# Subcellular Mechanical Imaging of Erythrocytes with Optically Correlated Scanning Ion Conductance Microscopy

**DOI:** 10.1021/acsmeasuresciau.5c00019

**Published:** 2025-04-02

**Authors:** Yunong Wang, Malavika Shashishekar, Dana M. Spence, Lane A. Baker

**Affiliations:** † Department of Chemistry, 14736 Texas A&M University, College Station, Texas 77843, United States; ‡ Department of Biomedical Engineering, Institute for Quantitative Health Science and Engineering, Michigan State University, East Lansing, Michigan 48824, United States

**Keywords:** red blood cell mechanical properties, scanning ion conductance microscopy, mechanical imaging, single-cell imaging, noninvasive biological imaging, optical correlation

## Abstract

We report mapping the mechanical properties of human red blood cells at submicron scales. Mapping is achieved via a new approach to scanning ion conductance microscopy correlated with optical microscopy. A three-point calibration and affine transformation are utilized to correlate pixel locations registered in optical images with pipette position, which facilitates initial targeting and subsequent tracking and analysis of red blood cells. By recording the response of pipette approach curves and sample compliance at each approach, maps of the Young’s modulus of samples and pipette indentation are recorded at subcellular spatial resolution. Comparison of normal and diamide-treated red blood cells shows a significant increase in cell stiffness and a concomitant decrease in deformability, clearly demonstrating the quantitative abilities of the correlative approach taken here for stiffness measurements of intact cellular samples.

## Graphical abstract

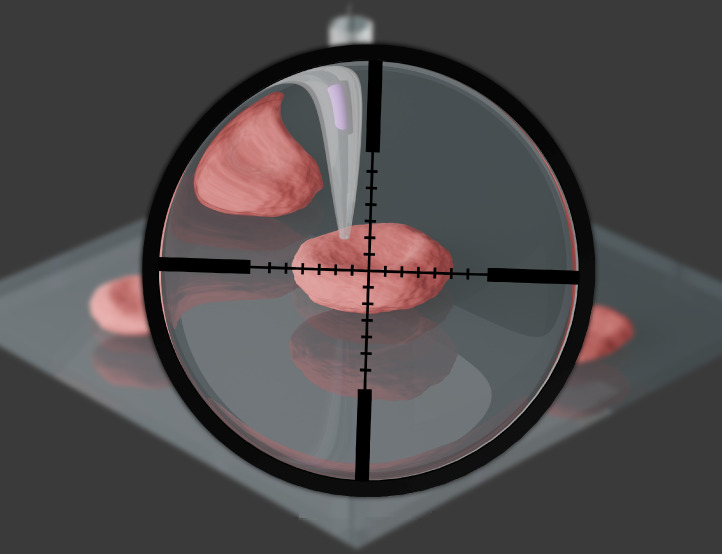

## 1 Introduction

The mechanical properties of red blood cells (RBCs) have long attracted significant interest due to the essential roles of hemorheology and hemodynamics in cell and organism function, including blood shear rates and viscosity, properties especially relevant to microcirculation.^1,2^ The RBC membrane is supported by a spectrin network, a quasi-hexagonal lattice of protein filaments linked by actin and anchored to the cell bilayer through protein complexes, such as ankyrin and band 3, which are crucial for maintaining mechanical stability and deformability.^3–5^ Disturbances or disruptions in the spectrin network are commonly associated with genetic defects, autoimmune disorders, and general RBC health. Membrane stiffness is a phenotype that can report on compositional changes in the membrane or oxidative stress. Additionally, environmental changes in *ex vivo* processes, such as blood storage, could potentially result in significant changes in RBC membrane mechanics, which may, in turn, impair normal function.^6–10^

A variety of techniques have been developed to investigate mechanical changes in individual RBCs, with the goal of developing a deeper understanding of membrane stiffness and RBC functionality.^11–13^ Among these techniques, atomic force microscopy (AFM) has been widely adopted for mapping the local mechanical properties of single RBCs.^14–17^ However, in many AFM-based imaging experiments on biological samples, the force exerted by the cantilever tip can exceed the mechanical threshold of the lipid bilayer, causing cells to collapse or rupture.^18–20^ Additionally, in many AFM measurements where membrane properties have been assessed, RBCs were air-dried or chemically fixed, which drives the cells away from a normal physiological state and inherently alters membrane stiffness.^14,21,22^ Further, AFM measurements tend to overestimate the magnitude of Young’s modulus due to the extreme softness of the RBC and inconsistencies in determining the point of contact between the AFM tip and the cell surface.^23^ To address these limitations, alternate approaches to better probe the relatively soft RBC membrane by decreasing the force on the sample have been developed. A highly promising approach has centered on use of scanning ion conductance microscopy (SICM) to apply localized hydrostatic pressure from the probe tip in a noncontact fashion.^24^ In 2008, Sánchez et al. applied SICM to the mechanical measurement of various types of living cells by ramping the hydrostatic pressure applied to samples through the tip of a nanopipette and highlighted its utility for single-point mechanical measurement of RBCs.^25^ However, this method suffered from limited spatial information and long measurement times (seconds per single point) due to the modulation of the pipette pressure. Subsequently, Rheinlaender and Schäffer developed improved protocols to image deformation and calculate the stiffness of single living fibroblast cells and human platelets using SICM.^26,27^ These advances demonstrated the practical utility of SICM in high-speed, high-resolution imaging scenarios for imaging and quantifying the properties of cell cytoskeletons, even for thin samples where overestimations of stiffness could be corrected.^26,27^ Rheinlaender and Schäffer clearly demonstrated that SICM provides a reliable approach for rapid and accurate quantitative mechanical measurements of living cells at subcellular spatial resolution. However, challenges remain in achieving precise stiffness mapping of RBCs and accounting for localized indentation effects caused by the scanning pipette. Optical microscopy has long been utilized in conjunction with SICM, in various automated forms, to determine the scanning region of interest, most predominantly by manual correlation, which is tedious and complicates runtime image processing.^28–30^ The existence of multiple RBCs within the SICM scanning area requires an efficient sequential imaging technique to facilitate targeting of multiple cells before and after experimental condition changes.

Herein, we present the design of an optically correlated pressurized-SICM system for imaging the stiffness of human erythrocytes and topographic changes induced by pipette indentation. The scanning protocol employs a three-point calibration and affine transformation to correlate pixel locations from an optical image of RBCs with XY scanner movement, enabling the selection of a targeted region by nanopipette through a simple software user interface without complicated runtime processing, such as image segmentation. Comparative analysis of 10 data sets from single, fresh RBCs and the same RBCs subjected to diamide stiffening was performed to validate the performance of the method. Results indicate that the average cell stiffness increased to approximately 4.9-fold (43.5–257.0 Pa), and the averaged vertical indentation caused by the pipette decreased by roughly 20% after diamide treatment. Moreover, the approach developed for optically correlated SICM measurements is generally tractable for any sample on a transparent substrate, which is likely to find significant utility in many applications of electrochemical microscopy, including SICM, scanning electrochemical microscopy, and scanning electrochemical cell microscopy.

## 2 Materials and Methods

### 2.1 Chemicals

Solutions were prepared with deionized water (18.20 MΩ·cm resistivity) from a filtration system (GenPure Pro UV-TOC, Thermo Scientific). Chemicals were used without further purification: sodium chloride (NaCl, VWR BDH Chemicals), potassium chloride (KCl, Sigma-Aldrich), sodium phosphate dibasic anhydrous (Na_2_HPO_4_, Macron Chemicals), potassium phosphate monobasic (KH_2_PO_4_, J.T. Baker), tetramethylazodicarboxamide (C_6_H_12_N_4_O_2_, diamide, Sigma-Aldrich), and agar (BD Bacto). SYLGARD 184 silicone elastomer base and curing agent (polydimethylsiloxane (PDMS), Dow) were also used. Phosphate-buffered saline (PBS) used in experiments contained 137.0 mM NaCl, 2.7 mM KCl, 10.0 mM Na_2_HPO_4_, and 1.8 mM KH_2_PO_4_. The pH was adjusted to 7.4 before use. The salt bridge was prepared by melting agar into heated PBS buffer with a 1:100 mass ratio and was pipetted into 10 μL graduated tips (TipOne, USA Scientific Inc.). The salt bridge was ready to use after it cooled to room temperature.

### 2.2 Nanopipette Fabrication

Pipettes with an inner tip diameter of ∼220 nm were fabricated using a pipette puller (P-1000, Sutter Instruments) and a single-barrel capillary (1.0 mm outer diameter, 0.7 mm inner diameter, 10 cm in length, Sutter Instruments) under the following parameters: HEAT = 505, PULL = 65, VEL = 78, DELAY = 167, PRESSURE = 500, and RAMP = 505 in Delay Mode. Typical top-down and side-view images of the pipette geometry are shown in Figure S1.

### 2.3 Blood Sample Preparation

Blood samples were collected from lab volunteers using a lancing device and lancets (CVS Pharmacy) and were transferred to plastic test tubes using a hematocrit tube (Hemato-Clad Mylar 75 MM Wrapped Hematocrit Tubes, Drummond Scientific Company). The original blood sample was diluted to a hematocrit of ∼0.7% and centrifuged at 5000 rpm for 3 min. The supernatant containing blood plasma and the buffy coat was then aspirated and removed, and fresh buffer was pipetted into the sample, followed by mixing with a vortex device for 6 s. This cleaning procedure was repeated 4 times. Blood samples were then pipetted onto a poly l-lysine coated glass slide (Poly-Prep Slides, Sigma-Aldrich) inside a regular culturing Petri dish, and the sample was left to rest for 30 min to allow RBCs to settle and promote adhesion to the lysine-coated slide. To mitigate substrate drift, polydimethylsiloxane (PDMS) was applied between the glass slide and the sample dish 1 day prior to blood preparation, and the assembly was enclosed in aluminum foil and cured on a hot plate at 80 °C overnight. An additional ∼4 mL of fresh buffer was then transferred into the Petri dish, and the sample was measured by SICM. For diamide stiffening, the sample solution was replaced with a PBS solution containing 100 μM diamide, and the RBC sample was allowed to incubate for 30 min. The buffer was then exchanged with normal, fresh PBS prior to imaging.

### 2.4 Pressurized-SICM Instrumentation

To develop the correlated optical-SICM approach, the SICM was set up over an inverted optical microscope (Eclipse TE2000-U, Nikon, with DMK 37BUR0234 CMOS sensor, The Imaging Source; Figure 1). The optical microscope was placed on an antivibration stage (AVI-200S/LP, Herzan). Pipette movement was achieved using a *Z*-axis stepper motor (M-112.1DG1, Physik Instrumente), a *Z*-axis piezoelectric actuator (P-753.21C, Physik Instrumente), and an *XY*-axis piezoelectric actuator (P-621.2CL, Physik Instrumente). Nanopipettes were pressurized through a high-pressure nitrogen gas line controlled by a pressure valve (TM-200, Narishige) with a custom-designed pipette holder. The SICM potentiostat (Axopatch 200B, Axon Instruments) was connected to the pipette to apply voltage and measure ion current. A multichannel interface (Axon Digidata 1550B, Axon Instruments) was connected to a computer for monitoring the signal channels. An FPGA board (NI-7845R OEM, National Instruments) was programmed to communicate with a home-built LabVIEW (2024, National Instruments) + Python (3.9.5) hybrid-designed software on the computer.

**Figure 1:**
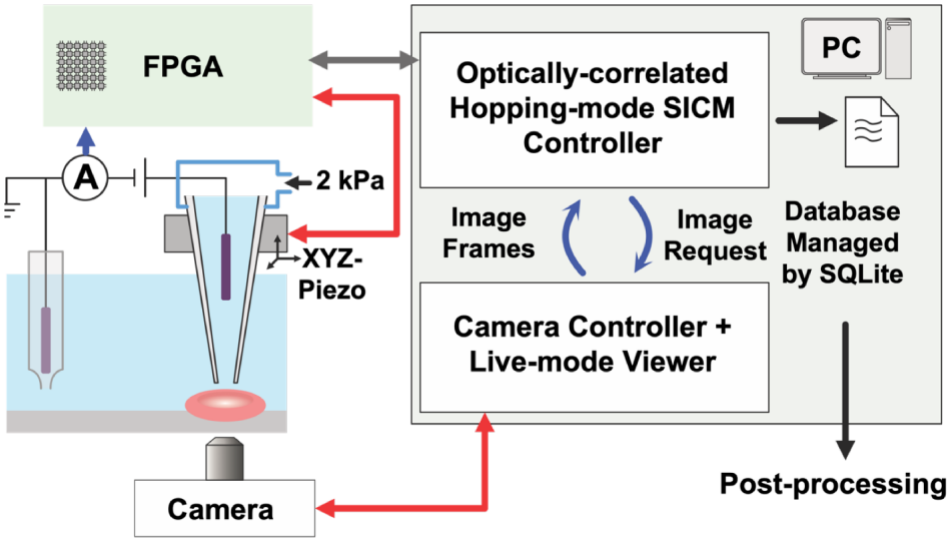
Instrumental architecture of optically correlated SICM. Pressurized pipette approaches a targeted cell with 2 kPa back pressure, with a potential difference applied between a pipette electrode and a bath electrode inserted in a phosphate-buffered salt bridge. Ion current is measured via an SICM potentiostat and sampled through analog-to-digital converters (ADCs) on an FPGA board. A host-PC communicates with the FPGA and the camera. The SICM controller sends requests to and obtains optical images from the camera controller to correlate the optical image to the piezo movement coordinates.

### 2.5 Pressurized-SICM Scanning Protocol

The pipette was back-pressurized with 2 kPa (*P*_0_) of nitrogen gas and then brought to a position ∼12.5 μm away from the sample surface using an automated approach protocol (Figure S2). A potential of +100 mV was applied to the pipette to generate an ion current, which was filtered with a 1 kHz analog low-pass 4-pole Bessel filter. Prior to imaging, the probe was repeatedly approached and retracted (or “hopped”) 200 times at an *XY* position of the substrate that was cell-free to obtain 200 ion current approach curves. These approach curves were then used to calculate the substrate reference. The region of the sample to be imaged was selected via optical correlation, as described in the following results section. The pipette was raster-scanned over RBCs in this region of interest, with a set of approach curves collected at each pixel. In the first approach curve, the pipette approached the surface at a rate of 8 μm·s^–1^ until a threshold of 0.5% current reduction was reached. This data was used to calculate the topography of the sample at a set point of 0.5%, where minimal tip–sample interaction is exhibited, and is taken as a noninteracting region of measurement.^31^ The pipette was then retracted for 1 μm at a rate of 15 μm·s^–1^. In the second approach curve at this same pixel, the pipette was approached to the surface at a smaller approach rate (1 μm·s^–1^) until a set point of 2.0% current threshold reduction was reached. A complete current-displacement response for this second approach curve was recorded by the FPGA and used to calculate the local mechanical response of the cell, as described below. Ion current and *z*-piezo position measurements were synchronized with the FPGA, and a 4-point averaging filter was applied to both ion current and *z*-piezo position in real time to improve the signal-to-noise ratio. Then, the pipette was retracted 5 μm from the surface at a rate of 15 μm·s^–1^ and advanced to the next pixel, where this process was repeated. A detailed procedure of the scanning protocol and the approach curve recording timing is demonstrated in Figure S3. Ion currents and piezo positions were extracted at runtime and saved as data files. All codes based on LabVIEW and Python for the automated pressurized-SICM scanning protocol are available at https://github.com/KLDistance/oc_pressurized_sicm.

### 2.6 Data Post Processing

Approach curves were extracted and analyzed using custom-built Python scripts. Two separate topographic images of the same RBC were obtained by extracting the *z*-piezo position at 0.5% and 2.0% current reduction (as described above) of a scanned area. A reference response for the mechanical properties of the substrate alone was obtained by analyzing 200 approach curves recorded over the cell-free substrate. For analysis, the region between 1% and 2% current reduction thresholds was extracted, and linear regression was performed on this dataset to generate approach curve slopes. The slope in this more extreme region of the approach curve effectively represents the compliance of the surface under the applied pressure. The reference response of the substrate, generated from these slopes, *s*_∞_, was determined from the median value of the set of slopes. The response of the RBC sample at each *XY* pixel was determined in a similar fashion (e.g., linear regression of the 1% to 2% region of approach curves) to generate a sample slope map, *s*. The Young’s modulus at each pixel could then be calculated from the values, *s*_∞_ and *s*, in eq 1 reported by Rheinlaender et al.^26^

1 $$E=AP_{0}{\left(\frac{s_{\infty }}{s}-1\right)}^{-1}$$

To isolate the location of the red blood cells, pixels indicative of cell regions were extracted by applying the Watershed imaging segmentation (skimage.segmentation library, Python) to topography images at a 2.0% ion current reduction threshold. The finite element method (FEM, COMSOL Multiphysics v6.1) was used to determine the geometry-dependent empirical parameter, *A* (eq 1), as described by Rhinelander et al., with details in SI.^26^ Histograms of the Young’s modulus for both normal and diamide-treated RBC samples were determined by analyzing the data at all pixels in each sample type.

## 3 Results and Discussion

### 3.1 Optically Correlated SICM

Hardware used to develop the SICM portion of the pressurized optically correlated SICM illustrated in Figure 1 was based on the platform we reported previously.^32^ A nanopipette was filled with phosphate-buffered saline. An Ag/AgCl quasi-reference counter electrode (QRCE) was back-inserted into this pipette, and the assembly was mounted on an *XYZ* piezo stage. A stepper motor was then used to submerge the distal end of the pipette into a PBS bath solution. To prevent contamination of the bath solution with Ag^+^, which has been shown to be deleterious to the lipid bilayer structure of RBCs, the reference electrode was inserted inside a phosphate-buffered salt bridge.^33,34^ Application of a potential difference between the two Ag/AgCl electrodes was used to generate a steady-state ion current through the pipette tip. A constant pressure of 2 kPa was applied at the back of the pipette through a custom-built pipette holder, creating a pressurized nanofluidic flow. An FPGA was used to control the piezo movement and to sample the ion current, asynchronously transferring *z*-piezo positions and ion current signals to the host PC.

On the host PC, two programs were developed: a camera controller and a hopping-mode SICM controller (Figure 1), to correlate the optical microscope images with the SICM *XY* piezo movement and facilitate real-time optical image viewing. The camera controller manages the camera sensor and continuously captures optical images, while the hopping-mode SICM controller interfaces with hardware via the FPGA. When operating independently, the camera controller serves solely as a live-mode viewer for the optical microscope, monitoring the pipette position and sample conditions. During SICM scanning, however, the SICM controller communicates with the camera controller via localhost transmission control protocol (TCP). Upon receiving a request notification from the SICM controller, the camera controller immediately suspends the live-mode imaging function and responds to the SICM controller with the latest optical image frame. To achieve image correlation, we implemented a three-point calibration technique, associating the optical image coordinates and piezo movement coordinates using affine transformation, as depicted in Figure 2. To efficiently identify the pipette tip location in the optical image while minimizing runtime computational complexity, a differential optical image is obtained by subtracting the images of the pipette in the extended state and the retracted state, when the pipette is at the same *XY* pixel. A threshold map is then obtained by applying 95% brightness threshold to the differential image, and the center of the white cluster (which represents the pipette position) is determined by averaging the *X* and *Y* indices of white pixels (Figure 2b). Repeating this step three times in total determines three different *XY* locations and yields three pairs of optical image and piezo movement coordinates, which are subsequently used to compute the affine transformation matrix. This matrix bridges the optical image locations and piezo movement locations. Details of the algorithm and a computational instance are described and demonstrated, respectively, in the SI, where a full calibration process was recorded and is shown as a movie in Movie S1. This approach leverages the brightness variation caused by the pipette’s shadow and avoids analyzing complicated sample morphology at runtime. Nevertheless, this technique is highly sensitive to sample movement and substrate drift during the calibration process and is well-suited for sample types with minimal lateral movement.

**Figure 2:**
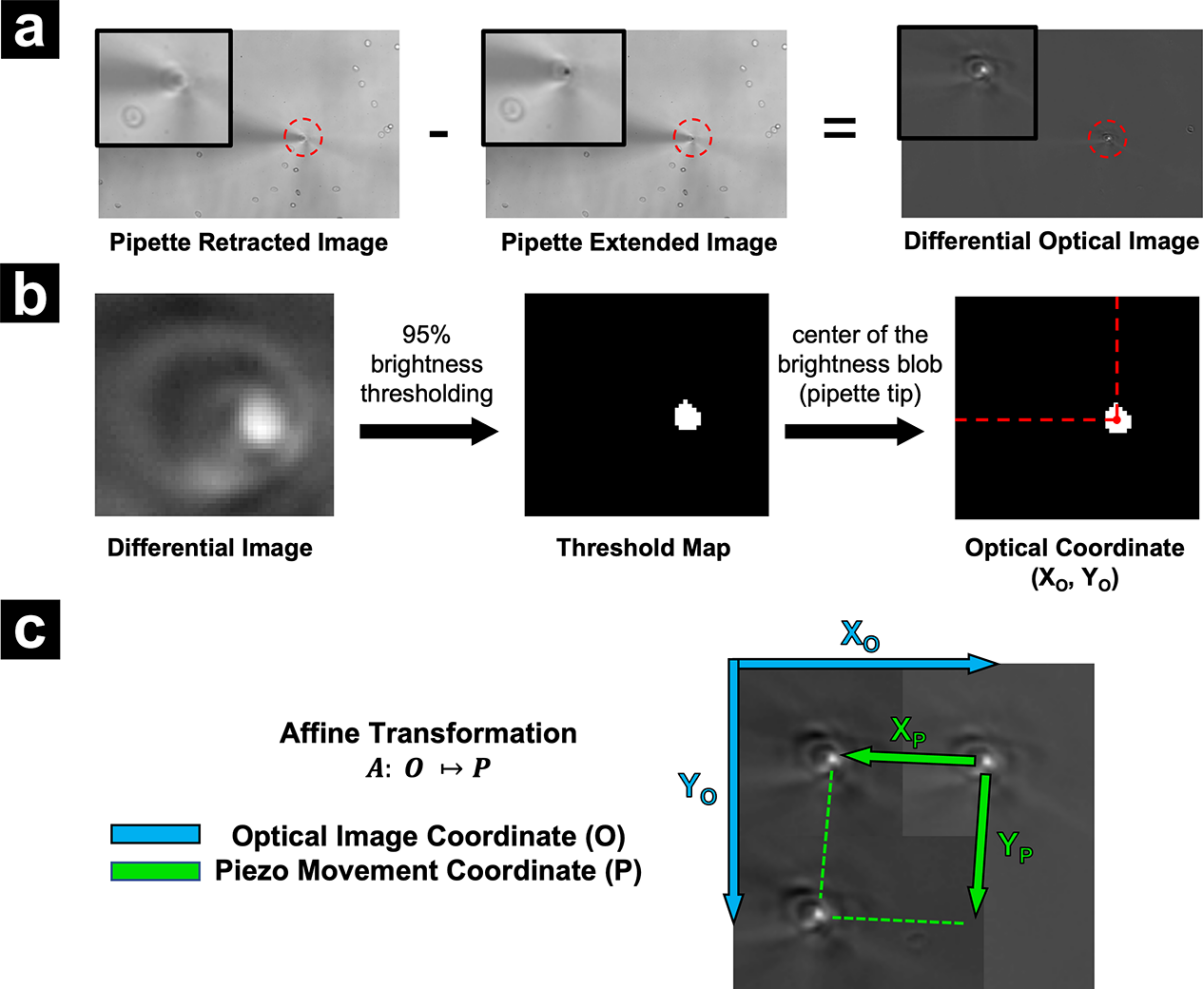
Procedure for three-point calibration/correlation of pipette tip position for optical image coordinates and piezo movement coordinates. (a) A differential optical image is generated from images with the pipette in retracted and extended states. A red dashed circle indicates the region magnified in the top left of each image. (b) Optical coordinates for the pipette position are determined by thresholding the differential map. (c) Steps a and b are repeated three times in total to obtain three *XY* piezo positions relative to the optical coordinate. A linear transformation matrix *A* is obtained by calculating the matrix product *PO* ^–1^. Green arrows represent the piezo movement coordinate, and the cyan arrows refer to the optical image coordinate.

### 3.2 Young’s Modulus Mapping on Human Red Blood Cells

Pressurized-SICM imaging was performed on immobilized single healthy RBCs, generating two types of SICM topographic images. One image at a set point of 0.5% and a second at a set point of 2.0%, as described in the methods section. The two topographic images were denoted separately as *h*_0.5%_ (height at 0.5%) and *h*_2.0%_(height at 2.0%). During imaging, the pipette approached the surface at 8 μm·s^–1^ until a 0.5% current reduction threshold was reached. Since the local indentation of the RBC caused by the pipette pressure was minimal at this set point, the RBC displayed normal discocyte (biconcave-disk) morphology (Figure 3a). After this initial approach, the pipette was retracted 1 μm from the surface and then reapproached at 1 μm·s^–1^ until a 2.0% current reduction threshold was reached. At this elevated set point, the local indentation of the RBC is significant and produced an apparent change in morphology that is directly related to the susceptibility of the RBC to mechanical deformation (Figure 3b), where the apparent change may be a convolution of the stiffness of the RBC with the true physical shape of the cell. The adoption of a smaller approach rate during the second approach was utilized to minimize bulk convection and overshoot of the *z*-piezo from rapid pipette translation. The deformability of RBCs is exhibited in the cross-section (shown at *Y* = 5.5 μm) in Figure 3c. A pronounced indentation with nominal decrease in height (at the center of the RBC, blue arrow A in Figure 3b) from 1.85 μm (measured at *h*_0.5%_, red trace) to 1.05 μm (measured at *h*_2.0%_, blue trace) is observed, a result of the increased pressure as the tip-to-sample distance decreases at higher set points of current threshold. Of note, the decrease in height and volume visually represented in the 2.0% threshold topographic map does not reflect actual cell shrinkage but rather a transient local deformation, reflective of the cell response at each individual pixel, as all pixels were not sampled at the same time. To readily visualize the magnitude of indentation over cells, an indentation map was developed as shown in Figure 3d, by subtracting the *h*_2.0%_ from the *h*_0.5%_. The lower values at center compared to the surrounding region of the RBC in the indentation map (Figure 3d) indicate a smaller deformation in the central region. The approach curves from all pixels were extracted. At each pixel, a linear regression was applied between 1.0% and 2.0% current reduction to determine a slope at that XY coordinate. The local Young’s modulus at each pixel was evaluated using this slope, as described in eq 1. This can be used to generate a map of the local Young’s modulus, as shown in Figure 3e. For clarification, approach curves at the limits measured here over the relatively “soft” center of a single RBC (point A in Figure 3b) and over a relatively “stiff” glass substrate (point B in Figure 3b) are shown in Figure 3f. The center of the cell shown in the example image displayed a stiffness of 87.1 Pa compared to the relatively softer edges of 24.8 Pa in the pressure map. These findings corroborate previous fluorescence imaging studies by Nowak et al., which demonstrated a denser spectrin-F-actin network distribution in the RBC’s central concave regions.^35^ For statistical purposes, pixels of the RBC were isolated from the entire image by the application of the Watershed image segmentation algorithm to the *h*_2.0%_ topography. The segmentation process generated a binary mask, which is subsequently used to localize the sample pixel locations and exclude the substrate pixels from further statistical analysis. This process is demonstrated in Figure S4. The cyan color in the pressure map background indicates the excluded substrate. This isolation step is particularly necessary. First, because pixels measured at the substrate are so stiff relative to the cell sample (stiffness values in excess of 10 kPa) that it can inundate “softer” response of RBCs, where stiffness values in the range of 100 Pa are relevant. Further, at the edges of the RBC, the approach of the pipette to the surface is complex, where the three-dimensional geometry of the pipette approaching the edge of the cell with a high radius of curvature deviates significantly from the ideal approach generated at a flat surface (Figure S5). This effect is manifested as high-indentation rings surrounding the cells in the indentation maps. This complex current–distance relationship created by the geometry of the RBC edges generates deviations that cannot be accounted for in the analysis conducted here. For these reasons, the edges of the RBCs are intentionally excluded.

**Figure 3:**
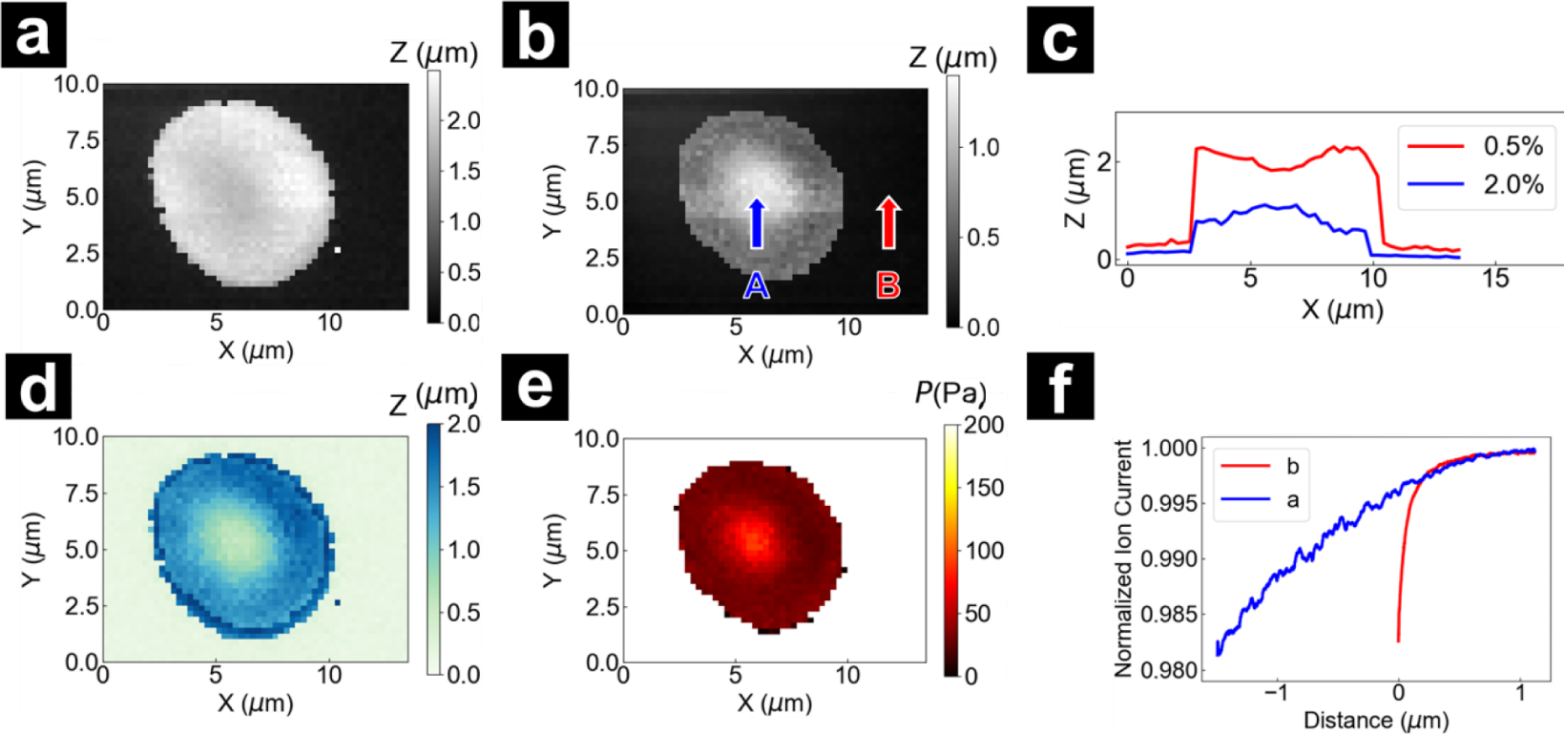
Pressure mapping of a single human RBC. Topography obtained at ion current thresholds (a) 0.5% and (b) 2.0%. (c) Line cut across the two topographic maps in (a) and (b) at Y = 5.5 μm indicating the height changes under different threshold. (d) Indentation map of the RBC. (e) The Young’s Modulus map of the RBC. (f) Normalized ion current approach curves extracted from the pixel in (b) over the (A) center of the RBC, and (B) the glass substrate.

### 3.3 Mechanical Comparison Between Normal and Chemically Stiffened RBC

To validate the quantification for the pressurized-SICM method employed here, comparative experiments were conducted on normal and chemically stiffened RBCs. The same scanning routine was used to generate a topography and pressure map of a single red blood cell. The same RBC was treated with 100 μM diamide in PBS buffer for 30 min, a recipe routinely used in RBC stiffening experiments.^36,37^ The same RBC was imaged again. As shown in Figure 4a,b, comparing the normal RBC to the diamide-treated RBC, the topography exhibited a minimal change in center height (∼1.68 μm). However, pixels near the edges changed from ∼0.79 to ∼1.61 μm. Indentation maps (Figure 4c,d) also revealed the same trend of change in height, where the diamide-treated cell showed a very small overall indentation depth. Corresponding Young’s modulus maps (Figure 4e,f) showed increased stiffness at the cell center from 62.1 to 244.1 Pa, whereas pixels near the edges increased from 36.2 to 80.8 Pa. Notably, a few single pixels in the images have high stiffness values >150 Pa, but surrounding pixels have relatively normal stiffness, which suggests these pixels are imaging artifacts caused by deviations in approach curve slopes from increased noise after buffer exchange and from vibration. In an effort to provide unadulterated data, we have left these pixels in the images shown. The pixel count of these high-stiffness artifacts can be visualized in Figure 5 and does not influence the overall trends observed. The fact that there was a greater increase in stiffness at the cell center relative to the pixels near the edges, also suggests diamide cross-linking was more effective at the center due to a denser spectrin network near the concave region of the RBC.

**Figure 4:**
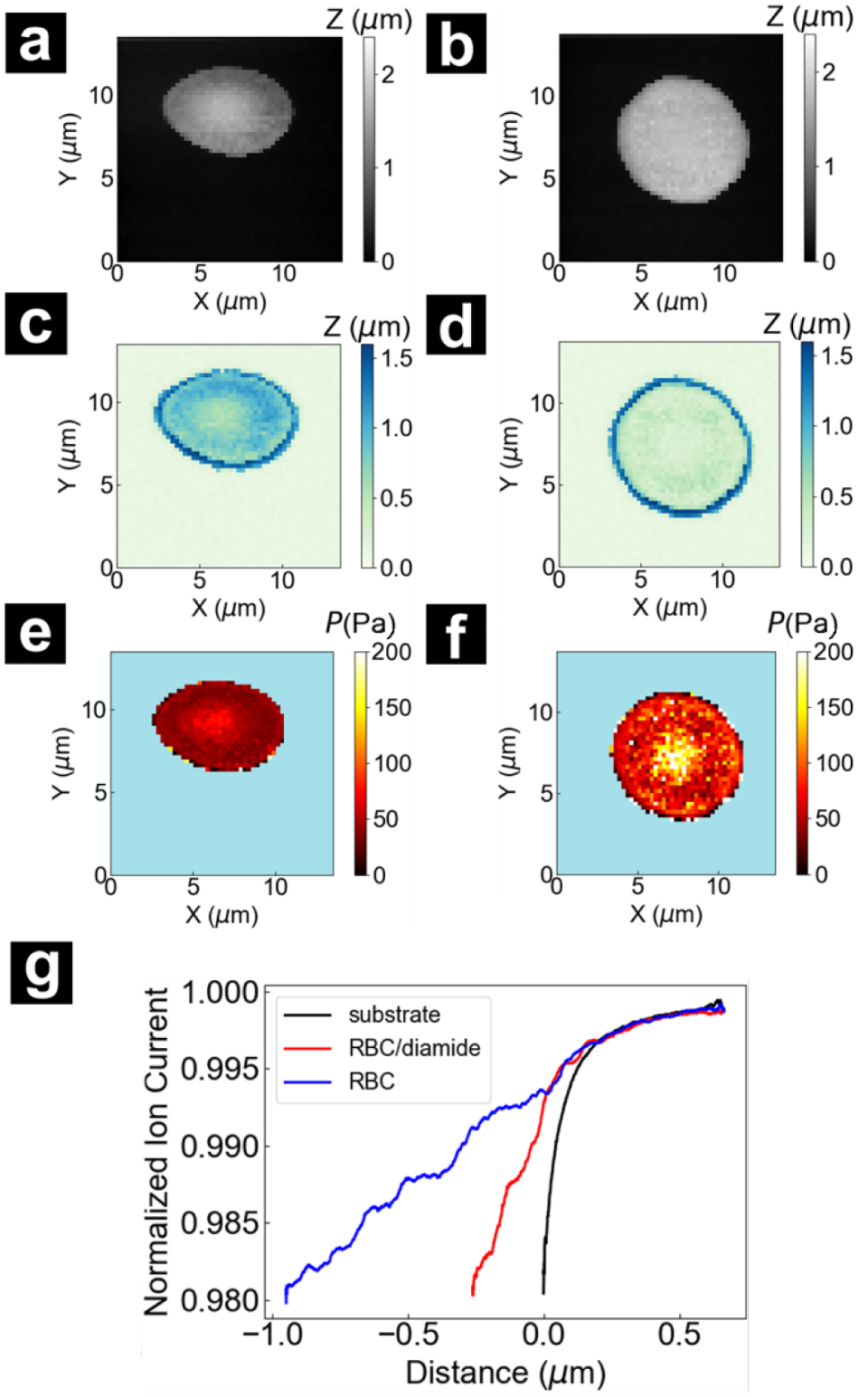
RBC images taken (a,c,e) before and (b,d,f) after the 100 μM tetramethylazodicarboxamide (diamide) treatment for 30 min. Topography of (a) normal and (b) same RBC treated with diamide at set point of 2.0%, (c,d) corresponding indentation maps and (e,f) the background-removed Young’s modulus maps of the same RBC. (g) The approach curves extracted over the glass substrate, the center of the RBC without diamide treatment, and the center of the diamide-treated RBC.

**Figure 5:**
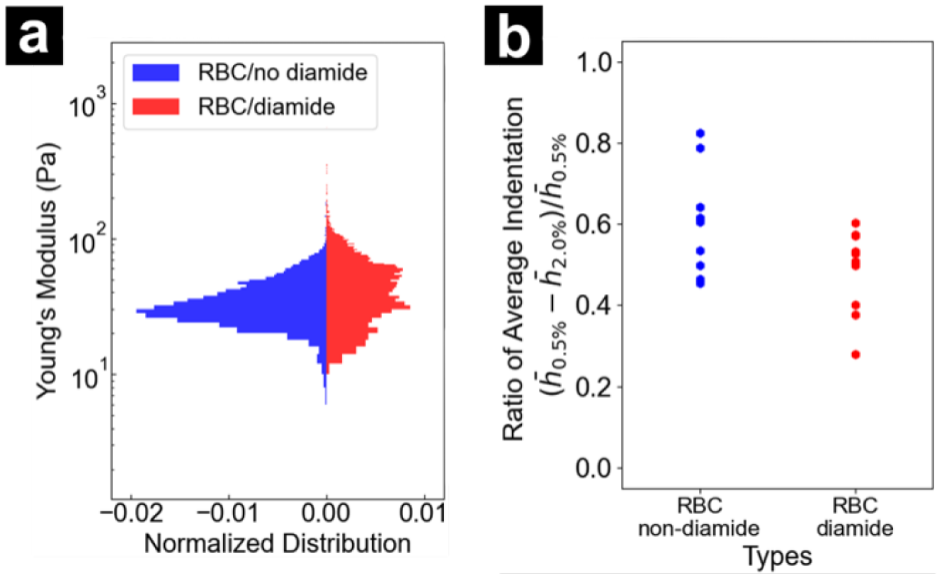
(a) Pixelwise normalized distribution and (b) ratio of average indentation of 10 normal and 10 diamide-treated RBCs.

This procedure was applied across multiple experiments to measure a total of 10 RBCs before and after the diamide treatment. Stiffness changes from diamide treatment, displayed as histograms of Young’s modulus values for 10 normal RBCs and the same cells post-diamide treatment are compared in Figure 5a. Non-diamide and diamide-treated RBCs showed different average stiffness values of 43.5 and 257.0 Pa, respectively, as confirmed by an independent *t*-test (*t* = 6.64 > 1.96, *p* < 3.35 × 10^–11^). This statistically validates a significant change in Young’s modulus before and after diamide treatment. Changes in deformability of RBCs, in terms of the indentation observed, were evaluated by averaging the *h*_0.5%_ and *h*_2.0%_ over all the RBC pixels in each sample to generate 10 pairs of ${\mathrm{h̅}}_{0.5\%}$ and ${\mathrm{h̅}}_{2.0\%}$ . The ratio of average indentation is defined as $\left({\mathrm{h̅}}_{0.5\%}-{\mathrm{h̅}}_{2.0\%}\right)/{\mathrm{h̅}}_{0.5\%}$ , which indicates indentation relative to the original sample height. The average indentation ratio of normal RBCs was 0.60, whereas the diamide-treated RBCs exhibited 0.48. The two distributions showed a significant difference according to a Student’s *t*-test (*t* = 2.84 > 2.26, *p* < 0.01). Notably, the subcellular approach taken here allows for statistical consideration that may overestimate the stiffness for diamide-treated RBCs, since extremely high Young’s modulus values in the central region of RBCs (>5 kPa) and possible image artifacts may exist, as shown in Figure 4f, as described above.

## 4 Conclusions

We described the design and application of optically correlated SICM for mechanical mapping of single human red blood cells and the quantification of stiffness changes following diamide treatment. The optical correlation technique was implemented using three-point calibration and affine transformation, which enabled the precise localization of regions of interest. Through Young’s modulus mapping, double-threshold topography, and indentation mapping of single RBCs, optical correlation was combined with pressurized SICM to reveal the spatial distribution of local cell stiffness and the magnitude of cell deformation at subcellular resolution. Pressurized SICM was used to quantify spatial variations in RBC stiffness, as demonstrated by the effects of diamide-induced stiffening.

As developed here, optically correlated SICM holds significant promise for diverse applications in electrochemical microscopy. Particularly, the biological behavior or electrochemical activity of micron- or submicron-sized single entities on optically transparent substrates can be localized and monitored using an inverted optical microscope in conjunction with scanning probes, as we have demonstrated previously in different applications of SICM.^38–42^ Additionally, digitization and automated control of the correlation afford the opportunity to integrate advanced computer vision algorithms, which can further enhance SICM in terms of the selection of sample areas and dynamic on-the-fly data analysis.

## Supplementary Material









## Data Availability

Data reported and used to generate results and other findings of this study are available from the corresponding author upon reasonable request.

## Notes

Detailed description of instrumentation and data processing procedures; SEM micrographs of nanopipettes; illustrations for automated approach and current-displacement signal recording scanning protocol; detailed three-point calibration computing process; RBC image segmentation; ion current response at RBC edges; finite element simulation details (PDF)
Three-point calibration runtime process (MP4)
Pressure hard results of pressurized-SICM (PDF)
Pressure soft results of pressurized-SICM (PDF)

## References

[1] Hamlin S. K., Benedik P. S. (2014). Basic Concepts of Hemorheology in Microvascular Hemodynamics. Crit. Care Nurs. Clin. North Am..

[2] Yedgar S., Koshkaryev A., Barshtein G. (2002). The Red Blood Cell in Vascular Occlusion. Pathophysiol. Haemost. Thromb..

[3] Grey J. L., Kodippili G. C., Simon K., Low P. S. (2012). Identification of
Contact Sites between Ankyrin and Band 3 in the Human Erythrocyte
Membrane. Biochemistry.

[4] Shotton D., Burke B., Branton D. (1978). The Shape
of Spectrin Molecules from
Human Erythrocyte Membranes. Biochim. Biophys.
Acta..

[5] Ralston G. B. (1975). Proteins
of the Camel Erythrocyte Membrane. Biochim.
Biophys. Acta..

[6] Barshtein G., Pajic-Lijakovic I., Gural A. (2021). Deformability of Stored
Red Blood
Cells. Front. Physiol..

[7] Barcellini W., Bianchi P., Fermo E., Imperiali F. G., Marcello A. P., Vercellati C., Zaninoni A., Zanella A. (2011). Hereditary
Red Cell Membrane Defects: Diagnostic and Clinical Aspects. Blood Transfus..

[8] Andolfo I., Russo R., Gambale A., Iolascon A. (2016). New Insights on Hereditary
Erythrocyte Membrane Defects. Haematologica.

[9] Yuan J., Bunyaratvej A., Fucharoen S., Fung C., Shinar E., Schrier S. L. (1995). The Instability
of the Membrane Skeleton in Thalassemic
Red Blood Cells. Blood.

[10] Groen K., Maltby V. E., Sanders K. A., Scott R. J., Tajouri L., Lechner-Scott J. (2016). Erythrocytes
in Multiple Sclerosis – Forgotten
Contributors to the Pathophysiology?. Mult.
Scler. J. - Exp. Transl. Clin..

[11] Weihs D., Mason T. G., Teitell M. A. (2006). Bio-Microrheology:
A Frontier in
Microrheology. Biophys. J..

[12] Hochmuth R. M. (2000). Micropipette
Aspiration of Living Cells. J. Biomech..

[13] Bronkhorst P. J., Streekstra G. J., Grimbergen J., Nijhof E. J., Sixma J. J., Brakenhoff G. J. (1995). A New Method to Study Shape Recovery of Red Blood Cells
Using Multiple Optical Trapping. Biophys. J..

[14] Sergunova V., Leesment S., Kozlov A., Inozemtsev V., Platitsina P., Lyapunova S., Onufrievich A., Polyakov V., Sherstyukova E. (2022). Investigation of Red Blood Cells
by Atomic Force Microscopy. Sensors.

[15] Dulińska I., Targosz M., Strojny W., Lekka M., Czuba P., Balwierz W., Szymoński M. (2006). Stiffness
of Normal and Pathological
Erythrocytes Studied by Means of Atomic Force Microscopy. J. Biochem Biophys Methods..

[16] Evans E. A., Waugh R. (1977). Osmotic Correction
to Elastic Area Compressibility Measurements on
Red Cell Membrane. Biophys. J..

[17] Lekka M., Fornal M., Pyka-Fościak G., Lebed K., Wizner B., Grodzicki T., Styczeń J. (2005). Erythrocyte Stiffness Probed Using
Atomic Force Microscope. Biorheology.

[18] Seifert J., Rheinlaender J., Novak P., Korchev Y. E., Schäffer T. E. (2015). Comparison
of Atomic Force Microscopy and Scanning Ion Conductance Microscopy
for Live Cell Imaging. Langmuir.

[19] Henderson E., Haydon P. G., Sakaguchi D. S. (1992). Actin Filament Dynamics in Living
Glial Cells Imaged by Atomic Force Microscopy. Science.

[20] You H. X., Lau J. M., Zhang S., Yu L. (2000). Atomic Force Microscopy
Imaging of Living Cells: A Preliminary Study of the Disruptive Effect
of the Cantilever Tip on Cell Morphology. Ultramicroscopy.

[21] Takeuchi M., Miyamoto H., Sako Y., Komizu H., Kusumi A. (1998). Structure
of the Erythrocyte Membrane Skeleton as Observed by Atomic Force Microscopy. Biophys. J..

[22] Nowakowski R., Luckham P. (2002). Imaging the Surface
Details of Red Blood Cells with
Atomic Force Microscopy. Surf. Interface Anal..

[23] Crick S. L., Yin F. C.-P. (2007). Assessing Micromechanical
Properties of Cells with
Atomic Force Microscopy: Importance of the Contact Point. Biomech. Model. Mechanobiol..

[24] Hansma P. K., Drake B., Marti O., Gould S. A. C., Prater C. B. (1989). The Scanning
Ion-Conductance Microscope. Science.

[25] Sánchez D., Johnson N., Li C., Novak P., Rheinlaender J., Zhang Y., Anand U., Anand P., Gorelik J., Frolenkov G. I., Benham C., Lab M., Ostanin V. P., Schäffer T. E., Klenerman D., Korchev Y. E. (2008). Noncontact Measurement
of the Local Mechanical Properties of Living Cells Using Pressure
Applied via a Pipette. Biophys. J..

[26] Rheinlaender J., Schäffer T. E. (2013). Mapping the Mechanical Stiffness of Live Cells with
the Scanning Ion Conductance Microscope. Soft
Matter..

[27] Rheinlaender J., Schäffer T. E. (2020). The Effect of Finite Sample Thickness in Scanning Ion
Conductance Microscopy Stiffness Measurements. Appl. Phys. Lett..

[28] Shevchuk A. I., Gorelik J., Harding S. E., Lab M. J., Klenerman D., Korchev Y. E. (2001). Simultaneous Measurement
of Ca2+ and Cellular Dynamics:
Combined Scanning Ion Conductance and Optical Microscopy to Study
Contracting Cardiac Myocytes. Biophys. J..

[29] Takahashi Y., Zhou Y., Miyamoto T., Higashi H., Nakamichi N., Takeda Y., Kato Y., Korchev Y., Fukuma T. (2020). High-Speed
SICM for the Visualization of Nanoscale Dynamic Structural Changes
in Hippocampal Neurons. Anal. Chem..

[30] Gu S., Zhuang J., Wang T., Hu S., Song W., Liao X. (2024). The Target
Region Focused Imaging Method for Scanning Ion Conductance
Microscopy. Ultramicroscopy.

[31] Del
Linz S., Willman E., Caldwell M., Klenerman D., Fernández A., Moss G. (2014). Contact-Free Scanning and Imaging
with the Scanning Ion Conductance Microscope. Anal. Chem..

[32] Wang Y., Rodriguez C., Alden S. E., Choi M., Alanis K., Srinivasan R., Baker L. A. (2024). Electrochemical Imaging of Neurotransmitter
Release with Fast-Scan Voltammetric Ion Conductance Microscopy. Sci. Adv..

[33] Sopjani M., Föller M., Haendeler J., Götz F., Lang F. (2009). Silver Ion-Induced Suicidal Erythrocyte Death. J. Appl. Toxicol..

[34] Zhu C., Shi W., Daleke D. L., Baker L. A. (2018). Monitoring Dynamic Spiculation in
Red Blood Cells with Scanning Ion Conductance Microscopy. Analyst.

[35] Nowak R. B., Alimohamadi H., Pestonjamasp K., Rangamani P., Fowler V. M. (2022). Nanoscale Dynamics
of Actin Filaments in the Red Blood
Cell Membrane Skeleton. Mol. Biol. Cell..

[36] Sridharan M., Sprague R. S., Adderley S. P., Bowles E. A., Ellsworth M. L., Stephenson A. H. (2010). Diamide Decreases Deformability of
Rabbit Erythrocytes
and Attenuates Low Oxygen Tension-Induced ATP Release. Exp. Biol. Med. Maywood..

[37] Price A. K., Martin R. S., Spence D. M. (2006). Monitoring
Erythrocytes in a Microchip
Channel That Narrows Uniformly: Towards an Improved Microfluidic-Based
Mimic of the Microcirculation. J. Chromatogr.
A.

[38] Shi W., Baker L. A. (2015). Imaging Heterogeneity and Transport of Degraded Nafion
Membranes. RSC Adv..

[39] Zhou Y., Chen C.-C., Baker L. A. (2012). Heterogeneity
of Multiple-Pore Membranes
Investigated with Ion Conductance Microscopy. Anal. Chem..

[40] Morris C. A., Chen C.-C., Ito T., Baker L. A. (2013). Local pH Measurement
with Scanning Ion Conductance Microscopy. J.
Electrochem. Soc..

[41] Zhou Y., Chen C.-C., Weber A. E., Zhou L., Baker L. A. (2014). Potentiometric-Scanning
Ion Conductance Microscopy. Langmuir.

[42] Zhu C., Zhou L., Choi M., Baker L. A. (2018). Mapping Surface
Charge of Individual Microdomains with Scanning Ion Conductance Microscopy. ChemElectrochem.

