# Supplementary material for: Subcellular Mechanical Imaging of Erythrocytes with Optically Correlated Scanning Ion Conductance Microscopy
Source: ACS Meas Sci Au. 2025 Apr 2;5(3):345–52. doi: 10.1021/acsmeasuresciau.5c00019 (PMC12183587; doi:10.1021/acsmeasuresciau.5c00019)
Supplement: Supplementary file 1 [file tg5c00019_si_001.pdf]

Supporting Information

Subcellular Mechanical Imaging of Erythrocytes with Optically-Correlated Scanning Ion Conductance Microscopy

Yunong Wang[1], Malavika Shashishekar.[1], Dana M. Spence[2*], Lane A. Baker[1*]

[1]*Department of Chemistry, Texas A&M University, College Station, Texas 77843, United States*

[2]*Department of Biomedical Engineering, Michigan State University, East Lansing, Michigan 48824, United States*

[2]*Institute for Quantitative Health Science and Engineering, Michigan State University, East Lansing, Michigan 48824, United States*

*Corresponding Authors:

Lane A. Baker – Email: lane.baker@tamu.edu; Phone: (979)-845-4721

Dana M. Spence – Email: spenceda@msu.edu; Phone: (517)-353-1116

Table of Contents

S1. Characterization of Nanopipette…………………………………………………………..S-3

S2. Scanning Protocol – Automated Approach Protocol…………………………………….S-4

S3. Scanning Protocol – Timing of Current-displacement Signal Recording…………..S-4

S4. Detailed Runtime Computing Process of 3-point Calibration………………………..S-5

S5. RBC Isolation Using Imaging Segmentation………………………………………..S-7

S6. Ion Current Response at RBC Edges……………………………………………….S-7

S7. Finite Element Method (FEM) Simulations…………………………………………S-8

Supplemental Methods

S1. Characterization of Nanopipettes

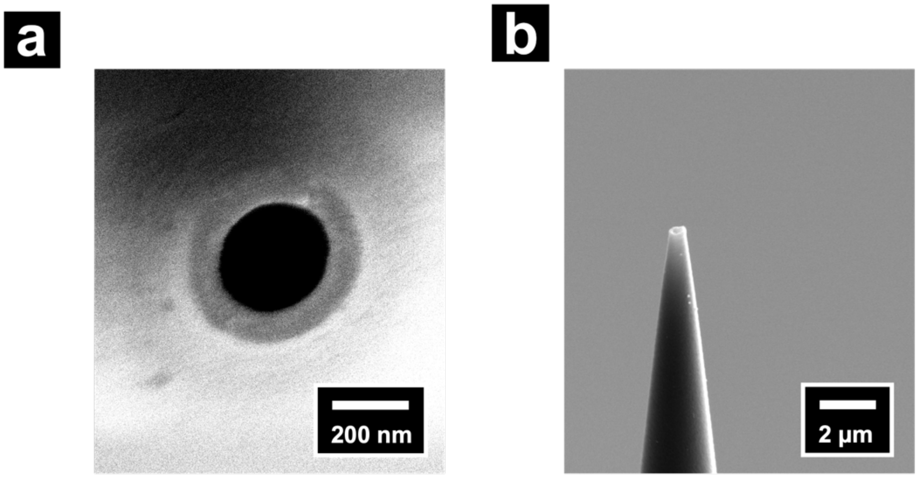

Figure S1. Scanning electron micrographs of (a) top view and (b) side view of the borosilicate single-barrel nanopipettes. The typical pipette geometry from the fabrication is ~220 nm I.D., ~360 nm O.D. with the half-cone angle at ~7.59º.

Borosilicate pipettes fabricated as described in the methods section showed a consistent geometry with an average inner diameter of 226±13 nm and outer diameter of 361±18 nm, with a pipette tip half-cone angle of 7.5±0.4º (n=10 pipettes). Scanning electron micrographs in Figure S1 show the typical geometry of a fabricated pipette from top and side view.

S2. Scanning Protocol – Automated Approach Protocol

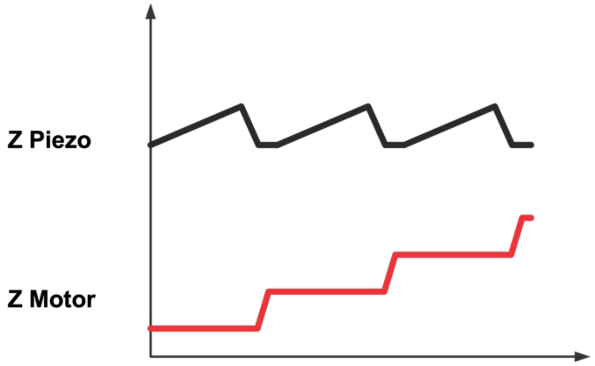

Figure S2. Automated approach protocol. The z piezo and stepper motor proceeds towards sample surface alternatively. First, the Z piezo ramps 2.5 μm towards sample surface at 20 μm s^{-1}. If a current decrease that exceeds a 1.2% threshold (setpoint) is not detected, then piezo is retracted at 45 μm s^{-1}. The z stepper motoradvanced to the surface by 2 μm. This process repeats until the pipette engages the surface (i.e., reaches a threshold current decrease of 1.2%). Final motor position is adjusted to leave ~12.5 μm tip-sample separation according to the piezo position at which the setpoint was reached.

S3. Scanning Protocol – Timing of Current-displacement Signal Recording

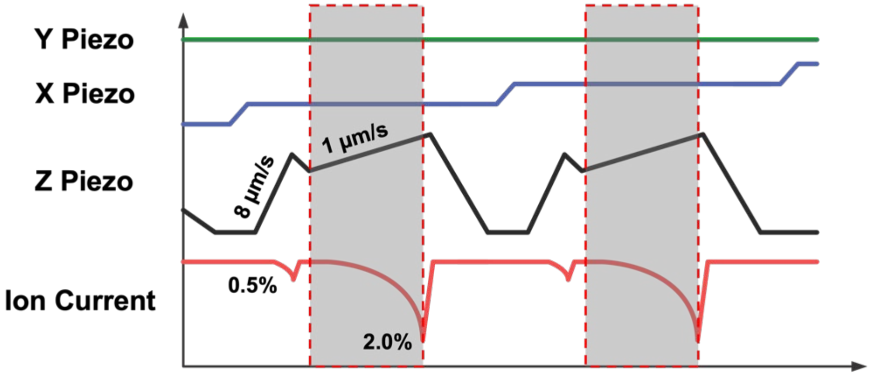

Figure S3. Traces of the X, Y, Z piezo and ion current feedback during a hopping-mode pressure mapping SICM experiment. At each XY pixel, the Z piezo uses a two-stage approach. In the first stage, a fast approach at 8 μm/s to reach a current reduction threshold of 0.5% is employed, followed by a second stage, where a fine approach at 1 μm/s is used until a current reduction threshold of 2.0% is reached. A 1 μm retraction between the phases allows sufficcient tip-to-sample separation for signal recording. The gray box in the figure indicates the time window in which the FPGA records the current-displacement curve.

S4. Detailed Runtime Computing Process of 3-point Calibration

1. At piezo X, Y position $x_p = 0$ μm and $y_p = 0$ μm , denoted as $C_p(0,0) = (0\ \mu\text{m}, 0\ \mu\text{m})$, the Z piezo is firstly held at retracted state $z_p = 0$ μm. Then, the SICM controller in host PC requests an optical image from the camera controller bridged through the TCP loopback communication between these two applications. The obtained optical image is a 2D matrix, which is denoted as $I_o(x_p, y_p, z_p) = I_o(0,0,0)$. For the next step, pipette is approached to the surface at 1.9% current threshold and its position is held near the surface. The SICM controller then requests another optical image, denoted as $I_o(0,0,1)$. The differential image is denoted as $D_o(x_p, y_p) = D_o(0,0) = I_o(0,0,0) - I_o(0,0,1)$. To get the pixel XY indices at the very middle of the bright spot in the differential image, the pixels above top 5% brightness, $D_{o,95\%}(0,0)$, are extracted and are visualized as a threshold map indicated in Figure 2b.

$$D_{o,\text{max}}(0,0) = \max\big(D_o(0,0)\big)$$

$$D_{o,\text{min}}(0,0) = \max\big(D_o(0,0)\big)$$

$$D_{o,95\%}(0,0) \geq D_{o,\text{min}}(0,0) + \Big(D_{o,\text{max}}(0,0) - D_{o,\text{min}}(0,0)\Big) \times 0.95$$

   Then, average the X and Y indices of the pixels with top 5% brightness respectively to get the central pixel point of the brightest spot $C_o(0,0) = (\bar{x}, \bar{y})$, where n is the number of pixels involved, and the $\bar{x}$ and $\bar{y}$ are simply the mean values of the indices:

$$\bar{x} = \frac{\sum x_{D_{o,95\%}(0,0)}}{n}$$

$$\bar{y} = \frac{\sum y_{D_{o,95\%}(0,0)}}{n}$$

   Here, for example, the obtained $C_o(0,0) = (1320.35,693.52)$.

2. Repeat step 1 at point $C_p(40,0) = (40\ \mu\text{m}, 0\ \mu\text{m})$ and $C_p(40,40) = (40\ \mu\text{m}, 40\ \mu\text{m})$ and get the other two central pixels: $C_o(40,0) = (1148.43,689.82)$ and $C_o(40,40) = (1148.93,861.13)$.

3. To create the standard affine transformation from optical coordinate O to piezo coordinate P, $A: O \mapsto P$, padding is required such that 3 by 3 transformation matrix can be established:

$$C_p(0,0) = \begin{bmatrix} 0 \\ 0 \\ 1 \end{bmatrix}, \quad C_p(40,0) = \begin{bmatrix} 40 \\ 0 \\ 1 \end{bmatrix}, \quad C_p(40,40) = \begin{bmatrix} 40 \\ 40 \\ 1 \end{bmatrix}$$

$$C_o(0,0) = \begin{bmatrix} 1320.35 \\ 693.52 \\ 1 \end{bmatrix}, \quad C_o(40,0) = \begin{bmatrix} 1148.43 \\ 689.82 \\ 1 \end{bmatrix}, \quad C_o(40,40) = \begin{bmatrix} 1148.93 \\ 861.13 \\ 1 \end{bmatrix}$$

The $A: O \mapsto P$, then, is represented as:

$$AO = P$$

$$A[C_o(0,0) \quad C_o(40,0) \quad C_o(40,40)] = \left[C_p(0,0) \quad C_p(40,0) \quad C_p(40,40)\right]$$

$$A = \left[C_p(0,0) \quad C_p(40,0) \quad C_p(40,40)\right][C_o(0,0) \quad C_o(40,0) \quad C_o(40,40)]^{-1}$$

$$A = \begin{bmatrix} 0 & 40 & 40 \\ 0 & 0 & 40 \\ 1 & 1 & 1 \end{bmatrix} \begin{bmatrix} 1320.35 & 1148.43 & 1148.93 \\ 693.52 & 689.82 & 861.13 \\ 1 & 1 & 1 \end{bmatrix}^{-1}$$

$$= \begin{bmatrix} -2.327 \times 10^{-1} & 6.791 \times 10^{-4} & 3.067 \times 10^{2} \\ -5.026 \times 10^{-3} & 2.335 \times 10^{-1} & -1.553 \times 10^{2} \\ -1.355 \times 10^{-19} & 0 & 0 \end{bmatrix}$$

4. The optical image determined by the 3-point calibration is extracted and allow user to click where to scan. For example, when two points are picked, $C_{o'1} = (1200,720)$ and $C_{o'2} = (1300,750)$ as a rectangular scanning region, the corresponding piezo can be derived:

$$P' = AO' = A \begin{bmatrix} 1200 & 1300 \\ 720 & 750 \\ 1 & 1 \end{bmatrix} = \begin{bmatrix} 27.95 & 6.79 \\ 4.70 & 13.29 \\ 0 & 0 \end{bmatrix}$$

The extracted two points under piezo movement coordinate are $C_{p'1} = (27.95\ \mu\text{m}, 6.79\ \mu\text{m})$ and $C_{p'2} = (4.70\ \mu\text{m}, 13.29\ \mu\text{m})$.

5. Start SICM hopping-mode raster scanning at the XY point:

$$\left(\min\left(C_{p'1x}, C_{p'2x}\right), \min\left(C_{p'1y}, C_{p'2y}\right)\right) = (4.70\ \mu\text{m}, 6.79\ \mu\text{m})$$

and end the scanning at the XY point:

$$\left(\max\left(C_{p'1x}, C_{p'2x}\right), \max\left(C_{p'1y}, C_{p'2y}\right)\right) = (27.95\ \mu\text{m}, 13.29\ \mu\text{m})$$

S5. RBC Isolation Using Imaging Segmentation

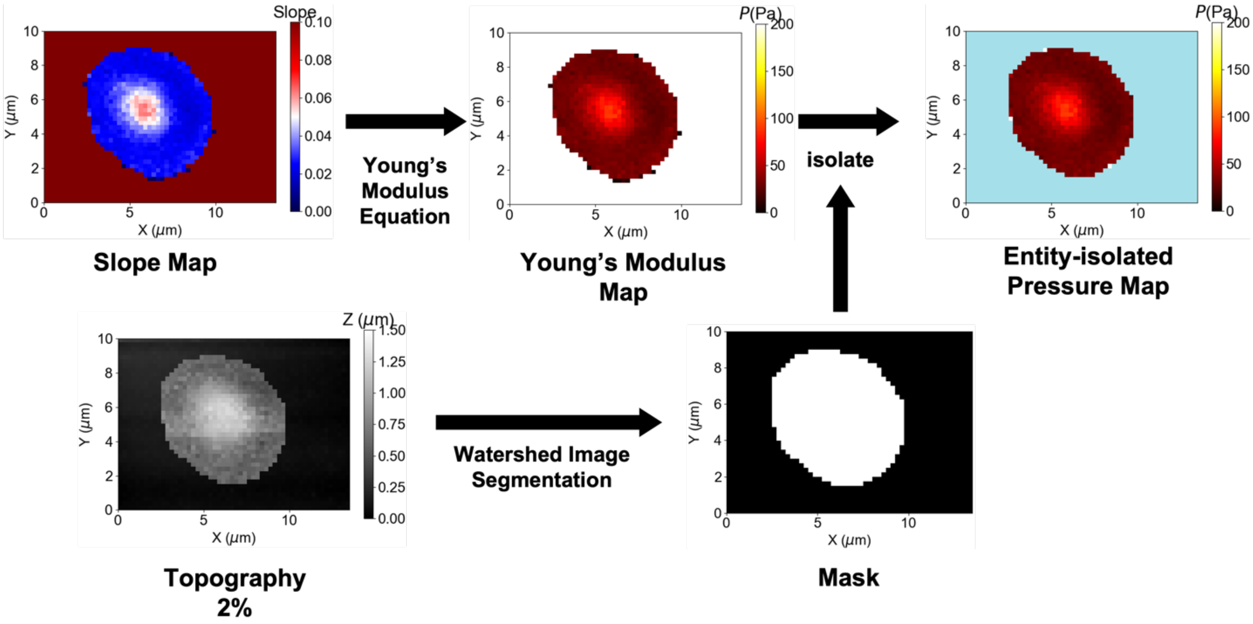

Figure S4. Single red blood cell image segmentation at post-processing phase.

S6. Ion Current Response at RBC Edges

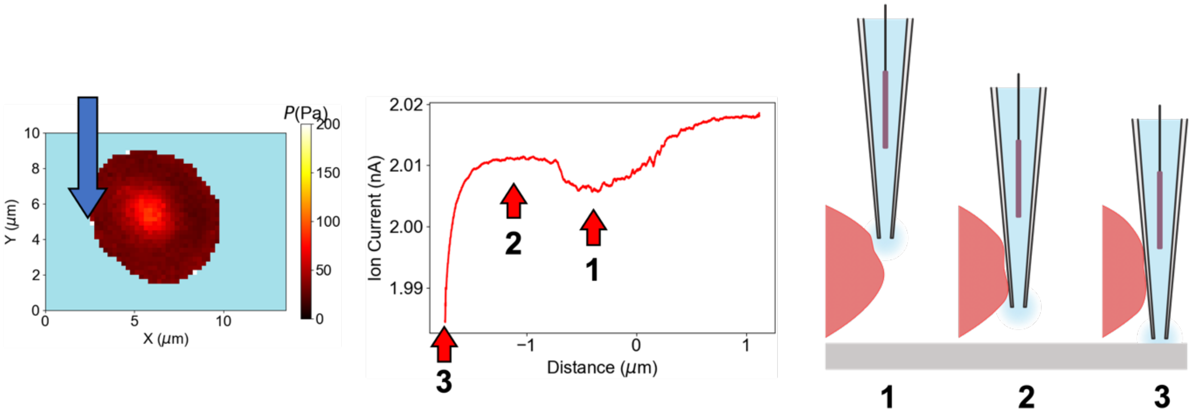

Figure S5. Approach curve responses at the edges of red blood cells, showing a non-conventional approach curve shape. The approach curve is separated into three phases: In phase 1, pipette approached near the RBC edge and caused slight deformation, with decreased ion current. In phase 2, pipette bypassed the edge and continued approaching, sensing a larger gap under the RBC edge with smaller tip-sample hinderance and increased ion current. In phase 3, pipette approached near the substrate and ion current decreased. More specifically, $h_{0.5\%}$ was sampled in phase 1, and $h_{2.0\%}$ was sampled in phase 3. This explains the existence of indentation rings in indentation maps.

S7. Finite Element Method (FEM) Simulations

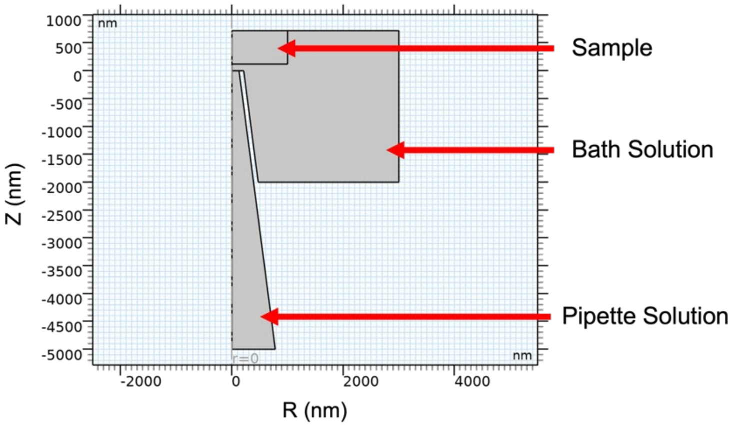

Figure S6. 2D axis symmetric geometric setup of the pressurized-SICM in COMSOL.

Finite element method simulations of Pressurized-SICM were established using COMSOL Multiphysics v6.1 based on the model previously reported by Rheinlaender and Schäffer with some adaptive modifications.[1] Two 2D axis symmetric models were produced to discuss pipette hydrostatic force on hard sample (Young's modulus $\mathrm{E} = 8 \times 10^8$ Pa) and soft sample ($\mathrm{E} = 8 \times 10^3$ Pa) respectively. The models used a combination of physics interfaces including moving mesh, deformed geometry, transport of diluted species, electrostatics, solid mechanics, laminar flow, and fluid-structure interaction to mimic the process of pipette approach in a bath solution towards a sample over a substrate.

The geometry setup included the pipette solution, bath solution and sample of interest, as shown in Figure S6, and matches the fabricated pipette geometry dimensions. The simulation adopted a stationary study with a parametric sweep of the tip-to-sample distance at 5 μm, followed by a range of 2500 nm down to 120 nm with a step size of 5 nm. 5 μm here serves the purpose of simulating the baseline current of pipette fully retracted state. The domain of pipette solution and bath solution used a moving mesh, where a laminar flow inlet of 4 kPa at the boundary of the back of the pipette was initialized, and the edge of the bath was set as a liquid outlet. To obtain an accurate simulation of ion migration under deformed substrate with hydrostatic flow, three Multiphysics couplings are implemented: Space Charge Density Coupling, Potential Coupling and fully

coupled Fluid-Structure Interaction. All the detailed settings were demonstrated in the attached reports: “pressure_hard_report.pdf” and “pressure_soft_report.pdf”.

The ion current evaluated from the model used the following equation is as follows:

$$I = |(\mathrm{flux}_{\mathrm{K}^+} - \mathrm{flux}_{\mathrm{Cl}^-}) \times F| \qquad (1)$$

where, the I is the net ion current, F is the Faraday constant. $\mathrm{flux}_{\mathrm{K}^+}$ and $\mathrm{flux}_{\mathrm{Cl}^-}$ are the flux of potassium and chloride ions through the orifice of the pipette tip. Two approach curves were extracted as shown in Figure S7. The , $\boldsymbol{s}_\infty$ and $\boldsymbol{s}$ from the approach curves were calculated by applying linear regression to the segment of 1% to 2% current reduction threshold. The geometry-dependent empirical parameter ***A*** is derived as 0.1617 from the calculation.

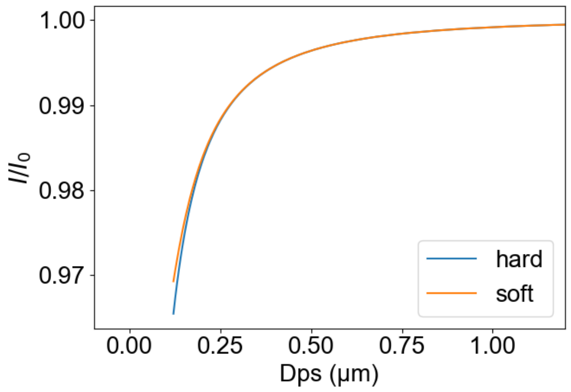

Figure S7. Simulated approach curves over hard substrate ($\mathrm{E} = 8 \times 10^8$ Pa) and soft sample surface ($\mathrm{E} = 8 \times 10^3$ Pa).

References

(1) Rheinlaender, J.; Schäffer, T. E. Mapping the Mechanical Stiffness of Live Cells with the Scanning Ion Conductance Microscope. *Soft Matter* **2013**, *9* (12), 3230–3236.
