# Supplementary material for: Subcellular Mechanical Imaging of Erythrocytes with Optically Correlated Scanning Ion Conductance Microscopy
Source: ACS Meas Sci Au. 2025 Apr 2;5(3):345–52. doi: 10.1021/acsmeasuresciau.5c00019 (PMC12183587; doi:10.1021/acsmeasuresciau.5c00019)
Supplement: Supplementary file 3 [file tg5c00019_si_003.pdf]

Pressure hard result

Report date	Feb 2, 2025, 7:40:01 PM

Contents

1. Global Definitions 3

1.1. Parameters 3

1.2. Shared Properties 4

2. Component 1 5

2.1. Definitions 5

2.2. Geometry 1 6

2.3. Materials 8

2.4. Moving Mesh 16

2.5. Deformed Geometry 17

2.6. Transport of Diluted Species 18

2.7. Electrostatics 39

2.8. Solid Mechanics 60

2.9. Laminar Flow 143

2.10. Multiphysics 176

2.11. Mesh 183

3. study1 190

3.1. Parametric Sweep 190

3.2. Stationary 190

3.3. Solver Configurations 191

4. Results 3069

4.1. Datasets 3069

4.2. Derived Values 3073

4.3. Tables 3074

4.4. Plot Groups 3088

1 Global Definitions

Date	Dec 9, 2024, 12:34:41 AM

GLOBAL SETTINGS

Name	Pressure hard result.mph
Path	/data/yunongwang/pressure_sicm/deform_geometry/sim3_improved_moving_mesh/pressure_hard_result.mph
Version	COMSOL Multiphysics 6.1 (Build: 252)
Unit system	SI

USED PRODUCTS

COMSOL Multiphysics
Chemical Reaction Engineering Module
MEMS Module

COMPUTER INFORMATION

CPU	AMD EPYC 7402P 24-Core Processor, 24 cores, 124.89 GB RAM
Operating system	Linux

1.1 PARAMETERS

PARAMETERS 1

Name	Expression	Value	Description
d	1.78E-7[m]	1.78E−7 m	dps
rhos	0.01[C/m^2]	0.01 C/m²	surface charge density on substrate
rhop	-0.001[C/m^2]	−0.001 C/m²	surface charge density on pipette
c	140[mM]	140 mol/m³	concentration of bulk
theta	7.42[deg]	0.1295 rad	inner half cone angle
roi	1.65	1.65	outer to inner radius ratio
pr	128[nm]	1.28E−7 m	inner pipette radius
pl	5000[nm]	5E−6 m	probe length
sh	0.6[um]	6E−7 m	thickness of sample with finite stiffness
sw	1[um]	1E−6 m	half width of sample with finite stiffness
p0	4e3[Pa]	4000 Pa	
dpert	5[nm]	5E−9 m	
hpert	5[nm]	5E−9 m	

1.2 SHARED PROPERTIES

1.2.1 Default Model Inputs

Tag	cminpt

2 Component 1

Date	Aug 18, 2021, 3:15:55 PM

SETTINGS

Description	Value
Unit system	Same as global system (SI)
Geometry shape function	Automatic

SPATIAL FRAME COORDINATES

First	Second	Third
r	phi	z

MATERIAL FRAME COORDINATES

First	Second	Third
R	PHI	Z

GEOMETRY FRAME COORDINATES

First	Second	Third
Rg	PHIg	Zg

MESH FRAME COORDINATES

First	Second	Third
Rm	PHIm	Zm

2.1 DEFINITIONS

2.1.1 Coordinate Systems

Boundary System 1

Coordinate system type	Boundary system
Tag	sys1

COORDINATE NAMES

First	Second	Third
t1	to	n

2.2 GEOMETRY 1

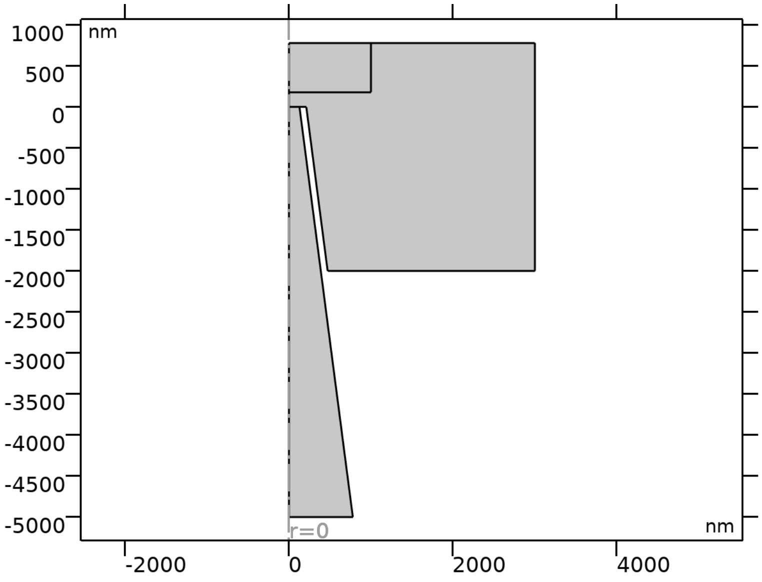

Geometry 1

UNITS

Length unit	nm
Angular unit	deg

GEOMETRY STATISTICS

Description	Value
Space dimension	2
Number of domains	3
Number of boundaries	20
Number of vertices	18

2.2.1 bulk (pol2)

OBJECT TYPE

Description	Value
Type	Solid

COORDINATES

Description	Value
Data source	Table

COORDINATES

r (nm)	z (nm)

r (nm)	z (nm)
0	0
pr	0
pr*roi	0
pr+500*tan(theta) + pr*(roi-1)	-500
pr+2000*tan(theta) + pr*(roi-1)	-2000
3000	-2000
3000	-1500
3000	d+sh
1500	d+sh
sw	d+sh
0	d+sh
0	0

2.2.2 pipette (pol1)

OBJECT TYPE

Description	Value
Type	Solid

COORDINATES

Description	Value
Data source	Table

COORDINATES

r (nm)	z (nm)
0	0
pr	0
pr+500*tan(theta)	-500
pr+pl*tan(theta)	-pl
0	-pl
0	-pl*0.15
0	-pl*0.001
0	0

2.2.3 soft sample (pol3)

OBJECT TYPE

Description	Value

Description	Value
Type	Solid

COORDINATES

Description	Value
Data source	Table

COORDINATES

r (nm)	z (nm)
0	d
sw	d
sw	d+sh
0	d+sh
0	d

2.3 MATERIALS

2.3.1 water

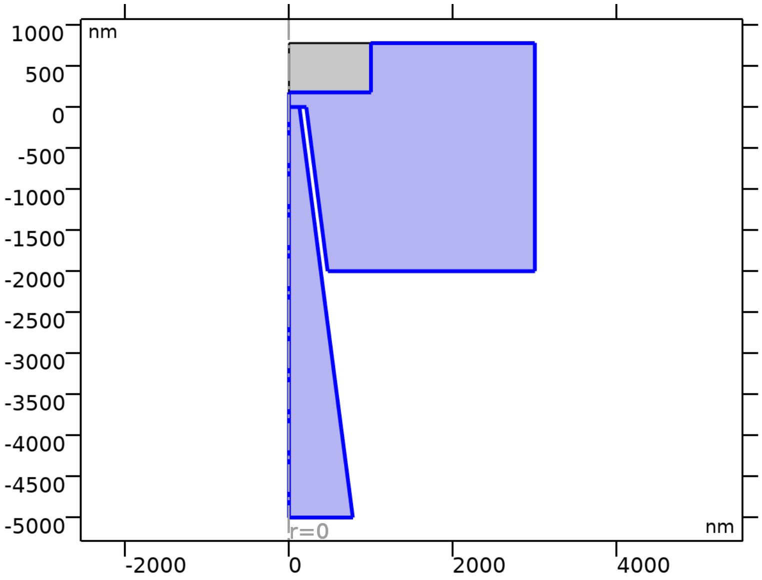

water

SELECTION

Geometric entity level	Domain
Selection	Geometry geom1: Dimension 2: Domains 1–2

MATERIAL PARAMETERS

Name	Value	Unit	Property group

Name	Value	Unit	Property group
Dynamic viscosity	eta(T)	Pa·s	Basic
Density	rho(T)	kg/m³	Basic
Relative permittivity	80	1	Basic

BASIC

Description	Value	Unit
Dynamic viscosity	eta(T)	Pa·s
Density	rho(T)	kg/m³
Relative permittivity	80	1

FUNCTIONS

Function name	Type
eta	Piecewise
Cp	Piecewise
rho	Piecewise
k	Piecewise
cs	Interpolation
alpha_p	Analytic
gamma_w	Analytic
muB	Analytic

Piecewise

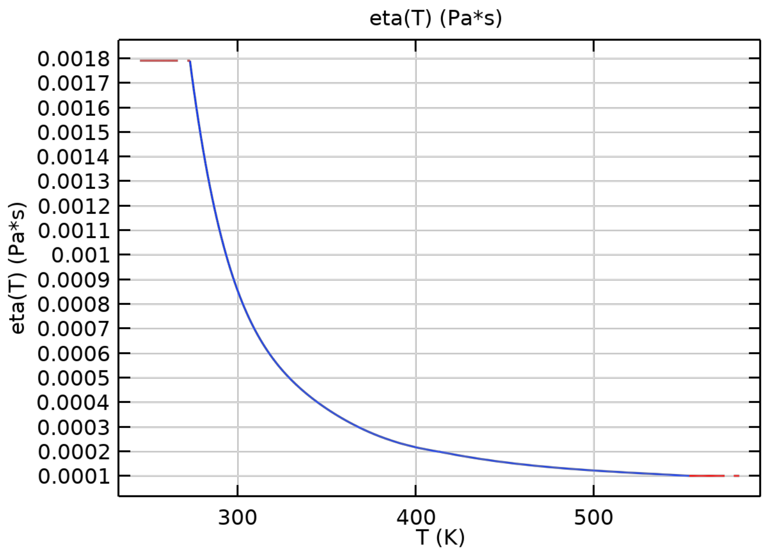

eta

Piecewise 2

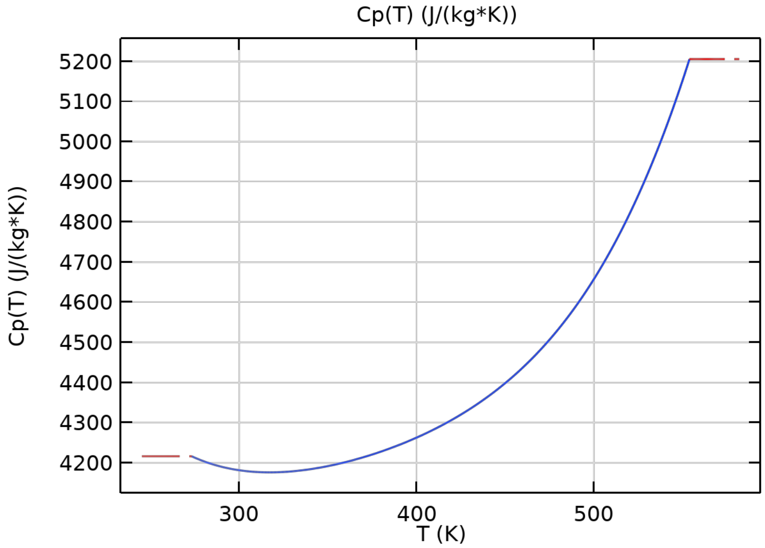

Cp

Piecewise 3

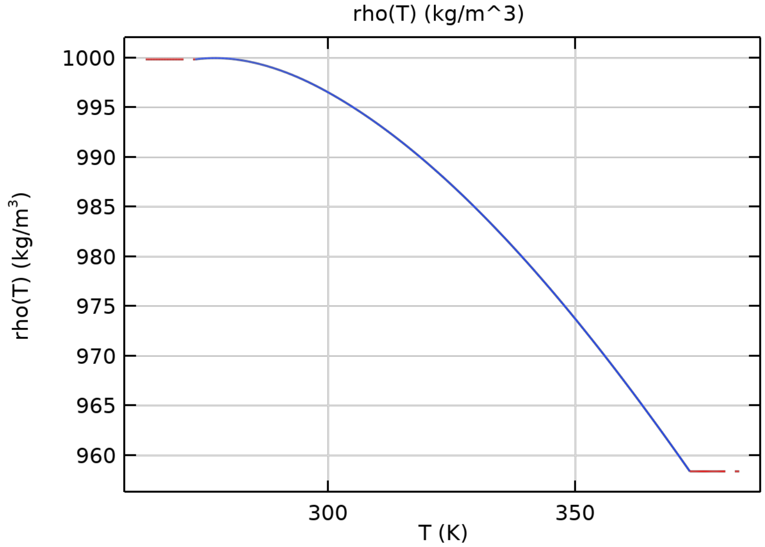

rho

Piecewise 4

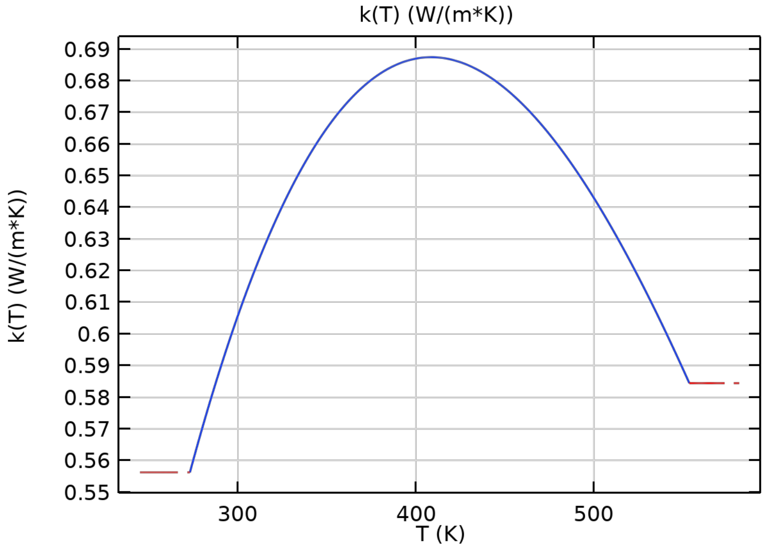

k

Interpolation

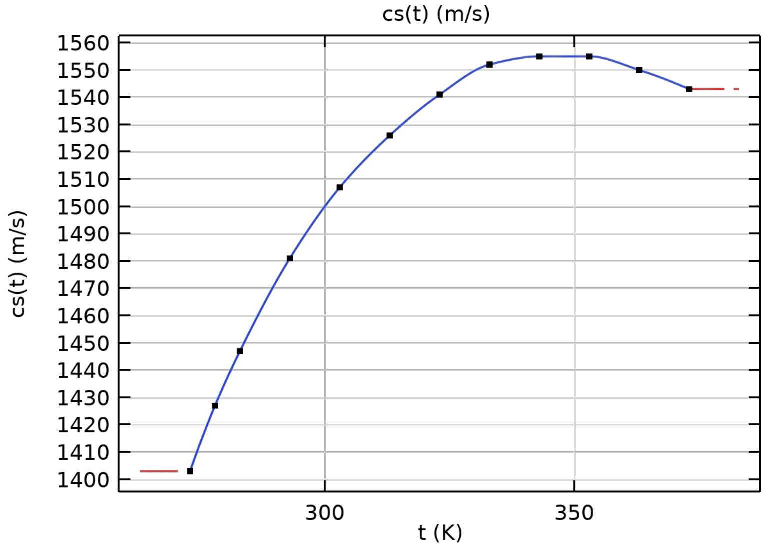

cs

Analytic 1

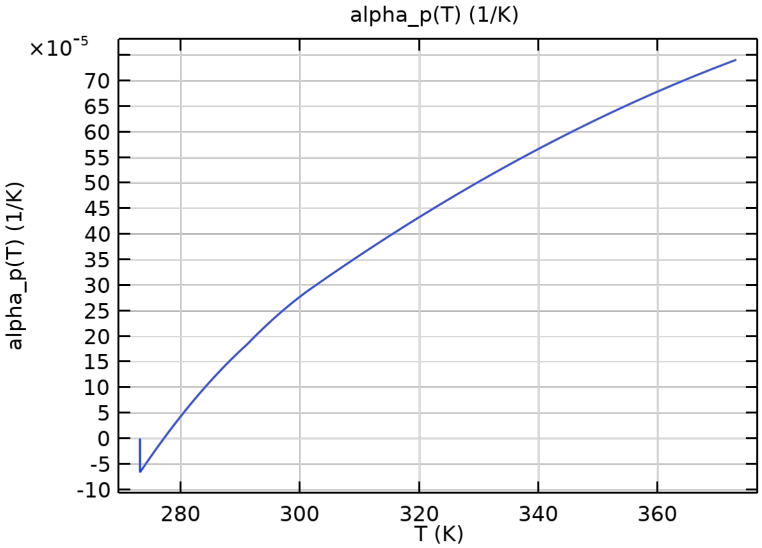

alpha_p

Analytic 2

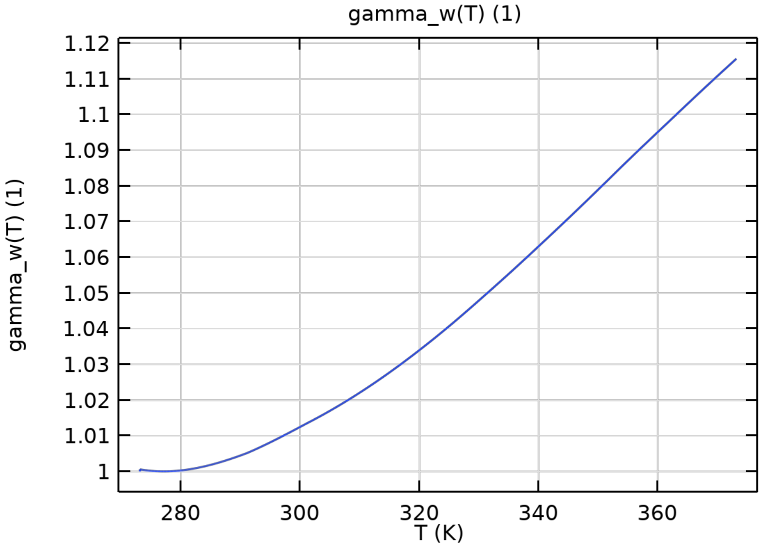

gamma_w

Analytic 3

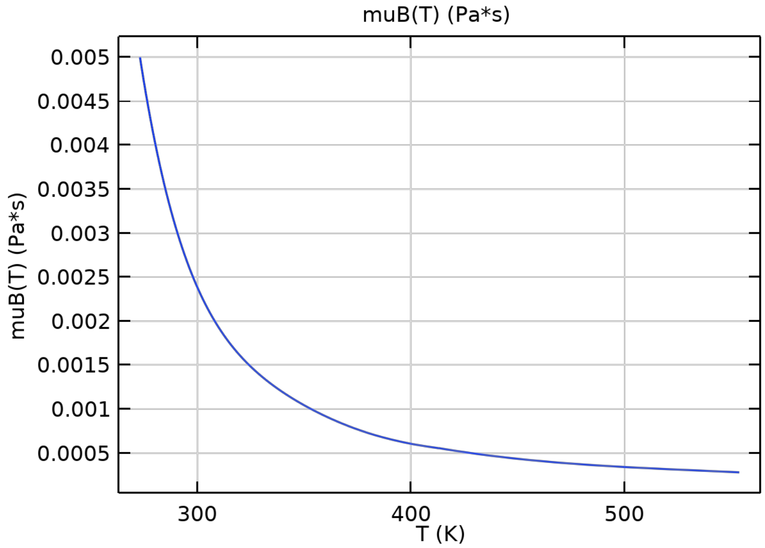

muB

2.3.2 polystyrene

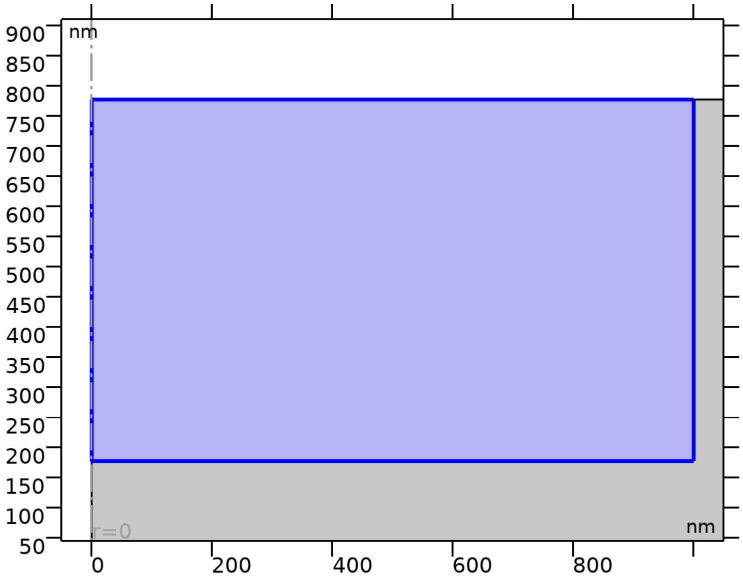

polystyrene

SELECTION

Geometric entity level	Domain
Selection	Geometry geom1: Dimension 2: Domain 3

MATERIAL PARAMETERS

Name	Value	Unit	Property group

Name	Value	Unit	Property group
Density	1	kg/m³	Basic
Young's modulus	8E8	Pa	Young's modulus and Poisson's ratio
Poisson's ratio	0.4999	1	Young's modulus and Poisson's ratio

BASIC

Description	Value	Unit
Density	1	kg/m³

FUNCTIONS

Function name	Type
TD	Piecewise

Piecewise

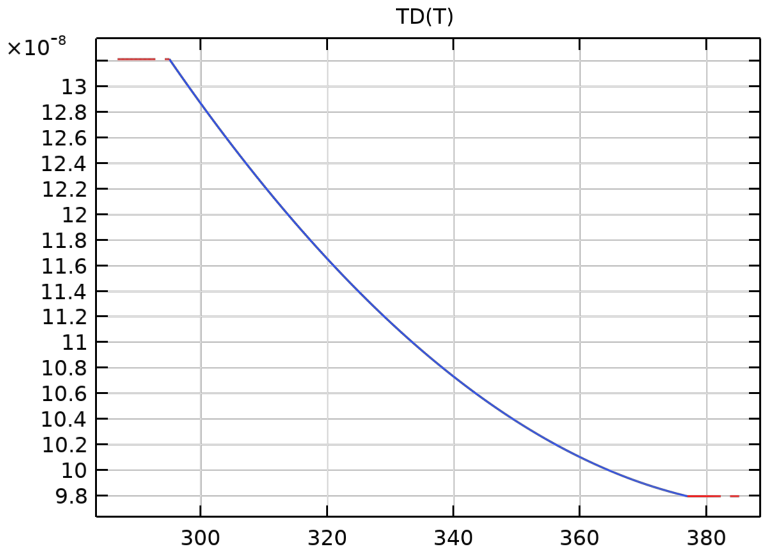

TD

YOUNG'S MODULUS AND POISSON'S RATIO

Description	Value	Unit
Young's modulus	8E8	Pa
Poisson's ratio	0.4999	1

FUNCTIONS

Function name	Type
E	Piecewise
nu	Piecewise

Piecewise

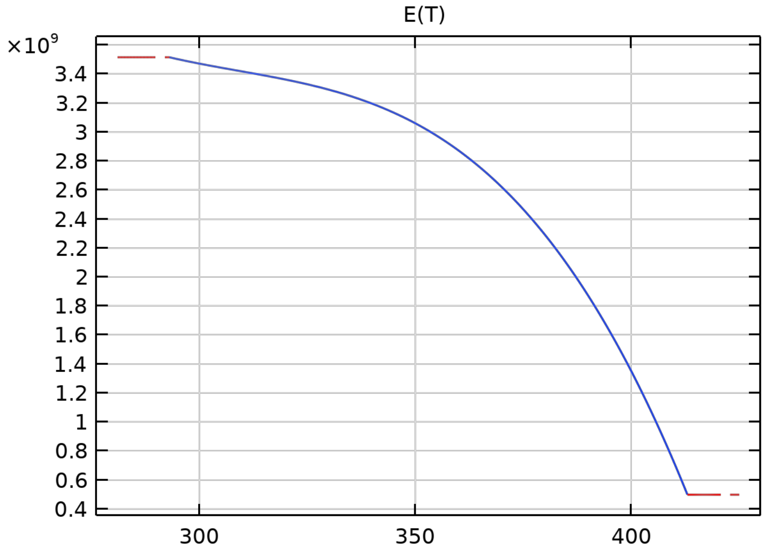

E

Piecewise 1

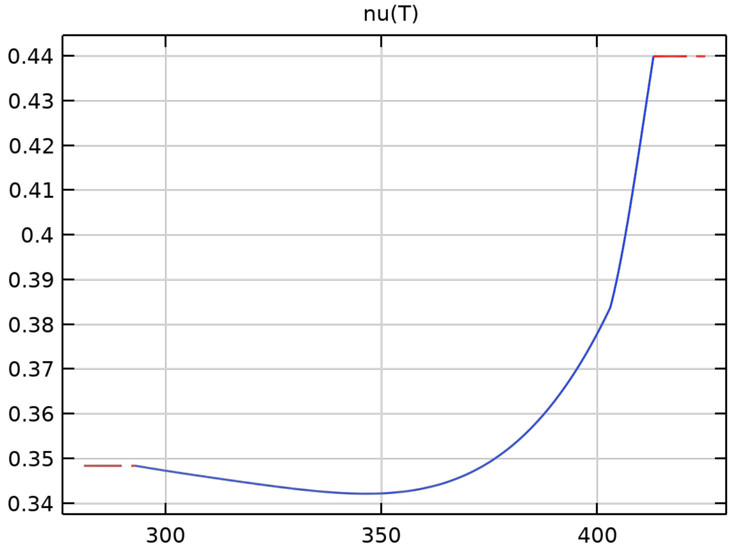

nu

FUNCTIONS

Function name	Type
mu	Piecewise
kappa	Piecewise

Piecewise

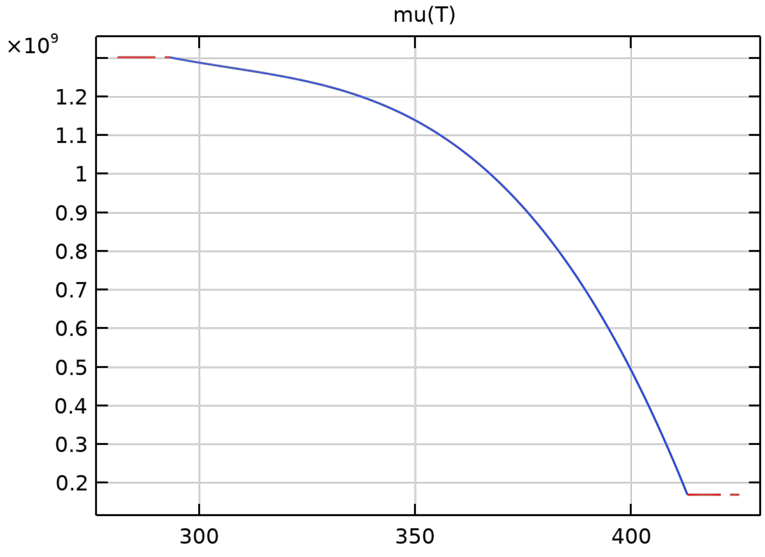

mu

Piecewise 1

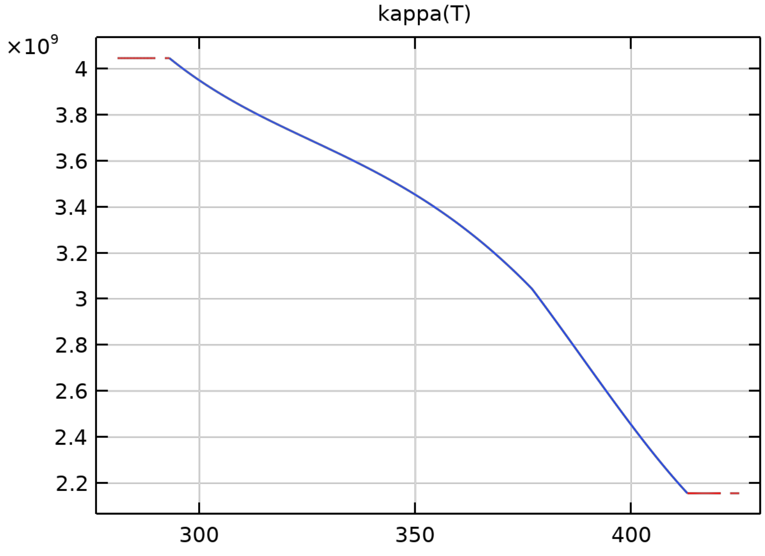

kappa

2.4 MOVING MESH

2.4.1 Deforming Domain 1

Tag	free1

SELECTION

Geometric entity level	Domain
Selection	Geometry geom1: Dimension 2: Domain 2

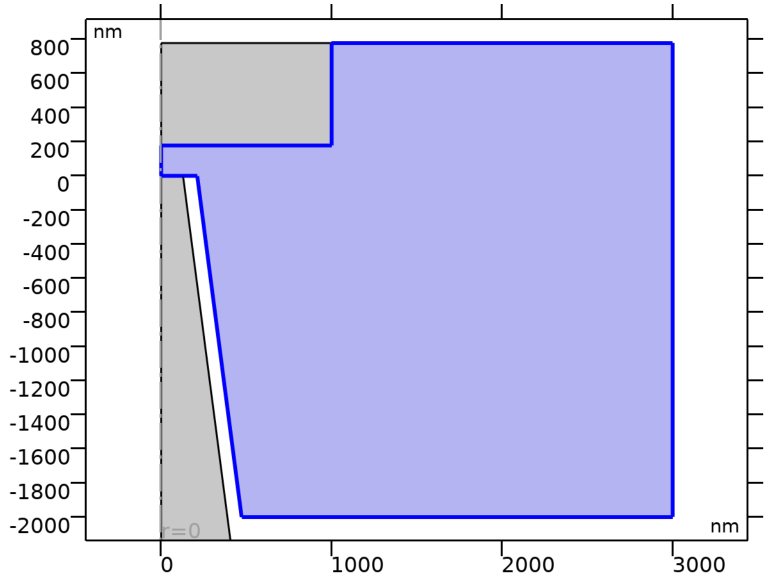

Selection

SMOOTHING

Description	Value
Stiffening factor	1E8

INITIAL DEFORMATION

Description	Value
Initial deformation	{0, 0, 0}

2.5 DEFORMED GEOMETRY

2.5.1 Prescribed Deformation 1

Tag	pres1

SELECTION

Geometric entity level	Domain
Selection	Geometry geom1: Dimension 2: Domain 3

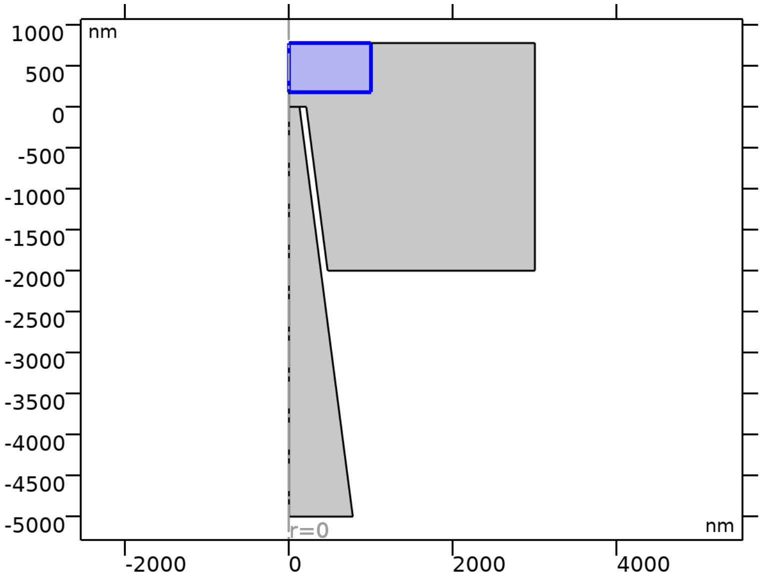

Selection

PRESCRIBED DEFORMATION

Description	Value
Prescribed deformation	{0, 0, 0}

2.6 TRANSPORT OF DILUTED SPECIES

USED PRODUCTS

COMSOL Multiphysics
Chemical Reaction Engineering Module

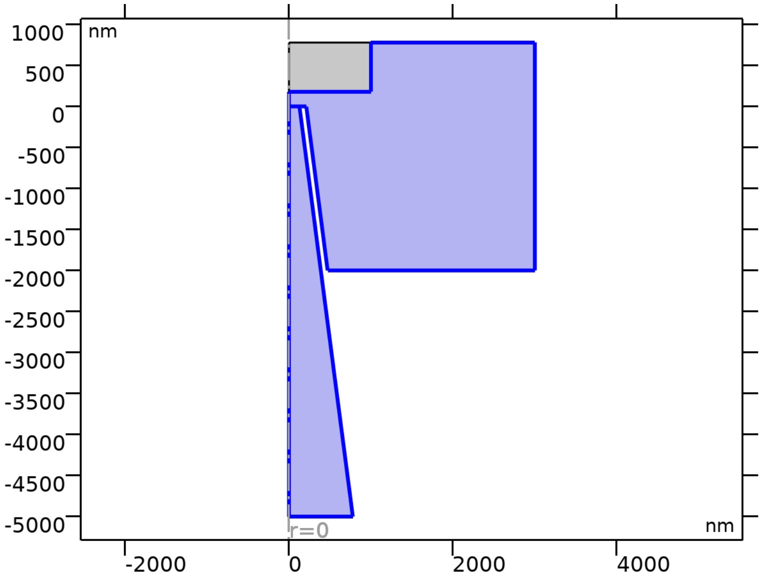

Transport of Diluted Species

SELECTION

Geometric entity level	Domain
Selection	Geometry geom1: Dimension 2: Domains 1–2

EQUATIONS

$$\nabla \cdot \mathbf{J}_i = R_i$$
$$\mathbf{J}_i = -D_i \nabla c_i - z_i u_{m,i} F c_i \nabla V$$

2.6.1 Interface Settings

Discretization

SETTINGS

Description	Value
Concentration	Linear

SETTINGS

Description	Value
Equation form	Study controlled

Species Activity

SETTINGS

Description	Value
Species activity	Ideal

Transport Mechanisms

SETTINGS

Description	Value
Convection	Off
Migration in electric field	On
Mass transfer in porous media	Off

2.6.2 Variables

Name	Expression	Unit	Description	Selection	Details
tds.d	1	1	Out-of-plane geometry extension	Global	
tds.f_K	1	1	Activity coefficient	Domains 1–2	
tds.f_Cl	1	1	Activity coefficient	Domains 1–2	
tds.nr	nr	1	Normal vector, r-component	Boundary 6	

Name	Expression	Unit	Description	Selection	Details
tds.nphi	0	1	Normal vector, phi-component	Boundary 6	
tds.nz	nz	1	Normal vector, z-component	Boundary 6	
tds.nr	unr	1	Normal vector, r-component	Boundaries 8, 16	
tds.nphi	0	1	Normal vector, phi-component	Boundaries 8, 16	
tds.nz	unz	1	Normal vector, z-component	Boundaries 8, 16	
tds.nr	dnr	1	Normal vector, r-component	Boundaries 1–5, 10–15, 17–20	
tds.nphi	0	1	Normal vector, phi-component	Boundaries 1–5, 10–15, 17–20	
tds.nz	dnz	1	Normal vector, z-component	Boundaries 1–5, 10–15, 17–20	
tds.nrmesh	nrmesh	1	Normal vector (mesh), r-component	Boundary 6	
tds.nphimesh	0	1	Normal vector (mesh), phi-component	Boundary 6	
tds.nzmesh	nzmesh	1	Normal vector (mesh), z-component	Boundary 6	
tds.nrmesh	unrmesh	1	Normal vector (mesh), r-component	Boundaries 8, 16	
tds.nphimesh	0	1	Normal vector (mesh), phi-component	Boundaries 8, 16	
tds.nzmesh	unzmesh	1	Normal vector (mesh), z-component	Boundaries 8, 16	
tds.nrmesh	dnrmesh	1	Normal vector (mesh), r-component	Boundaries 1–5, 10–15, 17–20	
tds.nphimesh	0	1	Normal vector (mesh), phi-	Boundaries 1–5, 10–15, 17–	

Name	Expression	Unit	Description	Selection	Details
			component	20	
tds.nzmesh	dnzmesh	1	Normal vector (mesh), z-component	Boundaries 1–5, 10–15, 17–20	
tds.nrc	-root.nrc/tds.ncLen	1	Normal vector, r-component	Boundaries 8, 16	
tds.nphic	0	1	Normal vector, phi-component	Boundaries 8, 16	
tds.nzc	-root.nzc/tds.ncLen	1	Normal vector, z-component	Boundaries 8, 16	
tds.nrc	root.nrc/tds.ncLen	1	Normal vector, r-component	Boundaries 1–6, 10–15, 17–20	
tds.nphic	0	1	Normal vector, phi-component	Boundaries 1–6, 10–15, 17–20	
tds.nzc	root.nzc/tds.ncLen	1	Normal vector, z-component	Boundaries 1–6, 10–15, 17–20	
tds.ncLen	sqrt(root.nrc^2+root.nzc^2+eps)	1	Help variable	Boundaries 1–6, 8, 10–20	
tds.R_K	0	mol/(m³·s)	Total rate expression	Domains 1–2	+ operation
tds.cP_K	0	mol/kg	Concentration species adsorbed to the solid	Domains 1–2	+ operation
tds.cP_K	0	mol/kg	Concentration species adsorbed to the solid	Boundaries 1–6, 8, 10–20	+ operation
tds.KP_K	0	m³/kg	Adsorption isotherm, first concentration derivative	Domains 1–2	+ operation
tds.KP_K	0	m³/kg	Adsorption isotherm, first concentration derivative	Boundaries 1–6, 8, 10–20	+ operation
tds.Rads_K	0	mol/(m³·s)	Total adsorption rate	Domains 1–2	+ operation
tds.DiT_K	0	m²/s	Turbulent diffusivity	Domains 1–2	
tds.cVar_K	K	mol/m³	Species	Boundaries 1–	

Name	Expression	Unit	Description	Selection	Details
				6, 8, 10–20	
tds.cVar_K	K	mol/m³	Species	Points 1–5, 7–18	
tds.R_Cl	0	mol/(m³·s)	Total rate expression	Domains 1–2	+ operation
tds.cP_Cl	0	mol/kg	Concentration species adsorbed to the solid	Domains 1–2	+ operation
tds.cP_Cl	0	mol/kg	Concentration species adsorbed to the solid	Boundaries 1–6, 8, 10–20	+ operation
tds.KP_Cl	0	m³/kg	Adsorption isotherm, first concentration derivative	Domains 1–2	+ operation
tds.KP_Cl	0	m³/kg	Adsorption isotherm, first concentration derivative	Boundaries 1–6, 8, 10–20	+ operation
tds.Rads_Cl	0	mol/(m³·s)	Total adsorption rate	Domains 1–2	+ operation
tds.DiT_Cl	0	m²/s	Turbulent diffusivity	Domains 1–2	
tds.cVar_Cl	Cl	mol/m³	Species	Boundaries 1–6, 8, 10–20	
tds.cVar_Cl	Cl	mol/m³	Species	Points 1–5, 7–18	
tds.poro	1	1	Porosity	Domains 1–2	
tds.theta_g	0	1	Gas volume fraction	Domains 1–2	
tds.theta_l	1	1	Liquid volume fraction	Domains 1–2	
tds.theta	tds.poro	1	Mobile fluid volume fraction	Domains 1–2	

2.6.3 Species Charges

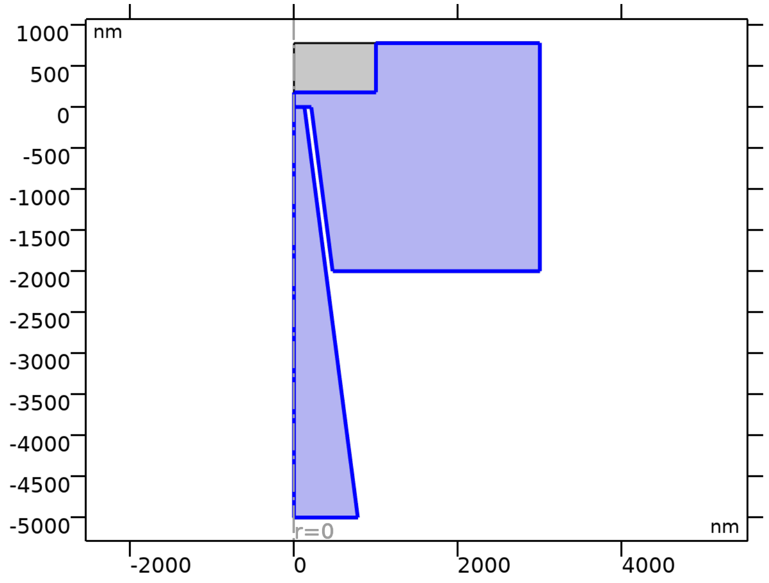

Species Charges

SELECTION

Geometric entity level	Domain
Selection	Geometry geom1: Dimension 2: All domains

Charge

SETTINGS

Description	Value
Charge number	{1, -1}

Model Input

SETTINGS

Description	Value
Temperature	Common model input

Variables

Name	Expression	Unit	Description	Selection
tds.z_K	1	1	Charge number	Domains 1–2
tds.z_Cl	-1	1	Charge number	Domains 1–2

2.6.4 Transport Properties 1

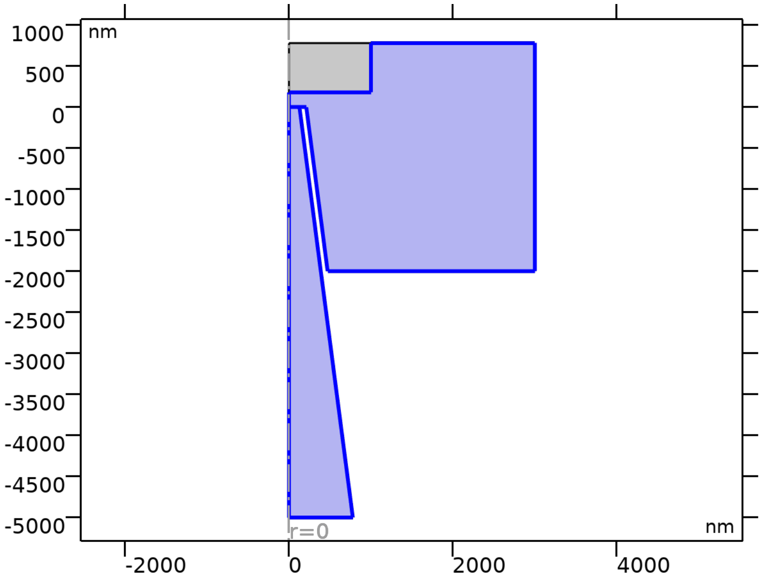

Transport Properties 1

SELECTION

Geometric entity level	Domain
Selection	Geometry geom1: Dimension 2: All domains

EQUATIONS

$$\nabla \cdot \mathbf{J}_i = R_i$$
$$\mathbf{J}_i = -D_i \nabla c_i - z_i u_{m,i} F c_i \nabla V$$

Diffusion

SETTINGS

**Description**	**Value**	**Unit**
Source	Material	
Material	water (mat1)	
Diffusion coefficient	User defined	
Diffusion coefficient	1.957E-9	m^2/s
Diffusion coefficient	User defined	
Diffusion coefficient	2.032E-9	m^2/s

Migration in Electric Field

SETTINGS

**Description**	**Value**
Mobility	Nernst - Einstein relation

Coordinate System Selection

SETTINGS

Description	Value
Coordinate system	Global coordinate system

Model Input

SETTINGS

Description	Value	Unit
Temperature	User defined	
Temperature	293.15	K

Variables

Name	Expression	Unit	Description	Selection	Details
domflux.Kr	2*(tds.dflux_Kr+tds.mflux_Kr)*pi*r*tds.d	mol/(m·s)	Domain flux, r-component	Domains 1–2	
domflux.Kz	2*(tds.dflux_Kz+tds.mflux_Kz)*pi*r*tds.d	mol/(m·s)	Domain flux, z-component	Domains 1–2	
domflux.Clr	2*(tds.dflux_Clr+tds.mflux_Clr)*pi*r*tds.d	mol/(m·s)	Domain flux, r-component	Domains 1–2	
domflux.Clz	2*(tds.dflux_Clz+tds.mflux_Clz)*pi*r*tds.d	mol/(m·s)	Domain flux, z-component	Domains 1–2	
tds.ndflux_K	tds.dflux_Kr*tds.nrc+tds.dflux_Kphi*tds.nphic+tds.dflux_Kz*tds.nzc	mol/(m²·s)	Normal diffusive flux	Boundaries 2, 6, 8, 10–20	
tds.nmflux_K	tds.mflux_Kr*tds.nrc+tds.mflux_Kphi*tds.nphic+tds.mflux_Kz*tds.nzc	mol/(m²·s)	Normal electrophoretic flux	Boundaries 2, 6, 8, 10–20	
tds.ntflux_K	tds.bndFlux_K	mol/(m²·s)	Normal total flux	Boundaries 2, 6, 8, 10–20	
tds.ndflux_Cl	tds.dflux_Clr*tds.nrc+tds.dflux_Clphi*tds.nphic+tds.dflux_Clz*tds.nzc	mol/(m²·s)	Normal diffusive flux	Boundaries 2, 6, 8, 10–20	
tds.nmflux_Cl	tds.mflux_Clr*tds.nrc+tds.mflux_Clphi*tds.nphic+tds.mflux_Clz*tds.nzc	mol/(m²·s)	Normal electrophoretic flux	Boundaries 2, 6, 8, 10–20	
tds.ntflux_Cl	tds.bndFlux_Cl	mol/(m²·s)	Normal total flux	Boundaries 2,	

Name	Expression	Unit	Description	Selection	Details
				6, 8, 10–20	
tds.bndFlux_K	0.25*(uflux_spatial(K)-dflux_spatial(K))/(pi*r*tds.d)	mol/(m²·s)	Boundary flux	Boundary 6	Meta
tds.bndFlux_K	-0.5*uflux_spatial(K)/(pi*r*tds.d)	mol/(m²·s)	Boundary flux	Boundaries 8, 16	Meta
tds.bndFlux_K	-dflux_spatial(K)/tds.d	mol/(m²·s)	Boundary flux	Boundaries 1, 3–5	
tds.bndFlux_K	-0.5*dflux_spatial(K)/(pi*r*tds.d)	mol/(m²·s)	Boundary flux	Boundaries 2, 10–15, 17–20	Meta
tds.bndFlux_Cl	0.25*(uflux_spatial(Cl)-dflux_spatial(Cl))/(pi*r*tds.d)	mol/(m²·s)	Boundary flux	Boundary 6	Meta
tds.bndFlux_Cl	-0.5*uflux_spatial(Cl)/(pi*r*tds.d)	mol/(m²·s)	Boundary flux	Boundaries 8, 16	Meta
tds.bndFlux_Cl	-dflux_spatial(Cl)/tds.d	mol/(m²·s)	Boundary flux	Boundaries 1, 3–5	
tds.bndFlux_Cl	-0.5*dflux_spatial(Cl)/(pi*r*tds.d)	mol/(m²·s)	Boundary flux	Boundaries 2, 10–15, 17–20	Meta
tds.DF_Krr	1.957E-9[m^2/s]	m²/s	Fluid diffusion coefficient, rr-component	Domains 1–2	
tds.DF_Kphir	0	m²/s	Fluid diffusion coefficient, phir-component	Domains 1–2	
tds.DF_Kzr	0	m²/s	Fluid diffusion coefficient, zr-component	Domains 1–2	
tds.DF_Krphi	0	m²/s	Fluid diffusion coefficient, rphi-component	Domains 1–2	
tds.DF_Kphiphi	1.957E-9[m^2/s]	m²/s	Fluid diffusion coefficient, phiphi-component	Domains 1–2	

Name	Expression	Unit	Description	Selection	Details
tds.DF_Kzphi	0	m²/s	Fluid diffusion coefficient, zphi-component	Domains 1–2	
tds.DF_Krz	0	m²/s	Fluid diffusion coefficient, rz-component	Domains 1–2	
tds.DF_Kphiz	0	m²/s	Fluid diffusion coefficient, phiz-component	Domains 1–2	
tds.DF_Kzz	1.957E-9[m^2/s]	m²/s	Fluid diffusion coefficient, zz-component	Domains 1–2	
tds.D_Krr	tds.DF_Krr+tds.DiT_K	m²/s	Diffusion coefficient, rr-component	Domains 1–2	+ operation
tds.D_Kphir	tds.DF_Kphir	m²/s	Diffusion coefficient, phir-component	Domains 1–2	+ operation
tds.D_Kzr	tds.DF_Kzr	m²/s	Diffusion coefficient, zr-component	Domains 1–2	+ operation
tds.D_Krphi	tds.DF_Krphi	m²/s	Diffusion coefficient, rphi-component	Domains 1–2	+ operation
tds.D_Kphiphi	tds.DF_Kphiphi+tds.DiT_K	m²/s	Diffusion coefficient, phiphi-component	Domains 1–2	+ operation
tds.D_Kzphi	tds.DF_Kzphi	m²/s	Diffusion coefficient, zphi-component	Domains 1–2	+ operation
tds.D_Krz	tds.DF_Krz	m²/s	Diffusion coefficient, rz-component	Domains 1–2	+ operation
tds.D_Kphiz	tds.DF_Kphiz	m²/s	Diffusion coefficient, phiz-component	Domains 1–2	+ operation
tds.D_Kzz	tds.DF_Kzz+tds.DiT_K	m²/s	Diffusion coefficient, zz-component	Domains 1–2	+ operation
tds.DF_Clrr	2.032E-9[m^2/s]	m²/s	Fluid diffusion coefficient, rr-component	Domains 1–2	

Name	Expression	Unit	Description	Selection	Details
tds.DF_Clphir	0	m²/s	Fluid diffusion coefficient, phir-component	Domains 1–2	
tds.DF_Clzr	0	m²/s	Fluid diffusion coefficient, zr-component	Domains 1–2	
tds.DF_Clrphi	0	m²/s	Fluid diffusion coefficient, rphi-component	Domains 1–2	
tds.DF_Clphiphi	2.032E-9[m^2/s]	m²/s	Fluid diffusion coefficient, phiphi-component	Domains 1–2	
tds.DF_Clzphi	0	m²/s	Fluid diffusion coefficient, zphi-component	Domains 1–2	
tds.DF_Clrz	0	m²/s	Fluid diffusion coefficient, rz-component	Domains 1–2	
tds.DF_Clphiz	0	m²/s	Fluid diffusion coefficient, phiz-component	Domains 1–2	
tds.DF_Clzz	2.032E-9[m^2/s]	m²/s	Fluid diffusion coefficient, zz-component	Domains 1–2	
tds.D_Clrr	tds.DF_Clrr+tds.DiT_Cl	m²/s	Diffusion coefficient, rr-component	Domains 1–2	+ operation
tds.D_Clphir	tds.DF_Clphir	m²/s	Diffusion coefficient, phir-component	Domains 1–2	+ operation
tds.D_Clzr	tds.DF_Clzr	m²/s	Diffusion coefficient, zr-component	Domains 1–2	+ operation
tds.D_Clrphi	tds.DF_Clrphi	m²/s	Diffusion coefficient, rphi-component	Domains 1–2	+ operation
tds.D_Clphiphi	tds.DF_Clphiphi+tds.DiT_Cl	m²/s	Diffusion coefficient, phiphi-component	Domains 1–2	+ operation
tds.D_Clzphi	tds.DF_Clzphi	m²/s	Diffusion coefficient, zphi-	Domains 1–2	+ operation

Name	Expression	Unit	Description	Selection	Details
			component		
tds.D_Clrz	tds.DF_Clrz	m²/s	Diffusion coefficient, rz-component	Domains 1–2	+ operation
tds.D_Clphiz	tds.DF_Clphiz	m²/s	Diffusion coefficient, phiz-component	Domains 1–2	+ operation
tds.D_Clzz	tds.DF_Clzz+tds.DiT_Cl	m²/s	Diffusion coefficient, zz-component	Domains 1–2	+ operation
tds.Dav_K	0.5*(tds.D_Krr+tds.D_Kzz)	m²/s	Average diffusion coefficient	Domains 1–2	
tds.Dav_Cl	0.5*(tds.D_Clrr+tds.D_Clzz)	m²/s	Average diffusion coefficient	Domains 1–2	
tds.tflux_Kr	tds.dflux_Kr+tds.mflux_Kr	mol/(m²·s)	Total flux, r-component	Domains 1–2	+ operation
tds.tflux_Kphi	tds.dflux_Kphi+tds.mflux_Kphi	mol/(m²·s)	Total flux, phi-component	Domains 1–2	+ operation
tds.tflux_Kz	tds.dflux_Kz+tds.mflux_Kz	mol/(m²·s)	Total flux, z-component	Domains 1–2	+ operation
tds.dfluxMag_K	sqrt(tds.dflux_Kr^2+tds.dflux_Kphi^2+tds.dflux_Kz^2)	mol/(m²·s)	Diffusive flux magnitude	Domains 1–2	
tds.tfluxMag_K	sqrt(tds.tflux_Kr^2+tds.tflux_Kphi^2+tds.tflux_Kz^2)	mol/(m²·s)	Total flux magnitude	Domains 1–2	
tds.dpflux_Kr	0	mol/(m²·s)	Dispersive flux, r-component	Domains 1–2	
tds.dpflux_Kphi	0	mol/(m²·s)	Dispersive flux, phi-component	Domains 1–2	
tds.dpflux_Kz	0	mol/(m²·s)	Dispersive flux, z-component	Domains 1–2	
tds.mflux_Kr	tds.z_K*F_const*K*(-tds.um_Krr*d(tds.V,r)-tds.um_Krz*d(tds.V,z))	mol/(m²·s)	Electrophoretic flux, r-component	Domains 1–2	
tds.mflux_Kphi	tds.z_K*F_const*K*(-tds.um_Kphir*d(tds.V,r)-	mol/(m²·s)	Electrophoretic flux, phi-component	Domains 1–2	

Name	Expression	Unit	Description	Selection	Details
	tds.um_Kphiz*d(tds.V,z))				
tds.mflux_Kz	tds.z_K*F_const*K*(-tds.um_Kzr*d(tds.V,r)-tds.um_Kzz*d(tds.V,z))	mol/(m²·s)	Electrophoretic flux, z-component	Domains 1–2	
tds.mfluxMag_K	sqrt(tds.mflux_Kr^2+tds.mflux_Kphi^2+tds.mflux_Kz^2)	mol/(m²·s)	Electrophoretic flux magnitude	Domains 1–2	
tds.tflux_Clr	tds.dflux_Clr+tds.mflux_Clr	mol/(m²·s)	Total flux, r-component	Domains 1–2	+ operation
tds.tflux_Clphi	tds.dflux_Clphi+tds.mflux_Clphi	mol/(m²·s)	Total flux, phi-component	Domains 1–2	+ operation
tds.tflux_Clz	tds.dflux_Clz+tds.mflux_Clz	mol/(m²·s)	Total flux, z-component	Domains 1–2	+ operation
tds.dfluxMag_Cl	sqrt(tds.dflux_Clr^2+tds.dflux_Clphi^2+tds.dflux_Clz^2)	mol/(m²·s)	Diffusive flux magnitude	Domains 1–2	
tds.tfluxMag_Cl	sqrt(tds.tflux_Clr^2+tds.tflux_Clphi^2+tds.tflux_Clz^2)	mol/(m²·s)	Total flux magnitude	Domains 1–2	
tds.dpflux_Clr	0	mol/(m²·s)	Dispersive flux, r-component	Domains 1–2	
tds.dpflux_Clphi	0	mol/(m²·s)	Dispersive flux, phi-component	Domains 1–2	
tds.dpflux_Clz	0	mol/(m²·s)	Dispersive flux, z-component	Domains 1–2	
tds.mflux_Clr	tds.z_Cl*F_const*Cl*(-tds.um_Clrr*d(tds.V,r)-tds.um_Clrz*d(tds.V,z))	mol/(m²·s)	Electrophoretic flux, r-component	Domains 1–2	
tds.mflux_Clphi	tds.z_Cl*F_const*Cl*(-tds.um_Clphir*d(tds.V,r)-tds.um_Clphiz*d(tds.V,z))	mol/(m²·s)	Electrophoretic flux, phi-component	Domains 1–2	
tds.mflux_Clz	tds.z_Cl*F_const*Cl*(-	mol/(m²·s)	Electrophoretic flux, z-component	Domains 1–2	

Name	Expression	Unit	Description	Selection	Details
	tds.um_Clzr*d(tds.V,r)-tds.um_Clzz*d(tds.V,z))				
tds.mfluxMag_Cl	sqrt(tds.mflux_Clr^2+tds.mflux_Clphi^2+tds.mflux_Clz^2)	mol/(m²·s)	Electrophoretic flux magnitude	Domains 1–2	
Krt	KrTIME-Krr*d(r,TIME)-Krz*d(z,TIME)	mol/(m⁴·s)	Gradient of K, r component, first time derivative	Domains 1–2	
Kzt	KzTIME-Kzr*d(r,TIME)-Kzz*d(z,TIME)	mol/(m⁴·s)	Gradient of K, z component, first time derivative	Domains 1–2	
Krtt	d(KrTIME-Krr*d(r,TIME)-Krz*d(z,TIME),TIME)-d(KrTIME-Krr*d(r,TIME)-Krz*d(z,TIME),r)*d(r,TIME)-d(KrTIME-Krr*d(r,TIME)-Krz*d(z,TIME),z)*d(z,TIME)	mol/(m⁴·s²)	Gradient of K, r component, second time derivative	Domains 1–2	
Kztt	d(KzTIME-Kzr*d(r,TIME)-Kzz*d(z,TIME),TIME)-d(KzTIME-Kzr*d(r,TIME)-Kzz*d(z,TIME),r)*d(r,TIME)-d(KzTIME-Kzr*d(r,TIME)-Kzz*d(z,TIME),z)*d(z,TIME)	mol/(m⁴·s²)	Gradient of K, z component, second time derivative	Domains 1–2	
Kt	KTIME-Kr*d(r,TIME)-Kz*d(z,TIME)	mol/(m³·s)	Concentration, first time derivative	Domains 1–2	
Ktt	d(KTIME-Kr*d(r,TIME)-Kz*d(z,TIME),TIME)-d(KTIME-Kr*d(r,TIME)-Kz*d(z,TIME),r)*d(r,TIME)-d(KTIME-Kr*d(r,TIME)-Kz*d(z,TIME),z)*d(z,	mol/(m³·s²)	Concentration, second time derivative	Domains 1–2	

Name	Expression	Unit	Description	Selection	Details
	TIME)				
tds.dflux_Kr	-tds.D_Krr*Kr-tds.D_Krz*Kz	mol/(m²·s)	Diffusive flux, r-component	Domains 1–2	+ operation
tds.dflux_Kphi	-tds.D_Kphir*Kr-tds.D_Kphiz*Kz	mol/(m²·s)	Diffusive flux, phi-component	Domains 1–2	+ operation
tds.dflux_Kz	-tds.D_Kzr*Kr-tds.D_Kzz*Kz	mol/(m²·s)	Diffusive flux, z-component	Domains 1–2	+ operation
tds.grad_Kr	Kr	mol/m⁴	Concentration gradient, r-component	Domains 1–2	
tds.grad_Kphi	0	mol/m⁴	Concentration gradient, phi-component	Domains 1–2	
tds.grad_Kz	Kz	mol/m⁴	Concentration gradient, z-component	Domains 1–2	
Clrt	ClrTIME-Clrr*d(r,TIME)-Clrz*d(z,TIME)	mol/(m⁴·s)	Gradient of Cl, r component, first time derivative	Domains 1–2	
Clzt	ClzTIME-Clzr*d(r,TIME)-Clzz*d(z,TIME)	mol/(m⁴·s)	Gradient of Cl, z component, first time derivative	Domains 1–2	
Clrtt	d(ClrTIME-Clrr*d(r,TIME)-Clrz*d(z,TIME),TIME)-d(ClrTIME-Clrr*d(r,TIME)-Clrz*d(z,TIME),r)*d(r,TIME)-d(ClrTIME-Clrr*d(r,TIME)-Clrz*d(z,TIME),z)*d(z,TIME)	mol/(m⁴·s²)	Gradient of Cl, r component, second time derivative	Domains 1–2	
Clztt	d(ClzTIME-Clzr*d(r,TIME)-Clzz*d(z,TIME),TIME)-d(ClzTIME-Clzr*d(r,TIME)-Clzz*d(z,TIME),r)*d(r,TIME)-d(ClzTIME-Clzr*d(r,TIME)-Clzz*d(z,TIME),z)*d(z,TIME)	mol/(m⁴·s²)	Gradient of Cl, z component, second time derivative	Domains 1–2	
Clt	ClTIME-Clr*d(r,TIME)-	mol/(m³·s)	Concentration, first time	Domains 1–2	

Name	Expression	Unit	Description	Selection	Details
	Clz*d(z,TIME)		derivative		
Cltt	d(ClTIME-Clr*d(r,TIME)-Clz*d(z,TIME),TIME)-d(ClTIME-Clr*d(r,TIME)-Clz*d(z,TIME),r)*d(r,TIME)-d(ClTIME-Clr*d(r,TIME)-Clz*d(z,TIME),z)*d(z,TIME)	mol/(m³·s²)	Concentration, second time derivative	Domains 1–2	
tds.dflux_Clr	-tds.D_Clrr*Clr-tds.D_Clrz*Clz	mol/(m²·s)	Diffusive flux, r-component	Domains 1–2	+ operation
tds.dflux_Clphi	-tds.D_Clphir*Clr-tds.D_Clphiz*Clz	mol/(m²·s)	Diffusive flux, phi-component	Domains 1–2	+ operation
tds.dflux_Clz	-tds.D_Clzr*Clr-tds.D_Clzz*Clz	mol/(m²·s)	Diffusive flux, z-component	Domains 1–2	+ operation
tds.grad_Clr	Clr	mol/m⁴	Concentration gradient, r-component	Domains 1–2	
tds.grad_Clphi	0	mol/m⁴	Concentration gradient, phi-component	Domains 1–2	
tds.grad_Clz	Clz	mol/m⁴	Concentration gradient, z-component	Domains 1–2	
tds.um_Krr	tds.D_Krr/(R_const*tds.T)	s·mol/kg	Mobility, rr-component	Domains 1–2	
tds.um_Kphir	tds.D_Kphir/(R_const*tds.T)	s·mol/kg	Mobility, phir-component	Domains 1–2	
tds.um_Kzr	tds.D_Kzr/(R_const*tds.T)	s·mol/kg	Mobility, zr-component	Domains 1–2	
tds.um_Krphi	tds.D_Krphi/(R_const*tds.T)	s·mol/kg	Mobility, rphi-component	Domains 1–2	
tds.um_Kphiphi	tds.D_Kphiphi/(R_const*tds.T)	s·mol/kg	Mobility, phiphi-component	Domains 1–2	
tds.um_Kzphi	tds.D_Kzphi/(R_const*tds.T)	s·mol/kg	Mobility, zphi-component	Domains 1–2	
tds.um_Krz	tds.D_Krz/(R_const*tds.T)	s·mol/kg	Mobility, rz-component	Domains 1–2	
tds.um_Kphiz	tds.D_Kphiz/(R_const*tds.T)	s·mol/kg	Mobility, phiz-component	Domains 1–2	

Name	Expression	Unit	Description	Selection	Details
tds.um_Kzz	tds.D_Kzz/(R_const*tds.T)	s·mol/kg	Mobility, zz-component	Domains 1–2	
tds.um_Clrr	tds.D_Clrr/(R_const*tds.T)	s·mol/kg	Mobility, rr-component	Domains 1–2	
tds.um_Clphir	tds.D_Clphir/(R_const*tds.T)	s·mol/kg	Mobility, phir-component	Domains 1–2	
tds.um_Clzr	tds.D_Clzr/(R_const*tds.T)	s·mol/kg	Mobility, zr-component	Domains 1–2	
tds.um_Clrphi	tds.D_Clrphi/(R_const*tds.T)	s·mol/kg	Mobility, rphi-component	Domains 1–2	
tds.um_Clphiphi	tds.D_Clphiphi/(R_const*tds.T)	s·mol/kg	Mobility, phiphi-component	Domains 1–2	
tds.um_Clzphi	tds.D_Clzphi/(R_const*tds.T)	s·mol/kg	Mobility, zphi-component	Domains 1–2	
tds.um_Clrz	tds.D_Clrz/(R_const*tds.T)	s·mol/kg	Mobility, rz-component	Domains 1–2	
tds.um_Clphiz	tds.D_Clphiz/(R_const*tds.T)	s·mol/kg	Mobility, phiz-component	Domains 1–2	
tds.um_Clzz	tds.D_Clzz/(R_const*tds.T)	s·mol/kg	Mobility, zz-component	Domains 1–2	
tds.V	model.input.V	V	Electric potential	Domains 1–2	Meta
tds.T	tds.cdm1.minput_temperature	K	Temperature	Domains 1–2	
tds.Rlin_K	0	1/s	Linear source term coefficient	Domains 1–2	+ operation
tds.Res_K	d(K*tds.z_K*F_const*(-tds.um_Krr*d(tds.V,r)-tds.um_Krz*d(tds.V,z)),r)+if(abs(r)<0.001*h_spatial,d(K*tds.z_K*F_const*(-tds.um_Krr*d(tds.V,r)-tds.um_Krz*d(tds.V,z)),r),K*tds.z_K*F_const*(-tds.um_Krr*d(tds.V,r)-tds.um_Krz*d(tds.V,z))/r)+d(K*tds.z_K*F_const*(-	mol/(m³·s)	Equation residual	Domains 1–2	

Name	Expression	Unit	Description	Selection	Details
	tds.um_Kzr*d(tds.V, r)- tds.um_Kzz*d(tds.V, z)),z)-K*tds.Rlin_K- tds.R_K				
tds.Rlin_Cl	0	1/s	Linear source term coefficient	Domains 1–2	+ operation
tds.Res_Cl	d(Cl*tds.z_Cl*F_const*(- tds.um_Clrr*d(tds.V, r)- tds.um_Clrz*d(tds.V, z)),r)+if(abs(r)<0.001*h_spatial,d(Cl*tds.z_Cl*F_const*(- tds.um_Clrr*d(tds.V, r)- tds.um_Clrz*d(tds.V, z)),r),Cl*tds.z_Cl*F_const*(- tds.um_Clrr*d(tds.V, r)- tds.um_Clrz*d(tds.V, z))/r)+d(Cl*tds.z_Cl*F_const*(- tds.um_Clzr*d(tds.V, r)- tds.um_Clzz*d(tds.V,z)),z)- Cl*tds.Rlin_Cl- tds.R_Cl	mol/(m³·s)	Equation residual	Domains 1–2	

Shape functions

Name	Shape function	Unit	Description	Shape frame	Selection
K	Lagrange (Linear)	mol/m³	Concentration	Spatial	Domains 1–2
Cl	Lagrange (Linear)	mol/m³	Concentration	Spatial	Domains 1–2

Weak Expressions

Weak expression	Integration order	Integration frame	Selection
2*(tds.dflux_Kr*test(Kr)+tds.dflux_Kz*test(Kz))*tds.d*pi*r	2	Spatial	Domains 1–2
2*(tds.dflux_Clr*test(Clr)+tds.dflux_Clz*test(Clz))*tds.d*pi*r	2	Spatial	Domains 1–2
2*tds.z_K*F_const*K*((-	2	Spatial	Domains 1–2

Weak expression	Integration order	Integration frame	Selection
tds.um_Krr*d(tds.V,r)-tds.um_Krz*d(tds.V,z))*test(Kr)+(-tds.um_Kzr*d(tds.V,r)-tds.um_Kzz*d(tds.V,z))*test(Kz))*tds.d*pi*r			
2*tds.z_Cl*F_const*Cl*((-tds.um_Clrr*d(tds.V,r)-tds.um_Clrz*d(tds.V,z))*test(Clr)+(-tds.um_Clzr*d(tds.V,r)-tds.um_Clzz*d(tds.V,z))*test(Clz))*tds.d*pi*r	2	Spatial	Domains 1–2
2*tds.streamline*(isScalingSystemDomain==0)*tds.d*pi*r	2	Spatial	Domains 1–2
2*tds.crosswind*(isScalingSystemDomain==0)*tds.d*pi*r	4	Spatial	Domains 1–2

2.6.5 Axial Symmetry 1

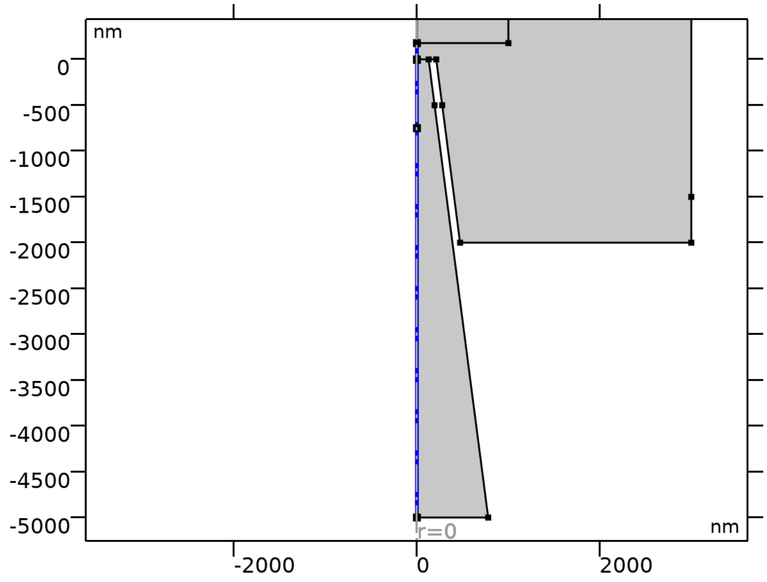

Axial Symmetry 1

SELECTION

Geometric entity level	Boundary
Selection	Geometry geom1: Dimension 1: All boundaries

2.6.6 No Flux 1

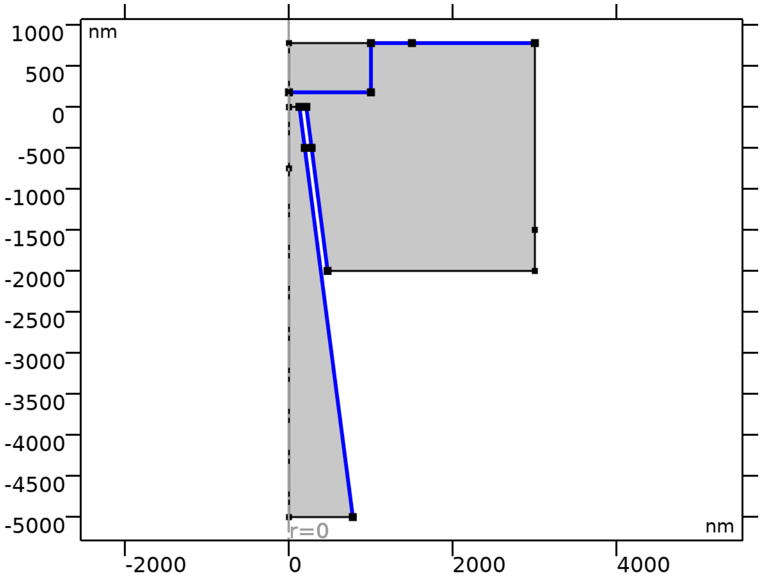

No Flux 1

SELECTION

Geometric entity level	Boundary
Selection	Geometry geom1: Dimension 1: All boundaries

EQUATIONS

$$-\mathbf{n}\cdot\mathbf{J}_i = 0$$

2.6.7 Initial Values 1

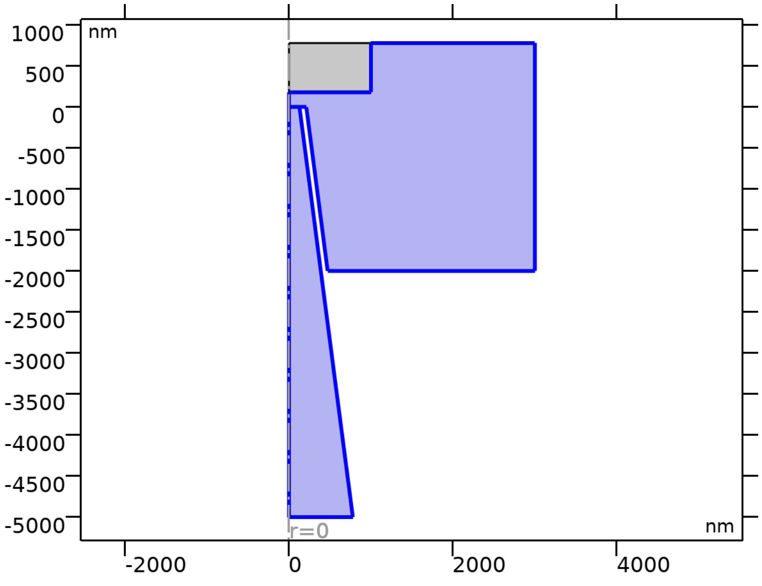

Initial Values 1

SELECTION

Geometric entity level	Domain
Selection	Geometry geom1: Dimension 2: All domains

Initial Values

SETTINGS

Description	Value	Unit
Concentration	{c, c}	mol/m^3

Variables

Name	Expression	Unit	Description	Selection	Details
tds.c0_K	c	mol/m^3	Concentration	Domains 1–2	+ operation
tds.c0_Cl	c	mol/m^3	Concentration	Domains 1–2	+ operation

2.6.8 Concentration 1

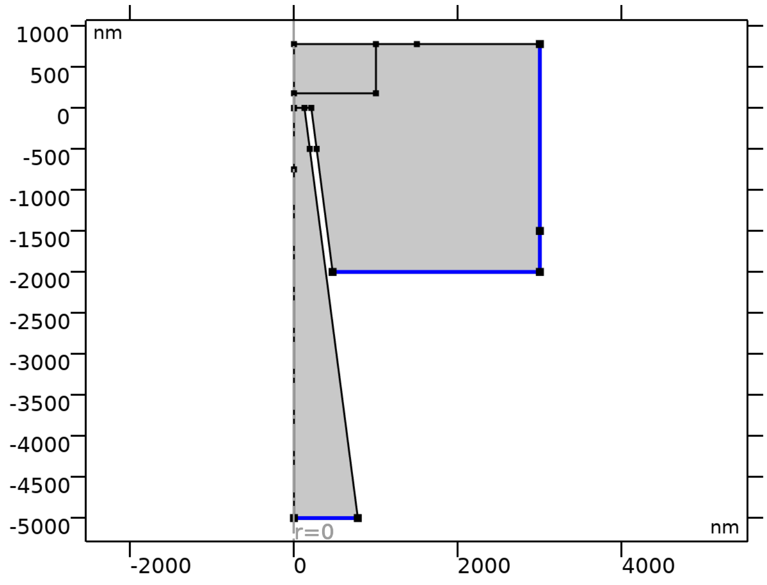

Concentration 1

SELECTION

Geometric entity level	Boundary
Selection	Geometry geom1: Dimension 1: Boundaries 2, 15, 19–20

EQUATIONS

$$c_i = c_{0,i}$$

Concentration

SETTINGS

Description	Value	Unit
Species K	On	
Species Cl	On	
Concentration	{c, c}	mol/m³

Variables

Name	Expression	Unit	Description	Selection	Details
tds.c0_K	c	mol/m³	Concentration	Boundaries 2, 15, 19–20	+ operation
tds.c0_Cl	c	mol/m³	Concentration	Boundaries 2, 15, 19–20	+ operation
tds.conc1.nmflow_K	tds.conc1.int(2*tds.ntflux_K*pi*r)*tds.d	mol/s	Normal molar flow rate	Global	
tds.conc1.nmflow_Cl	tds.conc1.int(2*tds.ntflux_Cl*pi*r)*tds.d	mol/s	Normal molar flow rate	Global	

Constraints

Constraint	Constraint force	Shape function	Selection	Details
-tds.cVar_K+tds.c0_K	test(-tds.cVar_K+tds.c0_K)	Lagrange (Linear)	Boundaries 2, 15, 19–20	Elemental
-tds.cVar_Cl+tds.c0_Cl	test(-tds.cVar_Cl+tds.c0_Cl)	Lagrange (Linear)	Boundaries 2, 15, 19–20	Elemental

2.7 ELECTROSTATICS

USED PRODUCTS

COMSOL Multiphysics

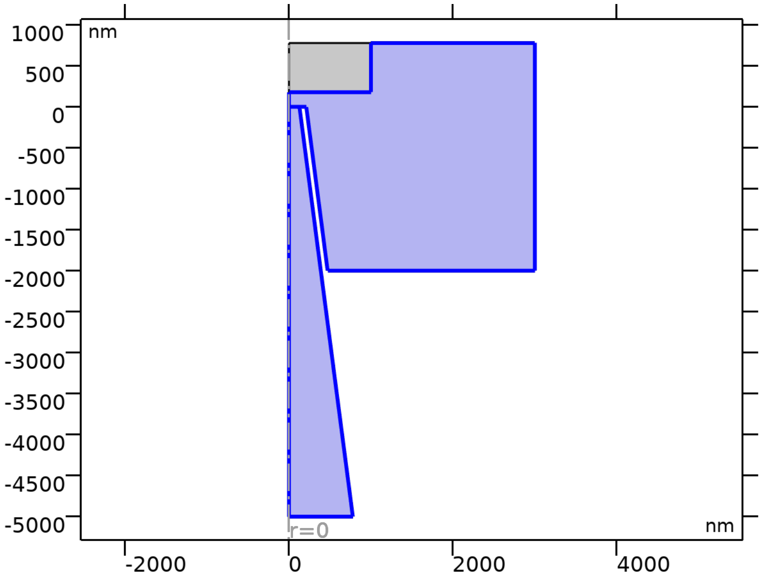

Electrostatics

SELECTION

Geometric entity level	Domain
Selection	Geometry geom1: Dimension 2: Domains 1–2

EQUATIONS

$$\nabla \cdot \mathbf{D} = \rho_v$$

$$\mathbf{E} = -\nabla V$$

2.7.1 Interface Settings

Discretization

SETTINGS

Description	Value
Electric potential	Quadratic

Manual Terminal Sweep Settings

SETTINGS

Description	Value	Unit
Use manual terminal sweep	Off	
Reference impedance	50	Ω

2.7.2 Variables

Name	Expression	Unit	Description	Selection	Details
es.d	1	1	Contribution	Domains 1–2	

Name	Expression	Unit	Description	Selection	Details
es.nr	nr		Normal vector, r-component	Boundary 6	
es.nphi	0		Normal vector, phi-component	Boundary 6	
es.nz	nz		Normal vector, z-component	Boundary 6	
es.nr	unr		Normal vector, r-component	Boundaries 8, 16	
es.nphi	0		Normal vector, phi-component	Boundaries 8, 16	
es.nz	unz		Normal vector, z-component	Boundaries 8, 16	
es.nr	dnr		Normal vector, r-component	Boundaries 1–5, 10–15, 17–20	
es.nphi	0		Normal vector, phi-component	Boundaries 1–5, 10–15, 17–20	
es.nz	dnz		Normal vector, z-component	Boundaries 1–5, 10–15, 17–20	
es.nmeshr	nrmesh		Mesh normal vector, r-component	Boundary 6	
es.nmeshphi	0		Mesh normal vector, phi-component	Boundary 6	
es.nmeshz	nzmesh		Mesh normal vector, z-component	Boundary 6	
es.nmeshr	unrmesh		Mesh normal vector, r-component	Boundaries 8, 16	
es.nmeshphi	0		Mesh normal vector, phi-component	Boundaries 8, 16	
es.nmeshz	unzmesh		Mesh normal vector, z-component	Boundaries 8, 16	
es.nmeshr	dnrmesh		Mesh normal vector, r-component	Boundaries 1–5, 10–15, 17–20	

Name	Expression	Unit	Description	Selection	Details
es.nmeshphi	0		Mesh normal vector, phi-component	Boundaries 1–5, 10–15, 17–20	
es.nmeshz	dnzmesh		Mesh normal vector, z-component	Boundaries 1–5, 10–15, 17–20	
es.unmeshr	unrmesh		Mesh normal vector, upside, r-component	Boundaries 1–6, 8, 10–20	
es.unmeshphi	0		Mesh normal vector, upside, phi-component	Boundaries 1–6, 8, 10–20	
es.unmeshz	unzmesh		Mesh normal vector, upside, z-component	Boundaries 1–6, 8, 10–20	
es.dnmeshr	dnrmesh		Mesh normal vector, downside, r-component	Boundaries 1–6, 8, 10–20	
es.dnmeshphi	0		Mesh normal vector, downside, phi-component	Boundaries 1–6, 8, 10–20	
es.dnmeshz	dnzmesh		Mesh normal vector, downside, z-component	Boundaries 1–6, 8, 10–20	
es.I_sRR	(spatial.invF11*(spatial.invF11*es.I_srr+spatial.invF31*es.I_szr)+spatial.invF31*(spatial.invF11*es.I_srz+spatial.invF31*es.I_szz))*spatial.detF	1	Spatial identity matrix, material frame, RR-component	Domains 1–2	
es.I_sPHIR	if(Rg>0.001*h,R/r,Rr)*(spatial.invF11*es.I_sphir+spatial.invF31*es.I_sphiz)*spatial.detF	1	Spatial identity matrix, material frame, PHIR-component	Domains 1–2	
es.I_sZR	(spatial.invF11*(spatial.invF13*es.I_srr+spatial.invF33*es.I_szr)+spatial.invF31*(spatial.invF13*es.I_srz+spatial.invF33*es.I_szz))*spatial.d	1	Spatial identity matrix, material frame, ZR-component	Domains 1–2	

Name	Expression	Unit	Description	Selection	Details
	etF				
es.I_sRPHI	if(Rg>0.001*h,R/r,Rr)*(spatial.invF11*es.I_srphi+spatial.invF31*es.I_szphi)*spatial.detF	1	Spatial identity matrix, material frame, RPHI-component	Domains 1–2	
es.I_sPHIPHI	if(Rg>0.001*h,R/r,Rr)^2*es.I_sphiphi*spatial.detF	1	Spatial identity matrix, material frame, PHIPHI-component	Domains 1–2	
es.I_sZPHI	if(Rg>0.001*h,R/r,Rr)*(spatial.invF13*es.I_srphi+spatial.invF33*es.I_szphi)*spatial.detF	1	Spatial identity matrix, material frame, ZPHI-component	Domains 1–2	
es.I_sRZ	(spatial.invF13*(spatial.invF11*es.I_srr+spatial.invF31*es.I_szr)+spatial.invF33*(spatial.invF11*es.I_srz+spatial.invF31*es.I_szz))*spatial.detF	1	Spatial identity matrix, material frame, RZ-component	Domains 1–2	
es.I_sPHIZ	if(Rg>0.001*h,R/r,Rr)*(spatial.invF13*es.I_sphir+spatial.invF33*es.I_sphiz)*spatial.detF	1	Spatial identity matrix, material frame, PHIZ-component	Domains 1–2	
es.I_sZZ	(spatial.invF13*(spatial.invF13*es.I_srr+spatial.invF33*es.I_szr)+spatial.invF33*(spatial.invF13*es.I_srz+spatial.invF33*es.I_szz))*spatial.detF	1	Spatial identity matrix, material frame, ZZ-component	Domains 1–2	
es.I_srr	1	1	Spatial identity matrix, rr-component	Domains 1–2	
es.I_sphir	0	1	Spatial identity matrix, phir-component	Domains 1–2	
es.I_szr	0	1	Spatial identity matrix, zr-component	Domains 1–2	
es.I_srphi	0	1	Spatial identity matrix, rphi-component	Domains 1–2	
es.I_sphiphi	1	1	Spatial identity	Domains 1–2	

Name	Expression	Unit	Description	Selection	Details
			matrix, phiphi-component		
es.I_szphi	0	1	Spatial identity matrix, zphi-component	Domains 1–2	
es.I_srz	0	1	Spatial identity matrix, rz-component	Domains 1–2	
es.I_sphiz	0	1	Spatial identity matrix, phiz-component	Domains 1–2	
es.I_szz	1	1	Spatial identity matrix, zz-component	Domains 1–2	
es.unTr	es.unTer	Pa	Maxwell upward surface stress tensor, r-component	Boundaries 1–6, 8, 10–20	
es.unTphi	es.unTephi	Pa	Maxwell upward surface stress tensor, phi-component	Boundaries 1–6, 8, 10–20	
es.unTz	es.unTez	Pa	Maxwell upward surface stress tensor, z-component	Boundaries 1–6, 8, 10–20	
es.dnTr	es.dnTer	Pa	Maxwell downward surface stress tensor, r-component	Boundaries 1–6, 8, 10–20	
es.dnTphi	es.dnTephi	Pa	Maxwell downward surface stress tensor, phi-component	Boundaries 1–6, 8, 10–20	
es.dnTz	es.dnTez	Pa	Maxwell downward surface stress tensor, z-component	Boundaries 1–6, 8, 10–20	
es.unr	unr		Normal vector up direction, r-component	Boundaries 1–6, 8, 10–20	

Name	Expression	Unit	Description	Selection	Details
es.unphi	0		Normal vector up direction, phi-component	Boundaries 1–6, 8, 10–20	
es.unz	unz		Normal vector up direction, z-component	Boundaries 1–6, 8, 10–20	
es.dnr	dnr		Normal vector down direction, r-component	Boundaries 1–6, 8, 10–20	
es.dnphi	0		Normal vector down direction, phi-component	Boundaries 1–6, 8, 10–20	
es.dnz	dnz		Normal vector down direction, z-component	Boundaries 1–6, 8, 10–20	
es.unTer	-0.5*es.dnr*(real(up(es.Dr))*real(up(es.Er))+real(up(es.Dphi))*real(up(es.Ephi))+real(up(es.Dz))*real(up(es.Ez)))+real(up(es.Dr))*(real(up(es.Er))*es.dnr+real(up(es.Ephi))*es.dnphi+real(up(es.Ez))*es.dnz)	Pa	Maxwell upward electric surface stress tensor, r-component	Boundaries 6, 8, 16	
es.unTephi	-0.5*es.dnphi*(real(up(es.Dr))*real(up(es.Er))+real(up(es.Dphi))*real(up(es.Ephi))+real(up(es.Dz))*real(up(es.Ez)))+real(up(es.Dphi))*(real(up(es.Er))*es.dnr+real(up(es.Ephi))*es.dnphi+real(up(es.Ez))*es.dnz)	Pa	Maxwell upward electric surface stress tensor, phi-component	Boundaries 6, 8, 16	
es.unTez	-0.5*es.dnz*(real(up(es.Dr))*real(up(es.Er))+real(up(es.Dphi))*real(up(es.Ephi))+real(up(es.Dz))*real(up(es.Ez)))+real(up(es.Dz))*(real(up(es.Er))*es.dnr+real(up(es.Ephi))*es.dnphi+re	Pa	Maxwell upward electric surface stress tensor, z-component	Boundaries 6, 8, 16	

Name	Expression	Unit	Description	Selection	Details
	al(up(es.Ez))*es.dnz)				
es.unTer	0	Pa	Maxwell upward electric surface stress tensor, r-component	Boundaries 1–5, 10–15, 17–20	
es.unTephi	0	Pa	Maxwell upward electric surface stress tensor, phi-component	Boundaries 1–5, 10–15, 17–20	
es.unTez	0	Pa	Maxwell upward electric surface stress tensor, z-component	Boundaries 1–5, 10–15, 17–20	
es.dnTer	-0.5*es.unr*(real(down(es.Dr))*real(down(es.Er))+real(down(es.Dphi))*real(down(es.Ephi))+real(down(es.Dz))*real(down(es.Ez)))+real(down(es.Dr))*(real(down(es.Er))*es.unr+real(down(es.Ephi))*es.unphi+real(down(es.Ez))*es.unz)	Pa	Maxwell downward electric surface stress tensor, r-component	Boundaries 1–6, 10–15, 17–20	
es.dnTephi	-0.5*es.unphi*(real(down(es.Dr))*real(down(es.Er))+real(down(es.Dphi))*real(down(es.Ephi))+real(down(es.Dz))*real(down(es.Ez)))+real(down(es.Dphi))*(real(down(es.Er))*es.unr+real(down(es.Ephi))*es.unphi+real(down(es.Ez))*es.unz)	Pa	Maxwell downward electric surface stress tensor, phi-component	Boundaries 1–6, 10–15, 17–20	
es.dnTez	-0.5*es.unz*(real(down(es.Dr))*real(down(es.Er))+real(down(es.Dphi))*real(down(es.Ephi))+real(down(es.Dz))*real(down(es.Ez)))+real(down(es.Dz))*(real(do	Pa	Maxwell downward electric surface stress tensor, z-component	Boundaries 1–6, 10–15, 17–20	

Name	Expression	Unit	Description	Selection	Details
	wn(es.Er))*es.unr+real(down(es.Ephi))*es.unphi+real(down(es.Ez))*es.unz)				
es.dnTer	0	Pa	Maxwell downward electric surface stress tensor, r-component	Boundaries 8, 16	
es.dnTephi	0	Pa	Maxwell downward electric surface stress tensor, phi-component	Boundaries 8, 16	
es.dnTez	0	Pa	Maxwell downward electric surface stress tensor, z-component	Boundaries 8, 16	
es.intWe	es.int_We(es.d*es.dWe)	J	Total electric energy	Global	+ operation
es.zref	50[ohm]	Ω	Reference impedance	Global	

2.7.3 Charge Conservation 1

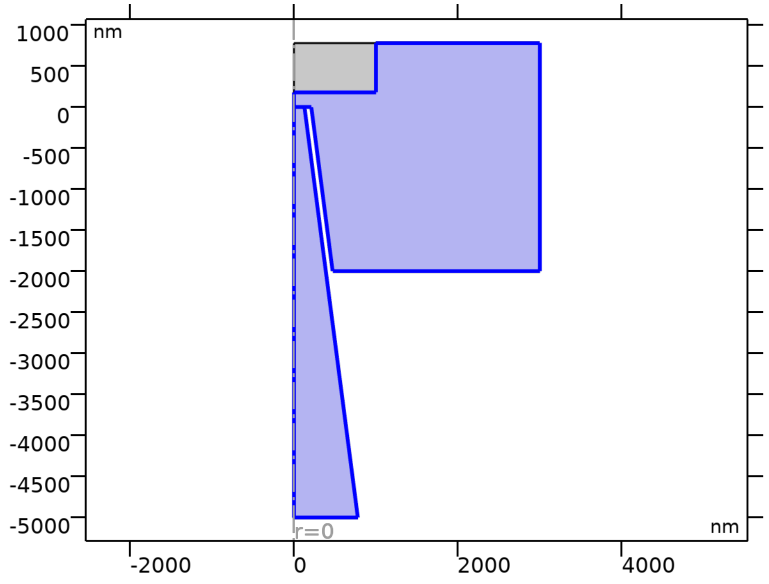

Charge Conservation 1

SELECTION

Geometric entity level	Domain
Selection	Geometry geom1: Dimension 2: All domains

EQUATIONS

$$\mathbf{E} = -\nabla V$$
$$\nabla \cdot (\epsilon_0 \epsilon_r \mathbf{E}) = \rho_v$$

Constitutive Relation D-E

SETTINGS

Description	Value
Dielectric model	Relative permittivity
Relative permittivity	From material

Coordinate System Selection

SETTINGS

Description	Value
Coordinate system	Global coordinate system

PROPERTIES FROM MATERIAL

Property	Material	Property group
Relative permittivity	water	Basic

Variables

Name	Expression	Unit	Description	Selection	Details
es.nD	0	C/m^2	Surface charge density	Boundaries 1–6, 8, 10–20	+ operation
es.epsilonrRR	material.epsilonr11	1	Relative permittivity, RR-component	Domains 1–2	Meta
es.epsilonrPHIR	material.epsilonr21	1	Relative permittivity, PHIR-component	Domains 1–2	Meta
es.epsilonrZR	material.epsilonr31	1	Relative permittivity, ZR-component	Domains 1–2	Meta
es.epsilonrRPHI	material.epsilonr12	1	Relative permittivity, RPHI-component	Domains 1–2	Meta
es.epsilonrPHIPHI	material.epsilonr22	1	Relative permittivity, PHIPHI-	Domains 1–2	Meta

Name	Expression	Unit	Description	Selection	Details
			component		
es.epsilonrZPHI	material.epsilonr32	1	Relative permittivity, ZPHI-component	Domains 1–2	Meta
es.epsilonrRZ	material.epsilonr13	1	Relative permittivity, RZ-component	Domains 1–2	Meta
es.epsilonrPHIZ	material.epsilonr23	1	Relative permittivity, PHIZ-component	Domains 1–2	Meta
es.epsilonrZZ	material.epsilonr33	1	Relative permittivity, ZZ-component	Domains 1–2	Meta
es.epsilonr_iso	material.epsilonr_iso	1	Relative permittivity, isotropic value	Domains 1–2	Meta
es.DrR	0	C/m²	Remanent electric displacement, R-component	Domains 1–2	
es.DrPHI	0	C/m²	Remanent electric displacement, PHI-component	Domains 1–2	
es.DrZ	0	C/m²	Remanent electric displacement, Z-component	Domains 1–2	
es.Dr	(spatial.F11*es.DR+spatial.F31*es.DZ)*spatial.detInvF	C/m²	Electric displacement field, r-component	Domains 1–2	
es.Dphi	if(Rg>0.001*h,r/R,rR)*es.DPHI*spatial.detInvF	C/m²	Electric displacement field, phi-component	Domains 1–2	
es.Dz	(spatial.F13*es.DR+spatial.F33*es.DZ)*spatial.detInvF	C/m²	Electric displacement field, z-component	Domains 1–2	
es.Pr	(spatial.F11*es.PR+spatial.F31*es.PZ)*spatial.detInvF	C/m²	Polarization, r-component	Domains 1–2	

Name	Expression	Unit	Description	Selection	Details
es.Pphi	if(Rg>0.001*h,r/R,rR)*es.PPHI*spatial.detInvF	C/m²	Polarization, phi-component	Domains 1–2	
es.Pz	(spatial.F13*es.PR+spatial.F33*es.PZ)*spatial.detInvF	C/m²	Polarization, z-component	Domains 1–2	
es.normD	sqrt(realdot(es.Dr,es.Dr)+realdot(es.Dphi,es.Dphi)+realdot(es.Dz,es.Dz))	C/m²	Electric displacement field norm	Domains 1–2	
es.normP	sqrt(realdot(es.Pr,es.Pr)+realdot(es.Pphi,es.Pphi)+realdot(es.Pz,es.Pz))	C/m²	Polarization norm	Domains 1–2	
es.PeR	0	C/m²	Polarization contribution, R-component	Domains 1–2	+ operation
es.PePHI	0	C/m²	Polarization contribution, PHI-component	Domains 1–2	+ operation
es.PeZ	0	C/m²	Polarization contribution, Z-component	Domains 1–2	+ operation
es.PhR	0	C/m²	Polarization contribution, R-component	Domains 1–2	+ operation
es.PhPHI	0	C/m²	Polarization contribution, PHI-component	Domains 1–2	+ operation
es.PhZ	0	C/m²	Polarization contribution, Z-component	Domains 1–2	+ operation
es.DR	epsilon0_const*es.I_sRR*es.ER+epsilon0_const*es.I_sRPHI*es.EPHI+epsilon0_const*es.I_sRZ*es.EZ+es.PR+es.PeR+es.PhR	C/m²	Electric displacement field, R-component	Domains 1–2	
es.DPHI	epsilon0_const*es.I_sPHIR*es.ER+epsilon0_const*es.I_sPHIPHI*es.EPHI+epsilon0_const*es.I_sPHIZ*es.EZ+es.PPHI+es.PePHI+	C/m²	Electric displacement field, PHI-component	Domains 1–2	

Name	Expression	Unit	Description	Selection	Details
	es.PhPHI				
es.DZ	epsilon0_const*es.I_sZR*es.ER+epsilon0_const*es.I_sZPHI*es.EPHI+epsilon0_const*es.I_sZZ*es.EZ+es.PZ+es.PeZ+es.PhZ	C/m²	Electric displacement field, Z-component	Domains 1–2	
es.PR	epsilon0_const*(es.chiRR*es.ER+es.chiRPHI*es.EPHI+es.chiRZ*es.EZ)	C/m²	Polarization, R-component	Domains 1–2	+ operation
es.PPHI	epsilon0_const*(es.chiPHIR*es.ER+es.chiPHIPHI*es.EPHI+es.chiPHIZ*es.EZ)	C/m²	Polarization, PHI-component	Domains 1–2	+ operation
es.PZ	epsilon0_const*(es.chiZR*es.ER+es.chiZPHI*es.EPHI+es.chiZZ*es.EZ)	C/m²	Polarization, Z-component	Domains 1–2	+ operation
es.chiRR	-1+es.epsilonrRR	1	Electric susceptibility, RR-component	Domains 1–2	
es.chiPHIR	es.epsilonrPHIR	1	Electric susceptibility, PHIR-component	Domains 1–2	
es.chiZR	es.epsilonrZR	1	Electric susceptibility, ZR-component	Domains 1–2	
es.chiRPHI	es.epsilonrRPHI	1	Electric susceptibility, RPHI-component	Domains 1–2	
es.chiPHIPHI	-1+es.epsilonrPHIPHI	1	Electric susceptibility, PHIPHI-component	Domains 1–2	
es.chiZPHI	es.epsilonrZPHI	1	Electric susceptibility, ZPHI-component	Domains 1–2	
es.chiRZ	es.epsilonrRZ	1	Electric susceptibility, RZ-component	Domains 1–2	
es.chiPHIZ	es.epsilonrPHIZ	1	Electric susceptibility,	Domains 1–2	

Name	Expression	Unit	Description	Selection	Details
			PHIZ-component		
es.chiZZ	-1+es.epsilonrZZ	1	Electric susceptibility, ZZ-component	Domains 1–2	
es.Er	spatial.invF11*es.ER+spatial.invF13*es.EZ	V/m	Electric field, r-component	Domains 1–2	
es.Ephi	if(Rg>0.001*h,R/r,Rr)*es.EPHI	V/m	Electric field, phi-component	Domains 1–2	
es.Ez	spatial.invF31*es.ER+spatial.invF33*es.EZ	V/m	Electric field, z-component	Domains 1–2	
es.tEr	spatial.invF11*es.tER+spatial.invF13*es.tEZ	V/m	Tangential electric field, r-component	Boundaries 1–6, 8, 10–20	
es.tEphi	if(Rg>0.001*h,R/r,Rr)*es.tEPHI	V/m	Tangential electric field, phi-component	Boundaries 1–6, 8, 10–20	
es.tEz	spatial.invF31*es.tER+spatial.invF33*es.tEZ	V/m	Tangential electric field, z-component	Boundaries 1–6, 8, 10–20	
es.ER	-VR	V/m	Electric field, R-component	Domains 1–2	+ operation
es.EPHI	0	V/m	Electric field, PHI-component	Domains 1–2	+ operation
es.EZ	-VZ	V/m	Electric field, Z-component	Domains 1–2	+ operation
es.tER	-VTR	V/m	Tangential electric field, R-component	Boundaries 1–6, 8, 10–20	
es.tEPHI	0	V/m	Tangential electric field, PHI-component	Boundaries 1–6, 8, 10–20	
es.tEZ	-VTZ	V/m	Tangential electric field, Z-component	Boundaries 1–6, 8, 10–20	
es.normE	sqrt(realdot(es.Er,es.Er)+realdot(es.Ephi,es.Ephi)+realdot(es.Ez,es.Ez))	V/m	Electric field norm	Domains 1–2	
es.Jr	(spatial.F11*es.JdR+spatial.F31*es.JdZ)*spatial.detInvF	A/m²	Current density, r-component	Domains 1–2	+ operation
es.Jphi	if(Rg>0.001*h,r/R,rR)	A/m²	Current density,	Domains 1–2	+ operation

Name	Expression	Unit	Description	Selection	Details
	*es.JdPHI*spatial.detInvF		phi-component		
es.Jz	(spatial.F13*es.JdR+spatial.F33*es.JdZ)*spatial.detInvF	A/m²	Current density, z-component	Domains 1–2	+ operation
es.JR	es.JdR	A/m²	Current density, R-component	Domains 1–2	+ operation
es.JPHI	es.JdPHI	A/m²	Current density, PHI-component	Domains 1–2	+ operation
es.JZ	es.JdZ	A/m²	Current density, Z-component	Domains 1–2	+ operation
es.JdR	0	A/m²	Displacement current density, R-component	Domains 1–2	
es.JdPHI	0	A/m²	Displacement current density, PHI-component	Domains 1–2	
es.JdZ	0	A/m²	Displacement current density, Z-component	Domains 1–2	
es.normJ	sqrt(realdot(es.JR,es.JR)+realdot(es.JPHI,es.JPHI)+realdot(es.JZ,es.JZ))	A/m²	Current density norm	Domains 1–2	
es.ccn1.nJ	es.dnr*(spatial.invF11*up(es.JR)+spatial.invF13*up(es.JZ))+es.dnphi*if(Rg>0.001*h,R/r,Rr)*up(es.JPHI)+es.dnz*(spatial.invF31*up(es.JR)+spatial.invF33*up(es.JZ))	A/m²	Inward current density	Boundaries 8, 16	
es.ccn1.nJ	es.unr*(spatial.invF11*down(es.JR)+spatial.invF13*down(es.JZ))+es.unphi*if(Rg>0.001*h,R/r,Rr)*down(es.JPHI)+es.unz*(spatial.invF31*down(es.JR)+spatial.invF33*down(es.JZ))	A/m²	Inward current density	Boundaries 1–5, 10–15, 17–20	
es.W	es.We	J/m³	Energy density	Domains 1–2	+ operation
es.dWe	2*es.We*pi*R	J/m²	Integrand for	Domains 1–2	Meta

Name	Expression	Unit	Description	Selection	Details
			total electric energy		
es.We	0.5*epsilon0_const*(((es.I_sRR+es.chiRR)*es.ER+(es.I_sRPHI+es.chiRPHI)*es.EPHI+(es.I_sRZ+es.chiRZ)*es.EZ)*es.ER+((es.I_sPHIR+es.chiPHIR)*es.ER+(es.I_sPHIPHI+es.chiPHIPHI)*es.EPHI+(es.I_sPHIZ+es.chiPHIZ)*es.EZ)*es.EPHI+((es.I_sZR+es.chiZR)*es.ER+(es.I_sZPHI+es.chiZPHI)*es.EPHI+(es.I_sZZ+es.chiZZ)*es.EZ)*es.EZ)*spatial.detInvF	J/m^3	Electric energy density	Domains 1–2	

Shape functions

Name	Shape function	Unit	Description	Shape frame	Selection
V	Lagrange (Quadratic)	V	Electric potential	Material	Domains 1–2
V	Lagrange (Quadratic)	V	Electric potential	Spatial	Domains 1–2
V	Lagrange (Quadratic)	V	Electric potential	Geometry	Domains 1–2
V	Lagrange (Quadratic)	V	Electric potential	Mesh	Domains 1–2

Weak Expressions

Weak expression	Integration order	Integration frame	Selection
-2*(es.DR*test(VR)+es.DZ*test(VZ))*es.d*pi*R	4	Material	Domains 1–2

2.7.4 Axial Symmetry 1

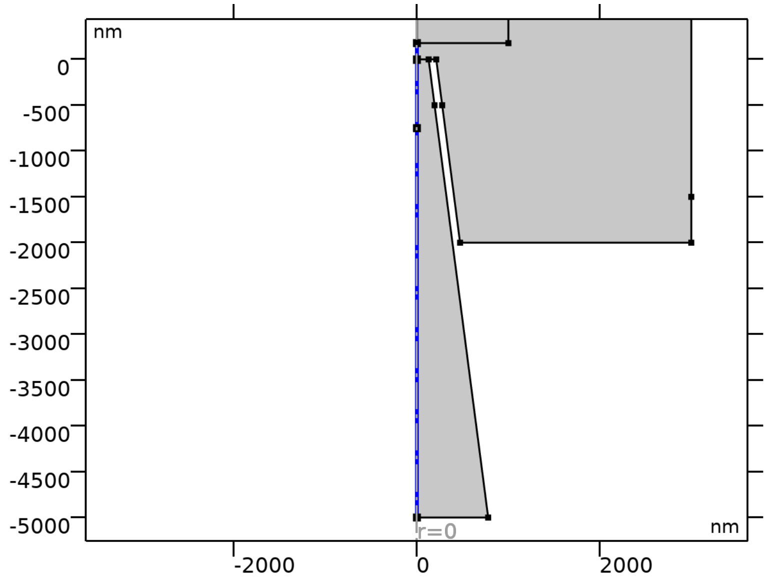

Axial Symmetry 1

SELECTION

Geometric entity level	Boundary
Selection	Geometry geom1: Dimension 1: All boundaries

2.7.5 Zero Charge 1

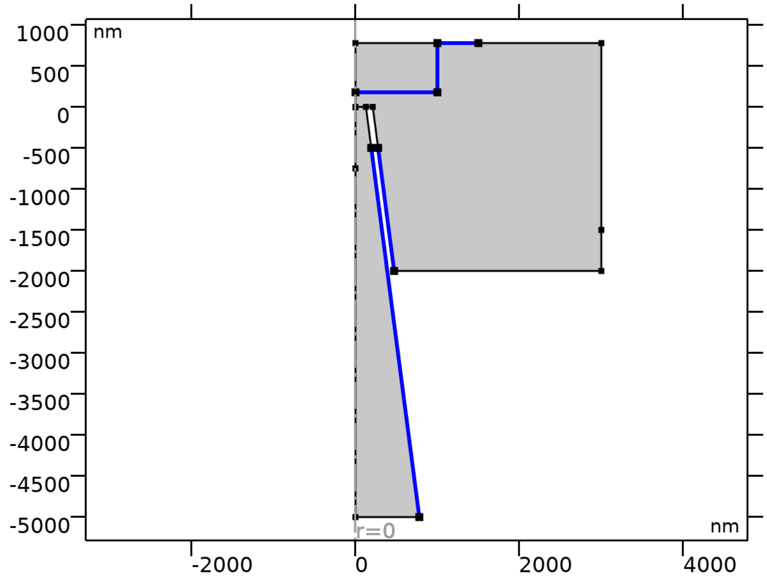

Zero Charge 1

SELECTION

Geometric entity level	Boundary

Selection	Geometry geom1: Dimension 1: All boundaries

EQUATIONS

$$\mathbf{n} \cdot \mathbf{D} = 0$$

Variables

Name	Expression	Unit	Description	Selection	Details
es.nD	0	C/m^2	Surface charge density	Boundaries 8, 12, 14, 16–17	+ operation

Shape functions

Name	Shape function	Unit	Description	Shape frame	Selection	Details
V	Lagrange (Quadratic)	V	Electric potential	Spatial	No boundaries	Slit
V	Lagrange (Quadratic)	V	Electric potential	Material	No boundaries	Slit
V	Lagrange (Quadratic)	V	Electric potential	Geometry	No boundaries	Slit
V	Lagrange (Quadratic)	V	Electric potential	Mesh	No boundaries	Slit

2.7.6 Initial Values 1

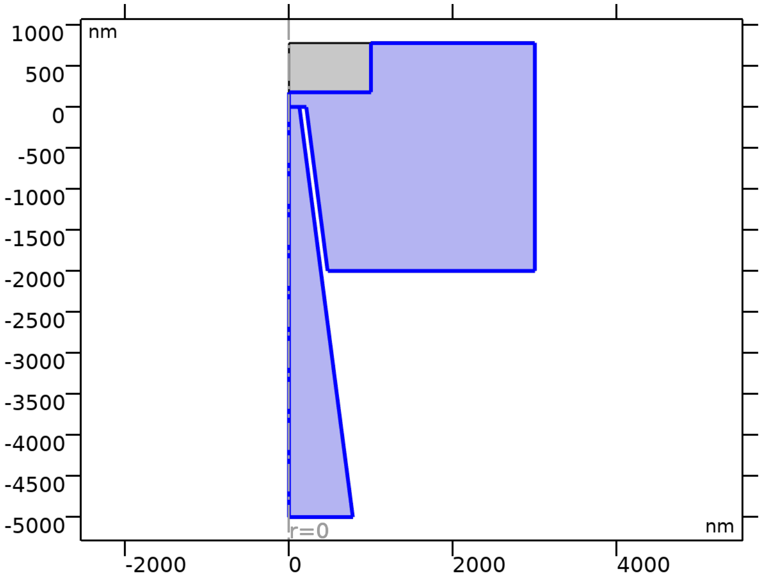

Initial Values 1

SELECTION

Geometric entity level	Domain

Selection	Geometry geom1: Dimension 2: All domains

SETTINGS

Description	Value	Unit
Electric potential	0.2	V

2.7.7 Pipette Potential

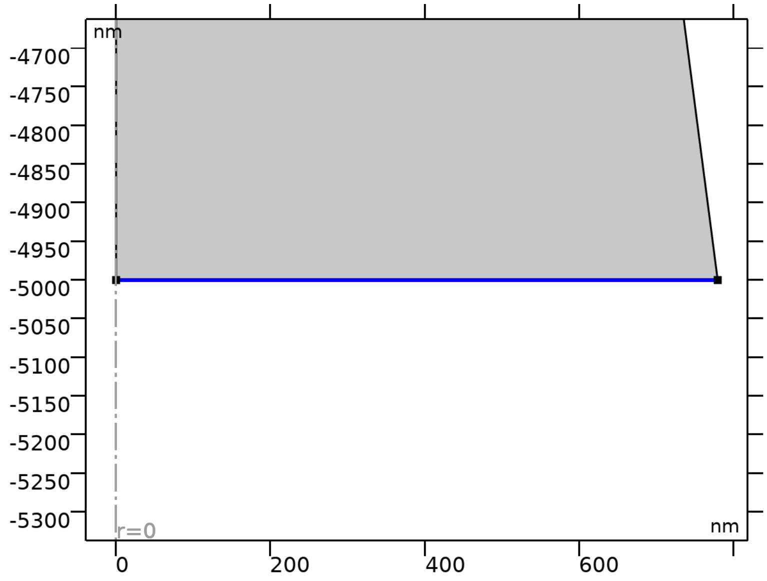

Pipette Potential

SELECTION

Geometric entity level	Boundary
Selection	Geometry geom1: Dimension 1: Boundary 2

EQUATIONS

$$V = V_0$$

Electric Potential

SETTINGS

Description	Value	Unit
Electric potential	0.1	V

Variables

Name	Expression	Unit	Description	Selection	Details
es.nD	(es.unr*(spatial.F11*down(es.DR)+spatial.F31*down(es.DZ))+es.unphi*if(Rg>0.001*h,r/R,rR)*down(es.DPHI)+es.unz*(spatial.F	C/m^2	Surface charge density	Boundary 2	+ operation

Name	Expression	Unit	Description	Selection	Details
	13*down(es.DR)+spatial.F33*down(es.DZ)))*spatial.detInvF				
es.V0	0.1	V	Electric potential	Boundary 2	

Constraints

Constraint	Constraint force	Shape function	Selection	Details
es.V0-V	test(es.V0-V)	Lagrange (Quadratic)	Boundary 2	Elemental

2.7.8 Pipette Ground

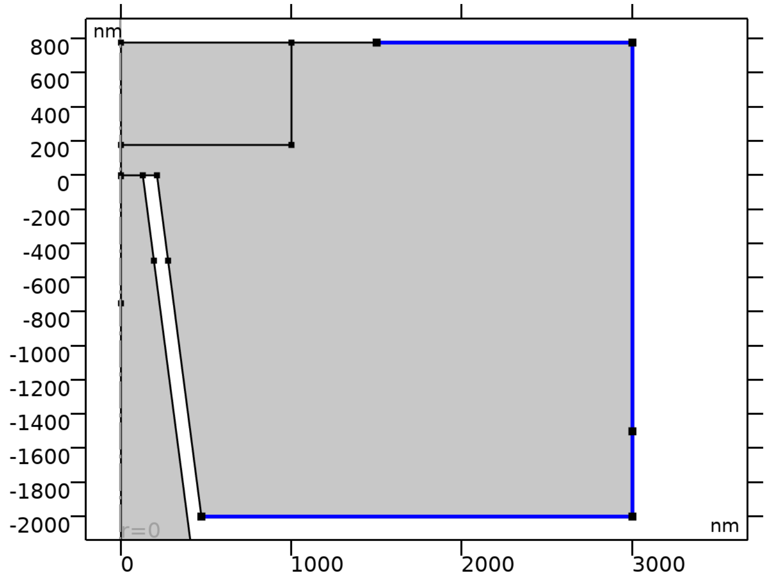

Pipette Ground

SELECTION

Geometric entity level	Boundary
Selection	Geometry geom1: Dimension 1: Boundaries 15, 18–20

EQUATIONS

$$V = 0$$

Variables

Name	Expression	Unit	Description	Selection	Details
es.nD	(es.unr*(spatial.F11*down(es.DR)+spatial.F31*down(es.DZ))+es.unphi*if(Rg>0.001*h,r/R,rR)*down(es.DPHI)+es.unz*(spatial.F13*down(es.DR)+spatial.F33*do	C/m²	Surface charge density	Boundaries 15, 18–20	+ operation

Name	Expression	Unit	Description	Selection	Details
	wn(es.DZ)))*spatial.detInvF				

Constraints

Constraint	Constraint force	Shape function	Selection	Details
-V	test(-V)	Lagrange (Quadratic)	Boundaries 15, 18–20	Elemental

2.7.9 Surface Charge of Pipette

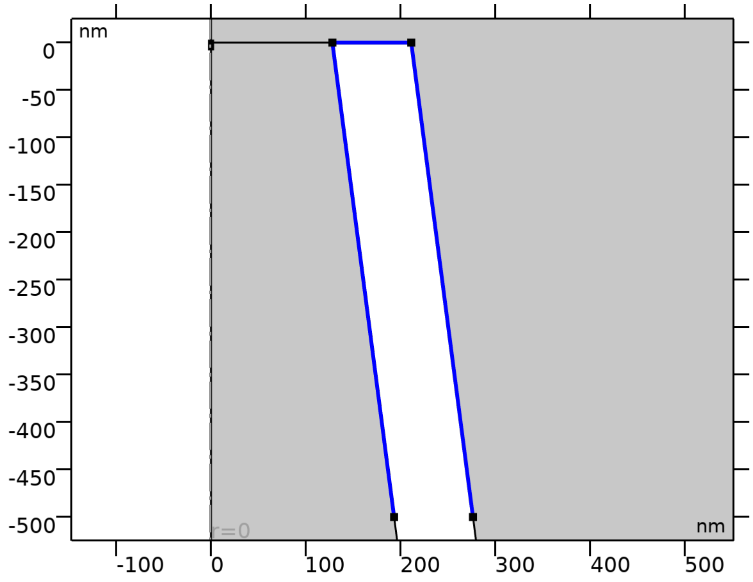

Surface Charge of Pipette

SELECTION

Geometric entity level	Boundary
Selection	Geometry geom1: Dimension 1: Boundaries 10–11, 13

EQUATIONS

$$\mathbf{n} \cdot (\mathbf{D}_1 - \mathbf{D}_2) = \rho_s$$

Surface Charge Density

SETTINGS

Description	Value	Unit
Surface charge density	rhop	C/m²

Coordinate System Selection

SETTINGS

Description	Value
Coordinate system	Global coordinate system

Variables

Name	Expression	Unit	Description	Selection	Details
es.nD	es.sfcd1.rhoqs	C/m^2	Surface charge density	Boundaries 10–11, 13	+ operation
es.sfcd1.rhoqs	rhop	C/m^2	Surface charge density	Boundaries 10–11, 13	

Weak Expressions

Weak expression	Integration order	Integration frame	Selection
-2*es.sfcd1.rhoqs*test(V)*es.d*pi*r	4	Spatial	Boundaries 10–11, 13

2.8 SOLID MECHANICS

USED PRODUCTS

COMSOL Multiphysics

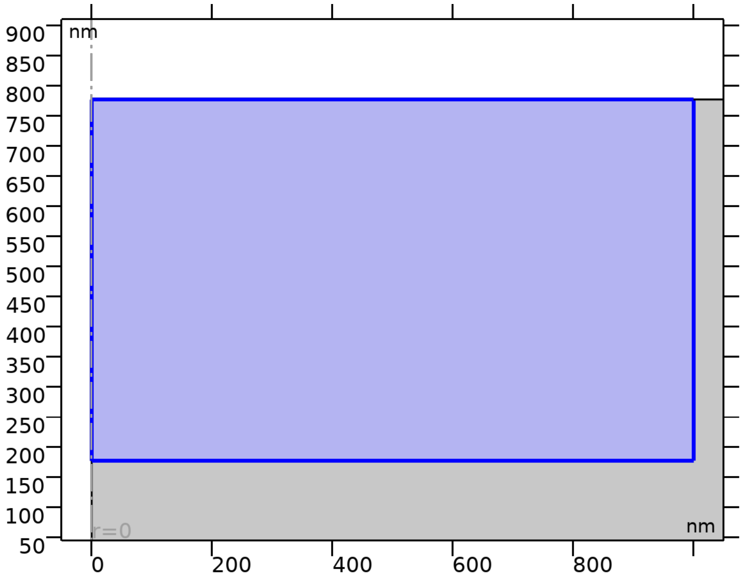

Solid Mechanics

SELECTION

Geometric entity level	Domain
Selection	Geometry geom1: Dimension 2: Domain 3

EQUATIONS

$$0 = \nabla \cdot (FS)^T + \mathbf{F}_V, \quad F = I + \nabla \mathbf{u}$$
$$\mathbf{u}(R, \Phi, Z) \rightarrow (u, 0, w)^T$$

2.8.1 Interface Settings

Physics Symbols

SETTINGS

Description	Value
Enable physics symbols	Off

Discretization

SETTINGS

Description	Value
Displacement field	Quadratic serendipity

SETTINGS

Description	Value
Equation form	Study controlled

Axial Symmetry Approximation

SETTINGS

Description	Value
Circumferential mode extension	Off
Include circumferential displacement	Off

SETTINGS

Description	Value
Structural transient behavior	Include inertial terms

Transient Solver Settings

SETTINGS

Description	Value
Description	Changes made to these settings only take effect when the default solver is generated.
Maximum frequency to resolve	Off

Typical Wave Speed for Perfectly Matched Layers

SETTINGS

Description	Value	Unit
Typical wave speed for perfectly matched layers	solid.cp	m/s

2.8.2 Variables

Name	Expression	Unit	Description	Selection	Details
solid.R	R	m	Material coordinates, R-component	Domain 3	
solid.PHI	0	m	Material coordinates, PHI-component	Domain 3	
solid.Z	Z	m	Material coordinates, Z-component	Domain 3	
solid.R	R	m	Material coordinates, R-component	Boundaries 7–9, 16	
solid.PHI	0	m	Material coordinates, PHI-component	Boundaries 7–9, 16	
solid.Z	Z	m	Material coordinates, Z-component	Boundaries 7–9, 16	
solid.r	r	m	Spatial coordinates, r-component	Domain 3	
solid.phi	0	m	Spatial coordinates, phi-component	Domain 3	
solid.z	z	m	Spatial coordinates, z-component	Domain 3	
solid.r	r	m	Spatial coordinates, r-component	Boundaries 7–9, 16	
solid.phi	0	m	Spatial coordinates, phi-component	Boundaries 7–9, 16	
solid.z	z	m	Spatial coordinates, z-component	Boundaries 7–9, 16	
solid.uf	u	m	Displacement field, R-component	Domain 3	
solid.vf	0	m	Displacement field, PHI-	Domain 3	

Name	Expression	Unit	Description	Selection	Details
			component		
solid.wf	w	m	Displacement field, Z-component	Domain 3	
solid.uf	u	m	Displacement field, R-component	Boundaries 7–9, 16	
solid.vf	0	m	Displacement field, PHI-component	Boundaries 7–9, 16	
solid.wf	w	m	Displacement field, Z-component	Boundaries 7–9, 16	
solid.nR	dnR	1	Normal vector, R-component	Boundaries 7–9, 16	
solid.nPHI	0	1	Normal vector, PHI-component	Boundaries 7–9, 16	
solid.nZ	dnZ	1	Normal vector, Z-component	Boundaries 7–9, 16	
solid.nr	dnr	1	Normal vector, r-component	Boundaries 7–9, 16	
solid.nphi	0	1	Normal vector, phi-component	Boundaries 7–9, 16	
solid.nz	dnz	1	Normal vector, z-component	Boundaries 7–9, 16	
solid.nRmesh	dnRmesh	1	Normal vector (mesh), R-component	Boundaries 7–9, 16	
solid.nPHImesh	0	1	Normal vector (mesh), PHI-component	Boundaries 7–9, 16	
solid.nZmesh	dnZmesh	1	Normal vector (mesh), Z-component	Boundaries 7–9, 16	
solid.nrmesh	dnrmesh	1	Normal vector (mesh), r-component	Boundaries 7–9, 16	
solid.nphimesh	0	1	Normal vector (mesh), phi-component	Boundaries 7–9, 16	
solid.nzmesh	dnzmesh	1	Normal vector (mesh), z-	Boundaries 7–9, 16	

Name	Expression	Unit	Description	Selection	Details
			component		
solid.beta_dK	0	s	Stiffness damping parameter	Domain 3	+ operation
solid.mk	0	1	Azimuthal mode number	Global	
solid.cref	solid.cp	m/s	Typical wave speed for perfectly matched layers	Domain 3	
solid.isGeomNon	1	1	Geometric nonlinearity variable	Global	
solid.RFtotalr	solid.sumreaction(0)	N	Total reaction force, r-component	Global	+ operation
solid.RFtotalphi	solid.sumreaction(0)	N	Total reaction force, phi-component	Global	+ operation
solid.RFtotalz	solid.sumreaction(solid.RFz)+solid.RFfsz+solid.RFfdz	N	Total reaction force, z-component	Global	+ operation
solid.RMtotalr	0	N·m	Total reaction moment, r-component	Global	+ operation
solid.RMtotalphi	0	N·m	Total reaction moment, phi-component	Global	+ operation
solid.RMtotalz	0	N·m	Total reaction moment, z-component	Global	+ operation
rt	d(r,TIME)	m/s	Mesh velocity, r-component	Global	
phit	0	m/s	Mesh velocity, phi-component	Global	
zt	d(z,TIME)	m/s	Mesh velocity, z-component	Global	

2.8.3 Linear Elastic Material 1

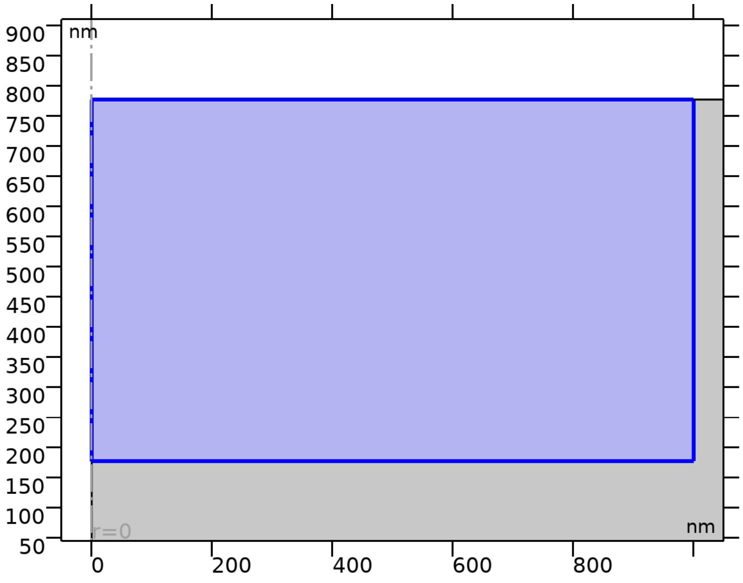

Linear Elastic Material 1

SELECTION

Geometric entity level	Domain
Selection	Geometry geom1: Dimension 2: All domains

EQUATIONS

$$0=\nabla\cdot(\mathrm{F\,S})^{T}+\mathbf{F}_{V},\quad \mathrm{F}=\mathrm{I}+\nabla\mathbf{u}$$

$$\mathbf{u}(R,\Phi,Z)\rightarrow(u,0,w)^{T}$$

$$\mathrm{S}=\mathrm{S}_{\mathrm{inel}}+\mathrm{S}_{\mathrm{el}}+\left(-\frac{1}{3}\mathrm{trace}(\mathbf{C}:\epsilon_{\mathrm{el}})-p_{w}\right)\mathrm{I},\quad \epsilon_{\mathrm{el}}=\frac{1}{2}\left(\mathrm{F}_{\mathrm{el}}^{T}\mathrm{F}_{\mathrm{el}}-\mathrm{I}\right),\quad \mathrm{F}_{\mathrm{el}}=\mathrm{F}\mathrm{F}_{\mathrm{inel}}^{-1}$$

$$\mathrm{S}_{\mathrm{el}}=J_{\mathrm{i}}\mathrm{F}_{\mathrm{inel}}^{-1}\left(\mathbf{C}:\epsilon_{\mathrm{el}}\right)\mathrm{F}_{\mathrm{inel}}^{-T}$$

$$\mathrm{S}_{\mathrm{inel}}=\mathrm{S}_{0}+\mathrm{S}_{\mathrm{ext}}+\mathrm{S}_{\mathrm{q}}$$

$$\epsilon=\frac{1}{2}\left[(\nabla\mathbf{u})^{T}+\nabla\mathbf{u}+(\nabla\mathbf{u})^{T}\nabla\mathbf{u}\right]$$

$$\mathbf{C}=\mathbf{C}(E,\nu)$$

Linear Elastic Material

SETTINGS

Description	Value
Material symmetry	Isotropic
Specify	Young's modulus and Poisson's ratio
Young's modulus	From material
Poisson's ratio	From material
Density	From material

Description	Value
Use mixed formulation	Pressure formulation

Geometric Nonlinearity

SETTINGS

Description	Value
Geometrically linear formulation	Off
Additive strain decomposition	Off

Quadrature Settings

SETTINGS

Description	Value
Reduced integration	Off

Coordinate System Selection

SETTINGS

Description	Value
Coordinate system	Global coordinate system

Model Input

SETTINGS

Description	Value
Volume reference temperature	Common model input
Temperature	Common model input

PROPERTIES FROM MATERIAL

Property	Material	Property group
Young's modulus	polystyrene	Young's modulus and Poisson's ratio
Poisson's ratio	polystyrene	Young's modulus and Poisson's ratio
Density	polystyrene	Basic

Variables

Name	Expression	Unit	Description	Selection	Details
solid.disp	sqrteps(real(u)^2+real(w)^2)	m	Displacement magnitude	Domain 3	
solid.curlUR	0	1	Curl of displacement, R-component	Domain 3	
solid.curlUPHI	solid.gradUrZ-	1	Curl of displacement,	Domain 3	

Name	Expression	Unit	Description	Selection	Details
	solid.gradUzR		PHI-component		
solid.curlUZ	0	1	Curl of displacement, Z-component	Domain 3	
solid.gradUrR	uR	1	Displacement gradient, rR-component	Domain 3	
solid.gradUphiR	0	1	Displacement gradient, phiR-component	Domain 3	
solid.gradUzR	wR	1	Displacement gradient, zR-component	Domain 3	
solid.gradUrPHI	0	1	Displacement gradient, rPHI-component	Domain 3	
solid.gradUphiPHI	if(abs(R)<0.001*h,uR,u/R)	1	Displacement gradient, phiPHI-component	Domain 3	
solid.gradUzPHI	0	1	Displacement gradient, zPHI-component	Domain 3	
solid.gradUrZ	uZ	1	Displacement gradient, rZ-component	Domain 3	
solid.gradUphiZ	0	1	Displacement gradient, phiZ-component	Domain 3	
solid.gradUzZ	wZ	1	Displacement gradient, zZ-component	Domain 3	
solid.FdrR	1+solid.gradUrR	1	Deformation gradient, rR-component	Domain 3	
solid.FdphiR	solid.gradUphiR	1	Deformation gradient, phiR-component	Domain 3	
solid.FdzR	solid.gradUzR	1	Deformation gradient, zR-component	Domain 3	
solid.FdrPHI	solid.gradUrPHI	1	Deformation gradient, rPHI-	Domain 3	

Name	Expression	Unit	Description	Selection	Details
			component		
solid.FdphiPHI	1+solid.gradUphiPHI	1	Deformation gradient, phiPHI-component	Domain 3	
solid.FdzPHI	solid.gradUzPHI	1	Deformation gradient, zPHI-component	Domain 3	
solid.FdrZ	solid.gradUrZ	1	Deformation gradient, rZ-component	Domain 3	
solid.FdphiZ	solid.gradUphiZ	1	Deformation gradient, phiZ-component	Domain 3	
solid.FdzZ	1+solid.gradUzZ	1	Deformation gradient, zZ-component	Domain 3	
solid.FdiRr	(solid.FdphiPHI*solid.FdzZ-solid.FdphiZ*solid.FdzPHI)/(solid.FdrR*solid.FdphiPHI*solid.FdzZ+solid.FdrPHI*solid.FdphiZ*solid.FdzR+solid.FdrZ*solid.FdphiR*solid.FdzPHI-solid.FdrR*solid.FdphiZ*solid.FdzPHI-solid.FdrPHI*solid.FdphiR*solid.FdzZ-solid.FdrZ*solid.FdphiPHI*solid.FdzR)	1	Deformation gradient inverse, Rr-component	Domain 3	
solid.FdiPHIr	(solid.FdphiZ*solid.FdzR-solid.FdphiR*solid.FdzZ)/(solid.FdrR*solid.FdphiPHI*solid.FdzZ+solid.FdrPHI*solid.FdphiZ*solid.FdzR+solid.FdrZ*solid.FdphiR*solid.FdzPHI-solid.FdrR*solid.FdphiZ*solid.FdzPHI-	1	Deformation gradient inverse, PHIr-component	Domain 3	

Name	Expression	Unit	Description	Selection	Details
	solid.FdrPHI*solid.FdphiR*solid.FdzZ-solid.FdrZ*solid.FdphiPHI*solid.FdzR)				
solid.FdiZr	(solid.FdphiR*solid.FdzPHI-solid.FdphiPHI*solid.FdzR)/(solid.FdrR*solid.FdphiPHI*solid.FdzZ+solid.FdrPHI*solid.FdphiZ*solid.FdzR+solid.FdrZ*solid.FdphiR*solid.FdzPHI-solid.FdrR*solid.FdphiZ*solid.FdzPHI-solid.FdrPHI*solid.FdphiR*solid.FdzZ-solid.FdrZ*solid.FdphiPHI*solid.FdzR)	1	Deformation gradient inverse, Zr-component	Domain 3	
solid.FdiRphi	(solid.FdrZ*solid.FdzPHI-solid.FdrPHI*solid.FdzZ)/(solid.FdrR*solid.FdphiPHI*solid.FdzZ+solid.FdrPHI*solid.FdphiZ*solid.FdzR+solid.FdrZ*solid.FdphiR*solid.FdzPHI-solid.FdrR*solid.FdphiZ*solid.FdzPHI-solid.FdrPHI*solid.FdphiR*solid.FdzZ-solid.FdrZ*solid.FdphiPHI*solid.FdzR)	1	Deformation gradient inverse, Rphi-component	Domain 3	
solid.FdiPHIphi	(solid.FdrR*solid.FdzZ-solid.FdrZ*solid.FdzR)/(solid.FdrR*solid.FdphiPHI*solid.FdzZ+solid.FdrPHI*solid.FdphiZ*solid.FdzR+solid.FdrZ*solid.FdphiR*solid.FdzPHI-	1	Deformation gradient inverse, PHIphi-component	Domain 3	

Name	Expression	Unit	Description	Selection	Details
	solid.FdrR*solid.FdphiZ*solid.FdzPHI-solid.FdrPHI*solid.FdphiR*solid.FdzZ-solid.FdrZ*solid.FdphiPHI*solid.FdzR)				
solid.FdiZphi	(solid.FdrPHI*solid.FdzR-solid.FdrR*solid.FdzPHI)/(solid.FdrR*solid.FdphiPHI*solid.FdzZ+solid.FdrPHI*solid.FdphiZ*solid.FdzR+solid.FdrZ*solid.FdphiR*solid.FdzPHI-solid.FdrR*solid.FdphiZ*solid.FdzPHI-solid.FdrPHI*solid.FdphiR*solid.FdzZ-solid.FdrZ*solid.FdphiPHI*solid.FdzR)	1	Deformation gradient inverse, Zphi-component	Domain 3	
solid.FdiRz	(solid.FdrPHI*solid.FdphiZ-solid.FdrZ*solid.FdphiPHI)/(solid.FdrR*solid.FdphiPHI*solid.FdzZ+solid.FdrPHI*solid.FdphiZ*solid.FdzR+solid.FdrZ*solid.FdphiR*solid.FdzPHI-solid.FdrR*solid.FdphiZ*solid.FdzPHI-solid.FdrPHI*solid.FdphiR*solid.FdzZ-solid.FdrZ*solid.FdphiPHI*solid.FdzR)	1	Deformation gradient inverse, Rz-component	Domain 3	
solid.FdiPHIz	(solid.FdrZ*solid.FdphiR-solid.FdrR*solid.FdphiZ)/(solid.FdrR*solid.FdphiPHI*solid.FdzZ+solid.FdrPHI*solid.FdphiZ*solid.FdzR+solid.FdrZ*solid.	1	Deformation gradient inverse, PHIz-component	Domain 3	

Name	Expression	Unit	Description	Selection	Details
	FdphiR*solid.FdzPHI-solid.FdrR*solid.FdphiZ*solid.FdzPHI-solid.FdrPHI*solid.FdphiR*solid.FdzZ-solid.FdrZ*solid.FdphiPHI*solid.FdzR)				
solid.FdiZz	(solid.FdrR*solid.FdphiPHI-solid.FdrPHI*solid.FdphiR)/(solid.FdrR*solid.FdphiPHI*solid.FdzZ+solid.FdrPHI*solid.FdphiZ*solid.FdzR+solid.FdrZ*solid.FdphiR*solid.FdzPHI-solid.FdrR*solid.FdphiZ*solid.FdzPHI-solid.FdrPHI*solid.FdphiR*solid.FdzZ-solid.FdrZ*solid.FdphiPHI*solid.FdzR)	1	Deformation gradient inverse, Zz-component	Domain 3	
solid.J	solid.FdrR*solid.FdphiPHI*solid.FdzZ+solid.FdrPHI*solid.FdphiZ*solid.FdzR+solid.FdrZ*solid.FdphiR*solid.FdzPHI-solid.FdrR*solid.FdphiZ*solid.FdzPHI-solid.FdrPHI*solid.FdphiR*solid.FdzZ-solid.FdrZ*solid.FdphiPHI*solid.FdzR	1	Volume ratio	Domain 3	
solid.Ji	1/(solid.Fiil11*solid.Fiil22*solid.Fiil33+solid.Fiil12*solid.Fiil23*solid.Fiil31+solid.Fiil13*solid.Fiil21*solid.Fiil32-solid.Fiil11*solid.Fiil23*solid.Fiil32-solid.Fiil12*solid.Fiil21*solid.Fiil33-	1	Inelastic volume ratio	Domain 3	

Name	Expression	Unit	Description	Selection	Details
	solid.Fiil13*solid.Fiil22*solid.Fiil31)				
solid.Fdlr1	solid.FdrR	1	Deformation gradient, local, r1-component	Domain 3	
solid.Fdlphi1	solid.FdphiR	1	Deformation gradient, local, phi1-component	Domain 3	
solid.Fdlz1	solid.FdzR	1	Deformation gradient, local, z1-component	Domain 3	
solid.Fdlr2	solid.FdrPHI	1	Deformation gradient, local, r2-component	Domain 3	
solid.Fdlphi2	solid.FdphiPHI	1	Deformation gradient, local, phi2-component	Domain 3	
solid.Fdlz2	solid.FdzPHI	1	Deformation gradient, local, z2-component	Domain 3	
solid.Fdlr3	solid.FdrZ	1	Deformation gradient, local, r3-component	Domain 3	
solid.Fdlphi3	solid.FdphiZ	1	Deformation gradient, local, phi3-component	Domain 3	
solid.Fdlz3	solid.FdzZ	1	Deformation gradient, local, z3-component	Domain 3	
solid.Fil11	(solid.Fiil22*solid.Fiil33-solid.Fiil23*solid.Fiil32)/(solid.Fiil11*solid.Fiil22*solid.Fiil33+solid.Fiil12*solid.Fiil23*solid.Fiil31+solid.Fiil13*solid.Fiil21*solid.Fiil32-solid.Fiil11*solid.Fiil23*solid.Fiil32-solid.Fiil12*solid.Fiil	1	Inelastic deformation gradient, local coordinate system, 11-component	Domain 3	Matrix multiplication

Name	Expression	Unit	Description	Selection	Details
	21*solid.Fiil33-solid.Fiil13*solid.Fiil22*solid.Fiil31)				
solid.Fil21	(solid.Fiil23*solid.Fiil31-solid.Fiil21*solid.Fiil33)/(solid.Fiil11*solid.Fiil22*solid.Fiil33+solid.Fiil12*solid.Fiil23*solid.Fiil31+solid.Fiil13*solid.Fiil21*solid.Fiil32-solid.Fiil11*solid.Fiil23*solid.Fiil32-solid.Fiil12*solid.Fiil21*solid.Fiil33-solid.Fiil13*solid.Fiil22*solid.Fiil31)	1	Inelastic deformation gradient, local coordinate system, 21-component	Domain 3	Matrix multiplication
solid.Fil31	(solid.Fiil21*solid.Fiil32-solid.Fiil22*solid.Fiil31)/(solid.Fiil11*solid.Fiil22*solid.Fiil33+solid.Fiil12*solid.Fiil23*solid.Fiil31+solid.Fiil13*solid.Fiil21*solid.Fiil32-solid.Fiil11*solid.Fiil23*solid.Fiil32-solid.Fiil12*solid.Fiil21*solid.Fiil33-solid.Fiil13*solid.Fiil22*solid.Fiil31)	1	Inelastic deformation gradient, local coordinate system, 31-component	Domain 3	Matrix multiplication
solid.Fil12	(solid.Fiil13*solid.Fiil32-solid.Fiil12*solid.Fiil33)/(solid.Fiil11*solid.Fiil22*solid.Fiil33+solid.Fiil12*solid.Fiil23*solid.Fiil31+solid.Fiil13*solid.Fiil21*solid.Fiil32-solid.Fiil11*solid.Fiil23*solid.Fiil32-solid.Fiil12*solid.Fiil21*solid.Fiil33-	1	Inelastic deformation gradient, local coordinate system, 12-component	Domain 3	Matrix multiplication

Name	Expression	Unit	Description	Selection	Details
	solid.Fiil13*solid.Fiil22*solid.Fiil31)				
solid.FiI22	(solid.Fiil11*solid.Fiil33-solid.Fiil13*solid.Fiil31)/(solid.Fiil11*solid.Fiil22*solid.Fiil33+solid.Fiil12*solid.Fiil23*solid.Fiil31+solid.Fiil13*solid.Fiil21*solid.Fiil32-solid.Fiil11*solid.Fiil23*solid.Fiil32-solid.Fiil12*solid.Fiil21*solid.Fiil33-solid.Fiil13*solid.Fiil22*solid.Fiil31)	1	Inelastic deformation gradient, local coordinate system, 22-component	Domain 3	Matrix multiplication
solid.FiI32	(solid.Fiil12*solid.Fiil31-solid.Fiil11*solid.Fiil32)/(solid.Fiil11*solid.Fiil22*solid.Fiil33+solid.Fiil12*solid.Fiil23*solid.Fiil31+solid.Fiil13*solid.Fiil21*solid.Fiil32-solid.Fiil11*solid.Fiil23*solid.Fiil32-solid.Fiil12*solid.Fiil21*solid.Fiil33-solid.Fiil13*solid.Fiil22*solid.Fiil31)	1	Inelastic deformation gradient, local coordinate system, 32-component	Domain 3	Matrix multiplication
solid.FiI13	(solid.Fiil12*solid.Fiil23-solid.Fiil13*solid.Fiil22)/(solid.Fiil11*solid.Fiil22*solid.Fiil33+solid.Fiil12*solid.Fiil23*solid.Fiil31+solid.Fiil13*solid.Fiil21*solid.Fiil32-solid.Fiil11*solid.Fiil23*solid.Fiil32-solid.Fiil12*solid.Fiil21*solid.Fiil33-solid.Fiil13*solid.Fiil	1	Inelastic deformation gradient, local coordinate system, 13-component	Domain 3	Matrix multiplication

Name	Expression	Unit	Description	Selection	Details
	22*solid.Fiil31)				
solid.FiI23	(solid.Fiil13*solid.Fiil21-solid.Fiil11*solid.Fiil23)/(solid.Fiil11*solid.Fiil22*solid.Fiil33+solid.Fiil12*solid.Fiil23*solid.Fiil31+solid.Fiil13*solid.Fiil21*solid.Fiil32-solid.Fiil11*solid.Fiil23*solid.Fiil32-solid.Fiil12*solid.Fiil21*solid.Fiil33-solid.Fiil13*solid.Fiil22*solid.Fiil31)	1	Inelastic deformation gradient, local coordinate system, 23-component	Domain 3	Matrix multiplication
solid.FiI33	(solid.Fiil11*solid.Fiil22-solid.Fiil12*solid.Fiil21)/(solid.Fiil11*solid.Fiil22*solid.Fiil33+solid.Fiil12*solid.Fiil23*solid.Fiil31+solid.Fiil13*solid.Fiil21*solid.Fiil32-solid.Fiil11*solid.Fiil23*solid.Fiil32-solid.Fiil12*solid.Fiil21*solid.Fiil33-solid.Fiil13*solid.Fiil22*solid.Fiil31)	1	Inelastic deformation gradient, local coordinate system, 33-component	Domain 3	Matrix multiplication
solid.Fiil11	1	1	Inelastic deformation gradient inverse, local coordinate system, 11-component	Domain 3	Matrix multiplication
solid.Fiil21	0	1	Inelastic deformation gradient inverse, local coordinate system, 21-component	Domain 3	Matrix multiplication
solid.Fiil31	0	1	Inelastic	Domain 3	Matrix

Name	Expression	Unit	Description	Selection	Details
			deformation gradient inverse, local coordinate system, 31-component		multiplication
solid.Fiil12	0	1	Inelastic deformation gradient inverse, local coordinate system, 12-component	Domain 3	Matrix multiplication
solid.Fiil22	1	1	Inelastic deformation gradient inverse, local coordinate system, 22-component	Domain 3	Matrix multiplication
solid.Fiil32	0	1	Inelastic deformation gradient inverse, local coordinate system, 32-component	Domain 3	Matrix multiplication
solid.Fiil13	0	1	Inelastic deformation gradient inverse, local coordinate system, 13-component	Domain 3	Matrix multiplication
solid.Fiil23	0	1	Inelastic deformation gradient inverse, local coordinate system, 23-component	Domain 3	Matrix multiplication
solid.Fiil33	1	1	Inelastic deformation gradient inverse, local	Domain 3	Matrix multiplication

Name	Expression	Unit	Description	Selection	Details
			coordinate system, 33-component		
solid.Felr1	solid.Fdlr1*solid.Fiil11+solid.Fdlr2*solid.Fiil21+solid.Fdlr3*solid.Fiil31	1	Elastic deformation gradient, local coordinate system, r1-component	Domain 3	
solid.Felphi1	solid.Fdlphi1*solid.Fiil11+solid.Fdlphi2*solid.Fiil21+solid.Fdlphi3*solid.Fiil31	1	Elastic deformation gradient, local coordinate system, phi1-component	Domain 3	
solid.Felz1	solid.Fdlz1*solid.Fiil11+solid.Fdlz2*solid.Fiil21+solid.Fdlz3*solid.Fiil31	1	Elastic deformation gradient, local coordinate system, z1-component	Domain 3	
solid.Felr2	solid.Fdlr1*solid.Fiil12+solid.Fdlr2*solid.Fiil22+solid.Fdlr3*solid.Fiil32	1	Elastic deformation gradient, local coordinate system, r2-component	Domain 3	
solid.Felphi2	solid.Fdlphi1*solid.Fiil12+solid.Fdlphi2*solid.Fiil22+solid.Fdlphi3*solid.Fiil32	1	Elastic deformation gradient, local coordinate system, phi2-component	Domain 3	
solid.Felz2	solid.Fdlz1*solid.Fiil12+solid.Fdlz2*solid.Fiil22+solid.Fdlz3*solid.Fiil32	1	Elastic deformation gradient, local coordinate system, z2-component	Domain 3	
solid.Felr3	solid.Fdlr1*solid.Fiil13+solid.Fdlr2*solid.Fiil23+solid.Fdlr3*solid.Fiil33	1	Elastic deformation gradient, local coordinate system, r3-component	Domain 3	

Name	Expression	Unit	Description	Selection	Details
solid.Felphi3	solid.Fdlphi1*solid.Fiil13+solid.Fdlphi2*solid.Fiil23+solid.Fdlphi3*solid.Fiil33	1	Elastic deformation gradient, local coordinate system, phi3-component	Domain 3	
solid.Felz3	solid.Fdlz1*solid.Fiil13+solid.Fdlz2*solid.Fiil23+solid.Fdlz3*solid.Fiil33	1	Elastic deformation gradient, local coordinate system, z3-component	Domain 3	
solid.Jel	sqrt(solid.Cel11*solid.Cel22*solid.Cel33+2*solid.Cel12*solid.Cel23*solid.Cel13-solid.Cel11*solid.Cel23^2-solid.Cel12^2*solid.Cel33-solid.Cel22*solid.Cel13^2)	1	Elastic volume ratio	Domain 3	
solid.CRR	solid.FdrR^2+solid.FdphiR^2+solid.FdzR^2	1	Cauchy-Green tensor, RR-component	Domain 3	
solid.CRPHI	solid.FdrR*solid.FdrPHI+solid.FdphiR*solid.FdphiPHI+solid.FdzR*solid.FdzPHI	1	Cauchy-Green tensor, RPHI-component	Domain 3	
solid.CRZ	solid.FdrR*solid.FdrZ+solid.FdphiR*solid.FdphiZ+solid.FdzR*solid.FdzZ	1	Cauchy-Green tensor, RZ-component	Domain 3	
solid.CPHIPHI	solid.FdrPHI^2+solid.FdphiPHI^2+solid.FdzPHI^2	1	Cauchy-Green tensor, PHIPHI-component	Domain 3	
solid.CPHIZ	solid.FdrPHI*solid.FdrZ+solid.FdphiPHI*solid.FdphiZ+solid.FdzPHI*solid.FdzZ	1	Cauchy-Green tensor, PHIZ-component	Domain 3	
solid.CZZ	solid.FdrZ^2+solid.FdphiZ^2+solid.FdzZ^2	1	Cauchy-Green tensor, ZZ-component	Domain 3	

Name	Expression	Unit	Description	Selection	Details
solid.Cl11	1+2*solid.el11	1	Cauchy-Green tensor, local coordinate system, 11-component	Domain 3	
solid.Cl12	2*solid.el12	1	Cauchy-Green tensor, local coordinate system, 12-component	Domain 3	
solid.Cl13	2*solid.el13	1	Cauchy-Green tensor, local coordinate system, 13-component	Domain 3	
solid.Cl22	1+2*solid.el22	1	Cauchy-Green tensor, local coordinate system, 22-component	Domain 3	
solid.Cl23	2*solid.el23	1	Cauchy-Green tensor, local coordinate system, 23-component	Domain 3	
solid.Cl33	1+2*solid.el33	1	Cauchy-Green tensor, local coordinate system, 33-component	Domain 3	
solid.Cel11	(solid.Fiil11*solid.Cl11+solid.Fiil21*solid.Cl12+solid.Fiil31*solid.Cl13)*solid.Fiil11+(solid.Fiil11*solid.Cl12+solid.Fiil21*solid.Cl22+solid.Fiil31*solid.Cl23)*solid.Fiil21+(solid.Fiil11*solid.Cl13+solid.Fiil21*solid.Cl23+solid.Fiil31*solid.Cl33)*solid.Fiil31	1	Elastic Cauchy-Green tensor, local coordinate system, 11-component	Domain 3	
solid.Cel12	(solid.Fiil11*solid.Cl11+solid.Fiil21*soli	1	Elastic Cauchy-Green tensor,	Domain 3	

Name	Expression	Unit	Description	Selection	Details
	d.Cl12+solid.Fiil31*solid.Cl13)*solid.Fiil12+(solid.Fiil11*solid.Cl12+solid.Fiil21*solid.Cl22+solid.Fiil31*solid.Cl23)*solid.Fiil22+(solid.Fiil11*solid.Cl13+solid.Fiil21*solid.Cl23+solid.Fiil31*solid.Cl33)*solid.Fiil32		local coordinate system, 12-component		
solid.Cel13	(solid.Fiil11*solid.Cl11+solid.Fiil21*solid.Cl12+solid.Fiil31*solid.Cl13)*solid.Fiil13+(solid.Fiil11*solid.Cl12+solid.Fiil21*solid.Cl22+solid.Fiil31*solid.Cl23)*solid.Fiil23+(solid.Fiil11*solid.Cl13+solid.Fiil21*solid.Cl23+solid.Fiil31*solid.Cl33)*solid.Fiil33	1	Elastic Cauchy-Green tensor, local coordinate system, 13-component	Domain 3	
solid.Cel22	(solid.Fiil12*solid.Cl11+solid.Fiil22*solid.Cl12+solid.Fiil32*solid.Cl13)*solid.Fiil12+(solid.Fiil12*solid.Cl12+solid.Fiil22*solid.Cl22+solid.Fiil32*solid.Cl23)*solid.Fiil22+(solid.Fiil12*solid.Cl13+solid.Fiil22*solid.Cl23+solid.Fiil32*solid.Cl33)*solid.Fiil32	1	Elastic Cauchy-Green tensor, local coordinate system, 22-component	Domain 3	
solid.Cel23	(solid.Fiil12*solid.Cl11+solid.Fiil22*solid.Cl12+solid.Fiil32*solid.Cl13)*solid.Fiil13+(solid.Fiil12*solid.Cl12+solid.Fiil22*solid.Cl22+solid.Fiil32*solid.Cl23)*solid.Fiil23+(solid.Fiil12*	1	Elastic Cauchy-Green tensor, local coordinate system, 23-component	Domain 3	

Name	Expression	Unit	Description	Selection	Details
	solid.Cl13+solid.Fiil22*solid.Cl23+solid.Fiil32*solid.Cl33)*solid.Fiil33				
solid.Cel33	(solid.Fiil13*solid.Cl11+solid.Fiil23*solid.Cl12+solid.Fiil33*solid.Cl13)*solid.Fiil13+(solid.Fiil13*solid.Cl12+solid.Fiil23*solid.Cl22+solid.Fiil33*solid.Cl23)*solid.Fiil23+(solid.Fiil13*solid.Cl13+solid.Fiil23*solid.Cl23+solid.Fiil33*solid.Cl33)*solid.Fiil33	1	Elastic Cauchy-Green tensor, local coordinate system, 33-component	Domain 3	
solid.Ceil11	(solid.Cel22*solid.Cel33-solid.Cel23^2)/(solid.Cel11*solid.Cel22*solid.Cel33+2*solid.Cel12*solid.Cel23*solid.Cel13-solid.Cel11*solid.Cel23^2-solid.Cel12^2*solid.Cel33-solid.Cel22*solid.Cel13^2)	1	Elastic Cauchy-Green tensor inverse, local coordinate system, 11-component	Domain 3	
solid.Ceil12	(solid.Cel23*solid.Cel13-solid.Cel12*solid.Cel33)/(solid.Cel11*solid.Cel22*solid.Cel33+2*solid.Cel12*solid.Cel23*solid.Cel13-solid.Cel11*solid.Cel23^2-solid.Cel12^2*solid.Cel33-solid.Cel22*solid.Cel13^2)	1	Elastic Cauchy-Green tensor inverse, local coordinate system, 12-component	Domain 3	
solid.Ceil13	(solid.Cel12*solid.Cel23-	1	Elastic Cauchy-Green tensor	Domain 3	

Name	Expression	Unit	Description	Selection	Details
	solid.Cel22*solid.Cel13)/(solid.Cel11*solid.Cel22*solid.Cel33+2*solid.Cel12*solid.Cel23*solid.Cel13-solid.Cel11*solid.Cel23^2-solid.Cel12^2*solid.Cel33-solid.Cel22*solid.Cel13^2)		inverse, local coordinate system, 13-component		
solid.Ceil22	(solid.Cel11*solid.Cel33-solid.Cel13^2)/(solid.Cel11*solid.Cel22*solid.Cel33+2*solid.Cel12*solid.Cel23*solid.Cel13-solid.Cel11*solid.Cel23^2-solid.Cel12^2*solid.Cel33-solid.Cel22*solid.Cel13^2)	1	Elastic Cauchy-Green tensor inverse, local coordinate system, 22-component	Domain 3	
solid.Ceil23	(solid.Cel12*solid.Cel13-solid.Cel11*solid.Cel23)/(solid.Cel11*solid.Cel22*solid.Cel33+2*solid.Cel12*solid.Cel23*solid.Cel13-solid.Cel11*solid.Cel23^2-solid.Cel12^2*solid.Cel33-solid.Cel22*solid.Cel13^2)	1	Elastic Cauchy-Green tensor inverse, local coordinate system, 23-component	Domain 3	
solid.Ceil33	(solid.Cel11*solid.Cel22-solid.Cel12^2)/(solid.Cel11*solid.Cel22*solid.Cel33+2*solid.Cel12*solid.Cel23*solid.Cel13-	1	Elastic Cauchy-Green tensor inverse, local coordinate system, 33-component	Domain 3	

Name	Expression	Unit	Description	Selection	Details
	solid.Cel11*solid.Cel23^2-solid.Cel12^2*solid.Cel33-solid.Cel22*solid.Cel13^2)				
solid.eRR	0.5*(2*solid.gradUrR+solid.gradUrR^2+solid.gradUphiR^2+solid.gradUzR^2)	1	Strain tensor, RR-component	Domain 3	+ operation
solid.eRPHI	0.5*(solid.gradUrPHI+solid.gradUphiR+solid.gradUrR*solid.gradUrPHI+solid.gradUphiR*solid.gradUphiPHI+solid.gradUzR*solid.gradUzPHI)	1	Strain tensor, RPHI-component	Domain 3	+ operation
solid.eRZ	0.5*(solid.gradUrZ+solid.gradUzR+solid.gradUrR*solid.gradUrZ+solid.gradUphiR*solid.gradUphiZ+solid.gradUzR*solid.gradUzZ)	1	Strain tensor, RZ-component	Domain 3	+ operation
solid.ePHIPHI	0.5*(2*solid.gradUphiPHI+solid.gradUrPHI^2+solid.gradUphiPHI^2+solid.gradUzPHI^2)	1	Strain tensor, PHIPHI-component	Domain 3	+ operation
solid.ePHIZ	0.5*(solid.gradUphiZ+solid.gradUzPHI+solid.gradUrPHI*solid.gradUrZ+solid.gradUphiPHI*solid.gradUphiZ+solid.gradUzPHI*solid.gradUzZ)	1	Strain tensor, PHIZ-component	Domain 3	+ operation
solid.eZZ	0.5*(2*solid.gradUzZ+solid.gradUrZ^2+solid.gradUphiZ^2+solid.gradUzZ^2)	1	Strain tensor, ZZ-component	Domain 3	+ operation
solid.el11	solid.eRR	1	Strain tensor,	Domain 3	+ operation

Name	Expression	Unit	Description	Selection	Details
			local coordinate system, 11-component		
solid.el12	solid.eRPHI	1	Strain tensor, local coordinate system, 12-component	Domain 3	+ operation
solid.el13	solid.eRZ	1	Strain tensor, local coordinate system, 13-component	Domain 3	+ operation
solid.el22	solid.ePHIPHI	1	Strain tensor, local coordinate system, 22-component	Domain 3	+ operation
solid.el23	solid.ePHIZ	1	Strain tensor, local coordinate system, 23-component	Domain 3	+ operation
solid.el33	solid.eZZ	1	Strain tensor, local coordinate system, 33-component	Domain 3	+ operation
solid.evol	solid.eRR+solid.ePHIPHI+solid.eZZ	1	Volumetric strain	Domain 3	
solid.eldev11	solid.el11-solid.evol/3	1	Deviatoric strain tensor, local coordinate system, 11-component	Domain 3	
solid.eldev12	solid.el12	1	Deviatoric strain tensor, local coordinate system, 12-component	Domain 3	
solid.eldev13	solid.el13	1	Deviatoric strain tensor, local coordinate system, 13-component	Domain 3	
solid.eldev22	solid.el22-solid.evol/3	1	Deviatoric strain tensor, local coordinate system, 22-	Domain 3	

Name	Expression	Unit	Description	Selection	Details
			component		
solid.eldev23	solid.el23	1	Deviatoric strain tensor, local coordinate system, 23-component	Domain 3	
solid.eldev33	solid.el33-solid.evol/3	1	Deviatoric strain tensor, local coordinate system, 33-component	Domain 3	
solid.edeve	sqrt(2*(solid.eldev11^2+2*solid.eldev12^2+2*solid.eldev13^2+solid.eldev22^2+2*solid.eldev23^2+solid.eldev33^2)/3+eps^2)	1	Equivalent deviatoric strain	Domain 3	
solid.eel11	0.5*(-1+solid.Cel11)	1	Elastic strain tensor, local coordinate system, 11-component	Domain 3	
solid.eel12	0.5*solid.Cel12	1	Elastic strain tensor, local coordinate system, 12-component	Domain 3	
solid.eel13	0.5*solid.Cel13	1	Elastic strain tensor, local coordinate system, 13-component	Domain 3	
solid.eel22	0.5*(-1+solid.Cel22)	1	Elastic strain tensor, local coordinate system, 22-component	Domain 3	
solid.eel23	0.5*solid.Cel23	1	Elastic strain tensor, local coordinate system, 23-component	Domain 3	
solid.eel33	0.5*(-1+solid.Cel33)	1	Elastic strain	Domain 3	

Name	Expression	Unit	Description	Selection	Details
			tensor, local coordinate system, 33-component		
solid.eil11	0	1	Initial strain tensor, local coordinate system, 11-component	Domain 3	+ operation
solid.eil12	0	1	Initial strain tensor, local coordinate system, 12-component	Domain 3	+ operation
solid.eil13	0	1	Initial strain tensor, local coordinate system, 13-component	Domain 3	+ operation
solid.eil22	0	1	Initial strain tensor, local coordinate system, 22-component	Domain 3	+ operation
solid.eil23	0	1	Initial strain tensor, local coordinate system, 23-component	Domain 3	+ operation
solid.eil33	0	1	Initial strain tensor, local coordinate system, 33-component	Domain 3	+ operation
solid.eiel11	solid.eil11	1	Inelastic strain tensor, local coordinate system, 11-component	Domain 3	+ operation
solid.eiel12	solid.eil12	1	Inelastic strain tensor, local coordinate system, 12-component	Domain 3	+ operation

Name	Expression	Unit	Description	Selection	Details
solid.eiel13	solid.eil13	1	Inelastic strain tensor, local coordinate system, 13-component	Domain 3	+ operation
solid.eiel22	solid.eil22	1	Inelastic strain tensor, local coordinate system, 22-component	Domain 3	+ operation
solid.eiel23	solid.eil23	1	Inelastic strain tensor, local coordinate system, 23-component	Domain 3	+ operation
solid.eiel33	solid.eil33	1	Inelastic strain tensor, local coordinate system, 33-component	Domain 3	+ operation
solid.II2eel	0.5*(solid.eeldev11^2+2*solid.eeldev12^2+2*solid.eeldev13^2+solid.eeldev22^2+2*solid.eeldev23^2+solid.eeldev33^2)	1	Second invariant of deviatoric elastic strain tensor	Domain 3	
solid.eelvol	solid.eel11+solid.eel22+solid.eel33	1	First invariant of elastic strain tensor	Domain 3	
solid.eeldev11	solid.eel11-(solid.eel11+solid.eel22+solid.eel33)/3	1	Deviatoric elastic strain tensor, local coordinate system, 11-component	Domain 3	
solid.eeldev12	solid.eel12	1	Deviatoric elastic strain tensor, local coordinate system, 12-component	Domain 3	
solid.eeldev13	solid.eel13	1	Deviatoric elastic strain tensor, local	Domain 3	

Name	Expression	Unit	Description	Selection	Details
			coordinate system, 13-component		
solid.eeldev22	solid.eel22-(solid.eel11+solid.eel22+solid.eel33)/3	1	Deviatoric elastic strain tensor, local coordinate system, 22-component	Domain 3	
solid.eeldev23	solid.eel23	1	Deviatoric elastic strain tensor, local coordinate system, 23-component	Domain 3	
solid.eeldev33	solid.eel33-(solid.eel11+solid.eel22+solid.eel33)/3	1	Deviatoric elastic strain tensor, local coordinate system, 33-component	Domain 3	
solid.eeRR	solid.eel11	1	Elastic strain tensor, local coordinate system,	Domain 3	
solid.eeRPHI	solid.eel12	1	Elastic strain tensor, local coordinate system,	Domain 3	
solid.eeRZ	solid.eel13	1	Elastic strain tensor, local coordinate system,	Domain 3	
solid.eePHIPHI	solid.eel22	1	Elastic strain tensor, local coordinate system,	Domain 3	
solid.eePHIZ	solid.eel23	1	Elastic strain tensor, local coordinate system,	Domain 3	
solid.eeZZ	solid.eel33	1	Elastic strain tensor, local coordinate	Domain 3	

Name	Expression	Unit	Description	Selection	Details
			system,		
solid.sel11	solid.D11*solid.eel11+solid.D12*solid.eel22+solid.D13*solid.eel33+2*solid.D14*solid.eel12+2*solid.D15*solid.eel23+2*solid.D16*solid.eel13	N/m²	Elastic stress tensor, local coordinate system, 11-component	Domain 3	
solid.sel12	solid.D14*solid.eel11+solid.D24*solid.eel22+solid.D34*solid.eel33+2*solid.D44*solid.eel12+2*solid.D45*solid.eel23+2*solid.D46*solid.eel13	N/m²	Elastic stress tensor, local coordinate system, 12-component	Domain 3	
solid.sel13	solid.D16*solid.eel11+solid.D26*solid.eel22+solid.D36*solid.eel33+2*solid.D46*solid.eel12+2*solid.D56*solid.eel23+2*solid.D66*solid.eel13	N/m²	Elastic stress tensor, local coordinate system, 13-component	Domain 3	
solid.sel22	solid.D12*solid.eel11+solid.D22*solid.eel22+solid.D23*solid.eel33+2*solid.D24*solid.eel12+2*solid.D25*solid.eel23+2*solid.D26*solid.eel13	N/m²	Elastic stress tensor, local coordinate system, 22-component	Domain 3	
solid.sel23	solid.D15*solid.eel11+solid.D25*solid.eel22+solid.D35*solid.eel33+2*solid.D45*solid.eel12+2*solid.D55*solid.eel23+2*solid.D56*solid.eel13	N/m²	Elastic stress tensor, local coordinate system, 23-component	Domain 3	
solid.sel33	solid.D13*solid.eel11+solid.D23*solid.eel22+solid.D33*solid.eel33+2*solid.D3	N/m²	Elastic stress tensor, local coordinate system, 33-	Domain 3	

Name	Expression	Unit	Description	Selection	Details
	4*solid.eel12+2*solid.D35*solid.eel23+2*solid.D36*solid.eel13		component		
solid.siel11	((solid.Fil11*solid.Siel11+solid.Fil12*solid.Siel12+solid.Fil13*solid.Siel13)*solid.Fil11+(solid.Fil11*solid.Siel12+solid.Fil12*solid.Siel22+solid.Fil13*solid.Siel23)*solid.Fil12+(solid.Fil11*solid.Siel13+solid.Fil12*solid.Siel23+solid.Fil13*solid.Siel33)*solid.Fil13)/solid.Ji	N/m²	Inelastic stress tensor, local coordinate system, 11-component	Domain 3	
solid.siel12	((solid.Fil11*solid.Siel11+solid.Fil12*solid.Siel12+solid.Fil13*solid.Siel13)*solid.Fil21+(solid.Fil11*solid.Siel12+solid.Fil12*solid.Siel22+solid.Fil13*solid.Siel23)*solid.Fil22+(solid.Fil11*solid.Siel13+solid.Fil12*solid.Siel23+solid.Fil13*solid.Siel33)*solid.Fil23)/solid.Ji	N/m²	Inelastic stress tensor, local coordinate system, 12-component	Domain 3	
solid.siel13	((solid.Fil11*solid.Siel11+solid.Fil12*solid.Siel12+solid.Fil13*solid.Siel13)*solid.Fil31+(solid.Fil11*solid.Siel12+solid.Fil12*solid.Siel22+solid.Fil13*solid.Siel23)*solid.Fil32+(solid.Fil11*solid.Siel13+solid.Fil12*solid.Siel23+solid.Fil13*solid.Siel33)*solid.Fil33)/solid.Ji	N/m²	Inelastic stress tensor, local coordinate system, 13-component	Domain 3	

Name	Expression	Unit	Description	Selection	Details
solid.siel22	((solid.Fil21*solid.Siel11+solid.Fil22*solid.Siel12+solid.Fil23*solid.Siel13)*solid.Fil21+(solid.Fil21*solid.Siel12+solid.Fil22*solid.Siel22+solid.Fil23*solid.Siel23)*solid.Fil22+(solid.Fil21*solid.Siel13+solid.Fil22*solid.Siel23+solid.Fil23*solid.Siel33)*solid.Fil23)/solid.Ji	N/m²	Inelastic stress tensor, local coordinate system, 22-component	Domain 3	
solid.siel23	((solid.Fil21*solid.Siel11+solid.Fil22*solid.Siel12+solid.Fil23*solid.Siel13)*solid.Fil31+(solid.Fil21*solid.Siel12+solid.Fil22*solid.Siel22+solid.Fil23*solid.Siel23)*solid.Fil32+(solid.Fil21*solid.Siel13+solid.Fil22*solid.Siel23+solid.Fil23*solid.Siel33)*solid.Fil33)/solid.Ji	N/m²	Inelastic stress tensor, local coordinate system, 23-component	Domain 3	
solid.siel33	((solid.Fil31*solid.Siel11+solid.Fil32*solid.Siel12+solid.Fil33*solid.Siel13)*solid.Fil31+(solid.Fil31*solid.Siel12+solid.Fil32*solid.Siel22+solid.Fil33*solid.Siel23)*solid.Fil32+(solid.Fil31*solid.Siel13+solid.Fil32*solid.Siel23+solid.Fil33*solid.Siel33)*solid.Fil33)/solid.Ji	N/m²	Inelastic stress tensor, local coordinate system, 33-component	Domain 3	
solid.srr	((solid.Fdlr1*solid.Sl11+solid.Fdlr2*solid.Sl12+solid.Fdlr3*solid.Sl13)*solid.Fdlr	N/m²	Stress tensor, rr-component	Domain 3	

Name	Expression	Unit	Description	Selection	Details
	1+(solid.Fdlr1*solid.Sl12+solid.Fdlr2*solid.Sl22+solid.Fdlr3*solid.Sl23)*solid.Fdlr2+(solid.Fdlr1*solid.Sl13+solid.Fdlr2*solid.Sl23+solid.Fdlr3*solid.Sl33)*solid.Fdlr3)/solid.J				
solid.srphi	((solid.Fdlr1*solid.Sl11+solid.Fdlr2*solid.Sl12+solid.Fdlr3*solid.Sl13)*solid.Fdlphi1+(solid.Fdlr1*solid.Sl12+solid.Fdlr2*solid.Sl22+solid.Fdlr3*solid.Sl23)*solid.Fdlphi2+(solid.Fdlr1*solid.Sl13+solid.Fdlr2*solid.Sl23+solid.Fdlr3*solid.Sl33)*solid.Fdlphi3)/solid.J	N/m²	Stress tensor, rphi-component	Domain 3	
solid.srz	((solid.Fdlr1*solid.Sl11+solid.Fdlr2*solid.Sl12+solid.Fdlr3*solid.Sl13)*solid.Fdlz1+(solid.Fdlr1*solid.Sl12+solid.Fdlr2*solid.Sl22+solid.Fdlr3*solid.Sl23)*solid.Fdlz2+(solid.Fdlr1*solid.Sl13+solid.Fdlr2*solid.Sl23+solid.Fdlr3*solid.Sl33)*solid.Fdlz3)/solid.J	N/m²	Stress tensor, rz-component	Domain 3	
solid.sphiphi	((solid.Fdlphi1*solid.Sl11+solid.Fdlphi2*solid.Sl12+solid.Fdlphi3*solid.Sl13)*solid.Fdlphi1+(solid.Fdlphi1*solid.Sl12+solid.Fdlphi2*solid.Sl22+solid.Fdlphi3*solid.Sl23)*solid.Fdlphi2+(solid.Fdlphi1*solid.Sl13+solid.Fdlphi	N/m²	Stress tensor, phiphi-component	Domain 3	

Name	Expression	Unit	Description	Selection	Details
	2*solid.Sl23+solid.Fdlphi3*solid.Sl33)*solid.Fdlphi3)/solid.J				
solid.sphiz	((solid.Fdlphi1*solid.Sl11+solid.Fdlphi2*solid.Sl12+solid.Fdlphi3*solid.Sl13)*solid.Fdlz1+(solid.Fdlphi1*solid.Sl12+solid.Fdlphi2*solid.Sl22+solid.Fdlphi3*solid.Sl23)*solid.Fdlz2+(solid.Fdlphi1*solid.Sl13+solid.Fdlphi2*solid.Sl23+solid.Fdlphi3*solid.Sl33)*solid.Fdlz3)/solid.J	N/m²	Stress tensor, phiz-component	Domain 3	
solid.szz	((solid.Fdlz1*solid.Sl11+solid.Fdlz2*solid.Sl12+solid.Fdlz3*solid.Sl13)*solid.Fdlz1+(solid.Fdlz1*solid.Sl12+solid.Fdlz2*solid.Sl22+solid.Fdlz3*solid.Sl23)*solid.Fdlz2+(solid.Fdlz1*solid.Sl13+solid.Fdlz2*solid.Sl23+solid.Fdlz3*solid.Sl33)*solid.Fdlz3)/solid.J	N/m²	Stress tensor, zz-component	Domain 3	
solid.sl11	solid.srr	N/m²	Stress tensor, local coordinate system, 11-component	Domain 3	
solid.sl12	solid.srphi	N/m²	Stress tensor, local coordinate system, 12-component	Domain 3	
solid.sl13	solid.srz	N/m²	Stress tensor, local coordinate system, 13-component	Domain 3	
solid.sl22	solid.sphiphi	N/m²	Stress tensor, local coordinate system, 22-	Domain 3	

Name	Expression	Unit	Description	Selection	Details
			component		
solid.sl23	solid.sphiz	N/m²	Stress tensor, local coordinate system, 23-component	Domain 3	
solid.sl33	solid.szz	N/m²	Stress tensor, local coordinate system, 33-component	Domain 3	
solid.sdevrr	solid.srr-(solid.srr+solid.sphiphi+solid.szz)/3	N/m²	Deviatoric stress tensor, rr-component	Domain 3	
solid.sdevrphi	solid.srphi	N/m²	Deviatoric stress tensor, rphi-component	Domain 3	
solid.sdevrz	solid.srz	N/m²	Deviatoric stress tensor, rz-component	Domain 3	
solid.sdevphiphi	solid.sphiphi-(solid.srr+solid.sphiphi+solid.szz)/3	N/m²	Deviatoric stress tensor, phiphi-component	Domain 3	
solid.sdevphiz	solid.sphiz	N/m²	Deviatoric stress tensor, phiz-component	Domain 3	
solid.sdevzz	solid.szz-(solid.srr+solid.sphiphi+solid.szz)/3	N/m²	Deviatoric stress tensor, zz-component	Domain 3	
solid.sldev11	solid.sl11-(solid.sl11+solid.sl22+solid.sl33)/3	N/m²	Deviatoric stress tensor, local coordinate system, 11-component	Domain 3	
solid.sldev12	solid.sl12	N/m²	Deviatoric stress tensor, local coordinate system, 12-component	Domain 3	
solid.sldev13	solid.sl13	N/m²	Deviatoric stress tensor, local coordinate system, 13-component	Domain 3	
solid.sldev22	solid.sl22-	N/m²	Deviatoric stress	Domain 3	

Name	Expression	Unit	Description	Selection	Details
	(solid.sl11+solid.sl22+solid.sl33)/3		tensor, local coordinate system, 22-component		
solid.sldev23	solid.sl23	N/m²	Deviatoric stress tensor, local coordinate system, 23-component	Domain 3	
solid.sldev33	solid.sl33-(solid.sl11+solid.sl22+solid.sl33)/3	N/m²	Deviatoric stress tensor, local coordinate system, 33-component	Domain 3	
solid.I1s	solid.sl11+solid.sl22+solid.sl33	N/m²	First principal invariant of stress	Domain 3	
solid.I2s	0.5*(solid.I1s^2-solid.sl11^2-2*solid.sl12^2-2*solid.sl13^2-solid.sl22^2-2*solid.sl23^2-solid.sl33^2)	$kg^2/(m^2 \cdot s^4)$	Second principal invariant of stress	Domain 3	
solid.I3s	solid.sl11*solid.sl22*solid.sl33+2*solid.sl12*solid.sl23*solid.sl13-solid.sl11*solid.sl23^2-solid.sl12^2*solid.sl33-solid.sl22*solid.sl13^2	$kg^3/(m^3 \cdot s^6)$	Third principal invariant of stress	Domain 3	
solid.II2s	0.5*(solid.sldev11^2+2*solid.sldev12^2+2*solid.sldev13^2+solid.sldev22^2+2*solid.sldev23^2+solid.sldev33^2)	$kg^2/(m^2 \cdot s^4)$	Second invariant of stress deviator	Domain 3	
solid.II3s	solid.sldev11*solid.sldev22*solid.sldev33+2*solid.sldev12*solid.sldev23*solid.sldev13-	$kg^3/(m^3 \cdot s^6)$	Third invariant of stress deviator	Domain 3	

Name	Expression	Unit	Description	Selection	Details
	solid.sldev11*solid.sldev23^2-solid.sldev12^2*solid.sldev33-solid.sldev22*solid.sldev13^2				
solid.Sel11	solid.Ji*((solid.Fiil11*solid.sel11+solid.Fiil12*solid.sel12+solid.Fiil13*solid.sel13)*solid.Fiil11+(solid.Fiil11*solid.sel12+solid.Fiil12*solid.sel22+solid.Fiil13*solid.sel23)*solid.Fiil12+(solid.Fiil11*solid.sel13+solid.Fiil12*solid.sel23+solid.Fiil13*solid.sel33)*solid.Fiil13)	N/m²	Elastic second Piola-Kirchhoff stress, local coordinate system, 11-component	Domain 3	
solid.Sel12	solid.Ji*((solid.Fiil11*solid.sel11+solid.Fiil12*solid.sel12+solid.Fiil13*solid.sel13)*solid.Fiil21+(solid.Fiil11*solid.sel12+solid.Fiil12*solid.sel22+solid.Fiil13*solid.sel23)*solid.Fiil22+(solid.Fiil11*solid.sel13+solid.Fiil12*solid.sel23+solid.Fiil13*solid.sel33)*solid.Fiil23)	N/m²	Elastic second Piola-Kirchhoff stress, local coordinate system, 12-component	Domain 3	
solid.Sel13	solid.Ji*((solid.Fiil11*solid.sel11+solid.Fiil12*solid.sel12+solid.Fiil13*solid.sel13)*solid.Fiil31+(solid.Fiil11*solid.sel12+solid.Fiil12*solid.sel22+solid.Fiil13*solid.sel23)*solid.Fiil32+(solid.Fiil11*solid.sel13+solid.Fiil12*solid.sel23+solid.Fiil13	N/m²	Elastic second Piola-Kirchhoff stress, local coordinate system, 13-component	Domain 3	

Name	Expression	Unit	Description	Selection	Details
	*solid.sel33)*solid.Fiil33)				
solid.Sel22	solid.Ji*((solid.Fiil21*solid.sel11+solid.Fiil22*solid.sel12+solid.Fiil23*solid.sel13)*solid.Fiil21+(solid.Fiil21*solid.sel12+solid.Fiil22*solid.sel22+solid.Fiil23*solid.sel23)*solid.Fiil22+(solid.Fiil21*solid.sel13+solid.Fiil22*solid.sel23+solid.Fiil23*solid.sel33)*solid.Fiil23)	N/m²	Elastic second Piola-Kirchhoff stress, local coordinate system, 22-component	Domain 3	
solid.Sel23	solid.Ji*((solid.Fiil21*solid.sel11+solid.Fiil22*solid.sel12+solid.Fiil23*solid.sel13)*solid.Fiil31+(solid.Fiil21*solid.sel12+solid.Fiil22*solid.sel22+solid.Fiil23*solid.sel23)*solid.Fiil32+(solid.Fiil21*solid.sel13+solid.Fiil22*solid.sel23+solid.Fiil23*solid.sel33)*solid.Fiil33)	N/m²	Elastic second Piola-Kirchhoff stress, local coordinate system, 23-component	Domain 3	
solid.Sel33	solid.Ji*((solid.Fiil31*solid.sel11+solid.Fiil32*solid.sel12+solid.Fiil33*solid.sel13)*solid.Fiil31+(solid.Fiil31*solid.sel12+solid.Fiil32*solid.sel22+solid.Fiil33*solid.sel23)*solid.Fiil32+(solid.Fiil31*solid.sel13+solid.Fiil32*solid.sel23+solid.Fiil33*solid.sel33)*solid.Fiil33)	N/m²	Elastic second Piola-Kirchhoff stress, local coordinate system, 33-component	Domain 3	
solid.Siel11	0	N/m²	Inelastic second Piola-Kirchhoff	Domain 3	+ operation

Name	Expression	Unit	Description	Selection	Details
			stress tensor, local coordinate system, 11-component		
solid.Siel12	0	N/m²	Inelastic second Piola-Kirchhoff stress tensor, local coordinate system, 12-component	Domain 3	+ operation
solid.Siel13	0	N/m²	Inelastic second Piola-Kirchhoff stress tensor, local coordinate system, 13-component	Domain 3	+ operation
solid.Siel22	0	N/m²	Inelastic second Piola-Kirchhoff stress tensor, local coordinate system, 22-component	Domain 3	+ operation
solid.Siel23	0	N/m²	Inelastic second Piola-Kirchhoff stress tensor, local coordinate system, 23-component	Domain 3	+ operation
solid.Siel33	0	N/m²	Inelastic second Piola-Kirchhoff stress tensor, local coordinate system, 33-component	Domain 3	+ operation
solid.Sl11	solid.Siel11+solid.Sel11+(solid.pp-solid.pw)*solid.Ji*(solid.Fiil11^2+solid.Fiil12^2+solid.Fiil13^2)	N/m²	Second Piola-Kirchhoff stress, local coordinate system, 11-component	Domain 3	+ operation
solid.Sl12	solid.Siel12+solid.Sel12+(solid.pp-solid.pw)*solid.Ji*(solid.Fiil11*solid.Fiil21+solid.Fiil12*solid.	N/m²	Second Piola-Kirchhoff stress, local coordinate system, 12-component	Domain 3	+ operation

Name	Expression	Unit	Description	Selection	Details
	Fiil22+solid.Fiil13*solid.Fiil23)				
solid.Sl13	solid.Siel13+solid.Sel13+(solid.pp-solid.pw)*solid.Ji*(solid.Fiil11*solid.Fiil31+solid.Fiil12*solid.Fiil32+solid.Fiil13*solid.Fiil33)	N/m²	Second Piola-Kirchhoff stress, local coordinate system, 13-component	Domain 3	+ operation
solid.Sl22	solid.Siel22+solid.Sel22+(solid.pp-solid.pw)*solid.Ji*(solid.Fiil21^2+solid.Fiil22^2+solid.Fiil23^2)	N/m²	Second Piola-Kirchhoff stress, local coordinate system, 22-component	Domain 3	+ operation
solid.Sl23	solid.Siel23+solid.Sel23+(solid.pp-solid.pw)*solid.Ji*(solid.Fiil21*solid.Fiil31+solid.Fiil22*solid.Fiil32+solid.Fiil23*solid.Fiil33)	N/m²	Second Piola-Kirchhoff stress, local coordinate system, 23-component	Domain 3	+ operation
solid.Sl33	solid.Siel33+solid.Sel33+(solid.pp-solid.pw)*solid.Ji*(solid.Fiil31^2+solid.Fiil32^2+solid.Fiil33^2)	N/m²	Second Piola-Kirchhoff stress, local coordinate system, 33-component	Domain 3	+ operation
solid.SRR	solid.Sl11	N/m²	Second Piola-Kirchhoff stress, RR-component	Domain 3	
solid.SRPHI	solid.Sl12	N/m²	Second Piola-Kirchhoff stress, RPHI-component	Domain 3	
solid.SRZ	solid.Sl13	N/m²	Second Piola-Kirchhoff stress, RZ-component	Domain 3	
solid.SPHIPHI	solid.Sl22	N/m²	Second Piola-Kirchhoff stress, PHIPHI-component	Domain 3	
solid.SPHIZ	solid.Sl23	N/m²	Second Piola-Kirchhoff stress,	Domain 3	

Name	Expression	Unit	Description	Selection	Details
			PHIZ-component		
solid.SZZ	solid.Sl33	N/m²	Second Piola-Kirchhoff stress, ZZ-component	Domain 3	
solid.SdevRR	solid.SRR-(solid.SRR+solid.SPHIPHI+solid.SZZ)/3	N/m²	Deviatoric second Piola-Kirchhoff stress, RR-component	Domain 3	
solid.SdevRPHI	solid.SRPHI	N/m²	Deviatoric second Piola-Kirchhoff stress, RPHI-component	Domain 3	
solid.SdevRZ	solid.SRZ	N/m²	Deviatoric second Piola-Kirchhoff stress, RZ-component	Domain 3	
solid.SdevPHIPHI	solid.SPHIPHI-(solid.SRR+solid.SPHIPHI+solid.SZZ)/3	N/m²	Deviatoric second Piola-Kirchhoff stress, PHIPHI-component	Domain 3	
solid.SdevPHIZ	solid.SPHIZ	N/m²	Deviatoric second Piola-Kirchhoff stress, PHIZ-component	Domain 3	
solid.SdevZZ	solid.SZZ-(solid.SRR+solid.SPHIPHI+solid.SZZ)/3	N/m²	Deviatoric second Piola-Kirchhoff stress, ZZ-component	Domain 3	
solid.PrR	solid.FdrR*solid.SRR+solid.FdrPHI*solid.SRPHI+solid.FdrZ*solid.SRZ	N/m²	First Piola-Kirchhoff stress, rR-component	Domain 3	
solid.PphiR	solid.FdphiR*solid.SRR+solid.FdphiPHI*solid.SRPHI+solid.FdphiZ*solid.SRZ	N/m²	First Piola-Kirchhoff stress, phiR-component	Domain 3	
solid.PzR	solid.FdzR*solid.SRR+solid.FdzPHI*solid.SRPHI+solid.FdzZ*solid.SRZ	N/m²	First Piola-Kirchhoff stress, zR-component	Domain 3	

Name	Expression	Unit	Description	Selection	Details
solid.PrPHI	solid.FdrR*solid.SRPHI+solid.FdrPHI*solid.SPHIPHI+solid.FdrZ*solid.SPHIZ	N/m²	First Piola-Kirchhoff stress, rPHI-component	Domain 3	
solid.PphiPHI	solid.FdphiR*solid.SRPHI+solid.FdphiPHI*solid.SPHIPHI+solid.FdphiZ*solid.SPHIZ	N/m²	First Piola-Kirchhoff stress, phiPHI-component	Domain 3	
solid.PzPHI	solid.FdzR*solid.SRPHI+solid.FdzPHI*solid.SPHIPHI+solid.FdzZ*solid.SPHIZ	N/m²	First Piola-Kirchhoff stress, zPHI-component	Domain 3	
solid.PrZ	solid.FdrR*solid.SRZ+solid.FdrPHI*solid.SPHIZ+solid.FdrZ*solid.SZZ	N/m²	First Piola-Kirchhoff stress, rZ-component	Domain 3	
solid.PphiZ	solid.FdphiR*solid.SRZ+solid.FdphiPHI*solid.SPHIZ+solid.FdphiZ*solid.SZZ	N/m²	First Piola-Kirchhoff stress, phiZ-component	Domain 3	
solid.PzZ	solid.FdzR*solid.SRZ+solid.FdzPHI*solid.SPHIZ+solid.FdzZ*solid.SZZ	N/m²	First Piola-Kirchhoff stress, zZ-component	Domain 3	
solid.Sil11	0	N/m²	Initial stress tensor, local coordinate system, 11-component	Domain 3	+ operation
solid.Sil12	0	N/m²	Initial stress tensor, local coordinate system, 12-component	Domain 3	+ operation
solid.Sil13	0	N/m²	Initial stress tensor, local coordinate system, 13-component	Domain 3	+ operation
solid.Sil22	0	N/m²	Initial stress tensor, local coordinate system, 22-	Domain 3	+ operation

Name	Expression	Unit	Description	Selection	Details
			component		
solid.Sil23	0	N/m²	Initial stress tensor, local coordinate system, 23-component	Domain 3	+ operation
solid.Sil33	0	N/m²	Initial stress tensor, local coordinate system, 33-component	Domain 3	+ operation
solid.mises	sqrteps(3*solid.II2s)	N/m²	von Mises stress	Domain 3	
solid.pm	-(solid.sl11+solid.sl22+solid.sl33)/3	N/m²	Pressure	Domain 3	
solid.pw	solid.lemm1.pw	N/m²	Auxiliary pressure	Domain 3	
solid.SlGp11	solid.gpeval(solid.Sl11)	N/m²	Second Piola-Kirchhoff stress, local coordinate system, Gauss point evaluation, 11-component	Domain 3	
solid.SlGp12	solid.gpeval(solid.Sl12)	N/m²	Second Piola-Kirchhoff stress, local coordinate system, Gauss point evaluation, 12-component	Domain 3	
solid.SlGp13	solid.gpeval(solid.Sl13)	N/m²	Second Piola-Kirchhoff stress, local coordinate system, Gauss point evaluation, 13-component	Domain 3	
solid.SlGp22	solid.gpeval(solid.Sl22)	N/m²	Second Piola-Kirchhoff stress, local coordinate system, Gauss point evaluation, 22-	Domain 3	

Name	Expression	Unit	Description	Selection	Details
			component		
solid.SlGp23	solid.gpeval(solid.Sl 23)	N/m²	Second Piola-Kirchhoff stress, local coordinate system, Gauss point evaluation, 23-component	Domain 3	
solid.SlGp33	solid.gpeval(solid.Sl 33)	N/m²	Second Piola-Kirchhoff stress, local coordinate system, Gauss point evaluation, 33-component	Domain 3	
solid.misesGp	max(0,solid.gpeval(solid.mises))	N/m²	von Mises stress, Gauss point evaluation	Domain 3	
solid.trescaGp	max(0,solid.gpeval(solid.tresca))	N/m²	Tresca stress, Gauss point evaluation	Domain 3	
solid.SGpRR	solid.gpeval(solid.S RR)	N/m²	Second Piola-Kirchhoff stress, Gauss point evaluation, RR-component	Domain 3	
solid.SGpRPHI	solid.gpeval(solid.S RPHI)	N/m²	Second Piola-Kirchhoff stress, Gauss point evaluation, RPHI-component	Domain 3	
solid.SGpRZ	solid.gpeval(solid.S RZ)	N/m²	Second Piola-Kirchhoff stress, Gauss point evaluation, RZ-component	Domain 3	
solid.SGpPHIPHI	solid.gpeval(solid.S PHIPHI)	N/m²	Second Piola-Kirchhoff stress, Gauss point evaluation, PHIPHI-component	Domain 3	
solid.SGpPHIZ	solid.gpeval(solid.S	N/m²	Second Piola-	Domain 3	

Name	Expression	Unit	Description	Selection	Details
	PHIZ)		Kirchhoff stress, Gauss point evaluation, PHIZ-component		
solid.SGpZZ	solid.gpeval(solid.SZZ)	N/m²	Second Piola-Kirchhoff stress, Gauss point evaluation, ZZ-component	Domain 3	
solid.SdevGpRR	solid.gpeval(solid.SdevRR)	N/m²	Deviatoric second Piola-Kirchhoff stress, Gauss point evaluation, RR-component	Domain 3	
solid.SdevGpRPHI	solid.gpeval(solid.SdevRPHI)	N/m²	Deviatoric second Piola-Kirchhoff stress, Gauss point evaluation, RPHI-component	Domain 3	
solid.SdevGpRZ	solid.gpeval(solid.SdevRZ)	N/m²	Deviatoric second Piola-Kirchhoff stress, Gauss point evaluation, RZ-component	Domain 3	
solid.SdevGpPHIPHI	solid.gpeval(solid.SdevPHIPHI)	N/m²	Deviatoric second Piola-Kirchhoff stress, Gauss point evaluation, PHIPHI-component	Domain 3	
solid.SdevGpPHIZ	solid.gpeval(solid.SdevPHIZ)	N/m²	Deviatoric second Piola-Kirchhoff stress, Gauss point evaluation, PHIZ-component	Domain 3	
solid.SdevGpZZ	solid.gpeval(solid.SdevZZ)	N/m²	Deviatoric second Piola-	Domain 3	

Name	Expression	Unit	Description	Selection	Details
			Kirchhoff stress, Gauss point evaluation, ZZ-component		
solid.sGprr	solid.gpeval(solid.sr r)	N/m²	Stress tensor, Gauss point evaluation, rr-component	Domain 3	
solid.sGprphi	solid.gpeval(solid.sr phi)	N/m²	Stress tensor, Gauss point evaluation, rphi-component	Domain 3	
solid.sGprz	solid.gpeval(solid.sr z)	N/m²	Stress tensor, Gauss point evaluation, rz-component	Domain 3	
solid.sGpphiphi	solid.gpeval(solid.s phiphi)	N/m²	Stress tensor, Gauss point evaluation, phiphi-component	Domain 3	
solid.sGpphiz	solid.gpeval(solid.s phiz)	N/m²	Stress tensor, Gauss point evaluation, phiz-component	Domain 3	
solid.sGpzz	solid.gpeval(solid.sz z)	N/m²	Stress tensor, Gauss point evaluation, zz-component	Domain 3	
solid.slGp11	solid.gpeval(solid.sl 11)	N/m²	Stress tensor, local coordinate system, Gauss point evaluation, 11-component	Domain 3	
solid.slGp12	solid.gpeval(solid.sl 12)	N/m²	Stress tensor, local coordinate system, Gauss point evaluation, 12-component	Domain 3	
solid.slGp13	solid.gpeval(solid.sl 13)	N/m²	Stress tensor, local coordinate	Domain 3	

Name	Expression	Unit	Description	Selection	Details
			system, Gauss point evaluation, 13-component		
solid.slGp22	solid.gpeval(solid.sl22)	N/m²	Stress tensor, local coordinate system, Gauss point evaluation, 22-component	Domain 3	
solid.slGp23	solid.gpeval(solid.sl23)	N/m²	Stress tensor, local coordinate system, Gauss point evaluation, 23-component	Domain 3	
solid.slGp33	solid.gpeval(solid.sl33)	N/m²	Stress tensor, local coordinate system, Gauss point evaluation, 33-component	Domain 3	
solid.sdevGprr	solid.gpeval(solid.sdevrr)	N/m²	Deviatoric stress tensor, Gauss point evaluation, rr-component	Domain 3	
solid.sdevGprphi	solid.gpeval(solid.sdevrphi)	N/m²	Deviatoric stress tensor, Gauss point evaluation, rphi-component	Domain 3	
solid.sdevGprz	solid.gpeval(solid.sdevrz)	N/m²	Deviatoric stress tensor, Gauss point evaluation, rz-component	Domain 3	
solid.sdevGpphiphi	solid.gpeval(solid.sdevphiphi)	N/m²	Deviatoric stress tensor, Gauss point evaluation, phiphi-component	Domain 3	
solid.sdevGpphiz	solid.gpeval(solid.sdevphiz)	N/m²	Deviatoric stress tensor, Gauss	Domain 3	

Name	Expression	Unit	Description	Selection	Details
			point evaluation, phiz-component		
solid.sdevGpzz	solid.gpeval(solid.sdevzz)	N/m²	Deviatoric stress tensor, Gauss point evaluation, zz-component	Domain 3	
solid.PGprR	solid.gpeval(solid.PrR)	N/m²	First Piola-Kirchhoff stress, Gauss point evaluation, rR-component	Domain 3	
solid.PGpphiR	solid.gpeval(solid.PphiR)	N/m²	First Piola-Kirchhoff stress, Gauss point evaluation, phiR-component	Domain 3	
solid.PGpzR	solid.gpeval(solid.PzR)	N/m²	First Piola-Kirchhoff stress, Gauss point evaluation, zR-component	Domain 3	
solid.PGprPHI	solid.gpeval(solid.PrPHI)	N/m²	First Piola-Kirchhoff stress, Gauss point evaluation, rPHI-component	Domain 3	
solid.PGpphiPHI	solid.gpeval(solid.PphiPHI)	N/m²	First Piola-Kirchhoff stress, Gauss point evaluation, phiPHI-component	Domain 3	
solid.PGpzPHI	solid.gpeval(solid.PzPHI)	N/m²	First Piola-Kirchhoff stress, Gauss point evaluation, zPHI-component	Domain 3	
solid.PGprZ	solid.gpeval(solid.PrZ)	N/m²	First Piola-Kirchhoff stress,	Domain 3	

Name	Expression	Unit	Description	Selection	Details
			Gauss point evaluation, rZ-component		
solid.PGpphiZ	solid.gpeval(solid.PphiZ)	N/m²	First Piola-Kirchhoff stress, Gauss point evaluation, phiZ-component	Domain 3	
solid.PGpzZ	solid.gpeval(solid.PzZ)	N/m²	First Piola-Kirchhoff stress, Gauss point evaluation, zZ-component	Domain 3	
solid.pGp	solid.gpeval(solid.p)	N/m²	Pressure, Gauss point evaluation	Domain 3	
solid.pmGp	solid.gpeval(solid.pm)	N/m²	Pressure, Gauss point evaluation	Domain 3	
solid.Ws	0.5*(solid.Sel11*solid.eel11+2*solid.Sel12*solid.eel12+2*solid.Sel13*solid.eel13+solid.Sel22*solid.eel22+2*solid.Sel23*solid.eel23+solid.Sel33*solid.eel33)	J/m³	Elastic strain energy density	Domain 3	+ operation
solid.Wh	solid.Ws	J/m³	Stored energy density	Domain 3	+ operation
solid.Ws_tot	solid.lemm1.intDom(solid.Ws*solid.lemm1.integrand*solid.lemm1.gsv)	J	Total elastic strain energy	Global	+ operation
solid.Wh_tot	solid.lemm1.intDom(solid.Wh*solid.lemm1.integrand*solid.lemm1.gsv)	J	Total stored energy	Global	+ operation
solid.Eequ	solid.E	Pa	Equivalent Young's modulus	Domain 3	
solid.nuequ	solid.nu	1	Equivalent Poisson's ratio	Domain 3	
solid.Gequ	solid.G	N/m²	Equivalent shear modulus	Domain 3	

Name	Expression	Unit	Description	Selection	Details
solid.K	solid.E/(3*(1-2*solid.nu))	N/m²	Bulk modulus	Domain 3	* operation
solid.D11	solid.E*(1-solid.nu)/((1+solid.nu)*(1-2*solid.nu))	Pa	Elasticity matrix, 11-component	Domain 3	* operation
solid.D12	solid.E*solid.nu/((1+solid.nu)*(1-2*solid.nu))	Pa	Elasticity matrix, 12-component	Domain 3	* operation
solid.D13	solid.E*solid.nu/((1+solid.nu)*(1-2*solid.nu))	Pa	Elasticity matrix, 13-component	Domain 3	* operation
solid.D14	0	Pa	Elasticity matrix, 14-component	Domain 3	* operation
solid.D15	0	Pa	Elasticity matrix, 15-component	Domain 3	* operation
solid.D16	0	Pa	Elasticity matrix, 16-component	Domain 3	* operation
solid.D22	solid.E*(1-solid.nu)/((1+solid.nu)*(1-2*solid.nu))	Pa	Elasticity matrix, 22-component	Domain 3	* operation
solid.D23	solid.E*solid.nu/((1+solid.nu)*(1-2*solid.nu))	Pa	Elasticity matrix, 23-component	Domain 3	* operation
solid.D24	0	Pa	Elasticity matrix, 24-component	Domain 3	* operation
solid.D25	0	Pa	Elasticity matrix, 25-component	Domain 3	* operation
solid.D26	0	Pa	Elasticity matrix, 26-component	Domain 3	* operation
solid.D33	solid.E*(1-solid.nu)/((1+solid.nu)*(1-2*solid.nu))	Pa	Elasticity matrix, 33-component	Domain 3	* operation
solid.D34	0	Pa	Elasticity matrix, 34-component	Domain 3	* operation
solid.D35	0	Pa	Elasticity matrix, 35-component	Domain 3	* operation
solid.D36	0	Pa	Elasticity matrix, 36-component	Domain 3	* operation
solid.D44	0.5*solid.E/(1+solid.nu)	Pa	Elasticity matrix, 44-component	Domain 3	* operation
solid.D45	0	Pa	Elasticity matrix,	Domain 3	* operation

Name	Expression	Unit	Description	Selection	Details
			45-component		
solid.D46	0	Pa	Elasticity matrix, 46-component	Domain 3	* operation
solid.D55	0.5*solid.E/(1+solid.nu)	Pa	Elasticity matrix, 55-component	Domain 3	* operation
solid.D56	0	Pa	Elasticity matrix, 56-component	Domain 3	* operation
solid.D66	0.5*solid.E/(1+solid.nu)	Pa	Elasticity matrix, 66-component	Domain 3	* operation
solid.DVo11	solid.D11	Pa	Elasticity matrix, Voigt notation, 11-component	Domain 3	
solid.DVo12	solid.D12	Pa	Elasticity matrix, Voigt notation, 12-component	Domain 3	
solid.DVo13	solid.D13	Pa	Elasticity matrix, Voigt notation, 13-component	Domain 3	
solid.DVo14	solid.D15	Pa	Elasticity matrix, Voigt notation, 14-component	Domain 3	
solid.DVo15	solid.D16	Pa	Elasticity matrix, Voigt notation, 15-component	Domain 3	
solid.DVo16	solid.D14	Pa	Elasticity matrix, Voigt notation, 16-component	Domain 3	
solid.DVo22	solid.D22	Pa	Elasticity matrix, Voigt notation, 22-component	Domain 3	
solid.DVo23	solid.D23	Pa	Elasticity matrix, Voigt notation, 23-component	Domain 3	
solid.DVo24	solid.D25	Pa	Elasticity matrix, Voigt notation, 24-component	Domain 3	
solid.DVo25	solid.D26	Pa	Elasticity matrix, Voigt notation, 25-component	Domain 3	
solid.DVo26	solid.D24	Pa	Elasticity matrix, Voigt notation, 26-component	Domain 3	

Name	Expression	Unit	Description	Selection	Details
solid.DVo33	solid.D33	Pa	Elasticity matrix, Voigt notation, 33-component	Domain 3	
solid.DVo34	solid.D35	Pa	Elasticity matrix, Voigt notation, 34-component	Domain 3	
solid.DVo35	solid.D36	Pa	Elasticity matrix, Voigt notation, 35-component	Domain 3	
solid.DVo36	solid.D34	Pa	Elasticity matrix, Voigt notation, 36-component	Domain 3	
solid.DVo44	solid.D55	Pa	Elasticity matrix, Voigt notation, 44-component	Domain 3	
solid.DVo45	solid.D56	Pa	Elasticity matrix, Voigt notation, 45-component	Domain 3	
solid.DVo46	solid.D45	Pa	Elasticity matrix, Voigt notation, 46-component	Domain 3	
solid.DVo55	solid.D66	Pa	Elasticity matrix, Voigt notation, 55-component	Domain 3	
solid.DVo56	solid.D46	Pa	Elasticity matrix, Voigt notation, 56-component	Domain 3	
solid.DVo66	solid.D44	Pa	Elasticity matrix, Voigt notation, 66-component	Domain 3	
solid.Dg11	solid.D11	Pa	Elasticity matrix (global), 11-component	Domain 3	
solid.Dg12	solid.D12	Pa	Elasticity matrix (global), 12-component	Domain 3	
solid.Dg13	solid.D13	Pa	Elasticity matrix (global), 13-component	Domain 3	
solid.Dg14	solid.D14	Pa	Elasticity matrix (global), 14-component	Domain 3	

Name	Expression	Unit	Description	Selection	Details
solid.Dg15	solid.D15	Pa	Elasticity matrix (global), 15-component	Domain 3	
solid.Dg16	solid.D16	Pa	Elasticity matrix (global), 16-component	Domain 3	
solid.Dg22	solid.D22	Pa	Elasticity matrix (global), 22-component	Domain 3	
solid.Dg23	solid.D23	Pa	Elasticity matrix (global), 23-component	Domain 3	
solid.Dg24	solid.D24	Pa	Elasticity matrix (global), 24-component	Domain 3	
solid.Dg25	solid.D25	Pa	Elasticity matrix (global), 25-component	Domain 3	
solid.Dg26	solid.D26	Pa	Elasticity matrix (global), 26-component	Domain 3	
solid.Dg33	solid.D33	Pa	Elasticity matrix (global), 33-component	Domain 3	
solid.Dg34	solid.D34	Pa	Elasticity matrix (global), 34-component	Domain 3	
solid.Dg35	solid.D35	Pa	Elasticity matrix (global), 35-component	Domain 3	
solid.Dg36	solid.D36	Pa	Elasticity matrix (global), 36-component	Domain 3	
solid.Dg44	solid.D44	Pa	Elasticity matrix (global), 44-component	Domain 3	
solid.Dg45	solid.D45	Pa	Elasticity matrix (global), 45-component	Domain 3	
solid.Dg46	solid.D46	Pa	Elasticity matrix (global), 46-component	Domain 3	

Name	Expression	Unit	Description	Selection	Details
solid.Dg55	solid.D55	Pa	Elasticity matrix (global), 55-component	Domain 3	
solid.Dg56	solid.D56	Pa	Elasticity matrix (global), 56-component	Domain 3	
solid.Dg66	solid.D66	Pa	Elasticity matrix (global), 66-component	Domain 3	
solid.DVog11	solid.DVo11	Pa	Elasticity matrix, Voigt notation (global), 11-component	Domain 3	
solid.DVog12	solid.DVo12	Pa	Elasticity matrix, Voigt notation (global), 12-component	Domain 3	
solid.DVog13	solid.DVo13	Pa	Elasticity matrix, Voigt notation (global), 13-component	Domain 3	
solid.DVog14	solid.DVo14	Pa	Elasticity matrix, Voigt notation (global), 14-component	Domain 3	
solid.DVog15	solid.DVo15	Pa	Elasticity matrix, Voigt notation (global), 15-component	Domain 3	
solid.DVog16	solid.DVo16	Pa	Elasticity matrix, Voigt notation (global), 16-component	Domain 3	
solid.DVog22	solid.DVo22	Pa	Elasticity matrix, Voigt notation (global), 22-component	Domain 3	
solid.DVog23	solid.DVo23	Pa	Elasticity matrix, Voigt notation (global), 23-component	Domain 3	
solid.DVog24	solid.DVo24	Pa	Elasticity matrix, Voigt notation (global), 24-	Domain 3	

Name	Expression	Unit	Description	Selection	Details
			component		
solid.DVog25	solid.DVo25	Pa	Elasticity matrix, Voigt notation (global), 25-component	Domain 3	
solid.DVog26	solid.DVo26	Pa	Elasticity matrix, Voigt notation (global), 26-component	Domain 3	
solid.DVog33	solid.DVo33	Pa	Elasticity matrix, Voigt notation (global), 33-component	Domain 3	
solid.DVog34	solid.DVo34	Pa	Elasticity matrix, Voigt notation (global), 34-component	Domain 3	
solid.DVog35	solid.DVo35	Pa	Elasticity matrix, Voigt notation (global), 35-component	Domain 3	
solid.DVog36	solid.DVo36	Pa	Elasticity matrix, Voigt notation (global), 36-component	Domain 3	
solid.DVog44	solid.DVo44	Pa	Elasticity matrix, Voigt notation (global), 44-component	Domain 3	
solid.DVog45	solid.DVo45	Pa	Elasticity matrix, Voigt notation (global), 45-component	Domain 3	
solid.DVog46	solid.DVo46	Pa	Elasticity matrix, Voigt notation (global), 46-component	Domain 3	
solid.DVog55	solid.DVo55	Pa	Elasticity matrix, Voigt notation (global), 55-component	Domain 3	
solid.DVog56	solid.DVo56	Pa	Elasticity matrix, Voigt notation (global), 56-	Domain 3	

Name	Expression	Unit	Description	Selection	Details
			component		
solid.DVog66	solid.DVo66	Pa	Elasticity matrix, Voigt notation (global), 66-component	Domain 3	
solid.Kpar0	0		Instantaneous bulk modulus	Domain 3	+ operation
solid.Qser0	0	1/Pa	Instantaneous bulk compliance	Domain 3	+ operation
solid.Jser0	0	1/Pa	Instantaneous shear compliance	Domain 3	+ operation
solid.Gpar0	0		Instantaneous shear modulus	Domain 3	+ operation
solid.K0	1/(1/(solid.Kequ+solid.Kpar0)+solid.Qser0)	N/m²	Instantaneous bulk modulus	Domain 3	
solid.G0	1/(1/(solid.Gequ+solid.Gpar0)+solid.Jser0)	N/m²	Instantaneous shear modulus	Domain 3	
solid.Qh	0	W/m³	Total power dissipation density	Domain 3	+ operation
solid.rho	material.rho	kg/m³	Density	Domain 3	Meta, * operation
solid.lemm1.integrand	2*pi*R	m	Intermediate variable	Domain 3	Meta
solid.lemm1.gsv	1		Geometry scale factor (volume)	Domain 3	
solid.cp	sqrt((solid.K+4*solid.G/3)/solid.rho)	m/s	Equivalent speed of pressure wave	Domain 3	
solid.cs	sqrt(solid.G/solid.rho)	m/s	Equivalent speed of shear wave	Domain 3	
solid.E	material.E	Pa	Young's modulus	Domain 3	Meta, * operation
solid.nu	material.nu	1	Poisson's ratio	Domain 3	Meta
solid.G	0.5*solid.E/(1+solid.nu)	N/m²	Shear modulus	Domain 3	* operation
solid.lambLame	solid.E*solid.nu/((1+solid.nu)*(1-	N/m²	Lamé parameter λ	Domain 3	* operation

Name	Expression	Unit	Description	Selection	Details
	2*solid.nu))				
solid.muLame	0.5*solid.E/(1+solid.nu)	N/m²	Lamé parameter μ	Domain 3	* operation
solid.Cgl11	solid.D11+solid.D12+solid.D13	Pa	C:g tensor, local coordinate system, 11-component	Domain 3	
solid.Cgl12	solid.D14+solid.D24+solid.D34	Pa	C:g tensor, local coordinate system, 12-component	Domain 3	
solid.Cgl13	solid.D16+solid.D26+solid.D36	Pa	C:g tensor, local coordinate system, 13-component	Domain 3	
solid.Cgl22	solid.D12+solid.D22+solid.D23	Pa	C:g tensor, local coordinate system, 22-component	Domain 3	
solid.Cgl23	solid.D15+solid.D25+solid.D35	Pa	C:g tensor, local coordinate system, 23-component	Domain 3	
solid.Cgl33	solid.D13+solid.D23+solid.D33	Pa	C:g tensor, local coordinate system, 33-component	Domain 3	
solid.elogrr	log(solid.stchp1)*solid.LW11^2+log(solid.stchp2)*solid.LW12^2+log(solid.stchp3)*solid.LW13^2	1	Logarithmic strain tensor, rr-component	Domain 3	
solid.elogrphi	log(solid.stchp1)*solid.LW11*solid.LW21+log(solid.stchp2)*solid.LW12*solid.LW22+log(solid.stchp3)*solid.LW13*solid.LW23	1	Logarithmic strain tensor, rphi-component	Domain 3	
solid.elogrz	log(solid.stchp1)*solid.LW11*solid.LW31+log(solid.stchp2)*solid.LW12*solid.LW32+log(solid.stch	1	Logarithmic strain tensor, rz-component	Domain 3	

Name	Expression	Unit	Description	Selection	Details
	p3)*solid.LW13*solid.LW33				
solid.elogphiphi	log(solid.stchp1)*solid.LW21^2+log(solid.stchp2)*solid.LW22^2+log(solid.stchp3)*solid.LW23^2	1	Logarithmic strain tensor, phiphi-component	Domain 3	
solid.elogphiz	log(solid.stchp1)*solid.LW21*solid.LW31+log(solid.stchp2)*solid.LW22*solid.LW32+log(solid.stchp3)*solid.LW23*solid.LW33	1	Logarithmic strain tensor, phiz-component	Domain 3	
solid.elogzz	log(solid.stchp1)*solid.LW31^2+log(solid.stchp2)*solid.LW32^2+log(solid.stchp3)*solid.LW33^2	1	Logarithmic strain tensor, zz-component	Domain 3	
solid.ellog11	(solid.sysT11*solid.elogrr+solid.sysT12*solid.elogrphi+solid.sysT13*solid.elogrz)*solid.sysT11+(solid.sysT11*solid.elogrphi+solid.sysT12*solid.elogphiphi+solid.sysT13*solid.elogphiz)*solid.sysT12+(solid.sysT11*solid.elogrz+solid.sysT12*solid.elogphiz+solid.sysT13*solid.elogzz)*solid.sysT13	1	Logarithmic strain tensor, local coordinate system, 11-component	Domain 3	
solid.ellog12	(solid.sysT11*solid.elogrr+solid.sysT12*solid.elogrphi+solid.sysT13*solid.elogrz)*solid.sysT21+(solid.sysT11*solid.elogrphi+solid.sysT12*solid.elogphiphi+solid.sysT13*solid.elogphiz)*solid.sysT22+(solid.sysT11*solid.elogrz+solid.sysT	1	Logarithmic strain tensor, local coordinate system, 12-component	Domain 3	

Name	Expression	Unit	Description	Selection	Details
	12*solid.elogphiz+solid.sysT13*solid.elogzz)*solid.sysT23				
solid.ellog13	(solid.sysT11*solid.elogrr+solid.sysT12*solid.elogrphi+solid.sysT13*solid.elogrz)*solid.sysT31+(solid.sysT11*solid.elogrphi+solid.sysT12*solid.elogphiphi+solid.sysT13*solid.elogphiz)*solid.sysT32+(solid.sysT11*solid.elogrz+solid.sysT12*solid.elogphiz+solid.sysT13*solid.elogzz)*solid.sysT33	1	Logarithmic strain tensor, local coordinate system, 13-component	Domain 3	
solid.ellog22	(solid.sysT21*solid.elogrr+solid.sysT22*solid.elogrphi+solid.sysT23*solid.elogrz)*solid.sysT21+(solid.sysT21*solid.elogrphi+solid.sysT22*solid.elogphiphi+solid.sysT23*solid.elogphiz)*solid.sysT22+(solid.sysT21*solid.elogrz+solid.sysT22*solid.elogphiz+solid.sysT23*solid.elogzz)*solid.sysT23	1	Logarithmic strain tensor, local coordinate system, 22-component	Domain 3	
solid.ellog23	(solid.sysT21*solid.elogrr+solid.sysT22*solid.elogrphi+solid.sysT23*solid.elogrz)*solid.sysT31+(solid.sysT21*solid.elogrphi+solid.sysT22*solid.elogphiphi+solid.sysT23*solid.elogphiz)*solid.sysT32+(solid.sysT21*solid.elogrz+solid.sysT22*solid.elogphiz+s	1	Logarithmic strain tensor, local coordinate system, 23-component	Domain 3	

Name	Expression	Unit	Description	Selection	Details
	olid.sysT23*solid.elogzz)*solid.sysT33				
solid.ellog33	(solid.sysT31*solid.elogrr+solid.sysT32*solid.elogrphi+solid.sysT33*solid.elogrz)*solid.sysT31+(solid.sysT31*solid.elogrphi+solid.sysT32*solid.elogphiphi+solid.sysT33*solid.elogphiz)*solid.sysT32+(solid.sysT31*solid.elogrz+solid.sysT32*solid.elogphiz+solid.sysT33*solid.elogzz)*solid.sysT33	1	Logarithmic strain tensor, local coordinate system, 33-component	Domain 3	
solid.u_ttR	d(uTIME-uR*d(R,TIME)-uZ*d(Z,TIME),TIME)-d(uTIME-uR*d(R,TIME)-uZ*d(Z,TIME),R)*d(R,TIME)-d(uTIME-uR*d(R,TIME)-uZ*d(Z,TIME),Z)*d(Z,TIME)	m/s²	Acceleration, R-component	Domain 3	
solid.u_ttPHI	0	m/s²	Acceleration, PHI-component	Domain 3	
solid.u_ttZ	d(wTIME-wR*d(R,TIME)-wZ*d(Z,TIME),TIME)-d(wTIME-wR*d(R,TIME)-wZ*d(Z,TIME),R)*d(R,TIME)-d(wTIME-wR*d(R,TIME)-wZ*d(Z,TIME),Z)*d(Z,TIME)	m/s²	Acceleration, Z-component	Domain 3	
solid.u_tR	uTIME-uR*d(R,TIME)-uZ*d(Z,TIME)	m/s	Velocity, R-component	Domain 3	
solid.u_tPHI	0	m/s	Velocity, PHI-component	Domain 3	
solid.u_tZ	wTIME-	m/s	Velocity, Z-	Domain 3	

Name	Expression	Unit	Description	Selection	Details
	wR*d(R,TIME)-wZ*d(Z,TIME)		component		
solid.afR	0	m/s²	Frame acceleration, R-component	Domain 3	+ operation
solid.afPHI	0	m/s²	Frame acceleration, PHI-component	Domain 3	+ operation
solid.afZ	0	m/s²	Frame acceleration, Z-component	Domain 3	+ operation
solid.accR	solid.u_ttR	m/s²	Effective acceleration, R-component	Domain 3	+ operation
solid.accPHI	solid.u_ttPHI	m/s²	Effective acceleration, PHI-component	Domain 3	+ operation
solid.accZ	solid.u_ttZ	m/s²	Effective acceleration, Z-component	Domain 3	+ operation
solid.vel	sqrteps(real(solid.u_tR)^2+real(solid.u_tPHI)^2+real(solid.u_tZ)^2)	m/s	Velocity magnitude	Domain 3	
solid.acc	sqrteps(real(solid.accR)^2+real(solid.accPHI)^2+real(solid.accZ)^2)	m/s²	Effective acceleration magnitude	Domain 3	
solid.u_tt	sqrteps(real(solid.u_ttR)^2+real(solid.u_ttPHI)^2+real(solid.u_ttZ)^2)	m/s²	Acceleration magnitude	Domain 3	
solid.thetaL	atan2(sqrt(max(0.14814814814814814*solid.II2s^3-solid.II3s^2,eps)),solid.II3s)/3	rad	Lode angle	Domain 3	
solid.tresca	max(max(abs(solid.sp1-solid.sp2),abs(solid.sp1-solid.sp3)),abs(solid.sp2-solid.sp3))	N/m²	Tresca stress	Domain 3	

Name	Expression	Unit	Description	Selection	Details
solid.Tar	mean(solid.srr)*solid.nr+mean(solid.srphi)*solid.nphi+mean(solid.srz)*solid.nz	N/m²	Traction (force/area), r-component	Boundaries 7–9, 16	
solid.Taphi	mean(solid.srphi)*solid.nr+mean(solid.sphiphi)*solid.nphi+mean(solid.sphiz)*solid.nz	N/m²	Traction (force/area), phi-component	Boundaries 7–9, 16	
solid.Taz	mean(solid.srz)*solid.nr+mean(solid.sphiz)*solid.nphi+mean(solid.szz)*solid.nz	N/m²	Traction (force/area), z-component	Boundaries 7–9, 16	
solid.stn	solid.Tar*solid.nr+solid.Taphi*solid.nphi+solid.Taz*solid.nz	N/m²	Normal stress	Boundaries 7–9, 16	
solid.Tanr	solid.stn*solid.nr	N/m²	Normal component of traction, r-component	Boundaries 7–9, 16	
solid.Tanphi	solid.stn*solid.nphi	N/m²	Normal component of traction, phi-component	Boundaries 7–9, 16	
solid.Tanz	solid.stn*solid.nz	N/m²	Normal component of traction, z-component	Boundaries 7–9, 16	
solid.Tatr	solid.Tar-solid.Tanr	N/m²	Shear component of traction, r-component	Boundaries 7–9, 16	
solid.Tatphi	solid.Taphi-solid.Tanphi	N/m²	Shear component of traction, phi-component	Boundaries 7–9, 16	
solid.Tatz	solid.Taz-solid.Tanz	N/m²	Shear component of traction, z-component	Boundaries 7–9, 16	
solid.stt	sqrt(solid.Tatr^2+solid.Tatphi^2+solid	N/m²	Shear stress	Boundaries 7–9, 16	

Name	Expression	Unit	Description	Selection	Details
	.Tatz^2)				
solid.sysT11	1	1	Transform to global system, 11-component	Domain 3	
solid.sysT21	0	1	Transform to global system, 21-component	Domain 3	
solid.sysT31	0	1	Transform to global system, 31-component	Domain 3	
solid.sysT12	0	1	Transform to global system, 12-component	Domain 3	
solid.sysT22	1	1	Transform to global system, 22-component	Domain 3	
solid.sysT32	0	1	Transform to global system, 32-component	Domain 3	
solid.sysT13	0	1	Transform to global system, 13-component	Domain 3	
solid.sysT23	0	1	Transform to global system, 23-component	Domain 3	
solid.sysT33	1	1	Transform to global system, 33-component	Domain 3	
uRt	uRTIME-uRR*d(R,TIME)-uRZ*d(Z,TIME)	1/s	Gradient of u, R component, first time derivative	Domain 3	
uZt	uZTIME-uZR*d(R,TIME)-uZZ*d(Z,TIME)	1/s	Gradient of u, Z component, first time derivative	Domain 3	
uRtt	d(uRTIME-uRR*d(R,TIME)-uRZ*d(Z,TIME),TIME)-d(uRTIME-uRR*d(R,TIME)-uRZ*d(Z,TIME),R)*d(R,TIME)-d(uRTIME-uRR*d(R,TIME)-uRZ*d(Z,TIME),Z)*d(	$1/s^2$	Gradient of u, R component, second time derivative	Domain 3	

Name	Expression	Unit	Description	Selection	Details
	Z,TIME)				
uZtt	d(uZTIME-uZR*d(R,TIME)-uZZ*d(Z,TIME),TIME)-d(uZTIME-uZR*d(R,TIME)-uZZ*d(Z,TIME),R)*d(R,TIME)-d(uZTIME-uZR*d(R,TIME)-uZZ*d(Z,TIME),Z)*d(Z,TIME)	$1/s^2$	Gradient of u, Z component, second time derivative	Domain 3	
wRt	wRTIME-wRR*d(R,TIME)-wRZ*d(Z,TIME)	1/s	Gradient of w, R component, first time derivative	Domain 3	
wZt	wZTIME-wZR*d(R,TIME)-wZZ*d(Z,TIME)	1/s	Gradient of w, Z component, first time derivative	Domain 3	
wRtt	d(wRTIME-wRR*d(R,TIME)-wRZ*d(Z,TIME),TIME)-d(wRTIME-wRR*d(R,TIME)-wRZ*d(Z,TIME),R)*d(R,TIME)-d(wRTIME-wRR*d(R,TIME)-wRZ*d(Z,TIME),Z)*d(Z,TIME)	$1/s^2$	Gradient of w, R component, second time derivative	Domain 3	
wZtt	d(wZTIME-wZR*d(R,TIME)-wZZ*d(Z,TIME),TIME)-d(wZTIME-wZR*d(R,TIME)-wZZ*d(Z,TIME),R)*d(R,TIME)-d(wZTIME-wZR*d(R,TIME)-wZZ*d(Z,TIME),Z)*d(Z,TIME)	$1/s^2$	Gradient of w, Z component, second time derivative	Domain 3	
ut	uTIME-uR*d(R,TIME)-uZ*d(Z,TIME)	m/s	Structural velocity field, R-component	Domain 3	
wt	wTIME-wR*d(R,TIME)-wZ*d(Z,TIME)	m/s	Structural velocity field, Z-component	Domain 3	

Name	Expression	Unit	Description	Selection	Details
utt	d(uTIME-uR*d(R,TIME)-uZ*d(Z,TIME),TIME)-d(uTIME-uR*d(R,TIME)-uZ*d(Z,TIME),R)*d(R,TIME)-d(uTIME-uR*d(R,TIME)-uZ*d(Z,TIME),Z)*d(Z,TIME)	m/s^2	Acceleration field, R-component	Domain 3	
wtt	d(wTIME-wR*d(R,TIME)-wZ*d(Z,TIME),TIME)-d(wTIME-wR*d(R,TIME)-wZ*d(Z,TIME),R)*d(R,TIME)-d(wTIME-wR*d(R,TIME)-wZ*d(Z,TIME),Z)*d(Z,TIME)	m/s^2	Acceleration field, Z-component	Domain 3	
solid.pp	-solid.K*solid.eelvol	N/m^2	Pressure with reference to material area	Domain 3	
solid.lemm1.pwRt	solid.lemm1.pwRTIME-solid.lemm1.pwRR*d(R,TIME)-solid.lemm1.pwRZ*d(Z,TIME)	W/m^4	Gradient of pw, R component, first time derivative	Domain 3	
solid.lemm1.pwZt	solid.lemm1.pwZTIME-solid.lemm1.pwZR*d(R,TIME)-solid.lemm1.pwZZ*d(Z,TIME)	W/m^4	Gradient of pw, Z component, first time derivative	Domain 3	
solid.lemm1.pwRtt	d(solid.lemm1.pwRTIME-solid.lemm1.pwRR*d(R,TIME)-solid.lemm1.pwRZ*d(Z,TIME),TIME)-d(solid.lemm1.pwRTIME-solid.lemm1.pwRR*d(R,TIME)-solid.lemm1.pwRZ*	$kg/(m^2 \cdot s^4)$	Gradient of pw, R component, second time derivative	Domain 3	

Name	Expression	Unit	Description	Selection	Details
	d(Z,TIME),R)*d(R,TIME)-d(solid.lemm1.pwRTIME-solid.lemm1.pwRR*d(R,TIME)-solid.lemm1.pwRZ*d(Z,TIME),Z)*d(Z,TIME)				
solid.lemm1.pwZtt	d(solid.lemm1.pwZTIME-solid.lemm1.pwZR*d(R,TIME)-solid.lemm1.pwZZ*d(Z,TIME),TIME)-d(solid.lemm1.pwZTIME-solid.lemm1.pwZR*d(R,TIME)-solid.lemm1.pwZZ*d(Z,TIME),R)*d(R,TIME)-d(solid.lemm1.pwZTIME-solid.lemm1.pwZR*d(R,TIME)-solid.lemm1.pwZZ*d(Z,TIME),Z)*d(Z,TIME)	kg/(m²·s⁴)	Gradient of pw, Z component, second time derivative	Domain 3	
solid.lemm1.pwt	solid.lemm1.pwTIME-solid.lemm1.pwR*d(R,TIME)-solid.lemm1.pwZ*d(Z,TIME)	W/m³	Auxiliary pressure, first time derivative	Domain 3	
solid.lemm1.pwtt	d(solid.lemm1.pwTIME-solid.lemm1.pwR*d(R,TIME)-solid.lemm1.pwZ*d(Z,TIME),TIME)-d(solid.lemm1.pwTIME-solid.lemm1.pwR*d(R,TIME)-solid.lemm1.pwZ*d(	Pa/s²	Auxiliary pressure, second time derivative	Domain 3	

Name	Expression	Unit	Description	Selection	Details
	Z,TIME),R)*d(R,TIME)-d(solid.lemm1.pwTIME-solid.lemm1.pwR*d(R,TIME)-solid.lemm1.pwZ*d(Z,TIME),Z)*d(Z,TIME)				
solid.Ldr	((d(solid.FdrR,TIME)-d(solid.FdrR,R)*d(R,TIME)-d(solid.FdrR,Z)*d(Z,TIME))*(solid.FdphiPHI*solid.FdzZ-solid.FdphiZ*solid.FdzPHI)+(d(solid.FdrPHI,TIME)-d(solid.FdrPHI,R)*d(R,TIME)-d(solid.FdrPHI,Z)*d(Z,TIME))*(solid.FdphiZ*solid.FdzR-solid.FdphiR*solid.FdzZ)+(d(solid.FdrZ,TIME)-d(solid.FdrZ,R)*d(R,TIME)-d(solid.FdrZ,Z)*d(Z,TIME))*(solid.FdphiR*solid.FdzPHI-solid.FdphiPHI*solid.FdzR))/(solid.FdrR*solid.FdphiPHI*solid.FdzZ+solid.FdrPHI*solid.FdphiZ*solid.FdzR+solid.FdrZ*solid.FdphiR*solid.FdzPHI-solid.FdrR*solid.FdphiZ*solid.FdzPHI-solid.FdrPHI*solid.FdphiR*solid.FdzZ-solid.FdrZ*solid.FdphiPHI*solid.FdzR)	1/s	Rate of strain tensor, r-component	Domain 3	
solid.Ldrphi	0.5*((d(solid.FdrR,TI	1/s	Rate of strain	Domain 3	

Name	Expression	Unit	Description	Selection	Details
	ME)-d(solid.FdrR,R)*d(R,TIME)-d(solid.FdrR,Z)*d(Z,TIME))*(solid.FdrZ*solid.FdzPHI-solid.FdrPHI*solid.FdzZ)+(d(solid.FdrPHI,TIME)-d(solid.FdrPHI,R)*d(R,TIME)-d(solid.FdrPHI,Z)*d(Z,TIME))*(solid.FdrR*solid.FdzZ-solid.FdrZ*solid.FdzR)+(d(solid.FdrZ,TIME)-d(solid.FdrZ,R)*d(R,TIME)-d(solid.FdrZ,Z)*d(Z,TIME))*(solid.FdrPHI*solid.FdzR-solid.FdrR*solid.FdzPHI)+(d(solid.FdphiR,TIME)-d(solid.FdphiR,R)*d(R,TIME)-d(solid.FdphiR,Z)*d(Z,TIME))*(solid.FdphiPHI*solid.FdzZ-solid.FdphiZ*solid.FdzPHI)+(d(solid.FdphiPHI,TIME)-d(solid.FdphiPHI,R)*d(R,TIME)-d(solid.FdphiPHI,Z)*d(Z,TIME))*(solid.FdphiZ*solid.FdzR-solid.FdphiR*solid.FdzZ)+(d(solid.FdphiZ,TIME)-d(solid.FdphiZ,R)*d(R,TIME)-d(solid.FdphiZ,Z)*d(Z,TIME))*(solid.FdphiR*solid.FdzPHI-solid.FdphiPHI*solid.FdzR))/(solid.FdrR		tensor, rphi-component		

Name	Expression	Unit	Description	Selection	Details
	*solid.FdphiPHI*solid.FdzZ+solid.FdrPHI*solid.FdphiZ*solid.FdzR+solid.FdrZ*solid.FdphiR*solid.FdzPHI-solid.FdrR*solid.FdphiZ*solid.FdzPHI-solid.FdrPHI*solid.FdphiR*solid.FdzZ-solid.FdrZ*solid.FdphiPHI*solid.FdzR)				
solid.Ldrz	0.5*((d(solid.FdrR,TIME)-d(solid.FdrR,R)*d(R,TIME)-d(solid.FdrR,Z)*d(Z,TIME))*(solid.FdrPHI*solid.FdphiZ-solid.FdrZ*solid.FdphiPHI)+(d(solid.FdrPHI,TIME)-d(solid.FdrPHI,R)*d(R,TIME)-d(solid.FdrPHI,Z)*d(Z,TIME))*(solid.FdrZ*solid.FdphiR-solid.FdrR*solid.FdphiZ)+(d(solid.FdrZ,TIME)-d(solid.FdrZ,R)*d(R,TIME)-d(solid.FdrZ,Z)*d(Z,TIME))*(solid.FdrR*solid.FdphiPHI-solid.FdrPHI*solid.FdphiR)+(d(solid.FdzR,TIME)-d(solid.FdzR,R)*d(R,TIME)-d(solid.FdzR,Z)*d(Z,TIME))*(solid.FdphiPHI*solid.FdzZ-solid.FdphiZ*solid.FdzPHI)+(d(solid.FdzPHI,TIME)-d(solid.FdzPHI,R)*d(	1/s	Rate of strain tensor, rz-component	Domain 3	

Name	Expression	Unit	Description	Selection	Details
	R,TIME)-d(solid.FdzPHI,Z)*d(Z,TIME))*(solid.FdphiZ*solid.FdzR-solid.FdphiR*solid.FdzZ)+(d(solid.FdzZ,TIME)-d(solid.FdzZ,R)*d(R,TIME)-d(solid.FdzZ,Z)*d(Z,TIME))*(solid.FdphiR*solid.FdzPHI-solid.FdphiPHI*solid.FdzR))/(solid.FdrR*solid.FdphiPHI*solid.FdzZ+solid.FdrPHI*solid.FdphiZ*solid.FdzR+solid.FdrZ*solid.FdphiR*solid.FdzPHI-solid.FdrR*solid.FdphiZ*solid.FdzPHI-solid.FdrPHI*solid.FdphiR*solid.FdzZ-solid.FdrZ*solid.FdphiPHI*solid.FdzR)				
solid.Ldphi	((d(solid.FdphiR,TIME)-d(solid.FdphiR,R)*d(R,TIME)-d(solid.FdphiR,Z)*d(Z,TIME))*(solid.FdrZ*solid.FdzPHI-solid.FdrPHI*solid.FdzZ)+(d(solid.FdphiPHI,TIME)-d(solid.FdphiPHI,R)*d(R,TIME)-d(solid.FdphiPHI,Z)*d(Z,TIME))*(solid.FdrR*solid.FdzZ-solid.FdrZ*solid.FdzR)+(d(solid.FdphiZ,TIME)-d(solid.FdphiZ,R)*d(R,TIME)-d(solid.FdphiZ,Z)*d(	1/s	Rate of strain tensor, phi-component	Domain 3	

Name	Expression	Unit	Description	Selection	Details
	Z,TIME))*(solid.FdrPHI*solid.FdzR-solid.FdrR*solid.FdzPHI))/(solid.FdrR*solid.FdphiPHI*solid.FdzZ+solid.FdrPHI*solid.FdphiZ*solid.FdzR+solid.FdrZ*solid.FdphiR*solid.FdzPHI-solid.FdrR*solid.FdphiZ*solid.FdzPHI-solid.FdrPHI*solid.FdphiR*solid.FdzZ-solid.FdrZ*solid.FdphiPHI*solid.FdzR)				
solid.Ldphiz	0.5*((d(solid.FdphiR,TIME)-d(solid.FdphiR,R)*d(R,TIME)-d(solid.FdphiR,Z)*d(Z,TIME))*(solid.FdrPHI*solid.FdphiZ-solid.FdrZ*solid.FdphiPHI)+(d(solid.FdphiPHI,TIME)-d(solid.FdphiPHI,R)*d(R,TIME)-d(solid.FdphiPHI,Z)*d(Z,TIME))*(solid.FdrZ*solid.FdphiR-solid.FdrR*solid.FdphiZ)+(d(solid.FdphiZ,TIME)-d(solid.FdphiZ,R)*d(R,TIME)-d(solid.FdphiZ,Z)*d(Z,TIME))*(solid.FdrR*solid.FdphiPHI-solid.FdrPHI*solid.FdphiR)+(d(solid.FdzR,TIME)-d(solid.FdzR,R)*d(R,TIME)-d(solid.FdzR,Z)*d(Z,TIME))*(solid.FdrZ*solid.FdzPHI-	1/s	Rate of strain tensor, phiz-component	Domain 3	

Name	Expression	Unit	Description	Selection	Details
	solid.FdrPHI*solid.FdzZ)+(d(solid.FdzPHI,TIME)-d(solid.FdzPHI,R)*d(R,TIME)-d(solid.FdzPHI,Z)*d(Z,TIME))*(solid.FdrR*solid.FdzZ-solid.FdrZ*solid.FdzR)+(d(solid.FdzZ,TIME)-d(solid.FdzZ,R)*d(R,TIME)-d(solid.FdzZ,Z)*d(Z,TIME))*(solid.FdrPHI*solid.FdzR-solid.FdrR*solid.FdzPHI))/(solid.FdrR*solid.FdphiPHI*solid.FdzZ+solid.FdrPHI*solid.FdphiZ*solid.FdzR+solid.FdrZ*solid.FdphiR*solid.FdzPHI-solid.FdrR*solid.FdphiZ*solid.FdzPHI-solid.FdrPHI*solid.FdphiR*solid.FdzZ-solid.FdrZ*solid.FdphiPHI*solid.FdzR)				
solid.Ldz	((d(solid.FdzR,TIME)-d(solid.FdzR,R)*d(R,TIME)-d(solid.FdzR,Z)*d(Z,TIME))*(solid.FdrPHI*solid.FdphiZ-solid.FdrZ*solid.FdphiPHI)+(d(solid.FdzPHI,TIME)-d(solid.FdzPHI,R)*d(R,TIME)-d(solid.FdzPHI,Z)*d(Z,TIME))*(solid.FdrZ*solid.FdphiR-solid.FdrR*solid.FdphiZ)+(d(solid.FdzZ,T	1/s	Rate of strain tensor, z-component	Domain 3	

Name	Expression	Unit	Description	Selection	Details
	IME)-d(solid.FdzZ,R)*d(R,TIME)-d(solid.FdzZ,Z)*d(Z,TIME))*(solid.FdrR*solid.FdphiPHI-solid.FdrPHI*solid.FdphiR))/(solid.FdrR*solid.FdphiPHI*solid.FdzZ+solid.FdrPHI*solid.FdphiZ*solid.FdzR+solid.FdrZ*solid.FdphiR*solid.FdzPHI-solid.FdrR*solid.FdphiZ*solid.FdzPHI-solid.FdrPHI*solid.FdphiR*solid.FdzZ-solid.FdrZ*solid.FdphiPHI*solid.FdzR)				
solid.Lwr	0	1/s	Spin tensor, r-component	Domain 3	
solid.Lwrphi	0.5*((d(solid.FdrR,TIME)-d(solid.FdrR,R)*d(R,TIME)-d(solid.FdrR,Z)*d(Z,TIME))*(solid.FdrZ*solid.FdzPHI-solid.FdrPHI*solid.FdzZ)+(d(solid.FdrPHI,TIME)-d(solid.FdrPHI,R)*d(R,TIME)-d(solid.FdrPHI,Z)*d(Z,TIME))*(solid.FdrR*solid.FdzZ-solid.FdrZ*solid.FdzR)+(d(solid.FdrZ,TIME)-d(solid.FdrZ,R)*d(R,TIME)-d(solid.FdrZ,Z)*d(Z,TIME))*(solid.FdrPHI*solid.FdzR-solid.FdrR*solid.FdzPHI)-	1/s	Spin tensor, rphi-component	Domain 3	

Name	Expression	Unit	Description	Selection	Details
	(d(solid.FdphiR,TIME)-d(solid.FdphiR,R)*d(R,TIME)-d(solid.FdphiR,Z)*d(Z,TIME))*(solid.FdphiPHI*solid.FdzZ-solid.FdphiZ*solid.FdzPHI)-(d(solid.FdphiPHI,TIME)-d(solid.FdphiPHI,R)*d(R,TIME)-d(solid.FdphiPHI,Z)*d(Z,TIME))*(solid.FdphiZ*solid.FdzR-solid.FdphiR*solid.FdzZ)-(d(solid.FdphiZ,TIME)-d(solid.FdphiZ,R)*d(R,TIME)-d(solid.FdphiZ,Z)*d(Z,TIME))*(solid.FdphiR*solid.FdzPHI-solid.FdphiPHI*solid.FdzR))/(solid.FdrR*solid.FdphiPHI*solid.FdzZ+solid.FdrPHI*solid.FdphiZ*solid.FdzR+solid.FdrZ*solid.FdphiR*solid.FdzPHI-solid.FdrR*solid.FdphiZ*solid.FdzPHI-solid.FdrPHI*solid.FdphiR*solid.FdzZ-solid.FdrZ*solid.FdphiPHI*solid.FdzR)				
solid.Lwrz	0.5*((d(solid.FdrR,TIME)-d(solid.FdrR,R)*d(R,TIME)-d(solid.FdrR,Z)*d(Z,TIME))*(solid.FdrPHI*solid.FdphiZ-solid.FdrZ*solid.Fdp	1/s	Spin tensor, rz-component	Domain 3	

Name	Expression	Unit	Description	Selection	Details
	hiPHI)+(d(solid.FdrPHI,TIME)-d(solid.FdrPHI,R)*d(R,TIME)-d(solid.FdrPHI,Z)*d(Z,TIME))*(solid.FdrZ*solid.FdphiR-solid.FdrR*solid.FdphiZ)+(d(solid.FdrZ,TIME)-d(solid.FdrZ,R)*d(R,TIME)-d(solid.FdrZ,Z)*d(Z,TIME))*(solid.FdrR*solid.FdphiPHI-solid.FdrPHI*solid.FdphiR)-(d(solid.FdzR,TIME)-d(solid.FdzR,R)*d(R,TIME)-d(solid.FdzR,Z)*d(Z,TIME))*(solid.FdphiPHI*solid.FdzZ-solid.FdphiZ*solid.FdzPHI)-(d(solid.FdzPHI,TIME)-d(solid.FdzPHI,R)*d(R,TIME)-d(solid.FdzPHI,Z)*d(Z,TIME))*(solid.FdphiZ*solid.FdzR-solid.FdphiR*solid.FdzZ)-(d(solid.FdzZ,TIME)-d(solid.FdzZ,R)*d(R,TIME)-d(solid.FdzZ,Z)*d(Z,TIME))*(solid.FdphiR*solid.FdzPHI-solid.FdphiPHI*solid.FdzR))/(solid.FdrR*solid.FdphiPHI*solid.FdzZ+solid.FdrPHI*solid.FdphiZ*solid.FdzR+solid.FdrZ*solid.FdphiR*solid.FdzPHI-				

Name	Expression	Unit	Description	Selection	Details
	solid.FdrR*solid.FdphiZ*solid.FdzPHI-solid.FdrPHI*solid.FdphiR*solid.FdzZ-solid.FdrZ*solid.FdphiPHI*solid.FdzR)				
solid.Lwphi	0	1/s	Spin tensor, phi-component	Domain 3	
solid.Lwphiz	0.5*((d(solid.FdphiR,TIME)-d(solid.FdphiR,R)*d(R,TIME)-d(solid.FdphiR,Z)*d(Z,TIME))*(solid.FdrPHI*solid.FdphiZ-solid.FdrZ*solid.FdphiPHI)+(d(solid.FdphiPHI,TIME)-d(solid.FdphiPHI,R)*d(R,TIME)-d(solid.FdphiPHI,Z)*d(Z,TIME))*(solid.FdrZ*solid.FdphiR-solid.FdrR*solid.FdphiZ)+(d(solid.FdphiZ,TIME)-d(solid.FdphiZ,R)*d(R,TIME)-d(solid.FdphiZ,Z)*d(Z,TIME))*(solid.FdrR*solid.FdphiPHI-solid.FdrPHI*solid.FdphiR)-(d(solid.FdzR,TIME)-d(solid.FdzR,R)*d(R,TIME)-d(solid.FdzR,Z)*d(Z,TIME))*(solid.FdrZ*solid.FdzPHI-solid.FdrPHI*solid.FdzZ)-(d(solid.FdzPHI,TIME)-d(solid.FdzPHI,R)*d(R,TIME)-d(solid.FdzPHI,Z)*d(Z,TIME))*(solid.FdrR	1/s	Spin tensor, phiz-component	Domain 3	

Name	Expression	Unit	Description	Selection	Details
	*solid.FdzZ-solid.FdrZ*solid.FdzR)-(d(solid.FdzZ,TIME)-d(solid.FdzZ,R)*d(R,TIME)-d(solid.FdzZ,Z)*d(Z,TIME))*(solid.FdrPHI*solid.FdzR-solid.FdrR*solid.FdzPHI))/(solid.FdrR*solid.FdphiPHI*solid.FdzZ+solid.FdrPHI*solid.FdphiZ*solid.FdzR+solid.FdrZ*solid.FdphiR*solid.FdzPHI-solid.FdrR*solid.FdphiZ*solid.FdzPHI-solid.FdrPHI*solid.FdphiR*solid.FdzZ-solid.FdrZ*solid.FdphiPHI*solid.FdzR)				
solid.Lwz	0	1/s	Spin tensor, z-component	Domain 3	
solid.sp1Gp	solid.gpeval(solid.sp1)	N/m²	First principal stress, Gauss point evaluation	Domain 3	
solid.sp2Gp	solid.gpeval(solid.sp2)	N/m²	Second principal stress, Gauss point evaluation	Domain 3	
solid.sp3Gp	solid.gpeval(solid.sp3)	N/m²	Third principal stress, Gauss point evaluation	Domain 3	
solid.RFr	reacf(u)	N	Reaction force, r-component	Domain 3	
solid.RFphi	0	N	Reaction force, phi-component	Domain 3	
solid.RFz	reacf(w)	N	Reaction force, z-component	Domain 3	
solid.RMr	-solid.RFz*solid.refpntphi-solid.RFphi*(z-	N·m	Reaction moment, r-component	Domain 3	

Name	Expression	Unit	Description	Selection	Details
	solid.refpntz)				
solid.RMphi	-solid.RFz*(r-solid.refpntr)+solid.RFr*(z-solid.refpntz)	N·m	Reaction moment, phi-component	Domain 3	
solid.RMz	solid.RFphi*(r-solid.refpntr)+solid.RFr*solid.refpntphi	N·m	Reaction moment, z-component	Domain 3	

Shape functions

Name	Shape function	Unit	Description	Shape frame	Selection	Details
u	Nodal serendipity (Quadratic)	m	Displacement field, R-component	Material	Domain 3	
w	Nodal serendipity (Quadratic)	m	Displacement field, Z-component	Material	Domain 3	
solid.lemm1.pw	Nodal serendipity	N/m²	Auxiliary pressure	Material	Domain 3	+ operation
solid.lemm1.pw	Nodal serendipity	N/m²	Auxiliary pressure	Material	Boundaries 7–9, 16	+ operation, Slit

Weak Expressions

Weak expression	Integration order	Integration frame	Selection
2*(-solid.Sl11*var(solid.el11,u)-2*solid.Sl12*var(solid.el12,u)-2*solid.Sl13*var(solid.el13,u)-solid.Sl22*var(solid.el22,u)-2*solid.Sl23*var(solid.el23,u)-solid.Sl33*var(solid.el33,u))*pi*R	4	Material	Domain 3
2*(solid.lemm1.pw-solid.pp)*test(solid.lemm1.pw)*pi*R/solid.K	4	Material	Domain 3

2.8.4 Axial Symmetry 1

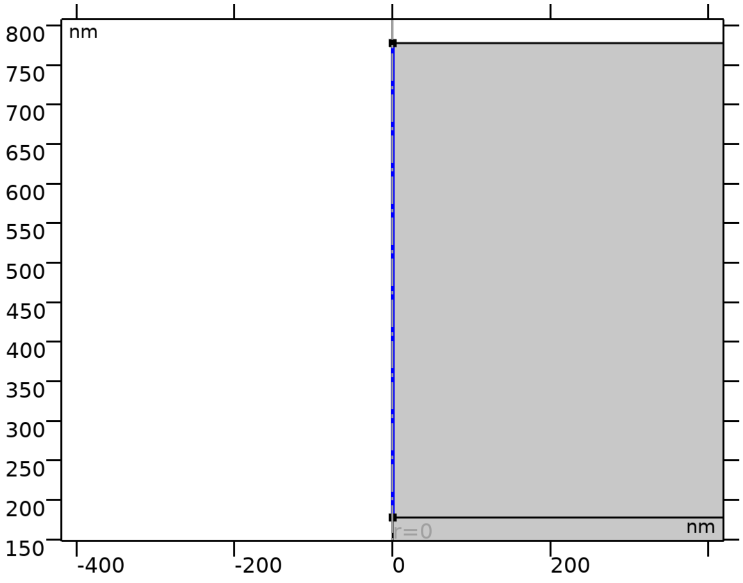

Axial Symmetry 1

SELECTION

Geometric entity level	Boundary
Selection	Geometry geom1: Dimension 1: All boundaries

Variables

Name	Expression	Unit	Description	Selection	Details
solid.axi1.usr	u	m	Displacement, spatial coordinate system, r-component	Boundary 7	+ operation
solid.axi1.usphi	0	m	Displacement, spatial coordinate system, phi-component	Boundary 7	+ operation
solid.axi1.usz	w	m	Displacement, spatial coordinate system, z-component	Boundary 7	+ operation

Constraints

Constraint	Constraint force	Shape function	Selection	Details
-solid.axi1.usr	test(-solid.axi1.usr)	Nodal serendipity (Quadratic)	Boundary 7	Nodal

Constraint	Constraint force	Shape function	Selection	Details
0	0		Boundary 7	Nodal
0	0	Nodal serendipity (Quadratic)	Boundary 7	Nodal

2.8.5 Free 1

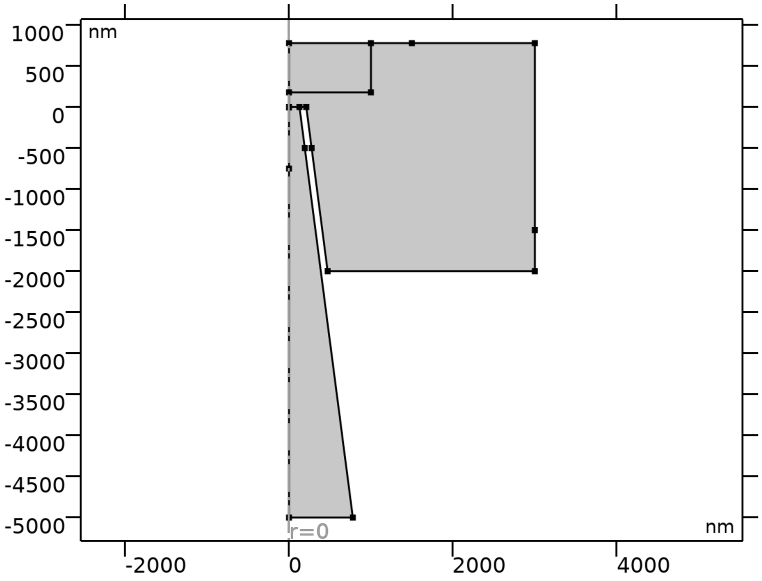

Free 1

SELECTION

Geometric entity level	Boundary
Selection	Geometry geom1: Dimension 1: All boundaries

2.8.6 Initial Values 1

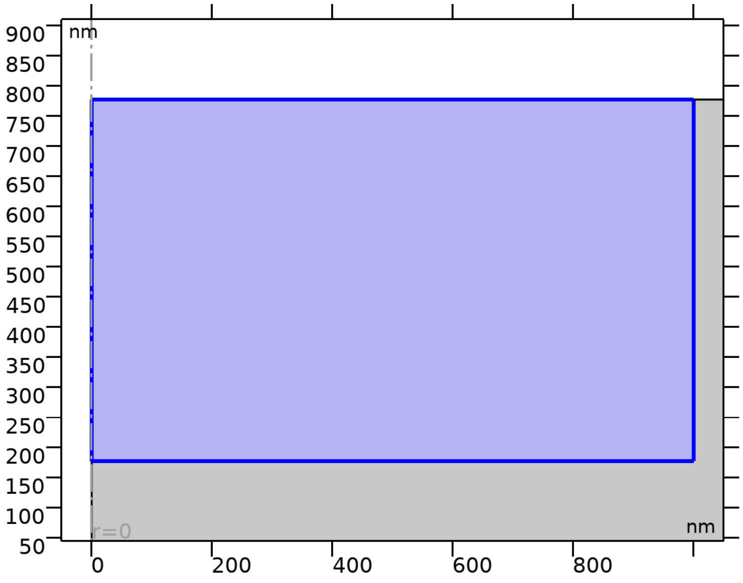

Initial Values 1

SELECTION

Geometric entity level	Domain
Selection	Geometry geom1: Dimension 2: All domains

Initial Values

SETTINGS

**Description**	**Value**	**Unit**
Displacement field, R-component	0	m
Displacement field, PHI-component	0	m
Displacement field, Z-component	0	m
Structural velocity field, R-component	0	m/s
Structural velocity field, PHI-component	0	m/s
Structural velocity field, Z-component	0	m/s

Coordinate System Selection

SETTINGS

**Description**	**Value**
Coordinate system	Global coordinate system

Variables

**Name**	**Expression**	**Unit**	**Description**	**Selection**
solid.init1.u1	0	m	Initial value of	Domain 3

Name	Expression	Unit	Description	Selection
			displacement, 1-component	
solid.init1.u2	0	m	Initial value of displacement, 2-component	Domain 3
solid.init1.u3	0	m	Initial value of displacement, 3-component	Domain 3
solid.init1.ut1	0	m/s	Initial value of structural velocity, 1-component	Domain 3
solid.init1.ut2	0	m/s	Initial value of structural velocity, 2-component	Domain 3
solid.init1.ut3	0	m/s	Initial value of structural velocity, 3-component	Domain 3
solid.uInitr	solid.init1.u1	m	Initial value of displacement, r-component	Domain 3
solid.uInitphi	solid.init1.u2	m	Initial value of displacement, phi-component	Domain 3
solid.uInitz	solid.init1.u3	m	Initial value of displacement, z-component	Domain 3
solid.utInitr	solid.init1.ut1	m/s	Initial value of structural velocity, r-component	Domain 3
solid.utInitphi	solid.init1.ut2	m/s	Initial value of structural velocity, phi-component	Domain 3
solid.utInitz	solid.init1.ut3	m/s	Initial value of structural velocity, z-component	Domain 3

2.8.7 Fixed Constraint 1

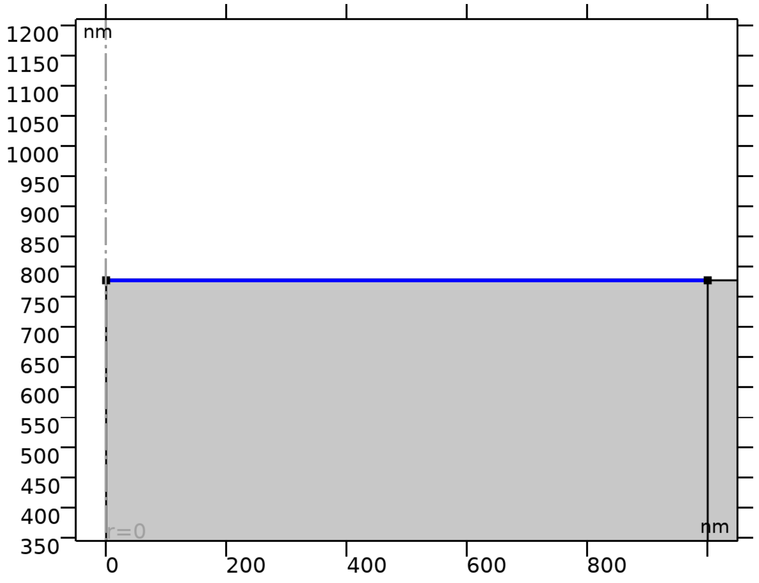

Fixed Constraint 1

SELECTION

Geometric entity level	Boundary
Selection	Geometry geom1: Dimension 1: Boundary 9

EQUATIONS

$$\mathbf{u} = \underset{\cdots}{\mathbf{0}}$$

Variables

Name	Expression	Unit	Description	Selection	Details
solid.fix1.usr	u	m	Displacement, spatial coordinate system, r-component	Boundary 9	+ operation
solid.fix1.usphi	0	m	Displacement, spatial coordinate system, phi-component	Boundary 9	+ operation
solid.fix1.usz	w	m	Displacement, spatial coordinate system, z-component	Boundary 9	+ operation

Constraints

Constraint	Constraint force	Shape function	Selection	Details

Constraint	Constraint force	Shape function	Selection	Details
-solid.fix1.usr	test(-solid.fix1.usr)	Nodal serendipity (Quadratic)	Boundary 9	Nodal
-solid.fix1.usphi	test(-solid.fix1.usphi)		Boundary 9	Nodal
-solid.fix1.usz	test(-solid.fix1.usz)	Nodal serendipity (Quadratic)	Boundary 9	Nodal

2.9 LAMINAR FLOW

USED PRODUCTS

COMSOL Multiphysics

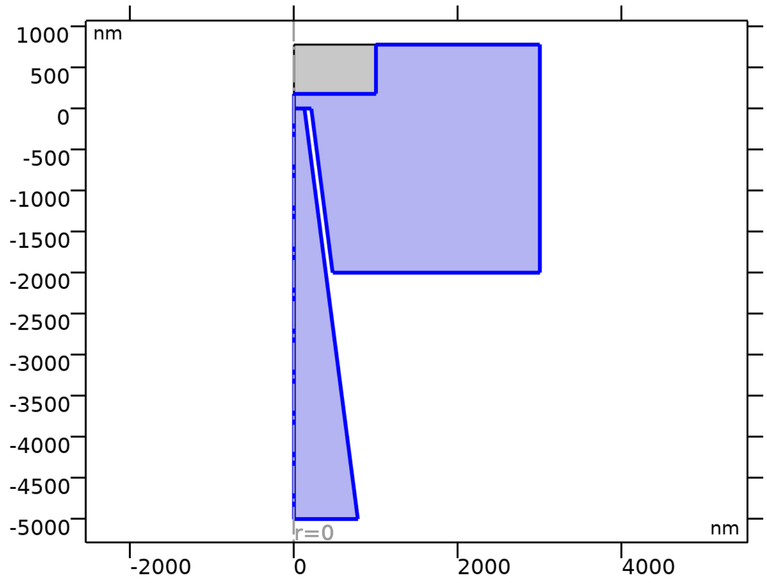

Laminar Flow

SELECTION

Geometric entity level	Domain
Selection	Geometry geom1: Dimension 2: Domains 1–2

EQUATIONS

$$\rho(\mathbf{u2}\cdot\nabla)\mathbf{u2} = \nabla\cdot[-p\mathbf{I} + \mathbf{K}] + \mathbf{F}$$
$$\rho\nabla\cdot\mathbf{u2} = 0$$

2.9.1 Interface Settings

Discretization

SETTINGS

Description	Value
Discretization of fluids	P1 + P1

SETTINGS

Description	Value
Equation form	Study controlled

Physical Model

SETTINGS

Description	Value	Unit
Neglect inertial term (Stokes flow)	Off	
Compressibility	Incompressible flow	
Enable porous media domains	Off	
Include gravity	Off	
Reference temperature	User defined	
Reference temperature	293.15	K
Reference pressure level	1.0133E5	Pa

Turbulence

SETTINGS

Description	Value
Turbulence model type	None

2.9.2 Variables

Name	Expression	Unit	Description	Selection	Details
spf.Tref	model.input.Tref	K	Reference temperature	Global	Meta
spf.dz	1	m	Thickness	Domains 1–2	
spf.pref	1[atm]	Pa	Reference pressure level	Domains 1–2	
spf.pA	p+spf.pref	Pa	Absolute pressure	Domains 1–2	
spf.hasWF	0		Help variable	Boundaries 1–5, 8, 10–20	
spf.hasWF_u	0		Help variable	Boundary 6	

Name	Expression	Unit	Description	Selection	Details
spf.hasWF_d	0		Help variable	Boundary 6	
spf.dt_CFL	1/max(spf.maxop(sqrt(emetric_spatial(u2-d(r,TIME),w2-d(z,TIME)))),eps)	s	Time step, CFL=1	Global	
spf.Qvd_tot	spf.intop(2*spf.Qvd*pi*r)	W	Total viscous dissipation	Global	
spf.K_stressr	spf.K_stress_tensorrr*spf.nrmesh+spf.K_stress_tensorrphi*spf.nphimesh+spf.K_stress_tensorrz*spf.nzmesh	N/m²	Viscous force, exterior boundaries, r-component	Boundaries 1–5, 8, 10–20	
spf.K_stressphi	spf.K_stress_tensorphir*spf.nrmesh+spf.K_stress_tensorphiphi*spf.nphimesh+spf.K_stress_tensorphiz*spf.nzmesh	N/m²	Viscous force, exterior boundaries, phi-component	Boundaries 1–5, 8, 10–20	
spf.K_stressz	spf.K_stress_tensorzr*spf.nrmesh+spf.K_stress_tensorzphi*spf.nphimesh+spf.K_stress_tensorzz*spf.nzmesh	N/m²	Viscous force, exterior boundaries, z-component	Boundaries 1–5, 8, 10–20	
spf.T_stressr	spf.T_stress_tensorrr*spf.nrmesh+spf.T_stress_tensorrphi*spf.nphimesh+spf.T_stress_tensorrz*spf.nzmesh	N/m²	Total traction, exterior boundaries, r-component	Boundaries 1–5, 8, 10–20	
spf.T_stressphi	spf.T_stress_tensorphir*spf.nrmesh+spf.T_stress_tensorphiphi*spf.nphimesh+spf.T_stress_tensorphiz*spf.nzmesh	N/m²	Total traction, exterior boundaries, phi-component	Boundaries 1–5, 8, 10–20	
spf.T_stressz	spf.T_stress_tensorzr*spf.nrmesh+spf.T_stress_tensorzphi*spf.nphimesh+spf.T_stress_tensorzz*spf.nzmesh	N/m²	Total traction, exterior boundaries, z-component	Boundaries 1–5, 8, 10–20	
spf.K_stress_dr	down(spf.K_stress_tensorrr)*spf.nrmesh+down(spf.K_stress_tensorrphi)*spf.nphimesh+down(spf.K_stress_tens	N/m²	Viscous force, interior boundaries, downside, r-component	Boundary 6	

Name	Expression	Unit	Description	Selection	Details
	orrz)*spf.nzmesh				
spf.K_stress_dphi	down(spf.K_stress_tensorphir)*spf.nrmesh+down(spf.K_stress_tensorphiphi)*spf.nphimesh+down(spf.K_stress_tensorphiz)*spf.nzmesh	N/m²	Viscous force, interior boundaries, downside, phi-component	Boundary 6	
spf.K_stress_dz	down(spf.K_stress_tensorzr)*spf.nrmesh+down(spf.K_stress_tensorzphi)*spf.nphimesh+down(spf.K_stress_tensorzz)*spf.nzmesh	N/m²	Viscous force, interior boundaries, downside, z-component	Boundary 6	
spf.K_stress_dr	down(spf.K_stress_tensorrr)*spf.dnrmesh+down(spf.K_stress_tensorrphi)*spf.dnphimesh+down(spf.K_stress_tensorrz)*spf.dnzmesh	N/m²	Viscous force, interior boundaries, downside, r-component	Boundaries 1–5, 10–15, 17–20	
spf.K_stress_dphi	down(spf.K_stress_tensorphir)*spf.dnrmesh+down(spf.K_stress_tensorphiphi)*spf.dnphimesh+down(spf.K_stress_tensorphiz)*spf.dnzmesh	N/m²	Viscous force, interior boundaries, downside, phi-component	Boundaries 1–5, 10–15, 17–20	
spf.K_stress_dz	down(spf.K_stress_tensorzr)*spf.dnrmesh+down(spf.K_stress_tensorzphi)*spf.dnphimesh+down(spf.K_stress_tensorzz)*spf.dnzmesh	N/m²	Viscous force, interior boundaries, downside, z-component	Boundaries 1–5, 10–15, 17–20	
spf.K_stress_ur	-up(spf.K_stress_tensorrr)*spf.nrmesh-up(spf.K_stress_tensorrphi)*spf.nphimesh-up(spf.K_stress_tensorrz)*spf.nzmesh	N/m²	Viscous force, interior boundaries, upside, r-component	Boundary 6	
spf.K_stress_uphi	-up(spf.K_stress_tensorphir)*spf.nrmesh-up(spf.K_stress_tensorphiphi)*spf.nphimesh-up(spf.K_stress_tensor	N/m²	Viscous force, interior boundaries, upside, phi-component	Boundary 6	

Name	Expression	Unit	Description	Selection	Details
	phiz)*spf.nzmesh				
spf.K_stress_uz	-up(spf.K_stress_tensorzr)*spf.nrmesh-up(spf.K_stress_tensorzphi)*spf.nphimesh-up(spf.K_stress_tensorzz)*spf.nzmesh	N/m²	Viscous force, interior boundaries, upside, z-component	Boundary 6	
spf.K_stress_ur	up(spf.K_stress_tensorrr)*spf.unrmesh+up(spf.K_stress_tensorrphi)*spf.unphimesh+up(spf.K_stress_tensorrz)*spf.unzmesh	N/m²	Viscous force, interior boundaries, upside, r-component	Boundaries 8, 16	
spf.K_stress_uphi	up(spf.K_stress_tensorphir)*spf.unrmesh+up(spf.K_stress_tensorphiphi)*spf.unphimesh+up(spf.K_stress_tensorphiz)*spf.unzmesh	N/m²	Viscous force, interior boundaries, upside, phi-component	Boundaries 8, 16	
spf.K_stress_uz	up(spf.K_stress_tensorzr)*spf.unrmesh+up(spf.K_stress_tensorzphi)*spf.unphimesh+up(spf.K_stress_tensorzz)*spf.unzmesh	N/m²	Viscous force, interior boundaries, upside, z-component	Boundaries 8, 16	
spf.T_stress_dr	down(spf.T_stress_tensorrr)*spf.nrmesh+down(spf.T_stress_tensorrphi)*spf.nphimesh+down(spf.T_stress_tensorrz)*spf.nzmesh	N/m²	Total traction, interior boundaries, downside, r-component	Boundary 6	
spf.T_stress_dphi	down(spf.T_stress_tensorphir)*spf.nrmesh+down(spf.T_stress_tensorphiphi)*spf.nphimesh+down(spf.T_stress_tensorphiz)*spf.nzmesh	N/m²	Total traction, interior boundaries, downside, phi-component	Boundary 6	
spf.T_stress_dz	down(spf.T_stress_tensorzr)*spf.nrmesh+down(spf.T_stress_tensorzphi)*spf.nphimesh+down(spf.T_stress_tensorzz)*spf.nzmesh	N/m²	Total traction, interior boundaries, downside, z-component	Boundary 6	
spf.T_stress_dr	down(spf.T_stress_ten	N/m²	Total traction,	Boundaries 1–	

Name	Expression	Unit	Description	Selection	Details
	sorrr)*spf.dnrmesh+down(spf.T_stress_tensorrphi)*spf.dnphimesh+down(spf.T_stress_tensorrz)*spf.dnzmesh		interior boundaries, downside, r-component	5, 10–15, 17–20	
spf.T_stress_dphi	down(spf.T_stress_tensorphir)*spf.dnrmesh+down(spf.T_stress_tensorphiphi)*spf.dnphimesh+down(spf.T_stress_tensorphiz)*spf.dnzmesh	N/m²	Total traction, interior boundaries, downside, phi-component	Boundaries 1–5, 10–15, 17–20	
spf.T_stress_dz	down(spf.T_stress_tensorzr)*spf.dnrmesh+down(spf.T_stress_tensorzphi)*spf.dnphimesh+down(spf.T_stress_tensorzz)*spf.dnzmesh	N/m²	Total traction, interior boundaries, downside, z-component	Boundaries 1–5, 10–15, 17–20	
spf.T_stress_ur	-up(spf.T_stress_tensorrr)*spf.nrmesh-up(spf.T_stress_tensorrphi)*spf.nphimesh-up(spf.T_stress_tensorrz)*spf.nzmesh	N/m²	Total traction, interior boundaries, upside, r-component	Boundary 6	
spf.T_stress_uphi	-up(spf.T_stress_tensorphir)*spf.nrmesh-up(spf.T_stress_tensorphiphi)*spf.nphimesh-up(spf.T_stress_tensorphiz)*spf.nzmesh	N/m²	Total traction, interior boundaries, upside, phi-component	Boundary 6	
spf.T_stress_uz	-up(spf.T_stress_tensorzr)*spf.nrmesh-up(spf.T_stress_tensorzphi)*spf.nphimesh-up(spf.T_stress_tensorzz)*spf.nzmesh	N/m²	Total traction, interior boundaries, upside, z-component	Boundary 6	
spf.T_stress_ur	up(spf.T_stress_tensorrr)*spf.unrmesh+up(spf.T_stress_tensorrphi)*spf.unphimesh+up(spf.T_stress_tensorrz)*spf.unzmesh	N/m²	Total traction, interior boundaries, upside, r-component	Boundaries 8, 16	

Name	Expression	Unit	Description	Selection	Details
spf.T_stress_uphi	up(spf.T_stress_tensorphir)*spf.unrmesh+up(spf.T_stress_tensorphiphi)*spf.unphimesh+up(spf.T_stress_tensorphiz)*spf.unzmesh	N/m²	Total traction, interior boundaries, upside, phi-component	Boundaries 8, 16	
spf.T_stress_uz	up(spf.T_stress_tensorzr)*spf.unrmesh+up(spf.T_stress_tensorzphi)*spf.unphimesh+up(spf.T_stress_tensorzz)*spf.unzmesh	N/m²	Total traction, interior boundaries, upside, z-component	Boundaries 8, 16	
spf.usePseudoTimeStepping	isrunningpseudotimestepping	1	Help variable	Global	
spf.localCFLvalue	1.3^min(niterCMP,9)+if(niterCMP>=25,9*1.3^min(-25+niterCMP,9),0)+if(niterCMP>=45,90*1.3^min(-45+niterCMP,9),0)		Local CFL number	Domains 1–2	
spf.locCFL	max(CFLCMP,sqrt(eps))	1	Local CFL number	Global	
spf.geometryLengthScale	7.5E-7	m	Geometry length scale	Domains 1–2	
spf.time_step_inv	max(sqrt(emetric_spatial(u2,w2)*2^gmg_level^2),spf.nu/spf.geometryLengthScale^2)	Hz	Inverse time step	Domains 1–2	
spf.tsti	nojac(spf.time_step_inv/spf.locCFL)	1/s	Help variable	Domains 1–2	
spf.nr	nr	1	Normal vector, r-component	Boundary 6	
spf.nphi	0	1	Normal vector, phi-component	Boundary 6	
spf.nz	nz	1	Normal vector, z-component	Boundary 6	
spf.nr	unr	1	Normal vector, r-component	Boundaries 8, 16	
spf.nphi	0	1	Normal vector, phi-component	Boundaries 8, 16	

Name	Expression	Unit	Description	Selection	Details
spf.nz	unz	1	Normal vector, z-component	Boundaries 8, 16	
spf.nr	dnr	1	Normal vector, r-component	Boundaries 1–5, 10–15, 17–20	
spf.nphi	0	1	Normal vector, phi-component	Boundaries 1–5, 10–15, 17–20	
spf.nz	dnz	1	Normal vector, z-component	Boundaries 1–5, 10–15, 17–20	
spf.nrmesh	nrmesh	1	Normal vector, r-component	Boundary 6	
spf.nphimesh	0	1	Normal vector, phi-component	Boundary 6	
spf.nzmesh	nzmesh	1	Normal vector, z-component	Boundary 6	
spf.nrmesh	unrmesh	1	Normal vector, r-component	Boundaries 8, 16	
spf.nphimesh	0	1	Normal vector, phi-component	Boundaries 8, 16	
spf.nzmesh	unzmesh	1	Normal vector, z-component	Boundaries 8, 16	
spf.nrmesh	dnrmesh	1	Normal vector, r-component	Boundaries 1–5, 10–15, 17–20	
spf.nphimesh	0	1	Normal vector, phi-component	Boundaries 1–5, 10–15, 17–20	
spf.nzmesh	dnzmesh	1	Normal vector, z-component	Boundaries 1–5, 10–15, 17–20	

2.9.3 Fluid Properties 1

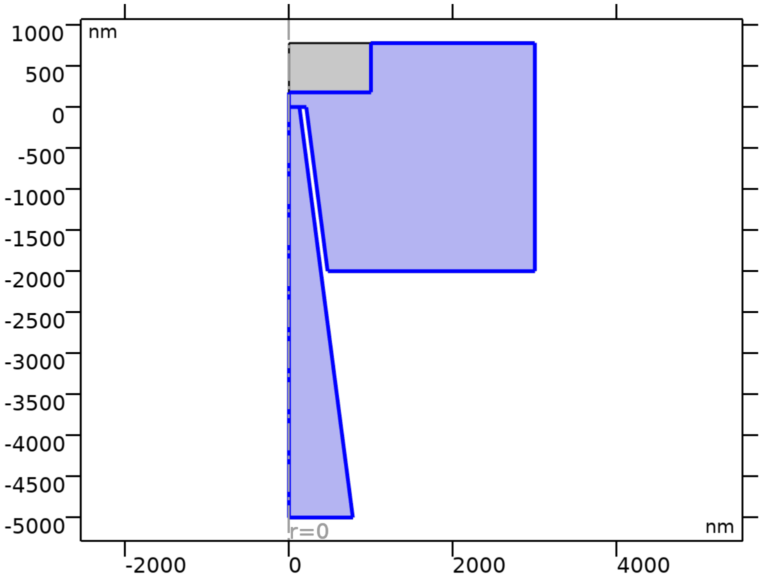

Fluid Properties 1

SELECTION

Geometric entity level	Domain
Selection	Geometry geom1: Dimension 2: All domains

EQUATIONS

$$\rho(\mathbf{u2}\cdot\nabla)\mathbf{u2} = \nabla\cdot[-p\mathbf{I} + \mathbf{K}] + \mathbf{F}$$
$$\rho\nabla\cdot\mathbf{u2} = 0$$
$$\mathbf{K} = \mu\left(\nabla\mathbf{u2} + (\nabla\mathbf{u2})^T\right)$$

Fluid Properties

SETTINGS

Description	Value
Density	From material
	Specify dynamic viscosity
Dynamic viscosity	From material

Model Input

SETTINGS

Description	Value
Temperature	Common model input

PROPERTIES FROM MATERIAL

Property	Material	Property group
Density	water	Basic
Dynamic viscosity	water	Basic

Variables

Name	Expression	Unit	Description	Selection	Details
spf.mu	material.mu	Pa·s	Dynamic viscosity	Domains 1–2	Meta
spf.rho	subst(material.rho,spf.fp1.minput_temperature,spf.Trho,spf.fp1.minput_pressure,spf.prho)	kg/m³	Density	Domains 1–2	Meta
prt	prTIME-prr*d(r,TIME)-prz*d(z,TIME)	W/m⁴	Gradient of p, r component, first time derivative	Domains 1–2	
pzt	pzTIME-pzr*d(r,TIME)-pzz*d(z,TIME)	W/m⁴	Gradient of p, z component, first time derivative	Domains 1–2	
prtt	d(prTIME-prr*d(r,TIME)-prz*d(z,TIME),TIME)-d(prTIME-prr*d(r,TIME)-prz*d(z,TIME),r)*d(r,TIME)-d(prTIME-prr*d(r,TIME)-prz*d(z,TIME),z)*d(z,TIME)	kg/(m²·s⁴)	Gradient of p, r component, second time derivative	Domains 1–2	
pztt	d(pzTIME-pzr*d(r,TIME)-pzz*d(z,TIME),TIME)-d(pzTIME-pzr*d(r,TIME)-pzz*d(z,TIME),r)*d(r,TIME)-d(pzTIME-pzr*d(r,TIME)-pzz*d(z,TIME),z)*d(z,TIME)	kg/(m²·s⁴)	Gradient of p, z component, second time derivative	Domains 1–2	
pt	pTIME-pr*d(r,TIME)-pz*d(z,TIME)	Pa/s	Pressure, first time derivative	Domains 1–2	
ptt	d(pTIME-pr*d(r,TIME)-pz*d(z,TIME),TIME)-d(pTIME-	Pa/s²	Pressure, second time derivative	Domains 1–2	

Name	Expression	Unit	Description	Selection	Details
	pr*d(r,TIME)-pz*d(z,TIME),r)*d(r,TIME)-d(pTIME-pr*d(r,TIME)-pz*d(z,TIME),z)*d(z,TIME)				
u2rt	u2rTIME-u2rr*d(r,TIME)-u2rz*d(z,TIME)	$1/s^2$	Gradient of u2, r component, first time derivative	Domains 1–2	
u2zt	u2zTIME-u2zr*d(r,TIME)-u2zz*d(z,TIME)	$1/s^2$	Gradient of u2, z component, first time derivative	Domains 1–2	
u2rtt	d(u2rTIME-u2rr*d(r,TIME)-u2rz*d(z,TIME),TIME)-d(u2rTIME-u2rr*d(r,TIME)-u2rz*d(z,TIME),r)*d(r,TIME)-d(u2rTIME-u2rr*d(r,TIME)-u2rz*d(z,TIME),z)*d(z,TIME)	$1/s^3$	Gradient of u2, r component, second time derivative	Domains 1–2	
u2ztt	d(u2zTIME-u2zr*d(r,TIME)-u2zz*d(z,TIME),TIME)-d(u2zTIME-u2zr*d(r,TIME)-u2zz*d(z,TIME),r)*d(r,TIME)-d(u2zTIME-u2zr*d(r,TIME)-u2zz*d(z,TIME),z)*d(z,TIME)	$1/s^3$	Gradient of u2, z component, second time derivative	Domains 1–2	
w2rt	w2rTIME-w2rr*d(r,TIME)-w2rz*d(z,TIME)	$1/s^2$	Gradient of w2, r component, first time derivative	Domains 1–2	
w2zt	w2zTIME-w2zr*d(r,TIME)-w2zz*d(z,TIME)	$1/s^2$	Gradient of w2, z component, first time derivative	Domains 1–2	
w2rtt	d(w2rTIME-w2rr*d(r,TIME)-w2rz*d(z,TIME),TIME)-d(w2rTIME-w2rr*d(r,TIME)-w2rz*d(z,TIME),r)*d(r,TIME)-d(w2rTIME-	$1/s^3$	Gradient of w2, r component, second time derivative	Domains 1–2	

Name	Expression	Unit	Description	Selection	Details
	w2rr*d(r,TIME)-w2rz*d(z,TIME),z)*d(z,TIME)				
w2ztt	d(w2zTIME-w2zr*d(r,TIME)-w2zz*d(z,TIME),TIME)-d(w2zTIME-w2zr*d(r,TIME)-w2zz*d(z,TIME),r)*d(r,TIME)-d(w2zTIME-w2zr*d(r,TIME)-w2zz*d(z,TIME),z)*d(z,TIME)	1/s³	Gradient of w2, z component, second time derivative	Domains 1–2	
u2t	u2TIME-u2r*d(r,TIME)-u2z*d(z,TIME)	m/s²	Velocity field, first time derivative, r-component	Domains 1–2	
w2t	w2TIME-w2r*d(r,TIME)-w2z*d(z,TIME)	m/s²	Velocity field, first time derivative, z-component	Domains 1–2	
u2tt	d(u2TIME-u2r*d(r,TIME)-u2z*d(z,TIME),TIME)-d(u2TIME-u2r*d(r,TIME)-u2z*d(z,TIME),r)*d(r,TIME)-d(u2TIME-u2r*d(r,TIME)-u2z*d(z,TIME),z)*d(z,TIME)	m/s³	Velocity field, second time derivative, r-component	Domains 1–2	
w2tt	d(w2TIME-w2r*d(r,TIME)-w2z*d(z,TIME),TIME)-d(w2TIME-w2r*d(r,TIME)-w2z*d(z,TIME),r)*d(r,TIME)-d(w2TIME-w2r*d(r,TIME)-w2z*d(z,TIME),z)*d(z,TIME)	m/s³	Velocity field, second time derivative, z-component	Domains 1–2	
spf.Trho	spf.Tref	K	Temperature for density evaluation	Domains 1–2	
spf.prho	spf.pref	Pa	Pressure for the evaluation of density	Domains 1–2	

Name	Expression	Unit	Description	Selection	Details
spf.rhoref	subst(material.rho,spf.fp1.minput_temperature,spf.Tref,spf.fp1.minput_pressure,spf.pref)	kg/m³	Reference density	Domains 1–2	Meta
spf.mumat	material.mu	Pa·s	Dynamic viscosity	Domains 1–2	Meta
spf.srijrr	u2r	1/s	Strain rate tensor, rr-component	Domains 1–2	
spf.srijphir	0	1/s	Strain rate tensor, phir-component	Domains 1–2	
spf.srijzr	0.5*(w2r+u2z)	1/s	Strain rate tensor, zr-component	Domains 1–2	
spf.srijrphi	0	1/s	Strain rate tensor, rphi-component	Domains 1–2	
spf.srijphiphi	if(abs(r)<0.001*h_spatial,u2r,u2/r)	1/s	Strain rate tensor, phiphi-component	Domains 1–2	
spf.srijzphi	0	1/s	Strain rate tensor, zphi-component	Domains 1–2	
spf.srijrz	0.5*(u2z+w2r)	1/s	Strain rate tensor, rz-component	Domains 1–2	
spf.srijphiz	0	1/s	Strain rate tensor, phiz-component	Domains 1–2	
spf.srijzz	w2z	1/s	Strain rate tensor, zz-component	Domains 1–2	
spf.rrijrr	0	1/s	Rotation rate tensor, rr-component	Domains 1–2	
spf.rrijphir	0	1/s	Rotation rate tensor, phir-component	Domains 1–2	
spf.rrijzr	0.5*(w2r-u2z)	1/s	Rotation rate tensor, zr-component	Domains 1–2	
spf.rrijrphi	0	1/s	Rotation rate tensor, rphi-component	Domains 1–2	
spf.rrijphiphi	0	1/s	Rotation rate tensor, phiphi-component	Domains 1–2	
spf.rrijzphi	0	1/s	Rotation rate tensor, zphi-	Domains 1–2	

Name	Expression	Unit	Description	Selection	Details
			component		
spf.rrijrz	0.5*(u2z-w2r)	1/s	Rotation rate tensor, rz-component	Domains 1–2	
spf.rrijphiz	0	1/s	Rotation rate tensor, phiz-component	Domains 1–2	
spf.rrijzz	0	1/s	Rotation rate tensor, zz-component	Domains 1–2	
spf.sr	sqrt(2*spf.srijrr^2+2*spf.srijrphi^2+2*spf.srijrz^2+2*spf.srijphir^2+2*spf.srijphiphi^2+2*spf.srijphiz^2+2*spf.srijzr^2+2*spf.srijzphi^2+2*spf.srijzz^2+eps)	1/s	Shear rate	Domains 1–2	
spf.rr	sqrt(2*spf.rrijrr^2+2*spf.rrijrphi^2+2*spf.rrijrz^2+2*spf.rrijphir^2+2*spf.rrijphiphi^2+2*spf.rrijphiz^2+2*spf.rrijzr^2+2*spf.rrijzphi^2+2*spf.rrijzz^2+eps)	1/s	Rotation rate	Domains 1–2	
spf.divu	u2r+if(abs(r)<0.001*h_spatial,u2r,u2/r)+w2z	1/s	Divergence of velocity field	Domains 1–2	
spf.Fr	0	N/m³	Volume force, r-component	Domains 1–2	+ operation
spf.Fphi	0	N/m³	Volume force, phi-component	Domains 1–2	+ operation
spf.Fz	0	N/m³	Volume force, z-component	Domains 1–2	+ operation
spf.U	sqrt(u2^2+w2^2)	m/s	Velocity magnitude	Domains 1–2	
spf.vorticityr	0	1/s	Vorticity field, r-component	Domains 1–2	
spf.vorticityphi	-w2r+u2z	1/s	Vorticity field, phi-component	Domains 1–2	
spf.vorticityz	0	1/s	Vorticity field, z-component	Domains 1–2	

Name	Expression	Unit	Description	Selection	Details
spf.vort_magn	sqrt(spf.vorticityr^2+spf.vorticityphi^2+spf.vorticityz^2)	1/s	Vorticity magnitude	Domains 1–2	
spf.cellRe	0.25*spf.rho*sqrt(emetric_spatial(u2-d(r,TIME),w2-d(z,TIME))/emetric2_spatial)/spf.mu	1	Cell Reynolds number	Domains 1–2	
spf.nu	spf.mu/spf.rho	m^2/s	Kinematic viscosity	Domains 1–2	
spf.betaT	0	1/Pa	Isothermal compressibility coefficient	Domains 1–2	
spf.Qm	0	$kg/(m^3\cdot s)$	Source term	Domains 1–2	+ operation
spf.Fgtotr	0	N/m^3	Gravity force, r-component	Domains 1–2	+ operation
spf.Fgtotphi	0	N/m^3	Gravity force, phi-component	Domains 1–2	+ operation
spf.Fgtotz	0	N/m^3	Gravity force, z-component	Domains 1–2	+ operation
spf.Qm_aco	0	$kg/(m^3\cdot s)$	Acoustic mass source	Domains 1–2	
spf.F_acor	0	N/m^3	Acoustic volume force, r-component	Domains 1–2	
spf.F_acophi	0	N/m^3	Acoustic volume force, phi-component	Domains 1–2	
spf.F_acoz	0	N/m^3	Acoustic volume force, z-component	Domains 1–2	
spf.gamma_sr	sqrt(2*spf.srijrr^2+2*spf.srijrphi^2+2*spf.srijrz^2+2*spf.srijphir^2+2*spf.srijphiphi^2+2*spf.srijphiz^2+2*spf.srijzr^2+2*spf.srijzphi^2+2*spf.srijzz^2+eps)	1/s	Shear rate	Domains 1–2	
spf.mu_eff	spf.mu+spf.muT	Pa·s	Effective dynamic viscosity	Domains 1–2	
spf.muT	0	Pa·s	Turbulent	Domains 1–2	+ operation

Name	Expression	Unit	Description	Selection	Details
			dynamic viscosity		
spf.T_stress_tensorr r	spf.K_stress_tensorr r-p	N/m²	Total stress tensor, rr-component	Domains 1–2	+ operation
spf.T_stress_tensor phir	spf.K_stress_tensor phir	N/m²	Total stress tensor, phir-component	Domains 1–2	+ operation
spf.T_stress_tensor zr	spf.K_stress_tensorz r	N/m²	Total stress tensor, zr-component	Domains 1–2	+ operation
spf.T_stress_tensorr phi	spf.K_stress_tensorr phi	N/m²	Total stress tensor, rphi-component	Domains 1–2	+ operation
spf.T_stress_tensor phiphi	spf.K_stress_tensor phiphi-p	N/m²	Total stress tensor, phiphi-component	Domains 1–2	+ operation
spf.T_stress_tensor zphi	spf.K_stress_tensorz phi	N/m²	Total stress tensor, zphi-component	Domains 1–2	+ operation
spf.T_stress_tensorr z	spf.K_stress_tensorr z	N/m²	Total stress tensor, rz-component	Domains 1–2	+ operation
spf.T_stress_tensor phiz	spf.K_stress_tensor phiz	N/m²	Total stress tensor, phiz-component	Domains 1–2	+ operation
spf.T_stress_tensor zz	spf.K_stress_tensorz z-p	N/m²	Total stress tensor, zz-component	Domains 1–2	+ operation
spf.K_stress_tensorr r	2*spf.mu_eff*u2r	N/m²	Viscous stress tensor, rr-component	Domains 1–2	+ operation
spf.K_stress_tensor phir	0	N/m²	Viscous stress tensor, phir-component	Domains 1–2	+ operation
spf.K_stress_tensor zr	spf.mu_eff*(w2r+u2 z)	N/m²	Viscous stress tensor, zr-component	Domains 1–2	+ operation
spf.K_stress_tensorr phi	0	N/m²	Viscous stress tensor, rphi-component	Domains 1–2	+ operation
spf.K_stress_tensor phiphi	2*spf.mu_eff*if(abs(r)<0.001*h_spatial,u 2r,u2/r)	N/m²	Viscous stress tensor, phiphi-component	Domains 1–2	+ operation

Name	Expression	Unit	Description	Selection	Details
spf.K_stress_tensor zphi	0	N/m²	Viscous stress tensor, zphi-component	Domains 1–2	+ operation
spf.K_stress_tensorr z	spf.mu_eff*(u2z+w2 r)	N/m²	Viscous stress tensor, rz-component	Domains 1–2	+ operation
spf.K_stress_tensor phiz	0	N/m²	Viscous stress tensor, phiz-component	Domains 1–2	+ operation
spf.K_stress_tensor zz	2*spf.mu_eff*w2z	N/m²	Viscous stress tensor, zz-component	Domains 1–2	+ operation
spf.K_stress_tensor _testrr	2*spf.mu_eff*test(u 2r)	N/m²	Viscous stress tensor test, rr-component	Domains 1–2	+ operation
spf.K_stress_tensor _testphir	0	N/m²	Viscous stress tensor test, phir-component	Domains 1–2	+ operation
spf.K_stress_tensor _testzr	spf.mu_eff*(test(w2r)+test(u2z))	N/m²	Viscous stress tensor test, zr-component	Domains 1–2	+ operation
spf.K_stress_tensor _testrphi	0	N/m²	Viscous stress tensor test, rphi-component	Domains 1–2	+ operation
spf.K_stress_tensor _testphiphi	2*spf.mu_eff*if(abs(r)<0.001*h_spatial,t est(u2r),test(u2)/r)	N/m²	Viscous stress tensor test, phiphi-component	Domains 1–2	+ operation
spf.K_stress_tensor _testzphi	0	N/m²	Viscous stress tensor test, zphi-component	Domains 1–2	+ operation
spf.K_stress_tensor _testrz	spf.mu_eff*(test(u2z)+test(w2r))	N/m²	Viscous stress tensor test, rz-component	Domains 1–2	+ operation
spf.K_stress_tensor _testphiz	0	N/m²	Viscous stress tensor test, phiz-component	Domains 1–2	+ operation
spf.K_stress_tensor _testzz	2*spf.mu_eff*test(w 2z)	N/m²	Viscous stress tensor test, zz-component	Domains 1–2	+ operation
spf.upwind_helpr	u2-d(r,TIME)	m/s	Upwind term, r-component	Domains 1–2	+ operation

Name	Expression	Unit	Description	Selection	Details
spf.upwind_helpphi	0	m/s	Upwind term, phi-component	Domains 1–2	+ operation
spf.upwind_helpz	w2-d(z,TIME)	m/s	Upwind term, z-component	Domains 1–2	+ operation
spf.continuityEquation	spf.rho*spf.divu	kg/(m³·s)	Continuity equation	Domains 1–2	
spf.contCoeff	spf.rho	kg/m³	Help variable	Domains 1–2	
spf.tau_vdrr	2*spf.mu*spf.srijrr	Pa	Viscous stress tensor, rr-component	Domains 1–2	+ operation
spf.tau_vdphir	2*spf.mu*spf.srijphir	Pa	Viscous stress tensor, phir-component	Domains 1–2	+ operation
spf.tau_vdzr	2*spf.mu*spf.srijzr	Pa	Viscous stress tensor, zr-component	Domains 1–2	+ operation
spf.tau_vdrphi	2*spf.mu*spf.srijrphi	Pa	Viscous stress tensor, rphi-component	Domains 1–2	+ operation
spf.tau_vdphiphi	2*spf.mu*spf.srijphiphi	Pa	Viscous stress tensor, phiphi-component	Domains 1–2	+ operation
spf.tau_vdzphi	2*spf.mu*spf.srijzphi	Pa	Viscous stress tensor, zphi-component	Domains 1–2	+ operation
spf.tau_vdrz	2*spf.mu*spf.srijrz	Pa	Viscous stress tensor, rz-component	Domains 1–2	+ operation
spf.tau_vdphiz	2*spf.mu*spf.srijphiz	Pa	Viscous stress tensor, phiz-component	Domains 1–2	+ operation
spf.tau_vdzz	2*spf.mu*spf.srijzz	Pa	Viscous stress tensor, zz-component	Domains 1–2	+ operation
spf.Qvd	spf.tau_vdrr*u2r+spf.tau_vdrz*u2z+spf.tau_vdphiphi*if(abs(r)<0.001*h_spatial,u2r,u2/r)+spf.tau_vdzr*w2r+spf.tau_vdzz*w2z	W/m³	Viscous dissipation	Domains 1–2	+ operation
spf.epsilon_p	1	1	Porosity	Domains 1–2	

Name	Expression	Unit	Description	Selection	Details
spf.epsilon_p_pos	1	1	Positive porosity	Domains 1–2	
spf.Fst_tensorrr	0	N/m²	Surface tension force, rr-component	Domains 1–2	+ operation
spf.Fst_tensorphir	0	N/m²	Surface tension force, phir-component	Domains 1–2	+ operation
spf.Fst_tensorzr	0	N/m²	Surface tension force, zr-component	Domains 1–2	+ operation
spf.Fst_tensorrphi	0	N/m²	Surface tension force, rphi-component	Domains 1–2	+ operation
spf.Fst_tensorphiphi	0	N/m²	Surface tension force, phiphi-component	Domains 1–2	+ operation
spf.Fst_tensorzphi	0	N/m²	Surface tension force, zphi-component	Domains 1–2	+ operation
spf.Fst_tensorrz	0	N/m²	Surface tension force, rz-component	Domains 1–2	+ operation
spf.Fst_tensorphiz	0	N/m²	Surface tension force, phiz-component	Domains 1–2	+ operation
spf.Fst_tensorzz	0	N/m²	Surface tension force, zz-component	Domains 1–2	+ operation
spf.res_u	pr+spf.rho*u2*u2r+spf.rho*w2*u2z-(d(2*u2r,r)+if(abs(r)<0.001*h_spatial,d(2*u2r,r),2*u2r/r)+d(u2z+w2r,z)-2*if(abs(r)<0.001*h_spatial,u2r,u2/r)/r)*spf.mu-spf.Fr	N/m³	Equation residual	Domains 1–2	
spf.res_v	-spf.Fphi	N/m³	Equation residual	Domains 1–2	
spf.res_w	spf.rho*u2*w2r+pz+spf.rho*w2*w2z-(d(w2r+u2z,r)+if(abs(r)<0.001*h_spatial,d(w2r+u2z,r),(w2r+	N/m³	Equation residual	Domains 1–2	

Name	Expression	Unit	Description	Selection	Details
	u2z)/r)+d(2*w2z,z))*spf.mu-spf.Fz				
spf.res_p	spf.rho*spf.divu	kg/(m³·s)	Pressure equation residual	Domains 1–2	

Shape functions

Name	Shape function	Unit	Description	Shape frame	Selection
u2	Lagrange (Linear)	m/s	Velocity field, r-component	Spatial	Domains 1–2
w2	Lagrange (Linear)	m/s	Velocity field, z-component	Spatial	Domains 1–2
p	Lagrange (Linear)	Pa	Pressure	Spatial	Domains 1–2

Weak Expressions

Weak expression	Integration order	Integration frame	Selection
2*((p-spf.K_stress_tensorrr)*test(u2r)-spf.K_stress_tensorrz*test(u2z)+(p-spf.K_stress_tensorphiphi)*if(abs(r)<0.001*h_spatial,test(u2r),test(u2)/r)-spf.K_stress_tensorzr*test(w2r)+(p-spf.K_stress_tensorzz)*test(w2z))*pi*r	2	Spatial	Domains 1–2
2*(spf.Fr*test(u2)+spf.Fz*test(w2))*pi*r	2	Spatial	Domains 1–2
2*spf.rho*(-(d(u2,r)*u2+d(u2,z)*w2)*test(u2)-(d(w2,r)*u2+d(w2,z)*w2)*test(w2))*pi*r	2	Spatial	Domains 1–2
-2*spf.continuityEquation*test(p)*pi*r	2	Spatial	Domains 1–2
2*spf.streamlinens*pi*r	2	Spatial	Domains 1–2
2*spf.crosswindns*pi*r	2	Spatial	Domains 1–2
2*if(spf.usePseudoTimeStepping>0,spf.rho*spf.tsti,0)*(-(u2-nojac(u2))*test(u2)-(w2-nojac(w2))*test(w2))*pi*r	2	Spatial	Domains 1–2

2.9.4 Initial Values 1

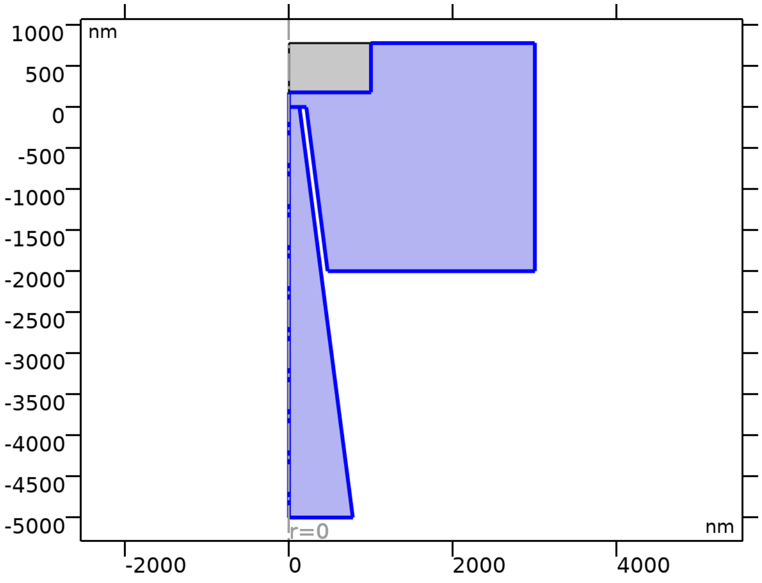

Initial Values 1

SELECTION

Geometric entity level	Domain
Selection	Geometry geom1: Dimension 2: All domains

Initial Values

SETTINGS

Description	Value	Unit
Velocity field, r-component	0	m/s
Velocity field, phi-component	0	m/s
Velocity field, z-component	0	m/s
Pressure	0	Pa

Coordinate System Selection

SETTINGS

Description	Value
Coordinate system	Global coordinate system

Variables

Name	Expression	Unit	Description	Selection
spf.u_initr	0	m/s	Velocity field, r-component	Domains 1–2
spf.u_initphi	0	m/s	Velocity field, phi-component	Domains 1–2
spf.u_initz	0	m/s	Velocity field, z-component	Domains 1–2

Name	Expression	Unit	Description	Selection
spf.p_init	0	Pa	Pressure	Domains 1–2

2.9.5 Axial Symmetry 1

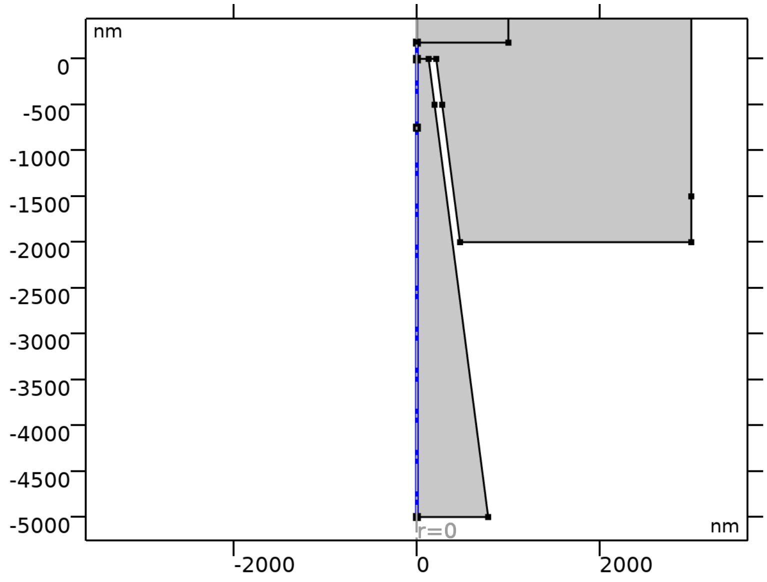

Axial Symmetry 1

SELECTION

Geometric entity level	Boundary
Selection	Geometry geom1: Dimension 1: All boundaries

Constraints

Constraint	Constraint force	Shape function	Selection	Details
-u2	test(-u2)	Lagrange (Linear)	Boundaries 1, 3–5	Elemental

2.9.6 Wall 1

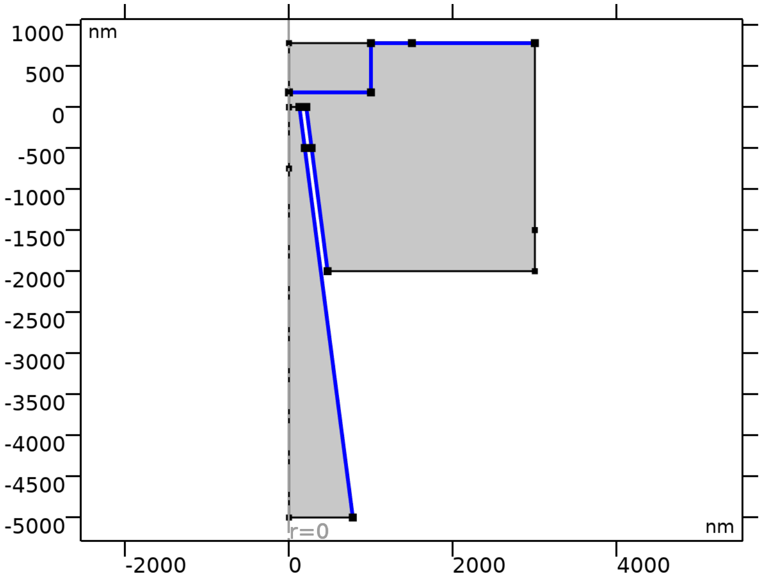

Wall 1

SELECTION

Geometric entity level	Boundary
Selection	Geometry geom1: Dimension 1: All boundaries

EQUATIONS

$$\mathbf{u2} = \mathbf{u}_{tr}$$

Boundary Condition

SETTINGS

Description	Value
Wall condition	No slip

Wall Movement

SETTINGS

Description	Value
Translational velocity	Automatic from frame
Sliding wall	Off

Variables

Name	Expression	Unit	Description	Selection	Details
spf.KStressn_avr	spf.K_stress_tensorrr*spf.nrmesh+spf.K_stress_tensorrphi*spf.np	N/m^2	Average viscous stress, r-component	Boundaries 8, 16	

Name	Expression	Unit	Description	Selection	Details
	himesh+spf.K_stress_tensorrz*spf.nzmesh				
spf.KStressn_avphi	spf.K_stress_tensorphir*spf.nrmesh+spf.K_stress_tensorphiphi*spf.nphimesh+spf.K_stress_tensorphiz*spf.nzmesh	N/m²	Average viscous stress, phi-component	Boundaries 8, 16	
spf.KStressn_avz	spf.K_stress_tensorzr*spf.nrmesh+spf.K_stress_tensorzphi*spf.nphimesh+spf.K_stress_tensorzz*spf.nzmesh	N/m²	Average viscous stress, z-component	Boundaries 8, 16	
spf.KStressTestn_avr	spf.K_stress_tensor_testrr*spf.nrmesh+spf.K_stress_tensor_testrphi*spf.nphimesh+spf.K_stress_tensor_testrz*spf.nzmesh	N/m²	Average viscous stress, r-component	Boundaries 8, 16	
spf.KStressTestn_avphi	spf.K_stress_tensor_testphir*spf.nrmesh+spf.K_stress_tensor_testphiphi*spf.nphimesh+spf.K_stress_tensor_testphiz*spf.nzmesh	N/m²	Average viscous stress, phi-component	Boundaries 8, 16	
spf.KStressTestn_avz	spf.K_stress_tensor_testzr*spf.nrmesh+spf.K_stress_tensor_testzphi*spf.nphimesh+spf.K_stress_tensor_testzz*spf.nzmesh	N/m²	Average viscous stress, z-component	Boundaries 8, 16	
spf.ujumpr	spf.u_herer-spf.u_therer	m/s	Velocity jump, r-component	Boundaries 8, 16	
spf.ujumpphi	spf.u_herephi-spf.u_therephi	m/s	Velocity jump, phi-component	Boundaries 8, 16	
spf.ujumpz	spf.u_herez-spf.u_therez	m/s	Velocity jump, z-component	Boundaries 8, 16	
spf.meshVol	meshvol_spatial	m		Boundaries 8, 16	
spf.meshVolInt	up(meshvol_spatial)	m²	Volume of interior mesh element	Boundaries 8, 16	
spf.sigma_dg_ns	4*spf.c_here	kg/(m²·s)		Boundaries 8, 16	

Name	Expression	Unit	Description	Selection	Details
spf.rhoFace	up(spf.rho)	kg/m^3	Density face value	Boundaries 8, 16	
spf.umxTnFace	(spf.upwind_helpr*spf.nrmesh+spf.upwind_helpphi*spf.nphimesh+spf.upwind_helpz*spf.nzmesh<0)*(spf.upwind_helpr*spf.nrmesh+spf.upwind_helpphi*spf.nphimesh+spf.upwind_helpz*spf.nzmesh)	m/s	Relative velocity on face	Boundaries 8, 16	
spf.ubndr	spf.utrr+spf.usr	m/s	Velocity at boundary, r-component	Boundaries 8, 10–14, 16–18	
spf.ubndphi	spf.utrphi+spf.usphi	m/s	Velocity at boundary, phi-component	Boundaries 8, 10–14, 16–18	
spf.ubndz	spf.utrz+spf.usz	m/s	Velocity at boundary, z-component	Boundaries 8, 10–14, 16–18	
spf.usr	0	m/s	Velocity of sliding wall, r-component	Boundaries 8, 10–14, 16–18	
spf.usphi	0	m/s	Velocity of sliding wall, phi-component	Boundaries 8, 10–14, 16–18	
spf.usz	0	m/s	Velocity of sliding wall, z-component	Boundaries 8, 10–14, 16–18	
spf.utrr	d(r,TIME)	m/s	Velocity of moving wall, r-component	Boundaries 8, 16	
spf.utrphi	0	m/s	Velocity of moving wall, phi-component	Boundaries 8, 16	
spf.utrz	d(z,TIME)	m/s	Velocity of moving wall, z-component	Boundaries 8, 16	
spf.utrr	0	m/s	Velocity of moving wall, r-component	Boundaries 10–14, 17–18	
spf.utrphi	0	m/s	Velocity of	Boundaries	

Name	Expression	Unit	Description	Selection	Details
			moving wall, phi-component	10–14, 17–18	
spf.utrz	0	m/s	Velocity of moving wall, z-component	Boundaries 10–14, 17–18	
spf.uLeakager	0	m/s	Leakage velocity, r-component	Boundaries 8, 10–14, 16–18	+ operation
spf.uLeakagephi	0	m/s	Leakage velocity, phi-component	Boundaries 8, 10–14, 16–18	+ operation
spf.uLeakagez	0	m/s	Leakage velocity, z-component	Boundaries 8, 10–14, 16–18	+ operation
spf.noSlipWall	1	1	Help variable	Boundaries 8, 10–14, 16–18	
spf.u_herer	u2	m/s	Intermediate variable, r-component	Boundaries 8, 16	
spf.u_herephi	0	m/s	Intermediate variable, phi-component	Boundaries 8, 16	
spf.u_herez	w2	m/s	Intermediate variable, z-component	Boundaries 8, 16	
spf.u_therer	spf.ubndr+spf.uLeakager	m/s	Intermediate variable, r-component	Boundaries 8, 16	
spf.u_therephi	spf.ubndphi+spf.uLeakagephi	m/s	Intermediate variable, phi-component	Boundaries 8, 16	
spf.u_therez	spf.ubndz+spf.uLeakagez	m/s	Intermediate variable, z-component	Boundaries 8, 16	
spf.c_here	24*nojac(up(spf.mu+spf.muT))*spf.meshVol/spf.meshVolInt	Pa·s/m	Intermediate variable	Boundaries 8, 16	
spf.contCoeffFace	up(spf.contCoeff)	kg/m³	Help variable	Boundaries 8, 16	
spf.upwind_ns	spf.rhoFace*spf.umxTnFace*(spf.ujumpr*test(spf.u_herer)+spf.ujumpphi*test(spf.u_herephi)+spf.ujumpz*test(spf.u_herez))	W/m²	Upwind term	Boundaries 8, 16	

Name	Expression	Unit	Description	Selection	Details
spf.upwindCont	spf.contCoeffFace*(spf.ujumpr*spf.nrmesh+spf.ujumpphi*spf.nphimesh+spf.ujumpz*spf.nzmesh)*test(p)	$kg^2/(m^3\cdot s^3)$	Upwind term for continuity equation	Boundaries 8, 16	
spf.pFace	p	Pa	Pressure face value	Boundaries 8, 16	
spf.consFlux	spf.pFace*(-test(spf.u_herer)*spf.nrmesh-test(spf.u_herephi)*spf.nphimesh-test(spf.u_herez)*spf.nzmesh)	W/m^2	Conservative flux	Boundaries 8, 16	+ operation

Weak Expressions

Weak expression	Integration order	Integration frame	Selection
2*(spf.KStressn_avr*test(spf.u_herer)+spf.KStressn_avphi*test(spf.u_herephi)+spf.KStressn_avz*test(spf.u_herez)+spf.KStressTestn_avr*spf.ujumpr+spf.KStressTestn_avphi*spf.ujumpphi+spf.KStressTestn_avz*spf.ujumpz-spf.sigma_dg_ns*spf.ujumpr*test(spf.u_herer)-spf.sigma_dg_ns*spf.ujumpphi*test(spf.u_herephi)-spf.sigma_dg_ns*spf.ujumpz*test(spf.u_herez)+spf.upwind_ns+spf.upwindCont+spf.consFlux)*pi*r	2	Spatial	Boundaries 8, 16

Constraints

Constraint	Constraint force	Shape function	Selection	Details
-u2+spf.ubndr+spf.uLeakager	test(-u2)	Lagrange (Linear)	Boundaries 10–14, 17–18	Elemental
spf.ubndphi+spf.uLeakagephi	0		Boundaries 10–14, 17–18	Elemental
-w2+spf.ubndz+spf.uLeakagez	test(-w2)	Lagrange (Linear)	Boundaries 10–14, 17–18	Elemental

2.9.7 Inlet 1

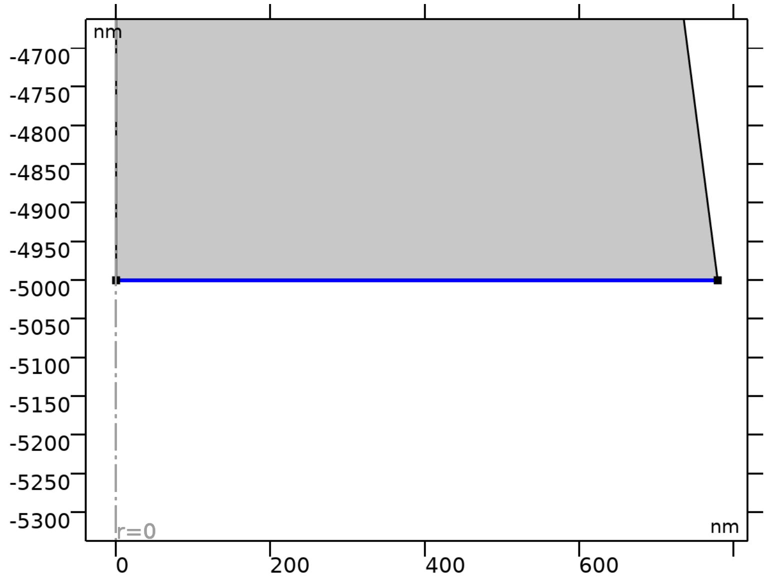

Inlet 1

SELECTION

Geometric entity level	Boundary
Selection	Geometry geom1: Dimension 1: Boundary 2

EQUATIONS

$$\mathbf{n}^T[-p\mathbf{I}+\mathbf{K}]\mathbf{n} = -\hat{p}_0$$
$$\hat{p}_0 \geq p_0\,,\ \mathbf{u2}\cdot\mathbf{t} = 0$$

Boundary Condition

SETTINGS

Description	Value
Boundary condition	Pressure

Pressure Conditions

SETTINGS

Description	Value	Unit
Pressure	Static	
Pressure	p0	Pa
Suppress backflow	On	
Flow direction	Normal flow	

Constraint Settings

SETTINGS

Description	Value
Apply reaction terms on	All physics (symmetric)
Use weak constraints	Off
Constraint method	Elemental

Variables

Name	Expression	Unit	Description	Selection
spf.KStressn_avr	spf.K_stress_tensorrr*spf.nrmesh+spf.K_stress_tensorrphi*spf.nphimesh+spf.K_stress_tensorrz*spf.nzmesh	N/m²	Average viscous stress, r-component	Boundary 2
spf.KStressn_avphi	spf.K_stress_tensorphir*spf.nrmesh+spf.K_stress_tensorphiphi*spf.nphimesh+spf.K_stress_tensorphiz*spf.nzmesh	N/m²	Average viscous stress, phi-component	Boundary 2
spf.KStressn_avz	spf.K_stress_tensorzr*spf.nrmesh+spf.K_stress_tensorzphi*spf.nphimesh+spf.K_stress_tensorzz*spf.nzmesh	N/m²	Average viscous stress, z-component	Boundary 2
spf.KStressTestn_avr	spf.K_stress_tensor_testrr*spf.nrmesh+spf.K_stress_tensor_testrphi*spf.nphimesh+spf.K_stress_tensor_testrz*spf.nzmesh	N/m²	Average viscous stress, r-component	Boundary 2
spf.KStressTestn_avphi	spf.K_stress_tensor_testphir*spf.nrmesh+spf.K_stress_tensor_testphiphi*spf.nphimesh+spf.K_stress_tensor_testphiz*spf.nzmesh	N/m²	Average viscous stress, phi-component	Boundary 2
spf.KStressTestn_avz	spf.K_stress_tensor_testzr*spf.nrmesh+spf.K_stress_tensor_testzphi*spf.nphimesh+spf.K_stress_tensor_testzz*spf.nzmesh	N/m²	Average viscous stress, z-component	Boundary 2
spf.ujumpr	spf.ut_herer-spf.ut_therer	m/s	Velocity jump, r-component	Boundary 2
spf.ujumpphi	spf.ut_herephi-spf.ut_therephi	m/s	Velocity jump, phi-component	Boundary 2
spf.ujumpz	spf.ut_herez-spf.ut_therez	m/s	Velocity jump, z-component	Boundary 2
spf.meshVol	meshvol_spatial	m		Boundary 2
spf.meshVolInt	down(meshvol_spatial)	m²	Volume of	Boundary 2

Name	Expression	Unit	Description	Selection
			interior mesh element	
spf.sigma_dg_ns	4*spf.ct_here	kg/(m²·s)		Boundary 2
spf.rhoFace	down(spf.rho)	kg/m³	Density face value	Boundary 2
spf.umxTnFace	spf.upwind_helpr*spf.nrmesh+spf.upwind_helpphi*spf.nphimesh+spf.upwind_helpz*spf.nzmesh	m/s	Relative velocity on face	Boundary 2
spf.p0	p0	Pa	Pressure	Boundary 2
spf.f0	spf.p0+spf.uNormal*(spf.backflowPenaltyDiff+spf.backflowPenaltyConv)*(spf.uNormal>0)	N/m²	Normal stress	Boundary 2
spf.uNormal	u2*nojac(spf.nrmesh)+w2*nojac(spf.nzmesh)	m/s	Normal velocity	Boundary 2
spf.backflowPenaltyDiff	spf.inl1.c_here*min((down(spf.mu)+spf.muT)*spf.meshVol/spf.meshVolInt,down(spf.rho)*abs(spf.uNormal)/down(spf.epsilon_p))	Pa·s/m	Backflow penalty parameter, diffusive contribution	Boundary 2
spf.backflowPenaltyConv	spf.rhoFace*spf.umxTnFace/spf.epsilon_p^2	kg/(m²·s)	Backflow penalty parameter, convective contribution	Boundary 2
spf.inl1.dz	spf.dz	m	Channel thickness	Boundary 2
spf.inl1.c_here	96/spf.epsilon_p	1	Intermediate variable	Boundary 2
spf.inl1.upwind_ns	spf.backflowPenaltyConv*spf.uNormal	Pa	Upwind term	Boundary 2
spf.un_here	u2*nojac(spf.nrmesh)+w2*nojac(spf.nzmesh)	m/s	Intermediate variable	Boundary 2
spf.ut_herer	u2-spf.un_here*nojac(spf.nrmesh)	m/s	Intermediate variable, r-component	Boundary 2
spf.ut_herephi	-spf.un_here*nojac(spf.nphimesh)	m/s	Intermediate variable, phi-component	Boundary 2
spf.ut_herez	w2-spf.un_here*nojac(spf.nzm	m/s	Intermediate variable, z-	Boundary 2

Name	Expression	Unit	Description	Selection
	esh)		component	
spf.un_there	0	m/s	Intermediate variable	Boundary 2
spf.ut_therer	-spf.un_there*nojac(spf.nrmesh)	m/s	Intermediate variable, r-component	Boundary 2
spf.ut_therephi	-spf.un_there*nojac(spf.nphimesh)	m/s	Intermediate variable, phi-component	Boundary 2
spf.ut_therez	-spf.un_there*nojac(spf.nzmesh)	m/s	Intermediate variable, z-component	Boundary 2
spf.ct_here	24*nojac(down((spf.mu+spf.muT)/spf.epsilon_p))*spf.meshVol/spf.meshVolInt	Pa·s/m	Intermediate variable	Boundary 2
spf.inl1.volumeFlowRate	spf.inl1.intop(2*(u2*spf.nrmesh+w2*spf.nzmesh)*pi*r)	m^3/s	Outward volume flow rate across feature selection	Global
spf.inl1.massFlowRate	spf.inl1.intop(2*spf.rho*(u2*spf.nrmesh+w2*spf.nzmesh)*pi*r)	kg/s	Outward mass flow rate across feature selection	Global
spf.inl1.pAverage	spf.inl1.intop(2*p*pi*r)/max(spf.inl1.intop(2*pi*r),1000*eps)	Pa	Pressure average over feature selection	Global

Weak Expressions

Weak expression	Integration order	Integration frame	Selection
-2*spf.f0*(test(u2)*spf.nrmesh+test(w2)*spf.nzmesh)*pi*r	2	Spatial	Boundary 2
2*(spf.KStressn_avr*test(spf.ut_herer)+spf.KStressn_avphi*test(spf.ut_herephi)+spf.KStressn_avz*test(spf.ut_herez)+spf.KStressTestn_avr*spf.ujumpr+spf.KStressTestn_avphi*spf.ujumpphi+spf.KStressTestn_avz*spf.ujumpz-spf.sigma_dg_ns*spf.ujumpr*test(spf.ut_herer)-spf.sigma_dg_ns*spf.ujumpphi*test(spf.ut_herephi)-spf.sigma_dg_ns*spf.ujumpz*test(spf.ut_herez))*pi*r	2	Spatial	Boundary 2

2.9.8 Outlet 1

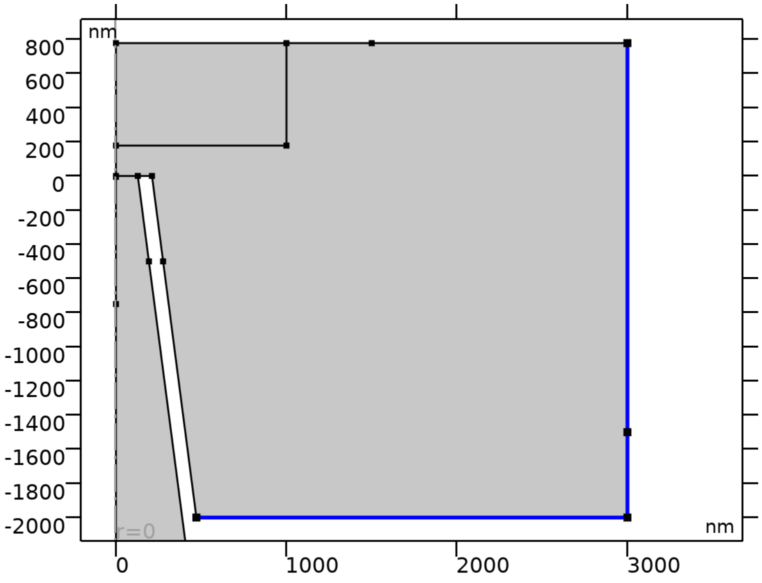

Outlet 1

SELECTION

Geometric entity level	Boundary
Selection	Geometry geom1: Dimension 1: Boundaries 15, 19–20

EQUATIONS

$$[-p\mathbf{I} + \mathbf{K}]\mathbf{n} = -\hat{p}_0\mathbf{n}$$

$$\hat{p}_0 \leq p_0 \,,$$

Boundary Condition

SETTINGS

Description	Value
Boundary condition	Pressure

Pressure Conditions

SETTINGS

Description	Value	Unit
Pressure	Static	
Pressure	0	Pa
Normal flow	Off	
Suppress backflow	On	

Constraint Settings

SETTINGS

Description	Value
Apply reaction terms on	All physics (symmetric)
Use weak constraints	Off
Constraint method	Elemental

Variables

Name	Expression	Unit	Description	Selection
spf.meshVol	meshvol_spatial	m		Boundaries 15, 19–20
spf.meshVolInt	down(meshvol_spatial)	m²	Volume of interior mesh element	Boundaries 15, 19–20
spf.rhoFace	down(spf.rho)	kg/m³	Density face value	Boundaries 15, 19–20
spf.umxTnFace	spf.upwind_helpr*spf.nrmesh+spf.upwind_helpphi*spf.nphimesh+spf.upwind_helpz*spf.nzmesh	m/s	Relative velocity on face	Boundaries 15, 19–20
spf.p0	0	Pa	Pressure	Boundaries 15, 19–20
spf.f0	spf.p0+spf.uNormal*(spf.backflowPenaltyDiff-spf.backflowPenaltyConv)*(spf.uNormal<0)	N/m²	Normal stress	Boundaries 15, 19–20
spf.uNormal	u2*nojac(spf.nrmesh)+w2*nojac(spf.nzmesh)	m/s	Normal velocity	Boundaries 15, 19–20
spf.backflowPenaltyDiff	spf.out1.c_here*min((down(spf.mu)+spf.muT)*spf.meshVol/spf.meshVolInt,down(spf.rho)*abs(spf.uNormal)/down(spf.epsilon_p))	Pa·s/m	Backflow penalty parameter, diffusive contribution	Boundaries 15, 19–20
spf.backflowPenaltyConv	spf.rhoFace*spf.umxTnFace/spf.epsilon_p^2	kg/(m²·s)	Backflow penalty parameter, convective contribution	Boundaries 15, 19–20
spf.out1.Uav	0	m/s	Average velocity	Global
spf.out1.p0avfdf	0	Pa	Average pressure	Global
spf.out1.dz	spf.dz	m	Channel thickness	Boundaries 15, 19–20
spf.out1.Mflow	spf.out1.massFlowRate	kg/s	Mass flow	Global

Name	Expression	Unit	Description	Selection
spf.out1.c_here	96/spf.epsilon_p	1	Intermediate variable	Boundaries 15, 19–20
spf.out1.upwind_ns	spf.backflowPenaltyConv*spf.uNormal	Pa	Upwind term	Boundaries 15, 19–20
spf.out1.volumeFlowRate	spf.out1.intop(2*(u2*spf.nrmesh+w2*spf.nzmesh)*pi*r)	m^3/s	Outward volume flow rate across feature selection	Global
spf.out1.massFlowRate	spf.out1.intop(2*spf.rho*(u2*spf.nrmesh+w2*spf.nzmesh)*pi*r)	kg/s	Outward mass flow rate across feature selection	Global
spf.out1.pAverage	spf.out1.intop(2*p*pi*r)/max(spf.out1.intop(2*pi*r),1000*eps)	Pa	Pressure average over feature selection	Global

Weak Expressions

Weak expression	Integration order	Integration frame	Selection
-2*spf.f0*(test(u2)*spf.nrmesh+test(w2)*spf.nzmesh)*pi*r	2	Spatial	Boundaries 15, 19–20

2.10 MULTIPHYSICS

2.10.1 Space Charge Density Coupling 1

USED PRODUCTS

COMSOL Multiphysics
Chemical Reaction Engineering Module

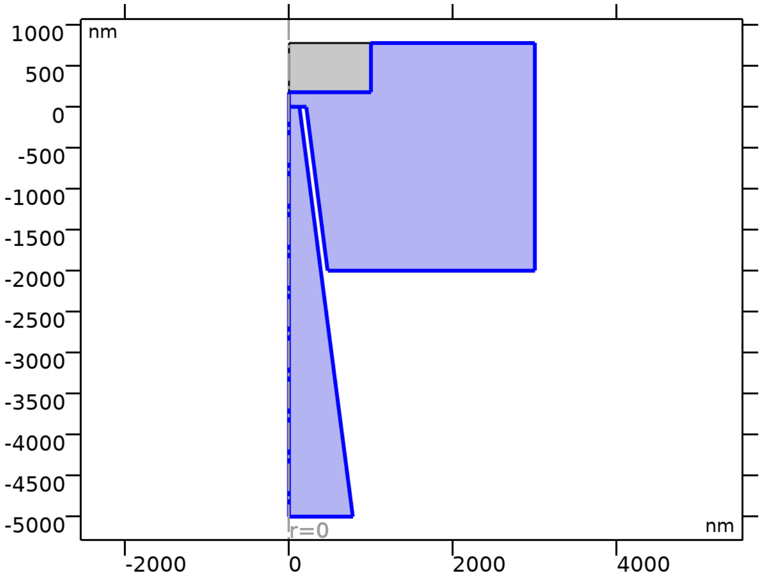

Space Charge Density Coupling 1

SELECTION

Geometric entity level	Domain
Selection	Geometry geom1: Dimension 2: Domains 1–2

EQUATIONS

$$\nabla \cdot \mathbf{D} = \rho_v, \quad \rho_v = F \sum_i z_i c_i$$

Coupled Interfaces

SETTINGS

Description	Value
Source	Transport of Diluted Species (tds)
Destination	Electrostatics (es)

Variables

Name	Expression	Unit	Description	Selection	Details
scdc1.sumzc	tds.z_K*K+tds.z_Cl*Cl	mol/m³	Help variable	Domains 1–2	+ operation
scdc1.rhoq	F_const*scdc1.sumzc	C/m³	Space charge density	Domains 1–2	
scdc1.sumztfluxr	tds.z_K*tds.tflux_Kr+tds.z_Cl*tds.tflux_Clr	mol/(m²·s)	Help variable, r-component	Domains 1–2	+ operation
scdc1.sumztfluxphi	tds.z_K*tds.tflux_Kphi+tds.z_Cl*tds.tflux_Clphi	mol/(m²·s)	Help variable, phi-component	Domains 1–2	+ operation

Name	Expression	Unit	Description	Selection	Details
scdc1.sumztfluxz	tds.z_K*tds.tflux_Kz+tds.z_Cl*tds.tflux_Clz	mol/(m²·s)	Help variable, z-component	Domains 1–2	+ operation
scdc1.Ilr	F_const*scdc1.sumztfluxr	A/m²	Electrolyte current density vector, r-component	Domains 1–2	
scdc1.Ilphi	F_const*scdc1.sumztfluxphi	A/m²	Electrolyte current density vector, phi-component	Domains 1–2	
scdc1.Ilz	F_const*scdc1.sumztfluxz	A/m²	Electrolyte current density vector, z-component	Domains 1–2	
scdc1.sumzntflux	tds.z_K*tds.ntflux_K+tds.z_Cl*tds.ntflux_Cl	mol/(m²·s)	Help variable	Boundaries 2, 6, 8, 10–20	+ operation
scdc1.nIl	F_const*scdc1.sumzntflux	A/m²	Normal electrolyte current density	Boundaries 2, 6, 8, 10–20	
es.rhoq	scdc1.rhoq	C/m³	Space charge density	Domains 1–2	+ operation

Weak Expressions

Weak expression	Integration order	Integration frame	Selection
-2*scdc1.rhoq*test(V)*es.d*pi*r	2	Spatial	Domains 1–2

2.10.2 Potential Coupling 1

USED PRODUCTS

COMSOL Multiphysics

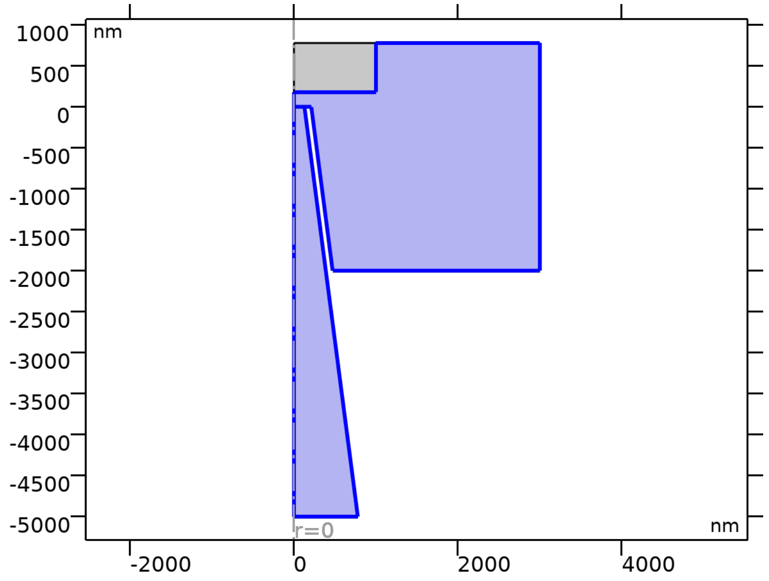

Potential Coupling 1

SELECTION

Geometric entity level	Domain
Selection	Geometry geom1: Dimension 2: All domains

Coupled Interfaces

SETTINGS

**Description**	**Value**
Source	Electrostatics (es)
Destination	Transport of Diluted Species (tds)

Variables

**Name**	**Expression**	**Unit**	**Description**	**Selection**
pc1.V	V	V	Electric potential	Domains 1–2

2.10.3 Fluid-Structure Interaction 1

USED PRODUCTS

COMSOL Multiphysics
MEMS Module

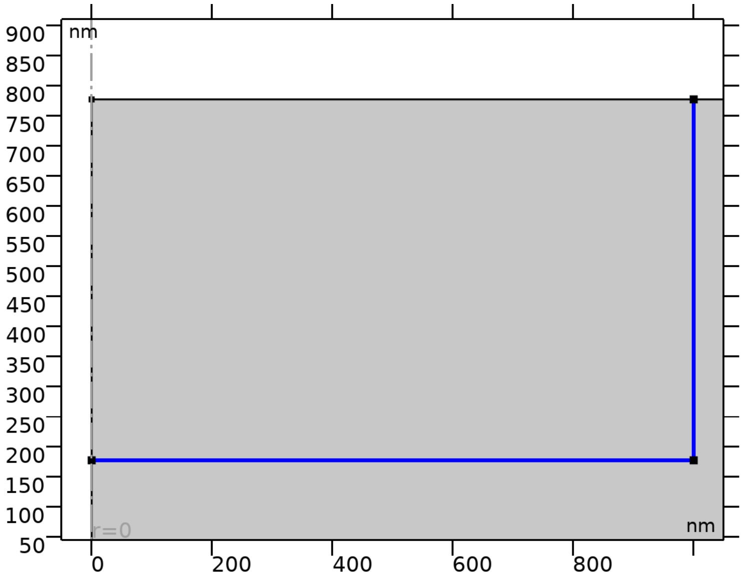

Fluid-Structure Interaction 1

SELECTION

Geometric entity level	Boundary
Selection	Geometry geom1: Dimension 1: Boundaries 8, 16

EQUATIONS

$$\mathbf{F}_A = [-p\mathbf{I} + \mathbf{K}] \cdot \mathbf{n}$$

Fixed Geometry

SETTINGS

Description	Value
Fixed geometry coupling type	Fully coupled

Coupled Interfaces

SETTINGS

Description	Value
Fluid	Laminar Flow (spf)
Structure	Solid Mechanics (solid)

Variables

Name	Expression	Unit	Description	Selection	Details
fsi1.u_fluidr	u2	m/s	Velocity field, r-component	Boundaries 8, 16	
fsi1.u_fluidphi	0	m/s	Velocity field, phi-component	Boundaries 8, 16	

Name	Expression	Unit	Description	Selection	Details
fsi1.u_fluidz	w2	m/s	Velocity field, z-component	Boundaries 8, 16	
solid.F_Ar	fsi1.F_Ar	N/m²	Load, r-component	Boundaries 8, 16	+ operation
solid.F_Aphi	fsi1.F_Aphi	N/m²	Load, phi-component	Boundaries 8, 16	+ operation
solid.F_Az	fsi1.F_Az	N/m²	Load, z-component	Boundaries 8, 16	+ operation
fsi1.F_Ar	-spf.T_stressr	N/m²	Load, r-component	Boundaries 8, 16	
fsi1.F_Aphi	-spf.T_stressphi	N/m²	Load, phi-component	Boundaries 8, 16	
fsi1.F_Az	-spf.T_stressz	N/m²	Load, z-component	Boundaries 8, 16	
fsi1.F_Anr	(fsi1.F_Ar*spf.nr+fsi1.F_Aphi*spf.nphi+fsi1.F_Az*spf.nz)*spf.nr	N/m²	Normal load, r-component	Boundaries 8, 16	
fsi1.F_Anphi	(fsi1.F_Ar*spf.nr+fsi1.F_Aphi*spf.nphi+fsi1.F_Az*spf.nz)*spf.nphi	N/m²	Normal load, phi-component	Boundaries 8, 16	
fsi1.F_Anz	(fsi1.F_Ar*spf.nr+fsi1.F_Aphi*spf.nphi+fsi1.F_Az*spf.nz)*spf.nz	N/m²	Normal load, z-component	Boundaries 8, 16	
fsi1.F_Atr	fsi1.F_Ar-fsi1.F_Anr	N/m²	Tangential load, r-component	Boundaries 8, 16	
fsi1.F_Atphi	fsi1.F_Aphi-fsi1.F_Anphi	N/m²	Tangential load, phi-component	Boundaries 8, 16	
fsi1.F_Atz	fsi1.F_Az-fsi1.F_Anz	N/m²	Tangential load, z-component	Boundaries 8, 16	
fsi1.F_A_Mag	sqrt(real(fsi1.F_Ar)^2+real(fsi1.F_Aphi)^2+real(fsi1.F_Az)^2)	N/m²	Load magnitude	Boundaries 8, 16	
fsi1.F_An_Mag	sqrt(real(fsi1.F_Anr)^2+real(fsi1.F_Anphi)^2+real(fsi1.F_Anz)^2)	N/m²	Normal load magnitude	Boundaries 8, 16	
fsi1.F_At_Mag	sqrt(real(fsi1.F_Atr)^2+real(fsi1.F_Atphi)^2+real(fsi1.F_Atz)^2)	N/m²	Tangential load magnitude	Boundaries 8, 16	
fsi1.u_solid1	u	m	Displacement, 1-component	Boundaries 8, 16	
fsi1.u_solid2	0	m	Displacement, 2-	Boundaries 8,	

Name	Expression	Unit	Description	Selection	Details
			component	16	
fsi1.u_solid3	w	m	Displacement, 3-component	Boundaries 8, 16	
fsi1.ASF	1	1	Area scaling for offset	Boundaries 8, 16	
fsi1.Tt_stress1	spf.T_stressr-(spf.T_stressr*spf.nr+spf.T_stressphi*spf.nphi+spf.T_stressz*spf.nz)*spf.nr	N/m²	Total stress tangential, 1-component	Boundaries 8, 16	
fsi1.Tt_stress2	spf.T_stressphi-(spf.T_stressr*spf.nr+spf.T_stressphi*spf.nphi+spf.T_stressz*spf.nz)*spf.nphi	N/m²	Total stress tangential, 2-component	Boundaries 8, 16	
fsi1.Tt_stress3	spf.T_stressz-(spf.T_stressr*spf.nr+spf.T_stressphi*spf.nphi+spf.T_stressz*spf.nz)*spf.nz	N/m²	Total stress tangential, 3-component	Boundaries 8, 16	

Weak Expressions

Weak expression	Integration order	Integration frame	Selection
2*(-spf.T_stressr*test(fsi1.u_solid1)-spf.T_stressphi*test(fsi1.u_solid2)-spf.T_stressz*test(fsi1.u_solid3))*fsi1.ASF*pi*r	4	Spatial	Boundaries 8, 16

2.11 MESH

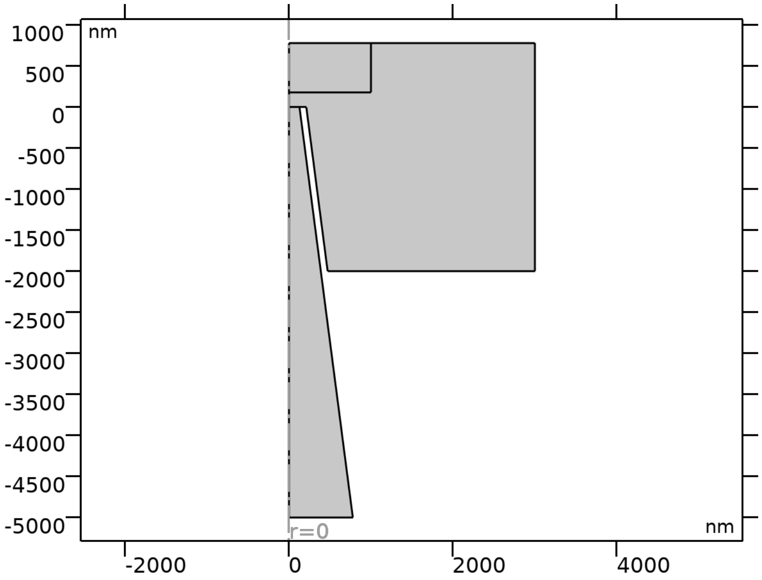

Mesh

MESH STATISTICS

Description	Value
Status	Empty mesh
Mesh vertices	0

2.11.1 Insignificant Size (size)

SETTINGS

Description	Value
Maximum element size	387
Minimum element size	1.73
Curvature factor	0.3
Maximum element growth rate	1.3

2.11.2 Focused Surface (size1)

SELECTION

Geometric entity level	Boundary
Selection	Geometry geom1: Dimension 1: Boundaries 10–11, 13, 16, 18

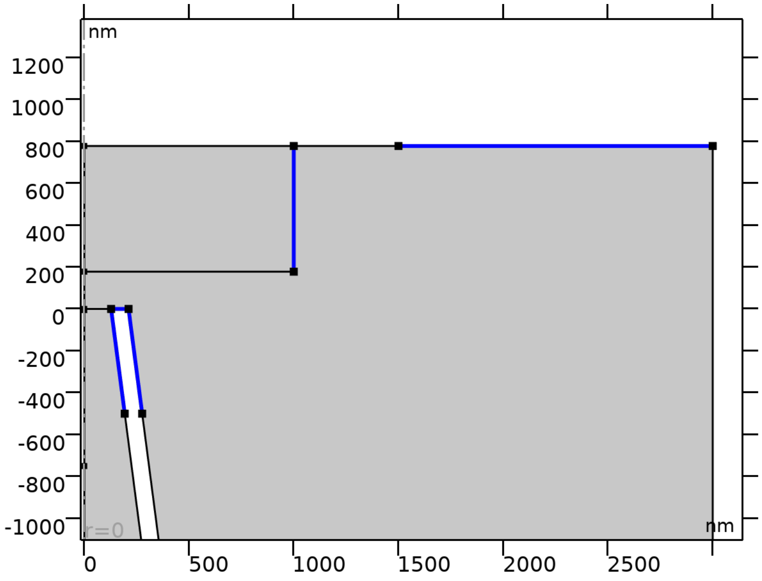

Focused Surface

SETTINGS

Description	Value
Minimum element size	0.02
Curvature factor	0.3
Curvature factor	Off
Resolution of narrow regions	Off
Custom element size	Custom

2.11.3 Focused Body (size2)

SELECTION

Geometric entity level	Domain
Selection	Geometry geom1: Dimension 2: Domain 3

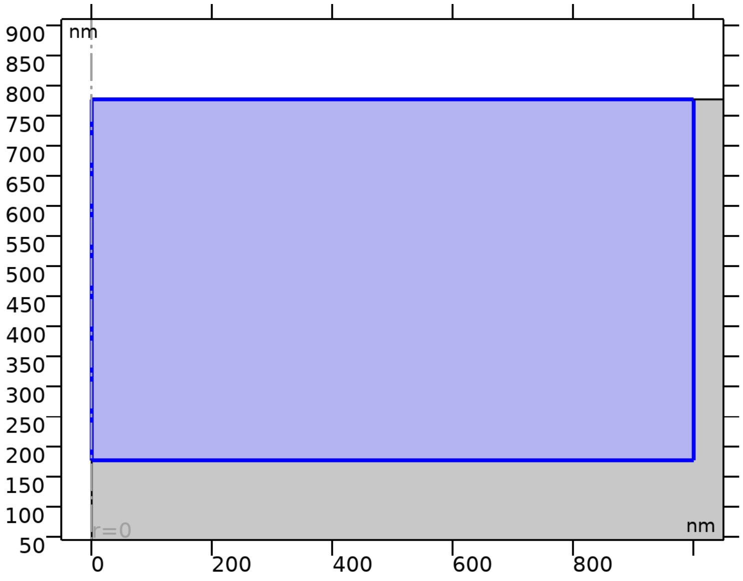

Focused Body

SETTINGS

Description	Value
Maximum element size	5
Minimum element size	1.68
Minimum element size	Off
Curvature factor	0.3
Curvature factor	Off
Resolution of narrow regions	Off
Maximum element growth rate	1.3
Maximum element growth rate	Off
Custom element size	Custom

2.11.4 Contact Point (size3)

SELECTION

Geometric entity level	Point
Selection	Geometry geom1: Dimension 0: No points

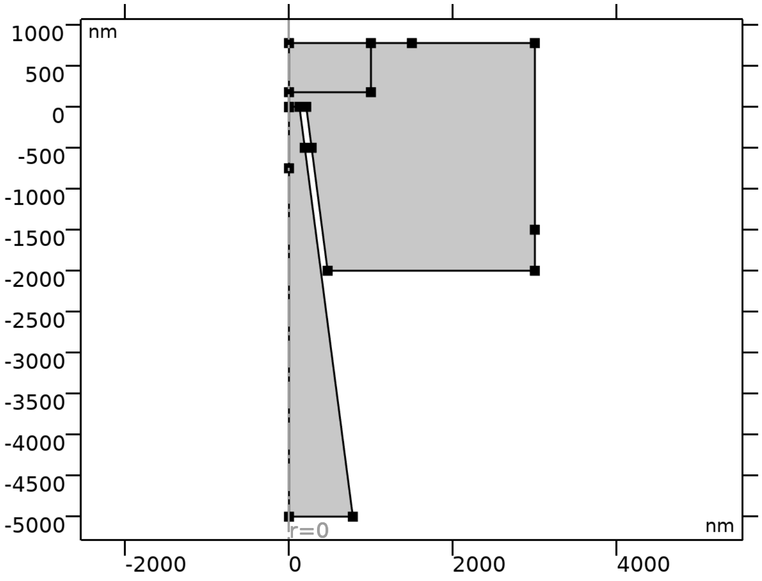

Contact Point

SETTINGS

Description	Value
Maximum element size	0.1
Minimum element size	0.01
Curvature factor	0.2
Curvature factor	Off
Resolution of narrow regions	Off
Maximum element growth rate	1.01
Predefined size	Extremely fine
Custom element size	Custom

2.11.5 Free Triangular Segmentation (ftri1)

SELECTION

Geometric entity level	Domain
Selection	Remaining

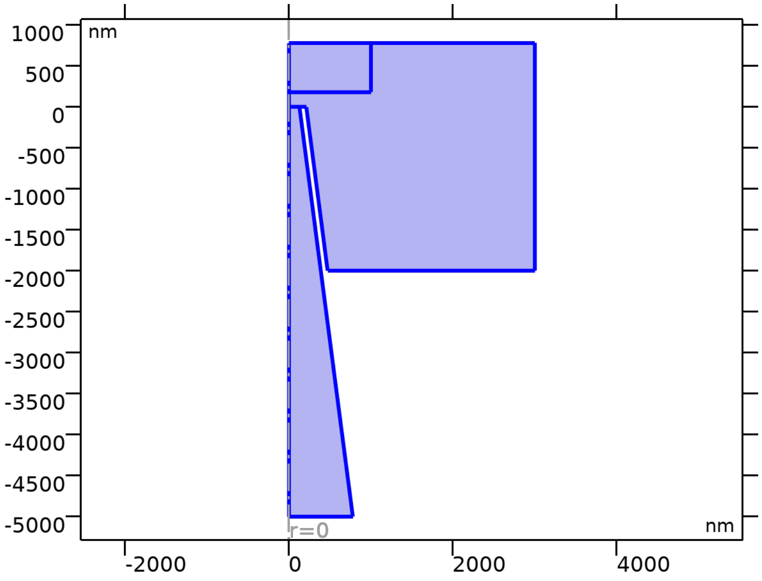

Free Triangular Segmentation

SETTINGS

Description	Value
Last build time	3
Built with	COMSOL 6.1.0.252 (glnxa64)|2025 - 02 - 01T12:59:34.647856

segps (size1)

SELECTION

Geometric entity level	Boundary
Selection	Geometry geom1: Dimension 1: Boundaries 3, 5

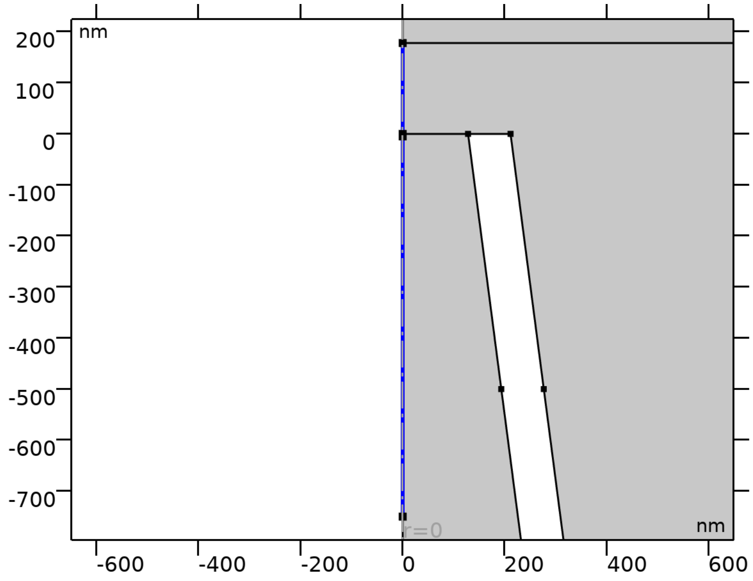

segps

SETTINGS

Description	Value
Maximum element size	2
Minimum element size	1.68
Minimum element size	Off
Curvature factor	0.3
Curvature factor	Off
Resolution of narrow regions	Off
Maximum element growth rate	1.01
Custom element size	Custom

Inner Pipette (size2)

SELECTION

Geometric entity level	Boundary
Selection	Geometry geom1: Dimension 1: Boundaries 1, 12

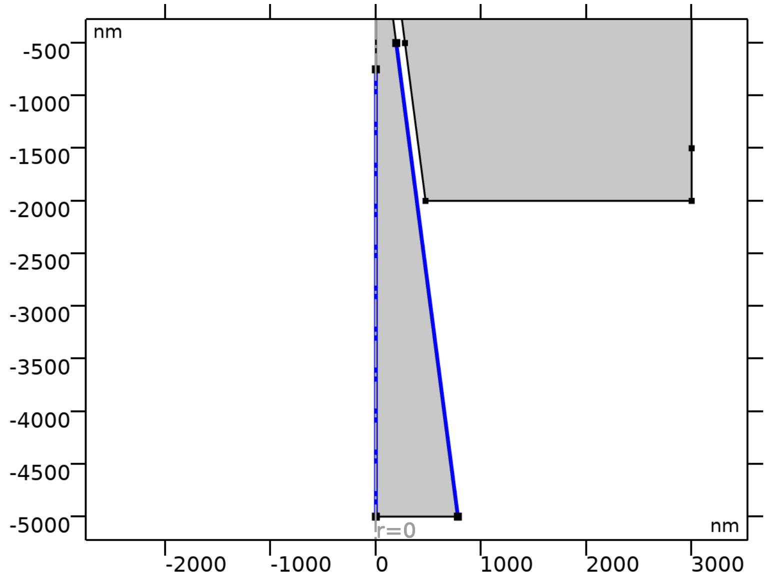

Inner Pipette

SETTINGS

Description	Value
Maximum element size	20
Minimum element size	1.68
Minimum element size	Off
Curvature factor	0.3
Curvature factor	Off
Resolution of narrow regions	Off
Maximum element growth rate	1.3
Maximum element growth rate	Off
Custom element size	Custom

3 study1

COMPUTATION INFORMATION

Computation time	20 h 14 min 16 s

3.1 PARAMETRIC SWEEP

Parameter name	Parameter value list	Parameter unit
d	5000, range(2500,-5,120)	nm

STUDY SETTINGS

Description	Value
Sweep type	All combinations
Parameter name	d
Unit	nm

PARAMETERS

Parameter name	Parameter value list	Parameter unit
d (dps)	5000, range(2500,-5,120)	nm

3.2 STATIONARY

STUDY SETTINGS

Description	Value
Include geometric nonlinearity	On

SETTINGS

Description	Value
Include geometric nonlinearity	On

PHYSICS AND VARIABLES SELECTION

Physics interface	Discretization
Transport of Diluted Species (tds)	physics
Electrostatics (es)	physics
Solid Mechanics (solid)	physics
Laminar Flow (spf)	physics

MESH SELECTION

Geometry	Mesh
Geometry 1 (geom1)	mesh1

3.3 SOLVER CONFIGURATIONS

3.3.1 Approach Curve Solver

Compile Equations: Stationary (st1)

STUDY AND STEP

Description	Value
Use study	study1
Use study step	Stationary

LOG

```
<---- Compile Equations: Stationary in study1/Approach Curve Solver (sol1) -----
Started at Feb 1, 2025, 12:59:29 PM.
Parameter d = 1.2000000000000002E-7 (m).
Geometry shape function: Linear serendipity
Number of vertex elements: 18
Number of boundary elements: 5123
Number of elements: 139097
Minimum element quality: 0.501
Time: 6 s.
Physical memory: 3.26 GB
Virtual memory: 15.24 GB
Ended at Feb 1, 2025, 12:59:35 PM.
----- Compile Equations: Stationary in study1/Approach Curve Solver (sol1) ---->
```

Dependent Variables 1 (v1)

GENERAL

Description	Value
Defined by study step	Stationary

LOG

```
<---- Dependent Variables 1 in study1/Approach Curve Solver (sol1) -------------
Started at Feb 1, 2025, 12:59:35 PM.
Solution time: 0 s.
Physical memory: 3.26 GB
Virtual memory: 15.24 GB
Ended at Feb 1, 2025, 12:59:35 PM.
----- Dependent Variables 1 in study1/Approach Curve Solver (sol1) ------------>
```

Concentration (comp1.Cl) (comp1_Cl)

GENERAL

Description	Value
Field components	comp1.Cl
Internal variables	{comp1.uflux.Cl, comp1.dflux.Cl}

Concentration (comp1.K) (comp1_K)

GENERAL

Description	Value
Field components	comp1.K
Internal variables	{comp1.uflux.K, comp1.dflux.K}

Pressure (comp1.p) (comp1_p)

GENERAL

Description	Value
Field components	comp1.p

Auxiliary pressure (comp1.solid.lemm1.pw) (comp1_solid_lemm1_pw)

GENERAL

Description	Value
Field components	comp1.solid.lemm1.pw

Spatial mesh displacement (comp1.spatial.disp) (comp1_spatial_disp)

GENERAL

Description	Value
Field components	{comp1.spatial.u, comp1.spatial.w}

SCALING

Description	Value
Method	Manual
Scale	1.1746E-9

Displacement field (comp1.u) (comp1_u)

GENERAL

Description	Value
Field components	{comp1.u, comp1.w}

Velocity field (spatial frame) (comp1.u2) (comp1_u2)

GENERAL

Description	Value
Field components	{comp1.u2, comp1.w2}
Internal variables	comp1.spf.isFluidHasBeenSolved

Electric potential (comp1.V) (comp1_V)

GENERAL

Description	Value
Field components	comp1.V

Stationary Solver 1 (s1)

GENERAL

Description	Value
Defined by study step	Stationary

RESULTS WHILE SOLVING

Description	Value
Probes	None

LOG

```
<---- Stationary Solver 1 in study1/Approach Curve Solver (sol1) ---------------
Started at Feb 1, 2025, 12:59:35 PM.
Segregated solver
Segregated Step 11 does not have any components solved for.
Number of degrees of freedom solved for: 695202 (plus 9669 internal DOFs).
Segregated solver iteration 1.
Merged variables
Nonsymmetric matrix found.
Scales for dependent variables:
Concentration (comp1.Cl): 1.4e+02
Concentration (comp1.K): 1.4e+02
Electric potential (comp1.V): 2e+02
Orthonormal null-space function used.
Iter      SolEst     Damping    Stepsize #Res #Jac #Sol   LinErr   LinRes
   1        0.81   0.3500000        0.81    2    1    1  1.7e-11  2.4e-15
   2        0.79   0.3500000        0.79    3    2    2  7.5e-11  2.4e-15
   3        0.77   0.3500000        0.77    4    3    3  8.6e-11  2.4e-15
Velocity u2, Pressure p
Nonsymmetric matrix found.
Scales for dependent variables:
Pressure (comp1.p): 1
Velocity field (spatial frame) (comp1.u2): 0.00014
Orthonormal null-space function used.
Iter      SolEst     Damping    Stepsize #Res #Jac #Sol   LinErr   LinRes
   1         1.1   0.5000000         1.1    2    1    1  1.1e-15    2e-15
Displacement field
Nonsymmetric matrix found.
Scales for dependent variables:
Auxiliary pressure (comp1.solid.lemm1.pw): 1
Displacement field (comp1.u): 2.8e-18
Orthonormal null-space function used.
Iter      SolEst     Damping    Stepsize #Res #Jac #Sol   LinErr   LinRes
   1         1.9   0.5000000         1.9    2    1    1  6.2e-14  3.1e-13
Spatial mesh displacement
Symmetric matrices found.
Scales for dependent variables:
Spatial mesh displacement (comp1.spatial.disp): 1.2e-09
Orthonormal null-space function used.
Iter      SolEst     Damping    Stepsize #Res #Jac #Sol   LinErr   LinRes
   1       5e-05   0.5000000       5e-05    2    1    1  1.1e-15  1.1e-15
Solution error estimates for segregated groups
1.3, 0.57, 0.93, 2.5e-05
Residual error estimates for segregated groups
7.1e+03, 7.4e+04, 2.9e+04, 2.3e+02
Segregated solver iteration 2.
Merged variables
Scales for dependent variables:
Concentration (comp1.Cl): 1.4e+02
Concentration (comp1.K): 1.4e+02
Electric potential (comp1.V): 0.067
Iter      SolEst     Damping    Stepsize #Res #Jac #Sol   LinErr   LinRes
   1        0.75   0.3500000        0.75    6    4    4  9.1e-14  2.3e-15
   2        0.73   0.3500000        0.73    7    5    5    1e-13  2.5e-15
   3        0.72   0.3500000        0.72    8    6    6  4.5e-14  2.3e-15
Velocity u2, Pressure p
Scales for dependent variables:
```

```
Pressure (comp1.p): 5e+02
Velocity field (spatial frame) (comp1.u2): 0.00061
Iter      SolEst     Damping    Stepsize #Res #Jac #Sol   LinErr   LinRes
   1        0.38   0.5000000        0.38    4    2    2  4.1e-15  7.8e-15
Displacement field
Scales for dependent variables:
Auxiliary pressure (comp1.solid.lemm1.pw): 14
Displacement field (comp1.u): 5.9e-15
Iter      SolEst     Damping    Stepsize #Res #Jac #Sol   LinErr   LinRes
   1        0.93   0.5000000        0.93    4    2    2  4.1e-14  2.8e-13
Spatial mesh displacement
Iter      SolEst     Damping    Stepsize #Res #Jac #Sol   LinErr   LinRes
   1     7.6e-05   0.5000000     7.6e-05    4    2    2  1.2e-15  1.2e-15
Solution error estimates for segregated groups
1.2, 0.19, 0.46, 3.8e-05
Residual error estimates for segregated groups
5.6e+02, 1e+04, 2.9e+04, 2.3e+03
Segregated solver iteration 3.
Merged variables
Iter      SolEst     Damping    Stepsize #Res #Jac #Sol   LinErr   LinRes
   1         0.7   0.3500000         0.7   10    7    7  1.8e-13  2.4e-15
   2        0.68   0.3500000        0.68   11    8    8  1.2e-13  2.4e-15
   3        0.66   0.3500000        0.66   12    9    9    7e-14  2.3e-15
Velocity u2, Pressure p
Iter      SolEst     Damping    Stepsize #Res #Jac #Sol   LinErr   LinRes
   1        0.16   0.5000000        0.16    6    3    3  2.2e-15  8.1e-15
Displacement field
Iter      SolEst     Damping    Stepsize #Res #Jac #Sol   LinErr   LinRes
   1         0.5   0.5000000         0.5    6    3    3  4.4e-14  2.7e-13
Spatial mesh displacement
Iter      SolEst     Damping    Stepsize #Res #Jac #Sol   LinErr   LinRes
   1     7.5e-05   0.5000000     7.5e-05    6    3    3  8.8e-16  1.2e-15
Solution error estimates for segregated groups
1.1, 0.081, 0.25, 3.8e-05
Residual error estimates for segregated groups
1.6e+02, 6.9e+03, 2.2e+04, 4.4e+03
Segregated solver iteration 4.
Merged variables
Iter      SolEst     Damping    Stepsize #Res #Jac #Sol   LinErr   LinRes
   1        0.64   0.3500000        0.64   14   10   10  1.3e-13  2.3e-15
   2        0.62   0.3500000        0.62   15   11   11  3.6e-14  2.3e-15
   3        0.44   0.3500000        0.44   16   12   12  1.6e-13  2.3e-15
Velocity u2, Pressure p
Iter      SolEst     Damping    Stepsize #Res #Jac #Sol   LinErr   LinRes
   1       0.076   0.5000000       0.076    8    4    4  4.1e-15  7.9e-15
Displacement field
Iter      SolEst     Damping    Stepsize #Res #Jac #Sol   LinErr   LinRes
   1        0.28   0.5000000        0.28    8    4    4  4.7e-14  2.8e-13
Spatial mesh displacement
Iter      SolEst     Damping    Stepsize #Res #Jac #Sol   LinErr   LinRes
   1     6.3e-05   0.5000000     6.3e-05    8    4    4  9.4e-16  1.2e-15
Solution error estimates for segregated groups
0.76, 0.038, 0.14, 3.1e-05
Residual error estimates for segregated groups
67, 5.6e+03, 1.4e+04, 4.4e+03
Segregated solver iteration 5.
```

```
Merged variables
Iter      SolEst     Damping    Stepsize #Res #Jac #Sol   LinErr   LinRes
   1         0.3   0.3500000         0.3   18   13   13  1.3e-13  2.4e-15
   2         0.2   0.3500000         0.2   19   14   14  8.2e-14  2.5e-15
   3        0.13   0.3500000        0.13   20   15   15  6.3e-14  2.3e-15
Velocity u2, Pressure p
Iter      SolEst     Damping    Stepsize #Res #Jac #Sol   LinErr   LinRes
   1       0.037   0.5000000       0.037   10    5    5  3.2e-15    8e-15
Displacement field
Iter      SolEst     Damping    Stepsize #Res #Jac #Sol   LinErr   LinRes
   1        0.16   0.5000000        0.16   10    5    5  4.6e-14  2.8e-13
Spatial mesh displacement
Iter      SolEst     Damping    Stepsize #Res #Jac #Sol   LinErr   LinRes
   1     4.7e-05   0.5000000     4.7e-05   10    5    5  9.7e-16  1.1e-15
Solution error estimates for segregated groups
0.23, 0.018, 0.081, 2.4e-05
Residual error estimates for segregated groups
28, 4.7e+03, 9e+03, 3.3e+03
Segregated solver iteration 6.
Merged variables
Iter      SolEst     Damping    Stepsize #Res #Jac #Sol   LinErr   LinRes
   1       0.086   0.3500000       0.086   22   16   16  8.4e-14  2.3e-15
   2       0.056   0.3500000       0.056   23   17   17  7.7e-14  2.3e-15
   3       0.037   0.3500000       0.037   24   18   18  7.6e-14  2.4e-15
Velocity u2, Pressure p
Iter      SolEst     Damping    Stepsize #Res #Jac #Sol   LinErr   LinRes
   1       0.018   0.5000000       0.018   12    6    6  3.3e-15  7.9e-15
Displacement field
Iter      SolEst     Damping    Stepsize #Res #Jac #Sol   LinErr   LinRes
   1       0.093   0.5000000       0.093   12    6    6  4.2e-14  2.8e-13
Spatial mesh displacement
Iter      SolEst     Damping    Stepsize #Res #Jac #Sol   LinErr   LinRes
   1     3.3e-05   0.5000000     3.3e-05   12    6    6  1.1e-15  1.2e-15
Solution error estimates for segregated groups
0.063, 0.009, 0.046, 1.6e-05
Residual error estimates for segregated groups
10, 3.8e+03, 5.4e+03, 2.2e+03
Segregated solver iteration 7.
Merged variables
Iter      SolEst     Damping    Stepsize #Res #Jac #Sol   LinErr   LinRes
   1       0.024   0.3500000       0.024   26   19   19  1.1e-13  2.3e-15
   2       0.016   0.3500000       0.016   27   20   20  8.3e-14  2.3e-15
   3        0.01   0.3500000        0.01   28   21   21  7.7e-14  2.4e-15
Velocity u2, Pressure p
Iter      SolEst     Damping    Stepsize #Res #Jac #Sol   LinErr   LinRes
   1       0.009   0.5000000       0.009   14    7    7  2.3e-15  7.9e-15
Displacement field
Iter      SolEst     Damping    Stepsize #Res #Jac #Sol   LinErr   LinRes
   1       0.052   0.5000000       0.052   14    7    7  4.1e-14  2.7e-13
Spatial mesh displacement
Iter      SolEst     Damping    Stepsize #Res #Jac #Sol   LinErr   LinRes
   1     2.2e-05   0.5000000     2.2e-05   14    7    7  1.3e-15  1.2e-15
Solution error estimates for segregated groups
0.017, 0.0045, 0.026, 1.1e-05
Residual error estimates for segregated groups
3.6, 2.9e+03, 3.1e+03, 1.3e+03
```

```
Segregated solver iteration 8.
Merged variables
Iter      SolEst     Damping    Stepsize #Res #Jac #Sol   LinErr   LinRes
   1      0.0066   0.3500000      0.0066   30   22   22  1.3e-13  2.4e-15
   2      0.0043   0.3500000      0.0043   31   23   23  1.2e-13  2.3e-15
   3      0.0028   0.3500000      0.0028   32   24   24  5.9e-14  2.4e-15
Velocity u2, Pressure p
Iter      SolEst     Damping    Stepsize #Res #Jac #Sol   LinErr   LinRes
   1      0.0045   0.5000000      0.0045   16    8    8  2.9e-15  7.9e-15
Displacement field
Iter      SolEst     Damping    Stepsize #Res #Jac #Sol   LinErr   LinRes
   1        0.03   0.5000000        0.03   16    8    8    4e-14  2.9e-13
Spatial mesh displacement
Iter      SolEst     Damping    Stepsize #Res #Jac #Sol   LinErr   LinRes
   1     1.4e-05   0.5000000     1.4e-05   16    8    8    1e-15  1.2e-15
Solution error estimates for segregated groups
0.0048, 0.0022, 0.015, 7.1e-06
Residual error estimates for segregated groups
1.2, 2.1e+03, 1.8e+03, 7.5e+02
Segregated solver iteration 9.
Merged variables
Iter      SolEst     Damping    Stepsize #Res #Jac #Sol   LinErr   LinRes
   1      0.0018   0.3500000      0.0018   34   25   25  7.4e-14  2.4e-15
   2      0.0012   0.3500000      0.0012   35   26   26  7.5e-14  2.4e-15
   3     0.00076   0.3500000     0.00076   36   27   27  7.5e-14  2.5e-15
Velocity u2, Pressure p
Iter      SolEst     Damping    Stepsize #Res #Jac #Sol   LinErr   LinRes
   1      0.0022   0.5000000      0.0022   18    9    9  2.4e-15  7.7e-15
Displacement field
Iter      SolEst     Damping    Stepsize #Res #Jac #Sol   LinErr   LinRes
   1       0.016   0.5000000       0.016   18    9    9    4e-14  2.8e-13
Spatial mesh displacement
Iter      SolEst     Damping    Stepsize #Res #Jac #Sol   LinErr   LinRes
   1     8.8e-06   0.5000000     8.8e-06   18    9    9  9.7e-16  1.1e-15
Solution error estimates for segregated groups
0.0013, 0.0011, 0.0082, 4.4e-06
Residual error estimates for segregated groups
0.39, 1.4e+03, 1e+03, 4.1e+02
Segregated solver iteration 10.
Merged variables
Iter      SolEst     Damping    Stepsize #Res #Jac #Sol   LinErr   LinRes
   1      0.0005   0.3500000      0.0005   38   28   28  8.3e-14  2.4e-15
   2     0.00032   0.3500000     0.00032   39   29   29  1.1e-13  2.4e-15
   3     0.00021   0.3500000     0.00021   40   30   30    7e-14  2.4e-15
Velocity u2, Pressure p
Iter      SolEst     Damping    Stepsize #Res #Jac #Sol   LinErr   LinRes
   1      0.0011   0.5000000      0.0011   20   10   10  3.9e-15  7.8e-15
Displacement field
Iter      SolEst     Damping    Stepsize #Res #Jac #Sol   LinErr   LinRes
   1      0.0091   0.5000000      0.0091   20   10   10  3.7e-14  2.8e-13
Spatial mesh displacement
Iter      SolEst     Damping    Stepsize #Res #Jac #Sol   LinErr   LinRes
   1     5.4e-06   0.5000000     5.4e-06   20   10   10  1.1e-15  1.1e-15
Solution error estimates for segregated groups
0.00036, 0.00056, 0.0045, 2.7e-06
Residual error estimates for segregated groups
```

```
0.12, 9.6e+02, 5.6e+02, 2.2e+02
Segregated solver iteration 11.
Merged variables
Iter      SolEst     Damping    Stepsize #Res #Jac #Sol   LinErr   LinRes
   1     0.00014   0.3500000     0.00014   42   31   31  6.5e-14  2.3e-15
   2     8.9e-05   0.3500000     8.9e-05   43   32   32  8.3e-14  2.4e-15
   3     5.8e-05   0.3500000     5.8e-05   44   33   33  8.3e-14  2.4e-15
Velocity u2, Pressure p
Iter      SolEst     Damping    Stepsize #Res #Jac #Sol   LinErr   LinRes
   1     0.00056   0.5000000     0.00056   22   11   11  4.6e-15  7.9e-15
Displacement field
Iter      SolEst     Damping    Stepsize #Res #Jac #Sol   LinErr   LinRes
   1       0.005   0.5000000       0.005   22   11   11  3.5e-14  2.8e-13
Spatial mesh displacement
Iter      SolEst     Damping    Stepsize #Res #Jac #Sol   LinErr   LinRes
   1     3.2e-06   0.5000000     3.2e-06   22   11   11    1e-15  1.1e-15
Solution error estimates for segregated groups
9.9e-05, 0.00028, 0.0025, 1.6e-06
Residual error estimates for segregated groups
0.038, 6.2e+02, 3.1e+02, 1.1e+02
Segregated solver iteration 12.
Merged variables
Iter      SolEst     Damping    Stepsize #Res #Jac #Sol   LinErr   LinRes
   1     3.7e-05   0.3500000     3.7e-05   46   34   34    1e-13  2.4e-15
   2     2.4e-05   0.3500000     2.4e-05   47   35   35    1e-13  2.4e-15
   3     1.6e-05   0.3500000     1.6e-05   48   36   36  7.8e-14  2.3e-15
Velocity u2, Pressure p
Iter      SolEst     Damping    Stepsize #Res #Jac #Sol   LinErr   LinRes
   1     0.00028   0.5000000     0.00028   24   12   12    3e-15  7.9e-15
Displacement field
Iter      SolEst     Damping    Stepsize #Res #Jac #Sol   LinErr   LinRes
   1      0.0027   0.5000000      0.0027   24   12   12  6.5e-14  2.7e-13
Spatial mesh displacement
Iter      SolEst     Damping    Stepsize #Res #Jac #Sol   LinErr   LinRes
   1     1.9e-06   0.5000000     1.9e-06   24   12   12  8.6e-16  1.1e-15
Solution error estimates for segregated groups
2.7e-05, 0.00014, 0.0014, 9.6e-07
Residual error estimates for segregated groups
0.012, 4e+02, 1.7e+02, 59
Segregated solver iteration 13.
Merged variables
Iter      SolEst     Damping    Stepsize #Res #Jac #Sol   LinErr   LinRes
   1       1e-05   0.3500000       1e-05   50   37   37  8.7e-14  2.4e-15
   2     6.7e-06   0.3500000     6.7e-06   51   38   38  1.3e-13  2.4e-15
   3     4.4e-06   0.3500000     4.4e-06   52   39   39  6.4e-14  2.3e-15
Velocity u2, Pressure p
Iter      SolEst     Damping    Stepsize #Res #Jac #Sol   LinErr   LinRes
   1     0.00014   0.5000000     0.00014   26   13   13  4.5e-15    8e-15
Displacement field
Iter      SolEst     Damping    Stepsize #Res #Jac #Sol   LinErr   LinRes
   1      0.0015   0.5000000      0.0015   26   13   13  3.8e-14  2.7e-13
Spatial mesh displacement
Iter      SolEst     Damping    Stepsize #Res #Jac #Sol   LinErr   LinRes
   1     1.1e-06   0.5000000     1.1e-06   26   13   13    1e-15  1.1e-15
Solution error estimates for segregated groups
7.5e-06, 6.9e-05, 0.00074, 5.6e-07
```

```
Residual error estimates for segregated groups
0.0036, 2.5e+02, 91, 30
Solution time: 129 s. (2 minutes, 9 seconds)
Physical memory: 4.93 GB
Virtual memory: 25.13 GB
Ended at Feb 1, 2025, 1:01:44 PM.
----- Stationary Solver 1 in study1/Approach Curve Solver (sol1) -------------->
```

Advanced (aDef)

ASSEMBLY SETTINGS

Description	Value
Reuse sparsity pattern	On

Segregated 1 (se1)

GENERAL

Description	Value
Maximum number of iterations	300

Merged variables (ss1)

GENERAL

Description	Value
Variables	{Electric potential (comp1.V), Concentration (comp1.K), Concentration (comp1.Cl)}
Linear solver	Direct (merged)

METHOD AND TERMINATION

Description	Value
Damping factor	0.35
Number of iterations	3

Velocity u2, Pressure p (ss2)

GENERAL

Description	Value
Variables	{Velocity field (spatial frame) (comp1.u2), Pressure (comp1.p)}
Linear solver	Direct (merged)

METHOD AND TERMINATION

Description	Value
Damping factor	0.5

Displacement field (ss3)

GENERAL

Description	Value

Description	Value
Variables	{Displacement field (comp1.u), Auxiliary pressure (comp1.solid.lemm1.pw)}
Linear solver	Direct (merged)

METHOD AND TERMINATION

Description	Value
Damping factor	0.5

Spatial mesh displacement (ss4)

GENERAL

Description	Value
Variables	Spatial mesh displacement (comp1.spatial.disp)
Linear solver	Direct (merged)

METHOD AND TERMINATION

Description	Value
Damping factor	0.5

Segregated Step 11 (material11)

GENERAL

Description	Value
Linear solver	Direct (merged)

Direct (merged) (d1)

GENERAL

Description	Value
Memory allocation factor	8

3.3.2 Parametric Solutions 1

d=5000 (su1)

GENERAL

Description	Value
Solution	d=5000

LOG

```
<---- Compile Equations: Stationary in study1/Approach Curve Solver (sol1) -----
Started at Jan 31, 2025, 4:47:31 PM.
Running on AMD EPYC 7402P 24-Core Processor.
Using 1 socket with 24 cores in total on chembakercomsol.
Available memory: 127.88 GB.
Parameter d = 5.0E-6 (m).
Geometry shape function: Linear serendipity
Number of vertex elements: 18
Number of boundary elements: 7662
Number of elements: 177960
Minimum element quality: 0.4769
Time: 15 s.
Physical memory: 1.38 GB
Virtual memory: 14.67 GB
Ended at Jan 31, 2025, 4:47:46 PM.
----- Compile Equations: Stationary in study1/Approach Curve Solver (sol1) ---->

<---- Dependent Variables 1 in study1/Approach Curve Solver (sol1) -------------
Started at Jan 31, 2025, 4:47:46 PM.
Solution time: 0 s.
Physical memory: 1.51 GB
Virtual memory: 14.69 GB
Ended at Jan 31, 2025, 4:47:46 PM.
----- Dependent Variables 1 in study1/Approach Curve Solver (sol1) ------------>

<---- Stationary Solver 1 in study1/Approach Curve Solver (sol1) ---------------
Started at Jan 31, 2025, 4:47:46 PM.
Segregated solver
Segregated Step 11 does not have any components solved for.
Number of degrees of freedom solved for: 920114 (plus 14747 internal DOFs).
Segregated solver iteration 1.
Merged variables
Nonsymmetric matrix found.
Scales for dependent variables:
Concentration (comp1.Cl): 1.4e+02
Concentration (comp1.K): 1.4e+02
Electric potential (comp1.V): 1.8e+02
Orthonormal null-space function used.
Iter      SolEst     Damping    Stepsize #Res #Jac #Sol   LinErr   LinRes
   1        0.84   0.3500000        0.84    2    1    1  2.6e-11  3.1e-15
   2        0.83   0.3500000        0.83    3    2    2  6.9e-11  3.1e-15
   3        0.82   0.3500000        0.82    4    3    3  5.5e-11    3e-15
Velocity u2, Pressure p
Nonsymmetric matrix found.
Scales for dependent variables:
Pressure (comp1.p): 1
Velocity field (spatial frame) (comp1.u2): 0.00022
Orthonormal null-space function used.
Iter      SolEst     Damping    Stepsize #Res #Jac #Sol   LinErr   LinRes
   1           1   0.5000000           1    2    1    1  1.6e-15  2.2e-15
Displacement field
Nonsymmetric matrix found.
Scales for dependent variables:
Auxiliary pressure (comp1.solid.lemm1.pw): 1
Displacement field (comp1.u): 2.8e-18
Orthonormal null-space function used.
```

```
Iter      SolEst     Damping    Stepsize #Res #Jac #Sol   LinErr   LinRes
   1         1.9   0.5000000         1.9    2    1    1  8.3e-14  1.4e-12
Spatial mesh displacement
Symmetric matrices found.
Scales for dependent variables:
Spatial mesh displacement (comp1.spatial.disp): 1.2e-09
Orthonormal null-space function used.
Iter      SolEst     Damping    Stepsize #Res #Jac #Sol   LinErr   LinRes
   1     1.2e-07   0.5000000     1.2e-07    2    1    1  2.1e-15  1.9e-15
Solution error estimates for segregated groups
1.4, 0.52, 0.94, 6.2e-08
Residual error estimates for segregated groups
8.9e+03, 9.3e+04, 2.3e+04, 1.1
Segregated solver iteration 2.
Merged variables
Scales for dependent variables:
Concentration (comp1.Cl): 1.4e+02
Concentration (comp1.K): 1.4e+02
Electric potential (comp1.V): 0.062
Iter      SolEst     Damping    Stepsize #Res #Jac #Sol   LinErr   LinRes
   1        0.81   0.3500000        0.81    6    4    4  3.3e-14  3.3e-15
   2         0.8   0.3500000         0.8    7    5    5  9.1e-14  3.3e-15
   3        0.79   0.3500000        0.79    8    6    6  4.7e-14  3.2e-15
Velocity u2, Pressure p
Scales for dependent variables:
Pressure (comp1.p): 2.8e+02
Velocity field (spatial frame) (comp1.u2): 0.00058
Iter      SolEst     Damping    Stepsize #Res #Jac #Sol   LinErr   LinRes
   1        0.35   0.5000000        0.35    4    2    2  2.5e-15  7.8e-15
Displacement field
Iter      SolEst     Damping    Stepsize #Res #Jac #Sol   LinErr   LinRes
   1        0.94   0.5000000        0.94    4    2    2  4.7e-14  1.4e-12
Spatial mesh displacement
Iter      SolEst     Damping    Stepsize #Res #Jac #Sol   LinErr   LinRes
   1     1.9e-07   0.5000000     1.9e-07    4    2    2  1.4e-15  1.9e-15
Solution error estimates for segregated groups
1.4, 0.17, 0.47, 9.3e-08
Residual error estimates for segregated groups
1e+03, 1.4e+04, 2.2e+04, 1.2
Segregated solver iteration 3.
Merged variables
Iter      SolEst     Damping    Stepsize #Res #Jac #Sol   LinErr   LinRes
   1        0.78   0.3500000        0.78   10    7    7    1e-13  3.1e-15
   2        0.77   0.3500000        0.77   11    8    8  7.5e-14  3.3e-15
   3        0.76   0.3500000        0.76   12    9    9  7.4e-14  3.3e-15
Velocity u2, Pressure p
Iter      SolEst     Damping    Stepsize #Res #Jac #Sol   LinErr   LinRes
   1        0.15   0.5000000        0.15    6    3    3  3.4e-15  7.7e-15
Displacement field
Iter      SolEst     Damping    Stepsize #Res #Jac #Sol   LinErr   LinRes
   1        0.51   0.5000000        0.51    6    3    3  1.5e-13  1.4e-12
Spatial mesh displacement
Iter      SolEst     Damping    Stepsize #Res #Jac #Sol   LinErr   LinRes
   1     1.8e-07   0.5000000     1.8e-07    6    3    3  1.4e-15  1.9e-15
Solution error estimates for segregated groups
1.3, 0.074, 0.26, 9.2e-08
```

```
Residual error estimates for segregated groups
2.9e+02, 8e+03, 1.7e+04, 1.2
Segregated solver iteration 4.
Merged variables
Iter      SolEst     Damping    Stepsize #Res #Jac #Sol   LinErr   LinRes
   1        0.75   0.3500000        0.75   14   10   10  3.9e-14  3.4e-15
   2        0.73   0.3500000        0.73   15   11   11  1.1e-13    4e-15
   3        0.72   0.3500000        0.72   16   12   12  6.2e-14  3.2e-15
Velocity u2, Pressure p
Iter      SolEst     Damping    Stepsize #Res #Jac #Sol   LinErr   LinRes
   1       0.069   0.5000000       0.069    8    4    4  2.7e-15  7.8e-15
Displacement field
Iter      SolEst     Damping    Stepsize #Res #Jac #Sol   LinErr   LinRes
   1        0.29   0.5000000        0.29    8    4    4  2.1e-13  1.4e-12
Spatial mesh displacement
Iter      SolEst     Damping    Stepsize #Res #Jac #Sol   LinErr   LinRes
   1     1.5e-07   0.5000000     1.5e-07    8    4    4  1.6e-15  1.9e-15
Solution error estimates for segregated groups
1.2, 0.035, 0.14, 7.7e-08
Residual error estimates for segregated groups
1e+02, 4.2e+03, 1.1e+04, 1.3
Segregated solver iteration 5.
Merged variables
Iter      SolEst     Damping    Stepsize #Res #Jac #Sol   LinErr   LinRes
   1        0.52   0.3500000        0.52   18   13   13  4.6e-14  3.1e-15
   2        0.35   0.3500000        0.35   19   14   14  6.6e-14  3.2e-15
   3        0.23   0.3500000        0.23   20   15   15  6.6e-14  3.4e-15
Velocity u2, Pressure p
Iter      SolEst     Damping    Stepsize #Res #Jac #Sol   LinErr   LinRes
   1       0.033   0.5000000       0.033   10    5    5    4e-15  7.8e-15
Displacement field
Iter      SolEst     Damping    Stepsize #Res #Jac #Sol   LinErr   LinRes
   1        0.16   0.5000000        0.16   10    5    5  8.4e-14  1.4e-12
Spatial mesh displacement
Iter      SolEst     Damping    Stepsize #Res #Jac #Sol   LinErr   LinRes
   1     1.2e-07   0.5000000     1.2e-07   10    5    5  1.7e-15  1.9e-15
Solution error estimates for segregated groups
0.4, 0.017, 0.082, 5.8e-08
Residual error estimates for segregated groups
39, 2.2e+03, 7e+03, 1.2
Segregated solver iteration 6.
Merged variables
Iter      SolEst     Damping    Stepsize #Res #Jac #Sol   LinErr   LinRes
   1        0.15   0.3500000        0.15   22   16   16  4.4e-14  3.4e-15
   2         0.1   0.3500000         0.1   23   17   17  4.4e-14  3.2e-15
   3       0.066   0.3500000       0.066   24   18   18  6.6e-14  3.4e-15
Velocity u2, Pressure p
Iter      SolEst     Damping    Stepsize #Res #Jac #Sol   LinErr   LinRes
   1       0.016   0.5000000       0.016   12    6    6  3.1e-15  7.8e-15
Displacement field
Iter      SolEst     Damping    Stepsize #Res #Jac #Sol   LinErr   LinRes
   1       0.094   0.5000000       0.094   12    6    6  1.6e-13  1.4e-12
Spatial mesh displacement
Iter      SolEst     Damping    Stepsize #Res #Jac #Sol   LinErr   LinRes
   1     8.1e-08   0.5000000     8.1e-08   12    6    6  1.6e-15  1.9e-15
Solution error estimates for segregated groups
```

```
0.11, 0.0082, 0.047, 4e-08
Residual error estimates for segregated groups
14, 1.1e+03, 4.3e+03, 1.2
Segregated solver iteration 7.
Merged variables
Iter      SolEst     Damping    Stepsize #Res #Jac #Sol   LinErr   LinRes
   1       0.043   0.3500000       0.043   26   19   19  6.4e-14  3.3e-15
   2       0.028   0.3500000       0.028   27   20   20  3.8e-14  3.3e-15
   3       0.018   0.3500000       0.018   28   21   21  4.4e-14  3.5e-15
Velocity u2, Pressure p
Iter      SolEst     Damping    Stepsize #Res #Jac #Sol   LinErr   LinRes
   1      0.0082   0.5000000      0.0082   14    7    7  5.1e-15  7.8e-15
Displacement field
Iter      SolEst     Damping    Stepsize #Res #Jac #Sol   LinErr   LinRes
   1       0.053   0.5000000       0.053   14    7    7  1.3e-13  1.4e-12
Spatial mesh displacement
Iter      SolEst     Damping    Stepsize #Res #Jac #Sol   LinErr   LinRes
   1     5.4e-08   0.5000000     5.4e-08   14    7    7  1.2e-15  1.9e-15
Solution error estimates for segregated groups
0.031, 0.0041, 0.027, 2.7e-08
Residual error estimates for segregated groups
4.7, 5.5e+02, 2.7e+03, 1.3
Segregated solver iteration 8.
Merged variables
Iter      SolEst     Damping    Stepsize #Res #Jac #Sol   LinErr   LinRes
   1       0.012   0.3500000       0.012   30   22   22  5.6e-14  3.2e-15
   2      0.0078   0.3500000      0.0078   31   23   23  4.2e-14  3.2e-15
   3       0.005   0.3500000       0.005   32   24   24  8.7e-14  3.4e-15
Velocity u2, Pressure p
Iter      SolEst     Damping    Stepsize #Res #Jac #Sol   LinErr   LinRes
   1      0.0041   0.5000000      0.0041   16    8    8  2.3e-15  7.7e-15
Displacement field
Iter      SolEst     Damping    Stepsize #Res #Jac #Sol   LinErr   LinRes
   1        0.03   0.5000000        0.03   16    8    8  1.1e-13  1.4e-12
Spatial mesh displacement
Iter      SolEst     Damping    Stepsize #Res #Jac #Sol   LinErr   LinRes
   1     3.4e-08   0.5000000     3.4e-08   16    8    8  1.9e-15  1.9e-15
Solution error estimates for segregated groups
0.0087, 0.002, 0.015, 1.7e-08
Residual error estimates for segregated groups
1.6, 2.8e+02, 1.8e+03, 1.3
Segregated solver iteration 9.
Merged variables
Iter      SolEst     Damping    Stepsize #Res #Jac #Sol   LinErr   LinRes
   1      0.0033   0.3500000      0.0033   34   25   25  4.3e-14  3.4e-15
   2      0.0021   0.3500000      0.0021   35   26   26    6e-14  3.3e-15
   3      0.0014   0.3500000      0.0014   36   27   27  8.2e-14  3.3e-15
Velocity u2, Pressure p
Iter      SolEst     Damping    Stepsize #Res #Jac #Sol   LinErr   LinRes
   1       0.002   0.5000000       0.002   18    9    9  2.6e-15  7.8e-15
Displacement field
Iter      SolEst     Damping    Stepsize #Res #Jac #Sol   LinErr   LinRes
   1       0.017   0.5000000       0.017   18    9    9  1.8e-13  1.4e-12
Spatial mesh displacement
Iter      SolEst     Damping    Stepsize #Res #Jac #Sol   LinErr   LinRes
   1     2.2e-08   0.5000000     2.2e-08   18    9    9  1.4e-15  1.9e-15
```

```
Solution error estimates for segregated groups
0.0024, 0.001, 0.0083, 1.1e-08
Residual error estimates for segregated groups
0.5, 1.4e+02, 1.4e+03, 1.3
Segregated solver iteration 10.
Merged variables
Iter      SolEst     Damping    Stepsize #Res #Jac #Sol   LinErr   LinRes
   1      0.0009   0.3500000      0.0009   38   28   28  8.8e-14  3.4e-15
   2     0.00059   0.3500000     0.00059   39   29   29  3.8e-14  3.2e-15
   3     0.00038   0.3500000     0.00038   40   30   30  5.6e-14  3.5e-15
Velocity u2, Pressure p
Iter      SolEst     Damping    Stepsize #Res #Jac #Sol   LinErr   LinRes
   1       0.001   0.5000000       0.001   20   10   10  2.4e-15  7.8e-15
Displacement field
Iter      SolEst     Damping    Stepsize #Res #Jac #Sol   LinErr   LinRes
   1      0.0092   0.5000000      0.0092   20   10   10  3.8e-14  1.3e-12
Spatial mesh displacement
Iter      SolEst     Damping    Stepsize #Res #Jac #Sol   LinErr   LinRes
   1     1.3e-08   0.5000000     1.3e-08   20   10   10  1.9e-15  1.9e-15
Solution error estimates for segregated groups
0.00065, 0.00051, 0.0046, 6.6e-09
Residual error estimates for segregated groups
0.16, 70, 1.2e+03, 1.3
Segregated solver iteration 11.
Merged variables
Iter      SolEst     Damping    Stepsize #Res #Jac #Sol   LinErr   LinRes
   1     0.00025   0.3500000     0.00025   42   31   31  5.8e-14  3.8e-15
   2     0.00016   0.3500000     0.00016   43   32   32  7.1e-14  3.3e-15
   3      0.0001   0.3500000      0.0001   44   33   33  5.4e-14  3.3e-15
Velocity u2, Pressure p
Iter      SolEst     Damping    Stepsize #Res #Jac #Sol   LinErr   LinRes
   1     0.00051   0.5000000     0.00051   22   11   11  2.6e-15  7.7e-15
Displacement field
Iter      SolEst     Damping    Stepsize #Res #Jac #Sol   LinErr   LinRes
   1       0.005   0.5000000       0.005   22   11   11    1e-13  1.4e-12
Spatial mesh displacement
Iter      SolEst     Damping    Stepsize #Res #Jac #Sol   LinErr   LinRes
   1     7.9e-09   0.5000000     7.9e-09   22   11   11    2e-15  1.9e-15
Solution error estimates for segregated groups
0.00018, 0.00025, 0.0025, 3.9e-09
Residual error estimates for segregated groups
0.049, 35, 1.2e+03, 1.3
Segregated solver iteration 12.
Merged variables
Iter      SolEst     Damping    Stepsize #Res #Jac #Sol   LinErr   LinRes
   1     6.8e-05   0.3500000     6.8e-05   46   34   34  4.7e-14  3.4e-15
   2     4.4e-05   0.3500000     4.4e-05   47   35   35    1e-13  3.4e-15
   3     2.9e-05   0.3500000     2.9e-05   48   36   36  3.5e-14  3.4e-15
Velocity u2, Pressure p
Iter      SolEst     Damping    Stepsize #Res #Jac #Sol   LinErr   LinRes
   1     0.00025   0.5000000     0.00025   24   12   12  3.3e-15  7.8e-15
Displacement field
Iter      SolEst     Damping    Stepsize #Res #Jac #Sol   LinErr   LinRes
   1      0.0027   0.5000000      0.0027   24   12   12  6.5e-14  1.4e-12
Spatial mesh displacement
Iter      SolEst     Damping    Stepsize #Res #Jac #Sol   LinErr   LinRes
```

```
   1     4.7e-09   0.5000000     4.7e-09   24    12    12  1.4e-15  1.9e-15
Solution error estimates for segregated groups
4.9e-05, 0.00013, 0.0014, 2.3e-09
Residual error estimates for segregated groups
0.015, 17, 1.2e+03, 1.3
Segregated solver iteration 13.
Merged variables
Iter      SolEst     Damping    Stepsize #Res #Jac #Sol   LinErr   LinRes
   1     1.9e-05   0.3500000     1.9e-05   50    37    37  3.1e-14  3.3e-15
   2     1.2e-05   0.3500000     1.2e-05   51    38    38  7.8e-14  3.1e-15
   3     7.9e-06   0.3500000     7.9e-06   52    39    39  4.3e-14  3.5e-15
Velocity u2, Pressure p
Iter      SolEst     Damping    Stepsize #Res #Jac #Sol   LinErr   LinRes
   1     0.00013   0.5000000     0.00013   26    13    13  4.8e-15  7.8e-15
Displacement field
Iter      SolEst     Damping    Stepsize #Res #Jac #Sol   LinErr   LinRes
   1      0.0015   0.5000000      0.0015   26    13    13  1.1e-13  1.4e-12
Spatial mesh displacement
Iter      SolEst     Damping    Stepsize #Res #Jac #Sol   LinErr   LinRes
   1     2.7e-09   0.5000000     2.7e-09   26    13    13  1.3e-15  1.9e-15
Solution error estimates for segregated groups
1.4e-05, 6.3e-05, 0.00074, 1.4e-09
Residual error estimates for segregated groups
0.0046, 8.7, 1.2e+03, 1.3
Solution time: 177 s. (2 minutes, 57 seconds)
Physical memory: 4.39 GB
Virtual memory: 26.72 GB
Ended at Jan 31, 2025, 4:50:43 PM.
----- Stationary Solver 1 in study1/Approach Curve Solver (sol1) -------------->
```

d=2500 (su2)

GENERAL

Description	Value
Solution	d=2500

LOG

```
<---- Compile Equations: Stationary in study1/Approach Curve Solver (sol1) -----
Started at Jan 31, 2025, 4:50:43 PM.
Parameter d = 2.5E-6 (m).
Geometry shape function: Linear serendipity
Number of vertex elements: 18
Number of boundary elements: 6332
Number of elements: 157844
Minimum element quality: 0.4841
Time: 9 s.
Physical memory: 2.56 GB
Virtual memory: 15.25 GB
Ended at Jan 31, 2025, 4:50:52 PM.
----- Compile Equations: Stationary in study1/Approach Curve Solver (sol1) ---->

<---- Dependent Variables 1 in study1/Approach Curve Solver (sol1) -------------
Started at Jan 31, 2025, 4:50:52 PM.
Solution time: 0 s.
Physical memory: 2.56 GB
Virtual memory: 15.19 GB
Ended at Jan 31, 2025, 4:50:52 PM.
----- Dependent Variables 1 in study1/Approach Curve Solver (sol1) ------------>

<---- Stationary Solver 1 in study1/Approach Curve Solver (sol1) ---------------
Started at Jan 31, 2025, 4:50:53 PM.
Segregated solver
Segregated Step 11 does not have any components solved for.
Number of degrees of freedom solved for: 803563 (plus 12087 internal DOFs).
Segregated solver iteration 1.
Merged variables
Nonsymmetric matrix found.
Scales for dependent variables:
Concentration (comp1.Cl): 1.4e+02
Concentration (comp1.K): 1.4e+02
Electric potential (comp1.V): 1.9e+02
Orthonormal null-space function used.
Iter      SolEst     Damping    Stepsize #Res #Jac #Sol   LinErr   LinRes
   1        0.83   0.3500000        0.83    2    1    1  4.1e-11  2.9e-15
   2        0.81   0.3500000        0.81    3    2    2  3.5e-11  2.9e-15
   3         0.8   0.3500000         0.8    4    3    3    4e-11  2.9e-15
Velocity u2, Pressure p
Nonsymmetric matrix found.
Scales for dependent variables:
Pressure (comp1.p): 1
Velocity field (spatial frame) (comp1.u2): 0.00019
Orthonormal null-space function used.
Iter      SolEst     Damping    Stepsize #Res #Jac #Sol   LinErr   LinRes
   1         1.1   0.5000000         1.1    2    1    1  1.5e-15  2.2e-15
Displacement field
Nonsymmetric matrix found.
Scales for dependent variables:
Auxiliary pressure (comp1.solid.lemm1.pw): 1
Displacement field (comp1.u): 2.8e-18
Orthonormal null-space function used.
Iter      SolEst     Damping    Stepsize #Res #Jac #Sol   LinErr   LinRes
   1         1.9   0.5000000         1.9    2    1    1  2.9e-14  1.5e-12
Spatial mesh displacement
```

```
Symmetric matrices found.
Scales for dependent variables:
Spatial mesh displacement (comp1.spatial.disp): 1.2e-09
Orthonormal null-space function used.
Iter      SolEst     Damping    Stepsize #Res #Jac #Sol   LinErr   LinRes
   1     5.5e-07   0.5000000     5.5e-07    2    1    1  1.5e-15  1.9e-15
Solution error estimates for segregated groups
1.4, 0.53, 0.95, 2.7e-07
Residual error estimates for segregated groups
8e+03, 8.4e+04, 2.2e+04, 0.17
Segregated solver iteration 2.
Merged variables
Scales for dependent variables:
Concentration (comp1.Cl): 1.4e+02
Concentration (comp1.K): 1.4e+02
Electric potential (comp1.V): 0.064
Iter      SolEst     Damping    Stepsize #Res #Jac #Sol   LinErr   LinRes
   1        0.79   0.3500000        0.79    6    4    4  5.9e-14  2.7e-15
   2        0.77   0.3500000        0.77    7    5    5  5.7e-14  2.7e-15
   3        0.76   0.3500000        0.76    8    6    6  4.9e-14  2.7e-15
Velocity u2, Pressure p
Scales for dependent variables:
Pressure (comp1.p): 3.5e+02
Velocity field (spatial frame) (comp1.u2): 0.0007
Iter      SolEst     Damping    Stepsize #Res #Jac #Sol   LinErr   LinRes
   1        0.36   0.5000000        0.36    4    2    2    3e-15    8e-15
Displacement field
Iter      SolEst     Damping    Stepsize #Res #Jac #Sol   LinErr   LinRes
   1        0.95   0.5000000        0.95    4    2    2  1.3e-13  1.5e-12
Spatial mesh displacement
Iter      SolEst     Damping    Stepsize #Res #Jac #Sol   LinErr   LinRes
   1     8.2e-07   0.5000000     8.2e-07    4    2    2  1.7e-15  1.8e-15
Solution error estimates for segregated groups
1.3, 0.18, 0.48, 4.1e-07
Residual error estimates for segregated groups
8.2e+02, 1.3e+04, 2.2e+04, 0.21
Segregated solver iteration 3.
Merged variables
Iter      SolEst     Damping    Stepsize #Res #Jac #Sol   LinErr   LinRes
   1        0.75   0.3500000        0.75   10    7    7  7.3e-14  2.7e-15
   2        0.74   0.3500000        0.74   11    8    8  5.8e-14  2.8e-15
   3        0.72   0.3500000        0.72   12    9    9  5.8e-14  2.8e-15
Velocity u2, Pressure p
Iter      SolEst     Damping    Stepsize #Res #Jac #Sol   LinErr   LinRes
   1        0.15   0.5000000        0.15    6    3    3  2.2e-15  7.9e-15
Displacement field
Iter      SolEst     Damping    Stepsize #Res #Jac #Sol   LinErr   LinRes
   1        0.52   0.5000000        0.52    6    3    3  4.6e-14  1.5e-12
Spatial mesh displacement
Iter      SolEst     Damping    Stepsize #Res #Jac #Sol   LinErr   LinRes
   1     8.2e-07   0.5000000     8.2e-07    6    3    3  1.7e-15  1.9e-15
Solution error estimates for segregated groups
1.2, 0.076, 0.26, 4.1e-07
Residual error estimates for segregated groups
2.3e+02, 7.3e+03, 1.6e+04, 0.2
Segregated solver iteration 4.
```

```
Merged variables
Iter      SolEst     Damping    Stepsize #Res #Jac #Sol   LinErr   LinRes
   1        0.71   0.3500000        0.71   14   10   10  4.2e-14  2.7e-15
   2        0.69   0.3500000        0.69   15   11   11  5.3e-14  2.7e-15
   3        0.61   0.3500000        0.61   16   12   12  5.8e-14  2.9e-15
Velocity u2, Pressure p
Iter      SolEst     Damping    Stepsize #Res #Jac #Sol   LinErr   LinRes
   1       0.071   0.5000000       0.071    8    4    4  2.7e-15  7.8e-15
Displacement field
Iter      SolEst     Damping    Stepsize #Res #Jac #Sol   LinErr   LinRes
   1        0.29   0.5000000        0.29    8    4    4  3.7e-14  1.5e-12
Spatial mesh displacement
Iter      SolEst     Damping    Stepsize #Res #Jac #Sol   LinErr   LinRes
   1     6.8e-07   0.5000000     6.8e-07    8    4    4  1.7e-15  1.9e-15
Solution error estimates for segregated groups
1, 0.036, 0.15, 3.4e-07
Residual error estimates for segregated groups
86, 3.8e+03, 1.1e+04, 0.2
Segregated solver iteration 5.
Merged variables
Iter      SolEst     Damping    Stepsize #Res #Jac #Sol   LinErr   LinRes
   1        0.42   0.3500000        0.42   18   13   13  9.3e-14  2.8e-15
   2        0.28   0.3500000        0.28   19   14   14  7.7e-14  2.8e-15
   3        0.19   0.3500000        0.19   20   15   15  5.4e-14  2.8e-15
Velocity u2, Pressure p
Iter      SolEst     Damping    Stepsize #Res #Jac #Sol   LinErr   LinRes
   1       0.034   0.5000000       0.034   10    5    5    3e-15  7.8e-15
Displacement field
Iter      SolEst     Damping    Stepsize #Res #Jac #Sol   LinErr   LinRes
   1        0.17   0.5000000        0.17   10    5    5  9.1e-14  1.5e-12
Spatial mesh displacement
Iter      SolEst     Damping    Stepsize #Res #Jac #Sol   LinErr   LinRes
   1     5.1e-07   0.5000000     5.1e-07   10    5    5  1.4e-15  1.9e-15
Solution error estimates for segregated groups
0.32, 0.017, 0.083, 2.6e-07
Residual error estimates for segregated groups
33, 2e+03, 6.9e+03, 0.22
Segregated solver iteration 6.
Merged variables
Iter      SolEst     Damping    Stepsize #Res #Jac #Sol   LinErr   LinRes
   1        0.12   0.3500000        0.12   22   16   16  7.5e-14  2.7e-15
   2        0.08   0.3500000        0.08   23   17   17  5.4e-14  2.7e-15
   3       0.053   0.3500000       0.053   24   18   18  4.8e-14  2.8e-15
Velocity u2, Pressure p
Iter      SolEst     Damping    Stepsize #Res #Jac #Sol   LinErr   LinRes
   1       0.017   0.5000000       0.017   12    6    6    4e-15  7.7e-15
Displacement field
Iter      SolEst     Damping    Stepsize #Res #Jac #Sol   LinErr   LinRes
   1       0.095   0.5000000       0.095   12    6    6  5.3e-14  1.5e-12
Spatial mesh displacement
Iter      SolEst     Damping    Stepsize #Res #Jac #Sol   LinErr   LinRes
   1     3.6e-07   0.5000000     3.6e-07   12    6    6  1.3e-15  1.9e-15
Solution error estimates for segregated groups
0.09, 0.0085, 0.048, 1.8e-07
Residual error estimates for segregated groups
12, 1e+03, 4.3e+03, 0.2
```

```
Segregated solver iteration 7.
Merged variables
Iter      SolEst     Damping    Stepsize #Res #Jac #Sol   LinErr   LinRes
   1       0.034   0.3500000       0.034   26   19   19  9.9e-14  2.7e-15
   2       0.022   0.3500000       0.022   27   20   20  4.2e-14  2.7e-15
   3       0.015   0.3500000       0.015   28   21   21  4.8e-14  2.8e-15
Velocity u2, Pressure p
Iter      SolEst     Damping    Stepsize #Res #Jac #Sol   LinErr   LinRes
   1      0.0084   0.5000000      0.0084   14    7    7  3.3e-15  7.9e-15
Displacement field
Iter      SolEst     Damping    Stepsize #Res #Jac #Sol   LinErr   LinRes
   1       0.054   0.5000000       0.054   14    7    7  1.1e-13  1.5e-12
Spatial mesh displacement
Iter      SolEst     Damping    Stepsize #Res #Jac #Sol   LinErr   LinRes
   1     2.4e-07   0.5000000     2.4e-07   14    7    7  1.6e-15  1.9e-15
Solution error estimates for segregated groups
0.025, 0.0042, 0.027, 1.2e-07
Residual error estimates for segregated groups
4.2, 5e+02, 2.7e+03, 0.22
Segregated solver iteration 8.
Merged variables
Iter      SolEst     Damping    Stepsize #Res #Jac #Sol   LinErr   LinRes
   1      0.0094   0.3500000      0.0094   30   22   22  6.9e-14  2.8e-15
   2      0.0061   0.3500000      0.0061   31   23   23    2e-13  2.7e-15
   3       0.004   0.3500000       0.004   32   24   24  6.8e-14  2.8e-15
Velocity u2, Pressure p
Iter      SolEst     Damping    Stepsize #Res #Jac #Sol   LinErr   LinRes
   1      0.0042   0.5000000      0.0042   16    8    8  1.9e-15  7.8e-15
Displacement field
Iter      SolEst     Damping    Stepsize #Res #Jac #Sol   LinErr   LinRes
   1        0.03   0.5000000        0.03   16    8    8  7.8e-14  1.5e-12
Spatial mesh displacement
Iter      SolEst     Damping    Stepsize #Res #Jac #Sol   LinErr   LinRes
   1     1.5e-07   0.5000000     1.5e-07   16    8    8  1.7e-15  1.9e-15
Solution error estimates for segregated groups
0.0069, 0.0021, 0.015, 7.7e-08
Residual error estimates for segregated groups
1.4, 2.5e+02, 1.8e+03, 0.22
Segregated solver iteration 9.
Merged variables
Iter      SolEst     Damping    Stepsize #Res #Jac #Sol   LinErr   LinRes
   1      0.0026   0.3500000      0.0026   34   25   25    7e-14  2.7e-15
   2      0.0017   0.3500000      0.0017   35   26   26  7.8e-14  2.9e-15
   3      0.0011   0.3500000      0.0011   36   27   27  5.8e-14  2.8e-15
Velocity u2, Pressure p
Iter      SolEst     Damping    Stepsize #Res #Jac #Sol   LinErr   LinRes
   1      0.0021   0.5000000      0.0021   18    9    9  2.9e-15  7.8e-15
Displacement field
Iter      SolEst     Damping    Stepsize #Res #Jac #Sol   LinErr   LinRes
   1       0.017   0.5000000       0.017   18    9    9  1.1e-13  1.5e-12
Spatial mesh displacement
Iter      SolEst     Damping    Stepsize #Res #Jac #Sol   LinErr   LinRes
   1     9.6e-08   0.5000000     9.6e-08   18    9    9  1.7e-15  1.8e-15
Solution error estimates for segregated groups
0.0019, 0.001, 0.0085, 4.8e-08
Residual error estimates for segregated groups
```

```
0.45, 1.3e+02, 1.4e+03, 0.23
Segregated solver iteration 10.
Merged variables
Iter      SolEst     Damping    Stepsize #Res #Jac #Sol   LinErr   LinRes
   1     0.00071   0.3500000     0.00071   38   28   28    5e-14  2.7e-15
   2     0.00046   0.3500000     0.00046   39   29   29  6.6e-14  2.8e-15
   3      0.0003   0.3500000      0.0003   40   30   30  7.9e-14  2.7e-15
Velocity u2, Pressure p
Iter      SolEst     Damping    Stepsize #Res #Jac #Sol   LinErr   LinRes
   1       0.001   0.5000000       0.001   20   10   10  2.3e-15  7.8e-15
Displacement field
Iter      SolEst     Damping    Stepsize #Res #Jac #Sol   LinErr   LinRes
   1      0.0093   0.5000000      0.0093   20   10   10  7.3e-14  1.5e-12
Spatial mesh displacement
Iter      SolEst     Damping    Stepsize #Res #Jac #Sol   LinErr   LinRes
   1     5.9e-08   0.5000000     5.9e-08   20   10   10  1.7e-15  1.8e-15
Solution error estimates for segregated groups
0.00052, 0.00052, 0.0047, 2.9e-08
Residual error estimates for segregated groups
0.14, 63, 1.2e+03, 0.22
Segregated solver iteration 11.
Merged variables
Iter      SolEst     Damping    Stepsize #Res #Jac #Sol   LinErr   LinRes
   1      0.0002   0.3500000      0.0002   42   31   31  8.6e-14  2.7e-15
   2     0.00013   0.3500000     0.00013   43   32   32  1.4e-13  2.9e-15
   3     8.3e-05   0.3500000     8.3e-05   44   33   33  6.1e-14  2.8e-15
Velocity u2, Pressure p
Iter      SolEst     Damping    Stepsize #Res #Jac #Sol   LinErr   LinRes
   1     0.00052   0.5000000     0.00052   22   11   11  1.8e-15  7.9e-15
Displacement field
Iter      SolEst     Damping    Stepsize #Res #Jac #Sol   LinErr   LinRes
   1      0.0051   0.5000000      0.0051   22   11   11  1.1e-13  1.5e-12
Spatial mesh displacement
Iter      SolEst     Damping    Stepsize #Res #Jac #Sol   LinErr   LinRes
   1     3.5e-08   0.5000000     3.5e-08   22   11   11  1.6e-15  1.9e-15
Solution error estimates for segregated groups
0.00014, 0.00026, 0.0026, 1.8e-08
Residual error estimates for segregated groups
0.044, 32, 1.2e+03, 0.22
Segregated solver iteration 12.
Merged variables
Iter      SolEst     Damping    Stepsize #Res #Jac #Sol   LinErr   LinRes
   1     5.4e-05   0.3500000     5.4e-05   46   34   34    5e-14  2.8e-15
   2     3.5e-05   0.3500000     3.5e-05   47   35   35  1.3e-13  2.7e-15
   3     2.3e-05   0.3500000     2.3e-05   48   36   36  7.7e-14  2.7e-15
Velocity u2, Pressure p
Iter      SolEst     Damping    Stepsize #Res #Jac #Sol   LinErr   LinRes
   1     0.00026   0.5000000     0.00026   24   12   12  2.1e-15  7.8e-15
Displacement field
Iter      SolEst     Damping    Stepsize #Res #Jac #Sol   LinErr   LinRes
   1      0.0028   0.5000000      0.0028   24   12   12  7.1e-14  1.5e-12
Spatial mesh displacement
Iter      SolEst     Damping    Stepsize #Res #Jac #Sol   LinErr   LinRes
   1     2.1e-08   0.5000000     2.1e-08   24   12   12  1.5e-15  1.9e-15
Solution error estimates for segregated groups
3.9e-05, 0.00013, 0.0014, 1e-08
```

```
Residual error estimates for segregated groups
0.013, 16, 1.2e+03, 0.21
Segregated solver iteration 13.
Merged variables
Iter      SolEst      Damping     Stepsize #Res #Jac #Sol   LinErr   LinRes
   1     1.5e-05    0.3500000     1.5e-05    50   37   37  8.3e-14  2.8e-15
   2     9.6e-06    0.3500000     9.6e-06    51   38   38  5.4e-14  2.8e-15
   3     6.2e-06    0.3500000     6.2e-06    52   39   39  8.8e-14  2.7e-15
Velocity u2, Pressure p
Iter      SolEst      Damping     Stepsize #Res #Jac #Sol   LinErr   LinRes
   1     0.00013    0.5000000     0.00013    26   13   13  2.3e-15  7.8e-15
Displacement field
Iter      SolEst      Damping     Stepsize #Res #Jac #Sol   LinErr   LinRes
   1      0.0015    0.5000000      0.0015    26   13   13  1.6e-13  1.5e-12
Spatial mesh displacement
Iter      SolEst      Damping     Stepsize #Res #Jac #Sol   LinErr   LinRes
   1     1.2e-08    0.5000000     1.2e-08    26   13   13  1.7e-15  1.9e-15
Solution error estimates for segregated groups
1.1e-05, 6.5e-05, 0.00076, 6.1e-09
Residual error estimates for segregated groups
0.0041, 7.9, 1.2e+03, 0.22
Solution time: 157 s. (2 minutes, 37 seconds)
Physical memory: 4.41 GB
Virtual memory: 25.66 GB
Ended at Jan 31, 2025, 4:53:29 PM.
----- Stationary Solver 1 in study1/Approach Curve Solver (sol1) -------------->
```

d=2495 (su3)

GENERAL

Description	Value
Solution	d=2495

LOG

```
<---- Compile Equations: Stationary in study1/Approach Curve Solver (sol1) -----
Started at Jan 31, 2025, 4:53:30 PM.
Parameter d = 2.4950000000000003E-6 (m).
Geometry shape function: Linear serendipity
Number of vertex elements: 18
Number of boundary elements: 6329
Number of elements: 157707
Minimum element quality: 0.4691
Time: 9 s.
Physical memory: 2.67 GB
Virtual memory: 15.2 GB
Ended at Jan 31, 2025, 4:53:39 PM.
----- Compile Equations: Stationary in study1/Approach Curve Solver (sol1) ---->

<---- Dependent Variables 1 in study1/Approach Curve Solver (sol1) -------------
Started at Jan 31, 2025, 4:53:39 PM.
Solution time: 0 s.
Physical memory: 2.67 GB
Virtual memory: 15.2 GB
Ended at Jan 31, 2025, 4:53:39 PM.
----- Dependent Variables 1 in study1/Approach Curve Solver (sol1) ------------>

<---- Stationary Solver 1 in study1/Approach Curve Solver (sol1) ---------------
Started at Jan 31, 2025, 4:53:39 PM.
Segregated solver
Segregated Step 11 does not have any components solved for.
Number of degrees of freedom solved for: 802822 (plus 12081 internal DOFs).
Segregated solver iteration 1.
Merged variables
Nonsymmetric matrix found.
Scales for dependent variables:
Concentration (comp1.Cl): 1.4e+02
Concentration (comp1.K): 1.4e+02
Electric potential (comp1.V): 1.9e+02
Orthonormal null-space function used.
Iter      SolEst     Damping    Stepsize #Res #Jac #Sol   LinErr   LinRes
   1        0.83   0.3500000        0.83    2    1    1  3.2e-11  2.8e-15
   2        0.81   0.3500000        0.81    3    2    2  2.7e-11  2.8e-15
   3         0.8   0.3500000         0.8    4    3    3  6.6e-11  2.8e-15
Velocity u2, Pressure p
Nonsymmetric matrix found.
Scales for dependent variables:
Pressure (comp1.p): 1
Velocity field (spatial frame) (comp1.u2): 0.00019
Orthonormal null-space function used.
Iter      SolEst     Damping    Stepsize #Res #Jac #Sol   LinErr   LinRes
   1         1.1   0.5000000         1.1    2    1    1  1.7e-15  2.3e-15
Displacement field
Nonsymmetric matrix found.
Scales for dependent variables:
Auxiliary pressure (comp1.solid.lemm1.pw): 1
Displacement field (comp1.u): 2.8e-18
Orthonormal null-space function used.
Iter      SolEst     Damping    Stepsize #Res #Jac #Sol   LinErr   LinRes
   1         1.9   0.5000000         1.9    2    1    1    1e-13  1.5e-12
Spatial mesh displacement
```

```
Symmetric matrices found.
Scales for dependent variables:
Spatial mesh displacement (comp1.spatial.disp): 1.2e-09
Orthonormal null-space function used.
Iter      SolEst     Damping    Stepsize #Res #Jac #Sol   LinErr   LinRes
   1     5.5e-07   0.5000000     5.5e-07    2    1    1  1.6e-15  1.9e-15
Solution error estimates for segregated groups
1.4, 0.53, 0.95, 2.7e-07
Residual error estimates for segregated groups
8e+03, 8.4e+04, 2.2e+04, 0.18
Segregated solver iteration 2.
Merged variables
Scales for dependent variables:
Concentration (comp1.Cl): 1.4e+02
Concentration (comp1.K): 1.4e+02
Electric potential (comp1.V): 0.064
Iter      SolEst     Damping    Stepsize #Res #Jac #Sol   LinErr   LinRes
   1        0.79   0.3500000        0.79    6    4    4  2.4e-13  7.2e-15
   2        0.77   0.3500000        0.77    7    5    5  1.8e-13  4.5e-15
   3        0.76   0.3500000        0.76    8    6    6  5.1e-13  7.7e-15
Velocity u2, Pressure p
Scales for dependent variables:
Pressure (comp1.p): 3.5e+02
Velocity field (spatial frame) (comp1.u2): 0.0007
Iter      SolEst     Damping    Stepsize #Res #Jac #Sol   LinErr   LinRes
   1        0.36   0.5000000        0.36    4    2    2  3.8e-15  7.9e-15
Displacement field
Iter      SolEst     Damping    Stepsize #Res #Jac #Sol   LinErr   LinRes
   1        0.95   0.5000000        0.95    4    2    2  7.6e-14  1.5e-12
Spatial mesh displacement
Iter      SolEst     Damping    Stepsize #Res #Jac #Sol   LinErr   LinRes
   1     8.2e-07   0.5000000     8.2e-07    4    2    2  1.2e-15  1.9e-15
Solution error estimates for segregated groups
1.3, 0.18, 0.48, 4.1e-07
Residual error estimates for segregated groups
8.2e+02, 1.3e+04, 2.2e+04, 0.2
Segregated solver iteration 3.
Merged variables
Iter      SolEst     Damping    Stepsize #Res #Jac #Sol   LinErr   LinRes
   1        0.75   0.3500000        0.75   10    7    7  1.5e-13  4.1e-15
   2        0.74   0.3500000        0.74   11    8    8  1.3e-13  6.9e-15
   3        0.72   0.3500000        0.72   12    9    9  9.8e-14  6.7e-15
Velocity u2, Pressure p
Iter      SolEst     Damping    Stepsize #Res #Jac #Sol   LinErr   LinRes
   1        0.15   0.5000000        0.15    6    3    3  2.4e-15  7.8e-15
Displacement field
Iter      SolEst     Damping    Stepsize #Res #Jac #Sol   LinErr   LinRes
   1        0.52   0.5000000        0.52    6    3    3  1.5e-13  1.5e-12
Spatial mesh displacement
Iter      SolEst     Damping    Stepsize #Res #Jac #Sol   LinErr   LinRes
   1     8.2e-07   0.5000000     8.2e-07    6    3    3  1.7e-15  1.9e-15
Solution error estimates for segregated groups
1.2, 0.076, 0.26, 4.1e-07
Residual error estimates for segregated groups
2.3e+02, 7.4e+03, 1.6e+04, 0.21
Segregated solver iteration 4.
```

```
Merged variables
Iter      SolEst     Damping    Stepsize #Res #Jac #Sol   LinErr   LinRes
   1        0.71   0.3500000        0.71   14   10   10  1.5e-13  4.1e-15
   2        0.69   0.3500000        0.69   15   11   11  1.4e-13  8.7e-15
   3        0.61   0.3500000        0.61   16   12   12  3.8e-13  9.6e-15
Velocity u2, Pressure p
Iter      SolEst     Damping    Stepsize #Res #Jac #Sol   LinErr   LinRes
   1       0.071   0.5000000       0.071    8    4    4  4.8e-15  7.8e-15
Displacement field
Iter      SolEst     Damping    Stepsize #Res #Jac #Sol   LinErr   LinRes
   1        0.29   0.5000000        0.29    8    4    4  6.1e-14  1.5e-12
Spatial mesh displacement
Iter      SolEst     Damping    Stepsize #Res #Jac #Sol   LinErr   LinRes
   1     6.9e-07   0.5000000     6.9e-07    8    4    4  1.3e-15  1.9e-15
Solution error estimates for segregated groups
1, 0.036, 0.15, 3.4e-07
Residual error estimates for segregated groups
86, 3.9e+03, 1.1e+04, 0.2
Segregated solver iteration 5.
Merged variables
Iter      SolEst     Damping    Stepsize #Res #Jac #Sol   LinErr   LinRes
   1        0.42   0.3500000        0.42   18   13   13  3.8e-13  8.1e-15
   2        0.28   0.3500000        0.28   19   14   14  1.4e-13  5.5e-15
   3        0.19   0.3500000        0.19   20   15   15  4.9e-14  4.4e-15
Velocity u2, Pressure p
Iter      SolEst     Damping    Stepsize #Res #Jac #Sol   LinErr   LinRes
   1       0.035   0.5000000       0.035   10    5    5  2.7e-15  7.8e-15
Displacement field
Iter      SolEst     Damping    Stepsize #Res #Jac #Sol   LinErr   LinRes
   1        0.17   0.5000000        0.17   10    5    5  1.1e-13  1.5e-12
Spatial mesh displacement
Iter      SolEst     Damping    Stepsize #Res #Jac #Sol   LinErr   LinRes
   1     5.2e-07   0.5000000     5.2e-07   10    5    5  1.8e-15  1.9e-15
Solution error estimates for segregated groups
0.32, 0.017, 0.083, 2.6e-07
Residual error estimates for segregated groups
33, 2e+03, 6.9e+03, 0.22
Segregated solver iteration 6.
Merged variables
Iter      SolEst     Damping    Stepsize #Res #Jac #Sol   LinErr   LinRes
   1        0.12   0.3500000        0.12   22   16   16  2.6e-13  5.7e-15
   2        0.08   0.3500000        0.08   23   17   17  2.5e-13  4.5e-15
   3       0.052   0.3500000       0.052   24   18   18  1.3e-13  5.5e-15
Velocity u2, Pressure p
Iter      SolEst     Damping    Stepsize #Res #Jac #Sol   LinErr   LinRes
   1       0.017   0.5000000       0.017   12    6    6  3.4e-15  7.9e-15
Displacement field
Iter      SolEst     Damping    Stepsize #Res #Jac #Sol   LinErr   LinRes
   1       0.095   0.5000000       0.095   12    6    6  9.8e-14  1.5e-12
Spatial mesh displacement
Iter      SolEst     Damping    Stepsize #Res #Jac #Sol   LinErr   LinRes
   1     3.6e-07   0.5000000     3.6e-07   12    6    6  1.7e-15  1.8e-15
Solution error estimates for segregated groups
0.09, 0.0085, 0.048, 1.8e-07
Residual error estimates for segregated groups
12, 1e+03, 4.3e+03, 0.21
```

```
Segregated solver iteration 7.
Merged variables
Iter      SolEst     Damping    Stepsize #Res #Jac #Sol   LinErr   LinRes
   1       0.034   0.3500000       0.034   26   19   19  2.1e-13  6.6e-15
   2       0.022   0.3500000       0.022   27   20   20  1.3e-13  4.2e-15
   3       0.014   0.3500000       0.014   28   21   21  1.5e-13  4.6e-15
Velocity u2, Pressure p
Iter      SolEst     Damping    Stepsize #Res #Jac #Sol   LinErr   LinRes
   1      0.0084   0.5000000      0.0084   14    7    7  2.1e-15  7.8e-15
Displacement field
Iter      SolEst     Damping    Stepsize #Res #Jac #Sol   LinErr   LinRes
   1       0.054   0.5000000       0.054   14    7    7  8.4e-14  1.5e-12
Spatial mesh displacement
Iter      SolEst     Damping    Stepsize #Res #Jac #Sol   LinErr   LinRes
   1     2.4e-07   0.5000000     2.4e-07   14    7    7  2.5e-15  1.9e-15
Solution error estimates for segregated groups
0.025, 0.0042, 0.027, 1.2e-07
Residual error estimates for segregated groups
4.2, 5.1e+02, 2.7e+03, 0.2
Segregated solver iteration 8.
Merged variables
Iter      SolEst     Damping    Stepsize #Res #Jac #Sol   LinErr   LinRes
   1      0.0094   0.3500000      0.0094   30   22   22  1.9e-13  5.8e-15
   2      0.0061   0.3500000      0.0061   31   23   23  1.1e-13    7e-15
   3       0.004   0.3500000       0.004   32   24   24  1.3e-13  5.4e-15
Velocity u2, Pressure p
Iter      SolEst     Damping    Stepsize #Res #Jac #Sol   LinErr   LinRes
   1      0.0042   0.5000000      0.0042   16    8    8  2.2e-15  7.8e-15
Displacement field
Iter      SolEst     Damping    Stepsize #Res #Jac #Sol   LinErr   LinRes
   1        0.03   0.5000000        0.03   16    8    8  1.8e-13  1.5e-12
Spatial mesh displacement
Iter      SolEst     Damping    Stepsize #Res #Jac #Sol   LinErr   LinRes
   1     1.5e-07   0.5000000     1.5e-07   16    8    8  1.4e-15  1.9e-15
Solution error estimates for segregated groups
0.0068, 0.0021, 0.015, 7.7e-08
Residual error estimates for segregated groups
1.4, 2.6e+02, 1.8e+03, 0.2
Segregated solver iteration 9.
Merged variables
Iter      SolEst     Damping    Stepsize #Res #Jac #Sol   LinErr   LinRes
   1      0.0026   0.3500000      0.0026   34   25   25  9.7e-14  4.1e-15
   2      0.0017   0.3500000      0.0017   35   26   26    8e-14  4.9e-15
   3      0.0011   0.3500000      0.0011   36   27   27  2.6e-13  7.1e-15
Velocity u2, Pressure p
Iter      SolEst     Damping    Stepsize #Res #Jac #Sol   LinErr   LinRes
   1      0.0021   0.5000000      0.0021   18    9    9  2.7e-15  7.8e-15
Displacement field
Iter      SolEst     Damping    Stepsize #Res #Jac #Sol   LinErr   LinRes
   1       0.017   0.5000000       0.017   18    9    9  5.1e-14  1.5e-12
Spatial mesh displacement
Iter      SolEst     Damping    Stepsize #Res #Jac #Sol   LinErr   LinRes
   1     9.7e-08   0.5000000     9.7e-08   18    9    9  1.6e-15  1.9e-15
Solution error estimates for segregated groups
0.0019, 0.001, 0.0085, 4.8e-08
Residual error estimates for segregated groups
```

```
0.45, 1.3e+02, 1.4e+03, 0.2
Segregated solver iteration 10.
Merged variables
Iter      SolEst     Damping    Stepsize #Res #Jac #Sol   LinErr   LinRes
   1     0.00071   0.3500000     0.00071   38   28   28  2.3e-13  5.6e-15
   2     0.00046   0.3500000     0.00046   39   29   29  2.4e-13  6.4e-15
   3      0.0003   0.3500000      0.0003   40   30   30    2e-13  5.6e-15
Velocity u2, Pressure p
Iter      SolEst     Damping    Stepsize #Res #Jac #Sol   LinErr   LinRes
   1       0.001   0.5000000       0.001   20   10   10  1.9e-15  7.8e-15
Displacement field
Iter      SolEst     Damping    Stepsize #Res #Jac #Sol   LinErr   LinRes
   1      0.0094   0.5000000      0.0094   20   10   10  2.5e-14  1.5e-12
Spatial mesh displacement
Iter      SolEst     Damping    Stepsize #Res #Jac #Sol   LinErr   LinRes
   1     5.9e-08   0.5000000     5.9e-08   20   10   10  1.9e-15  1.9e-15
Solution error estimates for segregated groups
0.00052, 0.00052, 0.0047, 3e-08
Residual error estimates for segregated groups
0.14, 64, 1.3e+03, 0.21
Segregated solver iteration 11.
Merged variables
Iter      SolEst     Damping    Stepsize #Res #Jac #Sol   LinErr   LinRes
   1      0.0002   0.3500000      0.0002   42   31   31  2.1e-13  5.8e-15
   2     0.00013   0.3500000     0.00013   43   32   32  2.3e-13  7.8e-15
   3     8.3e-05   0.3500000     8.3e-05   44   33   33  3.8e-13  6.2e-15
Velocity u2, Pressure p
Iter      SolEst     Damping    Stepsize #Res #Jac #Sol   LinErr   LinRes
   1     0.00052   0.5000000     0.00052   22   11   11  2.2e-15  7.9e-15
Displacement field
Iter      SolEst     Damping    Stepsize #Res #Jac #Sol   LinErr   LinRes
   1      0.0051   0.5000000      0.0051   22   11   11  1.6e-13  1.5e-12
Spatial mesh displacement
Iter      SolEst     Damping    Stepsize #Res #Jac #Sol   LinErr   LinRes
   1     3.5e-08   0.5000000     3.5e-08   22   11   11  1.5e-15  1.9e-15
Solution error estimates for segregated groups
0.00014, 0.00026, 0.0026, 1.8e-08
Residual error estimates for segregated groups
0.044, 32, 1.2e+03, 0.22
Segregated solver iteration 12.
Merged variables
Iter      SolEst     Damping    Stepsize #Res #Jac #Sol   LinErr   LinRes
   1     5.4e-05   0.3500000     5.4e-05   46   34   34    1e-13  7.1e-15
   2     3.5e-05   0.3500000     3.5e-05   47   35   35    2e-13  7.8e-15
   3     2.3e-05   0.3500000     2.3e-05   48   36   36  4.5e-13  8.3e-15
Velocity u2, Pressure p
Iter      SolEst     Damping    Stepsize #Res #Jac #Sol   LinErr   LinRes
   1     0.00026   0.5000000     0.00026   24   12   12  2.4e-15  7.8e-15
Displacement field
Iter      SolEst     Damping    Stepsize #Res #Jac #Sol   LinErr   LinRes
   1      0.0028   0.5000000      0.0028   24   12   12    9e-14  1.5e-12
Spatial mesh displacement
Iter      SolEst     Damping    Stepsize #Res #Jac #Sol   LinErr   LinRes
   1     2.1e-08   0.5000000     2.1e-08   24   12   12  1.5e-15  1.9e-15
Solution error estimates for segregated groups
3.9e-05, 0.00013, 0.0014, 1e-08
```

```
Residual error estimates for segregated groups
0.013, 16, 1.2e+03, 0.21
Segregated solver iteration 13.
Merged variables
Iter      SolEst     Damping    Stepsize #Res #Jac #Sol   LinErr   LinRes
   1     1.5e-05   0.3500000     1.5e-05   50   37   37  1.1e-13  4.4e-15
   2     9.6e-06   0.3500000     9.6e-06   51   38   38  4.1e-13    7e-15
   3     6.2e-06   0.3500000     6.2e-06   52   39   39  5.6e-13  9.1e-15
Velocity u2, Pressure p
Iter      SolEst     Damping    Stepsize #Res #Jac #Sol   LinErr   LinRes
   1     0.00013   0.5000000     0.00013   26   13   13  3.1e-15  7.8e-15
Displacement field
Iter      SolEst     Damping    Stepsize #Res #Jac #Sol   LinErr   LinRes
   1      0.0015   0.5000000      0.0015   26   13   13  7.5e-14  1.5e-12
Spatial mesh displacement
Iter      SolEst     Damping    Stepsize #Res #Jac #Sol   LinErr   LinRes
   1     1.2e-08   0.5000000     1.2e-08   26   13   13  1.3e-15  1.9e-15
Solution error estimates for segregated groups
1.1e-05, 6.5e-05, 0.00076, 6.1e-09
Residual error estimates for segregated groups
0.0041, 8, 1.2e+03, 0.2
Solution time: 157 s. (2 minutes, 37 seconds)
Physical memory: 4.45 GB
Virtual memory: 26.27 GB
Ended at Jan 31, 2025, 4:56:16 PM.
----- Stationary Solver 1 in study1/Approach Curve Solver (sol1) -------------->
```

d=2490 (su4)

GENERAL

Description	Value
Solution	d=2490

LOG

```
<---- Compile Equations: Stationary in study1/Approach Curve Solver (sol1) -----
Started at Jan 31, 2025, 4:56:16 PM.
Parameter d = 2.4900000000000003E-6 (m).
Geometry shape function: Linear serendipity
Number of vertex elements: 18
Number of boundary elements: 6326
Number of elements: 157754
Minimum element quality: 0.4704
Time: 9 s.
Physical memory: 2.75 GB
Virtual memory: 15.24 GB
Ended at Jan 31, 2025, 4:56:25 PM.
----- Compile Equations: Stationary in study1/Approach Curve Solver (sol1) ---->

<---- Dependent Variables 1 in study1/Approach Curve Solver (sol1) -------------
Started at Jan 31, 2025, 4:56:25 PM.
Solution time: 0 s.
Physical memory: 2.75 GB
Virtual memory: 15.24 GB
Ended at Jan 31, 2025, 4:56:25 PM.
----- Dependent Variables 1 in study1/Approach Curve Solver (sol1) ------------>

<---- Stationary Solver 1 in study1/Approach Curve Solver (sol1) ---------------
Started at Jan 31, 2025, 4:56:25 PM.
Segregated solver
Segregated Step 11 does not have any components solved for.
Number of degrees of freedom solved for: 802987 (plus 12075 internal DOFs).
Segregated solver iteration 1.
Merged variables
Nonsymmetric matrix found.
Scales for dependent variables:
Concentration (comp1.Cl): 1.4e+02
Concentration (comp1.K): 1.4e+02
Electric potential (comp1.V): 1.9e+02
Orthonormal null-space function used.
Iter      SolEst     Damping    Stepsize #Res #Jac #Sol   LinErr   LinRes
   1        0.83   0.3500000        0.83    2    1    1    3e-11  2.8e-15
   2        0.81   0.3500000        0.81    3    2    2    2e-11  2.8e-15
   3         0.8   0.3500000         0.8    4    3    3  5.2e-11  2.7e-15
Velocity u2, Pressure p
Nonsymmetric matrix found.
Scales for dependent variables:
Pressure (comp1.p): 1
Velocity field (spatial frame) (comp1.u2): 0.00019
Orthonormal null-space function used.
Iter      SolEst     Damping    Stepsize #Res #Jac #Sol   LinErr   LinRes
   1         1.1   0.5000000         1.1    2    1    1  1.5e-15  2.3e-15
Displacement field
Nonsymmetric matrix found.
Scales for dependent variables:
Auxiliary pressure (comp1.solid.lemm1.pw): 1
Displacement field (comp1.u): 2.8e-18
Orthonormal null-space function used.
Iter      SolEst     Damping    Stepsize #Res #Jac #Sol   LinErr   LinRes
   1         1.9   0.5000000         1.9    2    1    1  1.7e-13  1.5e-12
Spatial mesh displacement
```

```
Symmetric matrices found.
Scales for dependent variables:
Spatial mesh displacement (comp1.spatial.disp): 1.2e-09
Orthonormal null-space function used.
Iter      SolEst     Damping    Stepsize #Res #Jac #Sol   LinErr   LinRes
   1     5.5e-07   0.5000000     5.5e-07    2    1    1  2.4e-15  1.9e-15
Solution error estimates for segregated groups
1.4, 0.53, 0.95, 2.8e-07
Residual error estimates for segregated groups
8e+03, 8.3e+04, 2.2e+04, 0.18
Segregated solver iteration 2.
Merged variables
Scales for dependent variables:
Concentration (comp1.Cl): 1.4e+02
Concentration (comp1.K): 1.4e+02
Electric potential (comp1.V): 0.064
Iter      SolEst     Damping    Stepsize #Res #Jac #Sol   LinErr   LinRes
   1        0.79   0.3500000        0.79    6    4    4  1.6e-13  4.7e-15
   2        0.77   0.3500000        0.77    7    5    5  8.8e-14  5.7e-15
   3        0.76   0.3500000        0.76    8    6    6    1e-13  7.3e-15
Velocity u2, Pressure p
Scales for dependent variables:
Pressure (comp1.p): 3.5e+02
Velocity field (spatial frame) (comp1.u2): 0.00071
Iter      SolEst     Damping    Stepsize #Res #Jac #Sol   LinErr   LinRes
   1        0.36   0.5000000        0.36    4    2    2  2.6e-15  8.1e-15
Displacement field
Iter      SolEst     Damping    Stepsize #Res #Jac #Sol   LinErr   LinRes
   1        0.95   0.5000000        0.95    4    2    2  1.3e-13  1.5e-12
Spatial mesh displacement
Iter      SolEst     Damping    Stepsize #Res #Jac #Sol   LinErr   LinRes
   1     8.3e-07   0.5000000     8.3e-07    4    2    2  2.1e-15  1.9e-15
Solution error estimates for segregated groups
1.3, 0.18, 0.48, 4.1e-07
Residual error estimates for segregated groups
8.2e+02, 1.3e+04, 2.2e+04, 0.18
Segregated solver iteration 3.
Merged variables
Iter      SolEst     Damping    Stepsize #Res #Jac #Sol   LinErr   LinRes
   1        0.75   0.3500000        0.75   10    7    7  7.5e-14  5.1e-15
   2        0.74   0.3500000        0.74   11    8    8  7.8e-14    9e-15
   3        0.72   0.3500000        0.72   12    9    9  1.1e-13    6e-15
Velocity u2, Pressure p
Iter      SolEst     Damping    Stepsize #Res #Jac #Sol   LinErr   LinRes
   1        0.15   0.5000000        0.15    6    3    3    3e-15  7.8e-15
Displacement field
Iter      SolEst     Damping    Stepsize #Res #Jac #Sol   LinErr   LinRes
   1        0.52   0.5000000        0.52    6    3    3  3.4e-14  1.5e-12
Spatial mesh displacement
Iter      SolEst     Damping    Stepsize #Res #Jac #Sol   LinErr   LinRes
   1     8.3e-07   0.5000000     8.3e-07    6    3    3  1.9e-15  1.9e-15
Solution error estimates for segregated groups
1.2, 0.076, 0.26, 4.1e-07
Residual error estimates for segregated groups
2.3e+02, 7.3e+03, 1.6e+04, 0.2
Segregated solver iteration 4.
```

```
Merged variables
Iter      SolEst     Damping    Stepsize #Res #Jac #Sol   LinErr   LinRes
   1        0.71   0.3500000        0.71   14   10   10  1.5e-13  4.2e-15
   2        0.69   0.3500000        0.69   15   11   11  1.1e-13  4.4e-15
   3        0.61   0.3500000        0.61   16   12   12  1.2e-13  4.2e-15
Velocity u2, Pressure p
Iter      SolEst     Damping    Stepsize #Res #Jac #Sol   LinErr   LinRes
   1       0.071   0.5000000       0.071    8    4    4  2.9e-15    8e-15
Displacement field
Iter      SolEst     Damping    Stepsize #Res #Jac #Sol   LinErr   LinRes
   1        0.29   0.5000000        0.29    8    4    4  2.7e-14  1.5e-12
Spatial mesh displacement
Iter      SolEst     Damping    Stepsize #Res #Jac #Sol   LinErr   LinRes
   1     6.9e-07   0.5000000     6.9e-07    8    4    4  1.3e-15  1.9e-15
Solution error estimates for segregated groups
1, 0.036, 0.15, 3.4e-07
Residual error estimates for segregated groups
86, 3.9e+03, 1.1e+04, 0.21
Segregated solver iteration 5.
Merged variables
Iter      SolEst     Damping    Stepsize #Res #Jac #Sol   LinErr   LinRes
   1        0.42   0.3500000        0.42   18   13   13  9.7e-14  3.8e-15
   2        0.28   0.3500000        0.28   19   14   14  1.1e-13  5.2e-15
   3        0.19   0.3500000        0.19   20   15   15  1.2e-13  1.1e-14
Velocity u2, Pressure p
Iter      SolEst     Damping    Stepsize #Res #Jac #Sol   LinErr   LinRes
   1       0.035   0.5000000       0.035   10    5    5  2.8e-15  7.9e-15
Displacement field
Iter      SolEst     Damping    Stepsize #Res #Jac #Sol   LinErr   LinRes
   1        0.17   0.5000000        0.17   10    5    5    5e-14  1.5e-12
Spatial mesh displacement
Iter      SolEst     Damping    Stepsize #Res #Jac #Sol   LinErr   LinRes
   1     5.2e-07   0.5000000     5.2e-07   10    5    5    2e-15  1.9e-15
Solution error estimates for segregated groups
0.32, 0.017, 0.083, 2.6e-07
Residual error estimates for segregated groups
33, 2e+03, 6.9e+03, 0.21
Segregated solver iteration 6.
Merged variables
Iter      SolEst     Damping    Stepsize #Res #Jac #Sol   LinErr   LinRes
   1        0.12   0.3500000        0.12   22   16   16  1.2e-13  5.8e-15
   2        0.08   0.3500000        0.08   23   17   17  8.8e-14  4.4e-15
   3       0.052   0.3500000       0.052   24   18   18  1.4e-13  4.2e-15
Velocity u2, Pressure p
Iter      SolEst     Damping    Stepsize #Res #Jac #Sol   LinErr   LinRes
   1       0.017   0.5000000       0.017   12    6    6  2.6e-15  7.9e-15
Displacement field
Iter      SolEst     Damping    Stepsize #Res #Jac #Sol   LinErr   LinRes
   1       0.095   0.5000000       0.095   12    6    6  3.5e-14  1.5e-12
Spatial mesh displacement
Iter      SolEst     Damping    Stepsize #Res #Jac #Sol   LinErr   LinRes
   1     3.6e-07   0.5000000     3.6e-07   12    6    6  1.4e-15  1.9e-15
Solution error estimates for segregated groups
0.09, 0.0085, 0.048, 1.8e-07
Residual error estimates for segregated groups
12, 1e+03, 4.3e+03, 0.21
```

```
Segregated solver iteration 7.
Merged variables
Iter      SolEst     Damping    Stepsize #Res #Jac #Sol   LinErr   LinRes
   1       0.034   0.3500000       0.034   26   19   19  9.2e-14  3.8e-15
   2       0.022   0.3500000       0.022   27   20   20  7.5e-14  4.1e-15
   3       0.014   0.3500000       0.014   28   21   21  1.4e-13  4.8e-15
Velocity u2, Pressure p
Iter      SolEst     Damping    Stepsize #Res #Jac #Sol   LinErr   LinRes
   1      0.0084   0.5000000      0.0084   14    7    7  4.2e-15  7.9e-15
Displacement field
Iter      SolEst     Damping    Stepsize #Res #Jac #Sol   LinErr   LinRes
   1       0.054   0.5000000       0.054   14    7    7  8.3e-14  1.5e-12
Spatial mesh displacement
Iter      SolEst     Damping    Stepsize #Res #Jac #Sol   LinErr   LinRes
   1     2.4e-07   0.5000000     2.4e-07   14    7    7  1.5e-15  1.9e-15
Solution error estimates for segregated groups
0.025, 0.0042, 0.027, 1.2e-07
Residual error estimates for segregated groups
4.2, 5e+02, 2.7e+03, 0.2
Segregated solver iteration 8.
Merged variables
Iter      SolEst     Damping    Stepsize #Res #Jac #Sol   LinErr   LinRes
   1      0.0094   0.3500000      0.0094   30   22   22  1.1e-13  3.8e-15
   2      0.0061   0.3500000      0.0061   31   23   23  1.5e-13  3.4e-15
   3       0.004   0.3500000       0.004   32   24   24  8.2e-14  8.5e-15
Velocity u2, Pressure p
Iter      SolEst     Damping    Stepsize #Res #Jac #Sol   LinErr   LinRes
   1      0.0042   0.5000000      0.0042   16    8    8  2.9e-15  7.9e-15
Displacement field
Iter      SolEst     Damping    Stepsize #Res #Jac #Sol   LinErr   LinRes
   1        0.03   0.5000000        0.03   16    8    8  8.1e-14  1.5e-12
Spatial mesh displacement
Iter      SolEst     Damping    Stepsize #Res #Jac #Sol   LinErr   LinRes
   1     1.6e-07   0.5000000     1.6e-07   16    8    8  1.4e-15  1.8e-15
Solution error estimates for segregated groups
0.0068, 0.0021, 0.015, 7.8e-08
Residual error estimates for segregated groups
1.4, 2.5e+02, 1.8e+03, 0.21
Segregated solver iteration 9.
Merged variables
Iter      SolEst     Damping    Stepsize #Res #Jac #Sol   LinErr   LinRes
   1      0.0026   0.3500000      0.0026   34   25   25  1.6e-13    4e-15
   2      0.0017   0.3500000      0.0017   35   26   26  1.1e-13  8.5e-15
   3      0.0011   0.3500000      0.0011   36   27   27  1.4e-13  3.8e-15
Velocity u2, Pressure p
Iter      SolEst     Damping    Stepsize #Res #Jac #Sol   LinErr   LinRes
   1      0.0021   0.5000000      0.0021   18    9    9  5.8e-15  7.9e-15
Displacement field
Iter      SolEst     Damping    Stepsize #Res #Jac #Sol   LinErr   LinRes
   1       0.017   0.5000000       0.017   18    9    9  9.6e-14  1.5e-12
Spatial mesh displacement
Iter      SolEst     Damping    Stepsize #Res #Jac #Sol   LinErr   LinRes
   1     9.7e-08   0.5000000     9.7e-08   18    9    9  1.7e-15  1.8e-15
Solution error estimates for segregated groups
0.0019, 0.001, 0.0085, 4.9e-08
Residual error estimates for segregated groups
```

```
0.45, 1.3e+02, 1.4e+03, 0.2
Segregated solver iteration 10.
Merged variables
Iter      SolEst     Damping    Stepsize #Res #Jac #Sol   LinErr   LinRes
   1     0.00071   0.3500000     0.00071   38   28   28    8e-14  3.8e-15
   2     0.00046   0.3500000     0.00046   39   29   29  6.1e-14  5.2e-15
   3      0.0003   0.3500000      0.0003   40   30   30  9.6e-14  5.9e-15
Velocity u2, Pressure p
Iter      SolEst     Damping    Stepsize #Res #Jac #Sol   LinErr   LinRes
   1       0.001   0.5000000       0.001   20   10   10  4.2e-15  7.8e-15
Displacement field
Iter      SolEst     Damping    Stepsize #Res #Jac #Sol   LinErr   LinRes
   1      0.0093   0.5000000      0.0093   20   10   10    1e-13  1.5e-12
Spatial mesh displacement
Iter      SolEst     Damping    Stepsize #Res #Jac #Sol   LinErr   LinRes
   1     5.9e-08   0.5000000     5.9e-08   20   10   10  1.4e-15  1.9e-15
Solution error estimates for segregated groups
0.00052, 0.00052, 0.0047, 3e-08
Residual error estimates for segregated groups
0.14, 63, 1.3e+03, 0.21
Segregated solver iteration 11.
Merged variables
Iter      SolEst     Damping    Stepsize #Res #Jac #Sol   LinErr   LinRes
   1      0.0002   0.3500000      0.0002   42   31   31  7.5e-14  6.9e-15
   2     0.00013   0.3500000     0.00013   43   32   32  1.1e-13  8.5e-15
   3     8.3e-05   0.3500000     8.3e-05   44   33   33  1.1e-13  5.4e-15
Velocity u2, Pressure p
Iter      SolEst     Damping    Stepsize #Res #Jac #Sol   LinErr   LinRes
   1     0.00052   0.5000000     0.00052   22   11   11  2.4e-15  7.8e-15
Displacement field
Iter      SolEst     Damping    Stepsize #Res #Jac #Sol   LinErr   LinRes
   1      0.0051   0.5000000      0.0051   22   11   11  8.7e-14  1.5e-12
Spatial mesh displacement
Iter      SolEst     Damping    Stepsize #Res #Jac #Sol   LinErr   LinRes
   1     3.6e-08   0.5000000     3.6e-08   22   11   11  1.9e-15  1.9e-15
Solution error estimates for segregated groups
0.00014, 0.00026, 0.0026, 1.8e-08
Residual error estimates for segregated groups
0.044, 32, 1.2e+03, 0.2
Segregated solver iteration 12.
Merged variables
Iter      SolEst     Damping    Stepsize #Res #Jac #Sol   LinErr   LinRes
   1     5.4e-05   0.3500000     5.4e-05   46   34   34    1e-13  4.4e-15
   2     3.5e-05   0.3500000     3.5e-05   47   35   35  1.6e-13  5.2e-15
   3     2.3e-05   0.3500000     2.3e-05   48   36   36    1e-13  4.1e-15
Velocity u2, Pressure p
Iter      SolEst     Damping    Stepsize #Res #Jac #Sol   LinErr   LinRes
   1     0.00026   0.5000000     0.00026   24   12   12  3.2e-15  7.9e-15
Displacement field
Iter      SolEst     Damping    Stepsize #Res #Jac #Sol   LinErr   LinRes
   1      0.0028   0.5000000      0.0028   24   12   12  5.1e-14  1.5e-12
Spatial mesh displacement
Iter      SolEst     Damping    Stepsize #Res #Jac #Sol   LinErr   LinRes
   1     2.1e-08   0.5000000     2.1e-08   24   12   12  2.3e-15  1.9e-15
Solution error estimates for segregated groups
3.9e-05, 0.00013, 0.0014, 1.1e-08
```

```
Residual error estimates for segregated groups
0.013, 16, 1.2e+03, 0.22
Segregated solver iteration 13.
Merged variables
Iter      SolEst     Damping    Stepsize #Res #Jac #Sol   LinErr   LinRes
   1     1.5e-05   0.3500000     1.5e-05   50   37   37  1.1e-13  1.3e-14
   2     9.6e-06   0.3500000     9.6e-06   51   38   38  1.4e-13  6.9e-15
   3     6.2e-06   0.3500000     6.2e-06   52   39   39  1.1e-13  4.7e-15
Velocity u2, Pressure p
Iter      SolEst     Damping    Stepsize #Res #Jac #Sol   LinErr   LinRes
   1     0.00013   0.5000000     0.00013   26   13   13  2.6e-15  7.9e-15
Displacement field
Iter      SolEst     Damping    Stepsize #Res #Jac #Sol   LinErr   LinRes
   1      0.0015   0.5000000      0.0015   26   13   13    1e-13  1.5e-12
Spatial mesh displacement
Iter      SolEst     Damping    Stepsize #Res #Jac #Sol   LinErr   LinRes
   1     1.2e-08   0.5000000     1.2e-08   26   13   13  1.3e-15  1.9e-15
Solution error estimates for segregated groups
1.1e-05, 6.5e-05, 0.00076, 6.1e-09
Residual error estimates for segregated groups
0.0041, 7.9, 1.2e+03, 0.21
Solution time: 158 s. (2 minutes, 38 seconds)
Physical memory: 4.44 GB
Virtual memory: 25.83 GB
Ended at Jan 31, 2025, 4:59:03 PM.
----- Stationary Solver 1 in study1/Approach Curve Solver (sol1) -------------->
```

d=2485 (su5)

GENERAL

Description	Value
Solution	d=2485

LOG

```
<---- Compile Equations: Stationary in study1/Approach Curve Solver (sol1) -----
Started at Jan 31, 2025, 4:59:04 PM.
Parameter d = 2.4850000000000003E-6 (m).
Geometry shape function: Linear serendipity
Number of vertex elements: 18
Number of boundary elements: 6324
Number of elements: 157436
Minimum element quality: 0.4843
Time: 9 s.
Physical memory: 2.72 GB
Virtual memory: 15.2 GB
Ended at Jan 31, 2025, 4:59:13 PM.
----- Compile Equations: Stationary in study1/Approach Curve Solver (sol1) ---->

<---- Dependent Variables 1 in study1/Approach Curve Solver (sol1) -------------
Started at Jan 31, 2025, 4:59:13 PM.
Solution time: 0 s.
Physical memory: 2.72 GB
Virtual memory: 15.2 GB
Ended at Jan 31, 2025, 4:59:13 PM.
----- Dependent Variables 1 in study1/Approach Curve Solver (sol1) ------------>

<---- Stationary Solver 1 in study1/Approach Curve Solver (sol1) ---------------
Started at Jan 31, 2025, 4:59:13 PM.
Segregated solver
Segregated Step 11 does not have any components solved for.
Number of degrees of freedom solved for: 801423 (plus 12071 internal DOFs).
Segregated solver iteration 1.
Merged variables
Nonsymmetric matrix found.
Scales for dependent variables:
Concentration (comp1.Cl): 1.4e+02
Concentration (comp1.K): 1.4e+02
Electric potential (comp1.V): 1.9e+02
Orthonormal null-space function used.
Iter      SolEst     Damping    Stepsize #Res #Jac #Sol   LinErr   LinRes
   1        0.83   0.3500000        0.83    2    1    1  3.1e-11  2.8e-15
   2        0.81   0.3500000        0.81    3    2    2  4.6e-11  2.8e-15
   3         0.8   0.3500000         0.8    4    3    3    6e-11  2.7e-15
Velocity u2, Pressure p
Nonsymmetric matrix found.
Scales for dependent variables:
Pressure (comp1.p): 1
Velocity field (spatial frame) (comp1.u2): 0.00019
Orthonormal null-space function used.
Iter      SolEst     Damping    Stepsize #Res #Jac #Sol   LinErr   LinRes
   1         1.1   0.5000000         1.1    2    1    1    2e-15  2.3e-15
Displacement field
Nonsymmetric matrix found.
Scales for dependent variables:
Auxiliary pressure (comp1.solid.lemm1.pw): 1
Displacement field (comp1.u): 2.7e-18
Orthonormal null-space function used.
Iter      SolEst     Damping    Stepsize #Res #Jac #Sol   LinErr   LinRes
   1         1.9   0.5000000         1.9    2    1    1  1.4e-13  1.5e-12
Spatial mesh displacement
```

```
Symmetric matrices found.
Scales for dependent variables:
Spatial mesh displacement (comp1.spatial.disp): 1.2e-09
Orthonormal null-space function used.
Iter      SolEst     Damping    Stepsize #Res #Jac #Sol   LinErr   LinRes
   1     5.6e-07   0.5000000     5.6e-07    2    1    1  1.8e-15  1.9e-15
Solution error estimates for segregated groups
1.4, 0.54, 0.95, 2.8e-07
Residual error estimates for segregated groups
8e+03, 8.3e+04, 2.2e+04, 0.18
Segregated solver iteration 2.
Merged variables
Scales for dependent variables:
Concentration (comp1.Cl): 1.4e+02
Concentration (comp1.K): 1.4e+02
Electric potential (comp1.V): 0.064
Iter      SolEst     Damping    Stepsize #Res #Jac #Sol   LinErr   LinRes
   1        0.79   0.3500000        0.79    6    4    4  7.5e-14  2.8e-15
   2        0.77   0.3500000        0.77    7    5    5  1.2e-13  4.4e-15
   3        0.76   0.3500000        0.76    8    6    6  7.8e-14  3.4e-15
Velocity u2, Pressure p
Scales for dependent variables:
Pressure (comp1.p): 3.5e+02
Velocity field (spatial frame) (comp1.u2): 0.00071
Iter      SolEst     Damping    Stepsize #Res #Jac #Sol   LinErr   LinRes
   1        0.36   0.5000000        0.36    4    2    2  2.7e-15  7.8e-15
Displacement field
Iter      SolEst     Damping    Stepsize #Res #Jac #Sol   LinErr   LinRes
   1        0.95   0.5000000        0.95    4    2    2  2.2e-13  1.5e-12
Spatial mesh displacement
Iter      SolEst     Damping    Stepsize #Res #Jac #Sol   LinErr   LinRes
   1     8.3e-07   0.5000000     8.3e-07    4    2    2  1.5e-15  1.8e-15
Solution error estimates for segregated groups
1.3, 0.18, 0.48, 4.2e-07
Residual error estimates for segregated groups
8.2e+02, 1.3e+04, 2.2e+04, 0.2
Segregated solver iteration 3.
Merged variables
Iter      SolEst     Damping    Stepsize #Res #Jac #Sol   LinErr   LinRes
   1        0.75   0.3500000        0.75   10    7    7  8.4e-14  3.2e-15
   2        0.74   0.3500000        0.74   11    8    8  4.6e-14  3.9e-15
   3        0.72   0.3500000        0.72   12    9    9    6e-14  3.7e-15
Velocity u2, Pressure p
Iter      SolEst     Damping    Stepsize #Res #Jac #Sol   LinErr   LinRes
   1        0.15   0.5000000        0.15    6    3    3  2.6e-15  7.9e-15
Displacement field
Iter      SolEst     Damping    Stepsize #Res #Jac #Sol   LinErr   LinRes
   1        0.52   0.5000000        0.52    6    3    3  7.7e-14  1.5e-12
Spatial mesh displacement
Iter      SolEst     Damping    Stepsize #Res #Jac #Sol   LinErr   LinRes
   1     8.3e-07   0.5000000     8.3e-07    6    3    3  1.5e-15  1.9e-15
Solution error estimates for segregated groups
1.2, 0.076, 0.26, 4.2e-07
Residual error estimates for segregated groups
2.3e+02, 7.3e+03, 1.6e+04, 0.19
Segregated solver iteration 4.
```

```
Merged variables
Iter      SolEst     Damping    Stepsize #Res #Jac #Sol   LinErr   LinRes
   1        0.71   0.3500000        0.71   14   10   10  6.5e-14  3.6e-15
   2        0.69   0.3500000        0.69   15   11   11  6.5e-14  3.4e-15
   3        0.61   0.3500000        0.61   16   12   12  6.6e-14    3e-15
Velocity u2, Pressure p
Iter      SolEst     Damping    Stepsize #Res #Jac #Sol   LinErr   LinRes
   1       0.071   0.5000000       0.071    8    4    4  2.3e-15  7.8e-15
Displacement field
Iter      SolEst     Damping    Stepsize #Res #Jac #Sol   LinErr   LinRes
   1        0.29   0.5000000        0.29    8    4    4  1.6e-13  1.5e-12
Spatial mesh displacement
Iter      SolEst     Damping    Stepsize #Res #Jac #Sol   LinErr   LinRes
   1     6.9e-07   0.5000000     6.9e-07    8    4    4  1.7e-15  1.9e-15
Solution error estimates for segregated groups
1, 0.036, 0.15, 3.5e-07
Residual error estimates for segregated groups
86, 3.8e+03, 1.1e+04, 0.21
Segregated solver iteration 5.
Merged variables
Iter      SolEst     Damping    Stepsize #Res #Jac #Sol   LinErr   LinRes
   1        0.42   0.3500000        0.42   18   13   13  4.2e-14  3.4e-15
   2        0.28   0.3500000        0.28   19   14   14  5.8e-14  2.9e-15
   3        0.19   0.3500000        0.19   20   15   15  1.1e-13  3.1e-15
Velocity u2, Pressure p
Iter      SolEst     Damping    Stepsize #Res #Jac #Sol   LinErr   LinRes
   1       0.035   0.5000000       0.035   10    5    5  3.4e-15  7.9e-15
Displacement field
Iter      SolEst     Damping    Stepsize #Res #Jac #Sol   LinErr   LinRes
   1        0.17   0.5000000        0.17   10    5    5  1.6e-13  1.5e-12
Spatial mesh displacement
Iter      SolEst     Damping    Stepsize #Res #Jac #Sol   LinErr   LinRes
   1     5.2e-07   0.5000000     5.2e-07   10    5    5  1.7e-15  1.9e-15
Solution error estimates for segregated groups
0.32, 0.017, 0.083, 2.6e-07
Residual error estimates for segregated groups
33, 2e+03, 6.9e+03, 0.22
Segregated solver iteration 6.
Merged variables
Iter      SolEst     Damping    Stepsize #Res #Jac #Sol   LinErr   LinRes
   1        0.12   0.3500000        0.12   22   16   16  7.1e-14  3.3e-15
   2        0.08   0.3500000        0.08   23   17   17  1.1e-13  3.3e-15
   3       0.052   0.3500000       0.052   24   18   18  1.2e-13  3.8e-15
Velocity u2, Pressure p
Iter      SolEst     Damping    Stepsize #Res #Jac #Sol   LinErr   LinRes
   1       0.017   0.5000000       0.017   12    6    6  3.1e-15  7.9e-15
Displacement field
Iter      SolEst     Damping    Stepsize #Res #Jac #Sol   LinErr   LinRes
   1       0.095   0.5000000       0.095   12    6    6  7.5e-14  1.5e-12
Spatial mesh displacement
Iter      SolEst     Damping    Stepsize #Res #Jac #Sol   LinErr   LinRes
   1     3.6e-07   0.5000000     3.6e-07   12    6    6  1.7e-15  1.9e-15
Solution error estimates for segregated groups
0.09, 0.0085, 0.048, 1.8e-07
Residual error estimates for segregated groups
12, 9.9e+02, 4.2e+03, 0.21
```

```
Segregated solver iteration 7.
Merged variables
Iter      SolEst     Damping    Stepsize #Res #Jac #Sol   LinErr   LinRes
   1       0.034   0.3500000       0.034   26   19   19  3.2e-14    3e-15
   2       0.022   0.3500000       0.022   27   20   20  7.9e-14  3.1e-15
   3       0.014   0.3500000       0.014   28   21   21  5.6e-14  3.2e-15
Velocity u2, Pressure p
Iter      SolEst     Damping    Stepsize #Res #Jac #Sol   LinErr   LinRes
   1      0.0084   0.5000000      0.0084   14    7    7    3e-15  7.8e-15
Displacement field
Iter      SolEst     Damping    Stepsize #Res #Jac #Sol   LinErr   LinRes
   1       0.054   0.5000000       0.054   14    7    7  1.1e-13  1.5e-12
Spatial mesh displacement
Iter      SolEst     Damping    Stepsize #Res #Jac #Sol   LinErr   LinRes
   1     2.4e-07   0.5000000     2.4e-07   14    7    7  1.7e-15  1.9e-15
Solution error estimates for segregated groups
0.025, 0.0042, 0.027, 1.2e-07
Residual error estimates for segregated groups
4.2, 5e+02, 2.7e+03, 0.21
Segregated solver iteration 8.
Merged variables
Iter      SolEst     Damping    Stepsize #Res #Jac #Sol   LinErr   LinRes
   1      0.0094   0.3500000      0.0094   30   22   22  6.4e-14  4.6e-15
   2      0.0061   0.3500000      0.0061   31   23   23  1.7e-13  3.3e-15
   3       0.004   0.3500000       0.004   32   24   24  5.8e-14  3.2e-15
Velocity u2, Pressure p
Iter      SolEst     Damping    Stepsize #Res #Jac #Sol   LinErr   LinRes
   1      0.0042   0.5000000      0.0042   16    8    8  4.1e-15  7.9e-15
Displacement field
Iter      SolEst     Damping    Stepsize #Res #Jac #Sol   LinErr   LinRes
   1        0.03   0.5000000        0.03   16    8    8  1.4e-13  1.5e-12
Spatial mesh displacement
Iter      SolEst     Damping    Stepsize #Res #Jac #Sol   LinErr   LinRes
   1     1.6e-07   0.5000000     1.6e-07   16    8    8  1.6e-15  1.9e-15
Solution error estimates for segregated groups
0.0068, 0.0021, 0.015, 7.8e-08
Residual error estimates for segregated groups
1.4, 2.5e+02, 1.8e+03, 0.2
Segregated solver iteration 9.
Merged variables
Iter      SolEst     Damping    Stepsize #Res #Jac #Sol   LinErr   LinRes
   1      0.0026   0.3500000      0.0026   34   25   25    1e-13  2.8e-15
   2      0.0017   0.3500000      0.0017   35   26   26    5e-14  3.2e-15
   3      0.0011   0.3500000      0.0011   36   27   27  7.4e-14  3.7e-15
Velocity u2, Pressure p
Iter      SolEst     Damping    Stepsize #Res #Jac #Sol   LinErr   LinRes
   1      0.0021   0.5000000      0.0021   18    9    9  2.3e-15  7.8e-15
Displacement field
Iter      SolEst     Damping    Stepsize #Res #Jac #Sol   LinErr   LinRes
   1       0.017   0.5000000       0.017   18    9    9  5.9e-14  1.5e-12
Spatial mesh displacement
Iter      SolEst     Damping    Stepsize #Res #Jac #Sol   LinErr   LinRes
   1     9.8e-08   0.5000000     9.8e-08   18    9    9  1.6e-15  1.9e-15
Solution error estimates for segregated groups
0.0019, 0.001, 0.0085, 4.9e-08
Residual error estimates for segregated groups
```

```
0.45, 1.3e+02, 1.4e+03, 0.2
Segregated solver iteration 10.
Merged variables
Iter      SolEst     Damping    Stepsize #Res #Jac #Sol   LinErr   LinRes
   1     0.00071   0.3500000     0.00071   38   28   28  1.2e-13    4e-15
   2     0.00046   0.3500000     0.00046   39   29   29  1.4e-13  4.6e-15
   3      0.0003   0.3500000      0.0003   40   30   30  7.4e-14  3.1e-15
Velocity u2, Pressure p
Iter      SolEst     Damping    Stepsize #Res #Jac #Sol   LinErr   LinRes
   1       0.001   0.5000000       0.001   20   10   10    2e-15  7.9e-15
Displacement field
Iter      SolEst     Damping    Stepsize #Res #Jac #Sol   LinErr   LinRes
   1      0.0094   0.5000000      0.0094   20   10   10  6.4e-14  1.5e-12
Spatial mesh displacement
Iter      SolEst     Damping    Stepsize #Res #Jac #Sol   LinErr   LinRes
   1       6e-08   0.5000000       6e-08   20   10   10  2.9e-15  1.9e-15
Solution error estimates for segregated groups
0.00051, 0.00052, 0.0047, 3e-08
Residual error estimates for segregated groups
0.14, 63, 1.3e+03, 0.2
Segregated solver iteration 11.
Merged variables
Iter      SolEst     Damping    Stepsize #Res #Jac #Sol   LinErr   LinRes
   1     0.00019   0.3500000     0.00019   42   31   31    6e-14    3e-15
   2     0.00013   0.3500000     0.00013   43   32   32  6.5e-14  3.7e-15
   3     8.2e-05   0.3500000     8.2e-05   44   33   33  6.7e-14  3.2e-15
Velocity u2, Pressure p
Iter      SolEst     Damping    Stepsize #Res #Jac #Sol   LinErr   LinRes
   1     0.00052   0.5000000     0.00052   22   11   11  6.5e-15  7.8e-15
Displacement field
Iter      SolEst     Damping    Stepsize #Res #Jac #Sol   LinErr   LinRes
   1      0.0051   0.5000000      0.0051   22   11   11  1.4e-13  1.5e-12
Spatial mesh displacement
Iter      SolEst     Damping    Stepsize #Res #Jac #Sol   LinErr   LinRes
   1     3.6e-08   0.5000000     3.6e-08   22   11   11  1.9e-15  1.8e-15
Solution error estimates for segregated groups
0.00014, 0.00026, 0.0026, 1.8e-08
Residual error estimates for segregated groups
0.044, 31, 1.2e+03, 0.21
Segregated solver iteration 12.
Merged variables
Iter      SolEst     Damping    Stepsize #Res #Jac #Sol   LinErr   LinRes
   1     5.4e-05   0.3500000     5.4e-05   46   34   34  9.4e-14  3.6e-15
   2     3.5e-05   0.3500000     3.5e-05   47   35   35  1.2e-13    3e-15
   3     2.3e-05   0.3500000     2.3e-05   48   36   36  1.1e-13  3.6e-15
Velocity u2, Pressure p
Iter      SolEst     Damping    Stepsize #Res #Jac #Sol   LinErr   LinRes
   1     0.00026   0.5000000     0.00026   24   12   12  2.6e-15  7.8e-15
Displacement field
Iter      SolEst     Damping    Stepsize #Res #Jac #Sol   LinErr   LinRes
   1      0.0028   0.5000000      0.0028   24   12   12    4e-14  1.5e-12
Spatial mesh displacement
Iter      SolEst     Damping    Stepsize #Res #Jac #Sol   LinErr   LinRes
   1     2.1e-08   0.5000000     2.1e-08   24   12   12  1.7e-15  1.9e-15
Solution error estimates for segregated groups
3.9e-05, 0.00013, 0.0014, 1.1e-08
```

```
Residual error estimates for segregated groups
0.013, 16, 1.2e+03, 0.19
Segregated solver iteration 13.
Merged variables
Iter      SolEst     Damping    Stepsize #Res #Jac #Sol   LinErr   LinRes
   1     1.5e-05   0.3500000     1.5e-05   50   37   37  7.1e-14    4e-15
   2     9.6e-06   0.3500000     9.6e-06   51   38   38  8.1e-14  2.9e-15
   3     6.2e-06   0.3500000     6.2e-06   52   39   39  1.1e-13    3e-15
Velocity u2, Pressure p
Iter      SolEst     Damping    Stepsize #Res #Jac #Sol   LinErr   LinRes
   1     0.00013   0.5000000     0.00013   26   13   13  2.9e-15  7.9e-15
Displacement field
Iter      SolEst     Damping    Stepsize #Res #Jac #Sol   LinErr   LinRes
   1      0.0015   0.5000000      0.0015   26   13   13    7e-14  1.5e-12
Spatial mesh displacement
Iter      SolEst     Damping    Stepsize #Res #Jac #Sol   LinErr   LinRes
   1     1.2e-08   0.5000000     1.2e-08   26   13   13  1.7e-15  1.9e-15
Solution error estimates for segregated groups
1.1e-05, 6.5e-05, 0.00076, 6.2e-09
Residual error estimates for segregated groups
0.0041, 7.9, 1.2e+03, 0.21
Solution time: 158 s. (2 minutes, 38 seconds)
Physical memory: 4.46 GB
Virtual memory: 25.56 GB
Ended at Jan 31, 2025, 5:01:51 PM.
----- Stationary Solver 1 in study1/Approach Curve Solver (sol1) -------------->
```

d=2480 (su6)

GENERAL

Description	Value
Solution	d=2480

LOG

```
<---- Compile Equations: Stationary in study1/Approach Curve Solver (sol1) -----
Started at Jan 31, 2025, 5:01:51 PM.
Parameter d = 2.48E-6 (m).
Geometry shape function: Linear serendipity
Number of vertex elements: 18
Number of boundary elements: 6321
Number of elements: 157705
Minimum element quality: 0.4484
Time: 9 s.
Physical memory: 2.74 GB
Virtual memory: 15.2 GB
Ended at Jan 31, 2025, 5:02:00 PM.
----- Compile Equations: Stationary in study1/Approach Curve Solver (sol1) ---->

<---- Dependent Variables 1 in study1/Approach Curve Solver (sol1) -------------
Started at Jan 31, 2025, 5:02:00 PM.
Solution time: 0 s.
Physical memory: 2.74 GB
Virtual memory: 15.2 GB
Ended at Jan 31, 2025, 5:02:00 PM.
----- Dependent Variables 1 in study1/Approach Curve Solver (sol1) ------------>

<---- Stationary Solver 1 in study1/Approach Curve Solver (sol1) ---------------
Started at Jan 31, 2025, 5:02:00 PM.
Segregated solver
Segregated Step 11 does not have any components solved for.
Number of degrees of freedom solved for: 802593 (plus 12065 internal DOFs).
Segregated solver iteration 1.
Merged variables
Nonsymmetric matrix found.
Scales for dependent variables:
Concentration (comp1.Cl): 1.4e+02
Concentration (comp1.K): 1.4e+02
Electric potential (comp1.V): 1.9e+02
Orthonormal null-space function used.
Iter      SolEst     Damping    Stepsize #Res #Jac #Sol   LinErr   LinRes
   1        0.83   0.3500000        0.83    2    1    1  1.9e-11  2.8e-15
   2        0.81   0.3500000        0.81    3    2    2  5.1e-11  2.7e-15
   3         0.8   0.3500000         0.8    4    3    3    6e-11  2.7e-15
Velocity u2, Pressure p
Nonsymmetric matrix found.
Scales for dependent variables:
Pressure (comp1.p): 1
Velocity field (spatial frame) (comp1.u2): 0.00019
Orthonormal null-space function used.
Iter      SolEst     Damping    Stepsize #Res #Jac #Sol   LinErr   LinRes
   1         1.1   0.5000000         1.1    2    1    1  1.6e-15  2.3e-15
Displacement field
Nonsymmetric matrix found.
Scales for dependent variables:
Auxiliary pressure (comp1.solid.lemm1.pw): 1
Displacement field (comp1.u): 2.7e-18
Orthonormal null-space function used.
Iter      SolEst     Damping    Stepsize #Res #Jac #Sol   LinErr   LinRes
   1         1.9   0.5000000         1.9    2    1    1    4e-14  1.5e-12
Spatial mesh displacement
```

```
Symmetric matrices found.
Scales for dependent variables:
Spatial mesh displacement (comp1.spatial.disp): 1.2e-09
Orthonormal null-space function used.
Iter      SolEst     Damping    Stepsize #Res #Jac #Sol   LinErr   LinRes
   1     5.6e-07   0.5000000     5.6e-07    2    1    1    2e-15  1.9e-15
Solution error estimates for segregated groups
1.4, 0.53, 0.95, 2.8e-07
Residual error estimates for segregated groups
8e+03, 8.3e+04, 2.2e+04, 0.18
Segregated solver iteration 2.
Merged variables
Scales for dependent variables:
Concentration (comp1.Cl): 1.4e+02
Concentration (comp1.K): 1.4e+02
Electric potential (comp1.V): 0.064
Iter      SolEst     Damping    Stepsize #Res #Jac #Sol   LinErr   LinRes
   1        0.79   0.3500000        0.79    6    4    4  7.6e-14  2.7e-15
   2        0.77   0.3500000        0.77    7    5    5  4.8e-14  2.8e-15
   3        0.76   0.3500000        0.76    8    6    6  1.5e-13  2.8e-15
Velocity u2, Pressure p
Scales for dependent variables:
Pressure (comp1.p): 3.5e+02
Velocity field (spatial frame) (comp1.u2): 0.00071
Iter      SolEst     Damping    Stepsize #Res #Jac #Sol   LinErr   LinRes
   1        0.36   0.5000000        0.36    4    2    2  3.4e-15  7.9e-15
Displacement field
Iter      SolEst     Damping    Stepsize #Res #Jac #Sol   LinErr   LinRes
   1        0.95   0.5000000        0.95    4    2    2  5.2e-14  1.5e-12
Spatial mesh displacement
Iter      SolEst     Damping    Stepsize #Res #Jac #Sol   LinErr   LinRes
   1     8.3e-07   0.5000000     8.3e-07    4    2    2  1.4e-15  1.9e-15
Solution error estimates for segregated groups
1.3, 0.18, 0.48, 4.2e-07
Residual error estimates for segregated groups
8.2e+02, 1.4e+04, 2.2e+04, 0.2
Segregated solver iteration 3.
Merged variables
Iter      SolEst     Damping    Stepsize #Res #Jac #Sol   LinErr   LinRes
   1        0.75   0.3500000        0.75   10    7    7  8.8e-14  2.7e-15
   2        0.74   0.3500000        0.74   11    8    8  4.2e-14  2.7e-15
   3        0.72   0.3500000        0.72   12    9    9  5.8e-14  2.7e-15
Velocity u2, Pressure p
Iter      SolEst     Damping    Stepsize #Res #Jac #Sol   LinErr   LinRes
   1        0.15   0.5000000        0.15    6    3    3  4.7e-15  8.1e-15
Displacement field
Iter      SolEst     Damping    Stepsize #Res #Jac #Sol   LinErr   LinRes
   1        0.52   0.5000000        0.52    6    3    3    5e-14  1.5e-12
Spatial mesh displacement
Iter      SolEst     Damping    Stepsize #Res #Jac #Sol   LinErr   LinRes
   1     8.3e-07   0.5000000     8.3e-07    6    3    3  1.7e-15  1.9e-15
Solution error estimates for segregated groups
1.2, 0.076, 0.26, 4.2e-07
Residual error estimates for segregated groups
2.3e+02, 7.6e+03, 1.6e+04, 0.21
Segregated solver iteration 4.
```

```
Merged variables
Iter      SolEst     Damping    Stepsize #Res #Jac #Sol   LinErr   LinRes
   1        0.71   0.3500000        0.71   14   10   10  6.5e-14  2.8e-15
   2        0.69   0.3500000        0.69   15   11   11  5.5e-14  2.7e-15
   3        0.61   0.3500000        0.61   16   12   12    7e-14  2.8e-15
Velocity u2, Pressure p
Iter      SolEst     Damping    Stepsize #Res #Jac #Sol   LinErr   LinRes
   1       0.071   0.5000000       0.071    8    4    4  3.6e-15  7.8e-15
Displacement field
Iter      SolEst     Damping    Stepsize #Res #Jac #Sol   LinErr   LinRes
   1        0.29   0.5000000        0.29    8    4    4    1e-13  1.5e-12
Spatial mesh displacement
Iter      SolEst     Damping    Stepsize #Res #Jac #Sol   LinErr   LinRes
   1       7e-07   0.5000000       7e-07    8    4    4  1.5e-15  1.8e-15
Solution error estimates for segregated groups
1, 0.036, 0.15, 3.5e-07
Residual error estimates for segregated groups
86, 4e+03, 1.1e+04, 0.2
Segregated solver iteration 5.
Merged variables
Iter      SolEst     Damping    Stepsize #Res #Jac #Sol   LinErr   LinRes
   1        0.42   0.3500000        0.42   18   13   13  8.9e-14  2.8e-15
   2        0.28   0.3500000        0.28   19   14   14  1.4e-13  2.7e-15
   3        0.19   0.3500000        0.19   20   15   15  5.3e-14  2.8e-15
Velocity u2, Pressure p
Iter      SolEst     Damping    Stepsize #Res #Jac #Sol   LinErr   LinRes
   1       0.034   0.5000000       0.034   10    5    5  2.7e-15    8e-15
Displacement field
Iter      SolEst     Damping    Stepsize #Res #Jac #Sol   LinErr   LinRes
   1        0.17   0.5000000        0.17   10    5    5  5.8e-14  1.5e-12
Spatial mesh displacement
Iter      SolEst     Damping    Stepsize #Res #Jac #Sol   LinErr   LinRes
   1     5.2e-07   0.5000000     5.2e-07   10    5    5  1.7e-15  1.9e-15
Solution error estimates for segregated groups
0.32, 0.017, 0.083, 2.6e-07
Residual error estimates for segregated groups
33, 2.1e+03, 6.9e+03, 0.22
Segregated solver iteration 6.
Merged variables
Iter      SolEst     Damping    Stepsize #Res #Jac #Sol   LinErr   LinRes
   1        0.12   0.3500000        0.12   22   16   16    7e-14  2.8e-15
   2        0.08   0.3500000        0.08   23   17   17  4.7e-14  2.7e-15
   3       0.052   0.3500000       0.052   24   18   18  8.6e-14  2.8e-15
Velocity u2, Pressure p
Iter      SolEst     Damping    Stepsize #Res #Jac #Sol   LinErr   LinRes
   1       0.017   0.5000000       0.017   12    6    6  1.8e-15  7.9e-15
Displacement field
Iter      SolEst     Damping    Stepsize #Res #Jac #Sol   LinErr   LinRes
   1       0.095   0.5000000       0.095   12    6    6  9.4e-14  1.5e-12
Spatial mesh displacement
Iter      SolEst     Damping    Stepsize #Res #Jac #Sol   LinErr   LinRes
   1     3.7e-07   0.5000000     3.7e-07   12    6    6  1.6e-15  1.8e-15
Solution error estimates for segregated groups
0.09, 0.0085, 0.048, 1.8e-07
Residual error estimates for segregated groups
12, 1e+03, 4.3e+03, 0.22
```

```
Segregated solver iteration 7.
Merged variables
Iter      SolEst     Damping    Stepsize #Res #Jac #Sol   LinErr   LinRes
   1       0.034   0.3500000       0.034   26   19   19    5e-14  2.7e-15
   2       0.022   0.3500000       0.022   27   20   20  6.4e-14  2.9e-15
   3       0.014   0.3500000       0.014   28   21   21  6.9e-14  2.9e-15
Velocity u2, Pressure p
Iter      SolEst     Damping    Stepsize #Res #Jac #Sol   LinErr   LinRes
   1      0.0084   0.5000000      0.0084   14    7    7  2.4e-15  7.9e-15
Displacement field
Iter      SolEst     Damping    Stepsize #Res #Jac #Sol   LinErr   LinRes
   1       0.054   0.5000000       0.054   14    7    7    7e-14  1.5e-12
Spatial mesh displacement
Iter      SolEst     Damping    Stepsize #Res #Jac #Sol   LinErr   LinRes
   1     2.4e-07   0.5000000     2.4e-07   14    7    7  1.6e-15  1.9e-15
Solution error estimates for segregated groups
0.025, 0.0042, 0.027, 1.2e-07
Residual error estimates for segregated groups
4.2, 5.3e+02, 2.7e+03, 0.21
Segregated solver iteration 8.
Merged variables
Iter      SolEst     Damping    Stepsize #Res #Jac #Sol   LinErr   LinRes
   1      0.0094   0.3500000      0.0094   30   22   22  8.4e-14  2.7e-15
   2      0.0061   0.3500000      0.0061   31   23   23  7.5e-14  2.7e-15
   3       0.004   0.3500000       0.004   32   24   24  7.4e-14  2.8e-15
Velocity u2, Pressure p
Iter      SolEst     Damping    Stepsize #Res #Jac #Sol   LinErr   LinRes
   1      0.0042   0.5000000      0.0042   16    8    8  2.6e-15  7.9e-15
Displacement field
Iter      SolEst     Damping    Stepsize #Res #Jac #Sol   LinErr   LinRes
   1        0.03   0.5000000        0.03   16    8    8    1e-13  1.5e-12
Spatial mesh displacement
Iter      SolEst     Damping    Stepsize #Res #Jac #Sol   LinErr   LinRes
   1     1.6e-07   0.5000000     1.6e-07   16    8    8  1.5e-15  1.9e-15
Solution error estimates for segregated groups
0.0068, 0.0021, 0.015, 7.8e-08
Residual error estimates for segregated groups
1.4, 2.6e+02, 1.8e+03, 0.2
Segregated solver iteration 9.
Merged variables
Iter      SolEst     Damping    Stepsize #Res #Jac #Sol   LinErr   LinRes
   1      0.0026   0.3500000      0.0026   34   25   25  4.3e-14  2.8e-15
   2      0.0017   0.3500000      0.0017   35   26   26  9.1e-14  2.8e-15
   3      0.0011   0.3500000      0.0011   36   27   27  6.8e-14    3e-15
Velocity u2, Pressure p
Iter      SolEst     Damping    Stepsize #Res #Jac #Sol   LinErr   LinRes
   1      0.0021   0.5000000      0.0021   18    9    9  2.5e-15  7.8e-15
Displacement field
Iter      SolEst     Damping    Stepsize #Res #Jac #Sol   LinErr   LinRes
   1       0.017   0.5000000       0.017   18    9    9  4.9e-14  1.5e-12
Spatial mesh displacement
Iter      SolEst     Damping    Stepsize #Res #Jac #Sol   LinErr   LinRes
   1     9.8e-08   0.5000000     9.8e-08   18    9    9  1.3e-15  1.9e-15
Solution error estimates for segregated groups
0.0019, 0.001, 0.0085, 4.9e-08
Residual error estimates for segregated groups
```

```
0.45, 1.3e+02, 1.4e+03, 0.21
Segregated solver iteration 10.
Merged variables
Iter      SolEst     Damping    Stepsize #Res #Jac #Sol   LinErr   LinRes
   1     0.00071   0.3500000     0.00071   38   28   28  1.2e-13  2.7e-15
   2     0.00046   0.3500000     0.00046   39   29   29  6.7e-14  2.8e-15
   3      0.0003   0.3500000      0.0003   40   30   30  4.9e-14  2.8e-15
Velocity u2, Pressure p
Iter      SolEst     Damping    Stepsize #Res #Jac #Sol   LinErr   LinRes
   1       0.001   0.5000000       0.001   20   10   10  2.1e-15  7.9e-15
Displacement field
Iter      SolEst     Damping    Stepsize #Res #Jac #Sol   LinErr   LinRes
   1      0.0094   0.5000000      0.0094   20   10   10  7.2e-14  1.5e-12
Spatial mesh displacement
Iter      SolEst     Damping    Stepsize #Res #Jac #Sol   LinErr   LinRes
   1       6e-08   0.5000000       6e-08   20   10   10  1.4e-15  1.8e-15
Solution error estimates for segregated groups
0.00052, 0.00052, 0.0047, 3e-08
Residual error estimates for segregated groups
0.14, 66, 1.3e+03, 0.2
Segregated solver iteration 11.
Merged variables
Iter      SolEst     Damping    Stepsize #Res #Jac #Sol   LinErr   LinRes
   1     0.00019   0.3500000     0.00019   42   31   31  1.8e-13  2.7e-15
   2     0.00013   0.3500000     0.00013   43   32   32    1e-13  2.9e-15
   3     8.2e-05   0.3500000     8.2e-05   44   33   33  1.1e-13  2.8e-15
Velocity u2, Pressure p
Iter      SolEst     Damping    Stepsize #Res #Jac #Sol   LinErr   LinRes
   1     0.00052   0.5000000     0.00052   22   11   11  2.9e-15  7.8e-15
Displacement field
Iter      SolEst     Damping    Stepsize #Res #Jac #Sol   LinErr   LinRes
   1      0.0051   0.5000000      0.0051   22   11   11  6.5e-14  1.5e-12
Spatial mesh displacement
Iter      SolEst     Damping    Stepsize #Res #Jac #Sol   LinErr   LinRes
   1     3.6e-08   0.5000000     3.6e-08   22   11   11  1.5e-15  1.9e-15
Solution error estimates for segregated groups
0.00014, 0.00026, 0.0026, 1.8e-08
Residual error estimates for segregated groups
0.044, 33, 1.2e+03, 0.19
Segregated solver iteration 12.
Merged variables
Iter      SolEst     Damping    Stepsize #Res #Jac #Sol   LinErr   LinRes
   1     5.4e-05   0.3500000     5.4e-05   46   34   34  1.1e-13  2.8e-15
   2     3.5e-05   0.3500000     3.5e-05   47   35   35  9.1e-14  2.8e-15
   3     2.3e-05   0.3500000     2.3e-05   48   36   36  1.2e-13  2.7e-15
Velocity u2, Pressure p
Iter      SolEst     Damping    Stepsize #Res #Jac #Sol   LinErr   LinRes
   1     0.00026   0.5000000     0.00026   24   12   12  5.8e-15  7.9e-15
Displacement field
Iter      SolEst     Damping    Stepsize #Res #Jac #Sol   LinErr   LinRes
   1      0.0028   0.5000000      0.0028   24   12   12  2.7e-14  1.5e-12
Spatial mesh displacement
Iter      SolEst     Damping    Stepsize #Res #Jac #Sol   LinErr   LinRes
   1     2.1e-08   0.5000000     2.1e-08   24   12   12  1.4e-15  1.9e-15
Solution error estimates for segregated groups
3.9e-05, 0.00013, 0.0014, 1.1e-08
```

```
Residual error estimates for segregated groups
0.013, 17, 1.2e+03, 0.21
Segregated solver iteration 13.
Merged variables
Iter      SolEst      Damping    Stepsize #Res #Jac #Sol   LinErr   LinRes
   1      1.5e-05   0.3500000     1.5e-05   50   37   37  6.5e-14  2.8e-15
   2      9.6e-06   0.3500000     9.6e-06   51   38   38    5e-14  2.7e-15
   3      6.2e-06   0.3500000     6.2e-06   52   39   39  9.4e-14  2.8e-15
Velocity u2, Pressure p
Iter      SolEst      Damping    Stepsize #Res #Jac #Sol   LinErr   LinRes
   1      0.00013   0.5000000     0.00013   26   13   13  2.8e-15    8e-15
Displacement field
Iter      SolEst      Damping    Stepsize #Res #Jac #Sol   LinErr   LinRes
   1       0.0015   0.5000000      0.0015   26   13   13  4.9e-14  1.5e-12
Spatial mesh displacement
Iter      SolEst      Damping    Stepsize #Res #Jac #Sol   LinErr   LinRes
   1      1.2e-08   0.5000000     1.2e-08   26   13   13  1.8e-15  1.8e-15
Solution error estimates for segregated groups
1.1e-05, 6.5e-05, 0.00076, 6.2e-09
Residual error estimates for segregated groups
0.0041, 8.3, 1.2e+03, 0.21
Solution time: 158 s. (2 minutes, 38 seconds)
Physical memory: 4.48 GB
Virtual memory: 25.81 GB
Ended at Jan 31, 2025, 5:04:39 PM.
----- Stationary Solver 1 in study1/Approach Curve Solver (sol1) -------------->
```

d=2475 (su7)

GENERAL

Description	Value
Solution	d=2475

LOG

```
<---- Compile Equations: Stationary in study1/Approach Curve Solver (sol1) -----
Started at Jan 31, 2025, 5:04:39 PM.
Parameter d = 2.475E-6 (m).
Geometry shape function: Linear serendipity
Number of vertex elements: 18
Number of boundary elements: 6319
Number of elements: 157541
Minimum element quality: 0.4812
Time: 9 s.
Physical memory: 2.74 GB
Virtual memory: 15.18 GB
Ended at Jan 31, 2025, 5:04:48 PM.
----- Compile Equations: Stationary in study1/Approach Curve Solver (sol1) ---->

<---- Dependent Variables 1 in study1/Approach Curve Solver (sol1) -------------
Started at Jan 31, 2025, 5:04:48 PM.
Solution time: 0 s.
Physical memory: 2.74 GB
Virtual memory: 15.18 GB
Ended at Jan 31, 2025, 5:04:48 PM.
----- Dependent Variables 1 in study1/Approach Curve Solver (sol1) ------------>

<---- Stationary Solver 1 in study1/Approach Curve Solver (sol1) ---------------
Started at Jan 31, 2025, 5:04:48 PM.
Segregated solver
Segregated Step 11 does not have any components solved for.
Number of degrees of freedom solved for: 801820 (plus 12061 internal DOFs).
Segregated solver iteration 1.
Merged variables
Nonsymmetric matrix found.
Scales for dependent variables:
Concentration (comp1.Cl): 1.4e+02
Concentration (comp1.K): 1.4e+02
Electric potential (comp1.V): 1.9e+02
Orthonormal null-space function used.
Iter      SolEst     Damping    Stepsize #Res #Jac #Sol   LinErr   LinRes
   1        0.83   0.3500000        0.83    2    1    1  3.6e-11    3e-15
   2        0.81   0.3500000        0.81    3    2    2  2.6e-11  2.7e-15
   3         0.8   0.3500000         0.8    4    3    3  2.1e-11  2.8e-15
Velocity u2, Pressure p
Nonsymmetric matrix found.
Scales for dependent variables:
Pressure (comp1.p): 1
Velocity field (spatial frame) (comp1.u2): 0.00019
Orthonormal null-space function used.
Iter      SolEst     Damping    Stepsize #Res #Jac #Sol   LinErr   LinRes
   1         1.1   0.5000000         1.1    2    1    1    2e-15  2.3e-15
Displacement field
Nonsymmetric matrix found.
Scales for dependent variables:
Auxiliary pressure (comp1.solid.lemm1.pw): 1
Displacement field (comp1.u): 2.7e-18
Orthonormal null-space function used.
Iter      SolEst     Damping    Stepsize #Res #Jac #Sol   LinErr   LinRes
   1         1.9   0.5000000         1.9    2    1    1  1.2e-13  1.5e-12
Spatial mesh displacement
```

```
Symmetric matrices found.
Scales for dependent variables:
Spatial mesh displacement (comp1.spatial.disp): 1.2e-09
Orthonormal null-space function used.
Iter      SolEst     Damping    Stepsize #Res #Jac #Sol   LinErr   LinRes
   1     5.6e-07   0.5000000     5.6e-07    2    1    1  1.6e-15  1.9e-15
Solution error estimates for segregated groups
1.4, 0.53, 0.95, 2.8e-07
Residual error estimates for segregated groups
8e+03, 8.3e+04, 2.2e+04, 0.16
Segregated solver iteration 2.
Merged variables
Scales for dependent variables:
Concentration (comp1.Cl): 1.4e+02
Concentration (comp1.K): 1.4e+02
Electric potential (comp1.V): 0.064
Iter      SolEst     Damping    Stepsize #Res #Jac #Sol   LinErr   LinRes
   1        0.79   0.3500000        0.79    6    4    4  1.2e-13  2.6e-15
   2        0.77   0.3500000        0.77    7    5    5  9.4e-14  2.7e-15
   3        0.76   0.3500000        0.76    8    6    6    2e-13  2.7e-15
Velocity u2, Pressure p
Scales for dependent variables:
Pressure (comp1.p): 3.5e+02
Velocity field (spatial frame) (comp1.u2): 0.00071
Iter      SolEst     Damping    Stepsize #Res #Jac #Sol   LinErr   LinRes
   1        0.36   0.5000000        0.36    4    2    2  3.3e-15  7.7e-15
Displacement field
Iter      SolEst     Damping    Stepsize #Res #Jac #Sol   LinErr   LinRes
   1        0.95   0.5000000        0.95    4    2    2  4.4e-14  1.5e-12
Spatial mesh displacement
Iter      SolEst     Damping    Stepsize #Res #Jac #Sol   LinErr   LinRes
   1     8.4e-07   0.5000000     8.4e-07    4    2    2  1.4e-15  1.8e-15
Solution error estimates for segregated groups
1.3, 0.18, 0.48, 4.2e-07
Residual error estimates for segregated groups
8.2e+02, 1.3e+04, 2.2e+04, 0.2
Segregated solver iteration 3.
Merged variables
Iter      SolEst     Damping    Stepsize #Res #Jac #Sol   LinErr   LinRes
   1        0.75   0.3500000        0.75   10    7    7  4.2e-14  2.6e-15
   2        0.74   0.3500000        0.74   11    8    8    8e-14  2.8e-15
   3        0.72   0.3500000        0.72   12    9    9    8e-14  2.7e-15
Velocity u2, Pressure p
Iter      SolEst     Damping    Stepsize #Res #Jac #Sol   LinErr   LinRes
   1        0.15   0.5000000        0.15    6    3    3  2.8e-15    8e-15
Displacement field
Iter      SolEst     Damping    Stepsize #Res #Jac #Sol   LinErr   LinRes
   1        0.52   0.5000000        0.52    6    3    3  1.5e-13  1.5e-12
Spatial mesh displacement
Iter      SolEst     Damping    Stepsize #Res #Jac #Sol   LinErr   LinRes
   1     8.4e-07   0.5000000     8.4e-07    6    3    3  1.5e-15  1.9e-15
Solution error estimates for segregated groups
1.2, 0.076, 0.26, 4.2e-07
Residual error estimates for segregated groups
2.3e+02, 7.3e+03, 1.6e+04, 0.2
Segregated solver iteration 4.
```

```
Merged variables
Iter      SolEst     Damping    Stepsize #Res #Jac #Sol   LinErr   LinRes
   1        0.71   0.3500000        0.71   14   10   10  7.5e-14  2.8e-15
   2        0.69   0.3500000        0.69   15   11   11  4.3e-14  2.8e-15
   3        0.61   0.3500000        0.61   16   12   12  9.5e-14  2.7e-15
Velocity u2, Pressure p
Iter      SolEst     Damping    Stepsize #Res #Jac #Sol   LinErr   LinRes
   1       0.071   0.5000000       0.071    8    4    4  3.1e-15  7.9e-15
Displacement field
Iter      SolEst     Damping    Stepsize #Res #Jac #Sol   LinErr   LinRes
   1        0.29   0.5000000        0.29    8    4    4  9.8e-14  1.5e-12
Spatial mesh displacement
Iter      SolEst     Damping    Stepsize #Res #Jac #Sol   LinErr   LinRes
   1       7e-07   0.5000000       7e-07    8    4    4  1.5e-15  1.9e-15
Solution error estimates for segregated groups
1, 0.036, 0.15, 3.5e-07
Residual error estimates for segregated groups
86, 3.9e+03, 1.1e+04, 0.2
Segregated solver iteration 5.
Merged variables
Iter      SolEst     Damping    Stepsize #Res #Jac #Sol   LinErr   LinRes
   1        0.42   0.3500000        0.42   18   13   13  6.9e-14  2.7e-15
   2        0.28   0.3500000        0.28   19   14   14    1e-13  2.8e-15
   3        0.19   0.3500000        0.19   20   15   15  1.2e-13  2.7e-15
Velocity u2, Pressure p
Iter      SolEst     Damping    Stepsize #Res #Jac #Sol   LinErr   LinRes
   1       0.035   0.5000000       0.035   10    5    5  2.4e-15    8e-15
Displacement field
Iter      SolEst     Damping    Stepsize #Res #Jac #Sol   LinErr   LinRes
   1        0.17   0.5000000        0.17   10    5    5    1e-13  1.5e-12
Spatial mesh displacement
Iter      SolEst     Damping    Stepsize #Res #Jac #Sol   LinErr   LinRes
   1     5.2e-07   0.5000000     5.2e-07   10    5    5  1.2e-15  1.9e-15
Solution error estimates for segregated groups
0.32, 0.017, 0.083, 2.6e-07
Residual error estimates for segregated groups
33, 2e+03, 6.9e+03, 0.22
Segregated solver iteration 6.
Merged variables
Iter      SolEst     Damping    Stepsize #Res #Jac #Sol   LinErr   LinRes
   1        0.12   0.3500000        0.12   22   16   16  9.5e-14  2.7e-15
   2        0.08   0.3500000        0.08   23   17   17  5.2e-14  2.8e-15
   3       0.052   0.3500000       0.052   24   18   18  5.1e-14  2.7e-15
Velocity u2, Pressure p
Iter      SolEst     Damping    Stepsize #Res #Jac #Sol   LinErr   LinRes
   1       0.017   0.5000000       0.017   12    6    6  2.2e-15  7.9e-15
Displacement field
Iter      SolEst     Damping    Stepsize #Res #Jac #Sol   LinErr   LinRes
   1       0.095   0.5000000       0.095   12    6    6  4.6e-14  1.5e-12
Spatial mesh displacement
Iter      SolEst     Damping    Stepsize #Res #Jac #Sol   LinErr   LinRes
   1     3.7e-07   0.5000000     3.7e-07   12    6    6  1.8e-15  1.9e-15
Solution error estimates for segregated groups
0.09, 0.0085, 0.048, 1.8e-07
Residual error estimates for segregated groups
12, 1e+03, 4.3e+03, 0.21
```

```
Segregated solver iteration 7.
Merged variables
Iter      SolEst     Damping    Stepsize #Res #Jac #Sol   LinErr   LinRes
   1       0.034   0.3500000       0.034   26   19   19  6.4e-14  2.7e-15
   2       0.022   0.3500000       0.022   27   20   20  1.1e-13  2.7e-15
   3       0.014   0.3500000       0.014   28   21   21  5.6e-14  2.7e-15
Velocity u2, Pressure p
Iter      SolEst     Damping    Stepsize #Res #Jac #Sol   LinErr   LinRes
   1      0.0084   0.5000000      0.0084   14    7    7    4e-15  7.9e-15
Displacement field
Iter      SolEst     Damping    Stepsize #Res #Jac #Sol   LinErr   LinRes
   1       0.054   0.5000000       0.054   14    7    7  1.4e-13  1.5e-12
Spatial mesh displacement
Iter      SolEst     Damping    Stepsize #Res #Jac #Sol   LinErr   LinRes
   1     2.4e-07   0.5000000     2.4e-07   14    7    7  1.6e-15  1.8e-15
Solution error estimates for segregated groups
0.025, 0.0042, 0.027, 1.2e-07
Residual error estimates for segregated groups
4.2, 5.1e+02, 2.7e+03, 0.2
Segregated solver iteration 8.
Merged variables
Iter      SolEst     Damping    Stepsize #Res #Jac #Sol   LinErr   LinRes
   1      0.0094   0.3500000      0.0094   30   22   22  6.1e-14  2.7e-15
   2      0.0061   0.3500000      0.0061   31   23   23  6.5e-14  2.6e-15
   3       0.004   0.3500000       0.004   32   24   24  9.5e-14  2.7e-15
Velocity u2, Pressure p
Iter      SolEst     Damping    Stepsize #Res #Jac #Sol   LinErr   LinRes
   1      0.0042   0.5000000      0.0042   16    8    8  3.3e-15  7.9e-15
Displacement field
Iter      SolEst     Damping    Stepsize #Res #Jac #Sol   LinErr   LinRes
   1        0.03   0.5000000        0.03   16    8    8  1.9e-13  1.5e-12
Spatial mesh displacement
Iter      SolEst     Damping    Stepsize #Res #Jac #Sol   LinErr   LinRes
   1     1.6e-07   0.5000000     1.6e-07   16    8    8  1.6e-15  1.9e-15
Solution error estimates for segregated groups
0.0068, 0.0021, 0.015, 7.9e-08
Residual error estimates for segregated groups
1.4, 2.5e+02, 1.8e+03, 0.2
Segregated solver iteration 9.
Merged variables
Iter      SolEst     Damping    Stepsize #Res #Jac #Sol   LinErr   LinRes
   1      0.0026   0.3500000      0.0026   34   25   25  9.7e-14  2.7e-15
   2      0.0017   0.3500000      0.0017   35   26   26  9.8e-14  2.8e-15
   3      0.0011   0.3500000      0.0011   36   27   27  6.4e-14  2.8e-15
Velocity u2, Pressure p
Iter      SolEst     Damping    Stepsize #Res #Jac #Sol   LinErr   LinRes
   1      0.0021   0.5000000      0.0021   18    9    9  2.8e-15    8e-15
Displacement field
Iter      SolEst     Damping    Stepsize #Res #Jac #Sol   LinErr   LinRes
   1       0.017   0.5000000       0.017   18    9    9  2.3e-13  1.5e-12
Spatial mesh displacement
Iter      SolEst     Damping    Stepsize #Res #Jac #Sol   LinErr   LinRes
   1     9.8e-08   0.5000000     9.8e-08   18    9    9  1.5e-15  1.9e-15
Solution error estimates for segregated groups
0.0019, 0.001, 0.0085, 4.9e-08
Residual error estimates for segregated groups
```

```
0.45, 1.3e+02, 1.4e+03, 0.21
Segregated solver iteration 10.
Merged variables
Iter      SolEst     Damping    Stepsize #Res #Jac #Sol   LinErr   LinRes
   1     0.00071   0.3500000     0.00071   38   28   28  8.5e-14  2.6e-15
   2     0.00046   0.3500000     0.00046   39   29   29  7.2e-14  2.8e-15
   3      0.0003   0.3500000      0.0003   40   30   30  5.6e-14  2.8e-15
Velocity u2, Pressure p
Iter      SolEst     Damping    Stepsize #Res #Jac #Sol   LinErr   LinRes
   1       0.001   0.5000000       0.001   20   10   10  2.1e-15  7.8e-15
Displacement field
Iter      SolEst     Damping    Stepsize #Res #Jac #Sol   LinErr   LinRes
   1      0.0093   0.5000000      0.0093   20   10   10  5.6e-14  1.5e-12
Spatial mesh displacement
Iter      SolEst     Damping    Stepsize #Res #Jac #Sol   LinErr   LinRes
   1       6e-08   0.5000000       6e-08   20   10   10  1.6e-15  1.8e-15
Solution error estimates for segregated groups
0.00051, 0.00052, 0.0047, 3e-08
Residual error estimates for segregated groups
0.14, 63, 1.2e+03, 0.19
Segregated solver iteration 11.
Merged variables
Iter      SolEst     Damping    Stepsize #Res #Jac #Sol   LinErr   LinRes
   1     0.00019   0.3500000     0.00019   42   31   31    1e-13  2.7e-15
   2     0.00013   0.3500000     0.00013   43   32   32  9.6e-14  2.7e-15
   3     8.2e-05   0.3500000     8.2e-05   44   33   33  7.4e-14  2.6e-15
Velocity u2, Pressure p
Iter      SolEst     Damping    Stepsize #Res #Jac #Sol   LinErr   LinRes
   1     0.00052   0.5000000     0.00052   22   11   11  2.8e-15  7.9e-15
Displacement field
Iter      SolEst     Damping    Stepsize #Res #Jac #Sol   LinErr   LinRes
   1      0.0051   0.5000000      0.0051   22   11   11  3.9e-14  1.5e-12
Spatial mesh displacement
Iter      SolEst     Damping    Stepsize #Res #Jac #Sol   LinErr   LinRes
   1     3.6e-08   0.5000000     3.6e-08   22   11   11  1.6e-15  1.9e-15
Solution error estimates for segregated groups
0.00014, 0.00026, 0.0026, 1.8e-08
Residual error estimates for segregated groups
0.044, 32, 1.2e+03, 0.2
Segregated solver iteration 12.
Merged variables
Iter      SolEst     Damping    Stepsize #Res #Jac #Sol   LinErr   LinRes
   1     5.3e-05   0.3500000     5.3e-05   46   34   34  8.7e-14  2.8e-15
   2     3.5e-05   0.3500000     3.5e-05   47   35   35  5.9e-14  2.8e-15
   3     2.3e-05   0.3500000     2.3e-05   48   36   36  8.1e-14  2.7e-15
Velocity u2, Pressure p
Iter      SolEst     Damping    Stepsize #Res #Jac #Sol   LinErr   LinRes
   1     0.00026   0.5000000     0.00026   24   12   12  2.8e-15  7.8e-15
Displacement field
Iter      SolEst     Damping    Stepsize #Res #Jac #Sol   LinErr   LinRes
   1      0.0028   0.5000000      0.0028   24   12   12    1e-13  1.5e-12
Spatial mesh displacement
Iter      SolEst     Damping    Stepsize #Res #Jac #Sol   LinErr   LinRes
   1     2.1e-08   0.5000000     2.1e-08   24   12   12  1.5e-15  1.8e-15
Solution error estimates for segregated groups
3.9e-05, 0.00013, 0.0014, 1.1e-08
```

```
Residual error estimates for segregated groups
0.013, 16, 1.2e+03, 0.2
Segregated solver iteration 13.
Merged variables
Iter      SolEst     Damping    Stepsize #Res #Jac #Sol   LinErr   LinRes
   1     1.5e-05   0.3500000     1.5e-05   50   37   37  4.7e-14  2.7e-15
   2     9.5e-06   0.3500000     9.5e-06   51   38   38  7.8e-14  2.7e-15
   3     6.2e-06   0.3500000     6.2e-06   52   39   39  5.3e-14  2.7e-15
Velocity u2, Pressure p
Iter      SolEst     Damping    Stepsize #Res #Jac #Sol   LinErr   LinRes
   1     0.00013   0.5000000     0.00013   26   13   13  4.3e-15  7.9e-15
Displacement field
Iter      SolEst     Damping    Stepsize #Res #Jac #Sol   LinErr   LinRes
   1      0.0015   0.5000000      0.0015   26   13   13  1.5e-13  1.5e-12
Spatial mesh displacement
Iter      SolEst     Damping    Stepsize #Res #Jac #Sol   LinErr   LinRes
   1     1.2e-08   0.5000000     1.2e-08   26   13   13  1.4e-15  1.8e-15
Solution error estimates for segregated groups
1.1e-05, 6.5e-05, 0.00076, 6.2e-09
Residual error estimates for segregated groups
0.0041, 7.9, 1.2e+03, 0.2
Solution time: 158 s. (2 minutes, 38 seconds)
Physical memory: 4.48 GB
Virtual memory: 25.8 GB
Ended at Jan 31, 2025, 5:07:26 PM.
----- Stationary Solver 1 in study1/Approach Curve Solver (sol1) -------------->
```

d=2470 (su8)

GENERAL

Description	Value
Solution	d=2470

LOG

```
<---- Compile Equations: Stationary in study1/Approach Curve Solver (sol1) -----
Started at Jan 31, 2025, 5:07:26 PM.
Parameter d = 2.47E-6 (m).
Geometry shape function: Linear serendipity
Number of vertex elements: 18
Number of boundary elements: 6316
Number of elements: 157714
Minimum element quality: 0.4472
Time: 9 s.
Physical memory: 2.81 GB
Virtual memory: 15.24 GB
Ended at Jan 31, 2025, 5:07:35 PM.
----- Compile Equations: Stationary in study1/Approach Curve Solver (sol1) ---->

<---- Dependent Variables 1 in study1/Approach Curve Solver (sol1) -------------
Started at Jan 31, 2025, 5:07:35 PM.
Solution time: 0 s.
Physical memory: 2.81 GB
Virtual memory: 15.24 GB
Ended at Jan 31, 2025, 5:07:35 PM.
----- Dependent Variables 1 in study1/Approach Curve Solver (sol1) ------------>

<---- Stationary Solver 1 in study1/Approach Curve Solver (sol1) ---------------
Started at Jan 31, 2025, 5:07:35 PM.
Segregated solver
Segregated Step 11 does not have any components solved for.
Number of degrees of freedom solved for: 802718 (plus 12055 internal DOFs).
Segregated solver iteration 1.
Merged variables
Nonsymmetric matrix found.
Scales for dependent variables:
Concentration (comp1.Cl): 1.4e+02
Concentration (comp1.K): 1.4e+02
Electric potential (comp1.V): 1.9e+02
Orthonormal null-space function used.
Iter      SolEst     Damping    Stepsize #Res #Jac #Sol   LinErr   LinRes
   1        0.83   0.3500000        0.83    2    1    1  4.8e-11  2.9e-15
   2        0.81   0.3500000        0.81    3    2    2  4.5e-11  2.7e-15
   3         0.8   0.3500000         0.8    4    3    3  5.2e-11  2.7e-15
Velocity u2, Pressure p
Nonsymmetric matrix found.
Scales for dependent variables:
Pressure (comp1.p): 1
Velocity field (spatial frame) (comp1.u2): 0.00019
Orthonormal null-space function used.
Iter      SolEst     Damping    Stepsize #Res #Jac #Sol   LinErr   LinRes
   1         1.1   0.5000000         1.1    2    1    1  1.6e-15  2.3e-15
Displacement field
Nonsymmetric matrix found.
Scales for dependent variables:
Auxiliary pressure (comp1.solid.lemm1.pw): 1
Displacement field (comp1.u): 2.8e-18
Orthonormal null-space function used.
Iter      SolEst     Damping    Stepsize #Res #Jac #Sol   LinErr   LinRes
   1         1.9   0.5000000         1.9    2    1    1  8.4e-14  1.5e-12
Spatial mesh displacement
```

```
Symmetric matrices found.
Scales for dependent variables:
Spatial mesh displacement (comp1.spatial.disp): 1.2e-09
Orthonormal null-space function used.
Iter      SolEst     Damping    Stepsize #Res #Jac #Sol   LinErr   LinRes
   1     5.6e-07   0.5000000     5.6e-07    2    1    1  1.8e-15  1.9e-15
Solution error estimates for segregated groups
1.4, 0.53, 0.95, 2.8e-07
Residual error estimates for segregated groups
8e+03, 8.3e+04, 2.2e+04, 0.18
Segregated solver iteration 2.
Merged variables
Scales for dependent variables:
Concentration (comp1.Cl): 1.4e+02
Concentration (comp1.K): 1.4e+02
Electric potential (comp1.V): 0.064
Iter      SolEst     Damping    Stepsize #Res #Jac #Sol   LinErr   LinRes
   1        0.79   0.3500000        0.79    6    4    4  8.4e-14  4.2e-15
   2        0.77   0.3500000        0.77    7    5    5  7.2e-14  3.1e-15
   3        0.76   0.3500000        0.76    8    6    6  6.1e-14  3.3e-15
Velocity u2, Pressure p
Scales for dependent variables:
Pressure (comp1.p): 3.5e+02
Velocity field (spatial frame) (comp1.u2): 0.0007
Iter      SolEst     Damping    Stepsize #Res #Jac #Sol   LinErr   LinRes
   1        0.36   0.5000000        0.36    4    2    2  3.5e-15  7.8e-15
Displacement field
Iter      SolEst     Damping    Stepsize #Res #Jac #Sol   LinErr   LinRes
   1        0.95   0.5000000        0.95    4    2    2    1e-13  1.5e-12
Spatial mesh displacement
Iter      SolEst     Damping    Stepsize #Res #Jac #Sol   LinErr   LinRes
   1     8.4e-07   0.5000000     8.4e-07    4    2    2  1.7e-15  1.8e-15
Solution error estimates for segregated groups
1.3, 0.18, 0.48, 4.2e-07
Residual error estimates for segregated groups
8.2e+02, 1.4e+04, 2.2e+04, 0.18
Segregated solver iteration 3.
Merged variables
Iter      SolEst     Damping    Stepsize #Res #Jac #Sol   LinErr   LinRes
   1        0.75   0.3500000        0.75   10    7    7  7.8e-14    5e-15
   2        0.74   0.3500000        0.74   11    8    8    9e-14  3.7e-15
   3        0.72   0.3500000        0.72   12    9    9    7e-14  5.2e-15
Velocity u2, Pressure p
Iter      SolEst     Damping    Stepsize #Res #Jac #Sol   LinErr   LinRes
   1        0.15   0.5000000        0.15    6    3    3    4e-15  7.7e-15
Displacement field
Iter      SolEst     Damping    Stepsize #Res #Jac #Sol   LinErr   LinRes
   1        0.52   0.5000000        0.52    6    3    3  3.5e-14  1.5e-12
Spatial mesh displacement
Iter      SolEst     Damping    Stepsize #Res #Jac #Sol   LinErr   LinRes
   1     8.4e-07   0.5000000     8.4e-07    6    3    3  1.5e-15  1.8e-15
Solution error estimates for segregated groups
1.2, 0.076, 0.26, 4.2e-07
Residual error estimates for segregated groups
2.3e+02, 7.6e+03, 1.6e+04, 0.2
Segregated solver iteration 4.
```

```
Merged variables
Iter      SolEst     Damping    Stepsize #Res #Jac #Sol   LinErr   LinRes
   1        0.71   0.3500000        0.71   14   10   10  6.1e-14  4.7e-15
   2        0.69   0.3500000        0.69   15   11   11  8.9e-14  6.9e-15
   3        0.61   0.3500000        0.61   16   12   12  1.6e-13  3.7e-15
Velocity u2, Pressure p
Iter      SolEst     Damping    Stepsize #Res #Jac #Sol   LinErr   LinRes
   1       0.071   0.5000000       0.071    8    4    4  3.3e-15  7.8e-15
Displacement field
Iter      SolEst     Damping    Stepsize #Res #Jac #Sol   LinErr   LinRes
   1        0.29   0.5000000        0.29    8    4    4  6.2e-14  1.5e-12
Spatial mesh displacement
Iter      SolEst     Damping    Stepsize #Res #Jac #Sol   LinErr   LinRes
   1       7e-07   0.5000000       7e-07    8    4    4  1.8e-15  1.8e-15
Solution error estimates for segregated groups
1, 0.036, 0.15, 3.5e-07
Residual error estimates for segregated groups
86, 4e+03, 1.1e+04, 0.21
Segregated solver iteration 5.
Merged variables
Iter      SolEst     Damping    Stepsize #Res #Jac #Sol   LinErr   LinRes
   1        0.42   0.3500000        0.42   18   13   13  9.3e-14  3.2e-15
   2        0.28   0.3500000        0.28   19   14   14  8.8e-14    4e-15
   3        0.19   0.3500000        0.19   20   15   15  4.8e-14  3.4e-15
Velocity u2, Pressure p
Iter      SolEst     Damping    Stepsize #Res #Jac #Sol   LinErr   LinRes
   1       0.034   0.5000000       0.034   10    5    5  2.7e-15  7.9e-15
Displacement field
Iter      SolEst     Damping    Stepsize #Res #Jac #Sol   LinErr   LinRes
   1        0.17   0.5000000        0.17   10    5    5  7.6e-14  1.5e-12
Spatial mesh displacement
Iter      SolEst     Damping    Stepsize #Res #Jac #Sol   LinErr   LinRes
   1     5.3e-07   0.5000000     5.3e-07   10    5    5  1.6e-15  1.9e-15
Solution error estimates for segregated groups
0.32, 0.017, 0.084, 2.6e-07
Residual error estimates for segregated groups
33, 2.1e+03, 6.9e+03, 0.2
Segregated solver iteration 6.
Merged variables
Iter      SolEst     Damping    Stepsize #Res #Jac #Sol   LinErr   LinRes
   1        0.12   0.3500000        0.12   22   16   16  5.1e-14  4.9e-15
   2        0.08   0.3500000        0.08   23   17   17  1.2e-13  6.7e-15
   3       0.052   0.3500000       0.052   24   18   18  8.8e-14  3.1e-15
Velocity u2, Pressure p
Iter      SolEst     Damping    Stepsize #Res #Jac #Sol   LinErr   LinRes
   1       0.017   0.5000000       0.017   12    6    6  2.6e-15  7.9e-15
Displacement field
Iter      SolEst     Damping    Stepsize #Res #Jac #Sol   LinErr   LinRes
   1       0.095   0.5000000       0.095   12    6    6  6.8e-14  1.5e-12
Spatial mesh displacement
Iter      SolEst     Damping    Stepsize #Res #Jac #Sol   LinErr   LinRes
   1     3.7e-07   0.5000000     3.7e-07   12    6    6  1.5e-15  1.9e-15
Solution error estimates for segregated groups
0.09, 0.0085, 0.048, 1.8e-07
Residual error estimates for segregated groups
12, 1e+03, 4.3e+03, 0.21
```

```
Segregated solver iteration 7.
Merged variables
Iter      SolEst     Damping    Stepsize #Res #Jac #Sol   LinErr   LinRes
   1       0.034   0.3500000       0.034   26   19   19  9.5e-14  3.1e-15
   2       0.022   0.3500000       0.022   27   20   20  5.8e-14  1.1e-14
   3       0.014   0.3500000       0.014   28   21   21  6.9e-14  8.4e-15
Velocity u2, Pressure p
Iter      SolEst     Damping    Stepsize #Res #Jac #Sol   LinErr   LinRes
   1      0.0084   0.5000000      0.0084   14    7    7  2.3e-15  7.8e-15
Displacement field
Iter      SolEst     Damping    Stepsize #Res #Jac #Sol   LinErr   LinRes
   1       0.054   0.5000000       0.054   14    7    7  6.7e-14  1.5e-12
Spatial mesh displacement
Iter      SolEst     Damping    Stepsize #Res #Jac #Sol   LinErr   LinRes
   1     2.5e-07   0.5000000     2.5e-07   14    7    7  1.6e-15  1.9e-15
Solution error estimates for segregated groups
0.025, 0.0042, 0.027, 1.2e-07
Residual error estimates for segregated groups
4.2, 5.3e+02, 2.7e+03, 0.19
Segregated solver iteration 8.
Merged variables
Iter      SolEst     Damping    Stepsize #Res #Jac #Sol   LinErr   LinRes
   1      0.0094   0.3500000      0.0094   30   22   22  1.1e-13  4.2e-15
   2      0.0061   0.3500000      0.0061   31   23   23  7.8e-14  3.2e-15
   3       0.004   0.3500000       0.004   32   24   24  7.3e-14  3.6e-15
Velocity u2, Pressure p
Iter      SolEst     Damping    Stepsize #Res #Jac #Sol   LinErr   LinRes
   1      0.0042   0.5000000      0.0042   16    8    8  2.6e-15  7.8e-15
Displacement field
Iter      SolEst     Damping    Stepsize #Res #Jac #Sol   LinErr   LinRes
   1        0.03   0.5000000        0.03   16    8    8    4e-14  1.5e-12
Spatial mesh displacement
Iter      SolEst     Damping    Stepsize #Res #Jac #Sol   LinErr   LinRes
   1     1.6e-07   0.5000000     1.6e-07   16    8    8  1.7e-15  1.9e-15
Solution error estimates for segregated groups
0.0068, 0.0021, 0.015, 7.9e-08
Residual error estimates for segregated groups
1.4, 2.6e+02, 1.8e+03, 0.2
Segregated solver iteration 9.
Merged variables
Iter      SolEst     Damping    Stepsize #Res #Jac #Sol   LinErr   LinRes
   1      0.0026   0.3500000      0.0026   34   25   25  1.2e-13  3.4e-15
   2      0.0017   0.3500000      0.0017   35   26   26  1.5e-13  6.6e-15
   3      0.0011   0.3500000      0.0011   36   27   27  5.6e-14  3.2e-15
Velocity u2, Pressure p
Iter      SolEst     Damping    Stepsize #Res #Jac #Sol   LinErr   LinRes
   1      0.0021   0.5000000      0.0021   18    9    9  5.7e-15  7.8e-15
Displacement field
Iter      SolEst     Damping    Stepsize #Res #Jac #Sol   LinErr   LinRes
   1       0.017   0.5000000       0.017   18    9    9  6.3e-14  1.5e-12
Spatial mesh displacement
Iter      SolEst     Damping    Stepsize #Res #Jac #Sol   LinErr   LinRes
   1     9.9e-08   0.5000000     9.9e-08   18    9    9  1.4e-15  1.8e-15
Solution error estimates for segregated groups
0.0019, 0.001, 0.0085, 4.9e-08
Residual error estimates for segregated groups
```

```
0.45, 1.3e+02, 1.4e+03, 0.2
Segregated solver iteration 10.
Merged variables
Iter      SolEst     Damping    Stepsize #Res #Jac #Sol   LinErr   LinRes
   1     0.00071   0.3500000     0.00071   38   28   28  9.6e-14  7.3e-15
   2     0.00046   0.3500000     0.00046   39   29   29  8.1e-14  3.1e-15
   3      0.0003   0.3500000      0.0003   40   30   30  9.8e-14  3.3e-15
Velocity u2, Pressure p
Iter      SolEst     Damping    Stepsize #Res #Jac #Sol   LinErr   LinRes
   1       0.001   0.5000000       0.001   20   10   10  3.2e-15  7.8e-15
Displacement field
Iter      SolEst     Damping    Stepsize #Res #Jac #Sol   LinErr   LinRes
   1      0.0094   0.5000000      0.0094   20   10   10  3.9e-14  1.5e-12
Spatial mesh displacement
Iter      SolEst     Damping    Stepsize #Res #Jac #Sol   LinErr   LinRes
   1       6e-08   0.5000000       6e-08   20   10   10  1.5e-15  1.9e-15
Solution error estimates for segregated groups
0.00052, 0.00052, 0.0047, 3e-08
Residual error estimates for segregated groups
0.14, 66, 1.3e+03, 0.21
Segregated solver iteration 11.
Merged variables
Iter      SolEst     Damping    Stepsize #Res #Jac #Sol   LinErr   LinRes
   1      0.0002   0.3500000      0.0002   42   31   31  8.5e-14  6.1e-15
   2     0.00013   0.3500000     0.00013   43   32   32  5.3e-14  3.4e-15
   3     8.2e-05   0.3500000     8.2e-05   44   33   33  8.8e-14  3.1e-15
Velocity u2, Pressure p
Iter      SolEst     Damping    Stepsize #Res #Jac #Sol   LinErr   LinRes
   1     0.00052   0.5000000     0.00052   22   11   11  3.7e-15  7.9e-15
Displacement field
Iter      SolEst     Damping    Stepsize #Res #Jac #Sol   LinErr   LinRes
   1      0.0051   0.5000000      0.0051   22   11   11  4.4e-14  1.5e-12
Spatial mesh displacement
Iter      SolEst     Damping    Stepsize #Res #Jac #Sol   LinErr   LinRes
   1     3.6e-08   0.5000000     3.6e-08   22   11   11  1.5e-15  1.9e-15
Solution error estimates for segregated groups
0.00014, 0.00026, 0.0026, 1.8e-08
Residual error estimates for segregated groups
0.044, 33, 1.2e+03, 0.21
Segregated solver iteration 12.
Merged variables
Iter      SolEst     Damping    Stepsize #Res #Jac #Sol   LinErr   LinRes
   1     5.4e-05   0.3500000     5.4e-05   46   34   34  6.5e-14  5.9e-15
   2     3.5e-05   0.3500000     3.5e-05   47   35   35    7e-14  8.3e-15
   3     2.3e-05   0.3500000     2.3e-05   48   36   36  8.5e-14  6.1e-15
Velocity u2, Pressure p
Iter      SolEst     Damping    Stepsize #Res #Jac #Sol   LinErr   LinRes
   1     0.00026   0.5000000     0.00026   24   12   12  3.5e-15  7.7e-15
Displacement field
Iter      SolEst     Damping    Stepsize #Res #Jac #Sol   LinErr   LinRes
   1      0.0028   0.5000000      0.0028   24   12   12  5.3e-14  1.5e-12
Spatial mesh displacement
Iter      SolEst     Damping    Stepsize #Res #Jac #Sol   LinErr   LinRes
   1     2.1e-08   0.5000000     2.1e-08   24   12   12  1.5e-15  1.9e-15
Solution error estimates for segregated groups
3.9e-05, 0.00013, 0.0014, 1.1e-08
```

```
Residual error estimates for segregated groups
0.013, 17, 1.2e+03, 0.21
Segregated solver iteration 13.
Merged variables
Iter      SolEst     Damping    Stepsize #Res #Jac #Sol   LinErr   LinRes
   1     1.5e-05   0.3500000     1.5e-05   50   37   37  1.3e-13  5.3e-15
   2     9.6e-06   0.3500000     9.6e-06   51   38   38  1.4e-13    3e-15
   3     6.2e-06   0.3500000     6.2e-06   52   39   39  8.5e-14  6.6e-15
Velocity u2, Pressure p
Iter      SolEst     Damping    Stepsize #Res #Jac #Sol   LinErr   LinRes
   1     0.00013   0.5000000     0.00013   26   13   13  2.1e-15  7.9e-15
Displacement field
Iter      SolEst     Damping    Stepsize #Res #Jac #Sol   LinErr   LinRes
   1      0.0015   0.5000000      0.0015   26   13   13  1.4e-13  1.5e-12
Spatial mesh displacement
Iter      SolEst     Damping    Stepsize #Res #Jac #Sol   LinErr   LinRes
   1     1.2e-08   0.5000000     1.2e-08   26   13   13  1.4e-15  1.8e-15
Solution error estimates for segregated groups
1.1e-05, 6.5e-05, 0.00076, 6.2e-09
Residual error estimates for segregated groups
0.0041, 8.3, 1.2e+03, 0.2
Solution time: 157 s. (2 minutes, 37 seconds)
Physical memory: 4.52 GB
Virtual memory: 25.79 GB
Ended at Jan 31, 2025, 5:10:13 PM.
----- Stationary Solver 1 in study1/Approach Curve Solver (sol1) -------------->
```

d=2465 (su9)

GENERAL

Description	Value
Solution	d=2465

LOG

```
<---- Compile Equations: Stationary in study1/Approach Curve Solver (sol1) -----
Started at Jan 31, 2025, 5:10:13 PM.
Parameter d = 2.465E-6 (m).
Geometry shape function: Linear serendipity
Number of vertex elements: 18
Number of boundary elements: 6313
Number of elements: 157603
Minimum element quality: 0.4861
Time: 9 s.
Physical memory: 2.76 GB
Virtual memory: 15.18 GB
Ended at Jan 31, 2025, 5:10:22 PM.
----- Compile Equations: Stationary in study1/Approach Curve Solver (sol1) ---->

<---- Dependent Variables 1 in study1/Approach Curve Solver (sol1) -------------
Started at Jan 31, 2025, 5:10:22 PM.
Solution time: 0 s.
Physical memory: 2.76 GB
Virtual memory: 15.18 GB
Ended at Jan 31, 2025, 5:10:22 PM.
----- Dependent Variables 1 in study1/Approach Curve Solver (sol1) ------------>

<---- Stationary Solver 1 in study1/Approach Curve Solver (sol1) ---------------
Started at Jan 31, 2025, 5:10:22 PM.
Segregated solver
Segregated Step 11 does not have any components solved for.
Number of degrees of freedom solved for: 802134 (plus 12049 internal DOFs).
Segregated solver iteration 1.
Merged variables
Nonsymmetric matrix found.
Scales for dependent variables:
Concentration (comp1.Cl): 1.4e+02
Concentration (comp1.K): 1.4e+02
Electric potential (comp1.V): 1.9e+02
Orthonormal null-space function used.
Iter      SolEst     Damping    Stepsize #Res #Jac #Sol   LinErr   LinRes
   1        0.83   0.3500000        0.83    2    1    1  1.8e-11  2.8e-15
   2        0.81   0.3500000        0.81    3    2    2  2.2e-11  2.8e-15
   3         0.8   0.3500000         0.8    4    3    3  2.9e-11  2.8e-15
Velocity u2, Pressure p
Nonsymmetric matrix found.
Scales for dependent variables:
Pressure (comp1.p): 1
Velocity field (spatial frame) (comp1.u2): 0.00019
Orthonormal null-space function used.
Iter      SolEst     Damping    Stepsize #Res #Jac #Sol   LinErr   LinRes
   1         1.1   0.5000000         1.1    2    1    1  1.6e-15  2.3e-15
Displacement field
Nonsymmetric matrix found.
Scales for dependent variables:
Auxiliary pressure (comp1.solid.lemm1.pw): 1
Displacement field (comp1.u): 2.7e-18
Orthonormal null-space function used.
Iter      SolEst     Damping    Stepsize #Res #Jac #Sol   LinErr   LinRes
   1         1.9   0.5000000         1.9    2    1    1  6.2e-14  1.5e-12
Spatial mesh displacement
```

```
Symmetric matrices found.
Scales for dependent variables:
Spatial mesh displacement (comp1.spatial.disp): 1.2e-09
Orthonormal null-space function used.
Iter      SolEst     Damping    Stepsize #Res #Jac #Sol   LinErr   LinRes
   1     5.6e-07   0.5000000     5.6e-07    2    1    1  1.5e-15  1.8e-15
Solution error estimates for segregated groups
1.4, 0.54, 0.95, 2.8e-07
Residual error estimates for segregated groups
8e+03, 8.3e+04, 2.2e+04, 0.18
Segregated solver iteration 2.
Merged variables
Scales for dependent variables:
Concentration (comp1.Cl): 1.4e+02
Concentration (comp1.K): 1.4e+02
Electric potential (comp1.V): 0.064
Iter      SolEst     Damping    Stepsize #Res #Jac #Sol   LinErr   LinRes
   1        0.79   0.3500000        0.79    6    4    4  5.6e-14  2.7e-15
   2        0.77   0.3500000        0.77    7    5    5  4.6e-14  2.8e-15
   3        0.76   0.3500000        0.76    8    6    6  9.2e-14  2.7e-15
Velocity u2, Pressure p
Scales for dependent variables:
Pressure (comp1.p): 3.5e+02
Velocity field (spatial frame) (comp1.u2): 0.00071
Iter      SolEst     Damping    Stepsize #Res #Jac #Sol   LinErr   LinRes
   1        0.36   0.5000000        0.36    4    2    2  3.3e-15  7.9e-15
Displacement field
Iter      SolEst     Damping    Stepsize #Res #Jac #Sol   LinErr   LinRes
   1        0.95   0.5000000        0.95    4    2    2  3.8e-14  1.5e-12
Spatial mesh displacement
Iter      SolEst     Damping    Stepsize #Res #Jac #Sol   LinErr   LinRes
   1     8.4e-07   0.5000000     8.4e-07    4    2    2  1.4e-15  1.8e-15
Solution error estimates for segregated groups
1.3, 0.18, 0.48, 4.2e-07
Residual error estimates for segregated groups
8.2e+02, 1.4e+04, 2.2e+04, 0.19
Segregated solver iteration 3.
Merged variables
Iter      SolEst     Damping    Stepsize #Res #Jac #Sol   LinErr   LinRes
   1        0.75   0.3500000        0.75   10    7    7  4.3e-14  2.7e-15
   2        0.74   0.3500000        0.74   11    8    8  6.7e-14  2.7e-15
   3        0.72   0.3500000        0.72   12    9    9  7.2e-14  2.7e-15
Velocity u2, Pressure p
Iter      SolEst     Damping    Stepsize #Res #Jac #Sol   LinErr   LinRes
   1        0.15   0.5000000        0.15    6    3    3  2.8e-15  7.9e-15
Displacement field
Iter      SolEst     Damping    Stepsize #Res #Jac #Sol   LinErr   LinRes
   1        0.52   0.5000000        0.52    6    3    3  7.1e-14  1.5e-12
Spatial mesh displacement
Iter      SolEst     Damping    Stepsize #Res #Jac #Sol   LinErr   LinRes
   1     8.4e-07   0.5000000     8.4e-07    6    3    3  1.6e-15  1.8e-15
Solution error estimates for segregated groups
1.2, 0.076, 0.26, 4.2e-07
Residual error estimates for segregated groups
2.3e+02, 7.6e+03, 1.6e+04, 0.21
Segregated solver iteration 4.
```

```
Merged variables
Iter      SolEst     Damping    Stepsize #Res #Jac #Sol   LinErr   LinRes
   1        0.71   0.3500000        0.71   14   10   10  4.5e-14  2.8e-15
   2        0.69   0.3500000        0.69   15   11   11    1e-13  2.9e-15
   3        0.61   0.3500000        0.61   16   12   12    1e-13  2.8e-15
Velocity u2, Pressure p
Iter      SolEst     Damping    Stepsize #Res #Jac #Sol   LinErr   LinRes
   1       0.071   0.5000000       0.071    8    4    4  4.1e-15  7.9e-15
Displacement field
Iter      SolEst     Damping    Stepsize #Res #Jac #Sol   LinErr   LinRes
   1        0.29   0.5000000        0.29    8    4    4  1.3e-13  1.5e-12
Spatial mesh displacement
Iter      SolEst     Damping    Stepsize #Res #Jac #Sol   LinErr   LinRes
   1       7e-07   0.5000000       7e-07    8    4    4  1.6e-15  1.8e-15
Solution error estimates for segregated groups
1, 0.036, 0.15, 3.5e-07
Residual error estimates for segregated groups
86, 4e+03, 1.1e+04, 0.2
Segregated solver iteration 5.
Merged variables
Iter      SolEst     Damping    Stepsize #Res #Jac #Sol   LinErr   LinRes
   1        0.42   0.3500000        0.42   18   13   13  8.4e-14    3e-15
   2        0.28   0.3500000        0.28   19   14   14  7.6e-14  2.9e-15
   3        0.19   0.3500000        0.19   20   15   15  5.3e-14  2.7e-15
Velocity u2, Pressure p
Iter      SolEst     Damping    Stepsize #Res #Jac #Sol   LinErr   LinRes
   1       0.035   0.5000000       0.035   10    5    5  2.8e-15  7.8e-15
Displacement field
Iter      SolEst     Damping    Stepsize #Res #Jac #Sol   LinErr   LinRes
   1        0.17   0.5000000        0.17   10    5    5  4.5e-14  1.5e-12
Spatial mesh displacement
Iter      SolEst     Damping    Stepsize #Res #Jac #Sol   LinErr   LinRes
   1     5.3e-07   0.5000000     5.3e-07   10    5    5  1.7e-15  1.9e-15
Solution error estimates for segregated groups
0.32, 0.017, 0.083, 2.6e-07
Residual error estimates for segregated groups
33, 2.1e+03, 6.9e+03, 0.2
Segregated solver iteration 6.
Merged variables
Iter      SolEst     Damping    Stepsize #Res #Jac #Sol   LinErr   LinRes
   1        0.12   0.3500000        0.12   22   16   16    1e-13  2.7e-15
   2        0.08   0.3500000        0.08   23   17   17    1e-13  2.7e-15
   3       0.052   0.3500000       0.052   24   18   18  9.5e-14  2.9e-15
Velocity u2, Pressure p
Iter      SolEst     Damping    Stepsize #Res #Jac #Sol   LinErr   LinRes
   1       0.017   0.5000000       0.017   12    6    6  2.9e-15  7.7e-15
Displacement field
Iter      SolEst     Damping    Stepsize #Res #Jac #Sol   LinErr   LinRes
   1       0.095   0.5000000       0.095   12    6    6  6.3e-14  1.5e-12
Spatial mesh displacement
Iter      SolEst     Damping    Stepsize #Res #Jac #Sol   LinErr   LinRes
   1     3.7e-07   0.5000000     3.7e-07   12    6    6  2.2e-15  1.8e-15
Solution error estimates for segregated groups
0.09, 0.0085, 0.048, 1.8e-07
Residual error estimates for segregated groups
12, 1e+03, 4.2e+03, 0.19
```

```
Segregated solver iteration 7.
Merged variables
Iter      SolEst     Damping    Stepsize #Res #Jac #Sol   LinErr   LinRes
   1       0.034   0.3500000       0.034   26   19   19    1e-13  2.9e-15
   2       0.022   0.3500000       0.022   27   20   20  4.7e-14  2.7e-15
   3       0.014   0.3500000       0.014   28   21   21  5.8e-14  2.9e-15
Velocity u2, Pressure p
Iter      SolEst     Damping    Stepsize #Res #Jac #Sol   LinErr   LinRes
   1      0.0084   0.5000000      0.0084   14    7    7  2.1e-15  7.9e-15
Displacement field
Iter      SolEst     Damping    Stepsize #Res #Jac #Sol   LinErr   LinRes
   1       0.054   0.5000000       0.054   14    7    7  2.8e-14  1.5e-12
Spatial mesh displacement
Iter      SolEst     Damping    Stepsize #Res #Jac #Sol   LinErr   LinRes
   1     2.5e-07   0.5000000     2.5e-07   14    7    7  1.3e-15  1.9e-15
Solution error estimates for segregated groups
0.025, 0.0042, 0.027, 1.2e-07
Residual error estimates for segregated groups
4.2, 5.2e+02, 2.6e+03, 0.2
Segregated solver iteration 8.
Merged variables
Iter      SolEst     Damping    Stepsize #Res #Jac #Sol   LinErr   LinRes
   1      0.0094   0.3500000      0.0094   30   22   22  4.6e-14  2.7e-15
   2      0.0061   0.3500000      0.0061   31   23   23    7e-14  2.9e-15
   3       0.004   0.3500000       0.004   32   24   24  8.1e-14    3e-15
Velocity u2, Pressure p
Iter      SolEst     Damping    Stepsize #Res #Jac #Sol   LinErr   LinRes
   1      0.0042   0.5000000      0.0042   16    8    8    3e-15  7.8e-15
Displacement field
Iter      SolEst     Damping    Stepsize #Res #Jac #Sol   LinErr   LinRes
   1        0.03   0.5000000        0.03   16    8    8  7.8e-14  1.5e-12
Spatial mesh displacement
Iter      SolEst     Damping    Stepsize #Res #Jac #Sol   LinErr   LinRes
   1     1.6e-07   0.5000000     1.6e-07   16    8    8  1.9e-15  1.9e-15
Solution error estimates for segregated groups
0.0068, 0.0021, 0.015, 7.9e-08
Residual error estimates for segregated groups
1.4, 2.6e+02, 1.8e+03, 0.19
Segregated solver iteration 9.
Merged variables
Iter      SolEst     Damping    Stepsize #Res #Jac #Sol   LinErr   LinRes
   1      0.0026   0.3500000      0.0026   34   25   25  6.9e-14  2.8e-15
   2      0.0017   0.3500000      0.0017   35   26   26  5.9e-14  2.9e-15
   3      0.0011   0.3500000      0.0011   36   27   27  1.4e-13  2.7e-15
Velocity u2, Pressure p
Iter      SolEst     Damping    Stepsize #Res #Jac #Sol   LinErr   LinRes
   1      0.0021   0.5000000      0.0021   18    9    9  4.1e-15  7.9e-15
Displacement field
Iter      SolEst     Damping    Stepsize #Res #Jac #Sol   LinErr   LinRes
   1       0.017   0.5000000       0.017   18    9    9  7.9e-14  1.5e-12
Spatial mesh displacement
Iter      SolEst     Damping    Stepsize #Res #Jac #Sol   LinErr   LinRes
   1     9.9e-08   0.5000000     9.9e-08   18    9    9  1.4e-15  1.8e-15
Solution error estimates for segregated groups
0.0019, 0.001, 0.0085, 4.9e-08
Residual error estimates for segregated groups
```

```
0.45, 1.3e+02, 1.4e+03, 0.2
Segregated solver iteration 10.
Merged variables
Iter      SolEst     Damping    Stepsize #Res #Jac #Sol   LinErr   LinRes
   1     0.00071   0.3500000     0.00071   38   28   28  6.2e-14  2.9e-15
   2     0.00046   0.3500000     0.00046   39   29   29  6.9e-14  2.8e-15
   3      0.0003   0.3500000      0.0003   40   30   30  5.2e-14  2.8e-15
Velocity u2, Pressure p
Iter      SolEst     Damping    Stepsize #Res #Jac #Sol   LinErr   LinRes
   1       0.001   0.5000000       0.001   20   10   10  4.2e-15  7.9e-15
Displacement field
Iter      SolEst     Damping    Stepsize #Res #Jac #Sol   LinErr   LinRes
   1      0.0094   0.5000000      0.0094   20   10   10  5.3e-14  1.5e-12
Spatial mesh displacement
Iter      SolEst     Damping    Stepsize #Res #Jac #Sol   LinErr   LinRes
   1       6e-08   0.5000000       6e-08   20   10   10  1.7e-15  1.8e-15
Solution error estimates for segregated groups
0.00051, 0.00052, 0.0047, 3e-08
Residual error estimates for segregated groups
0.14, 66, 1.2e+03, 0.21
Segregated solver iteration 11.
Merged variables
Iter      SolEst     Damping    Stepsize #Res #Jac #Sol   LinErr   LinRes
   1     0.00019   0.3500000     0.00019   42   31   31    6e-14  2.7e-15
   2     0.00013   0.3500000     0.00013   43   32   32  5.7e-14  2.8e-15
   3     8.2e-05   0.3500000     8.2e-05   44   33   33  5.6e-14  2.7e-15
Velocity u2, Pressure p
Iter      SolEst     Damping    Stepsize #Res #Jac #Sol   LinErr   LinRes
   1     0.00052   0.5000000     0.00052   22   11   11  2.3e-15  7.8e-15
Displacement field
Iter      SolEst     Damping    Stepsize #Res #Jac #Sol   LinErr   LinRes
   1      0.0051   0.5000000      0.0051   22   11   11    1e-13  1.5e-12
Spatial mesh displacement
Iter      SolEst     Damping    Stepsize #Res #Jac #Sol   LinErr   LinRes
   1     3.6e-08   0.5000000     3.6e-08   22   11   11  1.3e-15  1.9e-15
Solution error estimates for segregated groups
0.00014, 0.00026, 0.0026, 1.8e-08
Residual error estimates for segregated groups
0.044, 33, 1.2e+03, 0.2
Segregated solver iteration 12.
Merged variables
Iter      SolEst     Damping    Stepsize #Res #Jac #Sol   LinErr   LinRes
   1     5.4e-05   0.3500000     5.4e-05   46   34   34  1.4e-13    3e-15
   2     3.5e-05   0.3500000     3.5e-05   47   35   35    1e-13  2.8e-15
   3     2.3e-05   0.3500000     2.3e-05   48   36   36  1.3e-13  2.7e-15
Velocity u2, Pressure p
Iter      SolEst     Damping    Stepsize #Res #Jac #Sol   LinErr   LinRes
   1     0.00026   0.5000000     0.00026   24   12   12  2.4e-15  7.8e-15
Displacement field
Iter      SolEst     Damping    Stepsize #Res #Jac #Sol   LinErr   LinRes
   1      0.0028   0.5000000      0.0028   24   12   12  5.3e-14  1.5e-12
Spatial mesh displacement
Iter      SolEst     Damping    Stepsize #Res #Jac #Sol   LinErr   LinRes
   1     2.1e-08   0.5000000     2.1e-08   24   12   12  1.8e-15  1.9e-15
Solution error estimates for segregated groups
3.9e-05, 0.00013, 0.0014, 1.1e-08
```

```
Residual error estimates for segregated groups
0.013, 16, 1.2e+03, 0.21
Segregated solver iteration 13.
Merged variables
Iter      SolEst      Damping    Stepsize #Res #Jac #Sol   LinErr   LinRes
   1     1.5e-05   0.3500000     1.5e-05   50   37   37  9.7e-14  2.7e-15
   2     9.6e-06   0.3500000     9.6e-06   51   38   38  9.7e-14  2.8e-15
   3     6.2e-06   0.3500000     6.2e-06   52   39   39  5.4e-14  2.8e-15
Velocity u2, Pressure p
Iter      SolEst      Damping    Stepsize #Res #Jac #Sol   LinErr   LinRes
   1     0.00013   0.5000000     0.00013   26   13   13  3.6e-15  7.8e-15
Displacement field
Iter      SolEst      Damping    Stepsize #Res #Jac #Sol   LinErr   LinRes
   1      0.0015   0.5000000      0.0015   26   13   13  7.8e-14  1.5e-12
Spatial mesh displacement
Iter      SolEst      Damping    Stepsize #Res #Jac #Sol   LinErr   LinRes
   1     1.2e-08   0.5000000     1.2e-08   26   13   13  1.5e-15  1.8e-15
Solution error estimates for segregated groups
1.1e-05, 6.5e-05, 0.00076, 6.2e-09
Residual error estimates for segregated groups
0.0041, 8.2, 1.2e+03, 0.22
Solution time: 159 s. (2 minutes, 39 seconds)
Physical memory: 4.5 GB
Virtual memory: 25.82 GB
Ended at Jan 31, 2025, 5:13:01 PM.
----- Stationary Solver 1 in study1/Approach Curve Solver (sol1) -------------->
```

d=2460 (su10)

GENERAL

Description	Value
Solution	d=2460

LOG

```
<---- Compile Equations: Stationary in study1/Approach Curve Solver (sol1) -----
Started at Jan 31, 2025, 5:13:01 PM.
Parameter d = 2.46E-6 (m).
Geometry shape function: Linear serendipity
Number of vertex elements: 18
Number of boundary elements: 6310
Number of elements: 157872
Minimum element quality: 0.4789
Time: 9 s.
Physical memory: 2.77 GB
Virtual memory: 15.18 GB
Ended at Jan 31, 2025, 5:13:10 PM.
----- Compile Equations: Stationary in study1/Approach Curve Solver (sol1) ---->

<---- Dependent Variables 1 in study1/Approach Curve Solver (sol1) -------------
Started at Jan 31, 2025, 5:13:10 PM.
Solution time: 0 s.
Physical memory: 2.77 GB
Virtual memory: 15.18 GB
Ended at Jan 31, 2025, 5:13:10 PM.
----- Dependent Variables 1 in study1/Approach Curve Solver (sol1) ------------>

<---- Stationary Solver 1 in study1/Approach Curve Solver (sol1) ---------------
Started at Jan 31, 2025, 5:13:10 PM.
Segregated solver
Segregated Step 11 does not have any components solved for.
Number of degrees of freedom solved for: 803454 (plus 12043 internal DOFs).
Segregated solver iteration 1.
Merged variables
Nonsymmetric matrix found.
Scales for dependent variables:
Concentration (comp1.Cl): 1.4e+02
Concentration (comp1.K): 1.4e+02
Electric potential (comp1.V): 1.9e+02
Orthonormal null-space function used.
Iter      SolEst     Damping    Stepsize #Res #Jac #Sol   LinErr   LinRes
   1        0.83   0.3500000        0.83    2    1    1  3.8e-11  2.8e-15
   2        0.81   0.3500000        0.81    3    2    2  2.2e-11  2.8e-15
   3         0.8   0.3500000         0.8    4    3    3  3.1e-11  2.7e-15
Velocity u2, Pressure p
Nonsymmetric matrix found.
Scales for dependent variables:
Pressure (comp1.p): 1
Velocity field (spatial frame) (comp1.u2): 0.00019
Orthonormal null-space function used.
Iter      SolEst     Damping    Stepsize #Res #Jac #Sol   LinErr   LinRes
   1         1.1   0.5000000         1.1    2    1    1  1.6e-15  2.3e-15
Displacement field
Nonsymmetric matrix found.
Scales for dependent variables:
Auxiliary pressure (comp1.solid.lemm1.pw): 1
Displacement field (comp1.u): 2.7e-18
Orthonormal null-space function used.
Iter      SolEst     Damping    Stepsize #Res #Jac #Sol   LinErr   LinRes
   1         1.9   0.5000000         1.9    2    1    1  5.1e-14  1.5e-12
Spatial mesh displacement
```

```
Symmetric matrices found.
Scales for dependent variables:
Spatial mesh displacement (comp1.spatial.disp): 1.2e-09
Orthonormal null-space function used.
Iter      SolEst     Damping    Stepsize #Res #Jac #Sol   LinErr   LinRes
   1     5.6e-07   0.5000000     5.6e-07    2    1    1  1.7e-15  1.9e-15
Solution error estimates for segregated groups
1.4, 0.53, 0.95, 2.8e-07
Residual error estimates for segregated groups
8e+03, 8.3e+04, 2.2e+04, 0.17
Segregated solver iteration 2.
Merged variables
Scales for dependent variables:
Concentration (comp1.Cl): 1.4e+02
Concentration (comp1.K): 1.4e+02
Electric potential (comp1.V): 0.064
Iter      SolEst     Damping    Stepsize #Res #Jac #Sol   LinErr   LinRes
   1        0.79   0.3500000        0.79    6    4    4  9.1e-14    3e-15
   2        0.77   0.3500000        0.77    7    5    5  4.9e-14  3.1e-15
   3        0.76   0.3500000        0.76    8    6    6  6.8e-14  3.1e-15
Velocity u2, Pressure p
Scales for dependent variables:
Pressure (comp1.p): 3.5e+02
Velocity field (spatial frame) (comp1.u2): 0.00071
Iter      SolEst     Damping    Stepsize #Res #Jac #Sol   LinErr   LinRes
   1        0.36   0.5000000        0.36    4    2    2  2.5e-15  7.8e-15
Displacement field
Iter      SolEst     Damping    Stepsize #Res #Jac #Sol   LinErr   LinRes
   1        0.95   0.5000000        0.95    4    2    2  5.1e-14  1.5e-12
Spatial mesh displacement
Iter      SolEst     Damping    Stepsize #Res #Jac #Sol   LinErr   LinRes
   1     8.4e-07   0.5000000     8.4e-07    4    2    2    2e-15  1.8e-15
Solution error estimates for segregated groups
1.3, 0.18, 0.48, 4.2e-07
Residual error estimates for segregated groups
8.2e+02, 1.3e+04, 2.2e+04, 0.2
Segregated solver iteration 3.
Merged variables
Iter      SolEst     Damping    Stepsize #Res #Jac #Sol   LinErr   LinRes
   1        0.75   0.3500000        0.75   10    7    7    7e-14  2.8e-15
   2        0.74   0.3500000        0.74   11    8    8  7.8e-14  3.2e-15
   3        0.72   0.3500000        0.72   12    9    9  8.4e-14    3e-15
Velocity u2, Pressure p
Iter      SolEst     Damping    Stepsize #Res #Jac #Sol   LinErr   LinRes
   1        0.15   0.5000000        0.15    6    3    3  2.4e-15  7.8e-15
Displacement field
Iter      SolEst     Damping    Stepsize #Res #Jac #Sol   LinErr   LinRes
   1        0.52   0.5000000        0.52    6    3    3  3.1e-14  1.5e-12
Spatial mesh displacement
Iter      SolEst     Damping    Stepsize #Res #Jac #Sol   LinErr   LinRes
   1     8.4e-07   0.5000000     8.4e-07    6    3    3  1.7e-15  1.8e-15
Solution error estimates for segregated groups
1.2, 0.076, 0.26, 4.2e-07
Residual error estimates for segregated groups
2.3e+02, 7.3e+03, 1.6e+04, 0.21
Segregated solver iteration 4.
```

```
Merged variables
Iter      SolEst     Damping    Stepsize #Res #Jac #Sol   LinErr   LinRes
   1        0.71   0.3500000        0.71   14   10   10  8.8e-14  3.1e-15
   2        0.69   0.3500000        0.69   15   11   11  5.3e-14  3.4e-15
   3        0.61   0.3500000        0.61   16   12   12  7.1e-14  3.2e-15
Velocity u2, Pressure p
Iter      SolEst     Damping    Stepsize #Res #Jac #Sol   LinErr   LinRes
   1       0.071   0.5000000       0.071    8    4    4  2.1e-15  7.8e-15
Displacement field
Iter      SolEst     Damping    Stepsize #Res #Jac #Sol   LinErr   LinRes
   1        0.29   0.5000000        0.29    8    4    4  6.7e-14  1.5e-12
Spatial mesh displacement
Iter      SolEst     Damping    Stepsize #Res #Jac #Sol   LinErr   LinRes
   1       7e-07   0.5000000       7e-07    8    4    4  1.7e-15  1.9e-15
Solution error estimates for segregated groups
1, 0.036, 0.15, 3.5e-07
Residual error estimates for segregated groups
86, 3.8e+03, 1.1e+04, 0.19
Segregated solver iteration 5.
Merged variables
Iter      SolEst     Damping    Stepsize #Res #Jac #Sol   LinErr   LinRes
   1        0.42   0.3500000        0.42   18   13   13  1.1e-13  3.1e-15
   2        0.28   0.3500000        0.28   19   14   14  6.5e-14  3.4e-15
   3        0.19   0.3500000        0.19   20   15   15  6.1e-14  2.9e-15
Velocity u2, Pressure p
Iter      SolEst     Damping    Stepsize #Res #Jac #Sol   LinErr   LinRes
   1       0.034   0.5000000       0.034   10    5    5    3e-15  7.8e-15
Displacement field
Iter      SolEst     Damping    Stepsize #Res #Jac #Sol   LinErr   LinRes
   1        0.17   0.5000000        0.17   10    5    5  5.4e-14  1.5e-12
Spatial mesh displacement
Iter      SolEst     Damping    Stepsize #Res #Jac #Sol   LinErr   LinRes
   1     5.3e-07   0.5000000     5.3e-07   10    5    5  1.6e-15  1.9e-15
Solution error estimates for segregated groups
0.32, 0.017, 0.084, 2.6e-07
Residual error estimates for segregated groups
33, 2e+03, 6.9e+03, 0.2
Segregated solver iteration 6.
Merged variables
Iter      SolEst     Damping    Stepsize #Res #Jac #Sol   LinErr   LinRes
   1        0.12   0.3500000        0.12   22   16   16  1.7e-13  3.2e-15
   2        0.08   0.3500000        0.08   23   17   17  5.4e-14  2.9e-15
   3       0.052   0.3500000       0.052   24   18   18  1.2e-13  3.1e-15
Velocity u2, Pressure p
Iter      SolEst     Damping    Stepsize #Res #Jac #Sol   LinErr   LinRes
   1       0.017   0.5000000       0.017   12    6    6  2.5e-15  7.9e-15
Displacement field
Iter      SolEst     Damping    Stepsize #Res #Jac #Sol   LinErr   LinRes
   1       0.095   0.5000000       0.095   12    6    6  8.3e-14  1.5e-12
Spatial mesh displacement
Iter      SolEst     Damping    Stepsize #Res #Jac #Sol   LinErr   LinRes
   1     3.7e-07   0.5000000     3.7e-07   12    6    6  1.9e-15  1.9e-15
Solution error estimates for segregated groups
0.09, 0.0085, 0.048, 1.8e-07
Residual error estimates for segregated groups
12, 9.9e+02, 4.3e+03, 0.2
```

```
Segregated solver iteration 7.
Merged variables
Iter      SolEst     Damping    Stepsize #Res #Jac #Sol   LinErr   LinRes
   1       0.034   0.3500000       0.034   26   19   19  1.1e-13  2.9e-15
   2       0.022   0.3500000       0.022   27   20   20    6e-14  3.1e-15
   3       0.014   0.3500000       0.014   28   21   21  1.1e-13    3e-15
Velocity u2, Pressure p
Iter      SolEst     Damping    Stepsize #Res #Jac #Sol   LinErr   LinRes
   1      0.0084   0.5000000      0.0084   14    7    7  2.1e-15    8e-15
Displacement field
Iter      SolEst     Damping    Stepsize #Res #Jac #Sol   LinErr   LinRes
   1       0.054   0.5000000       0.054   14    7    7  2.8e-14  1.5e-12
Spatial mesh displacement
Iter      SolEst     Damping    Stepsize #Res #Jac #Sol   LinErr   LinRes
   1     2.5e-07   0.5000000     2.5e-07   14    7    7  1.9e-15  1.9e-15
Solution error estimates for segregated groups
0.025, 0.0042, 0.027, 1.2e-07
Residual error estimates for segregated groups
4.2, 5e+02, 2.7e+03, 0.2
Segregated solver iteration 8.
Merged variables
Iter      SolEst     Damping    Stepsize #Res #Jac #Sol   LinErr   LinRes
   1      0.0094   0.3500000      0.0094   30   22   22  5.1e-14  3.4e-15
   2      0.0061   0.3500000      0.0061   31   23   23  6.6e-14  2.9e-15
   3       0.004   0.3500000       0.004   32   24   24    1e-13  2.9e-15
Velocity u2, Pressure p
Iter      SolEst     Damping    Stepsize #Res #Jac #Sol   LinErr   LinRes
   1      0.0042   0.5000000      0.0042   16    8    8  2.5e-15  7.8e-15
Displacement field
Iter      SolEst     Damping    Stepsize #Res #Jac #Sol   LinErr   LinRes
   1        0.03   0.5000000        0.03   16    8    8  2.7e-14  1.5e-12
Spatial mesh displacement
Iter      SolEst     Damping    Stepsize #Res #Jac #Sol   LinErr   LinRes
   1     1.6e-07   0.5000000     1.6e-07   16    8    8  1.5e-15  1.9e-15
Solution error estimates for segregated groups
0.0068, 0.0021, 0.015, 7.9e-08
Residual error estimates for segregated groups
1.4, 2.5e+02, 1.8e+03, 0.19
Segregated solver iteration 9.
Merged variables
Iter      SolEst     Damping    Stepsize #Res #Jac #Sol   LinErr   LinRes
   1      0.0026   0.3500000      0.0026   34   25   25  4.3e-14    3e-15
   2      0.0017   0.3500000      0.0017   35   26   26  1.1e-13  3.1e-15
   3      0.0011   0.3500000      0.0011   36   27   27  6.6e-14  3.1e-15
Velocity u2, Pressure p
Iter      SolEst     Damping    Stepsize #Res #Jac #Sol   LinErr   LinRes
   1      0.0021   0.5000000      0.0021   18    9    9  2.7e-15  7.9e-15
Displacement field
Iter      SolEst     Damping    Stepsize #Res #Jac #Sol   LinErr   LinRes
   1       0.017   0.5000000       0.017   18    9    9  1.7e-13  1.5e-12
Spatial mesh displacement
Iter      SolEst     Damping    Stepsize #Res #Jac #Sol   LinErr   LinRes
   1     9.9e-08   0.5000000     9.9e-08   18    9    9  1.7e-15  1.8e-15
Solution error estimates for segregated groups
0.0019, 0.001, 0.0085, 4.9e-08
Residual error estimates for segregated groups
```

```
0.45, 1.3e+02, 1.4e+03, 0.21
Segregated solver iteration 10.
Merged variables
Iter      SolEst     Damping    Stepsize #Res #Jac #Sol   LinErr   LinRes
   1     0.00071   0.3500000     0.00071   38   28   28  4.7e-14    3e-15
   2     0.00046   0.3500000     0.00046   39   29   29  5.3e-14  3.1e-15
   3      0.0003   0.3500000      0.0003   40   30   30  1.2e-13  3.1e-15
Velocity u2, Pressure p
Iter      SolEst     Damping    Stepsize #Res #Jac #Sol   LinErr   LinRes
   1       0.001   0.5000000       0.001   20   10   10  3.1e-15    8e-15
Displacement field
Iter      SolEst     Damping    Stepsize #Res #Jac #Sol   LinErr   LinRes
   1      0.0094   0.5000000      0.0094   20   10   10    7e-14  1.5e-12
Spatial mesh displacement
Iter      SolEst     Damping    Stepsize #Res #Jac #Sol   LinErr   LinRes
   1       6e-08   0.5000000       6e-08   20   10   10  2.5e-15  1.8e-15
Solution error estimates for segregated groups
0.00052, 0.00052, 0.0047, 3e-08
Residual error estimates for segregated groups
0.14, 63, 1.3e+03, 0.22
Segregated solver iteration 11.
Merged variables
Iter      SolEst     Damping    Stepsize #Res #Jac #Sol   LinErr   LinRes
   1      0.0002   0.3500000      0.0002   42   31   31  4.8e-14    3e-15
   2     0.00013   0.3500000     0.00013   43   32   32  5.9e-14    3e-15
   3     8.2e-05   0.3500000     8.2e-05   44   33   33  4.8e-14    3e-15
Velocity u2, Pressure p
Iter      SolEst     Damping    Stepsize #Res #Jac #Sol   LinErr   LinRes
   1     0.00052   0.5000000     0.00052   22   11   11  3.5e-15  7.7e-15
Displacement field
Iter      SolEst     Damping    Stepsize #Res #Jac #Sol   LinErr   LinRes
   1      0.0051   0.5000000      0.0051   22   11   11  4.4e-14  1.5e-12
Spatial mesh displacement
Iter      SolEst     Damping    Stepsize #Res #Jac #Sol   LinErr   LinRes
   1     3.6e-08   0.5000000     3.6e-08   22   11   11  1.6e-15  1.9e-15
Solution error estimates for segregated groups
0.00014, 0.00026, 0.0026, 1.8e-08
Residual error estimates for segregated groups
0.044, 31, 1.2e+03, 0.22
Segregated solver iteration 12.
Merged variables
Iter      SolEst     Damping    Stepsize #Res #Jac #Sol   LinErr   LinRes
   1     5.4e-05   0.3500000     5.4e-05   46   34   34  8.8e-14  4.1e-15
   2     3.5e-05   0.3500000     3.5e-05   47   35   35    8e-14  3.2e-15
   3     2.3e-05   0.3500000     2.3e-05   48   36   36  6.6e-14  3.2e-15
Velocity u2, Pressure p
Iter      SolEst     Damping    Stepsize #Res #Jac #Sol   LinErr   LinRes
   1     0.00026   0.5000000     0.00026   24   12   12  3.2e-15  7.9e-15
Displacement field
Iter      SolEst     Damping    Stepsize #Res #Jac #Sol   LinErr   LinRes
   1      0.0028   0.5000000      0.0028   24   12   12  8.9e-14  1.5e-12
Spatial mesh displacement
Iter      SolEst     Damping    Stepsize #Res #Jac #Sol   LinErr   LinRes
   1     2.1e-08   0.5000000     2.1e-08   24   12   12  1.8e-15  1.9e-15
Solution error estimates for segregated groups
3.9e-05, 0.00013, 0.0014, 1.1e-08
```

```
Residual error estimates for segregated groups
0.013, 16, 1.2e+03, 0.2
Segregated solver iteration 13.
Merged variables
Iter      SolEst      Damping    Stepsize #Res #Jac #Sol   LinErr   LinRes
   1      1.5e-05   0.3500000     1.5e-05   50   37   37  1.3e-13  3.2e-15
   2      9.6e-06   0.3500000     9.6e-06   51   38   38  8.6e-14    3e-15
   3      6.2e-06   0.3500000     6.2e-06   52   39   39  1.1e-13  2.9e-15
Velocity u2, Pressure p
Iter      SolEst      Damping    Stepsize #Res #Jac #Sol   LinErr   LinRes
   1      0.00013   0.5000000     0.00013   26   13   13  4.2e-15  7.8e-15
Displacement field
Iter      SolEst      Damping    Stepsize #Res #Jac #Sol   LinErr   LinRes
   1       0.0015   0.5000000      0.0015   26   13   13  6.1e-14  1.5e-12
Spatial mesh displacement
Iter      SolEst      Damping    Stepsize #Res #Jac #Sol   LinErr   LinRes
   1      1.3e-08   0.5000000     1.3e-08   26   13   13  1.7e-15  1.9e-15
Solution error estimates for segregated groups
1.1e-05, 6.5e-05, 0.00076, 6.3e-09
Residual error estimates for segregated groups
0.0041, 7.9, 1.2e+03, 0.2
Solution time: 158 s. (2 minutes, 38 seconds)
Physical memory: 4.54 GB
Virtual memory: 25.88 GB
Ended at Jan 31, 2025, 5:15:48 PM.
----- Stationary Solver 1 in study1/Approach Curve Solver (sol1) -------------->
```

d=2455 (su11)

GENERAL

Description	Value
Solution	d=2455

LOG

```
<---- Compile Equations: Stationary in study1/Approach Curve Solver (sol1) -----
Started at Jan 31, 2025, 5:15:48 PM.
Parameter d = 2.4550000000000002E-6 (m).
Geometry shape function: Linear serendipity
Number of vertex elements: 18
Number of boundary elements: 6308
Number of elements: 157250
Minimum element quality: 0.4881
Time: 9 s.
Physical memory: 2.78 GB
Virtual memory: 15.18 GB
Ended at Jan 31, 2025, 5:15:57 PM.
----- Compile Equations: Stationary in study1/Approach Curve Solver (sol1) ---->

<---- Dependent Variables 1 in study1/Approach Curve Solver (sol1) -------------
Started at Jan 31, 2025, 5:15:57 PM.
Solution time: 0 s.
Physical memory: 2.78 GB
Virtual memory: 15.18 GB
Ended at Jan 31, 2025, 5:15:57 PM.
----- Dependent Variables 1 in study1/Approach Curve Solver (sol1) ------------>

<---- Stationary Solver 1 in study1/Approach Curve Solver (sol1) ---------------
Started at Jan 31, 2025, 5:15:57 PM.
Segregated solver
Segregated Step 11 does not have any components solved for.
Number of degrees of freedom solved for: 800226 (plus 12039 internal DOFs).
Segregated solver iteration 1.
Merged variables
Nonsymmetric matrix found.
Scales for dependent variables:
Concentration (comp1.Cl): 1.4e+02
Concentration (comp1.K): 1.4e+02
Electric potential (comp1.V): 1.9e+02
Orthonormal null-space function used.
Iter      SolEst     Damping    Stepsize #Res #Jac #Sol   LinErr   LinRes
   1        0.83   0.3500000        0.83    2    1    1  4.5e-11  2.9e-15
   2        0.81   0.3500000        0.81    3    2    2  3.2e-11  2.7e-15
   3         0.8   0.3500000         0.8    4    3    3    2e-11  2.7e-15
Velocity u2, Pressure p
Nonsymmetric matrix found.
Scales for dependent variables:
Pressure (comp1.p): 1
Velocity field (spatial frame) (comp1.u2): 0.00019
Orthonormal null-space function used.
Iter      SolEst     Damping    Stepsize #Res #Jac #Sol   LinErr   LinRes
   1         1.1   0.5000000         1.1    2    1    1  1.8e-15  2.3e-15
Displacement field
Nonsymmetric matrix found.
Scales for dependent variables:
Auxiliary pressure (comp1.solid.lemm1.pw): 1
Displacement field (comp1.u): 2.8e-18
Orthonormal null-space function used.
Iter      SolEst     Damping    Stepsize #Res #Jac #Sol   LinErr   LinRes
   1         1.9   0.5000000         1.9    2    1    1  9.9e-14  1.5e-12
Spatial mesh displacement
```

```
Symmetric matrices found.
Scales for dependent variables:
Spatial mesh displacement (comp1.spatial.disp): 1.2e-09
Orthonormal null-space function used.
Iter      SolEst     Damping    Stepsize #Res #Jac #Sol   LinErr   LinRes
   1     5.7e-07   0.5000000     5.7e-07    2    1    1  1.6e-15  1.9e-15
Solution error estimates for segregated groups
1.4, 0.54, 0.95, 2.8e-07
Residual error estimates for segregated groups
8e+03, 8.3e+04, 2.2e+04, 0.16
Segregated solver iteration 2.
Merged variables
Scales for dependent variables:
Concentration (comp1.Cl): 1.4e+02
Concentration (comp1.K): 1.4e+02
Electric potential (comp1.V): 0.064
Iter      SolEst     Damping    Stepsize #Res #Jac #Sol   LinErr   LinRes
   1        0.79   0.3500000        0.79    6    4    4  1.2e-13  2.9e-15
   2        0.77   0.3500000        0.77    7    5    5  4.6e-14  2.8e-15
   3        0.76   0.3500000        0.76    8    6    6  4.9e-14  3.4e-15
Velocity u2, Pressure p
Scales for dependent variables:
Pressure (comp1.p): 3.5e+02
Velocity field (spatial frame) (comp1.u2): 0.00071
Iter      SolEst     Damping    Stepsize #Res #Jac #Sol   LinErr   LinRes
   1        0.36   0.5000000        0.36    4    2    2  3.7e-15  7.8e-15
Displacement field
Iter      SolEst     Damping    Stepsize #Res #Jac #Sol   LinErr   LinRes
   1        0.95   0.5000000        0.95    4    2    2  2.1e-13  1.5e-12
Spatial mesh displacement
Iter      SolEst     Damping    Stepsize #Res #Jac #Sol   LinErr   LinRes
   1     8.5e-07   0.5000000     8.5e-07    4    2    2  1.4e-15  1.9e-15
Solution error estimates for segregated groups
1.3, 0.18, 0.48, 4.3e-07
Residual error estimates for segregated groups
8.2e+02, 1.4e+04, 2.2e+04, 0.19
Segregated solver iteration 3.
Merged variables
Iter      SolEst     Damping    Stepsize #Res #Jac #Sol   LinErr   LinRes
   1        0.75   0.3500000        0.75   10    7    7    1e-13  2.8e-15
   2        0.74   0.3500000        0.74   11    8    8    7e-14  2.9e-15
   3        0.72   0.3500000        0.72   12    9    9  4.1e-14  2.9e-15
Velocity u2, Pressure p
Iter      SolEst     Damping    Stepsize #Res #Jac #Sol   LinErr   LinRes
   1        0.15   0.5000000        0.15    6    3    3    3e-15  7.8e-15
Displacement field
Iter      SolEst     Damping    Stepsize #Res #Jac #Sol   LinErr   LinRes
   1        0.52   0.5000000        0.52    6    3    3  7.7e-14  1.5e-12
Spatial mesh displacement
Iter      SolEst     Damping    Stepsize #Res #Jac #Sol   LinErr   LinRes
   1     8.5e-07   0.5000000     8.5e-07    6    3    3  1.3e-15  1.9e-15
Solution error estimates for segregated groups
1.2, 0.076, 0.26, 4.3e-07
Residual error estimates for segregated groups
2.3e+02, 7.6e+03, 1.6e+04, 0.21
Segregated solver iteration 4.
```

```
Merged variables
Iter      SolEst     Damping    Stepsize #Res #Jac #Sol   LinErr   LinRes
   1        0.71   0.3500000        0.71   14   10   10  1.1e-13  3.1e-15
   2        0.69   0.3500000        0.69   15   11   11  6.3e-14  2.8e-15
   3        0.61   0.3500000        0.61   16   12   12    7e-14  2.9e-15
Velocity u2, Pressure p
Iter      SolEst     Damping    Stepsize #Res #Jac #Sol   LinErr   LinRes
   1       0.071   0.5000000       0.071    8    4    4  2.6e-15  7.8e-15
Displacement field
Iter      SolEst     Damping    Stepsize #Res #Jac #Sol   LinErr   LinRes
   1        0.29   0.5000000        0.29    8    4    4    1e-13  1.5e-12
Spatial mesh displacement
Iter      SolEst     Damping    Stepsize #Res #Jac #Sol   LinErr   LinRes
   1     7.1e-07   0.5000000     7.1e-07    8    4    4  1.5e-15  1.8e-15
Solution error estimates for segregated groups
1, 0.036, 0.15, 3.5e-07
Residual error estimates for segregated groups
85, 4e+03, 1.1e+04, 0.19
Segregated solver iteration 5.
Merged variables
Iter      SolEst     Damping    Stepsize #Res #Jac #Sol   LinErr   LinRes
   1        0.42   0.3500000        0.42   18   13   13    9e-14    3e-15
   2        0.28   0.3500000        0.28   19   14   14  1.4e-13  2.8e-15
   3        0.19   0.3500000        0.19   20   15   15    5e-14  2.7e-15
Velocity u2, Pressure p
Iter      SolEst     Damping    Stepsize #Res #Jac #Sol   LinErr   LinRes
   1       0.035   0.5000000       0.035   10    5    5  2.2e-15  7.8e-15
Displacement field
Iter      SolEst     Damping    Stepsize #Res #Jac #Sol   LinErr   LinRes
   1        0.17   0.5000000        0.17   10    5    5  5.2e-14  1.5e-12
Spatial mesh displacement
Iter      SolEst     Damping    Stepsize #Res #Jac #Sol   LinErr   LinRes
   1     5.3e-07   0.5000000     5.3e-07   10    5    5  1.9e-15  1.9e-15
Solution error estimates for segregated groups
0.32, 0.017, 0.084, 2.7e-07
Residual error estimates for segregated groups
33, 2.1e+03, 6.9e+03, 0.21
Segregated solver iteration 6.
Merged variables
Iter      SolEst     Damping    Stepsize #Res #Jac #Sol   LinErr   LinRes
   1        0.12   0.3500000        0.12   22   16   16  8.9e-14  2.8e-15
   2        0.08   0.3500000        0.08   23   17   17    9e-14  2.8e-15
   3       0.052   0.3500000       0.052   24   18   18  1.1e-13    3e-15
Velocity u2, Pressure p
Iter      SolEst     Damping    Stepsize #Res #Jac #Sol   LinErr   LinRes
   1       0.017   0.5000000       0.017   12    6    6  1.9e-15  7.9e-15
Displacement field
Iter      SolEst     Damping    Stepsize #Res #Jac #Sol   LinErr   LinRes
   1       0.095   0.5000000       0.095   12    6    6  6.9e-14  1.5e-12
Spatial mesh displacement
Iter      SolEst     Damping    Stepsize #Res #Jac #Sol   LinErr   LinRes
   1     3.7e-07   0.5000000     3.7e-07   12    6    6  1.7e-15  1.8e-15
Solution error estimates for segregated groups
0.089, 0.0085, 0.048, 1.9e-07
Residual error estimates for segregated groups
12, 1e+03, 4.2e+03, 0.19
```

```
Segregated solver iteration 7.
Merged variables
Iter      SolEst     Damping    Stepsize #Res #Jac #Sol   LinErr   LinRes
   1       0.034   0.3500000       0.034   26   19   19  6.9e-14  2.8e-15
   2       0.022   0.3500000       0.022   27   20   20  1.3e-13  2.9e-15
   3       0.014   0.3500000       0.014   28   21   21  6.7e-14    3e-15
Velocity u2, Pressure p
Iter      SolEst     Damping    Stepsize #Res #Jac #Sol   LinErr   LinRes
   1      0.0084   0.5000000      0.0084   14    7    7  2.6e-15  7.7e-15
Displacement field
Iter      SolEst     Damping    Stepsize #Res #Jac #Sol   LinErr   LinRes
   1       0.054   0.5000000       0.054   14    7    7  1.4e-13  1.5e-12
Spatial mesh displacement
Iter      SolEst     Damping    Stepsize #Res #Jac #Sol   LinErr   LinRes
   1     2.5e-07   0.5000000     2.5e-07   14    7    7  1.6e-15  1.8e-15
Solution error estimates for segregated groups
0.025, 0.0042, 0.027, 1.2e-07
Residual error estimates for segregated groups
4.2, 5.2e+02, 2.7e+03, 0.19
Segregated solver iteration 8.
Merged variables
Iter      SolEst     Damping    Stepsize #Res #Jac #Sol   LinErr   LinRes
   1      0.0094   0.3500000      0.0094   30   22   22  1.2e-13  2.8e-15
   2      0.0061   0.3500000      0.0061   31   23   23  1.2e-13    3e-15
   3       0.004   0.3500000       0.004   32   24   24  6.2e-14  3.1e-15
Velocity u2, Pressure p
Iter      SolEst     Damping    Stepsize #Res #Jac #Sol   LinErr   LinRes
   1      0.0042   0.5000000      0.0042   16    8    8  5.1e-15  7.9e-15
Displacement field
Iter      SolEst     Damping    Stepsize #Res #Jac #Sol   LinErr   LinRes
   1        0.03   0.5000000        0.03   16    8    8  9.7e-14  1.5e-12
Spatial mesh displacement
Iter      SolEst     Damping    Stepsize #Res #Jac #Sol   LinErr   LinRes
   1     1.6e-07   0.5000000     1.6e-07   16    8    8    2e-15  1.9e-15
Solution error estimates for segregated groups
0.0068, 0.0021, 0.015, 8e-08
Residual error estimates for segregated groups
1.4, 2.6e+02, 1.8e+03, 0.21
Segregated solver iteration 9.
Merged variables
Iter      SolEst     Damping    Stepsize #Res #Jac #Sol   LinErr   LinRes
   1      0.0026   0.3500000      0.0026   34   25   25    1e-13    3e-15
   2      0.0017   0.3500000      0.0017   35   26   26  7.1e-14  2.9e-15
   3      0.0011   0.3500000      0.0011   36   27   27  8.1e-14    3e-15
Velocity u2, Pressure p
Iter      SolEst     Damping    Stepsize #Res #Jac #Sol   LinErr   LinRes
   1      0.0021   0.5000000      0.0021   18    9    9  2.3e-15  7.9e-15
Displacement field
Iter      SolEst     Damping    Stepsize #Res #Jac #Sol   LinErr   LinRes
   1       0.017   0.5000000       0.017   18    9    9  5.2e-14  1.5e-12
Spatial mesh displacement
Iter      SolEst     Damping    Stepsize #Res #Jac #Sol   LinErr   LinRes
   1       1e-07   0.5000000       1e-07   18    9    9  1.6e-15  1.9e-15
Solution error estimates for segregated groups
0.0019, 0.001, 0.0085, 5e-08
Residual error estimates for segregated groups
```

```
0.45, 1.3e+02, 1.4e+03, 0.2
Segregated solver iteration 10.
Merged variables
Iter      SolEst     Damping    Stepsize #Res #Jac #Sol   LinErr   LinRes
   1     0.00071   0.3500000     0.00071   38   28   28  8.4e-14  3.1e-15
   2     0.00046   0.3500000     0.00046   39   29   29  1.1e-13  2.9e-15
   3      0.0003   0.3500000      0.0003   40   30   30  1.1e-13  3.1e-15
Velocity u2, Pressure p
Iter      SolEst     Damping    Stepsize #Res #Jac #Sol   LinErr   LinRes
   1       0.001   0.5000000       0.001   20   10   10  2.4e-15  7.9e-15
Displacement field
Iter      SolEst     Damping    Stepsize #Res #Jac #Sol   LinErr   LinRes
   1      0.0094   0.5000000      0.0094   20   10   10  2.3e-14  1.5e-12
Spatial mesh displacement
Iter      SolEst     Damping    Stepsize #Res #Jac #Sol   LinErr   LinRes
   1     6.1e-08   0.5000000     6.1e-08   20   10   10  1.7e-15  1.8e-15
Solution error estimates for segregated groups
0.00051, 0.00052, 0.0047, 3e-08
Residual error estimates for segregated groups
0.14, 66, 1.3e+03, 0.2
Segregated solver iteration 11.
Merged variables
Iter      SolEst     Damping    Stepsize #Res #Jac #Sol   LinErr   LinRes
   1     0.00019   0.3500000     0.00019   42   31   31    1e-13  3.8e-15
   2     0.00013   0.3500000     0.00013   43   32   32  8.4e-14  2.9e-15
   3     8.2e-05   0.3500000     8.2e-05   44   33   33    7e-14  2.8e-15
Velocity u2, Pressure p
Iter      SolEst     Damping    Stepsize #Res #Jac #Sol   LinErr   LinRes
   1     0.00052   0.5000000     0.00052   22   11   11  2.4e-15  7.8e-15
Displacement field
Iter      SolEst     Damping    Stepsize #Res #Jac #Sol   LinErr   LinRes
   1      0.0051   0.5000000      0.0051   22   11   11  4.8e-14  1.5e-12
Spatial mesh displacement
Iter      SolEst     Damping    Stepsize #Res #Jac #Sol   LinErr   LinRes
   1     3.7e-08   0.5000000     3.7e-08   22   11   11  1.6e-15  1.9e-15
Solution error estimates for segregated groups
0.00014, 0.00026, 0.0026, 1.8e-08
Residual error estimates for segregated groups
0.044, 33, 1.2e+03, 0.2
Segregated solver iteration 12.
Merged variables
Iter      SolEst     Damping    Stepsize #Res #Jac #Sol   LinErr   LinRes
   1     5.3e-05   0.3500000     5.3e-05   46   34   34    5e-14  2.9e-15
   2     3.5e-05   0.3500000     3.5e-05   47   35   35  1.5e-13  2.8e-15
   3     2.3e-05   0.3500000     2.3e-05   48   36   36  5.3e-14  2.8e-15
Velocity u2, Pressure p
Iter      SolEst     Damping    Stepsize #Res #Jac #Sol   LinErr   LinRes
   1     0.00026   0.5000000     0.00026   24   12   12  2.2e-15  7.8e-15
Displacement field
Iter      SolEst     Damping    Stepsize #Res #Jac #Sol   LinErr   LinRes
   1      0.0028   0.5000000      0.0028   24   12   12  5.8e-14  1.5e-12
Spatial mesh displacement
Iter      SolEst     Damping    Stepsize #Res #Jac #Sol   LinErr   LinRes
   1     2.2e-08   0.5000000     2.2e-08   24   12   12  1.7e-15  1.9e-15
Solution error estimates for segregated groups
3.9e-05, 0.00013, 0.0014, 1.1e-08
```

```
Residual error estimates for segregated groups
0.013, 16, 1.2e+03, 0.21
Segregated solver iteration 13.
Merged variables
Iter      SolEst      Damping    Stepsize #Res #Jac #Sol   LinErr   LinRes
   1     1.5e-05   0.3500000     1.5e-05   50   37   37  6.7e-14  2.9e-15
   2     9.5e-06   0.3500000     9.5e-06   51   38   38  1.1e-13  2.9e-15
   3     6.2e-06   0.3500000     6.2e-06   52   39   39  7.6e-14  2.8e-15
Velocity u2, Pressure p
Iter      SolEst      Damping    Stepsize #Res #Jac #Sol   LinErr   LinRes
   1     0.00013   0.5000000     0.00013   26   13   13  2.5e-15  7.8e-15
Displacement field
Iter      SolEst      Damping    Stepsize #Res #Jac #Sol   LinErr   LinRes
   1      0.0015   0.5000000      0.0015   26   13   13    6e-14  1.5e-12
Spatial mesh displacement
Iter      SolEst      Damping    Stepsize #Res #Jac #Sol   LinErr   LinRes
   1     1.3e-08   0.5000000     1.3e-08   26   13   13  1.9e-15  1.9e-15
Solution error estimates for segregated groups
1.1e-05, 6.5e-05, 0.00076, 6.3e-09
Residual error estimates for segregated groups
0.0041, 8.2, 1.2e+03, 0.22
Solution time: 155 s. (2 minutes, 35 seconds)
Physical memory: 4.55 GB
Virtual memory: 25.54 GB
Ended at Jan 31, 2025, 5:18:32 PM.
----- Stationary Solver 1 in study1/Approach Curve Solver (sol1) -------------->
```

d=2450 (su12)

GENERAL

Description	Value
Solution	d=2450

LOG

```
<---- Compile Equations: Stationary in study1/Approach Curve Solver (sol1) -----
Started at Jan 31, 2025, 5:18:32 PM.
Parameter d = 2.4500000000000003E-6 (m).
Geometry shape function: Linear serendipity
Number of vertex elements: 18
Number of boundary elements: 6305
Number of elements: 157309
Minimum element quality: 0.4685
Time: 9 s.
Physical memory: 2.76 GB
Virtual memory: 15.15 GB
Ended at Jan 31, 2025, 5:18:41 PM.
----- Compile Equations: Stationary in study1/Approach Curve Solver (sol1) ---->

<---- Dependent Variables 1 in study1/Approach Curve Solver (sol1) -------------
Started at Jan 31, 2025, 5:18:41 PM.
Solution time: 0 s.
Physical memory: 2.76 GB
Virtual memory: 15.15 GB
Ended at Jan 31, 2025, 5:18:41 PM.
----- Dependent Variables 1 in study1/Approach Curve Solver (sol1) ------------>

<---- Stationary Solver 1 in study1/Approach Curve Solver (sol1) ---------------
Started at Jan 31, 2025, 5:18:41 PM.
Segregated solver
Segregated Step 11 does not have any components solved for.
Number of degrees of freedom solved for: 800583 (plus 12033 internal DOFs).
Segregated solver iteration 1.
Merged variables
Nonsymmetric matrix found.
Scales for dependent variables:
Concentration (comp1.Cl): 1.4e+02
Concentration (comp1.K): 1.4e+02
Electric potential (comp1.V): 1.9e+02
Orthonormal null-space function used.
Iter      SolEst     Damping    Stepsize #Res #Jac #Sol   LinErr   LinRes
   1        0.83   0.3500000        0.83    2    1    1    6e-11  2.7e-15
   2        0.81   0.3500000        0.81    3    2    2  4.6e-11  2.8e-15
   3         0.8   0.3500000         0.8    4    3    3  2.6e-11  2.7e-15
Velocity u2, Pressure p
Nonsymmetric matrix found.
Scales for dependent variables:
Pressure (comp1.p): 1
Velocity field (spatial frame) (comp1.u2): 0.00019
Orthonormal null-space function used.
Iter      SolEst     Damping    Stepsize #Res #Jac #Sol   LinErr   LinRes
   1         1.1   0.5000000         1.1    2    1    1  1.7e-15  2.3e-15
Displacement field
Nonsymmetric matrix found.
Scales for dependent variables:
Auxiliary pressure (comp1.solid.lemm1.pw): 1
Displacement field (comp1.u): 2.8e-18
Orthonormal null-space function used.
Iter      SolEst     Damping    Stepsize #Res #Jac #Sol   LinErr   LinRes
   1         1.9   0.5000000         1.9    2    1    1  5.7e-14  1.5e-12
Spatial mesh displacement
```

```
Symmetric matrices found.
Scales for dependent variables:
Spatial mesh displacement (comp1.spatial.disp): 1.2e-09
Orthonormal null-space function used.
Iter      SolEst     Damping    Stepsize #Res #Jac #Sol   LinErr   LinRes
   1     5.7e-07   0.5000000     5.7e-07    2    1    1  1.7e-15  1.9e-15
Solution error estimates for segregated groups
1.4, 0.53, 0.95, 2.8e-07
Residual error estimates for segregated groups
8e+03, 8.3e+04, 2.2e+04, 0.16
Segregated solver iteration 2.
Merged variables
Scales for dependent variables:
Concentration (comp1.Cl): 1.4e+02
Concentration (comp1.K): 1.4e+02
Electric potential (comp1.V): 0.064
Iter      SolEst     Damping    Stepsize #Res #Jac #Sol   LinErr   LinRes
   1        0.79   0.3500000        0.79    6    4    4  7.1e-14  2.8e-15
   2        0.77   0.3500000        0.77    7    5    5  5.5e-14  2.9e-15
   3        0.76   0.3500000        0.76    8    6    6  8.3e-14  3.1e-15
Velocity u2, Pressure p
Scales for dependent variables:
Pressure (comp1.p): 3.5e+02
Velocity field (spatial frame) (comp1.u2): 0.00071
Iter      SolEst     Damping    Stepsize #Res #Jac #Sol   LinErr   LinRes
   1        0.36   0.5000000        0.36    4    2    2  2.3e-15  7.8e-15
Displacement field
Iter      SolEst     Damping    Stepsize #Res #Jac #Sol   LinErr   LinRes
   1        0.95   0.5000000        0.95    4    2    2  6.9e-14  1.5e-12
Spatial mesh displacement
Iter      SolEst     Damping    Stepsize #Res #Jac #Sol   LinErr   LinRes
   1     8.5e-07   0.5000000     8.5e-07    4    2    2  1.4e-15  1.9e-15
Solution error estimates for segregated groups
1.3, 0.18, 0.48, 4.2e-07
Residual error estimates for segregated groups
8.2e+02, 1.4e+04, 2.2e+04, 0.19
Segregated solver iteration 3.
Merged variables
Iter      SolEst     Damping    Stepsize #Res #Jac #Sol   LinErr   LinRes
   1        0.75   0.3500000        0.75   10    7    7  1.2e-13  3.5e-15
   2        0.74   0.3500000        0.74   11    8    8  3.6e-14  2.8e-15
   3        0.72   0.3500000        0.72   12    9    9    1e-13  3.2e-15
Velocity u2, Pressure p
Iter      SolEst     Damping    Stepsize #Res #Jac #Sol   LinErr   LinRes
   1        0.15   0.5000000        0.15    6    3    3  2.7e-15  7.7e-15
Displacement field
Iter      SolEst     Damping    Stepsize #Res #Jac #Sol   LinErr   LinRes
   1        0.52   0.5000000        0.52    6    3    3  1.2e-13  1.5e-12
Spatial mesh displacement
Iter      SolEst     Damping    Stepsize #Res #Jac #Sol   LinErr   LinRes
   1     8.5e-07   0.5000000     8.5e-07    6    3    3  1.5e-15  1.9e-15
Solution error estimates for segregated groups
1.2, 0.076, 0.26, 4.2e-07
Residual error estimates for segregated groups
2.3e+02, 8e+03, 1.6e+04, 0.2
Segregated solver iteration 4.
```

```
Merged variables
Iter      SolEst     Damping    Stepsize #Res #Jac #Sol   LinErr   LinRes
   1        0.71   0.3500000        0.71   14   10   10  4.7e-14  3.2e-15
   2        0.69   0.3500000        0.69   15   11   11  8.4e-14  3.2e-15
   3        0.61   0.3500000        0.61   16   12   12  1.6e-13  2.8e-15
Velocity u2, Pressure p
Iter      SolEst     Damping    Stepsize #Res #Jac #Sol   LinErr   LinRes
   1       0.071   0.5000000       0.071    8    4    4  2.2e-15  7.7e-15
Displacement field
Iter      SolEst     Damping    Stepsize #Res #Jac #Sol   LinErr   LinRes
   1        0.29   0.5000000        0.29    8    4    4  1.2e-13  1.5e-12
Spatial mesh displacement
Iter      SolEst     Damping    Stepsize #Res #Jac #Sol   LinErr   LinRes
   1     7.1e-07   0.5000000     7.1e-07    8    4    4  1.6e-15  1.8e-15
Solution error estimates for segregated groups
1, 0.036, 0.15, 3.5e-07
Residual error estimates for segregated groups
86, 4.2e+03, 1.1e+04, 0.2
Segregated solver iteration 5.
Merged variables
Iter      SolEst     Damping    Stepsize #Res #Jac #Sol   LinErr   LinRes
   1        0.42   0.3500000        0.42   18   13   13    1e-13  3.4e-15
   2        0.28   0.3500000        0.28   19   14   14  7.6e-14  3.4e-15
   3        0.19   0.3500000        0.19   20   15   15  8.6e-14  3.1e-15
Velocity u2, Pressure p
Iter      SolEst     Damping    Stepsize #Res #Jac #Sol   LinErr   LinRes
   1       0.034   0.5000000       0.034   10    5    5  2.3e-15  7.9e-15
Displacement field
Iter      SolEst     Damping    Stepsize #Res #Jac #Sol   LinErr   LinRes
   1        0.17   0.5000000        0.17   10    5    5  1.6e-13  1.5e-12
Spatial mesh displacement
Iter      SolEst     Damping    Stepsize #Res #Jac #Sol   LinErr   LinRes
   1     5.3e-07   0.5000000     5.3e-07   10    5    5  1.8e-15  1.9e-15
Solution error estimates for segregated groups
0.32, 0.017, 0.084, 2.7e-07
Residual error estimates for segregated groups
33, 2.2e+03, 6.9e+03, 0.2
Segregated solver iteration 6.
Merged variables
Iter      SolEst     Damping    Stepsize #Res #Jac #Sol   LinErr   LinRes
   1        0.12   0.3500000        0.12   22   16   16  7.9e-14  3.2e-15
   2        0.08   0.3500000        0.08   23   17   17  5.3e-14  2.8e-15
   3       0.052   0.3500000       0.052   24   18   18  8.6e-14  3.1e-15
Velocity u2, Pressure p
Iter      SolEst     Damping    Stepsize #Res #Jac #Sol   LinErr   LinRes
   1       0.017   0.5000000       0.017   12    6    6  2.4e-15  7.8e-15
Displacement field
Iter      SolEst     Damping    Stepsize #Res #Jac #Sol   LinErr   LinRes
   1       0.095   0.5000000       0.095   12    6    6  4.8e-14  1.5e-12
Spatial mesh displacement
Iter      SolEst     Damping    Stepsize #Res #Jac #Sol   LinErr   LinRes
   1     3.7e-07   0.5000000     3.7e-07   12    6    6  1.9e-15  1.9e-15
Solution error estimates for segregated groups
0.09, 0.0085, 0.048, 1.9e-07
Residual error estimates for segregated groups
12, 1.1e+03, 4.2e+03, 0.19
```

```
Segregated solver iteration 7.
Merged variables
Iter      SolEst     Damping    Stepsize #Res #Jac #Sol   LinErr   LinRes
   1       0.034   0.3500000       0.034   26   19   19  7.6e-14  2.9e-15
   2       0.022   0.3500000       0.022   27   20   20    5e-14  3.2e-15
   3       0.014   0.3500000       0.014   28   21   21  6.4e-14  3.3e-15
Velocity u2, Pressure p
Iter      SolEst     Damping    Stepsize #Res #Jac #Sol   LinErr   LinRes
   1      0.0084   0.5000000      0.0084   14    7    7    3e-15  7.9e-15
Displacement field
Iter      SolEst     Damping    Stepsize #Res #Jac #Sol   LinErr   LinRes
   1       0.054   0.5000000       0.054   14    7    7  1.1e-13  1.5e-12
Spatial mesh displacement
Iter      SolEst     Damping    Stepsize #Res #Jac #Sol   LinErr   LinRes
   1     2.5e-07   0.5000000     2.5e-07   14    7    7  1.5e-15  1.9e-15
Solution error estimates for segregated groups
0.025, 0.0042, 0.027, 1.2e-07
Residual error estimates for segregated groups
4.2, 5.6e+02, 2.7e+03, 0.21
Segregated solver iteration 8.
Merged variables
Iter      SolEst     Damping    Stepsize #Res #Jac #Sol   LinErr   LinRes
   1      0.0094   0.3500000      0.0094   30   22   22  5.5e-14  3.2e-15
   2      0.0061   0.3500000      0.0061   31   23   23  7.8e-14  2.7e-15
   3       0.004   0.3500000       0.004   32   24   24  1.3e-13  3.2e-15
Velocity u2, Pressure p
Iter      SolEst     Damping    Stepsize #Res #Jac #Sol   LinErr   LinRes
   1      0.0042   0.5000000      0.0042   16    8    8  3.3e-15  7.8e-15
Displacement field
Iter      SolEst     Damping    Stepsize #Res #Jac #Sol   LinErr   LinRes
   1        0.03   0.5000000        0.03   16    8    8  6.8e-14  1.5e-12
Spatial mesh displacement
Iter      SolEst     Damping    Stepsize #Res #Jac #Sol   LinErr   LinRes
   1     1.6e-07   0.5000000     1.6e-07   16    8    8  1.6e-15  1.8e-15
Solution error estimates for segregated groups
0.0068, 0.0021, 0.015, 8e-08
Residual error estimates for segregated groups
1.4, 2.8e+02, 1.8e+03, 0.2
Segregated solver iteration 9.
Merged variables
Iter      SolEst     Damping    Stepsize #Res #Jac #Sol   LinErr   LinRes
   1      0.0026   0.3500000      0.0026   34   25   25  1.1e-13    3e-15
   2      0.0017   0.3500000      0.0017   35   26   26  3.9e-14  3.2e-15
   3      0.0011   0.3500000      0.0011   36   27   27  1.1e-13  2.9e-15
Velocity u2, Pressure p
Iter      SolEst     Damping    Stepsize #Res #Jac #Sol   LinErr   LinRes
   1      0.0021   0.5000000      0.0021   18    9    9  2.1e-15  7.8e-15
Displacement field
Iter      SolEst     Damping    Stepsize #Res #Jac #Sol   LinErr   LinRes
   1       0.017   0.5000000       0.017   18    9    9  7.8e-14  1.5e-12
Spatial mesh displacement
Iter      SolEst     Damping    Stepsize #Res #Jac #Sol   LinErr   LinRes
   1     9.9e-08   0.5000000     9.9e-08   18    9    9  1.5e-15  1.9e-15
Solution error estimates for segregated groups
0.0019, 0.001, 0.0085, 5e-08
Residual error estimates for segregated groups
```

```
0.45, 1.4e+02, 1.4e+03, 0.21
Segregated solver iteration 10.
Merged variables
Iter      SolEst     Damping    Stepsize #Res #Jac #Sol   LinErr   LinRes
   1     0.00071   0.3500000     0.00071   38   28   28  6.4e-14    3e-15
   2     0.00046   0.3500000     0.00046   39   29   29  6.2e-14  2.9e-15
   3      0.0003   0.3500000      0.0003   40   30   30  8.5e-14  2.9e-15
Velocity u2, Pressure p
Iter      SolEst     Damping    Stepsize #Res #Jac #Sol   LinErr   LinRes
   1       0.001   0.5000000       0.001   20   10   10    3e-15  7.8e-15
Displacement field
Iter      SolEst     Damping    Stepsize #Res #Jac #Sol   LinErr   LinRes
   1      0.0094   0.5000000      0.0094   20   10   10  9.5e-14  1.5e-12
Spatial mesh displacement
Iter      SolEst     Damping    Stepsize #Res #Jac #Sol   LinErr   LinRes
   1     6.1e-08   0.5000000     6.1e-08   20   10   10  1.3e-15  1.8e-15
Solution error estimates for segregated groups
0.00051, 0.00052, 0.0047, 3e-08
Residual error estimates for segregated groups
0.14, 70, 1.2e+03, 0.2
Segregated solver iteration 11.
Merged variables
Iter      SolEst     Damping    Stepsize #Res #Jac #Sol   LinErr   LinRes
   1     0.00019   0.3500000     0.00019   42   31   31  5.7e-14  3.2e-15
   2     0.00013   0.3500000     0.00013   43   32   32  9.9e-14  2.9e-15
   3     8.2e-05   0.3500000     8.2e-05   44   33   33  7.8e-14  2.9e-15
Velocity u2, Pressure p
Iter      SolEst     Damping    Stepsize #Res #Jac #Sol   LinErr   LinRes
   1     0.00052   0.5000000     0.00052   22   11   11  2.5e-15  7.8e-15
Displacement field
Iter      SolEst     Damping    Stepsize #Res #Jac #Sol   LinErr   LinRes
   1      0.0051   0.5000000      0.0051   22   11   11  7.8e-14  1.5e-12
Spatial mesh displacement
Iter      SolEst     Damping    Stepsize #Res #Jac #Sol   LinErr   LinRes
   1     3.6e-08   0.5000000     3.6e-08   22   11   11  1.5e-15  1.9e-15
Solution error estimates for segregated groups
0.00014, 0.00026, 0.0026, 1.8e-08
Residual error estimates for segregated groups
0.044, 35, 1.2e+03, 0.2
Segregated solver iteration 12.
Merged variables
Iter      SolEst     Damping    Stepsize #Res #Jac #Sol   LinErr   LinRes
   1     5.3e-05   0.3500000     5.3e-05   46   34   34  5.9e-14  3.7e-15
   2     3.5e-05   0.3500000     3.5e-05   47   35   35    1e-13    3e-15
   3     2.3e-05   0.3500000     2.3e-05   48   36   36  8.4e-14  2.8e-15
Velocity u2, Pressure p
Iter      SolEst     Damping    Stepsize #Res #Jac #Sol   LinErr   LinRes
   1     0.00026   0.5000000     0.00026   24   12   12  2.4e-15  7.8e-15
Displacement field
Iter      SolEst     Damping    Stepsize #Res #Jac #Sol   LinErr   LinRes
   1      0.0028   0.5000000      0.0028   24   12   12    9e-14  1.5e-12
Spatial mesh displacement
Iter      SolEst     Damping    Stepsize #Res #Jac #Sol   LinErr   LinRes
   1     2.2e-08   0.5000000     2.2e-08   24   12   12  1.5e-15  1.9e-15
Solution error estimates for segregated groups
3.9e-05, 0.00013, 0.0014, 1.1e-08
```

```
Residual error estimates for segregated groups
0.013, 17, 1.2e+03, 0.19
Segregated solver iteration 13.
Merged variables
Iter      SolEst     Damping    Stepsize #Res #Jac #Sol   LinErr   LinRes
   1      1.5e-05   0.3500000    1.5e-05   50   37   37  5.3e-14  3.2e-15
   2      9.5e-06   0.3500000    9.5e-06   51   38   38  7.3e-14  2.9e-15
   3      6.2e-06   0.3500000    6.2e-06   52   39   39  1.1e-13    3e-15
Velocity u2, Pressure p
Iter      SolEst     Damping    Stepsize #Res #Jac #Sol   LinErr   LinRes
   1      0.00013   0.5000000    0.00013   26   13   13  1.9e-15  7.8e-15
Displacement field
Iter      SolEst     Damping    Stepsize #Res #Jac #Sol   LinErr   LinRes
   1       0.0015   0.5000000     0.0015   26   13   13  7.9e-14  1.5e-12
Spatial mesh displacement
Iter      SolEst     Damping    Stepsize #Res #Jac #Sol   LinErr   LinRes
   1      1.3e-08   0.5000000    1.3e-08   26   13   13  1.7e-15  1.8e-15
Solution error estimates for segregated groups
1.1e-05, 6.5e-05, 0.00076, 6.3e-09
Residual error estimates for segregated groups
0.0041, 8.7, 1.2e+03, 0.21
Solution time: 160 s. (2 minutes, 40 seconds)
Physical memory: 4.57 GB
Virtual memory: 25.57 GB
Ended at Jan 31, 2025, 5:21:20 PM.
----- Stationary Solver 1 in study1/Approach Curve Solver (sol1) -------------->
```

d=2445 (su13)

GENERAL

Description	Value
Solution	d=2445

LOG

```
<---- Compile Equations: Stationary in study1/Approach Curve Solver (sol1) -----
Started at Jan 31, 2025, 5:21:21 PM.
Parameter d = 2.4450000000000003E-6 (m).
Geometry shape function: Linear serendipity
Number of vertex elements: 18
Number of boundary elements: 6304
Number of elements: 157276
Minimum element quality: 0.4719
Time: 9 s.
Physical memory: 2.79 GB
Virtual memory: 15.18 GB
Ended at Jan 31, 2025, 5:21:29 PM.
----- Compile Equations: Stationary in study1/Approach Curve Solver (sol1) ---->

<---- Dependent Variables 1 in study1/Approach Curve Solver (sol1) -------------
Started at Jan 31, 2025, 5:21:29 PM.
Solution time: 0 s.
Physical memory: 2.79 GB
Virtual memory: 15.18 GB
Ended at Jan 31, 2025, 5:21:29 PM.
----- Dependent Variables 1 in study1/Approach Curve Solver (sol1) ------------>

<---- Stationary Solver 1 in study1/Approach Curve Solver (sol1) ---------------
Started at Jan 31, 2025, 5:21:30 PM.
Segregated solver
Segregated Step 11 does not have any components solved for.
Number of degrees of freedom solved for: 800283 (plus 12031 internal DOFs).
Segregated solver iteration 1.
Merged variables
Nonsymmetric matrix found.
Scales for dependent variables:
Concentration (comp1.Cl): 1.4e+02
Concentration (comp1.K): 1.4e+02
Electric potential (comp1.V): 1.9e+02
Orthonormal null-space function used.
Iter      SolEst     Damping    Stepsize #Res #Jac #Sol   LinErr   LinRes
   1        0.83   0.3500000        0.83    2    1    1  1.9e-11  2.8e-15
   2        0.81   0.3500000        0.81    3    2    2  1.8e-11  2.7e-15
   3         0.8   0.3500000         0.8    4    3    3  5.8e-11  2.7e-15
Velocity u2, Pressure p
Nonsymmetric matrix found.
Scales for dependent variables:
Pressure (comp1.p): 1
Velocity field (spatial frame) (comp1.u2): 0.00019
Orthonormal null-space function used.
Iter      SolEst     Damping    Stepsize #Res #Jac #Sol   LinErr   LinRes
   1         1.1   0.5000000         1.1    2    1    1  1.9e-15  2.3e-15
Displacement field
Nonsymmetric matrix found.
Scales for dependent variables:
Auxiliary pressure (comp1.solid.lemm1.pw): 1
Displacement field (comp1.u): 2.8e-18
Orthonormal null-space function used.
Iter      SolEst     Damping    Stepsize #Res #Jac #Sol   LinErr   LinRes
   1         1.9   0.5000000         1.9    2    1    1  1.1e-13  1.5e-12
Spatial mesh displacement
```

```
Symmetric matrices found.
Scales for dependent variables:
Spatial mesh displacement (comp1.spatial.disp): 1.2e-09
Orthonormal null-space function used.
Iter      SolEst     Damping    Stepsize #Res #Jac #Sol   LinErr   LinRes
   1     5.7e-07   0.5000000     5.7e-07    2    1    1  1.7e-15  1.9e-15
Solution error estimates for segregated groups
1.4, 0.54, 0.95, 2.8e-07
Residual error estimates for segregated groups
7.9e+03, 8.3e+04, 2.2e+04, 0.17
Segregated solver iteration 2.
Merged variables
Scales for dependent variables:
Concentration (comp1.Cl): 1.4e+02
Concentration (comp1.K): 1.4e+02
Electric potential (comp1.V): 0.064
Iter      SolEst     Damping    Stepsize #Res #Jac #Sol   LinErr   LinRes
   1        0.79   0.3500000        0.79    6    4    4  9.1e-14    3e-15
   2        0.77   0.3500000        0.77    7    5    5  7.6e-14  2.8e-15
   3        0.76   0.3500000        0.76    8    6    6  3.9e-14  2.9e-15
Velocity u2, Pressure p
Scales for dependent variables:
Pressure (comp1.p): 3.5e+02
Velocity field (spatial frame) (comp1.u2): 0.00071
Iter      SolEst     Damping    Stepsize #Res #Jac #Sol   LinErr   LinRes
   1        0.36   0.5000000        0.36    4    2    2  1.8e-15  7.9e-15
Displacement field
Iter      SolEst     Damping    Stepsize #Res #Jac #Sol   LinErr   LinRes
   1        0.95   0.5000000        0.95    4    2    2  9.6e-14  1.5e-12
Spatial mesh displacement
Iter      SolEst     Damping    Stepsize #Res #Jac #Sol   LinErr   LinRes
   1     8.5e-07   0.5000000     8.5e-07    4    2    2  1.4e-15  1.9e-15
Solution error estimates for segregated groups
1.3, 0.18, 0.48, 4.3e-07
Residual error estimates for segregated groups
8.2e+02, 1.4e+04, 2.2e+04, 0.19
Segregated solver iteration 3.
Merged variables
Iter      SolEst     Damping    Stepsize #Res #Jac #Sol   LinErr   LinRes
   1        0.75   0.3500000        0.75   10    7    7  8.3e-14  2.7e-15
   2        0.74   0.3500000        0.74   11    8    8  8.5e-14    3e-15
   3        0.72   0.3500000        0.72   12    9    9  6.4e-14  2.8e-15
Velocity u2, Pressure p
Iter      SolEst     Damping    Stepsize #Res #Jac #Sol   LinErr   LinRes
   1        0.15   0.5000000        0.15    6    3    3    3e-15  7.8e-15
Displacement field
Iter      SolEst     Damping    Stepsize #Res #Jac #Sol   LinErr   LinRes
   1        0.52   0.5000000        0.52    6    3    3  6.4e-14  1.5e-12
Spatial mesh displacement
Iter      SolEst     Damping    Stepsize #Res #Jac #Sol   LinErr   LinRes
   1     8.5e-07   0.5000000     8.5e-07    6    3    3  1.4e-15  1.9e-15
Solution error estimates for segregated groups
1.2, 0.076, 0.26, 4.3e-07
Residual error estimates for segregated groups
2.3e+02, 7.7e+03, 1.6e+04, 0.2
Segregated solver iteration 4.
```

```
Merged variables
Iter      SolEst     Damping    Stepsize #Res #Jac #Sol   LinErr   LinRes
   1        0.71   0.3500000        0.71   14   10   10  7.6e-14    3e-15
   2        0.69   0.3500000        0.69   15   11   11  5.9e-14    3e-15
   3        0.61   0.3500000        0.61   16   12   12  7.9e-14  2.7e-15
Velocity u2, Pressure p
Iter      SolEst     Damping    Stepsize #Res #Jac #Sol   LinErr   LinRes
   1       0.071   0.5000000       0.071    8    4    4  7.4e-15  7.9e-15
Displacement field
Iter      SolEst     Damping    Stepsize #Res #Jac #Sol   LinErr   LinRes
   1        0.29   0.5000000        0.29    8    4    4  6.8e-14  1.5e-12
Spatial mesh displacement
Iter      SolEst     Damping    Stepsize #Res #Jac #Sol   LinErr   LinRes
   1     7.1e-07   0.5000000     7.1e-07    8    4    4  1.4e-15  1.8e-15
Solution error estimates for segregated groups
1, 0.036, 0.15, 3.6e-07
Residual error estimates for segregated groups
85, 4.1e+03, 1.1e+04, 0.19
Segregated solver iteration 5.
Merged variables
Iter      SolEst     Damping    Stepsize #Res #Jac #Sol   LinErr   LinRes
   1        0.42   0.3500000        0.42   18   13   13  1.4e-13    4e-15
   2        0.28   0.3500000        0.28   19   14   14  1.4e-13  3.1e-15
   3        0.19   0.3500000        0.19   20   15   15  5.7e-14  3.1e-15
Velocity u2, Pressure p
Iter      SolEst     Damping    Stepsize #Res #Jac #Sol   LinErr   LinRes
   1       0.035   0.5000000       0.035   10    5    5  2.7e-15  7.9e-15
Displacement field
Iter      SolEst     Damping    Stepsize #Res #Jac #Sol   LinErr   LinRes
   1        0.17   0.5000000        0.17   10    5    5  9.3e-14  1.5e-12
Spatial mesh displacement
Iter      SolEst     Damping    Stepsize #Res #Jac #Sol   LinErr   LinRes
   1     5.3e-07   0.5000000     5.3e-07   10    5    5  1.6e-15  1.8e-15
Solution error estimates for segregated groups
0.32, 0.017, 0.084, 2.7e-07
Residual error estimates for segregated groups
33, 2.1e+03, 6.9e+03, 0.19
Segregated solver iteration 6.
Merged variables
Iter      SolEst     Damping    Stepsize #Res #Jac #Sol   LinErr   LinRes
   1        0.12   0.3500000        0.12   22   16   16  1.4e-13  2.9e-15
   2        0.08   0.3500000        0.08   23   17   17  7.4e-14    4e-15
   3       0.052   0.3500000       0.052   24   18   18  7.1e-14  3.8e-15
Velocity u2, Pressure p
Iter      SolEst     Damping    Stepsize #Res #Jac #Sol   LinErr   LinRes
   1       0.017   0.5000000       0.017   12    6    6  3.1e-15    8e-15
Displacement field
Iter      SolEst     Damping    Stepsize #Res #Jac #Sol   LinErr   LinRes
   1       0.095   0.5000000       0.095   12    6    6  2.4e-14  1.5e-12
Spatial mesh displacement
Iter      SolEst     Damping    Stepsize #Res #Jac #Sol   LinErr   LinRes
   1     3.7e-07   0.5000000     3.7e-07   12    6    6  1.7e-15  1.8e-15
Solution error estimates for segregated groups
0.089, 0.0085, 0.048, 1.9e-07
Residual error estimates for segregated groups
12, 1.1e+03, 4.2e+03, 0.21
```

```
Segregated solver iteration 7.
Merged variables
Iter      SolEst     Damping    Stepsize #Res #Jac #Sol   LinErr   LinRes
   1       0.034   0.3500000       0.034   26   19   19  8.5e-14  2.9e-15
   2       0.022   0.3500000       0.022   27   20   20  5.2e-14  2.8e-15
   3       0.014   0.3500000       0.014   28   21   21    1e-13  2.7e-15
Velocity u2, Pressure p
Iter      SolEst     Damping    Stepsize #Res #Jac #Sol   LinErr   LinRes
   1      0.0084   0.5000000      0.0084   14    7    7  2.5e-15    8e-15
Displacement field
Iter      SolEst     Damping    Stepsize #Res #Jac #Sol   LinErr   LinRes
   1       0.054   0.5000000       0.054   14    7    7  6.3e-14  1.5e-12
Spatial mesh displacement
Iter      SolEst     Damping    Stepsize #Res #Jac #Sol   LinErr   LinRes
   1     2.5e-07   0.5000000     2.5e-07   14    7    7  1.5e-15  1.8e-15
Solution error estimates for segregated groups
0.025, 0.0042, 0.027, 1.2e-07
Residual error estimates for segregated groups
4.2, 5.3e+02, 2.7e+03, 0.21
Segregated solver iteration 8.
Merged variables
Iter      SolEst     Damping    Stepsize #Res #Jac #Sol   LinErr   LinRes
   1      0.0094   0.3500000      0.0094   30   22   22  7.2e-14  2.8e-15
   2      0.0061   0.3500000      0.0061   31   23   23  4.7e-14  2.7e-15
   3       0.004   0.3500000       0.004   32   24   24  1.1e-13  2.7e-15
Velocity u2, Pressure p
Iter      SolEst     Damping    Stepsize #Res #Jac #Sol   LinErr   LinRes
   1      0.0042   0.5000000      0.0042   16    8    8  2.5e-15  7.9e-15
Displacement field
Iter      SolEst     Damping    Stepsize #Res #Jac #Sol   LinErr   LinRes
   1        0.03   0.5000000        0.03   16    8    8  6.3e-14  1.5e-12
Spatial mesh displacement
Iter      SolEst     Damping    Stepsize #Res #Jac #Sol   LinErr   LinRes
   1     1.6e-07   0.5000000     1.6e-07   16    8    8  1.4e-15  1.9e-15
Solution error estimates for segregated groups
0.0068, 0.0021, 0.015, 8e-08
Residual error estimates for segregated groups
1.4, 2.7e+02, 1.8e+03, 0.19
Segregated solver iteration 9.
Merged variables
Iter      SolEst     Damping    Stepsize #Res #Jac #Sol   LinErr   LinRes
   1      0.0026   0.3500000      0.0026   34   25   25  5.4e-14  2.8e-15
   2      0.0017   0.3500000      0.0017   35   26   26  6.8e-14  2.9e-15
   3      0.0011   0.3500000      0.0011   36   27   27  4.3e-14  2.8e-15
Velocity u2, Pressure p
Iter      SolEst     Damping    Stepsize #Res #Jac #Sol   LinErr   LinRes
   1      0.0021   0.5000000      0.0021   18    9    9  2.6e-15  7.9e-15
Displacement field
Iter      SolEst     Damping    Stepsize #Res #Jac #Sol   LinErr   LinRes
   1       0.017   0.5000000       0.017   18    9    9  6.2e-14  1.5e-12
Spatial mesh displacement
Iter      SolEst     Damping    Stepsize #Res #Jac #Sol   LinErr   LinRes
   1       1e-07   0.5000000       1e-07   18    9    9  1.4e-15  1.8e-15
Solution error estimates for segregated groups
0.0019, 0.001, 0.0085, 5e-08
Residual error estimates for segregated groups
```

```
0.45, 1.3e+02, 1.4e+03, 0.21
Segregated solver iteration 10.
Merged variables
Iter      SolEst     Damping    Stepsize #Res #Jac #Sol   LinErr   LinRes
   1     0.00071   0.3500000     0.00071   38   28   28  5.8e-14  2.7e-15
   2     0.00046   0.3500000     0.00046   39   29   29  7.5e-14  2.8e-15
   3      0.0003   0.3500000      0.0003   40   30   30  6.8e-14  2.7e-15
Velocity u2, Pressure p
Iter      SolEst     Damping    Stepsize #Res #Jac #Sol   LinErr   LinRes
   1       0.001   0.5000000       0.001   20   10   10  3.4e-15    8e-15
Displacement field
Iter      SolEst     Damping    Stepsize #Res #Jac #Sol   LinErr   LinRes
   1      0.0094   0.5000000      0.0094   20   10   10  7.3e-14  1.5e-12
Spatial mesh displacement
Iter      SolEst     Damping    Stepsize #Res #Jac #Sol   LinErr   LinRes
   1     6.1e-08   0.5000000     6.1e-08   20   10   10  1.9e-15  1.8e-15
Solution error estimates for segregated groups
0.00051, 0.00052, 0.0047, 3.1e-08
Residual error estimates for segregated groups
0.14, 67, 1.2e+03, 0.2
Segregated solver iteration 11.
Merged variables
Iter      SolEst     Damping    Stepsize #Res #Jac #Sol   LinErr   LinRes
   1     0.00019   0.3500000     0.00019   42   31   31  1.4e-13  2.8e-15
   2     0.00013   0.3500000     0.00013   43   32   32    1e-13  2.8e-15
   3     8.2e-05   0.3500000     8.2e-05   44   33   33    9e-14  2.7e-15
Velocity u2, Pressure p
Iter      SolEst     Damping    Stepsize #Res #Jac #Sol   LinErr   LinRes
   1     0.00052   0.5000000     0.00052   22   11   11    3e-15  7.9e-15
Displacement field
Iter      SolEst     Damping    Stepsize #Res #Jac #Sol   LinErr   LinRes
   1      0.0051   0.5000000      0.0051   22   11   11    5e-14  1.5e-12
Spatial mesh displacement
Iter      SolEst     Damping    Stepsize #Res #Jac #Sol   LinErr   LinRes
   1     3.7e-08   0.5000000     3.7e-08   22   11   11  1.2e-15  1.9e-15
Solution error estimates for segregated groups
0.00014, 0.00026, 0.0026, 1.8e-08
Residual error estimates for segregated groups
0.044, 34, 1.2e+03, 0.2
Segregated solver iteration 12.
Merged variables
Iter      SolEst     Damping    Stepsize #Res #Jac #Sol   LinErr   LinRes
   1     5.3e-05   0.3500000     5.3e-05   46   34   34  8.5e-14  2.8e-15
   2     3.5e-05   0.3500000     3.5e-05   47   35   35  6.4e-14  2.8e-15
   3     2.3e-05   0.3500000     2.3e-05   48   36   36  7.5e-14  2.7e-15
Velocity u2, Pressure p
Iter      SolEst     Damping    Stepsize #Res #Jac #Sol   LinErr   LinRes
   1     0.00026   0.5000000     0.00026   24   12   12    3e-15  7.7e-15
Displacement field
Iter      SolEst     Damping    Stepsize #Res #Jac #Sol   LinErr   LinRes
   1      0.0028   0.5000000      0.0028   24   12   12    8e-14  1.5e-12
Spatial mesh displacement
Iter      SolEst     Damping    Stepsize #Res #Jac #Sol   LinErr   LinRes
   1     2.2e-08   0.5000000     2.2e-08   24   12   12  1.6e-15  1.9e-15
Solution error estimates for segregated groups
3.9e-05, 0.00013, 0.0014, 1.1e-08
```

```
Residual error estimates for segregated groups
0.013, 17, 1.2e+03, 0.19
Segregated solver iteration 13.
Merged variables
Iter      SolEst     Damping    Stepsize #Res #Jac #Sol   LinErr   LinRes
   1     1.5e-05   0.3500000     1.5e-05   50   37   37  9.9e-14  2.8e-15
   2     9.5e-06   0.3500000     9.5e-06   51   38   38  4.4e-14  2.7e-15
   3     6.2e-06   0.3500000     6.2e-06   52   39   39    1e-13  2.9e-15
Velocity u2, Pressure p
Iter      SolEst     Damping    Stepsize #Res #Jac #Sol   LinErr   LinRes
   1     0.00013   0.5000000     0.00013   26   13   13  2.7e-15  7.9e-15
Displacement field
Iter      SolEst     Damping    Stepsize #Res #Jac #Sol   LinErr   LinRes
   1      0.0015   0.5000000      0.0015   26   13   13  5.7e-14  1.5e-12
Spatial mesh displacement
Iter      SolEst     Damping    Stepsize #Res #Jac #Sol   LinErr   LinRes
   1     1.3e-08   0.5000000     1.3e-08   26   13   13    2e-15  1.8e-15
Solution error estimates for segregated groups
1.1e-05, 6.5e-05, 0.00076, 6.3e-09
Residual error estimates for segregated groups
0.0041, 8.4, 1.2e+03, 0.18
Solution time: 163 s. (2 minutes, 43 seconds)
Physical memory: 4.51 GB
Virtual memory: 25.8 GB
Ended at Jan 31, 2025, 5:24:12 PM.
----- Stationary Solver 1 in study1/Approach Curve Solver (sol1) -------------->
```

d=2440 (su14)

GENERAL

Description	Value
Solution	d=2440

LOG

```
<---- Compile Equations: Stationary in study1/Approach Curve Solver (sol1) -----
Started at Jan 31, 2025, 5:24:12 PM.
Parameter d = 2.4400000000000004E-6 (m).
Geometry shape function: Linear serendipity
Number of vertex elements: 18
Number of boundary elements: 6300
Number of elements: 157268
Minimum element quality: 0.4745
Time: 9 s.
Physical memory: 2.86 GB
Virtual memory: 15.25 GB
Ended at Jan 31, 2025, 5:24:21 PM.
----- Compile Equations: Stationary in study1/Approach Curve Solver (sol1) ---->

<---- Dependent Variables 1 in study1/Approach Curve Solver (sol1) -------------
Started at Jan 31, 2025, 5:24:21 PM.
Solution time: 0 s.
Physical memory: 2.86 GB
Virtual memory: 15.25 GB
Ended at Jan 31, 2025, 5:24:21 PM.
----- Dependent Variables 1 in study1/Approach Curve Solver (sol1) ------------>

<---- Stationary Solver 1 in study1/Approach Curve Solver (sol1) ---------------
Started at Jan 31, 2025, 5:24:21 PM.
Segregated solver
Segregated Step 11 does not have any components solved for.
Number of degrees of freedom solved for: 800431 (plus 12023 internal DOFs).
Segregated solver iteration 1.
Merged variables
Nonsymmetric matrix found.
Scales for dependent variables:
Concentration (comp1.Cl): 1.4e+02
Concentration (comp1.K): 1.4e+02
Electric potential (comp1.V): 1.9e+02
Orthonormal null-space function used.
Iter      SolEst     Damping    Stepsize #Res #Jac #Sol   LinErr   LinRes
   1        0.83   0.3500000        0.83    2    1    1  1.3e-11  2.8e-15
   2        0.81   0.3500000        0.81    3    2    2  3.6e-11  2.8e-15
   3         0.8   0.3500000         0.8    4    3    3    1e-10  2.7e-15
Velocity u2, Pressure p
Nonsymmetric matrix found.
Scales for dependent variables:
Pressure (comp1.p): 1
Velocity field (spatial frame) (comp1.u2): 0.00019
Orthonormal null-space function used.
Iter      SolEst     Damping    Stepsize #Res #Jac #Sol   LinErr   LinRes
   1         1.1   0.5000000         1.1    2    1    1  1.6e-15  2.2e-15
Displacement field
Nonsymmetric matrix found.
Scales for dependent variables:
Auxiliary pressure (comp1.solid.lemm1.pw): 1
Displacement field (comp1.u): 2.8e-18
Orthonormal null-space function used.
Iter      SolEst     Damping    Stepsize #Res #Jac #Sol   LinErr   LinRes
   1         1.9   0.5000000         1.9    2    1    1  3.4e-14  1.5e-12
Spatial mesh displacement
```

```
Symmetric matrices found.
Scales for dependent variables:
Spatial mesh displacement (comp1.spatial.disp): 1.2e-09
Orthonormal null-space function used.
Iter      SolEst     Damping    Stepsize #Res #Jac #Sol   LinErr   LinRes
   1     5.7e-07   0.5000000     5.7e-07    2    1    1  2.3e-15  1.9e-15
Solution error estimates for segregated groups
1.4, 0.53, 0.95, 2.9e-07
Residual error estimates for segregated groups
8e+03, 8.3e+04, 2.2e+04, 0.18
Segregated solver iteration 2.
Merged variables
Scales for dependent variables:
Concentration (comp1.Cl): 1.4e+02
Concentration (comp1.K): 1.4e+02
Electric potential (comp1.V): 0.064
Iter      SolEst     Damping    Stepsize #Res #Jac #Sol   LinErr   LinRes
   1        0.79   0.3500000        0.79    6    4    4  1.2e-13  3.8e-15
   2        0.77   0.3500000        0.77    7    5    5  7.9e-14  4.1e-15
   3        0.76   0.3500000        0.76    8    6    6  6.1e-14    3e-15
Velocity u2, Pressure p
Scales for dependent variables:
Pressure (comp1.p): 3.5e+02
Velocity field (spatial frame) (comp1.u2): 0.00071
Iter      SolEst     Damping    Stepsize #Res #Jac #Sol   LinErr   LinRes
   1        0.36   0.5000000        0.36    4    2    2  2.5e-15  7.9e-15
Displacement field
Iter      SolEst     Damping    Stepsize #Res #Jac #Sol   LinErr   LinRes
   1        0.95   0.5000000        0.95    4    2    2  9.2e-14  1.5e-12
Spatial mesh displacement
Iter      SolEst     Damping    Stepsize #Res #Jac #Sol   LinErr   LinRes
   1     8.6e-07   0.5000000     8.6e-07    4    2    2  1.8e-15  1.9e-15
Solution error estimates for segregated groups
1.3, 0.18, 0.48, 4.3e-07
Residual error estimates for segregated groups
8.2e+02, 1.5e+04, 2.2e+04, 0.2
Segregated solver iteration 3.
Merged variables
Iter      SolEst     Damping    Stepsize #Res #Jac #Sol   LinErr   LinRes
   1        0.75   0.3500000        0.75   10    7    7  7.6e-14    3e-15
   2        0.74   0.3500000        0.74   11    8    8  8.7e-14  3.6e-15
   3        0.72   0.3500000        0.72   12    9    9  9.1e-14  3.4e-15
Velocity u2, Pressure p
Iter      SolEst     Damping    Stepsize #Res #Jac #Sol   LinErr   LinRes
   1        0.15   0.5000000        0.15    6    3    3  4.3e-15  7.7e-15
Displacement field
Iter      SolEst     Damping    Stepsize #Res #Jac #Sol   LinErr   LinRes
   1        0.52   0.5000000        0.52    6    3    3  5.8e-14  1.5e-12
Spatial mesh displacement
Iter      SolEst     Damping    Stepsize #Res #Jac #Sol   LinErr   LinRes
   1     8.6e-07   0.5000000     8.6e-07    6    3    3  2.2e-15  1.9e-15
Solution error estimates for segregated groups
1.2, 0.076, 0.26, 4.3e-07
Residual error estimates for segregated groups
2.3e+02, 8.4e+03, 1.6e+04, 0.18
Segregated solver iteration 4.
```

```
Merged variables
Iter      SolEst     Damping    Stepsize #Res #Jac #Sol   LinErr   LinRes
   1        0.71   0.3500000        0.71   14   10   10  1.6e-13  3.3e-15
   2        0.69   0.3500000        0.69   15   11   11  1.4e-13  3.3e-15
   3        0.61   0.3500000        0.61   16   12   12  6.2e-14  3.1e-15
Velocity u2, Pressure p
Iter      SolEst     Damping    Stepsize #Res #Jac #Sol   LinErr   LinRes
   1       0.071   0.5000000       0.071    8    4    4  3.3e-15  7.9e-15
Displacement field
Iter      SolEst     Damping    Stepsize #Res #Jac #Sol   LinErr   LinRes
   1        0.29   0.5000000        0.29    8    4    4  3.5e-14  1.5e-12
Spatial mesh displacement
Iter      SolEst     Damping    Stepsize #Res #Jac #Sol   LinErr   LinRes
   1     7.2e-07   0.5000000     7.2e-07    8    4    4  1.5e-15  1.9e-15
Solution error estimates for segregated groups
1, 0.036, 0.15, 3.6e-07
Residual error estimates for segregated groups
86, 4.4e+03, 1.1e+04, 0.2
Segregated solver iteration 5.
Merged variables
Iter      SolEst     Damping    Stepsize #Res #Jac #Sol   LinErr   LinRes
   1        0.42   0.3500000        0.42   18   13   13  7.7e-14  3.4e-15
   2        0.28   0.3500000        0.28   19   14   14  6.8e-14    3e-15
   3        0.19   0.3500000        0.19   20   15   15    5e-14  3.6e-15
Velocity u2, Pressure p
Iter      SolEst     Damping    Stepsize #Res #Jac #Sol   LinErr   LinRes
   1       0.034   0.5000000       0.034   10    5    5  5.5e-15  7.9e-15
Displacement field
Iter      SolEst     Damping    Stepsize #Res #Jac #Sol   LinErr   LinRes
   1        0.17   0.5000000        0.17   10    5    5  5.7e-14  1.5e-12
Spatial mesh displacement
Iter      SolEst     Damping    Stepsize #Res #Jac #Sol   LinErr   LinRes
   1     5.4e-07   0.5000000     5.4e-07   10    5    5  1.8e-15  1.9e-15
Solution error estimates for segregated groups
0.32, 0.017, 0.084, 2.7e-07
Residual error estimates for segregated groups
33, 2.3e+03, 6.9e+03, 0.2
Segregated solver iteration 6.
Merged variables
Iter      SolEst     Damping    Stepsize #Res #Jac #Sol   LinErr   LinRes
   1        0.12   0.3500000        0.12   22   16   16  1.5e-13  4.1e-15
   2        0.08   0.3500000        0.08   23   17   17  4.6e-14  3.1e-15
   3       0.052   0.3500000       0.052   24   18   18  6.9e-14  3.4e-15
Velocity u2, Pressure p
Iter      SolEst     Damping    Stepsize #Res #Jac #Sol   LinErr   LinRes
   1       0.017   0.5000000       0.017   12    6    6  3.6e-15  7.9e-15
Displacement field
Iter      SolEst     Damping    Stepsize #Res #Jac #Sol   LinErr   LinRes
   1       0.095   0.5000000       0.095   12    6    6  1.2e-13  1.5e-12
Spatial mesh displacement
Iter      SolEst     Damping    Stepsize #Res #Jac #Sol   LinErr   LinRes
   1     3.8e-07   0.5000000     3.8e-07   12    6    6  1.4e-15  1.9e-15
Solution error estimates for segregated groups
0.09, 0.0085, 0.048, 1.9e-07
Residual error estimates for segregated groups
12, 1.2e+03, 4.2e+03, 0.2
```

```
Segregated solver iteration 7.
Merged variables
Iter      SolEst     Damping    Stepsize #Res #Jac #Sol   LinErr   LinRes
   1       0.034   0.3500000       0.034   26   19   19  1.2e-13  3.2e-15
   2       0.022   0.3500000       0.022   27   20   20  6.7e-14  3.5e-15
   3       0.014   0.3500000       0.014   28   21   21  1.1e-13  3.1e-15
Velocity u2, Pressure p
Iter      SolEst     Damping    Stepsize #Res #Jac #Sol   LinErr   LinRes
   1      0.0084   0.5000000      0.0084   14    7    7  5.1e-15  7.9e-15
Displacement field
Iter      SolEst     Damping    Stepsize #Res #Jac #Sol   LinErr   LinRes
   1       0.054   0.5000000       0.054   14    7    7  4.4e-14  1.5e-12
Spatial mesh displacement
Iter      SolEst     Damping    Stepsize #Res #Jac #Sol   LinErr   LinRes
   1     2.5e-07   0.5000000     2.5e-07   14    7    7  1.5e-15  1.9e-15
Solution error estimates for segregated groups
0.025, 0.0042, 0.027, 1.3e-07
Residual error estimates for segregated groups
4.2, 5.8e+02, 2.7e+03, 0.21
Segregated solver iteration 8.
Merged variables
Iter      SolEst     Damping    Stepsize #Res #Jac #Sol   LinErr   LinRes
   1      0.0094   0.3500000      0.0094   30   22   22    6e-14    3e-15
   2      0.0061   0.3500000      0.0061   31   23   23  8.5e-14  4.3e-15
   3       0.004   0.3500000       0.004   32   24   24  6.9e-14    4e-15
Velocity u2, Pressure p
Iter      SolEst     Damping    Stepsize #Res #Jac #Sol   LinErr   LinRes
   1      0.0042   0.5000000      0.0042   16    8    8  4.7e-15  7.9e-15
Displacement field
Iter      SolEst     Damping    Stepsize #Res #Jac #Sol   LinErr   LinRes
   1        0.03   0.5000000        0.03   16    8    8  4.2e-14  1.5e-12
Spatial mesh displacement
Iter      SolEst     Damping    Stepsize #Res #Jac #Sol   LinErr   LinRes
   1     1.6e-07   0.5000000     1.6e-07   16    8    8  1.5e-15  1.9e-15
Solution error estimates for segregated groups
0.0068, 0.0021, 0.015, 8.1e-08
Residual error estimates for segregated groups
1.4, 2.9e+02, 1.8e+03, 0.2
Segregated solver iteration 9.
Merged variables
Iter      SolEst     Damping    Stepsize #Res #Jac #Sol   LinErr   LinRes
   1      0.0026   0.3500000      0.0026   34   25   25  1.1e-13  3.2e-15
   2      0.0017   0.3500000      0.0017   35   26   26  3.8e-14  3.1e-15
   3      0.0011   0.3500000      0.0011   36   27   27  1.6e-13  3.5e-15
Velocity u2, Pressure p
Iter      SolEst     Damping    Stepsize #Res #Jac #Sol   LinErr   LinRes
   1      0.0021   0.5000000      0.0021   18    9    9  2.8e-15  7.8e-15
Displacement field
Iter      SolEst     Damping    Stepsize #Res #Jac #Sol   LinErr   LinRes
   1       0.017   0.5000000       0.017   18    9    9  3.7e-14  1.5e-12
Spatial mesh displacement
Iter      SolEst     Damping    Stepsize #Res #Jac #Sol   LinErr   LinRes
   1       1e-07   0.5000000       1e-07   18    9    9  1.3e-15  1.8e-15
Solution error estimates for segregated groups
0.0019, 0.001, 0.0085, 5e-08
Residual error estimates for segregated groups
```

```
0.45, 1.5e+02, 1.4e+03, 0.19
Segregated solver iteration 10.
Merged variables
Iter      SolEst     Damping    Stepsize #Res #Jac #Sol   LinErr   LinRes
   1     0.00071   0.3500000     0.00071   38   28   28  9.9e-14  3.2e-15
   2     0.00046   0.3500000     0.00046   39   29   29  1.1e-13    3e-15
   3      0.0003   0.3500000      0.0003   40   30   30  6.7e-14    3e-15
Velocity u2, Pressure p
Iter      SolEst     Damping    Stepsize #Res #Jac #Sol   LinErr   LinRes
   1       0.001   0.5000000       0.001   20   10   10  3.6e-15  7.9e-15
Displacement field
Iter      SolEst     Damping    Stepsize #Res #Jac #Sol   LinErr   LinRes
   1      0.0094   0.5000000      0.0094   20   10   10  5.7e-14  1.5e-12
Spatial mesh displacement
Iter      SolEst     Damping    Stepsize #Res #Jac #Sol   LinErr   LinRes
   1     6.2e-08   0.5000000     6.2e-08   20   10   10  1.4e-15  1.9e-15
Solution error estimates for segregated groups
0.00051, 0.00052, 0.0047, 3.1e-08
Residual error estimates for segregated groups
0.14, 73, 1.2e+03, 0.21
Segregated solver iteration 11.
Merged variables
Iter      SolEst     Damping    Stepsize #Res #Jac #Sol   LinErr   LinRes
   1     0.00019   0.3500000     0.00019   42   31   31  1.9e-13  3.5e-15
   2     0.00013   0.3500000     0.00013   43   32   32  8.7e-14  3.4e-15
   3     8.2e-05   0.3500000     8.2e-05   44   33   33  1.1e-13  3.1e-15
Velocity u2, Pressure p
Iter      SolEst     Damping    Stepsize #Res #Jac #Sol   LinErr   LinRes
   1     0.00052   0.5000000     0.00052   22   11   11  2.6e-15  7.8e-15
Displacement field
Iter      SolEst     Damping    Stepsize #Res #Jac #Sol   LinErr   LinRes
   1      0.0051   0.5000000      0.0051   22   11   11  7.1e-14  1.5e-12
Spatial mesh displacement
Iter      SolEst     Damping    Stepsize #Res #Jac #Sol   LinErr   LinRes
   1     3.7e-08   0.5000000     3.7e-08   22   11   11  1.5e-15  1.9e-15
Solution error estimates for segregated groups
0.00014, 0.00026, 0.0026, 1.9e-08
Residual error estimates for segregated groups
0.044, 36, 1.2e+03, 0.21
Segregated solver iteration 12.
Merged variables
Iter      SolEst     Damping    Stepsize #Res #Jac #Sol   LinErr   LinRes
   1     5.3e-05   0.3500000     5.3e-05   46   34   34  7.3e-14  4.6e-15
   2     3.5e-05   0.3500000     3.5e-05   47   35   35  4.4e-14  3.9e-15
   3     2.3e-05   0.3500000     2.3e-05   48   36   36  8.9e-14  3.2e-15
Velocity u2, Pressure p
Iter      SolEst     Damping    Stepsize #Res #Jac #Sol   LinErr   LinRes
   1     0.00026   0.5000000     0.00026   24   12   12  2.2e-15  7.9e-15
Displacement field
Iter      SolEst     Damping    Stepsize #Res #Jac #Sol   LinErr   LinRes
   1      0.0028   0.5000000      0.0028   24   12   12  1.2e-13  1.5e-12
Spatial mesh displacement
Iter      SolEst     Damping    Stepsize #Res #Jac #Sol   LinErr   LinRes
   1     2.2e-08   0.5000000     2.2e-08   24   12   12    2e-15  1.9e-15
Solution error estimates for segregated groups
3.9e-05, 0.00013, 0.0014, 1.1e-08
```

```
Residual error estimates for segregated groups
0.013, 18, 1.2e+03, 0.2
Segregated solver iteration 13.
Merged variables
Iter      SolEst     Damping    Stepsize #Res #Jac #Sol   LinErr   LinRes
   1     1.5e-05   0.3500000     1.5e-05   50   37   37  6.4e-14  3.4e-15
   2     9.5e-06   0.3500000     9.5e-06   51   38   38  1.1e-13  3.6e-15
   3     6.2e-06   0.3500000     6.2e-06   52   39   39  6.3e-14    3e-15
Velocity u2, Pressure p
Iter      SolEst     Damping    Stepsize #Res #Jac #Sol   LinErr   LinRes
   1     0.00013   0.5000000     0.00013   26   13   13  3.4e-15  7.8e-15
Displacement field
Iter      SolEst     Damping    Stepsize #Res #Jac #Sol   LinErr   LinRes
   1      0.0015   0.5000000      0.0015   26   13   13  3.1e-14  1.5e-12
Spatial mesh displacement
Iter      SolEst     Damping    Stepsize #Res #Jac #Sol   LinErr   LinRes
   1     1.3e-08   0.5000000     1.3e-08   26   13   13  1.7e-15  1.9e-15
Solution error estimates for segregated groups
1.1e-05, 6.5e-05, 0.00076, 6.4e-09
Residual error estimates for segregated groups
0.0041, 9.1, 1.2e+03, 0.19
Solution time: 155 s. (2 minutes, 35 seconds)
Physical memory: 4.5 GB
Virtual memory: 25.54 GB
Ended at Jan 31, 2025, 5:26:57 PM.
----- Stationary Solver 1 in study1/Approach Curve Solver (sol1) -------------->
```

d=2435 (su15)

GENERAL

Description	Value
Solution	d=2435

LOG

```
<---- Compile Equations: Stationary in study1/Approach Curve Solver (sol1) -----
Started at Jan 31, 2025, 5:26:57 PM.
Parameter d = 2.435E-6 (m).
Geometry shape function: Linear serendipity
Number of vertex elements: 18
Number of boundary elements: 6298
Number of elements: 157402
Minimum element quality: 0.4882
Time: 9 s.
Physical memory: 2.86 GB
Virtual memory: 15.24 GB
Ended at Jan 31, 2025, 5:27:06 PM.
----- Compile Equations: Stationary in study1/Approach Curve Solver (sol1) ---->

<---- Dependent Variables 1 in study1/Approach Curve Solver (sol1) -------------
Started at Jan 31, 2025, 5:27:06 PM.
Solution time: 0 s.
Physical memory: 2.86 GB
Virtual memory: 15.24 GB
Ended at Jan 31, 2025, 5:27:06 PM.
----- Dependent Variables 1 in study1/Approach Curve Solver (sol1) ------------>

<---- Stationary Solver 1 in study1/Approach Curve Solver (sol1) ---------------
Started at Jan 31, 2025, 5:27:06 PM.
Segregated solver
Segregated Step 11 does not have any components solved for.
Number of degrees of freedom solved for: 800863 (plus 12019 internal DOFs).
Segregated solver iteration 1.
Merged variables
Nonsymmetric matrix found.
Scales for dependent variables:
Concentration (comp1.Cl): 1.4e+02
Concentration (comp1.K): 1.4e+02
Electric potential (comp1.V): 1.9e+02
Orthonormal null-space function used.
Iter      SolEst     Damping    Stepsize #Res #Jac #Sol   LinErr   LinRes
   1        0.83   0.3500000        0.83    2    1    1  1.4e-11  2.8e-15
   2        0.81   0.3500000        0.81    3    2    2  2.5e-11  2.7e-15
   3         0.8   0.3500000         0.8    4    3    3  5.4e-11  2.7e-15
Velocity u2, Pressure p
Nonsymmetric matrix found.
Scales for dependent variables:
Pressure (comp1.p): 1
Velocity field (spatial frame) (comp1.u2): 0.00019
Orthonormal null-space function used.
Iter      SolEst     Damping    Stepsize #Res #Jac #Sol   LinErr   LinRes
   1         1.1   0.5000000         1.1    2    1    1  1.7e-15  2.3e-15
Displacement field
Nonsymmetric matrix found.
Scales for dependent variables:
Auxiliary pressure (comp1.solid.lemm1.pw): 1
Displacement field (comp1.u): 2.7e-18
Orthonormal null-space function used.
Iter      SolEst     Damping    Stepsize #Res #Jac #Sol   LinErr   LinRes
   1         1.9   0.5000000         1.9    2    1    1  1.4e-13  1.5e-12
Spatial mesh displacement
```

```
Symmetric matrices found.
Scales for dependent variables:
Spatial mesh displacement (comp1.spatial.disp): 1.2e-09
Orthonormal null-space function used.
Iter      SolEst     Damping    Stepsize #Res #Jac #Sol   LinErr   LinRes
   1     5.7e-07   0.5000000     5.7e-07    2    1    1  1.3e-15  1.9e-15
Solution error estimates for segregated groups
1.4, 0.53, 0.95, 2.9e-07
Residual error estimates for segregated groups
8e+03, 8.3e+04, 2.2e+04, 0.18
Segregated solver iteration 2.
Merged variables
Scales for dependent variables:
Concentration (comp1.Cl): 1.4e+02
Concentration (comp1.K): 1.4e+02
Electric potential (comp1.V): 0.064
Iter      SolEst     Damping    Stepsize #Res #Jac #Sol   LinErr   LinRes
   1        0.79   0.3500000        0.79    6    4    4  4.8e-14    4e-15
   2        0.77   0.3500000        0.77    7    5    5  7.8e-14  8.1e-15
   3        0.76   0.3500000        0.76    8    6    6  8.8e-14  3.6e-15
Velocity u2, Pressure p
Scales for dependent variables:
Pressure (comp1.p): 3.5e+02
Velocity field (spatial frame) (comp1.u2): 0.00071
Iter      SolEst     Damping    Stepsize #Res #Jac #Sol   LinErr   LinRes
   1        0.36   0.5000000        0.36    4    2    2  2.5e-15  7.9e-15
Displacement field
Iter      SolEst     Damping    Stepsize #Res #Jac #Sol   LinErr   LinRes
   1        0.95   0.5000000        0.95    4    2    2  2.5e-14  1.5e-12
Spatial mesh displacement
Iter      SolEst     Damping    Stepsize #Res #Jac #Sol   LinErr   LinRes
   1     8.6e-07   0.5000000     8.6e-07    4    2    2  1.3e-15  1.8e-15
Solution error estimates for segregated groups
1.3, 0.18, 0.48, 4.3e-07
Residual error estimates for segregated groups
8.2e+02, 1.5e+04, 2.2e+04, 0.2
Segregated solver iteration 3.
Merged variables
Iter      SolEst     Damping    Stepsize #Res #Jac #Sol   LinErr   LinRes
   1        0.75   0.3500000        0.75   10    7    7  1.5e-13  4.1e-15
   2        0.74   0.3500000        0.74   11    8    8  7.7e-14  3.1e-15
   3        0.72   0.3500000        0.72   12    9    9  5.8e-14    3e-15
Velocity u2, Pressure p
Iter      SolEst     Damping    Stepsize #Res #Jac #Sol   LinErr   LinRes
   1        0.15   0.5000000        0.15    6    3    3    2e-15  7.9e-15
Displacement field
Iter      SolEst     Damping    Stepsize #Res #Jac #Sol   LinErr   LinRes
   1        0.52   0.5000000        0.52    6    3    3  4.5e-14  1.5e-12
Spatial mesh displacement
Iter      SolEst     Damping    Stepsize #Res #Jac #Sol   LinErr   LinRes
   1     8.6e-07   0.5000000     8.6e-07    6    3    3    2e-15  1.8e-15
Solution error estimates for segregated groups
1.2, 0.076, 0.26, 4.3e-07
Residual error estimates for segregated groups
2.3e+02, 8.2e+03, 1.6e+04, 0.2
Segregated solver iteration 4.
```

```
Merged variables
Iter      SolEst     Damping    Stepsize #Res #Jac #Sol   LinErr   LinRes
   1        0.71   0.3500000        0.71   14   10   10  5.4e-14  2.9e-15
   2        0.69   0.3500000        0.69   15   11   11  5.4e-14  3.6e-15
   3        0.61   0.3500000        0.61   16   12   12    1e-13  2.9e-15
Velocity u2, Pressure p
Iter      SolEst     Damping    Stepsize #Res #Jac #Sol   LinErr   LinRes
   1       0.071   0.5000000       0.071    8    4    4  2.3e-15    8e-15
Displacement field
Iter      SolEst     Damping    Stepsize #Res #Jac #Sol   LinErr   LinRes
   1        0.29   0.5000000        0.29    8    4    4  1.2e-13  1.5e-12
Spatial mesh displacement
Iter      SolEst     Damping    Stepsize #Res #Jac #Sol   LinErr   LinRes
   1     7.2e-07   0.5000000     7.2e-07    8    4    4  1.6e-15  1.8e-15
Solution error estimates for segregated groups
1, 0.036, 0.15, 3.6e-07
Residual error estimates for segregated groups
85, 4.3e+03, 1.1e+04, 0.21
Segregated solver iteration 5.
Merged variables
Iter      SolEst     Damping    Stepsize #Res #Jac #Sol   LinErr   LinRes
   1        0.42   0.3500000        0.42   18   13   13  5.7e-14  3.2e-15
   2        0.28   0.3500000        0.28   19   14   14  8.3e-14  3.1e-15
   3        0.19   0.3500000        0.19   20   15   15  5.5e-14  5.2e-15
Velocity u2, Pressure p
Iter      SolEst     Damping    Stepsize #Res #Jac #Sol   LinErr   LinRes
   1       0.035   0.5000000       0.035   10    5    5  2.7e-15    8e-15
Displacement field
Iter      SolEst     Damping    Stepsize #Res #Jac #Sol   LinErr   LinRes
   1        0.17   0.5000000        0.17   10    5    5  9.4e-14  1.5e-12
Spatial mesh displacement
Iter      SolEst     Damping    Stepsize #Res #Jac #Sol   LinErr   LinRes
   1     5.4e-07   0.5000000     5.4e-07   10    5    5  1.4e-15  1.8e-15
Solution error estimates for segregated groups
0.32, 0.017, 0.084, 2.7e-07
Residual error estimates for segregated groups
33, 2.2e+03, 6.9e+03, 0.19
Segregated solver iteration 6.
Merged variables
Iter      SolEst     Damping    Stepsize #Res #Jac #Sol   LinErr   LinRes
   1        0.12   0.3500000        0.12   22   16   16  5.3e-14  2.8e-15
   2        0.08   0.3500000        0.08   23   17   17  7.2e-14  2.9e-15
   3       0.052   0.3500000       0.052   24   18   18  6.3e-14  3.4e-15
Velocity u2, Pressure p
Iter      SolEst     Damping    Stepsize #Res #Jac #Sol   LinErr   LinRes
   1       0.017   0.5000000       0.017   12    6    6  2.3e-15  7.9e-15
Displacement field
Iter      SolEst     Damping    Stepsize #Res #Jac #Sol   LinErr   LinRes
   1       0.095   0.5000000       0.095   12    6    6  2.1e-14  1.5e-12
Spatial mesh displacement
Iter      SolEst     Damping    Stepsize #Res #Jac #Sol   LinErr   LinRes
   1     3.8e-07   0.5000000     3.8e-07   12    6    6  1.6e-15  1.9e-15
Solution error estimates for segregated groups
0.089, 0.0085, 0.048, 1.9e-07
Residual error estimates for segregated groups
12, 1.1e+03, 4.3e+03, 0.2
```

```
Segregated solver iteration 7.
Merged variables
Iter      SolEst     Damping    Stepsize #Res #Jac #Sol   LinErr   LinRes
   1       0.034   0.3500000       0.034   26   19   19  9.5e-14  3.7e-15
   2       0.022   0.3500000       0.022   27   20   20  1.3e-13  3.5e-15
   3       0.014   0.3500000       0.014   28   21   21  8.7e-14  4.1e-15
Velocity u2, Pressure p
Iter      SolEst     Damping    Stepsize #Res #Jac #Sol   LinErr   LinRes
   1      0.0084   0.5000000      0.0084   14    7    7  2.3e-15  7.9e-15
Displacement field
Iter      SolEst     Damping    Stepsize #Res #Jac #Sol   LinErr   LinRes
   1       0.054   0.5000000       0.054   14    7    7  9.8e-14  1.5e-12
Spatial mesh displacement
Iter      SolEst     Damping    Stepsize #Res #Jac #Sol   LinErr   LinRes
   1     2.5e-07   0.5000000     2.5e-07   14    7    7  1.6e-15  1.9e-15
Solution error estimates for segregated groups
0.025, 0.0042, 0.027, 1.3e-07
Residual error estimates for segregated groups
4.2, 5.7e+02, 2.7e+03, 0.18
Segregated solver iteration 8.
Merged variables
Iter      SolEst     Damping    Stepsize #Res #Jac #Sol   LinErr   LinRes
   1      0.0094   0.3500000      0.0094   30   22   22  9.3e-14  2.9e-15
   2      0.0061   0.3500000      0.0061   31   23   23    5e-14  2.8e-15
   3       0.004   0.3500000       0.004   32   24   24  4.5e-14  3.1e-15
Velocity u2, Pressure p
Iter      SolEst     Damping    Stepsize #Res #Jac #Sol   LinErr   LinRes
   1      0.0042   0.5000000      0.0042   16    8    8  2.6e-15  7.7e-15
Displacement field
Iter      SolEst     Damping    Stepsize #Res #Jac #Sol   LinErr   LinRes
   1        0.03   0.5000000        0.03   16    8    8    8e-14  1.5e-12
Spatial mesh displacement
Iter      SolEst     Damping    Stepsize #Res #Jac #Sol   LinErr   LinRes
   1     1.6e-07   0.5000000     1.6e-07   16    8    8  1.8e-15  1.9e-15
Solution error estimates for segregated groups
0.0068, 0.0021, 0.015, 8.1e-08
Residual error estimates for segregated groups
1.4, 2.8e+02, 1.8e+03, 0.2
Segregated solver iteration 9.
Merged variables
Iter      SolEst     Damping    Stepsize #Res #Jac #Sol   LinErr   LinRes
   1      0.0026   0.3500000      0.0026   34   25   25  8.1e-14  2.9e-15
   2      0.0017   0.3500000      0.0017   35   26   26  9.2e-14  3.7e-15
   3      0.0011   0.3500000      0.0011   36   27   27  5.2e-14  2.8e-15
Velocity u2, Pressure p
Iter      SolEst     Damping    Stepsize #Res #Jac #Sol   LinErr   LinRes
   1      0.0021   0.5000000      0.0021   18    9    9  4.8e-15  7.8e-15
Displacement field
Iter      SolEst     Damping    Stepsize #Res #Jac #Sol   LinErr   LinRes
   1       0.017   0.5000000       0.017   18    9    9    4e-14  1.5e-12
Spatial mesh displacement
Iter      SolEst     Damping    Stepsize #Res #Jac #Sol   LinErr   LinRes
   1       1e-07   0.5000000       1e-07   18    9    9  1.7e-15  1.9e-15
Solution error estimates for segregated groups
0.0019, 0.001, 0.0085, 5.1e-08
Residual error estimates for segregated groups
```

```
0.45, 1.4e+02, 1.4e+03, 0.19
Segregated solver iteration 10.
Merged variables
Iter      SolEst     Damping    Stepsize #Res #Jac #Sol   LinErr   LinRes
   1     0.00071   0.3500000     0.00071   38   28   28  5.3e-14  2.9e-15
   2     0.00046   0.3500000     0.00046   39   29   29  5.5e-14    3e-15
   3      0.0003   0.3500000      0.0003   40   30   30  7.5e-14    3e-15
Velocity u2, Pressure p
Iter      SolEst     Damping    Stepsize #Res #Jac #Sol   LinErr   LinRes
   1       0.001   0.5000000       0.001   20   10   10    4e-15    8e-15
Displacement field
Iter      SolEst     Damping    Stepsize #Res #Jac #Sol   LinErr   LinRes
   1      0.0094   0.5000000      0.0094   20   10   10  4.7e-14  1.5e-12
Spatial mesh displacement
Iter      SolEst     Damping    Stepsize #Res #Jac #Sol   LinErr   LinRes
   1     6.2e-08   0.5000000     6.2e-08   20   10   10  1.6e-15  1.9e-15
Solution error estimates for segregated groups
0.00051, 0.00052, 0.0047, 3.1e-08
Residual error estimates for segregated groups
0.14, 71, 1.2e+03, 0.19
Segregated solver iteration 11.
Merged variables
Iter      SolEst     Damping    Stepsize #Res #Jac #Sol   LinErr   LinRes
   1     0.00019   0.3500000     0.00019   42   31   31  6.5e-14  2.8e-15
   2     0.00013   0.3500000     0.00013   43   32   32    9e-14    3e-15
   3     8.2e-05   0.3500000     8.2e-05   44   33   33  5.3e-14  2.8e-15
Velocity u2, Pressure p
Iter      SolEst     Damping    Stepsize #Res #Jac #Sol   LinErr   LinRes
   1     0.00052   0.5000000     0.00052   22   11   11  2.4e-15    8e-15
Displacement field
Iter      SolEst     Damping    Stepsize #Res #Jac #Sol   LinErr   LinRes
   1      0.0051   0.5000000      0.0051   22   11   11    4e-14  1.5e-12
Spatial mesh displacement
Iter      SolEst     Damping    Stepsize #Res #Jac #Sol   LinErr   LinRes
   1     3.7e-08   0.5000000     3.7e-08   22   11   11    2e-15  1.9e-15
Solution error estimates for segregated groups
0.00014, 0.00026, 0.0026, 1.9e-08
Residual error estimates for segregated groups
0.044, 36, 1.2e+03, 0.2
Segregated solver iteration 12.
Merged variables
Iter      SolEst     Damping    Stepsize #Res #Jac #Sol   LinErr   LinRes
   1     5.3e-05   0.3500000     5.3e-05   46   34   34  8.5e-14    3e-15
   2     3.5e-05   0.3500000     3.5e-05   47   35   35  5.5e-14    5e-15
   3     2.3e-05   0.3500000     2.3e-05   48   36   36  1.2e-13  3.1e-15
Velocity u2, Pressure p
Iter      SolEst     Damping    Stepsize #Res #Jac #Sol   LinErr   LinRes
   1     0.00026   0.5000000     0.00026   24   12   12  2.1e-15  7.8e-15
Displacement field
Iter      SolEst     Damping    Stepsize #Res #Jac #Sol   LinErr   LinRes
   1      0.0028   0.5000000      0.0028   24   12   12  4.3e-14  1.5e-12
Spatial mesh displacement
Iter      SolEst     Damping    Stepsize #Res #Jac #Sol   LinErr   LinRes
   1     2.2e-08   0.5000000     2.2e-08   24   12   12  1.3e-15  1.9e-15
Solution error estimates for segregated groups
3.9e-05, 0.00013, 0.0014, 1.1e-08
```

```
Residual error estimates for segregated groups
0.013, 18, 1.2e+03, 0.19
Segregated solver iteration 13.
Merged variables
Iter      SolEst     Damping    Stepsize #Res #Jac #Sol   LinErr   LinRes
   1     1.5e-05   0.3500000     1.5e-05   50   37   37    9e-14  2.9e-15
   2     9.5e-06   0.3500000     9.5e-06   51   38   38  1.3e-13  3.2e-15
   3     6.2e-06   0.3500000     6.2e-06   52   39   39  4.2e-14  5.9e-15
Velocity u2, Pressure p
Iter      SolEst     Damping    Stepsize #Res #Jac #Sol   LinErr   LinRes
   1     0.00013   0.5000000     0.00013   26   13   13  3.2e-15  7.9e-15
Displacement field
Iter      SolEst     Damping    Stepsize #Res #Jac #Sol   LinErr   LinRes
   1      0.0015   0.5000000      0.0015   26   13   13  7.1e-14  1.5e-12
Spatial mesh displacement
Iter      SolEst     Damping    Stepsize #Res #Jac #Sol   LinErr   LinRes
   1     1.3e-08   0.5000000     1.3e-08   26   13   13  1.4e-15  1.8e-15
Solution error estimates for segregated groups
1.1e-05, 6.5e-05, 0.00076, 6.4e-09
Residual error estimates for segregated groups
0.0041, 8.9, 1.2e+03, 0.2
Solution time: 159 s. (2 minutes, 39 seconds)
Physical memory: 4.57 GB
Virtual memory: 25.6 GB
Ended at Jan 31, 2025, 5:29:45 PM.
----- Stationary Solver 1 in study1/Approach Curve Solver (sol1) -------------->
```

d=2430 (su16)

GENERAL

Description	Value
Solution	d=2430

LOG

```
<---- Compile Equations: Stationary in study1/Approach Curve Solver (sol1) -----
Started at Jan 31, 2025, 5:29:45 PM.
Parameter d = 2.43E-6 (m).
Geometry shape function: Linear serendipity
Number of vertex elements: 18
Number of boundary elements: 6295
Number of elements: 156819
Minimum element quality: 0.4495
Time: 9 s.
Physical memory: 2.76 GB
Virtual memory: 15.13 GB
Ended at Jan 31, 2025, 5:29:53 PM.
----- Compile Equations: Stationary in study1/Approach Curve Solver (sol1) ---->

<---- Dependent Variables 1 in study1/Approach Curve Solver (sol1) -------------
Started at Jan 31, 2025, 5:29:53 PM.
Solution time: 0 s.
Physical memory: 2.76 GB
Virtual memory: 15.13 GB
Ended at Jan 31, 2025, 5:29:53 PM.
----- Dependent Variables 1 in study1/Approach Curve Solver (sol1) ------------>

<---- Stationary Solver 1 in study1/Approach Curve Solver (sol1) ---------------
Started at Jan 31, 2025, 5:29:54 PM.
Segregated solver
Segregated Step 11 does not have any components solved for.
Number of degrees of freedom solved for: 797991 (plus 12013 internal DOFs).
Segregated solver iteration 1.
Merged variables
Nonsymmetric matrix found.
Scales for dependent variables:
Concentration (comp1.Cl): 1.4e+02
Concentration (comp1.K): 1.4e+02
Electric potential (comp1.V): 1.9e+02
Orthonormal null-space function used.
Iter      SolEst     Damping    Stepsize #Res #Jac #Sol   LinErr   LinRes
   1        0.83   0.3500000        0.83    2    1    1  3.8e-11  2.7e-15
   2        0.81   0.3500000        0.81    3    2    2  4.4e-11    3e-15
   3         0.8   0.3500000         0.8    4    3    3  1.8e-11    3e-15
Velocity u2, Pressure p
Nonsymmetric matrix found.
Scales for dependent variables:
Pressure (comp1.p): 1
Velocity field (spatial frame) (comp1.u2): 0.00019
Orthonormal null-space function used.
Iter      SolEst     Damping    Stepsize #Res #Jac #Sol   LinErr   LinRes
   1         1.1   0.5000000         1.1    2    1    1  1.7e-15  2.2e-15
Displacement field
Nonsymmetric matrix found.
Scales for dependent variables:
Auxiliary pressure (comp1.solid.lemm1.pw): 1
Displacement field (comp1.u): 2.8e-18
Orthonormal null-space function used.
Iter      SolEst     Damping    Stepsize #Res #Jac #Sol   LinErr   LinRes
   1         1.9   0.5000000         1.9    2    1    1    6e-14  1.5e-12
Spatial mesh displacement
```

```
Symmetric matrices found.
Scales for dependent variables:
Spatial mesh displacement (comp1.spatial.disp): 1.2e-09
Orthonormal null-space function used.
Iter      SolEst     Damping    Stepsize #Res #Jac #Sol   LinErr   LinRes
   1     5.8e-07   0.5000000     5.8e-07    2    1    1  1.4e-15  1.8e-15
Solution error estimates for segregated groups
1.4, 0.54, 0.95, 2.9e-07
Residual error estimates for segregated groups
7.9e+03, 8.3e+04, 2.2e+04, 0.16
Segregated solver iteration 2.
Merged variables
Scales for dependent variables:
Concentration (comp1.Cl): 1.4e+02
Concentration (comp1.K): 1.4e+02
Electric potential (comp1.V): 0.064
Iter      SolEst     Damping    Stepsize #Res #Jac #Sol   LinErr   LinRes
   1        0.79   0.3500000        0.79    6    4    4  8.9e-14  2.8e-15
   2        0.77   0.3500000        0.77    7    5    5    1e-13  3.9e-15
   3        0.76   0.3500000        0.76    8    6    6  1.1e-13  2.9e-15
Velocity u2, Pressure p
Scales for dependent variables:
Pressure (comp1.p): 3.5e+02
Velocity field (spatial frame) (comp1.u2): 0.00071
Iter      SolEst     Damping    Stepsize #Res #Jac #Sol   LinErr   LinRes
   1        0.36   0.5000000        0.36    4    2    2  2.7e-15  7.8e-15
Displacement field
Iter      SolEst     Damping    Stepsize #Res #Jac #Sol   LinErr   LinRes
   1        0.95   0.5000000        0.95    4    2    2  1.4e-13  1.5e-12
Spatial mesh displacement
Iter      SolEst     Damping    Stepsize #Res #Jac #Sol   LinErr   LinRes
   1     8.7e-07   0.5000000     8.7e-07    4    2    2  1.5e-15  1.8e-15
Solution error estimates for segregated groups
1.3, 0.18, 0.48, 4.3e-07
Residual error estimates for segregated groups
8.1e+02, 1.4e+04, 2.2e+04, 0.19
Segregated solver iteration 3.
Merged variables
Iter      SolEst     Damping    Stepsize #Res #Jac #Sol   LinErr   LinRes
   1        0.75   0.3500000        0.75   10    7    7    1e-13    3e-15
   2        0.74   0.3500000        0.74   11    8    8  6.5e-14  3.5e-15
   3        0.72   0.3500000        0.72   12    9    9  6.8e-14  2.8e-15
Velocity u2, Pressure p
Iter      SolEst     Damping    Stepsize #Res #Jac #Sol   LinErr   LinRes
   1        0.15   0.5000000        0.15    6    3    3  2.8e-15  7.8e-15
Displacement field
Iter      SolEst     Damping    Stepsize #Res #Jac #Sol   LinErr   LinRes
   1        0.52   0.5000000        0.52    6    3    3  6.7e-14  1.5e-12
Spatial mesh displacement
Iter      SolEst     Damping    Stepsize #Res #Jac #Sol   LinErr   LinRes
   1     8.7e-07   0.5000000     8.7e-07    6    3    3  1.6e-15  1.8e-15
Solution error estimates for segregated groups
1.2, 0.076, 0.26, 4.3e-07
Residual error estimates for segregated groups
2.3e+02, 8.1e+03, 1.6e+04, 0.2
Segregated solver iteration 4.
```

```
Merged variables
Iter      SolEst     Damping    Stepsize #Res #Jac #Sol   LinErr   LinRes
   1        0.71   0.3500000        0.71   14   10   10  1.2e-13    3e-15
   2        0.69   0.3500000        0.69   15   11   11    1e-13  2.9e-15
   3        0.61   0.3500000        0.61   16   12   12  1.4e-13  3.1e-15
Velocity u2, Pressure p
Iter      SolEst     Damping    Stepsize #Res #Jac #Sol   LinErr   LinRes
   1       0.071   0.5000000       0.071    8    4    4  2.4e-15  7.9e-15
Displacement field
Iter      SolEst     Damping    Stepsize #Res #Jac #Sol   LinErr   LinRes
   1        0.29   0.5000000        0.29    8    4    4  7.1e-14  1.5e-12
Spatial mesh displacement
Iter      SolEst     Damping    Stepsize #Res #Jac #Sol   LinErr   LinRes
   1     7.2e-07   0.5000000     7.2e-07    8    4    4  1.9e-15  1.9e-15
Solution error estimates for segregated groups
1, 0.036, 0.15, 3.6e-07
Residual error estimates for segregated groups
85, 4.3e+03, 1.1e+04, 0.19
Segregated solver iteration 5.
Merged variables
Iter      SolEst     Damping    Stepsize #Res #Jac #Sol   LinErr   LinRes
   1        0.42   0.3500000        0.42   18   13   13  9.4e-14  3.1e-15
   2        0.28   0.3500000        0.28   19   14   14  9.3e-14  3.3e-15
   3        0.19   0.3500000        0.19   20   15   15    5e-14  3.2e-15
Velocity u2, Pressure p
Iter      SolEst     Damping    Stepsize #Res #Jac #Sol   LinErr   LinRes
   1       0.035   0.5000000       0.035   10    5    5  1.9e-15  7.7e-15
Displacement field
Iter      SolEst     Damping    Stepsize #Res #Jac #Sol   LinErr   LinRes
   1        0.17   0.5000000        0.17   10    5    5  6.9e-14  1.5e-12
Spatial mesh displacement
Iter      SolEst     Damping    Stepsize #Res #Jac #Sol   LinErr   LinRes
   1     5.4e-07   0.5000000     5.4e-07   10    5    5  1.7e-15  1.9e-15
Solution error estimates for segregated groups
0.32, 0.017, 0.084, 2.7e-07
Residual error estimates for segregated groups
33, 2.2e+03, 6.9e+03, 0.18
Segregated solver iteration 6.
Merged variables
Iter      SolEst     Damping    Stepsize #Res #Jac #Sol   LinErr   LinRes
   1        0.12   0.3500000        0.12   22   16   16  8.6e-14  3.1e-15
   2        0.08   0.3500000        0.08   23   17   17  7.8e-14    3e-15
   3       0.052   0.3500000       0.052   24   18   18  6.3e-14  3.5e-15
Velocity u2, Pressure p
Iter      SolEst     Damping    Stepsize #Res #Jac #Sol   LinErr   LinRes
   1       0.017   0.5000000       0.017   12    6    6  4.7e-15    8e-15
Displacement field
Iter      SolEst     Damping    Stepsize #Res #Jac #Sol   LinErr   LinRes
   1       0.095   0.5000000       0.095   12    6    6  9.3e-14  1.5e-12
Spatial mesh displacement
Iter      SolEst     Damping    Stepsize #Res #Jac #Sol   LinErr   LinRes
   1     3.8e-07   0.5000000     3.8e-07   12    6    6  1.7e-15  1.9e-15
Solution error estimates for segregated groups
0.089, 0.0085, 0.048, 1.9e-07
Residual error estimates for segregated groups
12, 1.1e+03, 4.2e+03, 0.2
```

```
Segregated solver iteration 7.
Merged variables
Iter      SolEst     Damping    Stepsize #Res #Jac #Sol   LinErr   LinRes
   1       0.034   0.3500000       0.034   26   19   19    1e-13  3.5e-15
   2       0.022   0.3500000       0.022   27   20   20  1.1e-13    3e-15
   3       0.014   0.3500000       0.014   28   21   21  7.8e-14    3e-15
Velocity u2, Pressure p
Iter      SolEst     Damping    Stepsize #Res #Jac #Sol   LinErr   LinRes
   1      0.0084   0.5000000      0.0084   14    7    7    3e-15  7.9e-15
Displacement field
Iter      SolEst     Damping    Stepsize #Res #Jac #Sol   LinErr   LinRes
   1       0.054   0.5000000       0.054   14    7    7  4.9e-14  1.5e-12
Spatial mesh displacement
Iter      SolEst     Damping    Stepsize #Res #Jac #Sol   LinErr   LinRes
   1     2.5e-07   0.5000000     2.5e-07   14    7    7  1.6e-15  1.8e-15
Solution error estimates for segregated groups
0.025, 0.0042, 0.027, 1.3e-07
Residual error estimates for segregated groups
4.2, 5.6e+02, 2.6e+03, 0.2
Segregated solver iteration 8.
Merged variables
Iter      SolEst     Damping    Stepsize #Res #Jac #Sol   LinErr   LinRes
   1      0.0093   0.3500000      0.0093   30   22   22    5e-14  2.9e-15
   2      0.0061   0.3500000      0.0061   31   23   23  7.9e-14  2.9e-15
   3      0.0039   0.3500000      0.0039   32   24   24    1e-13    3e-15
Velocity u2, Pressure p
Iter      SolEst     Damping    Stepsize #Res #Jac #Sol   LinErr   LinRes
   1      0.0042   0.5000000      0.0042   16    8    8    3e-15  7.8e-15
Displacement field
Iter      SolEst     Damping    Stepsize #Res #Jac #Sol   LinErr   LinRes
   1        0.03   0.5000000        0.03   16    8    8  6.8e-14  1.5e-12
Spatial mesh displacement
Iter      SolEst     Damping    Stepsize #Res #Jac #Sol   LinErr   LinRes
   1     1.6e-07   0.5000000     1.6e-07   16    8    8  1.9e-15  1.9e-15
Solution error estimates for segregated groups
0.0068, 0.0021, 0.015, 8.1e-08
Residual error estimates for segregated groups
1.4, 2.8e+02, 1.8e+03, 0.2
Segregated solver iteration 9.
Merged variables
Iter      SolEst     Damping    Stepsize #Res #Jac #Sol   LinErr   LinRes
   1      0.0026   0.3500000      0.0026   34   25   25    5e-14  3.3e-15
   2      0.0017   0.3500000      0.0017   35   26   26  1.4e-13    3e-15
   3      0.0011   0.3500000      0.0011   36   27   27  7.1e-14  3.5e-15
Velocity u2, Pressure p
Iter      SolEst     Damping    Stepsize #Res #Jac #Sol   LinErr   LinRes
   1      0.0021   0.5000000      0.0021   18    9    9    3e-15  7.8e-15
Displacement field
Iter      SolEst     Damping    Stepsize #Res #Jac #Sol   LinErr   LinRes
   1       0.017   0.5000000       0.017   18    9    9  1.3e-13  1.5e-12
Spatial mesh displacement
Iter      SolEst     Damping    Stepsize #Res #Jac #Sol   LinErr   LinRes
   1       1e-07   0.5000000       1e-07   18    9    9  1.5e-15  1.8e-15
Solution error estimates for segregated groups
0.0019, 0.001, 0.0085, 5.1e-08
Residual error estimates for segregated groups
```

```
0.45, 1.4e+02, 1.4e+03, 0.19
Segregated solver iteration 10.
Merged variables
Iter      SolEst     Damping    Stepsize #Res #Jac #Sol   LinErr   LinRes
   1      0.0007   0.3500000      0.0007   38   28   28  1.3e-13  3.1e-15
   2     0.00046   0.3500000     0.00046   39   29   29    5e-14    3e-15
   3      0.0003   0.3500000      0.0003   40   30   30  6.7e-14  3.2e-15
Velocity u2, Pressure p
Iter      SolEst     Damping    Stepsize #Res #Jac #Sol   LinErr   LinRes
   1       0.001   0.5000000       0.001   20   10   10  2.8e-15  7.8e-15
Displacement field
Iter      SolEst     Damping    Stepsize #Res #Jac #Sol   LinErr   LinRes
   1      0.0094   0.5000000      0.0094   20   10   10  1.9e-13  1.5e-12
Spatial mesh displacement
Iter      SolEst     Damping    Stepsize #Res #Jac #Sol   LinErr   LinRes
   1     6.2e-08   0.5000000     6.2e-08   20   10   10  1.4e-15  1.8e-15
Solution error estimates for segregated groups
0.00051, 0.00052, 0.0047, 3.1e-08
Residual error estimates for segregated groups
0.14, 70, 1.2e+03, 0.2
Segregated solver iteration 11.
Merged variables
Iter      SolEst     Damping    Stepsize #Res #Jac #Sol   LinErr   LinRes
   1     0.00019   0.3500000     0.00019   42   31   31  1.2e-13  3.4e-15
   2     0.00013   0.3500000     0.00013   43   32   32  1.2e-13  3.3e-15
   3     8.2e-05   0.3500000     8.2e-05   44   33   33  9.8e-14  3.2e-15
Velocity u2, Pressure p
Iter      SolEst     Damping    Stepsize #Res #Jac #Sol   LinErr   LinRes
   1     0.00052   0.5000000     0.00052   22   11   11  2.8e-15  7.9e-15
Displacement field
Iter      SolEst     Damping    Stepsize #Res #Jac #Sol   LinErr   LinRes
   1      0.0051   0.5000000      0.0051   22   11   11  4.6e-14  1.5e-12
Spatial mesh displacement
Iter      SolEst     Damping    Stepsize #Res #Jac #Sol   LinErr   LinRes
   1     3.7e-08   0.5000000     3.7e-08   22   11   11  1.7e-15  1.9e-15
Solution error estimates for segregated groups
0.00014, 0.00026, 0.0026, 1.9e-08
Residual error estimates for segregated groups
0.044, 35, 1.2e+03, 0.2
Segregated solver iteration 12.
Merged variables
Iter      SolEst     Damping    Stepsize #Res #Jac #Sol   LinErr   LinRes
   1     5.3e-05   0.3500000     5.3e-05   46   34   34  8.3e-14    3e-15
   2     3.5e-05   0.3500000     3.5e-05   47   35   35  5.7e-14    3e-15
   3     2.2e-05   0.3500000     2.2e-05   48   36   36  7.8e-14    3e-15
Velocity u2, Pressure p
Iter      SolEst     Damping    Stepsize #Res #Jac #Sol   LinErr   LinRes
   1     0.00026   0.5000000     0.00026   24   12   12  2.7e-15  7.7e-15
Displacement field
Iter      SolEst     Damping    Stepsize #Res #Jac #Sol   LinErr   LinRes
   1      0.0028   0.5000000      0.0028   24   12   12  8.9e-14  1.5e-12
Spatial mesh displacement
Iter      SolEst     Damping    Stepsize #Res #Jac #Sol   LinErr   LinRes
   1     2.2e-08   0.5000000     2.2e-08   24   12   12  1.6e-15  1.8e-15
Solution error estimates for segregated groups
3.9e-05, 0.00013, 0.0014, 1.1e-08
```

```
Residual error estimates for segregated groups
0.013, 18, 1.2e+03, 0.18
Segregated solver iteration 13.
Merged variables
Iter      SolEst     Damping    Stepsize #Res #Jac #Sol   LinErr   LinRes
   1     1.5e-05   0.3500000     1.5e-05   50   37   37  6.7e-14    3e-15
   2     9.5e-06   0.3500000     9.5e-06   51   38   38  9.2e-14  3.5e-15
   3     6.2e-06   0.3500000     6.2e-06   52   39   39  1.4e-13    3e-15
Velocity u2, Pressure p
Iter      SolEst     Damping    Stepsize #Res #Jac #Sol   LinErr   LinRes
   1     0.00013   0.5000000     0.00013   26   13   13    4e-15    8e-15
Displacement field
Iter      SolEst     Damping    Stepsize #Res #Jac #Sol   LinErr   LinRes
   1      0.0015   0.5000000      0.0015   26   13   13  8.8e-14  1.5e-12
Spatial mesh displacement
Iter      SolEst     Damping    Stepsize #Res #Jac #Sol   LinErr   LinRes
   1     1.3e-08   0.5000000     1.3e-08   26   13   13    2e-15  1.8e-15
Solution error estimates for segregated groups
1.1e-05, 6.5e-05, 0.00076, 6.4e-09
Residual error estimates for segregated groups
0.0041, 8.8, 1.2e+03, 0.2
Solution time: 161 s. (2 minutes, 41 seconds)
Physical memory: 4.58 GB
Virtual memory: 25.51 GB
Ended at Jan 31, 2025, 5:32:35 PM.
----- Stationary Solver 1 in study1/Approach Curve Solver (sol1) -------------->
```

d=2425 (su17)

GENERAL

Description	Value
Solution	d=2425

LOG

```
<---- Compile Equations: Stationary in study1/Approach Curve Solver (sol1) -----
Started at Jan 31, 2025, 5:32:35 PM.
Parameter d = 2.425E-6 (m).
Geometry shape function: Linear serendipity
Number of vertex elements: 18
Number of boundary elements: 6293
Number of elements: 156861
Minimum element quality: 0.4524
Time: 9 s.
Physical memory: 2.81 GB
Virtual memory: 15.18 GB
Ended at Jan 31, 2025, 5:32:44 PM.
----- Compile Equations: Stationary in study1/Approach Curve Solver (sol1) ---->

<---- Dependent Variables 1 in study1/Approach Curve Solver (sol1) -------------
Started at Jan 31, 2025, 5:32:44 PM.
Solution time: 0 s.
Physical memory: 2.81 GB
Virtual memory: 15.18 GB
Ended at Jan 31, 2025, 5:32:44 PM.
----- Dependent Variables 1 in study1/Approach Curve Solver (sol1) ------------>

<---- Stationary Solver 1 in study1/Approach Curve Solver (sol1) ---------------
Started at Jan 31, 2025, 5:32:44 PM.
Segregated solver
Segregated Step 11 does not have any components solved for.
Number of degrees of freedom solved for: 798097 (plus 12009 internal DOFs).
Segregated solver iteration 1.
Merged variables
Nonsymmetric matrix found.
Scales for dependent variables:
Concentration (comp1.Cl): 1.4e+02
Concentration (comp1.K): 1.4e+02
Electric potential (comp1.V): 1.9e+02
Orthonormal null-space function used.
Iter      SolEst     Damping    Stepsize #Res #Jac #Sol   LinErr   LinRes
   1        0.83   0.3500000        0.83    2    1    1  4.1e-11  2.8e-15
   2        0.81   0.3500000        0.81    3    2    2  2.7e-11  2.8e-15
   3         0.8   0.3500000         0.8    4    3    3    4e-11  2.8e-15
Velocity u2, Pressure p
Nonsymmetric matrix found.
Scales for dependent variables:
Pressure (comp1.p): 1
Velocity field (spatial frame) (comp1.u2): 0.00019
Orthonormal null-space function used.
Iter      SolEst     Damping    Stepsize #Res #Jac #Sol   LinErr   LinRes
   1         1.1   0.5000000         1.1    2    1    1  1.8e-15  2.3e-15
Displacement field
Nonsymmetric matrix found.
Scales for dependent variables:
Auxiliary pressure (comp1.solid.lemm1.pw): 1
Displacement field (comp1.u): 2.7e-18
Orthonormal null-space function used.
Iter      SolEst     Damping    Stepsize #Res #Jac #Sol   LinErr   LinRes
   1         1.9   0.5000000         1.9    2    1    1  4.9e-14  1.5e-12
Spatial mesh displacement
```

```
Symmetric matrices found.
Scales for dependent variables:
Spatial mesh displacement (comp1.spatial.disp): 1.2e-09
Orthonormal null-space function used.
Iter      SolEst     Damping    Stepsize #Res #Jac #Sol   LinErr   LinRes
   1     5.8e-07   0.5000000     5.8e-07    2    1    1  1.6e-15  1.9e-15
Solution error estimates for segregated groups
1.4, 0.54, 0.95, 2.9e-07
Residual error estimates for segregated groups
7.9e+03, 8.3e+04, 2.2e+04, 0.17
Segregated solver iteration 2.
Merged variables
Scales for dependent variables:
Concentration (comp1.Cl): 1.4e+02
Concentration (comp1.K): 1.4e+02
Electric potential (comp1.V): 0.064
Iter      SolEst     Damping    Stepsize #Res #Jac #Sol   LinErr   LinRes
   1        0.79   0.3500000        0.79    6    4    4  6.2e-14  3.2e-15
   2        0.77   0.3500000        0.77    7    5    5  6.2e-14  3.3e-15
   3        0.76   0.3500000        0.76    8    6    6    5e-14  2.9e-15
Velocity u2, Pressure p
Scales for dependent variables:
Pressure (comp1.p): 3.5e+02
Velocity field (spatial frame) (comp1.u2): 0.00071
Iter      SolEst     Damping    Stepsize #Res #Jac #Sol   LinErr   LinRes
   1        0.36   0.5000000        0.36    4    2    2  3.6e-15  7.9e-15
Displacement field
Iter      SolEst     Damping    Stepsize #Res #Jac #Sol   LinErr   LinRes
   1        0.95   0.5000000        0.95    4    2    2  1.7e-13  1.5e-12
Spatial mesh displacement
Iter      SolEst     Damping    Stepsize #Res #Jac #Sol   LinErr   LinRes
   1     8.7e-07   0.5000000     8.7e-07    4    2    2  1.7e-15  1.9e-15
Solution error estimates for segregated groups
1.3, 0.18, 0.48, 4.3e-07
Residual error estimates for segregated groups
8.1e+02, 1.4e+04, 2.2e+04, 0.18
Segregated solver iteration 3.
Merged variables
Iter      SolEst     Damping    Stepsize #Res #Jac #Sol   LinErr   LinRes
   1        0.75   0.3500000        0.75   10    7    7  4.4e-14    3e-15
   2        0.74   0.3500000        0.74   11    8    8  6.6e-14    3e-15
   3        0.72   0.3500000        0.72   12    9    9  8.7e-14  2.8e-15
Velocity u2, Pressure p
Iter      SolEst     Damping    Stepsize #Res #Jac #Sol   LinErr   LinRes
   1        0.15   0.5000000        0.15    6    3    3  3.7e-15  7.7e-15
Displacement field
Iter      SolEst     Damping    Stepsize #Res #Jac #Sol   LinErr   LinRes
   1        0.52   0.5000000        0.52    6    3    3  2.7e-14  1.5e-12
Spatial mesh displacement
Iter      SolEst     Damping    Stepsize #Res #Jac #Sol   LinErr   LinRes
   1     8.7e-07   0.5000000     8.7e-07    6    3    3  1.9e-15  1.8e-15
Solution error estimates for segregated groups
1.2, 0.076, 0.26, 4.3e-07
Residual error estimates for segregated groups
2.3e+02, 8e+03, 1.6e+04, 0.2
Segregated solver iteration 4.
```

```
Merged variables
Iter      SolEst     Damping    Stepsize #Res #Jac #Sol   LinErr   LinRes
   1        0.71   0.3500000        0.71   14   10   10  4.1e-14  3.2e-15
   2        0.69   0.3500000        0.69   15   11   11  8.4e-14  2.8e-15
   3        0.61   0.3500000        0.61   16   12   12  7.5e-14  3.2e-15
Velocity u2, Pressure p
Iter      SolEst     Damping    Stepsize #Res #Jac #Sol   LinErr   LinRes
   1       0.071   0.5000000       0.071    8    4    4  2.7e-15  7.8e-15
Displacement field
Iter      SolEst     Damping    Stepsize #Res #Jac #Sol   LinErr   LinRes
   1        0.29   0.5000000        0.29    8    4    4  5.1e-14  1.5e-12
Spatial mesh displacement
Iter      SolEst     Damping    Stepsize #Res #Jac #Sol   LinErr   LinRes
   1     7.2e-07   0.5000000     7.2e-07    8    4    4  1.7e-15  1.8e-15
Solution error estimates for segregated groups
1, 0.036, 0.15, 3.6e-07
Residual error estimates for segregated groups
85, 4.2e+03, 1.1e+04, 0.19
Segregated solver iteration 5.
Merged variables
Iter      SolEst     Damping    Stepsize #Res #Jac #Sol   LinErr   LinRes
   1        0.42   0.3500000        0.42   18   13   13  6.9e-14  3.4e-15
   2        0.28   0.3500000        0.28   19   14   14  6.5e-14    3e-15
   3        0.18   0.3500000        0.18   20   15   15    6e-14  2.8e-15
Velocity u2, Pressure p
Iter      SolEst     Damping    Stepsize #Res #Jac #Sol   LinErr   LinRes
   1       0.035   0.5000000       0.035   10    5    5  3.4e-15    8e-15
Displacement field
Iter      SolEst     Damping    Stepsize #Res #Jac #Sol   LinErr   LinRes
   1        0.17   0.5000000        0.17   10    5    5  7.3e-14  1.5e-12
Spatial mesh displacement
Iter      SolEst     Damping    Stepsize #Res #Jac #Sol   LinErr   LinRes
   1     5.4e-07   0.5000000     5.4e-07   10    5    5  1.5e-15  1.9e-15
Solution error estimates for segregated groups
0.32, 0.017, 0.084, 2.7e-07
Residual error estimates for segregated groups
33, 2.2e+03, 6.9e+03, 0.2
Segregated solver iteration 6.
Merged variables
Iter      SolEst     Damping    Stepsize #Res #Jac #Sol   LinErr   LinRes
   1        0.12   0.3500000        0.12   22   16   16    1e-13    3e-15
   2        0.08   0.3500000        0.08   23   17   17  1.1e-13  2.8e-15
   3       0.052   0.3500000       0.052   24   18   18  1.4e-13  3.1e-15
Velocity u2, Pressure p
Iter      SolEst     Damping    Stepsize #Res #Jac #Sol   LinErr   LinRes
   1       0.017   0.5000000       0.017   12    6    6  3.7e-15  7.7e-15
Displacement field
Iter      SolEst     Damping    Stepsize #Res #Jac #Sol   LinErr   LinRes
   1       0.095   0.5000000       0.095   12    6    6    9e-14  1.5e-12
Spatial mesh displacement
Iter      SolEst     Damping    Stepsize #Res #Jac #Sol   LinErr   LinRes
   1     3.8e-07   0.5000000     3.8e-07   12    6    6  1.3e-15  1.9e-15
Solution error estimates for segregated groups
0.089, 0.0085, 0.048, 1.9e-07
Residual error estimates for segregated groups
12, 1.1e+03, 4.2e+03, 0.2
```

```
Segregated solver iteration 7.
Merged variables
Iter      SolEst     Damping    Stepsize #Res #Jac #Sol   LinErr   LinRes
   1       0.034   0.3500000       0.034   26   19   19  4.9e-14    3e-15
   2       0.022   0.3500000       0.022   27   20   20  9.6e-14  2.9e-15
   3       0.014   0.3500000       0.014   28   21   21  1.2e-13  2.9e-15
Velocity u2, Pressure p
Iter      SolEst     Damping    Stepsize #Res #Jac #Sol   LinErr   LinRes
   1      0.0084   0.5000000      0.0084   14    7    7  3.3e-15  7.8e-15
Displacement field
Iter      SolEst     Damping    Stepsize #Res #Jac #Sol   LinErr   LinRes
   1       0.054   0.5000000       0.054   14    7    7  4.7e-14  1.5e-12
Spatial mesh displacement
Iter      SolEst     Damping    Stepsize #Res #Jac #Sol   LinErr   LinRes
   1     2.5e-07   0.5000000     2.5e-07   14    7    7  2.3e-15  1.8e-15
Solution error estimates for segregated groups
0.025, 0.0042, 0.027, 1.3e-07
Residual error estimates for segregated groups
4.2, 5.5e+02, 2.7e+03, 0.2
Segregated solver iteration 8.
Merged variables
Iter      SolEst     Damping    Stepsize #Res #Jac #Sol   LinErr   LinRes
   1      0.0093   0.3500000      0.0093   30   22   22  5.4e-14  2.8e-15
   2      0.0061   0.3500000      0.0061   31   23   23  1.3e-13  2.9e-15
   3      0.0039   0.3500000      0.0039   32   24   24  1.1e-13    3e-15
Velocity u2, Pressure p
Iter      SolEst     Damping    Stepsize #Res #Jac #Sol   LinErr   LinRes
   1      0.0042   0.5000000      0.0042   16    8    8  5.2e-15  7.8e-15
Displacement field
Iter      SolEst     Damping    Stepsize #Res #Jac #Sol   LinErr   LinRes
   1        0.03   0.5000000        0.03   16    8    8  4.6e-14  1.5e-12
Spatial mesh displacement
Iter      SolEst     Damping    Stepsize #Res #Jac #Sol   LinErr   LinRes
   1     1.6e-07   0.5000000     1.6e-07   16    8    8  1.7e-15  1.9e-15
Solution error estimates for segregated groups
0.0068, 0.0021, 0.015, 8.2e-08
Residual error estimates for segregated groups
1.4, 2.8e+02, 1.8e+03, 0.19
Segregated solver iteration 9.
Merged variables
Iter      SolEst     Damping    Stepsize #Res #Jac #Sol   LinErr   LinRes
   1      0.0026   0.3500000      0.0026   34   25   25  8.1e-14  3.1e-15
   2      0.0017   0.3500000      0.0017   35   26   26  1.3e-13  2.9e-15
   3      0.0011   0.3500000      0.0011   36   27   27  1.4e-13  3.1e-15
Velocity u2, Pressure p
Iter      SolEst     Damping    Stepsize #Res #Jac #Sol   LinErr   LinRes
   1      0.0021   0.5000000      0.0021   18    9    9  2.7e-15  7.9e-15
Displacement field
Iter      SolEst     Damping    Stepsize #Res #Jac #Sol   LinErr   LinRes
   1       0.017   0.5000000       0.017   18    9    9  1.1e-13  1.5e-12
Spatial mesh displacement
Iter      SolEst     Damping    Stepsize #Res #Jac #Sol   LinErr   LinRes
   1       1e-07   0.5000000       1e-07   18    9    9  1.4e-15  1.9e-15
Solution error estimates for segregated groups
0.0019, 0.001, 0.0085, 5.1e-08
Residual error estimates for segregated groups
```

```
0.45, 1.4e+02, 1.4e+03, 0.2
Segregated solver iteration 10.
Merged variables
Iter      SolEst     Damping    Stepsize #Res #Jac #Sol   LinErr   LinRes
   1      0.0007   0.3500000      0.0007   38   28   28    9e-14  2.8e-15
   2     0.00046   0.3500000     0.00046   39   29   29  9.1e-14    3e-15
   3      0.0003   0.3500000      0.0003   40   30   30  1.1e-13  3.1e-15
Velocity u2, Pressure p
Iter      SolEst     Damping    Stepsize #Res #Jac #Sol   LinErr   LinRes
   1       0.001   0.5000000       0.001   20   10   10  2.3e-15  7.9e-15
Displacement field
Iter      SolEst     Damping    Stepsize #Res #Jac #Sol   LinErr   LinRes
   1      0.0094   0.5000000      0.0094   20   10   10  4.9e-14  1.5e-12
Spatial mesh displacement
Iter      SolEst     Damping    Stepsize #Res #Jac #Sol   LinErr   LinRes
   1     6.2e-08   0.5000000     6.2e-08   20   10   10  1.4e-15  1.9e-15
Solution error estimates for segregated groups
0.00051, 0.00052, 0.0047, 3.1e-08
Residual error estimates for segregated groups
0.14, 69, 1.2e+03, 0.2
Segregated solver iteration 11.
Merged variables
Iter      SolEst     Damping    Stepsize #Res #Jac #Sol   LinErr   LinRes
   1     0.00019   0.3500000     0.00019   42   31   31  5.9e-14  2.8e-15
   2     0.00013   0.3500000     0.00013   43   32   32  9.4e-14  2.9e-15
   3     8.2e-05   0.3500000     8.2e-05   44   33   33  1.4e-13  2.8e-15
Velocity u2, Pressure p
Iter      SolEst     Damping    Stepsize #Res #Jac #Sol   LinErr   LinRes
   1     0.00052   0.5000000     0.00052   22   11   11  3.4e-15  7.8e-15
Displacement field
Iter      SolEst     Damping    Stepsize #Res #Jac #Sol   LinErr   LinRes
   1      0.0051   0.5000000      0.0051   22   11   11  1.7e-13  1.5e-12
Spatial mesh displacement
Iter      SolEst     Damping    Stepsize #Res #Jac #Sol   LinErr   LinRes
   1     3.7e-08   0.5000000     3.7e-08   22   11   11  1.1e-15  1.9e-15
Solution error estimates for segregated groups
0.00014, 0.00026, 0.0026, 1.9e-08
Residual error estimates for segregated groups
0.044, 35, 1.2e+03, 0.19
Segregated solver iteration 12.
Merged variables
Iter      SolEst     Damping    Stepsize #Res #Jac #Sol   LinErr   LinRes
   1     5.3e-05   0.3500000     5.3e-05   46   34   34  7.4e-14  2.9e-15
   2     3.5e-05   0.3500000     3.5e-05   47   35   35  4.4e-14    3e-15
   3     2.2e-05   0.3500000     2.2e-05   48   36   36  4.7e-14  2.9e-15
Velocity u2, Pressure p
Iter      SolEst     Damping    Stepsize #Res #Jac #Sol   LinErr   LinRes
   1     0.00026   0.5000000     0.00026   24   12   12  2.4e-15  7.8e-15
Displacement field
Iter      SolEst     Damping    Stepsize #Res #Jac #Sol   LinErr   LinRes
   1      0.0028   0.5000000      0.0028   24   12   12  9.1e-14  1.5e-12
Spatial mesh displacement
Iter      SolEst     Damping    Stepsize #Res #Jac #Sol   LinErr   LinRes
   1     2.2e-08   0.5000000     2.2e-08   24   12   12  1.6e-15  1.9e-15
Solution error estimates for segregated groups
3.9e-05, 0.00013, 0.0014, 1.1e-08
```

```
Residual error estimates for segregated groups
0.013, 17, 1.2e+03, 0.2
Segregated solver iteration 13.
Merged variables
Iter      SolEst     Damping    Stepsize #Res #Jac #Sol   LinErr   LinRes
   1     1.5e-05   0.3500000     1.5e-05   50   37   37  7.1e-14    3e-15
   2     9.5e-06   0.3500000     9.5e-06   51   38   38  1.2e-13  3.1e-15
   3     6.2e-06   0.3500000     6.2e-06   52   39   39  6.9e-14  2.9e-15
Velocity u2, Pressure p
Iter      SolEst     Damping    Stepsize #Res #Jac #Sol   LinErr   LinRes
   1     0.00013   0.5000000     0.00013   26   13   13  3.1e-15  7.7e-15
Displacement field
Iter      SolEst     Damping    Stepsize #Res #Jac #Sol   LinErr   LinRes
   1      0.0015   0.5000000      0.0015   26   13   13  1.7e-13  1.5e-12
Spatial mesh displacement
Iter      SolEst     Damping    Stepsize #Res #Jac #Sol   LinErr   LinRes
   1     1.3e-08   0.5000000     1.3e-08   26   13   13  1.7e-15  1.9e-15
Solution error estimates for segregated groups
1.1e-05, 6.5e-05, 0.00076, 6.4e-09
Residual error estimates for segregated groups
0.0041, 8.7, 1.2e+03, 0.19
Solution time: 158 s. (2 minutes, 38 seconds)
Physical memory: 4.59 GB
Virtual memory: 25.56 GB
Ended at Jan 31, 2025, 5:35:22 PM.
----- Stationary Solver 1 in study1/Approach Curve Solver (sol1) -------------->
```

d=2420 (su18)

GENERAL

Description	Value
Solution	d=2420

LOG

```
<---- Compile Equations: Stationary in study1/Approach Curve Solver (sol1) -----
Started at Jan 31, 2025, 5:35:22 PM.
Parameter d = 2.42E-6 (m).
Geometry shape function: Linear serendipity
Number of vertex elements: 18
Number of boundary elements: 6290
Number of elements: 156720
Minimum element quality: 0.4875
Time: 9 s.
Physical memory: 2.81 GB
Virtual memory: 15.17 GB
Ended at Jan 31, 2025, 5:35:31 PM.
----- Compile Equations: Stationary in study1/Approach Curve Solver (sol1) ---->

<---- Dependent Variables 1 in study1/Approach Curve Solver (sol1) -------------
Started at Jan 31, 2025, 5:35:31 PM.
Solution time: 0 s.
Physical memory: 2.81 GB
Virtual memory: 15.17 GB
Ended at Jan 31, 2025, 5:35:31 PM.
----- Dependent Variables 1 in study1/Approach Curve Solver (sol1) ------------>

<---- Stationary Solver 1 in study1/Approach Curve Solver (sol1) ---------------
Started at Jan 31, 2025, 5:35:31 PM.
Segregated solver
Segregated Step 11 does not have any components solved for.
Number of degrees of freedom solved for: 797404 (plus 12003 internal DOFs).
Segregated solver iteration 1.
Merged variables
Nonsymmetric matrix found.
Scales for dependent variables:
Concentration (comp1.Cl): 1.4e+02
Concentration (comp1.K): 1.4e+02
Electric potential (comp1.V): 1.9e+02
Orthonormal null-space function used.
Iter      SolEst     Damping    Stepsize #Res #Jac #Sol   LinErr   LinRes
   1        0.83   0.3500000        0.83    2    1    1  3.4e-11  2.7e-15
   2        0.81   0.3500000        0.81    3    2    2  1.6e-11  2.7e-15
   3         0.8   0.3500000         0.8    4    3    3  1.4e-11  2.7e-15
Velocity u2, Pressure p
Nonsymmetric matrix found.
Scales for dependent variables:
Pressure (comp1.p): 1
Velocity field (spatial frame) (comp1.u2): 0.00019
Orthonormal null-space function used.
Iter      SolEst     Damping    Stepsize #Res #Jac #Sol   LinErr   LinRes
   1         1.1   0.5000000         1.1    2    1    1  1.5e-15  2.2e-15
Displacement field
Nonsymmetric matrix found.
Scales for dependent variables:
Auxiliary pressure (comp1.solid.lemm1.pw): 1
Displacement field (comp1.u): 2.8e-18
Orthonormal null-space function used.
Iter      SolEst     Damping    Stepsize #Res #Jac #Sol   LinErr   LinRes
   1         1.9   0.5000000         1.9    2    1    1  5.7e-14  1.5e-12
Spatial mesh displacement
```

```
Symmetric matrices found.
Scales for dependent variables:
Spatial mesh displacement (comp1.spatial.disp): 1.2e-09
Orthonormal null-space function used.
Iter      SolEst     Damping    Stepsize #Res #Jac #Sol   LinErr   LinRes
   1     5.8e-07   0.5000000     5.8e-07    2    1    1  1.9e-15  1.9e-15
Solution error estimates for segregated groups
1.4, 0.54, 0.95, 2.9e-07
Residual error estimates for segregated groups
7.9e+03, 8.3e+04, 2.2e+04, 0.18
Segregated solver iteration 2.
Merged variables
Scales for dependent variables:
Concentration (comp1.Cl): 1.4e+02
Concentration (comp1.K): 1.4e+02
Electric potential (comp1.V): 0.064
Iter      SolEst     Damping    Stepsize #Res #Jac #Sol   LinErr   LinRes
   1        0.79   0.3500000        0.79    6    4    4  6.7e-14    3e-15
   2        0.77   0.3500000        0.77    7    5    5  1.2e-13  4.1e-15
   3        0.76   0.3500000        0.76    8    6    6  6.4e-14  3.2e-15
Velocity u2, Pressure p
Scales for dependent variables:
Pressure (comp1.p): 3.5e+02
Velocity field (spatial frame) (comp1.u2): 0.00071
Iter      SolEst     Damping    Stepsize #Res #Jac #Sol   LinErr   LinRes
   1        0.36   0.5000000        0.36    4    2    2  2.7e-15  7.8e-15
Displacement field
Iter      SolEst     Damping    Stepsize #Res #Jac #Sol   LinErr   LinRes
   1        0.95   0.5000000        0.95    4    2    2  4.3e-14  1.5e-12
Spatial mesh displacement
Iter      SolEst     Damping    Stepsize #Res #Jac #Sol   LinErr   LinRes
   1     8.7e-07   0.5000000     8.7e-07    4    2    2  1.6e-15  1.8e-15
Solution error estimates for segregated groups
1.3, 0.18, 0.48, 4.4e-07
Residual error estimates for segregated groups
8.1e+02, 1.4e+04, 2.2e+04, 0.18
Segregated solver iteration 3.
Merged variables
Iter      SolEst     Damping    Stepsize #Res #Jac #Sol   LinErr   LinRes
   1        0.75   0.3500000        0.75   10    7    7  7.9e-14  4.3e-15
   2        0.74   0.3500000        0.74   11    8    8  7.6e-14  4.4e-15
   3        0.72   0.3500000        0.72   12    9    9  5.5e-14  3.8e-15
Velocity u2, Pressure p
Iter      SolEst     Damping    Stepsize #Res #Jac #Sol   LinErr   LinRes
   1        0.15   0.5000000        0.15    6    3    3  2.5e-15  7.9e-15
Displacement field
Iter      SolEst     Damping    Stepsize #Res #Jac #Sol   LinErr   LinRes
   1        0.52   0.5000000        0.52    6    3    3  1.1e-13  1.5e-12
Spatial mesh displacement
Iter      SolEst     Damping    Stepsize #Res #Jac #Sol   LinErr   LinRes
   1     8.7e-07   0.5000000     8.7e-07    6    3    3  1.4e-15  1.9e-15
Solution error estimates for segregated groups
1.2, 0.076, 0.26, 4.4e-07
Residual error estimates for segregated groups
2.3e+02, 8.1e+03, 1.6e+04, 0.2
Segregated solver iteration 4.
```

```
Merged variables
Iter      SolEst     Damping    Stepsize #Res #Jac #Sol   LinErr   LinRes
   1        0.71   0.3500000        0.71   14   10   10  7.4e-14  3.5e-15
   2        0.69   0.3500000        0.69   15   11   11    6e-14  3.6e-15
   3        0.61   0.3500000        0.61   16   12   12  6.8e-14  4.2e-15
Velocity u2, Pressure p
Iter      SolEst     Damping    Stepsize #Res #Jac #Sol   LinErr   LinRes
   1       0.071   0.5000000       0.071    8    4    4  2.6e-15  7.8e-15
Displacement field
Iter      SolEst     Damping    Stepsize #Res #Jac #Sol   LinErr   LinRes
   1        0.29   0.5000000        0.29    8    4    4  8.2e-14  1.5e-12
Spatial mesh displacement
Iter      SolEst     Damping    Stepsize #Res #Jac #Sol   LinErr   LinRes
   1     7.3e-07   0.5000000     7.3e-07    8    4    4  1.3e-15  1.8e-15
Solution error estimates for segregated groups
1, 0.036, 0.15, 3.6e-07
Residual error estimates for segregated groups
85, 4.3e+03, 1.1e+04, 0.2
Segregated solver iteration 5.
Merged variables
Iter      SolEst     Damping    Stepsize #Res #Jac #Sol   LinErr   LinRes
   1        0.42   0.3500000        0.42   18   13   13  1.1e-13  4.5e-15
   2        0.28   0.3500000        0.28   19   14   14  1.2e-13  3.6e-15
   3        0.18   0.3500000        0.18   20   15   15  5.3e-14  3.7e-15
Velocity u2, Pressure p
Iter      SolEst     Damping    Stepsize #Res #Jac #Sol   LinErr   LinRes
   1       0.035   0.5000000       0.035   10    5    5  3.8e-15  7.9e-15
Displacement field
Iter      SolEst     Damping    Stepsize #Res #Jac #Sol   LinErr   LinRes
   1        0.17   0.5000000        0.17   10    5    5  7.1e-14  1.5e-12
Spatial mesh displacement
Iter      SolEst     Damping    Stepsize #Res #Jac #Sol   LinErr   LinRes
   1     5.4e-07   0.5000000     5.4e-07   10    5    5  1.5e-15  1.8e-15
Solution error estimates for segregated groups
0.32, 0.017, 0.084, 2.7e-07
Residual error estimates for segregated groups
33, 2.2e+03, 6.9e+03, 0.2
Segregated solver iteration 6.
Merged variables
Iter      SolEst     Damping    Stepsize #Res #Jac #Sol   LinErr   LinRes
   1        0.12   0.3500000        0.12   22   16   16  9.7e-14  3.6e-15
   2       0.079   0.3500000       0.079   23   17   17    6e-14  4.3e-15
   3       0.052   0.3500000       0.052   24   18   18  6.9e-14  4.1e-15
Velocity u2, Pressure p
Iter      SolEst     Damping    Stepsize #Res #Jac #Sol   LinErr   LinRes
   1       0.017   0.5000000       0.017   12    6    6  2.7e-15    8e-15
Displacement field
Iter      SolEst     Damping    Stepsize #Res #Jac #Sol   LinErr   LinRes
   1       0.095   0.5000000       0.095   12    6    6  5.8e-14  1.5e-12
Spatial mesh displacement
Iter      SolEst     Damping    Stepsize #Res #Jac #Sol   LinErr   LinRes
   1     3.8e-07   0.5000000     3.8e-07   12    6    6  1.5e-15  1.8e-15
Solution error estimates for segregated groups
0.089, 0.0085, 0.048, 1.9e-07
Residual error estimates for segregated groups
12, 1.1e+03, 4.2e+03, 0.2
```

```
Segregated solver iteration 7.
Merged variables
Iter      SolEst     Damping    Stepsize #Res #Jac #Sol   LinErr   LinRes
   1       0.034   0.3500000       0.034   26   19   19    7e-14  4.7e-15
   2       0.022   0.3500000       0.022   27   20   20  6.8e-14  4.3e-15
   3       0.014   0.3500000       0.014   28   21   21    8e-14  3.7e-15
Velocity u2, Pressure p
Iter      SolEst     Damping    Stepsize #Res #Jac #Sol   LinErr   LinRes
   1      0.0084   0.5000000      0.0084   14    7    7  3.7e-15  7.9e-15
Displacement field
Iter      SolEst     Damping    Stepsize #Res #Jac #Sol   LinErr   LinRes
   1       0.054   0.5000000       0.054   14    7    7  1.6e-13  1.5e-12
Spatial mesh displacement
Iter      SolEst     Damping    Stepsize #Res #Jac #Sol   LinErr   LinRes
   1     2.5e-07   0.5000000     2.5e-07   14    7    7  1.9e-15  1.8e-15
Solution error estimates for segregated groups
0.025, 0.0042, 0.027, 1.3e-07
Residual error estimates for segregated groups
4.2, 5.6e+02, 2.6e+03, 0.18
Segregated solver iteration 8.
Merged variables
Iter      SolEst     Damping    Stepsize #Res #Jac #Sol   LinErr   LinRes
   1      0.0093   0.3500000      0.0093   30   22   22  4.8e-14  4.3e-15
   2      0.0061   0.3500000      0.0061   31   23   23  1.1e-13  3.3e-15
   3      0.0039   0.3500000      0.0039   32   24   24  3.9e-14  4.7e-15
Velocity u2, Pressure p
Iter      SolEst     Damping    Stepsize #Res #Jac #Sol   LinErr   LinRes
   1      0.0042   0.5000000      0.0042   16    8    8  2.9e-15    8e-15
Displacement field
Iter      SolEst     Damping    Stepsize #Res #Jac #Sol   LinErr   LinRes
   1        0.03   0.5000000        0.03   16    8    8  7.2e-14  1.5e-12
Spatial mesh displacement
Iter      SolEst     Damping    Stepsize #Res #Jac #Sol   LinErr   LinRes
   1     1.6e-07   0.5000000     1.6e-07   16    8    8  1.7e-15  1.9e-15
Solution error estimates for segregated groups
0.0068, 0.0021, 0.015, 8.2e-08
Residual error estimates for segregated groups
1.4, 2.8e+02, 1.8e+03, 0.19
Segregated solver iteration 9.
Merged variables
Iter      SolEst     Damping    Stepsize #Res #Jac #Sol   LinErr   LinRes
   1      0.0026   0.3500000      0.0026   34   25   25  5.5e-14  4.4e-15
   2      0.0017   0.3500000      0.0017   35   26   26  4.9e-14  4.1e-15
   3      0.0011   0.3500000      0.0011   36   27   27  6.6e-14  5.2e-15
Velocity u2, Pressure p
Iter      SolEst     Damping    Stepsize #Res #Jac #Sol   LinErr   LinRes
   1      0.0021   0.5000000      0.0021   18    9    9  2.2e-15  7.7e-15
Displacement field
Iter      SolEst     Damping    Stepsize #Res #Jac #Sol   LinErr   LinRes
   1       0.017   0.5000000       0.017   18    9    9    1e-13  1.5e-12
Spatial mesh displacement
Iter      SolEst     Damping    Stepsize #Res #Jac #Sol   LinErr   LinRes
   1       1e-07   0.5000000       1e-07   18    9    9  1.7e-15  1.8e-15
Solution error estimates for segregated groups
0.0019, 0.001, 0.0085, 5.1e-08
Residual error estimates for segregated groups
```

```
0.45, 1.4e+02, 1.4e+03, 0.2
Segregated solver iteration 10.
Merged variables
Iter      SolEst     Damping    Stepsize #Res #Jac #Sol   LinErr   LinRes
   1      0.0007   0.3500000      0.0007   38   28   28  9.4e-14  3.8e-15
   2     0.00046   0.3500000     0.00046   39   29   29  7.1e-14  4.5e-15
   3      0.0003   0.3500000      0.0003   40   30   30  9.6e-14  3.1e-15
Velocity u2, Pressure p
Iter      SolEst     Damping    Stepsize #Res #Jac #Sol   LinErr   LinRes
   1       0.001   0.5000000       0.001   20   10   10  3.9e-15  7.8e-15
Displacement field
Iter      SolEst     Damping    Stepsize #Res #Jac #Sol   LinErr   LinRes
   1      0.0094   0.5000000      0.0094   20   10   10  1.2e-13  1.5e-12
Spatial mesh displacement
Iter      SolEst     Damping    Stepsize #Res #Jac #Sol   LinErr   LinRes
   1     6.2e-08   0.5000000     6.2e-08   20   10   10  1.4e-15  1.8e-15
Solution error estimates for segregated groups
0.00051, 0.00052, 0.0047, 3.1e-08
Residual error estimates for segregated groups
0.14, 70, 1.2e+03, 0.2
Segregated solver iteration 11.
Merged variables
Iter      SolEst     Damping    Stepsize #Res #Jac #Sol   LinErr   LinRes
   1     0.00019   0.3500000     0.00019   42   31   31  7.4e-14  3.2e-15
   2     0.00013   0.3500000     0.00013   43   32   32  6.7e-14  4.3e-15
   3     8.2e-05   0.3500000     8.2e-05   44   33   33  4.7e-14  3.1e-15
Velocity u2, Pressure p
Iter      SolEst     Damping    Stepsize #Res #Jac #Sol   LinErr   LinRes
   1     0.00052   0.5000000     0.00052   22   11   11  5.1e-15    8e-15
Displacement field
Iter      SolEst     Damping    Stepsize #Res #Jac #Sol   LinErr   LinRes
   1      0.0051   0.5000000      0.0051   22   11   11  9.4e-14  1.5e-12
Spatial mesh displacement
Iter      SolEst     Damping    Stepsize #Res #Jac #Sol   LinErr   LinRes
   1     3.7e-08   0.5000000     3.7e-08   22   11   11  2.1e-15  1.8e-15
Solution error estimates for segregated groups
0.00014, 0.00026, 0.0026, 1.9e-08
Residual error estimates for segregated groups
0.044, 35, 1.2e+03, 0.19
Segregated solver iteration 12.
Merged variables
Iter      SolEst     Damping    Stepsize #Res #Jac #Sol   LinErr   LinRes
   1     5.3e-05   0.3500000     5.3e-05   46   34   34  5.9e-14  4.2e-15
   2     3.5e-05   0.3500000     3.5e-05   47   35   35    1e-13  4.4e-15
   3     2.2e-05   0.3500000     2.2e-05   48   36   36  1.2e-13  3.3e-15
Velocity u2, Pressure p
Iter      SolEst     Damping    Stepsize #Res #Jac #Sol   LinErr   LinRes
   1     0.00026   0.5000000     0.00026   24   12   12  2.9e-15  7.7e-15
Displacement field
Iter      SolEst     Damping    Stepsize #Res #Jac #Sol   LinErr   LinRes
   1      0.0028   0.5000000      0.0028   24   12   12  3.4e-14  1.5e-12
Spatial mesh displacement
Iter      SolEst     Damping    Stepsize #Res #Jac #Sol   LinErr   LinRes
   1     2.2e-08   0.5000000     2.2e-08   24   12   12  1.4e-15  1.9e-15
Solution error estimates for segregated groups
3.9e-05, 0.00013, 0.0014, 1.1e-08
```

```
Residual error estimates for segregated groups
0.013, 18, 1.2e+03, 0.19
Segregated solver iteration 13.
Merged variables
Iter      SolEst     Damping    Stepsize #Res #Jac #Sol   LinErr   LinRes
   1     1.5e-05   0.3500000     1.5e-05   50   37   37  4.7e-14  3.6e-15
   2     9.5e-06   0.3500000     9.5e-06   51   38   38  1.1e-13  3.8e-15
   3     6.2e-06   0.3500000     6.2e-06   52   39   39  2.1e-13  3.3e-15
Velocity u2, Pressure p
Iter      SolEst     Damping    Stepsize #Res #Jac #Sol   LinErr   LinRes
   1     0.00013   0.5000000     0.00013   26   13   13  2.5e-15  7.9e-15
Displacement field
Iter      SolEst     Damping    Stepsize #Res #Jac #Sol   LinErr   LinRes
   1      0.0015   0.5000000      0.0015   26   13   13  1.5e-13  1.5e-12
Spatial mesh displacement
Iter      SolEst     Damping    Stepsize #Res #Jac #Sol   LinErr   LinRes
   1     1.3e-08   0.5000000     1.3e-08   26   13   13  1.3e-15  1.8e-15
Solution error estimates for segregated groups
1.1e-05, 6.5e-05, 0.00076, 6.4e-09
Residual error estimates for segregated groups
0.0041, 8.8, 1.2e+03, 0.2
Solution time: 161 s. (2 minutes, 41 seconds)
Physical memory: 4.56 GB
Virtual memory: 25.5 GB
Ended at Jan 31, 2025, 5:38:11 PM.
----- Stationary Solver 1 in study1/Approach Curve Solver (sol1) -------------->
```

d=2415 (su19)

GENERAL

Description	Value
Solution	d=2415

LOG

```
<---- Compile Equations: Stationary in study1/Approach Curve Solver (sol1) -----
Started at Jan 31, 2025, 5:38:12 PM.
Parameter d = 2.415E-6 (m).
Geometry shape function: Linear serendipity
Number of vertex elements: 18
Number of boundary elements: 6288
Number of elements: 157226
Minimum element quality: 0.4422
Time: 9 s.
Physical memory: 2.84 GB
Virtual memory: 15.21 GB
Ended at Jan 31, 2025, 5:38:20 PM.
----- Compile Equations: Stationary in study1/Approach Curve Solver (sol1) ---->

<---- Dependent Variables 1 in study1/Approach Curve Solver (sol1) -------------
Started at Jan 31, 2025, 5:38:20 PM.
Solution time: 0 s.
Physical memory: 2.84 GB
Virtual memory: 15.21 GB
Ended at Jan 31, 2025, 5:38:20 PM.
----- Dependent Variables 1 in study1/Approach Curve Solver (sol1) ------------>

<---- Stationary Solver 1 in study1/Approach Curve Solver (sol1) ---------------
Started at Jan 31, 2025, 5:38:21 PM.
Segregated solver
Segregated Step 11 does not have any components solved for.
Number of degrees of freedom solved for: 799936 (plus 11999 internal DOFs).
Segregated solver iteration 1.
Merged variables
Nonsymmetric matrix found.
Scales for dependent variables:
Concentration (comp1.Cl): 1.4e+02
Concentration (comp1.K): 1.4e+02
Electric potential (comp1.V): 1.9e+02
Orthonormal null-space function used.
Iter      SolEst     Damping    Stepsize #Res #Jac #Sol   LinErr   LinRes
   1        0.83   0.3500000        0.83    2    1    1  1.5e-11  2.8e-15
   2        0.81   0.3500000        0.81    3    2    2  4.1e-11  2.7e-15
   3         0.8   0.3500000         0.8    4    3    3  6.7e-11  2.8e-15
Velocity u2, Pressure p
Nonsymmetric matrix found.
Scales for dependent variables:
Pressure (comp1.p): 1
Velocity field (spatial frame) (comp1.u2): 0.00019
Orthonormal null-space function used.
Iter      SolEst     Damping    Stepsize #Res #Jac #Sol   LinErr   LinRes
   1         1.1   0.5000000         1.1    2    1    1  1.7e-15  2.3e-15
Displacement field
Nonsymmetric matrix found.
Scales for dependent variables:
Auxiliary pressure (comp1.solid.lemm1.pw): 1
Displacement field (comp1.u): 2.8e-18
Orthonormal null-space function used.
Iter      SolEst     Damping    Stepsize #Res #Jac #Sol   LinErr   LinRes
   1         1.9   0.5000000         1.9    2    1    1  7.2e-14  1.5e-12
Spatial mesh displacement
```

```
Symmetric matrices found.
Scales for dependent variables:
Spatial mesh displacement (comp1.spatial.disp): 1.2e-09
Orthonormal null-space function used.
Iter      SolEst     Damping    Stepsize #Res #Jac #Sol   LinErr   LinRes
   1     5.8e-07   0.5000000     5.8e-07    2    1    1  2.1e-15  1.9e-15
Solution error estimates for segregated groups
1.4, 0.54, 0.95, 2.9e-07
Residual error estimates for segregated groups
7.9e+03, 8.3e+04, 2.2e+04, 0.16
Segregated solver iteration 2.
Merged variables
Scales for dependent variables:
Concentration (comp1.Cl): 1.4e+02
Concentration (comp1.K): 1.4e+02
Electric potential (comp1.V): 0.064
Iter      SolEst     Damping    Stepsize #Res #Jac #Sol   LinErr   LinRes
   1        0.79   0.3500000        0.79    6    4    4  5.8e-14    3e-15
   2        0.77   0.3500000        0.77    7    5    5  8.7e-14    3e-15
   3        0.76   0.3500000        0.76    8    6    6  5.2e-14  3.2e-15
Velocity u2, Pressure p
Scales for dependent variables:
Pressure (comp1.p): 3.5e+02
Velocity field (spatial frame) (comp1.u2): 0.00071
Iter      SolEst     Damping    Stepsize #Res #Jac #Sol   LinErr   LinRes
   1        0.36   0.5000000        0.36    4    2    2  2.5e-15  7.6e-15
Displacement field
Iter      SolEst     Damping    Stepsize #Res #Jac #Sol   LinErr   LinRes
   1        0.95   0.5000000        0.95    4    2    2  8.1e-14  1.5e-12
Spatial mesh displacement
Iter      SolEst     Damping    Stepsize #Res #Jac #Sol   LinErr   LinRes
   1     8.7e-07   0.5000000     8.7e-07    4    2    2  1.6e-15  1.9e-15
Solution error estimates for segregated groups
1.3, 0.18, 0.48, 4.4e-07
Residual error estimates for segregated groups
8.1e+02, 1.6e+04, 2.2e+04, 0.19
Segregated solver iteration 3.
Merged variables
Iter      SolEst     Damping    Stepsize #Res #Jac #Sol   LinErr   LinRes
   1        0.75   0.3500000        0.75   10    7    7  7.8e-14  3.1e-15
   2        0.74   0.3500000        0.74   11    8    8  5.6e-14    3e-15
   3        0.72   0.3500000        0.72   12    9    9  1.1e-13  2.8e-15
Velocity u2, Pressure p
Iter      SolEst     Damping    Stepsize #Res #Jac #Sol   LinErr   LinRes
   1        0.15   0.5000000        0.15    6    3    3  3.5e-15  7.9e-15
Displacement field
Iter      SolEst     Damping    Stepsize #Res #Jac #Sol   LinErr   LinRes
   1        0.52   0.5000000        0.52    6    3    3  4.4e-14  1.5e-12
Spatial mesh displacement
Iter      SolEst     Damping    Stepsize #Res #Jac #Sol   LinErr   LinRes
   1     8.7e-07   0.5000000     8.7e-07    6    3    3  2.1e-15  1.8e-15
Solution error estimates for segregated groups
1.2, 0.076, 0.26, 4.4e-07
Residual error estimates for segregated groups
2.3e+02, 8.8e+03, 1.6e+04, 0.19
Segregated solver iteration 4.
```

```
Merged variables
Iter      SolEst     Damping    Stepsize #Res #Jac #Sol   LinErr   LinRes
   1        0.71   0.3500000        0.71   14   10   10  7.7e-14  2.8e-15
   2        0.69   0.3500000        0.69   15   11   11    1e-13  3.1e-15
   3        0.61   0.3500000        0.61   16   12   12    1e-13  2.8e-15
Velocity u2, Pressure p
Iter      SolEst     Damping    Stepsize #Res #Jac #Sol   LinErr   LinRes
   1       0.071   0.5000000       0.071    8    4    4  2.1e-15    8e-15
Displacement field
Iter      SolEst     Damping    Stepsize #Res #Jac #Sol   LinErr   LinRes
   1        0.29   0.5000000        0.29    8    4    4  4.6e-14  1.5e-12
Spatial mesh displacement
Iter      SolEst     Damping    Stepsize #Res #Jac #Sol   LinErr   LinRes
   1     7.3e-07   0.5000000     7.3e-07    8    4    4  1.5e-15  1.9e-15
Solution error estimates for segregated groups
1, 0.036, 0.15, 3.6e-07
Residual error estimates for segregated groups
85, 4.7e+03, 1.1e+04, 0.19
Segregated solver iteration 5.
Merged variables
Iter      SolEst     Damping    Stepsize #Res #Jac #Sol   LinErr   LinRes
   1        0.42   0.3500000        0.42   18   13   13  4.5e-14  2.9e-15
   2        0.28   0.3500000        0.28   19   14   14    1e-13  2.9e-15
   3        0.19   0.3500000        0.19   20   15   15  9.5e-14  2.8e-15
Velocity u2, Pressure p
Iter      SolEst     Damping    Stepsize #Res #Jac #Sol   LinErr   LinRes
   1       0.035   0.5000000       0.035   10    5    5  3.1e-15  7.7e-15
Displacement field
Iter      SolEst     Damping    Stepsize #Res #Jac #Sol   LinErr   LinRes
   1        0.17   0.5000000        0.17   10    5    5  1.1e-13  1.5e-12
Spatial mesh displacement
Iter      SolEst     Damping    Stepsize #Res #Jac #Sol   LinErr   LinRes
   1     5.5e-07   0.5000000     5.5e-07   10    5    5  1.8e-15  1.8e-15
Solution error estimates for segregated groups
0.32, 0.017, 0.084, 2.7e-07
Residual error estimates for segregated groups
33, 2.4e+03, 6.9e+03, 0.19
Segregated solver iteration 6.
Merged variables
Iter      SolEst     Damping    Stepsize #Res #Jac #Sol   LinErr   LinRes
   1        0.12   0.3500000        0.12   22   16   16  9.8e-14  3.1e-15
   2        0.08   0.3500000        0.08   23   17   17  1.1e-13  2.9e-15
   3       0.052   0.3500000       0.052   24   18   18  7.5e-14  2.9e-15
Velocity u2, Pressure p
Iter      SolEst     Damping    Stepsize #Res #Jac #Sol   LinErr   LinRes
   1       0.017   0.5000000       0.017   12    6    6  2.3e-15  7.8e-15
Displacement field
Iter      SolEst     Damping    Stepsize #Res #Jac #Sol   LinErr   LinRes
   1       0.095   0.5000000       0.095   12    6    6  1.2e-13  1.5e-12
Spatial mesh displacement
Iter      SolEst     Damping    Stepsize #Res #Jac #Sol   LinErr   LinRes
   1     3.8e-07   0.5000000     3.8e-07   12    6    6    2e-15  1.8e-15
Solution error estimates for segregated groups
0.089, 0.0085, 0.048, 1.9e-07
Residual error estimates for segregated groups
12, 1.2e+03, 4.2e+03, 0.18
```

```
Segregated solver iteration 7.
Merged variables
Iter      SolEst     Damping    Stepsize #Res #Jac #Sol   LinErr   LinRes
   1       0.034   0.3500000       0.034   26   19   19    1e-13  3.2e-15
   2       0.022   0.3500000       0.022   27   20   20  5.3e-14    3e-15
   3       0.014   0.3500000       0.014   28   21   21  7.3e-14    3e-15
Velocity u2, Pressure p
Iter      SolEst     Damping    Stepsize #Res #Jac #Sol   LinErr   LinRes
   1      0.0084   0.5000000      0.0084   14    7    7  2.7e-15  7.7e-15
Displacement field
Iter      SolEst     Damping    Stepsize #Res #Jac #Sol   LinErr   LinRes
   1       0.054   0.5000000       0.054   14    7    7  2.4e-14  1.5e-12
Spatial mesh displacement
Iter      SolEst     Damping    Stepsize #Res #Jac #Sol   LinErr   LinRes
   1     2.6e-07   0.5000000     2.6e-07   14    7    7  1.3e-15  1.9e-15
Solution error estimates for segregated groups
0.025, 0.0042, 0.027, 1.3e-07
Residual error estimates for segregated groups
4.2, 6.1e+02, 2.7e+03, 0.2
Segregated solver iteration 8.
Merged variables
Iter      SolEst     Damping    Stepsize #Res #Jac #Sol   LinErr   LinRes
   1      0.0094   0.3500000      0.0094   30   22   22  6.3e-14  2.9e-15
   2      0.0061   0.3500000      0.0061   31   23   23  5.9e-14  2.8e-15
   3       0.004   0.3500000       0.004   32   24   24  6.8e-14  3.1e-15
Velocity u2, Pressure p
Iter      SolEst     Damping    Stepsize #Res #Jac #Sol   LinErr   LinRes
   1      0.0042   0.5000000      0.0042   16    8    8  3.6e-15  7.8e-15
Displacement field
Iter      SolEst     Damping    Stepsize #Res #Jac #Sol   LinErr   LinRes
   1        0.03   0.5000000        0.03   16    8    8  6.4e-14  1.5e-12
Spatial mesh displacement
Iter      SolEst     Damping    Stepsize #Res #Jac #Sol   LinErr   LinRes
   1     1.6e-07   0.5000000     1.6e-07   16    8    8  1.8e-15  1.9e-15
Solution error estimates for segregated groups
0.0068, 0.0021, 0.015, 8.2e-08
Residual error estimates for segregated groups
1.4, 3.1e+02, 1.8e+03, 0.2
Segregated solver iteration 9.
Merged variables
Iter      SolEst     Damping    Stepsize #Res #Jac #Sol   LinErr   LinRes
   1      0.0026   0.3500000      0.0026   34   25   25  1.3e-13  3.1e-15
   2      0.0017   0.3500000      0.0017   35   26   26  8.4e-14  2.9e-15
   3      0.0011   0.3500000      0.0011   36   27   27  8.8e-14    3e-15
Velocity u2, Pressure p
Iter      SolEst     Damping    Stepsize #Res #Jac #Sol   LinErr   LinRes
   1      0.0021   0.5000000      0.0021   18    9    9  2.8e-15  7.7e-15
Displacement field
Iter      SolEst     Damping    Stepsize #Res #Jac #Sol   LinErr   LinRes
   1       0.017   0.5000000       0.017   18    9    9  6.7e-14  1.5e-12
Spatial mesh displacement
Iter      SolEst     Damping    Stepsize #Res #Jac #Sol   LinErr   LinRes
   1       1e-07   0.5000000       1e-07   18    9    9  1.2e-15  1.8e-15
Solution error estimates for segregated groups
0.0019, 0.001, 0.0085, 5.1e-08
Residual error estimates for segregated groups
```

```
0.45, 1.5e+02, 1.4e+03, 0.19
Segregated solver iteration 10.
Merged variables
Iter      SolEst     Damping    Stepsize #Res #Jac #Sol   LinErr   LinRes
   1     0.00071   0.3500000     0.00071   38   28   28  1.2e-13  2.8e-15
   2     0.00046   0.3500000     0.00046   39   29   29  5.5e-14  2.9e-15
   3      0.0003   0.3500000      0.0003   40   30   30  7.1e-14  2.9e-15
Velocity u2, Pressure p
Iter      SolEst     Damping    Stepsize #Res #Jac #Sol   LinErr   LinRes
   1       0.001   0.5000000       0.001   20   10   10  2.1e-15  7.7e-15
Displacement field
Iter      SolEst     Damping    Stepsize #Res #Jac #Sol   LinErr   LinRes
   1      0.0094   0.5000000      0.0094   20   10   10  1.1e-13  1.5e-12
Spatial mesh displacement
Iter      SolEst     Damping    Stepsize #Res #Jac #Sol   LinErr   LinRes
   1     6.3e-08   0.5000000     6.3e-08   20   10   10  1.3e-15  1.8e-15
Solution error estimates for segregated groups
0.00051, 0.00052, 0.0047, 3.1e-08
Residual error estimates for segregated groups
0.14, 77, 1.2e+03, 0.2
Segregated solver iteration 11.
Merged variables
Iter      SolEst     Damping    Stepsize #Res #Jac #Sol   LinErr   LinRes
   1     0.00019   0.3500000     0.00019   42   31   31    9e-14    3e-15
   2     0.00013   0.3500000     0.00013   43   32   32  1.1e-13  3.2e-15
   3     8.2e-05   0.3500000     8.2e-05   44   33   33  1.4e-13  2.9e-15
Velocity u2, Pressure p
Iter      SolEst     Damping    Stepsize #Res #Jac #Sol   LinErr   LinRes
   1     0.00052   0.5000000     0.00052   22   11   11  2.6e-15  7.7e-15
Displacement field
Iter      SolEst     Damping    Stepsize #Res #Jac #Sol   LinErr   LinRes
   1      0.0051   0.5000000      0.0051   22   11   11  7.4e-14  1.5e-12
Spatial mesh displacement
Iter      SolEst     Damping    Stepsize #Res #Jac #Sol   LinErr   LinRes
   1     3.8e-08   0.5000000     3.8e-08   22   11   11  1.4e-15  1.9e-15
Solution error estimates for segregated groups
0.00014, 0.00026, 0.0026, 1.9e-08
Residual error estimates for segregated groups
0.044, 38, 1.2e+03, 0.2
Segregated solver iteration 12.
Merged variables
Iter      SolEst     Damping    Stepsize #Res #Jac #Sol   LinErr   LinRes
   1     5.3e-05   0.3500000     5.3e-05   46   34   34  7.4e-14  3.1e-15
   2     3.5e-05   0.3500000     3.5e-05   47   35   35  1.2e-13  2.9e-15
   3     2.3e-05   0.3500000     2.3e-05   48   36   36  5.8e-14  2.9e-15
Velocity u2, Pressure p
Iter      SolEst     Damping    Stepsize #Res #Jac #Sol   LinErr   LinRes
   1     0.00026   0.5000000     0.00026   24   12   12  2.4e-15  7.8e-15
Displacement field
Iter      SolEst     Damping    Stepsize #Res #Jac #Sol   LinErr   LinRes
   1      0.0028   0.5000000      0.0028   24   12   12  5.3e-14  1.5e-12
Spatial mesh displacement
Iter      SolEst     Damping    Stepsize #Res #Jac #Sol   LinErr   LinRes
   1     2.2e-08   0.5000000     2.2e-08   24   12   12  1.5e-15  1.9e-15
Solution error estimates for segregated groups
3.9e-05, 0.00013, 0.0014, 1.1e-08
```

```
Residual error estimates for segregated groups
0.013, 19, 1.2e+03, 0.2
Segregated solver iteration 13.
Merged variables
Iter      SolEst     Damping    Stepsize #Res #Jac #Sol   LinErr   LinRes
   1     1.5e-05   0.3500000     1.5e-05   50   37   37  1.1e-13    3e-15
   2     9.5e-06   0.3500000     9.5e-06   51   38   38  1.3e-13  2.8e-15
   3     6.2e-06   0.3500000     6.2e-06   52   39   39  6.6e-14    3e-15
Velocity u2, Pressure p
Iter      SolEst     Damping    Stepsize #Res #Jac #Sol   LinErr   LinRes
   1     0.00013   0.5000000     0.00013   26   13   13  1.7e-15  7.8e-15
Displacement field
Iter      SolEst     Damping    Stepsize #Res #Jac #Sol   LinErr   LinRes
   1      0.0015   0.5000000      0.0015   26   13   13  3.4e-14  1.5e-12
Spatial mesh displacement
Iter      SolEst     Damping    Stepsize #Res #Jac #Sol   LinErr   LinRes
   1     1.3e-08   0.5000000     1.3e-08   26   13   13  1.7e-15  1.8e-15
Solution error estimates for segregated groups
1.1e-05, 6.5e-05, 0.00076, 6.5e-09
Residual error estimates for segregated groups
0.0041, 9.6, 1.2e+03, 0.2
Solution time: 156 s. (2 minutes, 36 seconds)
Physical memory: 4.57 GB
Virtual memory: 26.02 GB
Ended at Jan 31, 2025, 5:40:56 PM.
----- Stationary Solver 1 in study1/Approach Curve Solver (sol1) -------------->
```

d=2410 (su20)

GENERAL

Description	Value
Solution	d=2410

LOG

```
<---- Compile Equations: Stationary in study1/Approach Curve Solver (sol1) -----
Started at Jan 31, 2025, 5:40:56 PM.
Parameter d = 2.4100000000000002E-6 (m).
Geometry shape function: Linear serendipity
Number of vertex elements: 18
Number of boundary elements: 6286
Number of elements: 156768
Minimum element quality: 0.4824
Time: 9 s.
Physical memory: 2.77 GB
Virtual memory: 15.13 GB
Ended at Jan 31, 2025, 5:41:05 PM.
----- Compile Equations: Stationary in study1/Approach Curve Solver (sol1) ---->

<---- Dependent Variables 1 in study1/Approach Curve Solver (sol1) -------------
Started at Jan 31, 2025, 5:41:05 PM.
Solution time: 0 s.
Physical memory: 2.77 GB
Virtual memory: 15.13 GB
Ended at Jan 31, 2025, 5:41:05 PM.
----- Dependent Variables 1 in study1/Approach Curve Solver (sol1) ------------>

<---- Stationary Solver 1 in study1/Approach Curve Solver (sol1) ---------------
Started at Jan 31, 2025, 5:41:05 PM.
Segregated solver
Segregated Step 11 does not have any components solved for.
Number of degrees of freedom solved for: 797484 (plus 11995 internal DOFs).
Segregated solver iteration 1.
Merged variables
Nonsymmetric matrix found.
Scales for dependent variables:
Concentration (comp1.Cl): 1.4e+02
Concentration (comp1.K): 1.4e+02
Electric potential (comp1.V): 1.9e+02
Orthonormal null-space function used.
Iter      SolEst     Damping    Stepsize #Res #Jac #Sol   LinErr   LinRes
   1        0.83   0.3500000        0.83    2    1    1  2.4e-11  2.8e-15
   2        0.81   0.3500000        0.81    3    2    2  2.7e-11  2.7e-15
   3         0.8   0.3500000         0.8    4    3    3    3e-11  2.7e-15
Velocity u2, Pressure p
Nonsymmetric matrix found.
Scales for dependent variables:
Pressure (comp1.p): 1
Velocity field (spatial frame) (comp1.u2): 0.00019
Orthonormal null-space function used.
Iter      SolEst     Damping    Stepsize #Res #Jac #Sol   LinErr   LinRes
   1         1.1   0.5000000         1.1    2    1    1  1.6e-15  2.3e-15
Displacement field
Nonsymmetric matrix found.
Scales for dependent variables:
Auxiliary pressure (comp1.solid.lemm1.pw): 1
Displacement field (comp1.u): 2.8e-18
Orthonormal null-space function used.
Iter      SolEst     Damping    Stepsize #Res #Jac #Sol   LinErr   LinRes
   1         1.9   0.5000000         1.9    2    1    1  1.8e-13  1.5e-12
Spatial mesh displacement
```

```
Symmetric matrices found.
Scales for dependent variables:
Spatial mesh displacement (comp1.spatial.disp): 1.2e-09
Orthonormal null-space function used.
Iter      SolEst     Damping    Stepsize #Res #Jac #Sol   LinErr   LinRes
   1     5.9e-07   0.5000000     5.9e-07    2    1    1  1.6e-15  1.9e-15
Solution error estimates for segregated groups
1.4, 0.54, 0.95, 2.9e-07
Residual error estimates for segregated groups
7.9e+03, 8.3e+04, 2.2e+04, 0.17
Segregated solver iteration 2.
Merged variables
Scales for dependent variables:
Concentration (comp1.Cl): 1.4e+02
Concentration (comp1.K): 1.4e+02
Electric potential (comp1.V): 0.064
Iter      SolEst     Damping    Stepsize #Res #Jac #Sol   LinErr   LinRes
   1        0.79   0.3500000        0.79    6    4    4  6.7e-14  3.3e-15
   2        0.77   0.3500000        0.77    7    5    5  8.6e-14  2.8e-15
   3        0.76   0.3500000        0.76    8    6    6  7.8e-14  3.9e-15
Velocity u2, Pressure p
Scales for dependent variables:
Pressure (comp1.p): 3.5e+02
Velocity field (spatial frame) (comp1.u2): 0.00071
Iter      SolEst     Damping    Stepsize #Res #Jac #Sol   LinErr   LinRes
   1        0.36   0.5000000        0.36    4    2    2  3.3e-15  7.9e-15
Displacement field
Iter      SolEst     Damping    Stepsize #Res #Jac #Sol   LinErr   LinRes
   1        0.95   0.5000000        0.95    4    2    2  1.1e-13  1.5e-12
Spatial mesh displacement
Iter      SolEst     Damping    Stepsize #Res #Jac #Sol   LinErr   LinRes
   1     8.8e-07   0.5000000     8.8e-07    4    2    2  1.8e-15  1.9e-15
Solution error estimates for segregated groups
1.3, 0.18, 0.48, 4.4e-07
Residual error estimates for segregated groups
8.1e+02, 1.3e+04, 2.2e+04, 0.18
Segregated solver iteration 3.
Merged variables
Iter      SolEst     Damping    Stepsize #Res #Jac #Sol   LinErr   LinRes
   1        0.75   0.3500000        0.75   10    7    7  9.9e-14  2.9e-15
   2        0.74   0.3500000        0.74   11    8    8  1.1e-13    3e-15
   3        0.72   0.3500000        0.72   12    9    9  7.4e-14  3.4e-15
Velocity u2, Pressure p
Iter      SolEst     Damping    Stepsize #Res #Jac #Sol   LinErr   LinRes
   1        0.15   0.5000000        0.15    6    3    3  2.5e-15  7.8e-15
Displacement field
Iter      SolEst     Damping    Stepsize #Res #Jac #Sol   LinErr   LinRes
   1        0.52   0.5000000        0.52    6    3    3    3e-14  1.5e-12
Spatial mesh displacement
Iter      SolEst     Damping    Stepsize #Res #Jac #Sol   LinErr   LinRes
   1     8.8e-07   0.5000000     8.8e-07    6    3    3  1.3e-15  1.9e-15
Solution error estimates for segregated groups
1.2, 0.077, 0.26, 4.4e-07
Residual error estimates for segregated groups
2.2e+02, 7.4e+03, 1.6e+04, 0.18
Segregated solver iteration 4.
```

```
Merged variables
Iter      SolEst     Damping    Stepsize #Res #Jac #Sol   LinErr   LinRes
   1         0.7   0.3500000         0.7   14   10   10  1.4e-13  4.3e-15
   2        0.68   0.3500000        0.68   15   11   11  5.6e-14  3.2e-15
   3         0.6   0.3500000         0.6   16   12   12  6.3e-14    3e-15
Velocity u2, Pressure p
Iter      SolEst     Damping    Stepsize #Res #Jac #Sol   LinErr   LinRes
   1       0.071   0.5000000       0.071    8    4    4  2.4e-15  7.8e-15
Displacement field
Iter      SolEst     Damping    Stepsize #Res #Jac #Sol   LinErr   LinRes
   1        0.29   0.5000000        0.29    8    4    4    6e-14  1.5e-12
Spatial mesh displacement
Iter      SolEst     Damping    Stepsize #Res #Jac #Sol   LinErr   LinRes
   1     7.4e-07   0.5000000     7.4e-07    8    4    4  1.7e-15  1.8e-15
Solution error estimates for segregated groups
1, 0.036, 0.15, 3.7e-07
Residual error estimates for segregated groups
85, 3.9e+03, 1.1e+04, 0.19
Segregated solver iteration 5.
Merged variables
Iter      SolEst     Damping    Stepsize #Res #Jac #Sol   LinErr   LinRes
   1        0.42   0.3500000        0.42   18   13   13  5.2e-14  2.8e-15
   2        0.28   0.3500000        0.28   19   14   14  8.8e-14  3.6e-15
   3        0.18   0.3500000        0.18   20   15   15  2.7e-14  3.4e-15
Velocity u2, Pressure p
Iter      SolEst     Damping    Stepsize #Res #Jac #Sol   LinErr   LinRes
   1       0.035   0.5000000       0.035   10    5    5  2.8e-15  7.8e-15
Displacement field
Iter      SolEst     Damping    Stepsize #Res #Jac #Sol   LinErr   LinRes
   1        0.17   0.5000000        0.17   10    5    5  1.4e-13  1.5e-12
Spatial mesh displacement
Iter      SolEst     Damping    Stepsize #Res #Jac #Sol   LinErr   LinRes
   1     5.5e-07   0.5000000     5.5e-07   10    5    5  1.5e-15  1.9e-15
Solution error estimates for segregated groups
0.32, 0.017, 0.084, 2.8e-07
Residual error estimates for segregated groups
33, 2e+03, 6.9e+03, 0.18
Segregated solver iteration 6.
Merged variables
Iter      SolEst     Damping    Stepsize #Res #Jac #Sol   LinErr   LinRes
   1        0.12   0.3500000        0.12   22   16   16    1e-13  3.2e-15
   2       0.079   0.3500000       0.079   23   17   17  1.5e-13  2.9e-15
   3       0.052   0.3500000       0.052   24   18   18  3.5e-14  3.3e-15
Velocity u2, Pressure p
Iter      SolEst     Damping    Stepsize #Res #Jac #Sol   LinErr   LinRes
   1       0.017   0.5000000       0.017   12    6    6  3.7e-15  7.9e-15
Displacement field
Iter      SolEst     Damping    Stepsize #Res #Jac #Sol   LinErr   LinRes
   1       0.095   0.5000000       0.095   12    6    6  5.5e-14  1.5e-12
Spatial mesh displacement
Iter      SolEst     Damping    Stepsize #Res #Jac #Sol   LinErr   LinRes
   1     3.9e-07   0.5000000     3.9e-07   12    6    6  1.6e-15  1.9e-15
Solution error estimates for segregated groups
0.089, 0.0085, 0.048, 1.9e-07
Residual error estimates for segregated groups
12, 1e+03, 4.2e+03, 0.18
```

```
Segregated solver iteration 7.
Merged variables
Iter      SolEst     Damping    Stepsize #Res #Jac #Sol   LinErr   LinRes
   1       0.034   0.3500000       0.034   26   19   19  1.1e-13  3.3e-15
   2       0.022   0.3500000       0.022   27   20   20  6.1e-14    3e-15
   3       0.014   0.3500000       0.014   28   21   21  7.2e-14    3e-15
Velocity u2, Pressure p
Iter      SolEst     Damping    Stepsize #Res #Jac #Sol   LinErr   LinRes
   1      0.0084   0.5000000      0.0084   14    7    7  3.9e-15  7.8e-15
Displacement field
Iter      SolEst     Damping    Stepsize #Res #Jac #Sol   LinErr   LinRes
   1       0.054   0.5000000       0.054   14    7    7  5.1e-14  1.5e-12
Spatial mesh displacement
Iter      SolEst     Damping    Stepsize #Res #Jac #Sol   LinErr   LinRes
   1     2.6e-07   0.5000000     2.6e-07   14    7    7  1.6e-15  1.9e-15
Solution error estimates for segregated groups
0.025, 0.0042, 0.027, 1.3e-07
Residual error estimates for segregated groups
4.1, 5.1e+02, 2.7e+03, 0.18
Segregated solver iteration 8.
Merged variables
Iter      SolEst     Damping    Stepsize #Res #Jac #Sol   LinErr   LinRes
   1      0.0093   0.3500000      0.0093   30   22   22    1e-13  2.9e-15
   2       0.006   0.3500000       0.006   31   23   23  7.4e-14  3.1e-15
   3      0.0039   0.3500000      0.0039   32   24   24  4.7e-14  2.8e-15
Velocity u2, Pressure p
Iter      SolEst     Damping    Stepsize #Res #Jac #Sol   LinErr   LinRes
   1      0.0042   0.5000000      0.0042   16    8    8  4.2e-15    8e-15
Displacement field
Iter      SolEst     Damping    Stepsize #Res #Jac #Sol   LinErr   LinRes
   1        0.03   0.5000000        0.03   16    8    8  6.4e-14  1.5e-12
Spatial mesh displacement
Iter      SolEst     Damping    Stepsize #Res #Jac #Sol   LinErr   LinRes
   1     1.7e-07   0.5000000     1.7e-07   16    8    8  1.5e-15  1.9e-15
Solution error estimates for segregated groups
0.0068, 0.0021, 0.015, 8.3e-08
Residual error estimates for segregated groups
1.4, 2.6e+02, 1.8e+03, 0.19
Segregated solver iteration 9.
Merged variables
Iter      SolEst     Damping    Stepsize #Res #Jac #Sol   LinErr   LinRes
   1      0.0026   0.3500000      0.0026   34   25   25    1e-13  3.1e-15
   2      0.0017   0.3500000      0.0017   35   26   26  8.6e-14    3e-15
   3      0.0011   0.3500000      0.0011   36   27   27  4.9e-14  2.9e-15
Velocity u2, Pressure p
Iter      SolEst     Damping    Stepsize #Res #Jac #Sol   LinErr   LinRes
   1      0.0021   0.5000000      0.0021   18    9    9  2.4e-15  7.8e-15
Displacement field
Iter      SolEst     Damping    Stepsize #Res #Jac #Sol   LinErr   LinRes
   1       0.017   0.5000000       0.017   18    9    9  1.2e-13  1.5e-12
Spatial mesh displacement
Iter      SolEst     Damping    Stepsize #Res #Jac #Sol   LinErr   LinRes
   1       1e-07   0.5000000       1e-07   18    9    9  1.6e-15  1.9e-15
Solution error estimates for segregated groups
0.0019, 0.001, 0.0085, 5.2e-08
Residual error estimates for segregated groups
```

```
0.44, 1.3e+02, 1.4e+03, 0.2
Segregated solver iteration 10.
Merged variables
Iter      SolEst     Damping    Stepsize #Res #Jac #Sol   LinErr   LinRes
   1      0.0007   0.3500000      0.0007   38   28   28  7.9e-14    3e-15
   2     0.00046   0.3500000     0.00046   39   29   29  9.3e-14  2.9e-15
   3      0.0003   0.3500000      0.0003   40   30   30  6.6e-14  2.9e-15
Velocity u2, Pressure p
Iter      SolEst     Damping    Stepsize #Res #Jac #Sol   LinErr   LinRes
   1       0.001   0.5000000       0.001   20   10   10  4.8e-15  7.8e-15
Displacement field
Iter      SolEst     Damping    Stepsize #Res #Jac #Sol   LinErr   LinRes
   1      0.0094   0.5000000      0.0094   20   10   10  1.9e-13  1.5e-12
Spatial mesh displacement
Iter      SolEst     Damping    Stepsize #Res #Jac #Sol   LinErr   LinRes
   1     6.3e-08   0.5000000     6.3e-08   20   10   10  1.6e-15  1.8e-15
Solution error estimates for segregated groups
0.00051, 0.00052, 0.0047, 3.2e-08
Residual error estimates for segregated groups
0.14, 64, 1.3e+03, 0.19
Segregated solver iteration 11.
Merged variables
Iter      SolEst     Damping    Stepsize #Res #Jac #Sol   LinErr   LinRes
   1     0.00019   0.3500000     0.00019   42   31   31  3.1e-14  3.1e-15
   2     0.00013   0.3500000     0.00013   43   32   32  5.9e-14    3e-15
   3     8.2e-05   0.3500000     8.2e-05   44   33   33    1e-13  3.2e-15
Velocity u2, Pressure p
Iter      SolEst     Damping    Stepsize #Res #Jac #Sol   LinErr   LinRes
   1     0.00052   0.5000000     0.00052   22   11   11  6.2e-15  7.8e-15
Displacement field
Iter      SolEst     Damping    Stepsize #Res #Jac #Sol   LinErr   LinRes
   1      0.0051   0.5000000      0.0051   22   11   11    3e-14  1.5e-12
Spatial mesh displacement
Iter      SolEst     Damping    Stepsize #Res #Jac #Sol   LinErr   LinRes
   1     3.8e-08   0.5000000     3.8e-08   22   11   11  1.7e-15  1.9e-15
Solution error estimates for segregated groups
0.00014, 0.00026, 0.0026, 1.9e-08
Residual error estimates for segregated groups
0.044, 32, 1.2e+03, 0.19
Segregated solver iteration 12.
Merged variables
Iter      SolEst     Damping    Stepsize #Res #Jac #Sol   LinErr   LinRes
   1     5.3e-05   0.3500000     5.3e-05   46   34   34  7.2e-14  3.3e-15
   2     3.4e-05   0.3500000     3.4e-05   47   35   35  9.6e-14  2.9e-15
   3     2.2e-05   0.3500000     2.2e-05   48   36   36  8.9e-14  3.9e-15
Velocity u2, Pressure p
Iter      SolEst     Damping    Stepsize #Res #Jac #Sol   LinErr   LinRes
   1     0.00026   0.5000000     0.00026   24   12   12  2.7e-15  7.8e-15
Displacement field
Iter      SolEst     Damping    Stepsize #Res #Jac #Sol   LinErr   LinRes
   1      0.0028   0.5000000      0.0028   24   12   12    6e-14  1.5e-12
Spatial mesh displacement
Iter      SolEst     Damping    Stepsize #Res #Jac #Sol   LinErr   LinRes
   1     2.2e-08   0.5000000     2.2e-08   24   12   12  1.8e-15  1.9e-15
Solution error estimates for segregated groups
3.8e-05, 0.00013, 0.0014, 1.1e-08
```

```
Residual error estimates for segregated groups
0.013, 16, 1.2e+03, 0.18
Segregated solver iteration 13.
Merged variables
Iter      SolEst      Damping    Stepsize #Res #Jac #Sol   LinErr   LinRes
   1     1.5e-05   0.3500000     1.5e-05   50   37   37  7.5e-14    3e-15
   2     9.5e-06   0.3500000     9.5e-06   51   38   38    5e-14  2.8e-15
   3     6.1e-06   0.3500000     6.1e-06   52   39   39  1.9e-13    3e-15
Velocity u2, Pressure p
Iter      SolEst      Damping    Stepsize #Res #Jac #Sol   LinErr   LinRes
   1     0.00013   0.5000000     0.00013   26   13   13  4.6e-15  7.7e-15
Displacement field
Iter      SolEst      Damping    Stepsize #Res #Jac #Sol   LinErr   LinRes
   1      0.0015   0.5000000      0.0015   26   13   13  7.3e-14  1.5e-12
Spatial mesh displacement
Iter      SolEst      Damping    Stepsize #Res #Jac #Sol   LinErr   LinRes
   1     1.3e-08   0.5000000     1.3e-08   26   13   13  1.4e-15  1.9e-15
Solution error estimates for segregated groups
1.1e-05, 6.5e-05, 0.00076, 6.6e-09
Residual error estimates for segregated groups
0.0041, 8, 1.2e+03, 0.19
Solution time: 162 s. (2 minutes, 42 seconds)
Physical memory: 4.59 GB
Virtual memory: 25.54 GB
Ended at Jan 31, 2025, 5:43:47 PM.
----- Stationary Solver 1 in study1/Approach Curve Solver (sol1) -------------->
```

d=2405 (su21)

GENERAL

Description	Value
Solution	d=2405

LOG

```
<---- Compile Equations: Stationary in study1/Approach Curve Solver (sol1) -----
Started at Jan 31, 2025, 5:43:47 PM.
Parameter d = 2.4050000000000003E-6 (m).
Geometry shape function: Linear serendipity
Number of vertex elements: 18
Number of boundary elements: 6283
Number of elements: 156897
Minimum element quality: 0.4586
Time: 9 s.
Physical memory: 2.86 GB
Virtual memory: 15.22 GB
Ended at Jan 31, 2025, 5:43:56 PM.
----- Compile Equations: Stationary in study1/Approach Curve Solver (sol1) ---->

<---- Dependent Variables 1 in study1/Approach Curve Solver (sol1) -------------
Started at Jan 31, 2025, 5:43:56 PM.
Solution time: 0 s.
Physical memory: 2.86 GB
Virtual memory: 15.22 GB
Ended at Jan 31, 2025, 5:43:56 PM.
----- Dependent Variables 1 in study1/Approach Curve Solver (sol1) ------------>

<---- Stationary Solver 1 in study1/Approach Curve Solver (sol1) ---------------
Started at Jan 31, 2025, 5:43:56 PM.
Segregated solver
Segregated Step 11 does not have any components solved for.
Number of degrees of freedom solved for: 798018 (plus 11989 internal DOFs).
Segregated solver iteration 1.
Merged variables
Nonsymmetric matrix found.
Scales for dependent variables:
Concentration (comp1.Cl): 1.4e+02
Concentration (comp1.K): 1.4e+02
Electric potential (comp1.V): 1.9e+02
Orthonormal null-space function used.
Iter      SolEst     Damping    Stepsize #Res #Jac #Sol   LinErr   LinRes
   1        0.83   0.3500000        0.83    2    1    1  4.8e-11  2.9e-15
   2        0.81   0.3500000        0.81    3    2    2  1.6e-11  2.8e-15
   3         0.8   0.3500000         0.8    4    3    3  2.3e-11  2.7e-15
Velocity u2, Pressure p
Nonsymmetric matrix found.
Scales for dependent variables:
Pressure (comp1.p): 1
Velocity field (spatial frame) (comp1.u2): 0.00018
Orthonormal null-space function used.
Iter      SolEst     Damping    Stepsize #Res #Jac #Sol   LinErr   LinRes
   1         1.1   0.5000000         1.1    2    1    1  1.6e-15  2.3e-15
Displacement field
Nonsymmetric matrix found.
Scales for dependent variables:
Auxiliary pressure (comp1.solid.lemm1.pw): 1
Displacement field (comp1.u): 2.7e-18
Orthonormal null-space function used.
Iter      SolEst     Damping    Stepsize #Res #Jac #Sol   LinErr   LinRes
   1         1.9   0.5000000         1.9    2    1    1  5.9e-14  1.5e-12
Spatial mesh displacement
```

```
Symmetric matrices found.
Scales for dependent variables:
Spatial mesh displacement (comp1.spatial.disp): 1.2e-09
Orthonormal null-space function used.
Iter      SolEst     Damping    Stepsize #Res #Jac #Sol   LinErr   LinRes
   1     5.9e-07   0.5000000     5.9e-07    2    1    1  1.7e-15  1.9e-15
Solution error estimates for segregated groups
1.4, 0.54, 0.95, 3e-07
Residual error estimates for segregated groups
7.9e+03, 8.3e+04, 2.2e+04, 0.18
Segregated solver iteration 2.
Merged variables
Scales for dependent variables:
Concentration (comp1.Cl): 1.4e+02
Concentration (comp1.K): 1.4e+02
Electric potential (comp1.V): 0.064
Iter      SolEst     Damping    Stepsize #Res #Jac #Sol   LinErr   LinRes
   1        0.79   0.3500000        0.79    6    4    4  4.8e-14  2.7e-15
   2        0.77   0.3500000        0.77    7    5    5  4.7e-14  2.8e-15
   3        0.76   0.3500000        0.76    8    6    6  9.4e-14  2.8e-15
Velocity u2, Pressure p
Scales for dependent variables:
Pressure (comp1.p): 3.5e+02
Velocity field (spatial frame) (comp1.u2): 0.00071
Iter      SolEst     Damping    Stepsize #Res #Jac #Sol   LinErr   LinRes
   1        0.36   0.5000000        0.36    4    2    2  2.8e-15  7.9e-15
Displacement field
Iter      SolEst     Damping    Stepsize #Res #Jac #Sol   LinErr   LinRes
   1        0.95   0.5000000        0.95    4    2    2  2.5e-14  1.5e-12
Spatial mesh displacement
Iter      SolEst     Damping    Stepsize #Res #Jac #Sol   LinErr   LinRes
   1     8.9e-07   0.5000000     8.9e-07    4    2    2  1.4e-15  1.8e-15
Solution error estimates for segregated groups
1.3, 0.18, 0.48, 4.4e-07
Residual error estimates for segregated groups
8.1e+02, 1.3e+04, 2.2e+04, 0.18
Segregated solver iteration 3.
Merged variables
Iter      SolEst     Damping    Stepsize #Res #Jac #Sol   LinErr   LinRes
   1        0.75   0.3500000        0.75   10    7    7  8.6e-14  2.7e-15
   2        0.74   0.3500000        0.74   11    8    8  5.5e-14    3e-15
   3        0.72   0.3500000        0.72   12    9    9  4.4e-14  2.7e-15
Velocity u2, Pressure p
Iter      SolEst     Damping    Stepsize #Res #Jac #Sol   LinErr   LinRes
   1        0.15   0.5000000        0.15    6    3    3  2.4e-15  7.9e-15
Displacement field
Iter      SolEst     Damping    Stepsize #Res #Jac #Sol   LinErr   LinRes
   1        0.52   0.5000000        0.52    6    3    3  7.7e-14  1.5e-12
Spatial mesh displacement
Iter      SolEst     Damping    Stepsize #Res #Jac #Sol   LinErr   LinRes
   1     8.9e-07   0.5000000     8.9e-07    6    3    3  1.9e-15  1.8e-15
Solution error estimates for segregated groups
1.2, 0.077, 0.26, 4.4e-07
Residual error estimates for segregated groups
2.2e+02, 7e+03, 1.6e+04, 0.19
Segregated solver iteration 4.
```

```
Merged variables
Iter      SolEst     Damping    Stepsize #Res #Jac #Sol   LinErr   LinRes
   1         0.7   0.3500000         0.7   14   10   10  1.3e-13  2.8e-15
   2        0.68   0.3500000        0.68   15   11   11  7.1e-14  2.8e-15
   3         0.6   0.3500000         0.6   16   12   12  9.2e-14  2.7e-15
Velocity u2, Pressure p
Iter      SolEst     Damping    Stepsize #Res #Jac #Sol   LinErr   LinRes
   1       0.071   0.5000000       0.071    8    4    4  3.1e-15  7.9e-15
Displacement field
Iter      SolEst     Damping    Stepsize #Res #Jac #Sol   LinErr   LinRes
   1        0.29   0.5000000        0.29    8    4    4  3.4e-14  1.5e-12
Spatial mesh displacement
Iter      SolEst     Damping    Stepsize #Res #Jac #Sol   LinErr   LinRes
   1     7.4e-07   0.5000000     7.4e-07    8    4    4  1.8e-15  1.8e-15
Solution error estimates for segregated groups
1, 0.036, 0.15, 3.7e-07
Residual error estimates for segregated groups
85, 3.7e+03, 1.1e+04, 0.18
Segregated solver iteration 5.
Merged variables
Iter      SolEst     Damping    Stepsize #Res #Jac #Sol   LinErr   LinRes
   1        0.42   0.3500000        0.42   18   13   13  8.6e-14  2.9e-15
   2        0.28   0.3500000        0.28   19   14   14  6.8e-14  2.8e-15
   3        0.18   0.3500000        0.18   20   15   15    1e-13  2.8e-15
Velocity u2, Pressure p
Iter      SolEst     Damping    Stepsize #Res #Jac #Sol   LinErr   LinRes
   1       0.035   0.5000000       0.035   10    5    5  3.5e-15  7.8e-15
Displacement field
Iter      SolEst     Damping    Stepsize #Res #Jac #Sol   LinErr   LinRes
   1        0.17   0.5000000        0.17   10    5    5  8.6e-14  1.5e-12
Spatial mesh displacement
Iter      SolEst     Damping    Stepsize #Res #Jac #Sol   LinErr   LinRes
   1     5.5e-07   0.5000000     5.5e-07   10    5    5  1.5e-15  1.8e-15
Solution error estimates for segregated groups
0.32, 0.017, 0.084, 2.8e-07
Residual error estimates for segregated groups
33, 1.9e+03, 6.9e+03, 0.19
Segregated solver iteration 6.
Merged variables
Iter      SolEst     Damping    Stepsize #Res #Jac #Sol   LinErr   LinRes
   1        0.12   0.3500000        0.12   22   16   16  1.2e-13  2.9e-15
   2       0.079   0.3500000       0.079   23   17   17  6.5e-14  2.8e-15
   3       0.052   0.3500000       0.052   24   18   18  8.6e-14    3e-15
Velocity u2, Pressure p
Iter      SolEst     Damping    Stepsize #Res #Jac #Sol   LinErr   LinRes
   1       0.017   0.5000000       0.017   12    6    6  4.4e-15  7.8e-15
Displacement field
Iter      SolEst     Damping    Stepsize #Res #Jac #Sol   LinErr   LinRes
   1       0.095   0.5000000       0.095   12    6    6    8e-14  1.5e-12
Spatial mesh displacement
Iter      SolEst     Damping    Stepsize #Res #Jac #Sol   LinErr   LinRes
   1     3.9e-07   0.5000000     3.9e-07   12    6    6  2.6e-15  1.9e-15
Solution error estimates for segregated groups
0.089, 0.0085, 0.048, 1.9e-07
Residual error estimates for segregated groups
12, 9.5e+02, 4.2e+03, 0.19
```

```
Segregated solver iteration 7.
Merged variables
Iter      SolEst     Damping    Stepsize #Res #Jac #Sol   LinErr   LinRes
   1       0.034   0.3500000       0.034   26   19   19    7e-14  2.8e-15
   2       0.022   0.3500000       0.022   27   20   20  4.1e-14  2.8e-15
   3       0.014   0.3500000       0.014   28   21   21  8.2e-14  3.1e-15
Velocity u2, Pressure p
Iter      SolEst     Damping    Stepsize #Res #Jac #Sol   LinErr   LinRes
   1      0.0084   0.5000000      0.0084   14    7    7  5.2e-15  7.9e-15
Displacement field
Iter      SolEst     Damping    Stepsize #Res #Jac #Sol   LinErr   LinRes
   1       0.054   0.5000000       0.054   14    7    7  2.7e-13  1.5e-12
Spatial mesh displacement
Iter      SolEst     Damping    Stepsize #Res #Jac #Sol   LinErr   LinRes
   1     2.6e-07   0.5000000     2.6e-07   14    7    7  1.4e-15  1.8e-15
Solution error estimates for segregated groups
0.025, 0.0042, 0.027, 1.3e-07
Residual error estimates for segregated groups
4.1, 4.8e+02, 2.7e+03, 0.18
Segregated solver iteration 8.
Merged variables
Iter      SolEst     Damping    Stepsize #Res #Jac #Sol   LinErr   LinRes
   1      0.0093   0.3500000      0.0093   30   22   22  6.1e-14  2.9e-15
   2       0.006   0.3500000       0.006   31   23   23    5e-14  2.9e-15
   3      0.0039   0.3500000      0.0039   32   24   24  1.3e-13  2.9e-15
Velocity u2, Pressure p
Iter      SolEst     Damping    Stepsize #Res #Jac #Sol   LinErr   LinRes
   1      0.0042   0.5000000      0.0042   16    8    8  2.6e-15  7.9e-15
Displacement field
Iter      SolEst     Damping    Stepsize #Res #Jac #Sol   LinErr   LinRes
   1        0.03   0.5000000        0.03   16    8    8  1.1e-13  1.5e-12
Spatial mesh displacement
Iter      SolEst     Damping    Stepsize #Res #Jac #Sol   LinErr   LinRes
   1     1.7e-07   0.5000000     1.7e-07   16    8    8  2.1e-15  1.8e-15
Solution error estimates for segregated groups
0.0068, 0.0021, 0.015, 8.3e-08
Residual error estimates for segregated groups
1.4, 2.4e+02, 1.8e+03, 0.19
Segregated solver iteration 9.
Merged variables
Iter      SolEst     Damping    Stepsize #Res #Jac #Sol   LinErr   LinRes
   1      0.0026   0.3500000      0.0026   34   25   25  8.7e-14    3e-15
   2      0.0017   0.3500000      0.0017   35   26   26  7.8e-14  2.8e-15
   3      0.0011   0.3500000      0.0011   36   27   27  1.6e-13  2.8e-15
Velocity u2, Pressure p
Iter      SolEst     Damping    Stepsize #Res #Jac #Sol   LinErr   LinRes
   1      0.0021   0.5000000      0.0021   18    9    9  2.2e-15  7.7e-15
Displacement field
Iter      SolEst     Damping    Stepsize #Res #Jac #Sol   LinErr   LinRes
   1       0.017   0.5000000       0.017   18    9    9  6.9e-14  1.5e-12
Spatial mesh displacement
Iter      SolEst     Damping    Stepsize #Res #Jac #Sol   LinErr   LinRes
   1       1e-07   0.5000000       1e-07   18    9    9  1.4e-15  1.8e-15
Solution error estimates for segregated groups
0.0019, 0.001, 0.0085, 5.2e-08
Residual error estimates for segregated groups
```

```
0.44, 1.2e+02, 1.4e+03, 0.2
Segregated solver iteration 10.
Merged variables
Iter      SolEst     Damping    Stepsize #Res #Jac #Sol   LinErr   LinRes
   1      0.0007   0.3500000      0.0007   38   28   28  6.2e-14  2.8e-15
   2     0.00046   0.3500000     0.00046   39   29   29  4.8e-14  2.9e-15
   3      0.0003   0.3500000      0.0003   40   30   30  1.1e-13  2.8e-15
Velocity u2, Pressure p
Iter      SolEst     Damping    Stepsize #Res #Jac #Sol   LinErr   LinRes
   1       0.001   0.5000000       0.001   20   10   10  1.9e-15  7.9e-15
Displacement field
Iter      SolEst     Damping    Stepsize #Res #Jac #Sol   LinErr   LinRes
   1      0.0094   0.5000000      0.0094   20   10   10  4.4e-14  1.5e-12
Spatial mesh displacement
Iter      SolEst     Damping    Stepsize #Res #Jac #Sol   LinErr   LinRes
   1     6.3e-08   0.5000000     6.3e-08   20   10   10  1.8e-15  1.8e-15
Solution error estimates for segregated groups
0.00051, 0.00052, 0.0047, 3.2e-08
Residual error estimates for segregated groups
0.14, 60, 1.2e+03, 0.19
Segregated solver iteration 11.
Merged variables
Iter      SolEst     Damping    Stepsize #Res #Jac #Sol   LinErr   LinRes
   1     0.00019   0.3500000     0.00019   42   31   31  7.5e-14  2.8e-15
   2     0.00013   0.3500000     0.00013   43   32   32  6.9e-14  2.8e-15
   3     8.1e-05   0.3500000     8.1e-05   44   33   33  1.2e-13  2.9e-15
Velocity u2, Pressure p
Iter      SolEst     Damping    Stepsize #Res #Jac #Sol   LinErr   LinRes
   1     0.00052   0.5000000     0.00052   22   11   11    3e-15  7.8e-15
Displacement field
Iter      SolEst     Damping    Stepsize #Res #Jac #Sol   LinErr   LinRes
   1      0.0051   0.5000000      0.0051   22   11   11  2.7e-13  1.5e-12
Spatial mesh displacement
Iter      SolEst     Damping    Stepsize #Res #Jac #Sol   LinErr   LinRes
   1     3.8e-08   0.5000000     3.8e-08   22   11   11  1.4e-15  1.9e-15
Solution error estimates for segregated groups
0.00014, 0.00026, 0.0026, 1.9e-08
Residual error estimates for segregated groups
0.044, 30, 1.2e+03, 0.19
Segregated solver iteration 12.
Merged variables
Iter      SolEst     Damping    Stepsize #Res #Jac #Sol   LinErr   LinRes
   1     5.3e-05   0.3500000     5.3e-05   46   34   34  8.3e-14  2.9e-15
   2     3.4e-05   0.3500000     3.4e-05   47   35   35  1.6e-13  2.8e-15
   3     2.2e-05   0.3500000     2.2e-05   48   36   36  1.3e-13  2.8e-15
Velocity u2, Pressure p
Iter      SolEst     Damping    Stepsize #Res #Jac #Sol   LinErr   LinRes
   1     0.00026   0.5000000     0.00026   24   12   12  2.7e-15  7.8e-15
Displacement field
Iter      SolEst     Damping    Stepsize #Res #Jac #Sol   LinErr   LinRes
   1      0.0028   0.5000000      0.0028   24   12   12  7.4e-14  1.5e-12
Spatial mesh displacement
Iter      SolEst     Damping    Stepsize #Res #Jac #Sol   LinErr   LinRes
   1     2.2e-08   0.5000000     2.2e-08   24   12   12  1.6e-15  1.8e-15
Solution error estimates for segregated groups
3.8e-05, 0.00013, 0.0014, 1.1e-08
```

```
Residual error estimates for segregated groups
0.013, 15, 1.2e+03, 0.17
Segregated solver iteration 13.
Merged variables
Iter      SolEst      Damping    Stepsize #Res #Jac #Sol   LinErr   LinRes
   1     1.5e-05    0.3500000     1.5e-05    50   37   37  8.5e-14  2.8e-15
   2     9.5e-06    0.3500000     9.5e-06    51   38   38  1.3e-13  2.8e-15
   3     6.1e-06    0.3500000     6.1e-06    52   39   39  7.4e-14  2.8e-15
Velocity u2, Pressure p
Iter      SolEst      Damping    Stepsize #Res #Jac #Sol   LinErr   LinRes
   1     0.00013    0.5000000     0.00013    26   13   13  2.8e-15  7.9e-15
Displacement field
Iter      SolEst      Damping    Stepsize #Res #Jac #Sol   LinErr   LinRes
   1      0.0015    0.5000000      0.0015    26   13   13  8.6e-14  1.5e-12
Spatial mesh displacement
Iter      SolEst      Damping    Stepsize #Res #Jac #Sol   LinErr   LinRes
   1     1.3e-08    0.5000000     1.3e-08    26   13   13  1.8e-15  1.8e-15
Solution error estimates for segregated groups
1.1e-05, 6.5e-05, 0.00076, 6.6e-09
Residual error estimates for segregated groups
0.0041, 7.5, 1.2e+03, 0.19
Solution time: 158 s. (2 minutes, 38 seconds)
Physical memory: 4.51 GB
Virtual memory: 25.5 GB
Ended at Jan 31, 2025, 5:46:34 PM.
----- Stationary Solver 1 in study1/Approach Curve Solver (sol1) -------------->
```

d=2400 (su22)

GENERAL

Description	Value
Solution	d=2400

LOG

```
<---- Compile Equations: Stationary in study1/Approach Curve Solver (sol1) -----
Started at Jan 31, 2025, 5:46:34 PM.
Parameter d = 2.4000000000000003E-6 (m).
Geometry shape function: Linear serendipity
Number of vertex elements: 18
Number of boundary elements: 6279
Number of elements: 156857
Minimum element quality: 0.4767
Time: 8 s.
Physical memory: 2.84 GB
Virtual memory: 15.19 GB
Ended at Jan 31, 2025, 5:46:43 PM.
----- Compile Equations: Stationary in study1/Approach Curve Solver (sol1) ---->

<---- Dependent Variables 1 in study1/Approach Curve Solver (sol1) -------------
Started at Jan 31, 2025, 5:46:43 PM.
Solution time: 0 s.
Physical memory: 2.84 GB
Virtual memory: 15.19 GB
Ended at Jan 31, 2025, 5:46:43 PM.
----- Dependent Variables 1 in study1/Approach Curve Solver (sol1) ------------>

<---- Stationary Solver 1 in study1/Approach Curve Solver (sol1) ---------------
Started at Jan 31, 2025, 5:46:43 PM.
Segregated solver
Segregated Step 11 does not have any components solved for.
Number of degrees of freedom solved for: 798016 (plus 11981 internal DOFs).
Segregated solver iteration 1.
Merged variables
Nonsymmetric matrix found.
Scales for dependent variables:
Concentration (comp1.Cl): 1.4e+02
Concentration (comp1.K): 1.4e+02
Electric potential (comp1.V): 1.9e+02
Orthonormal null-space function used.
Iter      SolEst     Damping    Stepsize #Res #Jac #Sol   LinErr   LinRes
   1        0.83   0.3500000        0.83    2    1    1  3.2e-11  2.7e-15
   2        0.81   0.3500000        0.81    3    2    2  2.8e-11  2.8e-15
   3         0.8   0.3500000         0.8    4    3    3  1.2e-10  2.7e-15
Velocity u2, Pressure p
Nonsymmetric matrix found.
Scales for dependent variables:
Pressure (comp1.p): 1
Velocity field (spatial frame) (comp1.u2): 0.00019
Orthonormal null-space function used.
Iter      SolEst     Damping    Stepsize #Res #Jac #Sol   LinErr   LinRes
   1         1.1   0.5000000         1.1    2    1    1  1.6e-15  2.3e-15
Displacement field
Nonsymmetric matrix found.
Scales for dependent variables:
Auxiliary pressure (comp1.solid.lemm1.pw): 1
Displacement field (comp1.u): 2.8e-18
Orthonormal null-space function used.
Iter      SolEst     Damping    Stepsize #Res #Jac #Sol   LinErr   LinRes
   1         1.9   0.5000000         1.9    2    1    1  2.2e-14  1.5e-12
Spatial mesh displacement
```

```
Symmetric matrices found.
Scales for dependent variables:
Spatial mesh displacement (comp1.spatial.disp): 1.2e-09
Orthonormal null-space function used.
Iter      SolEst     Damping    Stepsize #Res #Jac #Sol   LinErr   LinRes
   1     5.9e-07   0.5000000     5.9e-07    2    1    1  1.8e-15  1.8e-15
Solution error estimates for segregated groups
1.4, 0.54, 0.95, 2.9e-07
Residual error estimates for segregated groups
7.9e+03, 8.3e+04, 2.2e+04, 0.17
Segregated solver iteration 2.
Merged variables
Scales for dependent variables:
Concentration (comp1.Cl): 1.4e+02
Concentration (comp1.K): 1.4e+02
Electric potential (comp1.V): 0.064
Iter      SolEst     Damping    Stepsize #Res #Jac #Sol   LinErr   LinRes
   1        0.79   0.3500000        0.79    6    4    4  1.4e-13  3.9e-15
   2        0.77   0.3500000        0.77    7    5    5  7.5e-14  4.9e-15
   3        0.76   0.3500000        0.76    8    6    6  8.9e-14  3.5e-15
Velocity u2, Pressure p
Scales for dependent variables:
Pressure (comp1.p): 3.5e+02
Velocity field (spatial frame) (comp1.u2): 0.00071
Iter      SolEst     Damping    Stepsize #Res #Jac #Sol   LinErr   LinRes
   1        0.36   0.5000000        0.36    4    2    2  4.5e-15  7.9e-15
Displacement field
Iter      SolEst     Damping    Stepsize #Res #Jac #Sol   LinErr   LinRes
   1        0.95   0.5000000        0.95    4    2    2  9.5e-14  1.5e-12
Spatial mesh displacement
Iter      SolEst     Damping    Stepsize #Res #Jac #Sol   LinErr   LinRes
   1     8.8e-07   0.5000000     8.8e-07    4    2    2  1.8e-15  1.8e-15
Solution error estimates for segregated groups
1.3, 0.18, 0.48, 4.4e-07
Residual error estimates for segregated groups
8.1e+02, 1.4e+04, 2.2e+04, 0.2
Segregated solver iteration 3.
Merged variables
Iter      SolEst     Damping    Stepsize #Res #Jac #Sol   LinErr   LinRes
   1        0.75   0.3500000        0.75   10    7    7  5.1e-14  3.5e-15
   2        0.74   0.3500000        0.74   11    8    8  8.6e-14  3.1e-15
   3        0.72   0.3500000        0.72   12    9    9  7.8e-14  4.3e-15
Velocity u2, Pressure p
Iter      SolEst     Damping    Stepsize #Res #Jac #Sol   LinErr   LinRes
   1        0.15   0.5000000        0.15    6    3    3    2e-15    8e-15
Displacement field
Iter      SolEst     Damping    Stepsize #Res #Jac #Sol   LinErr   LinRes
   1        0.52   0.5000000        0.52    6    3    3  2.3e-13  1.5e-12
Spatial mesh displacement
Iter      SolEst     Damping    Stepsize #Res #Jac #Sol   LinErr   LinRes
   1     8.8e-07   0.5000000     8.8e-07    6    3    3  1.3e-15  1.8e-15
Solution error estimates for segregated groups
1.2, 0.077, 0.26, 4.4e-07
Residual error estimates for segregated groups
2.2e+02, 7.7e+03, 1.6e+04, 0.19
Segregated solver iteration 4.
```

```
Merged variables
Iter      SolEst     Damping    Stepsize #Res #Jac #Sol   LinErr   LinRes
   1         0.7   0.3500000         0.7   14   10   10  1.1e-13  3.7e-15
   2        0.68   0.3500000        0.68   15   11   11  1.5e-13  4.5e-15
   3         0.6   0.3500000         0.6   16   12   12  1.3e-13  3.2e-15
Velocity u2, Pressure p
Iter      SolEst     Damping    Stepsize #Res #Jac #Sol   LinErr   LinRes
   1       0.071   0.5000000       0.071    8    4    4  3.2e-15  7.9e-15
Displacement field
Iter      SolEst     Damping    Stepsize #Res #Jac #Sol   LinErr   LinRes
   1        0.29   0.5000000        0.29    8    4    4  3.4e-14  1.5e-12
Spatial mesh displacement
Iter      SolEst     Damping    Stepsize #Res #Jac #Sol   LinErr   LinRes
   1     7.4e-07   0.5000000     7.4e-07    8    4    4  1.8e-15  1.8e-15
Solution error estimates for segregated groups
1, 0.036, 0.15, 3.7e-07
Residual error estimates for segregated groups
85, 4.1e+03, 1.1e+04, 0.18
Segregated solver iteration 5.
Merged variables
Iter      SolEst     Damping    Stepsize #Res #Jac #Sol   LinErr   LinRes
   1        0.42   0.3500000        0.42   18   13   13  9.2e-14  3.3e-15
   2        0.28   0.3500000        0.28   19   14   14  1.3e-13  3.1e-15
   3        0.18   0.3500000        0.18   20   15   15  9.8e-14  3.1e-15
Velocity u2, Pressure p
Iter      SolEst     Damping    Stepsize #Res #Jac #Sol   LinErr   LinRes
   1       0.035   0.5000000       0.035   10    5    5  3.1e-15  7.9e-15
Displacement field
Iter      SolEst     Damping    Stepsize #Res #Jac #Sol   LinErr   LinRes
   1        0.17   0.5000000        0.17   10    5    5  3.3e-14  1.5e-12
Spatial mesh displacement
Iter      SolEst     Damping    Stepsize #Res #Jac #Sol   LinErr   LinRes
   1     5.5e-07   0.5000000     5.5e-07   10    5    5  1.4e-15  1.9e-15
Solution error estimates for segregated groups
0.32, 0.017, 0.084, 2.8e-07
Residual error estimates for segregated groups
33, 2.1e+03, 6.9e+03, 0.19
Segregated solver iteration 6.
Merged variables
Iter      SolEst     Damping    Stepsize #Res #Jac #Sol   LinErr   LinRes
   1        0.12   0.3500000        0.12   22   16   16    5e-14  4.5e-15
   2       0.079   0.3500000       0.079   23   17   17  6.1e-14  3.1e-15
   3       0.052   0.3500000       0.052   24   18   18  5.4e-14  3.7e-15
Velocity u2, Pressure p
Iter      SolEst     Damping    Stepsize #Res #Jac #Sol   LinErr   LinRes
   1       0.017   0.5000000       0.017   12    6    6  2.6e-15    8e-15
Displacement field
Iter      SolEst     Damping    Stepsize #Res #Jac #Sol   LinErr   LinRes
   1       0.095   0.5000000       0.095   12    6    6  6.9e-14  1.5e-12
Spatial mesh displacement
Iter      SolEst     Damping    Stepsize #Res #Jac #Sol   LinErr   LinRes
   1     3.9e-07   0.5000000     3.9e-07   12    6    6  1.4e-15  1.8e-15
Solution error estimates for segregated groups
0.089, 0.0085, 0.048, 1.9e-07
Residual error estimates for segregated groups
12, 1.1e+03, 4.2e+03, 0.19
```

```
Segregated solver iteration 7.
Merged variables
Iter      SolEst     Damping    Stepsize #Res #Jac #Sol   LinErr   LinRes
   1       0.034   0.3500000       0.034   26   19   19  1.1e-13  3.3e-15
   2       0.022   0.3500000       0.022   27   20   20  1.1e-13  3.1e-15
   3       0.014   0.3500000       0.014   28   21   21  1.2e-13  3.3e-15
Velocity u2, Pressure p
Iter      SolEst     Damping    Stepsize #Res #Jac #Sol   LinErr   LinRes
   1      0.0084   0.5000000      0.0084   14    7    7    2e-15    8e-15
Displacement field
Iter      SolEst     Damping    Stepsize #Res #Jac #Sol   LinErr   LinRes
   1       0.054   0.5000000       0.054   14    7    7  7.9e-14  1.5e-12
Spatial mesh displacement
Iter      SolEst     Damping    Stepsize #Res #Jac #Sol   LinErr   LinRes
   1     2.6e-07   0.5000000     2.6e-07   14    7    7  1.5e-15  1.8e-15
Solution error estimates for segregated groups
0.025, 0.0042, 0.027, 1.3e-07
Residual error estimates for segregated groups
4.2, 5.3e+02, 2.6e+03, 0.19
Segregated solver iteration 8.
Merged variables
Iter      SolEst     Damping    Stepsize #Res #Jac #Sol   LinErr   LinRes
   1      0.0093   0.3500000      0.0093   30   22   22  1.4e-13    3e-15
   2      0.0061   0.3500000      0.0061   31   23   23  1.4e-13    3e-15
   3      0.0039   0.3500000      0.0039   32   24   24  1.3e-13    3e-15
Velocity u2, Pressure p
Iter      SolEst     Damping    Stepsize #Res #Jac #Sol   LinErr   LinRes
   1      0.0042   0.5000000      0.0042   16    8    8  2.6e-15  7.9e-15
Displacement field
Iter      SolEst     Damping    Stepsize #Res #Jac #Sol   LinErr   LinRes
   1        0.03   0.5000000        0.03   16    8    8  6.3e-14  1.5e-12
Spatial mesh displacement
Iter      SolEst     Damping    Stepsize #Res #Jac #Sol   LinErr   LinRes
   1     1.7e-07   0.5000000     1.7e-07   16    8    8  1.4e-15  1.8e-15
Solution error estimates for segregated groups
0.0068, 0.0021, 0.015, 8.3e-08
Residual error estimates for segregated groups
1.4, 2.7e+02, 1.8e+03, 0.19
Segregated solver iteration 9.
Merged variables
Iter      SolEst     Damping    Stepsize #Res #Jac #Sol   LinErr   LinRes
   1      0.0026   0.3500000      0.0026   34   25   25  7.1e-14  2.9e-15
   2      0.0017   0.3500000      0.0017   35   26   26  1.7e-13  3.3e-15
   3      0.0011   0.3500000      0.0011   36   27   27  1.1e-13  3.6e-15
Velocity u2, Pressure p
Iter      SolEst     Damping    Stepsize #Res #Jac #Sol   LinErr   LinRes
   1      0.0021   0.5000000      0.0021   18    9    9  2.9e-15  7.9e-15
Displacement field
Iter      SolEst     Damping    Stepsize #Res #Jac #Sol   LinErr   LinRes
   1       0.017   0.5000000       0.017   18    9    9  5.9e-14  1.5e-12
Spatial mesh displacement
Iter      SolEst     Damping    Stepsize #Res #Jac #Sol   LinErr   LinRes
   1       1e-07   0.5000000       1e-07   18    9    9  1.6e-15  1.8e-15
Solution error estimates for segregated groups
0.0019, 0.001, 0.0085, 5.2e-08
Residual error estimates for segregated groups
```

```
0.45, 1.3e+02, 1.4e+03, 0.2
Segregated solver iteration 10.
Merged variables
Iter      SolEst     Damping    Stepsize #Res #Jac #Sol   LinErr   LinRes
   1      0.0007   0.3500000      0.0007   38   28   28  1.6e-13  5.5e-15
   2     0.00046   0.3500000     0.00046   39   29   29  9.2e-14  3.4e-15
   3      0.0003   0.3500000      0.0003   40   30   30  1.5e-13  3.3e-15
Velocity u2, Pressure p
Iter      SolEst     Damping    Stepsize #Res #Jac #Sol   LinErr   LinRes
   1       0.001   0.5000000       0.001   20   10   10  2.4e-15  7.9e-15
Displacement field
Iter      SolEst     Damping    Stepsize #Res #Jac #Sol   LinErr   LinRes
   1      0.0094   0.5000000      0.0094   20   10   10    7e-14  1.5e-12
Spatial mesh displacement
Iter      SolEst     Damping    Stepsize #Res #Jac #Sol   LinErr   LinRes
   1     6.3e-08   0.5000000     6.3e-08   20   10   10  1.6e-15  1.8e-15
Solution error estimates for segregated groups
0.00051, 0.00052, 0.0047, 3.2e-08
Residual error estimates for segregated groups
0.14, 67, 1.2e+03, 0.19
Segregated solver iteration 11.
Merged variables
Iter      SolEst     Damping    Stepsize #Res #Jac #Sol   LinErr   LinRes
   1     0.00019   0.3500000     0.00019   42   31   31  8.5e-14  3.2e-15
   2     0.00013   0.3500000     0.00013   43   32   32  7.9e-14  3.4e-15
   3     8.2e-05   0.3500000     8.2e-05   44   33   33  6.2e-14  3.8e-15
Velocity u2, Pressure p
Iter      SolEst     Damping    Stepsize #Res #Jac #Sol   LinErr   LinRes
   1     0.00052   0.5000000     0.00052   22   11   11    3e-15    8e-15
Displacement field
Iter      SolEst     Damping    Stepsize #Res #Jac #Sol   LinErr   LinRes
   1      0.0051   0.5000000      0.0051   22   11   11    6e-14  1.5e-12
Spatial mesh displacement
Iter      SolEst     Damping    Stepsize #Res #Jac #Sol   LinErr   LinRes
   1     3.8e-08   0.5000000     3.8e-08   22   11   11    2e-15  1.8e-15
Solution error estimates for segregated groups
0.00014, 0.00026, 0.0026, 1.9e-08
Residual error estimates for segregated groups
0.044, 34, 1.2e+03, 0.19
Segregated solver iteration 12.
Merged variables
Iter      SolEst     Damping    Stepsize #Res #Jac #Sol   LinErr   LinRes
   1     5.3e-05   0.3500000     5.3e-05   46   34   34  1.3e-13  3.4e-15
   2     3.5e-05   0.3500000     3.5e-05   47   35   35  5.6e-14  4.4e-15
   3     2.2e-05   0.3500000     2.2e-05   48   36   36  8.3e-14    3e-15
Velocity u2, Pressure p
Iter      SolEst     Damping    Stepsize #Res #Jac #Sol   LinErr   LinRes
   1     0.00026   0.5000000     0.00026   24   12   12  3.1e-15  7.8e-15
Displacement field
Iter      SolEst     Damping    Stepsize #Res #Jac #Sol   LinErr   LinRes
   1      0.0028   0.5000000      0.0028   24   12   12  1.1e-13  1.5e-12
Spatial mesh displacement
Iter      SolEst     Damping    Stepsize #Res #Jac #Sol   LinErr   LinRes
   1     2.2e-08   0.5000000     2.2e-08   24   12   12  1.4e-15  1.8e-15
Solution error estimates for segregated groups
3.9e-05, 0.00013, 0.0014, 1.1e-08
```

```
Residual error estimates for segregated groups
0.013, 17, 1.2e+03, 0.19
Segregated solver iteration 13.
Merged variables
Iter      SolEst     Damping    Stepsize #Res #Jac #Sol   LinErr   LinRes
   1     1.5e-05   0.3500000     1.5e-05   50   37   37  1.1e-13  3.6e-15
   2     9.5e-06   0.3500000     9.5e-06   51   38   38  8.7e-14  3.4e-15
   3     6.2e-06   0.3500000     6.2e-06   52   39   39  8.1e-14  4.1e-15
Velocity u2, Pressure p
Iter      SolEst     Damping    Stepsize #Res #Jac #Sol   LinErr   LinRes
   1     0.00013   0.5000000     0.00013   26   13   13  2.7e-15    8e-15
Displacement field
Iter      SolEst     Damping    Stepsize #Res #Jac #Sol   LinErr   LinRes
   1      0.0015   0.5000000      0.0015   26   13   13  5.5e-14  1.5e-12
Spatial mesh displacement
Iter      SolEst     Damping    Stepsize #Res #Jac #Sol   LinErr   LinRes
   1     1.3e-08   0.5000000     1.3e-08   26   13   13  1.6e-15  1.8e-15
Solution error estimates for segregated groups
1.1e-05, 6.5e-05, 0.00076, 6.5e-09
Residual error estimates for segregated groups
0.0041, 8.4, 1.2e+03, 0.2
Solution time: 156 s. (2 minutes, 36 seconds)
Physical memory: 4.55 GB
Virtual memory: 25.85 GB
Ended at Jan 31, 2025, 5:49:20 PM.
----- Stationary Solver 1 in study1/Approach Curve Solver (sol1) -------------->
```

d=2395 (su23)

GENERAL

Description	Value
Solution	d=2395

LOG

```
<---- Compile Equations: Stationary in study1/Approach Curve Solver (sol1) -----
Started at Jan 31, 2025, 5:49:20 PM.
Parameter d = 2.395E-6 (m).
Geometry shape function: Linear serendipity
Number of vertex elements: 18
Number of boundary elements: 6277
Number of elements: 156693
Minimum element quality: 0.4727
Time: 9 s.
Physical memory: 2.84 GB
Virtual memory: 15.2 GB
Ended at Jan 31, 2025, 5:49:28 PM.
----- Compile Equations: Stationary in study1/Approach Curve Solver (sol1) ---->

<---- Dependent Variables 1 in study1/Approach Curve Solver (sol1) -------------
Started at Jan 31, 2025, 5:49:28 PM.
Solution time: 0 s.
Physical memory: 2.84 GB
Virtual memory: 15.2 GB
Ended at Jan 31, 2025, 5:49:29 PM.
----- Dependent Variables 1 in study1/Approach Curve Solver (sol1) ------------>

<---- Stationary Solver 1 in study1/Approach Curve Solver (sol1) ---------------
Started at Jan 31, 2025, 5:49:29 PM.
Segregated solver
Segregated Step 11 does not have any components solved for.
Number of degrees of freedom solved for: 797023 (plus 11977 internal DOFs).
Segregated solver iteration 1.
Merged variables
Nonsymmetric matrix found.
Scales for dependent variables:
Concentration (comp1.Cl): 1.4e+02
Concentration (comp1.K): 1.4e+02
Electric potential (comp1.V): 1.9e+02
Orthonormal null-space function used.
Iter      SolEst     Damping    Stepsize #Res #Jac #Sol   LinErr   LinRes
   1        0.83   0.3500000        0.83    2    1    1    2e-11  2.8e-15
   2        0.81   0.3500000        0.81    3    2    2  1.8e-11  2.7e-15
   3         0.8   0.3500000         0.8    4    3    3  4.9e-11  2.7e-15
Velocity u2, Pressure p
Nonsymmetric matrix found.
Scales for dependent variables:
Pressure (comp1.p): 1
Velocity field (spatial frame) (comp1.u2): 0.00018
Orthonormal null-space function used.
Iter      SolEst     Damping    Stepsize #Res #Jac #Sol   LinErr   LinRes
   1         1.1   0.5000000         1.1    2    1    1  1.6e-15  2.2e-15
Displacement field
Nonsymmetric matrix found.
Scales for dependent variables:
Auxiliary pressure (comp1.solid.lemm1.pw): 1
Displacement field (comp1.u): 2.8e-18
Orthonormal null-space function used.
Iter      SolEst     Damping    Stepsize #Res #Jac #Sol   LinErr   LinRes
   1         1.9   0.5000000         1.9    2    1    1  3.3e-14  1.5e-12
Spatial mesh displacement
```

```
Symmetric matrices found.
Scales for dependent variables:
Spatial mesh displacement (comp1.spatial.disp): 1.2e-09
Orthonormal null-space function used.
Iter      SolEst     Damping    Stepsize #Res #Jac #Sol   LinErr   LinRes
   1     5.9e-07   0.5000000     5.9e-07    2    1    1  1.7e-15  1.8e-15
Solution error estimates for segregated groups
1.4, 0.54, 0.95, 3e-07
Residual error estimates for segregated groups
7.9e+03, 8.3e+04, 2.2e+04, 0.18
Segregated solver iteration 2.
Merged variables
Scales for dependent variables:
Concentration (comp1.Cl): 1.4e+02
Concentration (comp1.K): 1.4e+02
Electric potential (comp1.V): 0.064
Iter      SolEst     Damping    Stepsize #Res #Jac #Sol   LinErr   LinRes
   1        0.79   0.3500000        0.79    6    4    4  5.3e-14  3.4e-15
   2        0.77   0.3500000        0.77    7    5    5  5.4e-14  3.4e-15
   3        0.76   0.3500000        0.76    8    6    6    8e-14  3.2e-15
Velocity u2, Pressure p
Scales for dependent variables:
Pressure (comp1.p): 3.5e+02
Velocity field (spatial frame) (comp1.u2): 0.00071
Iter      SolEst     Damping    Stepsize #Res #Jac #Sol   LinErr   LinRes
   1        0.36   0.5000000        0.36    4    2    2  3.2e-15  7.9e-15
Displacement field
Iter      SolEst     Damping    Stepsize #Res #Jac #Sol   LinErr   LinRes
   1        0.95   0.5000000        0.95    4    2    2  6.7e-14  1.5e-12
Spatial mesh displacement
Iter      SolEst     Damping    Stepsize #Res #Jac #Sol   LinErr   LinRes
   1     8.9e-07   0.5000000     8.9e-07    4    2    2  1.6e-15  1.9e-15
Solution error estimates for segregated groups
1.3, 0.18, 0.48, 4.4e-07
Residual error estimates for segregated groups
8.1e+02, 1.4e+04, 2.2e+04, 0.18
Segregated solver iteration 3.
Merged variables
Iter      SolEst     Damping    Stepsize #Res #Jac #Sol   LinErr   LinRes
   1        0.75   0.3500000        0.75   10    7    7  1.7e-13  3.1e-15
   2        0.74   0.3500000        0.74   11    8    8  8.3e-14  3.1e-15
   3        0.72   0.3500000        0.72   12    9    9  9.5e-14  3.5e-15
Velocity u2, Pressure p
Iter      SolEst     Damping    Stepsize #Res #Jac #Sol   LinErr   LinRes
   1        0.15   0.5000000        0.15    6    3    3  2.6e-15  7.8e-15
Displacement field
Iter      SolEst     Damping    Stepsize #Res #Jac #Sol   LinErr   LinRes
   1        0.52   0.5000000        0.52    6    3    3    4e-14  1.5e-12
Spatial mesh displacement
Iter      SolEst     Damping    Stepsize #Res #Jac #Sol   LinErr   LinRes
   1     8.9e-07   0.5000000     8.9e-07    6    3    3  1.7e-15  1.8e-15
Solution error estimates for segregated groups
1.2, 0.077, 0.26, 4.4e-07
Residual error estimates for segregated groups
2.2e+02, 8e+03, 1.6e+04, 0.2
Segregated solver iteration 4.
```

```
Merged variables
Iter      SolEst     Damping    Stepsize #Res #Jac #Sol   LinErr   LinRes
   1         0.7   0.3500000         0.7   14   10   10  1.4e-13  3.7e-15
   2        0.68   0.3500000        0.68   15   11   11  6.7e-14  5.6e-15
   3         0.6   0.3500000         0.6   16   12   12  6.7e-14    4e-15
Velocity u2, Pressure p
Iter      SolEst     Damping    Stepsize #Res #Jac #Sol   LinErr   LinRes
   1       0.071   0.5000000       0.071    8    4    4  2.9e-15  7.9e-15
Displacement field
Iter      SolEst     Damping    Stepsize #Res #Jac #Sol   LinErr   LinRes
   1        0.29   0.5000000        0.29    8    4    4  3.2e-14  1.5e-12
Spatial mesh displacement
Iter      SolEst     Damping    Stepsize #Res #Jac #Sol   LinErr   LinRes
   1     7.4e-07   0.5000000     7.4e-07    8    4    4  1.5e-15  1.8e-15
Solution error estimates for segregated groups
1, 0.036, 0.15, 3.7e-07
Residual error estimates for segregated groups
85, 4.2e+03, 1.1e+04, 0.21
Segregated solver iteration 5.
Merged variables
Iter      SolEst     Damping    Stepsize #Res #Jac #Sol   LinErr   LinRes
   1        0.42   0.3500000        0.42   18   13   13  1.3e-13  3.1e-15
   2        0.28   0.3500000        0.28   19   14   14  8.7e-14  3.3e-15
   3        0.18   0.3500000        0.18   20   15   15  7.3e-14  3.5e-15
Velocity u2, Pressure p
Iter      SolEst     Damping    Stepsize #Res #Jac #Sol   LinErr   LinRes
   1       0.035   0.5000000       0.035   10    5    5  3.4e-15    8e-15
Displacement field
Iter      SolEst     Damping    Stepsize #Res #Jac #Sol   LinErr   LinRes
   1        0.17   0.5000000        0.17   10    5    5  6.8e-14  1.5e-12
Spatial mesh displacement
Iter      SolEst     Damping    Stepsize #Res #Jac #Sol   LinErr   LinRes
   1     5.5e-07   0.5000000     5.5e-07   10    5    5  1.6e-15  1.9e-15
Solution error estimates for segregated groups
0.32, 0.017, 0.084, 2.8e-07
Residual error estimates for segregated groups
33, 2.2e+03, 6.9e+03, 0.18
Segregated solver iteration 6.
Merged variables
Iter      SolEst     Damping    Stepsize #Res #Jac #Sol   LinErr   LinRes
   1        0.12   0.3500000        0.12   22   16   16  9.9e-14  4.1e-15
   2       0.079   0.3500000       0.079   23   17   17  9.2e-14  3.8e-15
   3       0.052   0.3500000       0.052   24   18   18  1.2e-13  4.4e-15
Velocity u2, Pressure p
Iter      SolEst     Damping    Stepsize #Res #Jac #Sol   LinErr   LinRes
   1       0.017   0.5000000       0.017   12    6    6  2.9e-15    8e-15
Displacement field
Iter      SolEst     Damping    Stepsize #Res #Jac #Sol   LinErr   LinRes
   1       0.095   0.5000000       0.095   12    6    6  6.6e-14  1.5e-12
Spatial mesh displacement
Iter      SolEst     Damping    Stepsize #Res #Jac #Sol   LinErr   LinRes
   1     3.9e-07   0.5000000     3.9e-07   12    6    6  1.3e-15  1.8e-15
Solution error estimates for segregated groups
0.089, 0.0085, 0.048, 1.9e-07
Residual error estimates for segregated groups
12, 1.1e+03, 4.2e+03, 0.17
```

```
Segregated solver iteration 7.
Merged variables
Iter      SolEst     Damping    Stepsize #Res #Jac #Sol   LinErr   LinRes
   1       0.034   0.3500000       0.034   26   19   19  1.1e-13  3.8e-15
   2       0.022   0.3500000       0.022   27   20   20  1.1e-13  3.8e-15
   3       0.014   0.3500000       0.014   28   21   21  5.7e-14    4e-15
Velocity u2, Pressure p
Iter      SolEst     Damping    Stepsize #Res #Jac #Sol   LinErr   LinRes
   1      0.0084   0.5000000      0.0084   14    7    7  2.7e-15    8e-15
Displacement field
Iter      SolEst     Damping    Stepsize #Res #Jac #Sol   LinErr   LinRes
   1       0.054   0.5000000       0.054   14    7    7  4.4e-14  1.5e-12
Spatial mesh displacement
Iter      SolEst     Damping    Stepsize #Res #Jac #Sol   LinErr   LinRes
   1     2.6e-07   0.5000000     2.6e-07   14    7    7  1.6e-15  1.8e-15
Solution error estimates for segregated groups
0.025, 0.0042, 0.027, 1.3e-07
Residual error estimates for segregated groups
4.2, 5.5e+02, 2.6e+03, 0.19
Segregated solver iteration 8.
Merged variables
Iter      SolEst     Damping    Stepsize #Res #Jac #Sol   LinErr   LinRes
   1      0.0093   0.3500000      0.0093   30   22   22  7.2e-14    5e-15
   2       0.006   0.3500000       0.006   31   23   23  8.2e-14  3.2e-15
   3      0.0039   0.3500000      0.0039   32   24   24  1.7e-13  4.2e-15
Velocity u2, Pressure p
Iter      SolEst     Damping    Stepsize #Res #Jac #Sol   LinErr   LinRes
   1      0.0042   0.5000000      0.0042   16    8    8  2.8e-15  7.8e-15
Displacement field
Iter      SolEst     Damping    Stepsize #Res #Jac #Sol   LinErr   LinRes
   1        0.03   0.5000000        0.03   16    8    8  9.6e-14  1.5e-12
Spatial mesh displacement
Iter      SolEst     Damping    Stepsize #Res #Jac #Sol   LinErr   LinRes
   1     1.7e-07   0.5000000     1.7e-07   16    8    8  1.4e-15  1.9e-15
Solution error estimates for segregated groups
0.0067, 0.0021, 0.015, 8.3e-08
Residual error estimates for segregated groups
1.4, 2.8e+02, 1.8e+03, 0.19
Segregated solver iteration 9.
Merged variables
Iter      SolEst     Damping    Stepsize #Res #Jac #Sol   LinErr   LinRes
   1      0.0026   0.3500000      0.0026   34   25   25    1e-13  4.1e-15
   2      0.0017   0.3500000      0.0017   35   26   26  1.8e-13  3.8e-15
   3      0.0011   0.3500000      0.0011   36   27   27  7.2e-14  3.7e-15
Velocity u2, Pressure p
Iter      SolEst     Damping    Stepsize #Res #Jac #Sol   LinErr   LinRes
   1      0.0021   0.5000000      0.0021   18    9    9    3e-15  7.8e-15
Displacement field
Iter      SolEst     Damping    Stepsize #Res #Jac #Sol   LinErr   LinRes
   1       0.017   0.5000000       0.017   18    9    9  1.6e-13  1.5e-12
Spatial mesh displacement
Iter      SolEst     Damping    Stepsize #Res #Jac #Sol   LinErr   LinRes
   1       1e-07   0.5000000       1e-07   18    9    9  1.3e-15  1.8e-15
Solution error estimates for segregated groups
0.0019, 0.001, 0.0085, 5.2e-08
Residual error estimates for segregated groups
```

```
0.45, 1.4e+02, 1.4e+03, 0.18
Segregated solver iteration 10.
Merged variables
Iter      SolEst     Damping    Stepsize #Res #Jac #Sol   LinErr   LinRes
   1      0.0007   0.3500000      0.0007   38   28   28  1.9e-13    4e-15
   2     0.00046   0.3500000     0.00046   39   29   29  5.2e-14  3.6e-15
   3      0.0003   0.3500000      0.0003   40   30   30  7.3e-14  3.7e-15
Velocity u2, Pressure p
Iter      SolEst     Damping    Stepsize #Res #Jac #Sol   LinErr   LinRes
   1       0.001   0.5000000       0.001   20   10   10  2.2e-15  7.8e-15
Displacement field
Iter      SolEst     Damping    Stepsize #Res #Jac #Sol   LinErr   LinRes
   1      0.0094   0.5000000      0.0094   20   10   10  3.2e-14  1.5e-12
Spatial mesh displacement
Iter      SolEst     Damping    Stepsize #Res #Jac #Sol   LinErr   LinRes
   1     6.4e-08   0.5000000     6.4e-08   20   10   10  1.4e-15  1.8e-15
Solution error estimates for segregated groups
0.00051, 0.00052, 0.0047, 3.2e-08
Residual error estimates for segregated groups
0.14, 69, 1.2e+03, 0.2
Segregated solver iteration 11.
Merged variables
Iter      SolEst     Damping    Stepsize #Res #Jac #Sol   LinErr   LinRes
   1     0.00019   0.3500000     0.00019   42   31   31  7.3e-14    5e-15
   2     0.00013   0.3500000     0.00013   43   32   32  1.4e-13  3.3e-15
   3     8.1e-05   0.3500000     8.1e-05   44   33   33  8.2e-14  3.6e-15
Velocity u2, Pressure p
Iter      SolEst     Damping    Stepsize #Res #Jac #Sol   LinErr   LinRes
   1     0.00052   0.5000000     0.00052   22   11   11  2.2e-15  7.8e-15
Displacement field
Iter      SolEst     Damping    Stepsize #Res #Jac #Sol   LinErr   LinRes
   1      0.0051   0.5000000      0.0051   22   11   11  5.6e-14  1.5e-12
Spatial mesh displacement
Iter      SolEst     Damping    Stepsize #Res #Jac #Sol   LinErr   LinRes
   1     3.8e-08   0.5000000     3.8e-08   22   11   11  1.5e-15  1.8e-15
Solution error estimates for segregated groups
0.00014, 0.00026, 0.0026, 1.9e-08
Residual error estimates for segregated groups
0.044, 35, 1.2e+03, 0.19
Segregated solver iteration 12.
Merged variables
Iter      SolEst     Damping    Stepsize #Res #Jac #Sol   LinErr   LinRes
   1     5.3e-05   0.3500000     5.3e-05   46   34   34  5.9e-14  5.5e-15
   2     3.4e-05   0.3500000     3.4e-05   47   35   35  8.4e-14  4.3e-15
   3     2.2e-05   0.3500000     2.2e-05   48   36   36  1.1e-13  3.4e-15
Velocity u2, Pressure p
Iter      SolEst     Damping    Stepsize #Res #Jac #Sol   LinErr   LinRes
   1     0.00026   0.5000000     0.00026   24   12   12  2.5e-15  7.8e-15
Displacement field
Iter      SolEst     Damping    Stepsize #Res #Jac #Sol   LinErr   LinRes
   1      0.0028   0.5000000      0.0028   24   12   12  5.2e-14  1.5e-12
Spatial mesh displacement
Iter      SolEst     Damping    Stepsize #Res #Jac #Sol   LinErr   LinRes
   1     2.3e-08   0.5000000     2.3e-08   24   12   12  1.9e-15  1.8e-15
Solution error estimates for segregated groups
3.8e-05, 0.00013, 0.0014, 1.1e-08
```

```
Residual error estimates for segregated groups
0.013, 17, 1.2e+03, 0.19
Segregated solver iteration 13.
Merged variables
Iter      SolEst      Damping    Stepsize #Res #Jac #Sol   LinErr   LinRes
   1     1.5e-05   0.3500000     1.5e-05   50   37   37  9.9e-14  5.5e-15
   2     9.4e-06   0.3500000     9.4e-06   51   38   38  1.4e-13  3.6e-15
   3     6.1e-06   0.3500000     6.1e-06   52   39   39  6.3e-14  6.2e-15
Velocity u2, Pressure p
Iter      SolEst      Damping    Stepsize #Res #Jac #Sol   LinErr   LinRes
   1     0.00013   0.5000000     0.00013   26   13   13  2.6e-15  7.9e-15
Displacement field
Iter      SolEst      Damping    Stepsize #Res #Jac #Sol   LinErr   LinRes
   1      0.0015   0.5000000      0.0015   26   13   13  6.2e-14  1.5e-12
Spatial mesh displacement
Iter      SolEst      Damping    Stepsize #Res #Jac #Sol   LinErr   LinRes
   1     1.3e-08   0.5000000     1.3e-08   26   13   13    2e-15  1.8e-15
Solution error estimates for segregated groups
1.1e-05, 6.5e-05, 0.00076, 6.6e-09
Residual error estimates for segregated groups
0.0041, 8.7, 1.2e+03, 0.18
Solution time: 158 s. (2 minutes, 38 seconds)
Physical memory: 4.62 GB
Virtual memory: 25.91 GB
Ended at Jan 31, 2025, 5:52:07 PM.
----- Stationary Solver 1 in study1/Approach Curve Solver (sol1) -------------->
```

d=2390 (su24)

GENERAL

Description	Value
Solution	d=2390

LOG

```
<---- Compile Equations: Stationary in study1/Approach Curve Solver (sol1) -----
Started at Jan 31, 2025, 5:52:07 PM.
Parameter d = 2.39E-6 (m).
Geometry shape function: Linear serendipity
Number of vertex elements: 18
Number of boundary elements: 6274
Number of elements: 156850
Minimum element quality: 0.461
Time: 9 s.
Physical memory: 2.83 GB
Virtual memory: 15.18 GB
Ended at Jan 31, 2025, 5:52:16 PM.
----- Compile Equations: Stationary in study1/Approach Curve Solver (sol1) ---->

<---- Dependent Variables 1 in study1/Approach Curve Solver (sol1) -------------
Started at Jan 31, 2025, 5:52:16 PM.
Solution time: 0 s.
Physical memory: 2.83 GB
Virtual memory: 15.18 GB
Ended at Jan 31, 2025, 5:52:16 PM.
----- Dependent Variables 1 in study1/Approach Curve Solver (sol1) ------------>

<---- Stationary Solver 1 in study1/Approach Curve Solver (sol1) ---------------
Started at Jan 31, 2025, 5:52:16 PM.
Segregated solver
Segregated Step 11 does not have any components solved for.
Number of degrees of freedom solved for: 797809 (plus 11971 internal DOFs).
Segregated solver iteration 1.
Merged variables
Nonsymmetric matrix found.
Scales for dependent variables:
Concentration (comp1.Cl): 1.4e+02
Concentration (comp1.K): 1.4e+02
Electric potential (comp1.V): 1.9e+02
Orthonormal null-space function used.
Iter      SolEst     Damping    Stepsize #Res #Jac #Sol   LinErr   LinRes
   1        0.83   0.3500000        0.83    2    1    1    3e-11  2.8e-15
   2        0.81   0.3500000        0.81    3    2    2  2.8e-11  2.7e-15
   3         0.8   0.3500000         0.8    4    3    3  1.4e-11  2.7e-15
Velocity u2, Pressure p
Nonsymmetric matrix found.
Scales for dependent variables:
Pressure (comp1.p): 1
Velocity field (spatial frame) (comp1.u2): 0.00018
Orthonormal null-space function used.
Iter      SolEst     Damping    Stepsize #Res #Jac #Sol   LinErr   LinRes
   1         1.1   0.5000000         1.1    2    1    1  1.6e-15  2.3e-15
Displacement field
Nonsymmetric matrix found.
Scales for dependent variables:
Auxiliary pressure (comp1.solid.lemm1.pw): 1
Displacement field (comp1.u): 2.8e-18
Orthonormal null-space function used.
Iter      SolEst     Damping    Stepsize #Res #Jac #Sol   LinErr   LinRes
   1         1.9   0.5000000         1.9    2    1    1  8.1e-14  1.5e-12
Spatial mesh displacement
```

```
Symmetric matrices found.
Scales for dependent variables:
Spatial mesh displacement (comp1.spatial.disp): 1.2e-09
Orthonormal null-space function used.
Iter      SolEst     Damping    Stepsize #Res #Jac #Sol   LinErr   LinRes
   1     5.9e-07   0.5000000     5.9e-07    2    1    1  1.6e-15  1.8e-15
Solution error estimates for segregated groups
1.4, 0.54, 0.95, 3e-07
Residual error estimates for segregated groups
7.9e+03, 8.3e+04, 2.2e+04, 0.16
Segregated solver iteration 2.
Merged variables
Scales for dependent variables:
Concentration (comp1.Cl): 1.4e+02
Concentration (comp1.K): 1.4e+02
Electric potential (comp1.V): 0.064
Iter      SolEst     Damping    Stepsize #Res #Jac #Sol   LinErr   LinRes
   1        0.79   0.3500000        0.79    6    4    4  5.3e-14  5.8e-15
   2        0.77   0.3500000        0.77    7    5    5  6.9e-14    3e-15
   3        0.76   0.3500000        0.76    8    6    6    6e-14  4.6e-15
Velocity u2, Pressure p
Scales for dependent variables:
Pressure (comp1.p): 3.5e+02
Velocity field (spatial frame) (comp1.u2): 0.00071
Iter      SolEst     Damping    Stepsize #Res #Jac #Sol   LinErr   LinRes
   1        0.36   0.5000000        0.36    4    2    2  2.9e-15  7.8e-15
Displacement field
Iter      SolEst     Damping    Stepsize #Res #Jac #Sol   LinErr   LinRes
   1        0.95   0.5000000        0.95    4    2    2  4.4e-14  1.5e-12
Spatial mesh displacement
Iter      SolEst     Damping    Stepsize #Res #Jac #Sol   LinErr   LinRes
   1     8.9e-07   0.5000000     8.9e-07    4    2    2  1.3e-15  1.8e-15
Solution error estimates for segregated groups
1.3, 0.18, 0.48, 4.5e-07
Residual error estimates for segregated groups
8.1e+02, 1.4e+04, 2.2e+04, 0.18
Segregated solver iteration 3.
Merged variables
Iter      SolEst     Damping    Stepsize #Res #Jac #Sol   LinErr   LinRes
   1        0.75   0.3500000        0.75   10    7    7  4.7e-14  5.3e-15
   2        0.74   0.3500000        0.74   11    8    8  3.7e-14  3.7e-15
   3        0.72   0.3500000        0.72   12    9    9  7.9e-14  4.1e-15
Velocity u2, Pressure p
Iter      SolEst     Damping    Stepsize #Res #Jac #Sol   LinErr   LinRes
   1        0.15   0.5000000        0.15    6    3    3  3.9e-15  7.9e-15
Displacement field
Iter      SolEst     Damping    Stepsize #Res #Jac #Sol   LinErr   LinRes
   1        0.52   0.5000000        0.52    6    3    3  8.7e-14  1.5e-12
Spatial mesh displacement
Iter      SolEst     Damping    Stepsize #Res #Jac #Sol   LinErr   LinRes
   1     8.9e-07   0.5000000     8.9e-07    6    3    3  2.3e-15  1.9e-15
Solution error estimates for segregated groups
1.2, 0.077, 0.26, 4.5e-07
Residual error estimates for segregated groups
2.2e+02, 7.9e+03, 1.6e+04, 0.2
Segregated solver iteration 4.
```

```
Merged variables
Iter      SolEst     Damping    Stepsize #Res #Jac #Sol   LinErr   LinRes
   1         0.7   0.3500000         0.7   14   10   10  7.7e-14  8.9e-15
   2        0.68   0.3500000        0.68   15   11   11  1.1e-13  3.6e-15
   3         0.6   0.3500000         0.6   16   12   12  1.4e-13  3.7e-15
Velocity u2, Pressure p
Iter      SolEst     Damping    Stepsize #Res #Jac #Sol   LinErr   LinRes
   1       0.071   0.5000000       0.071    8    4    4  2.6e-15  7.8e-15
Displacement field
Iter      SolEst     Damping    Stepsize #Res #Jac #Sol   LinErr   LinRes
   1        0.29   0.5000000        0.29    8    4    4  1.1e-13  1.5e-12
Spatial mesh displacement
Iter      SolEst     Damping    Stepsize #Res #Jac #Sol   LinErr   LinRes
   1     7.4e-07   0.5000000     7.4e-07    8    4    4  1.3e-15  1.8e-15
Solution error estimates for segregated groups
1, 0.036, 0.15, 3.7e-07
Residual error estimates for segregated groups
85, 4.2e+03, 1.1e+04, 0.19
Segregated solver iteration 5.
Merged variables
Iter      SolEst     Damping    Stepsize #Res #Jac #Sol   LinErr   LinRes
   1        0.42   0.3500000        0.42   18   13   13    1e-13    7e-15
   2        0.28   0.3500000        0.28   19   14   14  5.6e-14  3.3e-15
   3        0.18   0.3500000        0.18   20   15   15  6.2e-14  8.6e-15
Velocity u2, Pressure p
Iter      SolEst     Damping    Stepsize #Res #Jac #Sol   LinErr   LinRes
   1       0.035   0.5000000       0.035   10    5    5  3.9e-15  7.8e-15
Displacement field
Iter      SolEst     Damping    Stepsize #Res #Jac #Sol   LinErr   LinRes
   1        0.17   0.5000000        0.17   10    5    5  7.3e-14  1.5e-12
Spatial mesh displacement
Iter      SolEst     Damping    Stepsize #Res #Jac #Sol   LinErr   LinRes
   1     5.6e-07   0.5000000     5.6e-07   10    5    5  1.4e-15  1.9e-15
Solution error estimates for segregated groups
0.32, 0.017, 0.084, 2.8e-07
Residual error estimates for segregated groups
33, 2.2e+03, 6.9e+03, 0.2
Segregated solver iteration 6.
Merged variables
Iter      SolEst     Damping    Stepsize #Res #Jac #Sol   LinErr   LinRes
   1        0.12   0.3500000        0.12   22   16   16  7.2e-14  3.2e-15
   2       0.079   0.3500000       0.079   23   17   17    6e-14    3e-15
   3       0.052   0.3500000       0.052   24   18   18  8.2e-14  5.6e-15
Velocity u2, Pressure p
Iter      SolEst     Damping    Stepsize #Res #Jac #Sol   LinErr   LinRes
   1       0.017   0.5000000       0.017   12    6    6  2.8e-15  7.8e-15
Displacement field
Iter      SolEst     Damping    Stepsize #Res #Jac #Sol   LinErr   LinRes
   1       0.095   0.5000000       0.095   12    6    6  5.7e-14  1.5e-12
Spatial mesh displacement
Iter      SolEst     Damping    Stepsize #Res #Jac #Sol   LinErr   LinRes
   1     3.9e-07   0.5000000     3.9e-07   12    6    6  1.4e-15  1.8e-15
Solution error estimates for segregated groups
0.089, 0.0085, 0.048, 2e-07
Residual error estimates for segregated groups
12, 1.1e+03, 4.2e+03, 0.21
```

```
Segregated solver iteration 7.
Merged variables
Iter      SolEst     Damping    Stepsize #Res #Jac #Sol   LinErr   LinRes
   1       0.034   0.3500000       0.034   26   19   19  5.6e-14  3.6e-15
   2       0.022   0.3500000       0.022   27   20   20  7.3e-14  4.2e-15
   3       0.014   0.3500000       0.014   28   21   21  6.1e-14    4e-15
Velocity u2, Pressure p
Iter      SolEst     Damping    Stepsize #Res #Jac #Sol   LinErr   LinRes
   1      0.0084   0.5000000      0.0084   14    7    7  3.6e-15  7.9e-15
Displacement field
Iter      SolEst     Damping    Stepsize #Res #Jac #Sol   LinErr   LinRes
   1       0.054   0.5000000       0.054   14    7    7  9.6e-14  1.5e-12
Spatial mesh displacement
Iter      SolEst     Damping    Stepsize #Res #Jac #Sol   LinErr   LinRes
   1     2.6e-07   0.5000000     2.6e-07   14    7    7  1.2e-15  1.8e-15
Solution error estimates for segregated groups
0.025, 0.0042, 0.027, 1.3e-07
Residual error estimates for segregated groups
4.2, 5.5e+02, 2.6e+03, 0.19
Segregated solver iteration 8.
Merged variables
Iter      SolEst     Damping    Stepsize #Res #Jac #Sol   LinErr   LinRes
   1      0.0093   0.3500000      0.0093   30   22   22  6.7e-14  5.7e-15
   2       0.006   0.3500000       0.006   31   23   23    5e-14  5.9e-15
   3      0.0039   0.3500000      0.0039   32   24   24  2.8e-14  6.6e-15
Velocity u2, Pressure p
Iter      SolEst     Damping    Stepsize #Res #Jac #Sol   LinErr   LinRes
   1      0.0042   0.5000000      0.0042   16    8    8  2.5e-15  7.8e-15
Displacement field
Iter      SolEst     Damping    Stepsize #Res #Jac #Sol   LinErr   LinRes
   1        0.03   0.5000000        0.03   16    8    8  1.1e-13  1.5e-12
Spatial mesh displacement
Iter      SolEst     Damping    Stepsize #Res #Jac #Sol   LinErr   LinRes
   1     1.7e-07   0.5000000     1.7e-07   16    8    8  1.5e-15  1.8e-15
Solution error estimates for segregated groups
0.0068, 0.0021, 0.015, 8.4e-08
Residual error estimates for segregated groups
1.4, 2.8e+02, 1.8e+03, 0.18
Segregated solver iteration 9.
Merged variables
Iter      SolEst     Damping    Stepsize #Res #Jac #Sol   LinErr   LinRes
   1      0.0026   0.3500000      0.0026   34   25   25  7.6e-14  3.5e-15
   2      0.0017   0.3500000      0.0017   35   26   26    6e-14  4.1e-15
   3      0.0011   0.3500000      0.0011   36   27   27  6.2e-14  6.7e-15
Velocity u2, Pressure p
Iter      SolEst     Damping    Stepsize #Res #Jac #Sol   LinErr   LinRes
   1      0.0021   0.5000000      0.0021   18    9    9  1.7e-15    8e-15
Displacement field
Iter      SolEst     Damping    Stepsize #Res #Jac #Sol   LinErr   LinRes
   1       0.017   0.5000000       0.017   18    9    9  8.4e-14  1.5e-12
Spatial mesh displacement
Iter      SolEst     Damping    Stepsize #Res #Jac #Sol   LinErr   LinRes
   1       1e-07   0.5000000       1e-07   18    9    9  1.9e-15  1.8e-15
Solution error estimates for segregated groups
0.0019, 0.001, 0.0085, 5.2e-08
Residual error estimates for segregated groups
```

```
0.45, 1.4e+02, 1.4e+03, 0.19
Segregated solver iteration 10.
Merged variables
Iter      SolEst     Damping    Stepsize #Res #Jac #Sol   LinErr   LinRes
   1      0.0007   0.3500000      0.0007   38   28   28  1.6e-13  3.1e-15
   2     0.00046   0.3500000     0.00046   39   29   29  1.2e-13  3.6e-15
   3      0.0003   0.3500000      0.0003   40   30   30  9.8e-14  5.7e-15
Velocity u2, Pressure p
Iter      SolEst     Damping    Stepsize #Res #Jac #Sol   LinErr   LinRes
   1       0.001   0.5000000       0.001   20   10   10  3.9e-15  7.8e-15
Displacement field
Iter      SolEst     Damping    Stepsize #Res #Jac #Sol   LinErr   LinRes
   1      0.0094   0.5000000      0.0094   20   10   10  2.7e-14  1.5e-12
Spatial mesh displacement
Iter      SolEst     Damping    Stepsize #Res #Jac #Sol   LinErr   LinRes
   1     6.4e-08   0.5000000     6.4e-08   20   10   10  1.6e-15  1.8e-15
Solution error estimates for segregated groups
0.00051, 0.00052, 0.0047, 3.2e-08
Residual error estimates for segregated groups
0.14, 69, 1.2e+03, 0.19
Segregated solver iteration 11.
Merged variables
Iter      SolEst     Damping    Stepsize #Res #Jac #Sol   LinErr   LinRes
   1     0.00019   0.3500000     0.00019   42   31   31  1.2e-13  5.9e-15
   2     0.00013   0.3500000     0.00013   43   32   32  3.7e-14  3.1e-15
   3     8.2e-05   0.3500000     8.2e-05   44   33   33  5.6e-14  4.4e-15
Velocity u2, Pressure p
Iter      SolEst     Damping    Stepsize #Res #Jac #Sol   LinErr   LinRes
   1     0.00052   0.5000000     0.00052   22   11   11  2.4e-15  7.9e-15
Displacement field
Iter      SolEst     Damping    Stepsize #Res #Jac #Sol   LinErr   LinRes
   1      0.0051   0.5000000      0.0051   22   11   11  8.4e-14  1.5e-12
Spatial mesh displacement
Iter      SolEst     Damping    Stepsize #Res #Jac #Sol   LinErr   LinRes
   1     3.8e-08   0.5000000     3.8e-08   22   11   11  1.4e-15  1.8e-15
Solution error estimates for segregated groups
0.00014, 0.00026, 0.0026, 1.9e-08
Residual error estimates for segregated groups
0.044, 35, 1.2e+03, 0.19
Segregated solver iteration 12.
Merged variables
Iter      SolEst     Damping    Stepsize #Res #Jac #Sol   LinErr   LinRes
   1     5.3e-05   0.3500000     5.3e-05   46   34   34  4.8e-14  1.2e-14
   2     3.4e-05   0.3500000     3.4e-05   47   35   35    1e-13  6.4e-15
   3     2.2e-05   0.3500000     2.2e-05   48   36   36  6.4e-14  6.4e-15
Velocity u2, Pressure p
Iter      SolEst     Damping    Stepsize #Res #Jac #Sol   LinErr   LinRes
   1     0.00026   0.5000000     0.00026   24   12   12    3e-15  7.8e-15
Displacement field
Iter      SolEst     Damping    Stepsize #Res #Jac #Sol   LinErr   LinRes
   1      0.0028   0.5000000      0.0028   24   12   12  5.7e-14  1.5e-12
Spatial mesh displacement
Iter      SolEst     Damping    Stepsize #Res #Jac #Sol   LinErr   LinRes
   1     2.3e-08   0.5000000     2.3e-08   24   12   12  1.8e-15  1.8e-15
Solution error estimates for segregated groups
3.8e-05, 0.00013, 0.0014, 1.1e-08
```

```
Residual error estimates for segregated groups
0.013, 17, 1.2e+03, 0.2
Segregated solver iteration 13.
Merged variables
Iter      SolEst     Damping    Stepsize #Res #Jac #Sol   LinErr   LinRes
   1     1.5e-05   0.3500000     1.5e-05   50   37   37    7e-14  3.4e-15
   2     9.5e-06   0.3500000     9.5e-06   51   38   38  1.3e-13  1.2e-14
   3     6.1e-06   0.3500000     6.1e-06   52   39   39  5.4e-14  4.2e-15
Velocity u2, Pressure p
Iter      SolEst     Damping    Stepsize #Res #Jac #Sol   LinErr   LinRes
   1     0.00013   0.5000000     0.00013   26   13   13  2.6e-15    8e-15
Displacement field
Iter      SolEst     Damping    Stepsize #Res #Jac #Sol   LinErr   LinRes
   1      0.0015   0.5000000      0.0015   26   13   13  5.9e-14  1.5e-12
Spatial mesh displacement
Iter      SolEst     Damping    Stepsize #Res #Jac #Sol   LinErr   LinRes
   1     1.3e-08   0.5000000     1.3e-08   26   13   13  1.9e-15  1.8e-15
Solution error estimates for segregated groups
1.1e-05, 6.5e-05, 0.00076, 6.6e-09
Residual error estimates for segregated groups
0.0041, 8.6, 1.2e+03, 0.17
Solution time: 158 s. (2 minutes, 38 seconds)
Physical memory: 4.59 GB
Virtual memory: 25.87 GB
Ended at Jan 31, 2025, 5:54:54 PM.
----- Stationary Solver 1 in study1/Approach Curve Solver (sol1) -------------->
```

d=2385 (su25)

GENERAL

Description	Value
Solution	d=2385

LOG

```
<---- Compile Equations: Stationary in study1/Approach Curve Solver (sol1) -----
Started at Jan 31, 2025, 5:54:54 PM.
Parameter d = 2.385E-6 (m).
Geometry shape function: Linear serendipity
Number of vertex elements: 18
Number of boundary elements: 6272
Number of elements: 156802
Minimum element quality: 0.4636
Time: 9 s.
Physical memory: 2.84 GB
Virtual memory: 15.2 GB
Ended at Jan 31, 2025, 5:55:03 PM.
----- Compile Equations: Stationary in study1/Approach Curve Solver (sol1) ---->

<---- Dependent Variables 1 in study1/Approach Curve Solver (sol1) -------------
Started at Jan 31, 2025, 5:55:03 PM.
Solution time: 0 s.
Physical memory: 2.84 GB
Virtual memory: 15.2 GB
Ended at Jan 31, 2025, 5:55:03 PM.
----- Dependent Variables 1 in study1/Approach Curve Solver (sol1) ------------>

<---- Stationary Solver 1 in study1/Approach Curve Solver (sol1) ---------------
Started at Jan 31, 2025, 5:55:03 PM.
Segregated solver
Segregated Step 11 does not have any components solved for.
Number of degrees of freedom solved for: 797588 (plus 11967 internal DOFs).
Segregated solver iteration 1.
Merged variables
Nonsymmetric matrix found.
Scales for dependent variables:
Concentration (comp1.Cl): 1.4e+02
Concentration (comp1.K): 1.4e+02
Electric potential (comp1.V): 1.9e+02
Orthonormal null-space function used.
Iter      SolEst     Damping    Stepsize #Res #Jac #Sol   LinErr   LinRes
   1        0.83   0.3500000        0.83    2    1    1  2.1e-11  2.8e-15
   2        0.81   0.3500000        0.81    3    2    2    7e-11  2.8e-15
   3         0.8   0.3500000         0.8    4    3    3    3e-11  2.7e-15
Velocity u2, Pressure p
Nonsymmetric matrix found.
Scales for dependent variables:
Pressure (comp1.p): 1
Velocity field (spatial frame) (comp1.u2): 0.00018
Orthonormal null-space function used.
Iter      SolEst     Damping    Stepsize #Res #Jac #Sol   LinErr   LinRes
   1         1.1   0.5000000         1.1    2    1    1  1.7e-15  2.3e-15
Displacement field
Nonsymmetric matrix found.
Scales for dependent variables:
Auxiliary pressure (comp1.solid.lemm1.pw): 1
Displacement field (comp1.u): 2.8e-18
Orthonormal null-space function used.
Iter      SolEst     Damping    Stepsize #Res #Jac #Sol   LinErr   LinRes
   1         1.9   0.5000000         1.9    2    1    1  1.1e-13  1.5e-12
Spatial mesh displacement
```

```
Symmetric matrices found.
Scales for dependent variables:
Spatial mesh displacement (comp1.spatial.disp): 1.2e-09
Orthonormal null-space function used.
Iter      SolEst     Damping    Stepsize #Res #Jac #Sol   LinErr   LinRes
   1       6e-07   0.5000000       6e-07    2    1    1  1.3e-15  1.9e-15
Solution error estimates for segregated groups
1.4, 0.54, 0.95, 3e-07
Residual error estimates for segregated groups
7.9e+03, 8.3e+04, 2.2e+04, 0.17
Segregated solver iteration 2.
Merged variables
Scales for dependent variables:
Concentration (comp1.Cl): 1.4e+02
Concentration (comp1.K): 1.4e+02
Electric potential (comp1.V): 0.064
Iter      SolEst     Damping    Stepsize #Res #Jac #Sol   LinErr   LinRes
   1        0.79   0.3500000        0.79    6    4    4  1.1e-13  2.6e-15
   2        0.77   0.3500000        0.77    7    5    5  3.8e-14  2.7e-15
   3        0.76   0.3500000        0.76    8    6    6  4.3e-14  3.4e-15
Velocity u2, Pressure p
Scales for dependent variables:
Pressure (comp1.p): 3.5e+02
Velocity field (spatial frame) (comp1.u2): 0.00071
Iter      SolEst     Damping    Stepsize #Res #Jac #Sol   LinErr   LinRes
   1        0.36   0.5000000        0.36    4    2    2  1.8e-15  7.9e-15
Displacement field
Iter      SolEst     Damping    Stepsize #Res #Jac #Sol   LinErr   LinRes
   1        0.95   0.5000000        0.95    4    2    2  5.7e-14  1.5e-12
Spatial mesh displacement
Iter      SolEst     Damping    Stepsize #Res #Jac #Sol   LinErr   LinRes
   1       9e-07   0.5000000       9e-07    4    2    2  1.6e-15  1.9e-15
Solution error estimates for segregated groups
1.3, 0.18, 0.48, 4.5e-07
Residual error estimates for segregated groups
8.1e+02, 1.3e+04, 2.2e+04, 0.18
Segregated solver iteration 3.
Merged variables
Iter      SolEst     Damping    Stepsize #Res #Jac #Sol   LinErr   LinRes
   1        0.75   0.3500000        0.75   10    7    7  3.1e-14  2.7e-15
   2        0.74   0.3500000        0.74   11    8    8    6e-14  2.8e-15
   3        0.72   0.3500000        0.72   12    9    9  6.6e-14  2.7e-15
Velocity u2, Pressure p
Iter      SolEst     Damping    Stepsize #Res #Jac #Sol   LinErr   LinRes
   1        0.15   0.5000000        0.15    6    3    3  4.2e-15  7.9e-15
Displacement field
Iter      SolEst     Damping    Stepsize #Res #Jac #Sol   LinErr   LinRes
   1        0.52   0.5000000        0.52    6    3    3  9.7e-14  1.5e-12
Spatial mesh displacement
Iter      SolEst     Damping    Stepsize #Res #Jac #Sol   LinErr   LinRes
   1       9e-07   0.5000000       9e-07    6    3    3  1.2e-15  1.9e-15
Solution error estimates for segregated groups
1.2, 0.077, 0.26, 4.5e-07
Residual error estimates for segregated groups
2.2e+02, 7.2e+03, 1.6e+04, 0.19
Segregated solver iteration 4.
```

```
Merged variables
Iter      SolEst     Damping    Stepsize #Res #Jac #Sol   LinErr   LinRes
   1         0.7   0.3500000         0.7   14   10   10  6.2e-14  2.7e-15
   2        0.68   0.3500000        0.68   15   11   11  6.4e-14  2.7e-15
   3         0.6   0.3500000         0.6   16   12   12  5.9e-14  2.6e-15
Velocity u2, Pressure p
Iter      SolEst     Damping    Stepsize #Res #Jac #Sol   LinErr   LinRes
   1       0.071   0.5000000       0.071    8    4    4  2.9e-15  7.8e-15
Displacement field
Iter      SolEst     Damping    Stepsize #Res #Jac #Sol   LinErr   LinRes
   1        0.29   0.5000000        0.29    8    4    4  1.4e-13  1.5e-12
Spatial mesh displacement
Iter      SolEst     Damping    Stepsize #Res #Jac #Sol   LinErr   LinRes
   1     7.5e-07   0.5000000     7.5e-07    8    4    4  1.6e-15  1.9e-15
Solution error estimates for segregated groups
1, 0.036, 0.15, 3.8e-07
Residual error estimates for segregated groups
85, 3.8e+03, 1.1e+04, 0.21
Segregated solver iteration 5.
Merged variables
Iter      SolEst     Damping    Stepsize #Res #Jac #Sol   LinErr   LinRes
   1        0.42   0.3500000        0.42   18   13   13    4e-14  2.7e-15
   2        0.28   0.3500000        0.28   19   14   14  9.2e-14  2.7e-15
   3        0.18   0.3500000        0.18   20   15   15  6.2e-14  2.6e-15
Velocity u2, Pressure p
Iter      SolEst     Damping    Stepsize #Res #Jac #Sol   LinErr   LinRes
   1       0.035   0.5000000       0.035   10    5    5  2.9e-15  7.8e-15
Displacement field
Iter      SolEst     Damping    Stepsize #Res #Jac #Sol   LinErr   LinRes
   1        0.17   0.5000000        0.17   10    5    5  4.1e-14  1.5e-12
Spatial mesh displacement
Iter      SolEst     Damping    Stepsize #Res #Jac #Sol   LinErr   LinRes
   1     5.6e-07   0.5000000     5.6e-07   10    5    5  1.3e-15  1.9e-15
Solution error estimates for segregated groups
0.32, 0.017, 0.084, 2.8e-07
Residual error estimates for segregated groups
33, 1.9e+03, 6.9e+03, 0.19
Segregated solver iteration 6.
Merged variables
Iter      SolEst     Damping    Stepsize #Res #Jac #Sol   LinErr   LinRes
   1        0.12   0.3500000        0.12   22   16   16  5.7e-14  3.2e-15
   2       0.079   0.3500000       0.079   23   17   17  1.6e-13  2.8e-15
   3       0.052   0.3500000       0.052   24   18   18  9.4e-14  2.8e-15
Velocity u2, Pressure p
Iter      SolEst     Damping    Stepsize #Res #Jac #Sol   LinErr   LinRes
   1       0.017   0.5000000       0.017   12    6    6  2.6e-15  7.7e-15
Displacement field
Iter      SolEst     Damping    Stepsize #Res #Jac #Sol   LinErr   LinRes
   1       0.095   0.5000000       0.095   12    6    6    1e-13  1.5e-12
Spatial mesh displacement
Iter      SolEst     Damping    Stepsize #Res #Jac #Sol   LinErr   LinRes
   1     3.9e-07   0.5000000     3.9e-07   12    6    6  1.7e-15  1.8e-15
Solution error estimates for segregated groups
0.089, 0.0085, 0.048, 2e-07
Residual error estimates for segregated groups
12, 9.8e+02, 4.2e+03, 0.2
```

```
Segregated solver iteration 7.
Merged variables
Iter      SolEst     Damping    Stepsize #Res #Jac #Sol   LinErr   LinRes
   1       0.034   0.3500000       0.034   26   19   19    6e-14  2.8e-15
   2       0.022   0.3500000       0.022   27   20   20  6.7e-14  2.9e-15
   3       0.014   0.3500000       0.014   28   21   21    1e-13    3e-15
Velocity u2, Pressure p
Iter      SolEst     Damping    Stepsize #Res #Jac #Sol   LinErr   LinRes
   1      0.0084   0.5000000      0.0084   14    7    7  3.6e-15    8e-15
Displacement field
Iter      SolEst     Damping    Stepsize #Res #Jac #Sol   LinErr   LinRes
   1       0.054   0.5000000       0.054   14    7    7  6.8e-14  1.5e-12
Spatial mesh displacement
Iter      SolEst     Damping    Stepsize #Res #Jac #Sol   LinErr   LinRes
   1     2.6e-07   0.5000000     2.6e-07   14    7    7  1.7e-15  1.9e-15
Solution error estimates for segregated groups
0.025, 0.0042, 0.027, 1.3e-07
Residual error estimates for segregated groups
4.2, 4.9e+02, 2.7e+03, 0.18
Segregated solver iteration 8.
Merged variables
Iter      SolEst     Damping    Stepsize #Res #Jac #Sol   LinErr   LinRes
   1      0.0093   0.3500000      0.0093   30   22   22  1.1e-13  2.7e-15
   2       0.006   0.3500000       0.006   31   23   23  4.2e-14  3.3e-15
   3      0.0039   0.3500000      0.0039   32   24   24  9.6e-14  2.9e-15
Velocity u2, Pressure p
Iter      SolEst     Damping    Stepsize #Res #Jac #Sol   LinErr   LinRes
   1      0.0042   0.5000000      0.0042   16    8    8  2.7e-15  7.9e-15
Displacement field
Iter      SolEst     Damping    Stepsize #Res #Jac #Sol   LinErr   LinRes
   1        0.03   0.5000000        0.03   16    8    8  6.6e-14  1.5e-12
Spatial mesh displacement
Iter      SolEst     Damping    Stepsize #Res #Jac #Sol   LinErr   LinRes
   1     1.7e-07   0.5000000     1.7e-07   16    8    8  1.6e-15  1.9e-15
Solution error estimates for segregated groups
0.0068, 0.0021, 0.015, 8.4e-08
Residual error estimates for segregated groups
1.4, 2.5e+02, 1.8e+03, 0.17
Segregated solver iteration 9.
Merged variables
Iter      SolEst     Damping    Stepsize #Res #Jac #Sol   LinErr   LinRes
   1      0.0026   0.3500000      0.0026   34   25   25  5.8e-14    3e-15
   2      0.0017   0.3500000      0.0017   35   26   26  1.1e-13  2.7e-15
   3      0.0011   0.3500000      0.0011   36   27   27  4.8e-14  2.8e-15
Velocity u2, Pressure p
Iter      SolEst     Damping    Stepsize #Res #Jac #Sol   LinErr   LinRes
   1      0.0021   0.5000000      0.0021   18    9    9  2.4e-15  7.9e-15
Displacement field
Iter      SolEst     Damping    Stepsize #Res #Jac #Sol   LinErr   LinRes
   1       0.017   0.5000000       0.017   18    9    9  1.3e-13  1.5e-12
Spatial mesh displacement
Iter      SolEst     Damping    Stepsize #Res #Jac #Sol   LinErr   LinRes
   1     1.1e-07   0.5000000     1.1e-07   18    9    9  1.6e-15  1.9e-15
Solution error estimates for segregated groups
0.0019, 0.001, 0.0085, 5.3e-08
Residual error estimates for segregated groups
```

```
0.45, 1.2e+02, 1.4e+03, 0.18
Segregated solver iteration 10.
Merged variables
Iter      SolEst     Damping    Stepsize #Res #Jac #Sol   LinErr   LinRes
   1      0.0007   0.3500000      0.0007   38   28   28  7.5e-14  2.9e-15
   2     0.00046   0.3500000     0.00046   39   29   29    9e-14  2.7e-15
   3      0.0003   0.3500000      0.0003   40   30   30    7e-14  3.3e-15
Velocity u2, Pressure p
Iter      SolEst     Damping    Stepsize #Res #Jac #Sol   LinErr   LinRes
   1       0.001   0.5000000       0.001   20   10   10  3.5e-15  7.9e-15
Displacement field
Iter      SolEst     Damping    Stepsize #Res #Jac #Sol   LinErr   LinRes
   1      0.0094   0.5000000      0.0094   20   10   10  8.4e-14  1.5e-12
Spatial mesh displacement
Iter      SolEst     Damping    Stepsize #Res #Jac #Sol   LinErr   LinRes
   1     6.4e-08   0.5000000     6.4e-08   20   10   10  1.4e-15  1.9e-15
Solution error estimates for segregated groups
0.00051, 0.00052, 0.0047, 3.2e-08
Residual error estimates for segregated groups
0.14, 62, 1.2e+03, 0.19
Segregated solver iteration 11.
Merged variables
Iter      SolEst     Damping    Stepsize #Res #Jac #Sol   LinErr   LinRes
   1     0.00019   0.3500000     0.00019   42   31   31  5.3e-14  2.7e-15
   2     0.00013   0.3500000     0.00013   43   32   32  4.6e-14  2.8e-15
   3     8.2e-05   0.3500000     8.2e-05   44   33   33  5.4e-14  2.9e-15
Velocity u2, Pressure p
Iter      SolEst     Damping    Stepsize #Res #Jac #Sol   LinErr   LinRes
   1     0.00052   0.5000000     0.00052   22   11   11  2.4e-15  7.6e-15
Displacement field
Iter      SolEst     Damping    Stepsize #Res #Jac #Sol   LinErr   LinRes
   1      0.0051   0.5000000      0.0051   22   11   11  4.1e-14  1.5e-12
Spatial mesh displacement
Iter      SolEst     Damping    Stepsize #Res #Jac #Sol   LinErr   LinRes
   1     3.9e-08   0.5000000     3.9e-08   22   11   11  1.8e-15  1.9e-15
Solution error estimates for segregated groups
0.00014, 0.00026, 0.0026, 1.9e-08
Residual error estimates for segregated groups
0.044, 31, 1.2e+03, 0.21
Segregated solver iteration 12.
Merged variables
Iter      SolEst     Damping    Stepsize #Res #Jac #Sol   LinErr   LinRes
   1     5.3e-05   0.3500000     5.3e-05   46   34   34  6.5e-14  2.8e-15
   2     3.4e-05   0.3500000     3.4e-05   47   35   35  6.9e-14  2.7e-15
   3     2.2e-05   0.3500000     2.2e-05   48   36   36  6.6e-14  2.7e-15
Velocity u2, Pressure p
Iter      SolEst     Damping    Stepsize #Res #Jac #Sol   LinErr   LinRes
   1     0.00026   0.5000000     0.00026   24   12   12  3.2e-15  7.8e-15
Displacement field
Iter      SolEst     Damping    Stepsize #Res #Jac #Sol   LinErr   LinRes
   1      0.0028   0.5000000      0.0028   24   12   12  2.8e-14  1.5e-12
Spatial mesh displacement
Iter      SolEst     Damping    Stepsize #Res #Jac #Sol   LinErr   LinRes
   1     2.3e-08   0.5000000     2.3e-08   24   12   12  1.5e-15  1.9e-15
Solution error estimates for segregated groups
3.8e-05, 0.00013, 0.0014, 1.1e-08
```

```
Residual error estimates for segregated groups
0.013, 16, 1.2e+03, 0.18
Segregated solver iteration 13.
Merged variables
Iter      SolEst     Damping    Stepsize #Res #Jac #Sol   LinErr   LinRes
   1     1.5e-05   0.3500000     1.5e-05   50   37   37  4.3e-14  2.8e-15
   2     9.5e-06   0.3500000     9.5e-06   51   38   38  7.3e-14  3.1e-15
   3     6.1e-06   0.3500000     6.1e-06   52   39   39  6.9e-14  3.2e-15
Velocity u2, Pressure p
Iter      SolEst     Damping    Stepsize #Res #Jac #Sol   LinErr   LinRes
   1     0.00013   0.5000000     0.00013   26   13   13  3.3e-15  7.9e-15
Displacement field
Iter      SolEst     Damping    Stepsize #Res #Jac #Sol   LinErr   LinRes
   1      0.0015   0.5000000      0.0015   26   13   13  1.3e-13  1.5e-12
Spatial mesh displacement
Iter      SolEst     Damping    Stepsize #Res #Jac #Sol   LinErr   LinRes
   1     1.3e-08   0.5000000     1.3e-08   26   13   13  2.9e-15  1.8e-15
Solution error estimates for segregated groups
1.1e-05, 6.5e-05, 0.00076, 6.7e-09
Residual error estimates for segregated groups
0.0041, 7.8, 1.2e+03, 0.18
Solution time: 156 s. (2 minutes, 36 seconds)
Physical memory: 4.51 GB
Virtual memory: 25.83 GB
Ended at Jan 31, 2025, 5:57:39 PM.
----- Stationary Solver 1 in study1/Approach Curve Solver (sol1) -------------->
```

d=2380 (su26)

GENERAL

Description	Value
Solution	d=2380

LOG

```
<---- Compile Equations: Stationary in study1/Approach Curve Solver (sol1) -----
Started at Jan 31, 2025, 5:57:39 PM.
Parameter d = 2.38E-6 (m).
Geometry shape function: Linear serendipity
Number of vertex elements: 18
Number of boundary elements: 6269
Number of elements: 156713
Minimum element quality: 0.4661
Time: 9 s.
Physical memory: 2.83 GB
Virtual memory: 15.18 GB
Ended at Jan 31, 2025, 5:57:48 PM.
----- Compile Equations: Stationary in study1/Approach Curve Solver (sol1) ---->

<---- Dependent Variables 1 in study1/Approach Curve Solver (sol1) -------------
Started at Jan 31, 2025, 5:57:48 PM.
Solution time: 0 s.
Physical memory: 2.83 GB
Virtual memory: 15.18 GB
Ended at Jan 31, 2025, 5:57:48 PM.
----- Dependent Variables 1 in study1/Approach Curve Solver (sol1) ------------>

<---- Stationary Solver 1 in study1/Approach Curve Solver (sol1) ---------------
Started at Jan 31, 2025, 5:57:48 PM.
Segregated solver
Segregated Step 11 does not have any components solved for.
Number of degrees of freedom solved for: 797133 (plus 11961 internal DOFs).
Segregated solver iteration 1.
Merged variables
Nonsymmetric matrix found.
Scales for dependent variables:
Concentration (comp1.Cl): 1.4e+02
Concentration (comp1.K): 1.4e+02
Electric potential (comp1.V): 1.9e+02
Orthonormal null-space function used.
Iter      SolEst     Damping    Stepsize #Res #Jac #Sol   LinErr   LinRes
   1        0.83   0.3500000        0.83    2    1    1  3.2e-11  2.8e-15
   2        0.81   0.3500000        0.81    3    2    2  4.1e-11  2.7e-15
   3         0.8   0.3500000         0.8    4    3    3  3.1e-11  2.7e-15
Velocity u2, Pressure p
Nonsymmetric matrix found.
Scales for dependent variables:
Pressure (comp1.p): 1
Velocity field (spatial frame) (comp1.u2): 0.00019
Orthonormal null-space function used.
Iter      SolEst     Damping    Stepsize #Res #Jac #Sol   LinErr   LinRes
   1         1.1   0.5000000         1.1    2    1    1  1.7e-15  2.3e-15
Displacement field
Nonsymmetric matrix found.
Scales for dependent variables:
Auxiliary pressure (comp1.solid.lemm1.pw): 1
Displacement field (comp1.u): 2.8e-18
Orthonormal null-space function used.
Iter      SolEst     Damping    Stepsize #Res #Jac #Sol   LinErr   LinRes
   1         1.9   0.5000000         1.9    2    1    1  3.2e-14  1.5e-12
Spatial mesh displacement
```

```
Symmetric matrices found.
Scales for dependent variables:
Spatial mesh displacement (comp1.spatial.disp): 1.2e-09
Orthonormal null-space function used.
Iter      SolEst     Damping    Stepsize #Res #Jac #Sol   LinErr   LinRes
   1       6e-07   0.5000000       6e-07    2    1    1  1.6e-15  1.9e-15
Solution error estimates for segregated groups
1.4, 0.54, 0.95, 3e-07
Residual error estimates for segregated groups
7.9e+03, 8.3e+04, 2.2e+04, 0.16
Segregated solver iteration 2.
Merged variables
Scales for dependent variables:
Concentration (comp1.Cl): 1.4e+02
Concentration (comp1.K): 1.4e+02
Electric potential (comp1.V): 0.064
Iter      SolEst     Damping    Stepsize #Res #Jac #Sol   LinErr   LinRes
   1        0.79   0.3500000        0.79    6    4    4  3.8e-13  2.2e-14
   2        0.77   0.3500000        0.77    7    5    5  2.5e-13  1.3e-14
   3        0.76   0.3500000        0.76    8    6    6  3.6e-13  7.9e-14
Velocity u2, Pressure p
Scales for dependent variables:
Pressure (comp1.p): 3.5e+02
Velocity field (spatial frame) (comp1.u2): 0.00071
Iter      SolEst     Damping    Stepsize #Res #Jac #Sol   LinErr   LinRes
   1        0.36   0.5000000        0.36    4    2    2    3e-15  7.9e-15
Displacement field
Iter      SolEst     Damping    Stepsize #Res #Jac #Sol   LinErr   LinRes
   1        0.95   0.5000000        0.95    4    2    2  1.2e-13  1.5e-12
Spatial mesh displacement
Iter      SolEst     Damping    Stepsize #Res #Jac #Sol   LinErr   LinRes
   1       9e-07   0.5000000       9e-07    4    2    2  1.7e-15  1.8e-15
Solution error estimates for segregated groups
1.3, 0.18, 0.48, 4.5e-07
Residual error estimates for segregated groups
8.1e+02, 1.4e+04, 2.2e+04, 0.19
Segregated solver iteration 3.
Merged variables
Iter      SolEst     Damping    Stepsize #Res #Jac #Sol   LinErr   LinRes
   1        0.75   0.3500000        0.75   10    7    7  1.6e-12  8.5e-14
   2        0.74   0.3500000        0.74   11    8    8  4.5e-13  5.9e-14
   3        0.72   0.3500000        0.72   12    9    9  1.1e-12  5.5e-14
Velocity u2, Pressure p
Iter      SolEst     Damping    Stepsize #Res #Jac #Sol   LinErr   LinRes
   1        0.15   0.5000000        0.15    6    3    3  2.7e-15  7.9e-15
Displacement field
Iter      SolEst     Damping    Stepsize #Res #Jac #Sol   LinErr   LinRes
   1        0.52   0.5000000        0.52    6    3    3  4.9e-14  1.5e-12
Spatial mesh displacement
Iter      SolEst     Damping    Stepsize #Res #Jac #Sol   LinErr   LinRes
   1       9e-07   0.5000000       9e-07    6    3    3  1.5e-15  1.8e-15
Solution error estimates for segregated groups
1.2, 0.077, 0.26, 4.5e-07
Residual error estimates for segregated groups
2.2e+02, 7.6e+03, 1.6e+04, 0.18
Segregated solver iteration 4.
```

```
Merged variables
Iter      SolEst     Damping    Stepsize #Res #Jac #Sol   LinErr   LinRes
   1         0.7   0.3500000         0.7   14   10   10  4.2e-13  2.1e-14
   2        0.68   0.3500000        0.68   15   11   11  1.5e-12  6.9e-14
   3         0.6   0.3500000         0.6   16   12   12    8e-13  3.6e-14
Velocity u2, Pressure p
Iter      SolEst     Damping    Stepsize #Res #Jac #Sol   LinErr   LinRes
   1       0.071   0.5000000       0.071    8    4    4  2.4e-15  7.8e-15
Displacement field
Iter      SolEst     Damping    Stepsize #Res #Jac #Sol   LinErr   LinRes
   1        0.29   0.5000000        0.29    8    4    4  6.3e-14  1.6e-12
Spatial mesh displacement
Iter      SolEst     Damping    Stepsize #Res #Jac #Sol   LinErr   LinRes
   1     7.5e-07   0.5000000     7.5e-07    8    4    4  1.4e-15  1.8e-15
Solution error estimates for segregated groups
1, 0.036, 0.15, 3.8e-07
Residual error estimates for segregated groups
85, 4e+03, 1.1e+04, 0.18
Segregated solver iteration 5.
Merged variables
Iter      SolEst     Damping    Stepsize #Res #Jac #Sol   LinErr   LinRes
   1        0.42   0.3500000        0.42   18   13   13  3.5e-13  3.2e-14
   2        0.28   0.3500000        0.28   19   14   14  3.9e-13  5.2e-14
   3        0.18   0.3500000        0.18   20   15   15  9.3e-13  6.8e-14
Velocity u2, Pressure p
Iter      SolEst     Damping    Stepsize #Res #Jac #Sol   LinErr   LinRes
   1       0.035   0.5000000       0.035   10    5    5  4.7e-15  7.9e-15
Displacement field
Iter      SolEst     Damping    Stepsize #Res #Jac #Sol   LinErr   LinRes
   1        0.17   0.5000000        0.17   10    5    5  1.1e-13  1.5e-12
Spatial mesh displacement
Iter      SolEst     Damping    Stepsize #Res #Jac #Sol   LinErr   LinRes
   1     5.6e-07   0.5000000     5.6e-07   10    5    5  1.4e-15  1.8e-15
Solution error estimates for segregated groups
0.32, 0.017, 0.084, 2.8e-07
Residual error estimates for segregated groups
33, 2.1e+03, 6.9e+03, 0.18
Segregated solver iteration 6.
Merged variables
Iter      SolEst     Damping    Stepsize #Res #Jac #Sol   LinErr   LinRes
   1        0.12   0.3500000        0.12   22   16   16  2.7e-13    2e-14
   2       0.079   0.3500000       0.079   23   17   17  4.6e-13  5.3e-14
   3       0.052   0.3500000       0.052   24   18   18  1.7e-13  5.6e-14
Velocity u2, Pressure p
Iter      SolEst     Damping    Stepsize #Res #Jac #Sol   LinErr   LinRes
   1       0.017   0.5000000       0.017   12    6    6  2.9e-15    8e-15
Displacement field
Iter      SolEst     Damping    Stepsize #Res #Jac #Sol   LinErr   LinRes
   1       0.095   0.5000000       0.095   12    6    6  5.3e-14  1.5e-12
Spatial mesh displacement
Iter      SolEst     Damping    Stepsize #Res #Jac #Sol   LinErr   LinRes
   1     3.9e-07   0.5000000     3.9e-07   12    6    6  1.7e-15  1.9e-15
Solution error estimates for segregated groups
0.089, 0.0085, 0.048, 2e-07
Residual error estimates for segregated groups
12, 1e+03, 4.2e+03, 0.19
```

```
Segregated solver iteration 7.
Merged variables
Iter      SolEst     Damping    Stepsize #Res #Jac #Sol   LinErr   LinRes
   1       0.034   0.3500000       0.034   26   19   19  8.8e-13  4.6e-14
   2       0.022   0.3500000       0.022   27   20   20  1.9e-12  8.9e-14
   3       0.014   0.3500000       0.014   28   21   21  5.4e-13    1e-13
Velocity u2, Pressure p
Iter      SolEst     Damping    Stepsize #Res #Jac #Sol   LinErr   LinRes
   1      0.0084   0.5000000      0.0084   14    7    7  3.3e-15  7.8e-15
Displacement field
Iter      SolEst     Damping    Stepsize #Res #Jac #Sol   LinErr   LinRes
   1       0.054   0.5000000       0.054   14    7    7  5.9e-14  1.5e-12
Spatial mesh displacement
Iter      SolEst     Damping    Stepsize #Res #Jac #Sol   LinErr   LinRes
   1     2.6e-07   0.5000000     2.6e-07   14    7    7  1.4e-15  1.8e-15
Solution error estimates for segregated groups
0.025, 0.0042, 0.027, 1.3e-07
Residual error estimates for segregated groups
4.2, 5.3e+02, 2.6e+03, 0.19
Segregated solver iteration 8.
Merged variables
Iter      SolEst     Damping    Stepsize #Res #Jac #Sol   LinErr   LinRes
   1      0.0093   0.3500000      0.0093   30   22   22  4.6e-13  2.7e-14
   2       0.006   0.3500000       0.006   31   23   23  4.2e-13  7.2e-14
   3      0.0039   0.3500000      0.0039   32   24   24  1.2e-12  6.4e-14
Velocity u2, Pressure p
Iter      SolEst     Damping    Stepsize #Res #Jac #Sol   LinErr   LinRes
   1      0.0042   0.5000000      0.0042   16    8    8  4.1e-15  7.8e-15
Displacement field
Iter      SolEst     Damping    Stepsize #Res #Jac #Sol   LinErr   LinRes
   1        0.03   0.5000000        0.03   16    8    8  6.2e-14  1.5e-12
Spatial mesh displacement
Iter      SolEst     Damping    Stepsize #Res #Jac #Sol   LinErr   LinRes
   1     1.7e-07   0.5000000     1.7e-07   16    8    8  1.7e-15  1.9e-15
Solution error estimates for segregated groups
0.0067, 0.0021, 0.015, 8.5e-08
Residual error estimates for segregated groups
1.4, 2.6e+02, 1.8e+03, 0.18
Segregated solver iteration 9.
Merged variables
Iter      SolEst     Damping    Stepsize #Res #Jac #Sol   LinErr   LinRes
   1      0.0026   0.3500000      0.0026   34   25   25  7.2e-13  2.6e-14
   2      0.0017   0.3500000      0.0017   35   26   26  9.5e-13  4.5e-14
   3      0.0011   0.3500000      0.0011   36   27   27  5.9e-13  3.8e-14
Velocity u2, Pressure p
Iter      SolEst     Damping    Stepsize #Res #Jac #Sol   LinErr   LinRes
   1      0.0021   0.5000000      0.0021   18    9    9  3.4e-15    8e-15
Displacement field
Iter      SolEst     Damping    Stepsize #Res #Jac #Sol   LinErr   LinRes
   1       0.017   0.5000000       0.017   18    9    9  7.4e-14  1.5e-12
Spatial mesh displacement
Iter      SolEst     Damping    Stepsize #Res #Jac #Sol   LinErr   LinRes
   1     1.1e-07   0.5000000     1.1e-07   18    9    9  1.7e-15  1.8e-15
Solution error estimates for segregated groups
0.0019, 0.001, 0.0085, 5.3e-08
Residual error estimates for segregated groups
```

```
0.45, 1.3e+02, 1.4e+03, 0.18
Segregated solver iteration 10.
Merged variables
Iter      SolEst     Damping    Stepsize #Res #Jac #Sol   LinErr   LinRes
   1      0.0007   0.3500000      0.0007   38   28   28  6.2e-13  4.2e-14
   2     0.00046   0.3500000     0.00046   39   29   29  2.8e-13  2.1e-14
   3      0.0003   0.3500000      0.0003   40   30   30  2.9e-13  3.3e-14
Velocity u2, Pressure p
Iter      SolEst     Damping    Stepsize #Res #Jac #Sol   LinErr   LinRes
   1       0.001   0.5000000       0.001   20   10   10  2.8e-15  7.7e-15
Displacement field
Iter      SolEst     Damping    Stepsize #Res #Jac #Sol   LinErr   LinRes
   1      0.0094   0.5000000      0.0094   20   10   10  1.1e-13  1.5e-12
Spatial mesh displacement
Iter      SolEst     Damping    Stepsize #Res #Jac #Sol   LinErr   LinRes
   1     6.5e-08   0.5000000     6.5e-08   20   10   10  1.9e-15  1.9e-15
Solution error estimates for segregated groups
0.00051, 0.00052, 0.0047, 3.2e-08
Residual error estimates for segregated groups
0.14, 66, 1.2e+03, 0.19
Segregated solver iteration 11.
Merged variables
Iter      SolEst     Damping    Stepsize #Res #Jac #Sol   LinErr   LinRes
   1     0.00019   0.3500000     0.00019   42   31   31  5.5e-13  5.4e-14
   2     0.00013   0.3500000     0.00013   43   32   32  5.1e-13  3.4e-14
   3     8.1e-05   0.3500000     8.1e-05   44   33   33  6.3e-13  3.3e-14
Velocity u2, Pressure p
Iter      SolEst     Damping    Stepsize #Res #Jac #Sol   LinErr   LinRes
   1     0.00052   0.5000000     0.00052   22   11   11    2e-15  7.8e-15
Displacement field
Iter      SolEst     Damping    Stepsize #Res #Jac #Sol   LinErr   LinRes
   1      0.0051   0.5000000      0.0051   22   11   11  2.8e-14  1.5e-12
Spatial mesh displacement
Iter      SolEst     Damping    Stepsize #Res #Jac #Sol   LinErr   LinRes
   1     3.9e-08   0.5000000     3.9e-08   22   11   11  1.6e-15  1.9e-15
Solution error estimates for segregated groups
0.00014, 0.00026, 0.0026, 1.9e-08
Residual error estimates for segregated groups
0.044, 33, 1.2e+03, 0.2
Segregated solver iteration 12.
Merged variables
Iter      SolEst     Damping    Stepsize #Res #Jac #Sol   LinErr   LinRes
   1     5.3e-05   0.3500000     5.3e-05   46   34   34  1.4e-12  8.5e-14
   2     3.4e-05   0.3500000     3.4e-05   47   35   35  3.8e-13  1.5e-14
   3     2.2e-05   0.3500000     2.2e-05   48   36   36  1.9e-13  2.5e-14
Velocity u2, Pressure p
Iter      SolEst     Damping    Stepsize #Res #Jac #Sol   LinErr   LinRes
   1     0.00026   0.5000000     0.00026   24   12   12  3.7e-15  7.9e-15
Displacement field
Iter      SolEst     Damping    Stepsize #Res #Jac #Sol   LinErr   LinRes
   1      0.0028   0.5000000      0.0028   24   12   12  9.9e-14  1.5e-12
Spatial mesh displacement
Iter      SolEst     Damping    Stepsize #Res #Jac #Sol   LinErr   LinRes
   1     2.3e-08   0.5000000     2.3e-08   24   12   12  1.3e-15  1.8e-15
Solution error estimates for segregated groups
3.8e-05, 0.00013, 0.0014, 1.1e-08
```

```
Residual error estimates for segregated groups
0.013, 17, 1.2e+03, 0.18
Segregated solver iteration 13.
Merged variables
Iter      SolEst     Damping    Stepsize #Res #Jac #Sol   LinErr   LinRes
   1     1.5e-05   0.3500000     1.5e-05   50   37   37  1.1e-12  6.9e-14
   2     9.4e-06   0.3500000     9.4e-06   51   38   38  1.4e-12  7.9e-14
   3     6.1e-06   0.3500000     6.1e-06   52   39   39  3.9e-13  7.6e-14
Velocity u2, Pressure p
Iter      SolEst     Damping    Stepsize #Res #Jac #Sol   LinErr   LinRes
   1     0.00013   0.5000000     0.00013   26   13   13  2.6e-15    8e-15
Displacement field
Iter      SolEst     Damping    Stepsize #Res #Jac #Sol   LinErr   LinRes
   1      0.0015   0.5000000      0.0015   26   13   13  4.1e-14  1.5e-12
Spatial mesh displacement
Iter      SolEst     Damping    Stepsize #Res #Jac #Sol   LinErr   LinRes
   1     1.3e-08   0.5000000     1.3e-08   26   13   13  1.5e-15  1.9e-15
Solution error estimates for segregated groups
1.1e-05, 6.5e-05, 0.00076, 6.7e-09
Residual error estimates for segregated groups
0.0041, 8.3, 1.2e+03, 0.18
Solution time: 157 s. (2 minutes, 37 seconds)
Physical memory: 4.67 GB
Virtual memory: 25.89 GB
Ended at Jan 31, 2025, 6:00:25 PM.
----- Stationary Solver 1 in study1/Approach Curve Solver (sol1) -------------->
```

d=2375 (su27)

GENERAL

Description	Value
Solution	d=2375

LOG

```
<---- Compile Equations: Stationary in study1/Approach Curve Solver (sol1) -----
Started at Jan 31, 2025, 6:00:25 PM.
Parameter d = 2.375E-6 (m).
Geometry shape function: Linear serendipity
Number of vertex elements: 18
Number of boundary elements: 6267
Number of elements: 156977
Minimum element quality: 0.4811
Time: 9 s.
Physical memory: 2.83 GB
Virtual memory: 15.18 GB
Ended at Jan 31, 2025, 6:00:34 PM.
----- Compile Equations: Stationary in study1/Approach Curve Solver (sol1) ---->

<---- Dependent Variables 1 in study1/Approach Curve Solver (sol1) -------------
Started at Jan 31, 2025, 6:00:34 PM.
Solution time: 0 s.
Physical memory: 2.83 GB
Virtual memory: 15.18 GB
Ended at Jan 31, 2025, 6:00:34 PM.
----- Dependent Variables 1 in study1/Approach Curve Solver (sol1) ------------>

<---- Stationary Solver 1 in study1/Approach Curve Solver (sol1) ---------------
Started at Jan 31, 2025, 6:00:34 PM.
Segregated solver
Segregated Step 11 does not have any components solved for.
Number of degrees of freedom solved for: 798368 (plus 11957 internal DOFs).
Segregated solver iteration 1.
Merged variables
Nonsymmetric matrix found.
Scales for dependent variables:
Concentration (comp1.Cl): 1.4e+02
Concentration (comp1.K): 1.4e+02
Electric potential (comp1.V): 1.9e+02
Orthonormal null-space function used.
Iter      SolEst     Damping    Stepsize #Res #Jac #Sol   LinErr   LinRes
   1        0.83   0.3500000        0.83    2    1    1  4.7e-11  2.8e-15
   2        0.81   0.3500000        0.81    3    2    2  2.3e-11  2.8e-15
   3         0.8   0.3500000         0.8    4    3    3  4.1e-11  2.7e-15
Velocity u2, Pressure p
Nonsymmetric matrix found.
Scales for dependent variables:
Pressure (comp1.p): 1
Velocity field (spatial frame) (comp1.u2): 0.00018
Orthonormal null-space function used.
Iter      SolEst     Damping    Stepsize #Res #Jac #Sol   LinErr   LinRes
   1         1.1   0.5000000         1.1    2    1    1  1.7e-15  2.3e-15
Displacement field
Nonsymmetric matrix found.
Scales for dependent variables:
Auxiliary pressure (comp1.solid.lemm1.pw): 1
Displacement field (comp1.u): 2.8e-18
Orthonormal null-space function used.
Iter      SolEst     Damping    Stepsize #Res #Jac #Sol   LinErr   LinRes
   1         1.9   0.5000000         1.9    2    1    1  3.6e-14  1.5e-12
Spatial mesh displacement
```

```
Symmetric matrices found.
Scales for dependent variables:
Spatial mesh displacement (comp1.spatial.disp): 1.2e-09
Orthonormal null-space function used.
Iter      SolEst     Damping    Stepsize #Res #Jac #Sol   LinErr   LinRes
   1       6e-07   0.5000000       6e-07    2    1    1  1.4e-15  1.9e-15
Solution error estimates for segregated groups
1.4, 0.54, 0.95, 3e-07
Residual error estimates for segregated groups
7.9e+03, 8.3e+04, 2.2e+04, 0.16
Segregated solver iteration 2.
Merged variables
Scales for dependent variables:
Concentration (comp1.Cl): 1.4e+02
Concentration (comp1.K): 1.4e+02
Electric potential (comp1.V): 0.064
Iter      SolEst     Damping    Stepsize #Res #Jac #Sol   LinErr   LinRes
   1        0.79   0.3500000        0.79    6    4    4  1.3e-13    3e-15
   2        0.77   0.3500000        0.77    7    5    5  5.3e-14  2.9e-15
   3        0.76   0.3500000        0.76    8    6    6  1.1e-13  3.8e-15
Velocity u2, Pressure p
Scales for dependent variables:
Pressure (comp1.p): 3.5e+02
Velocity field (spatial frame) (comp1.u2): 0.00071
Iter      SolEst     Damping    Stepsize #Res #Jac #Sol   LinErr   LinRes
   1        0.36   0.5000000        0.36    4    2    2  2.9e-15  7.9e-15
Displacement field
Iter      SolEst     Damping    Stepsize #Res #Jac #Sol   LinErr   LinRes
   1        0.95   0.5000000        0.95    4    2    2  1.1e-13  1.5e-12
Spatial mesh displacement
Iter      SolEst     Damping    Stepsize #Res #Jac #Sol   LinErr   LinRes
   1       9e-07   0.5000000       9e-07    4    2    2  1.7e-15  1.8e-15
Solution error estimates for segregated groups
1.3, 0.18, 0.48, 4.5e-07
Residual error estimates for segregated groups
8.1e+02, 1.4e+04, 2.2e+04, 0.17
Segregated solver iteration 3.
Merged variables
Iter      SolEst     Damping    Stepsize #Res #Jac #Sol   LinErr   LinRes
   1        0.75   0.3500000        0.75   10    7    7  9.2e-14  3.6e-15
   2        0.74   0.3500000        0.74   11    8    8  1.5e-13  2.9e-15
   3        0.72   0.3500000        0.72   12    9    9  5.4e-14  3.3e-15
Velocity u2, Pressure p
Iter      SolEst     Damping    Stepsize #Res #Jac #Sol   LinErr   LinRes
   1        0.15   0.5000000        0.15    6    3    3  2.2e-15    8e-15
Displacement field
Iter      SolEst     Damping    Stepsize #Res #Jac #Sol   LinErr   LinRes
   1        0.52   0.5000000        0.52    6    3    3  1.6e-13  1.5e-12
Spatial mesh displacement
Iter      SolEst     Damping    Stepsize #Res #Jac #Sol   LinErr   LinRes
   1       9e-07   0.5000000       9e-07    6    3    3  1.8e-15  1.9e-15
Solution error estimates for segregated groups
1.2, 0.076, 0.26, 4.5e-07
Residual error estimates for segregated groups
2.2e+02, 7.8e+03, 1.6e+04, 0.18
Segregated solver iteration 4.
```

```
Merged variables
Iter      SolEst     Damping    Stepsize #Res #Jac #Sol   LinErr   LinRes
   1         0.7   0.3500000         0.7   14   10   10  6.4e-14  3.1e-15
   2        0.68   0.3500000        0.68   15   11   11  6.5e-14  2.8e-15
   3         0.6   0.3500000         0.6   16   12   12  1.1e-13  2.9e-15
Velocity u2, Pressure p
Iter      SolEst     Damping    Stepsize #Res #Jac #Sol   LinErr   LinRes
   1       0.071   0.5000000       0.071    8    4    4  3.9e-15  7.9e-15
Displacement field
Iter      SolEst     Damping    Stepsize #Res #Jac #Sol   LinErr   LinRes
   1        0.29   0.5000000        0.29    8    4    4  5.1e-14  1.5e-12
Spatial mesh displacement
Iter      SolEst     Damping    Stepsize #Res #Jac #Sol   LinErr   LinRes
   1     7.5e-07   0.5000000     7.5e-07    8    4    4  2.2e-15  1.8e-15
Solution error estimates for segregated groups
1, 0.036, 0.15, 3.8e-07
Residual error estimates for segregated groups
85, 4.1e+03, 1.1e+04, 0.17
Segregated solver iteration 5.
Merged variables
Iter      SolEst     Damping    Stepsize #Res #Jac #Sol   LinErr   LinRes
   1        0.42   0.3500000        0.42   18   13   13  7.5e-14  4.1e-15
   2        0.28   0.3500000        0.28   19   14   14    1e-13  3.3e-15
   3        0.18   0.3500000        0.18   20   15   15  8.5e-14  2.9e-15
Velocity u2, Pressure p
Iter      SolEst     Damping    Stepsize #Res #Jac #Sol   LinErr   LinRes
   1       0.035   0.5000000       0.035   10    5    5  2.8e-15  7.8e-15
Displacement field
Iter      SolEst     Damping    Stepsize #Res #Jac #Sol   LinErr   LinRes
   1        0.17   0.5000000        0.17   10    5    5  7.9e-14  1.5e-12
Spatial mesh displacement
Iter      SolEst     Damping    Stepsize #Res #Jac #Sol   LinErr   LinRes
   1     5.6e-07   0.5000000     5.6e-07   10    5    5  2.2e-15  1.9e-15
Solution error estimates for segregated groups
0.32, 0.017, 0.084, 2.8e-07
Residual error estimates for segregated groups
33, 2.1e+03, 6.9e+03, 0.18
Segregated solver iteration 6.
Merged variables
Iter      SolEst     Damping    Stepsize #Res #Jac #Sol   LinErr   LinRes
   1        0.12   0.3500000        0.12   22   16   16  1.2e-13  3.4e-15
   2       0.079   0.3500000       0.079   23   17   17  5.7e-14  3.3e-15
   3       0.052   0.3500000       0.052   24   18   18  1.4e-13  3.1e-15
Velocity u2, Pressure p
Iter      SolEst     Damping    Stepsize #Res #Jac #Sol   LinErr   LinRes
   1       0.017   0.5000000       0.017   12    6    6    3e-15  7.9e-15
Displacement field
Iter      SolEst     Damping    Stepsize #Res #Jac #Sol   LinErr   LinRes
   1       0.095   0.5000000       0.095   12    6    6  3.7e-14  1.5e-12
Spatial mesh displacement
Iter      SolEst     Damping    Stepsize #Res #Jac #Sol   LinErr   LinRes
   1       4e-07   0.5000000       4e-07   12    6    6  1.9e-15  1.8e-15
Solution error estimates for segregated groups
0.089, 0.0085, 0.048, 2e-07
Residual error estimates for segregated groups
12, 1.1e+03, 4.2e+03, 0.2
```

```
Segregated solver iteration 7.
Merged variables
Iter      SolEst     Damping    Stepsize #Res #Jac #Sol   LinErr   LinRes
   1       0.034   0.3500000       0.034   26   19   19    1e-13  2.8e-15
   2       0.022   0.3500000       0.022   27   20   20  6.2e-14  4.5e-15
   3       0.014   0.3500000       0.014   28   21   21  7.8e-14  2.9e-15
Velocity u2, Pressure p
Iter      SolEst     Damping    Stepsize #Res #Jac #Sol   LinErr   LinRes
   1      0.0084   0.5000000      0.0084   14    7    7  3.6e-15  7.7e-15
Displacement field
Iter      SolEst     Damping    Stepsize #Res #Jac #Sol   LinErr   LinRes
   1       0.054   0.5000000       0.054   14    7    7  1.6e-13  1.5e-12
Spatial mesh displacement
Iter      SolEst     Damping    Stepsize #Res #Jac #Sol   LinErr   LinRes
   1     2.6e-07   0.5000000     2.6e-07   14    7    7  1.8e-15  1.8e-15
Solution error estimates for segregated groups
0.025, 0.0042, 0.027, 1.3e-07
Residual error estimates for segregated groups
4.2, 5.4e+02, 2.6e+03, 0.18
Segregated solver iteration 8.
Merged variables
Iter      SolEst     Damping    Stepsize #Res #Jac #Sol   LinErr   LinRes
   1      0.0093   0.3500000      0.0093   30   22   22  5.5e-14  3.7e-15
   2      0.0061   0.3500000      0.0061   31   23   23  6.8e-14  3.4e-15
   3      0.0039   0.3500000      0.0039   32   24   24  4.6e-14  4.3e-15
Velocity u2, Pressure p
Iter      SolEst     Damping    Stepsize #Res #Jac #Sol   LinErr   LinRes
   1      0.0042   0.5000000      0.0042   16    8    8    3e-15  7.7e-15
Displacement field
Iter      SolEst     Damping    Stepsize #Res #Jac #Sol   LinErr   LinRes
   1        0.03   0.5000000        0.03   16    8    8  7.8e-14  1.5e-12
Spatial mesh displacement
Iter      SolEst     Damping    Stepsize #Res #Jac #Sol   LinErr   LinRes
   1     1.7e-07   0.5000000     1.7e-07   16    8    8  2.1e-15  1.9e-15
Solution error estimates for segregated groups
0.0068, 0.0021, 0.015, 8.5e-08
Residual error estimates for segregated groups
1.4, 2.7e+02, 1.8e+03, 0.19
Segregated solver iteration 9.
Merged variables
Iter      SolEst     Damping    Stepsize #Res #Jac #Sol   LinErr   LinRes
   1      0.0026   0.3500000      0.0026   34   25   25    8e-14  2.8e-15
   2      0.0017   0.3500000      0.0017   35   26   26  4.4e-14  4.1e-15
   3      0.0011   0.3500000      0.0011   36   27   27  7.3e-14  3.4e-15
Velocity u2, Pressure p
Iter      SolEst     Damping    Stepsize #Res #Jac #Sol   LinErr   LinRes
   1      0.0021   0.5000000      0.0021   18    9    9  3.1e-15  7.8e-15
Displacement field
Iter      SolEst     Damping    Stepsize #Res #Jac #Sol   LinErr   LinRes
   1       0.017   0.5000000       0.017   18    9    9  3.4e-14  1.5e-12
Spatial mesh displacement
Iter      SolEst     Damping    Stepsize #Res #Jac #Sol   LinErr   LinRes
   1     1.1e-07   0.5000000     1.1e-07   18    9    9  1.9e-15  1.9e-15
Solution error estimates for segregated groups
0.0019, 0.001, 0.0085, 5.3e-08
Residual error estimates for segregated groups
```

```
0.45, 1.4e+02, 1.4e+03, 0.19
Segregated solver iteration 10.
Merged variables
Iter      SolEst     Damping    Stepsize #Res #Jac #Sol   LinErr   LinRes
   1      0.0007   0.3500000      0.0007   38   28   28    8e-14    3e-15
   2     0.00046   0.3500000     0.00046   39   29   29  4.5e-14  3.1e-15
   3      0.0003   0.3500000      0.0003   40   30   30    7e-14  3.3e-15
Velocity u2, Pressure p
Iter      SolEst     Damping    Stepsize #Res #Jac #Sol   LinErr   LinRes
   1       0.001   0.5000000       0.001   20   10   10  3.2e-15  7.8e-15
Displacement field
Iter      SolEst     Damping    Stepsize #Res #Jac #Sol   LinErr   LinRes
   1      0.0094   0.5000000      0.0094   20   10   10  1.4e-13  1.5e-12
Spatial mesh displacement
Iter      SolEst     Damping    Stepsize #Res #Jac #Sol   LinErr   LinRes
   1     6.5e-08   0.5000000     6.5e-08   20   10   10    2e-15  1.9e-15
Solution error estimates for segregated groups
0.00051, 0.00052, 0.0047, 3.2e-08
Residual error estimates for segregated groups
0.14, 68, 1.3e+03, 0.18
Segregated solver iteration 11.
Merged variables
Iter      SolEst     Damping    Stepsize #Res #Jac #Sol   LinErr   LinRes
   1     0.00019   0.3500000     0.00019   42   31   31  1.1e-13    3e-15
   2     0.00013   0.3500000     0.00013   43   32   32  6.5e-14  2.9e-15
   3     8.2e-05   0.3500000     8.2e-05   44   33   33    6e-14  3.2e-15
Velocity u2, Pressure p
Iter      SolEst     Damping    Stepsize #Res #Jac #Sol   LinErr   LinRes
   1     0.00052   0.5000000     0.00052   22   11   11  2.4e-15  7.9e-15
Displacement field
Iter      SolEst     Damping    Stepsize #Res #Jac #Sol   LinErr   LinRes
   1      0.0051   0.5000000      0.0051   22   11   11  3.9e-14  1.5e-12
Spatial mesh displacement
Iter      SolEst     Damping    Stepsize #Res #Jac #Sol   LinErr   LinRes
   1     3.9e-08   0.5000000     3.9e-08   22   11   11  1.6e-15  1.9e-15
Solution error estimates for segregated groups
0.00014, 0.00026, 0.0026, 1.9e-08
Residual error estimates for segregated groups
0.044, 34, 1.2e+03, 0.18
Segregated solver iteration 12.
Merged variables
Iter      SolEst     Damping    Stepsize #Res #Jac #Sol   LinErr   LinRes
   1     5.3e-05   0.3500000     5.3e-05   46   34   34  5.4e-14  2.9e-15
   2     3.4e-05   0.3500000     3.4e-05   47   35   35  5.2e-14    3e-15
   3     2.2e-05   0.3500000     2.2e-05   48   36   36  1.2e-13  2.9e-15
Velocity u2, Pressure p
Iter      SolEst     Damping    Stepsize #Res #Jac #Sol   LinErr   LinRes
   1     0.00026   0.5000000     0.00026   24   12   12  4.2e-15  7.9e-15
Displacement field
Iter      SolEst     Damping    Stepsize #Res #Jac #Sol   LinErr   LinRes
   1      0.0028   0.5000000      0.0028   24   12   12  3.9e-14  1.5e-12
Spatial mesh displacement
Iter      SolEst     Damping    Stepsize #Res #Jac #Sol   LinErr   LinRes
   1     2.3e-08   0.5000000     2.3e-08   24   12   12  1.5e-15  1.8e-15
Solution error estimates for segregated groups
3.8e-05, 0.00013, 0.0014, 1.1e-08
```

```
Residual error estimates for segregated groups
0.013, 17, 1.2e+03, 0.19
Segregated solver iteration 13.
Merged variables
Iter      SolEst     Damping    Stepsize #Res #Jac #Sol   LinErr   LinRes
   1      1.5e-05   0.3500000     1.5e-05   50   37   37  5.7e-14  3.3e-15
   2      9.5e-06   0.3500000     9.5e-06   51   38   38  5.2e-14  2.8e-15
   3      6.2e-06   0.3500000     6.2e-06   52   39   39  7.7e-14    3e-15
Velocity u2, Pressure p
Iter      SolEst     Damping    Stepsize #Res #Jac #Sol   LinErr   LinRes
   1      0.00013   0.5000000     0.00013   26   13   13  2.2e-15  7.8e-15
Displacement field
Iter      SolEst     Damping    Stepsize #Res #Jac #Sol   LinErr   LinRes
   1       0.0015   0.5000000      0.0015   26   13   13  2.9e-14  1.5e-12
Spatial mesh displacement
Iter      SolEst     Damping    Stepsize #Res #Jac #Sol   LinErr   LinRes
   1      1.3e-08   0.5000000     1.3e-08   26   13   13  1.4e-15  1.8e-15
Solution error estimates for segregated groups
1.1e-05, 6.5e-05, 0.00076, 6.7e-09
Residual error estimates for segregated groups
0.0041, 8.4, 1.2e+03, 0.19
Solution time: 158 s. (2 minutes, 38 seconds)
Physical memory: 4.58 GB
Virtual memory: 25.56 GB
Ended at Jan 31, 2025, 6:03:11 PM.
----- Stationary Solver 1 in study1/Approach Curve Solver (sol1) -------------->
```

d=2370 (su28)

GENERAL

Description	Value
Solution	d=2370

LOG

```
<---- Compile Equations: Stationary in study1/Approach Curve Solver (sol1) -----
Started at Jan 31, 2025, 6:03:12 PM.
Parameter d = 2.37E-6 (m).
Geometry shape function: Linear serendipity
Number of vertex elements: 18
Number of boundary elements: 6264
Number of elements: 156956
Minimum element quality: 0.4707
Time: 9 s.
Physical memory: 2.87 GB
Virtual memory: 15.22 GB
Ended at Jan 31, 2025, 6:03:20 PM.
----- Compile Equations: Stationary in study1/Approach Curve Solver (sol1) ---->

<---- Dependent Variables 1 in study1/Approach Curve Solver (sol1) -------------
Started at Jan 31, 2025, 6:03:20 PM.
Solution time: 0 s.
Physical memory: 2.87 GB
Virtual memory: 15.22 GB
Ended at Jan 31, 2025, 6:03:20 PM.
----- Dependent Variables 1 in study1/Approach Curve Solver (sol1) ------------>

<---- Stationary Solver 1 in study1/Approach Curve Solver (sol1) ---------------
Started at Jan 31, 2025, 6:03:21 PM.
Segregated solver
Segregated Step 11 does not have any components solved for.
Number of degrees of freedom solved for: 798365 (plus 11951 internal DOFs).
Segregated solver iteration 1.
Merged variables
Nonsymmetric matrix found.
Scales for dependent variables:
Concentration (comp1.Cl): 1.4e+02
Concentration (comp1.K): 1.4e+02
Electric potential (comp1.V): 1.9e+02
Orthonormal null-space function used.
Iter      SolEst     Damping    Stepsize #Res #Jac #Sol   LinErr   LinRes
   1        0.83   0.3500000        0.83    2    1    1  3.1e-11  2.8e-15
   2        0.81   0.3500000        0.81    3    2    2  3.7e-11  2.7e-15
   3         0.8   0.3500000         0.8    4    3    3  4.5e-11  2.7e-15
Velocity u2, Pressure p
Nonsymmetric matrix found.
Scales for dependent variables:
Pressure (comp1.p): 1
Velocity field (spatial frame) (comp1.u2): 0.00018
Orthonormal null-space function used.
Iter      SolEst     Damping    Stepsize #Res #Jac #Sol   LinErr   LinRes
   1         1.1   0.5000000         1.1    2    1    1  1.6e-15  2.3e-15
Displacement field
Nonsymmetric matrix found.
Scales for dependent variables:
Auxiliary pressure (comp1.solid.lemm1.pw): 1
Displacement field (comp1.u): 2.8e-18
Orthonormal null-space function used.
Iter      SolEst     Damping    Stepsize #Res #Jac #Sol   LinErr   LinRes
   1         1.9   0.5000000         1.9    2    1    1    1e-13  1.5e-12
Spatial mesh displacement
```

```
Symmetric matrices found.
Scales for dependent variables:
Spatial mesh displacement (comp1.spatial.disp): 1.2e-09
Orthonormal null-space function used.
Iter      SolEst     Damping    Stepsize #Res #Jac #Sol   LinErr   LinRes
   1       6e-07   0.5000000       6e-07    2    1    1  1.6e-15  1.9e-15
Solution error estimates for segregated groups
1.4, 0.54, 0.95, 3e-07
Residual error estimates for segregated groups
7.9e+03, 8.3e+04, 2.2e+04, 0.17
Segregated solver iteration 2.
Merged variables
Scales for dependent variables:
Concentration (comp1.Cl): 1.4e+02
Concentration (comp1.K): 1.4e+02
Electric potential (comp1.V): 0.064
Iter      SolEst     Damping    Stepsize #Res #Jac #Sol   LinErr   LinRes
   1        0.79   0.3500000        0.79    6    4    4    6e-14  2.9e-15
   2        0.77   0.3500000        0.77    7    5    5    1e-13  3.4e-15
   3        0.76   0.3500000        0.76    8    6    6  5.2e-14  3.3e-15
Velocity u2, Pressure p
Scales for dependent variables:
Pressure (comp1.p): 3.5e+02
Velocity field (spatial frame) (comp1.u2): 0.00071
Iter      SolEst     Damping    Stepsize #Res #Jac #Sol   LinErr   LinRes
   1        0.36   0.5000000        0.36    4    2    2  3.6e-15  7.8e-15
Displacement field
Iter      SolEst     Damping    Stepsize #Res #Jac #Sol   LinErr   LinRes
   1        0.95   0.5000000        0.95    4    2    2    8e-14  1.5e-12
Spatial mesh displacement
Iter      SolEst     Damping    Stepsize #Res #Jac #Sol   LinErr   LinRes
   1     9.1e-07   0.5000000     9.1e-07    4    2    2  1.5e-15  1.8e-15
Solution error estimates for segregated groups
1.3, 0.18, 0.48, 4.5e-07
Residual error estimates for segregated groups
8.1e+02, 1.3e+04, 2.2e+04, 0.19
Segregated solver iteration 3.
Merged variables
Iter      SolEst     Damping    Stepsize #Res #Jac #Sol   LinErr   LinRes
   1        0.75   0.3500000        0.75   10    7    7  7.8e-14  3.5e-15
   2        0.74   0.3500000        0.74   11    8    8    1e-13  3.2e-15
   3        0.72   0.3500000        0.72   12    9    9  1.2e-13  3.2e-15
Velocity u2, Pressure p
Iter      SolEst     Damping    Stepsize #Res #Jac #Sol   LinErr   LinRes
   1        0.15   0.5000000        0.15    6    3    3  4.3e-15  7.8e-15
Displacement field
Iter      SolEst     Damping    Stepsize #Res #Jac #Sol   LinErr   LinRes
   1        0.52   0.5000000        0.52    6    3    3  9.2e-14  1.5e-12
Spatial mesh displacement
Iter      SolEst     Damping    Stepsize #Res #Jac #Sol   LinErr   LinRes
   1     9.1e-07   0.5000000     9.1e-07    6    3    3  1.5e-15  1.9e-15
Solution error estimates for segregated groups
1.2, 0.076, 0.26, 4.5e-07
Residual error estimates for segregated groups
2.2e+02, 7.5e+03, 1.6e+04, 0.18
Segregated solver iteration 4.
```

```
Merged variables
Iter      SolEst     Damping    Stepsize #Res #Jac #Sol   LinErr   LinRes
   1         0.7   0.3500000         0.7   14   10   10  1.6e-13  2.9e-15
   2        0.68   0.3500000        0.68   15   11   11  1.1e-13  3.3e-15
   3         0.6   0.3500000         0.6   16   12   12    7e-14    3e-15
Velocity u2, Pressure p
Iter      SolEst     Damping    Stepsize #Res #Jac #Sol   LinErr   LinRes
   1       0.071   0.5000000       0.071    8    4    4  3.2e-15  7.9e-15
Displacement field
Iter      SolEst     Damping    Stepsize #Res #Jac #Sol   LinErr   LinRes
   1        0.29   0.5000000        0.29    8    4    4  6.1e-14  1.5e-12
Spatial mesh displacement
Iter      SolEst     Damping    Stepsize #Res #Jac #Sol   LinErr   LinRes
   1     7.6e-07   0.5000000     7.6e-07    8    4    4  1.4e-15  1.8e-15
Solution error estimates for segregated groups
1, 0.036, 0.15, 3.8e-07
Residual error estimates for segregated groups
85, 4e+03, 1.1e+04, 0.19
Segregated solver iteration 5.
Merged variables
Iter      SolEst     Damping    Stepsize #Res #Jac #Sol   LinErr   LinRes
   1        0.42   0.3500000        0.42   18   13   13  6.2e-14  2.9e-15
   2        0.28   0.3500000        0.28   19   14   14  1.1e-13  3.1e-15
   3        0.18   0.3500000        0.18   20   15   15    1e-13  3.3e-15
Velocity u2, Pressure p
Iter      SolEst     Damping    Stepsize #Res #Jac #Sol   LinErr   LinRes
   1       0.035   0.5000000       0.035   10    5    5  3.5e-15  7.8e-15
Displacement field
Iter      SolEst     Damping    Stepsize #Res #Jac #Sol   LinErr   LinRes
   1        0.17   0.5000000        0.17   10    5    5  6.2e-14  1.5e-12
Spatial mesh displacement
Iter      SolEst     Damping    Stepsize #Res #Jac #Sol   LinErr   LinRes
   1     5.7e-07   0.5000000     5.7e-07   10    5    5  1.3e-15  1.9e-15
Solution error estimates for segregated groups
0.32, 0.017, 0.084, 2.8e-07
Residual error estimates for segregated groups
33, 2e+03, 6.9e+03, 0.18
Segregated solver iteration 6.
Merged variables
Iter      SolEst     Damping    Stepsize #Res #Jac #Sol   LinErr   LinRes
   1        0.12   0.3500000        0.12   22   16   16  9.2e-14  3.1e-15
   2       0.079   0.3500000       0.079   23   17   17  6.2e-14    3e-15
   3       0.052   0.3500000       0.052   24   18   18  4.6e-14  3.2e-15
Velocity u2, Pressure p
Iter      SolEst     Damping    Stepsize #Res #Jac #Sol   LinErr   LinRes
   1       0.017   0.5000000       0.017   12    6    6  2.7e-15  7.9e-15
Displacement field
Iter      SolEst     Damping    Stepsize #Res #Jac #Sol   LinErr   LinRes
   1       0.095   0.5000000       0.095   12    6    6  6.4e-14  1.5e-12
Spatial mesh displacement
Iter      SolEst     Damping    Stepsize #Res #Jac #Sol   LinErr   LinRes
   1       4e-07   0.5000000       4e-07   12    6    6  1.6e-15  1.8e-15
Solution error estimates for segregated groups
0.089, 0.0085, 0.048, 2e-07
Residual error estimates for segregated groups
12, 1e+03, 4.2e+03, 0.19
```

```
Segregated solver iteration 7.
Merged variables
Iter      SolEst     Damping    Stepsize #Res #Jac #Sol   LinErr   LinRes
   1       0.034   0.3500000       0.034   26   19   19  1.2e-13  3.8e-15
   2       0.022   0.3500000       0.022   27   20   20  1.1e-13  3.1e-15
   3       0.014   0.3500000       0.014   28   21   21  6.5e-14  2.9e-15
Velocity u2, Pressure p
Iter      SolEst     Damping    Stepsize #Res #Jac #Sol   LinErr   LinRes
   1      0.0084   0.5000000      0.0084   14    7    7    5e-15  7.9e-15
Displacement field
Iter      SolEst     Damping    Stepsize #Res #Jac #Sol   LinErr   LinRes
   1       0.054   0.5000000       0.054   14    7    7  3.4e-14  1.5e-12
Spatial mesh displacement
Iter      SolEst     Damping    Stepsize #Res #Jac #Sol   LinErr   LinRes
   1     2.6e-07   0.5000000     2.6e-07   14    7    7  1.4e-15  1.8e-15
Solution error estimates for segregated groups
0.025, 0.0042, 0.027, 1.3e-07
Residual error estimates for segregated groups
4.2, 5.2e+02, 2.6e+03, 0.18
Segregated solver iteration 8.
Merged variables
Iter      SolEst     Damping    Stepsize #Res #Jac #Sol   LinErr   LinRes
   1      0.0093   0.3500000      0.0093   30   22   22  4.6e-14  2.8e-15
   2      0.0061   0.3500000      0.0061   31   23   23  9.2e-14  2.9e-15
   3      0.0039   0.3500000      0.0039   32   24   24  8.6e-14  3.1e-15
Velocity u2, Pressure p
Iter      SolEst     Damping    Stepsize #Res #Jac #Sol   LinErr   LinRes
   1      0.0042   0.5000000      0.0042   16    8    8    3e-15  7.8e-15
Displacement field
Iter      SolEst     Damping    Stepsize #Res #Jac #Sol   LinErr   LinRes
   1        0.03   0.5000000        0.03   16    8    8  4.7e-14  1.5e-12
Spatial mesh displacement
Iter      SolEst     Damping    Stepsize #Res #Jac #Sol   LinErr   LinRes
   1     1.7e-07   0.5000000     1.7e-07   16    8    8  1.4e-15  1.9e-15
Solution error estimates for segregated groups
0.0068, 0.0021, 0.015, 8.5e-08
Residual error estimates for segregated groups
1.4, 2.6e+02, 1.8e+03, 0.17
Segregated solver iteration 9.
Merged variables
Iter      SolEst     Damping    Stepsize #Res #Jac #Sol   LinErr   LinRes
   1      0.0026   0.3500000      0.0026   34   25   25  7.7e-14  2.9e-15
   2      0.0017   0.3500000      0.0017   35   26   26  1.3e-13  3.4e-15
   3      0.0011   0.3500000      0.0011   36   27   27  7.4e-14    4e-15
Velocity u2, Pressure p
Iter      SolEst     Damping    Stepsize #Res #Jac #Sol   LinErr   LinRes
   1      0.0021   0.5000000      0.0021   18    9    9    3e-15  7.9e-15
Displacement field
Iter      SolEst     Damping    Stepsize #Res #Jac #Sol   LinErr   LinRes
   1       0.017   0.5000000       0.017   18    9    9  1.8e-13  1.5e-12
Spatial mesh displacement
Iter      SolEst     Damping    Stepsize #Res #Jac #Sol   LinErr   LinRes
   1     1.1e-07   0.5000000     1.1e-07   18    9    9  1.6e-15  1.9e-15
Solution error estimates for segregated groups
0.0019, 0.001, 0.0085, 5.3e-08
Residual error estimates for segregated groups
```

```
0.45, 1.3e+02, 1.4e+03, 0.18
Segregated solver iteration 10.
Merged variables
Iter      SolEst     Damping    Stepsize #Res #Jac #Sol   LinErr   LinRes
   1      0.0007   0.3500000      0.0007   38   28   28  1.1e-13  3.2e-15
   2     0.00046   0.3500000     0.00046   39   29   29    1e-13  3.1e-15
   3      0.0003   0.3500000      0.0003   40   30   30  1.6e-13  3.9e-15
Velocity u2, Pressure p
Iter      SolEst     Damping    Stepsize #Res #Jac #Sol   LinErr   LinRes
   1       0.001   0.5000000       0.001   20   10   10  4.5e-15  7.9e-15
Displacement field
Iter      SolEst     Damping    Stepsize #Res #Jac #Sol   LinErr   LinRes
   1      0.0094   0.5000000      0.0094   20   10   10  5.6e-14  1.5e-12
Spatial mesh displacement
Iter      SolEst     Damping    Stepsize #Res #Jac #Sol   LinErr   LinRes
   1     6.5e-08   0.5000000     6.5e-08   20   10   10  1.9e-15  1.9e-15
Solution error estimates for segregated groups
0.00051, 0.00052, 0.0047, 3.3e-08
Residual error estimates for segregated groups
0.14, 65, 1.2e+03, 0.18
Segregated solver iteration 11.
Merged variables
Iter      SolEst     Damping    Stepsize #Res #Jac #Sol   LinErr   LinRes
   1     0.00019   0.3500000     0.00019   42   31   31    1e-13  2.9e-15
   2     0.00013   0.3500000     0.00013   43   32   32    9e-14    3e-15
   3     8.2e-05   0.3500000     8.2e-05   44   33   33  1.5e-13  2.9e-15
Velocity u2, Pressure p
Iter      SolEst     Damping    Stepsize #Res #Jac #Sol   LinErr   LinRes
   1     0.00052   0.5000000     0.00052   22   11   11  2.6e-15  8.1e-15
Displacement field
Iter      SolEst     Damping    Stepsize #Res #Jac #Sol   LinErr   LinRes
   1      0.0051   0.5000000      0.0051   22   11   11  4.3e-14  1.5e-12
Spatial mesh displacement
Iter      SolEst     Damping    Stepsize #Res #Jac #Sol   LinErr   LinRes
   1     3.9e-08   0.5000000     3.9e-08   22   11   11  1.7e-15  1.9e-15
Solution error estimates for segregated groups
0.00014, 0.00026, 0.0026, 2e-08
Residual error estimates for segregated groups
0.044, 33, 1.2e+03, 0.18
Segregated solver iteration 12.
Merged variables
Iter      SolEst     Damping    Stepsize #Res #Jac #Sol   LinErr   LinRes
   1     5.3e-05   0.3500000     5.3e-05   46   34   34  5.1e-14  3.5e-15
   2     3.4e-05   0.3500000     3.4e-05   47   35   35  5.4e-14  3.7e-15
   3     2.2e-05   0.3500000     2.2e-05   48   36   36  9.9e-14  2.8e-15
Velocity u2, Pressure p
Iter      SolEst     Damping    Stepsize #Res #Jac #Sol   LinErr   LinRes
   1     0.00026   0.5000000     0.00026   24   12   12  5.9e-15  7.9e-15
Displacement field
Iter      SolEst     Damping    Stepsize #Res #Jac #Sol   LinErr   LinRes
   1      0.0028   0.5000000      0.0028   24   12   12  4.1e-14  1.5e-12
Spatial mesh displacement
Iter      SolEst     Damping    Stepsize #Res #Jac #Sol   LinErr   LinRes
   1     2.3e-08   0.5000000     2.3e-08   24   12   12  1.9e-15  1.9e-15
Solution error estimates for segregated groups
3.8e-05, 0.00013, 0.0014, 1.2e-08
```

```
Residual error estimates for segregated groups
0.013, 16, 1.2e+03, 0.2
Segregated solver iteration 13.
Merged variables
Iter      SolEst     Damping    Stepsize #Res #Jac #Sol   LinErr   LinRes
   1     1.5e-05   0.3500000     1.5e-05   50   37   37  7.9e-14  2.9e-15
   2     9.5e-06   0.3500000     9.5e-06   51   38   38  6.5e-14  3.1e-15
   3     6.2e-06   0.3500000     6.2e-06   52   39   39  5.4e-14    3e-15
Velocity u2, Pressure p
Iter      SolEst     Damping    Stepsize #Res #Jac #Sol   LinErr   LinRes
   1     0.00013   0.5000000     0.00013   26   13   13  2.1e-15  7.8e-15
Displacement field
Iter      SolEst     Damping    Stepsize #Res #Jac #Sol   LinErr   LinRes
   1      0.0015   0.5000000      0.0015   26   13   13  6.2e-14  1.5e-12
Spatial mesh displacement
Iter      SolEst     Damping    Stepsize #Res #Jac #Sol   LinErr   LinRes
   1     1.3e-08   0.5000000     1.3e-08   26   13   13  1.4e-15  1.9e-15
Solution error estimates for segregated groups
1.1e-05, 6.5e-05, 0.00076, 6.7e-09
Residual error estimates for segregated groups
0.0041, 8.2, 1.2e+03, 0.18
Solution time: 158 s. (2 minutes, 38 seconds)
Physical memory: 4.61 GB
Virtual memory: 25.54 GB
Ended at Jan 31, 2025, 6:05:59 PM.
----- Stationary Solver 1 in study1/Approach Curve Solver (sol1) -------------->
```

d=2365 (su29)

GENERAL

Description	Value
Solution	d=2365

LOG

```
<---- Compile Equations: Stationary in study1/Approach Curve Solver (sol1) -----
Started at Jan 31, 2025, 6:05:59 PM.
Parameter d = 2.3650000000000002E-6 (m).
Geometry shape function: Linear serendipity
Number of vertex elements: 18
Number of boundary elements: 6262
Number of elements: 156632
Minimum element quality: 0.4924
Time: 9 s.
Physical memory: 2.84 GB
Virtual memory: 15.18 GB
Ended at Jan 31, 2025, 6:06:08 PM.
----- Compile Equations: Stationary in study1/Approach Curve Solver (sol1) ---->

<---- Dependent Variables 1 in study1/Approach Curve Solver (sol1) -------------
Started at Jan 31, 2025, 6:06:08 PM.
Solution time: 0 s.
Physical memory: 2.84 GB
Virtual memory: 15.18 GB
Ended at Jan 31, 2025, 6:06:08 PM.
----- Dependent Variables 1 in study1/Approach Curve Solver (sol1) ------------>

<---- Stationary Solver 1 in study1/Approach Curve Solver (sol1) ---------------
Started at Jan 31, 2025, 6:06:08 PM.
Segregated solver
Segregated Step 11 does not have any components solved for.
Number of degrees of freedom solved for: 796768 (plus 11947 internal DOFs).
Segregated solver iteration 1.
Merged variables
Nonsymmetric matrix found.
Scales for dependent variables:
Concentration (comp1.Cl): 1.4e+02
Concentration (comp1.K): 1.4e+02
Electric potential (comp1.V): 1.9e+02
Orthonormal null-space function used.
Iter      SolEst     Damping    Stepsize #Res #Jac #Sol   LinErr   LinRes
   1        0.83   0.3500000        0.83    2    1    1  1.7e-11  2.8e-15
   2        0.81   0.3500000        0.81    3    2    2  3.2e-11  2.9e-15
   3         0.8   0.3500000         0.8    4    3    3  6.8e-11  2.9e-15
Velocity u2, Pressure p
Nonsymmetric matrix found.
Scales for dependent variables:
Pressure (comp1.p): 1
Velocity field (spatial frame) (comp1.u2): 0.00018
Orthonormal null-space function used.
Iter      SolEst     Damping    Stepsize #Res #Jac #Sol   LinErr   LinRes
   1         1.1   0.5000000         1.1    2    1    1  1.8e-15  2.2e-15
Displacement field
Nonsymmetric matrix found.
Scales for dependent variables:
Auxiliary pressure (comp1.solid.lemm1.pw): 1
Displacement field (comp1.u): 2.8e-18
Orthonormal null-space function used.
Iter      SolEst     Damping    Stepsize #Res #Jac #Sol   LinErr   LinRes
   1         1.9   0.5000000         1.9    2    1    1  1.2e-13  1.5e-12
Spatial mesh displacement
```

```
Symmetric matrices found.
Scales for dependent variables:
Spatial mesh displacement (comp1.spatial.disp): 1.2e-09
Orthonormal null-space function used.
Iter      SolEst     Damping    Stepsize #Res #Jac #Sol   LinErr   LinRes
   1     6.1e-07   0.5000000     6.1e-07    2    1    1  1.5e-15  1.9e-15
Solution error estimates for segregated groups
1.4, 0.54, 0.95, 3e-07
Residual error estimates for segregated groups
7.9e+03, 8.3e+04, 2.2e+04, 0.16
Segregated solver iteration 2.
Merged variables
Scales for dependent variables:
Concentration (comp1.Cl): 1.4e+02
Concentration (comp1.K): 1.4e+02
Electric potential (comp1.V): 0.064
Iter      SolEst     Damping    Stepsize #Res #Jac #Sol   LinErr   LinRes
   1        0.79   0.3500000        0.79    6    4    4  9.2e-14  4.2e-15
   2        0.77   0.3500000        0.77    7    5    5  6.3e-14    4e-15
   3        0.76   0.3500000        0.76    8    6    6  1.2e-13    3e-15
Velocity u2, Pressure p
Scales for dependent variables:
Pressure (comp1.p): 3.5e+02
Velocity field (spatial frame) (comp1.u2): 0.00071
Iter      SolEst     Damping    Stepsize #Res #Jac #Sol   LinErr   LinRes
   1        0.36   0.5000000        0.36    4    2    2  3.1e-15    8e-15
Displacement field
Iter      SolEst     Damping    Stepsize #Res #Jac #Sol   LinErr   LinRes
   1        0.95   0.5000000        0.95    4    2    2  8.4e-14  1.5e-12
Spatial mesh displacement
Iter      SolEst     Damping    Stepsize #Res #Jac #Sol   LinErr   LinRes
   1     9.1e-07   0.5000000     9.1e-07    4    2    2  1.9e-15  1.8e-15
Solution error estimates for segregated groups
1.3, 0.18, 0.48, 4.6e-07
Residual error estimates for segregated groups
8.1e+02, 1.4e+04, 2.2e+04, 0.18
Segregated solver iteration 3.
Merged variables
Iter      SolEst     Damping    Stepsize #Res #Jac #Sol   LinErr   LinRes
   1        0.75   0.3500000        0.75   10    7    7  5.8e-14  3.2e-15
   2        0.74   0.3500000        0.74   11    8    8  6.1e-14    3e-15
   3        0.72   0.3500000        0.72   12    9    9  7.3e-14  2.9e-15
Velocity u2, Pressure p
Iter      SolEst     Damping    Stepsize #Res #Jac #Sol   LinErr   LinRes
   1        0.15   0.5000000        0.15    6    3    3  4.4e-15  7.8e-15
Displacement field
Iter      SolEst     Damping    Stepsize #Res #Jac #Sol   LinErr   LinRes
   1        0.52   0.5000000        0.52    6    3    3  1.3e-13  1.5e-12
Spatial mesh displacement
Iter      SolEst     Damping    Stepsize #Res #Jac #Sol   LinErr   LinRes
   1     9.1e-07   0.5000000     9.1e-07    6    3    3  1.5e-15  1.9e-15
Solution error estimates for segregated groups
1.2, 0.077, 0.26, 4.6e-07
Residual error estimates for segregated groups
2.2e+02, 7.6e+03, 1.6e+04, 0.17
Segregated solver iteration 4.
```

```
Merged variables
Iter      SolEst     Damping    Stepsize #Res #Jac #Sol   LinErr   LinRes
   1         0.7   0.3500000         0.7   14   10   10  4.1e-14  3.3e-15
   2        0.68   0.3500000        0.68   15   11   11  1.3e-13  3.4e-15
   3         0.6   0.3500000         0.6   16   12   12  8.9e-14    3e-15
Velocity u2, Pressure p
Iter      SolEst     Damping    Stepsize #Res #Jac #Sol   LinErr   LinRes
   1       0.071   0.5000000       0.071    8    4    4  3.7e-15  7.8e-15
Displacement field
Iter      SolEst     Damping    Stepsize #Res #Jac #Sol   LinErr   LinRes
   1        0.29   0.5000000        0.29    8    4    4  9.3e-14  1.5e-12
Spatial mesh displacement
Iter      SolEst     Damping    Stepsize #Res #Jac #Sol   LinErr   LinRes
   1     7.6e-07   0.5000000     7.6e-07    8    4    4  1.6e-15  1.8e-15
Solution error estimates for segregated groups
1, 0.036, 0.15, 3.8e-07
Residual error estimates for segregated groups
85, 4e+03, 1.1e+04, 0.17
Segregated solver iteration 5.
Merged variables
Iter      SolEst     Damping    Stepsize #Res #Jac #Sol   LinErr   LinRes
   1        0.42   0.3500000        0.42   18   13   13  7.7e-14    3e-15
   2        0.28   0.3500000        0.28   19   14   14  5.4e-14  3.3e-15
   3        0.18   0.3500000        0.18   20   15   15  1.1e-13  2.9e-15
Velocity u2, Pressure p
Iter      SolEst     Damping    Stepsize #Res #Jac #Sol   LinErr   LinRes
   1       0.035   0.5000000       0.035   10    5    5  3.8e-15  7.9e-15
Displacement field
Iter      SolEst     Damping    Stepsize #Res #Jac #Sol   LinErr   LinRes
   1        0.17   0.5000000        0.17   10    5    5  1.1e-13  1.5e-12
Spatial mesh displacement
Iter      SolEst     Damping    Stepsize #Res #Jac #Sol   LinErr   LinRes
   1     5.7e-07   0.5000000     5.7e-07   10    5    5  1.5e-15  1.8e-15
Solution error estimates for segregated groups
0.32, 0.017, 0.084, 2.9e-07
Residual error estimates for segregated groups
33, 2.1e+03, 6.9e+03, 0.19
Segregated solver iteration 6.
Merged variables
Iter      SolEst     Damping    Stepsize #Res #Jac #Sol   LinErr   LinRes
   1        0.12   0.3500000        0.12   22   16   16  7.6e-14  3.2e-15
   2       0.079   0.3500000       0.079   23   17   17  7.2e-14  4.3e-15
   3       0.052   0.3500000       0.052   24   18   18  5.4e-14  3.4e-15
Velocity u2, Pressure p
Iter      SolEst     Damping    Stepsize #Res #Jac #Sol   LinErr   LinRes
   1       0.017   0.5000000       0.017   12    6    6  2.7e-15  7.8e-15
Displacement field
Iter      SolEst     Damping    Stepsize #Res #Jac #Sol   LinErr   LinRes
   1       0.095   0.5000000       0.095   12    6    6  1.5e-13  1.5e-12
Spatial mesh displacement
Iter      SolEst     Damping    Stepsize #Res #Jac #Sol   LinErr   LinRes
   1       4e-07   0.5000000       4e-07   12    6    6  1.4e-15  1.8e-15
Solution error estimates for segregated groups
0.089, 0.0085, 0.048, 2e-07
Residual error estimates for segregated groups
12, 1e+03, 4.2e+03, 0.19
```

```
Segregated solver iteration 7.
Merged variables
Iter      SolEst     Damping    Stepsize #Res #Jac #Sol   LinErr   LinRes
   1       0.034   0.3500000       0.034   26   19   19  1.3e-13  3.4e-15
   2       0.022   0.3500000       0.022   27   20   20    1e-13    3e-15
   3       0.014   0.3500000       0.014   28   21   21  5.7e-14  2.8e-15
Velocity u2, Pressure p
Iter      SolEst     Damping    Stepsize #Res #Jac #Sol   LinErr   LinRes
   1      0.0084   0.5000000      0.0084   14    7    7  3.9e-15  7.7e-15
Displacement field
Iter      SolEst     Damping    Stepsize #Res #Jac #Sol   LinErr   LinRes
   1       0.054   0.5000000       0.054   14    7    7  2.9e-14  1.5e-12
Spatial mesh displacement
Iter      SolEst     Damping    Stepsize #Res #Jac #Sol   LinErr   LinRes
   1     2.7e-07   0.5000000     2.7e-07   14    7    7    2e-15  1.9e-15
Solution error estimates for segregated groups
0.025, 0.0042, 0.027, 1.3e-07
Residual error estimates for segregated groups
4.2, 5.2e+02, 2.6e+03, 0.19
Segregated solver iteration 8.
Merged variables
Iter      SolEst     Damping    Stepsize #Res #Jac #Sol   LinErr   LinRes
   1      0.0093   0.3500000      0.0093   30   22   22    1e-13  3.4e-15
   2       0.006   0.3500000       0.006   31   23   23  9.8e-14  2.9e-15
   3      0.0039   0.3500000      0.0039   32   24   24  9.7e-14  3.6e-15
Velocity u2, Pressure p
Iter      SolEst     Damping    Stepsize #Res #Jac #Sol   LinErr   LinRes
   1      0.0042   0.5000000      0.0042   16    8    8    5e-15  7.8e-15
Displacement field
Iter      SolEst     Damping    Stepsize #Res #Jac #Sol   LinErr   LinRes
   1        0.03   0.5000000        0.03   16    8    8  7.6e-14  1.5e-12
Spatial mesh displacement
Iter      SolEst     Damping    Stepsize #Res #Jac #Sol   LinErr   LinRes
   1     1.7e-07   0.5000000     1.7e-07   16    8    8  1.4e-15  1.8e-15
Solution error estimates for segregated groups
0.0067, 0.0021, 0.015, 8.6e-08
Residual error estimates for segregated groups
1.4, 2.6e+02, 1.8e+03, 0.18
Segregated solver iteration 9.
Merged variables
Iter      SolEst     Damping    Stepsize #Res #Jac #Sol   LinErr   LinRes
   1      0.0026   0.3500000      0.0026   34   25   25  3.6e-14  3.3e-15
   2      0.0017   0.3500000      0.0017   35   26   26  8.3e-14  3.1e-15
   3      0.0011   0.3500000      0.0011   36   27   27  5.3e-14  3.3e-15
Velocity u2, Pressure p
Iter      SolEst     Damping    Stepsize #Res #Jac #Sol   LinErr   LinRes
   1      0.0021   0.5000000      0.0021   18    9    9  6.1e-15    8e-15
Displacement field
Iter      SolEst     Damping    Stepsize #Res #Jac #Sol   LinErr   LinRes
   1       0.017   0.5000000       0.017   18    9    9  6.9e-14  1.5e-12
Spatial mesh displacement
Iter      SolEst     Damping    Stepsize #Res #Jac #Sol   LinErr   LinRes
   1     1.1e-07   0.5000000     1.1e-07   18    9    9  1.4e-15  1.8e-15
Solution error estimates for segregated groups
0.0019, 0.001, 0.0085, 5.4e-08
Residual error estimates for segregated groups
```

```
0.45, 1.3e+02, 1.4e+03, 0.18
Segregated solver iteration 10.
Merged variables
Iter      SolEst     Damping    Stepsize #Res #Jac #Sol   LinErr   LinRes
   1      0.0007   0.3500000      0.0007   38   28   28  7.3e-14  2.8e-15
   2     0.00046   0.3500000     0.00046   39   29   29  1.5e-13  3.7e-15
   3      0.0003   0.3500000      0.0003   40   30   30  9.3e-14  3.3e-15
Velocity u2, Pressure p
Iter      SolEst     Damping    Stepsize #Res #Jac #Sol   LinErr   LinRes
   1       0.001   0.5000000       0.001   20   10   10  2.8e-15  7.9e-15
Displacement field
Iter      SolEst     Damping    Stepsize #Res #Jac #Sol   LinErr   LinRes
   1      0.0094   0.5000000      0.0094   20   10   10  2.3e-13  1.5e-12
Spatial mesh displacement
Iter      SolEst     Damping    Stepsize #Res #Jac #Sol   LinErr   LinRes
   1     6.5e-08   0.5000000     6.5e-08   20   10   10  1.3e-15  1.8e-15
Solution error estimates for segregated groups
0.00051, 0.00052, 0.0047, 3.3e-08
Residual error estimates for segregated groups
0.14, 66, 1.2e+03, 0.18
Segregated solver iteration 11.
Merged variables
Iter      SolEst     Damping    Stepsize #Res #Jac #Sol   LinErr   LinRes
   1     0.00019   0.3500000     0.00019   42   31   31  5.8e-14  3.1e-15
   2     0.00013   0.3500000     0.00013   43   32   32  9.9e-14    3e-15
   3     8.1e-05   0.3500000     8.1e-05   44   33   33  9.9e-14  3.1e-15
Velocity u2, Pressure p
Iter      SolEst     Damping    Stepsize #Res #Jac #Sol   LinErr   LinRes
   1     0.00052   0.5000000     0.00052   22   11   11  2.7e-15  7.9e-15
Displacement field
Iter      SolEst     Damping    Stepsize #Res #Jac #Sol   LinErr   LinRes
   1      0.0051   0.5000000      0.0051   22   11   11  7.9e-14  1.5e-12
Spatial mesh displacement
Iter      SolEst     Damping    Stepsize #Res #Jac #Sol   LinErr   LinRes
   1     3.9e-08   0.5000000     3.9e-08   22   11   11  2.3e-15  1.9e-15
Solution error estimates for segregated groups
0.00014, 0.00026, 0.0026, 2e-08
Residual error estimates for segregated groups
0.044, 33, 1.2e+03, 0.18
Segregated solver iteration 12.
Merged variables
Iter      SolEst     Damping    Stepsize #Res #Jac #Sol   LinErr   LinRes
   1     5.3e-05   0.3500000     5.3e-05   46   34   34  6.3e-14  3.2e-15
   2     3.4e-05   0.3500000     3.4e-05   47   35   35  6.9e-14  3.4e-15
   3     2.2e-05   0.3500000     2.2e-05   48   36   36  4.1e-14  3.5e-15
Velocity u2, Pressure p
Iter      SolEst     Damping    Stepsize #Res #Jac #Sol   LinErr   LinRes
   1     0.00026   0.5000000     0.00026   24   12   12  2.8e-15  7.8e-15
Displacement field
Iter      SolEst     Damping    Stepsize #Res #Jac #Sol   LinErr   LinRes
   1      0.0028   0.5000000      0.0028   24   12   12  2.5e-14  1.5e-12
Spatial mesh displacement
Iter      SolEst     Damping    Stepsize #Res #Jac #Sol   LinErr   LinRes
   1     2.3e-08   0.5000000     2.3e-08   24   12   12  1.5e-15  1.8e-15
Solution error estimates for segregated groups
3.8e-05, 0.00013, 0.0014, 1.2e-08
```

```
Residual error estimates for segregated groups
0.013, 16, 1.2e+03, 0.18
Segregated solver iteration 13.
Merged variables
Iter      SolEst     Damping    Stepsize #Res #Jac #Sol   LinErr   LinRes
   1     1.5e-05   0.3500000     1.5e-05   50   37   37  4.2e-14  2.9e-15
   2     9.5e-06   0.3500000     9.5e-06   51   38   38  4.7e-14  3.1e-15
   3     6.1e-06   0.3500000     6.1e-06   52   39   39  1.5e-13  3.4e-15
Velocity u2, Pressure p
Iter      SolEst     Damping    Stepsize #Res #Jac #Sol   LinErr   LinRes
   1     0.00013   0.5000000     0.00013   26   13   13  2.4e-15  7.8e-15
Displacement field
Iter      SolEst     Damping    Stepsize #Res #Jac #Sol   LinErr   LinRes
   1      0.0015   0.5000000      0.0015   26   13   13  4.5e-14  1.5e-12
Spatial mesh displacement
Iter      SolEst     Damping    Stepsize #Res #Jac #Sol   LinErr   LinRes
   1     1.4e-08   0.5000000     1.4e-08   26   13   13    2e-15  1.8e-15
Solution error estimates for segregated groups
1.1e-05, 6.5e-05, 0.00076, 6.8e-09
Residual error estimates for segregated groups
0.0041, 8.2, 1.2e+03, 0.19
Solution time: 158 s. (2 minutes, 38 seconds)
Physical memory: 4.66 GB
Virtual memory: 25.57 GB
Ended at Jan 31, 2025, 6:08:46 PM.
----- Stationary Solver 1 in study1/Approach Curve Solver (sol1) -------------->
```

d=2360 (su30)

GENERAL

Description	Value
Solution	d=2360

LOG

```
<---- Compile Equations: Stationary in study1/Approach Curve Solver (sol1) -----
Started at Jan 31, 2025, 6:08:46 PM.
Parameter d = 2.3600000000000003E-6 (m).
Geometry shape function: Linear serendipity
Number of vertex elements: 18
Number of boundary elements: 6259
Number of elements: 156575
Minimum element quality: 0.4865
Time: 8 s.
Physical memory: 2.87 GB
Virtual memory: 15.2 GB
Ended at Jan 31, 2025, 6:08:55 PM.
----- Compile Equations: Stationary in study1/Approach Curve Solver (sol1) ---->

<---- Dependent Variables 1 in study1/Approach Curve Solver (sol1) -------------
Started at Jan 31, 2025, 6:08:55 PM.
Solution time: 0 s.
Physical memory: 2.87 GB
Virtual memory: 15.2 GB
Ended at Jan 31, 2025, 6:08:55 PM.
----- Dependent Variables 1 in study1/Approach Curve Solver (sol1) ------------>

<---- Stationary Solver 1 in study1/Approach Curve Solver (sol1) ---------------
Started at Jan 31, 2025, 6:08:55 PM.
Segregated solver
Segregated Step 11 does not have any components solved for.
Number of degrees of freedom solved for: 796179 (plus 11941 internal DOFs).
Segregated solver iteration 1.
Merged variables
Nonsymmetric matrix found.
Scales for dependent variables:
Concentration (comp1.Cl): 1.4e+02
Concentration (comp1.K): 1.4e+02
Electric potential (comp1.V): 1.9e+02
Orthonormal null-space function used.
Iter      SolEst     Damping    Stepsize #Res #Jac #Sol   LinErr   LinRes
   1        0.83   0.3500000        0.83    2    1    1    2e-11  2.8e-15
   2        0.81   0.3500000        0.81    3    2    2  1.6e-11  2.7e-15
   3         0.8   0.3500000         0.8    4    3    3    2e-11  2.7e-15
Velocity u2, Pressure p
Nonsymmetric matrix found.
Scales for dependent variables:
Pressure (comp1.p): 1
Velocity field (spatial frame) (comp1.u2): 0.00018
Orthonormal null-space function used.
Iter      SolEst     Damping    Stepsize #Res #Jac #Sol   LinErr   LinRes
   1         1.1   0.5000000         1.1    2    1    1  1.6e-15  2.3e-15
Displacement field
Nonsymmetric matrix found.
Scales for dependent variables:
Auxiliary pressure (comp1.solid.lemm1.pw): 1
Displacement field (comp1.u): 2.8e-18
Orthonormal null-space function used.
Iter      SolEst     Damping    Stepsize #Res #Jac #Sol   LinErr   LinRes
   1         1.9   0.5000000         1.9    2    1    1  1.2e-13  1.5e-12
Spatial mesh displacement
```

```
Symmetric matrices found.
Scales for dependent variables:
Spatial mesh displacement (comp1.spatial.disp): 1.2e-09
Orthonormal null-space function used.
Iter      SolEst     Damping    Stepsize #Res #Jac #Sol   LinErr   LinRes
   1     6.1e-07   0.5000000     6.1e-07    2    1    1  1.8e-15  1.8e-15
Solution error estimates for segregated groups
1.4, 0.54, 0.95, 3.1e-07
Residual error estimates for segregated groups
7.9e+03, 8.3e+04, 2.2e+04, 0.17
Segregated solver iteration 2.
Merged variables
Scales for dependent variables:
Concentration (comp1.Cl): 1.4e+02
Concentration (comp1.K): 1.4e+02
Electric potential (comp1.V): 0.064
Iter      SolEst     Damping    Stepsize #Res #Jac #Sol   LinErr   LinRes
   1        0.79   0.3500000        0.79    6    4    4  7.2e-14  5.5e-15
   2        0.77   0.3500000        0.77    7    5    5    1e-13  3.4e-15
   3        0.76   0.3500000        0.76    8    6    6  5.5e-14  4.8e-15
Velocity u2, Pressure p
Scales for dependent variables:
Pressure (comp1.p): 3.5e+02
Velocity field (spatial frame) (comp1.u2): 0.00071
Iter      SolEst     Damping    Stepsize #Res #Jac #Sol   LinErr   LinRes
   1        0.36   0.5000000        0.36    4    2    2  2.8e-15  7.9e-15
Displacement field
Iter      SolEst     Damping    Stepsize #Res #Jac #Sol   LinErr   LinRes
   1        0.95   0.5000000        0.95    4    2    2  8.2e-14  1.5e-12
Spatial mesh displacement
Iter      SolEst     Damping    Stepsize #Res #Jac #Sol   LinErr   LinRes
   1     9.2e-07   0.5000000     9.2e-07    4    2    2  1.5e-15  1.9e-15
Solution error estimates for segregated groups
1.3, 0.18, 0.48, 4.6e-07
Residual error estimates for segregated groups
8.1e+02, 1.3e+04, 2.2e+04, 0.17
Segregated solver iteration 3.
Merged variables
Iter      SolEst     Damping    Stepsize #Res #Jac #Sol   LinErr   LinRes
   1        0.75   0.3500000        0.75   10    7    7  7.1e-14    9e-15
   2        0.74   0.3500000        0.74   11    8    8  6.5e-14  3.6e-15
   3        0.72   0.3500000        0.72   12    9    9  8.6e-14  4.5e-15
Velocity u2, Pressure p
Iter      SolEst     Damping    Stepsize #Res #Jac #Sol   LinErr   LinRes
   1        0.15   0.5000000        0.15    6    3    3  4.2e-15  7.7e-15
Displacement field
Iter      SolEst     Damping    Stepsize #Res #Jac #Sol   LinErr   LinRes
   1        0.52   0.5000000        0.52    6    3    3  6.6e-14  1.5e-12
Spatial mesh displacement
Iter      SolEst     Damping    Stepsize #Res #Jac #Sol   LinErr   LinRes
   1     9.2e-07   0.5000000     9.2e-07    6    3    3  1.5e-15  1.9e-15
Solution error estimates for segregated groups
1.2, 0.077, 0.26, 4.6e-07
Residual error estimates for segregated groups
2.2e+02, 7.2e+03, 1.6e+04, 0.17
Segregated solver iteration 4.
```

```
Merged variables
Iter      SolEst     Damping    Stepsize #Res #Jac #Sol   LinErr   LinRes
   1         0.7   0.3500000         0.7   14   10   10  1.7e-13  2.4e-14
   2        0.68   0.3500000        0.68   15   11   11  1.5e-13  2.6e-14
   3         0.6   0.3500000         0.6   16   12   12  2.8e-13  1.5e-14
Velocity u2, Pressure p
Iter      SolEst     Damping    Stepsize #Res #Jac #Sol   LinErr   LinRes
   1       0.072   0.5000000       0.072    8    4    4  4.4e-15    8e-15
Displacement field
Iter      SolEst     Damping    Stepsize #Res #Jac #Sol   LinErr   LinRes
   1        0.29   0.5000000        0.29    8    4    4  2.6e-13  1.5e-12
Spatial mesh displacement
Iter      SolEst     Damping    Stepsize #Res #Jac #Sol   LinErr   LinRes
   1     7.6e-07   0.5000000     7.6e-07    8    4    4  1.6e-15  1.8e-15
Solution error estimates for segregated groups
1, 0.036, 0.15, 3.8e-07
Residual error estimates for segregated groups
84, 3.8e+03, 1.1e+04, 0.19
Segregated solver iteration 5.
Merged variables
Iter      SolEst     Damping    Stepsize #Res #Jac #Sol   LinErr   LinRes
   1        0.41   0.3500000        0.41   18   13   13  3.5e-13  6.5e-14
   2        0.28   0.3500000        0.28   19   14   14  6.2e-13  1.3e-14
   3        0.18   0.3500000        0.18   20   15   15  2.4e-13  4.4e-14
Velocity u2, Pressure p
Iter      SolEst     Damping    Stepsize #Res #Jac #Sol   LinErr   LinRes
   1       0.035   0.5000000       0.035   10    5    5  2.4e-15    8e-15
Displacement field
Iter      SolEst     Damping    Stepsize #Res #Jac #Sol   LinErr   LinRes
   1        0.17   0.5000000        0.17   10    5    5  4.7e-14  1.5e-12
Spatial mesh displacement
Iter      SolEst     Damping    Stepsize #Res #Jac #Sol   LinErr   LinRes
   1     5.7e-07   0.5000000     5.7e-07   10    5    5  1.4e-15  1.9e-15
Solution error estimates for segregated groups
0.32, 0.017, 0.084, 2.9e-07
Residual error estimates for segregated groups
33, 1.9e+03, 6.9e+03, 0.17
Segregated solver iteration 6.
Merged variables
Iter      SolEst     Damping    Stepsize #Res #Jac #Sol   LinErr   LinRes
   1        0.12   0.3500000        0.12   22   16   16  1.8e-13  2.8e-14
   2       0.079   0.3500000       0.079   23   17   17  1.5e-13  1.4e-14
   3       0.052   0.3500000       0.052   24   18   18  7.1e-13  3.1e-14
Velocity u2, Pressure p
Iter      SolEst     Damping    Stepsize #Res #Jac #Sol   LinErr   LinRes
   1       0.017   0.5000000       0.017   12    6    6    3e-15  7.8e-15
Displacement field
Iter      SolEst     Damping    Stepsize #Res #Jac #Sol   LinErr   LinRes
   1       0.095   0.5000000       0.095   12    6    6  2.6e-14  1.5e-12
Spatial mesh displacement
Iter      SolEst     Damping    Stepsize #Res #Jac #Sol   LinErr   LinRes
   1       4e-07   0.5000000       4e-07   12    6    6  1.3e-15  1.8e-15
Solution error estimates for segregated groups
0.088, 0.0085, 0.048, 2e-07
Residual error estimates for segregated groups
12, 9.8e+02, 4.2e+03, 0.19
```

```
Segregated solver iteration 7.
Merged variables
Iter      SolEst     Damping    Stepsize #Res #Jac #Sol   LinErr   LinRes
   1       0.034   0.3500000       0.034   26   19   19  2.1e-13  5.1e-14
   2       0.022   0.3500000       0.022   27   20   20  5.1e-13  1.1e-14
   3       0.014   0.3500000       0.014   28   21   21    2e-13  3.4e-14
Velocity u2, Pressure p
Iter      SolEst     Damping    Stepsize #Res #Jac #Sol   LinErr   LinRes
   1      0.0084   0.5000000      0.0084   14    7    7  6.1e-15  7.9e-15
Displacement field
Iter      SolEst     Damping    Stepsize #Res #Jac #Sol   LinErr   LinRes
   1       0.054   0.5000000       0.054   14    7    7  7.1e-14  1.5e-12
Spatial mesh displacement
Iter      SolEst     Damping    Stepsize #Res #Jac #Sol   LinErr   LinRes
   1     2.7e-07   0.5000000     2.7e-07   14    7    7  1.4e-15  1.8e-15
Solution error estimates for segregated groups
0.024, 0.0042, 0.027, 1.3e-07
Residual error estimates for segregated groups
4.1, 4.9e+02, 2.6e+03, 0.17
Segregated solver iteration 8.
Merged variables
Iter      SolEst     Damping    Stepsize #Res #Jac #Sol   LinErr   LinRes
   1      0.0093   0.3500000      0.0093   30   22   22  3.4e-13  7.9e-15
   2       0.006   0.3500000       0.006   31   23   23  3.2e-13  1.1e-14
   3      0.0039   0.3500000      0.0039   32   24   24  2.8e-13  5.9e-14
Velocity u2, Pressure p
Iter      SolEst     Damping    Stepsize #Res #Jac #Sol   LinErr   LinRes
   1      0.0042   0.5000000      0.0042   16    8    8  3.4e-15  7.9e-15
Displacement field
Iter      SolEst     Damping    Stepsize #Res #Jac #Sol   LinErr   LinRes
   1        0.03   0.5000000        0.03   16    8    8  4.7e-14  1.5e-12
Spatial mesh displacement
Iter      SolEst     Damping    Stepsize #Res #Jac #Sol   LinErr   LinRes
   1     1.7e-07   0.5000000     1.7e-07   16    8    8  1.3e-15  1.9e-15
Solution error estimates for segregated groups
0.0067, 0.0021, 0.015, 8.6e-08
Residual error estimates for segregated groups
1.4, 2.5e+02, 1.8e+03, 0.18
Segregated solver iteration 9.
Merged variables
Iter      SolEst     Damping    Stepsize #Res #Jac #Sol   LinErr   LinRes
   1      0.0025   0.3500000      0.0025   34   25   25  4.4e-13  1.5e-14
   2      0.0017   0.3500000      0.0017   35   26   26  2.3e-13  2.7e-14
   3      0.0011   0.3500000      0.0011   36   27   27  2.7e-13  2.2e-14
Velocity u2, Pressure p
Iter      SolEst     Damping    Stepsize #Res #Jac #Sol   LinErr   LinRes
   1      0.0021   0.5000000      0.0021   18    9    9  2.4e-15  7.8e-15
Displacement field
Iter      SolEst     Damping    Stepsize #Res #Jac #Sol   LinErr   LinRes
   1       0.017   0.5000000       0.017   18    9    9  2.8e-14  1.5e-12
Spatial mesh displacement
Iter      SolEst     Damping    Stepsize #Res #Jac #Sol   LinErr   LinRes
   1     1.1e-07   0.5000000     1.1e-07   18    9    9  1.9e-15  1.8e-15
Solution error estimates for segregated groups
0.0018, 0.001, 0.0085, 5.4e-08
Residual error estimates for segregated groups
```

```
0.44, 1.2e+02, 1.4e+03, 0.18
Segregated solver iteration 10.
Merged variables
Iter      SolEst     Damping    Stepsize #Res #Jac #Sol   LinErr   LinRes
   1      0.0007   0.3500000      0.0007   38   28   28  2.1e-13  4.2e-14
   2     0.00045   0.3500000     0.00045   39   29   29  1.7e-13  2.3e-14
   3      0.0003   0.3500000      0.0003   40   30   30  1.9e-13  8.9e-15
Velocity u2, Pressure p
Iter      SolEst     Damping    Stepsize #Res #Jac #Sol   LinErr   LinRes
   1       0.001   0.5000000       0.001   20   10   10  2.4e-15  7.8e-15
Displacement field
Iter      SolEst     Damping    Stepsize #Res #Jac #Sol   LinErr   LinRes
   1      0.0094   0.5000000      0.0094   20   10   10    2e-14  1.5e-12
Spatial mesh displacement
Iter      SolEst     Damping    Stepsize #Res #Jac #Sol   LinErr   LinRes
   1     6.6e-08   0.5000000     6.6e-08   20   10   10  1.2e-15  1.9e-15
Solution error estimates for segregated groups
0.00051, 0.00052, 0.0047, 3.3e-08
Residual error estimates for segregated groups
0.14, 62, 1.2e+03, 0.2
Segregated solver iteration 11.
Merged variables
Iter      SolEst     Damping    Stepsize #Res #Jac #Sol   LinErr   LinRes
   1     0.00019   0.3500000     0.00019   42   31   31  3.7e-13  6.4e-14
   2     0.00012   0.3500000     0.00012   43   32   32  2.1e-13  2.6e-14
   3     8.1e-05   0.3500000     8.1e-05   44   33   33  2.9e-13    7e-15
Velocity u2, Pressure p
Iter      SolEst     Damping    Stepsize #Res #Jac #Sol   LinErr   LinRes
   1     0.00052   0.5000000     0.00052   22   11   11  3.4e-15  7.8e-15
Displacement field
Iter      SolEst     Damping    Stepsize #Res #Jac #Sol   LinErr   LinRes
   1      0.0051   0.5000000      0.0051   22   11   11  2.4e-14  1.5e-12
Spatial mesh displacement
Iter      SolEst     Damping    Stepsize #Res #Jac #Sol   LinErr   LinRes
   1     3.9e-08   0.5000000     3.9e-08   22   11   11  1.3e-15  1.9e-15
Solution error estimates for segregated groups
0.00014, 0.00026, 0.0026, 2e-08
Residual error estimates for segregated groups
0.044, 31, 1.2e+03, 0.18
Segregated solver iteration 12.
Merged variables
Iter      SolEst     Damping    Stepsize #Res #Jac #Sol   LinErr   LinRes
   1     5.3e-05   0.3500000     5.3e-05   46   34   34  3.8e-13    8e-15
   2     3.4e-05   0.3500000     3.4e-05   47   35   35  9.3e-13    6e-14
   3     2.2e-05   0.3500000     2.2e-05   48   36   36  4.4e-13  2.7e-14
Velocity u2, Pressure p
Iter      SolEst     Damping    Stepsize #Res #Jac #Sol   LinErr   LinRes
   1     0.00026   0.5000000     0.00026   24   12   12  4.1e-15  7.9e-15
Displacement field
Iter      SolEst     Damping    Stepsize #Res #Jac #Sol   LinErr   LinRes
   1      0.0028   0.5000000      0.0028   24   12   12  6.2e-14  1.5e-12
Spatial mesh displacement
Iter      SolEst     Damping    Stepsize #Res #Jac #Sol   LinErr   LinRes
   1     2.3e-08   0.5000000     2.3e-08   24   12   12  1.6e-15  1.8e-15
Solution error estimates for segregated groups
3.8e-05, 0.00013, 0.0014, 1.2e-08
```

```
Residual error estimates for segregated groups
0.013, 16, 1.2e+03, 0.17
Segregated solver iteration 13.
Merged variables
Iter      SolEst     Damping    Stepsize #Res #Jac #Sol   LinErr   LinRes
   1     1.4e-05   0.3500000     1.4e-05   50   37   37  2.1e-13  7.5e-15
   2     9.4e-06   0.3500000     9.4e-06   51   38   38  4.4e-13  4.2e-14
   3     6.1e-06   0.3500000     6.1e-06   52   39   39  1.9e-13  1.1e-14
Velocity u2, Pressure p
Iter      SolEst     Damping    Stepsize #Res #Jac #Sol   LinErr   LinRes
   1     0.00013   0.5000000     0.00013   26   13   13  5.9e-15  7.9e-15
Displacement field
Iter      SolEst     Damping    Stepsize #Res #Jac #Sol   LinErr   LinRes
   1      0.0015   0.5000000      0.0015   26   13   13  7.5e-14  1.5e-12
Spatial mesh displacement
Iter      SolEst     Damping    Stepsize #Res #Jac #Sol   LinErr   LinRes
   1     1.4e-08   0.5000000     1.4e-08   26   13   13  1.5e-15  1.8e-15
Solution error estimates for segregated groups
1.1e-05, 6.5e-05, 0.00076, 6.8e-09
Residual error estimates for segregated groups
0.0041, 7.8, 1.2e+03, 0.18
Solution time: 157 s. (2 minutes, 37 seconds)
Physical memory: 4.64 GB
Virtual memory: 25.9 GB
Ended at Jan 31, 2025, 6:11:32 PM.
----- Stationary Solver 1 in study1/Approach Curve Solver (sol1) -------------->
```

d=2355 (su31)

GENERAL

Description	Value
Solution	d=2355

LOG

```
<---- Compile Equations: Stationary in study1/Approach Curve Solver (sol1) -----
Started at Jan 31, 2025, 6:11:32 PM.
Parameter d = 2.3550000000000003E-6 (m).
Geometry shape function: Linear serendipity
Number of vertex elements: 18
Number of boundary elements: 6257
Number of elements: 156279
Minimum element quality: 0.4504
Time: 9 s.
Physical memory: 2.8 GB
Virtual memory: 15.13 GB
Ended at Jan 31, 2025, 6:11:41 PM.
----- Compile Equations: Stationary in study1/Approach Curve Solver (sol1) ---->

<---- Dependent Variables 1 in study1/Approach Curve Solver (sol1) -------------
Started at Jan 31, 2025, 6:11:41 PM.
Solution time: 0 s.
Physical memory: 2.8 GB
Virtual memory: 15.13 GB
Ended at Jan 31, 2025, 6:11:41 PM.
----- Dependent Variables 1 in study1/Approach Curve Solver (sol1) ------------>

<---- Stationary Solver 1 in study1/Approach Curve Solver (sol1) ---------------
Started at Jan 31, 2025, 6:11:41 PM.
Segregated solver
Segregated Step 11 does not have any components solved for.
Number of degrees of freedom solved for: 794742 (plus 11937 internal DOFs).
Segregated solver iteration 1.
Merged variables
Nonsymmetric matrix found.
Scales for dependent variables:
Concentration (comp1.Cl): 1.4e+02
Concentration (comp1.K): 1.4e+02
Electric potential (comp1.V): 1.9e+02
Orthonormal null-space function used.
Iter      SolEst     Damping    Stepsize #Res #Jac #Sol   LinErr   LinRes
   1        0.83   0.3500000        0.83    2    1    1  2.2e-11  2.9e-15
   2        0.81   0.3500000        0.81    3    2    2  3.3e-11  2.8e-15
   3         0.8   0.3500000         0.8    4    3    3  3.4e-11  2.7e-15
Velocity u2, Pressure p
Nonsymmetric matrix found.
Scales for dependent variables:
Pressure (comp1.p): 1
Velocity field (spatial frame) (comp1.u2): 0.00018
Orthonormal null-space function used.
Iter      SolEst     Damping    Stepsize #Res #Jac #Sol   LinErr   LinRes
   1         1.1   0.5000000         1.1    2    1    1  2.3e-15  2.3e-15
Displacement field
Nonsymmetric matrix found.
Scales for dependent variables:
Auxiliary pressure (comp1.solid.lemm1.pw): 1
Displacement field (comp1.u): 2.8e-18
Orthonormal null-space function used.
Iter      SolEst     Damping    Stepsize #Res #Jac #Sol   LinErr   LinRes
   1         1.9   0.5000000         1.9    2    1    1  3.9e-14  1.5e-12
Spatial mesh displacement
```

```
Symmetric matrices found.
Scales for dependent variables:
Spatial mesh displacement (comp1.spatial.disp): 1.2e-09
Orthonormal null-space function used.
Iter      SolEst     Damping    Stepsize #Res #Jac #Sol   LinErr   LinRes
   1     6.2e-07   0.5000000     6.2e-07    2    1    1  1.4e-15  1.9e-15
Solution error estimates for segregated groups
1.4, 0.54, 0.95, 3.1e-07
Residual error estimates for segregated groups
7.9e+03, 8.3e+04, 2.2e+04, 0.16
Segregated solver iteration 2.
Merged variables
Scales for dependent variables:
Concentration (comp1.Cl): 1.4e+02
Concentration (comp1.K): 1.4e+02
Electric potential (comp1.V): 0.064
Iter      SolEst     Damping    Stepsize #Res #Jac #Sol   LinErr   LinRes
   1        0.79   0.3500000        0.79    6    4    4  4.2e-14  2.7e-15
   2        0.77   0.3500000        0.77    7    5    5  4.1e-14  2.7e-15
   3        0.76   0.3500000        0.76    8    6    6  6.6e-14  2.7e-15
Velocity u2, Pressure p
Scales for dependent variables:
Pressure (comp1.p): 3.5e+02
Velocity field (spatial frame) (comp1.u2): 0.00071
Iter      SolEst     Damping    Stepsize #Res #Jac #Sol   LinErr   LinRes
   1        0.36   0.5000000        0.36    4    2    2  2.7e-15  7.8e-15
Displacement field
Iter      SolEst     Damping    Stepsize #Res #Jac #Sol   LinErr   LinRes
   1        0.95   0.5000000        0.95    4    2    2  1.9e-13  1.5e-12
Spatial mesh displacement
Iter      SolEst     Damping    Stepsize #Res #Jac #Sol   LinErr   LinRes
   1     9.2e-07   0.5000000     9.2e-07    4    2    2  1.4e-15  1.8e-15
Solution error estimates for segregated groups
1.3, 0.18, 0.48, 4.6e-07
Residual error estimates for segregated groups
8e+02, 1.3e+04, 2.2e+04, 0.18
Segregated solver iteration 3.
Merged variables
Iter      SolEst     Damping    Stepsize #Res #Jac #Sol   LinErr   LinRes
   1        0.75   0.3500000        0.75   10    7    7  6.9e-14  2.6e-15
   2        0.74   0.3500000        0.74   11    8    8  7.8e-14  2.7e-15
   3        0.72   0.3500000        0.72   12    9    9  8.6e-14  2.7e-15
Velocity u2, Pressure p
Iter      SolEst     Damping    Stepsize #Res #Jac #Sol   LinErr   LinRes
   1        0.15   0.5000000        0.15    6    3    3  3.5e-15  7.9e-15
Displacement field
Iter      SolEst     Damping    Stepsize #Res #Jac #Sol   LinErr   LinRes
   1        0.52   0.5000000        0.52    6    3    3  5.3e-14  1.5e-12
Spatial mesh displacement
Iter      SolEst     Damping    Stepsize #Res #Jac #Sol   LinErr   LinRes
   1     9.2e-07   0.5000000     9.2e-07    6    3    3  1.9e-15  1.9e-15
Solution error estimates for segregated groups
1.2, 0.077, 0.26, 4.6e-07
Residual error estimates for segregated groups
2.2e+02, 7.3e+03, 1.6e+04, 0.17
Segregated solver iteration 4.
```

```
Merged variables
Iter      SolEst     Damping    Stepsize #Res #Jac #Sol   LinErr   LinRes
   1         0.7   0.3500000         0.7   14   10   10    5e-14  2.8e-15
   2        0.68   0.3500000        0.68   15   11   11  5.6e-14  2.8e-15
   3         0.6   0.3500000         0.6   16   12   12  4.3e-14  2.6e-15
Velocity u2, Pressure p
Iter      SolEst     Damping    Stepsize #Res #Jac #Sol   LinErr   LinRes
   1       0.071   0.5000000       0.071    8    4    4  2.8e-15    8e-15
Displacement field
Iter      SolEst     Damping    Stepsize #Res #Jac #Sol   LinErr   LinRes
   1        0.29   0.5000000        0.29    8    4    4    1e-13  1.5e-12
Spatial mesh displacement
Iter      SolEst     Damping    Stepsize #Res #Jac #Sol   LinErr   LinRes
   1     7.7e-07   0.5000000     7.7e-07    8    4    4  1.3e-15  1.8e-15
Solution error estimates for segregated groups
1, 0.036, 0.15, 3.9e-07
Residual error estimates for segregated groups
85, 3.9e+03, 1.1e+04, 0.19
Segregated solver iteration 5.
Merged variables
Iter      SolEst     Damping    Stepsize #Res #Jac #Sol   LinErr   LinRes
   1        0.41   0.3500000        0.41   18   13   13  5.2e-14  2.7e-15
   2        0.28   0.3500000        0.28   19   14   14    6e-14  2.7e-15
   3        0.18   0.3500000        0.18   20   15   15  6.3e-14  2.7e-15
Velocity u2, Pressure p
Iter      SolEst     Damping    Stepsize #Res #Jac #Sol   LinErr   LinRes
   1       0.035   0.5000000       0.035   10    5    5  2.8e-15  7.9e-15
Displacement field
Iter      SolEst     Damping    Stepsize #Res #Jac #Sol   LinErr   LinRes
   1        0.17   0.5000000        0.17   10    5    5    1e-13  1.5e-12
Spatial mesh displacement
Iter      SolEst     Damping    Stepsize #Res #Jac #Sol   LinErr   LinRes
   1     5.8e-07   0.5000000     5.8e-07   10    5    5  1.4e-15  1.9e-15
Solution error estimates for segregated groups
0.31, 0.017, 0.084, 2.9e-07
Residual error estimates for segregated groups
33, 2e+03, 6.9e+03, 0.17
Segregated solver iteration 6.
Merged variables
Iter      SolEst     Damping    Stepsize #Res #Jac #Sol   LinErr   LinRes
   1        0.12   0.3500000        0.12   22   16   16  5.7e-14  2.8e-15
   2       0.079   0.3500000       0.079   23   17   17  1.5e-13  2.6e-15
   3       0.051   0.3500000       0.051   24   18   18    7e-14  2.7e-15
Velocity u2, Pressure p
Iter      SolEst     Damping    Stepsize #Res #Jac #Sol   LinErr   LinRes
   1       0.017   0.5000000       0.017   12    6    6  2.3e-15  7.8e-15
Displacement field
Iter      SolEst     Damping    Stepsize #Res #Jac #Sol   LinErr   LinRes
   1       0.095   0.5000000       0.095   12    6    6  7.7e-14  1.5e-12
Spatial mesh displacement
Iter      SolEst     Damping    Stepsize #Res #Jac #Sol   LinErr   LinRes
   1       4e-07   0.5000000       4e-07   12    6    6    2e-15  1.8e-15
Solution error estimates for segregated groups
0.088, 0.0085, 0.048, 2e-07
Residual error estimates for segregated groups
12, 1e+03, 4.2e+03, 0.17
```

```
Segregated solver iteration 7.
Merged variables
Iter      SolEst     Damping    Stepsize #Res #Jac #Sol   LinErr   LinRes
   1       0.034   0.3500000       0.034   26   19   19  4.8e-14  2.8e-15
   2       0.022   0.3500000       0.022   27   20   20  1.5e-13  2.7e-15
   3       0.014   0.3500000       0.014   28   21   21  5.2e-14  2.7e-15
Velocity u2, Pressure p
Iter      SolEst     Damping    Stepsize #Res #Jac #Sol   LinErr   LinRes
   1      0.0084   0.5000000      0.0084   14    7    7  4.4e-15    8e-15
Displacement field
Iter      SolEst     Damping    Stepsize #Res #Jac #Sol   LinErr   LinRes
   1       0.054   0.5000000       0.054   14    7    7  7.6e-14  1.5e-12
Spatial mesh displacement
Iter      SolEst     Damping    Stepsize #Res #Jac #Sol   LinErr   LinRes
   1     2.7e-07   0.5000000     2.7e-07   14    7    7  1.7e-15  1.8e-15
Solution error estimates for segregated groups
0.024, 0.0042, 0.027, 1.3e-07
Residual error estimates for segregated groups
4.1, 5e+02, 2.6e+03, 0.19
Segregated solver iteration 8.
Merged variables
Iter      SolEst     Damping    Stepsize #Res #Jac #Sol   LinErr   LinRes
   1      0.0093   0.3500000      0.0093   30   22   22  4.9e-14  2.7e-15
   2       0.006   0.3500000       0.006   31   23   23  1.1e-13  2.6e-15
   3      0.0039   0.3500000      0.0039   32   24   24  8.9e-14  2.7e-15
Velocity u2, Pressure p
Iter      SolEst     Damping    Stepsize #Res #Jac #Sol   LinErr   LinRes
   1      0.0042   0.5000000      0.0042   16    8    8  3.1e-15  7.7e-15
Displacement field
Iter      SolEst     Damping    Stepsize #Res #Jac #Sol   LinErr   LinRes
   1        0.03   0.5000000        0.03   16    8    8  5.9e-14  1.5e-12
Spatial mesh displacement
Iter      SolEst     Damping    Stepsize #Res #Jac #Sol   LinErr   LinRes
   1     1.7e-07   0.5000000     1.7e-07   16    8    8  1.8e-15  1.9e-15
Solution error estimates for segregated groups
0.0067, 0.0021, 0.015, 8.7e-08
Residual error estimates for segregated groups
1.4, 2.5e+02, 1.8e+03, 0.17
Segregated solver iteration 9.
Merged variables
Iter      SolEst     Damping    Stepsize #Res #Jac #Sol   LinErr   LinRes
   1      0.0025   0.3500000      0.0025   34   25   25  7.7e-14  2.7e-15
   2      0.0017   0.3500000      0.0017   35   26   26  9.5e-14  2.7e-15
   3      0.0011   0.3500000      0.0011   36   27   27  7.1e-14  2.7e-15
Velocity u2, Pressure p
Iter      SolEst     Damping    Stepsize #Res #Jac #Sol   LinErr   LinRes
   1      0.0021   0.5000000      0.0021   18    9    9  2.4e-15  7.8e-15
Displacement field
Iter      SolEst     Damping    Stepsize #Res #Jac #Sol   LinErr   LinRes
   1       0.017   0.5000000       0.017   18    9    9  9.6e-14  1.5e-12
Spatial mesh displacement
Iter      SolEst     Damping    Stepsize #Res #Jac #Sol   LinErr   LinRes
   1     1.1e-07   0.5000000     1.1e-07   18    9    9  1.9e-15  1.8e-15
Solution error estimates for segregated groups
0.0018, 0.001, 0.0085, 5.4e-08
Residual error estimates for segregated groups
```

```
0.44, 1.3e+02, 1.4e+03, 0.19
Segregated solver iteration 10.
Merged variables
Iter      SolEst     Damping    Stepsize #Res #Jac #Sol   LinErr   LinRes
   1      0.0007   0.3500000      0.0007   38   28   28  7.3e-14  2.7e-15
   2     0.00045   0.3500000     0.00045   39   29   29  3.8e-14  2.7e-15
   3      0.0003   0.3500000      0.0003   40   30   30  6.8e-14  2.7e-15
Velocity u2, Pressure p
Iter      SolEst     Damping    Stepsize #Res #Jac #Sol   LinErr   LinRes
   1       0.001   0.5000000       0.001   20   10   10  2.2e-15  7.9e-15
Displacement field
Iter      SolEst     Damping    Stepsize #Res #Jac #Sol   LinErr   LinRes
   1      0.0094   0.5000000      0.0094   20   10   10  1.2e-13  1.5e-12
Spatial mesh displacement
Iter      SolEst     Damping    Stepsize #Res #Jac #Sol   LinErr   LinRes
   1     6.6e-08   0.5000000     6.6e-08   20   10   10  1.4e-15  1.8e-15
Solution error estimates for segregated groups
0.00051, 0.00052, 0.0047, 3.3e-08
Residual error estimates for segregated groups
0.14, 63, 1.2e+03, 0.19
Segregated solver iteration 11.
Merged variables
Iter      SolEst     Damping    Stepsize #Res #Jac #Sol   LinErr   LinRes
   1     0.00019   0.3500000     0.00019   42   31   31  3.7e-14  2.7e-15
   2     0.00012   0.3500000     0.00012   43   32   32  6.9e-14  2.7e-15
   3     8.1e-05   0.3500000     8.1e-05   44   33   33  1.1e-13  2.7e-15
Velocity u2, Pressure p
Iter      SolEst     Damping    Stepsize #Res #Jac #Sol   LinErr   LinRes
   1     0.00052   0.5000000     0.00052   22   11   11  4.1e-15  7.9e-15
Displacement field
Iter      SolEst     Damping    Stepsize #Res #Jac #Sol   LinErr   LinRes
   1      0.0051   0.5000000      0.0051   22   11   11  3.8e-14  1.5e-12
Spatial mesh displacement
Iter      SolEst     Damping    Stepsize #Res #Jac #Sol   LinErr   LinRes
   1       4e-08   0.5000000       4e-08   22   11   11  1.2e-15  1.8e-15
Solution error estimates for segregated groups
0.00014, 0.00026, 0.0026, 2e-08
Residual error estimates for segregated groups
0.044, 32, 1.2e+03, 0.18
Segregated solver iteration 12.
Merged variables
Iter      SolEst     Damping    Stepsize #Res #Jac #Sol   LinErr   LinRes
   1     5.3e-05   0.3500000     5.3e-05   46   34   34  5.8e-14  2.8e-15
   2     3.4e-05   0.3500000     3.4e-05   47   35   35  1.4e-13  2.7e-15
   3     2.2e-05   0.3500000     2.2e-05   48   36   36  1.1e-13  2.6e-15
Velocity u2, Pressure p
Iter      SolEst     Damping    Stepsize #Res #Jac #Sol   LinErr   LinRes
   1     0.00026   0.5000000     0.00026   24   12   12  4.8e-15  7.9e-15
Displacement field
Iter      SolEst     Damping    Stepsize #Res #Jac #Sol   LinErr   LinRes
   1      0.0028   0.5000000      0.0028   24   12   12  1.2e-13  1.5e-12
Spatial mesh displacement
Iter      SolEst     Damping    Stepsize #Res #Jac #Sol   LinErr   LinRes
   1     2.3e-08   0.5000000     2.3e-08   24   12   12    2e-15  1.9e-15
Solution error estimates for segregated groups
3.8e-05, 0.00013, 0.0014, 1.2e-08
```

```
Residual error estimates for segregated groups
0.013, 16, 1.2e+03, 0.18
Segregated solver iteration 13.
Merged variables
Iter      SolEst     Damping    Stepsize #Res #Jac #Sol   LinErr   LinRes
   1     1.4e-05   0.3500000     1.4e-05   50   37   37  8.4e-14  2.7e-15
   2     9.4e-06   0.3500000     9.4e-06   51   38   38  9.8e-14  2.7e-15
   3     6.1e-06   0.3500000     6.1e-06   52   39   39  4.5e-14  2.7e-15
Velocity u2, Pressure p
Iter      SolEst     Damping    Stepsize #Res #Jac #Sol   LinErr   LinRes
   1     0.00013   0.5000000     0.00013   26   13   13  2.4e-15  7.9e-15
Displacement field
Iter      SolEst     Damping    Stepsize #Res #Jac #Sol   LinErr   LinRes
   1      0.0015   0.5000000      0.0015   26   13   13    4e-14  1.5e-12
Spatial mesh displacement
Iter      SolEst     Damping    Stepsize #Res #Jac #Sol   LinErr   LinRes
   1     1.4e-08   0.5000000     1.4e-08   26   13   13  1.7e-15  1.8e-15
Solution error estimates for segregated groups
1e-05, 6.5e-05, 0.00076, 6.8e-09
Residual error estimates for segregated groups
0.0041, 7.9, 1.2e+03, 0.18
Solution time: 156 s. (2 minutes, 36 seconds)
Physical memory: 4.66 GB
Virtual memory: 25.99 GB
Ended at Jan 31, 2025, 6:14:17 PM.
----- Stationary Solver 1 in study1/Approach Curve Solver (sol1) -------------->
```

d=2350 (su32)

GENERAL

Description	Value
Solution	d=2350

LOG

```
<---- Compile Equations: Stationary in study1/Approach Curve Solver (sol1) -----
Started at Jan 31, 2025, 6:14:17 PM.
Parameter d = 2.35E-6 (m).
Geometry shape function: Linear serendipity
Number of vertex elements: 18
Number of boundary elements: 6254
Number of elements: 156162
Minimum element quality: 0.4933
Time: 9 s.
Physical memory: 2.85 GB
Virtual memory: 15.18 GB
Ended at Jan 31, 2025, 6:14:25 PM.
----- Compile Equations: Stationary in study1/Approach Curve Solver (sol1) ---->

<---- Dependent Variables 1 in study1/Approach Curve Solver (sol1) -------------
Started at Jan 31, 2025, 6:14:25 PM.
Solution time: 0 s.
Physical memory: 2.85 GB
Virtual memory: 15.18 GB
Ended at Jan 31, 2025, 6:14:25 PM.
----- Dependent Variables 1 in study1/Approach Curve Solver (sol1) ------------>

<---- Stationary Solver 1 in study1/Approach Curve Solver (sol1) ---------------
Started at Jan 31, 2025, 6:14:25 PM.
Segregated solver
Segregated Step 11 does not have any components solved for.
Number of degrees of freedom solved for: 794201 (plus 11931 internal DOFs).
Segregated solver iteration 1.
Merged variables
Nonsymmetric matrix found.
Scales for dependent variables:
Concentration (comp1.Cl): 1.4e+02
Concentration (comp1.K): 1.4e+02
Electric potential (comp1.V): 1.9e+02
Orthonormal null-space function used.
Iter      SolEst     Damping    Stepsize #Res #Jac #Sol   LinErr   LinRes
   1        0.83   0.3500000        0.83    2    1    1  1.3e-11  2.7e-15
   2        0.81   0.3500000        0.81    3    2    2    3e-11  2.9e-15
   3         0.8   0.3500000         0.8    4    3    3  3.1e-11  2.8e-15
Velocity u2, Pressure p
Nonsymmetric matrix found.
Scales for dependent variables:
Pressure (comp1.p): 1
Velocity field (spatial frame) (comp1.u2): 0.00018
Orthonormal null-space function used.
Iter      SolEst     Damping    Stepsize #Res #Jac #Sol   LinErr   LinRes
   1         1.1   0.5000000         1.1    2    1    1  1.5e-15  2.3e-15
Displacement field
Nonsymmetric matrix found.
Scales for dependent variables:
Auxiliary pressure (comp1.solid.lemm1.pw): 1
Displacement field (comp1.u): 2.8e-18
Orthonormal null-space function used.
Iter      SolEst     Damping    Stepsize #Res #Jac #Sol   LinErr   LinRes
   1         1.9   0.5000000         1.9    2    1    1  1.6e-13  1.5e-12
Spatial mesh displacement
```

```
Symmetric matrices found.
Scales for dependent variables:
Spatial mesh displacement (comp1.spatial.disp): 1.2e-09
Orthonormal null-space function used.
Iter      SolEst     Damping    Stepsize #Res #Jac #Sol   LinErr   LinRes
   1     6.2e-07   0.5000000     6.2e-07    2    1    1  1.9e-15  1.9e-15
Solution error estimates for segregated groups
1.4, 0.54, 0.95, 3.1e-07
Residual error estimates for segregated groups
7.9e+03, 8.3e+04, 2.2e+04, 0.15
Segregated solver iteration 2.
Merged variables
Scales for dependent variables:
Concentration (comp1.Cl): 1.4e+02
Concentration (comp1.K): 1.4e+02
Electric potential (comp1.V): 0.064
Iter      SolEst     Damping    Stepsize #Res #Jac #Sol   LinErr   LinRes
   1        0.79   0.3500000        0.79    6    4    4  5.6e-14    3e-15
   2        0.77   0.3500000        0.77    7    5    5    1e-13  3.3e-15
   3        0.76   0.3500000        0.76    8    6    6  4.9e-14  2.9e-15
Velocity u2, Pressure p
Scales for dependent variables:
Pressure (comp1.p): 3.6e+02
Velocity field (spatial frame) (comp1.u2): 0.00072
Iter      SolEst     Damping    Stepsize #Res #Jac #Sol   LinErr   LinRes
   1        0.36   0.5000000        0.36    4    2    2  2.2e-15  7.9e-15
Displacement field
Iter      SolEst     Damping    Stepsize #Res #Jac #Sol   LinErr   LinRes
   1        0.95   0.5000000        0.95    4    2    2  1.6e-13  1.5e-12
Spatial mesh displacement
Iter      SolEst     Damping    Stepsize #Res #Jac #Sol   LinErr   LinRes
   1     9.2e-07   0.5000000     9.2e-07    4    2    2  1.7e-15  1.9e-15
Solution error estimates for segregated groups
1.3, 0.18, 0.48, 4.6e-07
Residual error estimates for segregated groups
8e+02, 1.3e+04, 2.2e+04, 0.18
Segregated solver iteration 3.
Merged variables
Iter      SolEst     Damping    Stepsize #Res #Jac #Sol   LinErr   LinRes
   1        0.75   0.3500000        0.75   10    7    7  6.1e-14  2.9e-15
   2        0.73   0.3500000        0.73   11    8    8  1.6e-13  2.9e-15
   3        0.72   0.3500000        0.72   12    9    9  1.3e-13  2.9e-15
Velocity u2, Pressure p
Iter      SolEst     Damping    Stepsize #Res #Jac #Sol   LinErr   LinRes
   1        0.15   0.5000000        0.15    6    3    3  5.4e-15    8e-15
Displacement field
Iter      SolEst     Damping    Stepsize #Res #Jac #Sol   LinErr   LinRes
   1        0.52   0.5000000        0.52    6    3    3  6.3e-14  1.5e-12
Spatial mesh displacement
Iter      SolEst     Damping    Stepsize #Res #Jac #Sol   LinErr   LinRes
   1     9.2e-07   0.5000000     9.2e-07    6    3    3  1.7e-15  1.8e-15
Solution error estimates for segregated groups
1.2, 0.077, 0.26, 4.6e-07
Residual error estimates for segregated groups
2.2e+02, 7.4e+03, 1.6e+04, 0.18
Segregated solver iteration 4.
```

```
Merged variables
Iter      SolEst     Damping    Stepsize #Res #Jac #Sol   LinErr   LinRes
   1         0.7   0.3500000         0.7   14   10   10  8.7e-14  3.2e-15
   2        0.68   0.3500000        0.68   15   11   11    7e-14  3.3e-15
   3         0.6   0.3500000         0.6   16   12   12  4.4e-14  2.8e-15
Velocity u2, Pressure p
Iter      SolEst     Damping    Stepsize #Res #Jac #Sol   LinErr   LinRes
   1       0.072   0.5000000       0.072    8    4    4    3e-15  7.9e-15
Displacement field
Iter      SolEst     Damping    Stepsize #Res #Jac #Sol   LinErr   LinRes
   1        0.29   0.5000000        0.29    8    4    4  1.1e-13  1.5e-12
Spatial mesh displacement
Iter      SolEst     Damping    Stepsize #Res #Jac #Sol   LinErr   LinRes
   1     7.7e-07   0.5000000     7.7e-07    8    4    4  1.4e-15  1.9e-15
Solution error estimates for segregated groups
1, 0.036, 0.15, 3.9e-07
Residual error estimates for segregated groups
85, 3.9e+03, 1.1e+04, 0.18
Segregated solver iteration 5.
Merged variables
Iter      SolEst     Damping    Stepsize #Res #Jac #Sol   LinErr   LinRes
   1        0.41   0.3500000        0.41   18   13   13  4.5e-14    3e-15
   2        0.28   0.3500000        0.28   19   14   14  1.4e-13  3.4e-15
   3        0.18   0.3500000        0.18   20   15   15    1e-13  2.8e-15
Velocity u2, Pressure p
Iter      SolEst     Damping    Stepsize #Res #Jac #Sol   LinErr   LinRes
   1       0.035   0.5000000       0.035   10    5    5  3.2e-15  7.8e-15
Displacement field
Iter      SolEst     Damping    Stepsize #Res #Jac #Sol   LinErr   LinRes
   1        0.17   0.5000000        0.17   10    5    5    6e-14  1.5e-12
Spatial mesh displacement
Iter      SolEst     Damping    Stepsize #Res #Jac #Sol   LinErr   LinRes
   1     5.8e-07   0.5000000     5.8e-07   10    5    5  1.4e-15  1.9e-15
Solution error estimates for segregated groups
0.31, 0.017, 0.084, 2.9e-07
Residual error estimates for segregated groups
33, 2e+03, 6.9e+03, 0.18
Segregated solver iteration 6.
Merged variables
Iter      SolEst     Damping    Stepsize #Res #Jac #Sol   LinErr   LinRes
   1        0.12   0.3500000        0.12   22   16   16  1.4e-13  3.3e-15
   2       0.079   0.3500000       0.079   23   17   17  8.8e-14  2.9e-15
   3       0.051   0.3500000       0.051   24   18   18  6.6e-14    3e-15
Velocity u2, Pressure p
Iter      SolEst     Damping    Stepsize #Res #Jac #Sol   LinErr   LinRes
   1       0.017   0.5000000       0.017   12    6    6  2.2e-15  7.9e-15
Displacement field
Iter      SolEst     Damping    Stepsize #Res #Jac #Sol   LinErr   LinRes
   1       0.095   0.5000000       0.095   12    6    6  5.1e-14  1.5e-12
Spatial mesh displacement
Iter      SolEst     Damping    Stepsize #Res #Jac #Sol   LinErr   LinRes
   1       4e-07   0.5000000       4e-07   12    6    6  1.9e-15  1.9e-15
Solution error estimates for segregated groups
0.088, 0.0085, 0.048, 2e-07
Residual error estimates for segregated groups
12, 1e+03, 4.2e+03, 0.18
```

```
Segregated solver iteration 7.
Merged variables
Iter      SolEst     Damping    Stepsize #Res #Jac #Sol   LinErr   LinRes
   1       0.034   0.3500000       0.034   26   19   19  7.5e-14  2.8e-15
   2       0.022   0.3500000       0.022   27   20   20  6.5e-14  3.2e-15
   3       0.014   0.3500000       0.014   28   21   21  3.9e-14  3.2e-15
Velocity u2, Pressure p
Iter      SolEst     Damping    Stepsize #Res #Jac #Sol   LinErr   LinRes
   1      0.0084   0.5000000      0.0084   14    7    7    5e-15  7.9e-15
Displacement field
Iter      SolEst     Damping    Stepsize #Res #Jac #Sol   LinErr   LinRes
   1       0.054   0.5000000       0.054   14    7    7  1.9e-13  1.5e-12
Spatial mesh displacement
Iter      SolEst     Damping    Stepsize #Res #Jac #Sol   LinErr   LinRes
   1     2.7e-07   0.5000000     2.7e-07   14    7    7  1.8e-15  1.9e-15
Solution error estimates for segregated groups
0.024, 0.0042, 0.027, 1.3e-07
Residual error estimates for segregated groups
4.1, 5.1e+02, 2.6e+03, 0.18
Segregated solver iteration 8.
Merged variables
Iter      SolEst     Damping    Stepsize #Res #Jac #Sol   LinErr   LinRes
   1      0.0092   0.3500000      0.0092   30   22   22  6.3e-14  2.8e-15
   2       0.006   0.3500000       0.006   31   23   23  9.2e-14    3e-15
   3      0.0039   0.3500000      0.0039   32   24   24  1.2e-13  3.2e-15
Velocity u2, Pressure p
Iter      SolEst     Damping    Stepsize #Res #Jac #Sol   LinErr   LinRes
   1      0.0042   0.5000000      0.0042   16    8    8  3.9e-15  7.8e-15
Displacement field
Iter      SolEst     Damping    Stepsize #Res #Jac #Sol   LinErr   LinRes
   1        0.03   0.5000000        0.03   16    8    8  2.2e-13  1.5e-12
Spatial mesh displacement
Iter      SolEst     Damping    Stepsize #Res #Jac #Sol   LinErr   LinRes
   1     1.7e-07   0.5000000     1.7e-07   16    8    8  2.1e-15  1.9e-15
Solution error estimates for segregated groups
0.0067, 0.0021, 0.015, 8.7e-08
Residual error estimates for segregated groups
1.4, 2.6e+02, 1.8e+03, 0.17
Segregated solver iteration 9.
Merged variables
Iter      SolEst     Damping    Stepsize #Res #Jac #Sol   LinErr   LinRes
   1      0.0025   0.3500000      0.0025   34   25   25  6.3e-14    3e-15
   2      0.0017   0.3500000      0.0017   35   26   26  4.6e-14    3e-15
   3      0.0011   0.3500000      0.0011   36   27   27  5.9e-14  3.1e-15
Velocity u2, Pressure p
Iter      SolEst     Damping    Stepsize #Res #Jac #Sol   LinErr   LinRes
   1      0.0021   0.5000000      0.0021   18    9    9  4.6e-15  7.9e-15
Displacement field
Iter      SolEst     Damping    Stepsize #Res #Jac #Sol   LinErr   LinRes
   1       0.017   0.5000000       0.017   18    9    9    3e-14  1.5e-12
Spatial mesh displacement
Iter      SolEst     Damping    Stepsize #Res #Jac #Sol   LinErr   LinRes
   1     1.1e-07   0.5000000     1.1e-07   18    9    9  1.5e-15  1.8e-15
Solution error estimates for segregated groups
0.0018, 0.001, 0.0085, 5.4e-08
Residual error estimates for segregated groups
```

```
0.44, 1.3e+02, 1.4e+03, 0.19
Segregated solver iteration 10.
Merged variables
Iter      SolEst     Damping    Stepsize #Res #Jac #Sol   LinErr   LinRes
   1      0.0007   0.3500000      0.0007   38   28   28  5.7e-14  3.2e-15
   2     0.00045   0.3500000     0.00045   39   29   29  8.6e-14    3e-15
   3      0.0003   0.3500000      0.0003   40   30   30  1.3e-13  3.2e-15
Velocity u2, Pressure p
Iter      SolEst     Damping    Stepsize #Res #Jac #Sol   LinErr   LinRes
   1       0.001   0.5000000       0.001   20   10   10  2.3e-15  7.8e-15
Displacement field
Iter      SolEst     Damping    Stepsize #Res #Jac #Sol   LinErr   LinRes
   1      0.0094   0.5000000      0.0094   20   10   10  5.2e-14  1.5e-12
Spatial mesh displacement
Iter      SolEst     Damping    Stepsize #Res #Jac #Sol   LinErr   LinRes
   1     6.6e-08   0.5000000     6.6e-08   20   10   10  1.5e-15  1.9e-15
Solution error estimates for segregated groups
0.00051, 0.00052, 0.0047, 3.3e-08
Residual error estimates for segregated groups
0.14, 64, 1.2e+03, 0.18
Segregated solver iteration 11.
Merged variables
Iter      SolEst     Damping    Stepsize #Res #Jac #Sol   LinErr   LinRes
   1     0.00019   0.3500000     0.00019   42   31   31  5.6e-14  3.1e-15
   2     0.00012   0.3500000     0.00012   43   32   32  6.3e-14  3.1e-15
   3     8.1e-05   0.3500000     8.1e-05   44   33   33    2e-13  3.3e-15
Velocity u2, Pressure p
Iter      SolEst     Damping    Stepsize #Res #Jac #Sol   LinErr   LinRes
   1     0.00052   0.5000000     0.00052   22   11   11  2.5e-15  7.8e-15
Displacement field
Iter      SolEst     Damping    Stepsize #Res #Jac #Sol   LinErr   LinRes
   1      0.0051   0.5000000      0.0051   22   11   11  1.9e-13  1.5e-12
Spatial mesh displacement
Iter      SolEst     Damping    Stepsize #Res #Jac #Sol   LinErr   LinRes
   1       4e-08   0.5000000       4e-08   22   11   11  2.8e-15  1.9e-15
Solution error estimates for segregated groups
0.00014, 0.00026, 0.0026, 2e-08
Residual error estimates for segregated groups
0.044, 32, 1.2e+03, 0.18
Segregated solver iteration 12.
Merged variables
Iter      SolEst     Damping    Stepsize #Res #Jac #Sol   LinErr   LinRes
   1     5.3e-05   0.3500000     5.3e-05   46   34   34  8.8e-14  3.3e-15
   2     3.4e-05   0.3500000     3.4e-05   47   35   35  5.1e-14  3.1e-15
   3     2.2e-05   0.3500000     2.2e-05   48   36   36  1.1e-13  3.2e-15
Velocity u2, Pressure p
Iter      SolEst     Damping    Stepsize #Res #Jac #Sol   LinErr   LinRes
   1     0.00026   0.5000000     0.00026   24   12   12  1.8e-15  8.1e-15
Displacement field
Iter      SolEst     Damping    Stepsize #Res #Jac #Sol   LinErr   LinRes
   1      0.0028   0.5000000      0.0028   24   12   12  1.9e-13  1.5e-12
Spatial mesh displacement
Iter      SolEst     Damping    Stepsize #Res #Jac #Sol   LinErr   LinRes
   1     2.3e-08   0.5000000     2.3e-08   24   12   12  1.3e-15  1.9e-15
Solution error estimates for segregated groups
3.8e-05, 0.00013, 0.0014, 1.2e-08
```

```
Residual error estimates for segregated groups
0.013, 16, 1.2e+03, 0.17
Segregated solver iteration 13.
Merged variables
Iter      SolEst     Damping    Stepsize #Res #Jac #Sol   LinErr   LinRes
   1     1.4e-05   0.3500000     1.4e-05   50   37   37  9.1e-14    3e-15
   2     9.4e-06   0.3500000     9.4e-06   51   38   38  6.1e-14  3.1e-15
   3     6.1e-06   0.3500000     6.1e-06   52   39   39  8.1e-14  3.1e-15
Velocity u2, Pressure p
Iter      SolEst     Damping    Stepsize #Res #Jac #Sol   LinErr   LinRes
   1     0.00013   0.5000000     0.00013   26   13   13  3.9e-15  7.8e-15
Displacement field
Iter      SolEst     Damping    Stepsize #Res #Jac #Sol   LinErr   LinRes
   1      0.0015   0.5000000      0.0015   26   13   13  3.5e-14  1.5e-12
Spatial mesh displacement
Iter      SolEst     Damping    Stepsize #Res #Jac #Sol   LinErr   LinRes
   1     1.4e-08   0.5000000     1.4e-08   26   13   13  1.5e-15  1.9e-15
Solution error estimates for segregated groups
1e-05, 6.5e-05, 0.00076, 6.8e-09
Residual error estimates for segregated groups
0.0041, 8.1, 1.2e+03, 0.18
Solution time: 158 s. (2 minutes, 38 seconds)
Physical memory: 4.67 GB
Virtual memory: 25.85 GB
Ended at Jan 31, 2025, 6:17:03 PM.
----- Stationary Solver 1 in study1/Approach Curve Solver (sol1) -------------->
```

d=2345 (su33)

GENERAL

Description	Value
Solution	d=2345

LOG

```
<---- Compile Equations: Stationary in study1/Approach Curve Solver (sol1) -----
Started at Jan 31, 2025, 6:17:03 PM.
Parameter d = 2.345E-6 (m).
Geometry shape function: Linear serendipity
Number of vertex elements: 18
Number of boundary elements: 6252
Number of elements: 156670
Minimum element quality: 0.4848
Time: 8 s.
Physical memory: 2.86 GB
Virtual memory: 15.18 GB
Ended at Jan 31, 2025, 6:17:12 PM.
----- Compile Equations: Stationary in study1/Approach Curve Solver (sol1) ---->

<---- Dependent Variables 1 in study1/Approach Curve Solver (sol1) -------------
Started at Jan 31, 2025, 6:17:12 PM.
Solution time: 0 s.
Physical memory: 2.86 GB
Virtual memory: 15.18 GB
Ended at Jan 31, 2025, 6:17:12 PM.
----- Dependent Variables 1 in study1/Approach Curve Solver (sol1) ------------>

<---- Stationary Solver 1 in study1/Approach Curve Solver (sol1) ---------------
Started at Jan 31, 2025, 6:17:12 PM.
Segregated solver
Segregated Step 11 does not have any components solved for.
Number of degrees of freedom solved for: 796518 (plus 11927 internal DOFs).
Segregated solver iteration 1.
Merged variables
Nonsymmetric matrix found.
Scales for dependent variables:
Concentration (comp1.Cl): 1.4e+02
Concentration (comp1.K): 1.4e+02
Electric potential (comp1.V): 1.9e+02
Orthonormal null-space function used.
Iter      SolEst     Damping    Stepsize #Res #Jac #Sol   LinErr   LinRes
   1        0.83   0.3500000        0.83    2    1    1  5.3e-11  2.7e-15
   2        0.81   0.3500000        0.81    3    2    2  6.5e-11  2.7e-15
   3         0.8   0.3500000         0.8    4    3    3  3.7e-11  2.7e-15
Velocity u2, Pressure p
Nonsymmetric matrix found.
Scales for dependent variables:
Pressure (comp1.p): 1
Velocity field (spatial frame) (comp1.u2): 0.00018
Orthonormal null-space function used.
Iter      SolEst     Damping    Stepsize #Res #Jac #Sol   LinErr   LinRes
   1         1.1   0.5000000         1.1    2    1    1  2.1e-15  2.3e-15
Displacement field
Nonsymmetric matrix found.
Scales for dependent variables:
Auxiliary pressure (comp1.solid.lemm1.pw): 1
Displacement field (comp1.u): 2.8e-18
Orthonormal null-space function used.
Iter      SolEst     Damping    Stepsize #Res #Jac #Sol   LinErr   LinRes
   1         1.9   0.5000000         1.9    2    1    1  4.2e-14  1.5e-12
Spatial mesh displacement
```

```
Symmetric matrices found.
Scales for dependent variables:
Spatial mesh displacement (comp1.spatial.disp): 1.2e-09
Orthonormal null-space function used.
Iter      SolEst     Damping    Stepsize #Res #Jac #Sol   LinErr   LinRes
   1     6.2e-07   0.5000000     6.2e-07    2    1    1  1.3e-15  1.8e-15
Solution error estimates for segregated groups
1.4, 0.54, 0.95, 3.1e-07
Residual error estimates for segregated groups
7.9e+03, 8.3e+04, 2.2e+04, 0.16
Segregated solver iteration 2.
Merged variables
Scales for dependent variables:
Concentration (comp1.Cl): 1.4e+02
Concentration (comp1.K): 1.4e+02
Electric potential (comp1.V): 0.064
Iter      SolEst     Damping    Stepsize #Res #Jac #Sol   LinErr   LinRes
   1        0.79   0.3500000        0.79    6    4    4    5e-14  2.7e-15
   2        0.77   0.3500000        0.77    7    5    5  7.3e-14  2.8e-15
   3        0.76   0.3500000        0.76    8    6    6  8.2e-14  2.9e-15
Velocity u2, Pressure p
Scales for dependent variables:
Pressure (comp1.p): 3.5e+02
Velocity field (spatial frame) (comp1.u2): 0.00071
Iter      SolEst     Damping    Stepsize #Res #Jac #Sol   LinErr   LinRes
   1        0.36   0.5000000        0.36    4    2    2  2.1e-15    8e-15
Displacement field
Iter      SolEst     Damping    Stepsize #Res #Jac #Sol   LinErr   LinRes
   1        0.95   0.5000000        0.95    4    2    2  6.4e-14  1.5e-12
Spatial mesh displacement
Iter      SolEst     Damping    Stepsize #Res #Jac #Sol   LinErr   LinRes
   1     9.3e-07   0.5000000     9.3e-07    4    2    2  1.7e-15  1.8e-15
Solution error estimates for segregated groups
1.3, 0.18, 0.48, 4.7e-07
Residual error estimates for segregated groups
8e+02, 1.6e+04, 2.2e+04, 0.18
Segregated solver iteration 3.
Merged variables
Iter      SolEst     Damping    Stepsize #Res #Jac #Sol   LinErr   LinRes
   1        0.75   0.3500000        0.75   10    7    7  9.2e-14  2.8e-15
   2        0.74   0.3500000        0.74   11    8    8  7.9e-14  2.8e-15
   3        0.72   0.3500000        0.72   12    9    9  3.7e-14  2.8e-15
Velocity u2, Pressure p
Iter      SolEst     Damping    Stepsize #Res #Jac #Sol   LinErr   LinRes
   1        0.15   0.5000000        0.15    6    3    3    3e-15  7.8e-15
Displacement field
Iter      SolEst     Damping    Stepsize #Res #Jac #Sol   LinErr   LinRes
   1        0.52   0.5000000        0.52    6    3    3    6e-14  1.5e-12
Spatial mesh displacement
Iter      SolEst     Damping    Stepsize #Res #Jac #Sol   LinErr   LinRes
   1     9.3e-07   0.5000000     9.3e-07    6    3    3  1.5e-15  1.8e-15
Solution error estimates for segregated groups
1.2, 0.077, 0.26, 4.7e-07
Residual error estimates for segregated groups
2.2e+02, 8.8e+03, 1.6e+04, 0.17
Segregated solver iteration 4.
```

```
Merged variables
Iter      SolEst     Damping    Stepsize #Res #Jac #Sol   LinErr   LinRes
   1         0.7   0.3500000         0.7   14   10   10  7.8e-14  2.8e-15
   2        0.68   0.3500000        0.68   15   11   11  6.6e-14  2.8e-15
   3         0.6   0.3500000         0.6   16   12   12  6.3e-14  2.8e-15
Velocity u2, Pressure p
Iter      SolEst     Damping    Stepsize #Res #Jac #Sol   LinErr   LinRes
   1       0.071   0.5000000       0.071    8    4    4  2.7e-15  7.9e-15
Displacement field
Iter      SolEst     Damping    Stepsize #Res #Jac #Sol   LinErr   LinRes
   1        0.29   0.5000000        0.29    8    4    4  4.5e-14  1.5e-12
Spatial mesh displacement
Iter      SolEst     Damping    Stepsize #Res #Jac #Sol   LinErr   LinRes
   1     7.8e-07   0.5000000     7.8e-07    8    4    4  1.4e-15  1.9e-15
Solution error estimates for segregated groups
1, 0.036, 0.15, 3.9e-07
Residual error estimates for segregated groups
84, 4.6e+03, 1.1e+04, 0.17
Segregated solver iteration 5.
Merged variables
Iter      SolEst     Damping    Stepsize #Res #Jac #Sol   LinErr   LinRes
   1        0.41   0.3500000        0.41   18   13   13  7.1e-14  2.7e-15
   2        0.28   0.3500000        0.28   19   14   14  5.8e-14  2.8e-15
   3        0.18   0.3500000        0.18   20   15   15  1.3e-13  2.7e-15
Velocity u2, Pressure p
Iter      SolEst     Damping    Stepsize #Res #Jac #Sol   LinErr   LinRes
   1       0.035   0.5000000       0.035   10    5    5  3.1e-15  7.9e-15
Displacement field
Iter      SolEst     Damping    Stepsize #Res #Jac #Sol   LinErr   LinRes
   1        0.17   0.5000000        0.17   10    5    5  1.2e-13  1.5e-12
Spatial mesh displacement
Iter      SolEst     Damping    Stepsize #Res #Jac #Sol   LinErr   LinRes
   1     5.8e-07   0.5000000     5.8e-07   10    5    5  1.7e-15  1.9e-15
Solution error estimates for segregated groups
0.32, 0.017, 0.084, 2.9e-07
Residual error estimates for segregated groups
33, 2.4e+03, 6.9e+03, 0.17
Segregated solver iteration 6.
Merged variables
Iter      SolEst     Damping    Stepsize #Res #Jac #Sol   LinErr   LinRes
   1        0.12   0.3500000        0.12   22   16   16  1.6e-13  2.8e-15
   2       0.079   0.3500000       0.079   23   17   17  4.7e-14  2.7e-15
   3       0.052   0.3500000       0.052   24   18   18  6.1e-14  2.9e-15
Velocity u2, Pressure p
Iter      SolEst     Damping    Stepsize #Res #Jac #Sol   LinErr   LinRes
   1       0.017   0.5000000       0.017   12    6    6  2.3e-15    8e-15
Displacement field
Iter      SolEst     Damping    Stepsize #Res #Jac #Sol   LinErr   LinRes
   1       0.095   0.5000000       0.095   12    6    6  1.6e-13  1.5e-12
Spatial mesh displacement
Iter      SolEst     Damping    Stepsize #Res #Jac #Sol   LinErr   LinRes
   1     4.1e-07   0.5000000     4.1e-07   12    6    6  1.6e-15  1.8e-15
Solution error estimates for segregated groups
0.088, 0.0085, 0.048, 2e-07
Residual error estimates for segregated groups
12, 1.2e+03, 4.2e+03, 0.18
```

```
Segregated solver iteration 7.
Merged variables
Iter      SolEst     Damping    Stepsize #Res #Jac #Sol   LinErr   LinRes
   1       0.034   0.3500000       0.034   26   19   19  9.1e-14  2.7e-15
   2       0.022   0.3500000       0.022   27   20   20    4e-14  2.7e-15
   3       0.014   0.3500000       0.014   28   21   21  6.6e-14  2.9e-15
Velocity u2, Pressure p
Iter      SolEst     Damping    Stepsize #Res #Jac #Sol   LinErr   LinRes
   1      0.0084   0.5000000      0.0084   14    7    7  2.2e-15  7.8e-15
Displacement field
Iter      SolEst     Damping    Stepsize #Res #Jac #Sol   LinErr   LinRes
   1       0.054   0.5000000       0.054   14    7    7    5e-14  1.5e-12
Spatial mesh displacement
Iter      SolEst     Damping    Stepsize #Res #Jac #Sol   LinErr   LinRes
   1     2.7e-07   0.5000000     2.7e-07   14    7    7  1.3e-15  1.9e-15
Solution error estimates for segregated groups
0.024, 0.0042, 0.027, 1.4e-07
Residual error estimates for segregated groups
4.1, 6.1e+02, 2.7e+03, 0.18
Segregated solver iteration 8.
Merged variables
Iter      SolEst     Damping    Stepsize #Res #Jac #Sol   LinErr   LinRes
   1      0.0093   0.3500000      0.0093   30   22   22  1.1e-13  2.7e-15
   2       0.006   0.3500000       0.006   31   23   23  5.1e-14  2.8e-15
   3      0.0039   0.3500000      0.0039   32   24   24  8.1e-14  2.7e-15
Velocity u2, Pressure p
Iter      SolEst     Damping    Stepsize #Res #Jac #Sol   LinErr   LinRes
   1      0.0042   0.5000000      0.0042   16    8    8  4.5e-15  7.9e-15
Displacement field
Iter      SolEst     Damping    Stepsize #Res #Jac #Sol   LinErr   LinRes
   1        0.03   0.5000000        0.03   16    8    8  7.2e-14  1.5e-12
Spatial mesh displacement
Iter      SolEst     Damping    Stepsize #Res #Jac #Sol   LinErr   LinRes
   1     1.7e-07   0.5000000     1.7e-07   16    8    8  1.5e-15  1.9e-15
Solution error estimates for segregated groups
0.0067, 0.0021, 0.015, 8.7e-08
Residual error estimates for segregated groups
1.4, 3.1e+02, 1.8e+03, 0.18
Segregated solver iteration 9.
Merged variables
Iter      SolEst     Damping    Stepsize #Res #Jac #Sol   LinErr   LinRes
   1      0.0025   0.3500000      0.0025   34   25   25  1.1e-13  2.7e-15
   2      0.0017   0.3500000      0.0017   35   26   26  1.2e-13  2.8e-15
   3      0.0011   0.3500000      0.0011   36   27   27  8.3e-14  2.8e-15
Velocity u2, Pressure p
Iter      SolEst     Damping    Stepsize #Res #Jac #Sol   LinErr   LinRes
   1      0.0021   0.5000000      0.0021   18    9    9  3.4e-15  7.9e-15
Displacement field
Iter      SolEst     Damping    Stepsize #Res #Jac #Sol   LinErr   LinRes
   1       0.017   0.5000000       0.017   18    9    9    4e-14  1.5e-12
Spatial mesh displacement
Iter      SolEst     Damping    Stepsize #Res #Jac #Sol   LinErr   LinRes
   1     1.1e-07   0.5000000     1.1e-07   18    9    9  1.7e-15  1.8e-15
Solution error estimates for segregated groups
0.0018, 0.001, 0.0085, 5.5e-08
Residual error estimates for segregated groups
```

```
0.44, 1.5e+02, 1.4e+03, 0.17
Segregated solver iteration 10.
Merged variables
Iter      SolEst     Damping    Stepsize #Res #Jac #Sol   LinErr   LinRes
   1      0.0007   0.3500000      0.0007   38   28   28  5.6e-14  2.8e-15
   2     0.00045   0.3500000     0.00045   39   29   29  5.8e-14  2.8e-15
   3      0.0003   0.3500000      0.0003   40   30   30  1.4e-13  2.7e-15
Velocity u2, Pressure p
Iter      SolEst     Damping    Stepsize #Res #Jac #Sol   LinErr   LinRes
   1       0.001   0.5000000       0.001   20   10   10  5.1e-15  7.7e-15
Displacement field
Iter      SolEst     Damping    Stepsize #Res #Jac #Sol   LinErr   LinRes
   1      0.0094   0.5000000      0.0094   20   10   10  9.9e-14  1.5e-12
Spatial mesh displacement
Iter      SolEst     Damping    Stepsize #Res #Jac #Sol   LinErr   LinRes
   1     6.7e-08   0.5000000     6.7e-08   20   10   10  1.6e-15  1.8e-15
Solution error estimates for segregated groups
0.00051, 0.00052, 0.0047, 3.3e-08
Residual error estimates for segregated groups
0.14, 77, 1.2e+03, 0.17
Segregated solver iteration 11.
Merged variables
Iter      SolEst     Damping    Stepsize #Res #Jac #Sol   LinErr   LinRes
   1     0.00019   0.3500000     0.00019   42   31   31  6.6e-14  2.8e-15
   2     0.00012   0.3500000     0.00012   43   32   32  6.7e-14  2.9e-15
   3     8.1e-05   0.3500000     8.1e-05   44   33   33  6.2e-14  2.7e-15
Velocity u2, Pressure p
Iter      SolEst     Damping    Stepsize #Res #Jac #Sol   LinErr   LinRes
   1     0.00052   0.5000000     0.00052   22   11   11  3.4e-15  7.9e-15
Displacement field
Iter      SolEst     Damping    Stepsize #Res #Jac #Sol   LinErr   LinRes
   1      0.0051   0.5000000      0.0051   22   11   11  6.7e-14  1.5e-12
Spatial mesh displacement
Iter      SolEst     Damping    Stepsize #Res #Jac #Sol   LinErr   LinRes
   1       4e-08   0.5000000       4e-08   22   11   11  1.8e-15  1.9e-15
Solution error estimates for segregated groups
0.00014, 0.00026, 0.0026, 2e-08
Residual error estimates for segregated groups
0.044, 38, 1.2e+03, 0.17
Segregated solver iteration 12.
Merged variables
Iter      SolEst     Damping    Stepsize #Res #Jac #Sol   LinErr   LinRes
   1     5.3e-05   0.3500000     5.3e-05   46   34   34  6.7e-14  2.8e-15
   2     3.4e-05   0.3500000     3.4e-05   47   35   35    7e-14  2.7e-15
   3     2.2e-05   0.3500000     2.2e-05   48   36   36  9.6e-14  2.6e-15
Velocity u2, Pressure p
Iter      SolEst     Damping    Stepsize #Res #Jac #Sol   LinErr   LinRes
   1     0.00026   0.5000000     0.00026   24   12   12  2.8e-15  7.9e-15
Displacement field
Iter      SolEst     Damping    Stepsize #Res #Jac #Sol   LinErr   LinRes
   1      0.0028   0.5000000      0.0028   24   12   12  5.2e-14  1.5e-12
Spatial mesh displacement
Iter      SolEst     Damping    Stepsize #Res #Jac #Sol   LinErr   LinRes
   1     2.4e-08   0.5000000     2.4e-08   24   12   12  1.3e-15  1.8e-15
Solution error estimates for segregated groups
3.8e-05, 0.00013, 0.0014, 1.2e-08
```

```
Residual error estimates for segregated groups
0.013, 19, 1.2e+03, 0.17
Segregated solver iteration 13.
Merged variables
Iter      SolEst     Damping    Stepsize #Res #Jac #Sol   LinErr   LinRes
   1      1.4e-05   0.3500000    1.4e-05   50   37   37  9.4e-14  2.7e-15
   2      9.4e-06   0.3500000    9.4e-06   51   38   38  4.2e-14  2.8e-15
   3      6.1e-06   0.3500000    6.1e-06   52   39   39  8.2e-14  2.9e-15
Velocity u2, Pressure p
Iter      SolEst     Damping    Stepsize #Res #Jac #Sol   LinErr   LinRes
   1      0.00013   0.5000000    0.00013   26   13   13  3.3e-15  7.9e-15
Displacement field
Iter      SolEst     Damping    Stepsize #Res #Jac #Sol   LinErr   LinRes
   1       0.0015   0.5000000     0.0015   26   13   13  7.5e-14  1.5e-12
Spatial mesh displacement
Iter      SolEst     Damping    Stepsize #Res #Jac #Sol   LinErr   LinRes
   1      1.4e-08   0.5000000    1.4e-08   26   13   13  1.8e-15  1.8e-15
Solution error estimates for segregated groups
1.1e-05, 6.5e-05, 0.00076, 6.9e-09
Residual error estimates for segregated groups
0.0041, 9.6, 1.2e+03, 0.18
Solution time: 154 s. (2 minutes, 34 seconds)
Physical memory: 4.68 GB
Virtual memory: 25.92 GB
Ended at Jan 31, 2025, 6:19:46 PM.
----- Stationary Solver 1 in study1/Approach Curve Solver (sol1) -------------->
```

d=2340 (su34)

GENERAL

Description	Value
Solution	d=2340

LOG

```
<---- Compile Equations: Stationary in study1/Approach Curve Solver (sol1) -----
Started at Jan 31, 2025, 6:19:46 PM.
Parameter d = 2.34E-6 (m).
Geometry shape function: Linear serendipity
Number of vertex elements: 18
Number of boundary elements: 6249
Number of elements: 156491
Minimum element quality: 0.466
Time: 9 s.
Physical memory: 2.82 GB
Virtual memory: 15.19 GB
Ended at Jan 31, 2025, 6:19:55 PM.
----- Compile Equations: Stationary in study1/Approach Curve Solver (sol1) ---->

<---- Dependent Variables 1 in study1/Approach Curve Solver (sol1) -------------
Started at Jan 31, 2025, 6:19:55 PM.
Solution time: 0 s.
Physical memory: 2.81 GB
Virtual memory: 15.13 GB
Ended at Jan 31, 2025, 6:19:55 PM.
----- Dependent Variables 1 in study1/Approach Curve Solver (sol1) ------------>

<---- Stationary Solver 1 in study1/Approach Curve Solver (sol1) ---------------
Started at Jan 31, 2025, 6:19:55 PM.
Segregated solver
Segregated Step 11 does not have any components solved for.
Number of degrees of freedom solved for: 795778 (plus 11921 internal DOFs).
Segregated solver iteration 1.
Merged variables
Nonsymmetric matrix found.
Scales for dependent variables:
Concentration (comp1.Cl): 1.4e+02
Concentration (comp1.K): 1.4e+02
Electric potential (comp1.V): 1.9e+02
Orthonormal null-space function used.
Iter      SolEst     Damping    Stepsize #Res #Jac #Sol   LinErr   LinRes
   1        0.83   0.3500000        0.83    2    1    1  1.6e-11  2.8e-15
   2        0.81   0.3500000        0.81    3    2    2    4e-11  2.7e-15
   3         0.8   0.3500000         0.8    4    3    3  3.5e-11  2.7e-15
Velocity u2, Pressure p
Nonsymmetric matrix found.
Scales for dependent variables:
Pressure (comp1.p): 1
Velocity field (spatial frame) (comp1.u2): 0.00018
Orthonormal null-space function used.
Iter      SolEst     Damping    Stepsize #Res #Jac #Sol   LinErr   LinRes
   1         1.1   0.5000000         1.1    2    1    1  1.8e-15  2.3e-15
Displacement field
Nonsymmetric matrix found.
Scales for dependent variables:
Auxiliary pressure (comp1.solid.lemm1.pw): 1
Displacement field (comp1.u): 2.8e-18
Orthonormal null-space function used.
Iter      SolEst     Damping    Stepsize #Res #Jac #Sol   LinErr   LinRes
   1         1.9   0.5000000         1.9    2    1    1  2.8e-14  1.5e-12
Spatial mesh displacement
```

```
Symmetric matrices found.
Scales for dependent variables:
Spatial mesh displacement (comp1.spatial.disp): 1.2e-09
Orthonormal null-space function used.
Iter      SolEst     Damping    Stepsize #Res #Jac #Sol   LinErr   LinRes
   1     6.2e-07   0.5000000     6.2e-07    2    1    1  1.4e-15  1.8e-15
Solution error estimates for segregated groups
1.4, 0.54, 0.95, 3.1e-07
Residual error estimates for segregated groups
7.9e+03, 8.3e+04, 2.2e+04, 0.16
Segregated solver iteration 2.
Merged variables
Scales for dependent variables:
Concentration (comp1.Cl): 1.4e+02
Concentration (comp1.K): 1.4e+02
Electric potential (comp1.V): 0.064
Iter      SolEst     Damping    Stepsize #Res #Jac #Sol   LinErr   LinRes
   1        0.79   0.3500000        0.79    6    4    4  5.8e-14  2.8e-15
   2        0.77   0.3500000        0.77    7    5    5  5.7e-14  2.7e-15
   3        0.76   0.3500000        0.76    8    6    6  6.1e-14  2.8e-15
Velocity u2, Pressure p
Scales for dependent variables:
Pressure (comp1.p): 3.5e+02
Velocity field (spatial frame) (comp1.u2): 0.00071
Iter      SolEst     Damping    Stepsize #Res #Jac #Sol   LinErr   LinRes
   1        0.36   0.5000000        0.36    4    2    2  2.4e-15  7.9e-15
Displacement field
Iter      SolEst     Damping    Stepsize #Res #Jac #Sol   LinErr   LinRes
   1        0.95   0.5000000        0.95    4    2    2  8.3e-14  1.5e-12
Spatial mesh displacement
Iter      SolEst     Damping    Stepsize #Res #Jac #Sol   LinErr   LinRes
   1     9.3e-07   0.5000000     9.3e-07    4    2    2  1.5e-15  1.8e-15
Solution error estimates for segregated groups
1.3, 0.18, 0.48, 4.6e-07
Residual error estimates for segregated groups
8e+02, 1.3e+04, 2.2e+04, 0.19
Segregated solver iteration 3.
Merged variables
Iter      SolEst     Damping    Stepsize #Res #Jac #Sol   LinErr   LinRes
   1        0.75   0.3500000        0.75   10    7    7  1.2e-13  2.6e-15
   2        0.74   0.3500000        0.74   11    8    8  8.3e-14  2.8e-15
   3        0.72   0.3500000        0.72   12    9    9  4.3e-14  2.8e-15
Velocity u2, Pressure p
Iter      SolEst     Damping    Stepsize #Res #Jac #Sol   LinErr   LinRes
   1        0.15   0.5000000        0.15    6    3    3  2.5e-15  7.9e-15
Displacement field
Iter      SolEst     Damping    Stepsize #Res #Jac #Sol   LinErr   LinRes
   1        0.52   0.5000000        0.52    6    3    3  5.8e-14  1.5e-12
Spatial mesh displacement
Iter      SolEst     Damping    Stepsize #Res #Jac #Sol   LinErr   LinRes
   1     9.3e-07   0.5000000     9.3e-07    6    3    3  1.6e-15  1.9e-15
Solution error estimates for segregated groups
1.2, 0.077, 0.26, 4.6e-07
Residual error estimates for segregated groups
2.2e+02, 7.3e+03, 1.6e+04, 0.18
Segregated solver iteration 4.
```

```
Merged variables
Iter      SolEst     Damping    Stepsize #Res #Jac #Sol   LinErr   LinRes
   1         0.7   0.3500000         0.7   14   10   10  1.2e-13  2.8e-15
   2        0.68   0.3500000        0.68   15   11   11  4.8e-14  2.8e-15
   3         0.6   0.3500000         0.6   16   12   12  9.7e-14  2.9e-15
Velocity u2, Pressure p
Iter      SolEst     Damping    Stepsize #Res #Jac #Sol   LinErr   LinRes
   1       0.071   0.5000000       0.071    8    4    4  2.9e-15  7.7e-15
Displacement field
Iter      SolEst     Damping    Stepsize #Res #Jac #Sol   LinErr   LinRes
   1        0.29   0.5000000        0.29    8    4    4  5.6e-14  1.5e-12
Spatial mesh displacement
Iter      SolEst     Damping    Stepsize #Res #Jac #Sol   LinErr   LinRes
   1     7.7e-07   0.5000000     7.7e-07    8    4    4  2.6e-15  1.8e-15
Solution error estimates for segregated groups
1, 0.036, 0.15, 3.9e-07
Residual error estimates for segregated groups
84, 3.9e+03, 1.1e+04, 0.17
Segregated solver iteration 5.
Merged variables
Iter      SolEst     Damping    Stepsize #Res #Jac #Sol   LinErr   LinRes
   1        0.41   0.3500000        0.41   18   13   13  6.6e-14  2.8e-15
   2        0.28   0.3500000        0.28   19   14   14  9.7e-14  2.7e-15
   3        0.18   0.3500000        0.18   20   15   15  4.9e-14  2.7e-15
Velocity u2, Pressure p
Iter      SolEst     Damping    Stepsize #Res #Jac #Sol   LinErr   LinRes
   1       0.035   0.5000000       0.035   10    5    5  2.5e-15  7.9e-15
Displacement field
Iter      SolEst     Damping    Stepsize #Res #Jac #Sol   LinErr   LinRes
   1        0.17   0.5000000        0.17   10    5    5  1.4e-13  1.5e-12
Spatial mesh displacement
Iter      SolEst     Damping    Stepsize #Res #Jac #Sol   LinErr   LinRes
   1     5.8e-07   0.5000000     5.8e-07   10    5    5    2e-15  1.9e-15
Solution error estimates for segregated groups
0.32, 0.017, 0.084, 2.9e-07
Residual error estimates for segregated groups
33, 2e+03, 6.9e+03, 0.19
Segregated solver iteration 6.
Merged variables
Iter      SolEst     Damping    Stepsize #Res #Jac #Sol   LinErr   LinRes
   1        0.12   0.3500000        0.12   22   16   16  5.5e-14  2.8e-15
   2       0.079   0.3500000       0.079   23   17   17  8.3e-14  2.8e-15
   3       0.052   0.3500000       0.052   24   18   18  5.8e-14  2.9e-15
Velocity u2, Pressure p
Iter      SolEst     Damping    Stepsize #Res #Jac #Sol   LinErr   LinRes
   1       0.017   0.5000000       0.017   12    6    6  2.7e-15  7.9e-15
Displacement field
Iter      SolEst     Damping    Stepsize #Res #Jac #Sol   LinErr   LinRes
   1       0.095   0.5000000       0.095   12    6    6  1.3e-13  1.5e-12
Spatial mesh displacement
Iter      SolEst     Damping    Stepsize #Res #Jac #Sol   LinErr   LinRes
   1     4.1e-07   0.5000000     4.1e-07   12    6    6  1.4e-15  1.8e-15
Solution error estimates for segregated groups
0.088, 0.0085, 0.048, 2e-07
Residual error estimates for segregated groups
12, 1e+03, 4.2e+03, 0.19
```

```
Segregated solver iteration 7.
Merged variables
Iter      SolEst     Damping    Stepsize #Res #Jac #Sol   LinErr   LinRes
   1       0.034   0.3500000       0.034   26   19   19  1.1e-13  2.9e-15
   2       0.022   0.3500000       0.022   27   20   20  7.2e-14  2.7e-15
   3       0.014   0.3500000       0.014   28   21   21  1.2e-13  2.8e-15
Velocity u2, Pressure p
Iter      SolEst     Damping    Stepsize #Res #Jac #Sol   LinErr   LinRes
   1      0.0084   0.5000000      0.0084   14    7    7  2.2e-15  7.9e-15
Displacement field
Iter      SolEst     Damping    Stepsize #Res #Jac #Sol   LinErr   LinRes
   1       0.054   0.5000000       0.054   14    7    7  4.7e-14  1.5e-12
Spatial mesh displacement
Iter      SolEst     Damping    Stepsize #Res #Jac #Sol   LinErr   LinRes
   1     2.7e-07   0.5000000     2.7e-07   14    7    7  1.5e-15  1.8e-15
Solution error estimates for segregated groups
0.024, 0.0042, 0.027, 1.4e-07
Residual error estimates for segregated groups
4.1, 5.1e+02, 2.6e+03, 0.18
Segregated solver iteration 8.
Merged variables
Iter      SolEst     Damping    Stepsize #Res #Jac #Sol   LinErr   LinRes
   1      0.0093   0.3500000      0.0093   30   22   22  8.1e-14  2.8e-15
   2       0.006   0.3500000       0.006   31   23   23  3.6e-14  2.7e-15
   3      0.0039   0.3500000      0.0039   32   24   24  9.6e-14  2.8e-15
Velocity u2, Pressure p
Iter      SolEst     Damping    Stepsize #Res #Jac #Sol   LinErr   LinRes
   1      0.0042   0.5000000      0.0042   16    8    8  3.4e-15  7.8e-15
Displacement field
Iter      SolEst     Damping    Stepsize #Res #Jac #Sol   LinErr   LinRes
   1        0.03   0.5000000        0.03   16    8    8  5.1e-14  1.5e-12
Spatial mesh displacement
Iter      SolEst     Damping    Stepsize #Res #Jac #Sol   LinErr   LinRes
   1     1.7e-07   0.5000000     1.7e-07   16    8    8  1.6e-15  1.8e-15
Solution error estimates for segregated groups
0.0067, 0.0021, 0.015, 8.7e-08
Residual error estimates for segregated groups
1.4, 2.5e+02, 1.8e+03, 0.18
Segregated solver iteration 9.
Merged variables
Iter      SolEst     Damping    Stepsize #Res #Jac #Sol   LinErr   LinRes
   1      0.0025   0.3500000      0.0025   34   25   25  1.1e-13    3e-15
   2      0.0017   0.3500000      0.0017   35   26   26  8.2e-14  2.9e-15
   3      0.0011   0.3500000      0.0011   36   27   27  8.1e-14  2.7e-15
Velocity u2, Pressure p
Iter      SolEst     Damping    Stepsize #Res #Jac #Sol   LinErr   LinRes
   1      0.0021   0.5000000      0.0021   18    9    9  2.7e-15  7.8e-15
Displacement field
Iter      SolEst     Damping    Stepsize #Res #Jac #Sol   LinErr   LinRes
   1       0.017   0.5000000       0.017   18    9    9  1.3e-13  1.5e-12
Spatial mesh displacement
Iter      SolEst     Damping    Stepsize #Res #Jac #Sol   LinErr   LinRes
   1     1.1e-07   0.5000000     1.1e-07   18    9    9  1.7e-15  1.8e-15
Solution error estimates for segregated groups
0.0018, 0.001, 0.0085, 5.4e-08
Residual error estimates for segregated groups
```

```
0.44, 1.3e+02, 1.4e+03, 0.17
Segregated solver iteration 10.
Merged variables
Iter      SolEst     Damping    Stepsize #Res #Jac #Sol   LinErr   LinRes
   1      0.0007   0.3500000      0.0007   38   28   28  1.7e-13  2.8e-15
   2     0.00045   0.3500000     0.00045   39   29   29  8.3e-14  2.8e-15
   3      0.0003   0.3500000      0.0003   40   30   30  9.7e-14  2.9e-15
Velocity u2, Pressure p
Iter      SolEst     Damping    Stepsize #Res #Jac #Sol   LinErr   LinRes
   1       0.001   0.5000000       0.001   20   10   10  3.5e-15    8e-15
Displacement field
Iter      SolEst     Damping    Stepsize #Res #Jac #Sol   LinErr   LinRes
   1      0.0094   0.5000000      0.0094   20   10   10  3.8e-14  1.5e-12
Spatial mesh displacement
Iter      SolEst     Damping    Stepsize #Res #Jac #Sol   LinErr   LinRes
   1     6.6e-08   0.5000000     6.6e-08   20   10   10  1.7e-15  1.9e-15
Solution error estimates for segregated groups
0.00051, 0.00052, 0.0047, 3.3e-08
Residual error estimates for segregated groups
0.14, 64, 1.2e+03, 0.18
Segregated solver iteration 11.
Merged variables
Iter      SolEst     Damping    Stepsize #Res #Jac #Sol   LinErr   LinRes
   1     0.00019   0.3500000     0.00019   42   31   31  7.2e-14    3e-15
   2     0.00012   0.3500000     0.00012   43   32   32  1.1e-13  2.7e-15
   3     8.1e-05   0.3500000     8.1e-05   44   33   33  1.1e-13  2.9e-15
Velocity u2, Pressure p
Iter      SolEst     Damping    Stepsize #Res #Jac #Sol   LinErr   LinRes
   1     0.00052   0.5000000     0.00052   22   11   11  2.6e-15  7.9e-15
Displacement field
Iter      SolEst     Damping    Stepsize #Res #Jac #Sol   LinErr   LinRes
   1      0.0051   0.5000000      0.0051   22   11   11  2.3e-13  1.5e-12
Spatial mesh displacement
Iter      SolEst     Damping    Stepsize #Res #Jac #Sol   LinErr   LinRes
   1       4e-08   0.5000000       4e-08   22   11   11  1.7e-15  1.8e-15
Solution error estimates for segregated groups
0.00014, 0.00026, 0.0026, 2e-08
Residual error estimates for segregated groups
0.044, 32, 1.2e+03, 0.2
Segregated solver iteration 12.
Merged variables
Iter      SolEst     Damping    Stepsize #Res #Jac #Sol   LinErr   LinRes
   1     5.3e-05   0.3500000     5.3e-05   46   34   34  6.8e-14  2.9e-15
   2     3.4e-05   0.3500000     3.4e-05   47   35   35  4.8e-14  2.7e-15
   3     2.2e-05   0.3500000     2.2e-05   48   36   36    1e-13  2.6e-15
Velocity u2, Pressure p
Iter      SolEst     Damping    Stepsize #Res #Jac #Sol   LinErr   LinRes
   1     0.00026   0.5000000     0.00026   24   12   12  3.5e-15  7.9e-15
Displacement field
Iter      SolEst     Damping    Stepsize #Res #Jac #Sol   LinErr   LinRes
   1      0.0028   0.5000000      0.0028   24   12   12  1.1e-13  1.5e-12
Spatial mesh displacement
Iter      SolEst     Damping    Stepsize #Res #Jac #Sol   LinErr   LinRes
   1     2.4e-08   0.5000000     2.4e-08   24   12   12  1.4e-15  1.9e-15
Solution error estimates for segregated groups
3.8e-05, 0.00013, 0.0014, 1.2e-08
```

```
Residual error estimates for segregated groups
0.013, 16, 1.2e+03, 0.18
Segregated solver iteration 13.
Merged variables
Iter      SolEst     Damping    Stepsize #Res #Jac #Sol   LinErr   LinRes
   1     1.4e-05   0.3500000     1.4e-05   50   37   37  6.3e-14  2.8e-15
   2     9.4e-06   0.3500000     9.4e-06   51   38   38  6.1e-14  2.7e-15
   3     6.1e-06   0.3500000     6.1e-06   52   39   39  9.5e-14  2.8e-15
Velocity u2, Pressure p
Iter      SolEst     Damping    Stepsize #Res #Jac #Sol   LinErr   LinRes
   1     0.00013   0.5000000     0.00013   26   13   13    3e-15  7.8e-15
Displacement field
Iter      SolEst     Damping    Stepsize #Res #Jac #Sol   LinErr   LinRes
   1      0.0015   0.5000000      0.0015   26   13   13  6.1e-14  1.5e-12
Spatial mesh displacement
Iter      SolEst     Damping    Stepsize #Res #Jac #Sol   LinErr   LinRes
   1     1.4e-08   0.5000000     1.4e-08   26   13   13  1.6e-15  1.9e-15
Solution error estimates for segregated groups
1.1e-05, 6.5e-05, 0.00076, 6.9e-09
Residual error estimates for segregated groups
0.0041, 7.9, 1.2e+03, 0.18
Solution time: 156 s. (2 minutes, 36 seconds)
Physical memory: 4.59 GB
Virtual memory: 25.92 GB
Ended at Jan 31, 2025, 6:22:32 PM.
----- Stationary Solver 1 in study1/Approach Curve Solver (sol1) -------------->
```

d=2335 (su35)

GENERAL

Description	Value
Solution	d=2335

LOG

```
<---- Compile Equations: Stationary in study1/Approach Curve Solver (sol1) -----
Started at Jan 31, 2025, 6:22:32 PM.
Parameter d = 2.335E-6 (m).
Geometry shape function: Linear serendipity
Number of vertex elements: 18
Number of boundary elements: 6247
Number of elements: 156099
Minimum element quality: 0.4764
Time: 8 s.
Physical memory: 2.9 GB
Virtual memory: 15.22 GB
Ended at Jan 31, 2025, 6:22:40 PM.
----- Compile Equations: Stationary in study1/Approach Curve Solver (sol1) ---->

<---- Dependent Variables 1 in study1/Approach Curve Solver (sol1) -------------
Started at Jan 31, 2025, 6:22:40 PM.
Solution time: 0 s.
Physical memory: 2.9 GB
Virtual memory: 15.22 GB
Ended at Jan 31, 2025, 6:22:40 PM.
----- Dependent Variables 1 in study1/Approach Curve Solver (sol1) ------------>

<---- Stationary Solver 1 in study1/Approach Curve Solver (sol1) ---------------
Started at Jan 31, 2025, 6:22:41 PM.
Segregated solver
Segregated Step 11 does not have any components solved for.
Number of degrees of freedom solved for: 793741 (plus 11917 internal DOFs).
Segregated solver iteration 1.
Merged variables
Nonsymmetric matrix found.
Scales for dependent variables:
Concentration (comp1.Cl): 1.4e+02
Concentration (comp1.K): 1.4e+02
Electric potential (comp1.V): 1.9e+02
Orthonormal null-space function used.
Iter      SolEst     Damping    Stepsize #Res #Jac #Sol   LinErr   LinRes
   1        0.83   0.3500000        0.83    2    1    1  4.7e-11  2.8e-15
   2        0.81   0.3500000        0.81    3    2    2    2e-11  2.8e-15
   3         0.8   0.3500000         0.8    4    3    3  3.5e-11  2.8e-15
Velocity u2, Pressure p
Nonsymmetric matrix found.
Scales for dependent variables:
Pressure (comp1.p): 1
Velocity field (spatial frame) (comp1.u2): 0.00018
Orthonormal null-space function used.
Iter      SolEst     Damping    Stepsize #Res #Jac #Sol   LinErr   LinRes
   1         1.1   0.5000000         1.1    2    1    1  1.7e-15  2.3e-15
Displacement field
Nonsymmetric matrix found.
Scales for dependent variables:
Auxiliary pressure (comp1.solid.lemm1.pw): 1
Displacement field (comp1.u): 2.8e-18
Orthonormal null-space function used.
Iter      SolEst     Damping    Stepsize #Res #Jac #Sol   LinErr   LinRes
   1         1.9   0.5000000         1.9    2    1    1    4e-14  1.5e-12
Spatial mesh displacement
```

```
Symmetric matrices found.
Scales for dependent variables:
Spatial mesh displacement (comp1.spatial.disp): 1.2e-09
Orthonormal null-space function used.
Iter      SolEst     Damping    Stepsize #Res #Jac #Sol   LinErr   LinRes
   1     6.2e-07   0.5000000     6.2e-07    2    1    1  2.1e-15  1.9e-15
Solution error estimates for segregated groups
1.4, 0.54, 0.95, 3.1e-07
Residual error estimates for segregated groups
7.9e+03, 8.3e+04, 2.2e+04, 0.15
Segregated solver iteration 2.
Merged variables
Scales for dependent variables:
Concentration (comp1.Cl): 1.4e+02
Concentration (comp1.K): 1.4e+02
Electric potential (comp1.V): 0.064
Iter      SolEst     Damping    Stepsize #Res #Jac #Sol   LinErr   LinRes
   1        0.79   0.3500000        0.79    6    4    4  9.1e-14    3e-15
   2        0.77   0.3500000        0.77    7    5    5    5e-14    3e-15
   3        0.76   0.3500000        0.76    8    6    6  8.2e-14  2.9e-15
Velocity u2, Pressure p
Scales for dependent variables:
Pressure (comp1.p): 3.6e+02
Velocity field (spatial frame) (comp1.u2): 0.00071
Iter      SolEst     Damping    Stepsize #Res #Jac #Sol   LinErr   LinRes
   1        0.36   0.5000000        0.36    4    2    2    2e-15  7.8e-15
Displacement field
Iter      SolEst     Damping    Stepsize #Res #Jac #Sol   LinErr   LinRes
   1        0.95   0.5000000        0.95    4    2    2  1.7e-13  1.5e-12
Spatial mesh displacement
Iter      SolEst     Damping    Stepsize #Res #Jac #Sol   LinErr   LinRes
   1     9.4e-07   0.5000000     9.4e-07    4    2    2  1.5e-15  1.8e-15
Solution error estimates for segregated groups
1.3, 0.18, 0.48, 4.7e-07
Residual error estimates for segregated groups
8e+02, 1.4e+04, 2.2e+04, 0.17
Segregated solver iteration 3.
Merged variables
Iter      SolEst     Damping    Stepsize #Res #Jac #Sol   LinErr   LinRes
   1        0.75   0.3500000        0.75   10    7    7    4e-14    3e-15
   2        0.73   0.3500000        0.73   11    8    8  9.8e-14  3.5e-15
   3        0.72   0.3500000        0.72   12    9    9    7e-14    3e-15
Velocity u2, Pressure p
Iter      SolEst     Damping    Stepsize #Res #Jac #Sol   LinErr   LinRes
   1        0.15   0.5000000        0.15    6    3    3  1.8e-15  7.8e-15
Displacement field
Iter      SolEst     Damping    Stepsize #Res #Jac #Sol   LinErr   LinRes
   1        0.52   0.5000000        0.52    6    3    3  6.3e-14  1.5e-12
Spatial mesh displacement
Iter      SolEst     Damping    Stepsize #Res #Jac #Sol   LinErr   LinRes
   1     9.4e-07   0.5000000     9.4e-07    6    3    3  1.7e-15  1.8e-15
Solution error estimates for segregated groups
1.2, 0.077, 0.26, 4.7e-07
Residual error estimates for segregated groups
2.2e+02, 7.9e+03, 1.6e+04, 0.17
Segregated solver iteration 4.
```

```
Merged variables
Iter      SolEst     Damping    Stepsize #Res #Jac #Sol   LinErr   LinRes
   1         0.7   0.3500000         0.7   14   10   10  7.2e-14  4.3e-15
   2        0.68   0.3500000        0.68   15   11   11    5e-14  2.8e-15
   3         0.6   0.3500000         0.6   16   12   12  7.6e-14  3.6e-15
Velocity u2, Pressure p
Iter      SolEst     Damping    Stepsize #Res #Jac #Sol   LinErr   LinRes
   1       0.072   0.5000000       0.072    8    4    4  2.4e-15  7.8e-15
Displacement field
Iter      SolEst     Damping    Stepsize #Res #Jac #Sol   LinErr   LinRes
   1        0.29   0.5000000        0.29    8    4    4  5.5e-14  1.5e-12
Spatial mesh displacement
Iter      SolEst     Damping    Stepsize #Res #Jac #Sol   LinErr   LinRes
   1     7.8e-07   0.5000000     7.8e-07    8    4    4  2.4e-15  1.8e-15
Solution error estimates for segregated groups
1, 0.036, 0.15, 3.9e-07
Residual error estimates for segregated groups
84, 4.1e+03, 1.1e+04, 0.17
Segregated solver iteration 5.
Merged variables
Iter      SolEst     Damping    Stepsize #Res #Jac #Sol   LinErr   LinRes
   1        0.41   0.3500000        0.41   18   13   13  8.1e-14    3e-15
   2        0.28   0.3500000        0.28   19   14   14  4.8e-14  3.9e-15
   3        0.18   0.3500000        0.18   20   15   15  6.3e-14  2.9e-15
Velocity u2, Pressure p
Iter      SolEst     Damping    Stepsize #Res #Jac #Sol   LinErr   LinRes
   1       0.035   0.5000000       0.035   10    5    5  4.1e-15  7.9e-15
Displacement field
Iter      SolEst     Damping    Stepsize #Res #Jac #Sol   LinErr   LinRes
   1        0.17   0.5000000        0.17   10    5    5    4e-14  1.5e-12
Spatial mesh displacement
Iter      SolEst     Damping    Stepsize #Res #Jac #Sol   LinErr   LinRes
   1     5.8e-07   0.5000000     5.8e-07   10    5    5  1.7e-15  1.9e-15
Solution error estimates for segregated groups
0.31, 0.017, 0.084, 2.9e-07
Residual error estimates for segregated groups
33, 2.1e+03, 6.9e+03, 0.18
Segregated solver iteration 6.
Merged variables
Iter      SolEst     Damping    Stepsize #Res #Jac #Sol   LinErr   LinRes
   1        0.12   0.3500000        0.12   22   16   16  4.9e-14  2.8e-15
   2       0.079   0.3500000       0.079   23   17   17  8.2e-14  3.1e-15
   3       0.051   0.3500000       0.051   24   18   18  9.8e-14    3e-15
Velocity u2, Pressure p
Iter      SolEst     Damping    Stepsize #Res #Jac #Sol   LinErr   LinRes
   1       0.017   0.5000000       0.017   12    6    6  2.9e-15  7.8e-15
Displacement field
Iter      SolEst     Damping    Stepsize #Res #Jac #Sol   LinErr   LinRes
   1       0.095   0.5000000       0.095   12    6    6  1.6e-13  1.5e-12
Spatial mesh displacement
Iter      SolEst     Damping    Stepsize #Res #Jac #Sol   LinErr   LinRes
   1     4.1e-07   0.5000000     4.1e-07   12    6    6  1.8e-15  1.9e-15
Solution error estimates for segregated groups
0.088, 0.0085, 0.048, 2e-07
Residual error estimates for segregated groups
12, 1.1e+03, 4.2e+03, 0.17
```

```
Segregated solver iteration 7.
Merged variables
Iter      SolEst     Damping    Stepsize #Res #Jac #Sol   LinErr   LinRes
   1       0.033   0.3500000       0.033   26   19   19  1.1e-13  2.9e-15
   2       0.022   0.3500000       0.022   27   20   20  9.5e-14  3.2e-15
   3       0.014   0.3500000       0.014   28   21   21  6.5e-14  3.8e-15
Velocity u2, Pressure p
Iter      SolEst     Damping    Stepsize #Res #Jac #Sol   LinErr   LinRes
   1      0.0084   0.5000000      0.0084   14    7    7  3.3e-15  7.9e-15
Displacement field
Iter      SolEst     Damping    Stepsize #Res #Jac #Sol   LinErr   LinRes
   1       0.054   0.5000000       0.054   14    7    7  2.6e-14  1.5e-12
Spatial mesh displacement
Iter      SolEst     Damping    Stepsize #Res #Jac #Sol   LinErr   LinRes
   1     2.7e-07   0.5000000     2.7e-07   14    7    7  1.4e-15  1.9e-15
Solution error estimates for segregated groups
0.024, 0.0042, 0.027, 1.4e-07
Residual error estimates for segregated groups
4.1, 5.4e+02, 2.6e+03, 0.17
Segregated solver iteration 8.
Merged variables
Iter      SolEst     Damping    Stepsize #Res #Jac #Sol   LinErr   LinRes
   1      0.0092   0.3500000      0.0092   30   22   22  6.2e-14  2.9e-15
   2       0.006   0.3500000       0.006   31   23   23  8.4e-14  2.7e-15
   3      0.0039   0.3500000      0.0039   32   24   24  7.4e-14  4.1e-15
Velocity u2, Pressure p
Iter      SolEst     Damping    Stepsize #Res #Jac #Sol   LinErr   LinRes
   1      0.0042   0.5000000      0.0042   16    8    8  3.9e-15  7.9e-15
Displacement field
Iter      SolEst     Damping    Stepsize #Res #Jac #Sol   LinErr   LinRes
   1        0.03   0.5000000        0.03   16    8    8    6e-14  1.5e-12
Spatial mesh displacement
Iter      SolEst     Damping    Stepsize #Res #Jac #Sol   LinErr   LinRes
   1     1.8e-07   0.5000000     1.8e-07   16    8    8  1.6e-15  1.8e-15
Solution error estimates for segregated groups
0.0067, 0.0021, 0.015, 8.8e-08
Residual error estimates for segregated groups
1.4, 2.7e+02, 1.8e+03, 0.17
Segregated solver iteration 9.
Merged variables
Iter      SolEst     Damping    Stepsize #Res #Jac #Sol   LinErr   LinRes
   1      0.0025   0.3500000      0.0025   34   25   25  8.4e-14  2.9e-15
   2      0.0016   0.3500000      0.0016   35   26   26  5.9e-14  2.9e-15
   3      0.0011   0.3500000      0.0011   36   27   27  8.7e-14    3e-15
Velocity u2, Pressure p
Iter      SolEst     Damping    Stepsize #Res #Jac #Sol   LinErr   LinRes
   1      0.0021   0.5000000      0.0021   18    9    9    3e-15  7.9e-15
Displacement field
Iter      SolEst     Damping    Stepsize #Res #Jac #Sol   LinErr   LinRes
   1       0.017   0.5000000       0.017   18    9    9  1.7e-13  1.5e-12
Spatial mesh displacement
Iter      SolEst     Damping    Stepsize #Res #Jac #Sol   LinErr   LinRes
   1     1.1e-07   0.5000000     1.1e-07   18    9    9  1.6e-15  1.9e-15
Solution error estimates for segregated groups
0.0018, 0.001, 0.0085, 5.5e-08
Residual error estimates for segregated groups
```

```
0.44, 1.4e+02, 1.4e+03, 0.18
Segregated solver iteration 10.
Merged variables
Iter      SolEst     Damping    Stepsize #Res #Jac #Sol   LinErr   LinRes
   1      0.0007   0.3500000      0.0007   38   28   28  5.2e-14  2.9e-15
   2     0.00045   0.3500000     0.00045   39   29   29  4.2e-14  3.3e-15
   3     0.00029   0.3500000     0.00029   40   30   30  9.7e-14  3.2e-15
Velocity u2, Pressure p
Iter      SolEst     Damping    Stepsize #Res #Jac #Sol   LinErr   LinRes
   1       0.001   0.5000000       0.001   20   10   10  2.3e-15  7.8e-15
Displacement field
Iter      SolEst     Damping    Stepsize #Res #Jac #Sol   LinErr   LinRes
   1      0.0094   0.5000000      0.0094   20   10   10  4.3e-14  1.5e-12
Spatial mesh displacement
Iter      SolEst     Damping    Stepsize #Res #Jac #Sol   LinErr   LinRes
   1     6.7e-08   0.5000000     6.7e-08   20   10   10  1.5e-15  1.9e-15
Solution error estimates for segregated groups
0.00051, 0.00052, 0.0047, 3.4e-08
Residual error estimates for segregated groups
0.14, 68, 1.2e+03, 0.18
Segregated solver iteration 11.
Merged variables
Iter      SolEst     Damping    Stepsize #Res #Jac #Sol   LinErr   LinRes
   1     0.00019   0.3500000     0.00019   42   31   31  1.1e-13    3e-15
   2     0.00012   0.3500000     0.00012   43   32   32  6.8e-14  2.9e-15
   3     8.1e-05   0.3500000     8.1e-05   44   33   33  3.9e-14    3e-15
Velocity u2, Pressure p
Iter      SolEst     Damping    Stepsize #Res #Jac #Sol   LinErr   LinRes
   1     0.00052   0.5000000     0.00052   22   11   11  3.6e-15    8e-15
Displacement field
Iter      SolEst     Damping    Stepsize #Res #Jac #Sol   LinErr   LinRes
   1      0.0051   0.5000000      0.0051   22   11   11  1.1e-13  1.5e-12
Spatial mesh displacement
Iter      SolEst     Damping    Stepsize #Res #Jac #Sol   LinErr   LinRes
   1       4e-08   0.5000000       4e-08   22   11   11  1.4e-15  1.9e-15
Solution error estimates for segregated groups
0.00014, 0.00026, 0.0026, 2e-08
Residual error estimates for segregated groups
0.044, 34, 1.2e+03, 0.18
Segregated solver iteration 12.
Merged variables
Iter      SolEst     Damping    Stepsize #Res #Jac #Sol   LinErr   LinRes
   1     5.3e-05   0.3500000     5.3e-05   46   34   34  8.6e-14  3.3e-15
   2     3.4e-05   0.3500000     3.4e-05   47   35   35  7.3e-14    3e-15
   3     2.2e-05   0.3500000     2.2e-05   48   36   36  8.4e-14    3e-15
Velocity u2, Pressure p
Iter      SolEst     Damping    Stepsize #Res #Jac #Sol   LinErr   LinRes
   1     0.00026   0.5000000     0.00026   24   12   12  3.5e-15  7.9e-15
Displacement field
Iter      SolEst     Damping    Stepsize #Res #Jac #Sol   LinErr   LinRes
   1      0.0028   0.5000000      0.0028   24   12   12    2e-13  1.5e-12
Spatial mesh displacement
Iter      SolEst     Damping    Stepsize #Res #Jac #Sol   LinErr   LinRes
   1     2.4e-08   0.5000000     2.4e-08   24   12   12  1.3e-15  1.9e-15
Solution error estimates for segregated groups
3.8e-05, 0.00013, 0.0014, 1.2e-08
```

```
Residual error estimates for segregated groups
0.013, 17, 1.2e+03, 0.18
Segregated solver iteration 13.
Merged variables
Iter      SolEst      Damping    Stepsize #Res #Jac #Sol   LinErr   LinRes
   1      1.4e-05   0.3500000     1.4e-05   50   37   37  5.1e-14  2.8e-15
   2      9.4e-06   0.3500000     9.4e-06   51   38   38  8.7e-14  3.1e-15
   3      6.1e-06   0.3500000     6.1e-06   52   39   39  9.7e-14    3e-15
Velocity u2, Pressure p
Iter      SolEst      Damping    Stepsize #Res #Jac #Sol   LinErr   LinRes
   1      0.00013   0.5000000     0.00013   26   13   13  3.8e-15  7.8e-15
Displacement field
Iter      SolEst      Damping    Stepsize #Res #Jac #Sol   LinErr   LinRes
   1       0.0015   0.5000000      0.0015   26   13   13  7.3e-14  1.5e-12
Spatial mesh displacement
Iter      SolEst      Damping    Stepsize #Res #Jac #Sol   LinErr   LinRes
   1      1.4e-08   0.5000000     1.4e-08   26   13   13  1.4e-15  1.8e-15
Solution error estimates for segregated groups
1e-05, 6.5e-05, 0.00076, 6.9e-09
Residual error estimates for segregated groups
0.004, 8.5, 1.2e+03, 0.17
Solution time: 154 s. (2 minutes, 34 seconds)
Physical memory: 4.62 GB
Virtual memory: 25.87 GB
Ended at Jan 31, 2025, 6:25:14 PM.
----- Stationary Solver 1 in study1/Approach Curve Solver (sol1) -------------->
```

d=2330 (su36)

GENERAL

Description	Value
Solution	d=2330

LOG

```
<---- Compile Equations: Stationary in study1/Approach Curve Solver (sol1) -----
Started at Jan 31, 2025, 6:25:15 PM.
Parameter d = 2.33E-6 (m).
Geometry shape function: Linear serendipity
Number of vertex elements: 18
Number of boundary elements: 6244
Number of elements: 156248
Minimum element quality: 0.4765
Time: 8 s.
Physical memory: 2.89 GB
Virtual memory: 15.26 GB
Ended at Jan 31, 2025, 6:25:23 PM.
----- Compile Equations: Stationary in study1/Approach Curve Solver (sol1) ---->

<---- Dependent Variables 1 in study1/Approach Curve Solver (sol1) -------------
Started at Jan 31, 2025, 6:25:23 PM.
Solution time: 0 s.
Physical memory: 2.89 GB
Virtual memory: 15.2 GB
Ended at Jan 31, 2025, 6:25:23 PM.
----- Dependent Variables 1 in study1/Approach Curve Solver (sol1) ------------>

<---- Stationary Solver 1 in study1/Approach Curve Solver (sol1) ---------------
Started at Jan 31, 2025, 6:25:23 PM.
Segregated solver
Segregated Step 11 does not have any components solved for.
Number of degrees of freedom solved for: 794333 (plus 11911 internal DOFs).
Segregated solver iteration 1.
Merged variables
Nonsymmetric matrix found.
Scales for dependent variables:
Concentration (comp1.Cl): 1.4e+02
Concentration (comp1.K): 1.4e+02
Electric potential (comp1.V): 1.9e+02
Orthonormal null-space function used.
Iter      SolEst     Damping    Stepsize #Res #Jac #Sol   LinErr   LinRes
   1        0.83   0.3500000        0.83    2    1    1    2e-11  2.7e-15
   2        0.81   0.3500000        0.81    3    2    2  3.8e-11  2.7e-15
   3         0.8   0.3500000         0.8    4    3    3  4.2e-11  2.9e-15
Velocity u2, Pressure p
Nonsymmetric matrix found.
Scales for dependent variables:
Pressure (comp1.p): 1
Velocity field (spatial frame) (comp1.u2): 0.00018
Orthonormal null-space function used.
Iter      SolEst     Damping    Stepsize #Res #Jac #Sol   LinErr   LinRes
   1         1.1   0.5000000         1.1    2    1    1  1.8e-15  2.3e-15
Displacement field
Nonsymmetric matrix found.
Scales for dependent variables:
Auxiliary pressure (comp1.solid.lemm1.pw): 1
Displacement field (comp1.u): 2.8e-18
Orthonormal null-space function used.
Iter      SolEst     Damping    Stepsize #Res #Jac #Sol   LinErr   LinRes
   1         1.9   0.5000000         1.9    2    1    1    4e-14  1.5e-12
Spatial mesh displacement
```

```
Symmetric matrices found.
Scales for dependent variables:
Spatial mesh displacement (comp1.spatial.disp): 1.2e-09
Orthonormal null-space function used.
Iter      SolEst     Damping    Stepsize #Res #Jac #Sol   LinErr   LinRes
   1     6.3e-07   0.5000000     6.3e-07    2    1    1  1.5e-15  1.8e-15
Solution error estimates for segregated groups
1.4, 0.54, 0.95, 3.1e-07
Residual error estimates for segregated groups
7.9e+03, 8.3e+04, 2.2e+04, 0.15
Segregated solver iteration 2.
Merged variables
Scales for dependent variables:
Concentration (comp1.Cl): 1.4e+02
Concentration (comp1.K): 1.4e+02
Electric potential (comp1.V): 0.064
Iter      SolEst     Damping    Stepsize #Res #Jac #Sol   LinErr   LinRes
   1        0.78   0.3500000        0.78    6    4    4  4.1e-14  2.8e-15
   2        0.77   0.3500000        0.77    7    5    5  1.1e-13    3e-15
   3        0.76   0.3500000        0.76    8    6    6  7.5e-14  2.8e-15
Velocity u2, Pressure p
Scales for dependent variables:
Pressure (comp1.p): 3.6e+02
Velocity field (spatial frame) (comp1.u2): 0.00072
Iter      SolEst     Damping    Stepsize #Res #Jac #Sol   LinErr   LinRes
   1        0.36   0.5000000        0.36    4    2    2  2.8e-15    8e-15
Displacement field
Iter      SolEst     Damping    Stepsize #Res #Jac #Sol   LinErr   LinRes
   1        0.95   0.5000000        0.95    4    2    2  5.1e-14  1.5e-12
Spatial mesh displacement
Iter      SolEst     Damping    Stepsize #Res #Jac #Sol   LinErr   LinRes
   1     9.4e-07   0.5000000     9.4e-07    4    2    2  1.5e-15  1.8e-15
Solution error estimates for segregated groups
1.3, 0.18, 0.48, 4.7e-07
Residual error estimates for segregated groups
8e+02, 1.4e+04, 2.2e+04, 0.17
Segregated solver iteration 3.
Merged variables
Iter      SolEst     Damping    Stepsize #Res #Jac #Sol   LinErr   LinRes
   1        0.75   0.3500000        0.75   10    7    7  6.7e-14  2.8e-15
   2        0.73   0.3500000        0.73   11    8    8  7.2e-14  2.7e-15
   3        0.72   0.3500000        0.72   12    9    9  5.5e-14  2.9e-15
Velocity u2, Pressure p
Iter      SolEst     Damping    Stepsize #Res #Jac #Sol   LinErr   LinRes
   1        0.15   0.5000000        0.15    6    3    3  2.6e-15  7.8e-15
Displacement field
Iter      SolEst     Damping    Stepsize #Res #Jac #Sol   LinErr   LinRes
   1        0.52   0.5000000        0.52    6    3    3  1.4e-13  1.5e-12
Spatial mesh displacement
Iter      SolEst     Damping    Stepsize #Res #Jac #Sol   LinErr   LinRes
   1     9.4e-07   0.5000000     9.4e-07    6    3    3  1.6e-15  1.8e-15
Solution error estimates for segregated groups
1.2, 0.077, 0.26, 4.7e-07
Residual error estimates for segregated groups
2.2e+02, 8e+03, 1.6e+04, 0.17
Segregated solver iteration 4.
```

```
Merged variables
Iter      SolEst     Damping    Stepsize #Res #Jac #Sol   LinErr   LinRes
   1         0.7   0.3500000         0.7   14   10   10  8.5e-14  2.8e-15
   2        0.68   0.3500000        0.68   15   11   11    1e-13  3.1e-15
   3         0.6   0.3500000         0.6   16   12   12  5.8e-14  2.7e-15
Velocity u2, Pressure p
Iter      SolEst     Damping    Stepsize #Res #Jac #Sol   LinErr   LinRes
   1       0.072   0.5000000       0.072    8    4    4  4.6e-15  7.7e-15
Displacement field
Iter      SolEst     Damping    Stepsize #Res #Jac #Sol   LinErr   LinRes
   1        0.29   0.5000000        0.29    8    4    4  8.9e-14  1.5e-12
Spatial mesh displacement
Iter      SolEst     Damping    Stepsize #Res #Jac #Sol   LinErr   LinRes
   1     7.8e-07   0.5000000     7.8e-07    8    4    4  1.3e-15  1.8e-15
Solution error estimates for segregated groups
1, 0.036, 0.15, 3.9e-07
Residual error estimates for segregated groups
84, 4.2e+03, 1.1e+04, 0.17
Segregated solver iteration 5.
Merged variables
Iter      SolEst     Damping    Stepsize #Res #Jac #Sol   LinErr   LinRes
   1        0.41   0.3500000        0.41   18   13   13  5.6e-14  3.4e-15
   2        0.28   0.3500000        0.28   19   14   14  4.4e-14  2.7e-15
   3        0.18   0.3500000        0.18   20   15   15  5.9e-14  2.8e-15
Velocity u2, Pressure p
Iter      SolEst     Damping    Stepsize #Res #Jac #Sol   LinErr   LinRes
   1       0.035   0.5000000       0.035   10    5    5  2.8e-15  7.8e-15
Displacement field
Iter      SolEst     Damping    Stepsize #Res #Jac #Sol   LinErr   LinRes
   1        0.17   0.5000000        0.17   10    5    5  9.3e-14  1.5e-12
Spatial mesh displacement
Iter      SolEst     Damping    Stepsize #Res #Jac #Sol   LinErr   LinRes
   1     5.9e-07   0.5000000     5.9e-07   10    5    5  1.5e-15  1.8e-15
Solution error estimates for segregated groups
0.31, 0.017, 0.084, 2.9e-07
Residual error estimates for segregated groups
33, 2.2e+03, 6.9e+03, 0.17
Segregated solver iteration 6.
Merged variables
Iter      SolEst     Damping    Stepsize #Res #Jac #Sol   LinErr   LinRes
   1        0.12   0.3500000        0.12   22   16   16  1.3e-13  2.8e-15
   2       0.079   0.3500000       0.079   23   17   17  4.9e-14    3e-15
   3       0.051   0.3500000       0.051   24   18   18  5.2e-14    3e-15
Velocity u2, Pressure p
Iter      SolEst     Damping    Stepsize #Res #Jac #Sol   LinErr   LinRes
   1       0.017   0.5000000       0.017   12    6    6  2.6e-15  7.7e-15
Displacement field
Iter      SolEst     Damping    Stepsize #Res #Jac #Sol   LinErr   LinRes
   1       0.095   0.5000000       0.095   12    6    6  1.2e-13  1.5e-12
Spatial mesh displacement
Iter      SolEst     Damping    Stepsize #Res #Jac #Sol   LinErr   LinRes
   1     4.1e-07   0.5000000     4.1e-07   12    6    6  1.6e-15  1.8e-15
Solution error estimates for segregated groups
0.088, 0.0085, 0.048, 2.1e-07
Residual error estimates for segregated groups
12, 1.1e+03, 4.2e+03, 0.17
```

```
Segregated solver iteration 7.
Merged variables
Iter      SolEst     Damping    Stepsize #Res #Jac #Sol   LinErr   LinRes
   1       0.033   0.3500000       0.033   26   19   19  9.2e-14  2.8e-15
   2       0.022   0.3500000       0.022   27   20   20  1.4e-13  2.8e-15
   3       0.014   0.3500000       0.014   28   21   21  1.1e-13  2.8e-15
Velocity u2, Pressure p
Iter      SolEst     Damping    Stepsize #Res #Jac #Sol   LinErr   LinRes
   1      0.0085   0.5000000      0.0085   14    7    7  3.6e-15  7.9e-15
Displacement field
Iter      SolEst     Damping    Stepsize #Res #Jac #Sol   LinErr   LinRes
   1       0.054   0.5000000       0.054   14    7    7  4.9e-14  1.5e-12
Spatial mesh displacement
Iter      SolEst     Damping    Stepsize #Res #Jac #Sol   LinErr   LinRes
   1     2.7e-07   0.5000000     2.7e-07   14    7    7  1.3e-15  1.8e-15
Solution error estimates for segregated groups
0.024, 0.0042, 0.027, 1.4e-07
Residual error estimates for segregated groups
4.1, 5.6e+02, 2.6e+03, 0.19
Segregated solver iteration 8.
Merged variables
Iter      SolEst     Damping    Stepsize #Res #Jac #Sol   LinErr   LinRes
   1      0.0092   0.3500000      0.0092   30   22   22  3.1e-14  2.9e-15
   2       0.006   0.3500000       0.006   31   23   23  5.7e-14  2.8e-15
   3      0.0039   0.3500000      0.0039   32   24   24  7.8e-14  2.8e-15
Velocity u2, Pressure p
Iter      SolEst     Damping    Stepsize #Res #Jac #Sol   LinErr   LinRes
   1      0.0042   0.5000000      0.0042   16    8    8  2.5e-15  7.9e-15
Displacement field
Iter      SolEst     Damping    Stepsize #Res #Jac #Sol   LinErr   LinRes
   1        0.03   0.5000000        0.03   16    8    8  8.1e-14  1.5e-12
Spatial mesh displacement
Iter      SolEst     Damping    Stepsize #Res #Jac #Sol   LinErr   LinRes
   1     1.8e-07   0.5000000     1.8e-07   16    8    8  2.1e-15  1.9e-15
Solution error estimates for segregated groups
0.0067, 0.0021, 0.015, 8.8e-08
Residual error estimates for segregated groups
1.4, 2.8e+02, 1.8e+03, 0.18
Segregated solver iteration 9.
Merged variables
Iter      SolEst     Damping    Stepsize #Res #Jac #Sol   LinErr   LinRes
   1      0.0025   0.3500000      0.0025   34   25   25  9.3e-14  2.7e-15
   2      0.0016   0.3500000      0.0016   35   26   26  1.1e-13  2.8e-15
   3      0.0011   0.3500000      0.0011   36   27   27  7.7e-14  2.9e-15
Velocity u2, Pressure p
Iter      SolEst     Damping    Stepsize #Res #Jac #Sol   LinErr   LinRes
   1      0.0021   0.5000000      0.0021   18    9    9  2.1e-15  7.8e-15
Displacement field
Iter      SolEst     Damping    Stepsize #Res #Jac #Sol   LinErr   LinRes
   1       0.017   0.5000000       0.017   18    9    9  1.7e-13  1.5e-12
Spatial mesh displacement
Iter      SolEst     Damping    Stepsize #Res #Jac #Sol   LinErr   LinRes
   1     1.1e-07   0.5000000     1.1e-07   18    9    9  1.4e-15  1.8e-15
Solution error estimates for segregated groups
0.0018, 0.0011, 0.0085, 5.5e-08
Residual error estimates for segregated groups
```

```
0.44, 1.4e+02, 1.4e+03, 0.19
Segregated solver iteration 10.
Merged variables
Iter      SolEst     Damping    Stepsize #Res #Jac #Sol   LinErr   LinRes
   1      0.0007   0.3500000      0.0007   38   28   28  7.1e-14    3e-15
   2     0.00045   0.3500000     0.00045   39   29   29  6.5e-14  3.1e-15
   3     0.00029   0.3500000     0.00029   40   30   30  4.5e-14  3.3e-15
Velocity u2, Pressure p
Iter      SolEst     Damping    Stepsize #Res #Jac #Sol   LinErr   LinRes
   1       0.001   0.5000000       0.001   20   10   10  3.7e-15  7.9e-15
Displacement field
Iter      SolEst     Damping    Stepsize #Res #Jac #Sol   LinErr   LinRes
   1      0.0094   0.5000000      0.0094   20   10   10  1.4e-13  1.5e-12
Spatial mesh displacement
Iter      SolEst     Damping    Stepsize #Res #Jac #Sol   LinErr   LinRes
   1     6.7e-08   0.5000000     6.7e-08   20   10   10  1.5e-15  1.8e-15
Solution error estimates for segregated groups
0.0005, 0.00052, 0.0047, 3.4e-08
Residual error estimates for segregated groups
0.14, 70, 1.3e+03, 0.17
Segregated solver iteration 11.
Merged variables
Iter      SolEst     Damping    Stepsize #Res #Jac #Sol   LinErr   LinRes
   1     0.00019   0.3500000     0.00019   42   31   31  7.3e-14  3.5e-15
   2     0.00012   0.3500000     0.00012   43   32   32  4.7e-14  2.8e-15
   3     8.1e-05   0.3500000     8.1e-05   44   33   33  5.3e-14  2.8e-15
Velocity u2, Pressure p
Iter      SolEst     Damping    Stepsize #Res #Jac #Sol   LinErr   LinRes
   1     0.00052   0.5000000     0.00052   22   11   11  3.7e-15  7.8e-15
Displacement field
Iter      SolEst     Damping    Stepsize #Res #Jac #Sol   LinErr   LinRes
   1      0.0051   0.5000000      0.0051   22   11   11  7.2e-14  1.5e-12
Spatial mesh displacement
Iter      SolEst     Damping    Stepsize #Res #Jac #Sol   LinErr   LinRes
   1       4e-08   0.5000000       4e-08   22   11   11  1.2e-15  1.8e-15
Solution error estimates for segregated groups
0.00014, 0.00026, 0.0026, 2e-08
Residual error estimates for segregated groups
0.044, 35, 1.2e+03, 0.17
Segregated solver iteration 12.
Merged variables
Iter      SolEst     Damping    Stepsize #Res #Jac #Sol   LinErr   LinRes
   1     5.2e-05   0.3500000     5.2e-05   46   34   34  9.7e-14  3.4e-15
   2     3.4e-05   0.3500000     3.4e-05   47   35   35  8.5e-14  2.9e-15
   3     2.2e-05   0.3500000     2.2e-05   48   36   36  7.4e-14  3.1e-15
Velocity u2, Pressure p
Iter      SolEst     Damping    Stepsize #Res #Jac #Sol   LinErr   LinRes
   1     0.00026   0.5000000     0.00026   24   12   12  2.1e-15  7.9e-15
Displacement field
Iter      SolEst     Damping    Stepsize #Res #Jac #Sol   LinErr   LinRes
   1      0.0028   0.5000000      0.0028   24   12   12  4.3e-14  1.5e-12
Spatial mesh displacement
Iter      SolEst     Damping    Stepsize #Res #Jac #Sol   LinErr   LinRes
   1     2.4e-08   0.5000000     2.4e-08   24   12   12  1.7e-15  1.9e-15
Solution error estimates for segregated groups
3.8e-05, 0.00013, 0.0014, 1.2e-08
```

```
Residual error estimates for segregated groups
0.013, 17, 1.2e+03, 0.18
Segregated solver iteration 13.
Merged variables
Iter      SolEst     Damping    Stepsize #Res #Jac #Sol   LinErr   LinRes
   1     1.4e-05   0.3500000     1.4e-05   50   37   37  8.4e-14    3e-15
   2     9.4e-06   0.3500000     9.4e-06   51   38   38  1.4e-13    3e-15
   3     6.1e-06   0.3500000     6.1e-06   52   39   39    1e-13  2.9e-15
Velocity u2, Pressure p
Iter      SolEst     Damping    Stepsize #Res #Jac #Sol   LinErr   LinRes
   1     0.00013   0.5000000     0.00013   26   13   13  3.8e-15  7.8e-15
Displacement field
Iter      SolEst     Damping    Stepsize #Res #Jac #Sol   LinErr   LinRes
   1      0.0015   0.5000000      0.0015   26   13   13  1.9e-13  1.5e-12
Spatial mesh displacement
Iter      SolEst     Damping    Stepsize #Res #Jac #Sol   LinErr   LinRes
   1     1.4e-08   0.5000000     1.4e-08   26   13   13  1.8e-15  1.9e-15
Solution error estimates for segregated groups
1e-05, 6.6e-05, 0.00076, 7e-09
Residual error estimates for segregated groups
0.004, 8.7, 1.2e+03, 0.19
Solution time: 156 s. (2 minutes, 36 seconds)
Physical memory: 4.61 GB
Virtual memory: 25.53 GB
Ended at Jan 31, 2025, 6:27:59 PM.
----- Stationary Solver 1 in study1/Approach Curve Solver (sol1) -------------->
```

d=2325 (su37)

GENERAL

Description	Value
Solution	d=2325

LOG

```
<---- Compile Equations: Stationary in study1/Approach Curve Solver (sol1) -----
Started at Jan 31, 2025, 6:27:59 PM.
Parameter d = 2.325E-6 (m).
Geometry shape function: Linear serendipity
Number of vertex elements: 18
Number of boundary elements: 6242
Number of elements: 156436
Minimum element quality: 0.4697
Time: 8 s.
Physical memory: 2.87 GB
Virtual memory: 15.18 GB
Ended at Jan 31, 2025, 6:28:08 PM.
----- Compile Equations: Stationary in study1/Approach Curve Solver (sol1) ---->

<---- Dependent Variables 1 in study1/Approach Curve Solver (sol1) -------------
Started at Jan 31, 2025, 6:28:08 PM.
Solution time: 0 s.
Physical memory: 2.87 GB
Virtual memory: 15.18 GB
Ended at Jan 31, 2025, 6:28:08 PM.
----- Dependent Variables 1 in study1/Approach Curve Solver (sol1) ------------>

<---- Stationary Solver 1 in study1/Approach Curve Solver (sol1) ---------------
Started at Jan 31, 2025, 6:28:08 PM.
Segregated solver
Segregated Step 11 does not have any components solved for.
Number of degrees of freedom solved for: 795534 (plus 11907 internal DOFs).
Segregated solver iteration 1.
Merged variables
Nonsymmetric matrix found.
Scales for dependent variables:
Concentration (comp1.Cl): 1.4e+02
Concentration (comp1.K): 1.4e+02
Electric potential (comp1.V): 1.9e+02
Orthonormal null-space function used.
Iter      SolEst     Damping    Stepsize #Res #Jac #Sol   LinErr   LinRes
   1        0.83   0.3500000        0.83    2    1    1  4.2e-11  2.7e-15
   2        0.81   0.3500000        0.81    3    2    2    4e-11  2.7e-15
   3         0.8   0.3500000         0.8    4    3    3  5.7e-11  2.7e-15
Velocity u2, Pressure p
Nonsymmetric matrix found.
Scales for dependent variables:
Pressure (comp1.p): 1
Velocity field (spatial frame) (comp1.u2): 0.00018
Orthonormal null-space function used.
Iter      SolEst     Damping    Stepsize #Res #Jac #Sol   LinErr   LinRes
   1         1.1   0.5000000         1.1    2    1    1  1.9e-15  2.3e-15
Displacement field
Nonsymmetric matrix found.
Scales for dependent variables:
Auxiliary pressure (comp1.solid.lemm1.pw): 1
Displacement field (comp1.u): 2.8e-18
Orthonormal null-space function used.
Iter      SolEst     Damping    Stepsize #Res #Jac #Sol   LinErr   LinRes
   1         1.9   0.5000000         1.9    2    1    1  7.5e-14  1.5e-12
Spatial mesh displacement
```

```
Symmetric matrices found.
Scales for dependent variables:
Spatial mesh displacement (comp1.spatial.disp): 1.2e-09
Orthonormal null-space function used.
Iter      SolEst     Damping    Stepsize #Res #Jac #Sol   LinErr   LinRes
   1     6.3e-07   0.5000000     6.3e-07    2    1    1  1.6e-15  1.8e-15
Solution error estimates for segregated groups
1.4, 0.54, 0.95, 3.1e-07
Residual error estimates for segregated groups
7.9e+03, 8.3e+04, 2.2e+04, 0.15
Segregated solver iteration 2.
Merged variables
Scales for dependent variables:
Concentration (comp1.Cl): 1.4e+02
Concentration (comp1.K): 1.4e+02
Electric potential (comp1.V): 0.064
Iter      SolEst     Damping    Stepsize #Res #Jac #Sol   LinErr   LinRes
   1        0.79   0.3500000        0.79    6    4    4  6.1e-14  3.5e-15
   2        0.77   0.3500000        0.77    7    5    5  6.8e-14  3.4e-15
   3        0.76   0.3500000        0.76    8    6    6  1.1e-13  3.6e-15
Velocity u2, Pressure p
Scales for dependent variables:
Pressure (comp1.p): 3.5e+02
Velocity field (spatial frame) (comp1.u2): 0.00071
Iter      SolEst     Damping    Stepsize #Res #Jac #Sol   LinErr   LinRes
   1        0.36   0.5000000        0.36    4    2    2  4.3e-15  7.9e-15
Displacement field
Iter      SolEst     Damping    Stepsize #Res #Jac #Sol   LinErr   LinRes
   1        0.95   0.5000000        0.95    4    2    2  5.7e-14  1.5e-12
Spatial mesh displacement
Iter      SolEst     Damping    Stepsize #Res #Jac #Sol   LinErr   LinRes
   1     9.4e-07   0.5000000     9.4e-07    4    2    2  1.5e-15  1.9e-15
Solution error estimates for segregated groups
1.3, 0.18, 0.48, 4.7e-07
Residual error estimates for segregated groups
8e+02, 1.4e+04, 2.2e+04, 0.18
Segregated solver iteration 3.
Merged variables
Iter      SolEst     Damping    Stepsize #Res #Jac #Sol   LinErr   LinRes
   1        0.75   0.3500000        0.75   10    7    7  1.2e-13  5.3e-15
   2        0.73   0.3500000        0.73   11    8    8  1.9e-13  4.3e-15
   3        0.72   0.3500000        0.72   12    9    9  1.3e-13  3.3e-15
Velocity u2, Pressure p
Iter      SolEst     Damping    Stepsize #Res #Jac #Sol   LinErr   LinRes
   1        0.15   0.5000000        0.15    6    3    3  2.8e-15  7.8e-15
Displacement field
Iter      SolEst     Damping    Stepsize #Res #Jac #Sol   LinErr   LinRes
   1        0.52   0.5000000        0.52    6    3    3  3.7e-14  1.5e-12
Spatial mesh displacement
Iter      SolEst     Damping    Stepsize #Res #Jac #Sol   LinErr   LinRes
   1     9.4e-07   0.5000000     9.4e-07    6    3    3  1.5e-15  1.9e-15
Solution error estimates for segregated groups
1.2, 0.077, 0.26, 4.7e-07
Residual error estimates for segregated groups
2.2e+02, 8e+03, 1.6e+04, 0.18
Segregated solver iteration 4.
```

```
Merged variables
Iter      SolEst     Damping    Stepsize #Res #Jac #Sol   LinErr   LinRes
   1         0.7   0.3500000         0.7   14   10   10  1.1e-13  3.6e-15
   2        0.68   0.3500000        0.68   15   11   11  6.7e-14  7.1e-15
   3         0.6   0.3500000         0.6   16   12   12  1.7e-13  4.6e-15
Velocity u2, Pressure p
Iter      SolEst     Damping    Stepsize #Res #Jac #Sol   LinErr   LinRes
   1       0.071   0.5000000       0.071    8    4    4  3.6e-15  7.9e-15
Displacement field
Iter      SolEst     Damping    Stepsize #Res #Jac #Sol   LinErr   LinRes
   1        0.29   0.5000000        0.29    8    4    4  4.2e-14  1.5e-12
Spatial mesh displacement
Iter      SolEst     Damping    Stepsize #Res #Jac #Sol   LinErr   LinRes
   1     7.9e-07   0.5000000     7.9e-07    8    4    4  1.5e-15  1.9e-15
Solution error estimates for segregated groups
1, 0.036, 0.15, 3.9e-07
Residual error estimates for segregated groups
84, 4.2e+03, 1.1e+04, 0.18
Segregated solver iteration 5.
Merged variables
Iter      SolEst     Damping    Stepsize #Res #Jac #Sol   LinErr   LinRes
   1        0.41   0.3500000        0.41   18   13   13  6.1e-14  3.1e-15
   2        0.28   0.3500000        0.28   19   14   14  1.9e-13  3.8e-15
   3        0.18   0.3500000        0.18   20   15   15  9.2e-14  4.2e-15
Velocity u2, Pressure p
Iter      SolEst     Damping    Stepsize #Res #Jac #Sol   LinErr   LinRes
   1       0.035   0.5000000       0.035   10    5    5  2.4e-15  7.8e-15
Displacement field
Iter      SolEst     Damping    Stepsize #Res #Jac #Sol   LinErr   LinRes
   1        0.17   0.5000000        0.17   10    5    5  5.2e-14  1.5e-12
Spatial mesh displacement
Iter      SolEst     Damping    Stepsize #Res #Jac #Sol   LinErr   LinRes
   1     5.9e-07   0.5000000     5.9e-07   10    5    5  2.2e-15  1.9e-15
Solution error estimates for segregated groups
0.32, 0.017, 0.084, 2.9e-07
Residual error estimates for segregated groups
33, 2.2e+03, 6.9e+03, 0.18
Segregated solver iteration 6.
Merged variables
Iter      SolEst     Damping    Stepsize #Res #Jac #Sol   LinErr   LinRes
   1        0.12   0.3500000        0.12   22   16   16  1.4e-13  3.1e-15
   2       0.079   0.3500000       0.079   23   17   17  1.2e-13  3.6e-15
   3       0.052   0.3500000       0.052   24   18   18    9e-14  3.6e-15
Velocity u2, Pressure p
Iter      SolEst     Damping    Stepsize #Res #Jac #Sol   LinErr   LinRes
   1       0.017   0.5000000       0.017   12    6    6  2.1e-15  7.9e-15
Displacement field
Iter      SolEst     Damping    Stepsize #Res #Jac #Sol   LinErr   LinRes
   1       0.095   0.5000000       0.095   12    6    6  9.8e-14  1.5e-12
Spatial mesh displacement
Iter      SolEst     Damping    Stepsize #Res #Jac #Sol   LinErr   LinRes
   1     4.1e-07   0.5000000     4.1e-07   12    6    6  1.9e-15  1.8e-15
Solution error estimates for segregated groups
0.089, 0.0085, 0.048, 2.1e-07
Residual error estimates for segregated groups
12, 1.1e+03, 4.2e+03, 0.18
```

```
Segregated solver iteration 7.
Merged variables
Iter      SolEst     Damping    Stepsize #Res #Jac #Sol   LinErr   LinRes
   1       0.034   0.3500000       0.034   26   19   19    1e-13  3.1e-15
   2       0.022   0.3500000       0.022   27   20   20    1e-13  4.4e-15
   3       0.014   0.3500000       0.014   28   21   21  1.5e-13  2.9e-15
Velocity u2, Pressure p
Iter      SolEst     Damping    Stepsize #Res #Jac #Sol   LinErr   LinRes
   1      0.0084   0.5000000      0.0084   14    7    7  3.7e-15    8e-15
Displacement field
Iter      SolEst     Damping    Stepsize #Res #Jac #Sol   LinErr   LinRes
   1       0.054   0.5000000       0.054   14    7    7  1.3e-13  1.5e-12
Spatial mesh displacement
Iter      SolEst     Damping    Stepsize #Res #Jac #Sol   LinErr   LinRes
   1     2.8e-07   0.5000000     2.8e-07   14    7    7  1.7e-15  1.9e-15
Solution error estimates for segregated groups
0.024, 0.0042, 0.027, 1.4e-07
Residual error estimates for segregated groups
4.1, 5.5e+02, 2.6e+03, 0.17
Segregated solver iteration 8.
Merged variables
Iter      SolEst     Damping    Stepsize #Res #Jac #Sol   LinErr   LinRes
   1      0.0093   0.3500000      0.0093   30   22   22  8.5e-14    3e-15
   2       0.006   0.3500000       0.006   31   23   23  6.9e-14  6.2e-15
   3      0.0039   0.3500000      0.0039   32   24   24    1e-13  3.8e-15
Velocity u2, Pressure p
Iter      SolEst     Damping    Stepsize #Res #Jac #Sol   LinErr   LinRes
   1      0.0042   0.5000000      0.0042   16    8    8  2.1e-15  7.9e-15
Displacement field
Iter      SolEst     Damping    Stepsize #Res #Jac #Sol   LinErr   LinRes
   1        0.03   0.5000000        0.03   16    8    8  9.8e-14  1.5e-12
Spatial mesh displacement
Iter      SolEst     Damping    Stepsize #Res #Jac #Sol   LinErr   LinRes
   1     1.8e-07   0.5000000     1.8e-07   16    8    8  1.4e-15  1.9e-15
Solution error estimates for segregated groups
0.0067, 0.0021, 0.015, 8.8e-08
Residual error estimates for segregated groups
1.4, 2.8e+02, 1.8e+03, 0.16
Segregated solver iteration 9.
Merged variables
Iter      SolEst     Damping    Stepsize #Res #Jac #Sol   LinErr   LinRes
   1      0.0025   0.3500000      0.0025   34   25   25  1.2e-13  3.3e-15
   2      0.0017   0.3500000      0.0017   35   26   26  1.1e-13  3.1e-15
   3      0.0011   0.3500000      0.0011   36   27   27  9.4e-14  3.7e-15
Velocity u2, Pressure p
Iter      SolEst     Damping    Stepsize #Res #Jac #Sol   LinErr   LinRes
   1      0.0021   0.5000000      0.0021   18    9    9  4.1e-15  7.9e-15
Displacement field
Iter      SolEst     Damping    Stepsize #Res #Jac #Sol   LinErr   LinRes
   1       0.017   0.5000000       0.017   18    9    9  7.1e-14  1.5e-12
Spatial mesh displacement
Iter      SolEst     Damping    Stepsize #Res #Jac #Sol   LinErr   LinRes
   1     1.1e-07   0.5000000     1.1e-07   18    9    9  1.6e-15  1.8e-15
Solution error estimates for segregated groups
0.0018, 0.001, 0.0085, 5.5e-08
Residual error estimates for segregated groups
```

```
0.44, 1.4e+02, 1.4e+03, 0.17
Segregated solver iteration 10.
Merged variables
Iter      SolEst     Damping    Stepsize #Res #Jac #Sol   LinErr   LinRes
   1      0.0007   0.3500000      0.0007   38   28   28    1e-13  4.2e-15
   2     0.00045   0.3500000     0.00045   39   29   29  9.1e-14  8.8e-15
   3      0.0003   0.3500000      0.0003   40   30   30  1.2e-13    3e-15
Velocity u2, Pressure p
Iter      SolEst     Damping    Stepsize #Res #Jac #Sol   LinErr   LinRes
   1       0.001   0.5000000       0.001   20   10   10  2.6e-15  7.9e-15
Displacement field
Iter      SolEst     Damping    Stepsize #Res #Jac #Sol   LinErr   LinRes
   1      0.0094   0.5000000      0.0094   20   10   10  6.2e-14  1.5e-12
Spatial mesh displacement
Iter      SolEst     Damping    Stepsize #Res #Jac #Sol   LinErr   LinRes
   1     6.8e-08   0.5000000     6.8e-08   20   10   10  2.1e-15  1.9e-15
Solution error estimates for segregated groups
0.00051, 0.00052, 0.0047, 3.4e-08
Residual error estimates for segregated groups
0.14, 69, 1.2e+03, 0.18
Segregated solver iteration 11.
Merged variables
Iter      SolEst     Damping    Stepsize #Res #Jac #Sol   LinErr   LinRes
   1     0.00019   0.3500000     0.00019   42   31   31  1.5e-13  3.1e-15
   2     0.00012   0.3500000     0.00012   43   32   32    5e-14  8.1e-15
   3     8.1e-05   0.3500000     8.1e-05   44   33   33    9e-14  3.3e-15
Velocity u2, Pressure p
Iter      SolEst     Damping    Stepsize #Res #Jac #Sol   LinErr   LinRes
   1     0.00052   0.5000000     0.00052   22   11   11  4.1e-15  7.9e-15
Displacement field
Iter      SolEst     Damping    Stepsize #Res #Jac #Sol   LinErr   LinRes
   1      0.0051   0.5000000      0.0051   22   11   11  4.9e-14  1.5e-12
Spatial mesh displacement
Iter      SolEst     Damping    Stepsize #Res #Jac #Sol   LinErr   LinRes
   1     4.1e-08   0.5000000     4.1e-08   22   11   11  1.5e-15  1.8e-15
Solution error estimates for segregated groups
0.00014, 0.00026, 0.0026, 2e-08
Residual error estimates for segregated groups
0.044, 35, 1.2e+03, 0.17
Segregated solver iteration 12.
Merged variables
Iter      SolEst     Damping    Stepsize #Res #Jac #Sol   LinErr   LinRes
   1     5.3e-05   0.3500000     5.3e-05   46   34   34  6.7e-14  6.5e-15
   2     3.4e-05   0.3500000     3.4e-05   47   35   35  5.5e-14  4.6e-15
   3     2.2e-05   0.3500000     2.2e-05   48   36   36  1.3e-13  3.1e-15
Velocity u2, Pressure p
Iter      SolEst     Damping    Stepsize #Res #Jac #Sol   LinErr   LinRes
   1     0.00026   0.5000000     0.00026   24   12   12  3.7e-15  7.9e-15
Displacement field
Iter      SolEst     Damping    Stepsize #Res #Jac #Sol   LinErr   LinRes
   1      0.0028   0.5000000      0.0028   24   12   12  7.2e-14  1.5e-12
Spatial mesh displacement
Iter      SolEst     Damping    Stepsize #Res #Jac #Sol   LinErr   LinRes
   1     2.4e-08   0.5000000     2.4e-08   24   12   12  1.7e-15  1.9e-15
Solution error estimates for segregated groups
3.8e-05, 0.00013, 0.0014, 1.2e-08
```

```
Residual error estimates for segregated groups
0.013, 17, 1.2e+03, 0.17
Segregated solver iteration 13.
Merged variables
Iter      SolEst     Damping    Stepsize #Res #Jac #Sol   LinErr   LinRes
   1     1.4e-05   0.3500000     1.4e-05   50   37   37  3.5e-14  2.8e-15
   2     9.4e-06   0.3500000     9.4e-06   51   38   38  1.1e-13  2.9e-15
   3     6.1e-06   0.3500000     6.1e-06   52   39   39  9.8e-14  4.8e-15
Velocity u2, Pressure p
Iter      SolEst     Damping    Stepsize #Res #Jac #Sol   LinErr   LinRes
   1     0.00013   0.5000000     0.00013   26   13   13    2e-15  7.8e-15
Displacement field
Iter      SolEst     Damping    Stepsize #Res #Jac #Sol   LinErr   LinRes
   1      0.0015   0.5000000      0.0015   26   13   13  4.1e-14  1.5e-12
Spatial mesh displacement
Iter      SolEst     Damping    Stepsize #Res #Jac #Sol   LinErr   LinRes
   1     1.4e-08   0.5000000     1.4e-08   26   13   13  1.5e-15  1.8e-15
Solution error estimates for segregated groups
1.1e-05, 6.5e-05, 0.00076, 7e-09
Residual error estimates for segregated groups
0.0041, 8.6, 1.2e+03, 0.18
Solution time: 156 s. (2 minutes, 36 seconds)
Physical memory: 4.62 GB
Virtual memory: 25.51 GB
Ended at Jan 31, 2025, 6:30:44 PM.
----- Stationary Solver 1 in study1/Approach Curve Solver (sol1) -------------->
```

d=2320 (su38)

GENERAL

Description	Value
Solution	d=2320

LOG

```
<---- Compile Equations: Stationary in study1/Approach Curve Solver (sol1) -----
Started at Jan 31, 2025, 6:30:44 PM.
Parameter d = 2.3200000000000002E-6 (m).
Geometry shape function: Linear serendipity
Number of vertex elements: 18
Number of boundary elements: 6239
Number of elements: 156259
Minimum element quality: 0.4839
Time: 9 s.
Physical memory: 2.87 GB
Virtual memory: 15.18 GB
Ended at Jan 31, 2025, 6:30:52 PM.
----- Compile Equations: Stationary in study1/Approach Curve Solver (sol1) ---->

<---- Dependent Variables 1 in study1/Approach Curve Solver (sol1) -------------
Started at Jan 31, 2025, 6:30:52 PM.
Solution time: 0 s.
Physical memory: 2.87 GB
Virtual memory: 15.18 GB
Ended at Jan 31, 2025, 6:30:52 PM.
----- Dependent Variables 1 in study1/Approach Curve Solver (sol1) ------------>

<---- Stationary Solver 1 in study1/Approach Curve Solver (sol1) ---------------
Started at Jan 31, 2025, 6:30:53 PM.
Segregated solver
Segregated Step 11 does not have any components solved for.
Number of degrees of freedom solved for: 794569 (plus 11901 internal DOFs).
Segregated solver iteration 1.
Merged variables
Nonsymmetric matrix found.
Scales for dependent variables:
Concentration (comp1.Cl): 1.4e+02
Concentration (comp1.K): 1.4e+02
Electric potential (comp1.V): 1.9e+02
Orthonormal null-space function used.
Iter      SolEst     Damping    Stepsize #Res #Jac #Sol   LinErr   LinRes
   1        0.83   0.3500000        0.83    2    1    1    4e-11  2.8e-15
   2        0.81   0.3500000        0.81    3    2    2  1.5e-11  2.7e-15
   3         0.8   0.3500000         0.8    4    3    3  1.7e-11  2.7e-15
Velocity u2, Pressure p
Nonsymmetric matrix found.
Scales for dependent variables:
Pressure (comp1.p): 1
Velocity field (spatial frame) (comp1.u2): 0.00018
Orthonormal null-space function used.
Iter      SolEst     Damping    Stepsize #Res #Jac #Sol   LinErr   LinRes
   1         1.1   0.5000000         1.1    2    1    1  1.8e-15  2.2e-15
Displacement field
Nonsymmetric matrix found.
Scales for dependent variables:
Auxiliary pressure (comp1.solid.lemm1.pw): 1
Displacement field (comp1.u): 2.8e-18
Orthonormal null-space function used.
Iter      SolEst     Damping    Stepsize #Res #Jac #Sol   LinErr   LinRes
   1         1.9   0.5000000         1.9    2    1    1  4.6e-14  1.5e-12
Spatial mesh displacement
```

```
Symmetric matrices found.
Scales for dependent variables:
Spatial mesh displacement (comp1.spatial.disp): 1.2e-09
Orthonormal null-space function used.
Iter      SolEst     Damping    Stepsize #Res #Jac #Sol   LinErr   LinRes
   1     6.3e-07   0.5000000     6.3e-07    2    1    1  1.5e-15  1.9e-15
Solution error estimates for segregated groups
1.4, 0.54, 0.95, 3.1e-07
Residual error estimates for segregated groups
7.9e+03, 8.3e+04, 2.2e+04, 0.15
Segregated solver iteration 2.
Merged variables
Scales for dependent variables:
Concentration (comp1.Cl): 1.4e+02
Concentration (comp1.K): 1.4e+02
Electric potential (comp1.V): 0.064
Iter      SolEst     Damping    Stepsize #Res #Jac #Sol   LinErr   LinRes
   1        0.79   0.3500000        0.79    6    4    4  1.3e-13  2.8e-15
   2        0.77   0.3500000        0.77    7    5    5  1.1e-13  2.8e-15
   3        0.76   0.3500000        0.76    8    6    6  4.9e-14  2.8e-15
Velocity u2, Pressure p
Scales for dependent variables:
Pressure (comp1.p): 3.6e+02
Velocity field (spatial frame) (comp1.u2): 0.00072
Iter      SolEst     Damping    Stepsize #Res #Jac #Sol   LinErr   LinRes
   1        0.36   0.5000000        0.36    4    2    2  2.7e-15  7.9e-15
Displacement field
Iter      SolEst     Damping    Stepsize #Res #Jac #Sol   LinErr   LinRes
   1        0.95   0.5000000        0.95    4    2    2    6e-14  1.5e-12
Spatial mesh displacement
Iter      SolEst     Damping    Stepsize #Res #Jac #Sol   LinErr   LinRes
   1     9.4e-07   0.5000000     9.4e-07    4    2    2  1.7e-15  1.9e-15
Solution error estimates for segregated groups
1.3, 0.18, 0.48, 4.7e-07
Residual error estimates for segregated groups
8e+02, 1.3e+04, 2.2e+04, 0.16
Segregated solver iteration 3.
Merged variables
Iter      SolEst     Damping    Stepsize #Res #Jac #Sol   LinErr   LinRes
   1        0.75   0.3500000        0.75   10    7    7  9.8e-14  2.6e-15
   2        0.73   0.3500000        0.73   11    8    8    1e-13  2.7e-15
   3        0.72   0.3500000        0.72   12    9    9  3.6e-14  2.7e-15
Velocity u2, Pressure p
Iter      SolEst     Damping    Stepsize #Res #Jac #Sol   LinErr   LinRes
   1        0.15   0.5000000        0.15    6    3    3  3.7e-15  7.9e-15
Displacement field
Iter      SolEst     Damping    Stepsize #Res #Jac #Sol   LinErr   LinRes
   1        0.52   0.5000000        0.52    6    3    3  1.1e-13  1.5e-12
Spatial mesh displacement
Iter      SolEst     Damping    Stepsize #Res #Jac #Sol   LinErr   LinRes
   1     9.4e-07   0.5000000     9.4e-07    6    3    3  1.3e-15  1.8e-15
Solution error estimates for segregated groups
1.2, 0.077, 0.26, 4.7e-07
Residual error estimates for segregated groups
2.2e+02, 7.4e+03, 1.6e+04, 0.17
Segregated solver iteration 4.
```

```
Merged variables
Iter      SolEst     Damping    Stepsize #Res #Jac #Sol   LinErr   LinRes
   1         0.7   0.3500000         0.7   14   10   10  5.1e-14  2.8e-15
   2        0.68   0.3500000        0.68   15   11   11  5.9e-14  2.7e-15
   3         0.6   0.3500000         0.6   16   12   12  9.2e-14  2.8e-15
Velocity u2, Pressure p
Iter      SolEst     Damping    Stepsize #Res #Jac #Sol   LinErr   LinRes
   1       0.071   0.5000000       0.071    8    4    4  2.6e-15  7.9e-15
Displacement field
Iter      SolEst     Damping    Stepsize #Res #Jac #Sol   LinErr   LinRes
   1        0.29   0.5000000        0.29    8    4    4  2.2e-13  1.5e-12
Spatial mesh displacement
Iter      SolEst     Damping    Stepsize #Res #Jac #Sol   LinErr   LinRes
   1     7.8e-07   0.5000000     7.8e-07    8    4    4  1.3e-15  1.8e-15
Solution error estimates for segregated groups
1, 0.036, 0.15, 3.9e-07
Residual error estimates for segregated groups
84, 3.9e+03, 1.1e+04, 0.19
Segregated solver iteration 5.
Merged variables
Iter      SolEst     Damping    Stepsize #Res #Jac #Sol   LinErr   LinRes
   1        0.41   0.3500000        0.41   18   13   13    5e-14  2.7e-15
   2        0.28   0.3500000        0.28   19   14   14  4.8e-14  2.8e-15
   3        0.18   0.3500000        0.18   20   15   15  3.8e-14  2.8e-15
Velocity u2, Pressure p
Iter      SolEst     Damping    Stepsize #Res #Jac #Sol   LinErr   LinRes
   1       0.035   0.5000000       0.035   10    5    5  2.1e-15  7.9e-15
Displacement field
Iter      SolEst     Damping    Stepsize #Res #Jac #Sol   LinErr   LinRes
   1        0.17   0.5000000        0.17   10    5    5  7.3e-14  1.5e-12
Spatial mesh displacement
Iter      SolEst     Damping    Stepsize #Res #Jac #Sol   LinErr   LinRes
   1     5.9e-07   0.5000000     5.9e-07   10    5    5  1.5e-15  1.9e-15
Solution error estimates for segregated groups
0.31, 0.017, 0.084, 2.9e-07
Residual error estimates for segregated groups
33, 2e+03, 6.9e+03, 0.18
Segregated solver iteration 6.
Merged variables
Iter      SolEst     Damping    Stepsize #Res #Jac #Sol   LinErr   LinRes
   1        0.12   0.3500000        0.12   22   16   16  6.9e-14  2.9e-15
   2       0.079   0.3500000       0.079   23   17   17  5.7e-14  2.7e-15
   3       0.051   0.3500000       0.051   24   18   18  8.1e-14  2.7e-15
Velocity u2, Pressure p
Iter      SolEst     Damping    Stepsize #Res #Jac #Sol   LinErr   LinRes
   1       0.017   0.5000000       0.017   12    6    6  4.6e-15    8e-15
Displacement field
Iter      SolEst     Damping    Stepsize #Res #Jac #Sol   LinErr   LinRes
   1       0.095   0.5000000       0.095   12    6    6  6.6e-14  1.5e-12
Spatial mesh displacement
Iter      SolEst     Damping    Stepsize #Res #Jac #Sol   LinErr   LinRes
   1     4.1e-07   0.5000000     4.1e-07   12    6    6  1.6e-15  1.8e-15
Solution error estimates for segregated groups
0.088, 0.0085, 0.048, 2.1e-07
Residual error estimates for segregated groups
12, 1e+03, 4.2e+03, 0.17
```

```
Segregated solver iteration 7.
Merged variables
Iter      SolEst     Damping    Stepsize #Res #Jac #Sol   LinErr   LinRes
   1       0.034   0.3500000       0.034   26   19   19  7.7e-14  2.7e-15
   2       0.022   0.3500000       0.022   27   20   20  9.6e-14  2.6e-15
   3       0.014   0.3500000       0.014   28   21   21  5.5e-14  2.8e-15
Velocity u2, Pressure p
Iter      SolEst     Damping    Stepsize #Res #Jac #Sol   LinErr   LinRes
   1      0.0084   0.5000000      0.0084   14    7    7  2.1e-15    8e-15
Displacement field
Iter      SolEst     Damping    Stepsize #Res #Jac #Sol   LinErr   LinRes
   1       0.054   0.5000000       0.054   14    7    7  4.1e-14  1.5e-12
Spatial mesh displacement
Iter      SolEst     Damping    Stepsize #Res #Jac #Sol   LinErr   LinRes
   1     2.7e-07   0.5000000     2.7e-07   14    7    7  1.7e-15  1.8e-15
Solution error estimates for segregated groups
0.024, 0.0042, 0.027, 1.4e-07
Residual error estimates for segregated groups
4.1, 5.1e+02, 2.6e+03, 0.16
Segregated solver iteration 8.
Merged variables
Iter      SolEst     Damping    Stepsize #Res #Jac #Sol   LinErr   LinRes
   1      0.0092   0.3500000      0.0092   30   22   22  8.1e-14  2.7e-15
   2       0.006   0.3500000       0.006   31   23   23  5.3e-14  2.7e-15
   3      0.0039   0.3500000      0.0039   32   24   24    7e-14  2.8e-15
Velocity u2, Pressure p
Iter      SolEst     Damping    Stepsize #Res #Jac #Sol   LinErr   LinRes
   1      0.0042   0.5000000      0.0042   16    8    8  3.4e-15  8.1e-15
Displacement field
Iter      SolEst     Damping    Stepsize #Res #Jac #Sol   LinErr   LinRes
   1        0.03   0.5000000        0.03   16    8    8  1.2e-13  1.5e-12
Spatial mesh displacement
Iter      SolEst     Damping    Stepsize #Res #Jac #Sol   LinErr   LinRes
   1     1.8e-07   0.5000000     1.8e-07   16    8    8  1.4e-15  1.8e-15
Solution error estimates for segregated groups
0.0067, 0.0021, 0.015, 8.8e-08
Residual error estimates for segregated groups
1.4, 2.5e+02, 1.8e+03, 0.17
Segregated solver iteration 9.
Merged variables
Iter      SolEst     Damping    Stepsize #Res #Jac #Sol   LinErr   LinRes
   1      0.0025   0.3500000      0.0025   34   25   25  9.1e-14  2.7e-15
   2      0.0017   0.3500000      0.0017   35   26   26  5.2e-14  2.8e-15
   3      0.0011   0.3500000      0.0011   36   27   27  7.1e-14  2.8e-15
Velocity u2, Pressure p
Iter      SolEst     Damping    Stepsize #Res #Jac #Sol   LinErr   LinRes
   1      0.0021   0.5000000      0.0021   18    9    9  3.3e-15    8e-15
Displacement field
Iter      SolEst     Damping    Stepsize #Res #Jac #Sol   LinErr   LinRes
   1       0.017   0.5000000       0.017   18    9    9  5.7e-14  1.5e-12
Spatial mesh displacement
Iter      SolEst     Damping    Stepsize #Res #Jac #Sol   LinErr   LinRes
   1     1.1e-07   0.5000000     1.1e-07   18    9    9  1.2e-15  1.8e-15
Solution error estimates for segregated groups
0.0018, 0.001, 0.0085, 5.5e-08
Residual error estimates for segregated groups
```

```
0.44, 1.3e+02, 1.4e+03, 0.18
Segregated solver iteration 10.
Merged variables
Iter      SolEst     Damping    Stepsize #Res #Jac #Sol   LinErr   LinRes
   1      0.0007   0.3500000      0.0007   38   28   28  1.2e-13  2.7e-15
   2     0.00045   0.3500000     0.00045   39   29   29  4.5e-14  2.7e-15
   3      0.0003   0.3500000      0.0003   40   30   30  4.9e-14  2.8e-15
Velocity u2, Pressure p
Iter      SolEst     Damping    Stepsize #Res #Jac #Sol   LinErr   LinRes
   1       0.001   0.5000000       0.001   20   10   10  3.3e-15  7.9e-15
Displacement field
Iter      SolEst     Damping    Stepsize #Res #Jac #Sol   LinErr   LinRes
   1      0.0094   0.5000000      0.0094   20   10   10  9.1e-14  1.5e-12
Spatial mesh displacement
Iter      SolEst     Damping    Stepsize #Res #Jac #Sol   LinErr   LinRes
   1     6.7e-08   0.5000000     6.7e-08   20   10   10  2.2e-15  1.8e-15
Solution error estimates for segregated groups
0.00051, 0.00052, 0.0047, 3.4e-08
Residual error estimates for segregated groups
0.14, 64, 1.2e+03, 0.18
Segregated solver iteration 11.
Merged variables
Iter      SolEst     Damping    Stepsize #Res #Jac #Sol   LinErr   LinRes
   1     0.00019   0.3500000     0.00019   42   31   31  6.2e-14  2.7e-15
   2     0.00012   0.3500000     0.00012   43   32   32  7.4e-14  2.7e-15
   3     8.1e-05   0.3500000     8.1e-05   44   33   33  8.4e-14  2.6e-15
Velocity u2, Pressure p
Iter      SolEst     Damping    Stepsize #Res #Jac #Sol   LinErr   LinRes
   1     0.00052   0.5000000     0.00052   22   11   11  4.7e-15  7.9e-15
Displacement field
Iter      SolEst     Damping    Stepsize #Res #Jac #Sol   LinErr   LinRes
   1      0.0051   0.5000000      0.0051   22   11   11  2.6e-13  1.5e-12
Spatial mesh displacement
Iter      SolEst     Damping    Stepsize #Res #Jac #Sol   LinErr   LinRes
   1       4e-08   0.5000000       4e-08   22   11   11  1.4e-15  1.8e-15
Solution error estimates for segregated groups
0.00014, 0.00026, 0.0026, 2e-08
Residual error estimates for segregated groups
0.044, 32, 1.2e+03, 0.18
Segregated solver iteration 12.
Merged variables
Iter      SolEst     Damping    Stepsize #Res #Jac #Sol   LinErr   LinRes
   1     5.3e-05   0.3500000     5.3e-05   46   34   34  5.8e-14  2.8e-15
   2     3.4e-05   0.3500000     3.4e-05   47   35   35  5.6e-14  2.7e-15
   3     2.2e-05   0.3500000     2.2e-05   48   36   36  1.3e-13  2.6e-15
Velocity u2, Pressure p
Iter      SolEst     Damping    Stepsize #Res #Jac #Sol   LinErr   LinRes
   1     0.00026   0.5000000     0.00026   24   12   12  2.7e-15  7.9e-15
Displacement field
Iter      SolEst     Damping    Stepsize #Res #Jac #Sol   LinErr   LinRes
   1      0.0028   0.5000000      0.0028   24   12   12  4.7e-14  1.5e-12
Spatial mesh displacement
Iter      SolEst     Damping    Stepsize #Res #Jac #Sol   LinErr   LinRes
   1     2.4e-08   0.5000000     2.4e-08   24   12   12  1.3e-15  1.8e-15
Solution error estimates for segregated groups
3.8e-05, 0.00013, 0.0014, 1.2e-08
```

```
Residual error estimates for segregated groups
0.013, 16, 1.2e+03, 0.17
Segregated solver iteration 13.
Merged variables
Iter      SolEst      Damping    Stepsize #Res #Jac #Sol   LinErr   LinRes
   1      1.4e-05   0.3500000     1.4e-05   50   37   37  1.6e-13  2.7e-15
   2      9.4e-06   0.3500000     9.4e-06   51   38   38  7.6e-14  2.8e-15
   3      6.1e-06   0.3500000     6.1e-06   52   39   39  6.2e-14  2.8e-15
Velocity u2, Pressure p
Iter      SolEst      Damping    Stepsize #Res #Jac #Sol   LinErr   LinRes
   1      0.00013   0.5000000     0.00013   26   13   13  2.2e-15  7.8e-15
Displacement field
Iter      SolEst      Damping    Stepsize #Res #Jac #Sol   LinErr   LinRes
   1       0.0015   0.5000000      0.0015   26   13   13  5.8e-14  1.5e-12
Spatial mesh displacement
Iter      SolEst      Damping    Stepsize #Res #Jac #Sol   LinErr   LinRes
   1      1.4e-08   0.5000000     1.4e-08   26   13   13  1.4e-15  1.8e-15
Solution error estimates for segregated groups
1e-05, 6.5e-05, 0.00076, 7e-09
Residual error estimates for segregated groups
0.0041, 8, 1.2e+03, 0.19
Solution time: 155 s. (2 minutes, 35 seconds)
Physical memory: 4.66 GB
Virtual memory: 25.59 GB
Ended at Jan 31, 2025, 6:33:28 PM.
----- Stationary Solver 1 in study1/Approach Curve Solver (sol1) -------------->
```

d=2315 (su39)

GENERAL

Description	Value
Solution	d=2315

LOG

```
<---- Compile Equations: Stationary in study1/Approach Curve Solver (sol1) -----
Started at Jan 31, 2025, 6:33:28 PM.
Parameter d = 2.3150000000000003E-6 (m).
Geometry shape function: Linear serendipity
Number of vertex elements: 18
Number of boundary elements: 6237
Number of elements: 156337
Minimum element quality: 0.4793
Time: 8 s.
Physical memory: 2.88 GB
Virtual memory: 15.24 GB
Ended at Jan 31, 2025, 6:33:36 PM.
----- Compile Equations: Stationary in study1/Approach Curve Solver (sol1) ---->

<---- Dependent Variables 1 in study1/Approach Curve Solver (sol1) -------------
Started at Jan 31, 2025, 6:33:36 PM.
Solution time: 0 s.
Physical memory: 2.88 GB
Virtual memory: 15.18 GB
Ended at Jan 31, 2025, 6:33:36 PM.
----- Dependent Variables 1 in study1/Approach Curve Solver (sol1) ------------>

<---- Stationary Solver 1 in study1/Approach Curve Solver (sol1) ---------------
Started at Jan 31, 2025, 6:33:36 PM.
Segregated solver
Segregated Step 11 does not have any components solved for.
Number of degrees of freedom solved for: 794825 (plus 11897 internal DOFs).
Segregated solver iteration 1.
Merged variables
Nonsymmetric matrix found.
Scales for dependent variables:
Concentration (comp1.Cl): 1.4e+02
Concentration (comp1.K): 1.4e+02
Electric potential (comp1.V): 1.9e+02
Orthonormal null-space function used.
Iter      SolEst     Damping    Stepsize #Res #Jac #Sol   LinErr   LinRes
   1        0.83   0.3500000        0.83    2    1    1  1.7e-11  2.7e-15
   2        0.81   0.3500000        0.81    3    2    2  2.2e-11  2.7e-15
   3         0.8   0.3500000         0.8    4    3    3  2.4e-11  2.8e-15
Velocity u2, Pressure p
Nonsymmetric matrix found.
Scales for dependent variables:
Pressure (comp1.p): 1
Velocity field (spatial frame) (comp1.u2): 0.00018
Orthonormal null-space function used.
Iter      SolEst     Damping    Stepsize #Res #Jac #Sol   LinErr   LinRes
   1         1.1   0.5000000         1.1    2    1    1  1.5e-15  2.2e-15
Displacement field
Nonsymmetric matrix found.
Scales for dependent variables:
Auxiliary pressure (comp1.solid.lemm1.pw): 1
Displacement field (comp1.u): 2.8e-18
Orthonormal null-space function used.
Iter      SolEst     Damping    Stepsize #Res #Jac #Sol   LinErr   LinRes
   1         1.9   0.5000000         1.9    2    1    1  5.1e-14  1.5e-12
Spatial mesh displacement
```

```
Symmetric matrices found.
Scales for dependent variables:
Spatial mesh displacement (comp1.spatial.disp): 1.2e-09
Orthonormal null-space function used.
Iter      SolEst     Damping    Stepsize #Res #Jac #Sol   LinErr   LinRes
   1     6.3e-07   0.5000000     6.3e-07    2    1    1  1.3e-15  1.9e-15
Solution error estimates for segregated groups
1.4, 0.54, 0.95, 3.2e-07
Residual error estimates for segregated groups
7.9e+03, 8.3e+04, 2.2e+04, 0.15
Segregated solver iteration 2.
Merged variables
Scales for dependent variables:
Concentration (comp1.Cl): 1.4e+02
Concentration (comp1.K): 1.4e+02
Electric potential (comp1.V): 0.064
Iter      SolEst     Damping    Stepsize #Res #Jac #Sol   LinErr   LinRes
   1        0.79   0.3500000        0.79    6    4    4  8.2e-14  3.4e-15
   2        0.77   0.3500000        0.77    7    5    5  7.4e-14  4.2e-15
   3        0.76   0.3500000        0.76    8    6    6    1e-13  4.3e-15
Velocity u2, Pressure p
Scales for dependent variables:
Pressure (comp1.p): 3.6e+02
Velocity field (spatial frame) (comp1.u2): 0.00072
Iter      SolEst     Damping    Stepsize #Res #Jac #Sol   LinErr   LinRes
   1        0.36   0.5000000        0.36    4    2    2  3.2e-15  7.9e-15
Displacement field
Iter      SolEst     Damping    Stepsize #Res #Jac #Sol   LinErr   LinRes
   1        0.95   0.5000000        0.95    4    2    2  4.6e-14  1.5e-12
Spatial mesh displacement
Iter      SolEst     Damping    Stepsize #Res #Jac #Sol   LinErr   LinRes
   1     9.5e-07   0.5000000     9.5e-07    4    2    2  1.7e-15  1.9e-15
Solution error estimates for segregated groups
1.3, 0.18, 0.48, 4.8e-07
Residual error estimates for segregated groups
8e+02, 1.3e+04, 2.2e+04, 0.18
Segregated solver iteration 3.
Merged variables
Iter      SolEst     Damping    Stepsize #Res #Jac #Sol   LinErr   LinRes
   1        0.75   0.3500000        0.75   10    7    7  1.4e-13  5.5e-15
   2        0.73   0.3500000        0.73   11    8    8  1.1e-13  3.4e-15
   3        0.72   0.3500000        0.72   12    9    9  1.3e-13  4.3e-15
Velocity u2, Pressure p
Iter      SolEst     Damping    Stepsize #Res #Jac #Sol   LinErr   LinRes
   1        0.15   0.5000000        0.15    6    3    3  3.1e-15    8e-15
Displacement field
Iter      SolEst     Damping    Stepsize #Res #Jac #Sol   LinErr   LinRes
   1        0.52   0.5000000        0.52    6    3    3  1.6e-13  1.5e-12
Spatial mesh displacement
Iter      SolEst     Damping    Stepsize #Res #Jac #Sol   LinErr   LinRes
   1     9.5e-07   0.5000000     9.5e-07    6    3    3  1.4e-15  1.8e-15
Solution error estimates for segregated groups
1.2, 0.077, 0.26, 4.8e-07
Residual error estimates for segregated groups
2.2e+02, 7.6e+03, 1.6e+04, 0.17
Segregated solver iteration 4.
```

```
Merged variables
Iter      SolEst     Damping    Stepsize #Res #Jac #Sol   LinErr   LinRes
   1         0.7   0.3500000         0.7   14   10   10  1.1e-13  4.9e-15
   2        0.68   0.3500000        0.68   15   11   11  4.3e-14    4e-15
   3         0.6   0.3500000         0.6   16   12   12  1.1e-13  5.3e-15
Velocity u2, Pressure p
Iter      SolEst     Damping    Stepsize #Res #Jac #Sol   LinErr   LinRes
   1       0.072   0.5000000       0.072    8    4    4  2.7e-15  7.9e-15
Displacement field
Iter      SolEst     Damping    Stepsize #Res #Jac #Sol   LinErr   LinRes
   1        0.29   0.5000000        0.29    8    4    4  8.2e-14  1.5e-12
Spatial mesh displacement
Iter      SolEst     Damping    Stepsize #Res #Jac #Sol   LinErr   LinRes
   1     7.9e-07   0.5000000     7.9e-07    8    4    4  2.4e-15  1.9e-15
Solution error estimates for segregated groups
1, 0.036, 0.15, 4e-07
Residual error estimates for segregated groups
84, 4e+03, 1.1e+04, 0.17
Segregated solver iteration 5.
Merged variables
Iter      SolEst     Damping    Stepsize #Res #Jac #Sol   LinErr   LinRes
   1        0.41   0.3500000        0.41   18   13   13  7.7e-14  4.5e-15
   2        0.28   0.3500000        0.28   19   14   14  8.6e-14  4.2e-15
   3        0.18   0.3500000        0.18   20   15   15  7.4e-14  4.4e-15
Velocity u2, Pressure p
Iter      SolEst     Damping    Stepsize #Res #Jac #Sol   LinErr   LinRes
   1       0.035   0.5000000       0.035   10    5    5  2.2e-15  7.8e-15
Displacement field
Iter      SolEst     Damping    Stepsize #Res #Jac #Sol   LinErr   LinRes
   1        0.17   0.5000000        0.17   10    5    5  1.9e-13  1.5e-12
Spatial mesh displacement
Iter      SolEst     Damping    Stepsize #Res #Jac #Sol   LinErr   LinRes
   1     5.9e-07   0.5000000     5.9e-07   10    5    5  1.9e-15  1.8e-15
Solution error estimates for segregated groups
0.31, 0.017, 0.084, 3e-07
Residual error estimates for segregated groups
33, 2e+03, 6.9e+03, 0.17
Segregated solver iteration 6.
Merged variables
Iter      SolEst     Damping    Stepsize #Res #Jac #Sol   LinErr   LinRes
   1        0.12   0.3500000        0.12   22   16   16  1.4e-13  5.2e-15
   2       0.079   0.3500000       0.079   23   17   17  8.8e-14  5.3e-15
   3       0.051   0.3500000       0.051   24   18   18  7.2e-14  3.4e-15
Velocity u2, Pressure p
Iter      SolEst     Damping    Stepsize #Res #Jac #Sol   LinErr   LinRes
   1       0.017   0.5000000       0.017   12    6    6  3.1e-15  7.8e-15
Displacement field
Iter      SolEst     Damping    Stepsize #Res #Jac #Sol   LinErr   LinRes
   1       0.095   0.5000000       0.095   12    6    6  7.9e-14  1.5e-12
Spatial mesh displacement
Iter      SolEst     Damping    Stepsize #Res #Jac #Sol   LinErr   LinRes
   1     4.2e-07   0.5000000     4.2e-07   12    6    6  1.8e-15  1.9e-15
Solution error estimates for segregated groups
0.088, 0.0085, 0.048, 2.1e-07
Residual error estimates for segregated groups
12, 1e+03, 4.2e+03, 0.18
```

```
Segregated solver iteration 7.
Merged variables
Iter      SolEst     Damping    Stepsize #Res #Jac #Sol   LinErr   LinRes
   1       0.033   0.3500000       0.033   26   19   19  4.2e-14  3.5e-15
   2       0.022   0.3500000       0.022   27   20   20  7.9e-14  4.9e-15
   3       0.014   0.3500000       0.014   28   21   21  1.2e-13  3.6e-15
Velocity u2, Pressure p
Iter      SolEst     Damping    Stepsize #Res #Jac #Sol   LinErr   LinRes
   1      0.0084   0.5000000      0.0084   14    7    7  2.8e-15  7.8e-15
Displacement field
Iter      SolEst     Damping    Stepsize #Res #Jac #Sol   LinErr   LinRes
   1       0.054   0.5000000       0.054   14    7    7  5.8e-14  1.5e-12
Spatial mesh displacement
Iter      SolEst     Damping    Stepsize #Res #Jac #Sol   LinErr   LinRes
   1     2.8e-07   0.5000000     2.8e-07   14    7    7  1.7e-15  1.9e-15
Solution error estimates for segregated groups
0.024, 0.0042, 0.027, 1.4e-07
Residual error estimates for segregated groups
4.1, 5.2e+02, 2.6e+03, 0.18
Segregated solver iteration 8.
Merged variables
Iter      SolEst     Damping    Stepsize #Res #Jac #Sol   LinErr   LinRes
   1      0.0092   0.3500000      0.0092   30   22   22  3.9e-14  3.7e-15
   2       0.006   0.3500000       0.006   31   23   23  6.3e-14  3.5e-15
   3      0.0039   0.3500000      0.0039   32   24   24  9.8e-14  4.5e-15
Velocity u2, Pressure p
Iter      SolEst     Damping    Stepsize #Res #Jac #Sol   LinErr   LinRes
   1      0.0042   0.5000000      0.0042   16    8    8  4.6e-15    8e-15
Displacement field
Iter      SolEst     Damping    Stepsize #Res #Jac #Sol   LinErr   LinRes
   1        0.03   0.5000000        0.03   16    8    8  1.2e-13  1.5e-12
Spatial mesh displacement
Iter      SolEst     Damping    Stepsize #Res #Jac #Sol   LinErr   LinRes
   1     1.8e-07   0.5000000     1.8e-07   16    8    8  1.2e-15  1.9e-15
Solution error estimates for segregated groups
0.0067, 0.0021, 0.015, 8.9e-08
Residual error estimates for segregated groups
1.4, 2.6e+02, 1.8e+03, 0.17
Segregated solver iteration 9.
Merged variables
Iter      SolEst     Damping    Stepsize #Res #Jac #Sol   LinErr   LinRes
   1      0.0025   0.3500000      0.0025   34   25   25  9.1e-14  5.6e-15
   2      0.0017   0.3500000      0.0017   35   26   26  8.6e-14  5.3e-15
   3      0.0011   0.3500000      0.0011   36   27   27  4.5e-14  4.4e-15
Velocity u2, Pressure p
Iter      SolEst     Damping    Stepsize #Res #Jac #Sol   LinErr   LinRes
   1      0.0021   0.5000000      0.0021   18    9    9  5.3e-15  7.9e-15
Displacement field
Iter      SolEst     Damping    Stepsize #Res #Jac #Sol   LinErr   LinRes
   1       0.017   0.5000000       0.017   18    9    9  8.6e-14  1.5e-12
Spatial mesh displacement
Iter      SolEst     Damping    Stepsize #Res #Jac #Sol   LinErr   LinRes
   1     1.1e-07   0.5000000     1.1e-07   18    9    9  1.4e-15  1.8e-15
Solution error estimates for segregated groups
0.0018, 0.001, 0.0085, 5.6e-08
Residual error estimates for segregated groups
```

```
0.44, 1.3e+02, 1.4e+03, 0.17
Segregated solver iteration 10.
Merged variables
Iter      SolEst     Damping    Stepsize #Res #Jac #Sol   LinErr   LinRes
   1      0.0007   0.3500000      0.0007   38   28   28  7.8e-14  3.4e-15
   2     0.00045   0.3500000     0.00045   39   29   29  4.5e-14  3.7e-15
   3     0.00029   0.3500000     0.00029   40   30   30  9.7e-14  5.8e-15
Velocity u2, Pressure p
Iter      SolEst     Damping    Stepsize #Res #Jac #Sol   LinErr   LinRes
   1       0.001   0.5000000       0.001   20   10   10    5e-15  7.8e-15
Displacement field
Iter      SolEst     Damping    Stepsize #Res #Jac #Sol   LinErr   LinRes
   1      0.0094   0.5000000      0.0094   20   10   10  1.1e-13  1.5e-12
Spatial mesh displacement
Iter      SolEst     Damping    Stepsize #Res #Jac #Sol   LinErr   LinRes
   1     6.8e-08   0.5000000     6.8e-08   20   10   10  1.5e-15  1.8e-15
Solution error estimates for segregated groups
0.00051, 0.00052, 0.0047, 3.4e-08
Residual error estimates for segregated groups
0.14, 65, 1.2e+03, 0.17
Segregated solver iteration 11.
Merged variables
Iter      SolEst     Damping    Stepsize #Res #Jac #Sol   LinErr   LinRes
   1     0.00019   0.3500000     0.00019   42   31   31  1.3e-13  5.9e-15
   2     0.00012   0.3500000     0.00012   43   32   32  8.9e-14    4e-15
   3     8.1e-05   0.3500000     8.1e-05   44   33   33  8.1e-14  4.2e-15
Velocity u2, Pressure p
Iter      SolEst     Damping    Stepsize #Res #Jac #Sol   LinErr   LinRes
   1     0.00052   0.5000000     0.00052   22   11   11  3.5e-15  7.8e-15
Displacement field
Iter      SolEst     Damping    Stepsize #Res #Jac #Sol   LinErr   LinRes
   1      0.0051   0.5000000      0.0051   22   11   11  9.9e-14  1.5e-12
Spatial mesh displacement
Iter      SolEst     Damping    Stepsize #Res #Jac #Sol   LinErr   LinRes
   1     4.1e-08   0.5000000     4.1e-08   22   11   11  1.4e-15  1.9e-15
Solution error estimates for segregated groups
0.00014, 0.00026, 0.0026, 2e-08
Residual error estimates for segregated groups
0.044, 33, 1.2e+03, 0.17
Segregated solver iteration 12.
Merged variables
Iter      SolEst     Damping    Stepsize #Res #Jac #Sol   LinErr   LinRes
   1     5.3e-05   0.3500000     5.3e-05   46   34   34  8.6e-14  3.8e-15
   2     3.4e-05   0.3500000     3.4e-05   47   35   35    1e-13  5.1e-15
   3     2.2e-05   0.3500000     2.2e-05   48   36   36  1.1e-13  4.4e-15
Velocity u2, Pressure p
Iter      SolEst     Damping    Stepsize #Res #Jac #Sol   LinErr   LinRes
   1     0.00026   0.5000000     0.00026   24   12   12  3.1e-15  7.9e-15
Displacement field
Iter      SolEst     Damping    Stepsize #Res #Jac #Sol   LinErr   LinRes
   1      0.0028   0.5000000      0.0028   24   12   12  3.8e-14  1.5e-12
Spatial mesh displacement
Iter      SolEst     Damping    Stepsize #Res #Jac #Sol   LinErr   LinRes
   1     2.4e-08   0.5000000     2.4e-08   24   12   12  1.5e-15  1.8e-15
Solution error estimates for segregated groups
3.8e-05, 0.00013, 0.0014, 1.2e-08
```

```
Residual error estimates for segregated groups
0.013, 16, 1.2e+03, 0.18
Segregated solver iteration 13.
Merged variables
Iter      SolEst     Damping    Stepsize #Res #Jac #Sol   LinErr   LinRes
   1     1.4e-05   0.3500000     1.4e-05   50   37   37  1.5e-13  3.2e-15
   2     9.4e-06   0.3500000     9.4e-06   51   38   38    1e-13  4.5e-15
   3     6.1e-06   0.3500000     6.1e-06   52   39   39  8.2e-14    4e-15
Velocity u2, Pressure p
Iter      SolEst     Damping    Stepsize #Res #Jac #Sol   LinErr   LinRes
   1     0.00013   0.5000000     0.00013   26   13   13  2.2e-15  7.7e-15
Displacement field
Iter      SolEst     Damping    Stepsize #Res #Jac #Sol   LinErr   LinRes
   1      0.0015   0.5000000      0.0015   26   13   13  6.6e-14  1.5e-12
Spatial mesh displacement
Iter      SolEst     Damping    Stepsize #Res #Jac #Sol   LinErr   LinRes
   1     1.4e-08   0.5000000     1.4e-08   26   13   13  1.5e-15  1.9e-15
Solution error estimates for segregated groups
1e-05, 6.5e-05, 0.00076, 7e-09
Residual error estimates for segregated groups
0.004, 8.2, 1.2e+03, 0.18
Solution time: 155 s. (2 minutes, 35 seconds)
Physical memory: 4.66 GB
Virtual memory: 25.52 GB
Ended at Jan 31, 2025, 6:36:12 PM.
----- Stationary Solver 1 in study1/Approach Curve Solver (sol1) -------------->
```

d=2310 (su40)

GENERAL

Description	Value
Solution	d=2310

LOG

```
<---- Compile Equations: Stationary in study1/Approach Curve Solver (sol1) -----
Started at Jan 31, 2025, 6:36:12 PM.
Parameter d = 2.3100000000000003E-6 (m).
Geometry shape function: Linear serendipity
Number of vertex elements: 18
Number of boundary elements: 6234
Number of elements: 156110
Minimum element quality: 0.4797
Time: 8 s.
Physical memory: 2.94 GB
Virtual memory: 15.3 GB
Ended at Jan 31, 2025, 6:36:20 PM.
----- Compile Equations: Stationary in study1/Approach Curve Solver (sol1) ---->

<---- Dependent Variables 1 in study1/Approach Curve Solver (sol1) -------------
Started at Jan 31, 2025, 6:36:20 PM.
Solution time: 0 s.
Physical memory: 2.94 GB
Virtual memory: 15.24 GB
Ended at Jan 31, 2025, 6:36:20 PM.
----- Dependent Variables 1 in study1/Approach Curve Solver (sol1) ------------>

<---- Stationary Solver 1 in study1/Approach Curve Solver (sol1) ---------------
Started at Jan 31, 2025, 6:36:21 PM.
Segregated solver
Segregated Step 11 does not have any components solved for.
Number of degrees of freedom solved for: 793569 (plus 11891 internal DOFs).
Segregated solver iteration 1.
Merged variables
Nonsymmetric matrix found.
Scales for dependent variables:
Concentration (comp1.Cl): 1.4e+02
Concentration (comp1.K): 1.4e+02
Electric potential (comp1.V): 1.9e+02
Orthonormal null-space function used.
Iter      SolEst     Damping    Stepsize #Res #Jac #Sol   LinErr   LinRes
   1        0.83   0.3500000        0.83    2    1    1  6.3e-11  2.7e-15
   2        0.81   0.3500000        0.81    3    2    2  3.9e-11  2.7e-15
   3         0.8   0.3500000         0.8    4    3    3  3.8e-11  2.7e-15
Velocity u2, Pressure p
Nonsymmetric matrix found.
Scales for dependent variables:
Pressure (comp1.p): 1
Velocity field (spatial frame) (comp1.u2): 0.00018
Orthonormal null-space function used.
Iter      SolEst     Damping    Stepsize #Res #Jac #Sol   LinErr   LinRes
   1         1.1   0.5000000         1.1    2    1    1  1.6e-15  2.2e-15
Displacement field
Nonsymmetric matrix found.
Scales for dependent variables:
Auxiliary pressure (comp1.solid.lemm1.pw): 1
Displacement field (comp1.u): 2.8e-18
Orthonormal null-space function used.
Iter      SolEst     Damping    Stepsize #Res #Jac #Sol   LinErr   LinRes
   1         1.9   0.5000000         1.9    2    1    1    8e-14  1.5e-12
Spatial mesh displacement
```

```
Symmetric matrices found.
Scales for dependent variables:
Spatial mesh displacement (comp1.spatial.disp): 1.2e-09
Orthonormal null-space function used.
Iter      SolEst     Damping    Stepsize #Res #Jac #Sol   LinErr   LinRes
   1     6.4e-07   0.5000000     6.4e-07    2    1    1  1.5e-15  1.8e-15
Solution error estimates for segregated groups
1.4, 0.54, 0.95, 3.2e-07
Residual error estimates for segregated groups
7.9e+03, 8.3e+04, 2.2e+04, 0.15
Segregated solver iteration 2.
Merged variables
Scales for dependent variables:
Concentration (comp1.Cl): 1.4e+02
Concentration (comp1.K): 1.4e+02
Electric potential (comp1.V): 0.064
Iter      SolEst     Damping    Stepsize #Res #Jac #Sol   LinErr   LinRes
   1        0.78   0.3500000        0.78    6    4    4  5.6e-14  1.2e-14
   2        0.77   0.3500000        0.77    7    5    5  8.8e-14  5.9e-15
   3        0.76   0.3500000        0.76    8    6    6  5.3e-14  3.3e-15
Velocity u2, Pressure p
Scales for dependent variables:
Pressure (comp1.p): 3.6e+02
Velocity field (spatial frame) (comp1.u2): 0.00072
Iter      SolEst     Damping    Stepsize #Res #Jac #Sol   LinErr   LinRes
   1        0.36   0.5000000        0.36    4    2    2  1.7e-15  7.9e-15
Displacement field
Iter      SolEst     Damping    Stepsize #Res #Jac #Sol   LinErr   LinRes
   1        0.95   0.5000000        0.95    4    2    2    2e-13  1.5e-12
Spatial mesh displacement
Iter      SolEst     Damping    Stepsize #Res #Jac #Sol   LinErr   LinRes
   1     9.6e-07   0.5000000     9.6e-07    4    2    2  1.6e-15  1.9e-15
Solution error estimates for segregated groups
1.3, 0.18, 0.48, 4.8e-07
Residual error estimates for segregated groups
8e+02, 1.5e+04, 2.2e+04, 0.17
Segregated solver iteration 3.
Merged variables
Iter      SolEst     Damping    Stepsize #Res #Jac #Sol   LinErr   LinRes
   1        0.75   0.3500000        0.75   10    7    7  1.1e-13  3.9e-15
   2        0.73   0.3500000        0.73   11    8    8    7e-14  3.9e-15
   3        0.72   0.3500000        0.72   12    9    9  3.3e-14    6e-15
Velocity u2, Pressure p
Iter      SolEst     Damping    Stepsize #Res #Jac #Sol   LinErr   LinRes
   1        0.15   0.5000000        0.15    6    3    3  3.8e-15  7.9e-15
Displacement field
Iter      SolEst     Damping    Stepsize #Res #Jac #Sol   LinErr   LinRes
   1        0.52   0.5000000        0.52    6    3    3  9.5e-14  1.5e-12
Spatial mesh displacement
Iter      SolEst     Damping    Stepsize #Res #Jac #Sol   LinErr   LinRes
   1     9.6e-07   0.5000000     9.6e-07    6    3    3  1.4e-15  1.8e-15
Solution error estimates for segregated groups
1.2, 0.077, 0.26, 4.8e-07
Residual error estimates for segregated groups
2.2e+02, 8.2e+03, 1.6e+04, 0.18
Segregated solver iteration 4.
```

```
Merged variables
Iter      SolEst     Damping    Stepsize #Res #Jac #Sol   LinErr   LinRes
   1         0.7   0.3500000         0.7   14   10   10  6.2e-14    7e-15
   2        0.68   0.3500000        0.68   15   11   11  7.8e-14  8.2e-15
   3         0.6   0.3500000         0.6   16   12   12  8.7e-14  4.8e-15
Velocity u2, Pressure p
Iter      SolEst     Damping    Stepsize #Res #Jac #Sol   LinErr   LinRes
   1       0.072   0.5000000       0.072    8    4    4  2.9e-15  7.8e-15
Displacement field
Iter      SolEst     Damping    Stepsize #Res #Jac #Sol   LinErr   LinRes
   1        0.29   0.5000000        0.29    8    4    4  1.6e-13  1.5e-12
Spatial mesh displacement
Iter      SolEst     Damping    Stepsize #Res #Jac #Sol   LinErr   LinRes
   1       8e-07   0.5000000       8e-07    8    4    4  1.4e-15  1.9e-15
Solution error estimates for segregated groups
1, 0.036, 0.15, 4e-07
Residual error estimates for segregated groups
84, 4.3e+03, 1.1e+04, 0.18
Segregated solver iteration 5.
Merged variables
Iter      SolEst     Damping    Stepsize #Res #Jac #Sol   LinErr   LinRes
   1        0.41   0.3500000        0.41   18   13   13  6.2e-14  6.8e-15
   2        0.28   0.3500000        0.28   19   14   14  6.9e-14  4.2e-15
   3        0.18   0.3500000        0.18   20   15   15  9.6e-14  4.8e-15
Velocity u2, Pressure p
Iter      SolEst     Damping    Stepsize #Res #Jac #Sol   LinErr   LinRes
   1       0.035   0.5000000       0.035   10    5    5  5.3e-15  7.9e-15
Displacement field
Iter      SolEst     Damping    Stepsize #Res #Jac #Sol   LinErr   LinRes
   1        0.17   0.5000000        0.17   10    5    5  1.3e-13  1.5e-12
Spatial mesh displacement
Iter      SolEst     Damping    Stepsize #Res #Jac #Sol   LinErr   LinRes
   1       6e-07   0.5000000       6e-07   10    5    5  1.6e-15  1.9e-15
Solution error estimates for segregated groups
0.31, 0.017, 0.084, 3e-07
Residual error estimates for segregated groups
33, 2.2e+03, 6.9e+03, 0.17
Segregated solver iteration 6.
Merged variables
Iter      SolEst     Damping    Stepsize #Res #Jac #Sol   LinErr   LinRes
   1        0.12   0.3500000        0.12   22   16   16  1.1e-13  3.7e-15
   2       0.079   0.3500000       0.079   23   17   17  6.6e-14  4.5e-15
   3       0.051   0.3500000       0.051   24   18   18    7e-14  1.1e-14
Velocity u2, Pressure p
Iter      SolEst     Damping    Stepsize #Res #Jac #Sol   LinErr   LinRes
   1       0.017   0.5000000       0.017   12    6    6  4.9e-15  7.9e-15
Displacement field
Iter      SolEst     Damping    Stepsize #Res #Jac #Sol   LinErr   LinRes
   1       0.095   0.5000000       0.095   12    6    6  4.4e-14  1.5e-12
Spatial mesh displacement
Iter      SolEst     Damping    Stepsize #Res #Jac #Sol   LinErr   LinRes
   1     4.2e-07   0.5000000     4.2e-07   12    6    6  1.8e-15  1.8e-15
Solution error estimates for segregated groups
0.088, 0.0085, 0.048, 2.1e-07
Residual error estimates for segregated groups
12, 1.1e+03, 4.2e+03, 0.18
```

```
Segregated solver iteration 7.
Merged variables
Iter      SolEst     Damping    Stepsize #Res #Jac #Sol   LinErr   LinRes
   1       0.033   0.3500000       0.033   26   19   19  7.4e-14  5.3e-15
   2       0.022   0.3500000       0.022   27   20   20  1.2e-13  4.3e-15
   3       0.014   0.3500000       0.014   28   21   21  6.9e-14  4.4e-15
Velocity u2, Pressure p
Iter      SolEst     Damping    Stepsize #Res #Jac #Sol   LinErr   LinRes
   1      0.0084   0.5000000      0.0084   14    7    7  3.8e-15  7.9e-15
Displacement field
Iter      SolEst     Damping    Stepsize #Res #Jac #Sol   LinErr   LinRes
   1       0.054   0.5000000       0.054   14    7    7  2.8e-14  1.6e-12
Spatial mesh displacement
Iter      SolEst     Damping    Stepsize #Res #Jac #Sol   LinErr   LinRes
   1     2.8e-07   0.5000000     2.8e-07   14    7    7  1.4e-15  1.8e-15
Solution error estimates for segregated groups
0.024, 0.0042, 0.027, 1.4e-07
Residual error estimates for segregated groups
4.1, 5.7e+02, 2.6e+03, 0.19
Segregated solver iteration 8.
Merged variables
Iter      SolEst     Damping    Stepsize #Res #Jac #Sol   LinErr   LinRes
   1      0.0092   0.3500000      0.0092   30   22   22  5.3e-14  5.6e-15
   2       0.006   0.3500000       0.006   31   23   23  8.3e-14  6.4e-15
   3      0.0039   0.3500000      0.0039   32   24   24  6.7e-14  4.1e-15
Velocity u2, Pressure p
Iter      SolEst     Damping    Stepsize #Res #Jac #Sol   LinErr   LinRes
   1      0.0042   0.5000000      0.0042   16    8    8  2.9e-15  7.8e-15
Displacement field
Iter      SolEst     Damping    Stepsize #Res #Jac #Sol   LinErr   LinRes
   1        0.03   0.5000000        0.03   16    8    8    6e-14  1.5e-12
Spatial mesh displacement
Iter      SolEst     Damping    Stepsize #Res #Jac #Sol   LinErr   LinRes
   1     1.8e-07   0.5000000     1.8e-07   16    8    8  1.5e-15  1.8e-15
Solution error estimates for segregated groups
0.0067, 0.0021, 0.015, 9e-08
Residual error estimates for segregated groups
1.4, 2.8e+02, 1.8e+03, 0.18
Segregated solver iteration 9.
Merged variables
Iter      SolEst     Damping    Stepsize #Res #Jac #Sol   LinErr   LinRes
   1      0.0025   0.3500000      0.0025   34   25   25  1.4e-13  4.2e-15
   2      0.0016   0.3500000      0.0016   35   26   26  1.1e-13  8.3e-15
   3      0.0011   0.3500000      0.0011   36   27   27  6.7e-14  3.5e-15
Velocity u2, Pressure p
Iter      SolEst     Damping    Stepsize #Res #Jac #Sol   LinErr   LinRes
   1      0.0021   0.5000000      0.0021   18    9    9  2.3e-15  7.9e-15
Displacement field
Iter      SolEst     Damping    Stepsize #Res #Jac #Sol   LinErr   LinRes
   1       0.017   0.5000000       0.017   18    9    9  5.3e-14  1.5e-12
Spatial mesh displacement
Iter      SolEst     Damping    Stepsize #Res #Jac #Sol   LinErr   LinRes
   1     1.1e-07   0.5000000     1.1e-07   18    9    9  1.3e-15  1.8e-15
Solution error estimates for segregated groups
0.0018, 0.001, 0.0085, 5.6e-08
Residual error estimates for segregated groups
```

```
0.44, 1.4e+02, 1.4e+03, 0.17
Segregated solver iteration 10.
Merged variables
Iter      SolEst     Damping    Stepsize #Res #Jac #Sol   LinErr   LinRes
   1      0.0007   0.3500000      0.0007   38   28   28  5.4e-14  9.1e-15
   2     0.00045   0.3500000     0.00045   39   29   29  1.1e-13  4.9e-15
   3     0.00029   0.3500000     0.00029   40   30   30  6.5e-14  4.6e-15
Velocity u2, Pressure p
Iter      SolEst     Damping    Stepsize #Res #Jac #Sol   LinErr   LinRes
   1       0.001   0.5000000       0.001   20   10   10    3e-15  7.8e-15
Displacement field
Iter      SolEst     Damping    Stepsize #Res #Jac #Sol   LinErr   LinRes
   1      0.0094   0.5000000      0.0094   20   10   10  7.9e-14  1.5e-12
Spatial mesh displacement
Iter      SolEst     Damping    Stepsize #Res #Jac #Sol   LinErr   LinRes
   1     6.9e-08   0.5000000     6.9e-08   20   10   10  1.9e-15  1.9e-15
Solution error estimates for segregated groups
0.0005, 0.00052, 0.0047, 3.4e-08
Residual error estimates for segregated groups
0.14, 71, 1.2e+03, 0.18
Segregated solver iteration 11.
Merged variables
Iter      SolEst     Damping    Stepsize #Res #Jac #Sol   LinErr   LinRes
   1     0.00019   0.3500000     0.00019   42   31   31  4.7e-14    4e-15
   2     0.00012   0.3500000     0.00012   43   32   32    1e-13  3.9e-15
   3     8.1e-05   0.3500000     8.1e-05   44   33   33  8.4e-14  3.9e-15
Velocity u2, Pressure p
Iter      SolEst     Damping    Stepsize #Res #Jac #Sol   LinErr   LinRes
   1     0.00052   0.5000000     0.00052   22   11   11  5.4e-15  7.9e-15
Displacement field
Iter      SolEst     Damping    Stepsize #Res #Jac #Sol   LinErr   LinRes
   1      0.0051   0.5000000      0.0051   22   11   11  4.2e-14  1.5e-12
Spatial mesh displacement
Iter      SolEst     Damping    Stepsize #Res #Jac #Sol   LinErr   LinRes
   1     4.1e-08   0.5000000     4.1e-08   22   11   11  2.2e-15  1.9e-15
Solution error estimates for segregated groups
0.00014, 0.00026, 0.0026, 2.1e-08
Residual error estimates for segregated groups
0.044, 36, 1.2e+03, 0.17
Segregated solver iteration 12.
Merged variables
Iter      SolEst     Damping    Stepsize #Res #Jac #Sol   LinErr   LinRes
   1     5.2e-05   0.3500000     5.2e-05   46   34   34  5.9e-14  6.7e-15
   2     3.4e-05   0.3500000     3.4e-05   47   35   35  7.1e-14  5.4e-15
   3     2.2e-05   0.3500000     2.2e-05   48   36   36  7.3e-14  3.9e-15
Velocity u2, Pressure p
Iter      SolEst     Damping    Stepsize #Res #Jac #Sol   LinErr   LinRes
   1     0.00026   0.5000000     0.00026   24   12   12  2.1e-15  7.8e-15
Displacement field
Iter      SolEst     Damping    Stepsize #Res #Jac #Sol   LinErr   LinRes
   1      0.0028   0.5000000      0.0028   24   12   12  9.7e-14  1.5e-12
Spatial mesh displacement
Iter      SolEst     Damping    Stepsize #Res #Jac #Sol   LinErr   LinRes
   1     2.4e-08   0.5000000     2.4e-08   24   12   12  1.5e-15  1.9e-15
Solution error estimates for segregated groups
3.8e-05, 0.00013, 0.0014, 1.2e-08
```

```
Residual error estimates for segregated groups
0.013, 18, 1.2e+03, 0.17
Segregated solver iteration 13.
Merged variables
Iter      SolEst     Damping    Stepsize #Res #Jac #Sol   LinErr   LinRes
   1     1.4e-05   0.3500000     1.4e-05   50   37   37  1.4e-13    8e-15
   2     9.4e-06   0.3500000     9.4e-06   51   38   38  7.5e-14  4.7e-15
   3     6.1e-06   0.3500000     6.1e-06   52   39   39  6.4e-14  6.8e-15
Velocity u2, Pressure p
Iter      SolEst     Damping    Stepsize #Res #Jac #Sol   LinErr   LinRes
   1     0.00013   0.5000000     0.00013   26   13   13  4.2e-15  7.7e-15
Displacement field
Iter      SolEst     Damping    Stepsize #Res #Jac #Sol   LinErr   LinRes
   1      0.0015   0.5000000      0.0015   26   13   13  4.8e-14  1.5e-12
Spatial mesh displacement
Iter      SolEst     Damping    Stepsize #Res #Jac #Sol   LinErr   LinRes
   1     1.4e-08   0.5000000     1.4e-08   26   13   13  1.9e-15  1.8e-15
Solution error estimates for segregated groups
1e-05, 6.5e-05, 0.00076, 7.1e-09
Residual error estimates for segregated groups
0.004, 8.9, 1.2e+03, 0.17
Solution time: 159 s. (2 minutes, 39 seconds)
Physical memory: 4.65 GB
Virtual memory: 25.53 GB
Ended at Jan 31, 2025, 6:39:00 PM.
----- Stationary Solver 1 in study1/Approach Curve Solver (sol1) -------------->
```

d=2305 (su41)

GENERAL

Description	Value
Solution	d=2305

LOG

```
<---- Compile Equations: Stationary in study1/Approach Curve Solver (sol1) -----
Started at Jan 31, 2025, 6:39:00 PM.
Parameter d = 2.305E-6 (m).
Geometry shape function: Linear serendipity
Number of vertex elements: 18
Number of boundary elements: 6232
Number of elements: 155938
Minimum element quality: 0.4808
Time: 8 s.
Physical memory: 2.89 GB
Virtual memory: 15.18 GB
Ended at Jan 31, 2025, 6:39:08 PM.
----- Compile Equations: Stationary in study1/Approach Curve Solver (sol1) ---->

<---- Dependent Variables 1 in study1/Approach Curve Solver (sol1) -------------
Started at Jan 31, 2025, 6:39:08 PM.
Solution time: 0 s.
Physical memory: 2.89 GB
Virtual memory: 15.18 GB
Ended at Jan 31, 2025, 6:39:08 PM.
----- Dependent Variables 1 in study1/Approach Curve Solver (sol1) ------------>

<---- Stationary Solver 1 in study1/Approach Curve Solver (sol1) ---------------
Started at Jan 31, 2025, 6:39:09 PM.
Segregated solver
Segregated Step 11 does not have any components solved for.
Number of degrees of freedom solved for: 792802 (plus 11887 internal DOFs).
Segregated solver iteration 1.
Merged variables
Nonsymmetric matrix found.
Scales for dependent variables:
Concentration (comp1.Cl): 1.4e+02
Concentration (comp1.K): 1.4e+02
Electric potential (comp1.V): 1.9e+02
Orthonormal null-space function used.
Iter      SolEst     Damping    Stepsize #Res #Jac #Sol   LinErr   LinRes
   1        0.83   0.3500000        0.83    2    1    1  3.6e-11  2.7e-15
   2        0.81   0.3500000        0.81    3    2    2  2.2e-11  2.7e-15
   3         0.8   0.3500000         0.8    4    3    3  5.3e-11  2.7e-15
Velocity u2, Pressure p
Nonsymmetric matrix found.
Scales for dependent variables:
Pressure (comp1.p): 1
Velocity field (spatial frame) (comp1.u2): 0.00018
Orthonormal null-space function used.
Iter      SolEst     Damping    Stepsize #Res #Jac #Sol   LinErr   LinRes
   1         1.1   0.5000000         1.1    2    1    1  1.5e-15  2.2e-15
Displacement field
Nonsymmetric matrix found.
Scales for dependent variables:
Auxiliary pressure (comp1.solid.lemm1.pw): 1
Displacement field (comp1.u): 2.8e-18
Orthonormal null-space function used.
Iter      SolEst     Damping    Stepsize #Res #Jac #Sol   LinErr   LinRes
   1         1.9   0.5000000         1.9    2    1    1  5.8e-14  1.5e-12
Spatial mesh displacement
```

```
Symmetric matrices found.
Scales for dependent variables:
Spatial mesh displacement (comp1.spatial.disp): 1.2e-09
Orthonormal null-space function used.
Iter      SolEst     Damping    Stepsize #Res #Jac #Sol   LinErr   LinRes
   1     6.4e-07   0.5000000     6.4e-07    2    1    1  1.5e-15  1.8e-15
Solution error estimates for segregated groups
1.4, 0.54, 0.95, 3.2e-07
Residual error estimates for segregated groups
7.9e+03, 8.3e+04, 2.2e+04, 0.15
Segregated solver iteration 2.
Merged variables
Scales for dependent variables:
Concentration (comp1.Cl): 1.4e+02
Concentration (comp1.K): 1.4e+02
Electric potential (comp1.V): 0.064
Iter      SolEst     Damping    Stepsize #Res #Jac #Sol   LinErr   LinRes
   1        0.78   0.3500000        0.78    6    4    4  6.2e-14  2.7e-15
   2        0.77   0.3500000        0.77    7    5    5  6.2e-14  2.8e-15
   3        0.76   0.3500000        0.76    8    6    6  9.4e-14  2.7e-15
Velocity u2, Pressure p
Scales for dependent variables:
Pressure (comp1.p): 3.6e+02
Velocity field (spatial frame) (comp1.u2): 0.00072
Iter      SolEst     Damping    Stepsize #Res #Jac #Sol   LinErr   LinRes
   1        0.36   0.5000000        0.36    4    2    2  3.4e-15  7.8e-15
Displacement field
Iter      SolEst     Damping    Stepsize #Res #Jac #Sol   LinErr   LinRes
   1        0.95   0.5000000        0.95    4    2    2  1.3e-13  1.5e-12
Spatial mesh displacement
Iter      SolEst     Damping    Stepsize #Res #Jac #Sol   LinErr   LinRes
   1     9.5e-07   0.5000000     9.5e-07    4    2    2  1.4e-15  1.8e-15
Solution error estimates for segregated groups
1.3, 0.18, 0.48, 4.8e-07
Residual error estimates for segregated groups
8e+02, 1.4e+04, 2.2e+04, 0.17
Segregated solver iteration 3.
Merged variables
Iter      SolEst     Damping    Stepsize #Res #Jac #Sol   LinErr   LinRes
   1        0.75   0.3500000        0.75   10    7    7  5.6e-14  2.7e-15
   2        0.73   0.3500000        0.73   11    8    8  7.7e-14  2.7e-15
   3        0.72   0.3500000        0.72   12    9    9  4.6e-14  2.7e-15
Velocity u2, Pressure p
Iter      SolEst     Damping    Stepsize #Res #Jac #Sol   LinErr   LinRes
   1        0.15   0.5000000        0.15    6    3    3  2.3e-15  7.8e-15
Displacement field
Iter      SolEst     Damping    Stepsize #Res #Jac #Sol   LinErr   LinRes
   1        0.52   0.5000000        0.52    6    3    3  6.7e-14  1.5e-12
Spatial mesh displacement
Iter      SolEst     Damping    Stepsize #Res #Jac #Sol   LinErr   LinRes
   1     9.5e-07   0.5000000     9.5e-07    6    3    3  1.4e-15  1.9e-15
Solution error estimates for segregated groups
1.2, 0.077, 0.26, 4.8e-07
Residual error estimates for segregated groups
2.2e+02, 7.7e+03, 1.6e+04, 0.17
Segregated solver iteration 4.
```

```
Merged variables
Iter      SolEst     Damping    Stepsize #Res #Jac #Sol   LinErr   LinRes
   1         0.7   0.3500000         0.7   14   10   10  5.2e-14  2.8e-15
   2        0.68   0.3500000        0.68   15   11   11  4.2e-14  2.8e-15
   3         0.6   0.3500000         0.6   16   12   12  7.2e-14  2.7e-15
Velocity u2, Pressure p
Iter      SolEst     Damping    Stepsize #Res #Jac #Sol   LinErr   LinRes
   1       0.072   0.5000000       0.072    8    4    4  2.8e-15  7.9e-15
Displacement field
Iter      SolEst     Damping    Stepsize #Res #Jac #Sol   LinErr   LinRes
   1        0.29   0.5000000        0.29    8    4    4  1.1e-13  1.5e-12
Spatial mesh displacement
Iter      SolEst     Damping    Stepsize #Res #Jac #Sol   LinErr   LinRes
   1       8e-07   0.5000000       8e-07    8    4    4  1.5e-15  1.8e-15
Solution error estimates for segregated groups
1, 0.036, 0.15, 4e-07
Residual error estimates for segregated groups
84, 4.1e+03, 1.1e+04, 0.17
Segregated solver iteration 5.
Merged variables
Iter      SolEst     Damping    Stepsize #Res #Jac #Sol   LinErr   LinRes
   1        0.41   0.3500000        0.41   18   13   13    1e-13  2.8e-15
   2        0.28   0.3500000        0.28   19   14   14  3.9e-14  2.7e-15
   3        0.18   0.3500000        0.18   20   15   15  9.3e-14  2.7e-15
Velocity u2, Pressure p
Iter      SolEst     Damping    Stepsize #Res #Jac #Sol   LinErr   LinRes
   1       0.035   0.5000000       0.035   10    5    5  2.3e-15  7.9e-15
Displacement field
Iter      SolEst     Damping    Stepsize #Res #Jac #Sol   LinErr   LinRes
   1        0.17   0.5000000        0.17   10    5    5  4.7e-14  1.5e-12
Spatial mesh displacement
Iter      SolEst     Damping    Stepsize #Res #Jac #Sol   LinErr   LinRes
   1       6e-07   0.5000000       6e-07   10    5    5  1.5e-15  1.9e-15
Solution error estimates for segregated groups
0.31, 0.017, 0.084, 3e-07
Residual error estimates for segregated groups
33, 2.1e+03, 6.9e+03, 0.17
Segregated solver iteration 6.
Merged variables
Iter      SolEst     Damping    Stepsize #Res #Jac #Sol   LinErr   LinRes
   1        0.12   0.3500000        0.12   22   16   16  1.4e-13  2.7e-15
   2       0.079   0.3500000       0.079   23   17   17  5.2e-14  2.9e-15
   3       0.051   0.3500000       0.051   24   18   18    6e-14  2.7e-15
Velocity u2, Pressure p
Iter      SolEst     Damping    Stepsize #Res #Jac #Sol   LinErr   LinRes
   1       0.017   0.5000000       0.017   12    6    6  5.9e-15  7.8e-15
Displacement field
Iter      SolEst     Damping    Stepsize #Res #Jac #Sol   LinErr   LinRes
   1       0.095   0.5000000       0.095   12    6    6  7.3e-14  1.5e-12
Spatial mesh displacement
Iter      SolEst     Damping    Stepsize #Res #Jac #Sol   LinErr   LinRes
   1     4.2e-07   0.5000000     4.2e-07   12    6    6  1.3e-15  1.8e-15
Solution error estimates for segregated groups
0.088, 0.0085, 0.048, 2.1e-07
Residual error estimates for segregated groups
12, 1.1e+03, 4.2e+03, 0.18
```

```
Segregated solver iteration 7.
Merged variables
Iter      SolEst     Damping    Stepsize #Res #Jac #Sol   LinErr   LinRes
   1       0.033   0.3500000       0.033   26   19   19  6.3e-14  2.7e-15
   2       0.022   0.3500000       0.022   27   20   20  5.2e-14  2.6e-15
   3       0.014   0.3500000       0.014   28   21   21    4e-14  2.8e-15
Velocity u2, Pressure p
Iter      SolEst     Damping    Stepsize #Res #Jac #Sol   LinErr   LinRes
   1      0.0084   0.5000000      0.0084   14    7    7  2.7e-15  7.9e-15
Displacement field
Iter      SolEst     Damping    Stepsize #Res #Jac #Sol   LinErr   LinRes
   1       0.054   0.5000000       0.054   14    7    7  7.7e-14  1.5e-12
Spatial mesh displacement
Iter      SolEst     Damping    Stepsize #Res #Jac #Sol   LinErr   LinRes
   1     2.8e-07   0.5000000     2.8e-07   14    7    7    2e-15  1.8e-15
Solution error estimates for segregated groups
0.024, 0.0042, 0.027, 1.4e-07
Residual error estimates for segregated groups
4.1, 5.3e+02, 2.6e+03, 0.17
Segregated solver iteration 8.
Merged variables
Iter      SolEst     Damping    Stepsize #Res #Jac #Sol   LinErr   LinRes
   1      0.0092   0.3500000      0.0092   30   22   22  6.9e-14  2.8e-15
   2       0.006   0.3500000       0.006   31   23   23  7.6e-14  2.6e-15
   3      0.0039   0.3500000      0.0039   32   24   24  7.5e-14  2.7e-15
Velocity u2, Pressure p
Iter      SolEst     Damping    Stepsize #Res #Jac #Sol   LinErr   LinRes
   1      0.0042   0.5000000      0.0042   16    8    8  3.2e-15  7.8e-15
Displacement field
Iter      SolEst     Damping    Stepsize #Res #Jac #Sol   LinErr   LinRes
   1        0.03   0.5000000        0.03   16    8    8    5e-14  1.5e-12
Spatial mesh displacement
Iter      SolEst     Damping    Stepsize #Res #Jac #Sol   LinErr   LinRes
   1     1.8e-07   0.5000000     1.8e-07   16    8    8  1.7e-15  1.8e-15
Solution error estimates for segregated groups
0.0067, 0.0021, 0.015, 9e-08
Residual error estimates for segregated groups
1.4, 2.7e+02, 1.8e+03, 0.17
Segregated solver iteration 9.
Merged variables
Iter      SolEst     Damping    Stepsize #Res #Jac #Sol   LinErr   LinRes
   1      0.0025   0.3500000      0.0025   34   25   25  4.4e-14  2.6e-15
   2      0.0016   0.3500000      0.0016   35   26   26  4.9e-14  2.7e-15
   3      0.0011   0.3500000      0.0011   36   27   27  9.2e-14  2.7e-15
Velocity u2, Pressure p
Iter      SolEst     Damping    Stepsize #Res #Jac #Sol   LinErr   LinRes
   1      0.0021   0.5000000      0.0021   18    9    9  3.7e-15  7.9e-15
Displacement field
Iter      SolEst     Damping    Stepsize #Res #Jac #Sol   LinErr   LinRes
   1       0.017   0.5000000       0.017   18    9    9  6.9e-14  1.5e-12
Spatial mesh displacement
Iter      SolEst     Damping    Stepsize #Res #Jac #Sol   LinErr   LinRes
   1     1.1e-07   0.5000000     1.1e-07   18    9    9  1.5e-15  1.8e-15
Solution error estimates for segregated groups
0.0018, 0.001, 0.0085, 5.6e-08
Residual error estimates for segregated groups
```

```
0.44, 1.3e+02, 1.4e+03, 0.16
Segregated solver iteration 10.
Merged variables
Iter      SolEst     Damping    Stepsize #Res #Jac #Sol   LinErr   LinRes
   1      0.0007   0.3500000      0.0007   38   28   28  1.2e-13  2.6e-15
   2     0.00045   0.3500000     0.00045   39   29   29  7.6e-14  2.7e-15
   3     0.00029   0.3500000     0.00029   40   30   30  9.3e-14  2.7e-15
Velocity u2, Pressure p
Iter      SolEst     Damping    Stepsize #Res #Jac #Sol   LinErr   LinRes
   1       0.001   0.5000000       0.001   20   10   10  4.9e-15  7.8e-15
Displacement field
Iter      SolEst     Damping    Stepsize #Res #Jac #Sol   LinErr   LinRes
   1      0.0094   0.5000000      0.0094   20   10   10  4.3e-14  1.5e-12
Spatial mesh displacement
Iter      SolEst     Damping    Stepsize #Res #Jac #Sol   LinErr   LinRes
   1     6.8e-08   0.5000000     6.8e-08   20   10   10  1.3e-15  1.8e-15
Solution error estimates for segregated groups
0.0005, 0.00052, 0.0047, 3.4e-08
Residual error estimates for segregated groups
0.14, 67, 1.2e+03, 0.17
Segregated solver iteration 11.
Merged variables
Iter      SolEst     Damping    Stepsize #Res #Jac #Sol   LinErr   LinRes
   1     0.00019   0.3500000     0.00019   42   31   31  1.1e-13  2.7e-15
   2     0.00012   0.3500000     0.00012   43   32   32    6e-14  2.7e-15
   3     8.1e-05   0.3500000     8.1e-05   44   33   33  4.7e-14  2.6e-15
Velocity u2, Pressure p
Iter      SolEst     Damping    Stepsize #Res #Jac #Sol   LinErr   LinRes
   1     0.00052   0.5000000     0.00052   22   11   11  4.1e-15  7.8e-15
Displacement field
Iter      SolEst     Damping    Stepsize #Res #Jac #Sol   LinErr   LinRes
   1      0.0051   0.5000000      0.0051   22   11   11  5.5e-14  1.5e-12
Spatial mesh displacement
Iter      SolEst     Damping    Stepsize #Res #Jac #Sol   LinErr   LinRes
   1     4.1e-08   0.5000000     4.1e-08   22   11   11  1.2e-15  1.8e-15
Solution error estimates for segregated groups
0.00014, 0.00026, 0.0026, 2.1e-08
Residual error estimates for segregated groups
0.043, 33, 1.2e+03, 0.17
Segregated solver iteration 12.
Merged variables
Iter      SolEst     Damping    Stepsize #Res #Jac #Sol   LinErr   LinRes
   1     5.2e-05   0.3500000     5.2e-05   46   34   34  7.8e-14  2.8e-15
   2     3.4e-05   0.3500000     3.4e-05   47   35   35    6e-14  2.7e-15
   3     2.2e-05   0.3500000     2.2e-05   48   36   36  6.5e-14  2.7e-15
Velocity u2, Pressure p
Iter      SolEst     Damping    Stepsize #Res #Jac #Sol   LinErr   LinRes
   1     0.00026   0.5000000     0.00026   24   12   12  4.6e-15  7.9e-15
Displacement field
Iter      SolEst     Damping    Stepsize #Res #Jac #Sol   LinErr   LinRes
   1      0.0028   0.5000000      0.0028   24   12   12  9.2e-14  1.5e-12
Spatial mesh displacement
Iter      SolEst     Damping    Stepsize #Res #Jac #Sol   LinErr   LinRes
   1     2.4e-08   0.5000000     2.4e-08   24   12   12  1.9e-15  1.8e-15
Solution error estimates for segregated groups
3.8e-05, 0.00013, 0.0014, 1.2e-08
```

```
Residual error estimates for segregated groups
0.013, 17, 1.2e+03, 0.18
Segregated solver iteration 13.
Merged variables
Iter      SolEst     Damping    Stepsize #Res #Jac #Sol   LinErr   LinRes
   1     1.4e-05   0.3500000     1.4e-05   50   37   37    5e-14  2.7e-15
   2     9.4e-06   0.3500000     9.4e-06   51   38   38  9.1e-14  2.9e-15
   3     6.1e-06   0.3500000     6.1e-06   52   39   39  6.6e-14  2.8e-15
Velocity u2, Pressure p
Iter      SolEst     Damping    Stepsize #Res #Jac #Sol   LinErr   LinRes
   1     0.00013   0.5000000     0.00013   26   13   13  2.2e-15  7.9e-15
Displacement field
Iter      SolEst     Damping    Stepsize #Res #Jac #Sol   LinErr   LinRes
   1      0.0015   0.5000000      0.0015   26   13   13  6.1e-14  1.5e-12
Spatial mesh displacement
Iter      SolEst     Damping    Stepsize #Res #Jac #Sol   LinErr   LinRes
   1     1.4e-08   0.5000000     1.4e-08   26   13   13  2.3e-15  1.8e-15
Solution error estimates for segregated groups
1e-05, 6.5e-05, 0.00076, 7.1e-09
Residual error estimates for segregated groups
0.004, 8.3, 1.2e+03, 0.18
Solution time: 155 s. (2 minutes, 35 seconds)
Physical memory: 4.6 GB
Virtual memory: 25.5 GB
Ended at Jan 31, 2025, 6:41:43 PM.
----- Stationary Solver 1 in study1/Approach Curve Solver (sol1) -------------->
```

d=2300 (su42)

GENERAL

Description	Value
Solution	d=2300

LOG

```
<---- Compile Equations: Stationary in study1/Approach Curve Solver (sol1) -----
Started at Jan 31, 2025, 6:41:43 PM.
Parameter d = 2.3E-6 (m).
Geometry shape function: Linear serendipity
Number of vertex elements: 18
Number of boundary elements: 6229
Number of elements: 156221
Minimum element quality: 0.4896
Time: 8 s.
Physical memory: 2.92 GB
Virtual memory: 15.27 GB
Ended at Jan 31, 2025, 6:41:52 PM.
----- Compile Equations: Stationary in study1/Approach Curve Solver (sol1) ---->

<---- Dependent Variables 1 in study1/Approach Curve Solver (sol1) -------------
Started at Jan 31, 2025, 6:41:52 PM.
Solution time: 0 s.
Physical memory: 2.92 GB
Virtual memory: 15.2 GB
Ended at Jan 31, 2025, 6:41:52 PM.
----- Dependent Variables 1 in study1/Approach Curve Solver (sol1) ------------>

<---- Stationary Solver 1 in study1/Approach Curve Solver (sol1) ---------------
Started at Jan 31, 2025, 6:41:52 PM.
Segregated solver
Segregated Step 11 does not have any components solved for.
Number of degrees of freedom solved for: 794269 (plus 11881 internal DOFs).
Segregated solver iteration 1.
Merged variables
Nonsymmetric matrix found.
Scales for dependent variables:
Concentration (comp1.Cl): 1.4e+02
Concentration (comp1.K): 1.4e+02
Electric potential (comp1.V): 1.9e+02
Orthonormal null-space function used.
Iter      SolEst     Damping    Stepsize #Res #Jac #Sol   LinErr   LinRes
   1        0.83   0.3500000        0.83    2    1    1  2.8e-11  2.8e-15
   2        0.81   0.3500000        0.81    3    2    2    2e-11  2.8e-15
   3         0.8   0.3500000         0.8    4    3    3  3.2e-11  2.8e-15
Velocity u2, Pressure p
Nonsymmetric matrix found.
Scales for dependent variables:
Pressure (comp1.p): 1
Velocity field (spatial frame) (comp1.u2): 0.00018
Orthonormal null-space function used.
Iter      SolEst     Damping    Stepsize #Res #Jac #Sol   LinErr   LinRes
   1         1.1   0.5000000         1.1    2    1    1  1.8e-15  2.2e-15
Displacement field
Nonsymmetric matrix found.
Scales for dependent variables:
Auxiliary pressure (comp1.solid.lemm1.pw): 1
Displacement field (comp1.u): 2.8e-18
Orthonormal null-space function used.
Iter      SolEst     Damping    Stepsize #Res #Jac #Sol   LinErr   LinRes
   1         1.9   0.5000000         1.9    2    1    1  4.5e-14  1.5e-12
Spatial mesh displacement
```

```
Symmetric matrices found.
Scales for dependent variables:
Spatial mesh displacement (comp1.spatial.disp): 1.2e-09
Orthonormal null-space function used.
Iter      SolEst     Damping    Stepsize #Res #Jac #Sol   LinErr   LinRes
   1     6.4e-07   0.5000000     6.4e-07    2    1    1  1.2e-15  1.9e-15
Solution error estimates for segregated groups
1.4, 0.54, 0.95, 3.2e-07
Residual error estimates for segregated groups
7.9e+03, 8.3e+04, 2.2e+04, 0.15
Segregated solver iteration 2.
Merged variables
Scales for dependent variables:
Concentration (comp1.Cl): 1.4e+02
Concentration (comp1.K): 1.4e+02
Electric potential (comp1.V): 0.064
Iter      SolEst     Damping    Stepsize #Res #Jac #Sol   LinErr   LinRes
   1        0.79   0.3500000        0.79    6    4    4  3.7e-14  2.7e-15
   2        0.77   0.3500000        0.77    7    5    5  4.7e-14  2.7e-15
   3        0.76   0.3500000        0.76    8    6    6  7.3e-14  2.8e-15
Velocity u2, Pressure p
Scales for dependent variables:
Pressure (comp1.p): 3.6e+02
Velocity field (spatial frame) (comp1.u2): 0.00072
Iter      SolEst     Damping    Stepsize #Res #Jac #Sol   LinErr   LinRes
   1        0.36   0.5000000        0.36    4    2    2  2.2e-15  7.9e-15
Displacement field
Iter      SolEst     Damping    Stepsize #Res #Jac #Sol   LinErr   LinRes
   1        0.95   0.5000000        0.95    4    2    2    4e-14  1.5e-12
Spatial mesh displacement
Iter      SolEst     Damping    Stepsize #Res #Jac #Sol   LinErr   LinRes
   1     9.6e-07   0.5000000     9.6e-07    4    2    2  1.3e-15  1.8e-15
Solution error estimates for segregated groups
1.3, 0.18, 0.48, 4.8e-07
Residual error estimates for segregated groups
8e+02, 1.3e+04, 2.2e+04, 0.17
Segregated solver iteration 3.
Merged variables
Iter      SolEst     Damping    Stepsize #Res #Jac #Sol   LinErr   LinRes
   1        0.75   0.3500000        0.75   10    7    7  1.1e-13  2.6e-15
   2        0.73   0.3500000        0.73   11    8    8  8.8e-14  2.8e-15
   3        0.72   0.3500000        0.72   12    9    9  4.4e-14  2.7e-15
Velocity u2, Pressure p
Iter      SolEst     Damping    Stepsize #Res #Jac #Sol   LinErr   LinRes
   1        0.15   0.5000000        0.15    6    3    3  4.7e-15  7.9e-15
Displacement field
Iter      SolEst     Damping    Stepsize #Res #Jac #Sol   LinErr   LinRes
   1        0.52   0.5000000        0.52    6    3    3  5.6e-14  1.5e-12
Spatial mesh displacement
Iter      SolEst     Damping    Stepsize #Res #Jac #Sol   LinErr   LinRes
   1     9.6e-07   0.5000000     9.6e-07    6    3    3  1.6e-15  1.9e-15
Solution error estimates for segregated groups
1.2, 0.077, 0.26, 4.8e-07
Residual error estimates for segregated groups
2.2e+02, 7.4e+03, 1.6e+04, 0.17
Segregated solver iteration 4.
```

```
Merged variables
Iter      SolEst     Damping    Stepsize #Res #Jac #Sol   LinErr   LinRes
   1         0.7   0.3500000         0.7   14   10   10  6.5e-14  2.8e-15
   2        0.68   0.3500000        0.68   15   11   11  1.2e-13  2.7e-15
   3         0.6   0.3500000         0.6   16   12   12    1e-13  2.8e-15
Velocity u2, Pressure p
Iter      SolEst     Damping    Stepsize #Res #Jac #Sol   LinErr   LinRes
   1       0.071   0.5000000       0.071    8    4    4  2.1e-15  7.9e-15
Displacement field
Iter      SolEst     Damping    Stepsize #Res #Jac #Sol   LinErr   LinRes
   1        0.29   0.5000000        0.29    8    4    4  1.1e-13  1.5e-12
Spatial mesh displacement
Iter      SolEst     Damping    Stepsize #Res #Jac #Sol   LinErr   LinRes
   1       8e-07   0.5000000       8e-07    8    4    4  1.7e-15  1.8e-15
Solution error estimates for segregated groups
1, 0.036, 0.15, 4e-07
Residual error estimates for segregated groups
84, 3.9e+03, 1.1e+04, 0.17
Segregated solver iteration 5.
Merged variables
Iter      SolEst     Damping    Stepsize #Res #Jac #Sol   LinErr   LinRes
   1        0.41   0.3500000        0.41   18   13   13    1e-13  2.8e-15
   2        0.28   0.3500000        0.28   19   14   14  8.9e-14  2.7e-15
   3        0.18   0.3500000        0.18   20   15   15  5.6e-14  2.7e-15
Velocity u2, Pressure p
Iter      SolEst     Damping    Stepsize #Res #Jac #Sol   LinErr   LinRes
   1       0.035   0.5000000       0.035   10    5    5  2.1e-15  7.8e-15
Displacement field
Iter      SolEst     Damping    Stepsize #Res #Jac #Sol   LinErr   LinRes
   1        0.17   0.5000000        0.17   10    5    5  1.2e-13  1.5e-12
Spatial mesh displacement
Iter      SolEst     Damping    Stepsize #Res #Jac #Sol   LinErr   LinRes
   1       6e-07   0.5000000       6e-07   10    5    5  1.7e-15  1.9e-15
Solution error estimates for segregated groups
0.31, 0.017, 0.084, 3e-07
Residual error estimates for segregated groups
33, 2e+03, 6.9e+03, 0.18
Segregated solver iteration 6.
Merged variables
Iter      SolEst     Damping    Stepsize #Res #Jac #Sol   LinErr   LinRes
   1        0.12   0.3500000        0.12   22   16   16    1e-13  2.9e-15
   2       0.079   0.3500000       0.079   23   17   17  3.1e-14  2.7e-15
   3       0.051   0.3500000       0.051   24   18   18  7.8e-14  2.7e-15
Velocity u2, Pressure p
Iter      SolEst     Damping    Stepsize #Res #Jac #Sol   LinErr   LinRes
   1       0.017   0.5000000       0.017   12    6    6  5.3e-15  7.9e-15
Displacement field
Iter      SolEst     Damping    Stepsize #Res #Jac #Sol   LinErr   LinRes
   1       0.095   0.5000000       0.095   12    6    6  5.8e-14  1.5e-12
Spatial mesh displacement
Iter      SolEst     Damping    Stepsize #Res #Jac #Sol   LinErr   LinRes
   1     4.2e-07   0.5000000     4.2e-07   12    6    6  1.5e-15  1.9e-15
Solution error estimates for segregated groups
0.088, 0.0085, 0.048, 2.1e-07
Residual error estimates for segregated groups
12, 1e+03, 4.2e+03, 0.17
```

```
Segregated solver iteration 7.
Merged variables
Iter      SolEst     Damping    Stepsize #Res #Jac #Sol   LinErr   LinRes
   1       0.034   0.3500000       0.034   26   19   19  7.7e-14  2.7e-15
   2       0.022   0.3500000       0.022   27   20   20  1.1e-13  2.9e-15
   3       0.014   0.3500000       0.014   28   21   21  1.5e-13  2.8e-15
Velocity u2, Pressure p
Iter      SolEst     Damping    Stepsize #Res #Jac #Sol   LinErr   LinRes
   1      0.0084   0.5000000      0.0084   14    7    7  2.8e-15  7.9e-15
Displacement field
Iter      SolEst     Damping    Stepsize #Res #Jac #Sol   LinErr   LinRes
   1       0.054   0.5000000       0.054   14    7    7  3.5e-14  1.5e-12
Spatial mesh displacement
Iter      SolEst     Damping    Stepsize #Res #Jac #Sol   LinErr   LinRes
   1     2.8e-07   0.5000000     2.8e-07   14    7    7  1.6e-15  1.8e-15
Solution error estimates for segregated groups
0.024, 0.0042, 0.027, 1.4e-07
Residual error estimates for segregated groups
4.1, 5.1e+02, 2.6e+03, 0.17
Segregated solver iteration 8.
Merged variables
Iter      SolEst     Damping    Stepsize #Res #Jac #Sol   LinErr   LinRes
   1      0.0092   0.3500000      0.0092   30   22   22  1.3e-13  2.7e-15
   2       0.006   0.3500000       0.006   31   23   23  9.5e-14  2.7e-15
   3      0.0039   0.3500000      0.0039   32   24   24  9.7e-14  2.7e-15
Velocity u2, Pressure p
Iter      SolEst     Damping    Stepsize #Res #Jac #Sol   LinErr   LinRes
   1      0.0042   0.5000000      0.0042   16    8    8  2.2e-15  7.8e-15
Displacement field
Iter      SolEst     Damping    Stepsize #Res #Jac #Sol   LinErr   LinRes
   1        0.03   0.5000000        0.03   16    8    8  5.1e-14  1.5e-12
Spatial mesh displacement
Iter      SolEst     Damping    Stepsize #Res #Jac #Sol   LinErr   LinRes
   1     1.8e-07   0.5000000     1.8e-07   16    8    8  1.4e-15  1.9e-15
Solution error estimates for segregated groups
0.0067, 0.0021, 0.015, 9e-08
Residual error estimates for segregated groups
1.4, 2.5e+02, 1.8e+03, 0.17
Segregated solver iteration 9.
Merged variables
Iter      SolEst     Damping    Stepsize #Res #Jac #Sol   LinErr   LinRes
   1      0.0025   0.3500000      0.0025   34   25   25  9.2e-14  2.7e-15
   2      0.0017   0.3500000      0.0017   35   26   26  7.2e-14  2.7e-15
   3      0.0011   0.3500000      0.0011   36   27   27  6.8e-14  2.8e-15
Velocity u2, Pressure p
Iter      SolEst     Damping    Stepsize #Res #Jac #Sol   LinErr   LinRes
   1      0.0021   0.5000000      0.0021   18    9    9  2.5e-15  7.9e-15
Displacement field
Iter      SolEst     Damping    Stepsize #Res #Jac #Sol   LinErr   LinRes
   1       0.017   0.5000000       0.017   18    9    9    1e-13  1.5e-12
Spatial mesh displacement
Iter      SolEst     Damping    Stepsize #Res #Jac #Sol   LinErr   LinRes
   1     1.1e-07   0.5000000     1.1e-07   18    9    9  1.6e-15  1.8e-15
Solution error estimates for segregated groups
0.0018, 0.001, 0.0085, 5.6e-08
Residual error estimates for segregated groups
```

```
0.44, 1.3e+02, 1.4e+03, 0.17
Segregated solver iteration 10.
Merged variables
Iter      SolEst     Damping    Stepsize #Res #Jac #Sol   LinErr   LinRes
   1      0.0007   0.3500000      0.0007   38   28   28  9.7e-14  2.7e-15
   2     0.00045   0.3500000     0.00045   39   29   29  5.7e-14  2.7e-15
   3     0.00029   0.3500000     0.00029   40   30   30  5.4e-14  2.8e-15
Velocity u2, Pressure p
Iter      SolEst     Damping    Stepsize #Res #Jac #Sol   LinErr   LinRes
   1       0.001   0.5000000       0.001   20   10   10  3.1e-15  7.9e-15
Displacement field
Iter      SolEst     Damping    Stepsize #Res #Jac #Sol   LinErr   LinRes
   1      0.0094   0.5000000      0.0094   20   10   10  9.7e-14  1.5e-12
Spatial mesh displacement
Iter      SolEst     Damping    Stepsize #Res #Jac #Sol   LinErr   LinRes
   1     6.9e-08   0.5000000     6.9e-08   20   10   10    2e-15  1.9e-15
Solution error estimates for segregated groups
0.00051, 0.00052, 0.0047, 3.4e-08
Residual error estimates for segregated groups
0.14, 64, 1.2e+03, 0.16
Segregated solver iteration 11.
Merged variables
Iter      SolEst     Damping    Stepsize #Res #Jac #Sol   LinErr   LinRes
   1     0.00019   0.3500000     0.00019   42   31   31  7.4e-14  2.8e-15
   2     0.00012   0.3500000     0.00012   43   32   32  5.4e-14  2.7e-15
   3     8.1e-05   0.3500000     8.1e-05   44   33   33  7.9e-14  2.7e-15
Velocity u2, Pressure p
Iter      SolEst     Damping    Stepsize #Res #Jac #Sol   LinErr   LinRes
   1     0.00052   0.5000000     0.00052   22   11   11  2.9e-15    8e-15
Displacement field
Iter      SolEst     Damping    Stepsize #Res #Jac #Sol   LinErr   LinRes
   1      0.0051   0.5000000      0.0051   22   11   11  7.6e-14  1.5e-12
Spatial mesh displacement
Iter      SolEst     Damping    Stepsize #Res #Jac #Sol   LinErr   LinRes
   1     4.1e-08   0.5000000     4.1e-08   22   11   11  1.4e-15  1.9e-15
Solution error estimates for segregated groups
0.00014, 0.00026, 0.0026, 2.1e-08
Residual error estimates for segregated groups
0.044, 32, 1.2e+03, 0.16
Segregated solver iteration 12.
Merged variables
Iter      SolEst     Damping    Stepsize #Res #Jac #Sol   LinErr   LinRes
   1     5.3e-05   0.3500000     5.3e-05   46   34   34  6.5e-14  2.7e-15
   2     3.4e-05   0.3500000     3.4e-05   47   35   35  4.8e-14  2.9e-15
   3     2.2e-05   0.3500000     2.2e-05   48   36   36  1.1e-13  2.7e-15
Velocity u2, Pressure p
Iter      SolEst     Damping    Stepsize #Res #Jac #Sol   LinErr   LinRes
   1     0.00026   0.5000000     0.00026   24   12   12    5e-15  7.9e-15
Displacement field
Iter      SolEst     Damping    Stepsize #Res #Jac #Sol   LinErr   LinRes
   1      0.0028   0.5000000      0.0028   24   12   12  6.1e-14  1.5e-12
Spatial mesh displacement
Iter      SolEst     Damping    Stepsize #Res #Jac #Sol   LinErr   LinRes
   1     2.4e-08   0.5000000     2.4e-08   24   12   12  1.4e-15  1.8e-15
Solution error estimates for segregated groups
3.8e-05, 0.00013, 0.0014, 1.2e-08
```

```
Residual error estimates for segregated groups
0.013, 16, 1.2e+03, 0.18
Segregated solver iteration 13.
Merged variables
Iter      SolEst      Damping    Stepsize #Res #Jac #Sol   LinErr   LinRes
   1     1.4e-05    0.3500000     1.4e-05   50   37   37  5.9e-14  2.7e-15
   2     9.4e-06    0.3500000     9.4e-06   51   38   38  8.8e-14  2.7e-15
   3     6.1e-06    0.3500000     6.1e-06   52   39   39  1.2e-13  2.9e-15
Velocity u2, Pressure p
Iter      SolEst      Damping    Stepsize #Res #Jac #Sol   LinErr   LinRes
   1     0.00013    0.5000000     0.00013   26   13   13  2.9e-15  7.8e-15
Displacement field
Iter      SolEst      Damping    Stepsize #Res #Jac #Sol   LinErr   LinRes
   1      0.0015    0.5000000      0.0015   26   13   13  8.9e-14  1.5e-12
Spatial mesh displacement
Iter      SolEst      Damping    Stepsize #Res #Jac #Sol   LinErr   LinRes
   1     1.4e-08    0.5000000     1.4e-08   26   13   13  2.2e-15  1.9e-15
Solution error estimates for segregated groups
1e-05, 6.5e-05, 0.00076, 7.1e-09
Residual error estimates for segregated groups
0.0041, 8, 1.2e+03, 0.16
Solution time: 158 s. (2 minutes, 38 seconds)
Physical memory: 4.68 GB
Virtual memory: 26.03 GB
Ended at Jan 31, 2025, 6:44:30 PM.
----- Stationary Solver 1 in study1/Approach Curve Solver (sol1) -------------->
```

d=2295 (su43)

GENERAL

Description	Value
Solution	d=2295

LOG

```
<---- Compile Equations: Stationary in study1/Approach Curve Solver (sol1) -----
Started at Jan 31, 2025, 6:44:30 PM.
Parameter d = 2.295E-6 (m).
Geometry shape function: Linear serendipity
Number of vertex elements: 18
Number of boundary elements: 6227
Number of elements: 156325
Minimum element quality: 0.4795
Time: 8 s.
Physical memory: 2.93 GB
Virtual memory: 15.27 GB
Ended at Jan 31, 2025, 6:44:38 PM.
----- Compile Equations: Stationary in study1/Approach Curve Solver (sol1) ---->

<---- Dependent Variables 1 in study1/Approach Curve Solver (sol1) -------------
Started at Jan 31, 2025, 6:44:38 PM.
Solution time: 0 s.
Physical memory: 2.93 GB
Virtual memory: 15.27 GB
Ended at Jan 31, 2025, 6:44:38 PM.
----- Dependent Variables 1 in study1/Approach Curve Solver (sol1) ------------>

<---- Stationary Solver 1 in study1/Approach Curve Solver (sol1) ---------------
Started at Jan 31, 2025, 6:44:39 PM.
Segregated solver
Segregated Step 11 does not have any components solved for.
Number of degrees of freedom solved for: 794590 (plus 11877 internal DOFs).
Segregated solver iteration 1.
Merged variables
Nonsymmetric matrix found.
Scales for dependent variables:
Concentration (comp1.Cl): 1.4e+02
Concentration (comp1.K): 1.4e+02
Electric potential (comp1.V): 1.9e+02
Orthonormal null-space function used.
Iter      SolEst     Damping    Stepsize #Res #Jac #Sol   LinErr   LinRes
   1        0.83   0.3500000        0.83    2    1    1  3.2e-11  2.7e-15
   2        0.81   0.3500000        0.81    3    2    2  2.4e-11  2.7e-15
   3         0.8   0.3500000         0.8    4    3    3  3.1e-11  2.7e-15
Velocity u2, Pressure p
Nonsymmetric matrix found.
Scales for dependent variables:
Pressure (comp1.p): 1
Velocity field (spatial frame) (comp1.u2): 0.00018
Orthonormal null-space function used.
Iter      SolEst     Damping    Stepsize #Res #Jac #Sol   LinErr   LinRes
   1         1.1   0.5000000         1.1    2    1    1  2.5e-15  2.3e-15
Displacement field
Nonsymmetric matrix found.
Scales for dependent variables:
Auxiliary pressure (comp1.solid.lemm1.pw): 1
Displacement field (comp1.u): 2.8e-18
Orthonormal null-space function used.
Iter      SolEst     Damping    Stepsize #Res #Jac #Sol   LinErr   LinRes
   1         1.9   0.5000000         1.9    2    1    1  3.6e-14  1.5e-12
Spatial mesh displacement
```

```
Symmetric matrices found.
Scales for dependent variables:
Spatial mesh displacement (comp1.spatial.disp): 1.2e-09
Orthonormal null-space function used.
Iter      SolEst     Damping    Stepsize #Res #Jac #Sol   LinErr   LinRes
   1     6.5e-07   0.5000000     6.5e-07    2    1    1  1.8e-15  1.9e-15
Solution error estimates for segregated groups
1.4, 0.54, 0.95, 3.2e-07
Residual error estimates for segregated groups
7.9e+03, 8.3e+04, 2.2e+04, 0.15
Segregated solver iteration 2.
Merged variables
Scales for dependent variables:
Concentration (comp1.Cl): 1.4e+02
Concentration (comp1.K): 1.4e+02
Electric potential (comp1.V): 0.064
Iter      SolEst     Damping    Stepsize #Res #Jac #Sol   LinErr   LinRes
   1        0.78   0.3500000        0.78    6    4    4  7.7e-14    3e-15
   2        0.77   0.3500000        0.77    7    5    5  1.2e-13  2.9e-15
   3        0.76   0.3500000        0.76    8    6    6  1.2e-13  3.2e-15
Velocity u2, Pressure p
Scales for dependent variables:
Pressure (comp1.p): 3.6e+02
Velocity field (spatial frame) (comp1.u2): 0.00072
Iter      SolEst     Damping    Stepsize #Res #Jac #Sol   LinErr   LinRes
   1        0.36   0.5000000        0.36    4    2    2    2e-15  7.7e-15
Displacement field
Iter      SolEst     Damping    Stepsize #Res #Jac #Sol   LinErr   LinRes
   1        0.95   0.5000000        0.95    4    2    2  6.4e-14  1.5e-12
Spatial mesh displacement
Iter      SolEst     Damping    Stepsize #Res #Jac #Sol   LinErr   LinRes
   1     9.7e-07   0.5000000     9.7e-07    4    2    2  1.5e-15  1.8e-15
Solution error estimates for segregated groups
1.3, 0.18, 0.48, 4.9e-07
Residual error estimates for segregated groups
8e+02, 1.4e+04, 2.2e+04, 0.17
Segregated solver iteration 3.
Merged variables
Iter      SolEst     Damping    Stepsize #Res #Jac #Sol   LinErr   LinRes
   1        0.75   0.3500000        0.75   10    7    7  3.5e-14  2.9e-15
   2        0.73   0.3500000        0.73   11    8    8  7.6e-14  2.8e-15
   3        0.72   0.3500000        0.72   12    9    9  9.6e-14  2.8e-15
Velocity u2, Pressure p
Iter      SolEst     Damping    Stepsize #Res #Jac #Sol   LinErr   LinRes
   1        0.15   0.5000000        0.15    6    3    3    5e-15  7.8e-15
Displacement field
Iter      SolEst     Damping    Stepsize #Res #Jac #Sol   LinErr   LinRes
   1        0.52   0.5000000        0.52    6    3    3  7.6e-14  1.5e-12
Spatial mesh displacement
Iter      SolEst     Damping    Stepsize #Res #Jac #Sol   LinErr   LinRes
   1     9.7e-07   0.5000000     9.7e-07    6    3    3  1.5e-15  1.8e-15
Solution error estimates for segregated groups
1.2, 0.077, 0.26, 4.9e-07
Residual error estimates for segregated groups
2.2e+02, 7.7e+03, 1.6e+04, 0.17
Segregated solver iteration 4.
```

```
Merged variables
Iter      SolEst     Damping    Stepsize #Res #Jac #Sol   LinErr   LinRes
   1         0.7   0.3500000         0.7   14   10   10  1.1e-13  3.1e-15
   2        0.68   0.3500000        0.68   15   11   11  8.1e-14  3.2e-15
   3         0.6   0.3500000         0.6   16   12   12  5.1e-14  2.7e-15
Velocity u2, Pressure p
Iter      SolEst     Damping    Stepsize #Res #Jac #Sol   LinErr   LinRes
   1       0.071   0.5000000       0.071    8    4    4    2e-15  7.9e-15
Displacement field
Iter      SolEst     Damping    Stepsize #Res #Jac #Sol   LinErr   LinRes
   1        0.29   0.5000000        0.29    8    4    4  6.5e-14  1.5e-12
Spatial mesh displacement
Iter      SolEst     Damping    Stepsize #Res #Jac #Sol   LinErr   LinRes
   1     8.1e-07   0.5000000     8.1e-07    8    4    4  1.6e-15  1.8e-15
Solution error estimates for segregated groups
1, 0.036, 0.15, 4e-07
Residual error estimates for segregated groups
84, 4e+03, 1.1e+04, 0.17
Segregated solver iteration 5.
Merged variables
Iter      SolEst     Damping    Stepsize #Res #Jac #Sol   LinErr   LinRes
   1        0.41   0.3500000        0.41   18   13   13  1.1e-13  2.7e-15
   2        0.28   0.3500000        0.28   19   14   14    1e-13  3.3e-15
   3        0.18   0.3500000        0.18   20   15   15  5.7e-14  3.2e-15
Velocity u2, Pressure p
Iter      SolEst     Damping    Stepsize #Res #Jac #Sol   LinErr   LinRes
   1       0.035   0.5000000       0.035   10    5    5  3.6e-15  7.8e-15
Displacement field
Iter      SolEst     Damping    Stepsize #Res #Jac #Sol   LinErr   LinRes
   1        0.17   0.5000000        0.17   10    5    5  7.9e-14  1.5e-12
Spatial mesh displacement
Iter      SolEst     Damping    Stepsize #Res #Jac #Sol   LinErr   LinRes
   1     6.1e-07   0.5000000     6.1e-07   10    5    5  1.3e-15  1.9e-15
Solution error estimates for segregated groups
0.31, 0.017, 0.084, 3e-07
Residual error estimates for segregated groups
33, 2.1e+03, 6.9e+03, 0.16
Segregated solver iteration 6.
Merged variables
Iter      SolEst     Damping    Stepsize #Res #Jac #Sol   LinErr   LinRes
   1        0.12   0.3500000        0.12   22   16   16    9e-14    3e-15
   2       0.079   0.3500000       0.079   23   17   17    6e-14  2.8e-15
   3       0.051   0.3500000       0.051   24   18   18  1.4e-13  3.8e-15
Velocity u2, Pressure p
Iter      SolEst     Damping    Stepsize #Res #Jac #Sol   LinErr   LinRes
   1       0.017   0.5000000       0.017   12    6    6  4.3e-15  7.8e-15
Displacement field
Iter      SolEst     Damping    Stepsize #Res #Jac #Sol   LinErr   LinRes
   1       0.095   0.5000000       0.095   12    6    6  2.6e-14  1.5e-12
Spatial mesh displacement
Iter      SolEst     Damping    Stepsize #Res #Jac #Sol   LinErr   LinRes
   1     4.2e-07   0.5000000     4.2e-07   12    6    6  1.3e-15  1.9e-15
Solution error estimates for segregated groups
0.088, 0.0085, 0.048, 2.1e-07
Residual error estimates for segregated groups
12, 1.1e+03, 4.2e+03, 0.18
```

```
Segregated solver iteration 7.
Merged variables
Iter      SolEst     Damping    Stepsize #Res #Jac #Sol   LinErr   LinRes
   1       0.033   0.3500000       0.033   26   19   19  6.4e-14  3.4e-15
   2       0.022   0.3500000       0.022   27   20   20  1.1e-13  2.9e-15
   3       0.014   0.3500000       0.014   28   21   21  7.4e-14  3.1e-15
Velocity u2, Pressure p
Iter      SolEst     Damping    Stepsize #Res #Jac #Sol   LinErr   LinRes
   1      0.0084   0.5000000      0.0084   14    7    7  2.6e-15  7.8e-15
Displacement field
Iter      SolEst     Damping    Stepsize #Res #Jac #Sol   LinErr   LinRes
   1       0.054   0.5000000       0.054   14    7    7  5.7e-14  1.5e-12
Spatial mesh displacement
Iter      SolEst     Damping    Stepsize #Res #Jac #Sol   LinErr   LinRes
   1     2.8e-07   0.5000000     2.8e-07   14    7    7  2.4e-15  1.9e-15
Solution error estimates for segregated groups
0.024, 0.0042, 0.027, 1.4e-07
Residual error estimates for segregated groups
4.1, 5.3e+02, 2.6e+03, 0.18
Segregated solver iteration 8.
Merged variables
Iter      SolEst     Damping    Stepsize #Res #Jac #Sol   LinErr   LinRes
   1      0.0092   0.3500000      0.0092   30   22   22  1.1e-13  3.3e-15
   2       0.006   0.3500000       0.006   31   23   23    1e-13  3.1e-15
   3      0.0039   0.3500000      0.0039   32   24   24  7.7e-14  2.8e-15
Velocity u2, Pressure p
Iter      SolEst     Damping    Stepsize #Res #Jac #Sol   LinErr   LinRes
   1      0.0042   0.5000000      0.0042   16    8    8  3.2e-15  7.7e-15
Displacement field
Iter      SolEst     Damping    Stepsize #Res #Jac #Sol   LinErr   LinRes
   1        0.03   0.5000000        0.03   16    8    8  8.9e-14  1.5e-12
Spatial mesh displacement
Iter      SolEst     Damping    Stepsize #Res #Jac #Sol   LinErr   LinRes
   1     1.8e-07   0.5000000     1.8e-07   16    8    8  2.1e-15  1.9e-15
Solution error estimates for segregated groups
0.0067, 0.0021, 0.015, 9.1e-08
Residual error estimates for segregated groups
1.4, 2.7e+02, 1.8e+03, 0.17
Segregated solver iteration 9.
Merged variables
Iter      SolEst     Damping    Stepsize #Res #Jac #Sol   LinErr   LinRes
   1      0.0025   0.3500000      0.0025   34   25   25  6.2e-14  3.1e-15
   2      0.0016   0.3500000      0.0016   35   26   26  1.3e-13  3.8e-15
   3      0.0011   0.3500000      0.0011   36   27   27  7.4e-14  3.3e-15
Velocity u2, Pressure p
Iter      SolEst     Damping    Stepsize #Res #Jac #Sol   LinErr   LinRes
   1      0.0021   0.5000000      0.0021   18    9    9    3e-15  7.8e-15
Displacement field
Iter      SolEst     Damping    Stepsize #Res #Jac #Sol   LinErr   LinRes
   1       0.017   0.5000000       0.017   18    9    9  6.1e-14  1.5e-12
Spatial mesh displacement
Iter      SolEst     Damping    Stepsize #Res #Jac #Sol   LinErr   LinRes
   1     1.1e-07   0.5000000     1.1e-07   18    9    9  1.9e-15  1.9e-15
Solution error estimates for segregated groups
0.0018, 0.001, 0.0085, 5.7e-08
Residual error estimates for segregated groups
```

```
0.44, 1.3e+02, 1.4e+03, 0.17
Segregated solver iteration 10.
Merged variables
Iter      SolEst     Damping    Stepsize #Res #Jac #Sol   LinErr   LinRes
   1      0.0007   0.3500000      0.0007   38   28   28  9.2e-14  2.8e-15
   2     0.00045   0.3500000     0.00045   39   29   29  1.5e-13  2.9e-15
   3     0.00029   0.3500000     0.00029   40   30   30  3.8e-14  3.3e-15
Velocity u2, Pressure p
Iter      SolEst     Damping    Stepsize #Res #Jac #Sol   LinErr   LinRes
   1       0.001   0.5000000       0.001   20   10   10  2.4e-15  7.8e-15
Displacement field
Iter      SolEst     Damping    Stepsize #Res #Jac #Sol   LinErr   LinRes
   1      0.0094   0.5000000      0.0094   20   10   10  4.3e-14  1.5e-12
Spatial mesh displacement
Iter      SolEst     Damping    Stepsize #Res #Jac #Sol   LinErr   LinRes
   1       7e-08   0.5000000       7e-08   20   10   10  1.7e-15  1.9e-15
Solution error estimates for segregated groups
0.00051, 0.00052, 0.0047, 3.5e-08
Residual error estimates for segregated groups
0.14, 67, 1.2e+03, 0.17
Segregated solver iteration 11.
Merged variables
Iter      SolEst     Damping    Stepsize #Res #Jac #Sol   LinErr   LinRes
   1     0.00019   0.3500000     0.00019   42   31   31  7.6e-14  2.8e-15
   2     0.00012   0.3500000     0.00012   43   32   32  7.9e-14  3.1e-15
   3     8.1e-05   0.3500000     8.1e-05   44   33   33  9.7e-14  2.9e-15
Velocity u2, Pressure p
Iter      SolEst     Damping    Stepsize #Res #Jac #Sol   LinErr   LinRes
   1     0.00052   0.5000000     0.00052   22   11   11  4.4e-15  7.8e-15
Displacement field
Iter      SolEst     Damping    Stepsize #Res #Jac #Sol   LinErr   LinRes
   1      0.0051   0.5000000      0.0051   22   11   11    7e-14  1.5e-12
Spatial mesh displacement
Iter      SolEst     Damping    Stepsize #Res #Jac #Sol   LinErr   LinRes
   1     4.2e-08   0.5000000     4.2e-08   22   11   11    2e-15  1.9e-15
Solution error estimates for segregated groups
0.00014, 0.00026, 0.0026, 2.1e-08
Residual error estimates for segregated groups
0.044, 33, 1.2e+03, 0.17
Segregated solver iteration 12.
Merged variables
Iter      SolEst     Damping    Stepsize #Res #Jac #Sol   LinErr   LinRes
   1     5.3e-05   0.3500000     5.3e-05   46   34   34  4.8e-14    3e-15
   2     3.4e-05   0.3500000     3.4e-05   47   35   35  9.9e-14  3.6e-15
   3     2.2e-05   0.3500000     2.2e-05   48   36   36  9.1e-14  2.9e-15
Velocity u2, Pressure p
Iter      SolEst     Damping    Stepsize #Res #Jac #Sol   LinErr   LinRes
   1     0.00026   0.5000000     0.00026   24   12   12  3.9e-15  7.8e-15
Displacement field
Iter      SolEst     Damping    Stepsize #Res #Jac #Sol   LinErr   LinRes
   1      0.0028   0.5000000      0.0028   24   12   12  4.3e-14  1.5e-12
Spatial mesh displacement
Iter      SolEst     Damping    Stepsize #Res #Jac #Sol   LinErr   LinRes
   1     2.5e-08   0.5000000     2.5e-08   24   12   12  1.9e-15  1.9e-15
Solution error estimates for segregated groups
3.8e-05, 0.00013, 0.0014, 1.2e-08
```

```
Residual error estimates for segregated groups
0.013, 17, 1.2e+03, 0.17
Segregated solver iteration 13.
Merged variables
Iter      SolEst      Damping    Stepsize #Res #Jac #Sol   LinErr   LinRes
   1      1.4e-05   0.3500000     1.4e-05   50   37   37  5.5e-14  3.5e-15
   2      9.4e-06   0.3500000     9.4e-06   51   38   38  1.1e-13  3.4e-15
   3      6.1e-06   0.3500000     6.1e-06   52   39   39  1.1e-13  3.6e-15
Velocity u2, Pressure p
Iter      SolEst      Damping    Stepsize #Res #Jac #Sol   LinErr   LinRes
   1      0.00013   0.5000000     0.00013   26   13   13  1.9e-15  7.8e-15
Displacement field
Iter      SolEst      Damping    Stepsize #Res #Jac #Sol   LinErr   LinRes
   1       0.0015   0.5000000      0.0015   26   13   13  1.4e-13  1.5e-12
Spatial mesh displacement
Iter      SolEst      Damping    Stepsize #Res #Jac #Sol   LinErr   LinRes
   1      1.4e-08   0.5000000     1.4e-08   26   13   13  1.8e-15  1.9e-15
Solution error estimates for segregated groups
1e-05, 6.5e-05, 0.00076, 7.2e-09
Residual error estimates for segregated groups
0.0041, 8.3, 1.2e+03, 0.17
Solution time: 157 s. (2 minutes, 37 seconds)
Physical memory: 4.66 GB
Virtual memory: 25.59 GB
Ended at Jan 31, 2025, 6:47:15 PM.
----- Stationary Solver 1 in study1/Approach Curve Solver (sol1) -------------->
```

d=2290 (su44)

GENERAL

Description	Value
Solution	d=2290

LOG

```
<---- Compile Equations: Stationary in study1/Approach Curve Solver (sol1) -----
Started at Jan 31, 2025, 6:47:15 PM.
Parameter d = 2.29E-6 (m).
Geometry shape function: Linear serendipity
Number of vertex elements: 18
Number of boundary elements: 6224
Number of elements: 155980
Minimum element quality: 0.4775
Time: 8 s.
Physical memory: 2.92 GB
Virtual memory: 15.25 GB
Ended at Jan 31, 2025, 6:47:24 PM.
----- Compile Equations: Stationary in study1/Approach Curve Solver (sol1) ---->

<---- Dependent Variables 1 in study1/Approach Curve Solver (sol1) -------------
Started at Jan 31, 2025, 6:47:24 PM.
Solution time: 0 s.
Physical memory: 2.92 GB
Virtual memory: 15.25 GB
Ended at Jan 31, 2025, 6:47:24 PM.
----- Dependent Variables 1 in study1/Approach Curve Solver (sol1) ------------>

<---- Stationary Solver 1 in study1/Approach Curve Solver (sol1) ---------------
Started at Jan 31, 2025, 6:47:24 PM.
Segregated solver
Segregated Step 11 does not have any components solved for.
Number of degrees of freedom solved for: 792873 (plus 11871 internal DOFs).
Segregated solver iteration 1.
Merged variables
Nonsymmetric matrix found.
Scales for dependent variables:
Concentration (comp1.Cl): 1.4e+02
Concentration (comp1.K): 1.4e+02
Electric potential (comp1.V): 1.9e+02
Orthonormal null-space function used.
Iter      SolEst     Damping    Stepsize #Res #Jac #Sol   LinErr   LinRes
   1        0.83   0.3500000        0.83    2    1    1  3.4e-11  2.7e-15
   2        0.81   0.3500000        0.81    3    2    2  3.2e-11  2.7e-15
   3         0.8   0.3500000         0.8    4    3    3  2.3e-11  2.9e-15
Velocity u2, Pressure p
Nonsymmetric matrix found.
Scales for dependent variables:
Pressure (comp1.p): 1
Velocity field (spatial frame) (comp1.u2): 0.00018
Orthonormal null-space function used.
Iter      SolEst     Damping    Stepsize #Res #Jac #Sol   LinErr   LinRes
   1         1.1   0.5000000         1.1    2    1    1  1.8e-15  2.3e-15
Displacement field
Nonsymmetric matrix found.
Scales for dependent variables:
Auxiliary pressure (comp1.solid.lemm1.pw): 1
Displacement field (comp1.u): 2.8e-18
Orthonormal null-space function used.
Iter      SolEst     Damping    Stepsize #Res #Jac #Sol   LinErr   LinRes
   1         1.9   0.5000000         1.9    2    1    1  6.3e-14  1.5e-12
Spatial mesh displacement
```

```
Symmetric matrices found.
Scales for dependent variables:
Spatial mesh displacement (comp1.spatial.disp): 1.2e-09
Orthonormal null-space function used.
Iter      SolEst     Damping    Stepsize #Res #Jac #Sol   LinErr   LinRes
   1     6.4e-07   0.5000000     6.4e-07    2    1    1  1.7e-15  1.8e-15
Solution error estimates for segregated groups
1.4, 0.54, 0.95, 3.2e-07
Residual error estimates for segregated groups
7.9e+03, 8.3e+04, 2.2e+04, 0.15
Segregated solver iteration 2.
Merged variables
Scales for dependent variables:
Concentration (comp1.Cl): 1.4e+02
Concentration (comp1.K): 1.4e+02
Electric potential (comp1.V): 0.064
Iter      SolEst     Damping    Stepsize #Res #Jac #Sol   LinErr   LinRes
   1        0.78   0.3500000        0.78    6    4    4  1.3e-13  3.4e-15
   2        0.77   0.3500000        0.77    7    5    5  8.7e-14  3.3e-15
   3        0.76   0.3500000        0.76    8    6    6  5.9e-14  2.9e-15
Velocity u2, Pressure p
Scales for dependent variables:
Pressure (comp1.p): 3.6e+02
Velocity field (spatial frame) (comp1.u2): 0.00072
Iter      SolEst     Damping    Stepsize #Res #Jac #Sol   LinErr   LinRes
   1        0.36   0.5000000        0.36    4    2    2  4.1e-15  7.8e-15
Displacement field
Iter      SolEst     Damping    Stepsize #Res #Jac #Sol   LinErr   LinRes
   1        0.95   0.5000000        0.95    4    2    2  5.7e-14  1.5e-12
Spatial mesh displacement
Iter      SolEst     Damping    Stepsize #Res #Jac #Sol   LinErr   LinRes
   1     9.7e-07   0.5000000     9.7e-07    4    2    2  1.7e-15  1.9e-15
Solution error estimates for segregated groups
1.3, 0.18, 0.48, 4.8e-07
Residual error estimates for segregated groups
8e+02, 1.4e+04, 2.2e+04, 0.17
Segregated solver iteration 3.
Merged variables
Iter      SolEst     Damping    Stepsize #Res #Jac #Sol   LinErr   LinRes
   1        0.75   0.3500000        0.75   10    7    7  1.2e-13  3.7e-15
   2        0.73   0.3500000        0.73   11    8    8  5.3e-14  2.8e-15
   3        0.72   0.3500000        0.72   12    9    9  1.3e-13  3.1e-15
Velocity u2, Pressure p
Iter      SolEst     Damping    Stepsize #Res #Jac #Sol   LinErr   LinRes
   1        0.15   0.5000000        0.15    6    3    3  2.6e-15  7.9e-15
Displacement field
Iter      SolEst     Damping    Stepsize #Res #Jac #Sol   LinErr   LinRes
   1        0.52   0.5000000        0.52    6    3    3  4.7e-14  1.5e-12
Spatial mesh displacement
Iter      SolEst     Damping    Stepsize #Res #Jac #Sol   LinErr   LinRes
   1     9.7e-07   0.5000000     9.7e-07    6    3    3  1.4e-15  1.8e-15
Solution error estimates for segregated groups
1.2, 0.077, 0.26, 4.8e-07
Residual error estimates for segregated groups
2.2e+02, 7.7e+03, 1.6e+04, 0.17
Segregated solver iteration 4.
```

```
Merged variables
Iter      SolEst     Damping    Stepsize #Res #Jac #Sol   LinErr   LinRes
   1         0.7   0.3500000         0.7   14   10   10  8.5e-14  3.5e-15
   2        0.68   0.3500000        0.68   15   11   11  1.3e-13  3.2e-15
   3         0.6   0.3500000         0.6   16   12   12  1.1e-13    3e-15
Velocity u2, Pressure p
Iter      SolEst     Damping    Stepsize #Res #Jac #Sol   LinErr   LinRes
   1       0.072   0.5000000       0.072    8    4    4  3.5e-15    8e-15
Displacement field
Iter      SolEst     Damping    Stepsize #Res #Jac #Sol   LinErr   LinRes
   1        0.29   0.5000000        0.29    8    4    4  1.2e-13  1.5e-12
Spatial mesh displacement
Iter      SolEst     Damping    Stepsize #Res #Jac #Sol   LinErr   LinRes
   1       8e-07   0.5000000       8e-07    8    4    4  1.9e-15  1.8e-15
Solution error estimates for segregated groups
1, 0.036, 0.15, 4e-07
Residual error estimates for segregated groups
84, 4.1e+03, 1.1e+04, 0.18
Segregated solver iteration 5.
Merged variables
Iter      SolEst     Damping    Stepsize #Res #Jac #Sol   LinErr   LinRes
   1        0.41   0.3500000        0.41   18   13   13  6.6e-14  3.7e-15
   2        0.28   0.3500000        0.28   19   14   14    8e-14  3.2e-15
   3        0.18   0.3500000        0.18   20   15   15  8.5e-14  3.3e-15
Velocity u2, Pressure p
Iter      SolEst     Damping    Stepsize #Res #Jac #Sol   LinErr   LinRes
   1       0.035   0.5000000       0.035   10    5    5  2.6e-15  7.9e-15
Displacement field
Iter      SolEst     Damping    Stepsize #Res #Jac #Sol   LinErr   LinRes
   1        0.17   0.5000000        0.17   10    5    5  1.2e-13  1.5e-12
Spatial mesh displacement
Iter      SolEst     Damping    Stepsize #Res #Jac #Sol   LinErr   LinRes
   1       6e-07   0.5000000       6e-07   10    5    5    2e-15  1.9e-15
Solution error estimates for segregated groups
0.31, 0.017, 0.084, 3e-07
Residual error estimates for segregated groups
33, 2.1e+03, 6.9e+03, 0.16
Segregated solver iteration 6.
Merged variables
Iter      SolEst     Damping    Stepsize #Res #Jac #Sol   LinErr   LinRes
   1        0.12   0.3500000        0.12   22   16   16  1.5e-13  3.2e-15
   2       0.078   0.3500000       0.078   23   17   17  7.9e-14  3.4e-15
   3       0.051   0.3500000       0.051   24   18   18  7.8e-14  3.7e-15
Velocity u2, Pressure p
Iter      SolEst     Damping    Stepsize #Res #Jac #Sol   LinErr   LinRes
   1       0.017   0.5000000       0.017   12    6    6  4.1e-15  7.8e-15
Displacement field
Iter      SolEst     Damping    Stepsize #Res #Jac #Sol   LinErr   LinRes
   1       0.095   0.5000000       0.095   12    6    6  7.5e-14  1.5e-12
Spatial mesh displacement
Iter      SolEst     Damping    Stepsize #Res #Jac #Sol   LinErr   LinRes
   1     4.2e-07   0.5000000     4.2e-07   12    6    6  2.2e-15  1.8e-15
Solution error estimates for segregated groups
0.088, 0.0085, 0.048, 2.1e-07
Residual error estimates for segregated groups
12, 1.1e+03, 4.2e+03, 0.17
```

```
Segregated solver iteration 7.
Merged variables
Iter      SolEst     Damping    Stepsize #Res #Jac #Sol   LinErr   LinRes
   1       0.033   0.3500000       0.033   26   19   19  7.1e-14  2.9e-15
   2       0.022   0.3500000       0.022   27   20   20  6.4e-14    3e-15
   3       0.014   0.3500000       0.014   28   21   21  5.1e-14    3e-15
Velocity u2, Pressure p
Iter      SolEst     Damping    Stepsize #Res #Jac #Sol   LinErr   LinRes
   1      0.0084   0.5000000      0.0084   14    7    7  4.3e-15  7.8e-15
Displacement field
Iter      SolEst     Damping    Stepsize #Res #Jac #Sol   LinErr   LinRes
   1       0.054   0.5000000       0.054   14    7    7  1.2e-13  1.5e-12
Spatial mesh displacement
Iter      SolEst     Damping    Stepsize #Res #Jac #Sol   LinErr   LinRes
   1     2.8e-07   0.5000000     2.8e-07   14    7    7  1.7e-15  1.8e-15
Solution error estimates for segregated groups
0.024, 0.0042, 0.027, 1.4e-07
Residual error estimates for segregated groups
4.1, 5.3e+02, 2.6e+03, 0.17
Segregated solver iteration 8.
Merged variables
Iter      SolEst     Damping    Stepsize #Res #Jac #Sol   LinErr   LinRes
   1      0.0092   0.3500000      0.0092   30   22   22  6.5e-14  3.2e-15
   2       0.006   0.3500000       0.006   31   23   23    6e-14    3e-15
   3      0.0039   0.3500000      0.0039   32   24   24  6.3e-14  3.6e-15
Velocity u2, Pressure p
Iter      SolEst     Damping    Stepsize #Res #Jac #Sol   LinErr   LinRes
   1      0.0042   0.5000000      0.0042   16    8    8    3e-15  7.9e-15
Displacement field
Iter      SolEst     Damping    Stepsize #Res #Jac #Sol   LinErr   LinRes
   1        0.03   0.5000000        0.03   16    8    8  1.4e-13  1.5e-12
Spatial mesh displacement
Iter      SolEst     Damping    Stepsize #Res #Jac #Sol   LinErr   LinRes
   1     1.8e-07   0.5000000     1.8e-07   16    8    8  1.9e-15  1.8e-15
Solution error estimates for segregated groups
0.0067, 0.0021, 0.015, 9.1e-08
Residual error estimates for segregated groups
1.4, 2.7e+02, 1.8e+03, 0.18
Segregated solver iteration 9.
Merged variables
Iter      SolEst     Damping    Stepsize #Res #Jac #Sol   LinErr   LinRes
   1      0.0025   0.3500000      0.0025   34   25   25  8.9e-14    3e-15
   2      0.0016   0.3500000      0.0016   35   26   26  7.5e-14    3e-15
   3      0.0011   0.3500000      0.0011   36   27   27  1.7e-13  3.1e-15
Velocity u2, Pressure p
Iter      SolEst     Damping    Stepsize #Res #Jac #Sol   LinErr   LinRes
   1      0.0021   0.5000000      0.0021   18    9    9  3.6e-15    8e-15
Displacement field
Iter      SolEst     Damping    Stepsize #Res #Jac #Sol   LinErr   LinRes
   1       0.017   0.5000000       0.017   18    9    9  6.6e-14  1.5e-12
Spatial mesh displacement
Iter      SolEst     Damping    Stepsize #Res #Jac #Sol   LinErr   LinRes
   1     1.1e-07   0.5000000     1.1e-07   18    9    9  1.2e-15  1.8e-15
Solution error estimates for segregated groups
0.0018, 0.001, 0.0085, 5.7e-08
Residual error estimates for segregated groups
```

```
0.44, 1.3e+02, 1.4e+03, 0.16
Segregated solver iteration 10.
Merged variables
Iter      SolEst     Damping    Stepsize #Res #Jac #Sol   LinErr   LinRes
   1     0.00069   0.3500000     0.00069   38   28   28  6.1e-14  3.5e-15
   2     0.00045   0.3500000     0.00045   39   29   29  8.1e-14  3.6e-15
   3     0.00029   0.3500000     0.00029   40   30   30  5.9e-14    3e-15
Velocity u2, Pressure p
Iter      SolEst     Damping    Stepsize #Res #Jac #Sol   LinErr   LinRes
   1       0.001   0.5000000       0.001   20   10   10  2.6e-15  7.7e-15
Displacement field
Iter      SolEst     Damping    Stepsize #Res #Jac #Sol   LinErr   LinRes
   1      0.0094   0.5000000      0.0094   20   10   10  8.7e-14  1.5e-12
Spatial mesh displacement
Iter      SolEst     Damping    Stepsize #Res #Jac #Sol   LinErr   LinRes
   1     6.9e-08   0.5000000     6.9e-08   20   10   10  1.4e-15  1.9e-15
Solution error estimates for segregated groups
0.0005, 0.00052, 0.0047, 3.5e-08
Residual error estimates for segregated groups
0.14, 67, 1.2e+03, 0.18
Segregated solver iteration 11.
Merged variables
Iter      SolEst     Damping    Stepsize #Res #Jac #Sol   LinErr   LinRes
   1     0.00019   0.3500000     0.00019   42   31   31  6.8e-14  3.2e-15
   2     0.00012   0.3500000     0.00012   43   32   32  6.6e-14  3.3e-15
   3     8.1e-05   0.3500000     8.1e-05   44   33   33  1.2e-13  2.8e-15
Velocity u2, Pressure p
Iter      SolEst     Damping    Stepsize #Res #Jac #Sol   LinErr   LinRes
   1     0.00052   0.5000000     0.00052   22   11   11  4.7e-15  7.9e-15
Displacement field
Iter      SolEst     Damping    Stepsize #Res #Jac #Sol   LinErr   LinRes
   1      0.0051   0.5000000      0.0051   22   11   11  3.5e-14  1.5e-12
Spatial mesh displacement
Iter      SolEst     Damping    Stepsize #Res #Jac #Sol   LinErr   LinRes
   1     4.2e-08   0.5000000     4.2e-08   22   11   11  1.6e-15  1.8e-15
Solution error estimates for segregated groups
0.00014, 0.00026, 0.0026, 2.1e-08
Residual error estimates for segregated groups
0.043, 34, 1.2e+03, 0.17
Segregated solver iteration 12.
Merged variables
Iter      SolEst     Damping    Stepsize #Res #Jac #Sol   LinErr   LinRes
   1     5.2e-05   0.3500000     5.2e-05   46   34   34  6.6e-14  2.9e-15
   2     3.4e-05   0.3500000     3.4e-05   47   35   35  9.6e-14  3.1e-15
   3     2.2e-05   0.3500000     2.2e-05   48   36   36  9.8e-14  3.4e-15
Velocity u2, Pressure p
Iter      SolEst     Damping    Stepsize #Res #Jac #Sol   LinErr   LinRes
   1     0.00026   0.5000000     0.00026   24   12   12  5.4e-15  7.9e-15
Displacement field
Iter      SolEst     Damping    Stepsize #Res #Jac #Sol   LinErr   LinRes
   1      0.0028   0.5000000      0.0028   24   12   12  4.8e-14  1.5e-12
Spatial mesh displacement
Iter      SolEst     Damping    Stepsize #Res #Jac #Sol   LinErr   LinRes
   1     2.5e-08   0.5000000     2.5e-08   24   12   12  1.8e-15  1.8e-15
Solution error estimates for segregated groups
3.8e-05, 0.00013, 0.0014, 1.2e-08
```

```
Residual error estimates for segregated groups
0.013, 17, 1.2e+03, 0.17
Segregated solver iteration 13.
Merged variables
Iter      SolEst     Damping    Stepsize #Res #Jac #Sol   LinErr   LinRes
   1     1.4e-05   0.3500000     1.4e-05   50   37   37  4.3e-14    3e-15
   2     9.4e-06   0.3500000     9.4e-06   51   38   38  7.5e-14  3.2e-15
   3     6.1e-06   0.3500000     6.1e-06   52   39   39  1.6e-13  3.3e-15
Velocity u2, Pressure p
Iter      SolEst     Damping    Stepsize #Res #Jac #Sol   LinErr   LinRes
   1     0.00013   0.5000000     0.00013   26   13   13    3e-15  7.9e-15
Displacement field
Iter      SolEst     Damping    Stepsize #Res #Jac #Sol   LinErr   LinRes
   1      0.0015   0.5000000      0.0015   26   13   13  8.3e-14  1.5e-12
Spatial mesh displacement
Iter      SolEst     Damping    Stepsize #Res #Jac #Sol   LinErr   LinRes
   1     1.4e-08   0.5000000     1.4e-08   26   13   13  1.3e-15  1.8e-15
Solution error estimates for segregated groups
1e-05, 6.5e-05, 0.00076, 7.2e-09
Residual error estimates for segregated groups
0.004, 8.4, 1.1e+03, 0.17
Solution time: 157 s. (2 minutes, 37 seconds)
Physical memory: 4.72 GB
Virtual memory: 25.56 GB
Ended at Jan 31, 2025, 6:50:01 PM.
----- Stationary Solver 1 in study1/Approach Curve Solver (sol1) -------------->
```

d=2285 (su45)

GENERAL

Description	Value
Solution	d=2285

LOG

```
<---- Compile Equations: Stationary in study1/Approach Curve Solver (sol1) -----
Started at Jan 31, 2025, 6:50:01 PM.
Parameter d = 2.285E-6 (m).
Geometry shape function: Linear serendipity
Number of vertex elements: 18
Number of boundary elements: 6222
Number of elements: 155886
Minimum element quality: 0.4858
Time: 8 s.
Physical memory: 2.97 GB
Virtual memory: 15.24 GB
Ended at Jan 31, 2025, 6:50:10 PM.
----- Compile Equations: Stationary in study1/Approach Curve Solver (sol1) ---->

<---- Dependent Variables 1 in study1/Approach Curve Solver (sol1) -------------
Started at Jan 31, 2025, 6:50:10 PM.
Solution time: 0 s.
Physical memory: 2.97 GB
Virtual memory: 15.24 GB
Ended at Jan 31, 2025, 6:50:10 PM.
----- Dependent Variables 1 in study1/Approach Curve Solver (sol1) ------------>

<---- Stationary Solver 1 in study1/Approach Curve Solver (sol1) ---------------
Started at Jan 31, 2025, 6:50:10 PM.
Segregated solver
Segregated Step 11 does not have any components solved for.
Number of degrees of freedom solved for: 792481 (plus 11867 internal DOFs).
Segregated solver iteration 1.
Merged variables
Nonsymmetric matrix found.
Scales for dependent variables:
Concentration (comp1.Cl): 1.4e+02
Concentration (comp1.K): 1.4e+02
Electric potential (comp1.V): 1.9e+02
Orthonormal null-space function used.
Iter      SolEst     Damping    Stepsize #Res #Jac #Sol   LinErr   LinRes
   1        0.83   0.3500000        0.83    2    1    1    6e-11  3.1e-15
   2        0.81   0.3500000        0.81    3    2    2  2.4e-11  2.8e-15
   3         0.8   0.3500000         0.8    4    3    3    2e-11  2.7e-15
Velocity u2, Pressure p
Nonsymmetric matrix found.
Scales for dependent variables:
Pressure (comp1.p): 1
Velocity field (spatial frame) (comp1.u2): 0.00018
Orthonormal null-space function used.
Iter      SolEst     Damping    Stepsize #Res #Jac #Sol   LinErr   LinRes
   1         1.1   0.5000000         1.1    2    1    1  1.6e-15  2.3e-15
Displacement field
Nonsymmetric matrix found.
Scales for dependent variables:
Auxiliary pressure (comp1.solid.lemm1.pw): 1
Displacement field (comp1.u): 2.8e-18
Orthonormal null-space function used.
Iter      SolEst     Damping    Stepsize #Res #Jac #Sol   LinErr   LinRes
   1         1.9   0.5000000         1.9    2    1    1  7.6e-14  1.5e-12
Spatial mesh displacement
```

```
Symmetric matrices found.
Scales for dependent variables:
Spatial mesh displacement (comp1.spatial.disp): 1.2e-09
Orthonormal null-space function used.
Iter      SolEst     Damping    Stepsize #Res #Jac #Sol   LinErr   LinRes
   1     6.5e-07   0.5000000     6.5e-07    2    1    1  1.6e-15  1.9e-15
Solution error estimates for segregated groups
1.4, 0.54, 0.95, 3.2e-07
Residual error estimates for segregated groups
7.9e+03, 8.3e+04, 2.2e+04, 0.15
Segregated solver iteration 2.
Merged variables
Scales for dependent variables:
Concentration (comp1.Cl): 1.4e+02
Concentration (comp1.K): 1.4e+02
Electric potential (comp1.V): 0.064
Iter      SolEst     Damping    Stepsize #Res #Jac #Sol   LinErr   LinRes
   1        0.78   0.3500000        0.78    6    4    4  7.8e-14  3.8e-15
   2        0.77   0.3500000        0.77    7    5    5    1e-13  3.4e-15
   3        0.76   0.3500000        0.76    8    6    6  7.7e-14  4.5e-15
Velocity u2, Pressure p
Scales for dependent variables:
Pressure (comp1.p): 3.6e+02
Velocity field (spatial frame) (comp1.u2): 0.00072
Iter      SolEst     Damping    Stepsize #Res #Jac #Sol   LinErr   LinRes
   1        0.36   0.5000000        0.36    4    2    2    3e-15  7.7e-15
Displacement field
Iter      SolEst     Damping    Stepsize #Res #Jac #Sol   LinErr   LinRes
   1        0.95   0.5000000        0.95    4    2    2  4.9e-14  1.5e-12
Spatial mesh displacement
Iter      SolEst     Damping    Stepsize #Res #Jac #Sol   LinErr   LinRes
   1     9.7e-07   0.5000000     9.7e-07    4    2    2  1.8e-15  1.8e-15
Solution error estimates for segregated groups
1.3, 0.18, 0.48, 4.9e-07
Residual error estimates for segregated groups
7.9e+02, 1.5e+04, 2.2e+04, 0.15
Segregated solver iteration 3.
Merged variables
Iter      SolEst     Damping    Stepsize #Res #Jac #Sol   LinErr   LinRes
   1        0.75   0.3500000        0.75   10    7    7    4e-14  3.9e-15
   2        0.73   0.3500000        0.73   11    8    8  1.1e-13  4.4e-15
   3        0.72   0.3500000        0.72   12    9    9  1.3e-13  4.6e-15
Velocity u2, Pressure p
Iter      SolEst     Damping    Stepsize #Res #Jac #Sol   LinErr   LinRes
   1        0.15   0.5000000        0.15    6    3    3  2.8e-15  7.9e-15
Displacement field
Iter      SolEst     Damping    Stepsize #Res #Jac #Sol   LinErr   LinRes
   1        0.52   0.5000000        0.52    6    3    3  3.4e-14  1.5e-12
Spatial mesh displacement
Iter      SolEst     Damping    Stepsize #Res #Jac #Sol   LinErr   LinRes
   1     9.7e-07   0.5000000     9.7e-07    6    3    3  1.6e-15  1.9e-15
Solution error estimates for segregated groups
1.2, 0.077, 0.26, 4.9e-07
Residual error estimates for segregated groups
2.2e+02, 8.4e+03, 1.6e+04, 0.17
Segregated solver iteration 4.
```

```
Merged variables
Iter      SolEst     Damping    Stepsize #Res #Jac #Sol   LinErr   LinRes
   1         0.7   0.3500000         0.7   14   10   10  8.6e-14  3.1e-15
   2        0.68   0.3500000        0.68   15   11   11  1.2e-13    4e-15
   3         0.6   0.3500000         0.6   16   12   12  9.6e-14  5.4e-15
Velocity u2, Pressure p
Iter      SolEst     Damping    Stepsize #Res #Jac #Sol   LinErr   LinRes
   1       0.071   0.5000000       0.071    8    4    4  3.1e-15    8e-15
Displacement field
Iter      SolEst     Damping    Stepsize #Res #Jac #Sol   LinErr   LinRes
   1        0.29   0.5000000        0.29    8    4    4  3.9e-14  1.5e-12
Spatial mesh displacement
Iter      SolEst     Damping    Stepsize #Res #Jac #Sol   LinErr   LinRes
   1     8.1e-07   0.5000000     8.1e-07    8    4    4  1.3e-15  1.9e-15
Solution error estimates for segregated groups
1, 0.036, 0.15, 4.1e-07
Residual error estimates for segregated groups
84, 4.4e+03, 1.1e+04, 0.17
Segregated solver iteration 5.
Merged variables
Iter      SolEst     Damping    Stepsize #Res #Jac #Sol   LinErr   LinRes
   1        0.41   0.3500000        0.41   18   13   13  8.9e-14  3.6e-15
   2        0.28   0.3500000        0.28   19   14   14  1.1e-13  3.7e-15
   3        0.18   0.3500000        0.18   20   15   15  3.8e-14    4e-15
Velocity u2, Pressure p
Iter      SolEst     Damping    Stepsize #Res #Jac #Sol   LinErr   LinRes
   1       0.035   0.5000000       0.035   10    5    5  2.5e-15  7.9e-15
Displacement field
Iter      SolEst     Damping    Stepsize #Res #Jac #Sol   LinErr   LinRes
   1        0.17   0.5000000        0.17   10    5    5    6e-14  1.5e-12
Spatial mesh displacement
Iter      SolEst     Damping    Stepsize #Res #Jac #Sol   LinErr   LinRes
   1     6.1e-07   0.5000000     6.1e-07   10    5    5    2e-15  1.9e-15
Solution error estimates for segregated groups
0.31, 0.017, 0.084, 3e-07
Residual error estimates for segregated groups
33, 2.3e+03, 6.9e+03, 0.17
Segregated solver iteration 6.
Merged variables
Iter      SolEst     Damping    Stepsize #Res #Jac #Sol   LinErr   LinRes
   1        0.12   0.3500000        0.12   22   16   16  4.6e-14  3.4e-15
   2       0.079   0.3500000       0.079   23   17   17  1.4e-13  3.7e-15
   3       0.051   0.3500000       0.051   24   18   18  5.2e-14  4.1e-15
Velocity u2, Pressure p
Iter      SolEst     Damping    Stepsize #Res #Jac #Sol   LinErr   LinRes
   1       0.017   0.5000000       0.017   12    6    6  2.2e-15  7.7e-15
Displacement field
Iter      SolEst     Damping    Stepsize #Res #Jac #Sol   LinErr   LinRes
   1       0.095   0.5000000       0.095   12    6    6  1.3e-13  1.5e-12
Spatial mesh displacement
Iter      SolEst     Damping    Stepsize #Res #Jac #Sol   LinErr   LinRes
   1     4.3e-07   0.5000000     4.3e-07   12    6    6  1.6e-15  1.8e-15
Solution error estimates for segregated groups
0.088, 0.0085, 0.048, 2.1e-07
Residual error estimates for segregated groups
12, 1.2e+03, 4.2e+03, 0.17
```

```
Segregated solver iteration 7.
Merged variables
Iter      SolEst     Damping    Stepsize #Res #Jac #Sol   LinErr   LinRes
   1       0.033   0.3500000       0.033   26   19   19  6.3e-14  3.9e-15
   2       0.022   0.3500000       0.022   27   20   20    6e-14  3.9e-15
   3       0.014   0.3500000       0.014   28   21   21  5.4e-14  3.5e-15
Velocity u2, Pressure p
Iter      SolEst     Damping    Stepsize #Res #Jac #Sol   LinErr   LinRes
   1      0.0084   0.5000000      0.0084   14    7    7    3e-15    8e-15
Displacement field
Iter      SolEst     Damping    Stepsize #Res #Jac #Sol   LinErr   LinRes
   1       0.054   0.5000000       0.054   14    7    7  8.3e-14  1.5e-12
Spatial mesh displacement
Iter      SolEst     Damping    Stepsize #Res #Jac #Sol   LinErr   LinRes
   1     2.8e-07   0.5000000     2.8e-07   14    7    7  2.1e-15  1.9e-15
Solution error estimates for segregated groups
0.024, 0.0042, 0.027, 1.4e-07
Residual error estimates for segregated groups
4.1, 5.8e+02, 2.6e+03, 0.2
Segregated solver iteration 8.
Merged variables
Iter      SolEst     Damping    Stepsize #Res #Jac #Sol   LinErr   LinRes
   1      0.0092   0.3500000      0.0092   30   22   22  7.2e-14  4.1e-15
   2       0.006   0.3500000       0.006   31   23   23  6.7e-14  4.8e-15
   3      0.0039   0.3500000      0.0039   32   24   24    1e-13  4.9e-15
Velocity u2, Pressure p
Iter      SolEst     Damping    Stepsize #Res #Jac #Sol   LinErr   LinRes
   1      0.0042   0.5000000      0.0042   16    8    8  3.4e-15  7.8e-15
Displacement field
Iter      SolEst     Damping    Stepsize #Res #Jac #Sol   LinErr   LinRes
   1        0.03   0.5000000        0.03   16    8    8  1.6e-13  1.5e-12
Spatial mesh displacement
Iter      SolEst     Damping    Stepsize #Res #Jac #Sol   LinErr   LinRes
   1     1.8e-07   0.5000000     1.8e-07   16    8    8    2e-15  1.9e-15
Solution error estimates for segregated groups
0.0067, 0.0021, 0.015, 9.1e-08
Residual error estimates for segregated groups
1.4, 2.9e+02, 1.8e+03, 0.17
Segregated solver iteration 9.
Merged variables
Iter      SolEst     Damping    Stepsize #Res #Jac #Sol   LinErr   LinRes
   1      0.0025   0.3500000      0.0025   34   25   25  1.1e-13  3.5e-15
   2      0.0016   0.3500000      0.0016   35   26   26  7.9e-14    4e-15
   3      0.0011   0.3500000      0.0011   36   27   27  8.3e-14    4e-15
Velocity u2, Pressure p
Iter      SolEst     Damping    Stepsize #Res #Jac #Sol   LinErr   LinRes
   1      0.0021   0.5000000      0.0021   18    9    9  3.6e-15  7.8e-15
Displacement field
Iter      SolEst     Damping    Stepsize #Res #Jac #Sol   LinErr   LinRes
   1       0.017   0.5000000       0.017   18    9    9  3.1e-14  1.5e-12
Spatial mesh displacement
Iter      SolEst     Damping    Stepsize #Res #Jac #Sol   LinErr   LinRes
   1     1.1e-07   0.5000000     1.1e-07   18    9    9  1.4e-15  1.8e-15
Solution error estimates for segregated groups
0.0018, 0.001, 0.0085, 5.7e-08
Residual error estimates for segregated groups
```

```
0.44, 1.5e+02, 1.4e+03, 0.17
Segregated solver iteration 10.
Merged variables
Iter      SolEst     Damping    Stepsize #Res #Jac #Sol   LinErr   LinRes
   1      0.0007   0.3500000      0.0007   38   28   28  4.7e-14  4.9e-15
   2     0.00045   0.3500000     0.00045   39   29   29  8.4e-14  3.8e-15
   3     0.00029   0.3500000     0.00029   40   30   30  5.1e-14    3e-15
Velocity u2, Pressure p
Iter      SolEst     Damping    Stepsize #Res #Jac #Sol   LinErr   LinRes
   1       0.001   0.5000000       0.001   20   10   10  3.9e-15  7.8e-15
Displacement field
Iter      SolEst     Damping    Stepsize #Res #Jac #Sol   LinErr   LinRes
   1      0.0094   0.5000000      0.0094   20   10   10  9.6e-14  1.5e-12
Spatial mesh displacement
Iter      SolEst     Damping    Stepsize #Res #Jac #Sol   LinErr   LinRes
   1       7e-08   0.5000000       7e-08   20   10   10  1.7e-15  1.9e-15
Solution error estimates for segregated groups
0.0005, 0.00052, 0.0047, 3.5e-08
Residual error estimates for segregated groups
0.14, 73, 1.2e+03, 0.16
Segregated solver iteration 11.
Merged variables
Iter      SolEst     Damping    Stepsize #Res #Jac #Sol   LinErr   LinRes
   1     0.00019   0.3500000     0.00019   42   31   31    1e-13  3.2e-15
   2     0.00012   0.3500000     0.00012   43   32   32  1.1e-13  4.1e-15
   3     8.1e-05   0.3500000     8.1e-05   44   33   33  7.2e-14  3.8e-15
Velocity u2, Pressure p
Iter      SolEst     Damping    Stepsize #Res #Jac #Sol   LinErr   LinRes
   1     0.00052   0.5000000     0.00052   22   11   11  2.1e-15    8e-15
Displacement field
Iter      SolEst     Damping    Stepsize #Res #Jac #Sol   LinErr   LinRes
   1      0.0051   0.5000000      0.0051   22   11   11  1.3e-13  1.5e-12
Spatial mesh displacement
Iter      SolEst     Damping    Stepsize #Res #Jac #Sol   LinErr   LinRes
   1     4.2e-08   0.5000000     4.2e-08   22   11   11  1.7e-15  1.9e-15
Solution error estimates for segregated groups
0.00014, 0.00026, 0.0026, 2.1e-08
Residual error estimates for segregated groups
0.044, 37, 1.2e+03, 0.19
Segregated solver iteration 12.
Merged variables
Iter      SolEst     Damping    Stepsize #Res #Jac #Sol   LinErr   LinRes
   1     5.2e-05   0.3500000     5.2e-05   46   34   34  1.3e-13  3.3e-15
   2     3.4e-05   0.3500000     3.4e-05   47   35   35  4.9e-14  3.9e-15
   3     2.2e-05   0.3500000     2.2e-05   48   36   36  5.2e-14  4.1e-15
Velocity u2, Pressure p
Iter      SolEst     Damping    Stepsize #Res #Jac #Sol   LinErr   LinRes
   1     0.00026   0.5000000     0.00026   24   12   12  2.3e-15  7.9e-15
Displacement field
Iter      SolEst     Damping    Stepsize #Res #Jac #Sol   LinErr   LinRes
   1      0.0028   0.5000000      0.0028   24   12   12  5.6e-14  1.5e-12
Spatial mesh displacement
Iter      SolEst     Damping    Stepsize #Res #Jac #Sol   LinErr   LinRes
   1     2.5e-08   0.5000000     2.5e-08   24   12   12  1.6e-15  1.9e-15
Solution error estimates for segregated groups
3.8e-05, 0.00013, 0.0014, 1.2e-08
```

```
Residual error estimates for segregated groups
0.013, 18, 1.2e+03, 0.17
Segregated solver iteration 13.
Merged variables
Iter      SolEst      Damping    Stepsize #Res #Jac #Sol   LinErr   LinRes
   1      1.4e-05   0.3500000     1.4e-05   50   37   37  1.2e-13  5.2e-15
   2      9.4e-06   0.3500000     9.4e-06   51   38   38  1.2e-13  3.8e-15
   3      6.1e-06   0.3500000     6.1e-06   52   39   39    5e-14  7.5e-15
Velocity u2, Pressure p
Iter      SolEst      Damping    Stepsize #Res #Jac #Sol   LinErr   LinRes
   1      0.00013   0.5000000     0.00013   26   13   13  2.2e-15  7.7e-15
Displacement field
Iter      SolEst      Damping    Stepsize #Res #Jac #Sol   LinErr   LinRes
   1       0.0015   0.5000000      0.0015   26   13   13  1.6e-13  1.5e-12
Spatial mesh displacement
Iter      SolEst      Damping    Stepsize #Res #Jac #Sol   LinErr   LinRes
   1      1.4e-08   0.5000000     1.4e-08   26   13   13  1.9e-15  1.9e-15
Solution error estimates for segregated groups
1e-05, 6.5e-05, 0.00076, 7.2e-09
Residual error estimates for segregated groups
0.0041, 9.1, 1.2e+03, 0.17
Solution time: 156 s. (2 minutes, 36 seconds)
Physical memory: 4.61 GB
Virtual memory: 25.49 GB
Ended at Jan 31, 2025, 6:52:45 PM.
----- Stationary Solver 1 in study1/Approach Curve Solver (sol1) -------------->
```

d=2280 (su46)

GENERAL

Description	Value
Solution	d=2280

LOG

```
<---- Compile Equations: Stationary in study1/Approach Curve Solver (sol1) -----
Started at Jan 31, 2025, 6:52:46 PM.
Parameter d = 2.28E-6 (m).
Geometry shape function: Linear serendipity
Number of vertex elements: 18
Number of boundary elements: 6219
Number of elements: 155777
Minimum element quality: 0.4808
Time: 8 s.
Physical memory: 2.98 GB
Virtual memory: 15.25 GB
Ended at Jan 31, 2025, 6:52:54 PM.
----- Compile Equations: Stationary in study1/Approach Curve Solver (sol1) ---->

<---- Dependent Variables 1 in study1/Approach Curve Solver (sol1) -------------
Started at Jan 31, 2025, 6:52:54 PM.
Solution time: 0 s.
Physical memory: 2.98 GB
Virtual memory: 15.25 GB
Ended at Jan 31, 2025, 6:52:54 PM.
----- Dependent Variables 1 in study1/Approach Curve Solver (sol1) ------------>

<---- Stationary Solver 1 in study1/Approach Curve Solver (sol1) ---------------
Started at Jan 31, 2025, 6:52:54 PM.
Segregated solver
Segregated Step 11 does not have any components solved for.
Number of degrees of freedom solved for: 791768 (plus 11861 internal DOFs).
Segregated solver iteration 1.
Merged variables
Nonsymmetric matrix found.
Scales for dependent variables:
Concentration (comp1.Cl): 1.4e+02
Concentration (comp1.K): 1.4e+02
Electric potential (comp1.V): 1.9e+02
Orthonormal null-space function used.
Iter      SolEst     Damping    Stepsize #Res #Jac #Sol   LinErr   LinRes
   1        0.83   0.3500000        0.83    2    1    1  4.4e-11  2.7e-15
   2        0.81   0.3500000        0.81    3    2    2  4.5e-11  2.7e-15
   3         0.8   0.3500000         0.8    4    3    3    2e-11  2.6e-15
Velocity u2, Pressure p
Nonsymmetric matrix found.
Scales for dependent variables:
Pressure (comp1.p): 1
Velocity field (spatial frame) (comp1.u2): 0.00018
Orthonormal null-space function used.
Iter      SolEst     Damping    Stepsize #Res #Jac #Sol   LinErr   LinRes
   1         1.1   0.5000000         1.1    2    1    1  1.6e-15  2.2e-15
Displacement field
Nonsymmetric matrix found.
Scales for dependent variables:
Auxiliary pressure (comp1.solid.lemm1.pw): 1
Displacement field (comp1.u): 2.8e-18
Orthonormal null-space function used.
Iter      SolEst     Damping    Stepsize #Res #Jac #Sol   LinErr   LinRes
   1         1.9   0.5000000         1.9    2    1    1  1.2e-13  1.5e-12
Spatial mesh displacement
```

```
Symmetric matrices found.
Scales for dependent variables:
Spatial mesh displacement (comp1.spatial.disp): 1.2e-09
Orthonormal null-space function used.
Iter      SolEst     Damping    Stepsize #Res #Jac #Sol   LinErr   LinRes
   1     6.5e-07   0.5000000     6.5e-07    2    1    1  1.6e-15  1.8e-15
Solution error estimates for segregated groups
1.4, 0.54, 0.95, 3.3e-07
Residual error estimates for segregated groups
7.9e+03, 8.3e+04, 2.2e+04, 0.16
Segregated solver iteration 2.
Merged variables
Scales for dependent variables:
Concentration (comp1.Cl): 1.4e+02
Concentration (comp1.K): 1.4e+02
Electric potential (comp1.V): 0.064
Iter      SolEst     Damping    Stepsize #Res #Jac #Sol   LinErr   LinRes
   1        0.78   0.3500000        0.78    6    4    4  9.2e-14    9e-15
   2        0.77   0.3500000        0.77    7    5    5  1.1e-13  4.4e-15
   3        0.76   0.3500000        0.76    8    6    6  1.4e-13  4.7e-15
Velocity u2, Pressure p
Scales for dependent variables:
Pressure (comp1.p): 3.6e+02
Velocity field (spatial frame) (comp1.u2): 0.00072
Iter      SolEst     Damping    Stepsize #Res #Jac #Sol   LinErr   LinRes
   1        0.36   0.5000000        0.36    4    2    2  2.8e-15  7.8e-15
Displacement field
Iter      SolEst     Damping    Stepsize #Res #Jac #Sol   LinErr   LinRes
   1        0.95   0.5000000        0.95    4    2    2  9.7e-14  1.5e-12
Spatial mesh displacement
Iter      SolEst     Damping    Stepsize #Res #Jac #Sol   LinErr   LinRes
   1     9.8e-07   0.5000000     9.8e-07    4    2    2  2.2e-15  1.8e-15
Solution error estimates for segregated groups
1.3, 0.18, 0.48, 4.9e-07
Residual error estimates for segregated groups
8e+02, 1.4e+04, 2.2e+04, 0.16
Segregated solver iteration 3.
Merged variables
Iter      SolEst     Damping    Stepsize #Res #Jac #Sol   LinErr   LinRes
   1        0.75   0.3500000        0.75   10    7    7  1.5e-13  3.5e-15
   2        0.73   0.3500000        0.73   11    8    8  6.8e-14  4.4e-15
   3        0.72   0.3500000        0.72   12    9    9  6.7e-14  3.6e-15
Velocity u2, Pressure p
Iter      SolEst     Damping    Stepsize #Res #Jac #Sol   LinErr   LinRes
   1        0.15   0.5000000        0.15    6    3    3  3.5e-15  7.9e-15
Displacement field
Iter      SolEst     Damping    Stepsize #Res #Jac #Sol   LinErr   LinRes
   1        0.52   0.5000000        0.52    6    3    3  6.9e-14  1.5e-12
Spatial mesh displacement
Iter      SolEst     Damping    Stepsize #Res #Jac #Sol   LinErr   LinRes
   1     9.8e-07   0.5000000     9.8e-07    6    3    3  1.5e-15  1.9e-15
Solution error estimates for segregated groups
1.2, 0.077, 0.26, 4.9e-07
Residual error estimates for segregated groups
2.2e+02, 7.6e+03, 1.6e+04, 0.17
Segregated solver iteration 4.
```

```
Merged variables
Iter      SolEst     Damping    Stepsize #Res #Jac #Sol   LinErr   LinRes
   1         0.7   0.3500000         0.7   14   10   10  5.7e-14  6.9e-15
   2        0.68   0.3500000        0.68   15   11   11  6.6e-14  5.8e-15
   3         0.6   0.3500000         0.6   16   12   12  6.3e-14  3.4e-15
Velocity u2, Pressure p
Iter      SolEst     Damping    Stepsize #Res #Jac #Sol   LinErr   LinRes
   1       0.072   0.5000000       0.072    8    4    4  3.2e-15  7.9e-15
Displacement field
Iter      SolEst     Damping    Stepsize #Res #Jac #Sol   LinErr   LinRes
   1        0.29   0.5000000        0.29    8    4    4  8.9e-14  1.5e-12
Spatial mesh displacement
Iter      SolEst     Damping    Stepsize #Res #Jac #Sol   LinErr   LinRes
   1     8.2e-07   0.5000000     8.2e-07    8    4    4  1.8e-15  1.8e-15
Solution error estimates for segregated groups
1, 0.036, 0.15, 4.1e-07
Residual error estimates for segregated groups
84, 4e+03, 1.1e+04, 0.17
Segregated solver iteration 5.
Merged variables
Iter      SolEst     Damping    Stepsize #Res #Jac #Sol   LinErr   LinRes
   1        0.41   0.3500000        0.41   18   13   13  5.9e-14  3.8e-15
   2        0.28   0.3500000        0.28   19   14   14  9.8e-14  6.3e-15
   3        0.18   0.3500000        0.18   20   15   15  8.4e-14  3.1e-15
Velocity u2, Pressure p
Iter      SolEst     Damping    Stepsize #Res #Jac #Sol   LinErr   LinRes
   1       0.035   0.5000000       0.035   10    5    5  2.4e-15  7.8e-15
Displacement field
Iter      SolEst     Damping    Stepsize #Res #Jac #Sol   LinErr   LinRes
   1        0.17   0.5000000        0.17   10    5    5  8.2e-14  1.5e-12
Spatial mesh displacement
Iter      SolEst     Damping    Stepsize #Res #Jac #Sol   LinErr   LinRes
   1     6.1e-07   0.5000000     6.1e-07   10    5    5  2.1e-15  1.9e-15
Solution error estimates for segregated groups
0.31, 0.017, 0.084, 3.1e-07
Residual error estimates for segregated groups
33, 2.1e+03, 6.9e+03, 0.17
Segregated solver iteration 6.
Merged variables
Iter      SolEst     Damping    Stepsize #Res #Jac #Sol   LinErr   LinRes
   1        0.12   0.3500000        0.12   22   16   16  7.3e-14  8.6e-15
   2       0.078   0.3500000       0.078   23   17   17  1.2e-13  7.8e-15
   3       0.051   0.3500000       0.051   24   18   18  8.5e-14  6.9e-15
Velocity u2, Pressure p
Iter      SolEst     Damping    Stepsize #Res #Jac #Sol   LinErr   LinRes
   1       0.017   0.5000000       0.017   12    6    6  2.8e-15  7.8e-15
Displacement field
Iter      SolEst     Damping    Stepsize #Res #Jac #Sol   LinErr   LinRes
   1       0.095   0.5000000       0.095   12    6    6  6.7e-14  1.5e-12
Spatial mesh displacement
Iter      SolEst     Damping    Stepsize #Res #Jac #Sol   LinErr   LinRes
   1     4.3e-07   0.5000000     4.3e-07   12    6    6  1.3e-15  1.8e-15
Solution error estimates for segregated groups
0.088, 0.0085, 0.048, 2.1e-07
Residual error estimates for segregated groups
12, 1e+03, 4.2e+03, 0.18
```

```
Segregated solver iteration 7.
Merged variables
Iter      SolEst     Damping    Stepsize #Res #Jac #Sol   LinErr   LinRes
   1       0.033   0.3500000       0.033   26   19   19  1.3e-13  3.1e-15
   2       0.022   0.3500000       0.022   27   20   20  6.9e-14  7.7e-15
   3       0.014   0.3500000       0.014   28   21   21  7.4e-14  7.3e-15
Velocity u2, Pressure p
Iter      SolEst     Damping    Stepsize #Res #Jac #Sol   LinErr   LinRes
   1      0.0085   0.5000000      0.0085   14    7    7  5.3e-15  7.9e-15
Displacement field
Iter      SolEst     Damping    Stepsize #Res #Jac #Sol   LinErr   LinRes
   1       0.054   0.5000000       0.054   14    7    7  1.1e-13  1.5e-12
Spatial mesh displacement
Iter      SolEst     Damping    Stepsize #Res #Jac #Sol   LinErr   LinRes
   1     2.9e-07   0.5000000     2.9e-07   14    7    7  1.7e-15  1.8e-15
Solution error estimates for segregated groups
0.024, 0.0042, 0.027, 1.4e-07
Residual error estimates for segregated groups
4.1, 5.2e+02, 2.6e+03, 0.16
Segregated solver iteration 8.
Merged variables
Iter      SolEst     Damping    Stepsize #Res #Jac #Sol   LinErr   LinRes
   1      0.0092   0.3500000      0.0092   30   22   22  7.9e-14  3.4e-15
   2       0.006   0.3500000       0.006   31   23   23  6.6e-14  3.4e-15
   3      0.0039   0.3500000      0.0039   32   24   24  5.1e-14  5.3e-15
Velocity u2, Pressure p
Iter      SolEst     Damping    Stepsize #Res #Jac #Sol   LinErr   LinRes
   1      0.0042   0.5000000      0.0042   16    8    8  2.6e-15  7.8e-15
Displacement field
Iter      SolEst     Damping    Stepsize #Res #Jac #Sol   LinErr   LinRes
   1        0.03   0.5000000        0.03   16    8    8  2.6e-14  1.5e-12
Spatial mesh displacement
Iter      SolEst     Damping    Stepsize #Res #Jac #Sol   LinErr   LinRes
   1     1.8e-07   0.5000000     1.8e-07   16    8    8  1.7e-15  1.8e-15
Solution error estimates for segregated groups
0.0067, 0.0021, 0.015, 9.2e-08
Residual error estimates for segregated groups
1.4, 2.6e+02, 1.8e+03, 0.17
Segregated solver iteration 9.
Merged variables
Iter      SolEst     Damping    Stepsize #Res #Jac #Sol   LinErr   LinRes
   1      0.0025   0.3500000      0.0025   34   25   25  7.9e-14  7.7e-15
   2      0.0016   0.3500000      0.0016   35   26   26  9.5e-14  3.6e-15
   3      0.0011   0.3500000      0.0011   36   27   27  5.9e-14  5.6e-15
Velocity u2, Pressure p
Iter      SolEst     Damping    Stepsize #Res #Jac #Sol   LinErr   LinRes
   1      0.0021   0.5000000      0.0021   18    9    9  2.6e-15  7.9e-15
Displacement field
Iter      SolEst     Damping    Stepsize #Res #Jac #Sol   LinErr   LinRes
   1       0.017   0.5000000       0.017   18    9    9  2.6e-13  1.5e-12
Spatial mesh displacement
Iter      SolEst     Damping    Stepsize #Res #Jac #Sol   LinErr   LinRes
   1     1.2e-07   0.5000000     1.2e-07   18    9    9  1.7e-15  1.8e-15
Solution error estimates for segregated groups
0.0018, 0.0011, 0.0085, 5.8e-08
Residual error estimates for segregated groups
```

```
0.44, 1.3e+02, 1.4e+03, 0.17
Segregated solver iteration 10.
Merged variables
Iter      SolEst     Damping    Stepsize #Res #Jac #Sol   LinErr   LinRes
   1     0.00069   0.3500000     0.00069   38   28   28  7.7e-14  5.3e-15
   2     0.00045   0.3500000     0.00045   39   29   29  4.8e-14  3.3e-15
   3     0.00029   0.3500000     0.00029   40   30   30  8.6e-14  4.5e-15
Velocity u2, Pressure p
Iter      SolEst     Damping    Stepsize #Res #Jac #Sol   LinErr   LinRes
   1       0.001   0.5000000       0.001   20   10   10  2.7e-15  7.7e-15
Displacement field
Iter      SolEst     Damping    Stepsize #Res #Jac #Sol   LinErr   LinRes
   1      0.0094   0.5000000      0.0094   20   10   10  5.9e-14  1.5e-12
Spatial mesh displacement
Iter      SolEst     Damping    Stepsize #Res #Jac #Sol   LinErr   LinRes
   1       7e-08   0.5000000       7e-08   20   10   10  1.3e-15  1.8e-15
Solution error estimates for segregated groups
0.0005, 0.00052, 0.0047, 3.5e-08
Residual error estimates for segregated groups
0.14, 66, 1.2e+03, 0.16
Segregated solver iteration 11.
Merged variables
Iter      SolEst     Damping    Stepsize #Res #Jac #Sol   LinErr   LinRes
   1     0.00019   0.3500000     0.00019   42   31   31    9e-14  6.5e-15
   2     0.00012   0.3500000     0.00012   43   32   32  1.5e-13  4.5e-15
   3       8e-05   0.3500000       8e-05   44   33   33  6.2e-14  6.6e-15
Velocity u2, Pressure p
Iter      SolEst     Damping    Stepsize #Res #Jac #Sol   LinErr   LinRes
   1     0.00052   0.5000000     0.00052   22   11   11  3.2e-15  7.9e-15
Displacement field
Iter      SolEst     Damping    Stepsize #Res #Jac #Sol   LinErr   LinRes
   1      0.0051   0.5000000      0.0051   22   11   11  9.3e-14  1.5e-12
Spatial mesh displacement
Iter      SolEst     Damping    Stepsize #Res #Jac #Sol   LinErr   LinRes
   1     4.2e-08   0.5000000     4.2e-08   22   11   11  1.5e-15  1.9e-15
Solution error estimates for segregated groups
0.00014, 0.00026, 0.0026, 2.1e-08
Residual error estimates for segregated groups
0.043, 33, 1.2e+03, 0.16
Segregated solver iteration 12.
Merged variables
Iter      SolEst     Damping    Stepsize #Res #Jac #Sol   LinErr   LinRes
   1     5.2e-05   0.3500000     5.2e-05   46   34   34    6e-14  3.5e-15
   2     3.4e-05   0.3500000     3.4e-05   47   35   35  7.8e-14  5.5e-15
   3     2.2e-05   0.3500000     2.2e-05   48   36   36  5.4e-14  9.7e-15
Velocity u2, Pressure p
Iter      SolEst     Damping    Stepsize #Res #Jac #Sol   LinErr   LinRes
   1     0.00026   0.5000000     0.00026   24   12   12  3.3e-15  7.9e-15
Displacement field
Iter      SolEst     Damping    Stepsize #Res #Jac #Sol   LinErr   LinRes
   1      0.0028   0.5000000      0.0028   24   12   12  3.1e-14  1.5e-12
Spatial mesh displacement
Iter      SolEst     Damping    Stepsize #Res #Jac #Sol   LinErr   LinRes
   1     2.5e-08   0.5000000     2.5e-08   24   12   12  1.9e-15  1.8e-15
Solution error estimates for segregated groups
3.8e-05, 0.00013, 0.0014, 1.2e-08
```

```
Residual error estimates for segregated groups
0.013, 16, 1.2e+03, 0.17
Segregated solver iteration 13.
Merged variables
Iter      SolEst     Damping    Stepsize #Res #Jac #Sol   LinErr   LinRes
   1     1.4e-05   0.3500000     1.4e-05   50   37   37  1.4e-13  3.9e-15
   2     9.3e-06   0.3500000     9.3e-06   51   38   38  1.2e-13    8e-15
   3     6.1e-06   0.3500000     6.1e-06   52   39   39  8.4e-14  3.3e-15
Velocity u2, Pressure p
Iter      SolEst     Damping    Stepsize #Res #Jac #Sol   LinErr   LinRes
   1     0.00013   0.5000000     0.00013   26   13   13  1.9e-15  7.9e-15
Displacement field
Iter      SolEst     Damping    Stepsize #Res #Jac #Sol   LinErr   LinRes
   1      0.0015   0.5000000      0.0015   26   13   13  8.5e-14  1.5e-12
Spatial mesh displacement
Iter      SolEst     Damping    Stepsize #Res #Jac #Sol   LinErr   LinRes
   1     1.5e-08   0.5000000     1.5e-08   26   13   13  1.8e-15  1.9e-15
Solution error estimates for segregated groups
1e-05, 6.6e-05, 0.00076, 7.3e-09
Residual error estimates for segregated groups
0.004, 8.2, 1.2e+03, 0.16
Solution time: 157 s. (2 minutes, 37 seconds)
Physical memory: 4.73 GB
Virtual memory: 25.87 GB
Ended at Jan 31, 2025, 6:55:31 PM.
----- Stationary Solver 1 in study1/Approach Curve Solver (sol1) -------------->
```

d=2275 (su47)

GENERAL

Description	Value
Solution	d=2275

LOG

```
<---- Compile Equations: Stationary in study1/Approach Curve Solver (sol1) -----
Started at Jan 31, 2025, 6:55:31 PM.
Parameter d = 2.2750000000000002E-6 (m).
Geometry shape function: Linear serendipity
Number of vertex elements: 18
Number of boundary elements: 6217
Number of elements: 156917
Minimum element quality: 0.5143
Time: 15 s.
Physical memory: 2.97 GB
Virtual memory: 15.3 GB
Ended at Jan 31, 2025, 6:55:46 PM.
----- Compile Equations: Stationary in study1/Approach Curve Solver (sol1) ---->

<---- Dependent Variables 1 in study1/Approach Curve Solver (sol1) -------------
Started at Jan 31, 2025, 6:55:46 PM.
Solution time: 0 s.
Physical memory: 2.97 GB
Virtual memory: 15.3 GB
Ended at Jan 31, 2025, 6:55:46 PM.
----- Dependent Variables 1 in study1/Approach Curve Solver (sol1) ------------>

<---- Stationary Solver 1 in study1/Approach Curve Solver (sol1) ---------------
Started at Jan 31, 2025, 6:55:46 PM.
Segregated solver
Segregated Step 11 does not have any components solved for.
Number of degrees of freedom solved for: 797923 (plus 11857 internal DOFs).
Segregated solver iteration 1.
Merged variables
Nonsymmetric matrix found.
Scales for dependent variables:
Concentration (comp1.Cl): 1.4e+02
Concentration (comp1.K): 1.4e+02
Electric potential (comp1.V): 1.9e+02
Orthonormal null-space function used.
Iter      SolEst     Damping    Stepsize #Res #Jac #Sol   LinErr   LinRes
   1        0.83   0.3500000        0.83    2    1    1  6.1e-11  2.6e-15
   2        0.81   0.3500000        0.81    3    2    2    6e-11  2.6e-15
   3         0.8   0.3500000         0.8    4    3    3    2e-11  2.6e-15
Velocity u2, Pressure p
Nonsymmetric matrix found.
Scales for dependent variables:
Pressure (comp1.p): 1
Velocity field (spatial frame) (comp1.u2): 0.00018
Orthonormal null-space function used.
Iter      SolEst     Damping    Stepsize #Res #Jac #Sol   LinErr   LinRes
   1         1.1   0.5000000         1.1    2    1    1  1.9e-15  2.2e-15
Displacement field
Nonsymmetric matrix found.
Scales for dependent variables:
Auxiliary pressure (comp1.solid.lemm1.pw): 1
Displacement field (comp1.u): 2.7e-18
Orthonormal null-space function used.
Iter      SolEst     Damping    Stepsize #Res #Jac #Sol   LinErr   LinRes
   1         1.9   0.5000000         1.9    2    1    1  3.3e-14  1.5e-12
Spatial mesh displacement
```

```
Symmetric matrices found.
Scales for dependent variables:
Spatial mesh displacement (comp1.spatial.disp): 1.2e-09
Orthonormal null-space function used.
Iter      SolEst     Damping    Stepsize #Res #Jac #Sol   LinErr   LinRes
   1     6.4e-07   0.5000000     6.4e-07    2    1    1  1.7e-15  1.7e-15
Solution error estimates for segregated groups
1.4, 0.54, 0.95, 3.2e-07
Residual error estimates for segregated groups
7.9e+03, 8.3e+04, 2.2e+04, 0.13
Segregated solver iteration 2.
Merged variables
Scales for dependent variables:
Concentration (comp1.Cl): 1.4e+02
Concentration (comp1.K): 1.4e+02
Electric potential (comp1.V): 0.064
Iter      SolEst     Damping    Stepsize #Res #Jac #Sol   LinErr   LinRes
   1        0.79   0.3500000        0.79    6    4    4  7.9e-14    3e-15
   2        0.77   0.3500000        0.77    7    5    5  9.4e-14  2.9e-15
   3        0.76   0.3500000        0.76    8    6    6  3.4e-14  3.2e-15
Velocity u2, Pressure p
Scales for dependent variables:
Pressure (comp1.p): 3.5e+02
Velocity field (spatial frame) (comp1.u2): 0.00071
Iter      SolEst     Damping    Stepsize #Res #Jac #Sol   LinErr   LinRes
   1        0.36   0.5000000        0.36    4    2    2  2.8e-15  7.7e-15
Displacement field
Iter      SolEst     Damping    Stepsize #Res #Jac #Sol   LinErr   LinRes
   1        0.95   0.5000000        0.95    4    2    2  8.4e-14  1.5e-12
Spatial mesh displacement
Iter      SolEst     Damping    Stepsize #Res #Jac #Sol   LinErr   LinRes
   1     9.6e-07   0.5000000     9.6e-07    4    2    2  1.6e-15  1.7e-15
Solution error estimates for segregated groups
1.3, 0.18, 0.48, 4.8e-07
Residual error estimates for segregated groups
8.1e+02, 1.2e+04, 2.2e+04, 0.17
Segregated solver iteration 3.
Merged variables
Iter      SolEst     Damping    Stepsize #Res #Jac #Sol   LinErr   LinRes
   1        0.75   0.3500000        0.75   10    7    7  7.8e-14    5e-15
   2        0.74   0.3500000        0.74   11    8    8  8.4e-14  3.5e-15
   3        0.72   0.3500000        0.72   12    9    9  7.7e-14  3.1e-15
Velocity u2, Pressure p
Iter      SolEst     Damping    Stepsize #Res #Jac #Sol   LinErr   LinRes
   1        0.15   0.5000000        0.15    6    3    3  4.3e-15  7.8e-15
Displacement field
Iter      SolEst     Damping    Stepsize #Res #Jac #Sol   LinErr   LinRes
   1        0.52   0.5000000        0.52    6    3    3  1.6e-13  1.5e-12
Spatial mesh displacement
Iter      SolEst     Damping    Stepsize #Res #Jac #Sol   LinErr   LinRes
   1     9.6e-07   0.5000000     9.6e-07    6    3    3    2e-15  1.7e-15
Solution error estimates for segregated groups
1.2, 0.076, 0.26, 4.8e-07
Residual error estimates for segregated groups
2.2e+02, 6.4e+03, 1.6e+04, 0.18
Segregated solver iteration 4.
```

```
Merged variables
Iter      SolEst     Damping    Stepsize #Res #Jac #Sol   LinErr   LinRes
   1         0.7   0.3500000         0.7   14   10   10  5.6e-14  3.5e-15
   2        0.68   0.3500000        0.68   15   11   11  6.3e-14    3e-15
   3         0.6   0.3500000         0.6   16   12   12  5.9e-14  3.6e-15
Velocity u2, Pressure p
Iter      SolEst     Damping    Stepsize #Res #Jac #Sol   LinErr   LinRes
   1       0.071   0.5000000       0.071    8    4    4  3.5e-15  7.7e-15
Displacement field
Iter      SolEst     Damping    Stepsize #Res #Jac #Sol   LinErr   LinRes
   1        0.29   0.5000000        0.29    8    4    4  1.2e-13  1.5e-12
Spatial mesh displacement
Iter      SolEst     Damping    Stepsize #Res #Jac #Sol   LinErr   LinRes
   1       8e-07   0.5000000       8e-07    8    4    4  1.5e-15  1.7e-15
Solution error estimates for segregated groups
1, 0.036, 0.15, 4e-07
Residual error estimates for segregated groups
82, 3.3e+03, 1.1e+04, 0.17
Segregated solver iteration 5.
Merged variables
Iter      SolEst     Damping    Stepsize #Res #Jac #Sol   LinErr   LinRes
   1        0.42   0.3500000        0.42   18   13   13  5.2e-14  3.1e-15
   2        0.28   0.3500000        0.28   19   14   14  6.2e-14  3.1e-15
   3        0.18   0.3500000        0.18   20   15   15  4.9e-14  2.7e-15
Velocity u2, Pressure p
Iter      SolEst     Damping    Stepsize #Res #Jac #Sol   LinErr   LinRes
   1       0.035   0.5000000       0.035   10    5    5  2.5e-15  7.8e-15
Displacement field
Iter      SolEst     Damping    Stepsize #Res #Jac #Sol   LinErr   LinRes
   1        0.17   0.5000000        0.17   10    5    5  8.4e-14  1.5e-12
Spatial mesh displacement
Iter      SolEst     Damping    Stepsize #Res #Jac #Sol   LinErr   LinRes
   1       6e-07   0.5000000       6e-07   10    5    5  1.5e-15  1.8e-15
Solution error estimates for segregated groups
0.32, 0.017, 0.084, 3e-07
Residual error estimates for segregated groups
32, 1.7e+03, 6.8e+03, 0.17
Segregated solver iteration 6.
Merged variables
Iter      SolEst     Damping    Stepsize #Res #Jac #Sol   LinErr   LinRes
   1        0.12   0.3500000        0.12   22   16   16  9.3e-14  3.7e-15
   2       0.079   0.3500000       0.079   23   17   17  6.4e-14  3.1e-15
   3       0.052   0.3500000       0.052   24   18   18  7.2e-14    3e-15
Velocity u2, Pressure p
Iter      SolEst     Damping    Stepsize #Res #Jac #Sol   LinErr   LinRes
   1       0.017   0.5000000       0.017   12    6    6  4.3e-15  7.9e-15
Displacement field
Iter      SolEst     Damping    Stepsize #Res #Jac #Sol   LinErr   LinRes
   1       0.095   0.5000000       0.095   12    6    6  4.7e-14  1.5e-12
Spatial mesh displacement
Iter      SolEst     Damping    Stepsize #Res #Jac #Sol   LinErr   LinRes
   1     4.2e-07   0.5000000     4.2e-07   12    6    6  1.7e-15  1.7e-15
Solution error estimates for segregated groups
0.089, 0.0085, 0.048, 2.1e-07
Residual error estimates for segregated groups
12, 8.6e+02, 4.2e+03, 0.18
```

```
Segregated solver iteration 7.
Merged variables
Iter      SolEst     Damping    Stepsize #Res #Jac #Sol   LinErr   LinRes
   1       0.034   0.3500000       0.034   26   19   19  6.9e-14  3.6e-15
   2       0.022   0.3500000       0.022   27   20   20  8.4e-14  3.4e-15
   3       0.014   0.3500000       0.014   28   21   21  4.7e-14    4e-15
Velocity u2, Pressure p
Iter      SolEst     Damping    Stepsize #Res #Jac #Sol   LinErr   LinRes
   1      0.0084   0.5000000      0.0084   14    7    7    3e-15  7.8e-15
Displacement field
Iter      SolEst     Damping    Stepsize #Res #Jac #Sol   LinErr   LinRes
   1       0.054   0.5000000       0.054   14    7    7  7.4e-14  1.5e-12
Spatial mesh displacement
Iter      SolEst     Damping    Stepsize #Res #Jac #Sol   LinErr   LinRes
   1     2.8e-07   0.5000000     2.8e-07   14    7    7  1.6e-15  1.7e-15
Solution error estimates for segregated groups
0.025, 0.0042, 0.027, 1.4e-07
Residual error estimates for segregated groups
4, 4.3e+02, 2.6e+03, 0.18
Segregated solver iteration 8.
Merged variables
Iter      SolEst     Damping    Stepsize #Res #Jac #Sol   LinErr   LinRes
   1      0.0093   0.3500000      0.0093   30   22   22  5.7e-14  4.4e-15
   2      0.0061   0.3500000      0.0061   31   23   23  7.2e-14  2.9e-15
   3      0.0039   0.3500000      0.0039   32   24   24  8.2e-14  3.5e-15
Velocity u2, Pressure p
Iter      SolEst     Damping    Stepsize #Res #Jac #Sol   LinErr   LinRes
   1      0.0042   0.5000000      0.0042   16    8    8  7.1e-15  7.8e-15
Displacement field
Iter      SolEst     Damping    Stepsize #Res #Jac #Sol   LinErr   LinRes
   1        0.03   0.5000000        0.03   16    8    8  7.6e-14  1.5e-12
Spatial mesh displacement
Iter      SolEst     Damping    Stepsize #Res #Jac #Sol   LinErr   LinRes
   1     1.8e-07   0.5000000     1.8e-07   16    8    8  1.5e-15  1.7e-15
Solution error estimates for segregated groups
0.0068, 0.0021, 0.015, 9e-08
Residual error estimates for segregated groups
1.3, 2.2e+02, 1.8e+03, 0.18
Segregated solver iteration 9.
Merged variables
Iter      SolEst     Damping    Stepsize #Res #Jac #Sol   LinErr   LinRes
   1      0.0026   0.3500000      0.0026   34   25   25  8.9e-14  3.4e-15
   2      0.0017   0.3500000      0.0017   35   26   26  9.2e-14  2.9e-15
   3      0.0011   0.3500000      0.0011   36   27   27  9.9e-14  3.5e-15
Velocity u2, Pressure p
Iter      SolEst     Damping    Stepsize #Res #Jac #Sol   LinErr   LinRes
   1      0.0021   0.5000000      0.0021   18    9    9  2.1e-15  7.9e-15
Displacement field
Iter      SolEst     Damping    Stepsize #Res #Jac #Sol   LinErr   LinRes
   1       0.017   0.5000000       0.017   18    9    9  5.2e-14  1.5e-12
Spatial mesh displacement
Iter      SolEst     Damping    Stepsize #Res #Jac #Sol   LinErr   LinRes
   1     1.1e-07   0.5000000     1.1e-07   18    9    9  1.6e-15  1.7e-15
Solution error estimates for segregated groups
0.0019, 0.001, 0.0085, 5.7e-08
Residual error estimates for segregated groups
```

```
0.43, 1.1e+02, 1.4e+03, 0.17
Segregated solver iteration 10.
Merged variables
Iter      SolEst     Damping    Stepsize #Res #Jac #Sol   LinErr   LinRes
   1      0.0007   0.3500000      0.0007   38   28   28    2e-13  2.9e-15
   2     0.00046   0.3500000     0.00046   39   29   29  7.5e-14  4.9e-15
   3      0.0003   0.3500000      0.0003   40   30   30  1.1e-13  2.8e-15
Velocity u2, Pressure p
Iter      SolEst     Damping    Stepsize #Res #Jac #Sol   LinErr   LinRes
   1       0.001   0.5000000       0.001   20   10   10  2.7e-15  7.9e-15
Displacement field
Iter      SolEst     Damping    Stepsize #Res #Jac #Sol   LinErr   LinRes
   1      0.0094   0.5000000      0.0094   20   10   10  5.3e-14  1.5e-12
Spatial mesh displacement
Iter      SolEst     Damping    Stepsize #Res #Jac #Sol   LinErr   LinRes
   1     6.9e-08   0.5000000     6.9e-08   20   10   10  1.4e-15  1.7e-15
Solution error estimates for segregated groups
0.00051, 0.00052, 0.0047, 3.5e-08
Residual error estimates for segregated groups
0.14, 55, 1.2e+03, 0.18
Segregated solver iteration 11.
Merged variables
Iter      SolEst     Damping    Stepsize #Res #Jac #Sol   LinErr   LinRes
   1     0.00019   0.3500000     0.00019   42   31   31  6.7e-14  3.3e-15
   2     0.00013   0.3500000     0.00013   43   32   32  6.9e-14  3.6e-15
   3     8.2e-05   0.3500000     8.2e-05   44   33   33  1.1e-13  2.8e-15
Velocity u2, Pressure p
Iter      SolEst     Damping    Stepsize #Res #Jac #Sol   LinErr   LinRes
   1     0.00052   0.5000000     0.00052   22   11   11  3.4e-15  7.7e-15
Displacement field
Iter      SolEst     Damping    Stepsize #Res #Jac #Sol   LinErr   LinRes
   1      0.0051   0.5000000      0.0051   22   11   11  2.6e-14  1.5e-12
Spatial mesh displacement
Iter      SolEst     Damping    Stepsize #Res #Jac #Sol   LinErr   LinRes
   1     4.1e-08   0.5000000     4.1e-08   22   11   11  1.8e-15  1.7e-15
Solution error estimates for segregated groups
0.00014, 0.00026, 0.0026, 2.1e-08
Residual error estimates for segregated groups
0.043, 27, 1.2e+03, 0.17
Segregated solver iteration 12.
Merged variables
Iter      SolEst     Damping    Stepsize #Res #Jac #Sol   LinErr   LinRes
   1     5.3e-05   0.3500000     5.3e-05   46   34   34  1.1e-13  3.5e-15
   2     3.4e-05   0.3500000     3.4e-05   47   35   35    1e-13    3e-15
   3     2.2e-05   0.3500000     2.2e-05   48   36   36  1.2e-13    3e-15
Velocity u2, Pressure p
Iter      SolEst     Damping    Stepsize #Res #Jac #Sol   LinErr   LinRes
   1     0.00026   0.5000000     0.00026   24   12   12  2.3e-15  7.8e-15
Displacement field
Iter      SolEst     Damping    Stepsize #Res #Jac #Sol   LinErr   LinRes
   1      0.0028   0.5000000      0.0028   24   12   12  8.9e-14  1.5e-12
Spatial mesh displacement
Iter      SolEst     Damping    Stepsize #Res #Jac #Sol   LinErr   LinRes
   1     2.4e-08   0.5000000     2.4e-08   24   12   12  1.5e-15  1.7e-15
Solution error estimates for segregated groups
3.8e-05, 0.00013, 0.0014, 1.2e-08
```

```
Residual error estimates for segregated groups
0.013, 14, 1.2e+03, 0.18
Segregated solver iteration 13.
Merged variables
Iter      SolEst     Damping    Stepsize #Res #Jac #Sol   LinErr   LinRes
   1     1.5e-05   0.3500000     1.5e-05   50   37   37  6.3e-14  2.7e-15
   2     9.5e-06   0.3500000     9.5e-06   51   38   38  8.9e-14    3e-15
   3     6.2e-06   0.3500000     6.2e-06   52   39   39  1.1e-13  3.4e-15
Velocity u2, Pressure p
Iter      SolEst     Damping    Stepsize #Res #Jac #Sol   LinErr   LinRes
   1     0.00013   0.5000000     0.00013   26   13   13  3.8e-15  7.7e-15
Displacement field
Iter      SolEst     Damping    Stepsize #Res #Jac #Sol   LinErr   LinRes
   1      0.0015   0.5000000      0.0015   26   13   13    1e-13  1.5e-12
Spatial mesh displacement
Iter      SolEst     Damping    Stepsize #Res #Jac #Sol   LinErr   LinRes
   1     1.4e-08   0.5000000     1.4e-08   26   13   13  1.7e-15  1.7e-15
Solution error estimates for segregated groups
1.1e-05, 6.5e-05, 0.00076, 7.1e-09
Residual error estimates for segregated groups
0.004, 6.8, 1.2e+03, 0.17
Solution time: 155 s. (2 minutes, 35 seconds)
Physical memory: 4.72 GB
Virtual memory: 25.81 GB
Ended at Jan 31, 2025, 6:58:21 PM.
----- Stationary Solver 1 in study1/Approach Curve Solver (sol1) -------------->
```

d=2270 (su48)

GENERAL

Description	Value
Solution	d=2270

LOG

```
<---- Compile Equations: Stationary in study1/Approach Curve Solver (sol1) -----
Started at Jan 31, 2025, 6:58:21 PM.
Parameter d = 2.2700000000000003E-6 (m).
Geometry shape function: Linear serendipity
Number of vertex elements: 18
Number of boundary elements: 6214
Number of elements: 156228
Minimum element quality: 0.4685
Time: 8 s.
Physical memory: 2.91 GB
Virtual memory: 15.24 GB
Ended at Jan 31, 2025, 6:58:30 PM.
----- Compile Equations: Stationary in study1/Approach Curve Solver (sol1) ---->

<---- Dependent Variables 1 in study1/Approach Curve Solver (sol1) -------------
Started at Jan 31, 2025, 6:58:30 PM.
Solution time: 0 s.
Physical memory: 2.91 GB
Virtual memory: 15.24 GB
Ended at Jan 31, 2025, 6:58:30 PM.
----- Dependent Variables 1 in study1/Approach Curve Solver (sol1) ------------>

<---- Stationary Solver 1 in study1/Approach Curve Solver (sol1) ---------------
Started at Jan 31, 2025, 6:58:30 PM.
Segregated solver
Segregated Step 11 does not have any components solved for.
Number of degrees of freedom solved for: 793912 (plus 11851 internal DOFs).
Segregated solver iteration 1.
Merged variables
Nonsymmetric matrix found.
Scales for dependent variables:
Concentration (comp1.Cl): 1.4e+02
Concentration (comp1.K): 1.4e+02
Electric potential (comp1.V): 1.9e+02
Orthonormal null-space function used.
Iter      SolEst     Damping    Stepsize #Res #Jac #Sol   LinErr   LinRes
   1        0.83   0.3500000        0.83    2    1    1  1.6e-11  2.8e-15
   2        0.81   0.3500000        0.81    3    2    2  8.3e-11  2.7e-15
   3         0.8   0.3500000         0.8    4    3    3  1.3e-11  2.9e-15
Velocity u2, Pressure p
Nonsymmetric matrix found.
Scales for dependent variables:
Pressure (comp1.p): 1
Velocity field (spatial frame) (comp1.u2): 0.00018
Orthonormal null-space function used.
Iter      SolEst     Damping    Stepsize #Res #Jac #Sol   LinErr   LinRes
   1         1.1   0.5000000         1.1    2    1    1  1.7e-15  2.3e-15
Displacement field
Nonsymmetric matrix found.
Scales for dependent variables:
Auxiliary pressure (comp1.solid.lemm1.pw): 1
Displacement field (comp1.u): 2.7e-18
Orthonormal null-space function used.
Iter      SolEst     Damping    Stepsize #Res #Jac #Sol   LinErr   LinRes
   1         1.9   0.5000000         1.9    2    1    1  1.6e-13  1.5e-12
Spatial mesh displacement
```

```
Symmetric matrices found.
Scales for dependent variables:
Spatial mesh displacement (comp1.spatial.disp): 1.2e-09
Orthonormal null-space function used.
Iter      SolEst     Damping    Stepsize #Res #Jac #Sol   LinErr   LinRes
   1     6.6e-07   0.5000000     6.6e-07    2    1    1  1.5e-15  1.9e-15
Solution error estimates for segregated groups
1.4, 0.54, 0.95, 3.3e-07
Residual error estimates for segregated groups
7.9e+03, 8.3e+04, 2.2e+04, 0.15
Segregated solver iteration 2.
Merged variables
Scales for dependent variables:
Concentration (comp1.Cl): 1.4e+02
Concentration (comp1.K): 1.4e+02
Electric potential (comp1.V): 0.064
Iter      SolEst     Damping    Stepsize #Res #Jac #Sol   LinErr   LinRes
   1        0.78   0.3500000        0.78    6    4    4  9.7e-14  3.2e-15
   2        0.77   0.3500000        0.77    7    5    5    1e-13    3e-15
   3        0.76   0.3500000        0.76    8    6    6  7.1e-14  3.3e-15
Velocity u2, Pressure p
Scales for dependent variables:
Pressure (comp1.p): 3.6e+02
Velocity field (spatial frame) (comp1.u2): 0.00072
Iter      SolEst     Damping    Stepsize #Res #Jac #Sol   LinErr   LinRes
   1        0.36   0.5000000        0.36    4    2    2  6.2e-15  7.9e-15
Displacement field
Iter      SolEst     Damping    Stepsize #Res #Jac #Sol   LinErr   LinRes
   1        0.95   0.5000000        0.95    4    2    2  7.8e-14  1.5e-12
Spatial mesh displacement
Iter      SolEst     Damping    Stepsize #Res #Jac #Sol   LinErr   LinRes
   1     9.8e-07   0.5000000     9.8e-07    4    2    2  1.7e-15  1.9e-15
Solution error estimates for segregated groups
1.3, 0.18, 0.48, 4.9e-07
Residual error estimates for segregated groups
7.9e+02, 1.4e+04, 2.2e+04, 0.16
Segregated solver iteration 3.
Merged variables
Iter      SolEst     Damping    Stepsize #Res #Jac #Sol   LinErr   LinRes
   1        0.75   0.3500000        0.75   10    7    7  8.6e-14  2.9e-15
   2        0.73   0.3500000        0.73   11    8    8  5.4e-14    3e-15
   3        0.72   0.3500000        0.72   12    9    9  5.4e-14  3.1e-15
Velocity u2, Pressure p
Iter      SolEst     Damping    Stepsize #Res #Jac #Sol   LinErr   LinRes
   1        0.15   0.5000000        0.15    6    3    3  4.9e-15  8.1e-15
Displacement field
Iter      SolEst     Damping    Stepsize #Res #Jac #Sol   LinErr   LinRes
   1        0.52   0.5000000        0.52    6    3    3    1e-13  1.5e-12
Spatial mesh displacement
Iter      SolEst     Damping    Stepsize #Res #Jac #Sol   LinErr   LinRes
   1     9.8e-07   0.5000000     9.8e-07    6    3    3  1.7e-15  1.9e-15
Solution error estimates for segregated groups
1.2, 0.077, 0.26, 4.9e-07
Residual error estimates for segregated groups
2.2e+02, 7.8e+03, 1.6e+04, 0.17
Segregated solver iteration 4.
```

```
Merged variables
Iter      SolEst     Damping    Stepsize #Res #Jac #Sol   LinErr   LinRes
   1         0.7   0.3500000         0.7   14   10   10  9.7e-14  3.3e-15
   2        0.68   0.3500000        0.68   15   11   11  9.4e-14  2.9e-15
   3         0.6   0.3500000         0.6   16   12   12  8.4e-14  2.9e-15
Velocity u2, Pressure p
Iter      SolEst     Damping    Stepsize #Res #Jac #Sol   LinErr   LinRes
   1       0.072   0.5000000       0.072    8    4    4    4e-15  7.9e-15
Displacement field
Iter      SolEst     Damping    Stepsize #Res #Jac #Sol   LinErr   LinRes
   1        0.29   0.5000000        0.29    8    4    4  5.3e-14  1.5e-12
Spatial mesh displacement
Iter      SolEst     Damping    Stepsize #Res #Jac #Sol   LinErr   LinRes
   1     8.2e-07   0.5000000     8.2e-07    8    4    4  1.5e-15  1.8e-15
Solution error estimates for segregated groups
1, 0.036, 0.15, 4.1e-07
Residual error estimates for segregated groups
84, 4.1e+03, 1.1e+04, 0.17
Segregated solver iteration 5.
Merged variables
Iter      SolEst     Damping    Stepsize #Res #Jac #Sol   LinErr   LinRes
   1        0.41   0.3500000        0.41   18   13   13  6.3e-14  3.7e-15
   2        0.28   0.3500000        0.28   19   14   14  7.1e-14  2.9e-15
   3        0.18   0.3500000        0.18   20   15   15  7.8e-14    3e-15
Velocity u2, Pressure p
Iter      SolEst     Damping    Stepsize #Res #Jac #Sol   LinErr   LinRes
   1       0.035   0.5000000       0.035   10    5    5  2.2e-15  7.8e-15
Displacement field
Iter      SolEst     Damping    Stepsize #Res #Jac #Sol   LinErr   LinRes
   1        0.17   0.5000000        0.17   10    5    5  3.5e-14  1.5e-12
Spatial mesh displacement
Iter      SolEst     Damping    Stepsize #Res #Jac #Sol   LinErr   LinRes
   1     6.2e-07   0.5000000     6.2e-07   10    5    5  1.4e-15  1.8e-15
Solution error estimates for segregated groups
0.31, 0.017, 0.084, 3.1e-07
Residual error estimates for segregated groups
33, 2.1e+03, 6.9e+03, 0.16
Segregated solver iteration 6.
Merged variables
Iter      SolEst     Damping    Stepsize #Res #Jac #Sol   LinErr   LinRes
   1        0.12   0.3500000        0.12   22   16   16  9.6e-14  3.2e-15
   2       0.078   0.3500000       0.078   23   17   17  8.1e-14  2.9e-15
   3       0.051   0.3500000       0.051   24   18   18  9.9e-14  3.1e-15
Velocity u2, Pressure p
Iter      SolEst     Damping    Stepsize #Res #Jac #Sol   LinErr   LinRes
   1       0.017   0.5000000       0.017   12    6    6  2.8e-15  7.8e-15
Displacement field
Iter      SolEst     Damping    Stepsize #Res #Jac #Sol   LinErr   LinRes
   1       0.095   0.5000000       0.095   12    6    6  3.8e-14  1.5e-12
Spatial mesh displacement
Iter      SolEst     Damping    Stepsize #Res #Jac #Sol   LinErr   LinRes
   1     4.3e-07   0.5000000     4.3e-07   12    6    6  1.8e-15  1.9e-15
Solution error estimates for segregated groups
0.088, 0.0085, 0.048, 2.2e-07
Residual error estimates for segregated groups
12, 1.1e+03, 4.2e+03, 0.16
```

```
Segregated solver iteration 7.
Merged variables
Iter      SolEst     Damping    Stepsize #Res #Jac #Sol   LinErr   LinRes
   1       0.033   0.3500000       0.033   26   19   19    8e-14  2.9e-15
   2       0.022   0.3500000       0.022   27   20   20  7.5e-14  3.7e-15
   3       0.014   0.3500000       0.014   28   21   21  8.6e-14    3e-15
Velocity u2, Pressure p
Iter      SolEst     Damping    Stepsize #Res #Jac #Sol   LinErr   LinRes
   1      0.0084   0.5000000      0.0084   14    7    7  3.6e-15  7.8e-15
Displacement field
Iter      SolEst     Damping    Stepsize #Res #Jac #Sol   LinErr   LinRes
   1       0.054   0.5000000       0.054   14    7    7  7.6e-14  1.5e-12
Spatial mesh displacement
Iter      SolEst     Damping    Stepsize #Res #Jac #Sol   LinErr   LinRes
   1     2.9e-07   0.5000000     2.9e-07   14    7    7    2e-15  1.9e-15
Solution error estimates for segregated groups
0.024, 0.0042, 0.027, 1.4e-07
Residual error estimates for segregated groups
4.1, 5.4e+02, 2.6e+03, 0.16
Segregated solver iteration 8.
Merged variables
Iter      SolEst     Damping    Stepsize #Res #Jac #Sol   LinErr   LinRes
   1      0.0092   0.3500000      0.0092   30   22   22    9e-14  2.9e-15
   2       0.006   0.3500000       0.006   31   23   23  4.6e-14  2.9e-15
   3      0.0039   0.3500000      0.0039   32   24   24  1.3e-13  2.9e-15
Velocity u2, Pressure p
Iter      SolEst     Damping    Stepsize #Res #Jac #Sol   LinErr   LinRes
   1      0.0042   0.5000000      0.0042   16    8    8  7.9e-15  7.8e-15
Displacement field
Iter      SolEst     Damping    Stepsize #Res #Jac #Sol   LinErr   LinRes
   1        0.03   0.5000000        0.03   16    8    8  4.3e-14  1.5e-12
Spatial mesh displacement
Iter      SolEst     Damping    Stepsize #Res #Jac #Sol   LinErr   LinRes
   1     1.8e-07   0.5000000     1.8e-07   16    8    8  1.3e-15  1.9e-15
Solution error estimates for segregated groups
0.0067, 0.0021, 0.015, 9.2e-08
Residual error estimates for segregated groups
1.4, 2.7e+02, 1.8e+03, 0.16
Segregated solver iteration 9.
Merged variables
Iter      SolEst     Damping    Stepsize #Res #Jac #Sol   LinErr   LinRes
   1      0.0025   0.3500000      0.0025   34   25   25    1e-13  2.8e-15
   2      0.0016   0.3500000      0.0016   35   26   26  8.1e-14  3.2e-15
   3      0.0011   0.3500000      0.0011   36   27   27  1.1e-13  2.9e-15
Velocity u2, Pressure p
Iter      SolEst     Damping    Stepsize #Res #Jac #Sol   LinErr   LinRes
   1      0.0021   0.5000000      0.0021   18    9    9  3.7e-15  7.8e-15
Displacement field
Iter      SolEst     Damping    Stepsize #Res #Jac #Sol   LinErr   LinRes
   1       0.017   0.5000000       0.017   18    9    9  4.9e-14  1.5e-12
Spatial mesh displacement
Iter      SolEst     Damping    Stepsize #Res #Jac #Sol   LinErr   LinRes
   1     1.2e-07   0.5000000     1.2e-07   18    9    9  1.9e-15  1.8e-15
Solution error estimates for segregated groups
0.0018, 0.001, 0.0085, 5.8e-08
Residual error estimates for segregated groups
```

```
0.44, 1.4e+02, 1.4e+03, 0.18
Segregated solver iteration 10.
Merged variables
Iter      SolEst     Damping    Stepsize #Res #Jac #Sol   LinErr   LinRes
   1     0.00069   0.3500000     0.00069   38   28   28  1.3e-13    3e-15
   2     0.00045   0.3500000     0.00045   39   29   29  1.3e-13  3.4e-15
   3     0.00029   0.3500000     0.00029   40   30   30  5.3e-14  2.9e-15
Velocity u2, Pressure p
Iter      SolEst     Damping    Stepsize #Res #Jac #Sol   LinErr   LinRes
   1       0.001   0.5000000       0.001   20   10   10  4.3e-15  7.8e-15
Displacement field
Iter      SolEst     Damping    Stepsize #Res #Jac #Sol   LinErr   LinRes
   1      0.0094   0.5000000      0.0094   20   10   10  4.7e-14  1.5e-12
Spatial mesh displacement
Iter      SolEst     Damping    Stepsize #Res #Jac #Sol   LinErr   LinRes
   1     7.1e-08   0.5000000     7.1e-08   20   10   10  1.4e-15  1.9e-15
Solution error estimates for segregated groups
0.0005, 0.00052, 0.0047, 3.5e-08
Residual error estimates for segregated groups
0.14, 68, 1.2e+03, 0.17
Segregated solver iteration 11.
Merged variables
Iter      SolEst     Damping    Stepsize #Res #Jac #Sol   LinErr   LinRes
   1     0.00019   0.3500000     0.00019   42   31   31  6.5e-14    3e-15
   2     0.00012   0.3500000     0.00012   43   32   32  7.4e-14    3e-15
   3     8.1e-05   0.3500000     8.1e-05   44   33   33    1e-13  3.4e-15
Velocity u2, Pressure p
Iter      SolEst     Damping    Stepsize #Res #Jac #Sol   LinErr   LinRes
   1     0.00052   0.5000000     0.00052   22   11   11    3e-15  7.9e-15
Displacement field
Iter      SolEst     Damping    Stepsize #Res #Jac #Sol   LinErr   LinRes
   1      0.0051   0.5000000      0.0051   22   11   11  6.7e-14  1.5e-12
Spatial mesh displacement
Iter      SolEst     Damping    Stepsize #Res #Jac #Sol   LinErr   LinRes
   1     4.2e-08   0.5000000     4.2e-08   22   11   11  1.4e-15  1.8e-15
Solution error estimates for segregated groups
0.00014, 0.00026, 0.0026, 2.1e-08
Residual error estimates for segregated groups
0.043, 34, 1.2e+03, 0.18
Segregated solver iteration 12.
Merged variables
Iter      SolEst     Damping    Stepsize #Res #Jac #Sol   LinErr   LinRes
   1     5.2e-05   0.3500000     5.2e-05   46   34   34  7.4e-14    3e-15
   2     3.4e-05   0.3500000     3.4e-05   47   35   35  6.2e-14    3e-15
   3     2.2e-05   0.3500000     2.2e-05   48   36   36  6.6e-14  3.4e-15
Velocity u2, Pressure p
Iter      SolEst     Damping    Stepsize #Res #Jac #Sol   LinErr   LinRes
   1     0.00026   0.5000000     0.00026   24   12   12  2.6e-15  7.9e-15
Displacement field
Iter      SolEst     Damping    Stepsize #Res #Jac #Sol   LinErr   LinRes
   1      0.0028   0.5000000      0.0028   24   12   12  3.3e-14  1.5e-12
Spatial mesh displacement
Iter      SolEst     Damping    Stepsize #Res #Jac #Sol   LinErr   LinRes
   1     2.5e-08   0.5000000     2.5e-08   24   12   12  1.8e-15  1.9e-15
Solution error estimates for segregated groups
3.8e-05, 0.00013, 0.0014, 1.2e-08
```

```
Residual error estimates for segregated groups
0.013, 17, 1.2e+03, 0.17
Segregated solver iteration 13.
Merged variables
Iter      SolEst      Damping    Stepsize #Res #Jac #Sol   LinErr   LinRes
   1      1.4e-05   0.3500000     1.4e-05   50   37   37  1.3e-13    3e-15
   2      9.3e-06   0.3500000     9.3e-06   51   38   38  8.3e-14    3e-15
   3      6.1e-06   0.3500000     6.1e-06   52   39   39  9.4e-14  3.3e-15
Velocity u2, Pressure p
Iter      SolEst      Damping    Stepsize #Res #Jac #Sol   LinErr   LinRes
   1      0.00013   0.5000000     0.00013   26   13   13  3.2e-15  7.9e-15
Displacement field
Iter      SolEst      Damping    Stepsize #Res #Jac #Sol   LinErr   LinRes
   1       0.0015   0.5000000      0.0015   26   13   13  3.9e-14  1.5e-12
Spatial mesh displacement
Iter      SolEst      Damping    Stepsize #Res #Jac #Sol   LinErr   LinRes
   1      1.5e-08   0.5000000     1.5e-08   26   13   13  1.7e-15  1.8e-15
Solution error estimates for segregated groups
1e-05, 6.5e-05, 0.00076, 7.3e-09
Residual error estimates for segregated groups
0.004, 8.5, 1.2e+03, 0.17
Solution time: 156 s. (2 minutes, 36 seconds)
Physical memory: 4.66 GB
Virtual memory: 25.51 GB
Ended at Jan 31, 2025, 7:01:06 PM.
----- Stationary Solver 1 in study1/Approach Curve Solver (sol1) -------------->
```

d=2265 (su49)

GENERAL

Description	Value
Solution	d=2265

LOG

```
<---- Compile Equations: Stationary in study1/Approach Curve Solver (sol1) -----
Started at Jan 31, 2025, 7:01:06 PM.
Parameter d = 2.2650000000000003E-6 (m).
Geometry shape function: Linear serendipity
Number of vertex elements: 18
Number of boundary elements: 6212
Number of elements: 155820
Minimum element quality: 0.4731
Time: 8 s.
Physical memory: 2.92 GB
Virtual memory: 15.24 GB
Ended at Jan 31, 2025, 7:01:14 PM.
----- Compile Equations: Stationary in study1/Approach Curve Solver (sol1) ---->

<---- Dependent Variables 1 in study1/Approach Curve Solver (sol1) -------------
Started at Jan 31, 2025, 7:01:14 PM.
Solution time: 0 s.
Physical memory: 2.91 GB
Virtual memory: 15.18 GB
Ended at Jan 31, 2025, 7:01:14 PM.
----- Dependent Variables 1 in study1/Approach Curve Solver (sol1) ------------>

<---- Stationary Solver 1 in study1/Approach Curve Solver (sol1) ---------------
Started at Jan 31, 2025, 7:01:15 PM.
Segregated solver
Segregated Step 11 does not have any components solved for.
Number of degrees of freedom solved for: 791981 (plus 11847 internal DOFs).
Segregated solver iteration 1.
Merged variables
Nonsymmetric matrix found.
Scales for dependent variables:
Concentration (comp1.Cl): 1.4e+02
Concentration (comp1.K): 1.4e+02
Electric potential (comp1.V): 1.9e+02
Orthonormal null-space function used.
Iter      SolEst     Damping    Stepsize #Res #Jac #Sol   LinErr   LinRes
   1        0.83   0.3500000        0.83    2    1    1  1.6e-11  2.9e-15
   2        0.81   0.3500000        0.81    3    2    2  1.8e-11  2.7e-15
   3         0.8   0.3500000         0.8    4    3    3  1.7e-11  2.9e-15
Velocity u2, Pressure p
Nonsymmetric matrix found.
Scales for dependent variables:
Pressure (comp1.p): 1
Velocity field (spatial frame) (comp1.u2): 0.00018
Orthonormal null-space function used.
Iter      SolEst     Damping    Stepsize #Res #Jac #Sol   LinErr   LinRes
   1         1.1   0.5000000         1.1    2    1    1  1.6e-15  2.3e-15
Displacement field
Nonsymmetric matrix found.
Scales for dependent variables:
Auxiliary pressure (comp1.solid.lemm1.pw): 1
Displacement field (comp1.u): 2.8e-18
Orthonormal null-space function used.
Iter      SolEst     Damping    Stepsize #Res #Jac #Sol   LinErr   LinRes
   1         1.9   0.5000000         1.9    2    1    1  1.2e-13  1.5e-12
Spatial mesh displacement
```

```
Symmetric matrices found.
Scales for dependent variables:
Spatial mesh displacement (comp1.spatial.disp): 1.2e-09
Orthonormal null-space function used.
Iter      SolEst     Damping    Stepsize #Res #Jac #Sol   LinErr   LinRes
   1     6.6e-07   0.5000000     6.6e-07    2    1    1  1.5e-15  1.9e-15
Solution error estimates for segregated groups
1.4, 0.54, 0.95, 3.3e-07
Residual error estimates for segregated groups
7.9e+03, 8.3e+04, 2.2e+04, 0.15
Segregated solver iteration 2.
Merged variables
Scales for dependent variables:
Concentration (comp1.Cl): 1.4e+02
Concentration (comp1.K): 1.4e+02
Electric potential (comp1.V): 0.064
Iter      SolEst     Damping    Stepsize #Res #Jac #Sol   LinErr   LinRes
   1        0.78   0.3500000        0.78    6    4    4  4.2e-14  3.2e-15
   2        0.77   0.3500000        0.77    7    5    5  7.3e-14  2.9e-15
   3        0.76   0.3500000        0.76    8    6    6  7.1e-14  3.1e-15
Velocity u2, Pressure p
Scales for dependent variables:
Pressure (comp1.p): 3.6e+02
Velocity field (spatial frame) (comp1.u2): 0.00072
Iter      SolEst     Damping    Stepsize #Res #Jac #Sol   LinErr   LinRes
   1        0.36   0.5000000        0.36    4    2    2    3e-15    8e-15
Displacement field
Iter      SolEst     Damping    Stepsize #Res #Jac #Sol   LinErr   LinRes
   1        0.95   0.5000000        0.95    4    2    2    1e-13  1.5e-12
Spatial mesh displacement
Iter      SolEst     Damping    Stepsize #Res #Jac #Sol   LinErr   LinRes
   1     9.9e-07   0.5000000     9.9e-07    4    2    2  1.5e-15  1.9e-15
Solution error estimates for segregated groups
1.3, 0.18, 0.48, 4.9e-07
Residual error estimates for segregated groups
7.9e+02, 1.4e+04, 2.2e+04, 0.17
Segregated solver iteration 3.
Merged variables
Iter      SolEst     Damping    Stepsize #Res #Jac #Sol   LinErr   LinRes
   1        0.75   0.3500000        0.75   10    7    7  7.1e-14  2.9e-15
   2        0.73   0.3500000        0.73   11    8    8    7e-14  3.7e-15
   3        0.72   0.3500000        0.72   12    9    9  4.5e-14    3e-15
Velocity u2, Pressure p
Iter      SolEst     Damping    Stepsize #Res #Jac #Sol   LinErr   LinRes
   1        0.15   0.5000000        0.15    6    3    3  2.4e-15    8e-15
Displacement field
Iter      SolEst     Damping    Stepsize #Res #Jac #Sol   LinErr   LinRes
   1        0.52   0.5000000        0.52    6    3    3  7.4e-14  1.5e-12
Spatial mesh displacement
Iter      SolEst     Damping    Stepsize #Res #Jac #Sol   LinErr   LinRes
   1     9.9e-07   0.5000000     9.9e-07    6    3    3  1.6e-15  1.8e-15
Solution error estimates for segregated groups
1.2, 0.077, 0.26, 4.9e-07
Residual error estimates for segregated groups
2.2e+02, 7.6e+03, 1.6e+04, 0.16
Segregated solver iteration 4.
```

```
Merged variables
Iter      SolEst     Damping    Stepsize #Res #Jac #Sol   LinErr   LinRes
   1         0.7   0.3500000         0.7   14   10   10  8.5e-14  3.1e-15
   2        0.68   0.3500000        0.68   15   11   11  9.5e-14  2.7e-15
   3         0.6   0.3500000         0.6   16   12   12  7.1e-14  2.8e-15
Velocity u2, Pressure p
Iter      SolEst     Damping    Stepsize #Res #Jac #Sol   LinErr   LinRes
   1       0.072   0.5000000       0.072    8    4    4  6.7e-15    8e-15
Displacement field
Iter      SolEst     Damping    Stepsize #Res #Jac #Sol   LinErr   LinRes
   1        0.29   0.5000000        0.29    8    4    4  3.8e-14  1.5e-12
Spatial mesh displacement
Iter      SolEst     Damping    Stepsize #Res #Jac #Sol   LinErr   LinRes
   1     8.2e-07   0.5000000     8.2e-07    8    4    4  1.6e-15  1.8e-15
Solution error estimates for segregated groups
1, 0.036, 0.15, 4.1e-07
Residual error estimates for segregated groups
84, 4e+03, 1.1e+04, 0.17
Segregated solver iteration 5.
Merged variables
Iter      SolEst     Damping    Stepsize #Res #Jac #Sol   LinErr   LinRes
   1        0.41   0.3500000        0.41   18   13   13  6.8e-14  2.8e-15
   2        0.28   0.3500000        0.28   19   14   14  9.5e-14  2.7e-15
   3        0.18   0.3500000        0.18   20   15   15  6.3e-14  3.2e-15
Velocity u2, Pressure p
Iter      SolEst     Damping    Stepsize #Res #Jac #Sol   LinErr   LinRes
   1       0.035   0.5000000       0.035   10    5    5  3.9e-15  7.9e-15
Displacement field
Iter      SolEst     Damping    Stepsize #Res #Jac #Sol   LinErr   LinRes
   1        0.17   0.5000000        0.17   10    5    5    1e-13  1.5e-12
Spatial mesh displacement
Iter      SolEst     Damping    Stepsize #Res #Jac #Sol   LinErr   LinRes
   1     6.2e-07   0.5000000     6.2e-07   10    5    5  1.7e-15  1.9e-15
Solution error estimates for segregated groups
0.31, 0.017, 0.084, 3.1e-07
Residual error estimates for segregated groups
33, 2.1e+03, 6.9e+03, 0.16
Segregated solver iteration 6.
Merged variables
Iter      SolEst     Damping    Stepsize #Res #Jac #Sol   LinErr   LinRes
   1        0.12   0.3500000        0.12   22   16   16  6.1e-14  2.8e-15
   2       0.078   0.3500000       0.078   23   17   17  1.1e-13  2.9e-15
   3       0.051   0.3500000       0.051   24   18   18  6.8e-14  2.7e-15
Velocity u2, Pressure p
Iter      SolEst     Damping    Stepsize #Res #Jac #Sol   LinErr   LinRes
   1       0.017   0.5000000       0.017   12    6    6  3.6e-15    8e-15
Displacement field
Iter      SolEst     Damping    Stepsize #Res #Jac #Sol   LinErr   LinRes
   1       0.095   0.5000000       0.095   12    6    6  1.9e-13  1.5e-12
Spatial mesh displacement
Iter      SolEst     Damping    Stepsize #Res #Jac #Sol   LinErr   LinRes
   1     4.3e-07   0.5000000     4.3e-07   12    6    6  1.6e-15  1.8e-15
Solution error estimates for segregated groups
0.088, 0.0085, 0.048, 2.2e-07
Residual error estimates for segregated groups
12, 1e+03, 4.2e+03, 0.16
```

```
Segregated solver iteration 7.
Merged variables
Iter      SolEst     Damping    Stepsize #Res #Jac #Sol   LinErr   LinRes
   1       0.033   0.3500000       0.033   26   19   19  5.8e-14  2.9e-15
   2       0.022   0.3500000       0.022   27   20   20  6.5e-14  2.7e-15
   3       0.014   0.3500000       0.014   28   21   21  5.5e-14    3e-15
Velocity u2, Pressure p
Iter      SolEst     Damping    Stepsize #Res #Jac #Sol   LinErr   LinRes
   1      0.0085   0.5000000      0.0085   14    7    7  2.7e-15  7.9e-15
Displacement field
Iter      SolEst     Damping    Stepsize #Res #Jac #Sol   LinErr   LinRes
   1       0.054   0.5000000       0.054   14    7    7  5.8e-14  1.5e-12
Spatial mesh displacement
Iter      SolEst     Damping    Stepsize #Res #Jac #Sol   LinErr   LinRes
   1     2.9e-07   0.5000000     2.9e-07   14    7    7  1.8e-15  1.9e-15
Solution error estimates for segregated groups
0.024, 0.0042, 0.027, 1.4e-07
Residual error estimates for segregated groups
4.1, 5.2e+02, 2.6e+03, 0.17
Segregated solver iteration 8.
Merged variables
Iter      SolEst     Damping    Stepsize #Res #Jac #Sol   LinErr   LinRes
   1      0.0092   0.3500000      0.0092   30   22   22  5.4e-14  2.9e-15
   2       0.006   0.3500000       0.006   31   23   23  1.1e-13  2.8e-15
   3      0.0039   0.3500000      0.0039   32   24   24  4.3e-14  2.8e-15
Velocity u2, Pressure p
Iter      SolEst     Damping    Stepsize #Res #Jac #Sol   LinErr   LinRes
   1      0.0042   0.5000000      0.0042   16    8    8  6.6e-15    8e-15
Displacement field
Iter      SolEst     Damping    Stepsize #Res #Jac #Sol   LinErr   LinRes
   1        0.03   0.5000000        0.03   16    8    8  3.3e-14  1.5e-12
Spatial mesh displacement
Iter      SolEst     Damping    Stepsize #Res #Jac #Sol   LinErr   LinRes
   1     1.9e-07   0.5000000     1.9e-07   16    8    8  2.1e-15  1.8e-15
Solution error estimates for segregated groups
0.0067, 0.0021, 0.015, 9.3e-08
Residual error estimates for segregated groups
1.4, 2.6e+02, 1.8e+03, 0.17
Segregated solver iteration 9.
Merged variables
Iter      SolEst     Damping    Stepsize #Res #Jac #Sol   LinErr   LinRes
   1      0.0025   0.3500000      0.0025   34   25   25  3.8e-14  2.7e-15
   2      0.0016   0.3500000      0.0016   35   26   26  6.7e-14  2.8e-15
   3      0.0011   0.3500000      0.0011   36   27   27    8e-14    3e-15
Velocity u2, Pressure p
Iter      SolEst     Damping    Stepsize #Res #Jac #Sol   LinErr   LinRes
   1      0.0021   0.5000000      0.0021   18    9    9  2.4e-15  7.9e-15
Displacement field
Iter      SolEst     Damping    Stepsize #Res #Jac #Sol   LinErr   LinRes
   1       0.017   0.5000000       0.017   18    9    9  6.6e-14  1.5e-12
Spatial mesh displacement
Iter      SolEst     Damping    Stepsize #Res #Jac #Sol   LinErr   LinRes
   1     1.2e-07   0.5000000     1.2e-07   18    9    9  1.8e-15  1.8e-15
Solution error estimates for segregated groups
0.0018, 0.0011, 0.0085, 5.8e-08
Residual error estimates for segregated groups
```

```
0.44, 1.3e+02, 1.4e+03, 0.17
Segregated solver iteration 10.
Merged variables
Iter      SolEst     Damping    Stepsize #Res #Jac #Sol   LinErr   LinRes
   1     0.00069   0.3500000     0.00069   38   28   28  4.6e-14  2.8e-15
   2     0.00045   0.3500000     0.00045   39   29   29  5.7e-14  2.9e-15
   3     0.00029   0.3500000     0.00029   40   30   30  6.2e-14  2.9e-15
Velocity u2, Pressure p
Iter      SolEst     Damping    Stepsize #Res #Jac #Sol   LinErr   LinRes
   1       0.001   0.5000000       0.001   20   10   10  3.7e-15    8e-15
Displacement field
Iter      SolEst     Damping    Stepsize #Res #Jac #Sol   LinErr   LinRes
   1      0.0094   0.5000000      0.0094   20   10   10  1.2e-13  1.5e-12
Spatial mesh displacement
Iter      SolEst     Damping    Stepsize #Res #Jac #Sol   LinErr   LinRes
   1     7.1e-08   0.5000000     7.1e-08   20   10   10  1.6e-15  1.9e-15
Solution error estimates for segregated groups
0.0005, 0.00052, 0.0047, 3.5e-08
Residual error estimates for segregated groups
0.14, 66, 1.2e+03, 0.16
Segregated solver iteration 11.
Merged variables
Iter      SolEst     Damping    Stepsize #Res #Jac #Sol   LinErr   LinRes
   1     0.00019   0.3500000     0.00019   42   31   31  7.4e-14  2.7e-15
   2     0.00012   0.3500000     0.00012   43   32   32  5.4e-14  2.8e-15
   3       8e-05   0.3500000       8e-05   44   33   33  8.6e-14  2.7e-15
Velocity u2, Pressure p
Iter      SolEst     Damping    Stepsize #Res #Jac #Sol   LinErr   LinRes
   1     0.00052   0.5000000     0.00052   22   11   11  3.6e-15    8e-15
Displacement field
Iter      SolEst     Damping    Stepsize #Res #Jac #Sol   LinErr   LinRes
   1      0.0051   0.5000000      0.0051   22   11   11    1e-13  1.5e-12
Spatial mesh displacement
Iter      SolEst     Damping    Stepsize #Res #Jac #Sol   LinErr   LinRes
   1     4.2e-08   0.5000000     4.2e-08   22   11   11  1.6e-15  1.8e-15
Solution error estimates for segregated groups
0.00014, 0.00026, 0.0026, 2.1e-08
Residual error estimates for segregated groups
0.043, 33, 1.2e+03, 0.18
Segregated solver iteration 12.
Merged variables
Iter      SolEst     Damping    Stepsize #Res #Jac #Sol   LinErr   LinRes
   1     5.2e-05   0.3500000     5.2e-05   46   34   34  7.4e-14    3e-15
   2     3.4e-05   0.3500000     3.4e-05   47   35   35  8.9e-14    3e-15
   3     2.2e-05   0.3500000     2.2e-05   48   36   36  5.1e-14  2.9e-15
Velocity u2, Pressure p
Iter      SolEst     Damping    Stepsize #Res #Jac #Sol   LinErr   LinRes
   1     0.00026   0.5000000     0.00026   24   12   12  3.7e-15  7.9e-15
Displacement field
Iter      SolEst     Damping    Stepsize #Res #Jac #Sol   LinErr   LinRes
   1      0.0028   0.5000000      0.0028   24   12   12  8.1e-14  1.5e-12
Spatial mesh displacement
Iter      SolEst     Damping    Stepsize #Res #Jac #Sol   LinErr   LinRes
   1     2.5e-08   0.5000000     2.5e-08   24   12   12  1.7e-15  1.8e-15
Solution error estimates for segregated groups
3.8e-05, 0.00013, 0.0014, 1.3e-08
```

```
Residual error estimates for segregated groups
0.013, 16, 1.2e+03, 0.19
Segregated solver iteration 13.
Merged variables
Iter      SolEst      Damping    Stepsize #Res #Jac #Sol   LinErr   LinRes
   1      1.4e-05   0.3500000     1.4e-05   50   37   37  9.6e-14  2.9e-15
   2      9.3e-06   0.3500000     9.3e-06   51   38   38  5.3e-14  2.8e-15
   3      6.1e-06   0.3500000     6.1e-06   52   39   39  5.6e-14  2.8e-15
Velocity u2, Pressure p
Iter      SolEst      Damping    Stepsize #Res #Jac #Sol   LinErr   LinRes
   1      0.00013   0.5000000     0.00013   26   13   13  2.9e-15    8e-15
Displacement field
Iter      SolEst      Damping    Stepsize #Res #Jac #Sol   LinErr   LinRes
   1       0.0015   0.5000000      0.0015   26   13   13    7e-14  1.5e-12
Spatial mesh displacement
Iter      SolEst      Damping    Stepsize #Res #Jac #Sol   LinErr   LinRes
   1      1.5e-08   0.5000000     1.5e-08   26   13   13  1.6e-15  1.8e-15
Solution error estimates for segregated groups
1e-05, 6.6e-05, 0.00076, 7.3e-09
Residual error estimates for segregated groups
0.004, 8.2, 1.2e+03, 0.17
Solution time: 158 s. (2 minutes, 38 seconds)
Physical memory: 4.76 GB
Virtual memory: 25.58 GB
Ended at Jan 31, 2025, 7:03:53 PM.
----- Stationary Solver 1 in study1/Approach Curve Solver (sol1) -------------->
```

d=2260 (su50)

GENERAL

Description	Value
Solution	d=2260

LOG

```
<---- Compile Equations: Stationary in study1/Approach Curve Solver (sol1) -----
Started at Jan 31, 2025, 7:03:53 PM.
Parameter d = 2.26E-6 (m).
Geometry shape function: Linear serendipity
Number of vertex elements: 18
Number of boundary elements: 6209
Number of elements: 155921
Minimum element quality: 0.4818
Time: 8 s.
Physical memory: 2.95 GB
Virtual memory: 15.21 GB
Ended at Jan 31, 2025, 7:04:01 PM.
----- Compile Equations: Stationary in study1/Approach Curve Solver (sol1) ---->

<---- Dependent Variables 1 in study1/Approach Curve Solver (sol1) -------------
Started at Jan 31, 2025, 7:04:01 PM.
Solution time: 0 s.
Physical memory: 2.95 GB
Virtual memory: 15.21 GB
Ended at Jan 31, 2025, 7:04:01 PM.
----- Dependent Variables 1 in study1/Approach Curve Solver (sol1) ------------>

<---- Stationary Solver 1 in study1/Approach Curve Solver (sol1) ---------------
Started at Jan 31, 2025, 7:04:02 PM.
Segregated solver
Segregated Step 11 does not have any components solved for.
Number of degrees of freedom solved for: 792603 (plus 11841 internal DOFs).
Segregated solver iteration 1.
Merged variables
Nonsymmetric matrix found.
Scales for dependent variables:
Concentration (comp1.Cl): 1.4e+02
Concentration (comp1.K): 1.4e+02
Electric potential (comp1.V): 1.9e+02
Orthonormal null-space function used.
Iter      SolEst     Damping    Stepsize #Res #Jac #Sol   LinErr   LinRes
   1        0.83   0.3500000        0.83    2    1    1  2.9e-11  2.8e-15
   2        0.81   0.3500000        0.81    3    2    2  4.9e-11  2.8e-15
   3         0.8   0.3500000         0.8    4    3    3  2.9e-11  2.7e-15
Velocity u2, Pressure p
Nonsymmetric matrix found.
Scales for dependent variables:
Pressure (comp1.p): 1
Velocity field (spatial frame) (comp1.u2): 0.00018
Orthonormal null-space function used.
Iter      SolEst     Damping    Stepsize #Res #Jac #Sol   LinErr   LinRes
   1         1.1   0.5000000         1.1    2    1    1  1.6e-15  2.3e-15
Displacement field
Nonsymmetric matrix found.
Scales for dependent variables:
Auxiliary pressure (comp1.solid.lemm1.pw): 1
Displacement field (comp1.u): 2.8e-18
Orthonormal null-space function used.
Iter      SolEst     Damping    Stepsize #Res #Jac #Sol   LinErr   LinRes
   1         1.9   0.5000000         1.9    2    1    1  2.9e-14  1.5e-12
Spatial mesh displacement
```

```
Symmetric matrices found.
Scales for dependent variables:
Spatial mesh displacement (comp1.spatial.disp): 1.2e-09
Orthonormal null-space function used.
Iter      SolEst     Damping    Stepsize #Res #Jac #Sol   LinErr   LinRes
   1     6.6e-07   0.5000000     6.6e-07    2    1    1  1.4e-15  1.8e-15
Solution error estimates for segregated groups
1.4, 0.54, 0.95, 3.3e-07
Residual error estimates for segregated groups
7.9e+03, 8.3e+04, 2.2e+04, 0.15
Segregated solver iteration 2.
Merged variables
Scales for dependent variables:
Concentration (comp1.Cl): 1.4e+02
Concentration (comp1.K): 1.4e+02
Electric potential (comp1.V): 0.064
Iter      SolEst     Damping    Stepsize #Res #Jac #Sol   LinErr   LinRes
   1        0.78   0.3500000        0.78    6    4    4  8.8e-14    3e-15
   2        0.77   0.3500000        0.77    7    5    5  1.4e-13  3.2e-15
   3        0.76   0.3500000        0.76    8    6    6  5.4e-14  4.1e-15
Velocity u2, Pressure p
Scales for dependent variables:
Pressure (comp1.p): 3.6e+02
Velocity field (spatial frame) (comp1.u2): 0.00072
Iter      SolEst     Damping    Stepsize #Res #Jac #Sol   LinErr   LinRes
   1        0.36   0.5000000        0.36    4    2    2  3.3e-15  7.8e-15
Displacement field
Iter      SolEst     Damping    Stepsize #Res #Jac #Sol   LinErr   LinRes
   1        0.95   0.5000000        0.95    4    2    2  5.8e-14  1.5e-12
Spatial mesh displacement
Iter      SolEst     Damping    Stepsize #Res #Jac #Sol   LinErr   LinRes
   1     9.9e-07   0.5000000     9.9e-07    4    2    2  1.3e-15  1.9e-15
Solution error estimates for segregated groups
1.3, 0.18, 0.48, 5e-07
Residual error estimates for segregated groups
7.9e+02, 1.4e+04, 2.2e+04, 0.15
Segregated solver iteration 3.
Merged variables
Iter      SolEst     Damping    Stepsize #Res #Jac #Sol   LinErr   LinRes
   1        0.75   0.3500000        0.75   10    7    7  1.2e-13  5.9e-15
   2        0.73   0.3500000        0.73   11    8    8  1.9e-13  3.3e-15
   3        0.72   0.3500000        0.72   12    9    9    1e-13  4.1e-15
Velocity u2, Pressure p
Iter      SolEst     Damping    Stepsize #Res #Jac #Sol   LinErr   LinRes
   1        0.15   0.5000000        0.15    6    3    3  2.6e-15  7.8e-15
Displacement field
Iter      SolEst     Damping    Stepsize #Res #Jac #Sol   LinErr   LinRes
   1        0.52   0.5000000        0.52    6    3    3  4.6e-14  1.5e-12
Spatial mesh displacement
Iter      SolEst     Damping    Stepsize #Res #Jac #Sol   LinErr   LinRes
   1     9.9e-07   0.5000000     9.9e-07    6    3    3  1.7e-15  1.8e-15
Solution error estimates for segregated groups
1.2, 0.077, 0.26, 5e-07
Residual error estimates for segregated groups
2.2e+02, 7.7e+03, 1.6e+04, 0.17
Segregated solver iteration 4.
```

```
Merged variables
Iter      SolEst     Damping    Stepsize #Res #Jac #Sol   LinErr   LinRes
   1         0.7   0.3500000         0.7   14   10   10  1.6e-13  4.3e-15
   2        0.68   0.3500000        0.68   15   11   11  1.9e-13  5.3e-15
   3         0.6   0.3500000         0.6   16   12   12  1.1e-13    4e-15
Velocity u2, Pressure p
Iter      SolEst     Damping    Stepsize #Res #Jac #Sol   LinErr   LinRes
   1       0.072   0.5000000       0.072    8    4    4  2.8e-15    8e-15
Displacement field
Iter      SolEst     Damping    Stepsize #Res #Jac #Sol   LinErr   LinRes
   1        0.29   0.5000000        0.29    8    4    4  1.4e-13  1.5e-12
Spatial mesh displacement
Iter      SolEst     Damping    Stepsize #Res #Jac #Sol   LinErr   LinRes
   1     8.3e-07   0.5000000     8.3e-07    8    4    4  1.6e-15  1.8e-15
Solution error estimates for segregated groups
1, 0.036, 0.15, 4.1e-07
Residual error estimates for segregated groups
84, 4e+03, 1.1e+04, 0.17
Segregated solver iteration 5.
Merged variables
Iter      SolEst     Damping    Stepsize #Res #Jac #Sol   LinErr   LinRes
   1        0.41   0.3500000        0.41   18   13   13  7.1e-14  3.3e-15
   2        0.28   0.3500000        0.28   19   14   14    1e-13  3.5e-15
   3        0.18   0.3500000        0.18   20   15   15  1.1e-13  3.6e-15
Velocity u2, Pressure p
Iter      SolEst     Damping    Stepsize #Res #Jac #Sol   LinErr   LinRes
   1       0.035   0.5000000       0.035   10    5    5  2.6e-15  7.8e-15
Displacement field
Iter      SolEst     Damping    Stepsize #Res #Jac #Sol   LinErr   LinRes
   1        0.17   0.5000000        0.17   10    5    5  4.3e-14  1.5e-12
Spatial mesh displacement
Iter      SolEst     Damping    Stepsize #Res #Jac #Sol   LinErr   LinRes
   1     6.2e-07   0.5000000     6.2e-07   10    5    5  2.3e-15  1.9e-15
Solution error estimates for segregated groups
0.31, 0.017, 0.084, 3.1e-07
Residual error estimates for segregated groups
33, 2.1e+03, 6.8e+03, 0.17
Segregated solver iteration 6.
Merged variables
Iter      SolEst     Damping    Stepsize #Res #Jac #Sol   LinErr   LinRes
   1        0.12   0.3500000        0.12   22   16   16  1.8e-13  4.5e-15
   2       0.078   0.3500000       0.078   23   17   17  9.7e-14  6.3e-15
   3       0.051   0.3500000       0.051   24   18   18  7.7e-14  6.5e-15
Velocity u2, Pressure p
Iter      SolEst     Damping    Stepsize #Res #Jac #Sol   LinErr   LinRes
   1       0.017   0.5000000       0.017   12    6    6  3.6e-15  7.9e-15
Displacement field
Iter      SolEst     Damping    Stepsize #Res #Jac #Sol   LinErr   LinRes
   1       0.095   0.5000000       0.095   12    6    6    5e-14  1.5e-12
Spatial mesh displacement
Iter      SolEst     Damping    Stepsize #Res #Jac #Sol   LinErr   LinRes
   1     4.3e-07   0.5000000     4.3e-07   12    6    6  1.6e-15  1.8e-15
Solution error estimates for segregated groups
0.088, 0.0085, 0.048, 2.2e-07
Residual error estimates for segregated groups
12, 1e+03, 4.2e+03, 0.17
```

```
Segregated solver iteration 7.
Merged variables
Iter      SolEst     Damping    Stepsize #Res #Jac #Sol   LinErr   LinRes
   1       0.033   0.3500000       0.033   26   19   19  1.8e-13  3.7e-15
   2       0.022   0.3500000       0.022   27   20   20    1e-13  3.9e-15
   3       0.014   0.3500000       0.014   28   21   21  1.6e-13  5.1e-15
Velocity u2, Pressure p
Iter      SolEst     Damping    Stepsize #Res #Jac #Sol   LinErr   LinRes
   1      0.0084   0.5000000      0.0084   14    7    7  3.9e-15  7.7e-15
Displacement field
Iter      SolEst     Damping    Stepsize #Res #Jac #Sol   LinErr   LinRes
   1       0.054   0.5000000       0.054   14    7    7    1e-13  1.5e-12
Spatial mesh displacement
Iter      SolEst     Damping    Stepsize #Res #Jac #Sol   LinErr   LinRes
   1     2.9e-07   0.5000000     2.9e-07   14    7    7  1.5e-15  1.8e-15
Solution error estimates for segregated groups
0.024, 0.0042, 0.027, 1.4e-07
Residual error estimates for segregated groups
4.1, 5.3e+02, 2.6e+03, 0.17
Segregated solver iteration 8.
Merged variables
Iter      SolEst     Damping    Stepsize #Res #Jac #Sol   LinErr   LinRes
   1      0.0092   0.3500000      0.0092   30   22   22  8.6e-14  5.5e-15
   2       0.006   0.3500000       0.006   31   23   23    1e-13  5.9e-15
   3      0.0039   0.3500000      0.0039   32   24   24  9.7e-14  3.2e-15
Velocity u2, Pressure p
Iter      SolEst     Damping    Stepsize #Res #Jac #Sol   LinErr   LinRes
   1      0.0042   0.5000000      0.0042   16    8    8  3.1e-15  7.8e-15
Displacement field
Iter      SolEst     Damping    Stepsize #Res #Jac #Sol   LinErr   LinRes
   1        0.03   0.5000000        0.03   16    8    8  3.6e-14  1.5e-12
Spatial mesh displacement
Iter      SolEst     Damping    Stepsize #Res #Jac #Sol   LinErr   LinRes
   1     1.9e-07   0.5000000     1.9e-07   16    8    8  1.5e-15  1.9e-15
Solution error estimates for segregated groups
0.0067, 0.0021, 0.015, 9.3e-08
Residual error estimates for segregated groups
1.4, 2.6e+02, 1.8e+03, 0.16
Segregated solver iteration 9.
Merged variables
Iter      SolEst     Damping    Stepsize #Res #Jac #Sol   LinErr   LinRes
   1      0.0025   0.3500000      0.0025   34   25   25  7.9e-14  7.5e-15
   2      0.0016   0.3500000      0.0016   35   26   26  1.6e-13  3.9e-15
   3      0.0011   0.3500000      0.0011   36   27   27  1.5e-13  3.6e-15
Velocity u2, Pressure p
Iter      SolEst     Damping    Stepsize #Res #Jac #Sol   LinErr   LinRes
   1      0.0021   0.5000000      0.0021   18    9    9  3.8e-15  7.7e-15
Displacement field
Iter      SolEst     Damping    Stepsize #Res #Jac #Sol   LinErr   LinRes
   1       0.017   0.5000000       0.017   18    9    9  5.9e-14  1.5e-12
Spatial mesh displacement
Iter      SolEst     Damping    Stepsize #Res #Jac #Sol   LinErr   LinRes
   1     1.2e-07   0.5000000     1.2e-07   18    9    9  1.3e-15  1.8e-15
Solution error estimates for segregated groups
0.0018, 0.001, 0.0085, 5.8e-08
Residual error estimates for segregated groups
```

```
0.44, 1.3e+02, 1.4e+03, 0.17
Segregated solver iteration 10.
Merged variables
Iter      SolEst     Damping    Stepsize #Res #Jac #Sol   LinErr   LinRes
   1     0.00069   0.3500000     0.00069   38   28   28  5.1e-14  4.3e-15
   2     0.00045   0.3500000     0.00045   39   29   29  1.1e-13  4.6e-15
   3     0.00029   0.3500000     0.00029   40   30   30  1.4e-13  4.5e-15
Velocity u2, Pressure p
Iter      SolEst     Damping    Stepsize #Res #Jac #Sol   LinErr   LinRes
   1       0.001   0.5000000       0.001   20   10   10    3e-15  7.7e-15
Displacement field
Iter      SolEst     Damping    Stepsize #Res #Jac #Sol   LinErr   LinRes
   1      0.0094   0.5000000      0.0094   20   10   10  4.4e-14  1.5e-12
Spatial mesh displacement
Iter      SolEst     Damping    Stepsize #Res #Jac #Sol   LinErr   LinRes
   1     7.1e-08   0.5000000     7.1e-08   20   10   10  2.1e-15  1.8e-15
Solution error estimates for segregated groups
0.0005, 0.00052, 0.0047, 3.6e-08
Residual error estimates for segregated groups
0.14, 66, 1.2e+03, 0.17
Segregated solver iteration 11.
Merged variables
Iter      SolEst     Damping    Stepsize #Res #Jac #Sol   LinErr   LinRes
   1     0.00019   0.3500000     0.00019   42   31   31  2.1e-13  6.3e-15
   2     0.00012   0.3500000     0.00012   43   32   32  1.6e-13  3.6e-15
   3     8.1e-05   0.3500000     8.1e-05   44   33   33  1.9e-13  6.6e-15
Velocity u2, Pressure p
Iter      SolEst     Damping    Stepsize #Res #Jac #Sol   LinErr   LinRes
   1     0.00052   0.5000000     0.00052   22   11   11  3.5e-15  7.9e-15
Displacement field
Iter      SolEst     Damping    Stepsize #Res #Jac #Sol   LinErr   LinRes
   1      0.0051   0.5000000      0.0051   22   11   11  7.6e-14  1.5e-12
Spatial mesh displacement
Iter      SolEst     Damping    Stepsize #Res #Jac #Sol   LinErr   LinRes
   1     4.3e-08   0.5000000     4.3e-08   22   11   11  1.7e-15  1.8e-15
Solution error estimates for segregated groups
0.00014, 0.00026, 0.0026, 2.1e-08
Residual error estimates for segregated groups
0.043, 33, 1.2e+03, 0.17
Segregated solver iteration 12.
Merged variables
Iter      SolEst     Damping    Stepsize #Res #Jac #Sol   LinErr   LinRes
   1     5.2e-05   0.3500000     5.2e-05   46   34   34    1e-13  3.5e-15
   2     3.4e-05   0.3500000     3.4e-05   47   35   35  1.2e-13  3.3e-15
   3     2.2e-05   0.3500000     2.2e-05   48   36   36  7.4e-14  3.3e-15
Velocity u2, Pressure p
Iter      SolEst     Damping    Stepsize #Res #Jac #Sol   LinErr   LinRes
   1     0.00026   0.5000000     0.00026   24   12   12  4.1e-15  7.9e-15
Displacement field
Iter      SolEst     Damping    Stepsize #Res #Jac #Sol   LinErr   LinRes
   1      0.0028   0.5000000      0.0028   24   12   12  8.1e-14  1.5e-12
Spatial mesh displacement
Iter      SolEst     Damping    Stepsize #Res #Jac #Sol   LinErr   LinRes
   1     2.5e-08   0.5000000     2.5e-08   24   12   12  1.5e-15  1.8e-15
Solution error estimates for segregated groups
3.8e-05, 0.00013, 0.0014, 1.3e-08
```

```
Residual error estimates for segregated groups
0.013, 17, 1.2e+03, 0.17
Segregated solver iteration 13.
Merged variables
Iter      SolEst     Damping    Stepsize #Res #Jac #Sol   LinErr   LinRes
   1     1.4e-05   0.3500000     1.4e-05   50   37   37  5.3e-14  3.9e-15
   2     9.4e-06   0.3500000     9.4e-06   51   38   38  1.1e-13  3.8e-15
   3     6.1e-06   0.3500000     6.1e-06   52   39   39    1e-13  3.7e-15
Velocity u2, Pressure p
Iter      SolEst     Damping    Stepsize #Res #Jac #Sol   LinErr   LinRes
   1     0.00013   0.5000000     0.00013   26   13   13  2.2e-15  7.7e-15
Displacement field
Iter      SolEst     Damping    Stepsize #Res #Jac #Sol   LinErr   LinRes
   1      0.0015   0.5000000      0.0015   26   13   13  3.8e-14  1.5e-12
Spatial mesh displacement
Iter      SolEst     Damping    Stepsize #Res #Jac #Sol   LinErr   LinRes
   1     1.5e-08   0.5000000     1.5e-08   26   13   13  1.3e-15  1.8e-15
Solution error estimates for segregated groups
1e-05, 6.6e-05, 0.00076, 7.3e-09
Residual error estimates for segregated groups
0.004, 8.3, 1.2e+03, 0.16
Solution time: 155 s. (2 minutes, 35 seconds)
Physical memory: 4.73 GB
Virtual memory: 25.54 GB
Ended at Jan 31, 2025, 7:06:36 PM.
----- Stationary Solver 1 in study1/Approach Curve Solver (sol1) -------------->
```

d=2255 (su51)

GENERAL

Description	Value
Solution	d=2255

LOG

```
<---- Compile Equations: Stationary in study1/Approach Curve Solver (sol1) -----
Started at Jan 31, 2025, 7:06:36 PM.
Parameter d = 2.255E-6 (m).
Geometry shape function: Linear serendipity
Number of vertex elements: 18
Number of boundary elements: 6207
Number of elements: 155651
Minimum element quality: 0.4893
Time: 8 s.
Physical memory: 2.95 GB
Virtual memory: 15.28 GB
Ended at Jan 31, 2025, 7:06:45 PM.
----- Compile Equations: Stationary in study1/Approach Curve Solver (sol1) ---->

<---- Dependent Variables 1 in study1/Approach Curve Solver (sol1) -------------
Started at Jan 31, 2025, 7:06:45 PM.
Solution time: 0 s.
Physical memory: 2.95 GB
Virtual memory: 15.21 GB
Ended at Jan 31, 2025, 7:06:45 PM.
----- Dependent Variables 1 in study1/Approach Curve Solver (sol1) ------------>

<---- Stationary Solver 1 in study1/Approach Curve Solver (sol1) ---------------
Started at Jan 31, 2025, 7:06:45 PM.
Segregated solver
Segregated Step 11 does not have any components solved for.
Number of degrees of freedom solved for: 791029 (plus 11837 internal DOFs).
Segregated solver iteration 1.
Merged variables
Nonsymmetric matrix found.
Scales for dependent variables:
Concentration (comp1.Cl): 1.4e+02
Concentration (comp1.K): 1.4e+02
Electric potential (comp1.V): 1.9e+02
Orthonormal null-space function used.
Iter      SolEst     Damping    Stepsize #Res #Jac #Sol   LinErr   LinRes
   1        0.83   0.3500000        0.83    2    1    1  4.5e-11  2.8e-15
   2        0.81   0.3500000        0.81    3    2    2  3.1e-11  2.7e-15
   3         0.8   0.3500000         0.8    4    3    3  1.6e-11  2.8e-15
Velocity u2, Pressure p
Nonsymmetric matrix found.
Scales for dependent variables:
Pressure (comp1.p): 1
Velocity field (spatial frame) (comp1.u2): 0.00018
Orthonormal null-space function used.
Iter      SolEst     Damping    Stepsize #Res #Jac #Sol   LinErr   LinRes
   1         1.1   0.5000000         1.1    2    1    1  1.8e-15  2.2e-15
Displacement field
Nonsymmetric matrix found.
Scales for dependent variables:
Auxiliary pressure (comp1.solid.lemm1.pw): 1
Displacement field (comp1.u): 2.8e-18
Orthonormal null-space function used.
Iter      SolEst     Damping    Stepsize #Res #Jac #Sol   LinErr   LinRes
   1         1.9   0.5000000         1.9    2    1    1  1.3e-13  1.5e-12
Spatial mesh displacement
```

```
Symmetric matrices found.
Scales for dependent variables:
Spatial mesh displacement (comp1.spatial.disp): 1.2e-09
Orthonormal null-space function used.
Iter      SolEst     Damping    Stepsize #Res #Jac #Sol   LinErr   LinRes
   1     6.7e-07   0.5000000     6.7e-07    2    1    1  1.5e-15  1.8e-15
Solution error estimates for segregated groups
1.4, 0.54, 0.95, 3.3e-07
Residual error estimates for segregated groups
7.9e+03, 8.3e+04, 2.2e+04, 0.14
Segregated solver iteration 2.
Merged variables
Scales for dependent variables:
Concentration (comp1.Cl): 1.4e+02
Concentration (comp1.K): 1.4e+02
Electric potential (comp1.V): 0.064
Iter      SolEst     Damping    Stepsize #Res #Jac #Sol   LinErr   LinRes
   1        0.78   0.3500000        0.78    6    4    4  1.4e-13  2.8e-15
   2        0.77   0.3500000        0.77    7    5    5    9e-14    3e-15
   3        0.76   0.3500000        0.76    8    6    6  6.2e-14    3e-15
Velocity u2, Pressure p
Scales for dependent variables:
Pressure (comp1.p): 3.6e+02
Velocity field (spatial frame) (comp1.u2): 0.00072
Iter      SolEst     Damping    Stepsize #Res #Jac #Sol   LinErr   LinRes
   1        0.36   0.5000000        0.36    4    2    2  2.5e-15  7.7e-15
Displacement field
Iter      SolEst     Damping    Stepsize #Res #Jac #Sol   LinErr   LinRes
   1        0.95   0.5000000        0.95    4    2    2  4.2e-14  1.5e-12
Spatial mesh displacement
Iter      SolEst     Damping    Stepsize #Res #Jac #Sol   LinErr   LinRes
   1       1e-06   0.5000000       1e-06    4    2    2  1.6e-15  1.8e-15
Solution error estimates for segregated groups
1.3, 0.18, 0.48, 5e-07
Residual error estimates for segregated groups
7.9e+02, 1.3e+04, 2.2e+04, 0.15
Segregated solver iteration 3.
Merged variables
Iter      SolEst     Damping    Stepsize #Res #Jac #Sol   LinErr   LinRes
   1        0.75   0.3500000        0.75   10    7    7  1.5e-13    3e-15
   2        0.73   0.3500000        0.73   11    8    8  4.7e-14  2.8e-15
   3        0.72   0.3500000        0.72   12    9    9  5.2e-14  2.8e-15
Velocity u2, Pressure p
Iter      SolEst     Damping    Stepsize #Res #Jac #Sol   LinErr   LinRes
   1        0.15   0.5000000        0.15    6    3    3  4.3e-15  7.7e-15
Displacement field
Iter      SolEst     Damping    Stepsize #Res #Jac #Sol   LinErr   LinRes
   1        0.52   0.5000000        0.52    6    3    3  4.2e-14  1.5e-12
Spatial mesh displacement
Iter      SolEst     Damping    Stepsize #Res #Jac #Sol   LinErr   LinRes
   1       1e-06   0.5000000       1e-06    6    3    3  1.5e-15  1.8e-15
Solution error estimates for segregated groups
1.2, 0.077, 0.26, 5e-07
Residual error estimates for segregated groups
2.2e+02, 7.5e+03, 1.6e+04, 0.16
Segregated solver iteration 4.
```

```
Merged variables
Iter      SolEst     Damping    Stepsize #Res #Jac #Sol   LinErr   LinRes
   1         0.7   0.3500000         0.7   14   10   10  7.2e-14  2.9e-15
   2        0.68   0.3500000        0.68   15   11   11  4.6e-14  3.1e-15
   3         0.6   0.3500000         0.6   16   12   12  4.9e-14  3.1e-15
Velocity u2, Pressure p
Iter      SolEst     Damping    Stepsize #Res #Jac #Sol   LinErr   LinRes
   1       0.072   0.5000000       0.072    8    4    4  2.4e-15  7.8e-15
Displacement field
Iter      SolEst     Damping    Stepsize #Res #Jac #Sol   LinErr   LinRes
   1        0.29   0.5000000        0.29    8    4    4  1.1e-13  1.5e-12
Spatial mesh displacement
Iter      SolEst     Damping    Stepsize #Res #Jac #Sol   LinErr   LinRes
   1     8.3e-07   0.5000000     8.3e-07    8    4    4  1.4e-15  1.8e-15
Solution error estimates for segregated groups
1, 0.036, 0.15, 4.2e-07
Residual error estimates for segregated groups
84, 4e+03, 1.1e+04, 0.16
Segregated solver iteration 5.
Merged variables
Iter      SolEst     Damping    Stepsize #Res #Jac #Sol   LinErr   LinRes
   1        0.41   0.3500000        0.41   18   13   13    1e-13    3e-15
   2        0.27   0.3500000        0.27   19   14   14  8.2e-14  3.1e-15
   3        0.18   0.3500000        0.18   20   15   15  1.1e-13    3e-15
Velocity u2, Pressure p
Iter      SolEst     Damping    Stepsize #Res #Jac #Sol   LinErr   LinRes
   1       0.035   0.5000000       0.035   10    5    5  2.6e-15  7.8e-15
Displacement field
Iter      SolEst     Damping    Stepsize #Res #Jac #Sol   LinErr   LinRes
   1        0.17   0.5000000        0.17   10    5    5  2.6e-14  1.5e-12
Spatial mesh displacement
Iter      SolEst     Damping    Stepsize #Res #Jac #Sol   LinErr   LinRes
   1     6.2e-07   0.5000000     6.2e-07   10    5    5  1.3e-15  1.9e-15
Solution error estimates for segregated groups
0.31, 0.017, 0.084, 3.1e-07
Residual error estimates for segregated groups
33, 2e+03, 6.9e+03, 0.16
Segregated solver iteration 6.
Merged variables
Iter      SolEst     Damping    Stepsize #Res #Jac #Sol   LinErr   LinRes
   1        0.12   0.3500000        0.12   22   16   16  5.7e-14  2.9e-15
   2       0.078   0.3500000       0.078   23   17   17  1.1e-13  2.7e-15
   3       0.051   0.3500000       0.051   24   18   18  6.4e-14  2.8e-15
Velocity u2, Pressure p
Iter      SolEst     Damping    Stepsize #Res #Jac #Sol   LinErr   LinRes
   1       0.017   0.5000000       0.017   12    6    6  2.4e-15  7.8e-15
Displacement field
Iter      SolEst     Damping    Stepsize #Res #Jac #Sol   LinErr   LinRes
   1       0.095   0.5000000       0.095   12    6    6  1.4e-13  1.5e-12
Spatial mesh displacement
Iter      SolEst     Damping    Stepsize #Res #Jac #Sol   LinErr   LinRes
   1     4.4e-07   0.5000000     4.4e-07   12    6    6  1.7e-15  1.8e-15
Solution error estimates for segregated groups
0.088, 0.0085, 0.048, 2.2e-07
Residual error estimates for segregated groups
12, 1e+03, 4.2e+03, 0.16
```

```
Segregated solver iteration 7.
Merged variables
Iter      SolEst     Damping    Stepsize #Res #Jac #Sol   LinErr   LinRes
   1       0.033   0.3500000       0.033   26   19   19  9.4e-14  2.9e-15
   2       0.022   0.3500000       0.022   27   20   20  8.3e-14  2.8e-15
   3       0.014   0.3500000       0.014   28   21   21    1e-13  2.9e-15
Velocity u2, Pressure p
Iter      SolEst     Damping    Stepsize #Res #Jac #Sol   LinErr   LinRes
   1      0.0085   0.5000000      0.0085   14    7    7  2.6e-15    8e-15
Displacement field
Iter      SolEst     Damping    Stepsize #Res #Jac #Sol   LinErr   LinRes
   1       0.054   0.5000000       0.054   14    7    7  6.2e-14  1.5e-12
Spatial mesh displacement
Iter      SolEst     Damping    Stepsize #Res #Jac #Sol   LinErr   LinRes
   1     2.9e-07   0.5000000     2.9e-07   14    7    7  1.5e-15  1.8e-15
Solution error estimates for segregated groups
0.024, 0.0042, 0.027, 1.5e-07
Residual error estimates for segregated groups
4.1, 5.2e+02, 2.6e+03, 0.18
Segregated solver iteration 8.
Merged variables
Iter      SolEst     Damping    Stepsize #Res #Jac #Sol   LinErr   LinRes
   1      0.0092   0.3500000      0.0092   30   22   22  9.4e-14  2.7e-15
   2       0.006   0.3500000       0.006   31   23   23  4.6e-14  2.7e-15
   3      0.0039   0.3500000      0.0039   32   24   24  9.9e-14  2.8e-15
Velocity u2, Pressure p
Iter      SolEst     Damping    Stepsize #Res #Jac #Sol   LinErr   LinRes
   1      0.0042   0.5000000      0.0042   16    8    8  4.7e-15  7.7e-15
Displacement field
Iter      SolEst     Damping    Stepsize #Res #Jac #Sol   LinErr   LinRes
   1        0.03   0.5000000        0.03   16    8    8  4.7e-14  1.5e-12
Spatial mesh displacement
Iter      SolEst     Damping    Stepsize #Res #Jac #Sol   LinErr   LinRes
   1     1.9e-07   0.5000000     1.9e-07   16    8    8  1.7e-15  1.8e-15
Solution error estimates for segregated groups
0.0067, 0.0021, 0.015, 9.4e-08
Residual error estimates for segregated groups
1.4, 2.6e+02, 1.8e+03, 0.16
Segregated solver iteration 9.
Merged variables
Iter      SolEst     Damping    Stepsize #Res #Jac #Sol   LinErr   LinRes
   1      0.0025   0.3500000      0.0025   34   25   25    4e-14  3.7e-15
   2      0.0016   0.3500000      0.0016   35   26   26  5.1e-14  3.2e-15
   3      0.0011   0.3500000      0.0011   36   27   27    1e-13    3e-15
Velocity u2, Pressure p
Iter      SolEst     Damping    Stepsize #Res #Jac #Sol   LinErr   LinRes
   1      0.0021   0.5000000      0.0021   18    9    9  2.2e-15  7.8e-15
Displacement field
Iter      SolEst     Damping    Stepsize #Res #Jac #Sol   LinErr   LinRes
   1       0.017   0.5000000       0.017   18    9    9  3.7e-14  1.5e-12
Spatial mesh displacement
Iter      SolEst     Damping    Stepsize #Res #Jac #Sol   LinErr   LinRes
   1     1.2e-07   0.5000000     1.2e-07   18    9    9  1.5e-15  1.8e-15
Solution error estimates for segregated groups
0.0018, 0.0011, 0.0085, 5.9e-08
Residual error estimates for segregated groups
```

```
0.44, 1.3e+02, 1.4e+03, 0.16
Segregated solver iteration 10.
Merged variables
Iter      SolEst     Damping    Stepsize #Res #Jac #Sol   LinErr   LinRes
   1     0.00069   0.3500000     0.00069   38   28   28  4.1e-14  2.9e-15
   2     0.00045   0.3500000     0.00045   39   29   29  8.3e-14    3e-15
   3     0.00029   0.3500000     0.00029   40   30   30  1.4e-13  2.9e-15
Velocity u2, Pressure p
Iter      SolEst     Damping    Stepsize #Res #Jac #Sol   LinErr   LinRes
   1       0.001   0.5000000       0.001   20   10   10  2.4e-15  7.7e-15
Displacement field
Iter      SolEst     Damping    Stepsize #Res #Jac #Sol   LinErr   LinRes
   1      0.0094   0.5000000      0.0094   20   10   10    1e-13  1.5e-12
Spatial mesh displacement
Iter      SolEst     Damping    Stepsize #Res #Jac #Sol   LinErr   LinRes
   1     7.2e-08   0.5000000     7.2e-08   20   10   10  1.4e-15  1.8e-15
Solution error estimates for segregated groups
0.0005, 0.00052, 0.0047, 3.6e-08
Residual error estimates for segregated groups
0.14, 65, 1.2e+03, 0.16
Segregated solver iteration 11.
Merged variables
Iter      SolEst     Damping    Stepsize #Res #Jac #Sol   LinErr   LinRes
   1     0.00019   0.3500000     0.00019   42   31   31  1.1e-13  2.8e-15
   2     0.00012   0.3500000     0.00012   43   32   32  8.5e-14    3e-15
   3       8e-05   0.3500000       8e-05   44   33   33  5.4e-14  2.8e-15
Velocity u2, Pressure p
Iter      SolEst     Damping    Stepsize #Res #Jac #Sol   LinErr   LinRes
   1     0.00052   0.5000000     0.00052   22   11   11  5.1e-15  7.8e-15
Displacement field
Iter      SolEst     Damping    Stepsize #Res #Jac #Sol   LinErr   LinRes
   1      0.0051   0.5000000      0.0051   22   11   11  4.6e-14  1.5e-12
Spatial mesh displacement
Iter      SolEst     Damping    Stepsize #Res #Jac #Sol   LinErr   LinRes
   1     4.3e-08   0.5000000     4.3e-08   22   11   11  2.2e-15  1.8e-15
Solution error estimates for segregated groups
0.00014, 0.00026, 0.0026, 2.1e-08
Residual error estimates for segregated groups
0.043, 33, 1.2e+03, 0.16
Segregated solver iteration 12.
Merged variables
Iter      SolEst     Damping    Stepsize #Res #Jac #Sol   LinErr   LinRes
   1     5.2e-05   0.3500000     5.2e-05   46   34   34  7.5e-14  2.8e-15
   2     3.4e-05   0.3500000     3.4e-05   47   35   35  9.1e-14  2.8e-15
   3     2.2e-05   0.3500000     2.2e-05   48   36   36  2.8e-14  2.8e-15
Velocity u2, Pressure p
Iter      SolEst     Damping    Stepsize #Res #Jac #Sol   LinErr   LinRes
   1     0.00026   0.5000000     0.00026   24   12   12  3.3e-15  7.8e-15
Displacement field
Scales for dependent variables:
Auxiliary pressure (comp1.solid.lemm1.pw): 0.36
Displacement field (comp1.u): 2.8e-16
Iter      SolEst     Damping    Stepsize #Res #Jac #Sol   LinErr   LinRes
   1      0.0028   0.5000000      0.0028   24   12   12  1.5e-13  1.2e-12
Spatial mesh displacement
Iter      SolEst     Damping    Stepsize #Res #Jac #Sol   LinErr   LinRes
```

```
   1     2.5e-08   0.5000000     2.5e-08   24   12   12  1.7e-15  1.8e-15
Solution error estimates for segregated groups
3.8e-05, 0.00013, 0.0014, 1.3e-08
Residual error estimates for segregated groups
0.013, 16, 1.2e+03, 0.16
Segregated solver iteration 13.
Merged variables
Iter      SolEst     Damping    Stepsize #Res #Jac #Sol   LinErr   LinRes
   1     1.4e-05   0.3500000     1.4e-05   50   37   37  8.5e-14    3e-15
   2     9.3e-06   0.3500000     9.3e-06   51   38   38  6.2e-14  3.2e-15
   3     6.1e-06   0.3500000     6.1e-06   52   39   39  7.7e-14  3.1e-15
Velocity u2, Pressure p
Iter      SolEst     Damping    Stepsize #Res #Jac #Sol   LinErr   LinRes
   1     0.00013   0.5000000     0.00013   26   13   13  2.3e-15  7.7e-15
Displacement field
Iter      SolEst     Damping    Stepsize #Res #Jac #Sol   LinErr   LinRes
   1      0.0015   0.5000000      0.0015   26   13   13    1e-13  1.2e-12
Spatial mesh displacement
Iter      SolEst     Damping    Stepsize #Res #Jac #Sol   LinErr   LinRes
   1     1.5e-08   0.5000000     1.5e-08   26   13   13  1.7e-15  1.8e-15
Solution error estimates for segregated groups
1e-05, 6.6e-05, 0.00076, 7.4e-09
Residual error estimates for segregated groups
0.004, 8.2, 1.2e+03, 0.16
Solution time: 156 s. (2 minutes, 36 seconds)
Physical memory: 4.68 GB
Virtual memory: 25.84 GB
Ended at Jan 31, 2025, 7:09:21 PM.
----- Stationary Solver 1 in study1/Approach Curve Solver (sol1) -------------->
```

d=2250 (su52)

GENERAL

Description	Value
Solution	d=2250

LOG

```
<---- Compile Equations: Stationary in study1/Approach Curve Solver (sol1) -----
Started at Jan 31, 2025, 7:09:21 PM.
Parameter d = 2.25E-6 (m).
Geometry shape function: Linear serendipity
Number of vertex elements: 18
Number of boundary elements: 6204
Number of elements: 155528
Minimum element quality: 0.4983
Time: 8 s.
Physical memory: 2.96 GB
Virtual memory: 15.28 GB
Ended at Jan 31, 2025, 7:09:30 PM.
----- Compile Equations: Stationary in study1/Approach Curve Solver (sol1) ---->

<---- Dependent Variables 1 in study1/Approach Curve Solver (sol1) -------------
Started at Jan 31, 2025, 7:09:30 PM.
Solution time: 0 s.
Physical memory: 2.96 GB
Virtual memory: 15.22 GB
Ended at Jan 31, 2025, 7:09:30 PM.
----- Dependent Variables 1 in study1/Approach Curve Solver (sol1) ------------>

<---- Stationary Solver 1 in study1/Approach Curve Solver (sol1) ---------------
Started at Jan 31, 2025, 7:09:30 PM.
Segregated solver
Segregated Step 11 does not have any components solved for.
Number of degrees of freedom solved for: 790411 (plus 11831 internal DOFs).
Segregated solver iteration 1.
Merged variables
Nonsymmetric matrix found.
Scales for dependent variables:
Concentration (comp1.Cl): 1.4e+02
Concentration (comp1.K): 1.4e+02
Electric potential (comp1.V): 1.9e+02
Orthonormal null-space function used.
Iter      SolEst     Damping    Stepsize #Res #Jac #Sol   LinErr   LinRes
   1        0.83   0.3500000        0.83    2    1    1  4.3e-11  2.7e-15
   2        0.81   0.3500000        0.81    3    2    2  2.8e-11  2.7e-15
   3         0.8   0.3500000         0.8    4    3    3  1.4e-11  2.7e-15
Velocity u2, Pressure p
Nonsymmetric matrix found.
Scales for dependent variables:
Pressure (comp1.p): 1
Velocity field (spatial frame) (comp1.u2): 0.00018
Orthonormal null-space function used.
Iter      SolEst     Damping    Stepsize #Res #Jac #Sol   LinErr   LinRes
   1         1.1   0.5000000         1.1    2    1    1  2.2e-15  2.3e-15
Displacement field
Nonsymmetric matrix found.
Scales for dependent variables:
Auxiliary pressure (comp1.solid.lemm1.pw): 1
Displacement field (comp1.u): 2.8e-18
Orthonormal null-space function used.
Iter      SolEst     Damping    Stepsize #Res #Jac #Sol   LinErr   LinRes
   1         1.9   0.5000000         1.9    2    1    1  7.2e-14  1.5e-12
Spatial mesh displacement
```

```
Symmetric matrices found.
Scales for dependent variables:
Spatial mesh displacement (comp1.spatial.disp): 1.2e-09
Orthonormal null-space function used.
Iter      SolEst     Damping    Stepsize #Res #Jac #Sol   LinErr   LinRes
   1     6.7e-07   0.5000000     6.7e-07    2    1    1  1.9e-15  1.9e-15
Solution error estimates for segregated groups
1.4, 0.54, 0.95, 3.4e-07
Residual error estimates for segregated groups
7.9e+03, 8.3e+04, 2.2e+04, 0.14
Segregated solver iteration 2.
Merged variables
Scales for dependent variables:
Concentration (comp1.Cl): 1.4e+02
Concentration (comp1.K): 1.4e+02
Electric potential (comp1.V): 0.064
Iter      SolEst     Damping    Stepsize #Res #Jac #Sol   LinErr   LinRes
   1        0.78   0.3500000        0.78    6    4    4  9.2e-14  2.8e-15
   2        0.77   0.3500000        0.77    7    5    5  4.3e-14  2.8e-15
   3        0.76   0.3500000        0.76    8    6    6    3e-14  2.9e-15
Velocity u2, Pressure p
Scales for dependent variables:
Pressure (comp1.p): 3.6e+02
Velocity field (spatial frame) (comp1.u2): 0.00072
Iter      SolEst     Damping    Stepsize #Res #Jac #Sol   LinErr   LinRes
   1        0.36   0.5000000        0.36    4    2    2  2.4e-15    8e-15
Displacement field
Iter      SolEst     Damping    Stepsize #Res #Jac #Sol   LinErr   LinRes
   1        0.95   0.5000000        0.95    4    2    2  9.7e-14  1.5e-12
Spatial mesh displacement
Iter      SolEst     Damping    Stepsize #Res #Jac #Sol   LinErr   LinRes
   1       1e-06   0.5000000       1e-06    4    2    2  1.4e-15  1.9e-15
Solution error estimates for segregated groups
1.3, 0.18, 0.48, 5e-07
Residual error estimates for segregated groups
7.9e+02, 1.4e+04, 2.2e+04, 0.15
Segregated solver iteration 3.
Merged variables
Iter      SolEst     Damping    Stepsize #Res #Jac #Sol   LinErr   LinRes
   1        0.75   0.3500000        0.75   10    7    7  5.7e-14  2.7e-15
   2        0.73   0.3500000        0.73   11    8    8  9.6e-14  2.8e-15
   3        0.72   0.3500000        0.72   12    9    9  4.6e-14  2.9e-15
Velocity u2, Pressure p
Iter      SolEst     Damping    Stepsize #Res #Jac #Sol   LinErr   LinRes
   1        0.15   0.5000000        0.15    6    3    3  3.8e-15  7.8e-15
Displacement field
Iter      SolEst     Damping    Stepsize #Res #Jac #Sol   LinErr   LinRes
   1        0.52   0.5000000        0.52    6    3    3  3.5e-14  1.5e-12
Spatial mesh displacement
Iter      SolEst     Damping    Stepsize #Res #Jac #Sol   LinErr   LinRes
   1       1e-06   0.5000000       1e-06    6    3    3  1.4e-15  1.9e-15
Solution error estimates for segregated groups
1.2, 0.077, 0.26, 5e-07
Residual error estimates for segregated groups
2.2e+02, 7.6e+03, 1.6e+04, 0.16
Segregated solver iteration 4.
```

```
Merged variables
Iter      SolEst     Damping    Stepsize #Res #Jac #Sol   LinErr   LinRes
   1         0.7   0.3500000         0.7   14   10   10  7.9e-14  3.5e-15
   2        0.68   0.3500000        0.68   15   11   11  6.9e-14    3e-15
   3         0.6   0.3500000         0.6   16   12   12    5e-14  2.8e-15
Velocity u2, Pressure p
Iter      SolEst     Damping    Stepsize #Res #Jac #Sol   LinErr   LinRes
   1       0.072   0.5000000       0.072    8    4    4  2.5e-15  7.8e-15
Displacement field
Iter      SolEst     Damping    Stepsize #Res #Jac #Sol   LinErr   LinRes
   1        0.29   0.5000000        0.29    8    4    4  6.2e-14  1.5e-12
Spatial mesh displacement
Iter      SolEst     Damping    Stepsize #Res #Jac #Sol   LinErr   LinRes
   1     8.4e-07   0.5000000     8.4e-07    8    4    4  1.8e-15  1.9e-15
Solution error estimates for segregated groups
1, 0.036, 0.15, 4.2e-07
Residual error estimates for segregated groups
84, 4e+03, 1.1e+04, 0.15
Segregated solver iteration 5.
Merged variables
Iter      SolEst     Damping    Stepsize #Res #Jac #Sol   LinErr   LinRes
   1        0.41   0.3500000        0.41   18   13   13  8.5e-14  3.1e-15
   2        0.27   0.3500000        0.27   19   14   14  4.3e-14  2.9e-15
   3        0.18   0.3500000        0.18   20   15   15    7e-14  2.9e-15
Velocity u2, Pressure p
Iter      SolEst     Damping    Stepsize #Res #Jac #Sol   LinErr   LinRes
   1       0.035   0.5000000       0.035   10    5    5  3.7e-15  7.8e-15
Displacement field
Iter      SolEst     Damping    Stepsize #Res #Jac #Sol   LinErr   LinRes
   1        0.17   0.5000000        0.17   10    5    5  5.6e-14  1.5e-12
Spatial mesh displacement
Iter      SolEst     Damping    Stepsize #Res #Jac #Sol   LinErr   LinRes
   1     6.3e-07   0.5000000     6.3e-07   10    5    5  1.5e-15  1.9e-15
Solution error estimates for segregated groups
0.31, 0.017, 0.084, 3.1e-07
Residual error estimates for segregated groups
33, 2.1e+03, 6.8e+03, 0.16
Segregated solver iteration 6.
Merged variables
Iter      SolEst     Damping    Stepsize #Res #Jac #Sol   LinErr   LinRes
   1        0.12   0.3500000        0.12   22   16   16  8.8e-14    3e-15
   2       0.078   0.3500000       0.078   23   17   17  4.4e-14  2.9e-15
   3       0.051   0.3500000       0.051   24   18   18  4.9e-14  2.9e-15
Velocity u2, Pressure p
Iter      SolEst     Damping    Stepsize #Res #Jac #Sol   LinErr   LinRes
   1       0.017   0.5000000       0.017   12    6    6  2.4e-15    8e-15
Displacement field
Iter      SolEst     Damping    Stepsize #Res #Jac #Sol   LinErr   LinRes
   1       0.095   0.5000000       0.095   12    6    6  2.1e-13  1.5e-12
Spatial mesh displacement
Iter      SolEst     Damping    Stepsize #Res #Jac #Sol   LinErr   LinRes
   1     4.4e-07   0.5000000     4.4e-07   12    6    6    2e-15  1.9e-15
Solution error estimates for segregated groups
0.087, 0.0085, 0.048, 2.2e-07
Residual error estimates for segregated groups
12, 1e+03, 4.2e+03, 0.16
```

```
Segregated solver iteration 7.
Merged variables
Iter      SolEst     Damping    Stepsize #Res #Jac #Sol   LinErr   LinRes
   1       0.033   0.3500000       0.033   26   19   19  8.9e-14  2.9e-15
   2       0.022   0.3500000       0.022   27   20   20  6.4e-14  2.7e-15
   3       0.014   0.3500000       0.014   28   21   21  4.9e-14  2.9e-15
Velocity u2, Pressure p
Iter      SolEst     Damping    Stepsize #Res #Jac #Sol   LinErr   LinRes
   1      0.0085   0.5000000      0.0085   14    7    7  2.6e-15  7.8e-15
Displacement field
Iter      SolEst     Damping    Stepsize #Res #Jac #Sol   LinErr   LinRes
   1       0.054   0.5000000       0.054   14    7    7  6.6e-14  1.5e-12
Spatial mesh displacement
Iter      SolEst     Damping    Stepsize #Res #Jac #Sol   LinErr   LinRes
   1     2.9e-07   0.5000000     2.9e-07   14    7    7  1.3e-15  1.9e-15
Solution error estimates for segregated groups
0.024, 0.0042, 0.027, 1.5e-07
Residual error estimates for segregated groups
4.1, 5.2e+02, 2.6e+03, 0.15
Segregated solver iteration 8.
Merged variables
Iter      SolEst     Damping    Stepsize #Res #Jac #Sol   LinErr   LinRes
   1      0.0092   0.3500000      0.0092   30   22   22  1.6e-13    3e-15
   2       0.006   0.3500000       0.006   31   23   23  7.1e-14    3e-15
   3      0.0039   0.3500000      0.0039   32   24   24  6.6e-14  2.8e-15
Velocity u2, Pressure p
Iter      SolEst     Damping    Stepsize #Res #Jac #Sol   LinErr   LinRes
   1      0.0042   0.5000000      0.0042   16    8    8  4.4e-15  7.9e-15
Displacement field
Iter      SolEst     Damping    Stepsize #Res #Jac #Sol   LinErr   LinRes
   1        0.03   0.5000000        0.03   16    8    8  7.2e-14  1.5e-12
Spatial mesh displacement
Iter      SolEst     Damping    Stepsize #Res #Jac #Sol   LinErr   LinRes
   1     1.9e-07   0.5000000     1.9e-07   16    8    8  1.6e-15  1.8e-15
Solution error estimates for segregated groups
0.0066, 0.0021, 0.015, 9.4e-08
Residual error estimates for segregated groups
1.4, 2.6e+02, 1.8e+03, 0.16
Segregated solver iteration 9.
Merged variables
Iter      SolEst     Damping    Stepsize #Res #Jac #Sol   LinErr   LinRes
   1      0.0025   0.3500000      0.0025   34   25   25    9e-14    3e-15
   2      0.0016   0.3500000      0.0016   35   26   26  6.6e-14  2.8e-15
   3      0.0011   0.3500000      0.0011   36   27   27  4.5e-14    3e-15
Velocity u2, Pressure p
Iter      SolEst     Damping    Stepsize #Res #Jac #Sol   LinErr   LinRes
   1      0.0021   0.5000000      0.0021   18    9    9  2.9e-15  7.8e-15
Displacement field
Iter      SolEst     Damping    Stepsize #Res #Jac #Sol   LinErr   LinRes
   1       0.017   0.5000000       0.017   18    9    9  6.4e-14  1.5e-12
Spatial mesh displacement
Iter      SolEst     Damping    Stepsize #Res #Jac #Sol   LinErr   LinRes
   1     1.2e-07   0.5000000     1.2e-07   18    9    9  1.5e-15  1.9e-15
Solution error estimates for segregated groups
0.0018, 0.0011, 0.0085, 5.9e-08
Residual error estimates for segregated groups
```

```
0.44, 1.3e+02, 1.4e+03, 0.17
Segregated solver iteration 10.
Merged variables
Iter      SolEst     Damping    Stepsize #Res #Jac #Sol   LinErr   LinRes
   1     0.00069   0.3500000     0.00069   38   28   28  5.8e-14  2.8e-15
   2     0.00045   0.3500000     0.00045   39   29   29    1e-13  2.9e-15
   3     0.00029   0.3500000     0.00029   40   30   30  9.1e-14  2.9e-15
Velocity u2, Pressure p
Iter      SolEst     Damping    Stepsize #Res #Jac #Sol   LinErr   LinRes
   1       0.001   0.5000000       0.001   20   10   10  2.7e-15  7.8e-15
Displacement field
Iter      SolEst     Damping    Stepsize #Res #Jac #Sol   LinErr   LinRes
   1      0.0094   0.5000000      0.0094   20   10   10  8.3e-14  1.5e-12
Spatial mesh displacement
Iter      SolEst     Damping    Stepsize #Res #Jac #Sol   LinErr   LinRes
   1     7.2e-08   0.5000000     7.2e-08   20   10   10  1.6e-15  1.9e-15
Solution error estimates for segregated groups
0.0005, 0.00052, 0.0047, 3.6e-08
Residual error estimates for segregated groups
0.14, 66, 1.2e+03, 0.15
Segregated solver iteration 11.
Merged variables
Iter      SolEst     Damping    Stepsize #Res #Jac #Sol   LinErr   LinRes
   1     0.00019   0.3500000     0.00019   42   31   31  8.9e-14  2.8e-15
   2     0.00012   0.3500000     0.00012   43   32   32  5.5e-14  2.8e-15
   3       8e-05   0.3500000       8e-05   44   33   33  9.1e-14  2.8e-15
Velocity u2, Pressure p
Iter      SolEst     Damping    Stepsize #Res #Jac #Sol   LinErr   LinRes
   1     0.00052   0.5000000     0.00052   22   11   11  2.9e-15  7.9e-15
Displacement field
Scales for dependent variables:
Auxiliary pressure (comp1.solid.lemm1.pw): 0.36
Displacement field (comp1.u): 2.8e-16
Iter      SolEst     Damping    Stepsize #Res #Jac #Sol   LinErr   LinRes
   1      0.0051   0.5000000      0.0051   22   11   11  1.7e-13  1.2e-12
Spatial mesh displacement
Iter      SolEst     Damping    Stepsize #Res #Jac #Sol   LinErr   LinRes
   1     4.3e-08   0.5000000     4.3e-08   22   11   11  1.4e-15  1.9e-15
Solution error estimates for segregated groups
0.00014, 0.00026, 0.0026, 2.2e-08
Residual error estimates for segregated groups
0.043, 33, 1.2e+03, 0.17
Segregated solver iteration 12.
Merged variables
Iter      SolEst     Damping    Stepsize #Res #Jac #Sol   LinErr   LinRes
   1     5.2e-05   0.3500000     5.2e-05   46   34   34  8.9e-14    3e-15
   2     3.4e-05   0.3500000     3.4e-05   47   35   35  8.1e-14  2.9e-15
   3     2.2e-05   0.3500000     2.2e-05   48   36   36  6.6e-14  2.8e-15
Velocity u2, Pressure p
Iter      SolEst     Damping    Stepsize #Res #Jac #Sol   LinErr   LinRes
   1     0.00026   0.5000000     0.00026   24   12   12  2.8e-15  7.9e-15
Displacement field
Iter      SolEst     Damping    Stepsize #Res #Jac #Sol   LinErr   LinRes
   1      0.0028   0.5000000      0.0028   24   12   12  9.6e-14  1.2e-12
Spatial mesh displacement
Iter      SolEst     Damping    Stepsize #Res #Jac #Sol   LinErr   LinRes
```

```
   1      2.6e-08   0.5000000     2.6e-08   24    12    12  1.6e-15  1.8e-15
Solution error estimates for segregated groups
3.8e-05, 0.00013, 0.0014, 1.3e-08
Residual error estimates for segregated groups
0.013, 16, 1.2e+03, 0.18
Segregated solver iteration 13.
Merged variables
Iter      SolEst      Damping    Stepsize #Res #Jac #Sol   LinErr   LinRes
   1      1.4e-05   0.3500000     1.4e-05   50    37    37  5.1e-14  2.9e-15
   2      9.3e-06   0.3500000     9.3e-06   51    38    38  7.6e-14  3.1e-15
   3      6.1e-06   0.3500000     6.1e-06   52    39    39  6.1e-14  2.8e-15
Velocity u2, Pressure p
Iter      SolEst      Damping    Stepsize #Res #Jac #Sol   LinErr   LinRes
   1      0.00013   0.5000000     0.00013   26    13    13  2.6e-15  7.8e-15
Displacement field
Iter      SolEst      Damping    Stepsize #Res #Jac #Sol   LinErr   LinRes
   1       0.0015   0.5000000      0.0015   26    13    13  1.2e-13  1.2e-12
Spatial mesh displacement
Iter      SolEst      Damping    Stepsize #Res #Jac #Sol   LinErr   LinRes
   1      1.5e-08   0.5000000     1.5e-08   26    13    13  1.5e-15  1.8e-15
Solution error estimates for segregated groups
1e-05, 6.6e-05, 0.00076, 7.5e-09
Residual error estimates for segregated groups
0.004, 8.2, 1.2e+03, 0.17
Solution time: 155 s. (2 minutes, 35 seconds)
Physical memory: 4.66 GB
Virtual memory: 25.51 GB
Ended at Jan 31, 2025, 7:12:05 PM.
----- Stationary Solver 1 in study1/Approach Curve Solver (sol1) -------------->
```

d=2245 (su53)

GENERAL

Description	Value
Solution	d=2245

LOG

```
<---- Compile Equations: Stationary in study1/Approach Curve Solver (sol1) -----
Started at Jan 31, 2025, 7:12:05 PM.
Parameter d = 2.245E-6 (m).
Geometry shape function: Linear serendipity
Number of vertex elements: 18
Number of boundary elements: 6202
Number of elements: 155450
Minimum element quality: 0.4905
Time: 8 s.
Physical memory: 2.96 GB
Virtual memory: 15.29 GB
Ended at Jan 31, 2025, 7:12:13 PM.
----- Compile Equations: Stationary in study1/Approach Curve Solver (sol1) ---->

<---- Dependent Variables 1 in study1/Approach Curve Solver (sol1) -------------
Started at Jan 31, 2025, 7:12:13 PM.
Solution time: 0 s.
Physical memory: 2.96 GB
Virtual memory: 15.23 GB
Ended at Jan 31, 2025, 7:12:13 PM.
----- Dependent Variables 1 in study1/Approach Curve Solver (sol1) ------------>

<---- Stationary Solver 1 in study1/Approach Curve Solver (sol1) ---------------
Started at Jan 31, 2025, 7:12:13 PM.
Segregated solver
Segregated Step 11 does not have any components solved for.
Number of degrees of freedom solved for: 789979 (plus 11827 internal DOFs).
Segregated solver iteration 1.
Merged variables
Nonsymmetric matrix found.
Scales for dependent variables:
Concentration (comp1.Cl): 1.4e+02
Concentration (comp1.K): 1.4e+02
Electric potential (comp1.V): 1.9e+02
Orthonormal null-space function used.
Iter      SolEst     Damping    Stepsize #Res #Jac #Sol   LinErr   LinRes
   1        0.83   0.3500000        0.83    2    1    1    3e-11  2.7e-15
   2        0.81   0.3500000        0.81    3    2    2  2.2e-11  2.7e-15
   3         0.8   0.3500000         0.8    4    3    3  1.5e-11  2.7e-15
Velocity u2, Pressure p
Nonsymmetric matrix found.
Scales for dependent variables:
Pressure (comp1.p): 1
Velocity field (spatial frame) (comp1.u2): 0.00018
Orthonormal null-space function used.
Iter      SolEst     Damping    Stepsize #Res #Jac #Sol   LinErr   LinRes
   1         1.1   0.5000000         1.1    2    1    1  1.7e-15  2.3e-15
Displacement field
Nonsymmetric matrix found.
Scales for dependent variables:
Auxiliary pressure (comp1.solid.lemm1.pw): 1
Displacement field (comp1.u): 2.8e-18
Orthonormal null-space function used.
Iter      SolEst     Damping    Stepsize #Res #Jac #Sol   LinErr   LinRes
   1         1.9   0.5000000         1.9    2    1    1  4.2e-14  1.5e-12
Spatial mesh displacement
```

```
Symmetric matrices found.
Scales for dependent variables:
Spatial mesh displacement (comp1.spatial.disp): 1.2e-09
Orthonormal null-space function used.
Iter      SolEst     Damping    Stepsize #Res #Jac #Sol   LinErr   LinRes
   1     6.7e-07   0.5000000     6.7e-07    2    1    1  1.4e-15  1.9e-15
Solution error estimates for segregated groups
1.4, 0.54, 0.95, 3.4e-07
Residual error estimates for segregated groups
7.9e+03, 8.2e+04, 2.2e+04, 0.14
Segregated solver iteration 2.
Merged variables
Scales for dependent variables:
Concentration (comp1.Cl): 1.4e+02
Concentration (comp1.K): 1.4e+02
Electric potential (comp1.V): 0.064
Iter      SolEst     Damping    Stepsize #Res #Jac #Sol   LinErr   LinRes
   1        0.78   0.3500000        0.78    6    4    4  9.4e-14  2.7e-15
   2        0.77   0.3500000        0.77    7    5    5  6.3e-14  2.9e-15
   3        0.76   0.3500000        0.76    8    6    6  8.5e-14  2.7e-15
Velocity u2, Pressure p
Scales for dependent variables:
Pressure (comp1.p): 3.6e+02
Velocity field (spatial frame) (comp1.u2): 0.00072
Iter      SolEst     Damping    Stepsize #Res #Jac #Sol   LinErr   LinRes
   1        0.36   0.5000000        0.36    4    2    2  2.9e-15  7.8e-15
Displacement field
Iter      SolEst     Damping    Stepsize #Res #Jac #Sol   LinErr   LinRes
   1        0.95   0.5000000        0.95    4    2    2  8.4e-14  1.5e-12
Spatial mesh displacement
Iter      SolEst     Damping    Stepsize #Res #Jac #Sol   LinErr   LinRes
   1       1e-06   0.5000000       1e-06    4    2    2  1.6e-15  1.8e-15
Solution error estimates for segregated groups
1.3, 0.18, 0.48, 5e-07
Residual error estimates for segregated groups
7.9e+02, 1.3e+04, 2.2e+04, 0.16
Segregated solver iteration 3.
Merged variables
Iter      SolEst     Damping    Stepsize #Res #Jac #Sol   LinErr   LinRes
   1        0.75   0.3500000        0.75   10    7    7  7.6e-14  2.6e-15
   2        0.73   0.3500000        0.73   11    8    8  4.2e-14  2.7e-15
   3        0.72   0.3500000        0.72   12    9    9    1e-13  2.7e-15
Velocity u2, Pressure p
Iter      SolEst     Damping    Stepsize #Res #Jac #Sol   LinErr   LinRes
   1        0.15   0.5000000        0.15    6    3    3  1.8e-15  7.9e-15
Displacement field
Iter      SolEst     Damping    Stepsize #Res #Jac #Sol   LinErr   LinRes
   1        0.52   0.5000000        0.52    6    3    3  3.8e-14  1.5e-12
Spatial mesh displacement
Iter      SolEst     Damping    Stepsize #Res #Jac #Sol   LinErr   LinRes
   1       1e-06   0.5000000       1e-06    6    3    3  1.3e-15  1.9e-15
Solution error estimates for segregated groups
1.2, 0.077, 0.26, 5e-07
Residual error estimates for segregated groups
2.2e+02, 7.4e+03, 1.6e+04, 0.16
Segregated solver iteration 4.
```

```
Merged variables
Iter      SolEst     Damping    Stepsize #Res #Jac #Sol   LinErr   LinRes
   1         0.7   0.3500000         0.7   14   10   10  1.3e-13  2.7e-15
   2        0.68   0.3500000        0.68   15   11   11  7.2e-14  2.8e-15
   3        0.59   0.3500000        0.59   16   12   12  5.6e-14  2.6e-15
Velocity u2, Pressure p
Iter      SolEst     Damping    Stepsize #Res #Jac #Sol   LinErr   LinRes
   1       0.072   0.5000000       0.072    8    4    4  4.7e-15  7.8e-15
Displacement field
Iter      SolEst     Damping    Stepsize #Res #Jac #Sol   LinErr   LinRes
   1        0.29   0.5000000        0.29    8    4    4  4.3e-14  1.5e-12
Spatial mesh displacement
Iter      SolEst     Damping    Stepsize #Res #Jac #Sol   LinErr   LinRes
   1     8.4e-07   0.5000000     8.4e-07    8    4    4  1.4e-15  1.8e-15
Solution error estimates for segregated groups
1, 0.036, 0.15, 4.2e-07
Residual error estimates for segregated groups
84, 3.9e+03, 1.1e+04, 0.17
Segregated solver iteration 5.
Merged variables
Iter      SolEst     Damping    Stepsize #Res #Jac #Sol   LinErr   LinRes
   1        0.41   0.3500000        0.41   18   13   13  1.1e-13  2.7e-15
   2        0.27   0.3500000        0.27   19   14   14  4.8e-14  2.6e-15
   3        0.18   0.3500000        0.18   20   15   15  8.9e-14  2.8e-15
Velocity u2, Pressure p
Iter      SolEst     Damping    Stepsize #Res #Jac #Sol   LinErr   LinRes
   1       0.035   0.5000000       0.035   10    5    5  3.1e-15  7.7e-15
Displacement field
Iter      SolEst     Damping    Stepsize #Res #Jac #Sol   LinErr   LinRes
   1        0.17   0.5000000        0.17   10    5    5  1.6e-13  1.5e-12
Spatial mesh displacement
Iter      SolEst     Damping    Stepsize #Res #Jac #Sol   LinErr   LinRes
   1     6.3e-07   0.5000000     6.3e-07   10    5    5  1.3e-15  1.8e-15
Solution error estimates for segregated groups
0.31, 0.017, 0.084, 3.2e-07
Residual error estimates for segregated groups
33, 2e+03, 6.8e+03, 0.16
Segregated solver iteration 6.
Merged variables
Iter      SolEst     Damping    Stepsize #Res #Jac #Sol   LinErr   LinRes
   1        0.12   0.3500000        0.12   22   16   16  6.1e-14  2.8e-15
   2       0.078   0.3500000       0.078   23   17   17  9.7e-14  2.6e-15
   3       0.051   0.3500000       0.051   24   18   18  8.9e-14  2.7e-15
Velocity u2, Pressure p
Iter      SolEst     Damping    Stepsize #Res #Jac #Sol   LinErr   LinRes
   1       0.017   0.5000000       0.017   12    6    6    3e-15  7.9e-15
Displacement field
Iter      SolEst     Damping    Stepsize #Res #Jac #Sol   LinErr   LinRes
   1       0.095   0.5000000       0.095   12    6    6  3.6e-14  1.5e-12
Spatial mesh displacement
Iter      SolEst     Damping    Stepsize #Res #Jac #Sol   LinErr   LinRes
   1     4.4e-07   0.5000000     4.4e-07   12    6    6  1.5e-15  1.9e-15
Solution error estimates for segregated groups
0.087, 0.0085, 0.048, 2.2e-07
Residual error estimates for segregated groups
12, 1e+03, 4.2e+03, 0.16
```

```
Segregated solver iteration 7.
Merged variables
Iter      SolEst     Damping    Stepsize #Res #Jac #Sol   LinErr   LinRes
   1       0.033   0.3500000       0.033   26   19   19  1.2e-13  2.9e-15
   2       0.022   0.3500000       0.022   27   20   20  1.4e-13  2.6e-15
   3       0.014   0.3500000       0.014   28   21   21  7.6e-14  2.7e-15
Velocity u2, Pressure p
Iter      SolEst     Damping    Stepsize #Res #Jac #Sol   LinErr   LinRes
   1      0.0085   0.5000000      0.0085   14    7    7  2.7e-15  7.8e-15
Displacement field
Iter      SolEst     Damping    Stepsize #Res #Jac #Sol   LinErr   LinRes
   1       0.054   0.5000000       0.054   14    7    7  5.5e-14  1.5e-12
Spatial mesh displacement
Iter      SolEst     Damping    Stepsize #Res #Jac #Sol   LinErr   LinRes
   1     2.9e-07   0.5000000     2.9e-07   14    7    7  1.5e-15  1.9e-15
Solution error estimates for segregated groups
0.024, 0.0042, 0.027, 1.5e-07
Residual error estimates for segregated groups
4.1, 5.1e+02, 2.6e+03, 0.15
Segregated solver iteration 8.
Merged variables
Iter      SolEst     Damping    Stepsize #Res #Jac #Sol   LinErr   LinRes
   1      0.0091   0.3500000      0.0091   30   22   22  7.6e-14  2.7e-15
   2      0.0059   0.3500000      0.0059   31   23   23  4.9e-14  2.6e-15
   3      0.0039   0.3500000      0.0039   32   24   24  1.2e-13  2.7e-15
Velocity u2, Pressure p
Iter      SolEst     Damping    Stepsize #Res #Jac #Sol   LinErr   LinRes
   1      0.0042   0.5000000      0.0042   16    8    8  2.6e-15  7.9e-15
Displacement field
Iter      SolEst     Damping    Stepsize #Res #Jac #Sol   LinErr   LinRes
   1        0.03   0.5000000        0.03   16    8    8  1.2e-13  1.5e-12
Spatial mesh displacement
Iter      SolEst     Damping    Stepsize #Res #Jac #Sol   LinErr   LinRes
   1     1.9e-07   0.5000000     1.9e-07   16    8    8  1.6e-15  1.8e-15
Solution error estimates for segregated groups
0.0066, 0.0021, 0.015, 9.5e-08
Residual error estimates for segregated groups
1.4, 2.6e+02, 1.8e+03, 0.16
Segregated solver iteration 9.
Merged variables
Iter      SolEst     Damping    Stepsize #Res #Jac #Sol   LinErr   LinRes
   1      0.0025   0.3500000      0.0025   34   25   25  1.1e-13  2.8e-15
   2      0.0016   0.3500000      0.0016   35   26   26    4e-14  2.9e-15
   3      0.0011   0.3500000      0.0011   36   27   27  7.9e-14  2.7e-15
Velocity u2, Pressure p
Iter      SolEst     Damping    Stepsize #Res #Jac #Sol   LinErr   LinRes
   1      0.0021   0.5000000      0.0021   18    9    9  3.6e-15  7.9e-15
Displacement field
Iter      SolEst     Damping    Stepsize #Res #Jac #Sol   LinErr   LinRes
   1       0.017   0.5000000       0.017   18    9    9  7.7e-14  1.5e-12
Spatial mesh displacement
Iter      SolEst     Damping    Stepsize #Res #Jac #Sol   LinErr   LinRes
   1     1.2e-07   0.5000000     1.2e-07   18    9    9  1.5e-15  1.9e-15
Solution error estimates for segregated groups
0.0018, 0.0011, 0.0085, 5.9e-08
Residual error estimates for segregated groups
```

```
0.44, 1.3e+02, 1.4e+03, 0.16
Segregated solver iteration 10.
Merged variables
Iter      SolEst     Damping    Stepsize #Res #Jac #Sol   LinErr   LinRes
   1     0.00069   0.3500000     0.00069   38   28   28    5e-14  2.6e-15
   2     0.00045   0.3500000     0.00045   39   29   29  5.8e-14  2.7e-15
   3     0.00029   0.3500000     0.00029   40   30   30  8.6e-14  2.7e-15
Velocity u2, Pressure p
Iter      SolEst     Damping    Stepsize #Res #Jac #Sol   LinErr   LinRes
   1      0.0011   0.5000000      0.0011   20   10   10  3.8e-15  7.8e-15
Displacement field
Scales for dependent variables:
Auxiliary pressure (comp1.solid.lemm1.pw): 0.35
Displacement field (comp1.u): 2.8e-16
Iter      SolEst     Damping    Stepsize #Res #Jac #Sol   LinErr   LinRes
   1      0.0094   0.5000000      0.0094   20   10   10  2.1e-13  1.2e-12
Spatial mesh displacement
Iter      SolEst     Damping    Stepsize #Res #Jac #Sol   LinErr   LinRes
   1     7.2e-08   0.5000000     7.2e-08   20   10   10  1.6e-15  1.9e-15
Solution error estimates for segregated groups
0.0005, 0.00053, 0.0047, 3.6e-08
Residual error estimates for segregated groups
0.14, 64, 1.2e+03, 0.17
Segregated solver iteration 11.
Merged variables
Iter      SolEst     Damping    Stepsize #Res #Jac #Sol   LinErr   LinRes
   1     0.00019   0.3500000     0.00019   42   31   31  7.3e-14  2.7e-15
   2     0.00012   0.3500000     0.00012   43   32   32  5.2e-14  2.7e-15
   3       8e-05   0.3500000       8e-05   44   33   33  5.3e-14  2.6e-15
Velocity u2, Pressure p
Iter      SolEst     Damping    Stepsize #Res #Jac #Sol   LinErr   LinRes
   1     0.00052   0.5000000     0.00052   22   11   11  3.7e-15  7.9e-15
Displacement field
Iter      SolEst     Damping    Stepsize #Res #Jac #Sol   LinErr   LinRes
   1      0.0051   0.5000000      0.0051   22   11   11    1e-13  1.2e-12
Spatial mesh displacement
Iter      SolEst     Damping    Stepsize #Res #Jac #Sol   LinErr   LinRes
   1     4.3e-08   0.5000000     4.3e-08   22   11   11  1.9e-15  1.9e-15
Solution error estimates for segregated groups
0.00014, 0.00026, 0.0026, 2.2e-08
Residual error estimates for segregated groups
0.043, 32, 1.2e+03, 0.16
Segregated solver iteration 12.
Merged variables
Iter      SolEst     Damping    Stepsize #Res #Jac #Sol   LinErr   LinRes
   1     5.2e-05   0.3500000     5.2e-05   46   34   34  7.3e-14  2.7e-15
   2     3.4e-05   0.3500000     3.4e-05   47   35   35  5.9e-14  2.7e-15
   3     2.2e-05   0.3500000     2.2e-05   48   36   36  8.2e-14  2.6e-15
Velocity u2, Pressure p
Iter      SolEst     Damping    Stepsize #Res #Jac #Sol   LinErr   LinRes
   1     0.00026   0.5000000     0.00026   24   12   12  3.7e-15  7.8e-15
Displacement field
Iter      SolEst     Damping    Stepsize #Res #Jac #Sol   LinErr   LinRes
   1      0.0028   0.5000000      0.0028   24   12   12  1.2e-13  1.2e-12
Spatial mesh displacement
Iter      SolEst     Damping    Stepsize #Res #Jac #Sol   LinErr   LinRes
```

```
   1     2.6e-08   0.5000000     2.6e-08   24   12   12  1.3e-15  1.8e-15
Solution error estimates for segregated groups
3.8e-05, 0.00013, 0.0014, 1.3e-08
Residual error estimates for segregated groups
0.013, 16, 1.2e+03, 0.17
Segregated solver iteration 13.
Merged variables
Iter      SolEst     Damping    Stepsize #Res #Jac #Sol   LinErr   LinRes
   1     1.4e-05   0.3500000     1.4e-05   50   37   37  8.9e-14  2.8e-15
   2     9.3e-06   0.3500000     9.3e-06   51   38   38  7.5e-14  2.7e-15
   3       6e-06   0.3500000       6e-06   52   39   39  5.3e-14  2.8e-15
Velocity u2, Pressure p
Iter      SolEst     Damping    Stepsize #Res #Jac #Sol   LinErr   LinRes
   1     0.00013   0.5000000     0.00013   26   13   13  3.9e-15  7.7e-15
Displacement field
Iter      SolEst     Damping    Stepsize #Res #Jac #Sol   LinErr   LinRes
   1      0.0015   0.5000000      0.0015   26   13   13  1.1e-13  1.2e-12
Spatial mesh displacement
Iter      SolEst     Damping    Stepsize #Res #Jac #Sol   LinErr   LinRes
   1     1.5e-08   0.5000000     1.5e-08   26   13   13  1.4e-15  1.8e-15
Solution error estimates for segregated groups
1e-05, 6.6e-05, 0.00076, 7.5e-09
Residual error estimates for segregated groups
0.004, 8, 1.2e+03, 0.17
Solution time: 155 s. (2 minutes, 35 seconds)
Physical memory: 4.73 GB
Virtual memory: 25.55 GB
Ended at Jan 31, 2025, 7:14:48 PM.
----- Stationary Solver 1 in study1/Approach Curve Solver (sol1) -------------->
```

d=2240 (su54)

GENERAL

Description	Value
Solution	d=2240

LOG

```
<---- Compile Equations: Stationary in study1/Approach Curve Solver (sol1) -----
Started at Jan 31, 2025, 7:14:48 PM.
Parameter d = 2.24E-6 (m).
Geometry shape function: Linear serendipity
Number of vertex elements: 18
Number of boundary elements: 6199
Number of elements: 155665
Minimum element quality: 0.4774
Time: 8 s.
Physical memory: 2.96 GB
Virtual memory: 15.28 GB
Ended at Jan 31, 2025, 7:14:57 PM.
----- Compile Equations: Stationary in study1/Approach Curve Solver (sol1) ---->

<---- Dependent Variables 1 in study1/Approach Curve Solver (sol1) -------------
Started at Jan 31, 2025, 7:14:57 PM.
Solution time: 0 s.
Physical memory: 2.96 GB
Virtual memory: 15.22 GB
Ended at Jan 31, 2025, 7:14:57 PM.
----- Dependent Variables 1 in study1/Approach Curve Solver (sol1) ------------>

<---- Stationary Solver 1 in study1/Approach Curve Solver (sol1) ---------------
Started at Jan 31, 2025, 7:14:57 PM.
Segregated solver
Segregated Step 11 does not have any components solved for.
Number of degrees of freedom solved for: 791000 (plus 11821 internal DOFs).
Segregated solver iteration 1.
Merged variables
Nonsymmetric matrix found.
Scales for dependent variables:
Concentration (comp1.Cl): 1.4e+02
Concentration (comp1.K): 1.4e+02
Electric potential (comp1.V): 1.9e+02
Orthonormal null-space function used.
Iter      SolEst     Damping    Stepsize #Res #Jac #Sol   LinErr   LinRes
   1        0.83   0.3500000        0.83    2    1    1  1.7e-11  2.8e-15
   2        0.81   0.3500000        0.81    3    2    2  1.9e-11  2.7e-15
   3         0.8   0.3500000         0.8    4    3    3  2.2e-11  2.7e-15
Velocity u2, Pressure p
Nonsymmetric matrix found.
Scales for dependent variables:
Pressure (comp1.p): 1
Velocity field (spatial frame) (comp1.u2): 0.00018
Orthonormal null-space function used.
Iter      SolEst     Damping    Stepsize #Res #Jac #Sol   LinErr   LinRes
   1         1.1   0.5000000         1.1    2    1    1  1.7e-15  2.3e-15
Displacement field
Nonsymmetric matrix found.
Scales for dependent variables:
Auxiliary pressure (comp1.solid.lemm1.pw): 1
Displacement field (comp1.u): 2.8e-18
Orthonormal null-space function used.
Iter      SolEst     Damping    Stepsize #Res #Jac #Sol   LinErr   LinRes
   1         1.9   0.5000000         1.9    2    1    1  9.8e-14  1.5e-12
Spatial mesh displacement
```

```
Symmetric matrices found.
Scales for dependent variables:
Spatial mesh displacement (comp1.spatial.disp): 1.2e-09
Orthonormal null-space function used.
Iter      SolEst     Damping    Stepsize #Res #Jac #Sol   LinErr   LinRes
   1     6.8e-07   0.5000000     6.8e-07    2    1    1  1.3e-15  1.9e-15
Solution error estimates for segregated groups
1.4, 0.54, 0.95, 3.4e-07
Residual error estimates for segregated groups
7.9e+03, 8.3e+04, 2.2e+04, 0.14
Segregated solver iteration 2.
Merged variables
Scales for dependent variables:
Concentration (comp1.Cl): 1.4e+02
Concentration (comp1.K): 1.4e+02
Electric potential (comp1.V): 0.064
Iter      SolEst     Damping    Stepsize #Res #Jac #Sol   LinErr   LinRes
   1        0.78   0.3500000        0.78    6    4    4  1.1e-13  4.4e-15
   2        0.77   0.3500000        0.77    7    5    5  1.4e-13  3.6e-15
   3        0.76   0.3500000        0.76    8    6    6    1e-13  3.2e-15
Velocity u2, Pressure p
Scales for dependent variables:
Pressure (comp1.p): 3.6e+02
Velocity field (spatial frame) (comp1.u2): 0.00072
Iter      SolEst     Damping    Stepsize #Res #Jac #Sol   LinErr   LinRes
   1        0.36   0.5000000        0.36    4    2    2    3e-15  7.9e-15
Displacement field
Iter      SolEst     Damping    Stepsize #Res #Jac #Sol   LinErr   LinRes
   1        0.95   0.5000000        0.95    4    2    2  1.9e-13  1.5e-12
Spatial mesh displacement
Iter      SolEst     Damping    Stepsize #Res #Jac #Sol   LinErr   LinRes
   1       1e-06   0.5000000       1e-06    4    2    2  1.4e-15  1.8e-15
Solution error estimates for segregated groups
1.3, 0.18, 0.48, 5.1e-07
Residual error estimates for segregated groups
7.9e+02, 1.5e+04, 2.2e+04, 0.16
Segregated solver iteration 3.
Merged variables
Iter      SolEst     Damping    Stepsize #Res #Jac #Sol   LinErr   LinRes
   1        0.75   0.3500000        0.75   10    7    7  1.1e-13  3.9e-15
   2        0.73   0.3500000        0.73   11    8    8  1.2e-13  3.6e-15
   3        0.72   0.3500000        0.72   12    9    9  7.4e-14  3.7e-15
Velocity u2, Pressure p
Iter      SolEst     Damping    Stepsize #Res #Jac #Sol   LinErr   LinRes
   1        0.15   0.5000000        0.15    6    3    3  2.7e-15  7.9e-15
Displacement field
Iter      SolEst     Damping    Stepsize #Res #Jac #Sol   LinErr   LinRes
   1        0.52   0.5000000        0.52    6    3    3  7.9e-14  1.5e-12
Spatial mesh displacement
Iter      SolEst     Damping    Stepsize #Res #Jac #Sol   LinErr   LinRes
   1       1e-06   0.5000000       1e-06    6    3    3  1.4e-15  1.8e-15
Solution error estimates for segregated groups
1.2, 0.077, 0.26, 5.1e-07
Residual error estimates for segregated groups
2.2e+02, 8.6e+03, 1.6e+04, 0.16
Segregated solver iteration 4.
```

```
Merged variables
Iter      SolEst     Damping    Stepsize #Res #Jac #Sol   LinErr   LinRes
   1         0.7   0.3500000         0.7   14   10   10  1.4e-13  4.4e-15
   2        0.68   0.3500000        0.68   15   11   11  1.8e-13  3.1e-15
   3         0.6   0.3500000         0.6   16   12   12  9.4e-14  4.1e-15
Velocity u2, Pressure p
Iter      SolEst     Damping    Stepsize #Res #Jac #Sol   LinErr   LinRes
   1       0.072   0.5000000       0.072    8    4    4  3.7e-15  7.9e-15
Displacement field
Iter      SolEst     Damping    Stepsize #Res #Jac #Sol   LinErr   LinRes
   1        0.29   0.5000000        0.29    8    4    4  5.3e-14  1.5e-12
Spatial mesh displacement
Iter      SolEst     Damping    Stepsize #Res #Jac #Sol   LinErr   LinRes
   1     8.4e-07   0.5000000     8.4e-07    8    4    4  1.7e-15  1.8e-15
Solution error estimates for segregated groups
1, 0.036, 0.15, 4.2e-07
Residual error estimates for segregated groups
83, 4.6e+03, 1.1e+04, 0.16
Segregated solver iteration 5.
Merged variables
Iter      SolEst     Damping    Stepsize #Res #Jac #Sol   LinErr   LinRes
   1        0.41   0.3500000        0.41   18   13   13  7.1e-14  3.5e-15
   2        0.27   0.3500000        0.27   19   14   14  2.5e-13    4e-15
   3        0.18   0.3500000        0.18   20   15   15  9.8e-14  4.1e-15
Velocity u2, Pressure p
Iter      SolEst     Damping    Stepsize #Res #Jac #Sol   LinErr   LinRes
   1       0.035   0.5000000       0.035   10    5    5  4.4e-15  7.9e-15
Displacement field
Iter      SolEst     Damping    Stepsize #Res #Jac #Sol   LinErr   LinRes
   1        0.17   0.5000000        0.17   10    5    5  2.2e-14  1.5e-12
Spatial mesh displacement
Iter      SolEst     Damping    Stepsize #Res #Jac #Sol   LinErr   LinRes
   1     6.3e-07   0.5000000     6.3e-07   10    5    5  1.6e-15  1.9e-15
Solution error estimates for segregated groups
0.31, 0.017, 0.084, 3.2e-07
Residual error estimates for segregated groups
33, 2.3e+03, 6.9e+03, 0.16
Segregated solver iteration 6.
Merged variables
Iter      SolEst     Damping    Stepsize #Res #Jac #Sol   LinErr   LinRes
   1        0.12   0.3500000        0.12   22   16   16  9.4e-14  3.9e-15
   2       0.078   0.3500000       0.078   23   17   17  1.3e-13  3.8e-15
   3       0.051   0.3500000       0.051   24   18   18  8.6e-14  3.5e-15
Velocity u2, Pressure p
Iter      SolEst     Damping    Stepsize #Res #Jac #Sol   LinErr   LinRes
   1       0.017   0.5000000       0.017   12    6    6  4.2e-15  7.8e-15
Displacement field
Iter      SolEst     Damping    Stepsize #Res #Jac #Sol   LinErr   LinRes
   1       0.095   0.5000000       0.095   12    6    6  8.9e-14  1.5e-12
Spatial mesh displacement
Iter      SolEst     Damping    Stepsize #Res #Jac #Sol   LinErr   LinRes
   1     4.4e-07   0.5000000     4.4e-07   12    6    6  1.7e-15  1.8e-15
Solution error estimates for segregated groups
0.088, 0.0085, 0.048, 2.2e-07
Residual error estimates for segregated groups
12, 1.2e+03, 4.2e+03, 0.17
```

```
Segregated solver iteration 7.
Merged variables
Iter      SolEst     Damping    Stepsize #Res #Jac #Sol   LinErr   LinRes
   1       0.033   0.3500000       0.033   26   19   19  6.2e-14  3.5e-15
   2       0.022   0.3500000       0.022   27   20   20  1.6e-13  3.6e-15
   3       0.014   0.3500000       0.014   28   21   21  4.4e-14  3.8e-15
Velocity u2, Pressure p
Iter      SolEst     Damping    Stepsize #Res #Jac #Sol   LinErr   LinRes
   1      0.0085   0.5000000      0.0085   14    7    7  2.2e-15  7.7e-15
Displacement field
Iter      SolEst     Damping    Stepsize #Res #Jac #Sol   LinErr   LinRes
   1       0.054   0.5000000       0.054   14    7    7  8.9e-14  1.5e-12
Spatial mesh displacement
Iter      SolEst     Damping    Stepsize #Res #Jac #Sol   LinErr   LinRes
   1       3e-07   0.5000000       3e-07   14    7    7  1.5e-15  1.9e-15
Solution error estimates for segregated groups
0.024, 0.0042, 0.027, 1.5e-07
Residual error estimates for segregated groups
4.1, 6e+02, 2.6e+03, 0.15
Segregated solver iteration 8.
Merged variables
Iter      SolEst     Damping    Stepsize #Res #Jac #Sol   LinErr   LinRes
   1      0.0092   0.3500000      0.0092   30   22   22  1.4e-13    4e-15
   2       0.006   0.3500000       0.006   31   23   23  1.2e-13  3.5e-15
   3      0.0039   0.3500000      0.0039   32   24   24  1.2e-13  3.5e-15
Velocity u2, Pressure p
Iter      SolEst     Damping    Stepsize #Res #Jac #Sol   LinErr   LinRes
   1      0.0042   0.5000000      0.0042   16    8    8  3.9e-15  7.9e-15
Displacement field
Iter      SolEst     Damping    Stepsize #Res #Jac #Sol   LinErr   LinRes
   1        0.03   0.5000000        0.03   16    8    8  4.9e-14  1.5e-12
Spatial mesh displacement
Iter      SolEst     Damping    Stepsize #Res #Jac #Sol   LinErr   LinRes
   1     1.9e-07   0.5000000     1.9e-07   16    8    8  1.6e-15  1.8e-15
Solution error estimates for segregated groups
0.0066, 0.0021, 0.015, 9.5e-08
Residual error estimates for segregated groups
1.4, 3e+02, 1.8e+03, 0.16
Segregated solver iteration 9.
Merged variables
Iter      SolEst     Damping    Stepsize #Res #Jac #Sol   LinErr   LinRes
   1      0.0025   0.3500000      0.0025   34   25   25  6.5e-14  3.2e-15
   2      0.0016   0.3500000      0.0016   35   26   26  8.9e-14  3.5e-15
   3      0.0011   0.3500000      0.0011   36   27   27  1.4e-13  3.1e-15
Velocity u2, Pressure p
Iter      SolEst     Damping    Stepsize #Res #Jac #Sol   LinErr   LinRes
   1      0.0021   0.5000000      0.0021   18    9    9  3.3e-15  7.9e-15
Displacement field
Scales for dependent variables:
Auxiliary pressure (comp1.solid.lemm1.pw): 0.35
Displacement field (comp1.u): 2.8e-16
Iter      SolEst     Damping    Stepsize #Res #Jac #Sol   LinErr   LinRes
   1       0.017   0.5000000       0.017   18    9    9  1.2e-13  1.2e-12
Spatial mesh displacement
Iter      SolEst     Damping    Stepsize #Res #Jac #Sol   LinErr   LinRes
   1     1.2e-07   0.5000000     1.2e-07   18    9    9  1.5e-15  1.8e-15
```

```
Solution error estimates for segregated groups
0.0018, 0.0011, 0.0085, 5.9e-08
Residual error estimates for segregated groups
0.44, 1.5e+02, 1.4e+03, 0.16
Segregated solver iteration 10.
Merged variables
Iter      SolEst     Damping    Stepsize #Res #Jac #Sol   LinErr   LinRes
   1     0.00069   0.3500000     0.00069   38   28   28  4.3e-14  4.1e-15
   2     0.00045   0.3500000     0.00045   39   29   29  1.6e-13  4.8e-15
   3     0.00029   0.3500000     0.00029   40   30   30    1e-13    3e-15
Velocity u2, Pressure p
Iter      SolEst     Damping    Stepsize #Res #Jac #Sol   LinErr   LinRes
   1       0.001   0.5000000       0.001   20   10   10  2.8e-15  7.9e-15
Displacement field
Iter      SolEst     Damping    Stepsize #Res #Jac #Sol   LinErr   LinRes
   1      0.0094   0.5000000      0.0094   20   10   10    1e-13  1.2e-12
Spatial mesh displacement
Iter      SolEst     Damping    Stepsize #Res #Jac #Sol   LinErr   LinRes
   1     7.3e-08   0.5000000     7.3e-08   20   10   10  1.4e-15  1.8e-15
Solution error estimates for segregated groups
0.0005, 0.00052, 0.0047, 3.6e-08
Residual error estimates for segregated groups
0.14, 75, 1.2e+03, 0.15
Segregated solver iteration 11.
Merged variables
Iter      SolEst     Damping    Stepsize #Res #Jac #Sol   LinErr   LinRes
   1     0.00019   0.3500000     0.00019   42   31   31  1.4e-13  3.4e-15
   2     0.00012   0.3500000     0.00012   43   32   32  1.1e-13  3.5e-15
   3       8e-05   0.3500000       8e-05   44   33   33  1.8e-13    4e-15
Velocity u2, Pressure p
Iter      SolEst     Damping    Stepsize #Res #Jac #Sol   LinErr   LinRes
   1     0.00052   0.5000000     0.00052   22   11   11  3.3e-15  7.8e-15
Displacement field
Iter      SolEst     Damping    Stepsize #Res #Jac #Sol   LinErr   LinRes
   1      0.0051   0.5000000      0.0051   22   11   11  1.6e-13  1.2e-12
Spatial mesh displacement
Iter      SolEst     Damping    Stepsize #Res #Jac #Sol   LinErr   LinRes
   1     4.4e-08   0.5000000     4.4e-08   22   11   11  1.7e-15  1.9e-15
Solution error estimates for segregated groups
0.00014, 0.00026, 0.0026, 2.2e-08
Residual error estimates for segregated groups
0.043, 38, 1.2e+03, 0.17
Segregated solver iteration 12.
Merged variables
Iter      SolEst     Damping    Stepsize #Res #Jac #Sol   LinErr   LinRes
   1     5.2e-05   0.3500000     5.2e-05   46   34   34  1.4e-13  4.2e-15
   2     3.4e-05   0.3500000     3.4e-05   47   35   35    1e-13  3.9e-15
   3     2.2e-05   0.3500000     2.2e-05   48   36   36  1.6e-13  4.7e-15
Velocity u2, Pressure p
Iter      SolEst     Damping    Stepsize #Res #Jac #Sol   LinErr   LinRes
   1     0.00026   0.5000000     0.00026   24   12   12  2.7e-15  7.9e-15
Displacement field
Iter      SolEst     Damping    Stepsize #Res #Jac #Sol   LinErr   LinRes
   1      0.0028   0.5000000      0.0028   24   12   12  1.7e-13  1.2e-12
Spatial mesh displacement
Iter      SolEst     Damping    Stepsize #Res #Jac #Sol   LinErr   LinRes
```

```
   1     2.6e-08   0.5000000     2.6e-08   24   12   12  2.3e-15  1.8e-15
Solution error estimates for segregated groups
3.8e-05, 0.00013, 0.0014, 1.3e-08
Residual error estimates for segregated groups
0.013, 19, 1.2e+03, 0.16
Segregated solver iteration 13.
Merged variables
Iter      SolEst     Damping    Stepsize #Res #Jac #Sol   LinErr   LinRes
   1     1.4e-05   0.3500000     1.4e-05   50   37   37  1.1e-13  3.1e-15
   2     9.3e-06   0.3500000     9.3e-06   51   38   38  1.5e-13  3.6e-15
   3     6.1e-06   0.3500000     6.1e-06   52   39   39  6.4e-14  3.4e-15
Velocity u2, Pressure p
Iter      SolEst     Damping    Stepsize #Res #Jac #Sol   LinErr   LinRes
   1     0.00013   0.5000000     0.00013   26   13   13  2.6e-15  7.9e-15
Displacement field
Iter      SolEst     Damping    Stepsize #Res #Jac #Sol   LinErr   LinRes
   1      0.0015   0.5000000      0.0015   26   13   13  1.2e-13  1.2e-12
Spatial mesh displacement
Iter      SolEst     Damping    Stepsize #Res #Jac #Sol   LinErr   LinRes
   1     1.5e-08   0.5000000     1.5e-08   26   13   13  1.3e-15  1.8e-15
Solution error estimates for segregated groups
1e-05, 6.6e-05, 0.00076, 7.5e-09
Residual error estimates for segregated groups
0.004, 9.4, 1.2e+03, 0.16
Solution time: 154 s. (2 minutes, 34 seconds)
Physical memory: 4.65 GB
Virtual memory: 25.87 GB
Ended at Jan 31, 2025, 7:17:31 PM.
----- Stationary Solver 1 in study1/Approach Curve Solver (sol1) -------------->
```

d=2235 (su55)

GENERAL

Description	Value
Solution	d=2235

LOG

```
<---- Compile Equations: Stationary in study1/Approach Curve Solver (sol1) -----
Started at Jan 31, 2025, 7:17:31 PM.
Parameter d = 2.235E-6 (m).
Geometry shape function: Linear serendipity
Number of vertex elements: 18
Number of boundary elements: 6197
Number of elements: 155461
Minimum element quality: 0.4726
Time: 8 s.
Physical memory: 2.94 GB
Virtual memory: 15.27 GB
Ended at Jan 31, 2025, 7:17:39 PM.
----- Compile Equations: Stationary in study1/Approach Curve Solver (sol1) ---->

<---- Dependent Variables 1 in study1/Approach Curve Solver (sol1) -------------
Started at Jan 31, 2025, 7:17:39 PM.
Solution time: 0 s.
Physical memory: 2.94 GB
Virtual memory: 15.2 GB
Ended at Jan 31, 2025, 7:17:40 PM.
----- Dependent Variables 1 in study1/Approach Curve Solver (sol1) ------------>

<---- Stationary Solver 1 in study1/Approach Curve Solver (sol1) ---------------
Started at Jan 31, 2025, 7:17:40 PM.
Segregated solver
Segregated Step 11 does not have any components solved for.
Number of degrees of freedom solved for: 789941 (plus 11817 internal DOFs).
Segregated solver iteration 1.
Merged variables
Nonsymmetric matrix found.
Scales for dependent variables:
Concentration (comp1.Cl): 1.4e+02
Concentration (comp1.K): 1.4e+02
Electric potential (comp1.V): 1.9e+02
Orthonormal null-space function used.
Iter      SolEst     Damping    Stepsize #Res #Jac #Sol   LinErr   LinRes
   1        0.83   0.3500000        0.83    2    1    1    2e-11  2.7e-15
   2        0.81   0.3500000        0.81    3    2    2  2.9e-11  2.8e-15
   3         0.8   0.3500000         0.8    4    3    3  3.4e-11  2.7e-15
Velocity u2, Pressure p
Nonsymmetric matrix found.
Scales for dependent variables:
Pressure (comp1.p): 1
Velocity field (spatial frame) (comp1.u2): 0.00018
Orthonormal null-space function used.
Iter      SolEst     Damping    Stepsize #Res #Jac #Sol   LinErr   LinRes
   1         1.1   0.5000000         1.1    2    1    1  1.7e-15  2.3e-15
Displacement field
Nonsymmetric matrix found.
Scales for dependent variables:
Auxiliary pressure (comp1.solid.lemm1.pw): 1
Displacement field (comp1.u): 2.8e-18
Orthonormal null-space function used.
Iter      SolEst     Damping    Stepsize #Res #Jac #Sol   LinErr   LinRes
   1         1.9   0.5000000         1.9    2    1    1  4.6e-14  1.5e-12
Spatial mesh displacement
```

```
Symmetric matrices found.
Scales for dependent variables:
Spatial mesh displacement (comp1.spatial.disp): 1.2e-09
Orthonormal null-space function used.
Iter      SolEst     Damping    Stepsize #Res #Jac #Sol   LinErr   LinRes
   1     6.8e-07   0.5000000     6.8e-07    2    1    1  1.5e-15  1.8e-15
Solution error estimates for segregated groups
1.4, 0.54, 0.95, 3.4e-07
Residual error estimates for segregated groups
7.9e+03, 8.2e+04, 2.2e+04, 0.14
Segregated solver iteration 2.
Merged variables
Scales for dependent variables:
Concentration (comp1.Cl): 1.4e+02
Concentration (comp1.K): 1.4e+02
Electric potential (comp1.V): 0.064
Iter      SolEst     Damping    Stepsize #Res #Jac #Sol   LinErr   LinRes
   1        0.78   0.3500000        0.78    6    4    4  9.6e-14  3.2e-15
   2        0.77   0.3500000        0.77    7    5    5  1.1e-13  3.1e-15
   3        0.76   0.3500000        0.76    8    6    6  1.4e-13  4.8e-15
Velocity u2, Pressure p
Scales for dependent variables:
Pressure (comp1.p): 3.6e+02
Velocity field (spatial frame) (comp1.u2): 0.00072
Iter      SolEst     Damping    Stepsize #Res #Jac #Sol   LinErr   LinRes
   1        0.36   0.5000000        0.36    4    2    2  2.5e-15  8.1e-15
Displacement field
Iter      SolEst     Damping    Stepsize #Res #Jac #Sol   LinErr   LinRes
   1        0.95   0.5000000        0.95    4    2    2  3.1e-14  1.5e-12
Spatial mesh displacement
Iter      SolEst     Damping    Stepsize #Res #Jac #Sol   LinErr   LinRes
   1       1e-06   0.5000000       1e-06    4    2    2  1.8e-15  1.8e-15
Solution error estimates for segregated groups
1.3, 0.18, 0.48, 5.1e-07
Residual error estimates for segregated groups
7.9e+02, 1.2e+04, 2.2e+04, 0.15
Segregated solver iteration 3.
Merged variables
Iter      SolEst     Damping    Stepsize #Res #Jac #Sol   LinErr   LinRes
   1        0.75   0.3500000        0.75   10    7    7  1.2e-13  3.2e-15
   2        0.73   0.3500000        0.73   11    8    8  9.6e-14  3.4e-15
   3        0.72   0.3500000        0.72   12    9    9  6.6e-14  3.4e-15
Velocity u2, Pressure p
Iter      SolEst     Damping    Stepsize #Res #Jac #Sol   LinErr   LinRes
   1        0.15   0.5000000        0.15    6    3    3  2.6e-15  7.9e-15
Displacement field
Iter      SolEst     Damping    Stepsize #Res #Jac #Sol   LinErr   LinRes
   1        0.52   0.5000000        0.52    6    3    3  8.3e-14  1.5e-12
Spatial mesh displacement
Iter      SolEst     Damping    Stepsize #Res #Jac #Sol   LinErr   LinRes
   1       1e-06   0.5000000       1e-06    6    3    3  1.5e-15  1.8e-15
Solution error estimates for segregated groups
1.2, 0.077, 0.26, 5.1e-07
Residual error estimates for segregated groups
2.2e+02, 6.9e+03, 1.6e+04, 0.15
Segregated solver iteration 4.
```

```
Merged variables
Iter      SolEst     Damping    Stepsize #Res #Jac #Sol   LinErr   LinRes
   1         0.7   0.3500000         0.7   14   10   10  1.6e-13    4e-15
   2        0.68   0.3500000        0.68   15   11   11  5.9e-14  4.1e-15
   3        0.59   0.3500000        0.59   16   12   12  6.6e-14  4.7e-15
Velocity u2, Pressure p
Iter      SolEst     Damping    Stepsize #Res #Jac #Sol   LinErr   LinRes
   1       0.072   0.5000000       0.072    8    4    4  1.9e-15  7.8e-15
Displacement field
Iter      SolEst     Damping    Stepsize #Res #Jac #Sol   LinErr   LinRes
   1        0.29   0.5000000        0.29    8    4    4  4.5e-14  1.5e-12
Spatial mesh displacement
Iter      SolEst     Damping    Stepsize #Res #Jac #Sol   LinErr   LinRes
   1     8.4e-07   0.5000000     8.4e-07    8    4    4  1.5e-15  1.8e-15
Solution error estimates for segregated groups
1, 0.036, 0.15, 4.2e-07
Residual error estimates for segregated groups
84, 3.6e+03, 1.1e+04, 0.16
Segregated solver iteration 5.
Merged variables
Iter      SolEst     Damping    Stepsize #Res #Jac #Sol   LinErr   LinRes
   1        0.41   0.3500000        0.41   18   13   13  6.8e-14  4.7e-15
   2        0.27   0.3500000        0.27   19   14   14  1.3e-13  3.1e-15
   3        0.18   0.3500000        0.18   20   15   15  6.3e-14  3.5e-15
Velocity u2, Pressure p
Iter      SolEst     Damping    Stepsize #Res #Jac #Sol   LinErr   LinRes
   1       0.035   0.5000000       0.035   10    5    5  2.7e-15  7.9e-15
Displacement field
Iter      SolEst     Damping    Stepsize #Res #Jac #Sol   LinErr   LinRes
   1        0.17   0.5000000        0.17   10    5    5  1.6e-13  1.5e-12
Spatial mesh displacement
Iter      SolEst     Damping    Stepsize #Res #Jac #Sol   LinErr   LinRes
   1     6.3e-07   0.5000000     6.3e-07   10    5    5  1.4e-15  1.8e-15
Solution error estimates for segregated groups
0.31, 0.017, 0.084, 3.2e-07
Residual error estimates for segregated groups
33, 1.9e+03, 6.8e+03, 0.17
Segregated solver iteration 6.
Merged variables
Iter      SolEst     Damping    Stepsize #Res #Jac #Sol   LinErr   LinRes
   1        0.12   0.3500000        0.12   22   16   16  7.4e-14  3.1e-15
   2       0.078   0.3500000       0.078   23   17   17  8.6e-14  2.9e-15
   3       0.051   0.3500000       0.051   24   18   18  6.8e-14  3.4e-15
Velocity u2, Pressure p
Iter      SolEst     Damping    Stepsize #Res #Jac #Sol   LinErr   LinRes
   1       0.017   0.5000000       0.017   12    6    6  3.6e-15  7.9e-15
Displacement field
Iter      SolEst     Damping    Stepsize #Res #Jac #Sol   LinErr   LinRes
   1       0.095   0.5000000       0.095   12    6    6  9.6e-14  1.5e-12
Spatial mesh displacement
Iter      SolEst     Damping    Stepsize #Res #Jac #Sol   LinErr   LinRes
   1     4.4e-07   0.5000000     4.4e-07   12    6    6  1.4e-15  1.8e-15
Solution error estimates for segregated groups
0.087, 0.0085, 0.048, 2.2e-07
Residual error estimates for segregated groups
12, 9.4e+02, 4.2e+03, 0.16
```

```
Segregated solver iteration 7.
Merged variables
Iter      SolEst     Damping    Stepsize #Res #Jac #Sol   LinErr   LinRes
   1       0.033   0.3500000       0.033   26   19   19  1.2e-13  4.1e-15
   2       0.022   0.3500000       0.022   27   20   20  1.4e-13  5.3e-15
   3       0.014   0.3500000       0.014   28   21   21  1.4e-13  4.9e-15
Velocity u2, Pressure p
Iter      SolEst     Damping    Stepsize #Res #Jac #Sol   LinErr   LinRes
   1      0.0085   0.5000000      0.0085   14    7    7  2.5e-15  7.9e-15
Displacement field
Iter      SolEst     Damping    Stepsize #Res #Jac #Sol   LinErr   LinRes
   1       0.054   0.5000000       0.054   14    7    7  5.4e-14  1.5e-12
Spatial mesh displacement
Iter      SolEst     Damping    Stepsize #Res #Jac #Sol   LinErr   LinRes
   1       3e-07   0.5000000       3e-07   14    7    7  1.4e-15  1.8e-15
Solution error estimates for segregated groups
0.024, 0.0042, 0.027, 1.5e-07
Residual error estimates for segregated groups
4.1, 4.8e+02, 2.6e+03, 0.16
Segregated solver iteration 8.
Merged variables
Iter      SolEst     Damping    Stepsize #Res #Jac #Sol   LinErr   LinRes
   1      0.0091   0.3500000      0.0091   30   22   22  9.1e-14  4.6e-15
   2      0.0059   0.3500000      0.0059   31   23   23  5.8e-14  2.9e-15
   3      0.0039   0.3500000      0.0039   32   24   24  1.7e-13  3.7e-15
Velocity u2, Pressure p
Iter      SolEst     Damping    Stepsize #Res #Jac #Sol   LinErr   LinRes
   1      0.0042   0.5000000      0.0042   16    8    8  2.6e-15  7.9e-15
Displacement field
Iter      SolEst     Damping    Stepsize #Res #Jac #Sol   LinErr   LinRes
   1        0.03   0.5000000        0.03   16    8    8  3.2e-14  1.5e-12
Spatial mesh displacement
Iter      SolEst     Damping    Stepsize #Res #Jac #Sol   LinErr   LinRes
   1     1.9e-07   0.5000000     1.9e-07   16    8    8  1.8e-15  1.8e-15
Solution error estimates for segregated groups
0.0066, 0.0021, 0.015, 9.5e-08
Residual error estimates for segregated groups
1.4, 2.4e+02, 1.8e+03, 0.15
Segregated solver iteration 9.
Merged variables
Iter      SolEst     Damping    Stepsize #Res #Jac #Sol   LinErr   LinRes
   1      0.0025   0.3500000      0.0025   34   25   25  1.3e-13  3.9e-15
   2      0.0016   0.3500000      0.0016   35   26   26  9.3e-14  3.6e-15
   3      0.0011   0.3500000      0.0011   36   27   27  3.4e-14  3.9e-15
Velocity u2, Pressure p
Iter      SolEst     Damping    Stepsize #Res #Jac #Sol   LinErr   LinRes
   1      0.0021   0.5000000      0.0021   18    9    9  2.1e-15  7.9e-15
Displacement field
Scales for dependent variables:
Auxiliary pressure (comp1.solid.lemm1.pw): 0.36
Displacement field (comp1.u): 2.8e-16
Iter      SolEst     Damping    Stepsize #Res #Jac #Sol   LinErr   LinRes
   1       0.017   0.5000000       0.017   18    9    9    2e-13  1.2e-12
Spatial mesh displacement
Iter      SolEst     Damping    Stepsize #Res #Jac #Sol   LinErr   LinRes
   1     1.2e-07   0.5000000     1.2e-07   18    9    9  1.5e-15  1.8e-15
```

```
Solution error estimates for segregated groups
0.0018, 0.0011, 0.0085, 5.9e-08
Residual error estimates for segregated groups
0.44, 1.2e+02, 1.4e+03, 0.15
Segregated solver iteration 10.
Merged variables
Iter      SolEst     Damping    Stepsize #Res #Jac #Sol   LinErr   LinRes
   1     0.00069   0.3500000     0.00069   38   28   28  5.2e-14  3.4e-15
   2     0.00045   0.3500000     0.00045   39   29   29  9.3e-14  3.1e-15
   3     0.00029   0.3500000     0.00029   40   30   30  7.7e-14  2.9e-15
Velocity u2, Pressure p
Iter      SolEst     Damping    Stepsize #Res #Jac #Sol   LinErr   LinRes
   1      0.0011   0.5000000      0.0011   20   10   10  2.1e-15  7.9e-15
Displacement field
Iter      SolEst     Damping    Stepsize #Res #Jac #Sol   LinErr   LinRes
   1      0.0094   0.5000000      0.0094   20   10   10  1.2e-13  1.2e-12
Spatial mesh displacement
Iter      SolEst     Damping    Stepsize #Res #Jac #Sol   LinErr   LinRes
   1     7.3e-08   0.5000000     7.3e-08   20   10   10    2e-15  1.8e-15
Solution error estimates for segregated groups
0.0005, 0.00053, 0.0047, 3.6e-08
Residual error estimates for segregated groups
0.14, 60, 1.2e+03, 0.17
Segregated solver iteration 11.
Merged variables
Iter      SolEst     Damping    Stepsize #Res #Jac #Sol   LinErr   LinRes
   1     0.00019   0.3500000     0.00019   42   31   31  5.2e-14  3.7e-15
   2     0.00012   0.3500000     0.00012   43   32   32  1.4e-13  3.7e-15
   3       8e-05   0.3500000       8e-05   44   33   33  1.7e-13    4e-15
Velocity u2, Pressure p
Iter      SolEst     Damping    Stepsize #Res #Jac #Sol   LinErr   LinRes
   1     0.00052   0.5000000     0.00052   22   11   11  2.8e-15  7.9e-15
Displacement field
Iter      SolEst     Damping    Stepsize #Res #Jac #Sol   LinErr   LinRes
   1      0.0051   0.5000000      0.0051   22   11   11  1.5e-13  1.2e-12
Spatial mesh displacement
Iter      SolEst     Damping    Stepsize #Res #Jac #Sol   LinErr   LinRes
   1     4.4e-08   0.5000000     4.4e-08   22   11   11  1.5e-15  1.8e-15
Solution error estimates for segregated groups
0.00014, 0.00026, 0.0026, 2.2e-08
Residual error estimates for segregated groups
0.043, 30, 1.2e+03, 0.14
Segregated solver iteration 12.
Merged variables
Iter      SolEst     Damping    Stepsize #Res #Jac #Sol   LinErr   LinRes
   1     5.2e-05   0.3500000     5.2e-05   46   34   34  5.3e-14  3.7e-15
   2     3.4e-05   0.3500000     3.4e-05   47   35   35  1.3e-13  4.3e-15
   3     2.2e-05   0.3500000     2.2e-05   48   36   36    1e-13  3.6e-15
Velocity u2, Pressure p
Iter      SolEst     Damping    Stepsize #Res #Jac #Sol   LinErr   LinRes
   1     0.00026   0.5000000     0.00026   24   12   12    5e-15  7.9e-15
Displacement field
Iter      SolEst     Damping    Stepsize #Res #Jac #Sol   LinErr   LinRes
   1      0.0028   0.5000000      0.0028   24   12   12  1.1e-13  1.2e-12
Spatial mesh displacement
Iter      SolEst     Damping    Stepsize #Res #Jac #Sol   LinErr   LinRes
```

```
   1     2.6e-08   0.5000000     2.6e-08   24   12   12  1.4e-15  1.8e-15
Solution error estimates for segregated groups
3.8e-05, 0.00013, 0.0014, 1.3e-08
Residual error estimates for segregated groups
0.013, 15, 1.2e+03, 0.17
Segregated solver iteration 13.
Merged variables
Iter      SolEst     Damping    Stepsize #Res #Jac #Sol   LinErr   LinRes
   1     1.4e-05   0.3500000     1.4e-05   50   37   37  7.5e-14  3.2e-15
   2     9.3e-06   0.3500000     9.3e-06   51   38   38  1.6e-13  4.1e-15
   3       6e-06   0.3500000       6e-06   52   39   39  9.5e-14  3.6e-15
Velocity u2, Pressure p
Iter      SolEst     Damping    Stepsize #Res #Jac #Sol   LinErr   LinRes
   1     0.00013   0.5000000     0.00013   26   13   13  2.4e-15  7.8e-15
Displacement field
Iter      SolEst     Damping    Stepsize #Res #Jac #Sol   LinErr   LinRes
   1      0.0015   0.5000000      0.0015   26   13   13  8.5e-14  1.2e-12
Spatial mesh displacement
Iter      SolEst     Damping    Stepsize #Res #Jac #Sol   LinErr   LinRes
   1     1.5e-08   0.5000000     1.5e-08   26   13   13  1.8e-15  1.8e-15
Solution error estimates for segregated groups
1e-05, 6.6e-05, 0.00076, 7.5e-09
Residual error estimates for segregated groups
0.004, 7.5, 1.2e+03, 0.16
Solution time: 154 s. (2 minutes, 34 seconds)
Physical memory: 4.69 GB
Virtual memory: 25.51 GB
Ended at Jan 31, 2025, 7:20:14 PM.
----- Stationary Solver 1 in study1/Approach Curve Solver (sol1) -------------->
```

d=2230 (su56)

GENERAL

Description	Value
Solution	d=2230

LOG

```
<---- Compile Equations: Stationary in study1/Approach Curve Solver (sol1) -----
Started at Jan 31, 2025, 7:20:14 PM.
Parameter d = 2.2300000000000002E-6 (m).
Geometry shape function: Linear serendipity
Number of vertex elements: 18
Number of boundary elements: 6194
Number of elements: 155606
Minimum element quality: 0.4894
Time: 9 s.
Physical memory: 2.95 GB
Virtual memory: 15.21 GB
Ended at Jan 31, 2025, 7:20:23 PM.
----- Compile Equations: Stationary in study1/Approach Curve Solver (sol1) ---->

<---- Dependent Variables 1 in study1/Approach Curve Solver (sol1) -------------
Started at Jan 31, 2025, 7:20:23 PM.
Solution time: 0 s.
Physical memory: 2.95 GB
Virtual memory: 15.21 GB
Ended at Jan 31, 2025, 7:20:23 PM.
----- Dependent Variables 1 in study1/Approach Curve Solver (sol1) ------------>

<---- Stationary Solver 1 in study1/Approach Curve Solver (sol1) ---------------
Started at Jan 31, 2025, 7:20:23 PM.
Segregated solver
Segregated Step 11 does not have any components solved for.
Number of degrees of freedom solved for: 790553 (plus 11811 internal DOFs).
Segregated solver iteration 1.
Merged variables
Nonsymmetric matrix found.
Scales for dependent variables:
Concentration (comp1.Cl): 1.4e+02
Concentration (comp1.K): 1.4e+02
Electric potential (comp1.V): 1.9e+02
Orthonormal null-space function used.
Iter      SolEst     Damping    Stepsize #Res #Jac #Sol   LinErr   LinRes
   1        0.83   0.3500000        0.83    2    1    1  2.5e-11  2.8e-15
   2        0.81   0.3500000        0.81    3    2    2  3.2e-11  2.7e-15
   3         0.8   0.3500000         0.8    4    3    3  3.2e-11  2.8e-15
Velocity u2, Pressure p
Nonsymmetric matrix found.
Scales for dependent variables:
Pressure (comp1.p): 1
Velocity field (spatial frame) (comp1.u2): 0.00018
Orthonormal null-space function used.
Iter      SolEst     Damping    Stepsize #Res #Jac #Sol   LinErr   LinRes
   1         1.1   0.5000000         1.1    2    1    1  1.5e-15  2.3e-15
Displacement field
Nonsymmetric matrix found.
Scales for dependent variables:
Auxiliary pressure (comp1.solid.lemm1.pw): 1
Displacement field (comp1.u): 2.8e-18
Orthonormal null-space function used.
Iter      SolEst     Damping    Stepsize #Res #Jac #Sol   LinErr   LinRes
   1         1.9   0.5000000         1.9    2    1    1  1.8e-13  1.5e-12
Spatial mesh displacement
```

```
Symmetric matrices found.
Scales for dependent variables:
Spatial mesh displacement (comp1.spatial.disp): 1.2e-09
Orthonormal null-space function used.
Iter      SolEst     Damping    Stepsize #Res #Jac #Sol   LinErr   LinRes
   1     6.8e-07   0.5000000     6.8e-07    2    1    1  1.6e-15  1.8e-15
Solution error estimates for segregated groups
1.4, 0.54, 0.95, 3.4e-07
Residual error estimates for segregated groups
7.9e+03, 8.2e+04, 2.2e+04, 0.14
Segregated solver iteration 2.
Merged variables
Scales for dependent variables:
Concentration (comp1.Cl): 1.4e+02
Concentration (comp1.K): 1.4e+02
Electric potential (comp1.V): 0.064
Iter      SolEst     Damping    Stepsize #Res #Jac #Sol   LinErr   LinRes
   1        0.78   0.3500000        0.78    6    4    4  9.7e-14  3.9e-15
   2        0.77   0.3500000        0.77    7    5    5  4.2e-14    3e-15
   3        0.76   0.3500000        0.76    8    6    6  1.1e-13  3.3e-15
Velocity u2, Pressure p
Scales for dependent variables:
Pressure (comp1.p): 3.6e+02
Velocity field (spatial frame) (comp1.u2): 0.00072
Iter      SolEst     Damping    Stepsize #Res #Jac #Sol   LinErr   LinRes
   1        0.36   0.5000000        0.36    4    2    2    3e-15  7.9e-15
Displacement field
Iter      SolEst     Damping    Stepsize #Res #Jac #Sol   LinErr   LinRes
   1        0.95   0.5000000        0.95    4    2    2  5.8e-14  1.5e-12
Spatial mesh displacement
Iter      SolEst     Damping    Stepsize #Res #Jac #Sol   LinErr   LinRes
   1       1e-06   0.5000000       1e-06    4    2    2  1.5e-15  1.8e-15
Solution error estimates for segregated groups
1.3, 0.18, 0.48, 5.1e-07
Residual error estimates for segregated groups
7.9e+02, 1.5e+04, 2.2e+04, 0.15
Segregated solver iteration 3.
Merged variables
Iter      SolEst     Damping    Stepsize #Res #Jac #Sol   LinErr   LinRes
   1        0.75   0.3500000        0.75   10    7    7  6.4e-14  3.1e-15
   2        0.73   0.3500000        0.73   11    8    8  8.4e-14    4e-15
   3        0.72   0.3500000        0.72   12    9    9  1.1e-13  2.9e-15
Velocity u2, Pressure p
Iter      SolEst     Damping    Stepsize #Res #Jac #Sol   LinErr   LinRes
   1        0.15   0.5000000        0.15    6    3    3  2.8e-15  7.9e-15
Displacement field
Iter      SolEst     Damping    Stepsize #Res #Jac #Sol   LinErr   LinRes
   1        0.52   0.5000000        0.52    6    3    3  1.1e-13  1.5e-12
Spatial mesh displacement
Iter      SolEst     Damping    Stepsize #Res #Jac #Sol   LinErr   LinRes
   1       1e-06   0.5000000       1e-06    6    3    3  1.6e-15  1.8e-15
Solution error estimates for segregated groups
1.2, 0.077, 0.26, 5.1e-07
Residual error estimates for segregated groups
2.2e+02, 8.6e+03, 1.6e+04, 0.16
Segregated solver iteration 4.
```

```
Merged variables
Iter      SolEst     Damping    Stepsize #Res #Jac #Sol   LinErr   LinRes
   1         0.7   0.3500000         0.7   14   10   10    6e-13  3.2e-14
   2        0.68   0.3500000        0.68   15   11   11  3.1e-13  1.6e-14
   3        0.59   0.3500000        0.59   16   12   12  5.4e-13  3.1e-14
Velocity u2, Pressure p
Iter      SolEst     Damping    Stepsize #Res #Jac #Sol   LinErr   LinRes
   1       0.072   0.5000000       0.072    8    4    4  2.9e-15    8e-15
Displacement field
Iter      SolEst     Damping    Stepsize #Res #Jac #Sol   LinErr   LinRes
   1        0.29   0.5000000        0.29    8    4    4  6.2e-14  1.5e-12
Spatial mesh displacement
Iter      SolEst     Damping    Stepsize #Res #Jac #Sol   LinErr   LinRes
   1     8.5e-07   0.5000000     8.5e-07    8    4    4  1.4e-15  1.8e-15
Solution error estimates for segregated groups
1, 0.036, 0.15, 4.2e-07
Residual error estimates for segregated groups
83, 4.6e+03, 1.1e+04, 0.16
Segregated solver iteration 5.
Merged variables
Iter      SolEst     Damping    Stepsize #Res #Jac #Sol   LinErr   LinRes
   1        0.41   0.3500000        0.41   18   13   13  5.6e-13  2.5e-14
   2        0.27   0.3500000        0.27   19   14   14  3.1e-13  3.1e-14
   3        0.18   0.3500000        0.18   20   15   15  4.4e-13  3.8e-14
Velocity u2, Pressure p
Iter      SolEst     Damping    Stepsize #Res #Jac #Sol   LinErr   LinRes
   1       0.035   0.5000000       0.035   10    5    5  4.2e-15  7.9e-15
Displacement field
Iter      SolEst     Damping    Stepsize #Res #Jac #Sol   LinErr   LinRes
   1        0.17   0.5000000        0.17   10    5    5  1.2e-13  1.5e-12
Spatial mesh displacement
Iter      SolEst     Damping    Stepsize #Res #Jac #Sol   LinErr   LinRes
   1     6.4e-07   0.5000000     6.4e-07   10    5    5  1.3e-15  1.9e-15
Solution error estimates for segregated groups
0.31, 0.017, 0.084, 3.2e-07
Residual error estimates for segregated groups
33, 2.3e+03, 6.9e+03, 0.16
Segregated solver iteration 6.
Merged variables
Iter      SolEst     Damping    Stepsize #Res #Jac #Sol   LinErr   LinRes
   1        0.12   0.3500000        0.12   22   16   16  3.8e-13  3.6e-14
   2       0.078   0.3500000       0.078   23   17   17  5.5e-13  4.7e-14
   3       0.051   0.3500000       0.051   24   18   18  5.4e-13    2e-14
Velocity u2, Pressure p
Iter      SolEst     Damping    Stepsize #Res #Jac #Sol   LinErr   LinRes
   1       0.017   0.5000000       0.017   12    6    6  2.2e-15  7.7e-15
Displacement field
Iter      SolEst     Damping    Stepsize #Res #Jac #Sol   LinErr   LinRes
   1       0.095   0.5000000       0.095   12    6    6  9.4e-14  1.5e-12
Spatial mesh displacement
Iter      SolEst     Damping    Stepsize #Res #Jac #Sol   LinErr   LinRes
   1     4.4e-07   0.5000000     4.4e-07   12    6    6  1.5e-15  1.8e-15
Solution error estimates for segregated groups
0.087, 0.0085, 0.048, 2.2e-07
Residual error estimates for segregated groups
12, 1.2e+03, 4.2e+03, 0.15
```

```
Segregated solver iteration 7.
Merged variables
Iter      SolEst     Damping    Stepsize #Res #Jac #Sol   LinErr   LinRes
   1       0.033   0.3500000       0.033   26   19   19  3.6e-13  4.5e-14
   2       0.022   0.3500000       0.022   27   20   20  7.7e-13  4.4e-14
   3       0.014   0.3500000       0.014   28   21   21  1.1e-12  2.1e-14
Velocity u2, Pressure p
Iter      SolEst     Damping    Stepsize #Res #Jac #Sol   LinErr   LinRes
   1      0.0085   0.5000000      0.0085   14    7    7  2.6e-15  7.8e-15
Displacement field
Iter      SolEst     Damping    Stepsize #Res #Jac #Sol   LinErr   LinRes
   1       0.054   0.5000000       0.054   14    7    7  1.1e-13  1.5e-12
Spatial mesh displacement
Iter      SolEst     Damping    Stepsize #Res #Jac #Sol   LinErr   LinRes
   1       3e-07   0.5000000       3e-07   14    7    7  1.9e-15  1.8e-15
Solution error estimates for segregated groups
0.024, 0.0042, 0.027, 1.5e-07
Residual error estimates for segregated groups
4.1, 6e+02, 2.6e+03, 0.17
Segregated solver iteration 8.
Merged variables
Iter      SolEst     Damping    Stepsize #Res #Jac #Sol   LinErr   LinRes
   1      0.0091   0.3500000      0.0091   30   22   22  4.6e-13    4e-14
   2      0.0059   0.3500000      0.0059   31   23   23    8e-13  2.9e-14
   3      0.0039   0.3500000      0.0039   32   24   24  1.2e-12  1.6e-14
Velocity u2, Pressure p
Iter      SolEst     Damping    Stepsize #Res #Jac #Sol   LinErr   LinRes
   1      0.0042   0.5000000      0.0042   16    8    8  2.4e-15  7.8e-15
Displacement field
Iter      SolEst     Damping    Stepsize #Res #Jac #Sol   LinErr   LinRes
   1        0.03   0.5000000        0.03   16    8    8  3.2e-14  1.5e-12
Spatial mesh displacement
Iter      SolEst     Damping    Stepsize #Res #Jac #Sol   LinErr   LinRes
   1     1.9e-07   0.5000000     1.9e-07   16    8    8  1.9e-15  1.8e-15
Solution error estimates for segregated groups
0.0066, 0.0021, 0.015, 9.5e-08
Residual error estimates for segregated groups
1.4, 3e+02, 1.8e+03, 0.15
Segregated solver iteration 9.
Merged variables
Iter      SolEst     Damping    Stepsize #Res #Jac #Sol   LinErr   LinRes
   1      0.0025   0.3500000      0.0025   34   25   25  2.3e-13  1.7e-14
   2      0.0016   0.3500000      0.0016   35   26   26  7.6e-13  5.6e-14
   3      0.0011   0.3500000      0.0011   36   27   27  5.3e-13  2.2e-14
Velocity u2, Pressure p
Iter      SolEst     Damping    Stepsize #Res #Jac #Sol   LinErr   LinRes
   1      0.0021   0.5000000      0.0021   18    9    9  3.7e-15  7.9e-15
Displacement field
Scales for dependent variables:
Auxiliary pressure (comp1.solid.lemm1.pw): 0.36
Displacement field (comp1.u): 2.8e-16
Iter      SolEst     Damping    Stepsize #Res #Jac #Sol   LinErr   LinRes
   1       0.017   0.5000000       0.017   18    9    9  9.8e-14  1.2e-12
Spatial mesh displacement
Iter      SolEst     Damping    Stepsize #Res #Jac #Sol   LinErr   LinRes
   1     1.2e-07   0.5000000     1.2e-07   18    9    9  1.3e-15  1.8e-15
```

```
Solution error estimates for segregated groups
0.0018, 0.0011, 0.0085, 6e-08
Residual error estimates for segregated groups
0.44, 1.5e+02, 1.4e+03, 0.16
Segregated solver iteration 10.
Merged variables
Iter      SolEst     Damping    Stepsize #Res #Jac #Sol   LinErr   LinRes
   1     0.00069   0.3500000     0.00069   38   28   28  8.1e-13    4e-14
   2     0.00045   0.3500000     0.00045   39   29   29  5.8e-13    5e-14
   3     0.00029   0.3500000     0.00029   40   30   30  1.6e-12  2.8e-14
Velocity u2, Pressure p
Iter      SolEst     Damping    Stepsize #Res #Jac #Sol   LinErr   LinRes
   1      0.0011   0.5000000      0.0011   20   10   10  3.4e-15    8e-15
Displacement field
Iter      SolEst     Damping    Stepsize #Res #Jac #Sol   LinErr   LinRes
   1      0.0094   0.5000000      0.0094   20   10   10  1.3e-13  1.2e-12
Spatial mesh displacement
Iter      SolEst     Damping    Stepsize #Res #Jac #Sol   LinErr   LinRes
   1     7.3e-08   0.5000000     7.3e-08   20   10   10  1.3e-15  1.8e-15
Solution error estimates for segregated groups
0.0005, 0.00053, 0.0047, 3.6e-08
Residual error estimates for segregated groups
0.14, 75, 1.2e+03, 0.16
Segregated solver iteration 11.
Merged variables
Iter      SolEst     Damping    Stepsize #Res #Jac #Sol   LinErr   LinRes
   1     0.00019   0.3500000     0.00019   42   31   31  4.6e-13  3.2e-14
   2     0.00012   0.3500000     0.00012   43   32   32  1.1e-12  3.4e-14
   3       8e-05   0.3500000       8e-05   44   33   33  3.5e-13    2e-14
Velocity u2, Pressure p
Iter      SolEst     Damping    Stepsize #Res #Jac #Sol   LinErr   LinRes
   1     0.00052   0.5000000     0.00052   22   11   11  2.8e-15  7.8e-15
Displacement field
Iter      SolEst     Damping    Stepsize #Res #Jac #Sol   LinErr   LinRes
   1      0.0051   0.5000000      0.0051   22   11   11  9.3e-14  1.2e-12
Spatial mesh displacement
Iter      SolEst     Damping    Stepsize #Res #Jac #Sol   LinErr   LinRes
   1     4.4e-08   0.5000000     4.4e-08   22   11   11  1.6e-15  1.8e-15
Solution error estimates for segregated groups
0.00014, 0.00026, 0.0026, 2.2e-08
Residual error estimates for segregated groups
0.043, 38, 1.2e+03, 0.15
Segregated solver iteration 12.
Merged variables
Iter      SolEst     Damping    Stepsize #Res #Jac #Sol   LinErr   LinRes
   1     5.2e-05   0.3500000     5.2e-05   46   34   34  3.6e-13  3.8e-14
   2     3.4e-05   0.3500000     3.4e-05   47   35   35  8.2e-13    4e-14
   3     2.2e-05   0.3500000     2.2e-05   48   36   36  4.3e-13  2.1e-14
Velocity u2, Pressure p
Iter      SolEst     Damping    Stepsize #Res #Jac #Sol   LinErr   LinRes
   1     0.00026   0.5000000     0.00026   24   12   12  3.5e-15  7.9e-15
Displacement field
Iter      SolEst     Damping    Stepsize #Res #Jac #Sol   LinErr   LinRes
   1      0.0028   0.5000000      0.0028   24   12   12    1e-13  1.2e-12
Spatial mesh displacement
Iter      SolEst     Damping    Stepsize #Res #Jac #Sol   LinErr   LinRes
```

```
   1     2.6e-08   0.5000000     2.6e-08   24    12    12  1.6e-15  1.8e-15
Solution error estimates for segregated groups
3.8e-05, 0.00013, 0.0014, 1.3e-08
Residual error estimates for segregated groups
0.013, 19, 1.2e+03, 0.16
Segregated solver iteration 13.
Merged variables
Iter      SolEst     Damping    Stepsize #Res #Jac #Sol   LinErr   LinRes
   1     1.4e-05   0.3500000     1.4e-05   50    37    37  6.9e-13  3.9e-14
   2     9.3e-06   0.3500000     9.3e-06   51    38    38  6.3e-13  5.3e-14
   3       6e-06   0.3500000       6e-06   52    39    39  1.2e-12  5.4e-14
Velocity u2, Pressure p
Iter      SolEst     Damping    Stepsize #Res #Jac #Sol   LinErr   LinRes
   1     0.00013   0.5000000     0.00013   26    13    13  2.1e-15  7.7e-15
Displacement field
Iter      SolEst     Damping    Stepsize #Res #Jac #Sol   LinErr   LinRes
   1      0.0015   0.5000000      0.0015   26    13    13  1.2e-13  1.2e-12
Spatial mesh displacement
Iter      SolEst     Damping    Stepsize #Res #Jac #Sol   LinErr   LinRes
   1     1.5e-08   0.5000000     1.5e-08   26    13    13  1.6e-15  1.8e-15
Solution error estimates for segregated groups
1e-05, 6.6e-05, 0.00076, 7.5e-09
Residual error estimates for segregated groups
0.004, 9.4, 1.2e+03, 0.16
Solution time: 158 s. (2 minutes, 38 seconds)
Physical memory: 4.78 GB
Virtual memory: 25.59 GB
Ended at Jan 31, 2025, 7:23:01 PM.
----- Stationary Solver 1 in study1/Approach Curve Solver (sol1) -------------->
```

d=2225 (su57)

GENERAL

Description	Value
Solution	d=2225

LOG

```
<---- Compile Equations: Stationary in study1/Approach Curve Solver (sol1) -----
Started at Jan 31, 2025, 7:23:01 PM.
Parameter d = 2.2250000000000003E-6 (m).
Geometry shape function: Linear serendipity
Number of vertex elements: 18
Number of boundary elements: 6192
Number of elements: 155424
Minimum element quality: 0.4851
Time: 8 s.
Physical memory: 2.92 GB
Virtual memory: 15.25 GB
Ended at Jan 31, 2025, 7:23:10 PM.
----- Compile Equations: Stationary in study1/Approach Curve Solver (sol1) ---->

<---- Dependent Variables 1 in study1/Approach Curve Solver (sol1) -------------
Started at Jan 31, 2025, 7:23:10 PM.
Solution time: 0 s.
Physical memory: 2.92 GB
Virtual memory: 15.18 GB
Ended at Jan 31, 2025, 7:23:10 PM.
----- Dependent Variables 1 in study1/Approach Curve Solver (sol1) ------------>

<---- Stationary Solver 1 in study1/Approach Curve Solver (sol1) ---------------
Started at Jan 31, 2025, 7:23:10 PM.
Segregated solver
Segregated Step 11 does not have any components solved for.
Number of degrees of freedom solved for: 789649 (plus 11807 internal DOFs).
Segregated solver iteration 1.
Merged variables
Nonsymmetric matrix found.
Scales for dependent variables:
Concentration (comp1.Cl): 1.4e+02
Concentration (comp1.K): 1.4e+02
Electric potential (comp1.V): 1.9e+02
Orthonormal null-space function used.
Iter      SolEst     Damping    Stepsize #Res #Jac #Sol   LinErr   LinRes
   1        0.83   0.3500000        0.83    2    1    1  1.6e-11  2.7e-15
   2        0.81   0.3500000        0.81    3    2    2  1.7e-11  2.7e-15
   3         0.8   0.3500000         0.8    4    3    3  2.6e-11  2.8e-15
Velocity u2, Pressure p
Nonsymmetric matrix found.
Scales for dependent variables:
Pressure (comp1.p): 1
Velocity field (spatial frame) (comp1.u2): 0.00018
Orthonormal null-space function used.
Iter      SolEst     Damping    Stepsize #Res #Jac #Sol   LinErr   LinRes
   1         1.1   0.5000000         1.1    2    1    1  1.8e-15  2.3e-15
Displacement field
Nonsymmetric matrix found.
Scales for dependent variables:
Auxiliary pressure (comp1.solid.lemm1.pw): 1
Displacement field (comp1.u): 2.8e-18
Orthonormal null-space function used.
Iter      SolEst     Damping    Stepsize #Res #Jac #Sol   LinErr   LinRes
   1         1.9   0.5000000         1.9    2    1    1  6.9e-14  1.5e-12
Spatial mesh displacement
```

```
Symmetric matrices found.
Scales for dependent variables:
Spatial mesh displacement (comp1.spatial.disp): 1.2e-09
Orthonormal null-space function used.
Iter      SolEst     Damping    Stepsize #Res #Jac #Sol   LinErr   LinRes
   1     6.8e-07   0.5000000     6.8e-07    2    1    1  1.8e-15  1.9e-15
Solution error estimates for segregated groups
1.4, 0.54, 0.95, 3.4e-07
Residual error estimates for segregated groups
7.9e+03, 8.2e+04, 2.2e+04, 0.15
Segregated solver iteration 2.
Merged variables
Scales for dependent variables:
Concentration (comp1.Cl): 1.4e+02
Concentration (comp1.K): 1.4e+02
Electric potential (comp1.V): 0.064
Iter      SolEst     Damping    Stepsize #Res #Jac #Sol   LinErr   LinRes
   1        0.78   0.3500000        0.78    6    4    4  8.6e-14  7.3e-15
   2        0.77   0.3500000        0.77    7    5    5  1.2e-13  5.4e-15
   3        0.76   0.3500000        0.76    8    6    6  1.1e-13  1.1e-14
Velocity u2, Pressure p
Scales for dependent variables:
Pressure (comp1.p): 3.6e+02
Velocity field (spatial frame) (comp1.u2): 0.00072
Iter      SolEst     Damping    Stepsize #Res #Jac #Sol   LinErr   LinRes
   1        0.36   0.5000000        0.36    4    2    2  4.5e-15  7.8e-15
Displacement field
Iter      SolEst     Damping    Stepsize #Res #Jac #Sol   LinErr   LinRes
   1        0.95   0.5000000        0.95    4    2    2  8.5e-14  1.5e-12
Spatial mesh displacement
Iter      SolEst     Damping    Stepsize #Res #Jac #Sol   LinErr   LinRes
   1       1e-06   0.5000000       1e-06    4    2    2  1.9e-15  1.8e-15
Solution error estimates for segregated groups
1.3, 0.18, 0.48, 5.1e-07
Residual error estimates for segregated groups
7.9e+02, 1.4e+04, 2.2e+04, 0.15
Segregated solver iteration 3.
Merged variables
Iter      SolEst     Damping    Stepsize #Res #Jac #Sol   LinErr   LinRes
   1        0.75   0.3500000        0.75   10    7    7  8.1e-14  3.7e-15
   2        0.73   0.3500000        0.73   11    8    8    2e-13  4.4e-15
   3        0.72   0.3500000        0.72   12    9    9  1.8e-13  7.9e-15
Velocity u2, Pressure p
Iter      SolEst     Damping    Stepsize #Res #Jac #Sol   LinErr   LinRes
   1        0.15   0.5000000        0.15    6    3    3    3e-15  7.8e-15
Displacement field
Iter      SolEst     Damping    Stepsize #Res #Jac #Sol   LinErr   LinRes
   1        0.52   0.5000000        0.52    6    3    3  6.3e-14  1.5e-12
Spatial mesh displacement
Iter      SolEst     Damping    Stepsize #Res #Jac #Sol   LinErr   LinRes
   1       1e-06   0.5000000       1e-06    6    3    3  1.6e-15  1.8e-15
Solution error estimates for segregated groups
1.2, 0.077, 0.26, 5.1e-07
Residual error estimates for segregated groups
2.2e+02, 7.6e+03, 1.6e+04, 0.16
Segregated solver iteration 4.
```

```
Merged variables
Iter      SolEst     Damping    Stepsize #Res #Jac #Sol   LinErr   LinRes
   1         0.7   0.3500000         0.7   14   10   10  7.4e-14  3.4e-15
   2        0.68   0.3500000        0.68   15   11   11  8.5e-14  3.9e-15
   3        0.59   0.3500000        0.59   16   12   12  6.3e-14    3e-15
Velocity u2, Pressure p
Iter      SolEst     Damping    Stepsize #Res #Jac #Sol   LinErr   LinRes
   1       0.072   0.5000000       0.072    8    4    4  3.1e-15  7.7e-15
Displacement field
Iter      SolEst     Damping    Stepsize #Res #Jac #Sol   LinErr   LinRes
   1        0.29   0.5000000        0.29    8    4    4  1.2e-13  1.5e-12
Spatial mesh displacement
Iter      SolEst     Damping    Stepsize #Res #Jac #Sol   LinErr   LinRes
   1     8.5e-07   0.5000000     8.5e-07    8    4    4  1.4e-15  1.8e-15
Solution error estimates for segregated groups
1, 0.036, 0.15, 4.3e-07
Residual error estimates for segregated groups
83, 4e+03, 1.1e+04, 0.16
Segregated solver iteration 5.
Merged variables
Iter      SolEst     Damping    Stepsize #Res #Jac #Sol   LinErr   LinRes
   1        0.41   0.3500000        0.41   18   13   13  9.4e-14  3.4e-15
   2        0.27   0.3500000        0.27   19   14   14  7.6e-14    3e-15
   3        0.18   0.3500000        0.18   20   15   15  7.9e-14    3e-15
Velocity u2, Pressure p
Iter      SolEst     Damping    Stepsize #Res #Jac #Sol   LinErr   LinRes
   1       0.035   0.5000000       0.035   10    5    5  3.4e-15  7.9e-15
Displacement field
Iter      SolEst     Damping    Stepsize #Res #Jac #Sol   LinErr   LinRes
   1        0.17   0.5000000        0.17   10    5    5  9.9e-14  1.5e-12
Spatial mesh displacement
Iter      SolEst     Damping    Stepsize #Res #Jac #Sol   LinErr   LinRes
   1     6.4e-07   0.5000000     6.4e-07   10    5    5  1.6e-15  1.9e-15
Solution error estimates for segregated groups
0.31, 0.017, 0.084, 3.2e-07
Residual error estimates for segregated groups
32, 2.1e+03, 6.9e+03, 0.16
Segregated solver iteration 6.
Merged variables
Iter      SolEst     Damping    Stepsize #Res #Jac #Sol   LinErr   LinRes
   1        0.12   0.3500000        0.12   22   16   16  8.3e-14  5.1e-15
   2       0.078   0.3500000       0.078   23   17   17  1.2e-13  3.2e-15
   3       0.051   0.3500000       0.051   24   18   18  8.4e-14    3e-15
Velocity u2, Pressure p
Iter      SolEst     Damping    Stepsize #Res #Jac #Sol   LinErr   LinRes
   1       0.017   0.5000000       0.017   12    6    6  4.9e-15  8.1e-15
Displacement field
Iter      SolEst     Damping    Stepsize #Res #Jac #Sol   LinErr   LinRes
   1       0.095   0.5000000       0.095   12    6    6  5.7e-14  1.5e-12
Spatial mesh displacement
Iter      SolEst     Damping    Stepsize #Res #Jac #Sol   LinErr   LinRes
   1     4.5e-07   0.5000000     4.5e-07   12    6    6  1.9e-15  1.8e-15
Solution error estimates for segregated groups
0.087, 0.0085, 0.048, 2.2e-07
Residual error estimates for segregated groups
12, 1e+03, 4.2e+03, 0.15
```

```
Segregated solver iteration 7.
Merged variables
Iter      SolEst     Damping    Stepsize #Res #Jac #Sol   LinErr   LinRes
   1       0.033   0.3500000       0.033   26   19   19  9.5e-14    3e-15
   2       0.022   0.3500000       0.022   27   20   20  4.1e-14  3.1e-15
   3       0.014   0.3500000       0.014   28   21   21  8.4e-14  4.7e-15
Velocity u2, Pressure p
Iter      SolEst     Damping    Stepsize #Res #Jac #Sol   LinErr   LinRes
   1      0.0084   0.5000000      0.0084   14    7    7  2.3e-15  7.8e-15
Displacement field
Iter      SolEst     Damping    Stepsize #Res #Jac #Sol   LinErr   LinRes
   1       0.054   0.5000000       0.054   14    7    7  1.3e-13  1.5e-12
Spatial mesh displacement
Iter      SolEst     Damping    Stepsize #Res #Jac #Sol   LinErr   LinRes
   1       3e-07   0.5000000       3e-07   14    7    7  1.5e-15  1.9e-15
Solution error estimates for segregated groups
0.024, 0.0042, 0.027, 1.5e-07
Residual error estimates for segregated groups
4.1, 5.2e+02, 2.6e+03, 0.16
Segregated solver iteration 8.
Merged variables
Iter      SolEst     Damping    Stepsize #Res #Jac #Sol   LinErr   LinRes
   1      0.0091   0.3500000      0.0091   30   22   22  7.5e-14  3.2e-15
   2      0.0059   0.3500000      0.0059   31   23   23  9.7e-14  3.7e-15
   3      0.0039   0.3500000      0.0039   32   24   24  6.4e-14  4.2e-15
Velocity u2, Pressure p
Iter      SolEst     Damping    Stepsize #Res #Jac #Sol   LinErr   LinRes
   1      0.0042   0.5000000      0.0042   16    8    8    3e-15  7.7e-15
Displacement field
Iter      SolEst     Damping    Stepsize #Res #Jac #Sol   LinErr   LinRes
   1        0.03   0.5000000        0.03   16    8    8  8.3e-14  1.5e-12
Spatial mesh displacement
Iter      SolEst     Damping    Stepsize #Res #Jac #Sol   LinErr   LinRes
   1     1.9e-07   0.5000000     1.9e-07   16    8    8  1.6e-15  1.8e-15
Solution error estimates for segregated groups
0.0066, 0.0021, 0.015, 9.6e-08
Residual error estimates for segregated groups
1.4, 2.6e+02, 1.8e+03, 0.16
Segregated solver iteration 9.
Merged variables
Iter      SolEst     Damping    Stepsize #Res #Jac #Sol   LinErr   LinRes
   1      0.0025   0.3500000      0.0025   34   25   25  1.1e-13  3.7e-15
   2      0.0016   0.3500000      0.0016   35   26   26  5.7e-14  3.2e-15
   3      0.0011   0.3500000      0.0011   36   27   27  1.5e-13  3.8e-15
Velocity u2, Pressure p
Iter      SolEst     Damping    Stepsize #Res #Jac #Sol   LinErr   LinRes
   1      0.0021   0.5000000      0.0021   18    9    9  3.6e-15  7.9e-15
Displacement field
Scales for dependent variables:
Auxiliary pressure (comp1.solid.lemm1.pw): 0.36
Displacement field (comp1.u): 2.8e-16
Iter      SolEst     Damping    Stepsize #Res #Jac #Sol   LinErr   LinRes
   1       0.017   0.5000000       0.017   18    9    9  1.6e-13  1.2e-12
Spatial mesh displacement
Iter      SolEst     Damping    Stepsize #Res #Jac #Sol   LinErr   LinRes
   1     1.2e-07   0.5000000     1.2e-07   18    9    9  1.9e-15  1.9e-15
```

```
Solution error estimates for segregated groups
0.0018, 0.0011, 0.0085, 6e-08
Residual error estimates for segregated groups
0.44, 1.3e+02, 1.4e+03, 0.16
Segregated solver iteration 10.
Merged variables
Iter      SolEst     Damping    Stepsize #Res #Jac #Sol   LinErr   LinRes
   1     0.00069   0.3500000     0.00069   38   28   28    7e-14  3.3e-15
   2     0.00045   0.3500000     0.00045   39   29   29  1.3e-13    6e-15
   3     0.00029   0.3500000     0.00029   40   30   30  1.4e-13  3.2e-15
Velocity u2, Pressure p
Iter      SolEst     Damping    Stepsize #Res #Jac #Sol   LinErr   LinRes
   1       0.001   0.5000000       0.001   20   10   10  3.8e-15  7.8e-15
Displacement field
Iter      SolEst     Damping    Stepsize #Res #Jac #Sol   LinErr   LinRes
   1      0.0094   0.5000000      0.0094   20   10   10  1.2e-13  1.2e-12
Spatial mesh displacement
Iter      SolEst     Damping    Stepsize #Res #Jac #Sol   LinErr   LinRes
   1     7.3e-08   0.5000000     7.3e-08   20   10   10  1.4e-15  1.9e-15
Solution error estimates for segregated groups
0.0005, 0.00052, 0.0047, 3.7e-08
Residual error estimates for segregated groups
0.14, 66, 1.2e+03, 0.16
Segregated solver iteration 11.
Merged variables
Iter      SolEst     Damping    Stepsize #Res #Jac #Sol   LinErr   LinRes
   1     0.00019   0.3500000     0.00019   42   31   31  5.7e-14  3.2e-15
   2     0.00012   0.3500000     0.00012   43   32   32  8.3e-14  3.1e-15
   3       8e-05   0.3500000       8e-05   44   33   33  5.6e-14  4.8e-15
Velocity u2, Pressure p
Iter      SolEst     Damping    Stepsize #Res #Jac #Sol   LinErr   LinRes
   1     0.00052   0.5000000     0.00052   22   11   11  2.2e-15  7.9e-15
Displacement field
Iter      SolEst     Damping    Stepsize #Res #Jac #Sol   LinErr   LinRes
   1      0.0051   0.5000000      0.0051   22   11   11  1.3e-13  1.2e-12
Spatial mesh displacement
Iter      SolEst     Damping    Stepsize #Res #Jac #Sol   LinErr   LinRes
   1     4.4e-08   0.5000000     4.4e-08   22   11   11  1.5e-15  1.9e-15
Solution error estimates for segregated groups
0.00014, 0.00026, 0.0026, 2.2e-08
Residual error estimates for segregated groups
0.043, 33, 1.2e+03, 0.16
Segregated solver iteration 12.
Merged variables
Iter      SolEst     Damping    Stepsize #Res #Jac #Sol   LinErr   LinRes
   1     5.2e-05   0.3500000     5.2e-05   46   34   34  5.6e-14  3.4e-15
   2     3.4e-05   0.3500000     3.4e-05   47   35   35  9.3e-14  3.2e-15
   3     2.2e-05   0.3500000     2.2e-05   48   36   36  1.2e-13  3.1e-15
Velocity u2, Pressure p
Iter      SolEst     Damping    Stepsize #Res #Jac #Sol   LinErr   LinRes
   1     0.00026   0.5000000     0.00026   24   12   12    3e-15  7.8e-15
Displacement field
Iter      SolEst     Damping    Stepsize #Res #Jac #Sol   LinErr   LinRes
   1      0.0028   0.5000000      0.0028   24   12   12  1.8e-13  1.2e-12
Spatial mesh displacement
Iter      SolEst     Damping    Stepsize #Res #Jac #Sol   LinErr   LinRes
```

```
   1     2.6e-08   0.5000000     2.6e-08   24   12   12  1.4e-15  1.8e-15
Solution error estimates for segregated groups
3.8e-05, 0.00013, 0.0014, 1.3e-08
Residual error estimates for segregated groups
0.013, 16, 1.2e+03, 0.17
Segregated solver iteration 13.
Merged variables
Iter      SolEst      Damping    Stepsize #Res #Jac #Sol   LinErr   LinRes
   1     1.4e-05   0.3500000     1.4e-05   50   37   37  1.2e-13  4.2e-15
   2     9.3e-06   0.3500000     9.3e-06   51   38   38  4.7e-14  3.1e-15
   3       6e-06   0.3500000       6e-06   52   39   39  1.2e-13  4.5e-15
Velocity u2, Pressure p
Iter      SolEst      Damping    Stepsize #Res #Jac #Sol   LinErr   LinRes
   1     0.00013   0.5000000     0.00013   26   13   13    2e-15  7.9e-15
Displacement field
Iter      SolEst      Damping    Stepsize #Res #Jac #Sol   LinErr   LinRes
   1      0.0015   0.5000000      0.0015   26   13   13  1.2e-13  1.2e-12
Spatial mesh displacement
Iter      SolEst      Damping    Stepsize #Res #Jac #Sol   LinErr   LinRes
   1     1.5e-08   0.5000000     1.5e-08   26   13   13  1.5e-15  1.8e-15
Solution error estimates for segregated groups
1e-05, 6.6e-05, 0.00076, 7.6e-09
Residual error estimates for segregated groups
0.004, 8.2, 1.2e+03, 0.16
Solution time: 157 s. (2 minutes, 37 seconds)
Physical memory: 4.77 GB
Virtual memory: 25.59 GB
Ended at Jan 31, 2025, 7:25:47 PM.
----- Stationary Solver 1 in study1/Approach Curve Solver (sol1) -------------->
```

d=2220 (su58)

GENERAL

Description	Value
Solution	d=2220

LOG

```
<---- Compile Equations: Stationary in study1/Approach Curve Solver (sol1) -----
Started at Jan 31, 2025, 7:25:47 PM.
Parameter d = 2.2200000000000003E-6 (m).
Geometry shape function: Linear serendipity
Number of vertex elements: 18
Number of boundary elements: 6189
Number of elements: 155349
Minimum element quality: 0.4822
Time: 8 s.
Physical memory: 2.96 GB
Virtual memory: 15.28 GB
Ended at Jan 31, 2025, 7:25:56 PM.
----- Compile Equations: Stationary in study1/Approach Curve Solver (sol1) ---->

<---- Dependent Variables 1 in study1/Approach Curve Solver (sol1) -------------
Started at Jan 31, 2025, 7:25:56 PM.
Solution time: 0 s.
Physical memory: 2.96 GB
Virtual memory: 15.28 GB
Ended at Jan 31, 2025, 7:25:56 PM.
----- Dependent Variables 1 in study1/Approach Curve Solver (sol1) ------------>

<---- Stationary Solver 1 in study1/Approach Curve Solver (sol1) ---------------
Started at Jan 31, 2025, 7:25:56 PM.
Segregated solver
Segregated Step 11 does not have any components solved for.
Number of degrees of freedom solved for: 789333 (plus 11801 internal DOFs).
Segregated solver iteration 1.
Merged variables
Nonsymmetric matrix found.
Scales for dependent variables:
Concentration (comp1.Cl): 1.4e+02
Concentration (comp1.K): 1.4e+02
Electric potential (comp1.V): 1.9e+02
Orthonormal null-space function used.
Iter      SolEst     Damping    Stepsize #Res #Jac #Sol   LinErr   LinRes
   1        0.83   0.3500000        0.83    2    1    1  5.1e-11  2.9e-15
   2        0.81   0.3500000        0.81    3    2    2  3.9e-11  2.7e-15
   3         0.8   0.3500000         0.8    4    3    3  2.1e-11  2.7e-15
Velocity u2, Pressure p
Nonsymmetric matrix found.
Scales for dependent variables:
Pressure (comp1.p): 1
Velocity field (spatial frame) (comp1.u2): 0.00018
Orthonormal null-space function used.
Iter      SolEst     Damping    Stepsize #Res #Jac #Sol   LinErr   LinRes
   1         1.1   0.5000000         1.1    2    1    1  1.5e-15  2.3e-15
Displacement field
Nonsymmetric matrix found.
Scales for dependent variables:
Auxiliary pressure (comp1.solid.lemm1.pw): 1
Displacement field (comp1.u): 2.8e-18
Orthonormal null-space function used.
Iter      SolEst     Damping    Stepsize #Res #Jac #Sol   LinErr   LinRes
   1         1.9   0.5000000         1.9    2    1    1    1e-13  1.5e-12
Spatial mesh displacement
```

```
Symmetric matrices found.
Scales for dependent variables:
Spatial mesh displacement (comp1.spatial.disp): 1.2e-09
Orthonormal null-space function used.
Iter      SolEst     Damping    Stepsize #Res #Jac #Sol   LinErr   LinRes
   1     6.8e-07   0.5000000     6.8e-07    2    1    1  1.2e-15  1.8e-15
Solution error estimates for segregated groups
1.4, 0.54, 0.95, 3.4e-07
Residual error estimates for segregated groups
7.9e+03, 8.2e+04, 2.2e+04, 0.13
Segregated solver iteration 2.
Merged variables
Scales for dependent variables:
Concentration (comp1.Cl): 1.4e+02
Concentration (comp1.K): 1.4e+02
Electric potential (comp1.V): 0.064
Iter      SolEst     Damping    Stepsize #Res #Jac #Sol   LinErr   LinRes
   1        0.78   0.3500000        0.78    6    4    4  1.1e-13  2.9e-15
   2        0.77   0.3500000        0.77    7    5    5  8.8e-14  2.8e-15
   3        0.76   0.3500000        0.76    8    6    6  1.2e-13  3.1e-15
Velocity u2, Pressure p
Scales for dependent variables:
Pressure (comp1.p): 3.6e+02
Velocity field (spatial frame) (comp1.u2): 0.00072
Iter      SolEst     Damping    Stepsize #Res #Jac #Sol   LinErr   LinRes
   1        0.36   0.5000000        0.36    4    2    2  2.3e-15  7.9e-15
Displacement field
Iter      SolEst     Damping    Stepsize #Res #Jac #Sol   LinErr   LinRes
   1        0.95   0.5000000        0.95    4    2    2  6.7e-14  1.5e-12
Spatial mesh displacement
Iter      SolEst     Damping    Stepsize #Res #Jac #Sol   LinErr   LinRes
   1       1e-06   0.5000000       1e-06    4    2    2  1.8e-15  1.8e-15
Solution error estimates for segregated groups
1.3, 0.18, 0.48, 5.1e-07
Residual error estimates for segregated groups
7.9e+02, 1.4e+04, 2.2e+04, 0.14
Segregated solver iteration 3.
Merged variables
Iter      SolEst     Damping    Stepsize #Res #Jac #Sol   LinErr   LinRes
   1        0.75   0.3500000        0.75   10    7    7  1.1e-13  3.8e-15
   2        0.73   0.3500000        0.73   11    8    8  4.3e-14  2.9e-15
   3        0.72   0.3500000        0.72   12    9    9  9.7e-14    3e-15
Velocity u2, Pressure p
Iter      SolEst     Damping    Stepsize #Res #Jac #Sol   LinErr   LinRes
   1        0.15   0.5000000        0.15    6    3    3  2.4e-15  7.9e-15
Displacement field
Iter      SolEst     Damping    Stepsize #Res #Jac #Sol   LinErr   LinRes
   1        0.52   0.5000000        0.52    6    3    3  1.1e-13  1.5e-12
Spatial mesh displacement
Iter      SolEst     Damping    Stepsize #Res #Jac #Sol   LinErr   LinRes
   1       1e-06   0.5000000       1e-06    6    3    3  1.6e-15  1.8e-15
Solution error estimates for segregated groups
1.2, 0.077, 0.26, 5.1e-07
Residual error estimates for segregated groups
2.2e+02, 7.7e+03, 1.6e+04, 0.16
Segregated solver iteration 4.
```

```
Merged variables
Iter      SolEst     Damping    Stepsize #Res #Jac #Sol   LinErr   LinRes
   1         0.7   0.3500000         0.7   14   10   10  7.3e-14  3.1e-15
   2        0.68   0.3500000        0.68   15   11   11  6.6e-14  2.9e-15
   3        0.59   0.3500000        0.59   16   12   12  7.4e-14  2.9e-15
Velocity u2, Pressure p
Iter      SolEst     Damping    Stepsize #Res #Jac #Sol   LinErr   LinRes
   1       0.072   0.5000000       0.072    8    4    4  4.7e-15  7.9e-15
Displacement field
Iter      SolEst     Damping    Stepsize #Res #Jac #Sol   LinErr   LinRes
   1        0.29   0.5000000        0.29    8    4    4  6.7e-14  1.5e-12
Spatial mesh displacement
Iter      SolEst     Damping    Stepsize #Res #Jac #Sol   LinErr   LinRes
   1     8.5e-07   0.5000000     8.5e-07    8    4    4  1.9e-15  1.8e-15
Solution error estimates for segregated groups
1, 0.036, 0.15, 4.3e-07
Residual error estimates for segregated groups
83, 4.1e+03, 1.1e+04, 0.16
Segregated solver iteration 5.
Merged variables
Iter      SolEst     Damping    Stepsize #Res #Jac #Sol   LinErr   LinRes
   1        0.41   0.3500000        0.41   18   13   13  8.5e-14  2.9e-15
   2        0.27   0.3500000        0.27   19   14   14  5.5e-14  2.9e-15
   3        0.18   0.3500000        0.18   20   15   15  8.7e-14  2.8e-15
Velocity u2, Pressure p
Iter      SolEst     Damping    Stepsize #Res #Jac #Sol   LinErr   LinRes
   1       0.035   0.5000000       0.035   10    5    5  2.7e-15  7.9e-15
Displacement field
Iter      SolEst     Damping    Stepsize #Res #Jac #Sol   LinErr   LinRes
   1        0.17   0.5000000        0.17   10    5    5  4.7e-14  1.5e-12
Spatial mesh displacement
Iter      SolEst     Damping    Stepsize #Res #Jac #Sol   LinErr   LinRes
   1     6.4e-07   0.5000000     6.4e-07   10    5    5  1.5e-15  1.9e-15
Solution error estimates for segregated groups
0.31, 0.017, 0.084, 3.2e-07
Residual error estimates for segregated groups
32, 2.1e+03, 6.8e+03, 0.17
Segregated solver iteration 6.
Merged variables
Iter      SolEst     Damping    Stepsize #Res #Jac #Sol   LinErr   LinRes
   1        0.12   0.3500000        0.12   22   16   16  7.6e-14  3.5e-15
   2       0.078   0.3500000       0.078   23   17   17  6.9e-14  3.6e-15
   3       0.051   0.3500000       0.051   24   18   18  9.3e-14  2.8e-15
Velocity u2, Pressure p
Iter      SolEst     Damping    Stepsize #Res #Jac #Sol   LinErr   LinRes
   1       0.017   0.5000000       0.017   12    6    6  2.8e-15  7.9e-15
Displacement field
Iter      SolEst     Damping    Stepsize #Res #Jac #Sol   LinErr   LinRes
   1       0.095   0.5000000       0.095   12    6    6  7.7e-14  1.5e-12
Spatial mesh displacement
Iter      SolEst     Damping    Stepsize #Res #Jac #Sol   LinErr   LinRes
   1     4.5e-07   0.5000000     4.5e-07   12    6    6  1.3e-15  1.9e-15
Solution error estimates for segregated groups
0.087, 0.0085, 0.048, 2.2e-07
Residual error estimates for segregated groups
12, 1.1e+03, 4.2e+03, 0.15
```

```
Segregated solver iteration 7.
Merged variables
Iter      SolEst     Damping    Stepsize #Res #Jac #Sol   LinErr   LinRes
   1       0.033   0.3500000       0.033   26   19   19  1.1e-13  3.1e-15
   2       0.022   0.3500000       0.022   27   20   20    1e-13  3.4e-15
   3       0.014   0.3500000       0.014   28   21   21    7e-14  2.9e-15
Velocity u2, Pressure p
Iter      SolEst     Damping    Stepsize #Res #Jac #Sol   LinErr   LinRes
   1      0.0085   0.5000000      0.0085   14    7    7    3e-15  8.1e-15
Displacement field
Iter      SolEst     Damping    Stepsize #Res #Jac #Sol   LinErr   LinRes
   1       0.054   0.5000000       0.054   14    7    7  9.8e-14  1.5e-12
Spatial mesh displacement
Iter      SolEst     Damping    Stepsize #Res #Jac #Sol   LinErr   LinRes
   1       3e-07   0.5000000       3e-07   14    7    7  2.1e-15  1.8e-15
Solution error estimates for segregated groups
0.024, 0.0042, 0.027, 1.5e-07
Residual error estimates for segregated groups
4.1, 5.3e+02, 2.6e+03, 0.16
Segregated solver iteration 8.
Merged variables
Iter      SolEst     Damping    Stepsize #Res #Jac #Sol   LinErr   LinRes
   1      0.0091   0.3500000      0.0091   30   22   22  5.3e-14    3e-15
   2      0.0059   0.3500000      0.0059   31   23   23  1.2e-13    3e-15
   3      0.0039   0.3500000      0.0039   32   24   24  8.4e-14  3.7e-15
Velocity u2, Pressure p
Iter      SolEst     Damping    Stepsize #Res #Jac #Sol   LinErr   LinRes
   1      0.0042   0.5000000      0.0042   16    8    8  4.8e-15  7.9e-15
Displacement field
Iter      SolEst     Damping    Stepsize #Res #Jac #Sol   LinErr   LinRes
   1        0.03   0.5000000        0.03   16    8    8  1.2e-13  1.5e-12
Spatial mesh displacement
Iter      SolEst     Damping    Stepsize #Res #Jac #Sol   LinErr   LinRes
   1     1.9e-07   0.5000000     1.9e-07   16    8    8  1.3e-15  1.8e-15
Solution error estimates for segregated groups
0.0066, 0.0021, 0.015, 9.6e-08
Residual error estimates for segregated groups
1.4, 2.7e+02, 1.8e+03, 0.15
Segregated solver iteration 9.
Merged variables
Iter      SolEst     Damping    Stepsize #Res #Jac #Sol   LinErr   LinRes
   1      0.0025   0.3500000      0.0025   34   25   25  1.8e-13    3e-15
   2      0.0016   0.3500000      0.0016   35   26   26  6.6e-14    3e-15
   3      0.0011   0.3500000      0.0011   36   27   27  6.1e-14  3.2e-15
Velocity u2, Pressure p
Iter      SolEst     Damping    Stepsize #Res #Jac #Sol   LinErr   LinRes
   1      0.0021   0.5000000      0.0021   18    9    9  3.2e-15  7.8e-15
Displacement field
Scales for dependent variables:
Auxiliary pressure (comp1.solid.lemm1.pw): 0.36
Displacement field (comp1.u): 2.8e-16
Iter      SolEst     Damping    Stepsize #Res #Jac #Sol   LinErr   LinRes
   1       0.017   0.5000000       0.017   18    9    9  1.2e-13  1.2e-12
Spatial mesh displacement
Iter      SolEst     Damping    Stepsize #Res #Jac #Sol   LinErr   LinRes
   1     1.2e-07   0.5000000     1.2e-07   18    9    9  1.6e-15  1.8e-15
```

```
Solution error estimates for segregated groups
0.0018, 0.0011, 0.0085, 6e-08
Residual error estimates for segregated groups
0.44, 1.3e+02, 1.4e+03, 0.16
Segregated solver iteration 10.
Merged variables
Iter      SolEst     Damping    Stepsize #Res #Jac #Sol   LinErr   LinRes
   1     0.00069   0.3500000     0.00069   38   28   28  9.3e-14  3.4e-15
   2     0.00045   0.3500000     0.00045   39   29   29  6.3e-14  2.8e-15
   3     0.00029   0.3500000     0.00029   40   30   30  1.2e-13    3e-15
Velocity u2, Pressure p
Iter      SolEst     Damping    Stepsize #Res #Jac #Sol   LinErr   LinRes
   1       0.001   0.5000000       0.001   20   10   10  4.2e-15  7.9e-15
Displacement field
Iter      SolEst     Damping    Stepsize #Res #Jac #Sol   LinErr   LinRes
   1      0.0094   0.5000000      0.0094   20   10   10  1.5e-13  1.2e-12
Spatial mesh displacement
Iter      SolEst     Damping    Stepsize #Res #Jac #Sol   LinErr   LinRes
   1     7.3e-08   0.5000000     7.3e-08   20   10   10  1.2e-15  1.8e-15
Solution error estimates for segregated groups
0.0005, 0.00052, 0.0047, 3.7e-08
Residual error estimates for segregated groups
0.14, 67, 1.2e+03, 0.16
Segregated solver iteration 11.
Merged variables
Iter      SolEst     Damping    Stepsize #Res #Jac #Sol   LinErr   LinRes
   1     0.00019   0.3500000     0.00019   42   31   31  1.1e-13    3e-15
   2     0.00012   0.3500000     0.00012   43   32   32  1.1e-13  2.8e-15
   3       8e-05   0.3500000       8e-05   44   33   33  7.5e-14  2.8e-15
Velocity u2, Pressure p
Iter      SolEst     Damping    Stepsize #Res #Jac #Sol   LinErr   LinRes
   1     0.00052   0.5000000     0.00052   22   11   11  2.2e-15  7.9e-15
Displacement field
Iter      SolEst     Damping    Stepsize #Res #Jac #Sol   LinErr   LinRes
   1      0.0051   0.5000000      0.0051   22   11   11    1e-13  1.2e-12
Spatial mesh displacement
Iter      SolEst     Damping    Stepsize #Res #Jac #Sol   LinErr   LinRes
   1     4.4e-08   0.5000000     4.4e-08   22   11   11  1.3e-15  1.8e-15
Solution error estimates for segregated groups
0.00014, 0.00026, 0.0026, 2.2e-08
Residual error estimates for segregated groups
0.043, 33, 1.2e+03, 0.16
Segregated solver iteration 12.
Merged variables
Iter      SolEst     Damping    Stepsize #Res #Jac #Sol   LinErr   LinRes
   1     5.2e-05   0.3500000     5.2e-05   46   34   34    1e-13  2.8e-15
   2     3.4e-05   0.3500000     3.4e-05   47   35   35  1.3e-13  3.2e-15
   3     2.2e-05   0.3500000     2.2e-05   48   36   36  8.2e-14  4.1e-15
Velocity u2, Pressure p
Iter      SolEst     Damping    Stepsize #Res #Jac #Sol   LinErr   LinRes
   1     0.00026   0.5000000     0.00026   24   12   12  2.8e-15  7.9e-15
Displacement field
Iter      SolEst     Damping    Stepsize #Res #Jac #Sol   LinErr   LinRes
   1      0.0028   0.5000000      0.0028   24   12   12  1.6e-13  1.2e-12
Spatial mesh displacement
Iter      SolEst     Damping    Stepsize #Res #Jac #Sol   LinErr   LinRes
```

```
   1     2.6e-08   0.5000000     2.6e-08   24    12    12  1.5e-15  1.8e-15
Solution error estimates for segregated groups
3.8e-05, 0.00013, 0.0014, 1.3e-08
Residual error estimates for segregated groups
0.013, 17, 1.2e+03, 0.17
Segregated solver iteration 13.
Merged variables
Iter      SolEst     Damping    Stepsize #Res #Jac #Sol   LinErr   LinRes
   1     1.4e-05   0.3500000     1.4e-05   50    37    37  5.8e-14  2.9e-15
   2     9.3e-06   0.3500000     9.3e-06   51    38    38  1.1e-13  2.9e-15
   3       6e-06   0.3500000       6e-06   52    39    39  1.7e-13  3.1e-15
Velocity u2, Pressure p
Iter      SolEst     Damping    Stepsize #Res #Jac #Sol   LinErr   LinRes
   1     0.00013   0.5000000     0.00013   26    13    13  5.6e-15  7.9e-15
Displacement field
Iter      SolEst     Damping    Stepsize #Res #Jac #Sol   LinErr   LinRes
   1      0.0015   0.5000000      0.0015   26    13    13  1.1e-13  1.2e-12
Spatial mesh displacement
Iter      SolEst     Damping    Stepsize #Res #Jac #Sol   LinErr   LinRes
   1     1.5e-08   0.5000000     1.5e-08   26    13    13  1.6e-15  1.8e-15
Solution error estimates for segregated groups
1e-05, 6.6e-05, 0.00076, 7.6e-09
Residual error estimates for segregated groups
0.004, 8.3, 1.2e+03, 0.16
Solution time: 154 s. (2 minutes, 34 seconds)
Physical memory: 4.66 GB
Virtual memory: 25.49 GB
Ended at Jan 31, 2025, 7:28:30 PM.
----- Stationary Solver 1 in study1/Approach Curve Solver (sol1) -------------->
```

d=2215 (su59)

GENERAL

Description	Value
Solution	d=2215

LOG

```
<---- Compile Equations: Stationary in study1/Approach Curve Solver (sol1) -----
Started at Jan 31, 2025, 7:28:30 PM.
Parameter d = 2.215E-6 (m).
Geometry shape function: Linear serendipity
Number of vertex elements: 18
Number of boundary elements: 6187
Number of elements: 155477
Minimum element quality: 0.4766
Time: 8 s.
Physical memory: 2.96 GB
Virtual memory: 15.28 GB
Ended at Jan 31, 2025, 7:28:38 PM.
----- Compile Equations: Stationary in study1/Approach Curve Solver (sol1) ---->

<---- Dependent Variables 1 in study1/Approach Curve Solver (sol1) -------------
Started at Jan 31, 2025, 7:28:38 PM.
Solution time: 0 s.
Physical memory: 2.96 GB
Virtual memory: 15.22 GB
Ended at Jan 31, 2025, 7:28:38 PM.
----- Dependent Variables 1 in study1/Approach Curve Solver (sol1) ------------>

<---- Stationary Solver 1 in study1/Approach Curve Solver (sol1) ---------------
Started at Jan 31, 2025, 7:28:38 PM.
Segregated solver
Segregated Step 11 does not have any components solved for.
Number of degrees of freedom solved for: 789830 (plus 11797 internal DOFs).
Segregated solver iteration 1.
Merged variables
Nonsymmetric matrix found.
Scales for dependent variables:
Concentration (comp1.Cl): 1.4e+02
Concentration (comp1.K): 1.4e+02
Electric potential (comp1.V): 1.9e+02
Orthonormal null-space function used.
Iter      SolEst     Damping    Stepsize #Res #Jac #Sol   LinErr   LinRes
   1        0.83   0.3500000        0.83    2    1    1  2.7e-11  3.1e-15
   2        0.81   0.3500000        0.81    3    2    2  2.5e-11  2.7e-15
   3         0.8   0.3500000         0.8    4    3    3  4.2e-11  2.7e-15
Velocity u2, Pressure p
Nonsymmetric matrix found.
Scales for dependent variables:
Pressure (comp1.p): 1
Velocity field (spatial frame) (comp1.u2): 0.00018
Orthonormal null-space function used.
Iter      SolEst     Damping    Stepsize #Res #Jac #Sol   LinErr   LinRes
   1         1.1   0.5000000         1.1    2    1    1  1.8e-15  2.3e-15
Displacement field
Nonsymmetric matrix found.
Scales for dependent variables:
Auxiliary pressure (comp1.solid.lemm1.pw): 1
Displacement field (comp1.u): 2.8e-18
Orthonormal null-space function used.
Iter      SolEst     Damping    Stepsize #Res #Jac #Sol   LinErr   LinRes
   1         1.9   0.5000000         1.9    2    1    1  3.8e-14  1.5e-12
Spatial mesh displacement
```

```
Symmetric matrices found.
Scales for dependent variables:
Spatial mesh displacement (comp1.spatial.disp): 1.2e-09
Orthonormal null-space function used.
Iter      SolEst     Damping    Stepsize #Res #Jac #Sol   LinErr   LinRes
   1     6.9e-07   0.5000000     6.9e-07    2    1    1  1.2e-15  1.9e-15
Solution error estimates for segregated groups
1.4, 0.54, 0.95, 3.5e-07
Residual error estimates for segregated groups
7.9e+03, 8.2e+04, 2.2e+04, 0.14
Segregated solver iteration 2.
Merged variables
Scales for dependent variables:
Concentration (comp1.Cl): 1.4e+02
Concentration (comp1.K): 1.4e+02
Electric potential (comp1.V): 0.064
Iter      SolEst     Damping    Stepsize #Res #Jac #Sol   LinErr   LinRes
   1        0.78   0.3500000        0.78    6    4    4  1.1e-13  3.1e-15
   2        0.77   0.3500000        0.77    7    5    5  7.6e-14  3.9e-15
   3        0.76   0.3500000        0.76    8    6    6  8.5e-14  3.3e-15
Velocity u2, Pressure p
Scales for dependent variables:
Pressure (comp1.p): 3.6e+02
Velocity field (spatial frame) (comp1.u2): 0.00072
Iter      SolEst     Damping    Stepsize #Res #Jac #Sol   LinErr   LinRes
   1        0.36   0.5000000        0.36    4    2    2  2.1e-15  7.8e-15
Displacement field
Iter      SolEst     Damping    Stepsize #Res #Jac #Sol   LinErr   LinRes
   1        0.95   0.5000000        0.95    4    2    2  8.5e-14  1.5e-12
Spatial mesh displacement
Iter      SolEst     Damping    Stepsize #Res #Jac #Sol   LinErr   LinRes
   1       1e-06   0.5000000       1e-06    4    2    2  1.4e-15  1.9e-15
Solution error estimates for segregated groups
1.3, 0.18, 0.48, 5.2e-07
Residual error estimates for segregated groups
7.9e+02, 1.4e+04, 2.2e+04, 0.15
Segregated solver iteration 3.
Merged variables
Iter      SolEst     Damping    Stepsize #Res #Jac #Sol   LinErr   LinRes
   1        0.75   0.3500000        0.75   10    7    7  5.4e-14    3e-15
   2        0.73   0.3500000        0.73   11    8    8  1.1e-13  3.1e-15
   3        0.72   0.3500000        0.72   12    9    9  4.6e-14  3.1e-15
Velocity u2, Pressure p
Iter      SolEst     Damping    Stepsize #Res #Jac #Sol   LinErr   LinRes
   1        0.15   0.5000000        0.15    6    3    3  2.1e-15    8e-15
Displacement field
Iter      SolEst     Damping    Stepsize #Res #Jac #Sol   LinErr   LinRes
   1        0.52   0.5000000        0.52    6    3    3  3.1e-14  1.5e-12
Spatial mesh displacement
Iter      SolEst     Damping    Stepsize #Res #Jac #Sol   LinErr   LinRes
   1       1e-06   0.5000000       1e-06    6    3    3  1.5e-15  1.9e-15
Solution error estimates for segregated groups
1.2, 0.077, 0.26, 5.2e-07
Residual error estimates for segregated groups
2.2e+02, 8e+03, 1.6e+04, 0.16
Segregated solver iteration 4.
```

```
Merged variables
Iter      SolEst     Damping    Stepsize #Res #Jac #Sol   LinErr   LinRes
   1         0.7   0.3500000         0.7   14   10   10  1.1e-13  3.5e-15
   2        0.68   0.3500000        0.68   15   11   11  6.8e-14  2.9e-15
   3        0.59   0.3500000        0.59   16   12   12  7.2e-14  3.4e-15
Velocity u2, Pressure p
Iter      SolEst     Damping    Stepsize #Res #Jac #Sol   LinErr   LinRes
   1       0.072   0.5000000       0.072    8    4    4  2.4e-15  7.8e-15
Displacement field
Iter      SolEst     Damping    Stepsize #Res #Jac #Sol   LinErr   LinRes
   1        0.29   0.5000000        0.29    8    4    4  1.1e-13  1.5e-12
Spatial mesh displacement
Iter      SolEst     Damping    Stepsize #Res #Jac #Sol   LinErr   LinRes
   1     8.6e-07   0.5000000     8.6e-07    8    4    4    2e-15  1.8e-15
Solution error estimates for segregated groups
1, 0.036, 0.15, 4.3e-07
Residual error estimates for segregated groups
83, 4.2e+03, 1.1e+04, 0.15
Segregated solver iteration 5.
Merged variables
Iter      SolEst     Damping    Stepsize #Res #Jac #Sol   LinErr   LinRes
   1        0.41   0.3500000        0.41   18   13   13  6.7e-14  3.2e-15
   2        0.27   0.3500000        0.27   19   14   14  8.6e-14    3e-15
   3        0.18   0.3500000        0.18   20   15   15    9e-14  3.4e-15
Velocity u2, Pressure p
Iter      SolEst     Damping    Stepsize #Res #Jac #Sol   LinErr   LinRes
   1       0.035   0.5000000       0.035   10    5    5  2.9e-15  7.9e-15
Displacement field
Iter      SolEst     Damping    Stepsize #Res #Jac #Sol   LinErr   LinRes
   1        0.17   0.5000000        0.17   10    5    5  4.6e-14  1.5e-12
Spatial mesh displacement
Iter      SolEst     Damping    Stepsize #Res #Jac #Sol   LinErr   LinRes
   1     6.5e-07   0.5000000     6.5e-07   10    5    5  1.6e-15  1.9e-15
Solution error estimates for segregated groups
0.31, 0.017, 0.084, 3.2e-07
Residual error estimates for segregated groups
32, 2.2e+03, 6.8e+03, 0.15
Segregated solver iteration 6.
Merged variables
Iter      SolEst     Damping    Stepsize #Res #Jac #Sol   LinErr   LinRes
   1        0.12   0.3500000        0.12   22   16   16  7.3e-14  3.5e-15
   2       0.078   0.3500000       0.078   23   17   17  6.4e-14  3.3e-15
   3       0.051   0.3500000       0.051   24   18   18  1.6e-13  2.9e-15
Velocity u2, Pressure p
Iter      SolEst     Damping    Stepsize #Res #Jac #Sol   LinErr   LinRes
   1       0.017   0.5000000       0.017   12    6    6  3.4e-15  7.8e-15
Displacement field
Iter      SolEst     Damping    Stepsize #Res #Jac #Sol   LinErr   LinRes
   1       0.095   0.5000000       0.095   12    6    6  2.7e-14  1.5e-12
Spatial mesh displacement
Iter      SolEst     Damping    Stepsize #Res #Jac #Sol   LinErr   LinRes
   1     4.5e-07   0.5000000     4.5e-07   12    6    6  1.3e-15  1.8e-15
Solution error estimates for segregated groups
0.087, 0.0085, 0.048, 2.3e-07
Residual error estimates for segregated groups
12, 1.1e+03, 4.2e+03, 0.16
```

```
Segregated solver iteration 7.
Merged variables
Iter      SolEst     Damping    Stepsize #Res #Jac #Sol   LinErr   LinRes
   1       0.033   0.3500000       0.033   26   19   19  9.1e-14  3.4e-15
   2       0.022   0.3500000       0.022   27   20   20  9.5e-14  3.4e-15
   3       0.014   0.3500000       0.014   28   21   21  1.1e-13    3e-15
Velocity u2, Pressure p
Iter      SolEst     Damping    Stepsize #Res #Jac #Sol   LinErr   LinRes
   1      0.0085   0.5000000      0.0085   14    7    7  2.3e-15  7.8e-15
Displacement field
Iter      SolEst     Damping    Stepsize #Res #Jac #Sol   LinErr   LinRes
   1       0.054   0.5000000       0.054   14    7    7  3.5e-14  1.5e-12
Spatial mesh displacement
Iter      SolEst     Damping    Stepsize #Res #Jac #Sol   LinErr   LinRes
   1       3e-07   0.5000000       3e-07   14    7    7  1.4e-15  1.9e-15
Solution error estimates for segregated groups
0.024, 0.0042, 0.027, 1.5e-07
Residual error estimates for segregated groups
4.1, 5.5e+02, 2.6e+03, 0.15
Segregated solver iteration 8.
Merged variables
Iter      SolEst     Damping    Stepsize #Res #Jac #Sol   LinErr   LinRes
   1      0.0091   0.3500000      0.0091   30   22   22  1.1e-13    3e-15
   2      0.0059   0.3500000      0.0059   31   23   23  8.5e-14  3.4e-15
   3      0.0039   0.3500000      0.0039   32   24   24  5.3e-14  3.8e-15
Velocity u2, Pressure p
Iter      SolEst     Damping    Stepsize #Res #Jac #Sol   LinErr   LinRes
   1      0.0042   0.5000000      0.0042   16    8    8  3.6e-15  7.9e-15
Displacement field
Scales for dependent variables:
Auxiliary pressure (comp1.solid.lemm1.pw): 0.35
Displacement field (comp1.u): 2.8e-16
Iter      SolEst     Damping    Stepsize #Res #Jac #Sol   LinErr   LinRes
   1        0.03   0.5000000        0.03   16    8    8  1.6e-13  1.2e-12
Spatial mesh displacement
Iter      SolEst     Damping    Stepsize #Res #Jac #Sol   LinErr   LinRes
   1     1.9e-07   0.5000000     1.9e-07   16    8    8  1.6e-15  1.8e-15
Solution error estimates for segregated groups
0.0066, 0.0021, 0.015, 9.7e-08
Residual error estimates for segregated groups
1.4, 2.8e+02, 1.8e+03, 0.16
Segregated solver iteration 9.
Merged variables
Iter      SolEst     Damping    Stepsize #Res #Jac #Sol   LinErr   LinRes
   1      0.0025   0.3500000      0.0025   34   25   25  8.7e-14    3e-15
   2      0.0016   0.3500000      0.0016   35   26   26  8.8e-14  3.5e-15
   3      0.0011   0.3500000      0.0011   36   27   27    8e-14  3.4e-15
Velocity u2, Pressure p
Iter      SolEst     Damping    Stepsize #Res #Jac #Sol   LinErr   LinRes
   1      0.0021   0.5000000      0.0021   18    9    9  2.2e-15  7.9e-15
Displacement field
Iter      SolEst     Damping    Stepsize #Res #Jac #Sol   LinErr   LinRes
   1       0.017   0.5000000       0.017   18    9    9  1.3e-13  1.2e-12
Spatial mesh displacement
Iter      SolEst     Damping    Stepsize #Res #Jac #Sol   LinErr   LinRes
   1     1.2e-07   0.5000000     1.2e-07   18    9    9  1.7e-15  1.9e-15
```

```
Solution error estimates for segregated groups
0.0018, 0.0011, 0.0085, 6.1e-08
Residual error estimates for segregated groups
0.44, 1.4e+02, 1.4e+03, 0.15
Segregated solver iteration 10.
Merged variables
Iter      SolEst     Damping    Stepsize #Res #Jac #Sol   LinErr   LinRes
   1     0.00069   0.3500000     0.00069   38   28   28  8.8e-14  2.8e-15
   2     0.00045   0.3500000     0.00045   39   29   29  5.8e-14  3.1e-15
   3     0.00029   0.3500000     0.00029   40   30   30  8.7e-14  3.6e-15
Velocity u2, Pressure p
Iter      SolEst     Damping    Stepsize #Res #Jac #Sol   LinErr   LinRes
   1       0.001   0.5000000       0.001   20   10   10  3.5e-15  7.9e-15
Displacement field
Iter      SolEst     Damping    Stepsize #Res #Jac #Sol   LinErr   LinRes
   1      0.0094   0.5000000      0.0094   20   10   10  1.1e-13  1.2e-12
Spatial mesh displacement
Iter      SolEst     Damping    Stepsize #Res #Jac #Sol   LinErr   LinRes
   1     7.4e-08   0.5000000     7.4e-08   20   10   10    2e-15  1.9e-15
Solution error estimates for segregated groups
0.0005, 0.00052, 0.0047, 3.7e-08
Residual error estimates for segregated groups
0.14, 69, 1.2e+03, 0.15
Segregated solver iteration 11.
Merged variables
Iter      SolEst     Damping    Stepsize #Res #Jac #Sol   LinErr   LinRes
   1     0.00019   0.3500000     0.00019   42   31   31  6.6e-14    3e-15
   2     0.00012   0.3500000     0.00012   43   32   32  1.4e-13  3.2e-15
   3       8e-05   0.3500000       8e-05   44   33   33  7.7e-14  3.1e-15
Velocity u2, Pressure p
Iter      SolEst     Damping    Stepsize #Res #Jac #Sol   LinErr   LinRes
   1     0.00052   0.5000000     0.00052   22   11   11  2.6e-15  7.9e-15
Displacement field
Iter      SolEst     Damping    Stepsize #Res #Jac #Sol   LinErr   LinRes
   1      0.0051   0.5000000      0.0051   22   11   11  1.2e-13  1.2e-12
Spatial mesh displacement
Iter      SolEst     Damping    Stepsize #Res #Jac #Sol   LinErr   LinRes
   1     4.5e-08   0.5000000     4.5e-08   22   11   11  1.6e-15  1.9e-15
Solution error estimates for segregated groups
0.00014, 0.00026, 0.0026, 2.2e-08
Residual error estimates for segregated groups
0.043, 35, 1.2e+03, 0.17
Segregated solver iteration 12.
Merged variables
Iter      SolEst     Damping    Stepsize #Res #Jac #Sol   LinErr   LinRes
   1     5.2e-05   0.3500000     5.2e-05   46   34   34  6.4e-14  3.5e-15
   2     3.4e-05   0.3500000     3.4e-05   47   35   35  8.6e-14  3.1e-15
   3     2.2e-05   0.3500000     2.2e-05   48   36   36  5.6e-14  3.5e-15
Velocity u2, Pressure p
Iter      SolEst     Damping    Stepsize #Res #Jac #Sol   LinErr   LinRes
   1     0.00026   0.5000000     0.00026   24   12   12  2.6e-15  7.8e-15
Displacement field
Iter      SolEst     Damping    Stepsize #Res #Jac #Sol   LinErr   LinRes
   1      0.0028   0.5000000      0.0028   24   12   12  1.2e-13  1.2e-12
Spatial mesh displacement
Iter      SolEst     Damping    Stepsize #Res #Jac #Sol   LinErr   LinRes
```

```
   1     2.6e-08   0.5000000     2.6e-08   24    12    12  1.7e-15  1.8e-15
Solution error estimates for segregated groups
3.8e-05, 0.00013, 0.0014, 1.3e-08
Residual error estimates for segregated groups
0.013, 17, 1.2e+03, 0.16
Segregated solver iteration 13.
Merged variables
Iter      SolEst     Damping    Stepsize #Res #Jac #Sol   LinErr   LinRes
   1     1.4e-05   0.3500000     1.4e-05   50    37    37  5.1e-14  3.2e-15
   2     9.3e-06   0.3500000     9.3e-06   51    38    38    7e-14  3.1e-15
   3       6e-06   0.3500000       6e-06   52    39    39  8.3e-14    3e-15
Velocity u2, Pressure p
Iter      SolEst     Damping    Stepsize #Res #Jac #Sol   LinErr   LinRes
   1     0.00013   0.5000000     0.00013   26    13    13  2.9e-15  7.9e-15
Displacement field
Iter      SolEst     Damping    Stepsize #Res #Jac #Sol   LinErr   LinRes
   1      0.0015   0.5000000      0.0015   26    13    13  1.2e-13  1.2e-12
Spatial mesh displacement
Iter      SolEst     Damping    Stepsize #Res #Jac #Sol   LinErr   LinRes
   1     1.5e-08   0.5000000     1.5e-08   26    13    13  1.5e-15  1.9e-15
Solution error estimates for segregated groups
1e-05, 6.6e-05, 0.00076, 7.7e-09
Residual error estimates for segregated groups
0.004, 8.7, 1.2e+03, 0.15
Solution time: 154 s. (2 minutes, 34 seconds)
Physical memory: 4.69 GB
Virtual memory: 26.06 GB
Ended at Jan 31, 2025, 7:31:13 PM.
----- Stationary Solver 1 in study1/Approach Curve Solver (sol1) -------------->
```

d=2210 (su60)

GENERAL

Description	Value
Solution	d=2210

LOG

```
<---- Compile Equations: Stationary in study1/Approach Curve Solver (sol1) -----
Started at Jan 31, 2025, 7:31:13 PM.
Parameter d = 2.21E-6 (m).
Geometry shape function: Linear serendipity
Number of vertex elements: 18
Number of boundary elements: 6184
Number of elements: 155436
Minimum element quality: 0.4738
Time: 8 s.
Physical memory: 2.97 GB
Virtual memory: 15.29 GB
Ended at Jan 31, 2025, 7:31:21 PM.
----- Compile Equations: Stationary in study1/Approach Curve Solver (sol1) ---->

<---- Dependent Variables 1 in study1/Approach Curve Solver (sol1) -------------
Started at Jan 31, 2025, 7:31:21 PM.
Solution time: 0 s.
Physical memory: 2.97 GB
Virtual memory: 15.22 GB
Ended at Jan 31, 2025, 7:31:21 PM.
----- Dependent Variables 1 in study1/Approach Curve Solver (sol1) ------------>

<---- Stationary Solver 1 in study1/Approach Curve Solver (sol1) ---------------
Started at Jan 31, 2025, 7:31:21 PM.
Segregated solver
Segregated Step 11 does not have any components solved for.
Number of degrees of freedom solved for: 789685 (plus 11791 internal DOFs).
Segregated solver iteration 1.
Merged variables
Nonsymmetric matrix found.
Scales for dependent variables:
Concentration (comp1.Cl): 1.4e+02
Concentration (comp1.K): 1.4e+02
Electric potential (comp1.V): 1.9e+02
Orthonormal null-space function used.
Iter      SolEst     Damping    Stepsize #Res #Jac #Sol   LinErr   LinRes
   1        0.83   0.3500000        0.83    2    1    1  1.6e-11  2.8e-15
   2        0.81   0.3500000        0.81    3    2    2  3.9e-11  2.7e-15
   3         0.8   0.3500000         0.8    4    3    3  2.6e-11  2.7e-15
Velocity u2, Pressure p
Nonsymmetric matrix found.
Scales for dependent variables:
Pressure (comp1.p): 1
Velocity field (spatial frame) (comp1.u2): 0.00018
Orthonormal null-space function used.
Iter      SolEst     Damping    Stepsize #Res #Jac #Sol   LinErr   LinRes
   1         1.1   0.5000000         1.1    2    1    1    2e-15  2.2e-15
Displacement field
Nonsymmetric matrix found.
Scales for dependent variables:
Auxiliary pressure (comp1.solid.lemm1.pw): 1
Displacement field (comp1.u): 2.8e-18
Orthonormal null-space function used.
Iter      SolEst     Damping    Stepsize #Res #Jac #Sol   LinErr   LinRes
   1         1.9   0.5000000         1.9    2    1    1    4e-14  1.5e-12
Spatial mesh displacement
```

```
Symmetric matrices found.
Scales for dependent variables:
Spatial mesh displacement (comp1.spatial.disp): 1.2e-09
Orthonormal null-space function used.
Iter      SolEst     Damping    Stepsize #Res #Jac #Sol   LinErr   LinRes
   1     6.9e-07   0.5000000     6.9e-07    2    1    1  1.8e-15  1.8e-15
Solution error estimates for segregated groups
1.4, 0.54, 0.95, 3.5e-07
Residual error estimates for segregated groups
7.9e+03, 8.2e+04, 2.2e+04, 0.14
Segregated solver iteration 2.
Merged variables
Scales for dependent variables:
Concentration (comp1.Cl): 1.4e+02
Concentration (comp1.K): 1.4e+02
Electric potential (comp1.V): 0.064
Iter      SolEst     Damping    Stepsize #Res #Jac #Sol   LinErr   LinRes
   1        0.78   0.3500000        0.78    6    4    4  6.3e-14  2.9e-15
   2        0.77   0.3500000        0.77    7    5    5  9.3e-14    3e-15
   3        0.76   0.3500000        0.76    8    6    6  4.6e-14  2.8e-15
Velocity u2, Pressure p
Scales for dependent variables:
Pressure (comp1.p): 3.6e+02
Velocity field (spatial frame) (comp1.u2): 0.00072
Iter      SolEst     Damping    Stepsize #Res #Jac #Sol   LinErr   LinRes
   1        0.36   0.5000000        0.36    4    2    2  3.7e-15  7.8e-15
Displacement field
Iter      SolEst     Damping    Stepsize #Res #Jac #Sol   LinErr   LinRes
   1        0.95   0.5000000        0.95    4    2    2  1.4e-13  1.6e-12
Spatial mesh displacement
Iter      SolEst     Damping    Stepsize #Res #Jac #Sol   LinErr   LinRes
   1       1e-06   0.5000000       1e-06    4    2    2  1.4e-15  1.9e-15
Solution error estimates for segregated groups
1.3, 0.18, 0.48, 5.2e-07
Residual error estimates for segregated groups
7.8e+02, 1.3e+04, 2.2e+04, 0.16
Segregated solver iteration 3.
Merged variables
Iter      SolEst     Damping    Stepsize #Res #Jac #Sol   LinErr   LinRes
   1        0.75   0.3500000        0.75   10    7    7  1.8e-13  3.5e-15
   2        0.73   0.3500000        0.73   11    8    8  1.6e-13  3.3e-15
   3        0.72   0.3500000        0.72   12    9    9  1.6e-13  3.7e-15
Velocity u2, Pressure p
Iter      SolEst     Damping    Stepsize #Res #Jac #Sol   LinErr   LinRes
   1        0.15   0.5000000        0.15    6    3    3  3.4e-15  7.7e-15
Displacement field
Iter      SolEst     Damping    Stepsize #Res #Jac #Sol   LinErr   LinRes
   1        0.52   0.5000000        0.52    6    3    3  4.3e-14  1.5e-12
Spatial mesh displacement
Iter      SolEst     Damping    Stepsize #Res #Jac #Sol   LinErr   LinRes
   1       1e-06   0.5000000       1e-06    6    3    3  2.1e-15  1.8e-15
Solution error estimates for segregated groups
1.2, 0.077, 0.26, 5.2e-07
Residual error estimates for segregated groups
2.2e+02, 7.3e+03, 1.6e+04, 0.15
Segregated solver iteration 4.
```

```
Merged variables
Iter      SolEst     Damping    Stepsize #Res #Jac #Sol   LinErr   LinRes
   1         0.7   0.3500000         0.7   14   10   10  3.2e-13  6.6e-15
   2        0.68   0.3500000        0.68   15   11   11  8.2e-14  3.6e-15
   3        0.59   0.3500000        0.59   16   12   12  5.8e-14  3.6e-15
Velocity u2, Pressure p
Iter      SolEst     Damping    Stepsize #Res #Jac #Sol   LinErr   LinRes
   1       0.072   0.5000000       0.072    8    4    4  2.5e-15  7.9e-15
Displacement field
Iter      SolEst     Damping    Stepsize #Res #Jac #Sol   LinErr   LinRes
   1        0.29   0.5000000        0.29    8    4    4  6.4e-14  1.5e-12
Spatial mesh displacement
Iter      SolEst     Damping    Stepsize #Res #Jac #Sol   LinErr   LinRes
   1     8.6e-07   0.5000000     8.6e-07    8    4    4    2e-15  1.8e-15
Solution error estimates for segregated groups
1, 0.036, 0.15, 4.3e-07
Residual error estimates for segregated groups
83, 3.8e+03, 1.1e+04, 0.15
Segregated solver iteration 5.
Merged variables
Iter      SolEst     Damping    Stepsize #Res #Jac #Sol   LinErr   LinRes
   1        0.41   0.3500000        0.41   18   13   13  2.1e-13  5.6e-15
   2        0.27   0.3500000        0.27   19   14   14  7.7e-14  7.2e-15
   3        0.18   0.3500000        0.18   20   15   15  1.2e-13  3.7e-15
Velocity u2, Pressure p
Iter      SolEst     Damping    Stepsize #Res #Jac #Sol   LinErr   LinRes
   1       0.035   0.5000000       0.035   10    5    5  3.4e-15  7.9e-15
Displacement field
Iter      SolEst     Damping    Stepsize #Res #Jac #Sol   LinErr   LinRes
   1        0.17   0.5000000        0.17   10    5    5    6e-14  1.5e-12
Spatial mesh displacement
Iter      SolEst     Damping    Stepsize #Res #Jac #Sol   LinErr   LinRes
   1     6.5e-07   0.5000000     6.5e-07   10    5    5  1.6e-15  1.9e-15
Solution error estimates for segregated groups
0.31, 0.017, 0.084, 3.2e-07
Residual error estimates for segregated groups
32, 2e+03, 6.9e+03, 0.17
Segregated solver iteration 6.
Merged variables
Iter      SolEst     Damping    Stepsize #Res #Jac #Sol   LinErr   LinRes
   1        0.12   0.3500000        0.12   22   16   16  6.6e-14  4.4e-15
   2       0.078   0.3500000       0.078   23   17   17  1.2e-13  4.2e-15
   3       0.051   0.3500000       0.051   24   18   18  2.4e-13  4.7e-15
Velocity u2, Pressure p
Iter      SolEst     Damping    Stepsize #Res #Jac #Sol   LinErr   LinRes
   1       0.017   0.5000000       0.017   12    6    6  2.3e-15  7.8e-15
Displacement field
Iter      SolEst     Damping    Stepsize #Res #Jac #Sol   LinErr   LinRes
   1       0.095   0.5000000       0.095   12    6    6    4e-14  1.5e-12
Spatial mesh displacement
Iter      SolEst     Damping    Stepsize #Res #Jac #Sol   LinErr   LinRes
   1     4.5e-07   0.5000000     4.5e-07   12    6    6  1.6e-15  1.8e-15
Solution error estimates for segregated groups
0.087, 0.0085, 0.048, 2.3e-07
Residual error estimates for segregated groups
12, 1e+03, 4.2e+03, 0.16
```

```
Segregated solver iteration 7.
Merged variables
Iter      SolEst     Damping    Stepsize #Res #Jac #Sol   LinErr   LinRes
   1       0.033   0.3500000       0.033   26   19   19  1.3e-13  3.7e-15
   2       0.022   0.3500000       0.022   27   20   20  1.3e-13  3.2e-15
   3       0.014   0.3500000       0.014   28   21   21    2e-13    4e-15
Velocity u2, Pressure p
Iter      SolEst     Damping    Stepsize #Res #Jac #Sol   LinErr   LinRes
   1      0.0085   0.5000000      0.0085   14    7    7  2.9e-15  7.8e-15
Displacement field
Iter      SolEst     Damping    Stepsize #Res #Jac #Sol   LinErr   LinRes
   1       0.054   0.5000000       0.054   14    7    7  2.9e-14  1.5e-12
Spatial mesh displacement
Iter      SolEst     Damping    Stepsize #Res #Jac #Sol   LinErr   LinRes
   1       3e-07   0.5000000       3e-07   14    7    7  1.4e-15  1.9e-15
Solution error estimates for segregated groups
0.024, 0.0042, 0.027, 1.5e-07
Residual error estimates for segregated groups
4.1, 5e+02, 2.6e+03, 0.15
Segregated solver iteration 8.
Merged variables
Iter      SolEst     Damping    Stepsize #Res #Jac #Sol   LinErr   LinRes
   1      0.0091   0.3500000      0.0091   30   22   22  2.7e-13    5e-15
   2      0.0059   0.3500000      0.0059   31   23   23    7e-14  2.8e-15
   3      0.0039   0.3500000      0.0039   32   24   24  1.8e-13    5e-15
Velocity u2, Pressure p
Iter      SolEst     Damping    Stepsize #Res #Jac #Sol   LinErr   LinRes
   1      0.0042   0.5000000      0.0042   16    8    8  2.8e-15  7.8e-15
Displacement field
Iter      SolEst     Damping    Stepsize #Res #Jac #Sol   LinErr   LinRes
   1        0.03   0.5000000        0.03   16    8    8  7.7e-14  1.5e-12
Spatial mesh displacement
Iter      SolEst     Damping    Stepsize #Res #Jac #Sol   LinErr   LinRes
   1     1.9e-07   0.5000000     1.9e-07   16    8    8    2e-15  1.8e-15
Solution error estimates for segregated groups
0.0066, 0.0021, 0.015, 9.7e-08
Residual error estimates for segregated groups
1.4, 2.5e+02, 1.8e+03, 0.15
Segregated solver iteration 9.
Merged variables
Iter      SolEst     Damping    Stepsize #Res #Jac #Sol   LinErr   LinRes
   1      0.0025   0.3500000      0.0025   34   25   25  3.3e-13  6.5e-15
   2      0.0016   0.3500000      0.0016   35   26   26  1.2e-13    3e-15
   3      0.0011   0.3500000      0.0011   36   27   27  6.8e-14    5e-15
Velocity u2, Pressure p
Iter      SolEst     Damping    Stepsize #Res #Jac #Sol   LinErr   LinRes
   1      0.0021   0.5000000      0.0021   18    9    9  2.1e-15    8e-15
Displacement field
Scales for dependent variables:
Auxiliary pressure (comp1.solid.lemm1.pw): 0.36
Displacement field (comp1.u): 2.8e-16
Iter      SolEst     Damping    Stepsize #Res #Jac #Sol   LinErr   LinRes
   1       0.017   0.5000000       0.017   18    9    9  1.2e-13  1.2e-12
Spatial mesh displacement
Iter      SolEst     Damping    Stepsize #Res #Jac #Sol   LinErr   LinRes
   1     1.2e-07   0.5000000     1.2e-07   18    9    9  1.4e-15  1.8e-15
```

```
Solution error estimates for segregated groups
0.0018, 0.0011, 0.0085, 6.1e-08
Residual error estimates for segregated groups
0.44, 1.3e+02, 1.4e+03, 0.16
Segregated solver iteration 10.
Merged variables
Iter      SolEst     Damping    Stepsize #Res #Jac #Sol   LinErr   LinRes
   1     0.00069   0.3500000     0.00069   38   28   28  2.5e-13  3.8e-15
   2     0.00045   0.3500000     0.00045   39   29   29  1.7e-13    7e-15
   3     0.00029   0.3500000     0.00029   40   30   30  2.1e-13  5.5e-15
Velocity u2, Pressure p
Iter      SolEst     Damping    Stepsize #Res #Jac #Sol   LinErr   LinRes
   1       0.001   0.5000000       0.001   20   10   10    2e-15  7.9e-15
Displacement field
Iter      SolEst     Damping    Stepsize #Res #Jac #Sol   LinErr   LinRes
   1      0.0094   0.5000000      0.0094   20   10   10  1.2e-13  1.2e-12
Spatial mesh displacement
Iter      SolEst     Damping    Stepsize #Res #Jac #Sol   LinErr   LinRes
   1     7.4e-08   0.5000000     7.4e-08   20   10   10  1.7e-15  1.9e-15
Solution error estimates for segregated groups
0.0005, 0.00052, 0.0047, 3.7e-08
Residual error estimates for segregated groups
0.14, 63, 1.2e+03, 0.16
Segregated solver iteration 11.
Merged variables
Iter      SolEst     Damping    Stepsize #Res #Jac #Sol   LinErr   LinRes
   1     0.00019   0.3500000     0.00019   42   31   31  1.7e-13  5.9e-15
   2     0.00012   0.3500000     0.00012   43   32   32  1.5e-13  4.1e-15
   3       8e-05   0.3500000       8e-05   44   33   33  7.1e-14  4.5e-15
Velocity u2, Pressure p
Iter      SolEst     Damping    Stepsize #Res #Jac #Sol   LinErr   LinRes
   1     0.00052   0.5000000     0.00052   22   11   11  2.7e-15  7.9e-15
Displacement field
Iter      SolEst     Damping    Stepsize #Res #Jac #Sol   LinErr   LinRes
   1      0.0051   0.5000000      0.0051   22   11   11    9e-14  1.2e-12
Spatial mesh displacement
Iter      SolEst     Damping    Stepsize #Res #Jac #Sol   LinErr   LinRes
   1     4.5e-08   0.5000000     4.5e-08   22   11   11  1.9e-15  1.8e-15
Solution error estimates for segregated groups
0.00014, 0.00026, 0.0026, 2.2e-08
Residual error estimates for segregated groups
0.043, 31, 1.2e+03, 0.16
Segregated solver iteration 12.
Merged variables
Iter      SolEst     Damping    Stepsize #Res #Jac #Sol   LinErr   LinRes
   1     5.2e-05   0.3500000     5.2e-05   46   34   34  9.6e-14  5.7e-15
   2     3.4e-05   0.3500000     3.4e-05   47   35   35  1.1e-13  3.6e-15
   3     2.2e-05   0.3500000     2.2e-05   48   36   36  9.4e-14  3.7e-15
Velocity u2, Pressure p
Iter      SolEst     Damping    Stepsize #Res #Jac #Sol   LinErr   LinRes
   1     0.00026   0.5000000     0.00026   24   12   12  7.6e-15    8e-15
Displacement field
Iter      SolEst     Damping    Stepsize #Res #Jac #Sol   LinErr   LinRes
   1      0.0028   0.5000000      0.0028   24   12   12  1.1e-13  1.2e-12
Spatial mesh displacement
Iter      SolEst     Damping    Stepsize #Res #Jac #Sol   LinErr   LinRes
```

```
   1      2.6e-08   0.5000000     2.6e-08   24   12   12  1.3e-15  1.9e-15
Solution error estimates for segregated groups
3.8e-05, 0.00013, 0.0014, 1.3e-08
Residual error estimates for segregated groups
0.013, 16, 1.2e+03, 0.15
Segregated solver iteration 13.
Merged variables
Iter      SolEst     Damping    Stepsize #Res #Jac #Sol   LinErr   LinRes
   1      1.4e-05   0.3500000     1.4e-05   50   37   37  3.1e-13  4.8e-15
   2      9.3e-06   0.3500000     9.3e-06   51   38   38  1.2e-13    7e-15
   3        6e-06   0.3500000       6e-06   52   39   39  1.3e-13  4.5e-15
Velocity u2, Pressure p
Iter      SolEst     Damping    Stepsize #Res #Jac #Sol   LinErr   LinRes
   1      0.00013   0.5000000     0.00013   26   13   13  4.1e-15  7.9e-15
Displacement field
Iter      SolEst     Damping    Stepsize #Res #Jac #Sol   LinErr   LinRes
   1       0.0015   0.5000000      0.0015   26   13   13  1.1e-13  1.2e-12
Spatial mesh displacement
Iter      SolEst     Damping    Stepsize #Res #Jac #Sol   LinErr   LinRes
   1      1.5e-08   0.5000000     1.5e-08   26   13   13  2.1e-15  1.9e-15
Solution error estimates for segregated groups
1e-05, 6.6e-05, 0.00076, 7.7e-09
Residual error estimates for segregated groups
0.004, 7.9, 1.2e+03, 0.17
Solution time: 163 s. (2 minutes, 43 seconds)
Physical memory: 4.68 GB
Virtual memory: 25.88 GB
Ended at Jan 31, 2025, 7:34:04 PM.
----- Stationary Solver 1 in study1/Approach Curve Solver (sol1) -------------->
```

d=2205 (su61)

GENERAL

Description	Value
Solution	d=2205

LOG

```
<---- Compile Equations: Stationary in study1/Approach Curve Solver (sol1) -----
Started at Jan 31, 2025, 7:34:04 PM.
Parameter d = 2.205E-6 (m).
Geometry shape function: Linear serendipity
Number of vertex elements: 18
Number of boundary elements: 6176
Number of elements: 155472
Minimum element quality: 0.4745
Time: 8 s.
Physical memory: 2.95 GB
Virtual memory: 15.27 GB
Ended at Jan 31, 2025, 7:34:13 PM.
----- Compile Equations: Stationary in study1/Approach Curve Solver (sol1) ---->

<---- Dependent Variables 1 in study1/Approach Curve Solver (sol1) -------------
Started at Jan 31, 2025, 7:34:13 PM.
Solution time: 0 s.
Physical memory: 2.95 GB
Virtual memory: 15.2 GB
Ended at Jan 31, 2025, 7:34:13 PM.
----- Dependent Variables 1 in study1/Approach Curve Solver (sol1) ------------>

<---- Stationary Solver 1 in study1/Approach Curve Solver (sol1) ---------------
Started at Jan 31, 2025, 7:34:13 PM.
Segregated solver
Segregated Step 11 does not have any components solved for.
Number of degrees of freedom solved for: 789769 (plus 11775 internal DOFs).
Segregated solver iteration 1.
Merged variables
Nonsymmetric matrix found.
Scales for dependent variables:
Concentration (comp1.Cl): 1.4e+02
Concentration (comp1.K): 1.4e+02
Electric potential (comp1.V): 1.9e+02
Orthonormal null-space function used.
Iter      SolEst     Damping    Stepsize #Res #Jac #Sol   LinErr   LinRes
   1        0.83   0.3500000        0.83    2    1    1  3.3e-11  2.7e-15
   2        0.81   0.3500000        0.81    3    2    2  3.1e-11  2.7e-15
   3         0.8   0.3500000         0.8    4    3    3  7.9e-11  2.7e-15
Velocity u2, Pressure p
Nonsymmetric matrix found.
Scales for dependent variables:
Pressure (comp1.p): 1
Velocity field (spatial frame) (comp1.u2): 0.00018
Orthonormal null-space function used.
Iter      SolEst     Damping    Stepsize #Res #Jac #Sol   LinErr   LinRes
   1         1.1   0.5000000         1.1    2    1    1  1.6e-15  2.3e-15
Displacement field
Nonsymmetric matrix found.
Scales for dependent variables:
Auxiliary pressure (comp1.solid.lemm1.pw): 1
Displacement field (comp1.u): 2.8e-18
Orthonormal null-space function used.
Iter      SolEst     Damping    Stepsize #Res #Jac #Sol   LinErr   LinRes
   1         1.9   0.5000000         1.9    2    1    1  5.3e-14  1.5e-12
Spatial mesh displacement
```

```
Symmetric matrices found.
Scales for dependent variables:
Spatial mesh displacement (comp1.spatial.disp): 1.2e-09
Orthonormal null-space function used.
Iter      SolEst     Damping    Stepsize #Res #Jac #Sol   LinErr   LinRes
   1       7e-07   0.5000000       7e-07    2    1    1  1.8e-15  1.9e-15
Solution error estimates for segregated groups
1.4, 0.54, 0.95, 3.5e-07
Residual error estimates for segregated groups
7.9e+03, 8.2e+04, 2.2e+04, 0.14
Segregated solver iteration 2.
Merged variables
Scales for dependent variables:
Concentration (comp1.Cl): 1.4e+02
Concentration (comp1.K): 1.4e+02
Electric potential (comp1.V): 0.064
Iter      SolEst     Damping    Stepsize #Res #Jac #Sol   LinErr   LinRes
   1        0.78   0.3500000        0.78    6    4    4  5.6e-14  4.4e-15
   2        0.77   0.3500000        0.77    7    5    5  9.7e-14  3.1e-15
   3        0.76   0.3500000        0.76    8    6    6  9.1e-14  3.2e-15
Velocity u2, Pressure p
Scales for dependent variables:
Pressure (comp1.p): 3.6e+02
Velocity field (spatial frame) (comp1.u2): 0.00072
Iter      SolEst     Damping    Stepsize #Res #Jac #Sol   LinErr   LinRes
   1        0.36   0.5000000        0.36    4    2    2  3.5e-15  7.8e-15
Displacement field
Iter      SolEst     Damping    Stepsize #Res #Jac #Sol   LinErr   LinRes
   1        0.95   0.5000000        0.95    4    2    2  9.1e-14  1.5e-12
Spatial mesh displacement
Iter      SolEst     Damping    Stepsize #Res #Jac #Sol   LinErr   LinRes
   1       1e-06   0.5000000       1e-06    4    2    2  1.9e-15  1.9e-15
Solution error estimates for segregated groups
1.3, 0.18, 0.48, 5.2e-07
Residual error estimates for segregated groups
7.8e+02, 1.4e+04, 2.2e+04, 0.15
Segregated solver iteration 3.
Merged variables
Iter      SolEst     Damping    Stepsize #Res #Jac #Sol   LinErr   LinRes
   1        0.75   0.3500000        0.75   10    7    7  7.5e-14    5e-15
   2        0.73   0.3500000        0.73   11    8    8  8.6e-14  2.9e-15
   3        0.72   0.3500000        0.72   12    9    9  9.6e-14  2.9e-15
Velocity u2, Pressure p
Iter      SolEst     Damping    Stepsize #Res #Jac #Sol   LinErr   LinRes
   1        0.15   0.5000000        0.15    6    3    3  1.4e-15  7.8e-15
Displacement field
Iter      SolEst     Damping    Stepsize #Res #Jac #Sol   LinErr   LinRes
   1        0.52   0.5000000        0.52    6    3    3  7.1e-14  1.5e-12
Spatial mesh displacement
Iter      SolEst     Damping    Stepsize #Res #Jac #Sol   LinErr   LinRes
   1       1e-06   0.5000000       1e-06    6    3    3  1.3e-15  1.8e-15
Solution error estimates for segregated groups
1.2, 0.077, 0.26, 5.2e-07
Residual error estimates for segregated groups
2.2e+02, 8e+03, 1.6e+04, 0.16
Segregated solver iteration 4.
```

```
Merged variables
Iter      SolEst     Damping    Stepsize #Res #Jac #Sol   LinErr   LinRes
   1         0.7   0.3500000         0.7   14   10   10  6.3e-14  2.9e-15
   2        0.68   0.3500000        0.68   15   11   11  1.3e-13  3.4e-15
   3        0.59   0.3500000        0.59   16   12   12  8.7e-14  3.1e-15
Velocity u2, Pressure p
Iter      SolEst     Damping    Stepsize #Res #Jac #Sol   LinErr   LinRes
   1       0.072   0.5000000       0.072    8    4    4  3.3e-15  7.8e-15
Displacement field
Iter      SolEst     Damping    Stepsize #Res #Jac #Sol   LinErr   LinRes
   1        0.29   0.5000000        0.29    8    4    4    4e-14  1.5e-12
Spatial mesh displacement
Iter      SolEst     Damping    Stepsize #Res #Jac #Sol   LinErr   LinRes
   1     8.7e-07   0.5000000     8.7e-07    8    4    4  2.1e-15  1.8e-15
Solution error estimates for segregated groups
1, 0.036, 0.15, 4.3e-07
Residual error estimates for segregated groups
83, 4.2e+03, 1.1e+04, 0.14
Segregated solver iteration 5.
Merged variables
Iter      SolEst     Damping    Stepsize #Res #Jac #Sol   LinErr   LinRes
   1        0.41   0.3500000        0.41   18   13   13  6.2e-14  4.3e-15
   2        0.27   0.3500000        0.27   19   14   14  7.4e-14  4.4e-15
   3        0.18   0.3500000        0.18   20   15   15  6.9e-14    3e-15
Velocity u2, Pressure p
Iter      SolEst     Damping    Stepsize #Res #Jac #Sol   LinErr   LinRes
   1       0.035   0.5000000       0.035   10    5    5  3.6e-15  7.8e-15
Displacement field
Iter      SolEst     Damping    Stepsize #Res #Jac #Sol   LinErr   LinRes
   1        0.17   0.5000000        0.17   10    5    5  1.7e-13  1.5e-12
Spatial mesh displacement
Iter      SolEst     Damping    Stepsize #Res #Jac #Sol   LinErr   LinRes
   1     6.5e-07   0.5000000     6.5e-07   10    5    5  1.7e-15  1.9e-15
Solution error estimates for segregated groups
0.31, 0.017, 0.084, 3.3e-07
Residual error estimates for segregated groups
33, 2.2e+03, 6.8e+03, 0.16
Segregated solver iteration 6.
Merged variables
Iter      SolEst     Damping    Stepsize #Res #Jac #Sol   LinErr   LinRes
   1        0.12   0.3500000        0.12   22   16   16  6.5e-14  2.9e-15
   2       0.078   0.3500000       0.078   23   17   17  4.6e-14    5e-15
   3       0.051   0.3500000       0.051   24   18   18  1.1e-13  3.7e-15
Velocity u2, Pressure p
Iter      SolEst     Damping    Stepsize #Res #Jac #Sol   LinErr   LinRes
   1       0.017   0.5000000       0.017   12    6    6  2.4e-15  7.9e-15
Displacement field
Iter      SolEst     Damping    Stepsize #Res #Jac #Sol   LinErr   LinRes
   1       0.095   0.5000000       0.095   12    6    6  6.6e-14  1.5e-12
Spatial mesh displacement
Iter      SolEst     Damping    Stepsize #Res #Jac #Sol   LinErr   LinRes
   1     4.6e-07   0.5000000     4.6e-07   12    6    6  1.4e-15  1.9e-15
Solution error estimates for segregated groups
0.087, 0.0085, 0.048, 2.3e-07
Residual error estimates for segregated groups
12, 1.1e+03, 4.2e+03, 0.15
```

```
Segregated solver iteration 7.
Merged variables
Iter      SolEst     Damping    Stepsize #Res #Jac #Sol   LinErr   LinRes
   1       0.033   0.3500000       0.033   26   19   19  1.6e-13  3.3e-15
   2       0.022   0.3500000       0.022   27   20   20  1.2e-13  4.5e-15
   3       0.014   0.3500000       0.014   28   21   21  1.8e-13  4.3e-15
Velocity u2, Pressure p
Iter      SolEst     Damping    Stepsize #Res #Jac #Sol   LinErr   LinRes
   1      0.0085   0.5000000      0.0085   14    7    7  4.3e-15  7.9e-15
Displacement field
Iter      SolEst     Damping    Stepsize #Res #Jac #Sol   LinErr   LinRes
   1       0.054   0.5000000       0.054   14    7    7  5.5e-14  1.5e-12
Spatial mesh displacement
Iter      SolEst     Damping    Stepsize #Res #Jac #Sol   LinErr   LinRes
   1       3e-07   0.5000000       3e-07   14    7    7  1.9e-15  1.9e-15
Solution error estimates for segregated groups
0.024, 0.0042, 0.027, 1.5e-07
Residual error estimates for segregated groups
4.1, 5.5e+02, 2.6e+03, 0.15
Segregated solver iteration 8.
Merged variables
Iter      SolEst     Damping    Stepsize #Res #Jac #Sol   LinErr   LinRes
   1      0.0091   0.3500000      0.0091   30   22   22    7e-14  3.1e-15
   2      0.0059   0.3500000      0.0059   31   23   23    1e-13  3.8e-15
   3      0.0039   0.3500000      0.0039   32   24   24    9e-14  3.6e-15
Velocity u2, Pressure p
Iter      SolEst     Damping    Stepsize #Res #Jac #Sol   LinErr   LinRes
   1      0.0042   0.5000000      0.0042   16    8    8  2.5e-15  7.7e-15
Displacement field
Scales for dependent variables:
Auxiliary pressure (comp1.solid.lemm1.pw): 0.36
Displacement field (comp1.u): 2.8e-16
Iter      SolEst     Damping    Stepsize #Res #Jac #Sol   LinErr   LinRes
   1        0.03   0.5000000        0.03   16    8    8  1.2e-13  1.2e-12
Spatial mesh displacement
Iter      SolEst     Damping    Stepsize #Res #Jac #Sol   LinErr   LinRes
   1       2e-07   0.5000000       2e-07   16    8    8  1.5e-15  1.9e-15
Solution error estimates for segregated groups
0.0066, 0.0021, 0.015, 9.8e-08
Residual error estimates for segregated groups
1.4, 2.8e+02, 1.8e+03, 0.15
Segregated solver iteration 9.
Merged variables
Iter      SolEst     Damping    Stepsize #Res #Jac #Sol   LinErr   LinRes
   1      0.0025   0.3500000      0.0025   34   25   25  9.7e-14    4e-15
   2      0.0016   0.3500000      0.0016   35   26   26  9.9e-14    4e-15
   3      0.0011   0.3500000      0.0011   36   27   27  1.4e-13  4.4e-15
Velocity u2, Pressure p
Iter      SolEst     Damping    Stepsize #Res #Jac #Sol   LinErr   LinRes
   1      0.0021   0.5000000      0.0021   18    9    9  2.9e-15    8e-15
Displacement field
Iter      SolEst     Damping    Stepsize #Res #Jac #Sol   LinErr   LinRes
   1       0.017   0.5000000       0.017   18    9    9  1.6e-13  1.2e-12
Spatial mesh displacement
Iter      SolEst     Damping    Stepsize #Res #Jac #Sol   LinErr   LinRes
   1     1.2e-07   0.5000000     1.2e-07   18    9    9  1.4e-15  1.9e-15
```

```
Solution error estimates for segregated groups
0.0018, 0.0011, 0.0085, 6.1e-08
Residual error estimates for segregated groups
0.44, 1.4e+02, 1.4e+03, 0.16
Segregated solver iteration 10.
Merged variables
Iter      SolEst     Damping    Stepsize #Res #Jac #Sol   LinErr   LinRes
   1     0.00069   0.3500000     0.00069   38   28   28    1e-13  4.1e-15
   2     0.00045   0.3500000     0.00045   39   29   29  1.4e-13  3.4e-15
   3     0.00029   0.3500000     0.00029   40   30   30  9.3e-14  3.8e-15
Velocity u2, Pressure p
Iter      SolEst     Damping    Stepsize #Res #Jac #Sol   LinErr   LinRes
   1      0.0011   0.5000000      0.0011   20   10   10  4.1e-15  7.8e-15
Displacement field
Iter      SolEst     Damping    Stepsize #Res #Jac #Sol   LinErr   LinRes
   1      0.0094   0.5000000      0.0094   20   10   10  1.1e-13  1.2e-12
Spatial mesh displacement
Iter      SolEst     Damping    Stepsize #Res #Jac #Sol   LinErr   LinRes
   1     7.5e-08   0.5000000     7.5e-08   20   10   10  1.6e-15  1.9e-15
Solution error estimates for segregated groups
0.0005, 0.00053, 0.0047, 3.7e-08
Residual error estimates for segregated groups
0.14, 70, 1.2e+03, 0.16
Segregated solver iteration 11.
Merged variables
Iter      SolEst     Damping    Stepsize #Res #Jac #Sol   LinErr   LinRes
   1     0.00019   0.3500000     0.00019   42   31   31  9.2e-14  3.3e-15
   2     0.00012   0.3500000     0.00012   43   32   32  1.4e-13  3.5e-15
   3       8e-05   0.3500000       8e-05   44   33   33    6e-14  3.6e-15
Velocity u2, Pressure p
Iter      SolEst     Damping    Stepsize #Res #Jac #Sol   LinErr   LinRes
   1     0.00052   0.5000000     0.00052   22   11   11  2.9e-15  8.1e-15
Displacement field
Iter      SolEst     Damping    Stepsize #Res #Jac #Sol   LinErr   LinRes
   1      0.0051   0.5000000      0.0051   22   11   11  9.4e-14  1.2e-12
Spatial mesh displacement
Iter      SolEst     Damping    Stepsize #Res #Jac #Sol   LinErr   LinRes
   1     4.5e-08   0.5000000     4.5e-08   22   11   11  1.7e-15  1.9e-15
Solution error estimates for segregated groups
0.00014, 0.00026, 0.0026, 2.2e-08
Residual error estimates for segregated groups
0.043, 35, 1.2e+03, 0.17
Segregated solver iteration 12.
Merged variables
Iter      SolEst     Damping    Stepsize #Res #Jac #Sol   LinErr   LinRes
   1     5.2e-05   0.3500000     5.2e-05   46   34   34  1.2e-13  3.2e-15
   2     3.4e-05   0.3500000     3.4e-05   47   35   35  9.5e-14  3.3e-15
   3     2.2e-05   0.3500000     2.2e-05   48   36   36    5e-14  3.6e-15
Velocity u2, Pressure p
Iter      SolEst     Damping    Stepsize #Res #Jac #Sol   LinErr   LinRes
   1     0.00026   0.5000000     0.00026   24   12   12  2.3e-15  7.7e-15
Displacement field
Iter      SolEst     Damping    Stepsize #Res #Jac #Sol   LinErr   LinRes
   1      0.0028   0.5000000      0.0028   24   12   12  1.4e-13  1.2e-12
Spatial mesh displacement
Iter      SolEst     Damping    Stepsize #Res #Jac #Sol   LinErr   LinRes
```

```
   1      2.6e-08   0.5000000     2.6e-08   24    12    12  2.1e-15  1.8e-15
Solution error estimates for segregated groups
3.8e-05, 0.00013, 0.0014, 1.3e-08
Residual error estimates for segregated groups
0.013, 17, 1.2e+03, 0.15
Segregated solver iteration 13.
Merged variables
Iter      SolEst      Damping    Stepsize #Res #Jac #Sol   LinErr   LinRes
   1      1.4e-05   0.3500000     1.4e-05   50    37    37  8.5e-14  3.1e-15
   2      9.3e-06   0.3500000     9.3e-06   51    38    38  7.5e-14  3.5e-15
   3        6e-06   0.3500000       6e-06   52    39    39  1.1e-13    3e-15
Velocity u2, Pressure p
Iter      SolEst      Damping    Stepsize #Res #Jac #Sol   LinErr   LinRes
   1      0.00013   0.5000000     0.00013   26    13    13  1.8e-15  7.9e-15
Displacement field
Iter      SolEst      Damping    Stepsize #Res #Jac #Sol   LinErr   LinRes
   1       0.0015   0.5000000      0.0015   26    13    13  1.6e-13  1.2e-12
Spatial mesh displacement
Iter      SolEst      Damping    Stepsize #Res #Jac #Sol   LinErr   LinRes
   1      1.5e-08   0.5000000     1.5e-08   26    13    13  1.4e-15  1.9e-15
Solution error estimates for segregated groups
1e-05, 6.6e-05, 0.00076, 7.7e-09
Residual error estimates for segregated groups
0.004, 8.7, 1.2e+03, 0.15
Solution time: 155 s. (2 minutes, 35 seconds)
Physical memory: 4.7 GB
Virtual memory: 25.52 GB
Ended at Jan 31, 2025, 7:36:48 PM.
----- Stationary Solver 1 in study1/Approach Curve Solver (sol1) -------------->
```

d=2200 (su62)

GENERAL

Description	Value
Solution	d=2200

LOG

```
<---- Compile Equations: Stationary in study1/Approach Curve Solver (sol1) -----
Started at Jan 31, 2025, 7:36:48 PM.
Parameter d = 2.2E-6 (m).
Geometry shape function: Linear serendipity
Number of vertex elements: 18
Number of boundary elements: 6173
Number of elements: 155323
Minimum element quality: 0.4893
Time: 8 s.
Physical memory: 2.93 GB
Virtual memory: 15.25 GB
Ended at Jan 31, 2025, 7:36:56 PM.
----- Compile Equations: Stationary in study1/Approach Curve Solver (sol1) ---->

<---- Dependent Variables 1 in study1/Approach Curve Solver (sol1) -------------
Started at Jan 31, 2025, 7:36:56 PM.
Solution time: 0 s.
Physical memory: 2.93 GB
Virtual memory: 15.18 GB
Ended at Jan 31, 2025, 7:36:57 PM.
----- Dependent Variables 1 in study1/Approach Curve Solver (sol1) ------------>

<---- Stationary Solver 1 in study1/Approach Curve Solver (sol1) ---------------
Started at Jan 31, 2025, 7:36:57 PM.
Segregated solver
Segregated Step 11 does not have any components solved for.
Number of degrees of freedom solved for: 788970 (plus 11769 internal DOFs).
Segregated solver iteration 1.
Merged variables
Nonsymmetric matrix found.
Scales for dependent variables:
Concentration (comp1.Cl): 1.4e+02
Concentration (comp1.K): 1.4e+02
Electric potential (comp1.V): 1.9e+02
Orthonormal null-space function used.
Iter      SolEst     Damping    Stepsize #Res #Jac #Sol   LinErr   LinRes
   1        0.83   0.3500000        0.83    2    1    1  3.8e-11  2.7e-15
   2        0.81   0.3500000        0.81    3    2    2  1.5e-11  2.7e-15
   3         0.8   0.3500000         0.8    4    3    3  5.3e-11  2.7e-15
Velocity u2, Pressure p
Nonsymmetric matrix found.
Scales for dependent variables:
Pressure (comp1.p): 1
Velocity field (spatial frame) (comp1.u2): 0.00018
Orthonormal null-space function used.
Iter      SolEst     Damping    Stepsize #Res #Jac #Sol   LinErr   LinRes
   1         1.1   0.5000000         1.1    2    1    1  1.5e-15  2.3e-15
Displacement field
Nonsymmetric matrix found.
Scales for dependent variables:
Auxiliary pressure (comp1.solid.lemm1.pw): 1
Displacement field (comp1.u): 2.8e-18
Orthonormal null-space function used.
Iter      SolEst     Damping    Stepsize #Res #Jac #Sol   LinErr   LinRes
   1         1.9   0.5000000         1.9    2    1    1  3.5e-14  1.5e-12
Spatial mesh displacement
```

```
Symmetric matrices found.
Scales for dependent variables:
Spatial mesh displacement (comp1.spatial.disp): 1.2e-09
Orthonormal null-space function used.
Iter      SolEst     Damping    Stepsize #Res #Jac #Sol   LinErr   LinRes
   1       7e-07   0.5000000       7e-07    2    1    1  1.7e-15  1.8e-15
Solution error estimates for segregated groups
1.4, 0.54, 0.95, 3.5e-07
Residual error estimates for segregated groups
7.9e+03, 8.2e+04, 2.2e+04, 0.14
Segregated solver iteration 2.
Merged variables
Scales for dependent variables:
Concentration (comp1.Cl): 1.4e+02
Concentration (comp1.K): 1.4e+02
Electric potential (comp1.V): 0.064
Iter      SolEst     Damping    Stepsize #Res #Jac #Sol   LinErr   LinRes
   1        0.78   0.3500000        0.78    6    4    4  6.4e-14    3e-15
   2        0.77   0.3500000        0.77    7    5    5  9.3e-14  2.9e-15
   3        0.76   0.3500000        0.76    8    6    6  1.1e-13    3e-15
Velocity u2, Pressure p
Scales for dependent variables:
Pressure (comp1.p): 3.6e+02
Velocity field (spatial frame) (comp1.u2): 0.00072
Iter      SolEst     Damping    Stepsize #Res #Jac #Sol   LinErr   LinRes
   1        0.36   0.5000000        0.36    4    2    2    3e-15  7.8e-15
Displacement field
Iter      SolEst     Damping    Stepsize #Res #Jac #Sol   LinErr   LinRes
   1        0.95   0.5000000        0.95    4    2    2  4.2e-14  1.5e-12
Spatial mesh displacement
Iter      SolEst     Damping    Stepsize #Res #Jac #Sol   LinErr   LinRes
   1       1e-06   0.5000000       1e-06    4    2    2  1.3e-15  1.8e-15
Solution error estimates for segregated groups
1.3, 0.18, 0.48, 5.2e-07
Residual error estimates for segregated groups
7.8e+02, 1.6e+04, 2.2e+04, 0.16
Segregated solver iteration 3.
Merged variables
Iter      SolEst     Damping    Stepsize #Res #Jac #Sol   LinErr   LinRes
   1        0.75   0.3500000        0.75   10    7    7  1.3e-13    4e-15
   2        0.73   0.3500000        0.73   11    8    8    1e-13    3e-15
   3        0.72   0.3500000        0.72   12    9    9  6.9e-14    3e-15
Velocity u2, Pressure p
Iter      SolEst     Damping    Stepsize #Res #Jac #Sol   LinErr   LinRes
   1        0.15   0.5000000        0.15    6    3    3    5e-15  7.8e-15
Displacement field
Iter      SolEst     Damping    Stepsize #Res #Jac #Sol   LinErr   LinRes
   1        0.52   0.5000000        0.52    6    3    3  1.5e-13  1.5e-12
Spatial mesh displacement
Iter      SolEst     Damping    Stepsize #Res #Jac #Sol   LinErr   LinRes
   1       1e-06   0.5000000       1e-06    6    3    3  1.6e-15  1.8e-15
Solution error estimates for segregated groups
1.2, 0.077, 0.26, 5.2e-07
Residual error estimates for segregated groups
2.2e+02, 9.4e+03, 1.6e+04, 0.15
Segregated solver iteration 4.
```

```
Merged variables
Iter      SolEst     Damping    Stepsize #Res #Jac #Sol   LinErr   LinRes
   1         0.7   0.3500000         0.7   14   10   10    8e-14    3e-15
   2        0.68   0.3500000        0.68   15   11   11  6.8e-14  2.9e-15
   3        0.59   0.3500000        0.59   16   12   12  1.1e-13  3.2e-15
Velocity u2, Pressure p
Iter      SolEst     Damping    Stepsize #Res #Jac #Sol   LinErr   LinRes
   1       0.072   0.5000000       0.072    8    4    4  2.4e-15  7.8e-15
Displacement field
Iter      SolEst     Damping    Stepsize #Res #Jac #Sol   LinErr   LinRes
   1        0.29   0.5000000        0.29    8    4    4  3.5e-14  1.5e-12
Spatial mesh displacement
Iter      SolEst     Damping    Stepsize #Res #Jac #Sol   LinErr   LinRes
   1     8.7e-07   0.5000000     8.7e-07    8    4    4  1.4e-15  1.9e-15
Solution error estimates for segregated groups
1, 0.036, 0.15, 4.4e-07
Residual error estimates for segregated groups
83, 5e+03, 1.1e+04, 0.16
Segregated solver iteration 5.
Merged variables
Iter      SolEst     Damping    Stepsize #Res #Jac #Sol   LinErr   LinRes
   1        0.41   0.3500000        0.41   18   13   13  9.1e-14  3.1e-15
   2        0.27   0.3500000        0.27   19   14   14  1.3e-13  2.9e-15
   3        0.18   0.3500000        0.18   20   15   15  8.1e-14  3.1e-15
Velocity u2, Pressure p
Iter      SolEst     Damping    Stepsize #Res #Jac #Sol   LinErr   LinRes
   1       0.035   0.5000000       0.035   10    5    5  4.7e-15  7.9e-15
Displacement field
Iter      SolEst     Damping    Stepsize #Res #Jac #Sol   LinErr   LinRes
   1        0.17   0.5000000        0.17   10    5    5  6.6e-14  1.5e-12
Spatial mesh displacement
Iter      SolEst     Damping    Stepsize #Res #Jac #Sol   LinErr   LinRes
   1     6.5e-07   0.5000000     6.5e-07   10    5    5  1.3e-15  1.8e-15
Solution error estimates for segregated groups
0.31, 0.017, 0.084, 3.3e-07
Residual error estimates for segregated groups
32, 2.6e+03, 6.8e+03, 0.15
Segregated solver iteration 6.
Merged variables
Iter      SolEst     Damping    Stepsize #Res #Jac #Sol   LinErr   LinRes
   1        0.12   0.3500000        0.12   22   16   16    9e-14    3e-15
   2       0.078   0.3500000       0.078   23   17   17    9e-14  3.1e-15
   3       0.051   0.3500000       0.051   24   18   18  1.3e-13    3e-15
Velocity u2, Pressure p
Iter      SolEst     Damping    Stepsize #Res #Jac #Sol   LinErr   LinRes
   1       0.017   0.5000000       0.017   12    6    6  5.5e-15  7.7e-15
Displacement field
Iter      SolEst     Damping    Stepsize #Res #Jac #Sol   LinErr   LinRes
   1       0.095   0.5000000       0.095   12    6    6  1.1e-13  1.5e-12
Spatial mesh displacement
Iter      SolEst     Damping    Stepsize #Res #Jac #Sol   LinErr   LinRes
   1     4.6e-07   0.5000000     4.6e-07   12    6    6  1.3e-15  1.8e-15
Solution error estimates for segregated groups
0.087, 0.0085, 0.048, 2.3e-07
Residual error estimates for segregated groups
12, 1.3e+03, 4.2e+03, 0.15
```

```
Segregated solver iteration 7.
Merged variables
Iter      SolEst     Damping    Stepsize #Res #Jac #Sol   LinErr   LinRes
   1       0.033   0.3500000       0.033   26   19   19    1e-13  3.9e-15
   2       0.021   0.3500000       0.021   27   20   20    7e-14  2.8e-15
   3       0.014   0.3500000       0.014   28   21   21    8e-14    3e-15
Velocity u2, Pressure p
Iter      SolEst     Damping    Stepsize #Res #Jac #Sol   LinErr   LinRes
   1      0.0085   0.5000000      0.0085   14    7    7  2.5e-15  7.7e-15
Displacement field
Iter      SolEst     Damping    Stepsize #Res #Jac #Sol   LinErr   LinRes
   1       0.054   0.5000000       0.054   14    7    7  1.3e-13  1.5e-12
Spatial mesh displacement
Iter      SolEst     Damping    Stepsize #Res #Jac #Sol   LinErr   LinRes
   1     3.1e-07   0.5000000     3.1e-07   14    7    7  1.7e-15  1.9e-15
Solution error estimates for segregated groups
0.024, 0.0042, 0.027, 1.5e-07
Residual error estimates for segregated groups
4.1, 6.5e+02, 2.6e+03, 0.15
Segregated solver iteration 8.
Merged variables
Iter      SolEst     Damping    Stepsize #Res #Jac #Sol   LinErr   LinRes
   1      0.0091   0.3500000      0.0091   30   22   22  1.2e-13  2.9e-15
   2      0.0059   0.3500000      0.0059   31   23   23  6.5e-14    3e-15
   3      0.0038   0.3500000      0.0038   32   24   24  1.3e-13  3.4e-15
Velocity u2, Pressure p
Iter      SolEst     Damping    Stepsize #Res #Jac #Sol   LinErr   LinRes
   1      0.0042   0.5000000      0.0042   16    8    8  2.2e-15  7.9e-15
Displacement field
Scales for dependent variables:
Auxiliary pressure (comp1.solid.lemm1.pw): 0.36
Displacement field (comp1.u): 2.8e-16
Iter      SolEst     Damping    Stepsize #Res #Jac #Sol   LinErr   LinRes
   1        0.03   0.5000000        0.03   16    8    8    1e-13  1.2e-12
Spatial mesh displacement
Iter      SolEst     Damping    Stepsize #Res #Jac #Sol   LinErr   LinRes
   1       2e-07   0.5000000       2e-07   16    8    8  1.8e-15  1.8e-15
Solution error estimates for segregated groups
0.0066, 0.0021, 0.015, 9.8e-08
Residual error estimates for segregated groups
1.4, 3.3e+02, 1.8e+03, 0.15
Segregated solver iteration 9.
Merged variables
Iter      SolEst     Damping    Stepsize #Res #Jac #Sol   LinErr   LinRes
   1      0.0025   0.3500000      0.0025   34   25   25  6.5e-14  3.1e-15
   2      0.0016   0.3500000      0.0016   35   26   26    1e-13  2.9e-15
   3      0.0011   0.3500000      0.0011   36   27   27  5.7e-14    3e-15
Velocity u2, Pressure p
Iter      SolEst     Damping    Stepsize #Res #Jac #Sol   LinErr   LinRes
   1      0.0021   0.5000000      0.0021   18    9    9  3.7e-15  7.8e-15
Displacement field
Iter      SolEst     Damping    Stepsize #Res #Jac #Sol   LinErr   LinRes
   1       0.017   0.5000000       0.017   18    9    9  1.2e-13  1.2e-12
Spatial mesh displacement
Iter      SolEst     Damping    Stepsize #Res #Jac #Sol   LinErr   LinRes
   1     1.2e-07   0.5000000     1.2e-07   18    9    9  1.3e-15  1.8e-15
```

```
Solution error estimates for segregated groups
0.0018, 0.0011, 0.0085, 6.1e-08
Residual error estimates for segregated groups
0.44, 1.6e+02, 1.4e+03, 0.16
Segregated solver iteration 10.
Merged variables
Iter      SolEst     Damping    Stepsize #Res #Jac #Sol   LinErr   LinRes
   1     0.00069   0.3500000     0.00069   38   28   28    2e-13    3e-15
   2     0.00045   0.3500000     0.00045   39   29   29  8.1e-14  2.9e-15
   3     0.00029   0.3500000     0.00029   40   30   30  1.1e-13  3.1e-15
Velocity u2, Pressure p
Iter      SolEst     Damping    Stepsize #Res #Jac #Sol   LinErr   LinRes
   1      0.0011   0.5000000      0.0011   20   10   10  2.6e-15  7.9e-15
Displacement field
Iter      SolEst     Damping    Stepsize #Res #Jac #Sol   LinErr   LinRes
   1      0.0094   0.5000000      0.0094   20   10   10  1.2e-13  1.2e-12
Spatial mesh displacement
Iter      SolEst     Damping    Stepsize #Res #Jac #Sol   LinErr   LinRes
   1     7.5e-08   0.5000000     7.5e-08   20   10   10  1.7e-15  1.9e-15
Solution error estimates for segregated groups
0.0005, 0.00053, 0.0047, 3.8e-08
Residual error estimates for segregated groups
0.14, 82, 1.2e+03, 0.15
Segregated solver iteration 11.
Merged variables
Iter      SolEst     Damping    Stepsize #Res #Jac #Sol   LinErr   LinRes
   1     0.00019   0.3500000     0.00019   42   31   31  6.5e-14  2.9e-15
   2     0.00012   0.3500000     0.00012   43   32   32  1.3e-13    3e-15
   3       8e-05   0.3500000       8e-05   44   33   33  1.5e-13  2.8e-15
Velocity u2, Pressure p
Iter      SolEst     Damping    Stepsize #Res #Jac #Sol   LinErr   LinRes
   1     0.00053   0.5000000     0.00053   22   11   11    4e-15  7.7e-15
Displacement field
Iter      SolEst     Damping    Stepsize #Res #Jac #Sol   LinErr   LinRes
   1      0.0051   0.5000000      0.0051   22   11   11  1.4e-13  1.2e-12
Spatial mesh displacement
Iter      SolEst     Damping    Stepsize #Res #Jac #Sol   LinErr   LinRes
   1     4.5e-08   0.5000000     4.5e-08   22   11   11  1.5e-15  1.8e-15
Solution error estimates for segregated groups
0.00014, 0.00026, 0.0026, 2.3e-08
Residual error estimates for segregated groups
0.043, 41, 1.2e+03, 0.16
Segregated solver iteration 12.
Merged variables
Iter      SolEst     Damping    Stepsize #Res #Jac #Sol   LinErr   LinRes
   1     5.2e-05   0.3500000     5.2e-05   46   34   34  7.1e-14  2.9e-15
   2     3.4e-05   0.3500000     3.4e-05   47   35   35  8.4e-14    3e-15
   3     2.2e-05   0.3500000     2.2e-05   48   36   36  9.3e-14  3.3e-15
Velocity u2, Pressure p
Iter      SolEst     Damping    Stepsize #Res #Jac #Sol   LinErr   LinRes
   1     0.00026   0.5000000     0.00026   24   12   12  2.4e-15  7.9e-15
Displacement field
Iter      SolEst     Damping    Stepsize #Res #Jac #Sol   LinErr   LinRes
   1      0.0028   0.5000000      0.0028   24   12   12    1e-13  1.2e-12
Spatial mesh displacement
Iter      SolEst     Damping    Stepsize #Res #Jac #Sol   LinErr   LinRes
```

```
   1     2.7e-08   0.5000000     2.7e-08   24   12   12  1.6e-15  1.8e-15
Solution error estimates for segregated groups
3.8e-05, 0.00013, 0.0014, 1.3e-08
Residual error estimates for segregated groups
0.013, 20, 1.2e+03, 0.16
Segregated solver iteration 13.
Merged variables
Iter      SolEst     Damping    Stepsize #Res #Jac #Sol   LinErr   LinRes
   1     1.4e-05   0.3500000     1.4e-05   50   37   37  7.3e-14    3e-15
   2     9.3e-06   0.3500000     9.3e-06   51   38   38  6.6e-14  3.1e-15
   3       6e-06   0.3500000       6e-06   52   39   39  6.1e-14    3e-15
Velocity u2, Pressure p
Iter      SolEst     Damping    Stepsize #Res #Jac #Sol   LinErr   LinRes
   1     0.00013   0.5000000     0.00013   26   13   13  2.4e-15  7.9e-15
Displacement field
Iter      SolEst     Damping    Stepsize #Res #Jac #Sol   LinErr   LinRes
   1      0.0015   0.5000000      0.0015   26   13   13  1.1e-13  1.2e-12
Spatial mesh displacement
Iter      SolEst     Damping    Stepsize #Res #Jac #Sol   LinErr   LinRes
   1     1.6e-08   0.5000000     1.6e-08   26   13   13  1.9e-15  1.8e-15
Solution error estimates for segregated groups
1e-05, 6.6e-05, 0.00076, 7.8e-09
Residual error estimates for segregated groups
0.004, 10, 1.2e+03, 0.16
Solution time: 153 s. (2 minutes, 33 seconds)
Physical memory: 4.65 GB
Virtual memory: 25.54 GB
Ended at Jan 31, 2025, 7:39:30 PM.
----- Stationary Solver 1 in study1/Approach Curve Solver (sol1) -------------->
```

d=2195 (su63)

GENERAL

Description	Value
Solution	d=2195

LOG

```
<---- Compile Equations: Stationary in study1/Approach Curve Solver (sol1) -----
Started at Jan 31, 2025, 7:39:30 PM.
Parameter d = 2.195E-6 (m).
Geometry shape function: Linear serendipity
Number of vertex elements: 18
Number of boundary elements: 6171
Number of elements: 155077
Minimum element quality: 0.4866
Time: 8 s.
Physical memory: 2.94 GB
Virtual memory: 15.25 GB
Ended at Jan 31, 2025, 7:39:38 PM.
----- Compile Equations: Stationary in study1/Approach Curve Solver (sol1) ---->

<---- Dependent Variables 1 in study1/Approach Curve Solver (sol1) -------------
Started at Jan 31, 2025, 7:39:38 PM.
Solution time: 0 s.
Physical memory: 2.94 GB
Virtual memory: 15.18 GB
Ended at Jan 31, 2025, 7:39:39 PM.
----- Dependent Variables 1 in study1/Approach Curve Solver (sol1) ------------>

<---- Stationary Solver 1 in study1/Approach Curve Solver (sol1) ---------------
Started at Jan 31, 2025, 7:39:39 PM.
Segregated solver
Segregated Step 11 does not have any components solved for.
Number of degrees of freedom solved for: 787758 (plus 11765 internal DOFs).
Segregated solver iteration 1.
Merged variables
Nonsymmetric matrix found.
Scales for dependent variables:
Concentration (comp1.Cl): 1.4e+02
Concentration (comp1.K): 1.4e+02
Electric potential (comp1.V): 1.9e+02
Orthonormal null-space function used.
Iter      SolEst     Damping    Stepsize #Res #Jac #Sol   LinErr   LinRes
   1        0.83   0.3500000        0.83    2    1    1  2.1e-11  2.7e-15
   2        0.81   0.3500000        0.81    3    2    2  3.2e-11  2.6e-15
   3         0.8   0.3500000         0.8    4    3    3  1.8e-11  2.6e-15
Velocity u2, Pressure p
Nonsymmetric matrix found.
Scales for dependent variables:
Pressure (comp1.p): 1
Velocity field (spatial frame) (comp1.u2): 0.00018
Orthonormal null-space function used.
Iter      SolEst     Damping    Stepsize #Res #Jac #Sol   LinErr   LinRes
   1         1.1   0.5000000         1.1    2    1    1  1.5e-15  2.3e-15
Displacement field
Nonsymmetric matrix found.
Scales for dependent variables:
Auxiliary pressure (comp1.solid.lemm1.pw): 1
Displacement field (comp1.u): 2.8e-18
Orthonormal null-space function used.
Iter      SolEst     Damping    Stepsize #Res #Jac #Sol   LinErr   LinRes
   1         1.9   0.5000000         1.9    2    1    1  1.2e-13  1.5e-12
Spatial mesh displacement
```

```
Symmetric matrices found.
Scales for dependent variables:
Spatial mesh displacement (comp1.spatial.disp): 1.2e-09
Orthonormal null-space function used.
Iter      SolEst     Damping    Stepsize #Res #Jac #Sol   LinErr   LinRes
   1       7e-07   0.5000000       7e-07    2    1    1  1.7e-15  1.8e-15
Solution error estimates for segregated groups
1.4, 0.54, 0.95, 3.5e-07
Residual error estimates for segregated groups
7.9e+03, 8.2e+04, 2.2e+04, 0.14
Segregated solver iteration 2.
Merged variables
Scales for dependent variables:
Concentration (comp1.Cl): 1.4e+02
Concentration (comp1.K): 1.4e+02
Electric potential (comp1.V): 0.064
Iter      SolEst     Damping    Stepsize #Res #Jac #Sol   LinErr   LinRes
   1        0.78   0.3500000        0.78    6    4    4  8.5e-14  3.5e-15
   2        0.77   0.3500000        0.77    7    5    5  8.5e-14  3.1e-15
   3        0.76   0.3500000        0.76    8    6    6  5.2e-14  3.1e-15
Velocity u2, Pressure p
Scales for dependent variables:
Pressure (comp1.p): 3.6e+02
Velocity field (spatial frame) (comp1.u2): 0.00072
Iter      SolEst     Damping    Stepsize #Res #Jac #Sol   LinErr   LinRes
   1        0.36   0.5000000        0.36    4    2    2  5.8e-15  7.8e-15
Displacement field
Iter      SolEst     Damping    Stepsize #Res #Jac #Sol   LinErr   LinRes
   1        0.95   0.5000000        0.95    4    2    2  3.9e-14  1.5e-12
Spatial mesh displacement
Iter      SolEst     Damping    Stepsize #Res #Jac #Sol   LinErr   LinRes
   1     1.1e-06   0.5000000     1.1e-06    4    2    2  1.6e-15  1.9e-15
Solution error estimates for segregated groups
1.3, 0.18, 0.48, 5.3e-07
Residual error estimates for segregated groups
7.8e+02, 1.2e+04, 2.2e+04, 0.16
Segregated solver iteration 3.
Merged variables
Iter      SolEst     Damping    Stepsize #Res #Jac #Sol   LinErr   LinRes
   1        0.75   0.3500000        0.75   10    7    7  5.3e-14    3e-15
   2        0.73   0.3500000        0.73   11    8    8  6.5e-14  3.2e-15
   3        0.72   0.3500000        0.72   12    9    9  1.3e-13  3.1e-15
Velocity u2, Pressure p
Iter      SolEst     Damping    Stepsize #Res #Jac #Sol   LinErr   LinRes
   1        0.15   0.5000000        0.15    6    3    3  5.7e-15  7.8e-15
Displacement field
Iter      SolEst     Damping    Stepsize #Res #Jac #Sol   LinErr   LinRes
   1        0.52   0.5000000        0.52    6    3    3  1.2e-13  1.5e-12
Spatial mesh displacement
Iter      SolEst     Damping    Stepsize #Res #Jac #Sol   LinErr   LinRes
   1     1.1e-06   0.5000000     1.1e-06    6    3    3  1.5e-15  1.8e-15
Solution error estimates for segregated groups
1.2, 0.077, 0.26, 5.3e-07
Residual error estimates for segregated groups
2.2e+02, 6.9e+03, 1.6e+04, 0.14
Segregated solver iteration 4.
```

```
Merged variables
Iter      SolEst     Damping    Stepsize #Res #Jac #Sol   LinErr   LinRes
   1         0.7   0.3500000         0.7   14   10   10  7.3e-14  3.4e-15
   2        0.68   0.3500000        0.68   15   11   11  1.3e-13  3.6e-15
   3        0.59   0.3500000        0.59   16   12   12    5e-14  3.1e-15
Velocity u2, Pressure p
Iter      SolEst     Damping    Stepsize #Res #Jac #Sol   LinErr   LinRes
   1       0.072   0.5000000       0.072    8    4    4    3e-15  7.9e-15
Displacement field
Iter      SolEst     Damping    Stepsize #Res #Jac #Sol   LinErr   LinRes
   1        0.29   0.5000000        0.29    8    4    4    3e-14  1.5e-12
Spatial mesh displacement
Iter      SolEst     Damping    Stepsize #Res #Jac #Sol   LinErr   LinRes
   1     8.8e-07   0.5000000     8.8e-07    8    4    4  1.7e-15  1.8e-15
Solution error estimates for segregated groups
1, 0.036, 0.15, 4.4e-07
Residual error estimates for segregated groups
83, 3.6e+03, 1.1e+04, 0.14
Segregated solver iteration 5.
Merged variables
Iter      SolEst     Damping    Stepsize #Res #Jac #Sol   LinErr   LinRes
   1        0.41   0.3500000        0.41   18   13   13    7e-14  2.8e-15
   2        0.27   0.3500000        0.27   19   14   14  1.2e-13  3.3e-15
   3        0.18   0.3500000        0.18   20   15   15  5.5e-14  3.2e-15
Velocity u2, Pressure p
Iter      SolEst     Damping    Stepsize #Res #Jac #Sol   LinErr   LinRes
   1       0.035   0.5000000       0.035   10    5    5  2.3e-15    8e-15
Displacement field
Iter      SolEst     Damping    Stepsize #Res #Jac #Sol   LinErr   LinRes
   1        0.17   0.5000000        0.17   10    5    5    6e-14  1.5e-12
Spatial mesh displacement
Iter      SolEst     Damping    Stepsize #Res #Jac #Sol   LinErr   LinRes
   1     6.6e-07   0.5000000     6.6e-07   10    5    5  1.6e-15  1.8e-15
Solution error estimates for segregated groups
0.31, 0.017, 0.084, 3.3e-07
Residual error estimates for segregated groups
32, 1.9e+03, 6.8e+03, 0.15
Segregated solver iteration 6.
Merged variables
Iter      SolEst     Damping    Stepsize #Res #Jac #Sol   LinErr   LinRes
   1        0.12   0.3500000        0.12   22   16   16  1.4e-13  2.9e-15
   2       0.078   0.3500000       0.078   23   17   17  4.7e-14  2.9e-15
   3       0.051   0.3500000       0.051   24   18   18    7e-14    3e-15
Velocity u2, Pressure p
Iter      SolEst     Damping    Stepsize #Res #Jac #Sol   LinErr   LinRes
   1       0.017   0.5000000       0.017   12    6    6  2.1e-15  7.8e-15
Displacement field
Iter      SolEst     Damping    Stepsize #Res #Jac #Sol   LinErr   LinRes
   1       0.095   0.5000000       0.095   12    6    6  3.5e-14  1.5e-12
Spatial mesh displacement
Iter      SolEst     Damping    Stepsize #Res #Jac #Sol   LinErr   LinRes
   1     4.6e-07   0.5000000     4.6e-07   12    6    6  1.5e-15  1.8e-15
Solution error estimates for segregated groups
0.087, 0.0085, 0.048, 2.3e-07
Residual error estimates for segregated groups
12, 9.5e+02, 4.2e+03, 0.15
```

```
Segregated solver iteration 7.
Merged variables
Iter      SolEst     Damping    Stepsize #Res #Jac #Sol   LinErr   LinRes
   1       0.033   0.3500000       0.033   26   19   19  9.1e-14  2.9e-15
   2       0.021   0.3500000       0.021   27   20   20  6.9e-14  2.9e-15
   3       0.014   0.3500000       0.014   28   21   21  6.1e-14  2.9e-15
Velocity u2, Pressure p
Iter      SolEst     Damping    Stepsize #Res #Jac #Sol   LinErr   LinRes
   1      0.0085   0.5000000      0.0085   14    7    7  3.3e-15  7.8e-15
Displacement field
Iter      SolEst     Damping    Stepsize #Res #Jac #Sol   LinErr   LinRes
   1       0.054   0.5000000       0.054   14    7    7    3e-14  1.5e-12
Spatial mesh displacement
Iter      SolEst     Damping    Stepsize #Res #Jac #Sol   LinErr   LinRes
   1     3.1e-07   0.5000000     3.1e-07   14    7    7  1.4e-15  1.9e-15
Solution error estimates for segregated groups
0.024, 0.0042, 0.027, 1.5e-07
Residual error estimates for segregated groups
4.1, 4.8e+02, 2.6e+03, 0.15
Segregated solver iteration 8.
Merged variables
Iter      SolEst     Damping    Stepsize #Res #Jac #Sol   LinErr   LinRes
   1      0.0091   0.3500000      0.0091   30   22   22  9.4e-14  3.3e-15
   2      0.0059   0.3500000      0.0059   31   23   23    7e-14  3.1e-15
   3      0.0038   0.3500000      0.0038   32   24   24  9.4e-14  3.4e-15
Velocity u2, Pressure p
Iter      SolEst     Damping    Stepsize #Res #Jac #Sol   LinErr   LinRes
   1      0.0042   0.5000000      0.0042   16    8    8  3.2e-15  7.9e-15
Displacement field
Scales for dependent variables:
Auxiliary pressure (comp1.solid.lemm1.pw): 0.36
Displacement field (comp1.u): 2.8e-16
Iter      SolEst     Damping    Stepsize #Res #Jac #Sol   LinErr   LinRes
   1        0.03   0.5000000        0.03   16    8    8  2.1e-13  1.2e-12
Spatial mesh displacement
Iter      SolEst     Damping    Stepsize #Res #Jac #Sol   LinErr   LinRes
   1       2e-07   0.5000000       2e-07   16    8    8    2e-15  1.8e-15
Solution error estimates for segregated groups
0.0066, 0.0021, 0.015, 9.9e-08
Residual error estimates for segregated groups
1.4, 2.4e+02, 1.8e+03, 0.14
Segregated solver iteration 9.
Merged variables
Iter      SolEst     Damping    Stepsize #Res #Jac #Sol   LinErr   LinRes
   1      0.0025   0.3500000      0.0025   34   25   25  5.4e-14  3.2e-15
   2      0.0016   0.3500000      0.0016   35   26   26    1e-13  3.8e-15
   3      0.0011   0.3500000      0.0011   36   27   27  1.3e-13  2.9e-15
Velocity u2, Pressure p
Iter      SolEst     Damping    Stepsize #Res #Jac #Sol   LinErr   LinRes
   1      0.0021   0.5000000      0.0021   18    9    9  2.2e-15  7.9e-15
Displacement field
Iter      SolEst     Damping    Stepsize #Res #Jac #Sol   LinErr   LinRes
   1       0.017   0.5000000       0.017   18    9    9  9.5e-14  1.2e-12
Spatial mesh displacement
Iter      SolEst     Damping    Stepsize #Res #Jac #Sol   LinErr   LinRes
   1     1.2e-07   0.5000000     1.2e-07   18    9    9  1.5e-15  1.9e-15
```

```
Solution error estimates for segregated groups
0.0018, 0.0011, 0.0085, 6.2e-08
Residual error estimates for segregated groups
0.44, 1.2e+02, 1.4e+03, 0.15
Segregated solver iteration 10.
Merged variables
Iter      SolEst     Damping    Stepsize #Res #Jac #Sol   LinErr   LinRes
   1     0.00069   0.3500000     0.00069   38   28   28  9.5e-14  3.1e-15
   2     0.00045   0.3500000     0.00045   39   29   29  4.4e-14  3.4e-15
   3     0.00029   0.3500000     0.00029   40   30   30  7.1e-14    3e-15
Velocity u2, Pressure p
Iter      SolEst     Damping    Stepsize #Res #Jac #Sol   LinErr   LinRes
   1      0.0011   0.5000000      0.0011   20   10   10  3.4e-15  7.9e-15
Displacement field
Iter      SolEst     Damping    Stepsize #Res #Jac #Sol   LinErr   LinRes
   1      0.0094   0.5000000      0.0094   20   10   10  1.2e-13  1.2e-12
Spatial mesh displacement
Iter      SolEst     Damping    Stepsize #Res #Jac #Sol   LinErr   LinRes
   1     7.5e-08   0.5000000     7.5e-08   20   10   10  1.5e-15  1.9e-15
Solution error estimates for segregated groups
0.0005, 0.00053, 0.0047, 3.8e-08
Residual error estimates for segregated groups
0.14, 60, 1.2e+03, 0.16
Segregated solver iteration 11.
Merged variables
Iter      SolEst     Damping    Stepsize #Res #Jac #Sol   LinErr   LinRes
   1     0.00019   0.3500000     0.00019   42   31   31  6.9e-14    3e-15
   2     0.00012   0.3500000     0.00012   43   32   32  5.1e-14  3.1e-15
   3       8e-05   0.3500000       8e-05   44   33   33  9.6e-14  3.8e-15
Velocity u2, Pressure p
Iter      SolEst     Damping    Stepsize #Res #Jac #Sol   LinErr   LinRes
   1     0.00052   0.5000000     0.00052   22   11   11    2e-15  7.8e-15
Displacement field
Iter      SolEst     Damping    Stepsize #Res #Jac #Sol   LinErr   LinRes
   1      0.0051   0.5000000      0.0051   22   11   11  9.3e-14  1.2e-12
Spatial mesh displacement
Iter      SolEst     Damping    Stepsize #Res #Jac #Sol   LinErr   LinRes
   1     4.5e-08   0.5000000     4.5e-08   22   11   11  1.6e-15  1.8e-15
Solution error estimates for segregated groups
0.00014, 0.00026, 0.0026, 2.3e-08
Residual error estimates for segregated groups
0.043, 30, 1.2e+03, 0.14
Segregated solver iteration 12.
Merged variables
Iter      SolEst     Damping    Stepsize #Res #Jac #Sol   LinErr   LinRes
   1     5.2e-05   0.3500000     5.2e-05   46   34   34  6.4e-14    3e-15
   2     3.4e-05   0.3500000     3.4e-05   47   35   35  1.3e-13  2.8e-15
   3     2.2e-05   0.3500000     2.2e-05   48   36   36  5.8e-14  2.9e-15
Velocity u2, Pressure p
Iter      SolEst     Damping    Stepsize #Res #Jac #Sol   LinErr   LinRes
   1     0.00026   0.5000000     0.00026   24   12   12  3.8e-15  7.9e-15
Displacement field
Iter      SolEst     Damping    Stepsize #Res #Jac #Sol   LinErr   LinRes
   1      0.0028   0.5000000      0.0028   24   12   12    1e-13  1.2e-12
Spatial mesh displacement
Iter      SolEst     Damping    Stepsize #Res #Jac #Sol   LinErr   LinRes
```

```
   1     2.7e-08   0.5000000     2.7e-08   24   12   12  1.6e-15  1.9e-15
Solution error estimates for segregated groups
3.8e-05, 0.00013, 0.0014, 1.3e-08
Residual error estimates for segregated groups
0.013, 15, 1.2e+03, 0.14
Segregated solver iteration 13.
Merged variables
Iter      SolEst     Damping    Stepsize #Res #Jac #Sol   LinErr   LinRes
   1     1.4e-05   0.3500000     1.4e-05   50   37   37  7.6e-14  3.3e-15
   2     9.2e-06   0.3500000     9.2e-06   51   38   38  1.4e-13  3.4e-15
   3       6e-06   0.3500000       6e-06   52   39   39  5.6e-14  3.1e-15
Velocity u2, Pressure p
Iter      SolEst     Damping    Stepsize #Res #Jac #Sol   LinErr   LinRes
   1     0.00013   0.5000000     0.00013   26   13   13  4.6e-15  7.9e-15
Displacement field
Iter      SolEst     Damping    Stepsize #Res #Jac #Sol   LinErr   LinRes
   1      0.0015   0.5000000      0.0015   26   13   13  1.2e-13  1.2e-12
Spatial mesh displacement
Iter      SolEst     Damping    Stepsize #Res #Jac #Sol   LinErr   LinRes
   1     1.6e-08   0.5000000     1.6e-08   26   13   13  1.2e-15  1.8e-15
Solution error estimates for segregated groups
1e-05, 6.6e-05, 0.00076, 7.8e-09
Residual error estimates for segregated groups
0.004, 7.5, 1.2e+03, 0.17
Solution time: 155 s. (2 minutes, 35 seconds)
Physical memory: 4.72 GB
Virtual memory: 25.52 GB
Ended at Jan 31, 2025, 7:42:13 PM.
----- Stationary Solver 1 in study1/Approach Curve Solver (sol1) -------------->
```

d=2190 (su64)

GENERAL

Description	Value
Solution	d=2190

LOG

```
<---- Compile Equations: Stationary in study1/Approach Curve Solver (sol1) -----
Started at Jan 31, 2025, 7:42:13 PM.
Parameter d = 2.19E-6 (m).
Geometry shape function: Linear serendipity
Number of vertex elements: 18
Number of boundary elements: 6168
Number of elements: 155060
Minimum element quality: 0.4739
Time: 8 s.
Physical memory: 2.94 GB
Virtual memory: 15.18 GB
Ended at Jan 31, 2025, 7:42:22 PM.
----- Compile Equations: Stationary in study1/Approach Curve Solver (sol1) ---->

<---- Dependent Variables 1 in study1/Approach Curve Solver (sol1) -------------
Started at Jan 31, 2025, 7:42:22 PM.
Solution time: 0 s.
Physical memory: 2.94 GB
Virtual memory: 15.18 GB
Ended at Jan 31, 2025, 7:42:22 PM.
----- Dependent Variables 1 in study1/Approach Curve Solver (sol1) ------------>

<---- Stationary Solver 1 in study1/Approach Curve Solver (sol1) ---------------
Started at Jan 31, 2025, 7:42:22 PM.
Segregated solver
Segregated Step 11 does not have any components solved for.
Number of degrees of freedom solved for: 787611 (plus 11759 internal DOFs).
Segregated solver iteration 1.
Merged variables
Nonsymmetric matrix found.
Scales for dependent variables:
Concentration (comp1.Cl): 1.4e+02
Concentration (comp1.K): 1.4e+02
Electric potential (comp1.V): 1.9e+02
Orthonormal null-space function used.
Iter      SolEst     Damping    Stepsize #Res #Jac #Sol   LinErr   LinRes
   1        0.83   0.3500000        0.83    2    1    1    3e-11  2.7e-15
   2        0.81   0.3500000        0.81    3    2    2  2.5e-11  2.8e-15
   3         0.8   0.3500000         0.8    4    3    3  3.2e-11  2.8e-15
Velocity u2, Pressure p
Nonsymmetric matrix found.
Scales for dependent variables:
Pressure (comp1.p): 1
Velocity field (spatial frame) (comp1.u2): 0.00018
Orthonormal null-space function used.
Iter      SolEst     Damping    Stepsize #Res #Jac #Sol   LinErr   LinRes
   1         1.1   0.5000000         1.1    2    1    1  1.6e-15  2.3e-15
Displacement field
Nonsymmetric matrix found.
Scales for dependent variables:
Auxiliary pressure (comp1.solid.lemm1.pw): 1
Displacement field (comp1.u): 2.8e-18
Orthonormal null-space function used.
Iter      SolEst     Damping    Stepsize #Res #Jac #Sol   LinErr   LinRes
   1         1.9   0.5000000         1.9    2    1    1  1.2e-13  1.5e-12
Spatial mesh displacement
```

```
Symmetric matrices found.
Scales for dependent variables:
Spatial mesh displacement (comp1.spatial.disp): 1.2e-09
Orthonormal null-space function used.
Iter      SolEst     Damping    Stepsize #Res #Jac #Sol   LinErr   LinRes
   1       7e-07   0.5000000       7e-07    2    1    1  1.9e-15  1.9e-15
Solution error estimates for segregated groups
1.4, 0.54, 0.95, 3.5e-07
Residual error estimates for segregated groups
7.9e+03, 8.2e+04, 2.2e+04, 0.14
Segregated solver iteration 2.
Merged variables
Scales for dependent variables:
Concentration (comp1.Cl): 1.4e+02
Concentration (comp1.K): 1.4e+02
Electric potential (comp1.V): 0.064
Iter      SolEst     Damping    Stepsize #Res #Jac #Sol   LinErr   LinRes
   1        0.78   0.3500000        0.78    6    4    4  9.4e-14  2.6e-15
   2        0.77   0.3500000        0.77    7    5    5  6.2e-14  2.8e-15
   3        0.76   0.3500000        0.76    8    6    6  4.4e-14  2.7e-15
Velocity u2, Pressure p
Scales for dependent variables:
Pressure (comp1.p): 3.6e+02
Velocity field (spatial frame) (comp1.u2): 0.00072
Iter      SolEst     Damping    Stepsize #Res #Jac #Sol   LinErr   LinRes
   1        0.36   0.5000000        0.36    4    2    2  2.3e-15  7.8e-15
Displacement field
Iter      SolEst     Damping    Stepsize #Res #Jac #Sol   LinErr   LinRes
   1        0.95   0.5000000        0.95    4    2    2    9e-14  1.5e-12
Spatial mesh displacement
Iter      SolEst     Damping    Stepsize #Res #Jac #Sol   LinErr   LinRes
   1     1.1e-06   0.5000000     1.1e-06    4    2    2  1.6e-15  1.9e-15
Solution error estimates for segregated groups
1.3, 0.18, 0.48, 5.3e-07
Residual error estimates for segregated groups
7.8e+02, 1.5e+04, 2.2e+04, 0.14
Segregated solver iteration 3.
Merged variables
Iter      SolEst     Damping    Stepsize #Res #Jac #Sol   LinErr   LinRes
   1        0.75   0.3500000        0.75   10    7    7    4e-14  2.6e-15
   2        0.73   0.3500000        0.73   11    8    8  4.8e-14  2.7e-15
   3        0.72   0.3500000        0.72   12    9    9  1.1e-13  2.7e-15
Velocity u2, Pressure p
Iter      SolEst     Damping    Stepsize #Res #Jac #Sol   LinErr   LinRes
   1        0.15   0.5000000        0.15    6    3    3  2.9e-15  7.9e-15
Displacement field
Iter      SolEst     Damping    Stepsize #Res #Jac #Sol   LinErr   LinRes
   1        0.52   0.5000000        0.52    6    3    3    9e-14  1.5e-12
Spatial mesh displacement
Iter      SolEst     Damping    Stepsize #Res #Jac #Sol   LinErr   LinRes
   1     1.1e-06   0.5000000     1.1e-06    6    3    3  1.3e-15  1.8e-15
Solution error estimates for segregated groups
1.2, 0.077, 0.26, 5.3e-07
Residual error estimates for segregated groups
2.2e+02, 8.8e+03, 1.6e+04, 0.15
Segregated solver iteration 4.
```

```
Merged variables
Iter      SolEst     Damping    Stepsize #Res #Jac #Sol   LinErr   LinRes
   1         0.7   0.3500000         0.7   14   10   10  4.9e-14  2.7e-15
   2        0.68   0.3500000        0.68   15   11   11  6.3e-14  2.7e-15
   3        0.59   0.3500000        0.59   16   12   12  5.7e-14  2.6e-15
Velocity u2, Pressure p
Iter      SolEst     Damping    Stepsize #Res #Jac #Sol   LinErr   LinRes
   1       0.072   0.5000000       0.072    8    4    4  2.2e-15  7.9e-15
Displacement field
Iter      SolEst     Damping    Stepsize #Res #Jac #Sol   LinErr   LinRes
   1        0.29   0.5000000        0.29    8    4    4  1.2e-13  1.5e-12
Spatial mesh displacement
Iter      SolEst     Damping    Stepsize #Res #Jac #Sol   LinErr   LinRes
   1     8.8e-07   0.5000000     8.8e-07    8    4    4  1.4e-15  1.8e-15
Solution error estimates for segregated groups
1, 0.036, 0.15, 4.4e-07
Residual error estimates for segregated groups
83, 4.6e+03, 1.1e+04, 0.15
Segregated solver iteration 5.
Merged variables
Iter      SolEst     Damping    Stepsize #Res #Jac #Sol   LinErr   LinRes
   1        0.41   0.3500000        0.41   18   13   13  7.6e-14  2.7e-15
   2        0.27   0.3500000        0.27   19   14   14  6.7e-14  2.7e-15
   3        0.18   0.3500000        0.18   20   15   15  9.5e-14  2.6e-15
Velocity u2, Pressure p
Iter      SolEst     Damping    Stepsize #Res #Jac #Sol   LinErr   LinRes
   1       0.035   0.5000000       0.035   10    5    5  2.7e-15    8e-15
Displacement field
Iter      SolEst     Damping    Stepsize #Res #Jac #Sol   LinErr   LinRes
   1        0.17   0.5000000        0.17   10    5    5  4.3e-14  1.5e-12
Spatial mesh displacement
Iter      SolEst     Damping    Stepsize #Res #Jac #Sol   LinErr   LinRes
   1     6.6e-07   0.5000000     6.6e-07   10    5    5  1.7e-15  1.9e-15
Solution error estimates for segregated groups
0.31, 0.017, 0.084, 3.3e-07
Residual error estimates for segregated groups
32, 2.4e+03, 6.8e+03, 0.15
Segregated solver iteration 6.
Merged variables
Iter      SolEst     Damping    Stepsize #Res #Jac #Sol   LinErr   LinRes
   1        0.12   0.3500000        0.12   22   16   16  7.1e-14  2.7e-15
   2       0.077   0.3500000       0.077   23   17   17  6.3e-14  2.7e-15
   3       0.051   0.3500000       0.051   24   18   18  1.1e-13  2.7e-15
Velocity u2, Pressure p
Iter      SolEst     Damping    Stepsize #Res #Jac #Sol   LinErr   LinRes
   1       0.017   0.5000000       0.017   12    6    6  3.2e-15  7.9e-15
Displacement field
Iter      SolEst     Damping    Stepsize #Res #Jac #Sol   LinErr   LinRes
   1       0.095   0.5000000       0.095   12    6    6  5.9e-14  1.5e-12
Spatial mesh displacement
Iter      SolEst     Damping    Stepsize #Res #Jac #Sol   LinErr   LinRes
   1     4.6e-07   0.5000000     4.6e-07   12    6    6  2.1e-15  1.8e-15
Solution error estimates for segregated groups
0.087, 0.0085, 0.048, 2.3e-07
Residual error estimates for segregated groups
12, 1.2e+03, 4.2e+03, 0.16
```

```
Segregated solver iteration 7.
Merged variables
Iter      SolEst     Damping    Stepsize #Res #Jac #Sol   LinErr   LinRes
   1       0.033   0.3500000       0.033   26   19   19  4.6e-14  2.7e-15
   2       0.021   0.3500000       0.021   27   20   20  7.8e-14  2.7e-15
   3       0.014   0.3500000       0.014   28   21   21  5.4e-14  2.7e-15
Velocity u2, Pressure p
Iter      SolEst     Damping    Stepsize #Res #Jac #Sol   LinErr   LinRes
   1      0.0085   0.5000000      0.0085   14    7    7    2e-15    8e-15
Displacement field
Iter      SolEst     Damping    Stepsize #Res #Jac #Sol   LinErr   LinRes
   1       0.054   0.5000000       0.054   14    7    7  9.6e-14  1.5e-12
Spatial mesh displacement
Iter      SolEst     Damping    Stepsize #Res #Jac #Sol   LinErr   LinRes
   1     3.1e-07   0.5000000     3.1e-07   14    7    7  1.6e-15  1.9e-15
Solution error estimates for segregated groups
0.024, 0.0042, 0.027, 1.5e-07
Residual error estimates for segregated groups
4.1, 6.1e+02, 2.6e+03, 0.15
Segregated solver iteration 8.
Merged variables
Iter      SolEst     Damping    Stepsize #Res #Jac #Sol   LinErr   LinRes
   1      0.0091   0.3500000      0.0091   30   22   22  6.3e-14  2.7e-15
   2      0.0059   0.3500000      0.0059   31   23   23  6.3e-14  2.6e-15
   3      0.0038   0.3500000      0.0038   32   24   24  6.9e-14  2.7e-15
Velocity u2, Pressure p
Iter      SolEst     Damping    Stepsize #Res #Jac #Sol   LinErr   LinRes
   1      0.0042   0.5000000      0.0042   16    8    8    4e-15  7.9e-15
Displacement field
Scales for dependent variables:
Auxiliary pressure (comp1.solid.lemm1.pw): 0.36
Displacement field (comp1.u): 2.8e-16
Iter      SolEst     Damping    Stepsize #Res #Jac #Sol   LinErr   LinRes
   1        0.03   0.5000000        0.03   16    8    8  1.2e-13  1.2e-12
Spatial mesh displacement
Iter      SolEst     Damping    Stepsize #Res #Jac #Sol   LinErr   LinRes
   1       2e-07   0.5000000       2e-07   16    8    8  1.6e-15  1.9e-15
Solution error estimates for segregated groups
0.0066, 0.0021, 0.015, 9.9e-08
Residual error estimates for segregated groups
1.4, 3e+02, 1.8e+03, 0.15
Segregated solver iteration 9.
Merged variables
Iter      SolEst     Damping    Stepsize #Res #Jac #Sol   LinErr   LinRes
   1      0.0025   0.3500000      0.0025   34   25   25  6.1e-14  2.6e-15
   2      0.0016   0.3500000      0.0016   35   26   26  6.4e-14  2.7e-15
   3      0.0011   0.3500000      0.0011   36   27   27  1.1e-13  2.7e-15
Velocity u2, Pressure p
Iter      SolEst     Damping    Stepsize #Res #Jac #Sol   LinErr   LinRes
   1      0.0021   0.5000000      0.0021   18    9    9    3e-15  7.9e-15
Displacement field
Iter      SolEst     Damping    Stepsize #Res #Jac #Sol   LinErr   LinRes
   1       0.017   0.5000000       0.017   18    9    9  9.6e-14  1.2e-12
Spatial mesh displacement
Iter      SolEst     Damping    Stepsize #Res #Jac #Sol   LinErr   LinRes
   1     1.2e-07   0.5000000     1.2e-07   18    9    9  1.7e-15  1.9e-15
```

```
Solution error estimates for segregated groups
0.0018, 0.0011, 0.0085, 6.2e-08
Residual error estimates for segregated groups
0.44, 1.5e+02, 1.4e+03, 0.16
Segregated solver iteration 10.
Merged variables
Iter      SolEst     Damping    Stepsize #Res #Jac #Sol   LinErr   LinRes
   1     0.00069   0.3500000     0.00069   38   28   28    8e-14  2.7e-15
   2     0.00045   0.3500000     0.00045   39   29   29  6.9e-14  2.7e-15
   3     0.00029   0.3500000     0.00029   40   30   30  1.3e-13  2.7e-15
Velocity u2, Pressure p
Iter      SolEst     Damping    Stepsize #Res #Jac #Sol   LinErr   LinRes
   1      0.0011   0.5000000      0.0011   20   10   10  2.2e-15    8e-15
Displacement field
Iter      SolEst     Damping    Stepsize #Res #Jac #Sol   LinErr   LinRes
   1      0.0094   0.5000000      0.0094   20   10   10  1.4e-13  1.2e-12
Spatial mesh displacement
Iter      SolEst     Damping    Stepsize #Res #Jac #Sol   LinErr   LinRes
   1     7.6e-08   0.5000000     7.6e-08   20   10   10  1.4e-15  1.9e-15
Solution error estimates for segregated groups
0.0005, 0.00053, 0.0047, 3.8e-08
Residual error estimates for segregated groups
0.14, 76, 1.2e+03, 0.16
Segregated solver iteration 11.
Merged variables
Iter      SolEst     Damping    Stepsize #Res #Jac #Sol   LinErr   LinRes
   1     0.00019   0.3500000     0.00019   42   31   31  7.7e-14  2.6e-15
   2     0.00012   0.3500000     0.00012   43   32   32  7.2e-14  2.7e-15
   3       8e-05   0.3500000       8e-05   44   33   33  8.4e-14  2.7e-15
Velocity u2, Pressure p
Iter      SolEst     Damping    Stepsize #Res #Jac #Sol   LinErr   LinRes
   1     0.00053   0.5000000     0.00053   22   11   11  2.4e-15  7.8e-15
Displacement field
Iter      SolEst     Damping    Stepsize #Res #Jac #Sol   LinErr   LinRes
   1      0.0051   0.5000000      0.0051   22   11   11  1.5e-13  1.2e-12
Spatial mesh displacement
Iter      SolEst     Damping    Stepsize #Res #Jac #Sol   LinErr   LinRes
   1     4.5e-08   0.5000000     4.5e-08   22   11   11  1.8e-15  1.8e-15
Solution error estimates for segregated groups
0.00014, 0.00026, 0.0026, 2.3e-08
Residual error estimates for segregated groups
0.043, 38, 1.2e+03, 0.14
Segregated solver iteration 12.
Merged variables
Iter      SolEst     Damping    Stepsize #Res #Jac #Sol   LinErr   LinRes
   1     5.2e-05   0.3500000     5.2e-05   46   34   34  7.2e-14  2.7e-15
   2     3.4e-05   0.3500000     3.4e-05   47   35   35  7.2e-14  2.7e-15
   3     2.2e-05   0.3500000     2.2e-05   48   36   36  4.7e-14  2.6e-15
Velocity u2, Pressure p
Iter      SolEst     Damping    Stepsize #Res #Jac #Sol   LinErr   LinRes
   1     0.00026   0.5000000     0.00026   24   12   12  3.6e-15  7.8e-15
Displacement field
Iter      SolEst     Damping    Stepsize #Res #Jac #Sol   LinErr   LinRes
   1      0.0028   0.5000000      0.0028   24   12   12    1e-13  1.2e-12
Spatial mesh displacement
Iter      SolEst     Damping    Stepsize #Res #Jac #Sol   LinErr   LinRes
```

```
   1     2.7e-08   0.5000000     2.7e-08   24   12   12  1.6e-15  1.8e-15
Solution error estimates for segregated groups
3.8e-05, 0.00013, 0.0014, 1.3e-08
Residual error estimates for segregated groups
0.013, 19, 1.2e+03, 0.15
Segregated solver iteration 13.
Merged variables
Iter      SolEst     Damping    Stepsize #Res #Jac #Sol   LinErr   LinRes
   1     1.4e-05   0.3500000     1.4e-05   50   37   37  7.8e-14  2.7e-15
   2     9.2e-06   0.3500000     9.2e-06   51   38   38  5.6e-14  2.6e-15
   3       6e-06   0.3500000       6e-06   52   39   39  5.4e-14  2.7e-15
Velocity u2, Pressure p
Iter      SolEst     Damping    Stepsize #Res #Jac #Sol   LinErr   LinRes
   1     0.00013   0.5000000     0.00013   26   13   13  3.3e-15  7.9e-15
Displacement field
Iter      SolEst     Damping    Stepsize #Res #Jac #Sol   LinErr   LinRes
   1      0.0015   0.5000000      0.0015   26   13   13  1.1e-13  1.2e-12
Spatial mesh displacement
Iter      SolEst     Damping    Stepsize #Res #Jac #Sol   LinErr   LinRes
   1     1.6e-08   0.5000000     1.6e-08   26   13   13  1.4e-15  1.8e-15
Solution error estimates for segregated groups
1e-05, 6.6e-05, 0.00076, 7.8e-09
Residual error estimates for segregated groups
0.004, 9.6, 1.2e+03, 0.14
Solution time: 154 s. (2 minutes, 34 seconds)
Physical memory: 4.66 GB
Virtual memory: 25.51 GB
Ended at Jan 31, 2025, 7:44:56 PM.
----- Stationary Solver 1 in study1/Approach Curve Solver (sol1) -------------->
```

d=2185 (su65)

GENERAL

Description	Value
Solution	d=2185

LOG

```
<---- Compile Equations: Stationary in study1/Approach Curve Solver (sol1) -----
Started at Jan 31, 2025, 7:44:56 PM.
Parameter d = 2.1850000000000003E-6 (m).
Geometry shape function: Linear serendipity
Number of vertex elements: 18
Number of boundary elements: 6166
Number of elements: 155390
Minimum element quality: 0.448
Time: 8 s.
Physical memory: 2.9 GB
Virtual memory: 15.2 GB
Ended at Jan 31, 2025, 7:45:04 PM.
----- Compile Equations: Stationary in study1/Approach Curve Solver (sol1) ---->

<---- Dependent Variables 1 in study1/Approach Curve Solver (sol1) -------------
Started at Jan 31, 2025, 7:45:04 PM.
Solution time: 0 s.
Physical memory: 2.9 GB
Virtual memory: 15.14 GB
Ended at Jan 31, 2025, 7:45:04 PM.
----- Dependent Variables 1 in study1/Approach Curve Solver (sol1) ------------>

<---- Stationary Solver 1 in study1/Approach Curve Solver (sol1) ---------------
Started at Jan 31, 2025, 7:45:05 PM.
Segregated solver
Segregated Step 11 does not have any components solved for.
Number of degrees of freedom solved for: 789155 (plus 11755 internal DOFs).
Segregated solver iteration 1.
Merged variables
Nonsymmetric matrix found.
Scales for dependent variables:
Concentration (comp1.Cl): 1.4e+02
Concentration (comp1.K): 1.4e+02
Electric potential (comp1.V): 1.9e+02
Orthonormal null-space function used.
Iter      SolEst     Damping    Stepsize #Res #Jac #Sol   LinErr   LinRes
   1        0.83   0.3500000        0.83    2    1    1  6.1e-11  2.7e-15
   2        0.81   0.3500000        0.81    3    2    2    3e-11  3.1e-15
   3         0.8   0.3500000         0.8    4    3    3  2.3e-11  2.7e-15
Velocity u2, Pressure p
Nonsymmetric matrix found.
Scales for dependent variables:
Pressure (comp1.p): 1
Velocity field (spatial frame) (comp1.u2): 0.00018
Orthonormal null-space function used.
Iter      SolEst     Damping    Stepsize #Res #Jac #Sol   LinErr   LinRes
   1         1.1   0.5000000         1.1    2    1    1  1.5e-15  2.2e-15
Displacement field
Nonsymmetric matrix found.
Scales for dependent variables:
Auxiliary pressure (comp1.solid.lemm1.pw): 1
Displacement field (comp1.u): 2.7e-18
Orthonormal null-space function used.
Iter      SolEst     Damping    Stepsize #Res #Jac #Sol   LinErr   LinRes
   1         1.9   0.5000000         1.9    2    1    1  1.2e-13  1.5e-12
Spatial mesh displacement
```

```
Symmetric matrices found.
Scales for dependent variables:
Spatial mesh displacement (comp1.spatial.disp): 1.2e-09
Orthonormal null-space function used.
Iter      SolEst     Damping    Stepsize #Res #Jac #Sol   LinErr   LinRes
   1     7.1e-07   0.5000000     7.1e-07    2    1    1  1.9e-15  1.8e-15
Solution error estimates for segregated groups
1.4, 0.54, 0.95, 3.5e-07
Residual error estimates for segregated groups
7.9e+03, 8.2e+04, 2.2e+04, 0.13
Segregated solver iteration 2.
Merged variables
Scales for dependent variables:
Concentration (comp1.Cl): 1.4e+02
Concentration (comp1.K): 1.4e+02
Electric potential (comp1.V): 0.064
Iter      SolEst     Damping    Stepsize #Res #Jac #Sol   LinErr   LinRes
   1        0.78   0.3500000        0.78    6    4    4  9.8e-14  3.4e-15
   2        0.77   0.3500000        0.77    7    5    5  9.6e-14    6e-15
   3        0.76   0.3500000        0.76    8    6    6  7.5e-14  6.5e-15
Velocity u2, Pressure p
Scales for dependent variables:
Pressure (comp1.p): 3.6e+02
Velocity field (spatial frame) (comp1.u2): 0.00072
Iter      SolEst     Damping    Stepsize #Res #Jac #Sol   LinErr   LinRes
   1        0.36   0.5000000        0.36    4    2    2  2.3e-15  7.8e-15
Displacement field
Iter      SolEst     Damping    Stepsize #Res #Jac #Sol   LinErr   LinRes
   1        0.95   0.5000000        0.95    4    2    2  4.8e-14  1.5e-12
Spatial mesh displacement
Iter      SolEst     Damping    Stepsize #Res #Jac #Sol   LinErr   LinRes
   1     1.1e-06   0.5000000     1.1e-06    4    2    2  1.3e-15  1.8e-15
Solution error estimates for segregated groups
1.3, 0.18, 0.48, 5.3e-07
Residual error estimates for segregated groups
7.8e+02, 1.5e+04, 2.2e+04, 0.14
Segregated solver iteration 3.
Merged variables
Iter      SolEst     Damping    Stepsize #Res #Jac #Sol   LinErr   LinRes
   1        0.75   0.3500000        0.75   10    7    7  9.4e-14  5.1e-15
   2        0.73   0.3500000        0.73   11    8    8  1.3e-13  3.3e-15
   3        0.72   0.3500000        0.72   12    9    9  7.2e-14  3.4e-15
Velocity u2, Pressure p
Iter      SolEst     Damping    Stepsize #Res #Jac #Sol   LinErr   LinRes
   1        0.15   0.5000000        0.15    6    3    3  2.5e-15  7.7e-15
Displacement field
Iter      SolEst     Damping    Stepsize #Res #Jac #Sol   LinErr   LinRes
   1        0.52   0.5000000        0.52    6    3    3  9.7e-14  1.5e-12
Spatial mesh displacement
Iter      SolEst     Damping    Stepsize #Res #Jac #Sol   LinErr   LinRes
   1     1.1e-06   0.5000000     1.1e-06    6    3    3  1.8e-15  1.8e-15
Solution error estimates for segregated groups
1.2, 0.077, 0.26, 5.3e-07
Residual error estimates for segregated groups
2.2e+02, 8.7e+03, 1.6e+04, 0.16
Segregated solver iteration 4.
```

```
Merged variables
Iter      SolEst     Damping    Stepsize #Res #Jac #Sol   LinErr   LinRes
   1         0.7   0.3500000         0.7   14   10   10  4.8e-14  4.5e-15
   2        0.68   0.3500000        0.68   15   11   11  1.6e-13  1.4e-14
   3        0.59   0.3500000        0.59   16   12   12    7e-14  3.4e-15
Velocity u2, Pressure p
Iter      SolEst     Damping    Stepsize #Res #Jac #Sol   LinErr   LinRes
   1       0.072   0.5000000       0.072    8    4    4  5.4e-15  7.8e-15
Displacement field
Iter      SolEst     Damping    Stepsize #Res #Jac #Sol   LinErr   LinRes
   1        0.29   0.5000000        0.29    8    4    4  6.2e-14  1.5e-12
Spatial mesh displacement
Iter      SolEst     Damping    Stepsize #Res #Jac #Sol   LinErr   LinRes
   1     8.8e-07   0.5000000     8.8e-07    8    4    4  1.4e-15  1.8e-15
Solution error estimates for segregated groups
1, 0.036, 0.15, 4.4e-07
Residual error estimates for segregated groups
83, 4.6e+03, 1.1e+04, 0.15
Segregated solver iteration 5.
Merged variables
Iter      SolEst     Damping    Stepsize #Res #Jac #Sol   LinErr   LinRes
   1        0.41   0.3500000        0.41   18   13   13  6.4e-14  3.7e-15
   2        0.27   0.3500000        0.27   19   14   14    1e-13  3.7e-15
   3        0.18   0.3500000        0.18   20   15   15  9.5e-14  7.6e-15
Velocity u2, Pressure p
Iter      SolEst     Damping    Stepsize #Res #Jac #Sol   LinErr   LinRes
   1       0.035   0.5000000       0.035   10    5    5    3e-15  7.8e-15
Displacement field
Iter      SolEst     Damping    Stepsize #Res #Jac #Sol   LinErr   LinRes
   1        0.17   0.5000000        0.17   10    5    5  7.6e-14  1.5e-12
Spatial mesh displacement
Iter      SolEst     Damping    Stepsize #Res #Jac #Sol   LinErr   LinRes
   1     6.6e-07   0.5000000     6.6e-07   10    5    5  1.6e-15  1.8e-15
Solution error estimates for segregated groups
0.31, 0.017, 0.084, 3.3e-07
Residual error estimates for segregated groups
32, 2.4e+03, 6.8e+03, 0.15
Segregated solver iteration 6.
Merged variables
Iter      SolEst     Damping    Stepsize #Res #Jac #Sol   LinErr   LinRes
   1        0.12   0.3500000        0.12   22   16   16  7.5e-14  5.2e-15
   2       0.078   0.3500000       0.078   23   17   17  1.4e-13  7.5e-15
   3       0.051   0.3500000       0.051   24   18   18  9.8e-14  3.7e-15
Velocity u2, Pressure p
Iter      SolEst     Damping    Stepsize #Res #Jac #Sol   LinErr   LinRes
   1       0.017   0.5000000       0.017   12    6    6  3.2e-15  7.8e-15
Displacement field
Iter      SolEst     Damping    Stepsize #Res #Jac #Sol   LinErr   LinRes
   1       0.095   0.5000000       0.095   12    6    6  1.2e-13  1.5e-12
Spatial mesh displacement
Iter      SolEst     Damping    Stepsize #Res #Jac #Sol   LinErr   LinRes
   1     4.6e-07   0.5000000     4.6e-07   12    6    6  1.3e-15  1.8e-15
Solution error estimates for segregated groups
0.087, 0.0085, 0.048, 2.3e-07
Residual error estimates for segregated groups
12, 1.2e+03, 4.2e+03, 0.15
```

```
Segregated solver iteration 7.
Merged variables
Iter      SolEst     Damping    Stepsize #Res #Jac #Sol   LinErr   LinRes
   1       0.033   0.3500000       0.033   26   19   19  5.7e-14    6e-15
   2       0.021   0.3500000       0.021   27   20   20  1.4e-13  1.1e-14
   3       0.014   0.3500000       0.014   28   21   21  1.1e-13  3.6e-15
Velocity u2, Pressure p
Iter      SolEst     Damping    Stepsize #Res #Jac #Sol   LinErr   LinRes
   1      0.0085   0.5000000      0.0085   14    7    7  3.5e-15  7.8e-15
Displacement field
Scales for dependent variables:
Auxiliary pressure (comp1.solid.lemm1.pw): 0.35
Displacement field (comp1.u): 2.8e-16
Iter      SolEst     Damping    Stepsize #Res #Jac #Sol   LinErr   LinRes
   1       0.054   0.5000000       0.054   14    7    7  1.5e-13  1.2e-12
Spatial mesh displacement
Iter      SolEst     Damping    Stepsize #Res #Jac #Sol   LinErr   LinRes
   1     3.1e-07   0.5000000     3.1e-07   14    7    7  1.6e-15  1.8e-15
Solution error estimates for segregated groups
0.024, 0.0042, 0.027, 1.5e-07
Residual error estimates for segregated groups
4.1, 6e+02, 2.6e+03, 0.14
Segregated solver iteration 8.
Merged variables
Iter      SolEst     Damping    Stepsize #Res #Jac #Sol   LinErr   LinRes
   1      0.0091   0.3500000      0.0091   30   22   22  1.1e-13  4.6e-15
   2      0.0059   0.3500000      0.0059   31   23   23  5.9e-14  7.7e-15
   3      0.0038   0.3500000      0.0038   32   24   24  1.3e-13  5.7e-15
Velocity u2, Pressure p
Iter      SolEst     Damping    Stepsize #Res #Jac #Sol   LinErr   LinRes
   1      0.0042   0.5000000      0.0042   16    8    8  1.9e-15  7.9e-15
Displacement field
Iter      SolEst     Damping    Stepsize #Res #Jac #Sol   LinErr   LinRes
   1        0.03   0.5000000        0.03   16    8    8  1.1e-13  1.2e-12
Spatial mesh displacement
Iter      SolEst     Damping    Stepsize #Res #Jac #Sol   LinErr   LinRes
   1       2e-07   0.5000000       2e-07   16    8    8  1.8e-15  1.8e-15
Solution error estimates for segregated groups
0.0066, 0.0021, 0.015, 9.9e-08
Residual error estimates for segregated groups
1.4, 3e+02, 1.8e+03, 0.15
Segregated solver iteration 9.
Merged variables
Iter      SolEst     Damping    Stepsize #Res #Jac #Sol   LinErr   LinRes
   1      0.0025   0.3500000      0.0025   34   25   25  6.6e-14  3.8e-15
   2      0.0016   0.3500000      0.0016   35   26   26  8.3e-14  3.9e-15
   3      0.0011   0.3500000      0.0011   36   27   27  1.1e-13  5.5e-15
Velocity u2, Pressure p
Iter      SolEst     Damping    Stepsize #Res #Jac #Sol   LinErr   LinRes
   1      0.0021   0.5000000      0.0021   18    9    9  2.6e-15  7.8e-15
Displacement field
Iter      SolEst     Damping    Stepsize #Res #Jac #Sol   LinErr   LinRes
   1       0.017   0.5000000       0.017   18    9    9  1.1e-13  1.2e-12
Spatial mesh displacement
Iter      SolEst     Damping    Stepsize #Res #Jac #Sol   LinErr   LinRes
   1     1.2e-07   0.5000000     1.2e-07   18    9    9  1.4e-15  1.8e-15
```

```
Solution error estimates for segregated groups
0.0018, 0.0011, 0.0085, 6.2e-08
Residual error estimates for segregated groups
0.44, 1.5e+02, 1.4e+03, 0.16
Segregated solver iteration 10.
Merged variables
Iter      SolEst     Damping    Stepsize #Res #Jac #Sol   LinErr   LinRes
   1     0.00069   0.3500000     0.00069   38   28   28  7.6e-14  4.3e-15
   2     0.00045   0.3500000     0.00045   39   29   29  1.4e-13  3.4e-15
   3     0.00029   0.3500000     0.00029   40   30   30  9.7e-14  4.1e-15
Velocity u2, Pressure p
Iter      SolEst     Damping    Stepsize #Res #Jac #Sol   LinErr   LinRes
   1      0.0011   0.5000000      0.0011   20   10   10  2.8e-15  7.8e-15
Displacement field
Iter      SolEst     Damping    Stepsize #Res #Jac #Sol   LinErr   LinRes
   1      0.0094   0.5000000      0.0094   20   10   10    1e-13  1.2e-12
Spatial mesh displacement
Iter      SolEst     Damping    Stepsize #Res #Jac #Sol   LinErr   LinRes
   1     7.6e-08   0.5000000     7.6e-08   20   10   10  1.5e-15  1.8e-15
Solution error estimates for segregated groups
0.0005, 0.00053, 0.0047, 3.8e-08
Residual error estimates for segregated groups
0.14, 76, 1.2e+03, 0.15
Segregated solver iteration 11.
Merged variables
Iter      SolEst     Damping    Stepsize #Res #Jac #Sol   LinErr   LinRes
   1     0.00019   0.3500000     0.00019   42   31   31  1.2e-13  1.2e-14
   2     0.00012   0.3500000     0.00012   43   32   32  9.6e-14  3.4e-15
   3       8e-05   0.3500000       8e-05   44   33   33  1.1e-13  7.1e-15
Velocity u2, Pressure p
Iter      SolEst     Damping    Stepsize #Res #Jac #Sol   LinErr   LinRes
   1     0.00052   0.5000000     0.00052   22   11   11  2.5e-15  7.9e-15
Displacement field
Iter      SolEst     Damping    Stepsize #Res #Jac #Sol   LinErr   LinRes
   1      0.0051   0.5000000      0.0051   22   11   11    2e-13  1.2e-12
Spatial mesh displacement
Iter      SolEst     Damping    Stepsize #Res #Jac #Sol   LinErr   LinRes
   1     4.5e-08   0.5000000     4.5e-08   22   11   11  1.9e-15  1.8e-15
Solution error estimates for segregated groups
0.00014, 0.00026, 0.0026, 2.3e-08
Residual error estimates for segregated groups
0.043, 38, 1.2e+03, 0.14
Segregated solver iteration 12.
Merged variables
Iter      SolEst     Damping    Stepsize #Res #Jac #Sol   LinErr   LinRes
   1     5.2e-05   0.3500000     5.2e-05   46   34   34  7.5e-14  4.7e-15
   2     3.4e-05   0.3500000     3.4e-05   47   35   35  1.2e-13  4.6e-15
   3     2.2e-05   0.3500000     2.2e-05   48   36   36  5.9e-14  7.5e-15
Velocity u2, Pressure p
Iter      SolEst     Damping    Stepsize #Res #Jac #Sol   LinErr   LinRes
   1     0.00026   0.5000000     0.00026   24   12   12  3.5e-15  7.8e-15
Displacement field
Iter      SolEst     Damping    Stepsize #Res #Jac #Sol   LinErr   LinRes
   1      0.0028   0.5000000      0.0028   24   12   12  2.1e-13  1.2e-12
Spatial mesh displacement
Iter      SolEst     Damping    Stepsize #Res #Jac #Sol   LinErr   LinRes
```

```
   1     2.7e-08   0.5000000     2.7e-08   24    12    12  1.5e-15  1.8e-15
Solution error estimates for segregated groups
3.8e-05, 0.00013, 0.0014, 1.3e-08
Residual error estimates for segregated groups
0.013, 19, 1.2e+03, 0.15
Segregated solver iteration 13.
Merged variables
Iter      SolEst      Damping    Stepsize #Res #Jac #Sol   LinErr   LinRes
   1     1.4e-05   0.3500000     1.4e-05   50    37    37  1.1e-13  3.1e-15
   2     9.2e-06   0.3500000     9.2e-06   51    38    38  7.4e-14    6e-15
   3       6e-06   0.3500000       6e-06   52    39    39  8.3e-14  5.1e-15
Velocity u2, Pressure p
Iter      SolEst      Damping    Stepsize #Res #Jac #Sol   LinErr   LinRes
   1     0.00013   0.5000000     0.00013   26    13    13  2.4e-15  7.8e-15
Displacement field
Iter      SolEst      Damping    Stepsize #Res #Jac #Sol   LinErr   LinRes
   1      0.0015   0.5000000      0.0015   26    13    13  1.1e-13  1.2e-12
Spatial mesh displacement
Iter      SolEst      Damping    Stepsize #Res #Jac #Sol   LinErr   LinRes
   1     1.6e-08   0.5000000     1.6e-08   26    13    13  1.5e-15  1.9e-15
Solution error estimates for segregated groups
1e-05, 6.6e-05, 0.00076, 7.8e-09
Residual error estimates for segregated groups
0.004, 9.5, 1.2e+03, 0.15
Solution time: 155 s. (2 minutes, 35 seconds)
Physical memory: 4.67 GB
Virtual memory: 25.52 GB
Ended at Jan 31, 2025, 7:47:39 PM.
----- Stationary Solver 1 in study1/Approach Curve Solver (sol1) -------------->
```

d=2180 (su66)

GENERAL

Description	Value
Solution	d=2180

LOG

```
<---- Compile Equations: Stationary in study1/Approach Curve Solver (sol1) -----
Started at Jan 31, 2025, 7:47:40 PM.
Parameter d = 2.1800000000000003E-6 (m).
Geometry shape function: Linear serendipity
Number of vertex elements: 18
Number of boundary elements: 6156
Number of elements: 155130
Minimum element quality: 0.4803
Time: 8 s.
Physical memory: 2.97 GB
Virtual memory: 15.27 GB
Ended at Jan 31, 2025, 7:47:48 PM.
----- Compile Equations: Stationary in study1/Approach Curve Solver (sol1) ---->

<---- Dependent Variables 1 in study1/Approach Curve Solver (sol1) -------------
Started at Jan 31, 2025, 7:47:48 PM.
Solution time: 0 s.
Physical memory: 2.97 GB
Virtual memory: 15.21 GB
Ended at Jan 31, 2025, 7:47:48 PM.
----- Dependent Variables 1 in study1/Approach Curve Solver (sol1) ------------>

<---- Stationary Solver 1 in study1/Approach Curve Solver (sol1) ---------------
Started at Jan 31, 2025, 7:47:48 PM.
Segregated solver
Segregated Step 11 does not have any components solved for.
Number of degrees of freedom solved for: 787660 (plus 11735 internal DOFs).
Segregated solver iteration 1.
Merged variables
Nonsymmetric matrix found.
Scales for dependent variables:
Concentration (comp1.Cl): 1.4e+02
Concentration (comp1.K): 1.4e+02
Electric potential (comp1.V): 1.9e+02
Orthonormal null-space function used.
Iter      SolEst     Damping    Stepsize #Res #Jac #Sol   LinErr   LinRes
   1        0.83   0.3500000        0.83    2    1    1  1.9e-11  2.9e-15
   2        0.81   0.3500000        0.81    3    2    2  1.6e-11  2.7e-15
   3         0.8   0.3500000         0.8    4    3    3  1.8e-11  2.7e-15
Velocity u2, Pressure p
Nonsymmetric matrix found.
Scales for dependent variables:
Pressure (comp1.p): 1
Velocity field (spatial frame) (comp1.u2): 0.00018
Orthonormal null-space function used.
Iter      SolEst     Damping    Stepsize #Res #Jac #Sol   LinErr   LinRes
   1         1.1   0.5000000         1.1    2    1    1  1.6e-15  2.3e-15
Displacement field
Nonsymmetric matrix found.
Scales for dependent variables:
Auxiliary pressure (comp1.solid.lemm1.pw): 1
Displacement field (comp1.u): 2.8e-18
Orthonormal null-space function used.
Iter      SolEst     Damping    Stepsize #Res #Jac #Sol   LinErr   LinRes
   1         1.9   0.5000000         1.9    2    1    1  3.9e-14  1.5e-12
Spatial mesh displacement
```

```
Symmetric matrices found.
Scales for dependent variables:
Spatial mesh displacement (comp1.spatial.disp): 1.2e-09
Orthonormal null-space function used.
Iter      SolEst     Damping    Stepsize #Res #Jac #Sol   LinErr   LinRes
   1     7.1e-07   0.5000000     7.1e-07    2    1    1  1.5e-15  1.8e-15
Solution error estimates for segregated groups
1.4, 0.54, 0.95, 3.6e-07
Residual error estimates for segregated groups
7.8e+03, 8.2e+04, 2.2e+04, 0.14
Segregated solver iteration 2.
Merged variables
Scales for dependent variables:
Concentration (comp1.Cl): 1.4e+02
Concentration (comp1.K): 1.4e+02
Electric potential (comp1.V): 0.064
Iter      SolEst     Damping    Stepsize #Res #Jac #Sol   LinErr   LinRes
   1        0.78   0.3500000        0.78    6    4    4    7e-14  2.9e-15
   2        0.77   0.3500000        0.77    7    5    5  6.4e-14  2.8e-15
   3        0.76   0.3500000        0.76    8    6    6  6.4e-14  2.8e-15
Velocity u2, Pressure p
Scales for dependent variables:
Pressure (comp1.p): 3.6e+02
Velocity field (spatial frame) (comp1.u2): 0.00073
Iter      SolEst     Damping    Stepsize #Res #Jac #Sol   LinErr   LinRes
   1        0.36   0.5000000        0.36    4    2    2  2.5e-15    8e-15
Displacement field
Iter      SolEst     Damping    Stepsize #Res #Jac #Sol   LinErr   LinRes
   1        0.95   0.5000000        0.95    4    2    2  1.1e-13  1.5e-12
Spatial mesh displacement
Iter      SolEst     Damping    Stepsize #Res #Jac #Sol   LinErr   LinRes
   1     1.1e-06   0.5000000     1.1e-06    4    2    2  1.9e-15  1.8e-15
Solution error estimates for segregated groups
1.3, 0.18, 0.48, 5.3e-07
Residual error estimates for segregated groups
7.8e+02, 1.7e+04, 2.2e+04, 0.15
Segregated solver iteration 3.
Merged variables
Iter      SolEst     Damping    Stepsize #Res #Jac #Sol   LinErr   LinRes
   1        0.75   0.3500000        0.75   10    7    7  6.8e-14  2.9e-15
   2        0.73   0.3500000        0.73   11    8    8  8.5e-14  3.2e-15
   3        0.72   0.3500000        0.72   12    9    9  6.9e-14  2.9e-15
Velocity u2, Pressure p
Iter      SolEst     Damping    Stepsize #Res #Jac #Sol   LinErr   LinRes
   1        0.15   0.5000000        0.15    6    3    3  3.4e-15  7.8e-15
Displacement field
Iter      SolEst     Damping    Stepsize #Res #Jac #Sol   LinErr   LinRes
   1        0.52   0.5000000        0.52    6    3    3  9.9e-14  1.5e-12
Spatial mesh displacement
Iter      SolEst     Damping    Stepsize #Res #Jac #Sol   LinErr   LinRes
   1     1.1e-06   0.5000000     1.1e-06    6    3    3  1.7e-15  1.9e-15
Solution error estimates for segregated groups
1.2, 0.077, 0.26, 5.3e-07
Residual error estimates for segregated groups
2.2e+02, 9.5e+03, 1.6e+04, 0.14
Segregated solver iteration 4.
```

```
Merged variables
Iter      SolEst     Damping    Stepsize #Res #Jac #Sol   LinErr   LinRes
   1         0.7   0.3500000         0.7   14   10   10  7.8e-14    3e-15
   2        0.68   0.3500000        0.68   15   11   11  1.2e-13  2.8e-15
   3        0.59   0.3500000        0.59   16   12   12  3.8e-14  3.1e-15
Velocity u2, Pressure p
Iter      SolEst     Damping    Stepsize #Res #Jac #Sol   LinErr   LinRes
   1       0.072   0.5000000       0.072    8    4    4  3.5e-15  7.9e-15
Displacement field
Iter      SolEst     Damping    Stepsize #Res #Jac #Sol   LinErr   LinRes
   1        0.29   0.5000000        0.29    8    4    4    1e-13  1.5e-12
Spatial mesh displacement
Iter      SolEst     Damping    Stepsize #Res #Jac #Sol   LinErr   LinRes
   1     8.9e-07   0.5000000     8.9e-07    8    4    4  1.4e-15  1.9e-15
Solution error estimates for segregated groups
1, 0.036, 0.15, 4.5e-07
Residual error estimates for segregated groups
83, 5e+03, 1.1e+04, 0.16
Segregated solver iteration 5.
Merged variables
Iter      SolEst     Damping    Stepsize #Res #Jac #Sol   LinErr   LinRes
   1        0.41   0.3500000        0.41   18   13   13  8.4e-14  3.1e-15
   2        0.27   0.3500000        0.27   19   14   14  6.8e-14    3e-15
   3        0.18   0.3500000        0.18   20   15   15  7.2e-14    3e-15
Velocity u2, Pressure p
Iter      SolEst     Damping    Stepsize #Res #Jac #Sol   LinErr   LinRes
   1       0.035   0.5000000       0.035   10    5    5    3e-15  7.9e-15
Displacement field
Iter      SolEst     Damping    Stepsize #Res #Jac #Sol   LinErr   LinRes
   1        0.17   0.5000000        0.17   10    5    5  6.1e-14  1.5e-12
Spatial mesh displacement
Iter      SolEst     Damping    Stepsize #Res #Jac #Sol   LinErr   LinRes
   1     6.7e-07   0.5000000     6.7e-07   10    5    5  1.5e-15  1.9e-15
Solution error estimates for segregated groups
0.31, 0.017, 0.084, 3.3e-07
Residual error estimates for segregated groups
32, 2.6e+03, 6.8e+03, 0.16
Segregated solver iteration 6.
Merged variables
Iter      SolEst     Damping    Stepsize #Res #Jac #Sol   LinErr   LinRes
   1        0.12   0.3500000        0.12   22   16   16  5.6e-14  3.1e-15
   2       0.077   0.3500000       0.077   23   17   17  9.6e-14  2.9e-15
   3        0.05   0.3500000        0.05   24   18   18  4.7e-14  2.9e-15
Velocity u2, Pressure p
Iter      SolEst     Damping    Stepsize #Res #Jac #Sol   LinErr   LinRes
   1       0.017   0.5000000       0.017   12    6    6  2.5e-15  7.9e-15
Displacement field
Iter      SolEst     Damping    Stepsize #Res #Jac #Sol   LinErr   LinRes
   1       0.095   0.5000000       0.095   12    6    6  8.7e-14  1.5e-12
Spatial mesh displacement
Iter      SolEst     Damping    Stepsize #Res #Jac #Sol   LinErr   LinRes
   1     4.7e-07   0.5000000     4.7e-07   12    6    6  1.4e-15  1.9e-15
Solution error estimates for segregated groups
0.087, 0.0085, 0.048, 2.3e-07
Residual error estimates for segregated groups
12, 1.3e+03, 4.2e+03, 0.15
```

```
Segregated solver iteration 7.
Merged variables
Iter      SolEst     Damping    Stepsize #Res #Jac #Sol   LinErr   LinRes
   1       0.033   0.3500000       0.033   26   19   19  8.5e-14  3.1e-15
   2       0.021   0.3500000       0.021   27   20   20  6.1e-14  2.9e-15
   3       0.014   0.3500000       0.014   28   21   21  1.1e-13  3.3e-15
Velocity u2, Pressure p
Iter      SolEst     Damping    Stepsize #Res #Jac #Sol   LinErr   LinRes
   1      0.0085   0.5000000      0.0085   14    7    7  3.6e-15  7.9e-15
Displacement field
Scales for dependent variables:
Auxiliary pressure (comp1.solid.lemm1.pw): 0.36
Displacement field (comp1.u): 2.8e-16
Iter      SolEst     Damping    Stepsize #Res #Jac #Sol   LinErr   LinRes
   1       0.054   0.5000000       0.054   14    7    7  1.1e-13  1.2e-12
Spatial mesh displacement
Iter      SolEst     Damping    Stepsize #Res #Jac #Sol   LinErr   LinRes
   1     3.1e-07   0.5000000     3.1e-07   14    7    7  1.4e-15  1.8e-15
Solution error estimates for segregated groups
0.024, 0.0042, 0.027, 1.6e-07
Residual error estimates for segregated groups
4.1, 6.6e+02, 2.6e+03, 0.14
Segregated solver iteration 8.
Merged variables
Iter      SolEst     Damping    Stepsize #Res #Jac #Sol   LinErr   LinRes
   1      0.0091   0.3500000      0.0091   30   22   22  5.4e-14  2.8e-15
   2      0.0059   0.3500000      0.0059   31   23   23    8e-14  2.8e-15
   3      0.0038   0.3500000      0.0038   32   24   24    7e-14  2.8e-15
Velocity u2, Pressure p
Iter      SolEst     Damping    Stepsize #Res #Jac #Sol   LinErr   LinRes
   1      0.0042   0.5000000      0.0042   16    8    8  2.4e-15    8e-15
Displacement field
Iter      SolEst     Damping    Stepsize #Res #Jac #Sol   LinErr   LinRes
   1        0.03   0.5000000        0.03   16    8    8  1.2e-13  1.2e-12
Spatial mesh displacement
Iter      SolEst     Damping    Stepsize #Res #Jac #Sol   LinErr   LinRes
   1       2e-07   0.5000000       2e-07   16    8    8  1.5e-15  1.8e-15
Solution error estimates for segregated groups
0.0066, 0.0021, 0.015, 1e-07
Residual error estimates for segregated groups
1.4, 3.3e+02, 1.8e+03, 0.14
Segregated solver iteration 9.
Merged variables
Iter      SolEst     Damping    Stepsize #Res #Jac #Sol   LinErr   LinRes
   1      0.0025   0.3500000      0.0025   34   25   25    8e-14  2.8e-15
   2      0.0016   0.3500000      0.0016   35   26   26    1e-13  2.9e-15
   3      0.0011   0.3500000      0.0011   36   27   27  9.4e-14  3.3e-15
Velocity u2, Pressure p
Iter      SolEst     Damping    Stepsize #Res #Jac #Sol   LinErr   LinRes
   1      0.0021   0.5000000      0.0021   18    9    9  2.7e-15  7.9e-15
Displacement field
Iter      SolEst     Damping    Stepsize #Res #Jac #Sol   LinErr   LinRes
   1       0.017   0.5000000       0.017   18    9    9  1.2e-13  1.2e-12
Spatial mesh displacement
Iter      SolEst     Damping    Stepsize #Res #Jac #Sol   LinErr   LinRes
   1     1.3e-07   0.5000000     1.3e-07   18    9    9  1.9e-15  1.8e-15
```

```
Solution error estimates for segregated groups
0.0018, 0.0011, 0.0085, 6.3e-08
Residual error estimates for segregated groups
0.44, 1.7e+02, 1.4e+03, 0.14
Segregated solver iteration 10.
Merged variables
Iter      SolEst     Damping    Stepsize #Res #Jac #Sol   LinErr   LinRes
   1     0.00068   0.3500000     0.00068   38   28   28  1.2e-13    3e-15
   2     0.00044   0.3500000     0.00044   39   29   29  4.5e-14    3e-15
   3     0.00029   0.3500000     0.00029   40   30   30  7.1e-14  3.1e-15
Velocity u2, Pressure p
Iter      SolEst     Damping    Stepsize #Res #Jac #Sol   LinErr   LinRes
   1      0.0011   0.5000000      0.0011   20   10   10  2.1e-15  7.9e-15
Displacement field
Iter      SolEst     Damping    Stepsize #Res #Jac #Sol   LinErr   LinRes
   1      0.0094   0.5000000      0.0094   20   10   10  1.9e-13  1.2e-12
Spatial mesh displacement
Iter      SolEst     Damping    Stepsize #Res #Jac #Sol   LinErr   LinRes
   1     7.7e-08   0.5000000     7.7e-08   20   10   10  1.8e-15  1.8e-15
Solution error estimates for segregated groups
0.0005, 0.00053, 0.0047, 3.8e-08
Residual error estimates for segregated groups
0.14, 83, 1.2e+03, 0.15
Segregated solver iteration 11.
Merged variables
Iter      SolEst     Damping    Stepsize #Res #Jac #Sol   LinErr   LinRes
   1     0.00019   0.3500000     0.00019   42   31   31    7e-14    3e-15
   2     0.00012   0.3500000     0.00012   43   32   32  8.1e-14    3e-15
   3     7.9e-05   0.3500000     7.9e-05   44   33   33  7.9e-14  2.7e-15
Velocity u2, Pressure p
Iter      SolEst     Damping    Stepsize #Res #Jac #Sol   LinErr   LinRes
   1     0.00053   0.5000000     0.00053   22   11   11  3.6e-15    8e-15
Displacement field
Iter      SolEst     Damping    Stepsize #Res #Jac #Sol   LinErr   LinRes
   1      0.0051   0.5000000      0.0051   22   11   11    1e-13  1.2e-12
Spatial mesh displacement
Iter      SolEst     Damping    Stepsize #Res #Jac #Sol   LinErr   LinRes
   1     4.6e-08   0.5000000     4.6e-08   22   11   11  1.5e-15  1.8e-15
Solution error estimates for segregated groups
0.00014, 0.00026, 0.0026, 2.3e-08
Residual error estimates for segregated groups
0.043, 42, 1.2e+03, 0.15
Segregated solver iteration 12.
Merged variables
Iter      SolEst     Damping    Stepsize #Res #Jac #Sol   LinErr   LinRes
   1     5.2e-05   0.3500000     5.2e-05   46   34   34  9.7e-14  2.9e-15
   2     3.4e-05   0.3500000     3.4e-05   47   35   35    7e-14    3e-15
   3     2.2e-05   0.3500000     2.2e-05   48   36   36  6.5e-14    3e-15
Velocity u2, Pressure p
Iter      SolEst     Damping    Stepsize #Res #Jac #Sol   LinErr   LinRes
   1     0.00026   0.5000000     0.00026   24   12   12  2.5e-15  7.8e-15
Displacement field
Iter      SolEst     Damping    Stepsize #Res #Jac #Sol   LinErr   LinRes
   1      0.0028   0.5000000      0.0028   24   12   12  1.2e-13  1.2e-12
Spatial mesh displacement
Iter      SolEst     Damping    Stepsize #Res #Jac #Sol   LinErr   LinRes
```

```
   1      2.7e-08   0.5000000     2.7e-08   24   12   12  1.4e-15  1.8e-15
Solution error estimates for segregated groups
3.7e-05, 0.00013, 0.0014, 1.4e-08
Residual error estimates for segregated groups
0.013, 21, 1.2e+03, 0.15
Segregated solver iteration 13.
Merged variables
Iter      SolEst     Damping    Stepsize #Res #Jac #Sol   LinErr   LinRes
   1      1.4e-05   0.3500000     1.4e-05   50   37   37    9e-14    3e-15
   2      9.2e-06   0.3500000     9.2e-06   51   38   38  7.3e-14  2.9e-15
   3        6e-06   0.3500000       6e-06   52   39   39    9e-14    3e-15
Velocity u2, Pressure p
Iter      SolEst     Damping    Stepsize #Res #Jac #Sol   LinErr   LinRes
   1      0.00013   0.5000000     0.00013   26   13   13  3.3e-15  7.9e-15
Displacement field
Iter      SolEst     Damping    Stepsize #Res #Jac #Sol   LinErr   LinRes
   1       0.0015   0.5000000      0.0015   26   13   13  1.1e-13  1.2e-12
Spatial mesh displacement
Iter      SolEst     Damping    Stepsize #Res #Jac #Sol   LinErr   LinRes
   1      1.6e-08   0.5000000     1.6e-08   26   13   13  1.5e-15  1.9e-15
Solution error estimates for segregated groups
1e-05, 6.6e-05, 0.00076, 7.9e-09
Residual error estimates for segregated groups
0.004, 10, 1.2e+03, 0.14
Solution time: 155 s. (2 minutes, 35 seconds)
Physical memory: 4.72 GB
Virtual memory: 25.97 GB
Ended at Jan 31, 2025, 7:50:23 PM.
----- Stationary Solver 1 in study1/Approach Curve Solver (sol1) -------------->
```

d=2175 (su67)

GENERAL

Description	Value
Solution	d=2175

LOG

```
<---- Compile Equations: Stationary in study1/Approach Curve Solver (sol1) -----
Started at Jan 31, 2025, 7:50:23 PM.
Parameter d = 2.175E-6 (m).
Geometry shape function: Linear serendipity
Number of vertex elements: 18
Number of boundary elements: 6154
Number of elements: 154892
Minimum element quality: 0.4857
Time: 8 s.
Physical memory: 3 GB
Virtual memory: 15.24 GB
Ended at Jan 31, 2025, 7:50:31 PM.
----- Compile Equations: Stationary in study1/Approach Curve Solver (sol1) ---->

<---- Dependent Variables 1 in study1/Approach Curve Solver (sol1) -------------
Started at Jan 31, 2025, 7:50:31 PM.
Solution time: 0 s.
Physical memory: 3 GB
Virtual memory: 15.24 GB
Ended at Jan 31, 2025, 7:50:31 PM.
----- Dependent Variables 1 in study1/Approach Curve Solver (sol1) ------------>

<---- Stationary Solver 1 in study1/Approach Curve Solver (sol1) ---------------
Started at Jan 31, 2025, 7:50:31 PM.
Segregated solver
Segregated Step 11 does not have any components solved for.
Number of degrees of freedom solved for: 786686 (plus 11731 internal DOFs).
Segregated solver iteration 1.
Merged variables
Nonsymmetric matrix found.
Scales for dependent variables:
Concentration (comp1.Cl): 1.4e+02
Concentration (comp1.K): 1.4e+02
Electric potential (comp1.V): 1.9e+02
Orthonormal null-space function used.
Iter      SolEst     Damping    Stepsize #Res #Jac #Sol   LinErr   LinRes
   1        0.83   0.3500000        0.83    2    1    1  5.5e-11  2.8e-15
   2        0.81   0.3500000        0.81    3    2    2  5.3e-11  2.8e-15
   3         0.8   0.3500000         0.8    4    3    3  3.7e-11  2.7e-15
Velocity u2, Pressure p
Nonsymmetric matrix found.
Scales for dependent variables:
Pressure (comp1.p): 1
Velocity field (spatial frame) (comp1.u2): 0.00018
Orthonormal null-space function used.
Iter      SolEst     Damping    Stepsize #Res #Jac #Sol   LinErr   LinRes
   1         1.1   0.5000000         1.1    2    1    1  1.6e-15  2.3e-15
Displacement field
Nonsymmetric matrix found.
Scales for dependent variables:
Auxiliary pressure (comp1.solid.lemm1.pw): 1
Displacement field (comp1.u): 2.8e-18
Orthonormal null-space function used.
Iter      SolEst     Damping    Stepsize #Res #Jac #Sol   LinErr   LinRes
   1         1.9   0.5000000         1.9    2    1    1  4.1e-14  1.5e-12
Spatial mesh displacement
```

```
Symmetric matrices found.
Scales for dependent variables:
Spatial mesh displacement (comp1.spatial.disp): 1.2e-09
Orthonormal null-space function used.
Iter      SolEst     Damping    Stepsize #Res #Jac #Sol   LinErr   LinRes
   1     7.1e-07   0.5000000     7.1e-07    2    1    1  1.6e-15  1.8e-15
Solution error estimates for segregated groups
1.4, 0.54, 0.95, 3.6e-07
Residual error estimates for segregated groups
7.8e+03, 8.2e+04, 2.2e+04, 0.14
Segregated solver iteration 2.
Merged variables
Scales for dependent variables:
Concentration (comp1.Cl): 1.4e+02
Concentration (comp1.K): 1.4e+02
Electric potential (comp1.V): 0.064
Iter      SolEst     Damping    Stepsize #Res #Jac #Sol   LinErr   LinRes
   1        0.78   0.3500000        0.78    6    4    4  8.2e-14  3.6e-15
   2        0.77   0.3500000        0.77    7    5    5  7.4e-14  3.4e-15
   3        0.76   0.3500000        0.76    8    6    6    6e-14  3.3e-15
Velocity u2, Pressure p
Scales for dependent variables:
Pressure (comp1.p): 3.6e+02
Velocity field (spatial frame) (comp1.u2): 0.00072
Iter      SolEst     Damping    Stepsize #Res #Jac #Sol   LinErr   LinRes
   1        0.36   0.5000000        0.36    4    2    2    3e-15  7.9e-15
Displacement field
Iter      SolEst     Damping    Stepsize #Res #Jac #Sol   LinErr   LinRes
   1        0.95   0.5000000        0.95    4    2    2  7.9e-14  1.5e-12
Spatial mesh displacement
Iter      SolEst     Damping    Stepsize #Res #Jac #Sol   LinErr   LinRes
   1     1.1e-06   0.5000000     1.1e-06    4    2    2  1.5e-15  1.8e-15
Solution error estimates for segregated groups
1.3, 0.18, 0.48, 5.4e-07
Residual error estimates for segregated groups
7.8e+02, 1.6e+04, 2.2e+04, 0.16
Segregated solver iteration 3.
Merged variables
Iter      SolEst     Damping    Stepsize #Res #Jac #Sol   LinErr   LinRes
   1        0.75   0.3500000        0.75   10    7    7  8.3e-14  3.1e-15
   2        0.73   0.3500000        0.73   11    8    8  1.1e-13  4.7e-15
   3        0.72   0.3500000        0.72   12    9    9  6.2e-14  3.3e-15
Velocity u2, Pressure p
Iter      SolEst     Damping    Stepsize #Res #Jac #Sol   LinErr   LinRes
   1        0.15   0.5000000        0.15    6    3    3    2e-15    8e-15
Displacement field
Iter      SolEst     Damping    Stepsize #Res #Jac #Sol   LinErr   LinRes
   1        0.52   0.5000000        0.52    6    3    3  6.5e-14  1.5e-12
Spatial mesh displacement
Iter      SolEst     Damping    Stepsize #Res #Jac #Sol   LinErr   LinRes
   1     1.1e-06   0.5000000     1.1e-06    6    3    3  2.1e-15  1.9e-15
Solution error estimates for segregated groups
1.2, 0.077, 0.26, 5.4e-07
Residual error estimates for segregated groups
2.2e+02, 9.2e+03, 1.6e+04, 0.16
Segregated solver iteration 4.
```

```
Merged variables
Iter      SolEst     Damping    Stepsize #Res #Jac #Sol   LinErr   LinRes
   1         0.7   0.3500000         0.7   14   10   10  4.4e-14  4.2e-15
   2        0.68   0.3500000        0.68   15   11   11  7.2e-14  3.3e-15
   3        0.59   0.3500000        0.59   16   12   12  1.1e-13  3.1e-15
Velocity u2, Pressure p
Iter      SolEst     Damping    Stepsize #Res #Jac #Sol   LinErr   LinRes
   1       0.072   0.5000000       0.072    8    4    4    2e-15  7.9e-15
Displacement field
Iter      SolEst     Damping    Stepsize #Res #Jac #Sol   LinErr   LinRes
   1        0.29   0.5000000        0.29    8    4    4  4.3e-14  1.5e-12
Spatial mesh displacement
Iter      SolEst     Damping    Stepsize #Res #Jac #Sol   LinErr   LinRes
   1     8.9e-07   0.5000000     8.9e-07    8    4    4  1.4e-15  1.8e-15
Solution error estimates for segregated groups
1, 0.036, 0.15, 4.5e-07
Residual error estimates for segregated groups
83, 4.9e+03, 1.1e+04, 0.15
Segregated solver iteration 5.
Merged variables
Iter      SolEst     Damping    Stepsize #Res #Jac #Sol   LinErr   LinRes
   1        0.41   0.3500000        0.41   18   13   13  1.2e-13  3.2e-15
   2        0.27   0.3500000        0.27   19   14   14  9.8e-14  3.2e-15
   3        0.18   0.3500000        0.18   20   15   15  9.7e-14  5.4e-15
Velocity u2, Pressure p
Iter      SolEst     Damping    Stepsize #Res #Jac #Sol   LinErr   LinRes
   1       0.035   0.5000000       0.035   10    5    5    3e-15  7.7e-15
Displacement field
Iter      SolEst     Damping    Stepsize #Res #Jac #Sol   LinErr   LinRes
   1        0.17   0.5000000        0.17   10    5    5  4.1e-14  1.5e-12
Spatial mesh displacement
Iter      SolEst     Damping    Stepsize #Res #Jac #Sol   LinErr   LinRes
   1     6.7e-07   0.5000000     6.7e-07   10    5    5  1.5e-15  1.8e-15
Solution error estimates for segregated groups
0.31, 0.017, 0.084, 3.3e-07
Residual error estimates for segregated groups
32, 2.5e+03, 6.8e+03, 0.15
Segregated solver iteration 6.
Merged variables
Iter      SolEst     Damping    Stepsize #Res #Jac #Sol   LinErr   LinRes
   1        0.12   0.3500000        0.12   22   16   16    1e-13  3.4e-15
   2       0.077   0.3500000       0.077   23   17   17    8e-14  3.7e-15
   3        0.05   0.3500000        0.05   24   18   18  7.1e-14    3e-15
Velocity u2, Pressure p
Iter      SolEst     Damping    Stepsize #Res #Jac #Sol   LinErr   LinRes
   1       0.017   0.5000000       0.017   12    6    6  3.7e-15    8e-15
Displacement field
Iter      SolEst     Damping    Stepsize #Res #Jac #Sol   LinErr   LinRes
   1       0.095   0.5000000       0.095   12    6    6  6.7e-14  1.5e-12
Spatial mesh displacement
Iter      SolEst     Damping    Stepsize #Res #Jac #Sol   LinErr   LinRes
   1     4.7e-07   0.5000000     4.7e-07   12    6    6  1.3e-15  1.8e-15
Solution error estimates for segregated groups
0.087, 0.0085, 0.048, 2.3e-07
Residual error estimates for segregated groups
12, 1.3e+03, 4.2e+03, 0.14
```

```
Segregated solver iteration 7.
Merged variables
Iter      SolEst     Damping    Stepsize #Res #Jac #Sol   LinErr   LinRes
   1       0.033   0.3500000       0.033   26   19   19    1e-13  3.1e-15
   2       0.021   0.3500000       0.021   27   20   20    1e-13    3e-15
   3       0.014   0.3500000       0.014   28   21   21  7.5e-14  3.7e-15
Velocity u2, Pressure p
Iter      SolEst     Damping    Stepsize #Res #Jac #Sol   LinErr   LinRes
   1      0.0085   0.5000000      0.0085   14    7    7  4.4e-15    8e-15
Displacement field
Scales for dependent variables:
Auxiliary pressure (comp1.solid.lemm1.pw): 0.35
Displacement field (comp1.u): 2.8e-16
Iter      SolEst     Damping    Stepsize #Res #Jac #Sol   LinErr   LinRes
   1       0.054   0.5000000       0.054   14    7    7  1.1e-13  1.2e-12
Spatial mesh displacement
Iter      SolEst     Damping    Stepsize #Res #Jac #Sol   LinErr   LinRes
   1     3.1e-07   0.5000000     3.1e-07   14    7    7  1.5e-15  1.9e-15
Solution error estimates for segregated groups
0.024, 0.0042, 0.027, 1.6e-07
Residual error estimates for segregated groups
4.1, 6.4e+02, 2.6e+03, 0.14
Segregated solver iteration 8.
Merged variables
Iter      SolEst     Damping    Stepsize #Res #Jac #Sol   LinErr   LinRes
   1      0.0091   0.3500000      0.0091   30   22   22  6.7e-14  2.9e-15
   2      0.0059   0.3500000      0.0059   31   23   23  5.1e-14  3.7e-15
   3      0.0038   0.3500000      0.0038   32   24   24  7.8e-14  3.6e-15
Velocity u2, Pressure p
Iter      SolEst     Damping    Stepsize #Res #Jac #Sol   LinErr   LinRes
   1      0.0042   0.5000000      0.0042   16    8    8  2.4e-15  7.9e-15
Displacement field
Iter      SolEst     Damping    Stepsize #Res #Jac #Sol   LinErr   LinRes
   1        0.03   0.5000000        0.03   16    8    8  1.5e-13  1.2e-12
Spatial mesh displacement
Iter      SolEst     Damping    Stepsize #Res #Jac #Sol   LinErr   LinRes
   1       2e-07   0.5000000       2e-07   16    8    8  1.4e-15  1.9e-15
Solution error estimates for segregated groups
0.0066, 0.0021, 0.015, 1e-07
Residual error estimates for segregated groups
1.4, 3.2e+02, 1.8e+03, 0.15
Segregated solver iteration 9.
Merged variables
Iter      SolEst     Damping    Stepsize #Res #Jac #Sol   LinErr   LinRes
   1      0.0025   0.3500000      0.0025   34   25   25    1e-13  3.5e-15
   2      0.0016   0.3500000      0.0016   35   26   26  6.6e-14  3.2e-15
   3      0.0011   0.3500000      0.0011   36   27   27  4.3e-14    3e-15
Velocity u2, Pressure p
Iter      SolEst     Damping    Stepsize #Res #Jac #Sol   LinErr   LinRes
   1      0.0021   0.5000000      0.0021   18    9    9  2.9e-15  7.9e-15
Displacement field
Iter      SolEst     Damping    Stepsize #Res #Jac #Sol   LinErr   LinRes
   1       0.017   0.5000000       0.017   18    9    9  1.9e-13  1.2e-12
Spatial mesh displacement
Iter      SolEst     Damping    Stepsize #Res #Jac #Sol   LinErr   LinRes
   1     1.3e-07   0.5000000     1.3e-07   18    9    9  1.6e-15  1.8e-15
```

```
Solution error estimates for segregated groups
0.0018, 0.0011, 0.0085, 6.3e-08
Residual error estimates for segregated groups
0.44, 1.6e+02, 1.4e+03, 0.14
Segregated solver iteration 10.
Merged variables
Iter      SolEst     Damping    Stepsize #Res #Jac #Sol   LinErr   LinRes
   1     0.00069   0.3500000     0.00069   38   28   28  4.5e-14  2.9e-15
   2     0.00045   0.3500000     0.00045   39   29   29  9.9e-14  4.7e-15
   3     0.00029   0.3500000     0.00029   40   30   30  8.9e-14  3.1e-15
Velocity u2, Pressure p
Iter      SolEst     Damping    Stepsize #Res #Jac #Sol   LinErr   LinRes
   1      0.0011   0.5000000      0.0011   20   10   10    2e-15    8e-15
Displacement field
Iter      SolEst     Damping    Stepsize #Res #Jac #Sol   LinErr   LinRes
   1      0.0094   0.5000000      0.0094   20   10   10  1.6e-13  1.2e-12
Spatial mesh displacement
Iter      SolEst     Damping    Stepsize #Res #Jac #Sol   LinErr   LinRes
   1     7.7e-08   0.5000000     7.7e-08   20   10   10  1.9e-15  1.8e-15
Solution error estimates for segregated groups
0.0005, 0.00053, 0.0047, 3.8e-08
Residual error estimates for segregated groups
0.14, 81, 1.2e+03, 0.14
Segregated solver iteration 11.
Merged variables
Iter      SolEst     Damping    Stepsize #Res #Jac #Sol   LinErr   LinRes
   1     0.00019   0.3500000     0.00019   42   31   31  7.3e-14  5.2e-15
   2     0.00012   0.3500000     0.00012   43   32   32  4.8e-14  3.4e-15
   3     7.9e-05   0.3500000     7.9e-05   44   33   33  5.9e-14  2.9e-15
Velocity u2, Pressure p
Iter      SolEst     Damping    Stepsize #Res #Jac #Sol   LinErr   LinRes
   1     0.00053   0.5000000     0.00053   22   11   11  2.2e-15  7.8e-15
Displacement field
Iter      SolEst     Damping    Stepsize #Res #Jac #Sol   LinErr   LinRes
   1      0.0051   0.5000000      0.0051   22   11   11  1.1e-13  1.2e-12
Spatial mesh displacement
Iter      SolEst     Damping    Stepsize #Res #Jac #Sol   LinErr   LinRes
   1     4.6e-08   0.5000000     4.6e-08   22   11   11  1.2e-15  1.9e-15
Solution error estimates for segregated groups
0.00014, 0.00026, 0.0026, 2.3e-08
Residual error estimates for segregated groups
0.043, 40, 1.2e+03, 0.15
Segregated solver iteration 12.
Merged variables
Iter      SolEst     Damping    Stepsize #Res #Jac #Sol   LinErr   LinRes
   1     5.2e-05   0.3500000     5.2e-05   46   34   34  5.4e-14  3.6e-15
   2     3.4e-05   0.3500000     3.4e-05   47   35   35  1.2e-13  3.7e-15
   3     2.2e-05   0.3500000     2.2e-05   48   36   36  9.1e-14  2.9e-15
Velocity u2, Pressure p
Iter      SolEst     Damping    Stepsize #Res #Jac #Sol   LinErr   LinRes
   1     0.00026   0.5000000     0.00026   24   12   12  3.3e-15  7.9e-15
Displacement field
Iter      SolEst     Damping    Stepsize #Res #Jac #Sol   LinErr   LinRes
   1      0.0028   0.5000000      0.0028   24   12   12  1.1e-13  1.2e-12
Spatial mesh displacement
Iter      SolEst     Damping    Stepsize #Res #Jac #Sol   LinErr   LinRes
```

```
   1     2.7e-08   0.5000000     2.7e-08   24   12   12  1.4e-15  1.8e-15
Solution error estimates for segregated groups
3.7e-05, 0.00013, 0.0014, 1.4e-08
Residual error estimates for segregated groups
0.013, 20, 1.2e+03, 0.14
Segregated solver iteration 13.
Merged variables
Iter      SolEst     Damping    Stepsize #Res #Jac #Sol   LinErr   LinRes
   1     1.4e-05   0.3500000     1.4e-05   50   37   37  7.7e-14  3.3e-15
   2     9.2e-06   0.3500000     9.2e-06   51   38   38  6.8e-14  3.3e-15
   3       6e-06   0.3500000       6e-06   52   39   39  1.1e-13  3.3e-15
Velocity u2, Pressure p
Iter      SolEst     Damping    Stepsize #Res #Jac #Sol   LinErr   LinRes
   1     0.00013   0.5000000     0.00013   26   13   13  2.4e-15    8e-15
Displacement field
Iter      SolEst     Damping    Stepsize #Res #Jac #Sol   LinErr   LinRes
   1      0.0015   0.5000000      0.0015   26   13   13  1.1e-13  1.2e-12
Spatial mesh displacement
Iter      SolEst     Damping    Stepsize #Res #Jac #Sol   LinErr   LinRes
   1     1.6e-08   0.5000000     1.6e-08   26   13   13  1.4e-15  1.8e-15
Solution error estimates for segregated groups
1e-05, 6.6e-05, 0.00076, 7.9e-09
Residual error estimates for segregated groups
0.004, 10, 1.2e+03, 0.15
Solution time: 157 s. (2 minutes, 37 seconds)
Physical memory: 4.66 GB
Virtual memory: 25.51 GB
Ended at Jan 31, 2025, 7:53:08 PM.
----- Stationary Solver 1 in study1/Approach Curve Solver (sol1) -------------->
```

d=2170 (su68)

GENERAL

Description	Value
Solution	d=2170

LOG

```
<---- Compile Equations: Stationary in study1/Approach Curve Solver (sol1) -----
Started at Jan 31, 2025, 7:53:08 PM.
Parameter d = 2.17E-6 (m).
Geometry shape function: Linear serendipity
Number of vertex elements: 18
Number of boundary elements: 6151
Number of elements: 154657
Minimum element quality: 0.4704
Time: 8 s.
Physical memory: 2.95 GB
Virtual memory: 15.25 GB
Ended at Jan 31, 2025, 7:53:17 PM.
----- Compile Equations: Stationary in study1/Approach Curve Solver (sol1) ---->

<---- Dependent Variables 1 in study1/Approach Curve Solver (sol1) -------------
Started at Jan 31, 2025, 7:53:17 PM.
Solution time: 0 s.
Physical memory: 2.95 GB
Virtual memory: 15.18 GB
Ended at Jan 31, 2025, 7:53:17 PM.
----- Dependent Variables 1 in study1/Approach Curve Solver (sol1) ------------>

<---- Stationary Solver 1 in study1/Approach Curve Solver (sol1) ---------------
Started at Jan 31, 2025, 7:53:17 PM.
Segregated solver
Segregated Step 11 does not have any components solved for.
Number of degrees of freedom solved for: 785268 (plus 11725 internal DOFs).
Segregated solver iteration 1.
Merged variables
Nonsymmetric matrix found.
Scales for dependent variables:
Concentration (comp1.Cl): 1.4e+02
Concentration (comp1.K): 1.4e+02
Electric potential (comp1.V): 1.9e+02
Orthonormal null-space function used.
Iter      SolEst     Damping    Stepsize #Res #Jac #Sol   LinErr   LinRes
   1        0.83   0.3500000        0.83    2    1    1  1.9e-11  2.7e-15
   2        0.81   0.3500000        0.81    3    2    2  5.5e-11  2.7e-15
   3         0.8   0.3500000         0.8    4    3    3    4e-11  2.7e-15
Velocity u2, Pressure p
Nonsymmetric matrix found.
Scales for dependent variables:
Pressure (comp1.p): 1
Velocity field (spatial frame) (comp1.u2): 0.00018
Orthonormal null-space function used.
Iter      SolEst     Damping    Stepsize #Res #Jac #Sol   LinErr   LinRes
   1         1.1   0.5000000         1.1    2    1    1  1.7e-15  2.3e-15
Displacement field
Nonsymmetric matrix found.
Scales for dependent variables:
Auxiliary pressure (comp1.solid.lemm1.pw): 1
Displacement field (comp1.u): 2.7e-18
Orthonormal null-space function used.
Iter      SolEst     Damping    Stepsize #Res #Jac #Sol   LinErr   LinRes
   1         1.9   0.5000000         1.9    2    1    1  6.4e-14  1.5e-12
Spatial mesh displacement
```

```
Symmetric matrices found.
Scales for dependent variables:
Spatial mesh displacement (comp1.spatial.disp): 1.2e-09
Orthonormal null-space function used.
Iter      SolEst     Damping    Stepsize #Res #Jac #Sol   LinErr   LinRes
   1     7.1e-07   0.5000000     7.1e-07    2    1    1  1.7e-15  1.9e-15
Solution error estimates for segregated groups
1.4, 0.54, 0.95, 3.6e-07
Residual error estimates for segregated groups
7.8e+03, 8.2e+04, 2.2e+04, 0.13
Segregated solver iteration 2.
Merged variables
Scales for dependent variables:
Concentration (comp1.Cl): 1.4e+02
Concentration (comp1.K): 1.4e+02
Electric potential (comp1.V): 0.064
Iter      SolEst     Damping    Stepsize #Res #Jac #Sol   LinErr   LinRes
   1        0.78   0.3500000        0.78    6    4    4  6.6e-14  4.9e-15
   2        0.77   0.3500000        0.77    7    5    5  4.8e-14  6.1e-15
   3        0.76   0.3500000        0.76    8    6    6  8.3e-14  7.1e-15
Velocity u2, Pressure p
Scales for dependent variables:
Pressure (comp1.p): 3.6e+02
Velocity field (spatial frame) (comp1.u2): 0.00073
Iter      SolEst     Damping    Stepsize #Res #Jac #Sol   LinErr   LinRes
   1        0.36   0.5000000        0.36    4    2    2  2.6e-15  7.7e-15
Displacement field
Iter      SolEst     Damping    Stepsize #Res #Jac #Sol   LinErr   LinRes
   1        0.95   0.5000000        0.95    4    2    2  9.2e-14  1.5e-12
Spatial mesh displacement
Iter      SolEst     Damping    Stepsize #Res #Jac #Sol   LinErr   LinRes
   1     1.1e-06   0.5000000     1.1e-06    4    2    2  1.7e-15  1.9e-15
Solution error estimates for segregated groups
1.3, 0.18, 0.48, 5.4e-07
Residual error estimates for segregated groups
7.8e+02, 1.3e+04, 2.2e+04, 0.15
Segregated solver iteration 3.
Merged variables
Iter      SolEst     Damping    Stepsize #Res #Jac #Sol   LinErr   LinRes
   1        0.74   0.3500000        0.74   10    7    7  1.6e-13  6.7e-15
   2        0.73   0.3500000        0.73   11    8    8  9.4e-14  5.4e-15
   3        0.72   0.3500000        0.72   12    9    9  9.3e-14  1.2e-14
Velocity u2, Pressure p
Iter      SolEst     Damping    Stepsize #Res #Jac #Sol   LinErr   LinRes
   1        0.15   0.5000000        0.15    6    3    3  1.9e-15  7.9e-15
Displacement field
Iter      SolEst     Damping    Stepsize #Res #Jac #Sol   LinErr   LinRes
   1        0.52   0.5000000        0.52    6    3    3  5.3e-14  1.5e-12
Spatial mesh displacement
Iter      SolEst     Damping    Stepsize #Res #Jac #Sol   LinErr   LinRes
   1     1.1e-06   0.5000000     1.1e-06    6    3    3  1.4e-15  1.8e-15
Solution error estimates for segregated groups
1.2, 0.077, 0.26, 5.4e-07
Residual error estimates for segregated groups
2.2e+02, 7.5e+03, 1.6e+04, 0.14
Segregated solver iteration 4.
```

```
Merged variables
Iter      SolEst     Damping    Stepsize #Res #Jac #Sol   LinErr   LinRes
   1         0.7   0.3500000         0.7   14   10   10  6.9e-14  6.4e-15
   2        0.68   0.3500000        0.68   15   11   11  5.5e-14    4e-15
   3        0.59   0.3500000        0.59   16   12   12  1.2e-13  6.2e-15
Velocity u2, Pressure p
Iter      SolEst     Damping    Stepsize #Res #Jac #Sol   LinErr   LinRes
   1       0.072   0.5000000       0.072    8    4    4  3.1e-15  7.8e-15
Displacement field
Iter      SolEst     Damping    Stepsize #Res #Jac #Sol   LinErr   LinRes
   1        0.29   0.5000000        0.29    8    4    4  1.4e-13  1.5e-12
Spatial mesh displacement
Iter      SolEst     Damping    Stepsize #Res #Jac #Sol   LinErr   LinRes
   1     8.9e-07   0.5000000     8.9e-07    8    4    4  1.7e-15  1.8e-15
Solution error estimates for segregated groups
1, 0.036, 0.15, 4.5e-07
Residual error estimates for segregated groups
83, 3.9e+03, 1.1e+04, 0.14
Segregated solver iteration 5.
Merged variables
Iter      SolEst     Damping    Stepsize #Res #Jac #Sol   LinErr   LinRes
   1        0.41   0.3500000        0.41   18   13   13  8.9e-14  4.5e-15
   2        0.27   0.3500000        0.27   19   14   14  1.3e-13  4.3e-15
   3        0.18   0.3500000        0.18   20   15   15  8.9e-14  4.7e-15
Velocity u2, Pressure p
Iter      SolEst     Damping    Stepsize #Res #Jac #Sol   LinErr   LinRes
   1       0.035   0.5000000       0.035   10    5    5  4.2e-15  7.9e-15
Displacement field
Iter      SolEst     Damping    Stepsize #Res #Jac #Sol   LinErr   LinRes
   1        0.17   0.5000000        0.17   10    5    5  7.3e-14  1.5e-12
Spatial mesh displacement
Iter      SolEst     Damping    Stepsize #Res #Jac #Sol   LinErr   LinRes
   1     6.7e-07   0.5000000     6.7e-07   10    5    5  2.2e-15  1.8e-15
Solution error estimates for segregated groups
0.31, 0.017, 0.084, 3.4e-07
Residual error estimates for segregated groups
32, 2e+03, 6.8e+03, 0.15
Segregated solver iteration 6.
Merged variables
Iter      SolEst     Damping    Stepsize #Res #Jac #Sol   LinErr   LinRes
   1        0.12   0.3500000        0.12   22   16   16  9.1e-14  6.5e-15
   2       0.077   0.3500000       0.077   23   17   17  1.5e-13  1.3e-14
   3        0.05   0.3500000        0.05   24   18   18  8.7e-14  2.2e-14
Velocity u2, Pressure p
Iter      SolEst     Damping    Stepsize #Res #Jac #Sol   LinErr   LinRes
   1       0.017   0.5000000       0.017   12    6    6  4.5e-15  7.9e-15
Displacement field
Iter      SolEst     Damping    Stepsize #Res #Jac #Sol   LinErr   LinRes
   1       0.095   0.5000000       0.095   12    6    6  6.1e-14  1.5e-12
Spatial mesh displacement
Iter      SolEst     Damping    Stepsize #Res #Jac #Sol   LinErr   LinRes
   1     4.7e-07   0.5000000     4.7e-07   12    6    6  1.5e-15  1.8e-15
Solution error estimates for segregated groups
0.086, 0.0085, 0.048, 2.3e-07
Residual error estimates for segregated groups
12, 1e+03, 4.2e+03, 0.15
```

```
Segregated solver iteration 7.
Merged variables
Iter      SolEst     Damping    Stepsize #Res #Jac #Sol   LinErr   LinRes
   1       0.033   0.3500000       0.033   26   19   19  5.1e-14  8.7e-15
   2       0.021   0.3500000       0.021   27   20   20  1.2e-13  8.8e-15
   3       0.014   0.3500000       0.014   28   21   21  4.2e-14  5.7e-15
Velocity u2, Pressure p
Iter      SolEst     Damping    Stepsize #Res #Jac #Sol   LinErr   LinRes
   1      0.0085   0.5000000      0.0085   14    7    7  3.1e-15    8e-15
Displacement field
Scales for dependent variables:
Auxiliary pressure (comp1.solid.lemm1.pw): 0.35
Displacement field (comp1.u): 2.8e-16
Iter      SolEst     Damping    Stepsize #Res #Jac #Sol   LinErr   LinRes
   1       0.054   0.5000000       0.054   14    7    7  1.7e-13  1.2e-12
Spatial mesh displacement
Iter      SolEst     Damping    Stepsize #Res #Jac #Sol   LinErr   LinRes
   1     3.1e-07   0.5000000     3.1e-07   14    7    7  1.8e-15  1.8e-15
Solution error estimates for segregated groups
0.024, 0.0042, 0.027, 1.6e-07
Residual error estimates for segregated groups
4.1, 5.1e+02, 2.6e+03, 0.15
Segregated solver iteration 8.
Merged variables
Iter      SolEst     Damping    Stepsize #Res #Jac #Sol   LinErr   LinRes
   1       0.009   0.3500000       0.009   30   22   22  1.4e-13  5.3e-15
   2      0.0059   0.3500000      0.0059   31   23   23    2e-13    9e-15
   3      0.0038   0.3500000      0.0038   32   24   24  5.6e-14  5.5e-15
Velocity u2, Pressure p
Iter      SolEst     Damping    Stepsize #Res #Jac #Sol   LinErr   LinRes
   1      0.0042   0.5000000      0.0042   16    8    8  2.3e-15  7.9e-15
Displacement field
Iter      SolEst     Damping    Stepsize #Res #Jac #Sol   LinErr   LinRes
   1        0.03   0.5000000        0.03   16    8    8  1.7e-13  1.2e-12
Spatial mesh displacement
Iter      SolEst     Damping    Stepsize #Res #Jac #Sol   LinErr   LinRes
   1       2e-07   0.5000000       2e-07   16    8    8  1.4e-15  1.8e-15
Solution error estimates for segregated groups
0.0066, 0.0021, 0.015, 1e-07
Residual error estimates for segregated groups
1.4, 2.6e+02, 1.8e+03, 0.15
Segregated solver iteration 9.
Merged variables
Iter      SolEst     Damping    Stepsize #Res #Jac #Sol   LinErr   LinRes
   1      0.0025   0.3500000      0.0025   34   25   25  6.8e-14  4.3e-15
   2      0.0016   0.3500000      0.0016   35   26   26  8.6e-14    4e-15
   3       0.001   0.3500000       0.001   36   27   27  8.5e-14  3.7e-15
Velocity u2, Pressure p
Iter      SolEst     Damping    Stepsize #Res #Jac #Sol   LinErr   LinRes
   1      0.0021   0.5000000      0.0021   18    9    9  4.9e-15  7.8e-15
Displacement field
Iter      SolEst     Damping    Stepsize #Res #Jac #Sol   LinErr   LinRes
   1       0.017   0.5000000       0.017   18    9    9  1.5e-13  1.2e-12
Spatial mesh displacement
Iter      SolEst     Damping    Stepsize #Res #Jac #Sol   LinErr   LinRes
   1     1.3e-07   0.5000000     1.3e-07   18    9    9  1.9e-15  1.8e-15
```

```
Solution error estimates for segregated groups
0.0018, 0.0011, 0.0085, 6.3e-08
Residual error estimates for segregated groups
0.44, 1.3e+02, 1.4e+03, 0.15
Segregated solver iteration 10.
Merged variables
Iter      SolEst     Damping    Stepsize #Res #Jac #Sol   LinErr   LinRes
   1     0.00068   0.3500000     0.00068   38   28   28  9.9e-14  6.4e-15
   2     0.00044   0.3500000     0.00044   39   29   29  8.4e-14  6.7e-15
   3     0.00029   0.3500000     0.00029   40   30   30  8.1e-14  4.1e-15
Velocity u2, Pressure p
Iter      SolEst     Damping    Stepsize #Res #Jac #Sol   LinErr   LinRes
   1      0.0011   0.5000000      0.0011   20   10   10  2.7e-15  7.9e-15
Displacement field
Iter      SolEst     Damping    Stepsize #Res #Jac #Sol   LinErr   LinRes
   1      0.0094   0.5000000      0.0094   20   10   10  1.3e-13  1.2e-12
Spatial mesh displacement
Iter      SolEst     Damping    Stepsize #Res #Jac #Sol   LinErr   LinRes
   1     7.7e-08   0.5000000     7.7e-08   20   10   10  1.5e-15  1.8e-15
Solution error estimates for segregated groups
0.00049, 0.00053, 0.0047, 3.8e-08
Residual error estimates for segregated groups
0.14, 65, 1.2e+03, 0.15
Segregated solver iteration 11.
Merged variables
Iter      SolEst     Damping    Stepsize #Res #Jac #Sol   LinErr   LinRes
   1     0.00019   0.3500000     0.00019   42   31   31  1.1e-13  8.2e-15
   2     0.00012   0.3500000     0.00012   43   32   32  1.1e-13  3.9e-15
   3     7.9e-05   0.3500000     7.9e-05   44   33   33  2.3e-13  7.9e-15
Velocity u2, Pressure p
Iter      SolEst     Damping    Stepsize #Res #Jac #Sol   LinErr   LinRes
   1     0.00053   0.5000000     0.00053   22   11   11    3e-15    8e-15
Displacement field
Iter      SolEst     Damping    Stepsize #Res #Jac #Sol   LinErr   LinRes
   1      0.0051   0.5000000      0.0051   22   11   11  1.3e-13  1.2e-12
Spatial mesh displacement
Iter      SolEst     Damping    Stepsize #Res #Jac #Sol   LinErr   LinRes
   1     4.6e-08   0.5000000     4.6e-08   22   11   11  1.4e-15  1.8e-15
Solution error estimates for segregated groups
0.00014, 0.00026, 0.0026, 2.3e-08
Residual error estimates for segregated groups
0.043, 32, 1.2e+03, 0.14
Segregated solver iteration 12.
Merged variables
Iter      SolEst     Damping    Stepsize #Res #Jac #Sol   LinErr   LinRes
   1     5.1e-05   0.3500000     5.1e-05   46   34   34  1.4e-13  8.4e-15
   2     3.3e-05   0.3500000     3.3e-05   47   35   35  6.7e-14  4.8e-15
   3     2.2e-05   0.3500000     2.2e-05   48   36   36  1.3e-13  5.2e-15
Velocity u2, Pressure p
Iter      SolEst     Damping    Stepsize #Res #Jac #Sol   LinErr   LinRes
   1     0.00026   0.5000000     0.00026   24   12   12  3.1e-15    8e-15
Displacement field
Iter      SolEst     Damping    Stepsize #Res #Jac #Sol   LinErr   LinRes
   1      0.0028   0.5000000      0.0028   24   12   12    1e-13  1.2e-12
Spatial mesh displacement
Iter      SolEst     Damping    Stepsize #Res #Jac #Sol   LinErr   LinRes
```

```
   1     2.7e-08   0.5000000     2.7e-08   24   12   12  1.2e-15  1.8e-15
Solution error estimates for segregated groups
3.7e-05, 0.00013, 0.0014, 1.4e-08
Residual error estimates for segregated groups
0.013, 16, 1.2e+03, 0.16
Segregated solver iteration 13.
Merged variables
Iter      SolEst     Damping    Stepsize #Res #Jac #Sol   LinErr   LinRes
   1     1.4e-05   0.3500000     1.4e-05   50   37   37  1.3e-13  1.4e-14
   2     9.2e-06   0.3500000     9.2e-06   51   38   38  6.8e-14  3.3e-15
   3       6e-06   0.3500000       6e-06   52   39   39  1.2e-13  9.6e-15
Velocity u2, Pressure p
Iter      SolEst     Damping    Stepsize #Res #Jac #Sol   LinErr   LinRes
   1     0.00013   0.5000000     0.00013   26   13   13  2.2e-15  7.8e-15
Displacement field
Iter      SolEst     Damping    Stepsize #Res #Jac #Sol   LinErr   LinRes
   1      0.0015   0.5000000      0.0015   26   13   13  9.4e-14  1.2e-12
Spatial mesh displacement
Iter      SolEst     Damping    Stepsize #Res #Jac #Sol   LinErr   LinRes
   1     1.6e-08   0.5000000     1.6e-08   26   13   13  1.9e-15  1.8e-15
Solution error estimates for segregated groups
1e-05, 6.6e-05, 0.00076, 7.9e-09
Residual error estimates for segregated groups
0.004, 8.1, 1.2e+03, 0.16
Solution time: 155 s. (2 minutes, 35 seconds)
Physical memory: 4.63 GB
Virtual memory: 25.86 GB
Ended at Jan 31, 2025, 7:55:51 PM.
----- Stationary Solver 1 in study1/Approach Curve Solver (sol1) -------------->
```

d=2165 (su69)

GENERAL

Description	Value
Solution	d=2165

LOG

```
<---- Compile Equations: Stationary in study1/Approach Curve Solver (sol1) -----
Started at Jan 31, 2025, 7:55:52 PM.
Parameter d = 2.165E-6 (m).
Geometry shape function: Linear serendipity
Number of vertex elements: 18
Number of boundary elements: 6149
Number of elements: 154997
Minimum element quality: 0.4897
Time: 8 s.
Physical memory: 2.95 GB
Virtual memory: 15.18 GB
Ended at Jan 31, 2025, 7:56:00 PM.
----- Compile Equations: Stationary in study1/Approach Curve Solver (sol1) ---->

<---- Dependent Variables 1 in study1/Approach Curve Solver (sol1) -------------
Started at Jan 31, 2025, 7:56:00 PM.
Solution time: 0 s.
Physical memory: 2.95 GB
Virtual memory: 15.18 GB
Ended at Jan 31, 2025, 7:56:00 PM.
----- Dependent Variables 1 in study1/Approach Curve Solver (sol1) ------------>

<---- Stationary Solver 1 in study1/Approach Curve Solver (sol1) ---------------
Started at Jan 31, 2025, 7:56:00 PM.
Segregated solver
Segregated Step 11 does not have any components solved for.
Number of degrees of freedom solved for: 787039 (plus 11721 internal DOFs).
Segregated solver iteration 1.
Merged variables
Nonsymmetric matrix found.
Scales for dependent variables:
Concentration (comp1.Cl): 1.4e+02
Concentration (comp1.K): 1.4e+02
Electric potential (comp1.V): 1.9e+02
Orthonormal null-space function used.
Iter      SolEst     Damping    Stepsize #Res #Jac #Sol   LinErr   LinRes
   1        0.83   0.3500000        0.83    2    1    1  6.8e-11  2.7e-15
   2        0.81   0.3500000        0.81    3    2    2  3.7e-11  2.7e-15
   3         0.8   0.3500000         0.8    4    3    3  5.9e-11  2.7e-15
Velocity u2, Pressure p
Nonsymmetric matrix found.
Scales for dependent variables:
Pressure (comp1.p): 1
Velocity field (spatial frame) (comp1.u2): 0.00018
Orthonormal null-space function used.
Iter      SolEst     Damping    Stepsize #Res #Jac #Sol   LinErr   LinRes
   1         1.1   0.5000000         1.1    2    1    1  2.8e-15  2.2e-15
Displacement field
Nonsymmetric matrix found.
Scales for dependent variables:
Auxiliary pressure (comp1.solid.lemm1.pw): 1
Displacement field (comp1.u): 2.8e-18
Orthonormal null-space function used.
Iter      SolEst     Damping    Stepsize #Res #Jac #Sol   LinErr   LinRes
   1         1.9   0.5000000         1.9    2    1    1    4e-14  1.5e-12
Spatial mesh displacement
```

```
Symmetric matrices found.
Scales for dependent variables:
Spatial mesh displacement (comp1.spatial.disp): 1.2e-09
Orthonormal null-space function used.
Iter      SolEst     Damping    Stepsize #Res #Jac #Sol   LinErr   LinRes
   1     7.2e-07   0.5000000     7.2e-07    2    1    1  1.4e-15  1.9e-15
Solution error estimates for segregated groups
1.4, 0.54, 0.95, 3.6e-07
Residual error estimates for segregated groups
7.8e+03, 8.2e+04, 2.2e+04, 0.14
Segregated solver iteration 2.
Merged variables
Scales for dependent variables:
Concentration (comp1.Cl): 1.4e+02
Concentration (comp1.K): 1.4e+02
Electric potential (comp1.V): 0.064
Iter      SolEst     Damping    Stepsize #Res #Jac #Sol   LinErr   LinRes
   1        0.78   0.3500000        0.78    6    4    4  1.4e-13  2.8e-15
   2        0.77   0.3500000        0.77    7    5    5  9.5e-14  2.7e-15
   3        0.76   0.3500000        0.76    8    6    6  1.4e-13  2.7e-15
Velocity u2, Pressure p
Scales for dependent variables:
Pressure (comp1.p): 3.6e+02
Velocity field (spatial frame) (comp1.u2): 0.00072
Iter      SolEst     Damping    Stepsize #Res #Jac #Sol   LinErr   LinRes
   1        0.36   0.5000000        0.36    4    2    2  4.8e-15  7.8e-15
Displacement field
Iter      SolEst     Damping    Stepsize #Res #Jac #Sol   LinErr   LinRes
   1        0.95   0.5000000        0.95    4    2    2  1.4e-13  1.5e-12
Spatial mesh displacement
Iter      SolEst     Damping    Stepsize #Res #Jac #Sol   LinErr   LinRes
   1     1.1e-06   0.5000000     1.1e-06    4    2    2  1.4e-15  1.9e-15
Solution error estimates for segregated groups
1.3, 0.18, 0.48, 5.4e-07
Residual error estimates for segregated groups
7.8e+02, 1.7e+04, 2.2e+04, 0.14
Segregated solver iteration 3.
Merged variables
Iter      SolEst     Damping    Stepsize #Res #Jac #Sol   LinErr   LinRes
   1        0.75   0.3500000        0.75   10    7    7  5.3e-14  2.7e-15
   2        0.73   0.3500000        0.73   11    8    8    1e-13  2.6e-15
   3        0.72   0.3500000        0.72   12    9    9  5.7e-14  2.7e-15
Velocity u2, Pressure p
Iter      SolEst     Damping    Stepsize #Res #Jac #Sol   LinErr   LinRes
   1        0.15   0.5000000        0.15    6    3    3  3.6e-15  7.9e-15
Displacement field
Iter      SolEst     Damping    Stepsize #Res #Jac #Sol   LinErr   LinRes
   1        0.52   0.5000000        0.52    6    3    3  4.2e-14  1.5e-12
Spatial mesh displacement
Iter      SolEst     Damping    Stepsize #Res #Jac #Sol   LinErr   LinRes
   1     1.1e-06   0.5000000     1.1e-06    6    3    3  1.5e-15  1.8e-15
Solution error estimates for segregated groups
1.2, 0.077, 0.26, 5.4e-07
Residual error estimates for segregated groups
2.2e+02, 9.5e+03, 1.6e+04, 0.15
Segregated solver iteration 4.
```

```
Merged variables
Iter      SolEst     Damping    Stepsize #Res #Jac #Sol   LinErr   LinRes
   1         0.7   0.3500000         0.7   14   10   10  1.1e-13  2.9e-15
   2        0.68   0.3500000        0.68   15   11   11  4.1e-14  2.7e-15
   3        0.59   0.3500000        0.59   16   12   12  8.5e-14  2.7e-15
Velocity u2, Pressure p
Iter      SolEst     Damping    Stepsize #Res #Jac #Sol   LinErr   LinRes
   1       0.072   0.5000000       0.072    8    4    4  3.2e-15  7.8e-15
Displacement field
Iter      SolEst     Damping    Stepsize #Res #Jac #Sol   LinErr   LinRes
   1        0.29   0.5000000        0.29    8    4    4  1.4e-13  1.5e-12
Spatial mesh displacement
Iter      SolEst     Damping    Stepsize #Res #Jac #Sol   LinErr   LinRes
   1       9e-07   0.5000000       9e-07    8    4    4  1.5e-15  1.8e-15
Solution error estimates for segregated groups
1, 0.036, 0.15, 4.5e-07
Residual error estimates for segregated groups
83, 5.1e+03, 1.1e+04, 0.15
Segregated solver iteration 5.
Merged variables
Iter      SolEst     Damping    Stepsize #Res #Jac #Sol   LinErr   LinRes
   1        0.41   0.3500000        0.41   18   13   13  1.4e-13  2.8e-15
   2        0.27   0.3500000        0.27   19   14   14  6.5e-14  2.8e-15
   3        0.18   0.3500000        0.18   20   15   15  5.6e-14  2.7e-15
Velocity u2, Pressure p
Iter      SolEst     Damping    Stepsize #Res #Jac #Sol   LinErr   LinRes
   1       0.035   0.5000000       0.035   10    5    5  2.9e-15  7.8e-15
Displacement field
Iter      SolEst     Damping    Stepsize #Res #Jac #Sol   LinErr   LinRes
   1        0.17   0.5000000        0.17   10    5    5  5.9e-14  1.5e-12
Spatial mesh displacement
Iter      SolEst     Damping    Stepsize #Res #Jac #Sol   LinErr   LinRes
   1     6.8e-07   0.5000000     6.8e-07   10    5    5  2.1e-15  1.9e-15
Solution error estimates for segregated groups
0.31, 0.017, 0.084, 3.4e-07
Residual error estimates for segregated groups
32, 2.6e+03, 6.8e+03, 0.15
Segregated solver iteration 6.
Merged variables
Iter      SolEst     Damping    Stepsize #Res #Jac #Sol   LinErr   LinRes
   1        0.12   0.3500000        0.12   22   16   16    1e-13  2.7e-15
   2       0.077   0.3500000       0.077   23   17   17  8.8e-14  2.7e-15
   3        0.05   0.3500000        0.05   24   18   18  4.6e-14  2.8e-15
Velocity u2, Pressure p
Iter      SolEst     Damping    Stepsize #Res #Jac #Sol   LinErr   LinRes
   1       0.017   0.5000000       0.017   12    6    6  2.4e-15  7.9e-15
Displacement field
Iter      SolEst     Damping    Stepsize #Res #Jac #Sol   LinErr   LinRes
   1       0.095   0.5000000       0.095   12    6    6  4.5e-14  1.5e-12
Spatial mesh displacement
Iter      SolEst     Damping    Stepsize #Res #Jac #Sol   LinErr   LinRes
   1     4.7e-07   0.5000000     4.7e-07   12    6    6  1.5e-15  1.9e-15
Solution error estimates for segregated groups
0.087, 0.0085, 0.048, 2.4e-07
Residual error estimates for segregated groups
12, 1.3e+03, 4.2e+03, 0.15
```

```
Segregated solver iteration 7.
Merged variables
Iter      SolEst     Damping    Stepsize #Res #Jac #Sol   LinErr   LinRes
   1       0.033   0.3500000       0.033   26   19   19  6.7e-14  2.8e-15
   2       0.021   0.3500000       0.021   27   20   20  9.5e-14  2.8e-15
   3       0.014   0.3500000       0.014   28   21   21  6.4e-14  2.8e-15
Velocity u2, Pressure p
Iter      SolEst     Damping    Stepsize #Res #Jac #Sol   LinErr   LinRes
   1      0.0085   0.5000000      0.0085   14    7    7  2.9e-15    8e-15
Displacement field
Scales for dependent variables:
Auxiliary pressure (comp1.solid.lemm1.pw): 0.36
Displacement field (comp1.u): 2.8e-16
Iter      SolEst     Damping    Stepsize #Res #Jac #Sol   LinErr   LinRes
   1       0.054   0.5000000       0.054   14    7    7  1.2e-13  1.2e-12
Spatial mesh displacement
Iter      SolEst     Damping    Stepsize #Res #Jac #Sol   LinErr   LinRes
   1     3.2e-07   0.5000000     3.2e-07   14    7    7  1.8e-15  1.9e-15
Solution error estimates for segregated groups
0.024, 0.0042, 0.027, 1.6e-07
Residual error estimates for segregated groups
4.1, 6.6e+02, 2.6e+03, 0.14
Segregated solver iteration 8.
Merged variables
Iter      SolEst     Damping    Stepsize #Res #Jac #Sol   LinErr   LinRes
   1      0.0091   0.3500000      0.0091   30   22   22  3.6e-14  2.9e-15
   2      0.0059   0.3500000      0.0059   31   23   23  6.4e-14  2.6e-15
   3      0.0038   0.3500000      0.0038   32   24   24  7.5e-14  2.8e-15
Velocity u2, Pressure p
Iter      SolEst     Damping    Stepsize #Res #Jac #Sol   LinErr   LinRes
   1      0.0042   0.5000000      0.0042   16    8    8  2.1e-15  7.8e-15
Displacement field
Iter      SolEst     Damping    Stepsize #Res #Jac #Sol   LinErr   LinRes
   1        0.03   0.5000000        0.03   16    8    8  1.6e-13  1.2e-12
Spatial mesh displacement
Iter      SolEst     Damping    Stepsize #Res #Jac #Sol   LinErr   LinRes
   1       2e-07   0.5000000       2e-07   16    8    8  1.7e-15  1.9e-15
Solution error estimates for segregated groups
0.0066, 0.0021, 0.015, 1e-07
Residual error estimates for segregated groups
1.4, 3.3e+02, 1.8e+03, 0.14
Segregated solver iteration 9.
Merged variables
Iter      SolEst     Damping    Stepsize #Res #Jac #Sol   LinErr   LinRes
   1      0.0025   0.3500000      0.0025   34   25   25  6.8e-14  2.8e-15
   2      0.0016   0.3500000      0.0016   35   26   26    1e-13  2.7e-15
   3      0.0011   0.3500000      0.0011   36   27   27  8.5e-14  2.7e-15
Velocity u2, Pressure p
Iter      SolEst     Damping    Stepsize #Res #Jac #Sol   LinErr   LinRes
   1      0.0021   0.5000000      0.0021   18    9    9  3.5e-15  7.7e-15
Displacement field
Iter      SolEst     Damping    Stepsize #Res #Jac #Sol   LinErr   LinRes
   1       0.017   0.5000000       0.017   18    9    9  9.5e-14  1.2e-12
Spatial mesh displacement
Iter      SolEst     Damping    Stepsize #Res #Jac #Sol   LinErr   LinRes
   1     1.3e-07   0.5000000     1.3e-07   18    9    9  1.7e-15  1.9e-15
```

```
Solution error estimates for segregated groups
0.0018, 0.0011, 0.0085, 6.3e-08
Residual error estimates for segregated groups
0.44, 1.7e+02, 1.4e+03, 0.14
Segregated solver iteration 10.
Merged variables
Iter      SolEst     Damping    Stepsize #Res #Jac #Sol   LinErr   LinRes
   1     0.00068   0.3500000     0.00068   38   28   28    5e-14  2.8e-15
   2     0.00044   0.3500000     0.00044   39   29   29  5.8e-14  2.7e-15
   3     0.00029   0.3500000     0.00029   40   30   30  5.9e-14  2.9e-15
Velocity u2, Pressure p
Iter      SolEst     Damping    Stepsize #Res #Jac #Sol   LinErr   LinRes
   1      0.0011   0.5000000      0.0011   20   10   10  2.6e-15    8e-15
Displacement field
Iter      SolEst     Damping    Stepsize #Res #Jac #Sol   LinErr   LinRes
   1      0.0094   0.5000000      0.0094   20   10   10  1.2e-13  1.2e-12
Spatial mesh displacement
Iter      SolEst     Damping    Stepsize #Res #Jac #Sol   LinErr   LinRes
   1     7.8e-08   0.5000000     7.8e-08   20   10   10  1.6e-15  1.9e-15
Solution error estimates for segregated groups
0.0005, 0.00053, 0.0047, 3.9e-08
Residual error estimates for segregated groups
0.14, 84, 1.2e+03, 0.15
Segregated solver iteration 11.
Merged variables
Iter      SolEst     Damping    Stepsize #Res #Jac #Sol   LinErr   LinRes
   1     0.00019   0.3500000     0.00019   42   31   31  1.1e-13  2.7e-15
   2     0.00012   0.3500000     0.00012   43   32   32  6.4e-14  2.8e-15
   3     7.9e-05   0.3500000     7.9e-05   44   33   33  1.1e-13  2.7e-15
Velocity u2, Pressure p
Iter      SolEst     Damping    Stepsize #Res #Jac #Sol   LinErr   LinRes
   1     0.00053   0.5000000     0.00053   22   11   11    2e-15  7.8e-15
Displacement field
Iter      SolEst     Damping    Stepsize #Res #Jac #Sol   LinErr   LinRes
   1      0.0051   0.5000000      0.0051   22   11   11  2.4e-13  1.2e-12
Spatial mesh displacement
Iter      SolEst     Damping    Stepsize #Res #Jac #Sol   LinErr   LinRes
   1     4.7e-08   0.5000000     4.7e-08   22   11   11  1.4e-15  1.8e-15
Solution error estimates for segregated groups
0.00014, 0.00026, 0.0026, 2.3e-08
Residual error estimates for segregated groups
0.043, 42, 1.2e+03, 0.15
Segregated solver iteration 12.
Merged variables
Iter      SolEst     Damping    Stepsize #Res #Jac #Sol   LinErr   LinRes
   1     5.2e-05   0.3500000     5.2e-05   46   34   34  5.2e-14  2.9e-15
   2     3.4e-05   0.3500000     3.4e-05   47   35   35  8.5e-14  2.7e-15
   3     2.2e-05   0.3500000     2.2e-05   48   36   36    1e-13  2.6e-15
Velocity u2, Pressure p
Iter      SolEst     Damping    Stepsize #Res #Jac #Sol   LinErr   LinRes
   1     0.00026   0.5000000     0.00026   24   12   12  3.2e-15  7.8e-15
Displacement field
Iter      SolEst     Damping    Stepsize #Res #Jac #Sol   LinErr   LinRes
   1      0.0028   0.5000000      0.0028   24   12   12  1.3e-13  1.2e-12
Spatial mesh displacement
Iter      SolEst     Damping    Stepsize #Res #Jac #Sol   LinErr   LinRes
```

```
   1      2.8e-08   0.5000000     2.8e-08   24   12   12  1.5e-15  1.8e-15
Solution error estimates for segregated groups
3.7e-05, 0.00013, 0.0014, 1.4e-08
Residual error estimates for segregated groups
0.013, 21, 1.2e+03, 0.14
Segregated solver iteration 13.
Merged variables
Iter      SolEst     Damping    Stepsize #Res #Jac #Sol   LinErr   LinRes
   1      1.4e-05   0.3500000     1.4e-05   50   37   37  7.4e-14  2.9e-15
   2      9.2e-06   0.3500000     9.2e-06   51   38   38  9.6e-14  2.7e-15
   3        6e-06   0.3500000       6e-06   52   39   39  6.5e-14  2.7e-15
Velocity u2, Pressure p
Iter      SolEst     Damping    Stepsize #Res #Jac #Sol   LinErr   LinRes
   1      0.00013   0.5000000     0.00013   26   13   13  2.3e-15  7.8e-15
Displacement field
Iter      SolEst     Damping    Stepsize #Res #Jac #Sol   LinErr   LinRes
   1       0.0015   0.5000000      0.0015   26   13   13  1.4e-13  1.2e-12
Spatial mesh displacement
Iter      SolEst     Damping    Stepsize #Res #Jac #Sol   LinErr   LinRes
   1      1.6e-08   0.5000000     1.6e-08   26   13   13  1.7e-15  1.9e-15
Solution error estimates for segregated groups
1e-05, 6.6e-05, 0.00076, 8e-09
Residual error estimates for segregated groups
0.004, 10, 1.2e+03, 0.16
Solution time: 157 s. (2 minutes, 37 seconds)
Physical memory: 4.7 GB
Virtual memory: 25.59 GB
Ended at Jan 31, 2025, 7:58:37 PM.
----- Stationary Solver 1 in study1/Approach Curve Solver (sol1) -------------->
```

d=2160 (su70)

GENERAL

Description	Value
Solution	d=2160

LOG

```
<---- Compile Equations: Stationary in study1/Approach Curve Solver (sol1) -----
Started at Jan 31, 2025, 7:58:37 PM.
Parameter d = 2.16E-6 (m).
Geometry shape function: Linear serendipity
Number of vertex elements: 18
Number of boundary elements: 6146
Number of elements: 154906
Minimum element quality: 0.481
Time: 8 s.
Physical memory: 3.01 GB
Virtual memory: 15.31 GB
Ended at Jan 31, 2025, 7:58:45 PM.
----- Compile Equations: Stationary in study1/Approach Curve Solver (sol1) ---->

<---- Dependent Variables 1 in study1/Approach Curve Solver (sol1) -------------
Started at Jan 31, 2025, 7:58:45 PM.
Solution time: 0 s.
Physical memory: 3.01 GB
Virtual memory: 15.25 GB
Ended at Jan 31, 2025, 7:58:46 PM.
----- Dependent Variables 1 in study1/Approach Curve Solver (sol1) ------------>

<---- Stationary Solver 1 in study1/Approach Curve Solver (sol1) ---------------
Started at Jan 31, 2025, 7:58:46 PM.
Segregated solver
Segregated Step 11 does not have any components solved for.
Number of degrees of freedom solved for: 786535 (plus 11715 internal DOFs).
Segregated solver iteration 1.
Merged variables
Nonsymmetric matrix found.
Scales for dependent variables:
Concentration (comp1.Cl): 1.4e+02
Concentration (comp1.K): 1.4e+02
Electric potential (comp1.V): 1.9e+02
Orthonormal null-space function used.
Iter      SolEst     Damping    Stepsize #Res #Jac #Sol   LinErr   LinRes
   1        0.83   0.3500000        0.83    2    1    1  3.3e-11  2.7e-15
   2        0.81   0.3500000        0.81    3    2    2  3.8e-11  2.8e-15
   3         0.8   0.3500000         0.8    4    3    3  1.4e-11  2.6e-15
Velocity u2, Pressure p
Nonsymmetric matrix found.
Scales for dependent variables:
Pressure (comp1.p): 1
Velocity field (spatial frame) (comp1.u2): 0.00018
Orthonormal null-space function used.
Iter      SolEst     Damping    Stepsize #Res #Jac #Sol   LinErr   LinRes
   1         1.1   0.5000000         1.1    2    1    1  1.5e-15  2.3e-15
Displacement field
Nonsymmetric matrix found.
Scales for dependent variables:
Auxiliary pressure (comp1.solid.lemm1.pw): 1
Displacement field (comp1.u): 2.8e-18
Orthonormal null-space function used.
Iter      SolEst     Damping    Stepsize #Res #Jac #Sol   LinErr   LinRes
   1         1.9   0.5000000         1.9    2    1    1  3.4e-14  1.5e-12
Spatial mesh displacement
```

```
Symmetric matrices found.
Scales for dependent variables:
Spatial mesh displacement (comp1.spatial.disp): 1.2e-09
Orthonormal null-space function used.
Iter      SolEst     Damping    Stepsize #Res #Jac #Sol   LinErr   LinRes
   1     7.2e-07   0.5000000     7.2e-07    2    1    1  1.9e-15  1.8e-15
Solution error estimates for segregated groups
1.4, 0.54, 0.95, 3.6e-07
Residual error estimates for segregated groups
7.8e+03, 8.2e+04, 2.2e+04, 0.13
Segregated solver iteration 2.
Merged variables
Scales for dependent variables:
Concentration (comp1.Cl): 1.4e+02
Concentration (comp1.K): 1.4e+02
Electric potential (comp1.V): 0.064
Iter      SolEst     Damping    Stepsize #Res #Jac #Sol   LinErr   LinRes
   1        0.78   0.3500000        0.78    6    4    4  1.2e-13  3.5e-15
   2        0.77   0.3500000        0.77    7    5    5    8e-14  3.2e-15
   3        0.76   0.3500000        0.76    8    6    6  1.3e-13  4.5e-15
Velocity u2, Pressure p
Scales for dependent variables:
Pressure (comp1.p): 3.6e+02
Velocity field (spatial frame) (comp1.u2): 0.00073
Iter      SolEst     Damping    Stepsize #Res #Jac #Sol   LinErr   LinRes
   1        0.36   0.5000000        0.36    4    2    2  2.5e-15  7.7e-15
Displacement field
Iter      SolEst     Damping    Stepsize #Res #Jac #Sol   LinErr   LinRes
   1        0.95   0.5000000        0.95    4    2    2  1.5e-13  1.5e-12
Spatial mesh displacement
Iter      SolEst     Damping    Stepsize #Res #Jac #Sol   LinErr   LinRes
   1     1.1e-06   0.5000000     1.1e-06    4    2    2  1.6e-15  1.8e-15
Solution error estimates for segregated groups
1.3, 0.18, 0.48, 5.4e-07
Residual error estimates for segregated groups
7.8e+02, 1.6e+04, 2.2e+04, 0.14
Segregated solver iteration 3.
Merged variables
Iter      SolEst     Damping    Stepsize #Res #Jac #Sol   LinErr   LinRes
   1        0.74   0.3500000        0.74   10    7    7    1e-13  3.4e-15
   2        0.73   0.3500000        0.73   11    8    8  8.3e-14  4.5e-15
   3        0.72   0.3500000        0.72   12    9    9    1e-13  3.6e-15
Velocity u2, Pressure p
Iter      SolEst     Damping    Stepsize #Res #Jac #Sol   LinErr   LinRes
   1        0.15   0.5000000        0.15    6    3    3  2.7e-15  7.9e-15
Displacement field
Iter      SolEst     Damping    Stepsize #Res #Jac #Sol   LinErr   LinRes
   1        0.52   0.5000000        0.52    6    3    3  1.2e-13  1.5e-12
Spatial mesh displacement
Iter      SolEst     Damping    Stepsize #Res #Jac #Sol   LinErr   LinRes
   1     1.1e-06   0.5000000     1.1e-06    6    3    3  1.7e-15  1.8e-15
Solution error estimates for segregated groups
1.2, 0.077, 0.26, 5.4e-07
Residual error estimates for segregated groups
2.2e+02, 9.3e+03, 1.6e+04, 0.16
Segregated solver iteration 4.
```

```
Merged variables
Iter      SolEst     Damping    Stepsize #Res #Jac #Sol   LinErr   LinRes
   1         0.7   0.3500000         0.7   14   10   10  9.6e-14  6.9e-15
   2        0.68   0.3500000        0.68   15   11   11  6.6e-14  3.9e-15
   3        0.59   0.3500000        0.59   16   12   12    1e-13  3.9e-15
Velocity u2, Pressure p
Iter      SolEst     Damping    Stepsize #Res #Jac #Sol   LinErr   LinRes
   1       0.072   0.5000000       0.072    8    4    4  1.7e-15  7.8e-15
Displacement field
Iter      SolEst     Damping    Stepsize #Res #Jac #Sol   LinErr   LinRes
   1        0.29   0.5000000        0.29    8    4    4  4.1e-14  1.5e-12
Spatial mesh displacement
Iter      SolEst     Damping    Stepsize #Res #Jac #Sol   LinErr   LinRes
   1       9e-07   0.5000000       9e-07    8    4    4  1.9e-15  1.8e-15
Solution error estimates for segregated groups
1, 0.036, 0.15, 4.5e-07
Residual error estimates for segregated groups
83, 4.9e+03, 1.1e+04, 0.16
Segregated solver iteration 5.
Merged variables
Iter      SolEst     Damping    Stepsize #Res #Jac #Sol   LinErr   LinRes
   1        0.41   0.3500000        0.41   18   13   13  9.7e-14  6.3e-15
   2        0.27   0.3500000        0.27   19   14   14  1.2e-13  5.4e-15
   3        0.18   0.3500000        0.18   20   15   15  1.5e-13  4.5e-15
Velocity u2, Pressure p
Iter      SolEst     Damping    Stepsize #Res #Jac #Sol   LinErr   LinRes
   1       0.035   0.5000000       0.035   10    5    5  2.6e-15  7.8e-15
Displacement field
Iter      SolEst     Damping    Stepsize #Res #Jac #Sol   LinErr   LinRes
   1        0.17   0.5000000        0.17   10    5    5  1.5e-13  1.5e-12
Spatial mesh displacement
Iter      SolEst     Damping    Stepsize #Res #Jac #Sol   LinErr   LinRes
   1     6.8e-07   0.5000000     6.8e-07   10    5    5  2.4e-15  1.9e-15
Solution error estimates for segregated groups
0.31, 0.017, 0.084, 3.4e-07
Residual error estimates for segregated groups
32, 2.5e+03, 6.8e+03, 0.15
Segregated solver iteration 6.
Merged variables
Iter      SolEst     Damping    Stepsize #Res #Jac #Sol   LinErr   LinRes
   1        0.12   0.3500000        0.12   22   16   16    1e-13    4e-15
   2       0.077   0.3500000       0.077   23   17   17  1.3e-13  3.9e-15
   3        0.05   0.3500000        0.05   24   18   18  1.3e-13    4e-15
Velocity u2, Pressure p
Iter      SolEst     Damping    Stepsize #Res #Jac #Sol   LinErr   LinRes
   1       0.017   0.5000000       0.017   12    6    6  2.6e-15  7.8e-15
Displacement field
Iter      SolEst     Damping    Stepsize #Res #Jac #Sol   LinErr   LinRes
   1       0.095   0.5000000       0.095   12    6    6  6.1e-14  1.5e-12
Spatial mesh displacement
Iter      SolEst     Damping    Stepsize #Res #Jac #Sol   LinErr   LinRes
   1     4.7e-07   0.5000000     4.7e-07   12    6    6  1.4e-15  1.9e-15
Solution error estimates for segregated groups
0.086, 0.0085, 0.048, 2.4e-07
Residual error estimates for segregated groups
12, 1.3e+03, 4.2e+03, 0.14
```

```
Segregated solver iteration 7.
Merged variables
Iter      SolEst     Damping    Stepsize #Res #Jac #Sol   LinErr   LinRes
   1       0.033   0.3500000       0.033   26   19   19  7.9e-14  4.9e-15
   2       0.021   0.3500000       0.021   27   20   20  6.2e-14  3.9e-15
   3       0.014   0.3500000       0.014   28   21   21  7.6e-14  3.9e-15
Velocity u2, Pressure p
Iter      SolEst     Damping    Stepsize #Res #Jac #Sol   LinErr   LinRes
   1      0.0085   0.5000000      0.0085   14    7    7  2.4e-15  7.9e-15
Displacement field
Scales for dependent variables:
Auxiliary pressure (comp1.solid.lemm1.pw): 0.36
Displacement field (comp1.u): 2.8e-16
Iter      SolEst     Damping    Stepsize #Res #Jac #Sol   LinErr   LinRes
   1       0.054   0.5000000       0.054   14    7    7  2.3e-13  1.2e-12
Spatial mesh displacement
Iter      SolEst     Damping    Stepsize #Res #Jac #Sol   LinErr   LinRes
   1     3.2e-07   0.5000000     3.2e-07   14    7    7  1.4e-15  1.9e-15
Solution error estimates for segregated groups
0.024, 0.0042, 0.027, 1.6e-07
Residual error estimates for segregated groups
4.1, 6.5e+02, 2.6e+03, 0.14
Segregated solver iteration 8.
Merged variables
Iter      SolEst     Damping    Stepsize #Res #Jac #Sol   LinErr   LinRes
   1       0.009   0.3500000       0.009   30   22   22  1.3e-13    4e-15
   2      0.0059   0.3500000      0.0059   31   23   23  7.3e-14  4.9e-15
   3      0.0038   0.3500000      0.0038   32   24   24  1.7e-13  3.6e-15
Velocity u2, Pressure p
Iter      SolEst     Damping    Stepsize #Res #Jac #Sol   LinErr   LinRes
   1      0.0042   0.5000000      0.0042   16    8    8  5.1e-15  7.9e-15
Displacement field
Iter      SolEst     Damping    Stepsize #Res #Jac #Sol   LinErr   LinRes
   1        0.03   0.5000000        0.03   16    8    8  1.2e-13  1.2e-12
Spatial mesh displacement
Iter      SolEst     Damping    Stepsize #Res #Jac #Sol   LinErr   LinRes
   1       2e-07   0.5000000       2e-07   16    8    8  1.3e-15  1.8e-15
Solution error estimates for segregated groups
0.0066, 0.0021, 0.015, 1e-07
Residual error estimates for segregated groups
1.4, 3.2e+02, 1.8e+03, 0.15
Segregated solver iteration 9.
Merged variables
Iter      SolEst     Damping    Stepsize #Res #Jac #Sol   LinErr   LinRes
   1      0.0025   0.3500000      0.0025   34   25   25  7.9e-14  3.9e-15
   2      0.0016   0.3500000      0.0016   35   26   26  1.8e-13  5.4e-15
   3      0.0011   0.3500000      0.0011   36   27   27  9.9e-14    4e-15
Velocity u2, Pressure p
Iter      SolEst     Damping    Stepsize #Res #Jac #Sol   LinErr   LinRes
   1      0.0021   0.5000000      0.0021   18    9    9  4.7e-15  7.9e-15
Displacement field
Iter      SolEst     Damping    Stepsize #Res #Jac #Sol   LinErr   LinRes
   1       0.017   0.5000000       0.017   18    9    9  1.4e-13  1.2e-12
Spatial mesh displacement
Iter      SolEst     Damping    Stepsize #Res #Jac #Sol   LinErr   LinRes
   1     1.3e-07   0.5000000     1.3e-07   18    9    9  1.4e-15  1.9e-15
```

```
Solution error estimates for segregated groups
0.0018, 0.0011, 0.0085, 6.4e-08
Residual error estimates for segregated groups
0.44, 1.6e+02, 1.4e+03, 0.16
Segregated solver iteration 10.
Merged variables
Iter      SolEst     Damping    Stepsize #Res #Jac #Sol   LinErr   LinRes
   1     0.00068   0.3500000     0.00068   38   28   28  5.5e-14    4e-15
   2     0.00044   0.3500000     0.00044   39   29   29  5.7e-14  3.3e-15
   3     0.00029   0.3500000     0.00029   40   30   30  7.5e-14  4.7e-15
Velocity u2, Pressure p
Iter      SolEst     Damping    Stepsize #Res #Jac #Sol   LinErr   LinRes
   1      0.0011   0.5000000      0.0011   20   10   10  2.5e-15  7.9e-15
Displacement field
Iter      SolEst     Damping    Stepsize #Res #Jac #Sol   LinErr   LinRes
   1      0.0094   0.5000000      0.0094   20   10   10  1.1e-13  1.2e-12
Spatial mesh displacement
Iter      SolEst     Damping    Stepsize #Res #Jac #Sol   LinErr   LinRes
   1     7.8e-08   0.5000000     7.8e-08   20   10   10  1.3e-15  1.9e-15
Solution error estimates for segregated groups
0.0005, 0.00053, 0.0047, 3.9e-08
Residual error estimates for segregated groups
0.14, 81, 1.2e+03, 0.15
Segregated solver iteration 11.
Merged variables
Iter      SolEst     Damping    Stepsize #Res #Jac #Sol   LinErr   LinRes
   1     0.00019   0.3500000     0.00019   42   31   31  1.2e-13  4.5e-15
   2     0.00012   0.3500000     0.00012   43   32   32  8.6e-14  3.4e-15
   3     7.9e-05   0.3500000     7.9e-05   44   33   33  4.6e-14    4e-15
Velocity u2, Pressure p
Iter      SolEst     Damping    Stepsize #Res #Jac #Sol   LinErr   LinRes
   1     0.00053   0.5000000     0.00053   22   11   11  2.3e-15  7.7e-15
Displacement field
Iter      SolEst     Damping    Stepsize #Res #Jac #Sol   LinErr   LinRes
   1      0.0051   0.5000000      0.0051   22   11   11  1.1e-13  1.2e-12
Spatial mesh displacement
Iter      SolEst     Damping    Stepsize #Res #Jac #Sol   LinErr   LinRes
   1     4.7e-08   0.5000000     4.7e-08   22   11   11  1.3e-15  1.8e-15
Solution error estimates for segregated groups
0.00014, 0.00026, 0.0026, 2.3e-08
Residual error estimates for segregated groups
0.043, 41, 1.2e+03, 0.14
Segregated solver iteration 12.
Merged variables
Iter      SolEst     Damping    Stepsize #Res #Jac #Sol   LinErr   LinRes
   1     5.2e-05   0.3500000     5.2e-05   46   34   34  9.7e-14  4.9e-15
   2     3.3e-05   0.3500000     3.3e-05   47   35   35  8.8e-14    5e-15
   3     2.2e-05   0.3500000     2.2e-05   48   36   36  8.4e-14  4.7e-15
Velocity u2, Pressure p
Iter      SolEst     Damping    Stepsize #Res #Jac #Sol   LinErr   LinRes
   1     0.00026   0.5000000     0.00026   24   12   12  3.6e-15  7.8e-15
Displacement field
Iter      SolEst     Damping    Stepsize #Res #Jac #Sol   LinErr   LinRes
   1      0.0028   0.5000000      0.0028   24   12   12  1.1e-13  1.2e-12
Spatial mesh displacement
Iter      SolEst     Damping    Stepsize #Res #Jac #Sol   LinErr   LinRes
```

```
   1     2.8e-08   0.5000000     2.8e-08   24   12   12  1.4e-15  1.9e-15
Solution error estimates for segregated groups
3.7e-05, 0.00013, 0.0014, 1.4e-08
Residual error estimates for segregated groups
0.013, 20, 1.2e+03, 0.15
Segregated solver iteration 13.
Merged variables
Iter      SolEst     Damping    Stepsize #Res #Jac #Sol   LinErr   LinRes
   1     1.4e-05   0.3500000     1.4e-05   50   37   37  1.3e-13  3.9e-15
   2     9.2e-06   0.3500000     9.2e-06   51   38   38  1.2e-13  4.4e-15
   3       6e-06   0.3500000       6e-06   52   39   39  7.9e-14  4.6e-15
Velocity u2, Pressure p
Iter      SolEst     Damping    Stepsize #Res #Jac #Sol   LinErr   LinRes
   1     0.00013   0.5000000     0.00013   26   13   13    4e-15  7.8e-15
Displacement field
Iter      SolEst     Damping    Stepsize #Res #Jac #Sol   LinErr   LinRes
   1      0.0015   0.5000000      0.0015   26   13   13  2.5e-13  1.2e-12
Spatial mesh displacement
Iter      SolEst     Damping    Stepsize #Res #Jac #Sol   LinErr   LinRes
   1     1.6e-08   0.5000000     1.6e-08   26   13   13  1.5e-15  1.9e-15
Solution error estimates for segregated groups
1e-05, 6.6e-05, 0.00076, 8e-09
Residual error estimates for segregated groups
0.004, 10, 1.2e+03, 0.15
Solution time: 157 s. (2 minutes, 37 seconds)
Physical memory: 4.71 GB
Virtual memory: 25.52 GB
Ended at Jan 31, 2025, 8:01:22 PM.
----- Stationary Solver 1 in study1/Approach Curve Solver (sol1) -------------->
```

d=2155 (su71)

GENERAL

Description	Value
Solution	d=2155

LOG

```
<---- Compile Equations: Stationary in study1/Approach Curve Solver (sol1) -----
Started at Jan 31, 2025, 8:01:23 PM.
Parameter d = 2.155E-6 (m).
Geometry shape function: Linear serendipity
Number of vertex elements: 18
Number of boundary elements: 6144
Number of elements: 154668
Minimum element quality: 0.4727
Time: 8 s.
Physical memory: 2.97 GB
Virtual memory: 15.27 GB
Ended at Jan 31, 2025, 8:01:31 PM.
----- Compile Equations: Stationary in study1/Approach Curve Solver (sol1) ---->

<---- Dependent Variables 1 in study1/Approach Curve Solver (sol1) -------------
Started at Jan 31, 2025, 8:01:31 PM.
Solution time: 0 s.
Physical memory: 2.97 GB
Virtual memory: 15.2 GB
Ended at Jan 31, 2025, 8:01:31 PM.
----- Dependent Variables 1 in study1/Approach Curve Solver (sol1) ------------>

<---- Stationary Solver 1 in study1/Approach Curve Solver (sol1) ---------------
Started at Jan 31, 2025, 8:01:31 PM.
Segregated solver
Segregated Step 11 does not have any components solved for.
Number of degrees of freedom solved for: 785339 (plus 11711 internal DOFs).
Segregated solver iteration 1.
Merged variables
Nonsymmetric matrix found.
Scales for dependent variables:
Concentration (comp1.Cl): 1.4e+02
Concentration (comp1.K): 1.4e+02
Electric potential (comp1.V): 1.9e+02
Orthonormal null-space function used.
Iter      SolEst     Damping    Stepsize #Res #Jac #Sol   LinErr   LinRes
   1        0.83   0.3500000        0.83    2    1    1  2.8e-11  2.7e-15
   2        0.81   0.3500000        0.81    3    2    2  2.7e-11  2.7e-15
   3         0.8   0.3500000         0.8    4    3    3  5.3e-11  2.6e-15
Velocity u2, Pressure p
Nonsymmetric matrix found.
Scales for dependent variables:
Pressure (comp1.p): 1
Velocity field (spatial frame) (comp1.u2): 0.00018
Orthonormal null-space function used.
Iter      SolEst     Damping    Stepsize #Res #Jac #Sol   LinErr   LinRes
   1         1.1   0.5000000         1.1    2    1    1  1.8e-15  2.3e-15
Displacement field
Nonsymmetric matrix found.
Scales for dependent variables:
Auxiliary pressure (comp1.solid.lemm1.pw): 1
Displacement field (comp1.u): 2.8e-18
Orthonormal null-space function used.
Iter      SolEst     Damping    Stepsize #Res #Jac #Sol   LinErr   LinRes
   1         1.9   0.5000000         1.9    2    1    1  1.1e-13  1.5e-12
Spatial mesh displacement
```

```
Symmetric matrices found.
Scales for dependent variables:
Spatial mesh displacement (comp1.spatial.disp): 1.2e-09
Orthonormal null-space function used.
Iter      SolEst     Damping    Stepsize #Res #Jac #Sol   LinErr   LinRes
   1     7.2e-07   0.5000000     7.2e-07    2    1    1  1.4e-15  1.8e-15
Solution error estimates for segregated groups
1.4, 0.54, 0.95, 3.6e-07
Residual error estimates for segregated groups
7.8e+03, 8.2e+04, 2.2e+04, 0.12
Segregated solver iteration 2.
Merged variables
Scales for dependent variables:
Concentration (comp1.Cl): 1.4e+02
Concentration (comp1.K): 1.4e+02
Electric potential (comp1.V): 0.064
Iter      SolEst     Damping    Stepsize #Res #Jac #Sol   LinErr   LinRes
   1        0.78   0.3500000        0.78    6    4    4  7.4e-14  2.6e-15
   2        0.77   0.3500000        0.77    7    5    5  4.7e-14  2.6e-15
   3        0.76   0.3500000        0.76    8    6    6  7.5e-14  2.6e-15
Velocity u2, Pressure p
Scales for dependent variables:
Pressure (comp1.p): 3.6e+02
Velocity field (spatial frame) (comp1.u2): 0.00073
Iter      SolEst     Damping    Stepsize #Res #Jac #Sol   LinErr   LinRes
   1        0.36   0.5000000        0.36    4    2    2  2.3e-15  7.9e-15
Displacement field
Iter      SolEst     Damping    Stepsize #Res #Jac #Sol   LinErr   LinRes
   1        0.95   0.5000000        0.95    4    2    2  8.8e-14  1.5e-12
Spatial mesh displacement
Iter      SolEst     Damping    Stepsize #Res #Jac #Sol   LinErr   LinRes
   1     1.1e-06   0.5000000     1.1e-06    4    2    2  1.3e-15  1.8e-15
Solution error estimates for segregated groups
1.3, 0.18, 0.48, 5.4e-07
Residual error estimates for segregated groups
7.8e+02, 1.3e+04, 2.2e+04, 0.15
Segregated solver iteration 3.
Merged variables
Iter      SolEst     Damping    Stepsize #Res #Jac #Sol   LinErr   LinRes
   1        0.74   0.3500000        0.74   10    7    7  1.3e-13  2.6e-15
   2        0.73   0.3500000        0.73   11    8    8  1.1e-13  2.6e-15
   3        0.72   0.3500000        0.72   12    9    9  5.8e-14  2.6e-15
Velocity u2, Pressure p
Iter      SolEst     Damping    Stepsize #Res #Jac #Sol   LinErr   LinRes
   1        0.15   0.5000000        0.15    6    3    3  2.7e-15  7.8e-15
Displacement field
Iter      SolEst     Damping    Stepsize #Res #Jac #Sol   LinErr   LinRes
   1        0.52   0.5000000        0.52    6    3    3  1.5e-13  1.5e-12
Spatial mesh displacement
Iter      SolEst     Damping    Stepsize #Res #Jac #Sol   LinErr   LinRes
   1     1.1e-06   0.5000000     1.1e-06    6    3    3  1.6e-15  1.9e-15
Solution error estimates for segregated groups
1.2, 0.077, 0.26, 5.4e-07
Residual error estimates for segregated groups
2.2e+02, 7.5e+03, 1.6e+04, 0.16
Segregated solver iteration 4.
```

```
Merged variables
Iter      SolEst     Damping    Stepsize #Res #Jac #Sol   LinErr   LinRes
   1         0.7   0.3500000         0.7   14   10   10    6e-14  2.7e-15
   2        0.68   0.3500000        0.68   15   11   11    7e-14  2.6e-15
   3        0.59   0.3500000        0.59   16   12   12  6.7e-14  2.6e-15
Velocity u2, Pressure p
Iter      SolEst     Damping    Stepsize #Res #Jac #Sol   LinErr   LinRes
   1       0.072   0.5000000       0.072    8    4    4  2.5e-15  7.8e-15
Displacement field
Iter      SolEst     Damping    Stepsize #Res #Jac #Sol   LinErr   LinRes
   1        0.29   0.5000000        0.29    8    4    4  4.5e-14  1.5e-12
Spatial mesh displacement
Iter      SolEst     Damping    Stepsize #Res #Jac #Sol   LinErr   LinRes
   1     9.1e-07   0.5000000     9.1e-07    8    4    4  1.9e-15  1.8e-15
Solution error estimates for segregated groups
1, 0.036, 0.15, 4.5e-07
Residual error estimates for segregated groups
83, 4e+03, 1.1e+04, 0.15
Segregated solver iteration 5.
Merged variables
Iter      SolEst     Damping    Stepsize #Res #Jac #Sol   LinErr   LinRes
   1        0.41   0.3500000        0.41   18   13   13  1.6e-13  2.7e-15
   2        0.27   0.3500000        0.27   19   14   14  6.8e-14  2.7e-15
   3        0.18   0.3500000        0.18   20   15   15  8.5e-14  2.6e-15
Velocity u2, Pressure p
Iter      SolEst     Damping    Stepsize #Res #Jac #Sol   LinErr   LinRes
   1       0.035   0.5000000       0.035   10    5    5  2.4e-15  7.8e-15
Displacement field
Iter      SolEst     Damping    Stepsize #Res #Jac #Sol   LinErr   LinRes
   1        0.17   0.5000000        0.17   10    5    5  1.4e-13  1.5e-12
Spatial mesh displacement
Iter      SolEst     Damping    Stepsize #Res #Jac #Sol   LinErr   LinRes
   1     6.8e-07   0.5000000     6.8e-07   10    5    5  1.6e-15  1.8e-15
Solution error estimates for segregated groups
0.31, 0.017, 0.084, 3.4e-07
Residual error estimates for segregated groups
32, 2e+03, 6.8e+03, 0.14
Segregated solver iteration 6.
Merged variables
Iter      SolEst     Damping    Stepsize #Res #Jac #Sol   LinErr   LinRes
   1        0.12   0.3500000        0.12   22   16   16    5e-14  2.6e-15
   2       0.077   0.3500000       0.077   23   17   17  7.2e-14  2.6e-15
   3        0.05   0.3500000        0.05   24   18   18  7.5e-14  2.7e-15
Velocity u2, Pressure p
Iter      SolEst     Damping    Stepsize #Res #Jac #Sol   LinErr   LinRes
   1       0.017   0.5000000       0.017   12    6    6    3e-15  7.9e-15
Displacement field
Iter      SolEst     Damping    Stepsize #Res #Jac #Sol   LinErr   LinRes
   1       0.095   0.5000000       0.095   12    6    6  5.1e-14  1.5e-12
Spatial mesh displacement
Iter      SolEst     Damping    Stepsize #Res #Jac #Sol   LinErr   LinRes
   1     4.8e-07   0.5000000     4.8e-07   12    6    6  1.6e-15  1.8e-15
Solution error estimates for segregated groups
0.086, 0.0085, 0.048, 2.4e-07
Residual error estimates for segregated groups
12, 1e+03, 4.2e+03, 0.15
```

```
Segregated solver iteration 7.
Merged variables
Iter      SolEst     Damping    Stepsize #Res #Jac #Sol   LinErr   LinRes
   1       0.033   0.3500000       0.033   26   19   19  4.8e-14  2.7e-15
   2       0.021   0.3500000       0.021   27   20   20  9.9e-14  2.6e-15
   3       0.014   0.3500000       0.014   28   21   21  1.7e-13  2.7e-15
Velocity u2, Pressure p
Iter      SolEst     Damping    Stepsize #Res #Jac #Sol   LinErr   LinRes
   1      0.0085   0.5000000      0.0085   14    7    7  2.8e-15    8e-15
Displacement field
Scales for dependent variables:
Auxiliary pressure (comp1.solid.lemm1.pw): 0.36
Displacement field (comp1.u): 2.8e-16
Iter      SolEst     Damping    Stepsize #Res #Jac #Sol   LinErr   LinRes
   1       0.054   0.5000000       0.054   14    7    7  1.2e-13  1.2e-12
Spatial mesh displacement
Iter      SolEst     Damping    Stepsize #Res #Jac #Sol   LinErr   LinRes
   1     3.2e-07   0.5000000     3.2e-07   14    7    7  1.5e-15  1.9e-15
Solution error estimates for segregated groups
0.024, 0.0042, 0.027, 1.6e-07
Residual error estimates for segregated groups
4.1, 5.2e+02, 2.6e+03, 0.16
Segregated solver iteration 8.
Merged variables
Iter      SolEst     Damping    Stepsize #Res #Jac #Sol   LinErr   LinRes
   1       0.009   0.3500000       0.009   30   22   22  1.4e-13  2.6e-15
   2      0.0059   0.3500000      0.0059   31   23   23  9.6e-14  2.6e-15
   3      0.0038   0.3500000      0.0038   32   24   24  6.3e-14  2.6e-15
Velocity u2, Pressure p
Iter      SolEst     Damping    Stepsize #Res #Jac #Sol   LinErr   LinRes
   1      0.0042   0.5000000      0.0042   16    8    8  3.1e-15  7.9e-15
Displacement field
Iter      SolEst     Damping    Stepsize #Res #Jac #Sol   LinErr   LinRes
   1        0.03   0.5000000        0.03   16    8    8    1e-13  1.2e-12
Spatial mesh displacement
Iter      SolEst     Damping    Stepsize #Res #Jac #Sol   LinErr   LinRes
   1       2e-07   0.5000000       2e-07   16    8    8  1.5e-15  1.8e-15
Solution error estimates for segregated groups
0.0066, 0.0021, 0.015, 1e-07
Residual error estimates for segregated groups
1.4, 2.6e+02, 1.8e+03, 0.17
Segregated solver iteration 9.
Merged variables
Iter      SolEst     Damping    Stepsize #Res #Jac #Sol   LinErr   LinRes
   1      0.0025   0.3500000      0.0025   34   25   25  7.5e-14  2.6e-15
   2      0.0016   0.3500000      0.0016   35   26   26  7.5e-14  2.7e-15
   3       0.001   0.3500000       0.001   36   27   27  1.6e-13  2.7e-15
Velocity u2, Pressure p
Iter      SolEst     Damping    Stepsize #Res #Jac #Sol   LinErr   LinRes
   1      0.0021   0.5000000      0.0021   18    9    9  2.6e-15  7.9e-15
Displacement field
Iter      SolEst     Damping    Stepsize #Res #Jac #Sol   LinErr   LinRes
   1       0.017   0.5000000       0.017   18    9    9  9.7e-14  1.2e-12
Spatial mesh displacement
Iter      SolEst     Damping    Stepsize #Res #Jac #Sol   LinErr   LinRes
   1     1.3e-07   0.5000000     1.3e-07   18    9    9  1.4e-15  1.8e-15
```

```
Solution error estimates for segregated groups
0.0018, 0.0011, 0.0085, 6.4e-08
Residual error estimates for segregated groups
0.44, 1.3e+02, 1.4e+03, 0.15
Segregated solver iteration 10.
Merged variables
Iter      SolEst     Damping    Stepsize #Res #Jac #Sol   LinErr   LinRes
   1     0.00068   0.3500000     0.00068   38   28   28    1e-13  2.6e-15
   2     0.00044   0.3500000     0.00044   39   29   29  7.8e-14  2.7e-15
   3     0.00029   0.3500000     0.00029   40   30   30    7e-14  2.6e-15
Velocity u2, Pressure p
Iter      SolEst     Damping    Stepsize #Res #Jac #Sol   LinErr   LinRes
   1      0.0011   0.5000000      0.0011   20   10   10  2.1e-15  7.9e-15
Displacement field
Iter      SolEst     Damping    Stepsize #Res #Jac #Sol   LinErr   LinRes
   1      0.0094   0.5000000      0.0094   20   10   10  1.5e-13  1.2e-12
Spatial mesh displacement
Iter      SolEst     Damping    Stepsize #Res #Jac #Sol   LinErr   LinRes
   1     7.8e-08   0.5000000     7.8e-08   20   10   10  1.9e-15  1.8e-15
Solution error estimates for segregated groups
0.00049, 0.00053, 0.0047, 3.9e-08
Residual error estimates for segregated groups
0.14, 65, 1.2e+03, 0.14
Segregated solver iteration 11.
Merged variables
Iter      SolEst     Damping    Stepsize #Res #Jac #Sol   LinErr   LinRes
   1     0.00019   0.3500000     0.00019   42   31   31    6e-14  2.6e-15
   2     0.00012   0.3500000     0.00012   43   32   32    6e-14  2.6e-15
   3     7.9e-05   0.3500000     7.9e-05   44   33   33  1.2e-13  2.6e-15
Velocity u2, Pressure p
Iter      SolEst     Damping    Stepsize #Res #Jac #Sol   LinErr   LinRes
   1     0.00053   0.5000000     0.00053   22   11   11    3e-15  7.8e-15
Displacement field
Iter      SolEst     Damping    Stepsize #Res #Jac #Sol   LinErr   LinRes
   1      0.0051   0.5000000      0.0051   22   11   11  1.5e-13  1.2e-12
Spatial mesh displacement
Iter      SolEst     Damping    Stepsize #Res #Jac #Sol   LinErr   LinRes
   1     4.7e-08   0.5000000     4.7e-08   22   11   11  1.6e-15  1.8e-15
Solution error estimates for segregated groups
0.00014, 0.00026, 0.0026, 2.3e-08
Residual error estimates for segregated groups
0.043, 33, 1.2e+03, 0.15
Segregated solver iteration 12.
Merged variables
Iter      SolEst     Damping    Stepsize #Res #Jac #Sol   LinErr   LinRes
   1     5.1e-05   0.3500000     5.1e-05   46   34   34  7.5e-14  2.7e-15
   2     3.3e-05   0.3500000     3.3e-05   47   35   35  9.5e-14  2.6e-15
   3     2.2e-05   0.3500000     2.2e-05   48   36   36    6e-14  2.6e-15
Velocity u2, Pressure p
Iter      SolEst     Damping    Stepsize #Res #Jac #Sol   LinErr   LinRes
   1     0.00026   0.5000000     0.00026   24   12   12  2.3e-15  7.9e-15
Displacement field
Iter      SolEst     Damping    Stepsize #Res #Jac #Sol   LinErr   LinRes
   1      0.0028   0.5000000      0.0028   24   12   12  9.7e-14  1.2e-12
Spatial mesh displacement
Iter      SolEst     Damping    Stepsize #Res #Jac #Sol   LinErr   LinRes
```

```
   1     2.8e-08   0.5000000     2.8e-08   24   12   12  1.7e-15  1.8e-15
Solution error estimates for segregated groups
3.7e-05, 0.00013, 0.0014, 1.4e-08
Residual error estimates for segregated groups
0.013, 16, 1.1e+03, 0.15
Segregated solver iteration 13.
Merged variables
Iter      SolEst     Damping    Stepsize #Res #Jac #Sol   LinErr   LinRes
   1     1.4e-05   0.3500000     1.4e-05   50   37   37  7.8e-14  2.7e-15
   2     9.2e-06   0.3500000     9.2e-06   51   38   38  7.4e-14  2.6e-15
   3       6e-06   0.3500000       6e-06   52   39   39  4.2e-14  2.7e-15
Velocity u2, Pressure p
Iter      SolEst     Damping    Stepsize #Res #Jac #Sol   LinErr   LinRes
   1     0.00013   0.5000000     0.00013   26   13   13  2.1e-15  7.8e-15
Displacement field
Iter      SolEst     Damping    Stepsize #Res #Jac #Sol   LinErr   LinRes
   1      0.0015   0.5000000      0.0015   26   13   13    1e-13  1.2e-12
Spatial mesh displacement
Iter      SolEst     Damping    Stepsize #Res #Jac #Sol   LinErr   LinRes
   1     1.6e-08   0.5000000     1.6e-08   26   13   13  1.7e-15  1.8e-15
Solution error estimates for segregated groups
1e-05, 6.6e-05, 0.00076, 8.1e-09
Residual error estimates for segregated groups
0.004, 8.2, 1.2e+03, 0.15
Solution time: 156 s. (2 minutes, 36 seconds)
Physical memory: 4.72 GB
Virtual memory: 25.52 GB
Ended at Jan 31, 2025, 8:04:07 PM.
----- Stationary Solver 1 in study1/Approach Curve Solver (sol1) -------------->
```

d=2150 (su72)

GENERAL

Description	Value
Solution	d=2150

LOG

```
<---- Compile Equations: Stationary in study1/Approach Curve Solver (sol1) -----
Started at Jan 31, 2025, 8:04:07 PM.
Parameter d = 2.15E-6 (m).
Geometry shape function: Linear serendipity
Number of vertex elements: 18
Number of boundary elements: 6141
Number of elements: 154455
Minimum element quality: 0.4813
Time: 8 s.
Physical memory: 2.97 GB
Virtual memory: 15.27 GB
Ended at Jan 31, 2025, 8:04:15 PM.
----- Compile Equations: Stationary in study1/Approach Curve Solver (sol1) ---->

<---- Dependent Variables 1 in study1/Approach Curve Solver (sol1) -------------
Started at Jan 31, 2025, 8:04:15 PM.
Solution time: 0 s.
Physical memory: 2.97 GB
Virtual memory: 15.21 GB
Ended at Jan 31, 2025, 8:04:15 PM.
----- Dependent Variables 1 in study1/Approach Curve Solver (sol1) ------------>

<---- Stationary Solver 1 in study1/Approach Curve Solver (sol1) ---------------
Started at Jan 31, 2025, 8:04:15 PM.
Segregated solver
Segregated Step 11 does not have any components solved for.
Number of degrees of freedom solved for: 784130 (plus 11705 internal DOFs).
Segregated solver iteration 1.
Merged variables
Nonsymmetric matrix found.
Scales for dependent variables:
Concentration (comp1.Cl): 1.4e+02
Concentration (comp1.K): 1.4e+02
Electric potential (comp1.V): 1.9e+02
Orthonormal null-space function used.
Iter      SolEst     Damping    Stepsize #Res #Jac #Sol   LinErr   LinRes
   1        0.83   0.3500000        0.83    2    1    1  2.6e-11  2.7e-15
   2        0.81   0.3500000        0.81    3    2    2    4e-11  2.7e-15
   3         0.8   0.3500000         0.8    4    3    3  2.2e-11  2.8e-15
Velocity u2, Pressure p
Nonsymmetric matrix found.
Scales for dependent variables:
Pressure (comp1.p): 1
Velocity field (spatial frame) (comp1.u2): 0.00018
Orthonormal null-space function used.
Iter      SolEst     Damping    Stepsize #Res #Jac #Sol   LinErr   LinRes
   1         1.1   0.5000000         1.1    2    1    1  1.7e-15  2.3e-15
Displacement field
Nonsymmetric matrix found.
Scales for dependent variables:
Auxiliary pressure (comp1.solid.lemm1.pw): 1
Displacement field (comp1.u): 2.8e-18
Orthonormal null-space function used.
Iter      SolEst     Damping    Stepsize #Res #Jac #Sol   LinErr   LinRes
   1         1.9   0.5000000         1.9    2    1    1  1.1e-13  1.5e-12
Spatial mesh displacement
```

```
Symmetric matrices found.
Scales for dependent variables:
Spatial mesh displacement (comp1.spatial.disp): 1.2e-09
Orthonormal null-space function used.
Iter      SolEst     Damping    Stepsize #Res #Jac #Sol   LinErr   LinRes
   1     7.3e-07   0.5000000     7.3e-07    2    1    1  1.4e-15  1.8e-15
Solution error estimates for segregated groups
1.4, 0.54, 0.95, 3.6e-07
Residual error estimates for segregated groups
7.8e+03, 8.2e+04, 2.2e+04, 0.13
Segregated solver iteration 2.
Merged variables
Scales for dependent variables:
Concentration (comp1.Cl): 1.4e+02
Concentration (comp1.K): 1.4e+02
Electric potential (comp1.V): 0.064
Iter      SolEst     Damping    Stepsize #Res #Jac #Sol   LinErr   LinRes
   1        0.78   0.3500000        0.78    6    4    4  8.2e-14  2.9e-15
   2        0.77   0.3500000        0.77    7    5    5  6.2e-14  2.8e-15
   3        0.76   0.3500000        0.76    8    6    6  9.6e-14  2.9e-15
Velocity u2, Pressure p
Scales for dependent variables:
Pressure (comp1.p): 3.6e+02
Velocity field (spatial frame) (comp1.u2): 0.00073
Iter      SolEst     Damping    Stepsize #Res #Jac #Sol   LinErr   LinRes
   1        0.36   0.5000000        0.36    4    2    2  2.2e-15  7.9e-15
Displacement field
Iter      SolEst     Damping    Stepsize #Res #Jac #Sol   LinErr   LinRes
   1        0.95   0.5000000        0.95    4    2    2  1.1e-13  1.5e-12
Spatial mesh displacement
Iter      SolEst     Damping    Stepsize #Res #Jac #Sol   LinErr   LinRes
   1     1.1e-06   0.5000000     1.1e-06    4    2    2  1.8e-15  1.8e-15
Solution error estimates for segregated groups
1.3, 0.18, 0.48, 5.5e-07
Residual error estimates for segregated groups
7.8e+02, 1.6e+04, 2.2e+04, 0.13
Segregated solver iteration 3.
Merged variables
Iter      SolEst     Damping    Stepsize #Res #Jac #Sol   LinErr   LinRes
   1        0.74   0.3500000        0.74   10    7    7    8e-14  2.8e-15
   2        0.73   0.3500000        0.73   11    8    8    8e-14  3.1e-15
   3        0.72   0.3500000        0.72   12    9    9  3.2e-14  2.9e-15
Velocity u2, Pressure p
Iter      SolEst     Damping    Stepsize #Res #Jac #Sol   LinErr   LinRes
   1        0.15   0.5000000        0.15    6    3    3    4e-15    8e-15
Displacement field
Iter      SolEst     Damping    Stepsize #Res #Jac #Sol   LinErr   LinRes
   1        0.52   0.5000000        0.52    6    3    3  6.3e-14  1.5e-12
Spatial mesh displacement
Iter      SolEst     Damping    Stepsize #Res #Jac #Sol   LinErr   LinRes
   1     1.1e-06   0.5000000     1.1e-06    6    3    3  1.6e-15  1.9e-15
Solution error estimates for segregated groups
1.2, 0.077, 0.26, 5.5e-07
Residual error estimates for segregated groups
2.1e+02, 9.1e+03, 1.6e+04, 0.14
Segregated solver iteration 4.
```

```
Merged variables
Iter      SolEst     Damping    Stepsize #Res #Jac #Sol   LinErr   LinRes
   1         0.7   0.3500000         0.7   14   10   10  8.4e-14  2.8e-15
   2        0.68   0.3500000        0.68   15   11   11  1.1e-13  2.8e-15
   3        0.59   0.3500000        0.59   16   12   12    1e-13  2.9e-15
Velocity u2, Pressure p
Iter      SolEst     Damping    Stepsize #Res #Jac #Sol   LinErr   LinRes
   1       0.072   0.5000000       0.072    8    4    4  3.7e-15  7.9e-15
Displacement field
Iter      SolEst     Damping    Stepsize #Res #Jac #Sol   LinErr   LinRes
   1        0.29   0.5000000        0.29    8    4    4  8.3e-14  1.5e-12
Spatial mesh displacement
Iter      SolEst     Damping    Stepsize #Res #Jac #Sol   LinErr   LinRes
   1     9.1e-07   0.5000000     9.1e-07    8    4    4  1.4e-15  1.9e-15
Solution error estimates for segregated groups
1, 0.036, 0.15, 4.5e-07
Residual error estimates for segregated groups
82, 4.8e+03, 1.1e+04, 0.16
Segregated solver iteration 5.
Merged variables
Iter      SolEst     Damping    Stepsize #Res #Jac #Sol   LinErr   LinRes
   1         0.4   0.3500000         0.4   18   13   13  1.8e-13  3.1e-15
   2        0.27   0.3500000        0.27   19   14   14  6.2e-14  2.9e-15
   3        0.18   0.3500000        0.18   20   15   15  1.3e-13  2.9e-15
Velocity u2, Pressure p
Iter      SolEst     Damping    Stepsize #Res #Jac #Sol   LinErr   LinRes
   1       0.035   0.5000000       0.035   10    5    5  4.8e-15  8.2e-15
Displacement field
Iter      SolEst     Damping    Stepsize #Res #Jac #Sol   LinErr   LinRes
   1        0.17   0.5000000        0.17   10    5    5  1.2e-13  1.5e-12
Spatial mesh displacement
Iter      SolEst     Damping    Stepsize #Res #Jac #Sol   LinErr   LinRes
   1     6.8e-07   0.5000000     6.8e-07   10    5    5  1.1e-15  1.9e-15
Solution error estimates for segregated groups
0.31, 0.017, 0.084, 3.4e-07
Residual error estimates for segregated groups
32, 2.5e+03, 6.8e+03, 0.15
Segregated solver iteration 6.
Merged variables
Iter      SolEst     Damping    Stepsize #Res #Jac #Sol   LinErr   LinRes
   1        0.12   0.3500000        0.12   22   16   16  6.2e-14  2.9e-15
   2       0.077   0.3500000       0.077   23   17   17  6.4e-14  2.8e-15
   3        0.05   0.3500000        0.05   24   18   18  1.6e-13  2.9e-15
Velocity u2, Pressure p
Iter      SolEst     Damping    Stepsize #Res #Jac #Sol   LinErr   LinRes
   1       0.017   0.5000000       0.017   12    6    6  2.5e-15  7.8e-15
Displacement field
Iter      SolEst     Damping    Stepsize #Res #Jac #Sol   LinErr   LinRes
   1       0.095   0.5000000       0.095   12    6    6  6.5e-14  1.5e-12
Spatial mesh displacement
Iter      SolEst     Damping    Stepsize #Res #Jac #Sol   LinErr   LinRes
   1     4.8e-07   0.5000000     4.8e-07   12    6    6  1.3e-15  1.8e-15
Solution error estimates for segregated groups
0.086, 0.0085, 0.048, 2.4e-07
Residual error estimates for segregated groups
12, 1.3e+03, 4.2e+03, 0.15
```

```
Segregated solver iteration 7.
Merged variables
Iter      SolEst     Damping    Stepsize #Res #Jac #Sol   LinErr   LinRes
   1       0.033   0.3500000       0.033   26   19   19  1.2e-13  3.1e-15
   2       0.021   0.3500000       0.021   27   20   20  1.4e-13  2.9e-15
   3       0.014   0.3500000       0.014   28   21   21  1.3e-13    3e-15
Velocity u2, Pressure p
Iter      SolEst     Damping    Stepsize #Res #Jac #Sol   LinErr   LinRes
   1      0.0085   0.5000000      0.0085   14    7    7  6.3e-15  7.9e-15
Displacement field
Scales for dependent variables:
Auxiliary pressure (comp1.solid.lemm1.pw): 0.36
Displacement field (comp1.u): 2.8e-16
Iter      SolEst     Damping    Stepsize #Res #Jac #Sol   LinErr   LinRes
   1       0.054   0.5000000       0.054   14    7    7  1.7e-13  1.2e-12
Spatial mesh displacement
Iter      SolEst     Damping    Stepsize #Res #Jac #Sol   LinErr   LinRes
   1     3.2e-07   0.5000000     3.2e-07   14    7    7  1.6e-15  1.8e-15
Solution error estimates for segregated groups
0.024, 0.0042, 0.027, 1.6e-07
Residual error estimates for segregated groups
4.1, 6.4e+02, 2.6e+03, 0.15
Segregated solver iteration 8.
Merged variables
Iter      SolEst     Damping    Stepsize #Res #Jac #Sol   LinErr   LinRes
   1       0.009   0.3500000       0.009   30   22   22    1e-13  2.8e-15
   2      0.0059   0.3500000      0.0059   31   23   23    1e-13  2.9e-15
   3      0.0038   0.3500000      0.0038   32   24   24    1e-13  2.8e-15
Velocity u2, Pressure p
Iter      SolEst     Damping    Stepsize #Res #Jac #Sol   LinErr   LinRes
   1      0.0042   0.5000000      0.0042   16    8    8  2.7e-15  7.9e-15
Displacement field
Iter      SolEst     Damping    Stepsize #Res #Jac #Sol   LinErr   LinRes
   1        0.03   0.5000000        0.03   16    8    8  1.1e-13  1.2e-12
Spatial mesh displacement
Iter      SolEst     Damping    Stepsize #Res #Jac #Sol   LinErr   LinRes
   1       2e-07   0.5000000       2e-07   16    8    8  1.6e-15  1.9e-15
Solution error estimates for segregated groups
0.0065, 0.0021, 0.015, 1e-07
Residual error estimates for segregated groups
1.4, 3.2e+02, 1.8e+03, 0.15
Segregated solver iteration 9.
Merged variables
Iter      SolEst     Damping    Stepsize #Res #Jac #Sol   LinErr   LinRes
   1      0.0025   0.3500000      0.0025   34   25   25  6.3e-14    3e-15
   2      0.0016   0.3500000      0.0016   35   26   26  8.4e-14    3e-15
   3       0.001   0.3500000       0.001   36   27   27  1.1e-13  2.9e-15
Velocity u2, Pressure p
Iter      SolEst     Damping    Stepsize #Res #Jac #Sol   LinErr   LinRes
   1      0.0021   0.5000000      0.0021   18    9    9  2.8e-15  7.8e-15
Displacement field
Iter      SolEst     Damping    Stepsize #Res #Jac #Sol   LinErr   LinRes
   1       0.017   0.5000000       0.017   18    9    9  1.2e-13  1.2e-12
Spatial mesh displacement
Iter      SolEst     Damping    Stepsize #Res #Jac #Sol   LinErr   LinRes
   1     1.3e-07   0.5000000     1.3e-07   18    9    9  1.4e-15  1.8e-15
```

```
Solution error estimates for segregated groups
0.0018, 0.0011, 0.0085, 6.4e-08
Residual error estimates for segregated groups
0.44, 1.6e+02, 1.4e+03, 0.15
Segregated solver iteration 10.
Merged variables
Iter      SolEst     Damping    Stepsize #Res #Jac #Sol   LinErr   LinRes
   1     0.00068   0.3500000     0.00068   38   28   28  6.9e-14  2.9e-15
   2     0.00044   0.3500000     0.00044   39   29   29  3.9e-14  3.3e-15
   3     0.00029   0.3500000     0.00029   40   30   30    1e-13  3.2e-15
Velocity u2, Pressure p
Iter      SolEst     Damping    Stepsize #Res #Jac #Sol   LinErr   LinRes
   1      0.0011   0.5000000      0.0011   20   10   10    3e-15  7.8e-15
Displacement field
Iter      SolEst     Damping    Stepsize #Res #Jac #Sol   LinErr   LinRes
   1      0.0094   0.5000000      0.0094   20   10   10  1.3e-13  1.2e-12
Spatial mesh displacement
Iter      SolEst     Damping    Stepsize #Res #Jac #Sol   LinErr   LinRes
   1     7.8e-08   0.5000000     7.8e-08   20   10   10  1.5e-15  1.8e-15
Solution error estimates for segregated groups
0.00049, 0.00053, 0.0047, 3.9e-08
Residual error estimates for segregated groups
0.14, 80, 1.2e+03, 0.14
Segregated solver iteration 11.
Merged variables
Iter      SolEst     Damping    Stepsize #Res #Jac #Sol   LinErr   LinRes
   1     0.00019   0.3500000     0.00019   42   31   31  1.2e-13  2.8e-15
   2     0.00012   0.3500000     0.00012   43   32   32  6.4e-14  2.8e-15
   3     7.9e-05   0.3500000     7.9e-05   44   33   33  3.1e-14  2.8e-15
Velocity u2, Pressure p
Iter      SolEst     Damping    Stepsize #Res #Jac #Sol   LinErr   LinRes
   1     0.00053   0.5000000     0.00053   22   11   11  2.5e-15  7.8e-15
Displacement field
Iter      SolEst     Damping    Stepsize #Res #Jac #Sol   LinErr   LinRes
   1      0.0051   0.5000000      0.0051   22   11   11  1.1e-13  1.2e-12
Spatial mesh displacement
Iter      SolEst     Damping    Stepsize #Res #Jac #Sol   LinErr   LinRes
   1     4.7e-08   0.5000000     4.7e-08   22   11   11  1.5e-15  1.8e-15
Solution error estimates for segregated groups
0.00014, 0.00026, 0.0026, 2.3e-08
Residual error estimates for segregated groups
0.043, 40, 1.2e+03, 0.16
Segregated solver iteration 12.
Merged variables
Iter      SolEst     Damping    Stepsize #Res #Jac #Sol   LinErr   LinRes
   1     5.1e-05   0.3500000     5.1e-05   46   34   34  1.2e-13  2.9e-15
   2     3.3e-05   0.3500000     3.3e-05   47   35   35  6.6e-14    3e-15
   3     2.2e-05   0.3500000     2.2e-05   48   36   36  9.5e-14  2.8e-15
Velocity u2, Pressure p
Iter      SolEst     Damping    Stepsize #Res #Jac #Sol   LinErr   LinRes
   1     0.00026   0.5000000     0.00026   24   12   12  2.4e-15  7.9e-15
Displacement field
Iter      SolEst     Damping    Stepsize #Res #Jac #Sol   LinErr   LinRes
   1      0.0028   0.5000000      0.0028   24   12   12  1.4e-13  1.2e-12
Spatial mesh displacement
Iter      SolEst     Damping    Stepsize #Res #Jac #Sol   LinErr   LinRes
```

```
   1     2.8e-08   0.5000000     2.8e-08   24    12    12  1.4e-15  1.8e-15
Solution error estimates for segregated groups
3.7e-05, 0.00013, 0.0014, 1.4e-08
Residual error estimates for segregated groups
0.013, 20, 1.2e+03, 0.15
Segregated solver iteration 13.
Merged variables
Iter      SolEst     Damping    Stepsize #Res #Jac #Sol   LinErr   LinRes
   1     1.4e-05   0.3500000     1.4e-05   50    37    37    3e-14  2.8e-15
   2     9.2e-06   0.3500000     9.2e-06   51    38    38  7.8e-14  3.2e-15
   3       6e-06   0.3500000       6e-06   52    39    39  1.1e-13    3e-15
Velocity u2, Pressure p
Iter      SolEst     Damping    Stepsize #Res #Jac #Sol   LinErr   LinRes
   1     0.00013   0.5000000     0.00013   26    13    13  4.3e-15  7.9e-15
Displacement field
Iter      SolEst     Damping    Stepsize #Res #Jac #Sol   LinErr   LinRes
   1      0.0015   0.5000000      0.0015   26    13    13  1.5e-13  1.2e-12
Spatial mesh displacement
Iter      SolEst     Damping    Stepsize #Res #Jac #Sol   LinErr   LinRes
   1     1.6e-08   0.5000000     1.6e-08   26    13    13  1.8e-15  1.9e-15
Solution error estimates for segregated groups
1e-05, 6.6e-05, 0.00076, 8.1e-09
Residual error estimates for segregated groups
0.004, 10, 1.2e+03, 0.14
Solution time: 154 s. (2 minutes, 34 seconds)
Physical memory: 4.72 GB
Virtual memory: 26.01 GB
Ended at Jan 31, 2025, 8:06:49 PM.
----- Stationary Solver 1 in study1/Approach Curve Solver (sol1) -------------->
```

d=2145 (su73)

GENERAL

Description	Value
Solution	d=2145

LOG

```
<---- Compile Equations: Stationary in study1/Approach Curve Solver (sol1) -----
Started at Jan 31, 2025, 8:06:49 PM.
Parameter d = 2.1450000000000002E-6 (m).
Geometry shape function: Linear serendipity
Number of vertex elements: 18
Number of boundary elements: 6139
Number of elements: 154691
Minimum element quality: 0.4845
Time: 8 s.
Physical memory: 2.97 GB
Virtual memory: 15.21 GB
Ended at Jan 31, 2025, 8:06:58 PM.
----- Compile Equations: Stationary in study1/Approach Curve Solver (sol1) ---->

<---- Dependent Variables 1 in study1/Approach Curve Solver (sol1) -------------
Started at Jan 31, 2025, 8:06:58 PM.
Solution time: 0 s.
Physical memory: 2.97 GB
Virtual memory: 15.21 GB
Ended at Jan 31, 2025, 8:06:58 PM.
----- Dependent Variables 1 in study1/Approach Curve Solver (sol1) ------------>

<---- Stationary Solver 1 in study1/Approach Curve Solver (sol1) ---------------
Started at Jan 31, 2025, 8:06:58 PM.
Segregated solver
Segregated Step 11 does not have any components solved for.
Number of degrees of freedom solved for: 785363 (plus 11701 internal DOFs).
Segregated solver iteration 1.
Merged variables
Nonsymmetric matrix found.
Scales for dependent variables:
Concentration (comp1.Cl): 1.4e+02
Concentration (comp1.K): 1.4e+02
Electric potential (comp1.V): 1.9e+02
Orthonormal null-space function used.
Iter      SolEst     Damping    Stepsize #Res #Jac #Sol   LinErr   LinRes
   1        0.83   0.3500000        0.83    2    1    1  5.4e-11  2.7e-15
   2        0.81   0.3500000        0.81    3    2    2  2.6e-11  2.7e-15
   3         0.8   0.3500000         0.8    4    3    3  2.7e-11  2.7e-15
Velocity u2, Pressure p
Nonsymmetric matrix found.
Scales for dependent variables:
Pressure (comp1.p): 1
Velocity field (spatial frame) (comp1.u2): 0.00018
Orthonormal null-space function used.
Iter      SolEst     Damping    Stepsize #Res #Jac #Sol   LinErr   LinRes
   1         1.1   0.5000000         1.1    2    1    1  1.7e-15  2.2e-15
Displacement field
Nonsymmetric matrix found.
Scales for dependent variables:
Auxiliary pressure (comp1.solid.lemm1.pw): 1
Displacement field (comp1.u): 2.8e-18
Orthonormal null-space function used.
Iter      SolEst     Damping    Stepsize #Res #Jac #Sol   LinErr   LinRes
   1         1.9   0.5000000         1.9    2    1    1  1.6e-13  1.5e-12
Spatial mesh displacement
```

```
Symmetric matrices found.
Scales for dependent variables:
Spatial mesh displacement (comp1.spatial.disp): 1.2e-09
Orthonormal null-space function used.
Iter      SolEst     Damping    Stepsize #Res #Jac #Sol   LinErr   LinRes
   1     7.3e-07   0.5000000     7.3e-07    2    1    1  1.6e-15  1.8e-15
Solution error estimates for segregated groups
1.4, 0.54, 0.95, 3.7e-07
Residual error estimates for segregated groups
7.8e+03, 8.2e+04, 2.2e+04, 0.12
Segregated solver iteration 2.
Merged variables
Scales for dependent variables:
Concentration (comp1.Cl): 1.4e+02
Concentration (comp1.K): 1.4e+02
Electric potential (comp1.V): 0.064
Iter      SolEst     Damping    Stepsize #Res #Jac #Sol   LinErr   LinRes
   1        0.78   0.3500000        0.78    6    4    4  5.3e-14  3.4e-15
   2        0.77   0.3500000        0.77    7    5    5  1.3e-13  3.3e-15
   3        0.76   0.3500000        0.76    8    6    6    7e-14  5.1e-15
Velocity u2, Pressure p
Scales for dependent variables:
Pressure (comp1.p): 3.6e+02
Velocity field (spatial frame) (comp1.u2): 0.00073
Iter      SolEst     Damping    Stepsize #Res #Jac #Sol   LinErr   LinRes
   1        0.36   0.5000000        0.36    4    2    2  2.5e-15  7.9e-15
Displacement field
Iter      SolEst     Damping    Stepsize #Res #Jac #Sol   LinErr   LinRes
   1        0.95   0.5000000        0.95    4    2    2  5.6e-14  1.5e-12
Spatial mesh displacement
Iter      SolEst     Damping    Stepsize #Res #Jac #Sol   LinErr   LinRes
   1     1.1e-06   0.5000000     1.1e-06    4    2    2  1.6e-15  1.8e-15
Solution error estimates for segregated groups
1.3, 0.18, 0.48, 5.5e-07
Residual error estimates for segregated groups
7.8e+02, 1.7e+04, 2.2e+04, 0.15
Segregated solver iteration 3.
Merged variables
Iter      SolEst     Damping    Stepsize #Res #Jac #Sol   LinErr   LinRes
   1        0.74   0.3500000        0.74   10    7    7  8.9e-14  3.2e-15
   2        0.73   0.3500000        0.73   11    8    8  5.8e-14  4.1e-15
   3        0.72   0.3500000        0.72   12    9    9    5e-14    4e-15
Velocity u2, Pressure p
Iter      SolEst     Damping    Stepsize #Res #Jac #Sol   LinErr   LinRes
   1        0.15   0.5000000        0.15    6    3    3  2.5e-15    8e-15
Displacement field
Iter      SolEst     Damping    Stepsize #Res #Jac #Sol   LinErr   LinRes
   1        0.52   0.5000000        0.52    6    3    3    5e-14  1.5e-12
Spatial mesh displacement
Iter      SolEst     Damping    Stepsize #Res #Jac #Sol   LinErr   LinRes
   1     1.1e-06   0.5000000     1.1e-06    6    3    3  1.5e-15  1.8e-15
Solution error estimates for segregated groups
1.2, 0.077, 0.26, 5.5e-07
Residual error estimates for segregated groups
2.2e+02, 9.7e+03, 1.6e+04, 0.15
Segregated solver iteration 4.
```

```
Merged variables
Iter      SolEst     Damping    Stepsize #Res #Jac #Sol   LinErr   LinRes
   1         0.7   0.3500000         0.7   14   10   10  1.4e-13  3.6e-15
   2        0.68   0.3500000        0.68   15   11   11  4.7e-14  3.4e-15
   3        0.59   0.3500000        0.59   16   12   12  3.6e-14  4.6e-15
Velocity u2, Pressure p
Iter      SolEst     Damping    Stepsize #Res #Jac #Sol   LinErr   LinRes
   1       0.072   0.5000000       0.072    8    4    4  5.4e-15  7.8e-15
Displacement field
Iter      SolEst     Damping    Stepsize #Res #Jac #Sol   LinErr   LinRes
   1        0.29   0.5000000        0.29    8    4    4    7e-14  1.5e-12
Spatial mesh displacement
Iter      SolEst     Damping    Stepsize #Res #Jac #Sol   LinErr   LinRes
   1     9.1e-07   0.5000000     9.1e-07    8    4    4  1.8e-15  1.8e-15
Solution error estimates for segregated groups
1, 0.036, 0.15, 4.6e-07
Residual error estimates for segregated groups
82, 5.1e+03, 1.1e+04, 0.15
Segregated solver iteration 5.
Merged variables
Iter      SolEst     Damping    Stepsize #Res #Jac #Sol   LinErr   LinRes
   1         0.4   0.3500000         0.4   18   13   13  8.4e-14    3e-15
   2        0.27   0.3500000        0.27   19   14   14    1e-13    3e-15
   3        0.18   0.3500000        0.18   20   15   15  9.4e-14  3.7e-15
Velocity u2, Pressure p
Iter      SolEst     Damping    Stepsize #Res #Jac #Sol   LinErr   LinRes
   1       0.035   0.5000000       0.035   10    5    5  2.5e-15  7.9e-15
Displacement field
Iter      SolEst     Damping    Stepsize #Res #Jac #Sol   LinErr   LinRes
   1        0.17   0.5000000        0.17   10    5    5    1e-13  1.5e-12
Spatial mesh displacement
Iter      SolEst     Damping    Stepsize #Res #Jac #Sol   LinErr   LinRes
   1     6.9e-07   0.5000000     6.9e-07   10    5    5  1.6e-15  1.8e-15
Solution error estimates for segregated groups
0.31, 0.017, 0.084, 3.4e-07
Residual error estimates for segregated groups
32, 2.6e+03, 6.8e+03, 0.15
Segregated solver iteration 6.
Merged variables
Iter      SolEst     Damping    Stepsize #Res #Jac #Sol   LinErr   LinRes
   1        0.12   0.3500000        0.12   22   16   16  1.2e-13  2.9e-15
   2       0.077   0.3500000       0.077   23   17   17  1.1e-13  3.9e-15
   3        0.05   0.3500000        0.05   24   18   18    1e-13  3.2e-15
Velocity u2, Pressure p
Iter      SolEst     Damping    Stepsize #Res #Jac #Sol   LinErr   LinRes
   1       0.017   0.5000000       0.017   12    6    6  3.3e-15    8e-15
Displacement field
Iter      SolEst     Damping    Stepsize #Res #Jac #Sol   LinErr   LinRes
   1       0.095   0.5000000       0.095   12    6    6    8e-14  1.5e-12
Spatial mesh displacement
Iter      SolEst     Damping    Stepsize #Res #Jac #Sol   LinErr   LinRes
   1     4.8e-07   0.5000000     4.8e-07   12    6    6  2.2e-15  1.8e-15
Solution error estimates for segregated groups
0.086, 0.0085, 0.048, 2.4e-07
Residual error estimates for segregated groups
12, 1.3e+03, 4.2e+03, 0.14
```

```
Segregated solver iteration 7.
Merged variables
Iter      SolEst     Damping    Stepsize #Res #Jac #Sol   LinErr   LinRes
   1       0.033   0.3500000       0.033   26   19   19  5.6e-14  3.8e-15
   2       0.021   0.3500000       0.021   27   20   20  1.5e-13  3.3e-15
   3       0.014   0.3500000       0.014   28   21   21  8.4e-14    4e-15
Velocity u2, Pressure p
Iter      SolEst     Damping    Stepsize #Res #Jac #Sol   LinErr   LinRes
   1      0.0085   0.5000000      0.0085   14    7    7  2.3e-15  7.8e-15
Displacement field
Scales for dependent variables:
Auxiliary pressure (comp1.solid.lemm1.pw): 0.36
Displacement field (comp1.u): 2.9e-16
Iter      SolEst     Damping    Stepsize #Res #Jac #Sol   LinErr   LinRes
   1       0.054   0.5000000       0.054   14    7    7  1.7e-13  1.2e-12
Spatial mesh displacement
Iter      SolEst     Damping    Stepsize #Res #Jac #Sol   LinErr   LinRes
   1     3.2e-07   0.5000000     3.2e-07   14    7    7  1.6e-15  1.8e-15
Solution error estimates for segregated groups
0.024, 0.0042, 0.027, 1.6e-07
Residual error estimates for segregated groups
4.1, 6.8e+02, 2.6e+03, 0.15
Segregated solver iteration 8.
Merged variables
Iter      SolEst     Damping    Stepsize #Res #Jac #Sol   LinErr   LinRes
   1       0.009   0.3500000       0.009   30   22   22  1.3e-13  3.4e-15
   2      0.0059   0.3500000      0.0059   31   23   23  8.7e-14    3e-15
   3      0.0038   0.3500000      0.0038   32   24   24  7.5e-14    3e-15
Velocity u2, Pressure p
Iter      SolEst     Damping    Stepsize #Res #Jac #Sol   LinErr   LinRes
   1      0.0042   0.5000000      0.0042   16    8    8    2e-15  8.1e-15
Displacement field
Iter      SolEst     Damping    Stepsize #Res #Jac #Sol   LinErr   LinRes
   1        0.03   0.5000000        0.03   16    8    8  1.2e-13  1.2e-12
Spatial mesh displacement
Iter      SolEst     Damping    Stepsize #Res #Jac #Sol   LinErr   LinRes
   1     2.1e-07   0.5000000     2.1e-07   16    8    8  2.1e-15  1.8e-15
Solution error estimates for segregated groups
0.0066, 0.0021, 0.015, 1e-07
Residual error estimates for segregated groups
1.4, 3.4e+02, 1.8e+03, 0.14
Segregated solver iteration 9.
Merged variables
Iter      SolEst     Damping    Stepsize #Res #Jac #Sol   LinErr   LinRes
   1      0.0025   0.3500000      0.0025   34   25   25  5.1e-14  3.4e-15
   2      0.0016   0.3500000      0.0016   35   26   26  8.4e-14  7.2e-15
   3       0.001   0.3500000       0.001   36   27   27  7.6e-14  3.8e-15
Velocity u2, Pressure p
Iter      SolEst     Damping    Stepsize #Res #Jac #Sol   LinErr   LinRes
   1      0.0021   0.5000000      0.0021   18    9    9  2.6e-15    8e-15
Displacement field
Iter      SolEst     Damping    Stepsize #Res #Jac #Sol   LinErr   LinRes
   1       0.017   0.5000000       0.017   18    9    9  1.3e-13  1.2e-12
Spatial mesh displacement
Iter      SolEst     Damping    Stepsize #Res #Jac #Sol   LinErr   LinRes
   1     1.3e-07   0.5000000     1.3e-07   18    9    9  1.3e-15  1.8e-15
```

```
Solution error estimates for segregated groups
0.0018, 0.0011, 0.0085, 6.4e-08
Residual error estimates for segregated groups
0.44, 1.7e+02, 1.4e+03, 0.15
Segregated solver iteration 10.
Merged variables
Iter      SolEst     Damping    Stepsize #Res #Jac #Sol   LinErr   LinRes
   1     0.00068   0.3500000     0.00068   38   28   28  6.4e-14  5.5e-15
   2     0.00044   0.3500000     0.00044   39   29   29  9.4e-14  4.8e-15
   3     0.00029   0.3500000     0.00029   40   30   30  6.9e-14    4e-15
Velocity u2, Pressure p
Iter      SolEst     Damping    Stepsize #Res #Jac #Sol   LinErr   LinRes
   1      0.0011   0.5000000      0.0011   20   10   10  4.4e-15  7.9e-15
Displacement field
Iter      SolEst     Damping    Stepsize #Res #Jac #Sol   LinErr   LinRes
   1      0.0094   0.5000000      0.0094   20   10   10  1.3e-13  1.2e-12
Spatial mesh displacement
Iter      SolEst     Damping    Stepsize #Res #Jac #Sol   LinErr   LinRes
   1     7.9e-08   0.5000000     7.9e-08   20   10   10  1.6e-15  1.8e-15
Solution error estimates for segregated groups
0.00049, 0.00053, 0.0047, 3.9e-08
Residual error estimates for segregated groups
0.14, 85, 1.2e+03, 0.14
Segregated solver iteration 11.
Merged variables
Iter      SolEst     Damping    Stepsize #Res #Jac #Sol   LinErr   LinRes
   1     0.00019   0.3500000     0.00019   42   31   31  7.9e-14  5.3e-15
   2     0.00012   0.3500000     0.00012   43   32   32  1.2e-13  3.1e-15
   3     7.9e-05   0.3500000     7.9e-05   44   33   33  1.6e-13  3.3e-15
Velocity u2, Pressure p
Iter      SolEst     Damping    Stepsize #Res #Jac #Sol   LinErr   LinRes
   1     0.00053   0.5000000     0.00053   22   11   11  2.2e-15  7.8e-15
Displacement field
Iter      SolEst     Damping    Stepsize #Res #Jac #Sol   LinErr   LinRes
   1      0.0051   0.5000000      0.0051   22   11   11  1.3e-13  1.2e-12
Spatial mesh displacement
Iter      SolEst     Damping    Stepsize #Res #Jac #Sol   LinErr   LinRes
   1     4.7e-08   0.5000000     4.7e-08   22   11   11  2.1e-15  1.9e-15
Solution error estimates for segregated groups
0.00014, 0.00026, 0.0026, 2.4e-08
Residual error estimates for segregated groups
0.043, 42, 1.2e+03, 0.14
Segregated solver iteration 12.
Merged variables
Iter      SolEst     Damping    Stepsize #Res #Jac #Sol   LinErr   LinRes
   1     5.1e-05   0.3500000     5.1e-05   46   34   34  8.6e-14  3.9e-15
   2     3.3e-05   0.3500000     3.3e-05   47   35   35  4.3e-14  3.1e-15
   3     2.2e-05   0.3500000     2.2e-05   48   36   36    6e-14  4.1e-15
Velocity u2, Pressure p
Iter      SolEst     Damping    Stepsize #Res #Jac #Sol   LinErr   LinRes
   1     0.00026   0.5000000     0.00026   24   12   12  7.3e-15  7.9e-15
Displacement field
Iter      SolEst     Damping    Stepsize #Res #Jac #Sol   LinErr   LinRes
   1      0.0028   0.5000000      0.0028   24   12   12  1.2e-13  1.2e-12
Spatial mesh displacement
Iter      SolEst     Damping    Stepsize #Res #Jac #Sol   LinErr   LinRes
```

```
   1     2.8e-08   0.5000000     2.8e-08   24   12   12  1.6e-15  1.8e-15
Solution error estimates for segregated groups
3.7e-05, 0.00013, 0.0014, 1.4e-08
Residual error estimates for segregated groups
0.013, 21, 1.2e+03, 0.15
Segregated solver iteration 13.
Merged variables
Iter      SolEst      Damping    Stepsize #Res #Jac #Sol   LinErr   LinRes
   1     1.4e-05   0.3500000     1.4e-05   50   37   37  6.8e-14  3.4e-15
   2     9.2e-06   0.3500000     9.2e-06   51   38   38  4.5e-14  5.5e-15
   3       6e-06   0.3500000       6e-06   52   39   39  8.8e-14    3e-15
Velocity u2, Pressure p
Iter      SolEst      Damping    Stepsize #Res #Jac #Sol   LinErr   LinRes
   1     0.00013   0.5000000     0.00013   26   13   13  3.3e-15  7.9e-15
Displacement field
Iter      SolEst      Damping    Stepsize #Res #Jac #Sol   LinErr   LinRes
   1      0.0015   0.5000000      0.0015   26   13   13  9.9e-14  1.2e-12
Spatial mesh displacement
Iter      SolEst      Damping    Stepsize #Res #Jac #Sol   LinErr   LinRes
   1     1.6e-08   0.5000000     1.6e-08   26   13   13  1.6e-15  1.8e-15
Solution error estimates for segregated groups
1e-05, 6.6e-05, 0.00076, 8.1e-09
Residual error estimates for segregated groups
0.004, 11, 1.2e+03, 0.15
Solution time: 155 s. (2 minutes, 35 seconds)
Physical memory: 4.74 GB
Virtual memory: 25.55 GB
Ended at Jan 31, 2025, 8:09:33 PM.
----- Stationary Solver 1 in study1/Approach Curve Solver (sol1) -------------->
```

d=2140 (su74)

GENERAL

Description	Value
Solution	d=2140

LOG

```
<---- Compile Equations: Stationary in study1/Approach Curve Solver (sol1) -----
Started at Jan 31, 2025, 8:09:33 PM.
Parameter d = 2.1400000000000003E-6 (m).
Geometry shape function: Linear serendipity
Number of vertex elements: 18
Number of boundary elements: 6136
Number of elements: 154694
Minimum element quality: 0.4672
Time: 8 s.
Physical memory: 2.91 GB
Virtual memory: 15.14 GB
Ended at Jan 31, 2025, 8:09:41 PM.
----- Compile Equations: Stationary in study1/Approach Curve Solver (sol1) ---->

<---- Dependent Variables 1 in study1/Approach Curve Solver (sol1) -------------
Started at Jan 31, 2025, 8:09:41 PM.
Solution time: 0 s.
Physical memory: 2.91 GB
Virtual memory: 15.14 GB
Ended at Jan 31, 2025, 8:09:41 PM.
----- Dependent Variables 1 in study1/Approach Curve Solver (sol1) ------------>

<---- Stationary Solver 1 in study1/Approach Curve Solver (sol1) ---------------
Started at Jan 31, 2025, 8:09:42 PM.
Segregated solver
Segregated Step 11 does not have any components solved for.
Number of degrees of freedom solved for: 785412 (plus 11695 internal DOFs).
Segregated solver iteration 1.
Merged variables
Nonsymmetric matrix found.
Scales for dependent variables:
Concentration (comp1.Cl): 1.4e+02
Concentration (comp1.K): 1.4e+02
Electric potential (comp1.V): 1.9e+02
Orthonormal null-space function used.
Iter      SolEst     Damping    Stepsize #Res #Jac #Sol   LinErr   LinRes
   1        0.83   0.3500000        0.83    2    1    1  1.1e-11  2.8e-15
   2        0.81   0.3500000        0.81    3    2    2  4.7e-11  2.7e-15
   3         0.8   0.3500000         0.8    4    3    3  2.6e-11  2.7e-15
Velocity u2, Pressure p
Nonsymmetric matrix found.
Scales for dependent variables:
Pressure (comp1.p): 1
Velocity field (spatial frame) (comp1.u2): 0.00018
Orthonormal null-space function used.
Iter      SolEst     Damping    Stepsize #Res #Jac #Sol   LinErr   LinRes
   1         1.1   0.5000000         1.1    2    1    1  1.4e-15  2.2e-15
Displacement field
Nonsymmetric matrix found.
Scales for dependent variables:
Auxiliary pressure (comp1.solid.lemm1.pw): 1
Displacement field (comp1.u): 2.8e-18
Orthonormal null-space function used.
Iter      SolEst     Damping    Stepsize #Res #Jac #Sol   LinErr   LinRes
   1         1.9   0.5000000         1.9    2    1    1  5.2e-14  1.5e-12
Spatial mesh displacement
```

```
Symmetric matrices found.
Scales for dependent variables:
Spatial mesh displacement (comp1.spatial.disp): 1.2e-09
Orthonormal null-space function used.
Iter      SolEst     Damping    Stepsize #Res #Jac #Sol   LinErr   LinRes
   1     7.3e-07   0.5000000     7.3e-07    2    1    1  1.3e-15  1.8e-15
Solution error estimates for segregated groups
1.4, 0.54, 0.95, 3.7e-07
Residual error estimates for segregated groups
7.8e+03, 8.2e+04, 2.2e+04, 0.13
Segregated solver iteration 2.
Merged variables
Scales for dependent variables:
Concentration (comp1.Cl): 1.4e+02
Concentration (comp1.K): 1.4e+02
Electric potential (comp1.V): 0.064
Iter      SolEst     Damping    Stepsize #Res #Jac #Sol   LinErr   LinRes
   1        0.78   0.3500000        0.78    6    4    4    5e-14    3e-15
   2        0.77   0.3500000        0.77    7    5    5    7e-14  3.1e-15
   3        0.76   0.3500000        0.76    8    6    6    7e-14  2.8e-15
Velocity u2, Pressure p
Scales for dependent variables:
Pressure (comp1.p): 3.6e+02
Velocity field (spatial frame) (comp1.u2): 0.00073
Iter      SolEst     Damping    Stepsize #Res #Jac #Sol   LinErr   LinRes
   1        0.36   0.5000000        0.36    4    2    2  2.5e-15  7.9e-15
Displacement field
Iter      SolEst     Damping    Stepsize #Res #Jac #Sol   LinErr   LinRes
   1        0.95   0.5000000        0.95    4    2    2  7.4e-14  1.5e-12
Spatial mesh displacement
Iter      SolEst     Damping    Stepsize #Res #Jac #Sol   LinErr   LinRes
   1     1.1e-06   0.5000000     1.1e-06    4    2    2  1.8e-15  1.9e-15
Solution error estimates for segregated groups
1.3, 0.18, 0.48, 5.5e-07
Residual error estimates for segregated groups
7.7e+02, 1.3e+04, 2.2e+04, 0.16
Segregated solver iteration 3.
Merged variables
Iter      SolEst     Damping    Stepsize #Res #Jac #Sol   LinErr   LinRes
   1        0.74   0.3500000        0.74   10    7    7  4.6e-14  2.7e-15
   2        0.73   0.3500000        0.73   11    8    8  1.1e-13  2.8e-15
   3        0.72   0.3500000        0.72   12    9    9  9.4e-14  2.8e-15
Velocity u2, Pressure p
Iter      SolEst     Damping    Stepsize #Res #Jac #Sol   LinErr   LinRes
   1        0.15   0.5000000        0.15    6    3    3  2.6e-15  7.9e-15
Displacement field
Iter      SolEst     Damping    Stepsize #Res #Jac #Sol   LinErr   LinRes
   1        0.52   0.5000000        0.52    6    3    3  1.5e-13  1.5e-12
Spatial mesh displacement
Iter      SolEst     Damping    Stepsize #Res #Jac #Sol   LinErr   LinRes
   1     1.1e-06   0.5000000     1.1e-06    6    3    3  1.4e-15  1.8e-15
Solution error estimates for segregated groups
1.2, 0.077, 0.26, 5.5e-07
Residual error estimates for segregated groups
2.2e+02, 7.5e+03, 1.6e+04, 0.15
Segregated solver iteration 4.
```

```
Merged variables
Iter      SolEst     Damping    Stepsize #Res #Jac #Sol   LinErr   LinRes
   1         0.7   0.3500000         0.7   14   10   10  8.2e-14  3.3e-15
   2        0.68   0.3500000        0.68   15   11   11  5.3e-14    3e-15
   3        0.59   0.3500000        0.59   16   12   12    1e-13  3.5e-15
Velocity u2, Pressure p
Iter      SolEst     Damping    Stepsize #Res #Jac #Sol   LinErr   LinRes
   1       0.072   0.5000000       0.072    8    4    4  2.5e-15  7.8e-15
Displacement field
Iter      SolEst     Damping    Stepsize #Res #Jac #Sol   LinErr   LinRes
   1        0.29   0.5000000        0.29    8    4    4  3.5e-14  1.5e-12
Spatial mesh displacement
Iter      SolEst     Damping    Stepsize #Res #Jac #Sol   LinErr   LinRes
   1     9.2e-07   0.5000000     9.2e-07    8    4    4  1.8e-15  1.8e-15
Solution error estimates for segregated groups
1, 0.036, 0.15, 4.6e-07
Residual error estimates for segregated groups
83, 4e+03, 1.1e+04, 0.15
Segregated solver iteration 5.
Merged variables
Iter      SolEst     Damping    Stepsize #Res #Jac #Sol   LinErr   LinRes
   1         0.4   0.3500000         0.4   18   13   13  8.9e-14    3e-15
   2        0.27   0.3500000        0.27   19   14   14  1.1e-13  4.1e-15
   3        0.18   0.3500000        0.18   20   15   15  6.9e-14  3.1e-15
Velocity u2, Pressure p
Iter      SolEst     Damping    Stepsize #Res #Jac #Sol   LinErr   LinRes
   1       0.035   0.5000000       0.035   10    5    5  3.3e-15  7.8e-15
Displacement field
Iter      SolEst     Damping    Stepsize #Res #Jac #Sol   LinErr   LinRes
   1        0.17   0.5000000        0.17   10    5    5  5.6e-14  1.5e-12
Spatial mesh displacement
Iter      SolEst     Damping    Stepsize #Res #Jac #Sol   LinErr   LinRes
   1     6.9e-07   0.5000000     6.9e-07   10    5    5  1.5e-15  1.8e-15
Solution error estimates for segregated groups
0.31, 0.017, 0.084, 3.4e-07
Residual error estimates for segregated groups
32, 2e+03, 6.8e+03, 0.16
Segregated solver iteration 6.
Merged variables
Iter      SolEst     Damping    Stepsize #Res #Jac #Sol   LinErr   LinRes
   1        0.12   0.3500000        0.12   22   16   16  1.2e-13  3.1e-15
   2       0.077   0.3500000       0.077   23   17   17    5e-14  2.9e-15
   3        0.05   0.3500000        0.05   24   18   18  9.5e-14  2.9e-15
Velocity u2, Pressure p
Iter      SolEst     Damping    Stepsize #Res #Jac #Sol   LinErr   LinRes
   1       0.017   0.5000000       0.017   12    6    6  3.1e-15  7.8e-15
Displacement field
Iter      SolEst     Damping    Stepsize #Res #Jac #Sol   LinErr   LinRes
   1       0.095   0.5000000       0.095   12    6    6  2.6e-14  1.5e-12
Spatial mesh displacement
Iter      SolEst     Damping    Stepsize #Res #Jac #Sol   LinErr   LinRes
   1     4.8e-07   0.5000000     4.8e-07   12    6    6  1.6e-15  1.8e-15
Solution error estimates for segregated groups
0.086, 0.0085, 0.048, 2.4e-07
Residual error estimates for segregated groups
12, 1e+03, 4.2e+03, 0.15
```

```
Segregated solver iteration 7.
Merged variables
Iter      SolEst     Damping    Stepsize #Res #Jac #Sol   LinErr   LinRes
   1       0.033   0.3500000       0.033   26   19   19  1.1e-13  2.7e-15
   2       0.021   0.3500000       0.021   27   20   20  7.1e-14  3.5e-15
   3       0.014   0.3500000       0.014   28   21   21    5e-14  3.3e-15
Velocity u2, Pressure p
Iter      SolEst     Damping    Stepsize #Res #Jac #Sol   LinErr   LinRes
   1      0.0085   0.5000000      0.0085   14    7    7  2.6e-15  7.9e-15
Displacement field
Scales for dependent variables:
Auxiliary pressure (comp1.solid.lemm1.pw): 0.36
Displacement field (comp1.u): 2.9e-16
Iter      SolEst     Damping    Stepsize #Res #Jac #Sol   LinErr   LinRes
   1       0.054   0.5000000       0.054   14    7    7  1.1e-13  1.2e-12
Spatial mesh displacement
Iter      SolEst     Damping    Stepsize #Res #Jac #Sol   LinErr   LinRes
   1     3.2e-07   0.5000000     3.2e-07   14    7    7  1.7e-15  1.9e-15
Solution error estimates for segregated groups
0.024, 0.0042, 0.027, 1.6e-07
Residual error estimates for segregated groups
4.1, 5.2e+02, 2.6e+03, 0.14
Segregated solver iteration 8.
Merged variables
Iter      SolEst     Damping    Stepsize #Res #Jac #Sol   LinErr   LinRes
   1       0.009   0.3500000       0.009   30   22   22  6.3e-14  2.8e-15
   2      0.0059   0.3500000      0.0059   31   23   23  1.1e-13  3.2e-15
   3      0.0038   0.3500000      0.0038   32   24   24  5.2e-14  2.8e-15
Velocity u2, Pressure p
Iter      SolEst     Damping    Stepsize #Res #Jac #Sol   LinErr   LinRes
   1      0.0042   0.5000000      0.0042   16    8    8  2.3e-15    8e-15
Displacement field
Iter      SolEst     Damping    Stepsize #Res #Jac #Sol   LinErr   LinRes
   1        0.03   0.5000000        0.03   16    8    8    1e-13  1.2e-12
Spatial mesh displacement
Iter      SolEst     Damping    Stepsize #Res #Jac #Sol   LinErr   LinRes
   1     2.1e-07   0.5000000     2.1e-07   16    8    8  1.6e-15  1.8e-15
Solution error estimates for segregated groups
0.0066, 0.0021, 0.015, 1e-07
Residual error estimates for segregated groups
1.4, 2.6e+02, 1.8e+03, 0.14
Segregated solver iteration 9.
Merged variables
Iter      SolEst     Damping    Stepsize #Res #Jac #Sol   LinErr   LinRes
   1      0.0025   0.3500000      0.0025   34   25   25  1.2e-13  2.8e-15
   2      0.0016   0.3500000      0.0016   35   26   26  8.4e-14  2.9e-15
   3       0.001   0.3500000       0.001   36   27   27  4.5e-14  3.1e-15
Velocity u2, Pressure p
Iter      SolEst     Damping    Stepsize #Res #Jac #Sol   LinErr   LinRes
   1      0.0021   0.5000000      0.0021   18    9    9  2.2e-15  7.9e-15
Displacement field
Iter      SolEst     Damping    Stepsize #Res #Jac #Sol   LinErr   LinRes
   1       0.017   0.5000000       0.017   18    9    9  1.6e-13  1.2e-12
Spatial mesh displacement
Iter      SolEst     Damping    Stepsize #Res #Jac #Sol   LinErr   LinRes
   1     1.3e-07   0.5000000     1.3e-07   18    9    9  1.5e-15  1.8e-15
```

```
Solution error estimates for segregated groups
0.0018, 0.0011, 0.0085, 6.5e-08
Residual error estimates for segregated groups
0.44, 1.3e+02, 1.4e+03, 0.15
Segregated solver iteration 10.
Merged variables
Iter      SolEst     Damping    Stepsize #Res #Jac #Sol   LinErr   LinRes
   1     0.00068   0.3500000     0.00068   38   28   28    5e-14  3.1e-15
   2     0.00044   0.3500000     0.00044   39   29   29  9.9e-14  3.2e-15
   3     0.00029   0.3500000     0.00029   40   30   30  8.4e-14    3e-15
Velocity u2, Pressure p
Iter      SolEst     Damping    Stepsize #Res #Jac #Sol   LinErr   LinRes
   1      0.0011   0.5000000      0.0011   20   10   10  2.9e-15  7.8e-15
Displacement field
Iter      SolEst     Damping    Stepsize #Res #Jac #Sol   LinErr   LinRes
   1      0.0094   0.5000000      0.0094   20   10   10  1.1e-13  1.2e-12
Spatial mesh displacement
Iter      SolEst     Damping    Stepsize #Res #Jac #Sol   LinErr   LinRes
   1     7.9e-08   0.5000000     7.9e-08   20   10   10  1.8e-15  1.8e-15
Solution error estimates for segregated groups
0.00049, 0.00053, 0.0047, 3.9e-08
Residual error estimates for segregated groups
0.14, 65, 1.2e+03, 0.14
Segregated solver iteration 11.
Merged variables
Iter      SolEst     Damping    Stepsize #Res #Jac #Sol   LinErr   LinRes
   1     0.00019   0.3500000     0.00019   42   31   31  1.1e-13  2.7e-15
   2     0.00012   0.3500000     0.00012   43   32   32  1.5e-13  3.8e-15
   3     7.9e-05   0.3500000     7.9e-05   44   33   33  3.4e-14  2.7e-15
Velocity u2, Pressure p
Iter      SolEst     Damping    Stepsize #Res #Jac #Sol   LinErr   LinRes
   1     0.00053   0.5000000     0.00053   22   11   11  3.4e-15  7.8e-15
Displacement field
Iter      SolEst     Damping    Stepsize #Res #Jac #Sol   LinErr   LinRes
   1      0.0051   0.5000000      0.0051   22   11   11  1.1e-13  1.2e-12
Spatial mesh displacement
Iter      SolEst     Damping    Stepsize #Res #Jac #Sol   LinErr   LinRes
   1     4.7e-08   0.5000000     4.7e-08   22   11   11  1.7e-15  1.8e-15
Solution error estimates for segregated groups
0.00014, 0.00026, 0.0026, 2.4e-08
Residual error estimates for segregated groups
0.043, 32, 1.2e+03, 0.15
Segregated solver iteration 12.
Merged variables
Iter      SolEst     Damping    Stepsize #Res #Jac #Sol   LinErr   LinRes
   1     5.1e-05   0.3500000     5.1e-05   46   34   34  8.7e-14    3e-15
   2     3.3e-05   0.3500000     3.3e-05   47   35   35  8.2e-14  2.9e-15
   3     2.2e-05   0.3500000     2.2e-05   48   36   36    1e-13  2.7e-15
Velocity u2, Pressure p
Iter      SolEst     Damping    Stepsize #Res #Jac #Sol   LinErr   LinRes
   1     0.00026   0.5000000     0.00026   24   12   12  2.7e-15  7.9e-15
Displacement field
Iter      SolEst     Damping    Stepsize #Res #Jac #Sol   LinErr   LinRes
   1      0.0028   0.5000000      0.0028   24   12   12    1e-13  1.2e-12
Spatial mesh displacement
Iter      SolEst     Damping    Stepsize #Res #Jac #Sol   LinErr   LinRes
```

```
   1     2.8e-08   0.5000000     2.8e-08   24   12   12  1.4e-15  1.9e-15
Solution error estimates for segregated groups
3.7e-05, 0.00013, 0.0014, 1.4e-08
Residual error estimates for segregated groups
0.013, 16, 1.2e+03, 0.14
Segregated solver iteration 13.
Merged variables
Iter      SolEst     Damping    Stepsize #Res #Jac #Sol   LinErr   LinRes
   1     1.4e-05   0.3500000     1.4e-05   50   37   37    7e-14  2.8e-15
   2     9.2e-06   0.3500000     9.2e-06   51   38   38  6.4e-14  2.7e-15
   3       6e-06   0.3500000       6e-06   52   39   39  6.5e-14    3e-15
Velocity u2, Pressure p
Iter      SolEst     Damping    Stepsize #Res #Jac #Sol   LinErr   LinRes
   1     0.00013   0.5000000     0.00013   26   13   13  4.3e-15  7.9e-15
Displacement field
Iter      SolEst     Damping    Stepsize #Res #Jac #Sol   LinErr   LinRes
   1      0.0015   0.5000000      0.0015   26   13   13  1.4e-13  1.2e-12
Spatial mesh displacement
Iter      SolEst     Damping    Stepsize #Res #Jac #Sol   LinErr   LinRes
   1     1.6e-08   0.5000000     1.6e-08   26   13   13  1.2e-15  1.9e-15
Solution error estimates for segregated groups
1e-05, 6.6e-05, 0.00076, 8.2e-09
Residual error estimates for segregated groups
0.004, 8.1, 1.2e+03, 0.15
Solution time: 156 s. (2 minutes, 36 seconds)
Physical memory: 4.65 GB
Virtual memory: 25.51 GB
Ended at Jan 31, 2025, 8:12:17 PM.
----- Stationary Solver 1 in study1/Approach Curve Solver (sol1) -------------->
```

d=2135 (su75)

GENERAL

Description	Value
Solution	d=2135

LOG

```
<---- Compile Equations: Stationary in study1/Approach Curve Solver (sol1) -----
Started at Jan 31, 2025, 8:12:18 PM.
Parameter d = 2.1350000000000003E-6 (m).
Geometry shape function: Linear serendipity
Number of vertex elements: 18
Number of boundary elements: 6134
Number of elements: 154702
Minimum element quality: 0.4575
Time: 8 s.
Physical memory: 3 GB
Virtual memory: 15.29 GB
Ended at Jan 31, 2025, 8:12:26 PM.
----- Compile Equations: Stationary in study1/Approach Curve Solver (sol1) ---->

<---- Dependent Variables 1 in study1/Approach Curve Solver (sol1) -------------
Started at Jan 31, 2025, 8:12:26 PM.
Solution time: 0 s.
Physical memory: 3 GB
Virtual memory: 15.22 GB
Ended at Jan 31, 2025, 8:12:26 PM.
----- Dependent Variables 1 in study1/Approach Curve Solver (sol1) ------------>

<---- Stationary Solver 1 in study1/Approach Curve Solver (sol1) ---------------
Started at Jan 31, 2025, 8:12:26 PM.
Segregated solver
Segregated Step 11 does not have any components solved for.
Number of degrees of freedom solved for: 785441 (plus 11691 internal DOFs).
Segregated solver iteration 1.
Merged variables
Nonsymmetric matrix found.
Scales for dependent variables:
Concentration (comp1.Cl): 1.4e+02
Concentration (comp1.K): 1.4e+02
Electric potential (comp1.V): 1.9e+02
Orthonormal null-space function used.
Iter      SolEst     Damping    Stepsize #Res #Jac #Sol   LinErr   LinRes
   1        0.83   0.3500000        0.83    2    1    1    6e-11  2.7e-15
   2        0.81   0.3500000        0.81    3    2    2  4.2e-11  2.7e-15
   3         0.8   0.3500000         0.8    4    3    3    4e-11  2.8e-15
Velocity u2, Pressure p
Nonsymmetric matrix found.
Scales for dependent variables:
Pressure (comp1.p): 1
Velocity field (spatial frame) (comp1.u2): 0.00018
Orthonormal null-space function used.
Iter      SolEst     Damping    Stepsize #Res #Jac #Sol   LinErr   LinRes
   1         1.1   0.5000000         1.1    2    1    1    2e-15  2.2e-15
Displacement field
Nonsymmetric matrix found.
Scales for dependent variables:
Auxiliary pressure (comp1.solid.lemm1.pw): 1
Displacement field (comp1.u): 2.8e-18
Orthonormal null-space function used.
Iter      SolEst     Damping    Stepsize #Res #Jac #Sol   LinErr   LinRes
   1         1.9   0.5000000         1.9    2    1    1    3e-14  1.5e-12
Spatial mesh displacement
```

```
Symmetric matrices found.
Scales for dependent variables:
Spatial mesh displacement (comp1.spatial.disp): 1.2e-09
Orthonormal null-space function used.
Iter      SolEst     Damping    Stepsize #Res #Jac #Sol   LinErr   LinRes
   1     7.4e-07   0.5000000     7.4e-07    2    1    1  1.5e-15  1.8e-15
Solution error estimates for segregated groups
1.4, 0.54, 0.95, 3.7e-07
Residual error estimates for segregated groups
7.8e+03, 8.2e+04, 2.2e+04, 0.12
Segregated solver iteration 2.
Merged variables
Scales for dependent variables:
Concentration (comp1.Cl): 1.4e+02
Concentration (comp1.K): 1.4e+02
Electric potential (comp1.V): 0.064
Iter      SolEst     Damping    Stepsize #Res #Jac #Sol   LinErr   LinRes
   1        0.78   0.3500000        0.78    6    4    4  1.2e-13  2.8e-15
   2        0.77   0.3500000        0.77    7    5    5    6e-14  4.6e-15
   3        0.76   0.3500000        0.76    8    6    6  6.4e-14  4.2e-15
Velocity u2, Pressure p
Scales for dependent variables:
Pressure (comp1.p): 3.6e+02
Velocity field (spatial frame) (comp1.u2): 0.00073
Iter      SolEst     Damping    Stepsize #Res #Jac #Sol   LinErr   LinRes
   1        0.36   0.5000000        0.36    4    2    2  4.9e-15    8e-15
Displacement field
Iter      SolEst     Damping    Stepsize #Res #Jac #Sol   LinErr   LinRes
   1        0.95   0.5000000        0.95    4    2    2  3.7e-14  1.5e-12
Spatial mesh displacement
Iter      SolEst     Damping    Stepsize #Res #Jac #Sol   LinErr   LinRes
   1     1.1e-06   0.5000000     1.1e-06    4    2    2  1.8e-15  1.8e-15
Solution error estimates for segregated groups
1.3, 0.18, 0.48, 5.5e-07
Residual error estimates for segregated groups
7.7e+02, 1.4e+04, 2.2e+04, 0.12
Segregated solver iteration 3.
Merged variables
Iter      SolEst     Damping    Stepsize #Res #Jac #Sol   LinErr   LinRes
   1        0.74   0.3500000        0.74   10    7    7  7.7e-14  6.4e-15
   2        0.73   0.3500000        0.73   11    8    8  5.6e-14  3.1e-15
   3        0.72   0.3500000        0.72   12    9    9    1e-13  3.1e-15
Velocity u2, Pressure p
Iter      SolEst     Damping    Stepsize #Res #Jac #Sol   LinErr   LinRes
   1        0.15   0.5000000        0.15    6    3    3    4e-15  7.8e-15
Displacement field
Iter      SolEst     Damping    Stepsize #Res #Jac #Sol   LinErr   LinRes
   1        0.52   0.5000000        0.52    6    3    3  6.2e-14  1.5e-12
Spatial mesh displacement
Iter      SolEst     Damping    Stepsize #Res #Jac #Sol   LinErr   LinRes
   1     1.1e-06   0.5000000     1.1e-06    6    3    3  1.4e-15  1.8e-15
Solution error estimates for segregated groups
1.2, 0.077, 0.26, 5.5e-07
Residual error estimates for segregated groups
2.2e+02, 8e+03, 1.6e+04, 0.13
Segregated solver iteration 4.
```

```
Merged variables
Iter      SolEst     Damping    Stepsize #Res #Jac #Sol   LinErr   LinRes
   1         0.7   0.3500000         0.7   14   10   10  7.6e-14  5.9e-15
   2        0.68   0.3500000        0.68   15   11   11  1.2e-13  5.1e-15
   3        0.59   0.3500000        0.59   16   12   12  6.8e-14  3.1e-15
Velocity u2, Pressure p
Iter      SolEst     Damping    Stepsize #Res #Jac #Sol   LinErr   LinRes
   1       0.072   0.5000000       0.072    8    4    4  3.4e-15  7.9e-15
Displacement field
Iter      SolEst     Damping    Stepsize #Res #Jac #Sol   LinErr   LinRes
   1        0.29   0.5000000        0.29    8    4    4  4.8e-14  1.5e-12
Spatial mesh displacement
Iter      SolEst     Damping    Stepsize #Res #Jac #Sol   LinErr   LinRes
   1     9.2e-07   0.5000000     9.2e-07    8    4    4  1.7e-15  1.8e-15
Solution error estimates for segregated groups
1, 0.036, 0.15, 4.6e-07
Residual error estimates for segregated groups
82, 4.2e+03, 1.1e+04, 0.14
Segregated solver iteration 5.
Merged variables
Iter      SolEst     Damping    Stepsize #Res #Jac #Sol   LinErr   LinRes
   1        0.41   0.3500000        0.41   18   13   13  6.3e-14    5e-15
   2        0.27   0.3500000        0.27   19   14   14  1.9e-13    3e-15
   3        0.18   0.3500000        0.18   20   15   15  1.3e-13  5.2e-15
Velocity u2, Pressure p
Iter      SolEst     Damping    Stepsize #Res #Jac #Sol   LinErr   LinRes
   1       0.035   0.5000000       0.035   10    5    5  2.3e-15    8e-15
Displacement field
Iter      SolEst     Damping    Stepsize #Res #Jac #Sol   LinErr   LinRes
   1        0.17   0.5000000        0.17   10    5    5  5.8e-14  1.5e-12
Spatial mesh displacement
Iter      SolEst     Damping    Stepsize #Res #Jac #Sol   LinErr   LinRes
   1     6.9e-07   0.5000000     6.9e-07   10    5    5  1.5e-15  1.8e-15
Solution error estimates for segregated groups
0.31, 0.017, 0.084, 3.5e-07
Residual error estimates for segregated groups
32, 2.2e+03, 6.8e+03, 0.15
Segregated solver iteration 6.
Merged variables
Iter      SolEst     Damping    Stepsize #Res #Jac #Sol   LinErr   LinRes
   1        0.12   0.3500000        0.12   22   16   16  4.9e-14    4e-15
   2       0.077   0.3500000       0.077   23   17   17    1e-13  3.9e-15
   3        0.05   0.3500000        0.05   24   18   18  8.3e-14  5.5e-15
Velocity u2, Pressure p
Iter      SolEst     Damping    Stepsize #Res #Jac #Sol   LinErr   LinRes
   1       0.017   0.5000000       0.017   12    6    6  2.3e-15  7.9e-15
Displacement field
Iter      SolEst     Damping    Stepsize #Res #Jac #Sol   LinErr   LinRes
   1       0.095   0.5000000       0.095   12    6    6    9e-14  1.5e-12
Spatial mesh displacement
Iter      SolEst     Damping    Stepsize #Res #Jac #Sol   LinErr   LinRes
   1     4.8e-07   0.5000000     4.8e-07   12    6    6  1.7e-15  1.8e-15
Solution error estimates for segregated groups
0.086, 0.0085, 0.048, 2.4e-07
Residual error estimates for segregated groups
12, 1.1e+03, 4.2e+03, 0.14
```

```
Segregated solver iteration 7.
Merged variables
Iter      SolEst     Damping    Stepsize #Res #Jac #Sol   LinErr   LinRes
   1       0.033   0.3500000       0.033   26   19   19  1.3e-13  4.5e-15
   2       0.021   0.3500000       0.021   27   20   20  1.1e-13  4.7e-15
   3       0.014   0.3500000       0.014   28   21   21  1.4e-13  2.8e-15
Velocity u2, Pressure p
Iter      SolEst     Damping    Stepsize #Res #Jac #Sol   LinErr   LinRes
   1      0.0085   0.5000000      0.0085   14    7    7  2.3e-15  7.8e-15
Displacement field
Scales for dependent variables:
Auxiliary pressure (comp1.solid.lemm1.pw): 0.36
Displacement field (comp1.u): 2.9e-16
Iter      SolEst     Damping    Stepsize #Res #Jac #Sol   LinErr   LinRes
   1       0.054   0.5000000       0.054   14    7    7  1.1e-13  1.2e-12
Spatial mesh displacement
Iter      SolEst     Damping    Stepsize #Res #Jac #Sol   LinErr   LinRes
   1     3.2e-07   0.5000000     3.2e-07   14    7    7  1.3e-15  1.9e-15
Solution error estimates for segregated groups
0.024, 0.0042, 0.027, 1.6e-07
Residual error estimates for segregated groups
4.1, 5.5e+02, 2.6e+03, 0.14
Segregated solver iteration 8.
Merged variables
Iter      SolEst     Damping    Stepsize #Res #Jac #Sol   LinErr   LinRes
   1       0.009   0.3500000       0.009   30   22   22  7.7e-14  6.4e-15
   2      0.0059   0.3500000      0.0059   31   23   23  8.9e-14  5.5e-15
   3      0.0038   0.3500000      0.0038   32   24   24  7.1e-14  4.3e-15
Velocity u2, Pressure p
Iter      SolEst     Damping    Stepsize #Res #Jac #Sol   LinErr   LinRes
   1      0.0042   0.5000000      0.0042   16    8    8  2.9e-15  7.8e-15
Displacement field
Iter      SolEst     Damping    Stepsize #Res #Jac #Sol   LinErr   LinRes
   1        0.03   0.5000000        0.03   16    8    8    1e-13  1.2e-12
Spatial mesh displacement
Iter      SolEst     Damping    Stepsize #Res #Jac #Sol   LinErr   LinRes
   1     2.1e-07   0.5000000     2.1e-07   16    8    8  1.6e-15  1.8e-15
Solution error estimates for segregated groups
0.0066, 0.0021, 0.015, 1e-07
Residual error estimates for segregated groups
1.4, 2.8e+02, 1.8e+03, 0.15
Segregated solver iteration 9.
Merged variables
Iter      SolEst     Damping    Stepsize #Res #Jac #Sol   LinErr   LinRes
   1      0.0025   0.3500000      0.0025   34   25   25    1e-13  3.9e-15
   2      0.0016   0.3500000      0.0016   35   26   26  9.3e-14  2.9e-15
   3       0.001   0.3500000       0.001   36   27   27  1.2e-13  3.8e-15
Velocity u2, Pressure p
Iter      SolEst     Damping    Stepsize #Res #Jac #Sol   LinErr   LinRes
   1      0.0021   0.5000000      0.0021   18    9    9  2.8e-15  7.9e-15
Displacement field
Iter      SolEst     Damping    Stepsize #Res #Jac #Sol   LinErr   LinRes
   1       0.017   0.5000000       0.017   18    9    9  9.6e-14  1.2e-12
Spatial mesh displacement
Iter      SolEst     Damping    Stepsize #Res #Jac #Sol   LinErr   LinRes
   1     1.3e-07   0.5000000     1.3e-07   18    9    9  1.4e-15  1.8e-15
```

```
Solution error estimates for segregated groups
0.0018, 0.0011, 0.0085, 6.5e-08
Residual error estimates for segregated groups
0.44, 1.4e+02, 1.4e+03, 0.14
Segregated solver iteration 10.
Merged variables
Iter      SolEst     Damping    Stepsize #Res #Jac #Sol   LinErr   LinRes
   1     0.00068   0.3500000     0.00068   38   28   28  7.4e-14  6.9e-15
   2     0.00044   0.3500000     0.00044   39   29   29  7.7e-14  4.2e-15
   3     0.00029   0.3500000     0.00029   40   30   30  1.2e-13  3.1e-15
Velocity u2, Pressure p
Iter      SolEst     Damping    Stepsize #Res #Jac #Sol   LinErr   LinRes
   1      0.0011   0.5000000      0.0011   20   10   10  2.8e-15  7.9e-15
Displacement field
Iter      SolEst     Damping    Stepsize #Res #Jac #Sol   LinErr   LinRes
   1      0.0094   0.5000000      0.0094   20   10   10  1.1e-13  1.2e-12
Spatial mesh displacement
Iter      SolEst     Damping    Stepsize #Res #Jac #Sol   LinErr   LinRes
   1     7.9e-08   0.5000000     7.9e-08   20   10   10  1.8e-15  1.9e-15
Solution error estimates for segregated groups
0.00049, 0.00053, 0.0047, 4e-08
Residual error estimates for segregated groups
0.14, 70, 1.3e+03, 0.14
Segregated solver iteration 11.
Merged variables
Iter      SolEst     Damping    Stepsize #Res #Jac #Sol   LinErr   LinRes
   1     0.00019   0.3500000     0.00019   42   31   31  1.1e-13  3.2e-15
   2     0.00012   0.3500000     0.00012   43   32   32  1.9e-13  4.4e-15
   3     7.9e-05   0.3500000     7.9e-05   44   33   33  1.2e-13  3.4e-15
Velocity u2, Pressure p
Iter      SolEst     Damping    Stepsize #Res #Jac #Sol   LinErr   LinRes
   1     0.00053   0.5000000     0.00053   22   11   11  1.9e-15  7.8e-15
Displacement field
Iter      SolEst     Damping    Stepsize #Res #Jac #Sol   LinErr   LinRes
   1      0.0051   0.5000000      0.0051   22   11   11  1.4e-13  1.2e-12
Spatial mesh displacement
Iter      SolEst     Damping    Stepsize #Res #Jac #Sol   LinErr   LinRes
   1     4.8e-08   0.5000000     4.8e-08   22   11   11  1.7e-15  1.9e-15
Solution error estimates for segregated groups
0.00014, 0.00026, 0.0026, 2.4e-08
Residual error estimates for segregated groups
0.043, 35, 1.2e+03, 0.15
Segregated solver iteration 12.
Merged variables
Iter      SolEst     Damping    Stepsize #Res #Jac #Sol   LinErr   LinRes
   1     5.1e-05   0.3500000     5.1e-05   46   34   34  1.1e-13  3.3e-15
   2     3.3e-05   0.3500000     3.3e-05   47   35   35  7.5e-14  3.6e-15
   3     2.2e-05   0.3500000     2.2e-05   48   36   36  1.2e-13  4.3e-15
Velocity u2, Pressure p
Iter      SolEst     Damping    Stepsize #Res #Jac #Sol   LinErr   LinRes
   1     0.00026   0.5000000     0.00026   24   12   12  3.2e-15  7.9e-15
Displacement field
Iter      SolEst     Damping    Stepsize #Res #Jac #Sol   LinErr   LinRes
   1      0.0028   0.5000000      0.0028   24   12   12  1.1e-13  1.2e-12
Spatial mesh displacement
Iter      SolEst     Damping    Stepsize #Res #Jac #Sol   LinErr   LinRes
```

```
   1     2.8e-08   0.5000000     2.8e-08   24    12    12  1.4e-15  1.8e-15
Solution error estimates for segregated groups
3.7e-05, 0.00013, 0.0014, 1.4e-08
Residual error estimates for segregated groups
0.013, 17, 1.2e+03, 0.14
Segregated solver iteration 13.
Merged variables
Iter      SolEst     Damping    Stepsize #Res #Jac #Sol   LinErr   LinRes
   1     1.4e-05   0.3500000     1.4e-05   50    37    37  1.5e-13  4.7e-15
   2     9.2e-06   0.3500000     9.2e-06   51    38    38  1.1e-13  3.1e-15
   3       6e-06   0.3500000       6e-06   52    39    39  9.9e-14    3e-15
Velocity u2, Pressure p
Iter      SolEst     Damping    Stepsize #Res #Jac #Sol   LinErr   LinRes
   1     0.00013   0.5000000     0.00013   26    13    13  4.9e-15  7.9e-15
Displacement field
Iter      SolEst     Damping    Stepsize #Res #Jac #Sol   LinErr   LinRes
   1      0.0015   0.5000000      0.0015   26    13    13  1.6e-13  1.2e-12
Spatial mesh displacement
Iter      SolEst     Damping    Stepsize #Res #Jac #Sol   LinErr   LinRes
   1     1.6e-08   0.5000000     1.6e-08   26    13    13  1.6e-15  1.8e-15
Solution error estimates for segregated groups
1e-05, 6.6e-05, 0.00076, 8.2e-09
Residual error estimates for segregated groups
0.004, 8.7, 1.2e+03, 0.14
Solution time: 156 s. (2 minutes, 36 seconds)
Physical memory: 4.66 GB
Virtual memory: 25.53 GB
Ended at Jan 31, 2025, 8:15:01 PM.
----- Stationary Solver 1 in study1/Approach Curve Solver (sol1) -------------->
```

d=2130 (su76)

GENERAL

Description	Value
Solution	d=2130

LOG

```
<---- Compile Equations: Stationary in study1/Approach Curve Solver (sol1) -----
Started at Jan 31, 2025, 8:15:02 PM.
Parameter d = 2.13E-6 (m).
Geometry shape function: Linear serendipity
Number of vertex elements: 18
Number of boundary elements: 6131
Number of elements: 154189
Minimum element quality: 0.4823
Time: 8 s.
Physical memory: 2.99 GB
Virtual memory: 15.28 GB
Ended at Jan 31, 2025, 8:15:10 PM.
----- Compile Equations: Stationary in study1/Approach Curve Solver (sol1) ---->

<---- Dependent Variables 1 in study1/Approach Curve Solver (sol1) -------------
Started at Jan 31, 2025, 8:15:10 PM.
Solution time: 0 s.
Physical memory: 2.99 GB
Virtual memory: 15.21 GB
Ended at Jan 31, 2025, 8:15:10 PM.
----- Dependent Variables 1 in study1/Approach Curve Solver (sol1) ------------>

<---- Stationary Solver 1 in study1/Approach Curve Solver (sol1) ---------------
Started at Jan 31, 2025, 8:15:10 PM.
Segregated solver
Segregated Step 11 does not have any components solved for.
Number of degrees of freedom solved for: 782808 (plus 11685 internal DOFs).
Segregated solver iteration 1.
Merged variables
Nonsymmetric matrix found.
Scales for dependent variables:
Concentration (comp1.Cl): 1.4e+02
Concentration (comp1.K): 1.4e+02
Electric potential (comp1.V): 1.9e+02
Orthonormal null-space function used.
Iter      SolEst     Damping    Stepsize #Res #Jac #Sol   LinErr   LinRes
   1        0.83   0.3500000        0.83    2    1    1  2.1e-11  2.8e-15
   2        0.81   0.3500000        0.81    3    2    2  4.6e-11  2.7e-15
   3         0.8   0.3500000         0.8    4    3    3  3.6e-11  2.7e-15
Velocity u2, Pressure p
Nonsymmetric matrix found.
Scales for dependent variables:
Pressure (comp1.p): 1
Velocity field (spatial frame) (comp1.u2): 0.00018
Orthonormal null-space function used.
Iter      SolEst     Damping    Stepsize #Res #Jac #Sol   LinErr   LinRes
   1         1.1   0.5000000         1.1    2    1    1  1.8e-15  2.2e-15
Displacement field
Nonsymmetric matrix found.
Scales for dependent variables:
Auxiliary pressure (comp1.solid.lemm1.pw): 1
Displacement field (comp1.u): 2.8e-18
Orthonormal null-space function used.
Iter      SolEst     Damping    Stepsize #Res #Jac #Sol   LinErr   LinRes
   1         1.9   0.5000000         1.9    2    1    1  8.6e-14  1.5e-12
Spatial mesh displacement
```

```
Symmetric matrices found.
Scales for dependent variables:
Spatial mesh displacement (comp1.spatial.disp): 1.2e-09
Orthonormal null-space function used.
Iter      SolEst     Damping    Stepsize #Res #Jac #Sol   LinErr   LinRes
   1     7.4e-07   0.5000000     7.4e-07    2    1    1  1.5e-15  1.9e-15
Solution error estimates for segregated groups
1.4, 0.54, 0.95, 3.7e-07
Residual error estimates for segregated groups
7.8e+03, 8.2e+04, 2.1e+04, 0.12
Segregated solver iteration 2.
Merged variables
Scales for dependent variables:
Concentration (comp1.Cl): 1.4e+02
Concentration (comp1.K): 1.4e+02
Electric potential (comp1.V): 0.064
Iter      SolEst     Damping    Stepsize #Res #Jac #Sol   LinErr   LinRes
   1        0.78   0.3500000        0.78    6    4    4  6.9e-14  2.6e-15
   2        0.77   0.3500000        0.77    7    5    5  1.1e-13  2.7e-15
   3        0.76   0.3500000        0.76    8    6    6  5.1e-14  2.7e-15
Velocity u2, Pressure p
Scales for dependent variables:
Pressure (comp1.p): 3.6e+02
Velocity field (spatial frame) (comp1.u2): 0.00073
Iter      SolEst     Damping    Stepsize #Res #Jac #Sol   LinErr   LinRes
   1        0.36   0.5000000        0.36    4    2    2  3.1e-15  7.8e-15
Displacement field
Iter      SolEst     Damping    Stepsize #Res #Jac #Sol   LinErr   LinRes
   1        0.95   0.5000000        0.95    4    2    2  6.5e-14  1.4e-12
Spatial mesh displacement
Iter      SolEst     Damping    Stepsize #Res #Jac #Sol   LinErr   LinRes
   1     1.1e-06   0.5000000     1.1e-06    4    2    2  1.4e-15  1.8e-15
Solution error estimates for segregated groups
1.3, 0.18, 0.48, 5.6e-07
Residual error estimates for segregated groups
7.7e+02, 1.6e+04, 2.2e+04, 0.14
Segregated solver iteration 3.
Merged variables
Iter      SolEst     Damping    Stepsize #Res #Jac #Sol   LinErr   LinRes
   1        0.74   0.3500000        0.74   10    7    7  4.7e-14  2.7e-15
   2        0.73   0.3500000        0.73   11    8    8  7.6e-14  2.7e-15
   3        0.72   0.3500000        0.72   12    9    9  4.7e-14  2.7e-15
Velocity u2, Pressure p
Iter      SolEst     Damping    Stepsize #Res #Jac #Sol   LinErr   LinRes
   1        0.15   0.5000000        0.15    6    3    3  2.2e-15    8e-15
Displacement field
Iter      SolEst     Damping    Stepsize #Res #Jac #Sol   LinErr   LinRes
   1        0.52   0.5000000        0.52    6    3    3    4e-14  1.5e-12
Spatial mesh displacement
Iter      SolEst     Damping    Stepsize #Res #Jac #Sol   LinErr   LinRes
   1     1.1e-06   0.5000000     1.1e-06    6    3    3  1.9e-15  1.9e-15
Solution error estimates for segregated groups
1.2, 0.077, 0.26, 5.6e-07
Residual error estimates for segregated groups
2.1e+02, 9.3e+03, 1.6e+04, 0.13
Segregated solver iteration 4.
```

```
Merged variables
Iter      SolEst     Damping    Stepsize #Res #Jac #Sol   LinErr   LinRes
   1         0.7   0.3500000         0.7   14   10   10  7.9e-14  2.7e-15
   2        0.68   0.3500000        0.68   15   11   11  9.8e-14  2.8e-15
   3        0.59   0.3500000        0.59   16   12   12  9.6e-14  2.7e-15
Velocity u2, Pressure p
Iter      SolEst     Damping    Stepsize #Res #Jac #Sol   LinErr   LinRes
   1       0.072   0.5000000       0.072    8    4    4  2.2e-15  7.8e-15
Displacement field
Iter      SolEst     Damping    Stepsize #Res #Jac #Sol   LinErr   LinRes
   1        0.29   0.5000000        0.29    8    4    4  9.3e-14  1.5e-12
Spatial mesh displacement
Iter      SolEst     Damping    Stepsize #Res #Jac #Sol   LinErr   LinRes
   1     9.3e-07   0.5000000     9.3e-07    8    4    4  1.3e-15  1.8e-15
Solution error estimates for segregated groups
1, 0.036, 0.15, 4.7e-07
Residual error estimates for segregated groups
82, 4.9e+03, 1.1e+04, 0.13
Segregated solver iteration 5.
Merged variables
Iter      SolEst     Damping    Stepsize #Res #Jac #Sol   LinErr   LinRes
   1         0.4   0.3500000         0.4   18   13   13  6.7e-14  2.7e-15
   2        0.27   0.3500000        0.27   19   14   14  8.1e-14  2.7e-15
   3        0.18   0.3500000        0.18   20   15   15  4.2e-14  2.8e-15
Velocity u2, Pressure p
Iter      SolEst     Damping    Stepsize #Res #Jac #Sol   LinErr   LinRes
   1       0.035   0.5000000       0.035   10    5    5  3.3e-15  7.8e-15
Displacement field
Iter      SolEst     Damping    Stepsize #Res #Jac #Sol   LinErr   LinRes
   1        0.17   0.5000000        0.17   10    5    5  6.7e-14  1.5e-12
Spatial mesh displacement
Iter      SolEst     Damping    Stepsize #Res #Jac #Sol   LinErr   LinRes
   1       7e-07   0.5000000       7e-07   10    5    5  1.3e-15  1.9e-15
Solution error estimates for segregated groups
0.31, 0.017, 0.084, 3.5e-07
Residual error estimates for segregated groups
32, 2.5e+03, 6.8e+03, 0.15
Segregated solver iteration 6.
Merged variables
Iter      SolEst     Damping    Stepsize #Res #Jac #Sol   LinErr   LinRes
   1        0.12   0.3500000        0.12   22   16   16  8.5e-14  2.7e-15
   2       0.077   0.3500000       0.077   23   17   17  3.1e-14  2.7e-15
   3        0.05   0.3500000        0.05   24   18   18    8e-14  2.8e-15
Velocity u2, Pressure p
Iter      SolEst     Damping    Stepsize #Res #Jac #Sol   LinErr   LinRes
   1       0.017   0.5000000       0.017   12    6    6  4.2e-15  7.8e-15
Displacement field
Iter      SolEst     Damping    Stepsize #Res #Jac #Sol   LinErr   LinRes
   1       0.095   0.5000000       0.095   12    6    6  7.3e-14  1.5e-12
Spatial mesh displacement
Iter      SolEst     Damping    Stepsize #Res #Jac #Sol   LinErr   LinRes
   1     4.9e-07   0.5000000     4.9e-07   12    6    6  1.4e-15  1.8e-15
Solution error estimates for segregated groups
0.086, 0.0085, 0.048, 2.4e-07
Residual error estimates for segregated groups
12, 1.3e+03, 4.2e+03, 0.15
```

```
Segregated solver iteration 7.
Merged variables
Iter      SolEst     Damping    Stepsize #Res #Jac #Sol   LinErr   LinRes
   1       0.033   0.3500000       0.033   26   19   19  1.1e-13  2.7e-15
   2       0.021   0.3500000       0.021   27   20   20  5.9e-14  2.7e-15
   3       0.014   0.3500000       0.014   28   21   21  7.2e-14  2.7e-15
Velocity u2, Pressure p
Iter      SolEst     Damping    Stepsize #Res #Jac #Sol   LinErr   LinRes
   1      0.0085   0.5000000      0.0085   14    7    7  2.4e-15  7.9e-15
Displacement field
Scales for dependent variables:
Auxiliary pressure (comp1.solid.lemm1.pw): 0.37
Displacement field (comp1.u): 2.9e-16
Iter      SolEst     Damping    Stepsize #Res #Jac #Sol   LinErr   LinRes
   1       0.054   0.5000000       0.054   14    7    7  1.9e-13  1.2e-12
Spatial mesh displacement
Iter      SolEst     Damping    Stepsize #Res #Jac #Sol   LinErr   LinRes
   1     3.3e-07   0.5000000     3.3e-07   14    7    7  1.3e-15  1.8e-15
Solution error estimates for segregated groups
0.024, 0.0042, 0.027, 1.6e-07
Residual error estimates for segregated groups
4.1, 6.5e+02, 2.6e+03, 0.15
Segregated solver iteration 8.
Merged variables
Iter      SolEst     Damping    Stepsize #Res #Jac #Sol   LinErr   LinRes
   1       0.009   0.3500000       0.009   30   22   22  1.3e-13  2.8e-15
   2      0.0058   0.3500000      0.0058   31   23   23  1.1e-13  2.7e-15
   3      0.0038   0.3500000      0.0038   32   24   24    1e-13  2.7e-15
Velocity u2, Pressure p
Iter      SolEst     Damping    Stepsize #Res #Jac #Sol   LinErr   LinRes
   1      0.0042   0.5000000      0.0042   16    8    8  2.9e-15  7.9e-15
Displacement field
Iter      SolEst     Damping    Stepsize #Res #Jac #Sol   LinErr   LinRes
   1        0.03   0.5000000        0.03   16    8    8  1.2e-13  1.2e-12
Spatial mesh displacement
Iter      SolEst     Damping    Stepsize #Res #Jac #Sol   LinErr   LinRes
   1     2.1e-07   0.5000000     2.1e-07   16    8    8  2.1e-15  1.8e-15
Solution error estimates for segregated groups
0.0065, 0.0021, 0.015, 1e-07
Residual error estimates for segregated groups
1.4, 3.3e+02, 1.8e+03, 0.15
Segregated solver iteration 9.
Merged variables
Iter      SolEst     Damping    Stepsize #Res #Jac #Sol   LinErr   LinRes
   1      0.0025   0.3500000      0.0025   34   25   25  3.1e-14  2.6e-15
   2      0.0016   0.3500000      0.0016   35   26   26  7.2e-14  2.8e-15
   3       0.001   0.3500000       0.001   36   27   27    3e-14  2.7e-15
Velocity u2, Pressure p
Iter      SolEst     Damping    Stepsize #Res #Jac #Sol   LinErr   LinRes
   1      0.0021   0.5000000      0.0021   18    9    9  2.2e-15  7.9e-15
Displacement field
Iter      SolEst     Damping    Stepsize #Res #Jac #Sol   LinErr   LinRes
   1       0.017   0.5000000       0.017   18    9    9  1.7e-13  1.2e-12
Spatial mesh displacement
Iter      SolEst     Damping    Stepsize #Res #Jac #Sol   LinErr   LinRes
   1     1.3e-07   0.5000000     1.3e-07   18    9    9  1.8e-15  1.8e-15
```

```
Solution error estimates for segregated groups
0.0018, 0.0011, 0.0085, 6.6e-08
Residual error estimates for segregated groups
0.44, 1.6e+02, 1.4e+03, 0.14
Segregated solver iteration 10.
Merged variables
Iter      SolEst     Damping    Stepsize #Res #Jac #Sol   LinErr   LinRes
   1     0.00068   0.3500000     0.00068   38   28   28  8.2e-14  2.6e-15
   2     0.00044   0.3500000     0.00044   39   29   29    1e-13  2.7e-15
   3     0.00029   0.3500000     0.00029   40   30   30  6.8e-14  2.7e-15
Velocity u2, Pressure p
Iter      SolEst     Damping    Stepsize #Res #Jac #Sol   LinErr   LinRes
   1      0.0011   0.5000000      0.0011   20   10   10  2.1e-15  7.9e-15
Displacement field
Iter      SolEst     Damping    Stepsize #Res #Jac #Sol   LinErr   LinRes
   1      0.0094   0.5000000      0.0094   20   10   10  1.2e-13  1.2e-12
Spatial mesh displacement
Iter      SolEst     Damping    Stepsize #Res #Jac #Sol   LinErr   LinRes
   1       8e-08   0.5000000       8e-08   20   10   10  1.9e-15  1.9e-15
Solution error estimates for segregated groups
0.00049, 0.00053, 0.0047, 4e-08
Residual error estimates for segregated groups
0.14, 82, 1.2e+03, 0.14
Segregated solver iteration 11.
Merged variables
Iter      SolEst     Damping    Stepsize #Res #Jac #Sol   LinErr   LinRes
   1     0.00019   0.3500000     0.00019   42   31   31  6.5e-14  2.8e-15
   2     0.00012   0.3500000     0.00012   43   32   32  5.9e-14  2.8e-15
   3     7.9e-05   0.3500000     7.9e-05   44   33   33  4.9e-14  2.7e-15
Velocity u2, Pressure p
Iter      SolEst     Damping    Stepsize #Res #Jac #Sol   LinErr   LinRes
   1     0.00053   0.5000000     0.00053   22   11   11  2.1e-15  7.8e-15
Displacement field
Iter      SolEst     Damping    Stepsize #Res #Jac #Sol   LinErr   LinRes
   1      0.0051   0.5000000      0.0051   22   11   11  1.4e-13  1.2e-12
Spatial mesh displacement
Iter      SolEst     Damping    Stepsize #Res #Jac #Sol   LinErr   LinRes
   1     4.8e-08   0.5000000     4.8e-08   22   11   11  1.4e-15  1.8e-15
Solution error estimates for segregated groups
0.00014, 0.00026, 0.0026, 2.4e-08
Residual error estimates for segregated groups
0.043, 41, 1.2e+03, 0.14
Segregated solver iteration 12.
Merged variables
Iter      SolEst     Damping    Stepsize #Res #Jac #Sol   LinErr   LinRes
   1     5.1e-05   0.3500000     5.1e-05   46   34   34  8.4e-14  2.8e-15
   2     3.3e-05   0.3500000     3.3e-05   47   35   35  5.2e-14  2.7e-15
   3     2.2e-05   0.3500000     2.2e-05   48   36   36  4.4e-14  2.7e-15
Velocity u2, Pressure p
Iter      SolEst     Damping    Stepsize #Res #Jac #Sol   LinErr   LinRes
   1     0.00026   0.5000000     0.00026   24   12   12  2.9e-15  7.9e-15
Displacement field
Iter      SolEst     Damping    Stepsize #Res #Jac #Sol   LinErr   LinRes
   1      0.0028   0.5000000      0.0028   24   12   12  9.4e-14  1.2e-12
Spatial mesh displacement
Iter      SolEst     Damping    Stepsize #Res #Jac #Sol   LinErr   LinRes
```

```
   1     2.8e-08   0.5000000     2.8e-08   24   12   12  1.9e-15  1.8e-15
Solution error estimates for segregated groups
3.7e-05, 0.00013, 0.0014, 1.4e-08
Residual error estimates for segregated groups
0.013, 20, 1.2e+03, 0.15
Segregated solver iteration 13.
Merged variables
Iter      SolEst     Damping    Stepsize #Res #Jac #Sol   LinErr   LinRes
   1     1.4e-05   0.3500000     1.4e-05   50   37   37  7.3e-14  2.7e-15
   2     9.1e-06   0.3500000     9.1e-06   51   38   38  6.2e-14  2.7e-15
   3     5.9e-06   0.3500000     5.9e-06   52   39   39  4.6e-14  2.8e-15
Velocity u2, Pressure p
Iter      SolEst     Damping    Stepsize #Res #Jac #Sol   LinErr   LinRes
   1     0.00013   0.5000000     0.00013   26   13   13    5e-15  8.1e-15
Displacement field
Iter      SolEst     Damping    Stepsize #Res #Jac #Sol   LinErr   LinRes
   1      0.0015   0.5000000      0.0015   26   13   13  2.3e-13  1.2e-12
Spatial mesh displacement
Iter      SolEst     Damping    Stepsize #Res #Jac #Sol   LinErr   LinRes
   1     1.7e-08   0.5000000     1.7e-08   26   13   13  1.7e-15  1.8e-15
Solution error estimates for segregated groups
1e-05, 6.6e-05, 0.00076, 8.3e-09
Residual error estimates for segregated groups
0.004, 10, 1.2e+03, 0.15
Solution time: 157 s. (2 minutes, 37 seconds)
Physical memory: 4.72 GB
Virtual memory: 25.51 GB
Ended at Jan 31, 2025, 8:17:47 PM.
----- Stationary Solver 1 in study1/Approach Curve Solver (sol1) -------------->
```

d=2125 (su77)

GENERAL

Description	Value
Solution	d=2125

LOG

```
<---- Compile Equations: Stationary in study1/Approach Curve Solver (sol1) -----
Started at Jan 31, 2025, 8:17:47 PM.
Parameter d = 2.125E-6 (m).
Geometry shape function: Linear serendipity
Number of vertex elements: 18
Number of boundary elements: 6129
Number of elements: 154549
Minimum element quality: 0.4673
Time: 8 s.
Physical memory: 2.98 GB
Virtual memory: 15.2 GB
Ended at Jan 31, 2025, 8:17:55 PM.
----- Compile Equations: Stationary in study1/Approach Curve Solver (sol1) ---->

<---- Dependent Variables 1 in study1/Approach Curve Solver (sol1) -------------
Started at Jan 31, 2025, 8:17:55 PM.
Solution time: 0 s.
Physical memory: 2.98 GB
Virtual memory: 15.2 GB
Ended at Jan 31, 2025, 8:17:55 PM.
----- Dependent Variables 1 in study1/Approach Curve Solver (sol1) ------------>

<---- Stationary Solver 1 in study1/Approach Curve Solver (sol1) ---------------
Started at Jan 31, 2025, 8:17:55 PM.
Segregated solver
Segregated Step 11 does not have any components solved for.
Number of degrees of freedom solved for: 784563 (plus 11681 internal DOFs).
Segregated solver iteration 1.
Merged variables
Nonsymmetric matrix found.
Scales for dependent variables:
Concentration (comp1.Cl): 1.4e+02
Concentration (comp1.K): 1.4e+02
Electric potential (comp1.V): 1.9e+02
Orthonormal null-space function used.
Iter      SolEst     Damping    Stepsize #Res #Jac #Sol   LinErr   LinRes
   1        0.83   0.3500000        0.83    2    1    1  3.5e-11  2.7e-15
   2        0.81   0.3500000        0.81    3    2    2    3e-11  2.7e-15
   3         0.8   0.3500000         0.8    4    3    3  4.1e-11  2.7e-15
Velocity u2, Pressure p
Nonsymmetric matrix found.
Scales for dependent variables:
Pressure (comp1.p): 1
Velocity field (spatial frame) (comp1.u2): 0.00018
Orthonormal null-space function used.
Iter      SolEst     Damping    Stepsize #Res #Jac #Sol   LinErr   LinRes
   1         1.1   0.5000000         1.1    2    1    1  1.5e-15  2.3e-15
Displacement field
Nonsymmetric matrix found.
Scales for dependent variables:
Auxiliary pressure (comp1.solid.lemm1.pw): 1
Displacement field (comp1.u): 2.8e-18
Orthonormal null-space function used.
Iter      SolEst     Damping    Stepsize #Res #Jac #Sol   LinErr   LinRes
   1         1.9   0.5000000         1.9    2    1    1  1.3e-13  1.5e-12
Spatial mesh displacement
```

```
Symmetric matrices found.
Scales for dependent variables:
Spatial mesh displacement (comp1.spatial.disp): 1.2e-09
Orthonormal null-space function used.
Iter      SolEst     Damping    Stepsize #Res #Jac #Sol   LinErr   LinRes
   1     7.4e-07   0.5000000     7.4e-07    2    1    1  2.1e-15  1.8e-15
Solution error estimates for segregated groups
1.4, 0.54, 0.95, 3.7e-07
Residual error estimates for segregated groups
7.8e+03, 8.2e+04, 2.2e+04, 0.13
Segregated solver iteration 2.
Merged variables
Scales for dependent variables:
Concentration (comp1.Cl): 1.4e+02
Concentration (comp1.K): 1.4e+02
Electric potential (comp1.V): 0.064
Iter      SolEst     Damping    Stepsize #Res #Jac #Sol   LinErr   LinRes
   1        0.78   0.3500000        0.78    6    4    4  4.9e-14  2.9e-15
   2        0.77   0.3500000        0.77    7    5    5  5.6e-14  3.6e-15
   3        0.76   0.3500000        0.76    8    6    6  7.7e-14  2.8e-15
Velocity u2, Pressure p
Scales for dependent variables:
Pressure (comp1.p): 3.6e+02
Velocity field (spatial frame) (comp1.u2): 0.00073
Iter      SolEst     Damping    Stepsize #Res #Jac #Sol   LinErr   LinRes
   1        0.36   0.5000000        0.36    4    2    2  1.9e-15  7.9e-15
Displacement field
Iter      SolEst     Damping    Stepsize #Res #Jac #Sol   LinErr   LinRes
   1        0.95   0.5000000        0.95    4    2    2  5.1e-14  1.5e-12
Spatial mesh displacement
Iter      SolEst     Damping    Stepsize #Res #Jac #Sol   LinErr   LinRes
   1     1.1e-06   0.5000000     1.1e-06    4    2    2  1.7e-15  1.9e-15
Solution error estimates for segregated groups
1.3, 0.18, 0.48, 5.6e-07
Residual error estimates for segregated groups
7.7e+02, 1.4e+04, 2.2e+04, 0.14
Segregated solver iteration 3.
Merged variables
Iter      SolEst     Damping    Stepsize #Res #Jac #Sol   LinErr   LinRes
   1        0.74   0.3500000        0.74   10    7    7    1e-13  2.8e-15
   2        0.73   0.3500000        0.73   11    8    8    1e-13    3e-15
   3        0.72   0.3500000        0.72   12    9    9  6.3e-14  4.4e-15
Velocity u2, Pressure p
Iter      SolEst     Damping    Stepsize #Res #Jac #Sol   LinErr   LinRes
   1        0.15   0.5000000        0.15    6    3    3  2.9e-15  7.9e-15
Displacement field
Iter      SolEst     Damping    Stepsize #Res #Jac #Sol   LinErr   LinRes
   1        0.52   0.5000000        0.52    6    3    3  1.2e-13  1.5e-12
Spatial mesh displacement
Iter      SolEst     Damping    Stepsize #Res #Jac #Sol   LinErr   LinRes
   1     1.1e-06   0.5000000     1.1e-06    6    3    3  1.6e-15  1.9e-15
Solution error estimates for segregated groups
1.2, 0.077, 0.26, 5.6e-07
Residual error estimates for segregated groups
2.1e+02, 8e+03, 1.6e+04, 0.14
Segregated solver iteration 4.
```

```
Merged variables
Iter      SolEst     Damping    Stepsize #Res #Jac #Sol   LinErr   LinRes
   1         0.7   0.3500000         0.7   14   10   10  5.9e-14  4.9e-15
   2        0.68   0.3500000        0.68   15   11   11    1e-13  4.5e-15
   3        0.59   0.3500000        0.59   16   12   12  4.8e-14  2.8e-15
Velocity u2, Pressure p
Iter      SolEst     Damping    Stepsize #Res #Jac #Sol   LinErr   LinRes
   1       0.072   0.5000000       0.072    8    4    4    4e-15  7.8e-15
Displacement field
Iter      SolEst     Damping    Stepsize #Res #Jac #Sol   LinErr   LinRes
   1        0.29   0.5000000        0.29    8    4    4  8.6e-14  1.5e-12
Spatial mesh displacement
Iter      SolEst     Damping    Stepsize #Res #Jac #Sol   LinErr   LinRes
   1     9.3e-07   0.5000000     9.3e-07    8    4    4  1.5e-15  1.8e-15
Solution error estimates for segregated groups
1, 0.036, 0.15, 4.6e-07
Residual error estimates for segregated groups
82, 4.2e+03, 1.1e+04, 0.14
Segregated solver iteration 5.
Merged variables
Iter      SolEst     Damping    Stepsize #Res #Jac #Sol   LinErr   LinRes
   1         0.4   0.3500000         0.4   18   13   13  9.6e-14    3e-15
   2        0.27   0.3500000        0.27   19   14   14  1.2e-13  4.2e-15
   3        0.18   0.3500000        0.18   20   15   15  1.2e-13  2.7e-15
Velocity u2, Pressure p
Iter      SolEst     Damping    Stepsize #Res #Jac #Sol   LinErr   LinRes
   1       0.035   0.5000000       0.035   10    5    5  3.1e-15  7.8e-15
Displacement field
Iter      SolEst     Damping    Stepsize #Res #Jac #Sol   LinErr   LinRes
   1        0.17   0.5000000        0.17   10    5    5    4e-14  1.5e-12
Spatial mesh displacement
Iter      SolEst     Damping    Stepsize #Res #Jac #Sol   LinErr   LinRes
   1       7e-07   0.5000000       7e-07   10    5    5  1.7e-15  1.8e-15
Solution error estimates for segregated groups
0.31, 0.017, 0.084, 3.5e-07
Residual error estimates for segregated groups
32, 2.2e+03, 6.8e+03, 0.15
Segregated solver iteration 6.
Merged variables
Iter      SolEst     Damping    Stepsize #Res #Jac #Sol   LinErr   LinRes
   1        0.12   0.3500000        0.12   22   16   16    7e-14  3.8e-15
   2       0.077   0.3500000       0.077   23   17   17  8.7e-14  2.9e-15
   3        0.05   0.3500000        0.05   24   18   18  3.9e-14  3.2e-15
Velocity u2, Pressure p
Iter      SolEst     Damping    Stepsize #Res #Jac #Sol   LinErr   LinRes
   1       0.017   0.5000000       0.017   12    6    6    4e-15  7.8e-15
Displacement field
Iter      SolEst     Damping    Stepsize #Res #Jac #Sol   LinErr   LinRes
   1       0.095   0.5000000       0.095   12    6    6  1.8e-13  1.4e-12
Spatial mesh displacement
Iter      SolEst     Damping    Stepsize #Res #Jac #Sol   LinErr   LinRes
   1     4.9e-07   0.5000000     4.9e-07   12    6    6  1.6e-15  1.8e-15
Solution error estimates for segregated groups
0.086, 0.0085, 0.048, 2.4e-07
Residual error estimates for segregated groups
12, 1.1e+03, 4.2e+03, 0.14
```

```
Segregated solver iteration 7.
Merged variables
Iter      SolEst     Damping    Stepsize #Res #Jac #Sol   LinErr   LinRes
   1       0.033   0.3500000       0.033   26   19   19  9.8e-14  3.1e-15
   2       0.021   0.3500000       0.021   27   20   20    1e-13  2.8e-15
   3       0.014   0.3500000       0.014   28   21   21    6e-14  4.6e-15
Velocity u2, Pressure p
Iter      SolEst     Damping    Stepsize #Res #Jac #Sol   LinErr   LinRes
   1      0.0085   0.5000000      0.0085   14    7    7  3.8e-15  7.8e-15
Displacement field
Scales for dependent variables:
Auxiliary pressure (comp1.solid.lemm1.pw): 0.37
Displacement field (comp1.u): 2.9e-16
Iter      SolEst     Damping    Stepsize #Res #Jac #Sol   LinErr   LinRes
   1       0.054   0.5000000       0.054   14    7    7  2.1e-13  1.2e-12
Spatial mesh displacement
Iter      SolEst     Damping    Stepsize #Res #Jac #Sol   LinErr   LinRes
   1     3.3e-07   0.5000000     3.3e-07   14    7    7  1.6e-15  1.8e-15
Solution error estimates for segregated groups
0.024, 0.0042, 0.027, 1.6e-07
Residual error estimates for segregated groups
4.1, 5.5e+02, 2.6e+03, 0.13
Segregated solver iteration 8.
Merged variables
Iter      SolEst     Damping    Stepsize #Res #Jac #Sol   LinErr   LinRes
   1       0.009   0.3500000       0.009   30   22   22  1.1e-13  3.5e-15
   2      0.0059   0.3500000      0.0059   31   23   23  6.6e-14  3.2e-15
   3      0.0038   0.3500000      0.0038   32   24   24  6.5e-14  2.9e-15
Velocity u2, Pressure p
Iter      SolEst     Damping    Stepsize #Res #Jac #Sol   LinErr   LinRes
   1      0.0042   0.5000000      0.0042   16    8    8  4.8e-15  7.9e-15
Displacement field
Iter      SolEst     Damping    Stepsize #Res #Jac #Sol   LinErr   LinRes
   1        0.03   0.5000000        0.03   16    8    8  1.5e-13  1.2e-12
Spatial mesh displacement
Iter      SolEst     Damping    Stepsize #Res #Jac #Sol   LinErr   LinRes
   1     2.1e-07   0.5000000     2.1e-07   16    8    8  1.4e-15  1.8e-15
Solution error estimates for segregated groups
0.0065, 0.0021, 0.015, 1e-07
Residual error estimates for segregated groups
1.4, 2.8e+02, 1.8e+03, 0.14
Segregated solver iteration 9.
Merged variables
Iter      SolEst     Damping    Stepsize #Res #Jac #Sol   LinErr   LinRes
   1      0.0025   0.3500000      0.0025   34   25   25  5.8e-14    3e-15
   2      0.0016   0.3500000      0.0016   35   26   26  1.3e-13    3e-15
   3       0.001   0.3500000       0.001   36   27   27  1.8e-13  3.6e-15
Velocity u2, Pressure p
Iter      SolEst     Damping    Stepsize #Res #Jac #Sol   LinErr   LinRes
   1      0.0021   0.5000000      0.0021   18    9    9  5.3e-15    8e-15
Displacement field
Iter      SolEst     Damping    Stepsize #Res #Jac #Sol   LinErr   LinRes
   1       0.017   0.5000000       0.017   18    9    9  1.4e-13  1.2e-12
Spatial mesh displacement
Iter      SolEst     Damping    Stepsize #Res #Jac #Sol   LinErr   LinRes
   1     1.3e-07   0.5000000     1.3e-07   18    9    9  1.6e-15  1.8e-15
```

```
Solution error estimates for segregated groups
0.0018, 0.0011, 0.0085, 6.5e-08
Residual error estimates for segregated groups
0.44, 1.4e+02, 1.4e+03, 0.14
Segregated solver iteration 10.
Merged variables
Iter      SolEst     Damping    Stepsize #Res #Jac #Sol   LinErr   LinRes
   1     0.00068   0.3500000     0.00068   38   28   28  1.4e-13  2.9e-15
   2     0.00044   0.3500000     0.00044   39   29   29  1.3e-13    3e-15
   3     0.00029   0.3500000     0.00029   40   30   30  3.9e-14  3.1e-15
Velocity u2, Pressure p
Iter      SolEst     Damping    Stepsize #Res #Jac #Sol   LinErr   LinRes
   1      0.0011   0.5000000      0.0011   20   10   10  3.2e-15  7.9e-15
Displacement field
Iter      SolEst     Damping    Stepsize #Res #Jac #Sol   LinErr   LinRes
   1      0.0094   0.5000000      0.0094   20   10   10  1.3e-13  1.2e-12
Spatial mesh displacement
Iter      SolEst     Damping    Stepsize #Res #Jac #Sol   LinErr   LinRes
   1       8e-08   0.5000000       8e-08   20   10   10    2e-15  1.9e-15
Solution error estimates for segregated groups
0.00049, 0.00053, 0.0047, 4e-08
Residual error estimates for segregated groups
0.14, 69, 1.2e+03, 0.14
Segregated solver iteration 11.
Merged variables
Iter      SolEst     Damping    Stepsize #Res #Jac #Sol   LinErr   LinRes
   1     0.00019   0.3500000     0.00019   42   31   31  6.3e-14  2.9e-15
   2     0.00012   0.3500000     0.00012   43   32   32  1.1e-13  2.8e-15
   3     7.9e-05   0.3500000     7.9e-05   44   33   33  8.8e-14  3.1e-15
Velocity u2, Pressure p
Iter      SolEst     Damping    Stepsize #Res #Jac #Sol   LinErr   LinRes
   1     0.00053   0.5000000     0.00053   22   11   11  2.2e-15  7.7e-15
Displacement field
Iter      SolEst     Damping    Stepsize #Res #Jac #Sol   LinErr   LinRes
   1      0.0051   0.5000000      0.0051   22   11   11  1.2e-13  1.2e-12
Spatial mesh displacement
Iter      SolEst     Damping    Stepsize #Res #Jac #Sol   LinErr   LinRes
   1     4.8e-08   0.5000000     4.8e-08   22   11   11  1.3e-15  1.8e-15
Solution error estimates for segregated groups
0.00014, 0.00026, 0.0026, 2.4e-08
Residual error estimates for segregated groups
0.043, 35, 1.2e+03, 0.14
Segregated solver iteration 12.
Merged variables
Iter      SolEst     Damping    Stepsize #Res #Jac #Sol   LinErr   LinRes
   1     5.1e-05   0.3500000     5.1e-05   46   34   34  7.3e-14  4.2e-15
   2     3.3e-05   0.3500000     3.3e-05   47   35   35  1.6e-13  2.9e-15
   3     2.2e-05   0.3500000     2.2e-05   48   36   36  8.9e-14  3.6e-15
Velocity u2, Pressure p
Iter      SolEst     Damping    Stepsize #Res #Jac #Sol   LinErr   LinRes
   1     0.00026   0.5000000     0.00026   24   12   12  2.4e-15  7.9e-15
Displacement field
Iter      SolEst     Damping    Stepsize #Res #Jac #Sol   LinErr   LinRes
   1      0.0028   0.5000000      0.0028   24   12   12  1.3e-13  1.2e-12
Spatial mesh displacement
Iter      SolEst     Damping    Stepsize #Res #Jac #Sol   LinErr   LinRes
```

```
   1     2.8e-08   0.5000000     2.8e-08   24   12   12  1.2e-15  1.8e-15
Solution error estimates for segregated groups
3.7e-05, 0.00013, 0.0014, 1.4e-08
Residual error estimates for segregated groups
0.013, 17, 1.2e+03, 0.14
Segregated solver iteration 13.
Merged variables
Iter      SolEst     Damping    Stepsize #Res #Jac #Sol   LinErr   LinRes
   1     1.4e-05   0.3500000     1.4e-05   50   37   37  5.8e-14  2.8e-15
   2     9.2e-06   0.3500000     9.2e-06   51   38   38  1.3e-13  3.6e-15
   3       6e-06   0.3500000       6e-06   52   39   39  1.1e-13  2.7e-15
Velocity u2, Pressure p
Iter      SolEst     Damping    Stepsize #Res #Jac #Sol   LinErr   LinRes
   1     0.00013   0.5000000     0.00013   26   13   13  2.2e-15  7.8e-15
Displacement field
Iter      SolEst     Damping    Stepsize #Res #Jac #Sol   LinErr   LinRes
   1      0.0015   0.5000000      0.0015   26   13   13  1.9e-13  1.2e-12
Spatial mesh displacement
Iter      SolEst     Damping    Stepsize #Res #Jac #Sol   LinErr   LinRes
   1     1.7e-08   0.5000000     1.7e-08   26   13   13  1.5e-15  1.8e-15
Solution error estimates for segregated groups
1e-05, 6.6e-05, 0.00076, 8.3e-09
Residual error estimates for segregated groups
0.004, 8.7, 1.2e+03, 0.15
Solution time: 155 s. (2 minutes, 35 seconds)
Physical memory: 4.68 GB
Virtual memory: 25.5 GB
Ended at Jan 31, 2025, 8:20:30 PM.
----- Stationary Solver 1 in study1/Approach Curve Solver (sol1) -------------->
```

d=2120 (su78)

GENERAL

Description	Value
Solution	d=2120

LOG

```
<---- Compile Equations: Stationary in study1/Approach Curve Solver (sol1) -----
Started at Jan 31, 2025, 8:20:31 PM.
Parameter d = 2.12E-6 (m).
Geometry shape function: Linear serendipity
Number of vertex elements: 18
Number of boundary elements: 6126
Number of elements: 154596
Minimum element quality: 0.4734
Time: 8 s.
Physical memory: 3 GB
Virtual memory: 15.29 GB
Ended at Jan 31, 2025, 8:20:39 PM.
----- Compile Equations: Stationary in study1/Approach Curve Solver (sol1) ---->

<---- Dependent Variables 1 in study1/Approach Curve Solver (sol1) -------------
Started at Jan 31, 2025, 8:20:39 PM.
Solution time: 0 s.
Physical memory: 3 GB
Virtual memory: 15.22 GB
Ended at Jan 31, 2025, 8:20:39 PM.
----- Dependent Variables 1 in study1/Approach Curve Solver (sol1) ------------>

<---- Stationary Solver 1 in study1/Approach Curve Solver (sol1) ---------------
Started at Jan 31, 2025, 8:20:39 PM.
Segregated solver
Segregated Step 11 does not have any components solved for.
Number of degrees of freedom solved for: 784822 (plus 11675 internal DOFs).
Segregated solver iteration 1.
Merged variables
Nonsymmetric matrix found.
Scales for dependent variables:
Concentration (comp1.Cl): 1.4e+02
Concentration (comp1.K): 1.4e+02
Electric potential (comp1.V): 1.9e+02
Orthonormal null-space function used.
Iter      SolEst     Damping    Stepsize #Res #Jac #Sol   LinErr   LinRes
   1        0.83   0.3500000        0.83    2    1    1  4.4e-11  2.7e-15
   2        0.81   0.3500000        0.81    3    2    2  3.5e-11  2.8e-15
   3         0.8   0.3500000         0.8    4    3    3  3.1e-11  2.7e-15
Velocity u2, Pressure p
Nonsymmetric matrix found.
Scales for dependent variables:
Pressure (comp1.p): 1
Velocity field (spatial frame) (comp1.u2): 0.00018
Orthonormal null-space function used.
Iter      SolEst     Damping    Stepsize #Res #Jac #Sol   LinErr   LinRes
   1         1.1   0.5000000         1.1    2    1    1  1.6e-15  2.3e-15
Displacement field
Nonsymmetric matrix found.
Scales for dependent variables:
Auxiliary pressure (comp1.solid.lemm1.pw): 1
Displacement field (comp1.u): 2.8e-18
Orthonormal null-space function used.
Iter      SolEst     Damping    Stepsize #Res #Jac #Sol   LinErr   LinRes
   1         1.9   0.5000000         1.9    2    1    1  4.2e-14  1.5e-12
Spatial mesh displacement
```

```
Symmetric matrices found.
Scales for dependent variables:
Spatial mesh displacement (comp1.spatial.disp): 1.2e-09
Orthonormal null-space function used.
Iter      SolEst     Damping    Stepsize #Res #Jac #Sol   LinErr   LinRes
   1     7.5e-07   0.5000000     7.5e-07    2    1    1  1.8e-15  1.8e-15
Solution error estimates for segregated groups
1.4, 0.54, 0.95, 3.7e-07
Residual error estimates for segregated groups
7.8e+03, 8.2e+04, 2.1e+04, 0.13
Segregated solver iteration 2.
Merged variables
Scales for dependent variables:
Concentration (comp1.Cl): 1.4e+02
Concentration (comp1.K): 1.4e+02
Electric potential (comp1.V): 0.064
Iter      SolEst     Damping    Stepsize #Res #Jac #Sol   LinErr   LinRes
   1        0.78   0.3500000        0.78    6    4    4  4.1e-14  2.7e-15
   2        0.77   0.3500000        0.77    7    5    5  7.1e-14  2.8e-15
   3        0.76   0.3500000        0.76    8    6    6  3.3e-14  2.7e-15
Velocity u2, Pressure p
Scales for dependent variables:
Pressure (comp1.p): 3.6e+02
Velocity field (spatial frame) (comp1.u2): 0.00073
Iter      SolEst     Damping    Stepsize #Res #Jac #Sol   LinErr   LinRes
   1        0.36   0.5000000        0.36    4    2    2  2.8e-15  7.7e-15
Displacement field
Iter      SolEst     Damping    Stepsize #Res #Jac #Sol   LinErr   LinRes
   1        0.95   0.5000000        0.95    4    2    2  6.4e-14  1.5e-12
Spatial mesh displacement
Iter      SolEst     Damping    Stepsize #Res #Jac #Sol   LinErr   LinRes
   1     1.1e-06   0.5000000     1.1e-06    4    2    2  1.4e-15  1.9e-15
Solution error estimates for segregated groups
1.3, 0.18, 0.48, 5.6e-07
Residual error estimates for segregated groups
7.7e+02, 1.4e+04, 2.2e+04, 0.13
Segregated solver iteration 3.
Merged variables
Iter      SolEst     Damping    Stepsize #Res #Jac #Sol   LinErr   LinRes
   1        0.74   0.3500000        0.74   10    7    7  4.3e-14  2.7e-15
   2        0.73   0.3500000        0.73   11    8    8  6.9e-14  2.7e-15
   3        0.72   0.3500000        0.72   12    9    9  3.4e-14  2.7e-15
Velocity u2, Pressure p
Iter      SolEst     Damping    Stepsize #Res #Jac #Sol   LinErr   LinRes
   1        0.15   0.5000000        0.15    6    3    3  3.5e-15  7.8e-15
Displacement field
Iter      SolEst     Damping    Stepsize #Res #Jac #Sol   LinErr   LinRes
   1        0.52   0.5000000        0.52    6    3    3  3.9e-14  1.5e-12
Spatial mesh displacement
Iter      SolEst     Damping    Stepsize #Res #Jac #Sol   LinErr   LinRes
   1     1.1e-06   0.5000000     1.1e-06    6    3    3  1.7e-15  1.9e-15
Solution error estimates for segregated groups
1.2, 0.077, 0.26, 5.6e-07
Residual error estimates for segregated groups
2.1e+02, 8.1e+03, 1.6e+04, 0.14
Segregated solver iteration 4.
```

```
Merged variables
Iter      SolEst     Damping    Stepsize #Res #Jac #Sol   LinErr   LinRes
   1         0.7   0.3500000         0.7   14   10   10  1.2e-13  2.7e-15
   2        0.68   0.3500000        0.68   15   11   11  8.7e-14  2.8e-15
   3        0.59   0.3500000        0.59   16   12   12  6.6e-14  2.8e-15
Velocity u2, Pressure p
Iter      SolEst     Damping    Stepsize #Res #Jac #Sol   LinErr   LinRes
   1       0.072   0.5000000       0.072    8    4    4  2.8e-15  7.8e-15
Displacement field
Iter      SolEst     Damping    Stepsize #Res #Jac #Sol   LinErr   LinRes
   1        0.29   0.5000000        0.29    8    4    4  7.9e-14  1.5e-12
Spatial mesh displacement
Iter      SolEst     Damping    Stepsize #Res #Jac #Sol   LinErr   LinRes
   1     9.4e-07   0.5000000     9.4e-07    8    4    4  1.7e-15  1.8e-15
Solution error estimates for segregated groups
1, 0.036, 0.15, 4.7e-07
Residual error estimates for segregated groups
82, 4.3e+03, 1.1e+04, 0.14
Segregated solver iteration 5.
Merged variables
Iter      SolEst     Damping    Stepsize #Res #Jac #Sol   LinErr   LinRes
   1         0.4   0.3500000         0.4   18   13   13    1e-13  2.7e-15
   2        0.27   0.3500000        0.27   19   14   14  7.5e-14  2.7e-15
   3        0.18   0.3500000        0.18   20   15   15  1.2e-13  2.7e-15
Velocity u2, Pressure p
Iter      SolEst     Damping    Stepsize #Res #Jac #Sol   LinErr   LinRes
   1       0.035   0.5000000       0.035   10    5    5  2.1e-15  7.8e-15
Displacement field
Iter      SolEst     Damping    Stepsize #Res #Jac #Sol   LinErr   LinRes
   1        0.17   0.5000000        0.17   10    5    5  7.2e-14  1.5e-12
Spatial mesh displacement
Iter      SolEst     Damping    Stepsize #Res #Jac #Sol   LinErr   LinRes
   1       7e-07   0.5000000       7e-07   10    5    5  1.5e-15  1.9e-15
Solution error estimates for segregated groups
0.31, 0.017, 0.084, 3.5e-07
Residual error estimates for segregated groups
32, 2.2e+03, 6.8e+03, 0.14
Segregated solver iteration 6.
Merged variables
Iter      SolEst     Damping    Stepsize #Res #Jac #Sol   LinErr   LinRes
   1        0.12   0.3500000        0.12   22   16   16  5.6e-14  2.8e-15
   2       0.077   0.3500000       0.077   23   17   17  7.5e-14  2.7e-15
   3        0.05   0.3500000        0.05   24   18   18  9.7e-14  2.7e-15
Velocity u2, Pressure p
Iter      SolEst     Damping    Stepsize #Res #Jac #Sol   LinErr   LinRes
   1       0.017   0.5000000       0.017   12    6    6  4.3e-15  7.8e-15
Displacement field
Scales for dependent variables:
Auxiliary pressure (comp1.solid.lemm1.pw): 0.35
Displacement field (comp1.u): 2.8e-16
Iter      SolEst     Damping    Stepsize #Res #Jac #Sol   LinErr   LinRes
   1       0.095   0.5000000       0.095   12    6    6  1.2e-13  1.2e-12
Spatial mesh displacement
Iter      SolEst     Damping    Stepsize #Res #Jac #Sol   LinErr   LinRes
   1     4.9e-07   0.5000000     4.9e-07   12    6    6  1.8e-15  1.9e-15
Solution error estimates for segregated groups
```

```
0.086, 0.0085, 0.048, 2.5e-07
Residual error estimates for segregated groups
12, 1.1e+03, 4.2e+03, 0.14
Segregated solver iteration 7.
Merged variables
Iter      SolEst     Damping    Stepsize #Res #Jac #Sol   LinErr   LinRes
   1       0.033   0.3500000       0.033   26   19   19  8.9e-14  2.8e-15
   2       0.021   0.3500000       0.021   27   20   20  6.7e-14  2.6e-15
   3       0.014   0.3500000       0.014   28   21   21  9.7e-14  2.7e-15
Velocity u2, Pressure p
Iter      SolEst     Damping    Stepsize #Res #Jac #Sol   LinErr   LinRes
   1      0.0085   0.5000000      0.0085   14    7    7  2.4e-15  7.7e-15
Displacement field
Iter      SolEst     Damping    Stepsize #Res #Jac #Sol   LinErr   LinRes
   1       0.054   0.5000000       0.054   14    7    7    1e-13  1.2e-12
Spatial mesh displacement
Iter      SolEst     Damping    Stepsize #Res #Jac #Sol   LinErr   LinRes
   1     3.3e-07   0.5000000     3.3e-07   14    7    7  1.4e-15  1.9e-15
Solution error estimates for segregated groups
0.024, 0.0042, 0.027, 1.6e-07
Residual error estimates for segregated groups
4.1, 5.6e+02, 2.6e+03, 0.14
Segregated solver iteration 8.
Merged variables
Iter      SolEst     Damping    Stepsize #Res #Jac #Sol   LinErr   LinRes
   1       0.009   0.3500000       0.009   30   22   22  4.3e-14  2.7e-15
   2      0.0059   0.3500000      0.0059   31   23   23    8e-14  2.7e-15
   3      0.0038   0.3500000      0.0038   32   24   24    8e-14  2.7e-15
Velocity u2, Pressure p
Iter      SolEst     Damping    Stepsize #Res #Jac #Sol   LinErr   LinRes
   1      0.0042   0.5000000      0.0042   16    8    8  2.8e-15  7.9e-15
Displacement field
Iter      SolEst     Damping    Stepsize #Res #Jac #Sol   LinErr   LinRes
   1        0.03   0.5000000        0.03   16    8    8  1.2e-13  1.2e-12
Spatial mesh displacement
Iter      SolEst     Damping    Stepsize #Res #Jac #Sol   LinErr   LinRes
   1     2.1e-07   0.5000000     2.1e-07   16    8    8  1.5e-15  1.8e-15
Solution error estimates for segregated groups
0.0065, 0.0021, 0.015, 1.1e-07
Residual error estimates for segregated groups
1.4, 2.8e+02, 1.8e+03, 0.14
Segregated solver iteration 9.
Merged variables
Iter      SolEst     Damping    Stepsize #Res #Jac #Sol   LinErr   LinRes
   1      0.0025   0.3500000      0.0025   34   25   25  1.1e-13  2.7e-15
   2      0.0016   0.3500000      0.0016   35   26   26  9.1e-14  2.9e-15
   3       0.001   0.3500000       0.001   36   27   27  1.6e-13  2.7e-15
Velocity u2, Pressure p
Iter      SolEst     Damping    Stepsize #Res #Jac #Sol   LinErr   LinRes
   1      0.0021   0.5000000      0.0021   18    9    9  3.3e-15  7.8e-15
Displacement field
Iter      SolEst     Damping    Stepsize #Res #Jac #Sol   LinErr   LinRes
   1       0.017   0.5000000       0.017   18    9    9    1e-13  1.2e-12
Spatial mesh displacement
Iter      SolEst     Damping    Stepsize #Res #Jac #Sol   LinErr   LinRes
   1     1.3e-07   0.5000000     1.3e-07   18    9    9  1.6e-15  1.9e-15
```

```
Solution error estimates for segregated groups
0.0018, 0.0011, 0.0085, 6.6e-08
Residual error estimates for segregated groups
0.44, 1.4e+02, 1.4e+03, 0.14
Segregated solver iteration 10.
Merged variables
Iter      SolEst     Damping    Stepsize #Res #Jac #Sol   LinErr   LinRes
   1     0.00068   0.3500000     0.00068   38   28   28  7.1e-14  2.7e-15
   2     0.00044   0.3500000     0.00044   39   29   29  9.1e-14  2.7e-15
   3     0.00029   0.3500000     0.00029   40   30   30  7.4e-14  2.8e-15
Velocity u2, Pressure p
Iter      SolEst     Damping    Stepsize #Res #Jac #Sol   LinErr   LinRes
   1      0.0011   0.5000000      0.0011   20   10   10  2.5e-15  7.8e-15
Displacement field
Iter      SolEst     Damping    Stepsize #Res #Jac #Sol   LinErr   LinRes
   1      0.0094   0.5000000      0.0094   20   10   10  1.2e-13  1.2e-12
Spatial mesh displacement
Iter      SolEst     Damping    Stepsize #Res #Jac #Sol   LinErr   LinRes
   1       8e-08   0.5000000       8e-08   20   10   10  1.4e-15  1.9e-15
Solution error estimates for segregated groups
0.00049, 0.00053, 0.0047, 4e-08
Residual error estimates for segregated groups
0.14, 70, 1.2e+03, 0.15
Segregated solver iteration 11.
Merged variables
Iter      SolEst     Damping    Stepsize #Res #Jac #Sol   LinErr   LinRes
   1     0.00019   0.3500000     0.00019   42   31   31  1.2e-13  2.8e-15
   2     0.00012   0.3500000     0.00012   43   32   32    8e-14  2.7e-15
   3     7.9e-05   0.3500000     7.9e-05   44   33   33  8.5e-14  2.7e-15
Velocity u2, Pressure p
Iter      SolEst     Damping    Stepsize #Res #Jac #Sol   LinErr   LinRes
   1     0.00053   0.5000000     0.00053   22   11   11  3.2e-15  7.8e-15
Displacement field
Iter      SolEst     Damping    Stepsize #Res #Jac #Sol   LinErr   LinRes
   1      0.0051   0.5000000      0.0051   22   11   11  1.4e-13  1.2e-12
Spatial mesh displacement
Iter      SolEst     Damping    Stepsize #Res #Jac #Sol   LinErr   LinRes
   1     4.8e-08   0.5000000     4.8e-08   22   11   11  1.6e-15  1.9e-15
Solution error estimates for segregated groups
0.00014, 0.00026, 0.0026, 2.4e-08
Residual error estimates for segregated groups
0.043, 35, 1.2e+03, 0.14
Segregated solver iteration 12.
Merged variables
Iter      SolEst     Damping    Stepsize #Res #Jac #Sol   LinErr   LinRes
   1     5.1e-05   0.3500000     5.1e-05   46   34   34    6e-14  2.7e-15
   2     3.3e-05   0.3500000     3.3e-05   47   35   35  9.3e-14  2.7e-15
   3     2.2e-05   0.3500000     2.2e-05   48   36   36    8e-14  2.6e-15
Velocity u2, Pressure p
Iter      SolEst     Damping    Stepsize #Res #Jac #Sol   LinErr   LinRes
   1     0.00026   0.5000000     0.00026   24   12   12  3.1e-15  7.8e-15
Displacement field
Iter      SolEst     Damping    Stepsize #Res #Jac #Sol   LinErr   LinRes
   1      0.0028   0.5000000      0.0028   24   12   12  1.9e-13  1.2e-12
Spatial mesh displacement
Iter      SolEst     Damping    Stepsize #Res #Jac #Sol   LinErr   LinRes
```

```
   1     2.9e-08   0.5000000     2.9e-08   24   12   12  1.5e-15  1.8e-15
Solution error estimates for segregated groups
3.7e-05, 0.00013, 0.0014, 1.4e-08
Residual error estimates for segregated groups
0.013, 18, 1.2e+03, 0.14
Segregated solver iteration 13.
Merged variables
Iter      SolEst     Damping    Stepsize #Res #Jac #Sol   LinErr   LinRes
   1     1.4e-05   0.3500000     1.4e-05   50   37   37  6.2e-14  2.7e-15
   2     9.2e-06   0.3500000     9.2e-06   51   38   38  8.5e-14  2.8e-15
   3       6e-06   0.3500000       6e-06   52   39   39  9.6e-14  2.7e-15
Velocity u2, Pressure p
Iter      SolEst     Damping    Stepsize #Res #Jac #Sol   LinErr   LinRes
   1     0.00013   0.5000000     0.00013   26   13   13  3.5e-15  7.7e-15
Displacement field
Iter      SolEst     Damping    Stepsize #Res #Jac #Sol   LinErr   LinRes
   1      0.0015   0.5000000      0.0015   26   13   13    1e-13  1.2e-12
Spatial mesh displacement
Iter      SolEst     Damping    Stepsize #Res #Jac #Sol   LinErr   LinRes
   1     1.7e-08   0.5000000     1.7e-08   26   13   13  1.5e-15  1.9e-15
Solution error estimates for segregated groups
1e-05, 6.6e-05, 0.00076, 8.3e-09
Residual error estimates for segregated groups
0.004, 8.8, 1.2e+03, 0.14
Solution time: 155 s. (2 minutes, 35 seconds)
Physical memory: 4.67 GB
Virtual memory: 25.56 GB
Ended at Jan 31, 2025, 8:23:14 PM.
----- Stationary Solver 1 in study1/Approach Curve Solver (sol1) -------------->
```

d=2115 (su79)

GENERAL

Description	Value
Solution	d=2115

LOG

```
<---- Compile Equations: Stationary in study1/Approach Curve Solver (sol1) -----
Started at Jan 31, 2025, 8:23:14 PM.
Parameter d = 2.115E-6 (m).
Geometry shape function: Linear serendipity
Number of vertex elements: 18
Number of boundary elements: 6124
Number of elements: 154308
Minimum element quality: 0.4862
Time: 8 s.
Physical memory: 3 GB
Virtual memory: 15.27 GB
Ended at Jan 31, 2025, 8:23:23 PM.
----- Compile Equations: Stationary in study1/Approach Curve Solver (sol1) ---->

<---- Dependent Variables 1 in study1/Approach Curve Solver (sol1) -------------
Started at Jan 31, 2025, 8:23:23 PM.
Solution time: 0 s.
Physical memory: 3 GB
Virtual memory: 15.21 GB
Ended at Jan 31, 2025, 8:23:23 PM.
----- Dependent Variables 1 in study1/Approach Curve Solver (sol1) ------------>

<---- Stationary Solver 1 in study1/Approach Curve Solver (sol1) ---------------
Started at Jan 31, 2025, 8:23:23 PM.
Segregated solver
Segregated Step 11 does not have any components solved for.
Number of degrees of freedom solved for: 783169 (plus 11671 internal DOFs).
Segregated solver iteration 1.
Merged variables
Nonsymmetric matrix found.
Scales for dependent variables:
Concentration (comp1.Cl): 1.4e+02
Concentration (comp1.K): 1.4e+02
Electric potential (comp1.V): 1.9e+02
Orthonormal null-space function used.
Iter      SolEst     Damping    Stepsize #Res #Jac #Sol   LinErr   LinRes
   1        0.83   0.3500000        0.83    2    1    1  2.1e-11  2.8e-15
   2        0.81   0.3500000        0.81    3    2    2  5.5e-11  2.7e-15
   3         0.8   0.3500000         0.8    4    3    3  2.7e-11  2.7e-15
Velocity u2, Pressure p
Nonsymmetric matrix found.
Scales for dependent variables:
Pressure (comp1.p): 1
Velocity field (spatial frame) (comp1.u2): 0.00018
Orthonormal null-space function used.
Iter      SolEst     Damping    Stepsize #Res #Jac #Sol   LinErr   LinRes
   1         1.1   0.5000000         1.1    2    1    1  1.5e-15  2.3e-15
Displacement field
Nonsymmetric matrix found.
Scales for dependent variables:
Auxiliary pressure (comp1.solid.lemm1.pw): 1
Displacement field (comp1.u): 2.8e-18
Orthonormal null-space function used.
Iter      SolEst     Damping    Stepsize #Res #Jac #Sol   LinErr   LinRes
   1         1.9   0.5000000         1.9    2    1    1  3.5e-14  1.5e-12
Spatial mesh displacement
```

```
Symmetric matrices found.
Scales for dependent variables:
Spatial mesh displacement (comp1.spatial.disp): 1.2e-09
Orthonormal null-space function used.
Iter      SolEst     Damping    Stepsize #Res #Jac #Sol   LinErr   LinRes
   1     7.5e-07   0.5000000     7.5e-07    2    1    1  1.4e-15  1.8e-15
Solution error estimates for segregated groups
1.4, 0.54, 0.95, 3.8e-07
Residual error estimates for segregated groups
7.8e+03, 8.2e+04, 2.2e+04, 0.12
Segregated solver iteration 2.
Merged variables
Scales for dependent variables:
Concentration (comp1.Cl): 1.4e+02
Concentration (comp1.K): 1.4e+02
Electric potential (comp1.V): 0.064
Iter      SolEst     Damping    Stepsize #Res #Jac #Sol   LinErr   LinRes
   1        0.78   0.3500000        0.78    6    4    4  1.1e-13  2.9e-15
   2        0.77   0.3500000        0.77    7    5    5  9.1e-14  3.4e-15
   3        0.76   0.3500000        0.76    8    6    6  1.2e-13  3.1e-15
Velocity u2, Pressure p
Scales for dependent variables:
Pressure (comp1.p): 3.6e+02
Velocity field (spatial frame) (comp1.u2): 0.00073
Iter      SolEst     Damping    Stepsize #Res #Jac #Sol   LinErr   LinRes
   1        0.36   0.5000000        0.36    4    2    2  2.5e-15  7.9e-15
Displacement field
Iter      SolEst     Damping    Stepsize #Res #Jac #Sol   LinErr   LinRes
   1        0.95   0.5000000        0.95    4    2    2  1.5e-13  1.5e-12
Spatial mesh displacement
Iter      SolEst     Damping    Stepsize #Res #Jac #Sol   LinErr   LinRes
   1     1.1e-06   0.5000000     1.1e-06    4    2    2    2e-15  1.8e-15
Solution error estimates for segregated groups
1.3, 0.18, 0.48, 5.6e-07
Residual error estimates for segregated groups
7.7e+02, 1.6e+04, 2.2e+04, 0.14
Segregated solver iteration 3.
Merged variables
Iter      SolEst     Damping    Stepsize #Res #Jac #Sol   LinErr   LinRes
   1        0.74   0.3500000        0.74   10    7    7  5.4e-14  2.9e-15
   2        0.73   0.3500000        0.73   11    8    8  8.9e-14  2.7e-15
   3        0.72   0.3500000        0.72   12    9    9  1.1e-13    3e-15
Velocity u2, Pressure p
Iter      SolEst     Damping    Stepsize #Res #Jac #Sol   LinErr   LinRes
   1        0.15   0.5000000        0.15    6    3    3  1.9e-15  7.9e-15
Displacement field
Iter      SolEst     Damping    Stepsize #Res #Jac #Sol   LinErr   LinRes
   1        0.52   0.5000000        0.52    6    3    3  5.3e-14  1.5e-12
Spatial mesh displacement
Iter      SolEst     Damping    Stepsize #Res #Jac #Sol   LinErr   LinRes
   1     1.1e-06   0.5000000     1.1e-06    6    3    3  1.3e-15  1.8e-15
Solution error estimates for segregated groups
1.2, 0.077, 0.26, 5.6e-07
Residual error estimates for segregated groups
2.1e+02, 9.3e+03, 1.6e+04, 0.14
Segregated solver iteration 4.
```

```
Merged variables
Iter      SolEst     Damping    Stepsize #Res #Jac #Sol   LinErr   LinRes
   1         0.7   0.3500000         0.7   14   10   10  1.1e-13  2.9e-15
   2        0.68   0.3500000        0.68   15   11   11  4.9e-14  2.7e-15
   3        0.59   0.3500000        0.59   16   12   12  5.6e-14  2.6e-15
Velocity u2, Pressure p
Iter      SolEst     Damping    Stepsize #Res #Jac #Sol   LinErr   LinRes
   1       0.072   0.5000000       0.072    8    4    4  4.8e-15  8.1e-15
Displacement field
Iter      SolEst     Damping    Stepsize #Res #Jac #Sol   LinErr   LinRes
   1        0.29   0.5000000        0.29    8    4    4  1.2e-13  1.5e-12
Spatial mesh displacement
Iter      SolEst     Damping    Stepsize #Res #Jac #Sol   LinErr   LinRes
   1     9.4e-07   0.5000000     9.4e-07    8    4    4  1.5e-15  1.8e-15
Solution error estimates for segregated groups
1, 0.036, 0.15, 4.7e-07
Residual error estimates for segregated groups
82, 4.9e+03, 1.1e+04, 0.13
Segregated solver iteration 5.
Merged variables
Iter      SolEst     Damping    Stepsize #Res #Jac #Sol   LinErr   LinRes
   1         0.4   0.3500000         0.4   18   13   13  1.1e-13  2.7e-15
   2        0.27   0.3500000        0.27   19   14   14  5.6e-14  2.8e-15
   3        0.18   0.3500000        0.18   20   15   15  7.9e-14  2.7e-15
Velocity u2, Pressure p
Iter      SolEst     Damping    Stepsize #Res #Jac #Sol   LinErr   LinRes
   1       0.035   0.5000000       0.035   10    5    5  2.2e-15  7.9e-15
Displacement field
Iter      SolEst     Damping    Stepsize #Res #Jac #Sol   LinErr   LinRes
   1        0.17   0.5000000        0.17   10    5    5    9e-14  1.5e-12
Spatial mesh displacement
Iter      SolEst     Damping    Stepsize #Res #Jac #Sol   LinErr   LinRes
   1       7e-07   0.5000000       7e-07   10    5    5  1.2e-15  1.8e-15
Solution error estimates for segregated groups
0.31, 0.017, 0.084, 3.5e-07
Residual error estimates for segregated groups
32, 2.5e+03, 6.8e+03, 0.14
Segregated solver iteration 6.
Merged variables
Iter      SolEst     Damping    Stepsize #Res #Jac #Sol   LinErr   LinRes
   1        0.12   0.3500000        0.12   22   16   16  9.7e-14  2.8e-15
   2       0.077   0.3500000       0.077   23   17   17    1e-13  2.7e-15
   3        0.05   0.3500000        0.05   24   18   18  5.3e-14  2.7e-15
Velocity u2, Pressure p
Iter      SolEst     Damping    Stepsize #Res #Jac #Sol   LinErr   LinRes
   1       0.017   0.5000000       0.017   12    6    6    3e-15  7.8e-15
Displacement field
Scales for dependent variables:
Auxiliary pressure (comp1.solid.lemm1.pw): 0.35
Displacement field (comp1.u): 2.8e-16
Iter      SolEst     Damping    Stepsize #Res #Jac #Sol   LinErr   LinRes
   1       0.095   0.5000000       0.095   12    6    6  1.4e-13  1.2e-12
Spatial mesh displacement
Iter      SolEst     Damping    Stepsize #Res #Jac #Sol   LinErr   LinRes
   1     4.9e-07   0.5000000     4.9e-07   12    6    6  1.7e-15  1.8e-15
Solution error estimates for segregated groups
```

```
0.086, 0.0085, 0.048, 2.5e-07
Residual error estimates for segregated groups
12, 1.3e+03, 4.2e+03, 0.14
Segregated solver iteration 7.
Merged variables
Iter      SolEst     Damping    Stepsize #Res #Jac #Sol   LinErr   LinRes
   1       0.033   0.3500000       0.033   26   19   19  6.8e-14  2.8e-15
   2       0.021   0.3500000       0.021   27   20   20  5.4e-14  2.7e-15
   3       0.014   0.3500000       0.014   28   21   21  1.2e-13  2.7e-15
Velocity u2, Pressure p
Iter      SolEst     Damping    Stepsize #Res #Jac #Sol   LinErr   LinRes
   1      0.0085   0.5000000      0.0085   14    7    7  1.9e-15  7.9e-15
Displacement field
Iter      SolEst     Damping    Stepsize #Res #Jac #Sol   LinErr   LinRes
   1       0.054   0.5000000       0.054   14    7    7    1e-13  1.2e-12
Spatial mesh displacement
Iter      SolEst     Damping    Stepsize #Res #Jac #Sol   LinErr   LinRes
   1     3.3e-07   0.5000000     3.3e-07   14    7    7  1.5e-15  1.9e-15
Solution error estimates for segregated groups
0.024, 0.0042, 0.027, 1.6e-07
Residual error estimates for segregated groups
4.1, 6.5e+02, 2.6e+03, 0.14
Segregated solver iteration 8.
Merged variables
Iter      SolEst     Damping    Stepsize #Res #Jac #Sol   LinErr   LinRes
   1       0.009   0.3500000       0.009   30   22   22  6.1e-14  2.7e-15
   2      0.0058   0.3500000      0.0058   31   23   23  9.6e-14  2.6e-15
   3      0.0038   0.3500000      0.0038   32   24   24  4.1e-14  2.8e-15
Velocity u2, Pressure p
Iter      SolEst     Damping    Stepsize #Res #Jac #Sol   LinErr   LinRes
   1      0.0042   0.5000000      0.0042   16    8    8  3.5e-15  7.8e-15
Displacement field
Iter      SolEst     Damping    Stepsize #Res #Jac #Sol   LinErr   LinRes
   1        0.03   0.5000000        0.03   16    8    8  1.1e-13  1.2e-12
Spatial mesh displacement
Iter      SolEst     Damping    Stepsize #Res #Jac #Sol   LinErr   LinRes
   1     2.1e-07   0.5000000     2.1e-07   16    8    8  1.5e-15  1.8e-15
Solution error estimates for segregated groups
0.0065, 0.0021, 0.015, 1.1e-07
Residual error estimates for segregated groups
1.4, 3.3e+02, 1.8e+03, 0.15
Segregated solver iteration 9.
Merged variables
Iter      SolEst     Damping    Stepsize #Res #Jac #Sol   LinErr   LinRes
   1      0.0025   0.3500000      0.0025   34   25   25  7.1e-14  2.7e-15
   2      0.0016   0.3500000      0.0016   35   26   26  8.5e-14  2.8e-15
   3       0.001   0.3500000       0.001   36   27   27  3.7e-14  2.7e-15
Velocity u2, Pressure p
Iter      SolEst     Damping    Stepsize #Res #Jac #Sol   LinErr   LinRes
   1      0.0021   0.5000000      0.0021   18    9    9  2.4e-15  7.8e-15
Displacement field
Iter      SolEst     Damping    Stepsize #Res #Jac #Sol   LinErr   LinRes
   1       0.017   0.5000000       0.017   18    9    9  1.5e-13  1.2e-12
Spatial mesh displacement
Iter      SolEst     Damping    Stepsize #Res #Jac #Sol   LinErr   LinRes
   1     1.3e-07   0.5000000     1.3e-07   18    9    9  1.3e-15  1.8e-15
```

```
Solution error estimates for segregated groups
0.0018, 0.0011, 0.0085, 6.6e-08
Residual error estimates for segregated groups
0.44, 1.6e+02, 1.4e+03, 0.14
Segregated solver iteration 10.
Merged variables
Iter      SolEst     Damping    Stepsize #Res #Jac #Sol   LinErr   LinRes
   1     0.00068   0.3500000     0.00068   38   28   28  6.6e-14  2.6e-15
   2     0.00044   0.3500000     0.00044   39   29   29  4.3e-14  2.8e-15
   3     0.00029   0.3500000     0.00029   40   30   30  1.2e-13  2.8e-15
Velocity u2, Pressure p
Iter      SolEst     Damping    Stepsize #Res #Jac #Sol   LinErr   LinRes
   1      0.0011   0.5000000      0.0011   20   10   10  2.6e-15  7.8e-15
Displacement field
Iter      SolEst     Damping    Stepsize #Res #Jac #Sol   LinErr   LinRes
   1      0.0094   0.5000000      0.0094   20   10   10    9e-14  1.2e-12
Spatial mesh displacement
Iter      SolEst     Damping    Stepsize #Res #Jac #Sol   LinErr   LinRes
   1     8.1e-08   0.5000000     8.1e-08   20   10   10  1.5e-15  1.8e-15
Solution error estimates for segregated groups
0.00049, 0.00053, 0.0047, 4e-08
Residual error estimates for segregated groups
0.14, 82, 1.2e+03, 0.14
Segregated solver iteration 11.
Merged variables
Iter      SolEst     Damping    Stepsize #Res #Jac #Sol   LinErr   LinRes
   1     0.00019   0.3500000     0.00019   42   31   31  6.1e-14  2.7e-15
   2     0.00012   0.3500000     0.00012   43   32   32    5e-14  2.7e-15
   3     7.9e-05   0.3500000     7.9e-05   44   33   33  3.6e-14  2.7e-15
Velocity u2, Pressure p
Iter      SolEst     Damping    Stepsize #Res #Jac #Sol   LinErr   LinRes
   1     0.00053   0.5000000     0.00053   22   11   11  4.6e-15  7.9e-15
Displacement field
Iter      SolEst     Damping    Stepsize #Res #Jac #Sol   LinErr   LinRes
   1      0.0051   0.5000000      0.0051   22   11   11  1.9e-13  1.2e-12
Spatial mesh displacement
Iter      SolEst     Damping    Stepsize #Res #Jac #Sol   LinErr   LinRes
   1     4.8e-08   0.5000000     4.8e-08   22   11   11  1.5e-15  1.9e-15
Solution error estimates for segregated groups
0.00014, 0.00026, 0.0026, 2.4e-08
Residual error estimates for segregated groups
0.043, 41, 1.2e+03, 0.14
Segregated solver iteration 12.
Merged variables
Iter      SolEst     Damping    Stepsize #Res #Jac #Sol   LinErr   LinRes
   1     5.1e-05   0.3500000     5.1e-05   46   34   34  6.3e-14  2.7e-15
   2     3.3e-05   0.3500000     3.3e-05   47   35   35  4.2e-14  2.8e-15
   3     2.2e-05   0.3500000     2.2e-05   48   36   36  5.6e-14  2.7e-15
Velocity u2, Pressure p
Iter      SolEst     Damping    Stepsize #Res #Jac #Sol   LinErr   LinRes
   1     0.00026   0.5000000     0.00026   24   12   12  2.6e-15  7.9e-15
Displacement field
Iter      SolEst     Damping    Stepsize #Res #Jac #Sol   LinErr   LinRes
   1      0.0028   0.5000000      0.0028   24   12   12  1.3e-13  1.2e-12
Spatial mesh displacement
Iter      SolEst     Damping    Stepsize #Res #Jac #Sol   LinErr   LinRes
```

```
   1      2.9e-08   0.5000000     2.9e-08   24   12   12  1.6e-15  1.8e-15
Solution error estimates for segregated groups
3.7e-05, 0.00013, 0.0014, 1.4e-08
Residual error estimates for segregated groups
0.013, 20, 1.2e+03, 0.14
Segregated solver iteration 13.
Merged variables
Iter      SolEst     Damping    Stepsize #Res #Jac #Sol   LinErr   LinRes
   1      1.4e-05   0.3500000     1.4e-05   50   37   37  7.7e-14  2.8e-15
   2      9.1e-06   0.3500000     9.1e-06   51   38   38  9.4e-14  2.8e-15
   3      5.9e-06   0.3500000     5.9e-06   52   39   39  4.5e-14  2.7e-15
Velocity u2, Pressure p
Iter      SolEst     Damping    Stepsize #Res #Jac #Sol   LinErr   LinRes
   1      0.00013   0.5000000     0.00013   26   13   13  2.4e-15  7.9e-15
Displacement field
Iter      SolEst     Damping    Stepsize #Res #Jac #Sol   LinErr   LinRes
   1       0.0015   0.5000000      0.0015   26   13   13  1.4e-13  1.2e-12
Spatial mesh displacement
Iter      SolEst     Damping    Stepsize #Res #Jac #Sol   LinErr   LinRes
   1      1.7e-08   0.5000000     1.7e-08   26   13   13  2.1e-15  1.8e-15
Solution error estimates for segregated groups
1e-05, 6.6e-05, 0.00076, 8.4e-09
Residual error estimates for segregated groups
0.004, 10, 1.2e+03, 0.15
Solution time: 155 s. (2 minutes, 35 seconds)
Physical memory: 4.73 GB
Virtual memory: 25.5 GB
Ended at Jan 31, 2025, 8:25:58 PM.
----- Stationary Solver 1 in study1/Approach Curve Solver (sol1) -------------->
```

d=2110 (su80)

GENERAL

Description	Value
Solution	d=2110

LOG

```
<---- Compile Equations: Stationary in study1/Approach Curve Solver (sol1) -----
Started at Jan 31, 2025, 8:25:58 PM.
Parameter d = 2.11E-6 (m).
Geometry shape function: Linear serendipity
Number of vertex elements: 18
Number of boundary elements: 6121
Number of elements: 154363
Minimum element quality: 0.4611
Time: 8 s.
Physical memory: 3.03 GB
Virtual memory: 15.31 GB
Ended at Jan 31, 2025, 8:26:06 PM.
----- Compile Equations: Stationary in study1/Approach Curve Solver (sol1) ---->

<---- Dependent Variables 1 in study1/Approach Curve Solver (sol1) -------------
Started at Jan 31, 2025, 8:26:06 PM.
Solution time: 0 s.
Physical memory: 3.03 GB
Virtual memory: 15.31 GB
Ended at Jan 31, 2025, 8:26:06 PM.
----- Dependent Variables 1 in study1/Approach Curve Solver (sol1) ------------>

<---- Stationary Solver 1 in study1/Approach Curve Solver (sol1) ---------------
Started at Jan 31, 2025, 8:26:06 PM.
Segregated solver
Segregated Step 11 does not have any components solved for.
Number of degrees of freedom solved for: 783554 (plus 11665 internal DOFs).
Segregated solver iteration 1.
Merged variables
Nonsymmetric matrix found.
Scales for dependent variables:
Concentration (comp1.Cl): 1.4e+02
Concentration (comp1.K): 1.4e+02
Electric potential (comp1.V): 1.9e+02
Orthonormal null-space function used.
Iter      SolEst     Damping    Stepsize #Res #Jac #Sol   LinErr   LinRes
   1        0.83   0.3500000        0.83    2    1    1    3e-11  2.8e-15
   2        0.81   0.3500000        0.81    3    2    2  4.1e-11  2.6e-15
   3         0.8   0.3500000         0.8    4    3    3    5e-11  2.6e-15
Velocity u2, Pressure p
Nonsymmetric matrix found.
Scales for dependent variables:
Pressure (comp1.p): 1
Velocity field (spatial frame) (comp1.u2): 0.00018
Orthonormal null-space function used.
Iter      SolEst     Damping    Stepsize #Res #Jac #Sol   LinErr   LinRes
   1         1.1   0.5000000         1.1    2    1    1  1.8e-15  2.2e-15
Displacement field
Nonsymmetric matrix found.
Scales for dependent variables:
Auxiliary pressure (comp1.solid.lemm1.pw): 1
Displacement field (comp1.u): 2.8e-18
Orthonormal null-space function used.
Iter      SolEst     Damping    Stepsize #Res #Jac #Sol   LinErr   LinRes
   1         1.9   0.5000000         1.9    2    1    1  9.9e-14  1.5e-12
Spatial mesh displacement
```

```
Symmetric matrices found.
Scales for dependent variables:
Spatial mesh displacement (comp1.spatial.disp): 1.2e-09
Orthonormal null-space function used.
Iter      SolEst     Damping    Stepsize #Res #Jac #Sol   LinErr   LinRes
   1     7.5e-07   0.5000000     7.5e-07    2    1    1  1.6e-15  1.8e-15
Solution error estimates for segregated groups
1.4, 0.54, 0.95, 3.8e-07
Residual error estimates for segregated groups
7.8e+03, 8.2e+04, 2.1e+04, 0.13
Segregated solver iteration 2.
Merged variables
Scales for dependent variables:
Concentration (comp1.Cl): 1.4e+02
Concentration (comp1.K): 1.4e+02
Electric potential (comp1.V): 0.064
Iter      SolEst     Damping    Stepsize #Res #Jac #Sol   LinErr   LinRes
   1        0.78   0.3500000        0.78    6    4    4  8.2e-14  3.5e-15
   2        0.77   0.3500000        0.77    7    5    5  1.2e-13  3.3e-15
   3        0.76   0.3500000        0.76    8    6    6  1.2e-13  3.5e-15
Velocity u2, Pressure p
Scales for dependent variables:
Pressure (comp1.p): 3.6e+02
Velocity field (spatial frame) (comp1.u2): 0.00073
Iter      SolEst     Damping    Stepsize #Res #Jac #Sol   LinErr   LinRes
   1        0.36   0.5000000        0.36    4    2    2  4.1e-15  7.8e-15
Displacement field
Iter      SolEst     Damping    Stepsize #Res #Jac #Sol   LinErr   LinRes
   1        0.95   0.5000000        0.95    4    2    2  5.2e-14  1.5e-12
Spatial mesh displacement
Iter      SolEst     Damping    Stepsize #Res #Jac #Sol   LinErr   LinRes
   1     1.1e-06   0.5000000     1.1e-06    4    2    2  1.8e-15  1.8e-15
Solution error estimates for segregated groups
1.3, 0.18, 0.48, 5.6e-07
Residual error estimates for segregated groups
7.7e+02, 1.5e+04, 2.2e+04, 0.14
Segregated solver iteration 3.
Merged variables
Iter      SolEst     Damping    Stepsize #Res #Jac #Sol   LinErr   LinRes
   1        0.74   0.3500000        0.74   10    7    7  8.5e-14    3e-15
   2        0.73   0.3500000        0.73   11    8    8  4.3e-14  3.8e-15
   3        0.71   0.3500000        0.71   12    9    9  1.2e-13  3.1e-15
Velocity u2, Pressure p
Iter      SolEst     Damping    Stepsize #Res #Jac #Sol   LinErr   LinRes
   1        0.15   0.5000000        0.15    6    3    3  2.5e-15  7.9e-15
Displacement field
Iter      SolEst     Damping    Stepsize #Res #Jac #Sol   LinErr   LinRes
   1        0.52   0.5000000        0.52    6    3    3  5.5e-14  1.5e-12
Spatial mesh displacement
Iter      SolEst     Damping    Stepsize #Res #Jac #Sol   LinErr   LinRes
   1     1.1e-06   0.5000000     1.1e-06    6    3    3  1.8e-15  1.8e-15
Solution error estimates for segregated groups
1.2, 0.077, 0.26, 5.6e-07
Residual error estimates for segregated groups
2.1e+02, 8.7e+03, 1.6e+04, 0.14
Segregated solver iteration 4.
```

```
Merged variables
Iter      SolEst     Damping    Stepsize #Res #Jac #Sol   LinErr   LinRes
   1         0.7   0.3500000         0.7   14   10   10  8.1e-14  2.9e-15
   2        0.68   0.3500000        0.68   15   11   11  7.2e-14  2.9e-15
   3        0.59   0.3500000        0.59   16   12   12  8.8e-14  3.1e-15
Velocity u2, Pressure p
Iter      SolEst     Damping    Stepsize #Res #Jac #Sol   LinErr   LinRes
   1       0.072   0.5000000       0.072    8    4    4  4.6e-15  7.8e-15
Displacement field
Iter      SolEst     Damping    Stepsize #Res #Jac #Sol   LinErr   LinRes
   1        0.29   0.5000000        0.29    8    4    4    2e-13  1.5e-12
Spatial mesh displacement
Iter      SolEst     Damping    Stepsize #Res #Jac #Sol   LinErr   LinRes
   1     9.4e-07   0.5000000     9.4e-07    8    4    4  1.2e-15  1.8e-15
Solution error estimates for segregated groups
1, 0.036, 0.15, 4.7e-07
Residual error estimates for segregated groups
82, 4.6e+03, 1.1e+04, 0.14
Segregated solver iteration 5.
Merged variables
Iter      SolEst     Damping    Stepsize #Res #Jac #Sol   LinErr   LinRes
   1         0.4   0.3500000         0.4   18   13   13  9.8e-14  3.2e-15
   2        0.27   0.3500000        0.27   19   14   14  1.3e-13  3.5e-15
   3        0.18   0.3500000        0.18   20   15   15  1.4e-13  3.5e-15
Velocity u2, Pressure p
Iter      SolEst     Damping    Stepsize #Res #Jac #Sol   LinErr   LinRes
   1       0.035   0.5000000       0.035   10    5    5  3.8e-15  7.8e-15
Displacement field
Iter      SolEst     Damping    Stepsize #Res #Jac #Sol   LinErr   LinRes
   1        0.17   0.5000000        0.17   10    5    5  8.3e-14  1.5e-12
Spatial mesh displacement
Iter      SolEst     Damping    Stepsize #Res #Jac #Sol   LinErr   LinRes
   1       7e-07   0.5000000       7e-07   10    5    5  1.5e-15  1.8e-15
Solution error estimates for segregated groups
0.31, 0.017, 0.084, 3.5e-07
Residual error estimates for segregated groups
32, 2.4e+03, 6.8e+03, 0.14
Segregated solver iteration 6.
Merged variables
Iter      SolEst     Damping    Stepsize #Res #Jac #Sol   LinErr   LinRes
   1        0.12   0.3500000        0.12   22   16   16  5.3e-14  3.1e-15
   2       0.077   0.3500000       0.077   23   17   17  8.4e-14  3.1e-15
   3        0.05   0.3500000        0.05   24   18   18  6.2e-14    3e-15
Velocity u2, Pressure p
Iter      SolEst     Damping    Stepsize #Res #Jac #Sol   LinErr   LinRes
   1       0.017   0.5000000       0.017   12    6    6  1.6e-15  7.8e-15
Displacement field
Scales for dependent variables:
Auxiliary pressure (comp1.solid.lemm1.pw): 0.35
Displacement field (comp1.u): 2.8e-16
Iter      SolEst     Damping    Stepsize #Res #Jac #Sol   LinErr   LinRes
   1       0.095   0.5000000       0.095   12    6    6  1.7e-13  1.2e-12
Spatial mesh displacement
Iter      SolEst     Damping    Stepsize #Res #Jac #Sol   LinErr   LinRes
   1     4.9e-07   0.5000000     4.9e-07   12    6    6  1.4e-15  1.8e-15
Solution error estimates for segregated groups
```

```
0.086, 0.0085, 0.048, 2.5e-07
Residual error estimates for segregated groups
12, 1.2e+03, 4.2e+03, 0.13
Segregated solver iteration 7.
Merged variables
Iter      SolEst     Damping    Stepsize #Res #Jac #Sol   LinErr   LinRes
   1       0.033   0.3500000       0.033   26   19   19  1.1e-13    4e-15
   2       0.021   0.3500000       0.021   27   20   20  4.1e-14    3e-15
   3       0.014   0.3500000       0.014   28   21   21    6e-14  3.3e-15
Velocity u2, Pressure p
Iter      SolEst     Damping    Stepsize #Res #Jac #Sol   LinErr   LinRes
   1      0.0085   0.5000000      0.0085   14    7    7  1.9e-15  7.7e-15
Displacement field
Iter      SolEst     Damping    Stepsize #Res #Jac #Sol   LinErr   LinRes
   1       0.054   0.5000000       0.054   14    7    7  9.3e-14  1.2e-12
Spatial mesh displacement
Iter      SolEst     Damping    Stepsize #Res #Jac #Sol   LinErr   LinRes
   1     3.3e-07   0.5000000     3.3e-07   14    7    7  1.3e-15  1.8e-15
Solution error estimates for segregated groups
0.024, 0.0042, 0.027, 1.6e-07
Residual error estimates for segregated groups
4.1, 6e+02, 2.6e+03, 0.14
Segregated solver iteration 8.
Merged variables
Iter      SolEst     Damping    Stepsize #Res #Jac #Sol   LinErr   LinRes
   1       0.009   0.3500000       0.009   30   22   22    1e-13    3e-15
   2      0.0058   0.3500000      0.0058   31   23   23  9.5e-14  3.4e-15
   3      0.0038   0.3500000      0.0038   32   24   24  7.7e-14  3.2e-15
Velocity u2, Pressure p
Iter      SolEst     Damping    Stepsize #Res #Jac #Sol   LinErr   LinRes
   1      0.0042   0.5000000      0.0042   16    8    8    3e-15  7.8e-15
Displacement field
Iter      SolEst     Damping    Stepsize #Res #Jac #Sol   LinErr   LinRes
   1        0.03   0.5000000        0.03   16    8    8  1.3e-13  1.2e-12
Spatial mesh displacement
Iter      SolEst     Damping    Stepsize #Res #Jac #Sol   LinErr   LinRes
   1     2.1e-07   0.5000000     2.1e-07   16    8    8  1.6e-15  1.9e-15
Solution error estimates for segregated groups
0.0065, 0.0021, 0.015, 1.1e-07
Residual error estimates for segregated groups
1.4, 3e+02, 1.8e+03, 0.14
Segregated solver iteration 9.
Merged variables
Iter      SolEst     Damping    Stepsize #Res #Jac #Sol   LinErr   LinRes
   1      0.0025   0.3500000      0.0025   34   25   25  4.9e-14  2.8e-15
   2      0.0016   0.3500000      0.0016   35   26   26  7.3e-14    3e-15
   3       0.001   0.3500000       0.001   36   27   27  8.3e-14  3.4e-15
Velocity u2, Pressure p
Iter      SolEst     Damping    Stepsize #Res #Jac #Sol   LinErr   LinRes
   1      0.0021   0.5000000      0.0021   18    9    9  2.5e-15  7.8e-15
Displacement field
Iter      SolEst     Damping    Stepsize #Res #Jac #Sol   LinErr   LinRes
   1       0.017   0.5000000       0.017   18    9    9  1.5e-13  1.2e-12
Spatial mesh displacement
Iter      SolEst     Damping    Stepsize #Res #Jac #Sol   LinErr   LinRes
   1     1.3e-07   0.5000000     1.3e-07   18    9    9  2.4e-15  1.8e-15
```

```
Solution error estimates for segregated groups
0.0018, 0.0011, 0.0085, 6.6e-08
Residual error estimates for segregated groups
0.44, 1.5e+02, 1.4e+03, 0.13
Segregated solver iteration 10.
Merged variables
Iter      SolEst     Damping    Stepsize #Res #Jac #Sol   LinErr   LinRes
   1     0.00068   0.3500000     0.00068   38   28   28  9.2e-14    3e-15
   2     0.00044   0.3500000     0.00044   39   29   29  5.5e-14  3.3e-15
   3     0.00029   0.3500000     0.00029   40   30   30  9.8e-14    3e-15
Velocity u2, Pressure p
Iter      SolEst     Damping    Stepsize #Res #Jac #Sol   LinErr   LinRes
   1      0.0011   0.5000000      0.0011   20   10   10  2.4e-15  7.8e-15
Displacement field
Iter      SolEst     Damping    Stepsize #Res #Jac #Sol   LinErr   LinRes
   1      0.0094   0.5000000      0.0094   20   10   10  1.2e-13  1.2e-12
Spatial mesh displacement
Iter      SolEst     Damping    Stepsize #Res #Jac #Sol   LinErr   LinRes
   1     8.1e-08   0.5000000     8.1e-08   20   10   10  1.4e-15  1.8e-15
Solution error estimates for segregated groups
0.00049, 0.00053, 0.0047, 4e-08
Residual error estimates for segregated groups
0.14, 76, 1.2e+03, 0.14
Segregated solver iteration 11.
Merged variables
Iter      SolEst     Damping    Stepsize #Res #Jac #Sol   LinErr   LinRes
   1     0.00019   0.3500000     0.00019   42   31   31    8e-14  3.6e-15
   2     0.00012   0.3500000     0.00012   43   32   32  8.8e-14  3.4e-15
   3     7.9e-05   0.3500000     7.9e-05   44   33   33  9.7e-14  4.5e-15
Velocity u2, Pressure p
Iter      SolEst     Damping    Stepsize #Res #Jac #Sol   LinErr   LinRes
   1     0.00053   0.5000000     0.00053   22   11   11  2.8e-15  7.7e-15
Displacement field
Iter      SolEst     Damping    Stepsize #Res #Jac #Sol   LinErr   LinRes
   1      0.0051   0.5000000      0.0051   22   11   11  8.7e-14  1.2e-12
Spatial mesh displacement
Iter      SolEst     Damping    Stepsize #Res #Jac #Sol   LinErr   LinRes
   1     4.8e-08   0.5000000     4.8e-08   22   11   11  1.7e-15  1.8e-15
Solution error estimates for segregated groups
0.00014, 0.00026, 0.0026, 2.4e-08
Residual error estimates for segregated groups
0.043, 38, 1.2e+03, 0.14
Segregated solver iteration 12.
Merged variables
Iter      SolEst     Damping    Stepsize #Res #Jac #Sol   LinErr   LinRes
   1     5.1e-05   0.3500000     5.1e-05   46   34   34  5.5e-14  3.1e-15
   2     3.3e-05   0.3500000     3.3e-05   47   35   35  9.9e-14    3e-15
   3     2.2e-05   0.3500000     2.2e-05   48   36   36  4.1e-14  3.8e-15
Velocity u2, Pressure p
Iter      SolEst     Damping    Stepsize #Res #Jac #Sol   LinErr   LinRes
   1     0.00026   0.5000000     0.00026   24   12   12  2.1e-15  7.7e-15
Displacement field
Iter      SolEst     Damping    Stepsize #Res #Jac #Sol   LinErr   LinRes
   1      0.0028   0.5000000      0.0028   24   12   12  1.5e-13  1.2e-12
Spatial mesh displacement
Iter      SolEst     Damping    Stepsize #Res #Jac #Sol   LinErr   LinRes
```

```
   1     2.9e-08   0.5000000     2.9e-08   24   12   12  1.3e-15  1.8e-15
Solution error estimates for segregated groups
3.7e-05, 0.00013, 0.0014, 1.4e-08
Residual error estimates for segregated groups
0.013, 19, 1.2e+03, 0.14
Segregated solver iteration 13.
Merged variables
Iter      SolEst     Damping    Stepsize #Res #Jac #Sol   LinErr   LinRes
   1     1.4e-05   0.3500000     1.4e-05   50   37   37  6.5e-14    3e-15
   2     9.1e-06   0.3500000     9.1e-06   51   38   38    7e-14    3e-15
   3     5.9e-06   0.3500000     5.9e-06   52   39   39  8.7e-14  3.4e-15
Velocity u2, Pressure p
Iter      SolEst     Damping    Stepsize #Res #Jac #Sol   LinErr   LinRes
   1     0.00013   0.5000000     0.00013   26   13   13  4.5e-15  7.8e-15
Displacement field
Iter      SolEst     Damping    Stepsize #Res #Jac #Sol   LinErr   LinRes
   1      0.0015   0.5000000      0.0015   26   13   13  1.3e-13  1.2e-12
Spatial mesh displacement
Iter      SolEst     Damping    Stepsize #Res #Jac #Sol   LinErr   LinRes
   1     1.7e-08   0.5000000     1.7e-08   26   13   13  1.8e-15  1.9e-15
Solution error estimates for segregated groups
1e-05, 6.6e-05, 0.00076, 8.4e-09
Residual error estimates for segregated groups
0.004, 9.5, 1.2e+03, 0.15
Solution time: 154 s. (2 minutes, 34 seconds)
Physical memory: 4.68 GB
Virtual memory: 25.55 GB
Ended at Jan 31, 2025, 8:28:41 PM.
----- Stationary Solver 1 in study1/Approach Curve Solver (sol1) -------------->
```

d=2105 (su81)

GENERAL

Description	Value
Solution	d=2105

LOG

```
<---- Compile Equations: Stationary in study1/Approach Curve Solver (sol1) -----
Started at Jan 31, 2025, 8:28:41 PM.
Parameter d = 2.105E-6 (m).
Geometry shape function: Linear serendipity
Number of vertex elements: 18
Number of boundary elements: 6119
Number of elements: 153981
Minimum element quality: 0.4916
Time: 8 s.
Physical memory: 3.02 GB
Virtual memory: 15.29 GB
Ended at Jan 31, 2025, 8:28:49 PM.
----- Compile Equations: Stationary in study1/Approach Curve Solver (sol1) ---->

<---- Dependent Variables 1 in study1/Approach Curve Solver (sol1) -------------
Started at Jan 31, 2025, 8:28:49 PM.
Solution time: 0 s.
Physical memory: 3.02 GB
Virtual memory: 15.29 GB
Ended at Jan 31, 2025, 8:28:49 PM.
----- Dependent Variables 1 in study1/Approach Curve Solver (sol1) ------------>

<---- Stationary Solver 1 in study1/Approach Curve Solver (sol1) ---------------
Started at Jan 31, 2025, 8:28:49 PM.
Segregated solver
Segregated Step 11 does not have any components solved for.
Number of degrees of freedom solved for: 781716 (plus 11661 internal DOFs).
Segregated solver iteration 1.
Merged variables
Nonsymmetric matrix found.
Scales for dependent variables:
Concentration (comp1.Cl): 1.4e+02
Concentration (comp1.K): 1.4e+02
Electric potential (comp1.V): 1.9e+02
Orthonormal null-space function used.
Iter      SolEst     Damping    Stepsize #Res #Jac #Sol   LinErr   LinRes
   1        0.83   0.3500000        0.83    2    1    1  4.8e-11  2.7e-15
   2        0.81   0.3500000        0.81    3    2    2  5.3e-11  2.7e-15
   3         0.8   0.3500000         0.8    4    3    3  5.6e-11  2.6e-15
Velocity u2, Pressure p
Nonsymmetric matrix found.
Scales for dependent variables:
Pressure (comp1.p): 1
Velocity field (spatial frame) (comp1.u2): 0.00018
Orthonormal null-space function used.
Iter      SolEst     Damping    Stepsize #Res #Jac #Sol   LinErr   LinRes
   1         1.1   0.5000000         1.1    2    1    1  1.6e-15  2.3e-15
Displacement field
Nonsymmetric matrix found.
Scales for dependent variables:
Auxiliary pressure (comp1.solid.lemm1.pw): 1
Displacement field (comp1.u): 2.8e-18
Orthonormal null-space function used.
Iter      SolEst     Damping    Stepsize #Res #Jac #Sol   LinErr   LinRes
   1         1.9   0.5000000         1.9    2    1    1    7e-14  1.5e-12
Spatial mesh displacement
```

```
Symmetric matrices found.
Scales for dependent variables:
Spatial mesh displacement (comp1.spatial.disp): 1.2e-09
Orthonormal null-space function used.
Iter      SolEst     Damping    Stepsize #Res #Jac #Sol   LinErr   LinRes
   1     7.5e-07   0.5000000     7.5e-07    2    1    1  1.4e-15  1.8e-15
Solution error estimates for segregated groups
1.4, 0.54, 0.95, 3.8e-07
Residual error estimates for segregated groups
7.8e+03, 8.2e+04, 2.1e+04, 0.12
Segregated solver iteration 2.
Merged variables
Scales for dependent variables:
Concentration (comp1.Cl): 1.4e+02
Concentration (comp1.K): 1.4e+02
Electric potential (comp1.V): 0.064
Iter      SolEst     Damping    Stepsize #Res #Jac #Sol   LinErr   LinRes
   1        0.78   0.3500000        0.78    6    4    4  4.1e-14  2.8e-15
   2        0.77   0.3500000        0.77    7    5    5    5e-14    3e-15
   3        0.76   0.3500000        0.76    8    6    6  8.8e-14  2.8e-15
Velocity u2, Pressure p
Scales for dependent variables:
Pressure (comp1.p): 3.6e+02
Velocity field (spatial frame) (comp1.u2): 0.00073
Iter      SolEst     Damping    Stepsize #Res #Jac #Sol   LinErr   LinRes
   1        0.36   0.5000000        0.36    4    2    2  2.5e-15  7.8e-15
Displacement field
Iter      SolEst     Damping    Stepsize #Res #Jac #Sol   LinErr   LinRes
   1        0.95   0.5000000        0.95    4    2    2  2.2e-13  1.5e-12
Spatial mesh displacement
Iter      SolEst     Damping    Stepsize #Res #Jac #Sol   LinErr   LinRes
   1     1.1e-06   0.5000000     1.1e-06    4    2    2  1.6e-15  1.8e-15
Solution error estimates for segregated groups
1.3, 0.18, 0.48, 5.6e-07
Residual error estimates for segregated groups
7.7e+02, 1.3e+04, 2.1e+04, 0.14
Segregated solver iteration 3.
Merged variables
Iter      SolEst     Damping    Stepsize #Res #Jac #Sol   LinErr   LinRes
   1        0.74   0.3500000        0.74   10    7    7  8.3e-14  4.7e-15
   2        0.73   0.3500000        0.73   11    8    8  1.6e-13  7.2e-15
   3        0.71   0.3500000        0.71   12    9    9    1e-13  5.8e-15
Velocity u2, Pressure p
Iter      SolEst     Damping    Stepsize #Res #Jac #Sol   LinErr   LinRes
   1        0.15   0.5000000        0.15    6    3    3  2.7e-15  7.8e-15
Displacement field
Iter      SolEst     Damping    Stepsize #Res #Jac #Sol   LinErr   LinRes
   1        0.52   0.5000000        0.52    6    3    3  6.4e-14  1.5e-12
Spatial mesh displacement
Iter      SolEst     Damping    Stepsize #Res #Jac #Sol   LinErr   LinRes
   1     1.1e-06   0.5000000     1.1e-06    6    3    3  1.6e-15  1.8e-15
Solution error estimates for segregated groups
1.2, 0.077, 0.26, 5.6e-07
Residual error estimates for segregated groups
2.1e+02, 7e+03, 1.6e+04, 0.14
Segregated solver iteration 4.
```

```
Merged variables
Iter      SolEst     Damping    Stepsize #Res #Jac #Sol   LinErr   LinRes
   1         0.7   0.3500000         0.7   14   10   10  1.3e-13  4.2e-15
   2        0.68   0.3500000        0.68   15   11   11  1.1e-13  4.2e-15
   3        0.58   0.3500000        0.58   16   12   12  8.2e-14  5.5e-15
Velocity u2, Pressure p
Iter      SolEst     Damping    Stepsize #Res #Jac #Sol   LinErr   LinRes
   1       0.072   0.5000000       0.072    8    4    4  3.4e-15  7.9e-15
Displacement field
Iter      SolEst     Damping    Stepsize #Res #Jac #Sol   LinErr   LinRes
   1        0.29   0.5000000        0.29    8    4    4  2.6e-14  1.5e-12
Spatial mesh displacement
Iter      SolEst     Damping    Stepsize #Res #Jac #Sol   LinErr   LinRes
   1     9.4e-07   0.5000000     9.4e-07    8    4    4    2e-15  1.8e-15
Solution error estimates for segregated groups
1, 0.036, 0.15, 4.7e-07
Residual error estimates for segregated groups
82, 3.7e+03, 1.1e+04, 0.14
Segregated solver iteration 5.
Merged variables
Iter      SolEst     Damping    Stepsize #Res #Jac #Sol   LinErr   LinRes
   1         0.4   0.3500000         0.4   18   13   13  5.4e-14  3.4e-15
   2        0.27   0.3500000        0.27   19   14   14  5.6e-14  3.2e-15
   3        0.18   0.3500000        0.18   20   15   15  1.4e-13  7.1e-15
Velocity u2, Pressure p
Iter      SolEst     Damping    Stepsize #Res #Jac #Sol   LinErr   LinRes
   1       0.035   0.5000000       0.035   10    5    5  2.5e-15  7.8e-15
Displacement field
Iter      SolEst     Damping    Stepsize #Res #Jac #Sol   LinErr   LinRes
   1        0.17   0.5000000        0.17   10    5    5  1.2e-13  1.5e-12
Spatial mesh displacement
Iter      SolEst     Damping    Stepsize #Res #Jac #Sol   LinErr   LinRes
   1       7e-07   0.5000000       7e-07   10    5    5  1.2e-15  1.8e-15
Solution error estimates for segregated groups
0.31, 0.017, 0.084, 3.5e-07
Residual error estimates for segregated groups
32, 1.9e+03, 6.8e+03, 0.14
Segregated solver iteration 6.
Merged variables
Iter      SolEst     Damping    Stepsize #Res #Jac #Sol   LinErr   LinRes
   1        0.12   0.3500000        0.12   22   16   16    1e-13  4.5e-15
   2       0.077   0.3500000       0.077   23   17   17  1.1e-13  4.8e-15
   3        0.05   0.3500000        0.05   24   18   18  9.3e-14  8.5e-15
Velocity u2, Pressure p
Iter      SolEst     Damping    Stepsize #Res #Jac #Sol   LinErr   LinRes
   1       0.017   0.5000000       0.017   12    6    6  2.9e-15  7.9e-15
Displacement field
Scales for dependent variables:
Auxiliary pressure (comp1.solid.lemm1.pw): 0.35
Displacement field (comp1.u): 2.8e-16
Iter      SolEst     Damping    Stepsize #Res #Jac #Sol   LinErr   LinRes
   1       0.095   0.5000000       0.095   12    6    6  1.4e-13  1.2e-12
Spatial mesh displacement
Iter      SolEst     Damping    Stepsize #Res #Jac #Sol   LinErr   LinRes
   1     4.9e-07   0.5000000     4.9e-07   12    6    6  1.4e-15  1.8e-15
Solution error estimates for segregated groups
```

```
0.086, 0.0085, 0.048, 2.5e-07
Residual error estimates for segregated groups
12, 9.6e+02, 4.2e+03, 0.14
Segregated solver iteration 7.
Merged variables
Iter      SolEst     Damping    Stepsize #Res #Jac #Sol   LinErr   LinRes
   1       0.033   0.3500000       0.033   26   19   19  7.6e-14  4.2e-15
   2       0.021   0.3500000       0.021   27   20   20  1.1e-13  3.2e-15
   3       0.014   0.3500000       0.014   28   21   21  1.4e-13  4.5e-15
Velocity u2, Pressure p
Iter      SolEst     Damping    Stepsize #Res #Jac #Sol   LinErr   LinRes
   1      0.0085   0.5000000      0.0085   14    7    7  2.8e-15  7.9e-15
Displacement field
Iter      SolEst     Damping    Stepsize #Res #Jac #Sol   LinErr   LinRes
   1       0.054   0.5000000       0.054   14    7    7  1.6e-13  1.2e-12
Spatial mesh displacement
Iter      SolEst     Damping    Stepsize #Res #Jac #Sol   LinErr   LinRes
   1     3.3e-07   0.5000000     3.3e-07   14    7    7  1.2e-15  1.8e-15
Solution error estimates for segregated groups
0.024, 0.0042, 0.027, 1.6e-07
Residual error estimates for segregated groups
4.1, 4.8e+02, 2.6e+03, 0.14
Segregated solver iteration 8.
Merged variables
Iter      SolEst     Damping    Stepsize #Res #Jac #Sol   LinErr   LinRes
   1       0.009   0.3500000       0.009   30   22   22  5.8e-14  5.9e-15
   2      0.0058   0.3500000      0.0058   31   23   23  1.1e-13  3.9e-15
   3      0.0038   0.3500000      0.0038   32   24   24    9e-14  4.4e-15
Velocity u2, Pressure p
Iter      SolEst     Damping    Stepsize #Res #Jac #Sol   LinErr   LinRes
   1      0.0042   0.5000000      0.0042   16    8    8  2.6e-15  7.9e-15
Displacement field
Iter      SolEst     Damping    Stepsize #Res #Jac #Sol   LinErr   LinRes
   1        0.03   0.5000000        0.03   16    8    8  1.2e-13  1.2e-12
Spatial mesh displacement
Iter      SolEst     Damping    Stepsize #Res #Jac #Sol   LinErr   LinRes
   1     2.1e-07   0.5000000     2.1e-07   16    8    8  1.8e-15  1.8e-15
Solution error estimates for segregated groups
0.0065, 0.0021, 0.015, 1.1e-07
Residual error estimates for segregated groups
1.4, 2.4e+02, 1.8e+03, 0.14
Segregated solver iteration 9.
Merged variables
Iter      SolEst     Damping    Stepsize #Res #Jac #Sol   LinErr   LinRes
   1      0.0025   0.3500000      0.0025   34   25   25  6.8e-14  3.6e-15
   2      0.0016   0.3500000      0.0016   35   26   26  1.4e-13  5.4e-15
   3       0.001   0.3500000       0.001   36   27   27  1.5e-13  3.7e-15
Velocity u2, Pressure p
Iter      SolEst     Damping    Stepsize #Res #Jac #Sol   LinErr   LinRes
   1      0.0021   0.5000000      0.0021   18    9    9  2.6e-15  7.8e-15
Displacement field
Iter      SolEst     Damping    Stepsize #Res #Jac #Sol   LinErr   LinRes
   1       0.017   0.5000000       0.017   18    9    9  1.5e-13  1.2e-12
Spatial mesh displacement
Iter      SolEst     Damping    Stepsize #Res #Jac #Sol   LinErr   LinRes
   1     1.3e-07   0.5000000     1.3e-07   18    9    9  1.3e-15  1.8e-15
```

```
Solution error estimates for segregated groups
0.0018, 0.0011, 0.0085, 6.6e-08
Residual error estimates for segregated groups
0.44, 1.2e+02, 1.4e+03, 0.13
Segregated solver iteration 10.
Merged variables
Iter      SolEst     Damping    Stepsize #Res #Jac #Sol   LinErr   LinRes
   1     0.00068   0.3500000     0.00068   38   28   28    1e-13    4e-15
   2     0.00044   0.3500000     0.00044   39   29   29  1.2e-13  4.7e-15
   3     0.00029   0.3500000     0.00029   40   30   30  9.6e-14  3.3e-15
Velocity u2, Pressure p
Iter      SolEst     Damping    Stepsize #Res #Jac #Sol   LinErr   LinRes
   1      0.0011   0.5000000      0.0011   20   10   10  2.8e-15  7.9e-15
Displacement field
Iter      SolEst     Damping    Stepsize #Res #Jac #Sol   LinErr   LinRes
   1      0.0094   0.5000000      0.0094   20   10   10  1.5e-13  1.2e-12
Spatial mesh displacement
Iter      SolEst     Damping    Stepsize #Res #Jac #Sol   LinErr   LinRes
   1     8.1e-08   0.5000000     8.1e-08   20   10   10  1.4e-15  1.8e-15
Solution error estimates for segregated groups
0.00049, 0.00053, 0.0047, 4e-08
Residual error estimates for segregated groups
0.14, 61, 1.2e+03, 0.14
Segregated solver iteration 11.
Merged variables
Iter      SolEst     Damping    Stepsize #Res #Jac #Sol   LinErr   LinRes
   1     0.00019   0.3500000     0.00019   42   31   31  8.9e-14  4.9e-15
   2     0.00012   0.3500000     0.00012   43   32   32  1.1e-13  3.8e-15
   3     7.9e-05   0.3500000     7.9e-05   44   33   33  8.1e-14  5.8e-15
Velocity u2, Pressure p
Iter      SolEst     Damping    Stepsize #Res #Jac #Sol   LinErr   LinRes
   1     0.00053   0.5000000     0.00053   22   11   11  2.2e-15    8e-15
Displacement field
Iter      SolEst     Damping    Stepsize #Res #Jac #Sol   LinErr   LinRes
   1      0.0051   0.5000000      0.0051   22   11   11  1.4e-13  1.2e-12
Spatial mesh displacement
Iter      SolEst     Damping    Stepsize #Res #Jac #Sol   LinErr   LinRes
   1     4.8e-08   0.5000000     4.8e-08   22   11   11  1.4e-15  1.8e-15
Solution error estimates for segregated groups
0.00014, 0.00026, 0.0026, 2.4e-08
Residual error estimates for segregated groups
0.043, 30, 1.2e+03, 0.13
Segregated solver iteration 12.
Merged variables
Iter      SolEst     Damping    Stepsize #Res #Jac #Sol   LinErr   LinRes
   1     5.1e-05   0.3500000     5.1e-05   46   34   34  8.9e-14  3.7e-15
   2     3.3e-05   0.3500000     3.3e-05   47   35   35  7.8e-14  4.1e-15
   3     2.2e-05   0.3500000     2.2e-05   48   36   36  9.5e-14  3.6e-15
Velocity u2, Pressure p
Iter      SolEst     Damping    Stepsize #Res #Jac #Sol   LinErr   LinRes
   1     0.00026   0.5000000     0.00026   24   12   12  4.3e-15  7.9e-15
Displacement field
Iter      SolEst     Damping    Stepsize #Res #Jac #Sol   LinErr   LinRes
   1      0.0028   0.5000000      0.0028   24   12   12  1.4e-13  1.2e-12
Spatial mesh displacement
Iter      SolEst     Damping    Stepsize #Res #Jac #Sol   LinErr   LinRes
```

```
   1     2.9e-08   0.5000000     2.9e-08   24    12    12  1.9e-15  1.8e-15
Solution error estimates for segregated groups
3.7e-05, 0.00013, 0.0014, 1.4e-08
Residual error estimates for segregated groups
0.013, 15, 1.2e+03, 0.14
Segregated solver iteration 13.
Merged variables
Iter      SolEst     Damping    Stepsize #Res #Jac #Sol   LinErr   LinRes
   1     1.4e-05   0.3500000     1.4e-05   50    37    37  7.5e-14    6e-15
   2     9.1e-06   0.3500000     9.1e-06   51    38    38  8.3e-14  5.3e-15
   3     5.9e-06   0.3500000     5.9e-06   52    39    39  1.2e-13  4.3e-15
Velocity u2, Pressure p
Iter      SolEst     Damping    Stepsize #Res #Jac #Sol   LinErr   LinRes
   1     0.00013   0.5000000     0.00013   26    13    13  4.3e-15    8e-15
Displacement field
Iter      SolEst     Damping    Stepsize #Res #Jac #Sol   LinErr   LinRes
   1      0.0015   0.5000000      0.0015   26    13    13    1e-13  1.2e-12
Spatial mesh displacement
Iter      SolEst     Damping    Stepsize #Res #Jac #Sol   LinErr   LinRes
   1     1.7e-08   0.5000000     1.7e-08   26    13    13  1.9e-15  1.9e-15
Solution error estimates for segregated groups
1e-05, 6.6e-05, 0.00076, 8.3e-09
Residual error estimates for segregated groups
0.004, 7.6, 1.2e+03, 0.15
Solution time: 159 s. (2 minutes, 39 seconds)
Physical memory: 4.69 GB
Virtual memory: 25.57 GB
Ended at Jan 31, 2025, 8:31:28 PM.
----- Stationary Solver 1 in study1/Approach Curve Solver (sol1) -------------->
```

d=2100 (su82)

GENERAL

Description	Value
Solution	d=2100

LOG

```
<---- Compile Equations: Stationary in study1/Approach Curve Solver (sol1) -----
Started at Jan 31, 2025, 8:31:28 PM.
Parameter d = 2.1000000000000002E-6 (m).
Geometry shape function: Linear serendipity
Number of vertex elements: 18
Number of boundary elements: 6116
Number of elements: 154198
Minimum element quality: 0.4812
Time: 8 s.
Physical memory: 2.99 GB
Virtual memory: 15.25 GB
Ended at Jan 31, 2025, 8:31:36 PM.
----- Compile Equations: Stationary in study1/Approach Curve Solver (sol1) ---->

<---- Dependent Variables 1 in study1/Approach Curve Solver (sol1) -------------
Started at Jan 31, 2025, 8:31:36 PM.
Solution time: 0 s.
Physical memory: 2.99 GB
Virtual memory: 15.25 GB
Ended at Jan 31, 2025, 8:31:36 PM.
----- Dependent Variables 1 in study1/Approach Curve Solver (sol1) ------------>

<---- Stationary Solver 1 in study1/Approach Curve Solver (sol1) ---------------
Started at Jan 31, 2025, 8:31:36 PM.
Segregated solver
Segregated Step 11 does not have any components solved for.
Number of degrees of freedom solved for: 782552 (plus 11655 internal DOFs).
Segregated solver iteration 1.
Merged variables
Nonsymmetric matrix found.
Scales for dependent variables:
Concentration (comp1.Cl): 1.4e+02
Concentration (comp1.K): 1.4e+02
Electric potential (comp1.V): 1.9e+02
Orthonormal null-space function used.
Iter      SolEst     Damping    Stepsize #Res #Jac #Sol   LinErr   LinRes
   1        0.83   0.3500000        0.83    2    1    1  3.8e-11  2.7e-15
   2        0.81   0.3500000        0.81    3    2    2  1.9e-11  2.6e-15
   3         0.8   0.3500000         0.8    4    3    3  4.9e-11  2.6e-15
Velocity u2, Pressure p
Nonsymmetric matrix found.
Scales for dependent variables:
Pressure (comp1.p): 1
Velocity field (spatial frame) (comp1.u2): 0.00018
Orthonormal null-space function used.
Iter      SolEst     Damping    Stepsize #Res #Jac #Sol   LinErr   LinRes
   1         1.1   0.5000000         1.1    2    1    1  1.7e-15  2.3e-15
Displacement field
Nonsymmetric matrix found.
Scales for dependent variables:
Auxiliary pressure (comp1.solid.lemm1.pw): 1
Displacement field (comp1.u): 2.8e-18
Orthonormal null-space function used.
Iter      SolEst     Damping    Stepsize #Res #Jac #Sol   LinErr   LinRes
   1         1.9   0.5000000         1.9    2    1    1  1.6e-13  1.5e-12
Spatial mesh displacement
```

```
Symmetric matrices found.
Scales for dependent variables:
Spatial mesh displacement (comp1.spatial.disp): 1.2e-09
Orthonormal null-space function used.
Iter      SolEst     Damping    Stepsize #Res #Jac #Sol   LinErr   LinRes
   1     7.6e-07   0.5000000     7.6e-07    2    1    1  1.8e-15  1.8e-15
Solution error estimates for segregated groups
1.4, 0.54, 0.95, 3.8e-07
Residual error estimates for segregated groups
7.8e+03, 8.2e+04, 2.1e+04, 0.12
Segregated solver iteration 2.
Merged variables
Scales for dependent variables:
Concentration (comp1.Cl): 1.4e+02
Concentration (comp1.K): 1.4e+02
Electric potential (comp1.V): 0.064
Iter      SolEst     Damping    Stepsize #Res #Jac #Sol   LinErr   LinRes
   1        0.78   0.3500000        0.78    6    4    4    8e-14  1.1e-14
   2        0.77   0.3500000        0.77    7    5    5  8.2e-14  4.2e-15
   3        0.76   0.3500000        0.76    8    6    6  4.9e-14  5.8e-15
Velocity u2, Pressure p
Scales for dependent variables:
Pressure (comp1.p): 3.6e+02
Velocity field (spatial frame) (comp1.u2): 0.00073
Iter      SolEst     Damping    Stepsize #Res #Jac #Sol   LinErr   LinRes
   1        0.36   0.5000000        0.36    4    2    2  2.3e-15  7.9e-15
Displacement field
Iter      SolEst     Damping    Stepsize #Res #Jac #Sol   LinErr   LinRes
   1        0.95   0.5000000        0.95    4    2    2  1.1e-13  1.4e-12
Spatial mesh displacement
Iter      SolEst     Damping    Stepsize #Res #Jac #Sol   LinErr   LinRes
   1     1.1e-06   0.5000000     1.1e-06    4    2    2  1.1e-15  1.9e-15
Solution error estimates for segregated groups
1.3, 0.18, 0.48, 5.7e-07
Residual error estimates for segregated groups
7.7e+02, 1.7e+04, 2.1e+04, 0.14
Segregated solver iteration 3.
Merged variables
Iter      SolEst     Damping    Stepsize #Res #Jac #Sol   LinErr   LinRes
   1        0.74   0.3500000        0.74   10    7    7  1.4e-13    4e-15
   2        0.73   0.3500000        0.73   11    8    8  1.4e-13    1e-14
   3        0.71   0.3500000        0.71   12    9    9  8.4e-14  6.3e-15
Velocity u2, Pressure p
Iter      SolEst     Damping    Stepsize #Res #Jac #Sol   LinErr   LinRes
   1        0.15   0.5000000        0.15    6    3    3  3.4e-15  7.9e-15
Displacement field
Iter      SolEst     Damping    Stepsize #Res #Jac #Sol   LinErr   LinRes
   1        0.52   0.5000000        0.52    6    3    3  4.2e-14  1.5e-12
Spatial mesh displacement
Iter      SolEst     Damping    Stepsize #Res #Jac #Sol   LinErr   LinRes
   1     1.1e-06   0.5000000     1.1e-06    6    3    3  1.5e-15  1.9e-15
Solution error estimates for segregated groups
1.2, 0.077, 0.26, 5.7e-07
Residual error estimates for segregated groups
2.1e+02, 9.6e+03, 1.6e+04, 0.14
Segregated solver iteration 4.
```

```
Merged variables
Iter      SolEst     Damping    Stepsize #Res #Jac #Sol   LinErr   LinRes
   1         0.7   0.3500000         0.7   14   10   10  7.9e-14  3.6e-15
   2        0.68   0.3500000        0.68   15   11   11  7.2e-14    4e-15
   3        0.58   0.3500000        0.58   16   12   12  1.3e-13  5.5e-15
Velocity u2, Pressure p
Iter      SolEst     Damping    Stepsize #Res #Jac #Sol   LinErr   LinRes
   1       0.072   0.5000000       0.072    8    4    4  2.3e-15  7.9e-15
Displacement field
Iter      SolEst     Damping    Stepsize #Res #Jac #Sol   LinErr   LinRes
   1        0.29   0.5000000        0.29    8    4    4  5.3e-14  1.5e-12
Spatial mesh displacement
Iter      SolEst     Damping    Stepsize #Res #Jac #Sol   LinErr   LinRes
   1     9.5e-07   0.5000000     9.5e-07    8    4    4  1.6e-15  1.8e-15
Solution error estimates for segregated groups
1, 0.036, 0.15, 4.7e-07
Residual error estimates for segregated groups
82, 5.1e+03, 1.1e+04, 0.13
Segregated solver iteration 5.
Merged variables
Iter      SolEst     Damping    Stepsize #Res #Jac #Sol   LinErr   LinRes
   1         0.4   0.3500000         0.4   18   13   13  1.6e-13  4.7e-15
   2        0.27   0.3500000        0.27   19   14   14  2.4e-13  5.7e-15
   3        0.18   0.3500000        0.18   20   15   15  3.2e-13  6.1e-15
Velocity u2, Pressure p
Iter      SolEst     Damping    Stepsize #Res #Jac #Sol   LinErr   LinRes
   1       0.035   0.5000000       0.035   10    5    5  2.9e-15  7.8e-15
Displacement field
Iter      SolEst     Damping    Stepsize #Res #Jac #Sol   LinErr   LinRes
   1        0.17   0.5000000        0.17   10    5    5  6.3e-14  1.5e-12
Spatial mesh displacement
Iter      SolEst     Damping    Stepsize #Res #Jac #Sol   LinErr   LinRes
   1     7.1e-07   0.5000000     7.1e-07   10    5    5  1.6e-15  1.8e-15
Solution error estimates for segregated groups
0.31, 0.017, 0.084, 3.6e-07
Residual error estimates for segregated groups
32, 2.6e+03, 6.8e+03, 0.15
Segregated solver iteration 6.
Merged variables
Iter      SolEst     Damping    Stepsize #Res #Jac #Sol   LinErr   LinRes
   1        0.12   0.3500000        0.12   22   16   16  1.5e-13  6.1e-15
   2       0.076   0.3500000       0.076   23   17   17  7.1e-14  4.6e-15
   3        0.05   0.3500000        0.05   24   18   18  1.4e-13  5.8e-15
Velocity u2, Pressure p
Iter      SolEst     Damping    Stepsize #Res #Jac #Sol   LinErr   LinRes
   1       0.017   0.5000000       0.017   12    6    6  2.9e-15    8e-15
Displacement field
Scales for dependent variables:
Auxiliary pressure (comp1.solid.lemm1.pw): 0.35
Displacement field (comp1.u): 2.8e-16
Iter      SolEst     Damping    Stepsize #Res #Jac #Sol   LinErr   LinRes
   1       0.095   0.5000000       0.095   12    6    6  1.1e-13  1.2e-12
Spatial mesh displacement
Iter      SolEst     Damping    Stepsize #Res #Jac #Sol   LinErr   LinRes
   1       5e-07   0.5000000       5e-07   12    6    6  2.1e-15  1.8e-15
Solution error estimates for segregated groups
```

```
0.086, 0.0085, 0.048, 2.5e-07
Residual error estimates for segregated groups
12, 1.3e+03, 4.2e+03, 0.13
Segregated solver iteration 7.
Merged variables
Iter      SolEst     Damping    Stepsize #Res #Jac #Sol   LinErr   LinRes
   1       0.033   0.3500000       0.033   26   19   19  1.1e-13  6.4e-15
   2       0.021   0.3500000       0.021   27   20   20  7.1e-14    4e-15
   3       0.014   0.3500000       0.014   28   21   21  3.1e-13    7e-15
Velocity u2, Pressure p
Iter      SolEst     Damping    Stepsize #Res #Jac #Sol   LinErr   LinRes
   1      0.0085   0.5000000      0.0085   14    7    7  4.1e-15  7.9e-15
Displacement field
Iter      SolEst     Damping    Stepsize #Res #Jac #Sol   LinErr   LinRes
   1       0.054   0.5000000       0.054   14    7    7  1.3e-13  1.2e-12
Spatial mesh displacement
Iter      SolEst     Damping    Stepsize #Res #Jac #Sol   LinErr   LinRes
   1     3.3e-07   0.5000000     3.3e-07   14    7    7  1.5e-15  1.8e-15
Solution error estimates for segregated groups
0.024, 0.0042, 0.027, 1.7e-07
Residual error estimates for segregated groups
4.1, 6.7e+02, 2.6e+03, 0.15
Segregated solver iteration 8.
Merged variables
Iter      SolEst     Damping    Stepsize #Res #Jac #Sol   LinErr   LinRes
   1       0.009   0.3500000       0.009   30   22   22  1.2e-13  6.6e-15
   2      0.0058   0.3500000      0.0058   31   23   23  8.4e-14  1.1e-14
   3      0.0038   0.3500000      0.0038   32   24   24  1.7e-13  3.5e-15
Velocity u2, Pressure p
Iter      SolEst     Damping    Stepsize #Res #Jac #Sol   LinErr   LinRes
   1      0.0042   0.5000000      0.0042   16    8    8  1.9e-15  7.9e-15
Displacement field
Iter      SolEst     Damping    Stepsize #Res #Jac #Sol   LinErr   LinRes
   1        0.03   0.5000000        0.03   16    8    8  1.2e-13  1.2e-12
Spatial mesh displacement
Iter      SolEst     Damping    Stepsize #Res #Jac #Sol   LinErr   LinRes
   1     2.1e-07   0.5000000     2.1e-07   16    8    8  1.6e-15  1.8e-15
Solution error estimates for segregated groups
0.0065, 0.0021, 0.015, 1.1e-07
Residual error estimates for segregated groups
1.4, 3.4e+02, 1.8e+03, 0.14
Segregated solver iteration 9.
Merged variables
Iter      SolEst     Damping    Stepsize #Res #Jac #Sol   LinErr   LinRes
   1      0.0025   0.3500000      0.0025   34   25   25  1.7e-13    6e-15
   2      0.0016   0.3500000      0.0016   35   26   26  2.2e-13  6.4e-15
   3       0.001   0.3500000       0.001   36   27   27  1.8e-13    5e-15
Velocity u2, Pressure p
Iter      SolEst     Damping    Stepsize #Res #Jac #Sol   LinErr   LinRes
   1      0.0021   0.5000000      0.0021   18    9    9  3.8e-15  7.9e-15
Displacement field
Iter      SolEst     Damping    Stepsize #Res #Jac #Sol   LinErr   LinRes
   1       0.017   0.5000000       0.017   18    9    9  1.5e-13  1.2e-12
Spatial mesh displacement
Iter      SolEst     Damping    Stepsize #Res #Jac #Sol   LinErr   LinRes
   1     1.3e-07   0.5000000     1.3e-07   18    9    9  1.5e-15  1.8e-15
```

```
Solution error estimates for segregated groups
0.0018, 0.0011, 0.0085, 6.7e-08
Residual error estimates for segregated groups
0.44, 1.7e+02, 1.4e+03, 0.13
Segregated solver iteration 10.
Merged variables
Iter      SolEst     Damping    Stepsize #Res #Jac #Sol   LinErr   LinRes
   1     0.00068   0.3500000     0.00068   38   28   28  1.5e-13  4.7e-15
   2     0.00044   0.3500000     0.00044   39   29   29  8.6e-14  1.1e-14
   3     0.00029   0.3500000     0.00029   40   30   30  9.4e-14  3.5e-15
Velocity u2, Pressure p
Iter      SolEst     Damping    Stepsize #Res #Jac #Sol   LinErr   LinRes
   1      0.0011   0.5000000      0.0011   20   10   10  4.5e-15  7.9e-15
Displacement field
Iter      SolEst     Damping    Stepsize #Res #Jac #Sol   LinErr   LinRes
   1      0.0094   0.5000000      0.0094   20   10   10  1.1e-13  1.2e-12
Spatial mesh displacement
Iter      SolEst     Damping    Stepsize #Res #Jac #Sol   LinErr   LinRes
   1     8.2e-08   0.5000000     8.2e-08   20   10   10  1.5e-15  1.8e-15
Solution error estimates for segregated groups
0.00049, 0.00053, 0.0047, 4.1e-08
Residual error estimates for segregated groups
0.14, 84, 1.2e+03, 0.14
Segregated solver iteration 11.
Merged variables
Iter      SolEst     Damping    Stepsize #Res #Jac #Sol   LinErr   LinRes
   1     0.00019   0.3500000     0.00019   42   31   31  1.2e-13  3.4e-15
   2     0.00012   0.3500000     0.00012   43   32   32  8.9e-14  5.9e-15
   3     7.9e-05   0.3500000     7.9e-05   44   33   33  1.7e-13  3.9e-15
Velocity u2, Pressure p
Iter      SolEst     Damping    Stepsize #Res #Jac #Sol   LinErr   LinRes
   1     0.00053   0.5000000     0.00053   22   11   11  2.5e-15  7.9e-15
Displacement field
Iter      SolEst     Damping    Stepsize #Res #Jac #Sol   LinErr   LinRes
   1      0.0051   0.5000000      0.0051   22   11   11  2.1e-13  1.2e-12
Spatial mesh displacement
Iter      SolEst     Damping    Stepsize #Res #Jac #Sol   LinErr   LinRes
   1     4.9e-08   0.5000000     4.9e-08   22   11   11  1.4e-15  1.8e-15
Solution error estimates for segregated groups
0.00013, 0.00026, 0.0026, 2.4e-08
Residual error estimates for segregated groups
0.043, 42, 1.2e+03, 0.14
Segregated solver iteration 12.
Merged variables
Iter      SolEst     Damping    Stepsize #Res #Jac #Sol   LinErr   LinRes
   1     5.1e-05   0.3500000     5.1e-05   46   34   34  8.1e-14  5.1e-15
   2     3.3e-05   0.3500000     3.3e-05   47   35   35    1e-13  5.8e-15
   3     2.2e-05   0.3500000     2.2e-05   48   36   36  1.2e-13  9.8e-15
Velocity u2, Pressure p
Iter      SolEst     Damping    Stepsize #Res #Jac #Sol   LinErr   LinRes
   1     0.00026   0.5000000     0.00026   24   12   12  3.1e-15    8e-15
Displacement field
Iter      SolEst     Damping    Stepsize #Res #Jac #Sol   LinErr   LinRes
   1      0.0028   0.5000000      0.0028   24   12   12  1.1e-13  1.2e-12
Spatial mesh displacement
Iter      SolEst     Damping    Stepsize #Res #Jac #Sol   LinErr   LinRes
```

```
   1     2.9e-08   0.5000000     2.9e-08   24    12    12  1.1e-15  1.8e-15
Solution error estimates for segregated groups
3.7e-05, 0.00013, 0.0014, 1.4e-08
Residual error estimates for segregated groups
0.013, 21, 1.2e+03, 0.14
Segregated solver iteration 13.
Merged variables
Iter      SolEst     Damping    Stepsize #Res #Jac #Sol   LinErr   LinRes
   1     1.4e-05   0.3500000     1.4e-05   50    37    37  1.1e-13  5.9e-15
   2     9.1e-06   0.3500000     9.1e-06   51    38    38  1.1e-13  4.1e-15
   3     5.9e-06   0.3500000     5.9e-06   52    39    39  1.2e-13  4.6e-15
Velocity u2, Pressure p
Iter      SolEst     Damping    Stepsize #Res #Jac #Sol   LinErr   LinRes
   1     0.00013   0.5000000     0.00013   26    13    13  3.2e-15    8e-15
Displacement field
Iter      SolEst     Damping    Stepsize #Res #Jac #Sol   LinErr   LinRes
   1      0.0015   0.5000000      0.0015   26    13    13    1e-13  1.2e-12
Spatial mesh displacement
Iter      SolEst     Damping    Stepsize #Res #Jac #Sol   LinErr   LinRes
   1     1.7e-08   0.5000000     1.7e-08   26    13    13  1.5e-15  1.8e-15
Solution error estimates for segregated groups
1e-05, 6.6e-05, 0.00076, 8.4e-09
Residual error estimates for segregated groups
0.004, 11, 1.2e+03, 0.14
Solution time: 154 s. (2 minutes, 34 seconds)
Physical memory: 4.68 GB
Virtual memory: 25.55 GB
Ended at Jan 31, 2025, 8:34:10 PM.
----- Stationary Solver 1 in study1/Approach Curve Solver (sol1) -------------->
```

d=2095 (su83)

GENERAL

Description	Value
Solution	d=2095

LOG

```
<---- Compile Equations: Stationary in study1/Approach Curve Solver (sol1) -----
Started at Jan 31, 2025, 8:34:10 PM.
Parameter d = 2.0950000000000003E-6 (m).
Geometry shape function: Linear serendipity
Number of vertex elements: 18
Number of boundary elements: 6114
Number of elements: 154428
Minimum element quality: 0.4784
Time: 8 s.
Physical memory: 2.99 GB
Virtual memory: 15.25 GB
Ended at Jan 31, 2025, 8:34:18 PM.
----- Compile Equations: Stationary in study1/Approach Curve Solver (sol1) ---->

<---- Dependent Variables 1 in study1/Approach Curve Solver (sol1) -------------
Started at Jan 31, 2025, 8:34:18 PM.
Solution time: 0 s.
Physical memory: 2.99 GB
Virtual memory: 15.25 GB
Ended at Jan 31, 2025, 8:34:18 PM.
----- Dependent Variables 1 in study1/Approach Curve Solver (sol1) ------------>

<---- Stationary Solver 1 in study1/Approach Curve Solver (sol1) ---------------
Started at Jan 31, 2025, 8:34:19 PM.
Segregated solver
Segregated Step 11 does not have any components solved for.
Number of degrees of freedom solved for: 783754 (plus 11651 internal DOFs).
Segregated solver iteration 1.
Merged variables
Nonsymmetric matrix found.
Scales for dependent variables:
Concentration (comp1.Cl): 1.4e+02
Concentration (comp1.K): 1.4e+02
Electric potential (comp1.V): 1.9e+02
Orthonormal null-space function used.
Iter      SolEst     Damping    Stepsize #Res #Jac #Sol   LinErr   LinRes
   1        0.83   0.3500000        0.83    2    1    1  2.5e-11  2.7e-15
   2        0.81   0.3500000        0.81    3    2    2  4.3e-11  2.7e-15
   3         0.8   0.3500000         0.8    4    3    3  1.5e-11  2.7e-15
Velocity u2, Pressure p
Nonsymmetric matrix found.
Scales for dependent variables:
Pressure (comp1.p): 1
Velocity field (spatial frame) (comp1.u2): 0.00018
Orthonormal null-space function used.
Iter      SolEst     Damping    Stepsize #Res #Jac #Sol   LinErr   LinRes
   1         1.1   0.5000000         1.1    2    1    1  1.5e-15  2.3e-15
Displacement field
Nonsymmetric matrix found.
Scales for dependent variables:
Auxiliary pressure (comp1.solid.lemm1.pw): 1
Displacement field (comp1.u): 2.8e-18
Orthonormal null-space function used.
Iter      SolEst     Damping    Stepsize #Res #Jac #Sol   LinErr   LinRes
   1         1.9   0.5000000         1.9    2    1    1  3.6e-14  1.5e-12
Spatial mesh displacement
```

```
Symmetric matrices found.
Scales for dependent variables:
Spatial mesh displacement (comp1.spatial.disp): 1.2e-09
Orthonormal null-space function used.
Iter      SolEst     Damping    Stepsize #Res #Jac #Sol   LinErr   LinRes
   1     7.6e-07   0.5000000     7.6e-07    2    1    1  1.3e-15  1.8e-15
Solution error estimates for segregated groups
1.4, 0.54, 0.95, 3.8e-07
Residual error estimates for segregated groups
7.8e+03, 8.2e+04, 2.1e+04, 0.13
Segregated solver iteration 2.
Merged variables
Scales for dependent variables:
Concentration (comp1.Cl): 1.4e+02
Concentration (comp1.K): 1.4e+02
Electric potential (comp1.V): 0.064
Iter      SolEst     Damping    Stepsize #Res #Jac #Sol   LinErr   LinRes
   1        0.78   0.3500000        0.78    6    4    4  1.3e-13  2.9e-15
   2        0.77   0.3500000        0.77    7    5    5  9.4e-14  2.9e-15
   3        0.76   0.3500000        0.76    8    6    6  6.7e-14  2.9e-15
Velocity u2, Pressure p
Scales for dependent variables:
Pressure (comp1.p): 3.6e+02
Velocity field (spatial frame) (comp1.u2): 0.00073
Iter      SolEst     Damping    Stepsize #Res #Jac #Sol   LinErr   LinRes
   1        0.36   0.5000000        0.36    4    2    2    3e-15  7.7e-15
Displacement field
Iter      SolEst     Damping    Stepsize #Res #Jac #Sol   LinErr   LinRes
   1        0.95   0.5000000        0.95    4    2    2    1e-13  1.5e-12
Spatial mesh displacement
Iter      SolEst     Damping    Stepsize #Res #Jac #Sol   LinErr   LinRes
   1     1.1e-06   0.5000000     1.1e-06    4    2    2  1.4e-15  1.8e-15
Solution error estimates for segregated groups
1.3, 0.18, 0.48, 5.7e-07
Residual error estimates for segregated groups
7.7e+02, 1.6e+04, 2.2e+04, 0.13
Segregated solver iteration 3.
Merged variables
Iter      SolEst     Damping    Stepsize #Res #Jac #Sol   LinErr   LinRes
   1        0.74   0.3500000        0.74   10    7    7    6e-14  4.7e-15
   2        0.73   0.3500000        0.73   11    8    8  6.9e-14  4.5e-15
   3        0.72   0.3500000        0.72   12    9    9    8e-14  3.8e-15
Velocity u2, Pressure p
Iter      SolEst     Damping    Stepsize #Res #Jac #Sol   LinErr   LinRes
   1        0.15   0.5000000        0.15    6    3    3  3.9e-15  7.9e-15
Displacement field
Iter      SolEst     Damping    Stepsize #Res #Jac #Sol   LinErr   LinRes
   1        0.52   0.5000000        0.52    6    3    3  4.2e-14  1.5e-12
Spatial mesh displacement
Iter      SolEst     Damping    Stepsize #Res #Jac #Sol   LinErr   LinRes
   1     1.1e-06   0.5000000     1.1e-06    6    3    3  1.5e-15  1.8e-15
Solution error estimates for segregated groups
1.2, 0.077, 0.26, 5.7e-07
Residual error estimates for segregated groups
2.1e+02, 9.1e+03, 1.6e+04, 0.13
Segregated solver iteration 4.
```

```
Merged variables
Iter      SolEst     Damping    Stepsize #Res #Jac #Sol   LinErr   LinRes
   1         0.7   0.3500000         0.7   14   10   10  8.1e-14  3.9e-15
   2        0.68   0.3500000        0.68   15   11   11  1.9e-13  4.8e-15
   3        0.59   0.3500000        0.59   16   12   12  7.3e-14  3.4e-15
Velocity u2, Pressure p
Iter      SolEst     Damping    Stepsize #Res #Jac #Sol   LinErr   LinRes
   1       0.072   0.5000000       0.072    8    4    4  2.1e-15  7.8e-15
Displacement field
Iter      SolEst     Damping    Stepsize #Res #Jac #Sol   LinErr   LinRes
   1        0.29   0.5000000        0.29    8    4    4  6.5e-14  1.5e-12
Spatial mesh displacement
Iter      SolEst     Damping    Stepsize #Res #Jac #Sol   LinErr   LinRes
   1     9.5e-07   0.5000000     9.5e-07    8    4    4  1.6e-15  1.8e-15
Solution error estimates for segregated groups
1, 0.036, 0.15, 4.8e-07
Residual error estimates for segregated groups
82, 4.8e+03, 1.1e+04, 0.14
Segregated solver iteration 5.
Merged variables
Iter      SolEst     Damping    Stepsize #Res #Jac #Sol   LinErr   LinRes
   1         0.4   0.3500000         0.4   18   13   13  9.1e-14  4.7e-15
   2        0.27   0.3500000        0.27   19   14   14    6e-14  3.6e-15
   3        0.18   0.3500000        0.18   20   15   15  1.2e-13  3.1e-15
Velocity u2, Pressure p
Iter      SolEst     Damping    Stepsize #Res #Jac #Sol   LinErr   LinRes
   1       0.035   0.5000000       0.035   10    5    5  4.2e-15  7.8e-15
Displacement field
Iter      SolEst     Damping    Stepsize #Res #Jac #Sol   LinErr   LinRes
   1        0.17   0.5000000        0.17   10    5    5  4.1e-14  1.5e-12
Spatial mesh displacement
Iter      SolEst     Damping    Stepsize #Res #Jac #Sol   LinErr   LinRes
   1     7.2e-07   0.5000000     7.2e-07   10    5    5  1.5e-15  1.8e-15
Solution error estimates for segregated groups
0.31, 0.017, 0.084, 3.6e-07
Residual error estimates for segregated groups
32, 2.5e+03, 6.8e+03, 0.14
Segregated solver iteration 6.
Merged variables
Iter      SolEst     Damping    Stepsize #Res #Jac #Sol   LinErr   LinRes
   1        0.12   0.3500000        0.12   22   16   16  8.1e-14  3.3e-15
   2       0.077   0.3500000       0.077   23   17   17  7.3e-14  5.1e-15
   3        0.05   0.3500000        0.05   24   18   18  1.1e-13  5.6e-15
Velocity u2, Pressure p
Iter      SolEst     Damping    Stepsize #Res #Jac #Sol   LinErr   LinRes
   1       0.017   0.5000000       0.017   12    6    6  3.5e-15  7.9e-15
Displacement field
Scales for dependent variables:
Auxiliary pressure (comp1.solid.lemm1.pw): 0.36
Displacement field (comp1.u): 2.8e-16
Iter      SolEst     Damping    Stepsize #Res #Jac #Sol   LinErr   LinRes
   1       0.095   0.5000000       0.095   12    6    6  1.1e-13  1.2e-12
Spatial mesh displacement
Iter      SolEst     Damping    Stepsize #Res #Jac #Sol   LinErr   LinRes
   1       5e-07   0.5000000       5e-07   12    6    6  1.5e-15  1.8e-15
Solution error estimates for segregated groups
```

```
0.086, 0.0085, 0.048, 2.5e-07
Residual error estimates for segregated groups
12, 1.3e+03, 4.2e+03, 0.14
Segregated solver iteration 7.
Merged variables
Iter      SolEst     Damping    Stepsize #Res #Jac #Sol   LinErr   LinRes
   1       0.033   0.3500000       0.033   26   19   19    7e-14  3.2e-15
   2       0.021   0.3500000       0.021   27   20   20  6.3e-14  3.2e-15
   3       0.014   0.3500000       0.014   28   21   21  5.5e-14  3.6e-15
Velocity u2, Pressure p
Iter      SolEst     Damping    Stepsize #Res #Jac #Sol   LinErr   LinRes
   1      0.0085   0.5000000      0.0085   14    7    7  2.7e-15  7.9e-15
Displacement field
Iter      SolEst     Damping    Stepsize #Res #Jac #Sol   LinErr   LinRes
   1       0.054   0.5000000       0.054   14    7    7  1.6e-13  1.2e-12
Spatial mesh displacement
Iter      SolEst     Damping    Stepsize #Res #Jac #Sol   LinErr   LinRes
   1     3.3e-07   0.5000000     3.3e-07   14    7    7  1.5e-15  1.9e-15
Solution error estimates for segregated groups
0.024, 0.0042, 0.027, 1.7e-07
Residual error estimates for segregated groups
4.1, 6.3e+02, 2.6e+03, 0.13
Segregated solver iteration 8.
Merged variables
Iter      SolEst     Damping    Stepsize #Res #Jac #Sol   LinErr   LinRes
   1       0.009   0.3500000       0.009   30   22   22  8.3e-14    4e-15
   2      0.0058   0.3500000      0.0058   31   23   23  8.7e-14  3.3e-15
   3      0.0038   0.3500000      0.0038   32   24   24  6.6e-14    4e-15
Velocity u2, Pressure p
Iter      SolEst     Damping    Stepsize #Res #Jac #Sol   LinErr   LinRes
   1      0.0042   0.5000000      0.0042   16    8    8  2.5e-15  7.9e-15
Displacement field
Iter      SolEst     Damping    Stepsize #Res #Jac #Sol   LinErr   LinRes
   1        0.03   0.5000000        0.03   16    8    8  1.3e-13  1.2e-12
Spatial mesh displacement
Iter      SolEst     Damping    Stepsize #Res #Jac #Sol   LinErr   LinRes
   1     2.1e-07   0.5000000     2.1e-07   16    8    8  1.4e-15  1.9e-15
Solution error estimates for segregated groups
0.0065, 0.0021, 0.015, 1.1e-07
Residual error estimates for segregated groups
1.4, 3.2e+02, 1.8e+03, 0.14
Segregated solver iteration 9.
Merged variables
Iter      SolEst     Damping    Stepsize #Res #Jac #Sol   LinErr   LinRes
   1      0.0025   0.3500000      0.0025   34   25   25  6.1e-14    4e-15
   2      0.0016   0.3500000      0.0016   35   26   26  9.3e-14  4.4e-15
   3       0.001   0.3500000       0.001   36   27   27  5.3e-14  3.2e-15
Velocity u2, Pressure p
Iter      SolEst     Damping    Stepsize #Res #Jac #Sol   LinErr   LinRes
   1      0.0021   0.5000000      0.0021   18    9    9  2.1e-15  7.8e-15
Displacement field
Iter      SolEst     Damping    Stepsize #Res #Jac #Sol   LinErr   LinRes
   1       0.017   0.5000000       0.017   18    9    9  1.2e-13  1.2e-12
Spatial mesh displacement
Iter      SolEst     Damping    Stepsize #Res #Jac #Sol   LinErr   LinRes
   1     1.3e-07   0.5000000     1.3e-07   18    9    9  1.6e-15  1.8e-15
```

```
Solution error estimates for segregated groups
0.0018, 0.0011, 0.0085, 6.7e-08
Residual error estimates for segregated groups
0.44, 1.6e+02, 1.4e+03, 0.14
Segregated solver iteration 10.
Merged variables
Iter      SolEst     Damping    Stepsize #Res #Jac #Sol   LinErr   LinRes
   1     0.00068   0.3500000     0.00068   38   28   28  6.1e-14  3.9e-15
   2     0.00044   0.3500000     0.00044   39   29   29  8.9e-14  3.6e-15
   3     0.00029   0.3500000     0.00029   40   30   30  8.8e-14  6.6e-15
Velocity u2, Pressure p
Iter      SolEst     Damping    Stepsize #Res #Jac #Sol   LinErr   LinRes
   1      0.0011   0.5000000      0.0011   20   10   10  1.9e-15  7.9e-15
Displacement field
Iter      SolEst     Damping    Stepsize #Res #Jac #Sol   LinErr   LinRes
   1      0.0094   0.5000000      0.0094   20   10   10    1e-13  1.2e-12
Spatial mesh displacement
Iter      SolEst     Damping    Stepsize #Res #Jac #Sol   LinErr   LinRes
   1     8.2e-08   0.5000000     8.2e-08   20   10   10  1.6e-15  1.8e-15
Solution error estimates for segregated groups
0.00049, 0.00053, 0.0047, 4.1e-08
Residual error estimates for segregated groups
0.14, 79, 1.2e+03, 0.14
Segregated solver iteration 11.
Merged variables
Iter      SolEst     Damping    Stepsize #Res #Jac #Sol   LinErr   LinRes
   1     0.00019   0.3500000     0.00019   42   31   31  4.6e-14  5.7e-15
   2     0.00012   0.3500000     0.00012   43   32   32  4.5e-14  3.1e-15
   3     7.9e-05   0.3500000     7.9e-05   44   33   33  1.2e-13  3.9e-15
Velocity u2, Pressure p
Iter      SolEst     Damping    Stepsize #Res #Jac #Sol   LinErr   LinRes
   1     0.00053   0.5000000     0.00053   22   11   11  2.5e-15  7.9e-15
Displacement field
Iter      SolEst     Damping    Stepsize #Res #Jac #Sol   LinErr   LinRes
   1      0.0051   0.5000000      0.0051   22   11   11  2.2e-13  1.2e-12
Spatial mesh displacement
Iter      SolEst     Damping    Stepsize #Res #Jac #Sol   LinErr   LinRes
   1     4.9e-08   0.5000000     4.9e-08   22   11   11  1.6e-15  1.8e-15
Solution error estimates for segregated groups
0.00014, 0.00026, 0.0026, 2.5e-08
Residual error estimates for segregated groups
0.043, 40, 1.2e+03, 0.14
Segregated solver iteration 12.
Merged variables
Iter      SolEst     Damping    Stepsize #Res #Jac #Sol   LinErr   LinRes
   1     5.1e-05   0.3500000     5.1e-05   46   34   34  8.4e-14  5.9e-15
   2     3.3e-05   0.3500000     3.3e-05   47   35   35  1.5e-13  3.1e-15
   3     2.2e-05   0.3500000     2.2e-05   48   36   36  5.4e-14  4.9e-15
Velocity u2, Pressure p
Iter      SolEst     Damping    Stepsize #Res #Jac #Sol   LinErr   LinRes
   1     0.00026   0.5000000     0.00026   24   12   12  2.5e-15  7.7e-15
Displacement field
Iter      SolEst     Damping    Stepsize #Res #Jac #Sol   LinErr   LinRes
   1      0.0028   0.5000000      0.0028   24   12   12  1.3e-13  1.2e-12
Spatial mesh displacement
Iter      SolEst     Damping    Stepsize #Res #Jac #Sol   LinErr   LinRes
```

```
   1     2.9e-08   0.5000000     2.9e-08   24   12   12  1.8e-15  1.8e-15
Solution error estimates for segregated groups
3.7e-05, 0.00013, 0.0014, 1.5e-08
Residual error estimates for segregated groups
0.013, 20, 1.2e+03, 0.13
Segregated solver iteration 13.
Merged variables
Iter      SolEst     Damping    Stepsize #Res #Jac #Sol   LinErr   LinRes
   1     1.4e-05   0.3500000     1.4e-05   50   37   37  9.3e-14  3.2e-15
   2     9.1e-06   0.3500000     9.1e-06   51   38   38  3.3e-14  4.2e-15
   3     5.9e-06   0.3500000     5.9e-06   52   39   39  6.6e-14  2.9e-15
Velocity u2, Pressure p
Iter      SolEst     Damping    Stepsize #Res #Jac #Sol   LinErr   LinRes
   1     0.00013   0.5000000     0.00013   26   13   13  3.7e-15  7.9e-15
Displacement field
Iter      SolEst     Damping    Stepsize #Res #Jac #Sol   LinErr   LinRes
   1      0.0015   0.5000000      0.0015   26   13   13  1.2e-13  1.2e-12
Spatial mesh displacement
Iter      SolEst     Damping    Stepsize #Res #Jac #Sol   LinErr   LinRes
   1     1.7e-08   0.5000000     1.7e-08   26   13   13  1.5e-15  1.9e-15
Solution error estimates for segregated groups
1e-05, 6.6e-05, 0.00076, 8.5e-09
Residual error estimates for segregated groups
0.004, 9.9, 1.2e+03, 0.14
Solution time: 158 s. (2 minutes, 38 seconds)
Physical memory: 4.77 GB
Virtual memory: 25.6 GB
Ended at Jan 31, 2025, 8:36:56 PM.
----- Stationary Solver 1 in study1/Approach Curve Solver (sol1) -------------->
```

d=2090 (su84)

GENERAL

Description	Value
Solution	d=2090

LOG

```
<---- Compile Equations: Stationary in study1/Approach Curve Solver (sol1) -----
Started at Jan 31, 2025, 8:36:56 PM.
Parameter d = 2.0900000000000003E-6 (m).
Geometry shape function: Linear serendipity
Number of vertex elements: 18
Number of boundary elements: 6111
Number of elements: 154415
Minimum element quality: 0.4906
Time: 8 s.
Physical memory: 3.01 GB
Virtual memory: 15.27 GB
Ended at Jan 31, 2025, 8:37:05 PM.
----- Compile Equations: Stationary in study1/Approach Curve Solver (sol1) ---->

<---- Dependent Variables 1 in study1/Approach Curve Solver (sol1) -------------
Started at Jan 31, 2025, 8:37:05 PM.
Solution time: 0 s.
Physical memory: 3.01 GB
Virtual memory: 15.27 GB
Ended at Jan 31, 2025, 8:37:05 PM.
----- Dependent Variables 1 in study1/Approach Curve Solver (sol1) ------------>

<---- Stationary Solver 1 in study1/Approach Curve Solver (sol1) ---------------
Started at Jan 31, 2025, 8:37:05 PM.
Segregated solver
Segregated Step 11 does not have any components solved for.
Number of degrees of freedom solved for: 783729 (plus 11645 internal DOFs).
Segregated solver iteration 1.
Merged variables
Nonsymmetric matrix found.
Scales for dependent variables:
Concentration (comp1.Cl): 1.4e+02
Concentration (comp1.K): 1.4e+02
Electric potential (comp1.V): 1.9e+02
Orthonormal null-space function used.
Iter      SolEst     Damping    Stepsize #Res #Jac #Sol   LinErr   LinRes
   1        0.83   0.3500000        0.83    2    1    1    3e-11  2.8e-15
   2        0.81   0.3500000        0.81    3    2    2  2.8e-11  2.7e-15
   3         0.8   0.3500000         0.8    4    3    3  2.1e-11  2.7e-15
Velocity u2, Pressure p
Nonsymmetric matrix found.
Scales for dependent variables:
Pressure (comp1.p): 1
Velocity field (spatial frame) (comp1.u2): 0.00018
Orthonormal null-space function used.
Iter      SolEst     Damping    Stepsize #Res #Jac #Sol   LinErr   LinRes
   1         1.1   0.5000000         1.1    2    1    1  1.6e-15  2.2e-15
Displacement field
Nonsymmetric matrix found.
Scales for dependent variables:
Auxiliary pressure (comp1.solid.lemm1.pw): 1
Displacement field (comp1.u): 2.8e-18
Orthonormal null-space function used.
Iter      SolEst     Damping    Stepsize #Res #Jac #Sol   LinErr   LinRes
   1         1.9   0.5000000         1.9    2    1    1    5e-14  1.5e-12
Spatial mesh displacement
```

```
Symmetric matrices found.
Scales for dependent variables:
Spatial mesh displacement (comp1.spatial.disp): 1.2e-09
Orthonormal null-space function used.
Iter      SolEst     Damping    Stepsize #Res #Jac #Sol   LinErr   LinRes
   1     7.6e-07   0.5000000     7.6e-07    2    1    1  1.4e-15  1.8e-15
Solution error estimates for segregated groups
1.4, 0.54, 0.95, 3.8e-07
Residual error estimates for segregated groups
7.8e+03, 8.2e+04, 2.1e+04, 0.12
Segregated solver iteration 2.
Merged variables
Scales for dependent variables:
Concentration (comp1.Cl): 1.4e+02
Concentration (comp1.K): 1.4e+02
Electric potential (comp1.V): 0.064
Iter      SolEst     Damping    Stepsize #Res #Jac #Sol   LinErr   LinRes
   1        0.78   0.3500000        0.78    6    4    4  1.3e-13  3.3e-15
   2        0.77   0.3500000        0.77    7    5    5  6.3e-14  3.3e-15
   3        0.76   0.3500000        0.76    8    6    6  1.1e-13  3.1e-15
Velocity u2, Pressure p
Scales for dependent variables:
Pressure (comp1.p): 3.6e+02
Velocity field (spatial frame) (comp1.u2): 0.00073
Iter      SolEst     Damping    Stepsize #Res #Jac #Sol   LinErr   LinRes
   1        0.36   0.5000000        0.36    4    2    2  2.6e-15  7.8e-15
Displacement field
Iter      SolEst     Damping    Stepsize #Res #Jac #Sol   LinErr   LinRes
   1        0.95   0.5000000        0.95    4    2    2  5.1e-14  1.5e-12
Spatial mesh displacement
Iter      SolEst     Damping    Stepsize #Res #Jac #Sol   LinErr   LinRes
   1     1.1e-06   0.5000000     1.1e-06    4    2    2  1.6e-15  1.8e-15
Solution error estimates for segregated groups
1.3, 0.18, 0.48, 5.7e-07
Residual error estimates for segregated groups
7.7e+02, 1.5e+04, 2.1e+04, 0.13
Segregated solver iteration 3.
Merged variables
Iter      SolEst     Damping    Stepsize #Res #Jac #Sol   LinErr   LinRes
   1        0.74   0.3500000        0.74   10    7    7  1.3e-13  3.2e-15
   2        0.73   0.3500000        0.73   11    8    8  4.2e-14    3e-15
   3        0.71   0.3500000        0.71   12    9    9  6.2e-14  2.9e-15
Velocity u2, Pressure p
Iter      SolEst     Damping    Stepsize #Res #Jac #Sol   LinErr   LinRes
   1        0.15   0.5000000        0.15    6    3    3  2.1e-15    8e-15
Displacement field
Iter      SolEst     Damping    Stepsize #Res #Jac #Sol   LinErr   LinRes
   1        0.52   0.5000000        0.52    6    3    3  1.2e-13  1.4e-12
Spatial mesh displacement
Iter      SolEst     Damping    Stepsize #Res #Jac #Sol   LinErr   LinRes
   1     1.1e-06   0.5000000     1.1e-06    6    3    3  1.5e-15  1.8e-15
Solution error estimates for segregated groups
1.2, 0.077, 0.26, 5.7e-07
Residual error estimates for segregated groups
2.1e+02, 8.6e+03, 1.6e+04, 0.15
Segregated solver iteration 4.
```

```
Merged variables
Iter      SolEst     Damping    Stepsize #Res #Jac #Sol   LinErr   LinRes
   1         0.7   0.3500000         0.7   14   10   10  6.9e-14  3.1e-15
   2        0.68   0.3500000        0.68   15   11   11  1.2e-13  3.1e-15
   3        0.59   0.3500000        0.59   16   12   12    8e-14  2.9e-15
Velocity u2, Pressure p
Iter      SolEst     Damping    Stepsize #Res #Jac #Sol   LinErr   LinRes
   1       0.072   0.5000000       0.072    8    4    4  2.7e-15  7.7e-15
Displacement field
Iter      SolEst     Damping    Stepsize #Res #Jac #Sol   LinErr   LinRes
   1        0.29   0.5000000        0.29    8    4    4  3.6e-14  1.5e-12
Spatial mesh displacement
Iter      SolEst     Damping    Stepsize #Res #Jac #Sol   LinErr   LinRes
   1     9.5e-07   0.5000000     9.5e-07    8    4    4  1.4e-15  1.8e-15
Solution error estimates for segregated groups
1, 0.036, 0.15, 4.8e-07
Residual error estimates for segregated groups
82, 4.6e+03, 1.1e+04, 0.14
Segregated solver iteration 5.
Merged variables
Iter      SolEst     Damping    Stepsize #Res #Jac #Sol   LinErr   LinRes
   1         0.4   0.3500000         0.4   18   13   13    4e-14  3.2e-15
   2        0.27   0.3500000        0.27   19   14   14  5.7e-14  3.2e-15
   3        0.18   0.3500000        0.18   20   15   15  9.1e-14  3.2e-15
Velocity u2, Pressure p
Iter      SolEst     Damping    Stepsize #Res #Jac #Sol   LinErr   LinRes
   1       0.035   0.5000000       0.035   10    5    5  2.4e-15  7.8e-15
Displacement field
Iter      SolEst     Damping    Stepsize #Res #Jac #Sol   LinErr   LinRes
   1        0.17   0.5000000        0.17   10    5    5  1.3e-13  1.5e-12
Spatial mesh displacement
Iter      SolEst     Damping    Stepsize #Res #Jac #Sol   LinErr   LinRes
   1     7.2e-07   0.5000000     7.2e-07   10    5    5  1.6e-15  1.9e-15
Solution error estimates for segregated groups
0.31, 0.017, 0.084, 3.6e-07
Residual error estimates for segregated groups
32, 2.4e+03, 6.8e+03, 0.14
Segregated solver iteration 6.
Merged variables
Iter      SolEst     Damping    Stepsize #Res #Jac #Sol   LinErr   LinRes
   1        0.12   0.3500000        0.12   22   16   16  9.2e-14    3e-15
   2       0.077   0.3500000       0.077   23   17   17  5.7e-14  3.4e-15
   3        0.05   0.3500000        0.05   24   18   18  8.1e-14  3.2e-15
Velocity u2, Pressure p
Iter      SolEst     Damping    Stepsize #Res #Jac #Sol   LinErr   LinRes
   1       0.017   0.5000000       0.017   12    6    6  3.7e-15  7.9e-15
Displacement field
Scales for dependent variables:
Auxiliary pressure (comp1.solid.lemm1.pw): 0.36
Displacement field (comp1.u): 2.8e-16
Iter      SolEst     Damping    Stepsize #Res #Jac #Sol   LinErr   LinRes
   1       0.095   0.5000000       0.095   12    6    6  1.3e-13  1.2e-12
Spatial mesh displacement
Iter      SolEst     Damping    Stepsize #Res #Jac #Sol   LinErr   LinRes
   1       5e-07   0.5000000       5e-07   12    6    6  1.6e-15  1.8e-15
Solution error estimates for segregated groups
```

```
0.086, 0.0085, 0.048, 2.5e-07
Residual error estimates for segregated groups
12, 1.2e+03, 4.2e+03, 0.15
Segregated solver iteration 7.
Merged variables
Iter      SolEst     Damping    Stepsize #Res #Jac #Sol   LinErr   LinRes
   1       0.033   0.3500000       0.033   26   19   19     8e-14  2.9e-15
   2       0.021   0.3500000       0.021   27   20   20   4.8e-14  3.2e-15
   3       0.014   0.3500000       0.014   28   21   21   9.8e-14  2.8e-15
Velocity u2, Pressure p
Iter      SolEst     Damping    Stepsize #Res #Jac #Sol   LinErr   LinRes
   1      0.0085   0.5000000      0.0085   14    7    7   2.4e-15    8e-15
Displacement field
Iter      SolEst     Damping    Stepsize #Res #Jac #Sol   LinErr   LinRes
   1       0.054   0.5000000       0.054   14    7    7   1.5e-13  1.2e-12
Spatial mesh displacement
Iter      SolEst     Damping    Stepsize #Res #Jac #Sol   LinErr   LinRes
   1     3.3e-07   0.5000000     3.3e-07   14    7    7   1.4e-15  1.8e-15
Solution error estimates for segregated groups
0.024, 0.0042, 0.027, 1.7e-07
Residual error estimates for segregated groups
4.1, 6e+02, 2.6e+03, 0.14
Segregated solver iteration 8.
Merged variables
Iter      SolEst     Damping    Stepsize #Res #Jac #Sol   LinErr   LinRes
   1       0.009   0.3500000       0.009   30   22   22   1.4e-13  3.4e-15
   2      0.0058   0.3500000      0.0058   31   23   23   7.3e-14    3e-15
   3      0.0038   0.3500000      0.0038   32   24   24   8.1e-14  2.9e-15
Velocity u2, Pressure p
Iter      SolEst     Damping    Stepsize #Res #Jac #Sol   LinErr   LinRes
   1      0.0042   0.5000000      0.0042   16    8    8   3.2e-15  7.7e-15
Displacement field
Iter      SolEst     Damping    Stepsize #Res #Jac #Sol   LinErr   LinRes
   1        0.03   0.5000000        0.03   16    8    8     1e-13  1.2e-12
Spatial mesh displacement
Iter      SolEst     Damping    Stepsize #Res #Jac #Sol   LinErr   LinRes
   1     2.1e-07   0.5000000     2.1e-07   16    8    8   1.6e-15  1.8e-15
Solution error estimates for segregated groups
0.0065, 0.0021, 0.015, 1.1e-07
Residual error estimates for segregated groups
1.4, 3e+02, 1.8e+03, 0.15
Segregated solver iteration 9.
Merged variables
Iter      SolEst     Damping    Stepsize #Res #Jac #Sol   LinErr   LinRes
   1      0.0025   0.3500000      0.0025   34   25   25     9e-14  3.1e-15
   2      0.0016   0.3500000      0.0016   35   26   26     9e-14  2.9e-15
   3       0.001   0.3500000       0.001   36   27   27   9.9e-14  2.9e-15
Velocity u2, Pressure p
Iter      SolEst     Damping    Stepsize #Res #Jac #Sol   LinErr   LinRes
   1      0.0021   0.5000000      0.0021   18    9    9   2.7e-15  7.7e-15
Displacement field
Iter      SolEst     Damping    Stepsize #Res #Jac #Sol   LinErr   LinRes
   1       0.017   0.5000000       0.017   18    9    9   1.3e-13  1.2e-12
Spatial mesh displacement
Iter      SolEst     Damping    Stepsize #Res #Jac #Sol   LinErr   LinRes
   1     1.3e-07   0.5000000     1.3e-07   18    9    9   1.7e-15  1.8e-15
```

```
Solution error estimates for segregated groups
0.0018, 0.0011, 0.0085, 6.7e-08
Residual error estimates for segregated groups
0.44, 1.5e+02, 1.4e+03, 0.14
Segregated solver iteration 10.
Merged variables
Iter      SolEst     Damping    Stepsize #Res #Jac #Sol   LinErr   LinRes
   1     0.00068   0.3500000     0.00068   38   28   28  5.8e-14  3.3e-15
   2     0.00044   0.3500000     0.00044   39   29   29  7.2e-14  2.9e-15
   3     0.00029   0.3500000     0.00029   40   30   30  1.3e-13  2.9e-15
Velocity u2, Pressure p
Iter      SolEst     Damping    Stepsize #Res #Jac #Sol   LinErr   LinRes
   1      0.0011   0.5000000      0.0011   20   10   10  5.9e-15  7.7e-15
Displacement field
Iter      SolEst     Damping    Stepsize #Res #Jac #Sol   LinErr   LinRes
   1      0.0094   0.5000000      0.0094   20   10   10  1.4e-13  1.2e-12
Spatial mesh displacement
Iter      SolEst     Damping    Stepsize #Res #Jac #Sol   LinErr   LinRes
   1     8.2e-08   0.5000000     8.2e-08   20   10   10  1.4e-15  1.8e-15
Solution error estimates for segregated groups
0.00049, 0.00053, 0.0047, 4.1e-08
Residual error estimates for segregated groups
0.14, 75, 1.2e+03, 0.13
Segregated solver iteration 11.
Merged variables
Iter      SolEst     Damping    Stepsize #Res #Jac #Sol   LinErr   LinRes
   1     0.00019   0.3500000     0.00019   42   31   31  9.2e-14  3.2e-15
   2     0.00012   0.3500000     0.00012   43   32   32  1.4e-13  2.9e-15
   3     7.9e-05   0.3500000     7.9e-05   44   33   33  1.1e-13  3.1e-15
Velocity u2, Pressure p
Iter      SolEst     Damping    Stepsize #Res #Jac #Sol   LinErr   LinRes
   1     0.00053   0.5000000     0.00053   22   11   11  3.2e-15  7.9e-15
Displacement field
Iter      SolEst     Damping    Stepsize #Res #Jac #Sol   LinErr   LinRes
   1      0.0051   0.5000000      0.0051   22   11   11  1.2e-13  1.2e-12
Spatial mesh displacement
Iter      SolEst     Damping    Stepsize #Res #Jac #Sol   LinErr   LinRes
   1     4.9e-08   0.5000000     4.9e-08   22   11   11  1.8e-15  1.8e-15
Solution error estimates for segregated groups
0.00014, 0.00026, 0.0026, 2.5e-08
Residual error estimates for segregated groups
0.043, 38, 1.2e+03, 0.14
Segregated solver iteration 12.
Merged variables
Iter      SolEst     Damping    Stepsize #Res #Jac #Sol   LinErr   LinRes
   1     5.1e-05   0.3500000     5.1e-05   46   34   34  1.1e-13  3.1e-15
   2     3.3e-05   0.3500000     3.3e-05   47   35   35  7.2e-14    3e-15
   3     2.2e-05   0.3500000     2.2e-05   48   36   36  8.9e-14    4e-15
Velocity u2, Pressure p
Iter      SolEst     Damping    Stepsize #Res #Jac #Sol   LinErr   LinRes
   1     0.00026   0.5000000     0.00026   24   12   12    4e-15  7.8e-15
Displacement field
Iter      SolEst     Damping    Stepsize #Res #Jac #Sol   LinErr   LinRes
   1      0.0028   0.5000000      0.0028   24   12   12  1.4e-13  1.2e-12
Spatial mesh displacement
Iter      SolEst     Damping    Stepsize #Res #Jac #Sol   LinErr   LinRes
```

```
   1     2.9e-08   0.5000000     2.9e-08   24    12    12  2.3e-15  1.8e-15
Solution error estimates for segregated groups
3.7e-05, 0.00013, 0.0014, 1.5e-08
Residual error estimates for segregated groups
0.013, 19, 1.2e+03, 0.13
Segregated solver iteration 13.
Merged variables
Iter      SolEst      Damping    Stepsize #Res #Jac #Sol   LinErr   LinRes
   1     1.4e-05   0.3500000     1.4e-05   50    37    37  1.1e-13  3.8e-15
   2     9.1e-06   0.3500000     9.1e-06   51    38    38  9.4e-14    3e-15
   3     5.9e-06   0.3500000     5.9e-06   52    39    39    8e-14  3.2e-15
Velocity u2, Pressure p
Iter      SolEst      Damping    Stepsize #Res #Jac #Sol   LinErr   LinRes
   1     0.00013   0.5000000     0.00013   26    13    13  5.7e-15  7.7e-15
Displacement field
Iter      SolEst      Damping    Stepsize #Res #Jac #Sol   LinErr   LinRes
   1      0.0015   0.5000000      0.0015   26    13    13  1.1e-13  1.2e-12
Spatial mesh displacement
Iter      SolEst      Damping    Stepsize #Res #Jac #Sol   LinErr   LinRes
   1     1.7e-08   0.5000000     1.7e-08   26    13    13  1.4e-15  1.8e-15
Solution error estimates for segregated groups
1e-05, 6.6e-05, 0.00076, 8.5e-09
Residual error estimates for segregated groups
0.004, 9.4, 1.2e+03, 0.15
Solution time: 155 s. (2 minutes, 35 seconds)
Physical memory: 4.76 GB
Virtual memory: 26.04 GB
Ended at Jan 31, 2025, 8:39:40 PM.
----- Stationary Solver 1 in study1/Approach Curve Solver (sol1) -------------->
```

d=2085 (su85)

GENERAL

Description	Value
Solution	d=2085

LOG

```
<---- Compile Equations: Stationary in study1/Approach Curve Solver (sol1) -----
Started at Jan 31, 2025, 8:39:40 PM.
Parameter d = 2.085E-6 (m).
Geometry shape function: Linear serendipity
Number of vertex elements: 18
Number of boundary elements: 6109
Number of elements: 154107
Minimum element quality: 0.4771
Time: 8 s.
Physical memory: 3 GB
Virtual memory: 15.26 GB
Ended at Jan 31, 2025, 8:39:48 PM.
----- Compile Equations: Stationary in study1/Approach Curve Solver (sol1) ---->

<---- Dependent Variables 1 in study1/Approach Curve Solver (sol1) -------------
Started at Jan 31, 2025, 8:39:48 PM.
Solution time: 0 s.
Physical memory: 3 GB
Virtual memory: 15.26 GB
Ended at Jan 31, 2025, 8:39:49 PM.
----- Dependent Variables 1 in study1/Approach Curve Solver (sol1) ------------>

<---- Stationary Solver 1 in study1/Approach Curve Solver (sol1) ---------------
Started at Jan 31, 2025, 8:39:49 PM.
Segregated solver
Segregated Step 11 does not have any components solved for.
Number of degrees of freedom solved for: 782250 (plus 11641 internal DOFs).
Segregated solver iteration 1.
Merged variables
Nonsymmetric matrix found.
Scales for dependent variables:
Concentration (comp1.Cl): 1.4e+02
Concentration (comp1.K): 1.4e+02
Electric potential (comp1.V): 1.9e+02
Orthonormal null-space function used.
Iter      SolEst     Damping    Stepsize #Res #Jac #Sol   LinErr   LinRes
   1        0.83   0.3500000        0.83    2    1    1  4.5e-11  2.7e-15
   2        0.81   0.3500000        0.81    3    2    2  2.3e-11  2.7e-15
   3         0.8   0.3500000         0.8    4    3    3  3.2e-11  2.7e-15
Velocity u2, Pressure p
Nonsymmetric matrix found.
Scales for dependent variables:
Pressure (comp1.p): 1
Velocity field (spatial frame) (comp1.u2): 0.00018
Orthonormal null-space function used.
Iter      SolEst     Damping    Stepsize #Res #Jac #Sol   LinErr   LinRes
   1         1.1   0.5000000         1.1    2    1    1  2.5e-15  2.3e-15
Displacement field
Nonsymmetric matrix found.
Scales for dependent variables:
Auxiliary pressure (comp1.solid.lemm1.pw): 1
Displacement field (comp1.u): 2.8e-18
Orthonormal null-space function used.
Iter      SolEst     Damping    Stepsize #Res #Jac #Sol   LinErr   LinRes
   1         1.9   0.5000000         1.9    2    1    1  1.2e-13  1.5e-12
Spatial mesh displacement
```

```
Symmetric matrices found.
Scales for dependent variables:
Spatial mesh displacement (comp1.spatial.disp): 1.2e-09
Orthonormal null-space function used.
Iter      SolEst     Damping    Stepsize #Res #Jac #Sol   LinErr   LinRes
   1     7.7e-07   0.5000000     7.7e-07    2    1    1  1.6e-15  1.8e-15
Solution error estimates for segregated groups
1.4, 0.54, 0.95, 3.9e-07
Residual error estimates for segregated groups
7.8e+03, 8.2e+04, 2.1e+04, 0.13
Segregated solver iteration 2.
Merged variables
Scales for dependent variables:
Concentration (comp1.Cl): 1.4e+02
Concentration (comp1.K): 1.4e+02
Electric potential (comp1.V): 0.064
Iter      SolEst     Damping    Stepsize #Res #Jac #Sol   LinErr   LinRes
   1        0.78   0.3500000        0.78    6    4    4  5.7e-14  2.8e-15
   2        0.77   0.3500000        0.77    7    5    5    7e-14    3e-15
   3        0.76   0.3500000        0.76    8    6    6  7.3e-14  2.9e-15
Velocity u2, Pressure p
Scales for dependent variables:
Pressure (comp1.p): 3.6e+02
Velocity field (spatial frame) (comp1.u2): 0.00073
Iter      SolEst     Damping    Stepsize #Res #Jac #Sol   LinErr   LinRes
   1        0.36   0.5000000        0.36    4    2    2  2.2e-15  7.8e-15
Displacement field
Iter      SolEst     Damping    Stepsize #Res #Jac #Sol   LinErr   LinRes
   1        0.95   0.5000000        0.95    4    2    2  3.5e-14  1.5e-12
Spatial mesh displacement
Iter      SolEst     Damping    Stepsize #Res #Jac #Sol   LinErr   LinRes
   1     1.2e-06   0.5000000     1.2e-06    4    2    2  2.6e-15  1.8e-15
Solution error estimates for segregated groups
1.3, 0.18, 0.48, 5.8e-07
Residual error estimates for segregated groups
7.7e+02, 1.7e+04, 2.1e+04, 0.13
Segregated solver iteration 3.
Merged variables
Iter      SolEst     Damping    Stepsize #Res #Jac #Sol   LinErr   LinRes
   1        0.74   0.3500000        0.74   10    7    7  6.1e-14    3e-15
   2        0.73   0.3500000        0.73   11    8    8  8.1e-14  3.5e-15
   3        0.71   0.3500000        0.71   12    9    9  8.3e-14  2.8e-15
Velocity u2, Pressure p
Iter      SolEst     Damping    Stepsize #Res #Jac #Sol   LinErr   LinRes
   1        0.15   0.5000000        0.15    6    3    3  2.2e-15  7.8e-15
Displacement field
Iter      SolEst     Damping    Stepsize #Res #Jac #Sol   LinErr   LinRes
   1        0.52   0.5000000        0.52    6    3    3  1.2e-13  1.5e-12
Spatial mesh displacement
Iter      SolEst     Damping    Stepsize #Res #Jac #Sol   LinErr   LinRes
   1     1.2e-06   0.5000000     1.2e-06    6    3    3  1.7e-15  1.9e-15
Solution error estimates for segregated groups
1.2, 0.077, 0.26, 5.8e-07
Residual error estimates for segregated groups
2.1e+02, 9.5e+03, 1.6e+04, 0.14
Segregated solver iteration 4.
```

```
Merged variables
Iter      SolEst     Damping    Stepsize #Res #Jac #Sol   LinErr   LinRes
   1         0.7   0.3500000         0.7   14   10   10  1.2e-13  2.8e-15
   2        0.67   0.3500000        0.67   15   11   11  5.6e-14  2.7e-15
   3        0.58   0.3500000        0.58   16   12   12  1.5e-13  3.2e-15
Velocity u2, Pressure p
Iter      SolEst     Damping    Stepsize #Res #Jac #Sol   LinErr   LinRes
   1       0.072   0.5000000       0.072    8    4    4  2.8e-15    8e-15
Displacement field
Iter      SolEst     Damping    Stepsize #Res #Jac #Sol   LinErr   LinRes
   1        0.29   0.5000000        0.29    8    4    4  1.2e-13  1.4e-12
Spatial mesh displacement
Iter      SolEst     Damping    Stepsize #Res #Jac #Sol   LinErr   LinRes
   1     9.6e-07   0.5000000     9.6e-07    8    4    4  1.5e-15  1.9e-15
Solution error estimates for segregated groups
1, 0.036, 0.15, 4.8e-07
Residual error estimates for segregated groups
82, 5e+03, 1.1e+04, 0.13
Segregated solver iteration 5.
Merged variables
Iter      SolEst     Damping    Stepsize #Res #Jac #Sol   LinErr   LinRes
   1         0.4   0.3500000         0.4   18   13   13  1.3e-13  2.8e-15
   2        0.27   0.3500000        0.27   19   14   14  8.3e-14  3.2e-15
   3        0.18   0.3500000        0.18   20   15   15  6.4e-14  2.7e-15
Velocity u2, Pressure p
Iter      SolEst     Damping    Stepsize #Res #Jac #Sol   LinErr   LinRes
   1       0.035   0.5000000       0.035   10    5    5  2.8e-15  7.7e-15
Displacement field
Iter      SolEst     Damping    Stepsize #Res #Jac #Sol   LinErr   LinRes
   1        0.17   0.5000000        0.17   10    5    5  1.9e-13  1.5e-12
Spatial mesh displacement
Iter      SolEst     Damping    Stepsize #Res #Jac #Sol   LinErr   LinRes
   1     7.2e-07   0.5000000     7.2e-07   10    5    5  1.5e-15  1.8e-15
Solution error estimates for segregated groups
0.31, 0.017, 0.084, 3.6e-07
Residual error estimates for segregated groups
32, 2.6e+03, 6.8e+03, 0.14
Segregated solver iteration 6.
Merged variables
Iter      SolEst     Damping    Stepsize #Res #Jac #Sol   LinErr   LinRes
   1        0.12   0.3500000        0.12   22   16   16  3.2e-14  2.8e-15
   2       0.077   0.3500000       0.077   23   17   17  9.3e-14  2.8e-15
   3        0.05   0.3500000        0.05   24   18   18  7.4e-14  2.9e-15
Velocity u2, Pressure p
Iter      SolEst     Damping    Stepsize #Res #Jac #Sol   LinErr   LinRes
   1       0.017   0.5000000       0.017   12    6    6    3e-15  7.8e-15
Displacement field
Scales for dependent variables:
Auxiliary pressure (comp1.solid.lemm1.pw): 0.36
Displacement field (comp1.u): 2.9e-16
Iter      SolEst     Damping    Stepsize #Res #Jac #Sol   LinErr   LinRes
   1       0.095   0.5000000       0.095   12    6    6  1.5e-13  1.2e-12
Spatial mesh displacement
Iter      SolEst     Damping    Stepsize #Res #Jac #Sol   LinErr   LinRes
   1     5.1e-07   0.5000000     5.1e-07   12    6    6  1.5e-15  1.8e-15
Solution error estimates for segregated groups
```

```
0.086, 0.0085, 0.048, 2.5e-07
Residual error estimates for segregated groups
12, 1.3e+03, 4.2e+03, 0.14
Segregated solver iteration 7.
Merged variables
Iter      SolEst     Damping    Stepsize #Res #Jac #Sol   LinErr   LinRes
   1       0.033   0.3500000       0.033   26   19   19  1.2e-13  2.9e-15
   2       0.021   0.3500000       0.021   27   20   20  8.5e-14  2.8e-15
   3       0.014   0.3500000       0.014   28   21   21  1.1e-13  3.2e-15
Velocity u2, Pressure p
Iter      SolEst     Damping    Stepsize #Res #Jac #Sol   LinErr   LinRes
   1      0.0085   0.5000000      0.0085   14    7    7  2.3e-15  7.8e-15
Displacement field
Iter      SolEst     Damping    Stepsize #Res #Jac #Sol   LinErr   LinRes
   1       0.054   0.5000000       0.054   14    7    7  1.1e-13  1.2e-12
Spatial mesh displacement
Iter      SolEst     Damping    Stepsize #Res #Jac #Sol   LinErr   LinRes
   1     3.4e-07   0.5000000     3.4e-07   14    7    7  1.3e-15  1.8e-15
Solution error estimates for segregated groups
0.024, 0.0042, 0.027, 1.7e-07
Residual error estimates for segregated groups
4.1, 6.6e+02, 2.6e+03, 0.15
Segregated solver iteration 8.
Merged variables
Iter      SolEst     Damping    Stepsize #Res #Jac #Sol   LinErr   LinRes
   1       0.009   0.3500000       0.009   30   22   22  1.5e-13  3.1e-15
   2      0.0058   0.3500000      0.0058   31   23   23    1e-13  2.7e-15
   3      0.0038   0.3500000      0.0038   32   24   24  8.2e-14  3.2e-15
Velocity u2, Pressure p
Iter      SolEst     Damping    Stepsize #Res #Jac #Sol   LinErr   LinRes
   1      0.0042   0.5000000      0.0042   16    8    8  2.5e-15  7.8e-15
Displacement field
Iter      SolEst     Damping    Stepsize #Res #Jac #Sol   LinErr   LinRes
   1        0.03   0.5000000        0.03   16    8    8  1.3e-13  1.2e-12
Spatial mesh displacement
Iter      SolEst     Damping    Stepsize #Res #Jac #Sol   LinErr   LinRes
   1     2.2e-07   0.5000000     2.2e-07   16    8    8  1.5e-15  1.8e-15
Solution error estimates for segregated groups
0.0065, 0.0021, 0.015, 1.1e-07
Residual error estimates for segregated groups
1.4, 3.3e+02, 1.8e+03, 0.13
Segregated solver iteration 9.
Merged variables
Iter      SolEst     Damping    Stepsize #Res #Jac #Sol   LinErr   LinRes
   1      0.0025   0.3500000      0.0025   34   25   25  6.1e-14  2.7e-15
   2      0.0016   0.3500000      0.0016   35   26   26  5.7e-14  2.9e-15
   3       0.001   0.3500000       0.001   36   27   27  1.1e-13  3.9e-15
Velocity u2, Pressure p
Iter      SolEst     Damping    Stepsize #Res #Jac #Sol   LinErr   LinRes
   1      0.0021   0.5000000      0.0021   18    9    9    4e-15  7.9e-15
Displacement field
Iter      SolEst     Damping    Stepsize #Res #Jac #Sol   LinErr   LinRes
   1       0.017   0.5000000       0.017   18    9    9  1.1e-13  1.2e-12
Spatial mesh displacement
Iter      SolEst     Damping    Stepsize #Res #Jac #Sol   LinErr   LinRes
   1     1.4e-07   0.5000000     1.4e-07   18    9    9  1.4e-15  1.9e-15
```

```
Solution error estimates for segregated groups
0.0018, 0.0011, 0.0085, 6.8e-08
Residual error estimates for segregated groups
0.44, 1.7e+02, 1.4e+03, 0.14
Segregated solver iteration 10.
Merged variables
Iter      SolEst     Damping    Stepsize #Res #Jac #Sol   LinErr   LinRes
   1     0.00068   0.3500000     0.00068   38   28   28  1.1e-13  3.2e-15
   2     0.00044   0.3500000     0.00044   39   29   29  5.6e-14  3.3e-15
   3     0.00029   0.3500000     0.00029   40   30   30  9.1e-14  3.1e-15
Velocity u2, Pressure p
Iter      SolEst     Damping    Stepsize #Res #Jac #Sol   LinErr   LinRes
   1      0.0011   0.5000000      0.0011   20   10   10  2.1e-15  7.8e-15
Displacement field
Iter      SolEst     Damping    Stepsize #Res #Jac #Sol   LinErr   LinRes
   1      0.0094   0.5000000      0.0094   20   10   10  9.4e-14  1.2e-12
Spatial mesh displacement
Iter      SolEst     Damping    Stepsize #Res #Jac #Sol   LinErr   LinRes
   1     8.3e-08   0.5000000     8.3e-08   20   10   10  1.4e-15  1.8e-15
Solution error estimates for segregated groups
0.00049, 0.00053, 0.0047, 4.1e-08
Residual error estimates for segregated groups
0.14, 83, 1.2e+03, 0.14
Segregated solver iteration 11.
Merged variables
Iter      SolEst     Damping    Stepsize #Res #Jac #Sol   LinErr   LinRes
   1     0.00019   0.3500000     0.00019   42   31   31  8.9e-14  3.3e-15
   2     0.00012   0.3500000     0.00012   43   32   32  1.4e-13  2.8e-15
   3     7.9e-05   0.3500000     7.9e-05   44   33   33    9e-14  3.1e-15
Velocity u2, Pressure p
Iter      SolEst     Damping    Stepsize #Res #Jac #Sol   LinErr   LinRes
   1     0.00053   0.5000000     0.00053   22   11   11  5.6e-15  7.7e-15
Displacement field
Iter      SolEst     Damping    Stepsize #Res #Jac #Sol   LinErr   LinRes
   1      0.0051   0.5000000      0.0051   22   11   11  1.6e-13  1.2e-12
Spatial mesh displacement
Iter      SolEst     Damping    Stepsize #Res #Jac #Sol   LinErr   LinRes
   1       5e-08   0.5000000       5e-08   22   11   11  2.2e-15  1.8e-15
Solution error estimates for segregated groups
0.00014, 0.00026, 0.0026, 2.5e-08
Residual error estimates for segregated groups
0.043, 42, 1.2e+03, 0.14
Segregated solver iteration 12.
Merged variables
Iter      SolEst     Damping    Stepsize #Res #Jac #Sol   LinErr   LinRes
   1     5.1e-05   0.3500000     5.1e-05   46   34   34  4.9e-14  2.9e-15
   2     3.3e-05   0.3500000     3.3e-05   47   35   35  5.7e-14  2.8e-15
   3     2.2e-05   0.3500000     2.2e-05   48   36   36  7.8e-14  3.1e-15
Velocity u2, Pressure p
Iter      SolEst     Damping    Stepsize #Res #Jac #Sol   LinErr   LinRes
   1     0.00026   0.5000000     0.00026   24   12   12  2.2e-15  7.8e-15
Displacement field
Iter      SolEst     Damping    Stepsize #Res #Jac #Sol   LinErr   LinRes
   1      0.0028   0.5000000      0.0028   24   12   12    1e-13  1.2e-12
Spatial mesh displacement
Iter      SolEst     Damping    Stepsize #Res #Jac #Sol   LinErr   LinRes
```

```
   1      2.9e-08   0.5000000     2.9e-08   24    12    12  1.7e-15  1.8e-15
Solution error estimates for segregated groups
3.7e-05, 0.00013, 0.0014, 1.5e-08
Residual error estimates for segregated groups
0.013, 21, 1.2e+03, 0.15
Segregated solver iteration 13.
Merged variables
Iter      SolEst     Damping    Stepsize #Res #Jac #Sol   LinErr   LinRes
   1      1.4e-05   0.3500000     1.4e-05   50    37    37    7e-14    3e-15
   2      9.1e-06   0.3500000     9.1e-06   51    38    38  1.5e-13    3e-15
   3      5.9e-06   0.3500000     5.9e-06   52    39    39  7.5e-14  3.5e-15
Velocity u2, Pressure p
Iter      SolEst     Damping    Stepsize #Res #Jac #Sol   LinErr   LinRes
   1      0.00013   0.5000000     0.00013   26    13    13  2.3e-15  7.9e-15
Displacement field
Iter      SolEst     Damping    Stepsize #Res #Jac #Sol   LinErr   LinRes
   1       0.0015   0.5000000      0.0015   26    13    13  1.1e-13  1.2e-12
Spatial mesh displacement
Iter      SolEst     Damping    Stepsize #Res #Jac #Sol   LinErr   LinRes
   1      1.7e-08   0.5000000     1.7e-08   26    13    13  1.7e-15  1.8e-15
Solution error estimates for segregated groups
1e-05, 6.6e-05, 0.00076, 8.6e-09
Residual error estimates for segregated groups
0.004, 10, 1.2e+03, 0.14
Solution time: 153 s. (2 minutes, 33 seconds)
Physical memory: 4.74 GB
Virtual memory: 25.9 GB
Ended at Jan 31, 2025, 8:42:22 PM.
----- Stationary Solver 1 in study1/Approach Curve Solver (sol1) -------------->
```

d=2080 (su86)

GENERAL

Description	Value
Solution	d=2080

LOG

```
<---- Compile Equations: Stationary in study1/Approach Curve Solver (sol1) -----
Started at Jan 31, 2025, 8:42:22 PM.
Parameter d = 2.08E-6 (m).
Geometry shape function: Linear serendipity
Number of vertex elements: 18
Number of boundary elements: 6106
Number of elements: 154238
Minimum element quality: 0.4613
Time: 8 s.
Physical memory: 2.99 GB
Virtual memory: 15.25 GB
Ended at Jan 31, 2025, 8:42:30 PM.
----- Compile Equations: Stationary in study1/Approach Curve Solver (sol1) ---->

<---- Dependent Variables 1 in study1/Approach Curve Solver (sol1) -------------
Started at Jan 31, 2025, 8:42:30 PM.
Solution time: 0 s.
Physical memory: 2.99 GB
Virtual memory: 15.25 GB
Ended at Jan 31, 2025, 8:42:30 PM.
----- Dependent Variables 1 in study1/Approach Curve Solver (sol1) ------------>

<---- Stationary Solver 1 in study1/Approach Curve Solver (sol1) ---------------
Started at Jan 31, 2025, 8:42:30 PM.
Segregated solver
Segregated Step 11 does not have any components solved for.
Number of degrees of freedom solved for: 782707 (plus 11635 internal DOFs).
Segregated solver iteration 1.
Merged variables
Nonsymmetric matrix found.
Scales for dependent variables:
Concentration (comp1.Cl): 1.4e+02
Concentration (comp1.K): 1.4e+02
Electric potential (comp1.V): 1.9e+02
Orthonormal null-space function used.
Iter      SolEst     Damping    Stepsize #Res #Jac #Sol   LinErr   LinRes
   1        0.83   0.3500000        0.83    2    1    1  1.5e-11  2.7e-15
   2        0.81   0.3500000        0.81    3    2    2  2.3e-11  2.7e-15
   3         0.8   0.3500000         0.8    4    3    3    3e-11  2.7e-15
Velocity u2, Pressure p
Nonsymmetric matrix found.
Scales for dependent variables:
Pressure (comp1.p): 1
Velocity field (spatial frame) (comp1.u2): 0.00018
Orthonormal null-space function used.
Iter      SolEst     Damping    Stepsize #Res #Jac #Sol   LinErr   LinRes
   1         1.1   0.5000000         1.1    2    1    1  1.5e-15  2.3e-15
Displacement field
Nonsymmetric matrix found.
Scales for dependent variables:
Auxiliary pressure (comp1.solid.lemm1.pw): 1
Displacement field (comp1.u): 2.8e-18
Orthonormal null-space function used.
Iter      SolEst     Damping    Stepsize #Res #Jac #Sol   LinErr   LinRes
   1         1.9   0.5000000         1.9    2    1    1  3.2e-14  1.5e-12
Spatial mesh displacement
```

```
Symmetric matrices found.
Scales for dependent variables:
Spatial mesh displacement (comp1.spatial.disp): 1.2e-09
Orthonormal null-space function used.
Iter      SolEst     Damping    Stepsize #Res #Jac #Sol   LinErr   LinRes
   1     7.7e-07   0.5000000     7.7e-07    2    1    1  2.3e-15  1.8e-15
Solution error estimates for segregated groups
1.4, 0.54, 0.95, 3.9e-07
Residual error estimates for segregated groups
7.8e+03, 8.2e+04, 2.1e+04, 0.12
Segregated solver iteration 2.
Merged variables
Scales for dependent variables:
Concentration (comp1.Cl): 1.4e+02
Concentration (comp1.K): 1.4e+02
Electric potential (comp1.V): 0.064
Iter      SolEst     Damping    Stepsize #Res #Jac #Sol   LinErr   LinRes
   1        0.78   0.3500000        0.78    6    4    4  1.6e-13  7.1e-15
   2        0.77   0.3500000        0.77    7    5    5  6.7e-14  6.5e-15
   3        0.76   0.3500000        0.76    8    6    6    1e-13  3.5e-15
Velocity u2, Pressure p
Scales for dependent variables:
Pressure (comp1.p): 3.6e+02
Velocity field (spatial frame) (comp1.u2): 0.00073
Iter      SolEst     Damping    Stepsize #Res #Jac #Sol   LinErr   LinRes
   1        0.36   0.5000000        0.36    4    2    2  3.9e-15  7.9e-15
Displacement field
Iter      SolEst     Damping    Stepsize #Res #Jac #Sol   LinErr   LinRes
   1        0.95   0.5000000        0.95    4    2    2  9.8e-14  1.5e-12
Spatial mesh displacement
Iter      SolEst     Damping    Stepsize #Res #Jac #Sol   LinErr   LinRes
   1     1.2e-06   0.5000000     1.2e-06    4    2    2  1.6e-15  1.9e-15
Solution error estimates for segregated groups
1.3, 0.18, 0.48, 5.8e-07
Residual error estimates for segregated groups
7.7e+02, 1.5e+04, 2.1e+04, 0.12
Segregated solver iteration 3.
Merged variables
Iter      SolEst     Damping    Stepsize #Res #Jac #Sol   LinErr   LinRes
   1        0.74   0.3500000        0.74   10    7    7    8e-14  3.5e-15
   2        0.73   0.3500000        0.73   11    8    8  1.1e-13  4.6e-15
   3        0.71   0.3500000        0.71   12    9    9    1e-13  3.5e-15
Velocity u2, Pressure p
Iter      SolEst     Damping    Stepsize #Res #Jac #Sol   LinErr   LinRes
   1        0.15   0.5000000        0.15    6    3    3    2e-15  7.9e-15
Displacement field
Iter      SolEst     Damping    Stepsize #Res #Jac #Sol   LinErr   LinRes
   1        0.52   0.5000000        0.52    6    3    3  3.2e-14  1.5e-12
Spatial mesh displacement
Iter      SolEst     Damping    Stepsize #Res #Jac #Sol   LinErr   LinRes
   1     1.2e-06   0.5000000     1.2e-06    6    3    3  1.7e-15  1.8e-15
Solution error estimates for segregated groups
1.2, 0.077, 0.26, 5.8e-07
Residual error estimates for segregated groups
2.1e+02, 8.2e+03, 1.6e+04, 0.13
Segregated solver iteration 4.
```

```
Merged variables
Iter      SolEst     Damping    Stepsize #Res #Jac #Sol   LinErr   LinRes
   1         0.7   0.3500000         0.7   14   10   10  1.2e-13  1.1e-14
   2        0.67   0.3500000        0.67   15   11   11  1.5e-13  5.7e-15
   3        0.58   0.3500000        0.58   16   12   12    1e-13  7.5e-15
Velocity u2, Pressure p
Iter      SolEst     Damping    Stepsize #Res #Jac #Sol   LinErr   LinRes
   1       0.072   0.5000000       0.072    8    4    4  2.5e-15  7.9e-15
Displacement field
Iter      SolEst     Damping    Stepsize #Res #Jac #Sol   LinErr   LinRes
   1        0.29   0.5000000        0.29    8    4    4  3.6e-14  1.5e-12
Spatial mesh displacement
Iter      SolEst     Damping    Stepsize #Res #Jac #Sol   LinErr   LinRes
   1     9.7e-07   0.5000000     9.7e-07    8    4    4  1.5e-15  1.8e-15
Solution error estimates for segregated groups
1, 0.036, 0.15, 4.8e-07
Residual error estimates for segregated groups
82, 4.3e+03, 1.1e+04, 0.13
Segregated solver iteration 5.
Merged variables
Iter      SolEst     Damping    Stepsize #Res #Jac #Sol   LinErr   LinRes
   1         0.4   0.3500000         0.4   18   13   13  1.1e-13  6.6e-15
   2        0.27   0.3500000        0.27   19   14   14  1.5e-13  3.9e-15
   3        0.18   0.3500000        0.18   20   15   15  1.2e-13  8.7e-15
Velocity u2, Pressure p
Iter      SolEst     Damping    Stepsize #Res #Jac #Sol   LinErr   LinRes
   1       0.035   0.5000000       0.035   10    5    5  2.7e-15  7.8e-15
Displacement field
Iter      SolEst     Damping    Stepsize #Res #Jac #Sol   LinErr   LinRes
   1        0.17   0.5000000        0.17   10    5    5  3.2e-14  1.5e-12
Spatial mesh displacement
Iter      SolEst     Damping    Stepsize #Res #Jac #Sol   LinErr   LinRes
   1     7.3e-07   0.5000000     7.3e-07   10    5    5  1.7e-15  1.8e-15
Solution error estimates for segregated groups
0.31, 0.017, 0.084, 3.6e-07
Residual error estimates for segregated groups
32, 2.2e+03, 6.8e+03, 0.13
Segregated solver iteration 6.
Merged variables
Iter      SolEst     Damping    Stepsize #Res #Jac #Sol   LinErr   LinRes
   1        0.12   0.3500000        0.12   22   16   16    9e-14    1e-14
   2       0.076   0.3500000       0.076   23   17   17  1.2e-13    4e-15
   3        0.05   0.3500000        0.05   24   18   18  1.2e-13  5.5e-15
Velocity u2, Pressure p
Iter      SolEst     Damping    Stepsize #Res #Jac #Sol   LinErr   LinRes
   1       0.017   0.5000000       0.017   12    6    6  3.1e-15  7.8e-15
Displacement field
Scales for dependent variables:
Auxiliary pressure (comp1.solid.lemm1.pw): 0.36
Displacement field (comp1.u): 2.9e-16
Iter      SolEst     Damping    Stepsize #Res #Jac #Sol   LinErr   LinRes
   1       0.095   0.5000000       0.095   12    6    6  2.3e-13  1.2e-12
Spatial mesh displacement
Iter      SolEst     Damping    Stepsize #Res #Jac #Sol   LinErr   LinRes
   1     5.1e-07   0.5000000     5.1e-07   12    6    6  1.5e-15  1.9e-15
Solution error estimates for segregated groups
```

```
0.086, 0.0085, 0.048, 2.5e-07
Residual error estimates for segregated groups
12, 1.1e+03, 4.2e+03, 0.14
Segregated solver iteration 7.
Merged variables
Iter      SolEst     Damping    Stepsize #Res #Jac #Sol   LinErr   LinRes
   1       0.033   0.3500000       0.033   26   19   19  1.7e-13  3.4e-15
   2       0.021   0.3500000       0.021   27   20   20  8.2e-14  4.6e-15
   3       0.014   0.3500000       0.014   28   21   21    8e-14  5.6e-15
Velocity u2, Pressure p
Iter      SolEst     Damping    Stepsize #Res #Jac #Sol   LinErr   LinRes
   1      0.0085   0.5000000      0.0085   14    7    7  2.8e-15  7.9e-15
Displacement field
Iter      SolEst     Damping    Stepsize #Res #Jac #Sol   LinErr   LinRes
   1       0.054   0.5000000       0.054   14    7    7  1.2e-13  1.2e-12
Spatial mesh displacement
Iter      SolEst     Damping    Stepsize #Res #Jac #Sol   LinErr   LinRes
   1     3.4e-07   0.5000000     3.4e-07   14    7    7  1.8e-15  1.9e-15
Solution error estimates for segregated groups
0.024, 0.0042, 0.027, 1.7e-07
Residual error estimates for segregated groups
4.1, 5.7e+02, 2.6e+03, 0.14
Segregated solver iteration 8.
Merged variables
Iter      SolEst     Damping    Stepsize #Res #Jac #Sol   LinErr   LinRes
   1       0.009   0.3500000       0.009   30   22   22  7.1e-14  5.8e-15
   2      0.0058   0.3500000      0.0058   31   23   23  1.2e-13  3.5e-15
   3      0.0038   0.3500000      0.0038   32   24   24  9.3e-14  4.9e-15
Velocity u2, Pressure p
Iter      SolEst     Damping    Stepsize #Res #Jac #Sol   LinErr   LinRes
   1      0.0042   0.5000000      0.0042   16    8    8  3.9e-15  7.9e-15
Displacement field
Iter      SolEst     Damping    Stepsize #Res #Jac #Sol   LinErr   LinRes
   1        0.03   0.5000000        0.03   16    8    8  1.6e-13  1.2e-12
Spatial mesh displacement
Iter      SolEst     Damping    Stepsize #Res #Jac #Sol   LinErr   LinRes
   1     2.2e-07   0.5000000     2.2e-07   16    8    8  1.7e-15  1.8e-15
Solution error estimates for segregated groups
0.0065, 0.0021, 0.015, 1.1e-07
Residual error estimates for segregated groups
1.4, 2.9e+02, 1.8e+03, 0.13
Segregated solver iteration 9.
Merged variables
Iter      SolEst     Damping    Stepsize #Res #Jac #Sol   LinErr   LinRes
   1      0.0025   0.3500000      0.0025   34   25   25  8.9e-14  7.3e-15
   2      0.0016   0.3500000      0.0016   35   26   26    8e-14    5e-15
   3       0.001   0.3500000       0.001   36   27   27  2.1e-13  5.3e-15
Velocity u2, Pressure p
Iter      SolEst     Damping    Stepsize #Res #Jac #Sol   LinErr   LinRes
   1      0.0021   0.5000000      0.0021   18    9    9  2.3e-15  7.8e-15
Displacement field
Iter      SolEst     Damping    Stepsize #Res #Jac #Sol   LinErr   LinRes
   1       0.017   0.5000000       0.017   18    9    9  1.1e-13  1.2e-12
Spatial mesh displacement
Iter      SolEst     Damping    Stepsize #Res #Jac #Sol   LinErr   LinRes
   1     1.4e-07   0.5000000     1.4e-07   18    9    9  1.4e-15  1.8e-15
```

```
Solution error estimates for segregated groups
0.0018, 0.0011, 0.0085, 6.8e-08
Residual error estimates for segregated groups
0.44, 1.4e+02, 1.4e+03, 0.13
Segregated solver iteration 10.
Merged variables
Iter      SolEst     Damping    Stepsize #Res #Jac #Sol   LinErr   LinRes
   1     0.00068   0.3500000     0.00068   38   28   28  1.7e-13    5e-15
   2     0.00044   0.3500000     0.00044   39   29   29  8.3e-14  5.7e-15
   3     0.00029   0.3500000     0.00029   40   30   30  1.1e-13    8e-15
Velocity u2, Pressure p
Iter      SolEst     Damping    Stepsize #Res #Jac #Sol   LinErr   LinRes
   1      0.0011   0.5000000      0.0011   20   10   10  2.6e-15  7.9e-15
Displacement field
Iter      SolEst     Damping    Stepsize #Res #Jac #Sol   LinErr   LinRes
   1      0.0094   0.5000000      0.0094   20   10   10  1.2e-13  1.2e-12
Spatial mesh displacement
Iter      SolEst     Damping    Stepsize #Res #Jac #Sol   LinErr   LinRes
   1     8.3e-08   0.5000000     8.3e-08   20   10   10  2.4e-15  1.9e-15
Solution error estimates for segregated groups
0.00049, 0.00053, 0.0047, 4.2e-08
Residual error estimates for segregated groups
0.14, 71, 1.3e+03, 0.14
Segregated solver iteration 11.
Merged variables
Iter      SolEst     Damping    Stepsize #Res #Jac #Sol   LinErr   LinRes
   1     0.00019   0.3500000     0.00019   42   31   31  1.2e-13  3.4e-15
   2     0.00012   0.3500000     0.00012   43   32   32  1.5e-13  5.9e-15
   3     7.9e-05   0.3500000     7.9e-05   44   33   33    6e-14  5.1e-15
Velocity u2, Pressure p
Iter      SolEst     Damping    Stepsize #Res #Jac #Sol   LinErr   LinRes
   1     0.00053   0.5000000     0.00053   22   11   11  3.1e-15  7.8e-15
Displacement field
Iter      SolEst     Damping    Stepsize #Res #Jac #Sol   LinErr   LinRes
   1      0.0051   0.5000000      0.0051   22   11   11  1.5e-13  1.2e-12
Spatial mesh displacement
Iter      SolEst     Damping    Stepsize #Res #Jac #Sol   LinErr   LinRes
   1       5e-08   0.5000000       5e-08   22   11   11  1.3e-15  1.8e-15
Solution error estimates for segregated groups
0.00013, 0.00026, 0.0026, 2.5e-08
Residual error estimates for segregated groups
0.043, 36, 1.2e+03, 0.13
Segregated solver iteration 12.
Merged variables
Iter      SolEst     Damping    Stepsize #Res #Jac #Sol   LinErr   LinRes
   1     5.1e-05   0.3500000     5.1e-05   46   34   34  6.3e-14  5.3e-15
   2     3.3e-05   0.3500000     3.3e-05   47   35   35  1.3e-13    4e-15
   3     2.2e-05   0.3500000     2.2e-05   48   36   36  5.3e-14  4.1e-15
Velocity u2, Pressure p
Iter      SolEst     Damping    Stepsize #Res #Jac #Sol   LinErr   LinRes
   1     0.00026   0.5000000     0.00026   24   12   12  2.1e-15  7.8e-15
Displacement field
Iter      SolEst     Damping    Stepsize #Res #Jac #Sol   LinErr   LinRes
   1      0.0028   0.5000000      0.0028   24   12   12  1.6e-13  1.2e-12
Spatial mesh displacement
Iter      SolEst     Damping    Stepsize #Res #Jac #Sol   LinErr   LinRes
```

```
   1       3e-08   0.5000000       3e-08   24   12   12  1.6e-15  1.8e-15
Solution error estimates for segregated groups
3.7e-05, 0.00013, 0.0014, 1.5e-08
Residual error estimates for segregated groups
0.013, 18, 1.2e+03, 0.13
Segregated solver iteration 13.
Merged variables
Iter      SolEst      Damping    Stepsize #Res #Jac #Sol   LinErr   LinRes
   1     1.4e-05   0.3500000     1.4e-05   50   37   37  1.6e-13  1.3e-14
   2     9.1e-06   0.3500000     9.1e-06   51   38   38  1.4e-13  3.3e-15
   3     5.9e-06   0.3500000     5.9e-06   52   39   39  1.1e-13  3.4e-15
Velocity u2, Pressure p
Iter      SolEst      Damping    Stepsize #Res #Jac #Sol   LinErr   LinRes
   1     0.00013   0.5000000     0.00013   26   13   13  4.9e-15  7.9e-15
Displacement field
Iter      SolEst      Damping    Stepsize #Res #Jac #Sol   LinErr   LinRes
   1      0.0015   0.5000000      0.0015   26   13   13  1.4e-13  1.2e-12
Spatial mesh displacement
Iter      SolEst      Damping    Stepsize #Res #Jac #Sol   LinErr   LinRes
   1     1.7e-08   0.5000000     1.7e-08   26   13   13  1.3e-15  1.9e-15
Solution error estimates for segregated groups
1e-05, 6.6e-05, 0.00076, 8.6e-09
Residual error estimates for segregated groups
0.004, 8.9, 1.2e+03, 0.13
Solution time: 155 s. (2 minutes, 35 seconds)
Physical memory: 4.73 GB
Virtual memory: 25.59 GB
Ended at Jan 31, 2025, 8:45:06 PM.
----- Stationary Solver 1 in study1/Approach Curve Solver (sol1) -------------->
```

d=2075 (su87)

GENERAL

Description	Value
Solution	d=2075

LOG

```
<---- Compile Equations: Stationary in study1/Approach Curve Solver (sol1) -----
Started at Jan 31, 2025, 8:45:06 PM.
Parameter d = 2.075E-6 (m).
Geometry shape function: Linear serendipity
Number of vertex elements: 18
Number of boundary elements: 6104
Number of elements: 153924
Minimum element quality: 0.4886
Time: 8 s.
Physical memory: 2.99 GB
Virtual memory: 15.25 GB
Ended at Jan 31, 2025, 8:45:14 PM.
----- Compile Equations: Stationary in study1/Approach Curve Solver (sol1) ---->

<---- Dependent Variables 1 in study1/Approach Curve Solver (sol1) -------------
Started at Jan 31, 2025, 8:45:14 PM.
Solution time: 0 s.
Physical memory: 2.99 GB
Virtual memory: 15.25 GB
Ended at Jan 31, 2025, 8:45:14 PM.
----- Dependent Variables 1 in study1/Approach Curve Solver (sol1) ------------>

<---- Stationary Solver 1 in study1/Approach Curve Solver (sol1) ---------------
Started at Jan 31, 2025, 8:45:14 PM.
Segregated solver
Segregated Step 11 does not have any components solved for.
Number of degrees of freedom solved for: 781045 (plus 11631 internal DOFs).
Segregated solver iteration 1.
Merged variables
Nonsymmetric matrix found.
Scales for dependent variables:
Concentration (comp1.Cl): 1.4e+02
Concentration (comp1.K): 1.4e+02
Electric potential (comp1.V): 1.9e+02
Orthonormal null-space function used.
Iter      SolEst     Damping    Stepsize #Res #Jac #Sol   LinErr   LinRes
   1        0.83   0.3500000        0.83    2    1    1  2.1e-11  2.7e-15
   2        0.81   0.3500000        0.81    3    2    2  2.2e-11  2.7e-15
   3         0.8   0.3500000         0.8    4    3    3  5.6e-11  2.7e-15
Velocity u2, Pressure p
Nonsymmetric matrix found.
Scales for dependent variables:
Pressure (comp1.p): 1
Velocity field (spatial frame) (comp1.u2): 0.00018
Orthonormal null-space function used.
Iter      SolEst     Damping    Stepsize #Res #Jac #Sol   LinErr   LinRes
   1         1.1   0.5000000         1.1    2    1    1  2.2e-15  2.2e-15
Displacement field
Nonsymmetric matrix found.
Scales for dependent variables:
Auxiliary pressure (comp1.solid.lemm1.pw): 1
Displacement field (comp1.u): 2.8e-18
Orthonormal null-space function used.
Iter      SolEst     Damping    Stepsize #Res #Jac #Sol   LinErr   LinRes
   1         1.9   0.5000000         1.9    2    1    1  1.8e-13  1.5e-12
Spatial mesh displacement
```

```
Symmetric matrices found.
Scales for dependent variables:
Spatial mesh displacement (comp1.spatial.disp): 1.2e-09
Orthonormal null-space function used.
Iter      SolEst     Damping    Stepsize #Res #Jac #Sol   LinErr   LinRes
   1     7.8e-07   0.5000000     7.8e-07    2    1    1  1.5e-15  1.8e-15
Solution error estimates for segregated groups
1.4, 0.54, 0.95, 3.9e-07
Residual error estimates for segregated groups
7.8e+03, 8.2e+04, 2.1e+04, 0.12
Segregated solver iteration 2.
Merged variables
Scales for dependent variables:
Concentration (comp1.Cl): 1.4e+02
Concentration (comp1.K): 1.4e+02
Electric potential (comp1.V): 0.064
Iter      SolEst     Damping    Stepsize #Res #Jac #Sol   LinErr   LinRes
   1        0.78   0.3500000        0.78    6    4    4    1e-13  2.8e-15
   2        0.77   0.3500000        0.77    7    5    5  9.8e-14  2.9e-15
   3        0.76   0.3500000        0.76    8    6    6  4.6e-14  2.8e-15
Velocity u2, Pressure p
Scales for dependent variables:
Pressure (comp1.p): 3.7e+02
Velocity field (spatial frame) (comp1.u2): 0.00073
Iter      SolEst     Damping    Stepsize #Res #Jac #Sol   LinErr   LinRes
   1        0.36   0.5000000        0.36    4    2    2  2.4e-15  7.9e-15
Displacement field
Iter      SolEst     Damping    Stepsize #Res #Jac #Sol   LinErr   LinRes
   1        0.95   0.5000000        0.95    4    2    2  1.3e-13  1.5e-12
Spatial mesh displacement
Iter      SolEst     Damping    Stepsize #Res #Jac #Sol   LinErr   LinRes
   1     1.2e-06   0.5000000     1.2e-06    4    2    2  1.4e-15  1.8e-15
Solution error estimates for segregated groups
1.3, 0.18, 0.48, 5.8e-07
Residual error estimates for segregated groups
7.7e+02, 1.4e+04, 2.1e+04, 0.13
Segregated solver iteration 3.
Merged variables
Iter      SolEst     Damping    Stepsize #Res #Jac #Sol   LinErr   LinRes
   1        0.74   0.3500000        0.74   10    7    7  1.3e-13  2.7e-15
   2        0.73   0.3500000        0.73   11    8    8  3.9e-14    3e-15
   3        0.71   0.3500000        0.71   12    9    9  8.3e-14  2.9e-15
Velocity u2, Pressure p
Iter      SolEst     Damping    Stepsize #Res #Jac #Sol   LinErr   LinRes
   1        0.15   0.5000000        0.15    6    3    3  3.5e-15  7.7e-15
Displacement field
Iter      SolEst     Damping    Stepsize #Res #Jac #Sol   LinErr   LinRes
   1        0.52   0.5000000        0.52    6    3    3  6.8e-14  1.5e-12
Spatial mesh displacement
Iter      SolEst     Damping    Stepsize #Res #Jac #Sol   LinErr   LinRes
   1     1.2e-06   0.5000000     1.2e-06    6    3    3  1.4e-15  1.8e-15
Solution error estimates for segregated groups
1.2, 0.077, 0.26, 5.8e-07
Residual error estimates for segregated groups
2.1e+02, 7.7e+03, 1.6e+04, 0.14
Segregated solver iteration 4.
```

```
Merged variables
Iter      SolEst     Damping    Stepsize #Res #Jac #Sol   LinErr   LinRes
   1         0.7   0.3500000         0.7   14   10   10  1.4e-13  2.8e-15
   2        0.67   0.3500000        0.67   15   11   11  1.2e-13  2.8e-15
   3        0.58   0.3500000        0.58   16   12   12  8.2e-14  2.7e-15
Velocity u2, Pressure p
Iter      SolEst     Damping    Stepsize #Res #Jac #Sol   LinErr   LinRes
   1       0.072   0.5000000       0.072    8    4    4  3.2e-15  7.8e-15
Displacement field
Iter      SolEst     Damping    Stepsize #Res #Jac #Sol   LinErr   LinRes
   1        0.29   0.5000000        0.29    8    4    4  5.8e-14  1.5e-12
Spatial mesh displacement
Iter      SolEst     Damping    Stepsize #Res #Jac #Sol   LinErr   LinRes
   1     9.7e-07   0.5000000     9.7e-07    8    4    4  1.5e-15  1.9e-15
Solution error estimates for segregated groups
1, 0.036, 0.15, 4.9e-07
Residual error estimates for segregated groups
82, 4.1e+03, 1.1e+04, 0.15
Segregated solver iteration 5.
Merged variables
Iter      SolEst     Damping    Stepsize #Res #Jac #Sol   LinErr   LinRes
   1         0.4   0.3500000         0.4   18   13   13  6.8e-14  2.8e-15
   2        0.27   0.3500000        0.27   19   14   14  9.1e-14  2.8e-15
   3        0.18   0.3500000        0.18   20   15   15  7.4e-14  3.1e-15
Velocity u2, Pressure p
Iter      SolEst     Damping    Stepsize #Res #Jac #Sol   LinErr   LinRes
   1       0.035   0.5000000       0.035   10    5    5  1.7e-15  7.7e-15
Displacement field
Iter      SolEst     Damping    Stepsize #Res #Jac #Sol   LinErr   LinRes
   1        0.17   0.5000000        0.17   10    5    5  2.4e-13  1.4e-12
Spatial mesh displacement
Iter      SolEst     Damping    Stepsize #Res #Jac #Sol   LinErr   LinRes
   1     7.3e-07   0.5000000     7.3e-07   10    5    5  1.6e-15  1.8e-15
Solution error estimates for segregated groups
0.3, 0.017, 0.084, 3.7e-07
Residual error estimates for segregated groups
32, 2.1e+03, 6.8e+03, 0.13
Segregated solver iteration 6.
Merged variables
Iter      SolEst     Damping    Stepsize #Res #Jac #Sol   LinErr   LinRes
   1        0.12   0.3500000        0.12   22   16   16    1e-13  2.7e-15
   2       0.076   0.3500000       0.076   23   17   17  7.5e-14    3e-15
   3        0.05   0.3500000        0.05   24   18   18  8.4e-14  2.7e-15
Velocity u2, Pressure p
Iter      SolEst     Damping    Stepsize #Res #Jac #Sol   LinErr   LinRes
   1       0.017   0.5000000       0.017   12    6    6  1.9e-15  7.8e-15
Displacement field
Scales for dependent variables:
Auxiliary pressure (comp1.solid.lemm1.pw): 0.36
Displacement field (comp1.u): 2.9e-16
Iter      SolEst     Damping    Stepsize #Res #Jac #Sol   LinErr   LinRes
   1       0.095   0.5000000       0.095   12    6    6  1.2e-13  1.2e-12
Spatial mesh displacement
Iter      SolEst     Damping    Stepsize #Res #Jac #Sol   LinErr   LinRes
   1     5.1e-07   0.5000000     5.1e-07   12    6    6  1.4e-15  1.9e-15
Solution error estimates for segregated groups
```

```
0.085, 0.0085, 0.048, 2.6e-07
Residual error estimates for segregated groups
12, 1.1e+03, 4.2e+03, 0.14
Segregated solver iteration 7.
Merged variables
Iter      SolEst     Damping    Stepsize #Res #Jac #Sol   LinErr   LinRes
   1       0.032   0.3500000       0.032   26   19   19  9.4e-14  2.8e-15
   2       0.021   0.3500000       0.021   27   20   20  6.8e-14  2.7e-15
   3       0.014   0.3500000       0.014   28   21   21  1.4e-13  3.1e-15
Velocity u2, Pressure p
Iter      SolEst     Damping    Stepsize #Res #Jac #Sol   LinErr   LinRes
   1      0.0085   0.5000000      0.0085   14    7    7  2.8e-15  7.8e-15
Displacement field
Iter      SolEst     Damping    Stepsize #Res #Jac #Sol   LinErr   LinRes
   1       0.054   0.5000000       0.054   14    7    7  1.1e-13  1.2e-12
Spatial mesh displacement
Iter      SolEst     Damping    Stepsize #Res #Jac #Sol   LinErr   LinRes
   1     3.4e-07   0.5000000     3.4e-07   14    7    7  1.7e-15  1.8e-15
Solution error estimates for segregated groups
0.024, 0.0042, 0.027, 1.7e-07
Residual error estimates for segregated groups
4.1, 5.3e+02, 2.6e+03, 0.14
Segregated solver iteration 8.
Merged variables
Iter      SolEst     Damping    Stepsize #Res #Jac #Sol   LinErr   LinRes
   1      0.0089   0.3500000      0.0089   30   22   22  1.2e-13  2.9e-15
   2      0.0058   0.3500000      0.0058   31   23   23  9.5e-14  2.6e-15
   3      0.0038   0.3500000      0.0038   32   24   24  6.1e-14  3.1e-15
Velocity u2, Pressure p
Iter      SolEst     Damping    Stepsize #Res #Jac #Sol   LinErr   LinRes
   1      0.0042   0.5000000      0.0042   16    8    8  2.8e-15  7.7e-15
Displacement field
Iter      SolEst     Damping    Stepsize #Res #Jac #Sol   LinErr   LinRes
   1        0.03   0.5000000        0.03   16    8    8  1.5e-13  1.2e-12
Spatial mesh displacement
Iter      SolEst     Damping    Stepsize #Res #Jac #Sol   LinErr   LinRes
   1     2.2e-07   0.5000000     2.2e-07   16    8    8  1.5e-15  1.8e-15
Solution error estimates for segregated groups
0.0065, 0.0021, 0.015, 1.1e-07
Residual error estimates for segregated groups
1.3, 2.7e+02, 1.8e+03, 0.13
Segregated solver iteration 9.
Merged variables
Iter      SolEst     Damping    Stepsize #Res #Jac #Sol   LinErr   LinRes
   1      0.0025   0.3500000      0.0025   34   25   25  1.1e-13  3.1e-15
   2      0.0016   0.3500000      0.0016   35   26   26  4.3e-14  2.7e-15
   3       0.001   0.3500000       0.001   36   27   27  1.2e-13  2.7e-15
Velocity u2, Pressure p
Iter      SolEst     Damping    Stepsize #Res #Jac #Sol   LinErr   LinRes
   1      0.0021   0.5000000      0.0021   18    9    9  5.2e-15  7.7e-15
Displacement field
Iter      SolEst     Damping    Stepsize #Res #Jac #Sol   LinErr   LinRes
   1       0.017   0.5000000       0.017   18    9    9  1.6e-13  1.2e-12
Spatial mesh displacement
Iter      SolEst     Damping    Stepsize #Res #Jac #Sol   LinErr   LinRes
   1     1.4e-07   0.5000000     1.4e-07   18    9    9  1.1e-15  1.9e-15
```

```
Solution error estimates for segregated groups
0.0018, 0.0011, 0.0085, 6.8e-08
Residual error estimates for segregated groups
0.44, 1.3e+02, 1.4e+03, 0.12
Segregated solver iteration 10.
Merged variables
Iter      SolEst     Damping    Stepsize #Res #Jac #Sol   LinErr   LinRes
   1     0.00067   0.3500000     0.00067   38   28   28    7e-14  2.7e-15
   2     0.00044   0.3500000     0.00044   39   29   29  3.3e-14  2.9e-15
   3     0.00029   0.3500000     0.00029   40   30   30  2.5e-14  2.9e-15
Velocity u2, Pressure p
Iter      SolEst     Damping    Stepsize #Res #Jac #Sol   LinErr   LinRes
   1      0.0011   0.5000000      0.0011   20   10   10  3.8e-15  7.8e-15
Displacement field
Iter      SolEst     Damping    Stepsize #Res #Jac #Sol   LinErr   LinRes
   1      0.0094   0.5000000      0.0094   20   10   10  1.3e-13  1.2e-12
Spatial mesh displacement
Iter      SolEst     Damping    Stepsize #Res #Jac #Sol   LinErr   LinRes
   1     8.4e-08   0.5000000     8.4e-08   20   10   10    2e-15  1.9e-15
Solution error estimates for segregated groups
0.00049, 0.00053, 0.0047, 4.2e-08
Residual error estimates for segregated groups
0.14, 67, 1.2e+03, 0.12
Segregated solver iteration 11.
Merged variables
Iter      SolEst     Damping    Stepsize #Res #Jac #Sol   LinErr   LinRes
   1     0.00019   0.3500000     0.00019   42   31   31    1e-13  2.9e-15
   2     0.00012   0.3500000     0.00012   43   32   32  9.7e-14  2.9e-15
   3     7.8e-05   0.3500000     7.8e-05   44   33   33  6.8e-14  2.9e-15
Velocity u2, Pressure p
Iter      SolEst     Damping    Stepsize #Res #Jac #Sol   LinErr   LinRes
   1     0.00053   0.5000000     0.00053   22   11   11  3.1e-15  7.9e-15
Displacement field
Iter      SolEst     Damping    Stepsize #Res #Jac #Sol   LinErr   LinRes
   1      0.0051   0.5000000      0.0051   22   11   11  1.5e-13  1.2e-12
Spatial mesh displacement
Iter      SolEst     Damping    Stepsize #Res #Jac #Sol   LinErr   LinRes
   1       5e-08   0.5000000       5e-08   22   11   11  1.2e-15  1.8e-15
Solution error estimates for segregated groups
0.00013, 0.00026, 0.0026, 2.5e-08
Residual error estimates for segregated groups
0.043, 33, 1.2e+03, 0.14
Segregated solver iteration 12.
Merged variables
Iter      SolEst     Damping    Stepsize #Res #Jac #Sol   LinErr   LinRes
   1     5.1e-05   0.3500000     5.1e-05   46   34   34  5.3e-14  2.8e-15
   2     3.3e-05   0.3500000     3.3e-05   47   35   35    8e-14  2.9e-15
   3     2.2e-05   0.3500000     2.2e-05   48   36   36  4.1e-14  2.6e-15
Velocity u2, Pressure p
Iter      SolEst     Damping    Stepsize #Res #Jac #Sol   LinErr   LinRes
   1     0.00026   0.5000000     0.00026   24   12   12  4.9e-15  7.8e-15
Displacement field
Iter      SolEst     Damping    Stepsize #Res #Jac #Sol   LinErr   LinRes
   1      0.0028   0.5000000      0.0028   24   12   12  1.4e-13  1.2e-12
Spatial mesh displacement
Iter      SolEst     Damping    Stepsize #Res #Jac #Sol   LinErr   LinRes
```

```
   1       3e-08   0.5000000       3e-08   24   12   12  1.5e-15  1.8e-15
Solution error estimates for segregated groups
3.7e-05, 0.00013, 0.0014, 1.5e-08
Residual error estimates for segregated groups
0.013, 17, 1.2e+03, 0.13
Segregated solver iteration 13.
Merged variables
Iter      SolEst     Damping    Stepsize #Res #Jac #Sol   LinErr   LinRes
   1     1.4e-05   0.3500000     1.4e-05   50   37   37  7.4e-14  2.8e-15
   2     9.1e-06   0.3500000     9.1e-06   51   38   38  8.9e-14  3.3e-15
   3     5.9e-06   0.3500000     5.9e-06   52   39   39  6.3e-14  2.8e-15
Velocity u2, Pressure p
Iter      SolEst     Damping    Stepsize #Res #Jac #Sol   LinErr   LinRes
   1     0.00013   0.5000000     0.00013   26   13   13  5.5e-15  7.8e-15
Displacement field
Iter      SolEst     Damping    Stepsize #Res #Jac #Sol   LinErr   LinRes
   1      0.0015   0.5000000      0.0015   26   13   13    1e-13  1.2e-12
Spatial mesh displacement
Iter      SolEst     Damping    Stepsize #Res #Jac #Sol   LinErr   LinRes
   1     1.7e-08   0.5000000     1.7e-08   26   13   13  1.7e-15  1.8e-15
Solution error estimates for segregated groups
1e-05, 6.6e-05, 0.00076, 8.7e-09
Residual error estimates for segregated groups
0.004, 8.4, 1.2e+03, 0.13
Solution time: 151 s. (2 minutes, 31 seconds)
Physical memory: 4.68 GB
Virtual memory: 25.91 GB
Ended at Jan 31, 2025, 8:47:45 PM.
----- Stationary Solver 1 in study1/Approach Curve Solver (sol1) -------------->
```

d=2070 (su88)

GENERAL

Description	Value
Solution	d=2070

LOG

```
<---- Compile Equations: Stationary in study1/Approach Curve Solver (sol1) -----
Started at Jan 31, 2025, 8:47:45 PM.
Parameter d = 2.07E-6 (m).
Geometry shape function: Linear serendipity
Number of vertex elements: 18
Number of boundary elements: 6101
Number of elements: 154185
Minimum element quality: 0.4979
Time: 8 s.
Physical memory: 3.04 GB
Virtual memory: 15.3 GB
Ended at Jan 31, 2025, 8:47:53 PM.
----- Compile Equations: Stationary in study1/Approach Curve Solver (sol1) ---->

<---- Dependent Variables 1 in study1/Approach Curve Solver (sol1) -------------
Started at Jan 31, 2025, 8:47:53 PM.
Solution time: 0 s.
Physical memory: 3.04 GB
Virtual memory: 15.3 GB
Ended at Jan 31, 2025, 8:47:53 PM.
----- Dependent Variables 1 in study1/Approach Curve Solver (sol1) ------------>

<---- Stationary Solver 1 in study1/Approach Curve Solver (sol1) ---------------
Started at Jan 31, 2025, 8:47:53 PM.
Segregated solver
Segregated Step 11 does not have any components solved for.
Number of degrees of freedom solved for: 782307 (plus 11625 internal DOFs).
Segregated solver iteration 1.
Merged variables
Nonsymmetric matrix found.
Scales for dependent variables:
Concentration (comp1.Cl): 1.4e+02
Concentration (comp1.K): 1.4e+02
Electric potential (comp1.V): 1.9e+02
Orthonormal null-space function used.
Iter      SolEst     Damping    Stepsize #Res #Jac #Sol   LinErr   LinRes
   1        0.83   0.3500000        0.83    2    1    1  4.3e-11  2.7e-15
   2        0.81   0.3500000        0.81    3    2    2    5e-11  2.7e-15
   3         0.8   0.3500000         0.8    4    3    3  8.3e-11  2.7e-15
Velocity u2, Pressure p
Nonsymmetric matrix found.
Scales for dependent variables:
Pressure (comp1.p): 1
Velocity field (spatial frame) (comp1.u2): 0.00018
Orthonormal null-space function used.
Iter      SolEst     Damping    Stepsize #Res #Jac #Sol   LinErr   LinRes
   1         1.1   0.5000000         1.1    2    1    1  1.9e-15  2.3e-15
Displacement field
Nonsymmetric matrix found.
Scales for dependent variables:
Auxiliary pressure (comp1.solid.lemm1.pw): 1
Displacement field (comp1.u): 2.8e-18
Orthonormal null-space function used.
Iter      SolEst     Damping    Stepsize #Res #Jac #Sol   LinErr   LinRes
   1         1.9   0.5000000         1.9    2    1    1  3.3e-14  1.5e-12
Spatial mesh displacement
```

```
Symmetric matrices found.
Scales for dependent variables:
Spatial mesh displacement (comp1.spatial.disp): 1.2e-09
Orthonormal null-space function used.
Iter      SolEst     Damping    Stepsize #Res #Jac #Sol   LinErr   LinRes
   1     7.8e-07   0.5000000     7.8e-07    2    1    1  1.4e-15  1.8e-15
Solution error estimates for segregated groups
1.4, 0.54, 0.95, 3.9e-07
Residual error estimates for segregated groups
7.8e+03, 8.2e+04, 2.1e+04, 0.12
Segregated solver iteration 2.
Merged variables
Scales for dependent variables:
Concentration (comp1.Cl): 1.4e+02
Concentration (comp1.K): 1.4e+02
Electric potential (comp1.V): 0.064
Iter      SolEst     Damping    Stepsize #Res #Jac #Sol   LinErr   LinRes
   1        0.78   0.3500000        0.78    6    4    4  1.1e-13  3.2e-15
   2        0.77   0.3500000        0.77    7    5    5  6.1e-14  3.5e-15
   3        0.76   0.3500000        0.76    8    6    6  1.9e-13    4e-15
Velocity u2, Pressure p
Scales for dependent variables:
Pressure (comp1.p): 3.6e+02
Velocity field (spatial frame) (comp1.u2): 0.00073
Iter      SolEst     Damping    Stepsize #Res #Jac #Sol   LinErr   LinRes
   1        0.36   0.5000000        0.36    4    2    2  3.1e-15  7.9e-15
Displacement field
Iter      SolEst     Damping    Stepsize #Res #Jac #Sol   LinErr   LinRes
   1        0.95   0.5000000        0.95    4    2    2  5.3e-14  1.5e-12
Spatial mesh displacement
Iter      SolEst     Damping    Stepsize #Res #Jac #Sol   LinErr   LinRes
   1     1.2e-06   0.5000000     1.2e-06    4    2    2  1.7e-15  1.9e-15
Solution error estimates for segregated groups
1.3, 0.18, 0.48, 5.8e-07
Residual error estimates for segregated groups
7.6e+02, 1.4e+04, 2.2e+04, 0.13
Segregated solver iteration 3.
Merged variables
Iter      SolEst     Damping    Stepsize #Res #Jac #Sol   LinErr   LinRes
   1        0.74   0.3500000        0.74   10    7    7  7.2e-14  3.2e-15
   2        0.73   0.3500000        0.73   11    8    8  8.1e-14  3.1e-15
   3        0.71   0.3500000        0.71   12    9    9  1.1e-13  3.1e-15
Velocity u2, Pressure p
Iter      SolEst     Damping    Stepsize #Res #Jac #Sol   LinErr   LinRes
   1        0.15   0.5000000        0.15    6    3    3  2.7e-15  7.9e-15
Displacement field
Iter      SolEst     Damping    Stepsize #Res #Jac #Sol   LinErr   LinRes
   1        0.52   0.5000000        0.52    6    3    3  1.4e-13  1.5e-12
Spatial mesh displacement
Iter      SolEst     Damping    Stepsize #Res #Jac #Sol   LinErr   LinRes
   1     1.2e-06   0.5000000     1.2e-06    6    3    3  1.5e-15  1.8e-15
Solution error estimates for segregated groups
1.2, 0.077, 0.26, 5.9e-07
Residual error estimates for segregated groups
2.1e+02, 8.1e+03, 1.6e+04, 0.13
Segregated solver iteration 4.
```

```
Merged variables
Iter      SolEst     Damping    Stepsize #Res #Jac #Sol   LinErr   LinRes
   1         0.7   0.3500000         0.7   14   10   10  1.4e-13  2.9e-15
   2        0.67   0.3500000        0.67   15   11   11  1.8e-13  3.5e-15
   3        0.58   0.3500000        0.58   16   12   12  1.1e-13  4.4e-15
Velocity u2, Pressure p
Iter      SolEst     Damping    Stepsize #Res #Jac #Sol   LinErr   LinRes
   1       0.072   0.5000000       0.072    8    4    4    3e-15  7.9e-15
Displacement field
Iter      SolEst     Damping    Stepsize #Res #Jac #Sol   LinErr   LinRes
   1        0.29   0.5000000        0.29    8    4    4    1e-13  1.5e-12
Spatial mesh displacement
Iter      SolEst     Damping    Stepsize #Res #Jac #Sol   LinErr   LinRes
   1     9.8e-07   0.5000000     9.8e-07    8    4    4  1.4e-15  1.8e-15
Solution error estimates for segregated groups
1, 0.036, 0.15, 4.9e-07
Residual error estimates for segregated groups
82, 4.3e+03, 1.1e+04, 0.14
Segregated solver iteration 5.
Merged variables
Iter      SolEst     Damping    Stepsize #Res #Jac #Sol   LinErr   LinRes
   1         0.4   0.3500000         0.4   18   13   13  1.3e-13    4e-15
   2        0.27   0.3500000        0.27   19   14   14  7.4e-14  3.9e-15
   3        0.18   0.3500000        0.18   20   15   15  6.5e-14  3.7e-15
Velocity u2, Pressure p
Iter      SolEst     Damping    Stepsize #Res #Jac #Sol   LinErr   LinRes
   1       0.035   0.5000000       0.035   10    5    5    4e-15  7.9e-15
Displacement field
Iter      SolEst     Damping    Stepsize #Res #Jac #Sol   LinErr   LinRes
   1        0.17   0.5000000        0.17   10    5    5  6.5e-14  1.5e-12
Spatial mesh displacement
Iter      SolEst     Damping    Stepsize #Res #Jac #Sol   LinErr   LinRes
   1     7.3e-07   0.5000000     7.3e-07   10    5    5    2e-15  1.9e-15
Solution error estimates for segregated groups
0.3, 0.017, 0.084, 3.7e-07
Residual error estimates for segregated groups
32, 2.2e+03, 6.8e+03, 0.14
Segregated solver iteration 6.
Merged variables
Iter      SolEst     Damping    Stepsize #Res #Jac #Sol   LinErr   LinRes
   1        0.12   0.3500000        0.12   22   16   16    8e-14    4e-15
   2       0.076   0.3500000       0.076   23   17   17    9e-14  6.5e-15
   3        0.05   0.3500000        0.05   24   18   18  5.9e-14    4e-15
Velocity u2, Pressure p
Iter      SolEst     Damping    Stepsize #Res #Jac #Sol   LinErr   LinRes
   1       0.017   0.5000000       0.017   12    6    6  3.4e-15    8e-15
Displacement field
Scales for dependent variables:
Auxiliary pressure (comp1.solid.lemm1.pw): 0.36
Displacement field (comp1.u): 2.9e-16
Iter      SolEst     Damping    Stepsize #Res #Jac #Sol   LinErr   LinRes
   1       0.095   0.5000000       0.095   12    6    6  1.1e-13  1.2e-12
Spatial mesh displacement
Iter      SolEst     Damping    Stepsize #Res #Jac #Sol   LinErr   LinRes
   1     5.1e-07   0.5000000     5.1e-07   12    6    6  1.8e-15  1.8e-15
Solution error estimates for segregated groups
```

```
0.086, 0.0085, 0.048, 2.6e-07
Residual error estimates for segregated groups
12, 1.1e+03, 4.2e+03, 0.14
Segregated solver iteration 7.
Merged variables
Iter      SolEst     Damping    Stepsize #Res #Jac #Sol   LinErr   LinRes
   1       0.032   0.3500000       0.032   26   19   19  9.2e-14  4.5e-15
   2       0.021   0.3500000       0.021   27   20   20  1.7e-13  3.6e-15
   3       0.014   0.3500000       0.014   28   21   21  6.9e-14  5.9e-15
Velocity u2, Pressure p
Iter      SolEst     Damping    Stepsize #Res #Jac #Sol   LinErr   LinRes
   1      0.0085   0.5000000      0.0085   14    7    7  4.9e-15  7.9e-15
Displacement field
Iter      SolEst     Damping    Stepsize #Res #Jac #Sol   LinErr   LinRes
   1       0.054   0.5000000       0.054   14    7    7  1.1e-13  1.2e-12
Spatial mesh displacement
Iter      SolEst     Damping    Stepsize #Res #Jac #Sol   LinErr   LinRes
   1     3.4e-07   0.5000000     3.4e-07   14    7    7  1.7e-15  1.8e-15
Solution error estimates for segregated groups
0.024, 0.0042, 0.027, 1.7e-07
Residual error estimates for segregated groups
4.1, 5.6e+02, 2.6e+03, 0.14
Segregated solver iteration 8.
Merged variables
Iter      SolEst     Damping    Stepsize #Res #Jac #Sol   LinErr   LinRes
   1      0.0089   0.3500000      0.0089   30   22   22  9.7e-14  5.3e-15
   2      0.0058   0.3500000      0.0058   31   23   23    9e-14  3.6e-15
   3      0.0038   0.3500000      0.0038   32   24   24    1e-13  3.1e-15
Velocity u2, Pressure p
Iter      SolEst     Damping    Stepsize #Res #Jac #Sol   LinErr   LinRes
   1      0.0042   0.5000000      0.0042   16    8    8  2.2e-15  7.9e-15
Displacement field
Iter      SolEst     Damping    Stepsize #Res #Jac #Sol   LinErr   LinRes
   1        0.03   0.5000000        0.03   16    8    8  1.1e-13  1.2e-12
Spatial mesh displacement
Iter      SolEst     Damping    Stepsize #Res #Jac #Sol   LinErr   LinRes
   1     2.2e-07   0.5000000     2.2e-07   16    8    8  2.2e-15  1.8e-15
Solution error estimates for segregated groups
0.0065, 0.0021, 0.015, 1.1e-07
Residual error estimates for segregated groups
1.4, 2.8e+02, 1.8e+03, 0.14
Segregated solver iteration 9.
Merged variables
Iter      SolEst     Damping    Stepsize #Res #Jac #Sol   LinErr   LinRes
   1      0.0025   0.3500000      0.0025   34   25   25  8.4e-14  4.6e-15
   2      0.0016   0.3500000      0.0016   35   26   26  1.2e-13  4.9e-15
   3       0.001   0.3500000       0.001   36   27   27  7.4e-14  3.5e-15
Velocity u2, Pressure p
Iter      SolEst     Damping    Stepsize #Res #Jac #Sol   LinErr   LinRes
   1      0.0021   0.5000000      0.0021   18    9    9  2.4e-15  7.7e-15
Displacement field
Iter      SolEst     Damping    Stepsize #Res #Jac #Sol   LinErr   LinRes
   1       0.017   0.5000000       0.017   18    9    9  1.2e-13  1.2e-12
Spatial mesh displacement
Iter      SolEst     Damping    Stepsize #Res #Jac #Sol   LinErr   LinRes
   1     1.4e-07   0.5000000     1.4e-07   18    9    9  1.7e-15  1.8e-15
```

```
Solution error estimates for segregated groups
0.0018, 0.0011, 0.0085, 6.9e-08
Residual error estimates for segregated groups
0.44, 1.4e+02, 1.4e+03, 0.13
Segregated solver iteration 10.
Merged variables
Iter      SolEst     Damping    Stepsize #Res #Jac #Sol   LinErr   LinRes
   1     0.00068   0.3500000     0.00068   38   28   28  4.2e-14  3.2e-15
   2     0.00044   0.3500000     0.00044   39   29   29    8e-14  3.5e-15
   3     0.00029   0.3500000     0.00029   40   30   30  1.5e-13    4e-15
Velocity u2, Pressure p
Iter      SolEst     Damping    Stepsize #Res #Jac #Sol   LinErr   LinRes
   1      0.0011   0.5000000      0.0011   20   10   10  2.3e-15  7.9e-15
Displacement field
Iter      SolEst     Damping    Stepsize #Res #Jac #Sol   LinErr   LinRes
   1      0.0094   0.5000000      0.0094   20   10   10  1.5e-13  1.2e-12
Spatial mesh displacement
Iter      SolEst     Damping    Stepsize #Res #Jac #Sol   LinErr   LinRes
   1     8.4e-08   0.5000000     8.4e-08   20   10   10  1.8e-15  1.9e-15
Solution error estimates for segregated groups
0.00049, 0.00053, 0.0047, 4.2e-08
Residual error estimates for segregated groups
0.14, 70, 1.2e+03, 0.13
Segregated solver iteration 11.
Merged variables
Iter      SolEst     Damping    Stepsize #Res #Jac #Sol   LinErr   LinRes
   1     0.00019   0.3500000     0.00019   42   31   31  6.1e-14    5e-15
   2     0.00012   0.3500000     0.00012   43   32   32  7.9e-14  4.9e-15
   3     7.8e-05   0.3500000     7.8e-05   44   33   33  6.2e-14  5.2e-15
Velocity u2, Pressure p
Iter      SolEst     Damping    Stepsize #Res #Jac #Sol   LinErr   LinRes
   1     0.00053   0.5000000     0.00053   22   11   11  2.9e-15    8e-15
Displacement field
Iter      SolEst     Damping    Stepsize #Res #Jac #Sol   LinErr   LinRes
   1      0.0051   0.5000000      0.0051   22   11   11  1.2e-13  1.2e-12
Spatial mesh displacement
Iter      SolEst     Damping    Stepsize #Res #Jac #Sol   LinErr   LinRes
   1       5e-08   0.5000000       5e-08   22   11   11  1.6e-15  1.8e-15
Solution error estimates for segregated groups
0.00013, 0.00026, 0.0026, 2.5e-08
Residual error estimates for segregated groups
0.043, 35, 1.2e+03, 0.13
Segregated solver iteration 12.
Merged variables
Iter      SolEst     Damping    Stepsize #Res #Jac #Sol   LinErr   LinRes
   1     5.1e-05   0.3500000     5.1e-05   46   34   34  5.4e-14  4.2e-15
   2     3.3e-05   0.3500000     3.3e-05   47   35   35  5.9e-14    3e-15
   3     2.2e-05   0.3500000     2.2e-05   48   36   36  5.9e-14    3e-15
Velocity u2, Pressure p
Iter      SolEst     Damping    Stepsize #Res #Jac #Sol   LinErr   LinRes
   1     0.00026   0.5000000     0.00026   24   12   12  3.4e-15  7.9e-15
Displacement field
Iter      SolEst     Damping    Stepsize #Res #Jac #Sol   LinErr   LinRes
   1      0.0028   0.5000000      0.0028   24   12   12  1.2e-13  1.2e-12
Spatial mesh displacement
Iter      SolEst     Damping    Stepsize #Res #Jac #Sol   LinErr   LinRes
```

```
   1       3e-08   0.5000000       3e-08   24   12   12  1.6e-15  1.8e-15
Solution error estimates for segregated groups
3.7e-05, 0.00013, 0.0014, 1.5e-08
Residual error estimates for segregated groups
0.013, 18, 1.2e+03, 0.14
Segregated solver iteration 13.
Merged variables
Iter      SolEst     Damping    Stepsize #Res #Jac #Sol   LinErr   LinRes
   1     1.4e-05   0.3500000     1.4e-05   50   37   37  4.8e-14  5.5e-15
   2     9.1e-06   0.3500000     9.1e-06   51   38   38  9.2e-14  4.7e-15
   3     5.9e-06   0.3500000     5.9e-06   52   39   39  1.3e-13  3.8e-15
Velocity u2, Pressure p
Iter      SolEst     Damping    Stepsize #Res #Jac #Sol   LinErr   LinRes
   1     0.00013   0.5000000     0.00013   26   13   13  3.1e-15  7.8e-15
Displacement field
Iter      SolEst     Damping    Stepsize #Res #Jac #Sol   LinErr   LinRes
   1      0.0015   0.5000000      0.0015   26   13   13  1.1e-13  1.2e-12
Spatial mesh displacement
Iter      SolEst     Damping    Stepsize #Res #Jac #Sol   LinErr   LinRes
   1     1.7e-08   0.5000000     1.7e-08   26   13   13  1.3e-15  1.8e-15
Solution error estimates for segregated groups
1e-05, 6.6e-05, 0.00076, 8.7e-09
Residual error estimates for segregated groups
0.004, 8.8, 1.2e+03, 0.14
Solution time: 155 s. (2 minutes, 35 seconds)
Physical memory: 4.76 GB
Virtual memory: 26.05 GB
Ended at Jan 31, 2025, 8:50:28 PM.
----- Stationary Solver 1 in study1/Approach Curve Solver (sol1) -------------->
```

d=2065 (su89)

GENERAL

Description	Value
Solution	d=2065

LOG

```
<---- Compile Equations: Stationary in study1/Approach Curve Solver (sol1) -----
Started at Jan 31, 2025, 8:50:28 PM.
Parameter d = 2.065E-6 (m).
Geometry shape function: Linear serendipity
Number of vertex elements: 18
Number of boundary elements: 6099
Number of elements: 154055
Minimum element quality: 0.496
Time: 8 s.
Physical memory: 2.99 GB
Virtual memory: 15.25 GB
Ended at Jan 31, 2025, 8:50:36 PM.
----- Compile Equations: Stationary in study1/Approach Curve Solver (sol1) ---->

<---- Dependent Variables 1 in study1/Approach Curve Solver (sol1) -------------
Started at Jan 31, 2025, 8:50:36 PM.
Solution time: 0 s.
Physical memory: 2.99 GB
Virtual memory: 15.25 GB
Ended at Jan 31, 2025, 8:50:36 PM.
----- Dependent Variables 1 in study1/Approach Curve Solver (sol1) ------------>

<---- Stationary Solver 1 in study1/Approach Curve Solver (sol1) ---------------
Started at Jan 31, 2025, 8:50:37 PM.
Segregated solver
Segregated Step 11 does not have any components solved for.
Number of degrees of freedom solved for: 781757 (plus 11621 internal DOFs).
Segregated solver iteration 1.
Merged variables
Nonsymmetric matrix found.
Scales for dependent variables:
Concentration (comp1.Cl): 1.4e+02
Concentration (comp1.K): 1.4e+02
Electric potential (comp1.V): 1.9e+02
Orthonormal null-space function used.
Iter      SolEst     Damping    Stepsize #Res #Jac #Sol   LinErr   LinRes
   1        0.83   0.3500000        0.83    2    1    1  3.2e-11  2.7e-15
   2        0.81   0.3500000        0.81    3    2    2  5.1e-11  2.7e-15
   3         0.8   0.3500000         0.8    4    3    3  3.3e-11  2.7e-15
Velocity u2, Pressure p
Nonsymmetric matrix found.
Scales for dependent variables:
Pressure (comp1.p): 1
Velocity field (spatial frame) (comp1.u2): 0.00018
Orthonormal null-space function used.
Iter      SolEst     Damping    Stepsize #Res #Jac #Sol   LinErr   LinRes
   1         1.1   0.5000000         1.1    2    1    1    2e-15  2.3e-15
Displacement field
Nonsymmetric matrix found.
Scales for dependent variables:
Auxiliary pressure (comp1.solid.lemm1.pw): 1
Displacement field (comp1.u): 2.8e-18
Orthonormal null-space function used.
Iter      SolEst     Damping    Stepsize #Res #Jac #Sol   LinErr   LinRes
   1         1.9   0.5000000         1.9    2    1    1  7.1e-14  1.5e-12
Spatial mesh displacement
```

```
Symmetric matrices found.
Scales for dependent variables:
Spatial mesh displacement (comp1.spatial.disp): 1.2e-09
Orthonormal null-space function used.
Iter      SolEst     Damping    Stepsize #Res #Jac #Sol   LinErr   LinRes
   1     7.8e-07   0.5000000     7.8e-07    2    1    1  1.4e-15  1.8e-15
Solution error estimates for segregated groups
1.4, 0.54, 0.95, 3.9e-07
Residual error estimates for segregated groups
7.8e+03, 8.2e+04, 2.1e+04, 0.11
Segregated solver iteration 2.
Merged variables
Scales for dependent variables:
Concentration (comp1.Cl): 1.4e+02
Concentration (comp1.K): 1.4e+02
Electric potential (comp1.V): 0.064
Iter      SolEst     Damping    Stepsize #Res #Jac #Sol   LinErr   LinRes
   1        0.78   0.3500000        0.78    6    4    4  8.8e-14  3.6e-15
   2        0.77   0.3500000        0.77    7    5    5  9.8e-14  5.4e-15
   3        0.76   0.3500000        0.76    8    6    6  6.5e-14  5.7e-15
Velocity u2, Pressure p
Scales for dependent variables:
Pressure (comp1.p): 3.6e+02
Velocity field (spatial frame) (comp1.u2): 0.00073
Iter      SolEst     Damping    Stepsize #Res #Jac #Sol   LinErr   LinRes
   1        0.36   0.5000000        0.36    4    2    2    3e-15  7.9e-15
Displacement field
Iter      SolEst     Damping    Stepsize #Res #Jac #Sol   LinErr   LinRes
   1        0.95   0.5000000        0.95    4    2    2    9e-14  1.5e-12
Spatial mesh displacement
Iter      SolEst     Damping    Stepsize #Res #Jac #Sol   LinErr   LinRes
   1     1.2e-06   0.5000000     1.2e-06    4    2    2  1.4e-15  1.8e-15
Solution error estimates for segregated groups
1.3, 0.18, 0.48, 5.9e-07
Residual error estimates for segregated groups
7.6e+02, 1.4e+04, 2.1e+04, 0.13
Segregated solver iteration 3.
Merged variables
Iter      SolEst     Damping    Stepsize #Res #Jac #Sol   LinErr   LinRes
   1        0.74   0.3500000        0.74   10    7    7  1.6e-13  4.1e-15
   2        0.73   0.3500000        0.73   11    8    8  1.2e-13  3.8e-15
   3        0.71   0.3500000        0.71   12    9    9    1e-13  3.6e-15
Velocity u2, Pressure p
Iter      SolEst     Damping    Stepsize #Res #Jac #Sol   LinErr   LinRes
   1        0.15   0.5000000        0.15    6    3    3  3.3e-15  7.9e-15
Displacement field
Iter      SolEst     Damping    Stepsize #Res #Jac #Sol   LinErr   LinRes
   1        0.52   0.5000000        0.52    6    3    3  9.1e-14  1.5e-12
Spatial mesh displacement
Iter      SolEst     Damping    Stepsize #Res #Jac #Sol   LinErr   LinRes
   1     1.2e-06   0.5000000     1.2e-06    6    3    3  1.9e-15  1.8e-15
Solution error estimates for segregated groups
1.2, 0.077, 0.26, 5.9e-07
Residual error estimates for segregated groups
2.1e+02, 7.7e+03, 1.6e+04, 0.14
Segregated solver iteration 4.
```

```
Merged variables
Iter      SolEst     Damping    Stepsize #Res #Jac #Sol   LinErr   LinRes
   1         0.7   0.3500000         0.7   14   10   10  1.4e-13  3.2e-15
   2        0.67   0.3500000        0.67   15   11   11  8.3e-14  2.9e-15
   3        0.58   0.3500000        0.58   16   12   12  8.2e-14  3.7e-15
Velocity u2, Pressure p
Iter      SolEst     Damping    Stepsize #Res #Jac #Sol   LinErr   LinRes
   1       0.072   0.5000000       0.072    8    4    4  3.9e-15  7.8e-15
Displacement field
Iter      SolEst     Damping    Stepsize #Res #Jac #Sol   LinErr   LinRes
   1        0.29   0.5000000        0.29    8    4    4  6.1e-14  1.5e-12
Spatial mesh displacement
Iter      SolEst     Damping    Stepsize #Res #Jac #Sol   LinErr   LinRes
   1     9.8e-07   0.5000000     9.8e-07    8    4    4  1.9e-15  1.9e-15
Solution error estimates for segregated groups
1, 0.036, 0.15, 4.9e-07
Residual error estimates for segregated groups
82, 4.1e+03, 1.1e+04, 0.14
Segregated solver iteration 5.
Merged variables
Iter      SolEst     Damping    Stepsize #Res #Jac #Sol   LinErr   LinRes
   1         0.4   0.3500000         0.4   18   13   13  9.7e-14  3.1e-15
   2        0.27   0.3500000        0.27   19   14   14  1.2e-13  4.2e-15
   3        0.18   0.3500000        0.18   20   15   15  1.4e-13    3e-15
Velocity u2, Pressure p
Iter      SolEst     Damping    Stepsize #Res #Jac #Sol   LinErr   LinRes
   1       0.035   0.5000000       0.035   10    5    5  3.5e-15  7.9e-15
Displacement field
Iter      SolEst     Damping    Stepsize #Res #Jac #Sol   LinErr   LinRes
   1        0.17   0.5000000        0.17   10    5    5  1.5e-13  1.5e-12
Spatial mesh displacement
Iter      SolEst     Damping    Stepsize #Res #Jac #Sol   LinErr   LinRes
   1     7.3e-07   0.5000000     7.3e-07   10    5    5  1.7e-15  1.8e-15
Solution error estimates for segregated groups
0.31, 0.017, 0.084, 3.7e-07
Residual error estimates for segregated groups
32, 2.1e+03, 6.8e+03, 0.13
Segregated solver iteration 6.
Merged variables
Iter      SolEst     Damping    Stepsize #Res #Jac #Sol   LinErr   LinRes
   1        0.12   0.3500000        0.12   22   16   16  1.3e-13  3.3e-15
   2       0.076   0.3500000       0.076   23   17   17  8.7e-14  3.3e-15
   3        0.05   0.3500000        0.05   24   18   18  1.1e-13  3.2e-15
Velocity u2, Pressure p
Iter      SolEst     Damping    Stepsize #Res #Jac #Sol   LinErr   LinRes
   1       0.017   0.5000000       0.017   12    6    6  2.7e-15  7.9e-15
Displacement field
Scales for dependent variables:
Auxiliary pressure (comp1.solid.lemm1.pw): 0.36
Displacement field (comp1.u): 2.9e-16
Iter      SolEst     Damping    Stepsize #Res #Jac #Sol   LinErr   LinRes
   1       0.095   0.5000000       0.095   12    6    6  9.8e-14  1.2e-12
Spatial mesh displacement
Iter      SolEst     Damping    Stepsize #Res #Jac #Sol   LinErr   LinRes
   1     5.1e-07   0.5000000     5.1e-07   12    6    6  1.8e-15  1.9e-15
Solution error estimates for segregated groups
```

```
0.086, 0.0085, 0.048, 2.6e-07
Residual error estimates for segregated groups
12, 1.1e+03, 4.2e+03, 0.14
Segregated solver iteration 7.
Merged variables
Iter      SolEst     Damping    Stepsize #Res #Jac #Sol   LinErr   LinRes
   1       0.032   0.3500000       0.032   26   19   19    5e-14  2.9e-15
   2       0.021   0.3500000       0.021   27   20   20  7.9e-14  3.6e-15
   3       0.014   0.3500000       0.014   28   21   21  8.6e-14    3e-15
Velocity u2, Pressure p
Iter      SolEst     Damping    Stepsize #Res #Jac #Sol   LinErr   LinRes
   1      0.0085   0.5000000      0.0085   14    7    7  3.1e-15  7.8e-15
Displacement field
Iter      SolEst     Damping    Stepsize #Res #Jac #Sol   LinErr   LinRes
   1       0.054   0.5000000       0.054   14    7    7  1.1e-13  1.2e-12
Spatial mesh displacement
Iter      SolEst     Damping    Stepsize #Res #Jac #Sol   LinErr   LinRes
   1     3.4e-07   0.5000000     3.4e-07   14    7    7  1.4e-15  1.9e-15
Solution error estimates for segregated groups
0.024, 0.0042, 0.027, 1.7e-07
Residual error estimates for segregated groups
4.1, 5.4e+02, 2.6e+03, 0.14
Segregated solver iteration 8.
Merged variables
Iter      SolEst     Damping    Stepsize #Res #Jac #Sol   LinErr   LinRes
   1       0.009   0.3500000       0.009   30   22   22  1.3e-13  3.2e-15
   2      0.0058   0.3500000      0.0058   31   23   23  7.4e-14  4.3e-15
   3      0.0038   0.3500000      0.0038   32   24   24  9.9e-14  3.5e-15
Velocity u2, Pressure p
Iter      SolEst     Damping    Stepsize #Res #Jac #Sol   LinErr   LinRes
   1      0.0042   0.5000000      0.0042   16    8    8  3.8e-15  7.9e-15
Displacement field
Iter      SolEst     Damping    Stepsize #Res #Jac #Sol   LinErr   LinRes
   1        0.03   0.5000000        0.03   16    8    8    1e-13  1.2e-12
Spatial mesh displacement
Iter      SolEst     Damping    Stepsize #Res #Jac #Sol   LinErr   LinRes
   1     2.2e-07   0.5000000     2.2e-07   16    8    8  1.7e-15  1.8e-15
Solution error estimates for segregated groups
0.0065, 0.0021, 0.015, 1.1e-07
Residual error estimates for segregated groups
1.4, 2.7e+02, 1.8e+03, 0.12
Segregated solver iteration 9.
Merged variables
Iter      SolEst     Damping    Stepsize #Res #Jac #Sol   LinErr   LinRes
   1      0.0025   0.3500000      0.0025   34   25   25  1.6e-13  4.5e-15
   2      0.0016   0.3500000      0.0016   35   26   26  4.8e-14  3.7e-15
   3       0.001   0.3500000       0.001   36   27   27  1.1e-13  3.4e-15
Velocity u2, Pressure p
Iter      SolEst     Damping    Stepsize #Res #Jac #Sol   LinErr   LinRes
   1      0.0021   0.5000000      0.0021   18    9    9  3.5e-15  7.9e-15
Displacement field
Iter      SolEst     Damping    Stepsize #Res #Jac #Sol   LinErr   LinRes
   1       0.017   0.5000000       0.017   18    9    9  2.9e-13  1.2e-12
Spatial mesh displacement
Iter      SolEst     Damping    Stepsize #Res #Jac #Sol   LinErr   LinRes
   1     1.4e-07   0.5000000     1.4e-07   18    9    9  1.6e-15  1.8e-15
```

```
Solution error estimates for segregated groups
0.0018, 0.0011, 0.0085, 6.9e-08
Residual error estimates for segregated groups
0.44, 1.3e+02, 1.4e+03, 0.13
Segregated solver iteration 10.
Merged variables
Iter      SolEst     Damping    Stepsize #Res #Jac #Sol   LinErr   LinRes
   1     0.00068   0.3500000     0.00068   38   28   28  7.7e-14  2.9e-15
   2     0.00044   0.3500000     0.00044   39   29   29  1.3e-13  3.8e-15
   3     0.00029   0.3500000     0.00029   40   30   30  1.5e-13  3.3e-15
Velocity u2, Pressure p
Iter      SolEst     Damping    Stepsize #Res #Jac #Sol   LinErr   LinRes
   1      0.0011   0.5000000      0.0011   20   10   10  2.8e-15  7.9e-15
Displacement field
Iter      SolEst     Damping    Stepsize #Res #Jac #Sol   LinErr   LinRes
   1      0.0094   0.5000000      0.0094   20   10   10  1.3e-13  1.2e-12
Spatial mesh displacement
Iter      SolEst     Damping    Stepsize #Res #Jac #Sol   LinErr   LinRes
   1     8.4e-08   0.5000000     8.4e-08   20   10   10  1.6e-15  1.8e-15
Solution error estimates for segregated groups
0.00049, 0.00053, 0.0047, 4.2e-08
Residual error estimates for segregated groups
0.14, 67, 1.2e+03, 0.13
Segregated solver iteration 11.
Merged variables
Iter      SolEst     Damping    Stepsize #Res #Jac #Sol   LinErr   LinRes
   1     0.00019   0.3500000     0.00019   42   31   31  1.4e-13  2.9e-15
   2     0.00012   0.3500000     0.00012   43   32   32  7.1e-14  2.9e-15
   3     7.8e-05   0.3500000     7.8e-05   44   33   33  5.3e-14  2.9e-15
Velocity u2, Pressure p
Iter      SolEst     Damping    Stepsize #Res #Jac #Sol   LinErr   LinRes
   1     0.00053   0.5000000     0.00053   22   11   11  3.6e-15  7.8e-15
Displacement field
Iter      SolEst     Damping    Stepsize #Res #Jac #Sol   LinErr   LinRes
   1      0.0051   0.5000000      0.0051   22   11   11  1.2e-13  1.2e-12
Spatial mesh displacement
Iter      SolEst     Damping    Stepsize #Res #Jac #Sol   LinErr   LinRes
   1     5.1e-08   0.5000000     5.1e-08   22   11   11  1.6e-15  1.8e-15
Solution error estimates for segregated groups
0.00013, 0.00026, 0.0026, 2.5e-08
Residual error estimates for segregated groups
0.043, 34, 1.2e+03, 0.14
Segregated solver iteration 12.
Merged variables
Iter      SolEst     Damping    Stepsize #Res #Jac #Sol   LinErr   LinRes
   1     5.1e-05   0.3500000     5.1e-05   46   34   34  8.8e-14  3.2e-15
   2     3.3e-05   0.3500000     3.3e-05   47   35   35  1.2e-13  5.3e-15
   3     2.2e-05   0.3500000     2.2e-05   48   36   36  5.2e-14    3e-15
Velocity u2, Pressure p
Iter      SolEst     Damping    Stepsize #Res #Jac #Sol   LinErr   LinRes
   1     0.00026   0.5000000     0.00026   24   12   12    2e-15  7.8e-15
Displacement field
Iter      SolEst     Damping    Stepsize #Res #Jac #Sol   LinErr   LinRes
   1      0.0028   0.5000000      0.0028   24   12   12  1.4e-13  1.2e-12
Spatial mesh displacement
Iter      SolEst     Damping    Stepsize #Res #Jac #Sol   LinErr   LinRes
```

```
   1       3e-08   0.5000000       3e-08   24    12    12  1.5e-15  1.8e-15
Solution error estimates for segregated groups
3.7e-05, 0.00013, 0.0014, 1.5e-08
Residual error estimates for segregated groups
0.013, 17, 1.2e+03, 0.14
Segregated solver iteration 13.
Merged variables
Iter      SolEst      Damping    Stepsize #Res #Jac #Sol   LinErr   LinRes
   1     1.4e-05   0.3500000     1.4e-05   50    37    37  7.9e-14  3.3e-15
   2     9.1e-06   0.3500000     9.1e-06   51    38    38  1.4e-13  3.1e-15
   3     5.9e-06   0.3500000     5.9e-06   52    39    39  5.2e-14  3.8e-15
Velocity u2, Pressure p
Iter      SolEst      Damping    Stepsize #Res #Jac #Sol   LinErr   LinRes
   1     0.00013   0.5000000     0.00013   26    13    13    2e-15  7.9e-15
Displacement field
Iter      SolEst      Damping    Stepsize #Res #Jac #Sol   LinErr   LinRes
   1      0.0015   0.5000000      0.0015   26    13    13  1.3e-13  1.2e-12
Spatial mesh displacement
Iter      SolEst      Damping    Stepsize #Res #Jac #Sol   LinErr   LinRes
   1     1.7e-08   0.5000000     1.7e-08   26    13    13  1.7e-15  1.8e-15
Solution error estimates for segregated groups
1e-05, 6.6e-05, 0.00076, 8.7e-09
Residual error estimates for segregated groups
0.004, 8.4, 1.2e+03, 0.13
Solution time: 158 s. (2 minutes, 38 seconds)
Physical memory: 4.81 GB
Virtual memory: 25.64 GB
Ended at Jan 31, 2025, 8:53:14 PM.
----- Stationary Solver 1 in study1/Approach Curve Solver (sol1) -------------->
```

d=2060 (su90)

GENERAL

Description	Value
Solution	d=2060

LOG

```
<---- Compile Equations: Stationary in study1/Approach Curve Solver (sol1) -----
Started at Jan 31, 2025, 8:53:15 PM.
Parameter d = 2.06E-6 (m).
Geometry shape function: Linear serendipity
Number of vertex elements: 18
Number of boundary elements: 6096
Number of elements: 153896
Minimum element quality: 0.4733
Time: 8 s.
Physical memory: 3.01 GB
Virtual memory: 15.27 GB
Ended at Jan 31, 2025, 8:53:23 PM.
----- Compile Equations: Stationary in study1/Approach Curve Solver (sol1) ---->

<---- Dependent Variables 1 in study1/Approach Curve Solver (sol1) -------------
Started at Jan 31, 2025, 8:53:23 PM.
Solution time: 0 s.
Physical memory: 3.01 GB
Virtual memory: 15.27 GB
Ended at Jan 31, 2025, 8:53:23 PM.
----- Dependent Variables 1 in study1/Approach Curve Solver (sol1) ------------>

<---- Stationary Solver 1 in study1/Approach Curve Solver (sol1) ---------------
Started at Jan 31, 2025, 8:53:23 PM.
Segregated solver
Segregated Step 11 does not have any components solved for.
Number of degrees of freedom solved for: 780805 (plus 11615 internal DOFs).
Segregated solver iteration 1.
Merged variables
Nonsymmetric matrix found.
Scales for dependent variables:
Concentration (comp1.Cl): 1.4e+02
Concentration (comp1.K): 1.4e+02
Electric potential (comp1.V): 1.9e+02
Orthonormal null-space function used.
Iter      SolEst     Damping    Stepsize #Res #Jac #Sol   LinErr   LinRes
   1        0.83   0.3500000        0.83    2    1    1  3.5e-11  2.7e-15
   2        0.81   0.3500000        0.81    3    2    2  2.5e-11  2.7e-15
   3         0.8   0.3500000         0.8    4    3    3  3.3e-11  2.8e-15
Velocity u2, Pressure p
Nonsymmetric matrix found.
Scales for dependent variables:
Pressure (comp1.p): 1
Velocity field (spatial frame) (comp1.u2): 0.00018
Orthonormal null-space function used.
Iter      SolEst     Damping    Stepsize #Res #Jac #Sol   LinErr   LinRes
   1         1.1   0.5000000         1.1    2    1    1  1.6e-15  2.3e-15
Displacement field
Nonsymmetric matrix found.
Scales for dependent variables:
Auxiliary pressure (comp1.solid.lemm1.pw): 1
Displacement field (comp1.u): 2.8e-18
Orthonormal null-space function used.
Iter      SolEst     Damping    Stepsize #Res #Jac #Sol   LinErr   LinRes
   1         1.9   0.5000000         1.9    2    1    1  5.1e-14  1.4e-12
Spatial mesh displacement
```

```
Symmetric matrices found.
Scales for dependent variables:
Spatial mesh displacement (comp1.spatial.disp): 1.2e-09
Orthonormal null-space function used.
Iter      SolEst     Damping    Stepsize #Res #Jac #Sol   LinErr   LinRes
   1     7.9e-07   0.5000000     7.9e-07    2    1    1  1.5e-15  1.8e-15
Solution error estimates for segregated groups
1.4, 0.54, 0.95, 3.9e-07
Residual error estimates for segregated groups
7.8e+03, 8.2e+04, 2.1e+04, 0.12
Segregated solver iteration 2.
Merged variables
Scales for dependent variables:
Concentration (comp1.Cl): 1.4e+02
Concentration (comp1.K): 1.4e+02
Electric potential (comp1.V): 0.064
Iter      SolEst     Damping    Stepsize #Res #Jac #Sol   LinErr   LinRes
   1        0.78   0.3500000        0.78    6    4    4  4.4e-14  2.9e-15
   2        0.77   0.3500000        0.77    7    5    5  7.8e-14    4e-15
   3        0.76   0.3500000        0.76    8    6    6  1.1e-13  5.9e-15
Velocity u2, Pressure p
Scales for dependent variables:
Pressure (comp1.p): 3.7e+02
Velocity field (spatial frame) (comp1.u2): 0.00073
Iter      SolEst     Damping    Stepsize #Res #Jac #Sol   LinErr   LinRes
   1        0.36   0.5000000        0.36    4    2    2  2.7e-15  7.8e-15
Displacement field
Iter      SolEst     Damping    Stepsize #Res #Jac #Sol   LinErr   LinRes
   1        0.95   0.5000000        0.95    4    2    2  1.1e-13  1.5e-12
Spatial mesh displacement
Iter      SolEst     Damping    Stepsize #Res #Jac #Sol   LinErr   LinRes
   1     1.2e-06   0.5000000     1.2e-06    4    2    2  1.7e-15  1.8e-15
Solution error estimates for segregated groups
1.3, 0.18, 0.48, 5.9e-07
Residual error estimates for segregated groups
7.6e+02, 1.6e+04, 2.1e+04, 0.12
Segregated solver iteration 3.
Merged variables
Iter      SolEst     Damping    Stepsize #Res #Jac #Sol   LinErr   LinRes
   1        0.74   0.3500000        0.74   10    7    7  6.2e-14  3.6e-15
   2        0.73   0.3500000        0.73   11    8    8  5.7e-14    4e-15
   3        0.71   0.3500000        0.71   12    9    9  3.5e-14  3.3e-15
Velocity u2, Pressure p
Iter      SolEst     Damping    Stepsize #Res #Jac #Sol   LinErr   LinRes
   1        0.15   0.5000000        0.15    6    3    3    3e-15  7.8e-15
Displacement field
Iter      SolEst     Damping    Stepsize #Res #Jac #Sol   LinErr   LinRes
   1        0.52   0.5000000        0.52    6    3    3  4.2e-14  1.5e-12
Spatial mesh displacement
Iter      SolEst     Damping    Stepsize #Res #Jac #Sol   LinErr   LinRes
   1     1.2e-06   0.5000000     1.2e-06    6    3    3  1.5e-15  1.8e-15
Solution error estimates for segregated groups
1.2, 0.077, 0.26, 5.9e-07
Residual error estimates for segregated groups
2.1e+02, 8.9e+03, 1.6e+04, 0.13
Segregated solver iteration 4.
```

```
Merged variables
Iter      SolEst     Damping    Stepsize #Res #Jac #Sol   LinErr   LinRes
   1         0.7   0.3500000         0.7   14   10   10  7.9e-14  5.7e-15
   2        0.67   0.3500000        0.67   15   11   11  8.5e-14  3.3e-15
   3        0.58   0.3500000        0.58   16   12   12    1e-13  5.7e-15
Velocity u2, Pressure p
Iter      SolEst     Damping    Stepsize #Res #Jac #Sol   LinErr   LinRes
   1       0.072   0.5000000       0.072    8    4    4  2.2e-15  7.8e-15
Displacement field
Iter      SolEst     Damping    Stepsize #Res #Jac #Sol   LinErr   LinRes
   1        0.29   0.5000000        0.29    8    4    4  1.2e-13  1.5e-12
Spatial mesh displacement
Iter      SolEst     Damping    Stepsize #Res #Jac #Sol   LinErr   LinRes
   1     9.8e-07   0.5000000     9.8e-07    8    4    4  1.3e-15  1.8e-15
Solution error estimates for segregated groups
1, 0.036, 0.15, 4.9e-07
Residual error estimates for segregated groups
82, 4.7e+03, 1.1e+04, 0.14
Segregated solver iteration 5.
Merged variables
Iter      SolEst     Damping    Stepsize #Res #Jac #Sol   LinErr   LinRes
   1         0.4   0.3500000         0.4   18   13   13  6.7e-14  5.8e-15
   2        0.27   0.3500000        0.27   19   14   14  7.8e-14  3.6e-15
   3        0.18   0.3500000        0.18   20   15   15    9e-14  2.7e-15
Velocity u2, Pressure p
Iter      SolEst     Damping    Stepsize #Res #Jac #Sol   LinErr   LinRes
   1       0.035   0.5000000       0.035   10    5    5    3e-15  7.7e-15
Displacement field
Iter      SolEst     Damping    Stepsize #Res #Jac #Sol   LinErr   LinRes
   1        0.17   0.5000000        0.17   10    5    5  5.6e-14  1.4e-12
Spatial mesh displacement
Iter      SolEst     Damping    Stepsize #Res #Jac #Sol   LinErr   LinRes
   1     7.4e-07   0.5000000     7.4e-07   10    5    5  1.4e-15  1.8e-15
Solution error estimates for segregated groups
0.3, 0.017, 0.084, 3.7e-07
Residual error estimates for segregated groups
32, 2.4e+03, 6.8e+03, 0.14
Segregated solver iteration 6.
Merged variables
Iter      SolEst     Damping    Stepsize #Res #Jac #Sol   LinErr   LinRes
   1        0.12   0.3500000        0.12   22   16   16  8.1e-14  3.7e-15
   2       0.076   0.3500000       0.076   23   17   17  9.4e-14  2.8e-15
   3        0.05   0.3500000        0.05   24   18   18  3.2e-14  3.7e-15
Velocity u2, Pressure p
Iter      SolEst     Damping    Stepsize #Res #Jac #Sol   LinErr   LinRes
   1       0.017   0.5000000       0.017   12    6    6  2.6e-15    8e-15
Displacement field
Scales for dependent variables:
Auxiliary pressure (comp1.solid.lemm1.pw): 0.36
Displacement field (comp1.u): 2.9e-16
Iter      SolEst     Damping    Stepsize #Res #Jac #Sol   LinErr   LinRes
   1       0.095   0.5000000       0.095   12    6    6  1.1e-13  1.2e-12
Spatial mesh displacement
Iter      SolEst     Damping    Stepsize #Res #Jac #Sol   LinErr   LinRes
   1     5.2e-07   0.5000000     5.2e-07   12    6    6  1.4e-15  1.8e-15
Solution error estimates for segregated groups
```

```
0.085, 0.0086, 0.048, 2.6e-07
Residual error estimates for segregated groups
12, 1.2e+03, 4.2e+03, 0.14
Segregated solver iteration 7.
Merged variables
Iter      SolEst     Damping    Stepsize #Res #Jac #Sol   LinErr   LinRes
   1       0.032   0.3500000       0.032   26   19   19  9.3e-14  4.2e-15
   2       0.021   0.3500000       0.021   27   20   20    1e-13  3.4e-15
   3       0.014   0.3500000       0.014   28   21   21  1.4e-13  3.1e-15
Velocity u2, Pressure p
Iter      SolEst     Damping    Stepsize #Res #Jac #Sol   LinErr   LinRes
   1      0.0085   0.5000000      0.0085   14    7    7    2e-15  7.9e-15
Displacement field
Iter      SolEst     Damping    Stepsize #Res #Jac #Sol   LinErr   LinRes
   1       0.054   0.5000000       0.054   14    7    7  1.1e-13  1.2e-12
Spatial mesh displacement
Iter      SolEst     Damping    Stepsize #Res #Jac #Sol   LinErr   LinRes
   1     3.4e-07   0.5000000     3.4e-07   14    7    7  1.7e-15  1.8e-15
Solution error estimates for segregated groups
0.024, 0.0042, 0.027, 1.7e-07
Residual error estimates for segregated groups
4.1, 6.2e+02, 2.6e+03, 0.13
Segregated solver iteration 8.
Merged variables
Iter      SolEst     Damping    Stepsize #Res #Jac #Sol   LinErr   LinRes
   1      0.0089   0.3500000      0.0089   30   22   22  7.7e-14  4.1e-15
   2      0.0058   0.3500000      0.0058   31   23   23  1.5e-13  3.4e-15
   3      0.0038   0.3500000      0.0038   32   24   24  6.3e-14  3.7e-15
Velocity u2, Pressure p
Iter      SolEst     Damping    Stepsize #Res #Jac #Sol   LinErr   LinRes
   1      0.0042   0.5000000      0.0042   16    8    8  2.2e-15  7.8e-15
Displacement field
Iter      SolEst     Damping    Stepsize #Res #Jac #Sol   LinErr   LinRes
   1        0.03   0.5000000        0.03   16    8    8  1.5e-13  1.2e-12
Spatial mesh displacement
Iter      SolEst     Damping    Stepsize #Res #Jac #Sol   LinErr   LinRes
   1     2.2e-07   0.5000000     2.2e-07   16    8    8    2e-15  1.8e-15
Solution error estimates for segregated groups
0.0065, 0.0021, 0.015, 1.1e-07
Residual error estimates for segregated groups
1.3, 3.1e+02, 1.8e+03, 0.13
Segregated solver iteration 9.
Merged variables
Iter      SolEst     Damping    Stepsize #Res #Jac #Sol   LinErr   LinRes
   1      0.0025   0.3500000      0.0025   34   25   25    5e-14  3.6e-15
   2      0.0016   0.3500000      0.0016   35   26   26  5.5e-14  5.2e-15
   3       0.001   0.3500000       0.001   36   27   27  5.7e-14  2.9e-15
Velocity u2, Pressure p
Iter      SolEst     Damping    Stepsize #Res #Jac #Sol   LinErr   LinRes
   1      0.0021   0.5000000      0.0021   18    9    9  3.5e-15  7.9e-15
Displacement field
Iter      SolEst     Damping    Stepsize #Res #Jac #Sol   LinErr   LinRes
   1       0.017   0.5000000       0.017   18    9    9  1.3e-13  1.2e-12
Spatial mesh displacement
Iter      SolEst     Damping    Stepsize #Res #Jac #Sol   LinErr   LinRes
   1     1.4e-07   0.5000000     1.4e-07   18    9    9  1.8e-15  1.8e-15
```

```
Solution error estimates for segregated groups
0.0018, 0.0011, 0.0085, 6.9e-08
Residual error estimates for segregated groups
0.44, 1.6e+02, 1.4e+03, 0.14
Segregated solver iteration 10.
Merged variables
Iter      SolEst     Damping    Stepsize #Res #Jac #Sol   LinErr   LinRes
   1     0.00067   0.3500000     0.00067   38   28   28  8.2e-14  3.1e-15
   2     0.00044   0.3500000     0.00044   39   29   29  1.1e-13    3e-15
   3     0.00028   0.3500000     0.00028   40   30   30  8.7e-14    5e-15
Velocity u2, Pressure p
Iter      SolEst     Damping    Stepsize #Res #Jac #Sol   LinErr   LinRes
   1      0.0011   0.5000000      0.0011   20   10   10  2.6e-15    8e-15
Displacement field
Iter      SolEst     Damping    Stepsize #Res #Jac #Sol   LinErr   LinRes
   1      0.0094   0.5000000      0.0094   20   10   10  1.7e-13  1.2e-12
Spatial mesh displacement
Iter      SolEst     Damping    Stepsize #Res #Jac #Sol   LinErr   LinRes
   1     8.5e-08   0.5000000     8.5e-08   20   10   10  1.7e-15  1.9e-15
Solution error estimates for segregated groups
0.00049, 0.00053, 0.0047, 4.2e-08
Residual error estimates for segregated groups
0.14, 78, 1.3e+03, 0.13
Segregated solver iteration 11.
Merged variables
Iter      SolEst     Damping    Stepsize #Res #Jac #Sol   LinErr   LinRes
   1     0.00019   0.3500000     0.00019   42   31   31  6.9e-14  3.1e-15
   2     0.00012   0.3500000     0.00012   43   32   32    9e-14  3.6e-15
   3     7.8e-05   0.3500000     7.8e-05   44   33   33    1e-13  3.1e-15
Velocity u2, Pressure p
Iter      SolEst     Damping    Stepsize #Res #Jac #Sol   LinErr   LinRes
   1     0.00053   0.5000000     0.00053   22   11   11  1.8e-15  7.8e-15
Displacement field
Iter      SolEst     Damping    Stepsize #Res #Jac #Sol   LinErr   LinRes
   1      0.0051   0.5000000      0.0051   22   11   11  1.2e-13  1.2e-12
Spatial mesh displacement
Iter      SolEst     Damping    Stepsize #Res #Jac #Sol   LinErr   LinRes
   1     5.1e-08   0.5000000     5.1e-08   22   11   11  1.6e-15  1.8e-15
Solution error estimates for segregated groups
0.00013, 0.00026, 0.0026, 2.5e-08
Residual error estimates for segregated groups
0.043, 39, 1.2e+03, 0.13
Segregated solver iteration 12.
Merged variables
Iter      SolEst     Damping    Stepsize #Res #Jac #Sol   LinErr   LinRes
   1     5.1e-05   0.3500000     5.1e-05   46   34   34  4.2e-14  4.1e-15
   2     3.3e-05   0.3500000     3.3e-05   47   35   35  5.9e-14  3.8e-15
   3     2.1e-05   0.3500000     2.1e-05   48   36   36  5.9e-14  4.2e-15
Velocity u2, Pressure p
Iter      SolEst     Damping    Stepsize #Res #Jac #Sol   LinErr   LinRes
   1     0.00026   0.5000000     0.00026   24   12   12  3.3e-15  7.8e-15
Displacement field
Iter      SolEst     Damping    Stepsize #Res #Jac #Sol   LinErr   LinRes
   1      0.0028   0.5000000      0.0028   24   12   12  1.6e-13  1.2e-12
Spatial mesh displacement
Iter      SolEst     Damping    Stepsize #Res #Jac #Sol   LinErr   LinRes
```

```
   1       3e-08   0.5000000       3e-08   24   12   12  1.5e-15  1.8e-15
Solution error estimates for segregated groups
3.7e-05, 0.00013, 0.0014, 1.5e-08
Residual error estimates for segregated groups
0.013, 19, 1.2e+03, 0.13
Segregated solver iteration 13.
Merged variables
Iter      SolEst     Damping    Stepsize #Res #Jac #Sol   LinErr   LinRes
   1     1.4e-05   0.3500000     1.4e-05   50   37   37  1.6e-13    3e-15
   2     9.1e-06   0.3500000     9.1e-06   51   38   38  7.4e-14  3.4e-15
   3     5.9e-06   0.3500000     5.9e-06   52   39   39  7.4e-14  3.9e-15
Velocity u2, Pressure p
Iter      SolEst     Damping    Stepsize #Res #Jac #Sol   LinErr   LinRes
   1     0.00013   0.5000000     0.00013   26   13   13  4.8e-15    8e-15
Displacement field
Iter      SolEst     Damping    Stepsize #Res #Jac #Sol   LinErr   LinRes
   1      0.0015   0.5000000      0.0015   26   13   13  1.3e-13  1.2e-12
Spatial mesh displacement
Iter      SolEst     Damping    Stepsize #Res #Jac #Sol   LinErr   LinRes
   1     1.7e-08   0.5000000     1.7e-08   26   13   13  1.4e-15  1.8e-15
Solution error estimates for segregated groups
1e-05, 6.6e-05, 0.00076, 8.7e-09
Residual error estimates for segregated groups
0.004, 9.7, 1.2e+03, 0.13
Solution time: 152 s. (2 minutes, 32 seconds)
Physical memory: 4.75 GB
Virtual memory: 25.91 GB
Ended at Jan 31, 2025, 8:55:54 PM.
----- Stationary Solver 1 in study1/Approach Curve Solver (sol1) -------------->
```

d=2055 (su91)

GENERAL

Description	Value
Solution	d=2055

LOG

```
<---- Compile Equations: Stationary in study1/Approach Curve Solver (sol1) -----
Started at Jan 31, 2025, 8:55:55 PM.
Parameter d = 2.0550000000000002E-6 (m).
Geometry shape function: Linear serendipity
Number of vertex elements: 18
Number of boundary elements: 6094
Number of elements: 154022
Minimum element quality: 0.4831
Time: 8 s.
Physical memory: 3 GB
Virtual memory: 15.26 GB
Ended at Jan 31, 2025, 8:56:03 PM.
----- Compile Equations: Stationary in study1/Approach Curve Solver (sol1) ---->

<---- Dependent Variables 1 in study1/Approach Curve Solver (sol1) -------------
Started at Jan 31, 2025, 8:56:03 PM.
Solution time: 0 s.
Physical memory: 3 GB
Virtual memory: 15.26 GB
Ended at Jan 31, 2025, 8:56:03 PM.
----- Dependent Variables 1 in study1/Approach Curve Solver (sol1) ------------>

<---- Stationary Solver 1 in study1/Approach Curve Solver (sol1) ---------------
Started at Jan 31, 2025, 8:56:03 PM.
Segregated solver
Segregated Step 11 does not have any components solved for.
Number of degrees of freedom solved for: 781477 (plus 11611 internal DOFs).
Segregated solver iteration 1.
Merged variables
Nonsymmetric matrix found.
Scales for dependent variables:
Concentration (comp1.Cl): 1.4e+02
Concentration (comp1.K): 1.4e+02
Electric potential (comp1.V): 1.9e+02
Orthonormal null-space function used.
Iter      SolEst     Damping    Stepsize #Res #Jac #Sol   LinErr   LinRes
   1        0.83   0.3500000        0.83    2    1    1  3.8e-11  2.6e-15
   2        0.81   0.3500000        0.81    3    2    2  4.1e-11  2.7e-15
   3         0.8   0.3500000         0.8    4    3    3  3.1e-11  2.7e-15
Velocity u2, Pressure p
Nonsymmetric matrix found.
Scales for dependent variables:
Pressure (comp1.p): 1
Velocity field (spatial frame) (comp1.u2): 0.00018
Orthonormal null-space function used.
Iter      SolEst     Damping    Stepsize #Res #Jac #Sol   LinErr   LinRes
   1         1.1   0.5000000         1.1    2    1    1  1.8e-15  2.2e-15
Displacement field
Nonsymmetric matrix found.
Scales for dependent variables:
Auxiliary pressure (comp1.solid.lemm1.pw): 1
Displacement field (comp1.u): 2.8e-18
Orthonormal null-space function used.
Iter      SolEst     Damping    Stepsize #Res #Jac #Sol   LinErr   LinRes
   1         1.9   0.5000000         1.9    2    1    1  5.2e-14  1.5e-12
Spatial mesh displacement
```

```
Symmetric matrices found.
Scales for dependent variables:
Spatial mesh displacement (comp1.spatial.disp): 1.2e-09
Orthonormal null-space function used.
Iter      SolEst     Damping    Stepsize #Res #Jac #Sol   LinErr   LinRes
   1     7.9e-07   0.5000000     7.9e-07    2    1    1  1.8e-15  1.8e-15
Solution error estimates for segregated groups
1.4, 0.54, 0.95, 4e-07
Residual error estimates for segregated groups
7.8e+03, 8.2e+04, 2.1e+04, 0.11
Segregated solver iteration 2.
Merged variables
Scales for dependent variables:
Concentration (comp1.Cl): 1.4e+02
Concentration (comp1.K): 1.4e+02
Electric potential (comp1.V): 0.064
Iter      SolEst     Damping    Stepsize #Res #Jac #Sol   LinErr   LinRes
   1        0.78   0.3500000        0.78    6    4    4    8e-14  2.8e-15
   2        0.77   0.3500000        0.77    7    5    5  1.2e-13  2.9e-15
   3        0.76   0.3500000        0.76    8    6    6  5.7e-14  3.7e-15
Velocity u2, Pressure p
Scales for dependent variables:
Pressure (comp1.p): 3.7e+02
Velocity field (spatial frame) (comp1.u2): 0.00073
Iter      SolEst     Damping    Stepsize #Res #Jac #Sol   LinErr   LinRes
   1        0.36   0.5000000        0.36    4    2    2  2.3e-15  7.9e-15
Displacement field
Iter      SolEst     Damping    Stepsize #Res #Jac #Sol   LinErr   LinRes
   1        0.95   0.5000000        0.95    4    2    2  8.7e-14  1.5e-12
Spatial mesh displacement
Iter      SolEst     Damping    Stepsize #Res #Jac #Sol   LinErr   LinRes
   1     1.2e-06   0.5000000     1.2e-06    4    2    2  1.6e-15  1.8e-15
Solution error estimates for segregated groups
1.3, 0.18, 0.48, 5.9e-07
Residual error estimates for segregated groups
7.6e+02, 1.5e+04, 2.1e+04, 0.13
Segregated solver iteration 3.
Merged variables
Iter      SolEst     Damping    Stepsize #Res #Jac #Sol   LinErr   LinRes
   1        0.74   0.3500000        0.74   10    7    7  5.1e-14  3.4e-15
   2        0.73   0.3500000        0.73   11    8    8  7.4e-14  4.3e-15
   3        0.71   0.3500000        0.71   12    9    9  8.3e-14  3.8e-15
Velocity u2, Pressure p
Iter      SolEst     Damping    Stepsize #Res #Jac #Sol   LinErr   LinRes
   1        0.15   0.5000000        0.15    6    3    3  3.3e-15  7.8e-15
Displacement field
Iter      SolEst     Damping    Stepsize #Res #Jac #Sol   LinErr   LinRes
   1        0.52   0.5000000        0.52    6    3    3  3.6e-14  1.5e-12
Spatial mesh displacement
Iter      SolEst     Damping    Stepsize #Res #Jac #Sol   LinErr   LinRes
   1     1.2e-06   0.5000000     1.2e-06    6    3    3  1.4e-15  1.9e-15
Solution error estimates for segregated groups
1.2, 0.077, 0.26, 5.9e-07
Residual error estimates for segregated groups
2.1e+02, 8.5e+03, 1.6e+04, 0.13
Segregated solver iteration 4.
```

```
Merged variables
Iter      SolEst     Damping    Stepsize #Res #Jac #Sol   LinErr   LinRes
   1         0.7   0.3500000         0.7   14   10   10  5.6e-14    3e-15
   2        0.67   0.3500000        0.67   15   11   11    9e-14  3.3e-15
   3        0.58   0.3500000        0.58   16   12   12    7e-14  3.1e-15
Velocity u2, Pressure p
Iter      SolEst     Damping    Stepsize #Res #Jac #Sol   LinErr   LinRes
   1       0.072   0.5000000       0.072    8    4    4  2.8e-15  7.9e-15
Displacement field
Iter      SolEst     Damping    Stepsize #Res #Jac #Sol   LinErr   LinRes
   1        0.29   0.5000000        0.29    8    4    4  1.6e-13  1.5e-12
Spatial mesh displacement
Iter      SolEst     Damping    Stepsize #Res #Jac #Sol   LinErr   LinRes
   1     9.9e-07   0.5000000     9.9e-07    8    4    4  1.7e-15  1.8e-15
Solution error estimates for segregated groups
1, 0.036, 0.15, 4.9e-07
Residual error estimates for segregated groups
82, 4.5e+03, 1.1e+04, 0.13
Segregated solver iteration 5.
Merged variables
Iter      SolEst     Damping    Stepsize #Res #Jac #Sol   LinErr   LinRes
   1         0.4   0.3500000         0.4   18   13   13  6.7e-14  3.3e-15
   2        0.27   0.3500000        0.27   19   14   14    1e-13  2.8e-15
   3        0.18   0.3500000        0.18   20   15   15  1.2e-13  2.8e-15
Velocity u2, Pressure p
Iter      SolEst     Damping    Stepsize #Res #Jac #Sol   LinErr   LinRes
   1       0.035   0.5000000       0.035   10    5    5  2.5e-15  7.8e-15
Displacement field
Iter      SolEst     Damping    Stepsize #Res #Jac #Sol   LinErr   LinRes
   1        0.17   0.5000000        0.17   10    5    5  7.2e-14  1.5e-12
Spatial mesh displacement
Iter      SolEst     Damping    Stepsize #Res #Jac #Sol   LinErr   LinRes
   1     7.4e-07   0.5000000     7.4e-07   10    5    5  2.1e-15  1.8e-15
Solution error estimates for segregated groups
0.3, 0.017, 0.084, 3.7e-07
Residual error estimates for segregated groups
32, 2.3e+03, 6.8e+03, 0.14
Segregated solver iteration 6.
Merged variables
Iter      SolEst     Damping    Stepsize #Res #Jac #Sol   LinErr   LinRes
   1        0.12   0.3500000        0.12   22   16   16  7.9e-14  3.2e-15
   2       0.076   0.3500000       0.076   23   17   17  4.1e-14    3e-15
   3        0.05   0.3500000        0.05   24   18   18  5.6e-14  3.4e-15
Velocity u2, Pressure p
Iter      SolEst     Damping    Stepsize #Res #Jac #Sol   LinErr   LinRes
   1       0.017   0.5000000       0.017   12    6    6  3.2e-15  7.8e-15
Displacement field
Scales for dependent variables:
Auxiliary pressure (comp1.solid.lemm1.pw): 0.37
Displacement field (comp1.u): 2.9e-16
Iter      SolEst     Damping    Stepsize #Res #Jac #Sol   LinErr   LinRes
   1       0.095   0.5000000       0.095   12    6    6  1.1e-13  1.2e-12
Spatial mesh displacement
Iter      SolEst     Damping    Stepsize #Res #Jac #Sol   LinErr   LinRes
   1     5.2e-07   0.5000000     5.2e-07   12    6    6  1.7e-15  1.8e-15
Solution error estimates for segregated groups
```

```
0.085, 0.0086, 0.048, 2.6e-07
Residual error estimates for segregated groups
12, 1.2e+03, 4.2e+03, 0.13
Segregated solver iteration 7.
Merged variables
Iter      SolEst     Damping    Stepsize #Res #Jac #Sol   LinErr   LinRes
   1       0.032   0.3500000       0.032   26   19   19  4.2e-14  2.9e-15
   2       0.021   0.3500000       0.021   27   20   20  7.1e-14  3.1e-15
   3       0.014   0.3500000       0.014   28   21   21  1.1e-13  3.3e-15
Velocity u2, Pressure p
Iter      SolEst     Damping    Stepsize #Res #Jac #Sol   LinErr   LinRes
   1      0.0085   0.5000000      0.0085   14    7    7  3.6e-15  7.9e-15
Displacement field
Iter      SolEst     Damping    Stepsize #Res #Jac #Sol   LinErr   LinRes
   1       0.054   0.5000000       0.054   14    7    7  1.2e-13  1.2e-12
Spatial mesh displacement
Iter      SolEst     Damping    Stepsize #Res #Jac #Sol   LinErr   LinRes
   1     3.5e-07   0.5000000     3.5e-07   14    7    7  1.5e-15  1.8e-15
Solution error estimates for segregated groups
0.024, 0.0042, 0.027, 1.7e-07
Residual error estimates for segregated groups
4.1, 5.9e+02, 2.6e+03, 0.13
Segregated solver iteration 8.
Merged variables
Iter      SolEst     Damping    Stepsize #Res #Jac #Sol   LinErr   LinRes
   1      0.0089   0.3500000      0.0089   30   22   22  7.7e-14    3e-15
   2      0.0058   0.3500000      0.0058   31   23   23  7.9e-14  2.7e-15
   3      0.0038   0.3500000      0.0038   32   24   24  6.9e-14  2.7e-15
Velocity u2, Pressure p
Iter      SolEst     Damping    Stepsize #Res #Jac #Sol   LinErr   LinRes
   1      0.0042   0.5000000      0.0042   16    8    8  2.2e-15  7.9e-15
Displacement field
Iter      SolEst     Damping    Stepsize #Res #Jac #Sol   LinErr   LinRes
   1        0.03   0.5000000        0.03   16    8    8  9.3e-14  1.2e-12
Spatial mesh displacement
Iter      SolEst     Damping    Stepsize #Res #Jac #Sol   LinErr   LinRes
   1     2.2e-07   0.5000000     2.2e-07   16    8    8  1.5e-15  1.8e-15
Solution error estimates for segregated groups
0.0065, 0.0021, 0.015, 1.1e-07
Residual error estimates for segregated groups
1.3, 3e+02, 1.8e+03, 0.12
Segregated solver iteration 9.
Merged variables
Iter      SolEst     Damping    Stepsize #Res #Jac #Sol   LinErr   LinRes
   1      0.0025   0.3500000      0.0025   34   25   25  6.4e-14  3.2e-15
   2      0.0016   0.3500000      0.0016   35   26   26  4.7e-14  2.9e-15
   3       0.001   0.3500000       0.001   36   27   27  8.3e-14  3.8e-15
Velocity u2, Pressure p
Iter      SolEst     Damping    Stepsize #Res #Jac #Sol   LinErr   LinRes
   1      0.0021   0.5000000      0.0021   18    9    9  3.1e-15  7.8e-15
Displacement field
Iter      SolEst     Damping    Stepsize #Res #Jac #Sol   LinErr   LinRes
   1       0.017   0.5000000       0.017   18    9    9  1.5e-13  1.2e-12
Spatial mesh displacement
Iter      SolEst     Damping    Stepsize #Res #Jac #Sol   LinErr   LinRes
   1     1.4e-07   0.5000000     1.4e-07   18    9    9  1.8e-15  1.8e-15
```

```
Solution error estimates for segregated groups
0.0018, 0.0011, 0.0085, 7e-08
Residual error estimates for segregated groups
0.44, 1.5e+02, 1.4e+03, 0.13
Segregated solver iteration 10.
Merged variables
Iter      SolEst     Damping    Stepsize #Res #Jac #Sol   LinErr   LinRes
   1     0.00067   0.3500000     0.00067   38   28   28  9.9e-14  3.1e-15
   2     0.00044   0.3500000     0.00044   39   29   29  6.3e-14  4.2e-15
   3     0.00029   0.3500000     0.00029   40   30   30  7.6e-14  2.9e-15
Velocity u2, Pressure p
Iter      SolEst     Damping    Stepsize #Res #Jac #Sol   LinErr   LinRes
   1      0.0011   0.5000000      0.0011   20   10   10  2.4e-15  7.8e-15
Displacement field
Iter      SolEst     Damping    Stepsize #Res #Jac #Sol   LinErr   LinRes
   1      0.0094   0.5000000      0.0094   20   10   10  1.2e-13  1.2e-12
Spatial mesh displacement
Iter      SolEst     Damping    Stepsize #Res #Jac #Sol   LinErr   LinRes
   1     8.5e-08   0.5000000     8.5e-08   20   10   10  1.6e-15  1.8e-15
Solution error estimates for segregated groups
0.00049, 0.00053, 0.0047, 4.3e-08
Residual error estimates for segregated groups
0.14, 75, 1.2e+03, 0.13
Segregated solver iteration 11.
Merged variables
Iter      SolEst     Damping    Stepsize #Res #Jac #Sol   LinErr   LinRes
   1     0.00019   0.3500000     0.00019   42   31   31  6.2e-14  2.9e-15
   2     0.00012   0.3500000     0.00012   43   32   32  9.1e-14  3.4e-15
   3     7.8e-05   0.3500000     7.8e-05   44   33   33  1.2e-13  2.7e-15
Velocity u2, Pressure p
Iter      SolEst     Damping    Stepsize #Res #Jac #Sol   LinErr   LinRes
   1     0.00053   0.5000000     0.00053   22   11   11  3.2e-15    8e-15
Displacement field
Iter      SolEst     Damping    Stepsize #Res #Jac #Sol   LinErr   LinRes
   1      0.0051   0.5000000      0.0051   22   11   11  1.4e-13  1.2e-12
Spatial mesh displacement
Iter      SolEst     Damping    Stepsize #Res #Jac #Sol   LinErr   LinRes
   1     5.1e-08   0.5000000     5.1e-08   22   11   11  1.3e-15  1.8e-15
Solution error estimates for segregated groups
0.00013, 0.00026, 0.0026, 2.6e-08
Residual error estimates for segregated groups
0.043, 37, 1.2e+03, 0.13
Segregated solver iteration 12.
Merged variables
Iter      SolEst     Damping    Stepsize #Res #Jac #Sol   LinErr   LinRes
   1     5.1e-05   0.3500000     5.1e-05   46   34   34  7.6e-14  3.2e-15
   2     3.3e-05   0.3500000     3.3e-05   47   35   35  7.5e-14  3.1e-15
   3     2.1e-05   0.3500000     2.1e-05   48   36   36  4.7e-14    3e-15
Velocity u2, Pressure p
Iter      SolEst     Damping    Stepsize #Res #Jac #Sol   LinErr   LinRes
   1     0.00026   0.5000000     0.00026   24   12   12  2.9e-15  7.9e-15
Displacement field
Iter      SolEst     Damping    Stepsize #Res #Jac #Sol   LinErr   LinRes
   1      0.0028   0.5000000      0.0028   24   12   12  2.1e-13  1.2e-12
Spatial mesh displacement
Iter      SolEst     Damping    Stepsize #Res #Jac #Sol   LinErr   LinRes
```

```
   1       3e-08   0.5000000       3e-08   24   12   12  1.2e-15  1.8e-15
Solution error estimates for segregated groups
3.7e-05, 0.00013, 0.0014, 1.5e-08
Residual error estimates for segregated groups
0.013, 19, 1.2e+03, 0.12
Segregated solver iteration 13.
Merged variables
Iter      SolEst     Damping    Stepsize #Res #Jac #Sol   LinErr   LinRes
   1     1.4e-05   0.3500000     1.4e-05   50   37   37  5.6e-14  3.1e-15
   2     9.1e-06   0.3500000     9.1e-06   51   38   38  7.3e-14  2.8e-15
   3     5.9e-06   0.3500000     5.9e-06   52   39   39  5.7e-14  3.2e-15
Velocity u2, Pressure p
Iter      SolEst     Damping    Stepsize #Res #Jac #Sol   LinErr   LinRes
   1     0.00013   0.5000000     0.00013   26   13   13  3.1e-15  7.9e-15
Displacement field
Iter      SolEst     Damping    Stepsize #Res #Jac #Sol   LinErr   LinRes
   1      0.0015   0.5000000      0.0015   26   13   13    1e-13  1.2e-12
Spatial mesh displacement
Iter      SolEst     Damping    Stepsize #Res #Jac #Sol   LinErr   LinRes
   1     1.8e-08   0.5000000     1.8e-08   26   13   13  1.4e-15  1.9e-15
Solution error estimates for segregated groups
1e-05, 6.6e-05, 0.00076, 8.8e-09
Residual error estimates for segregated groups
0.004, 9.3, 1.2e+03, 0.13
Solution time: 156 s. (2 minutes, 36 seconds)
Physical memory: 4.75 GB
Virtual memory: 25.61 GB
Ended at Jan 31, 2025, 8:58:38 PM.
----- Stationary Solver 1 in study1/Approach Curve Solver (sol1) -------------->
```

d=2050 (su92)

GENERAL

Description	Value
Solution	d=2050

LOG

```
<---- Compile Equations: Stationary in study1/Approach Curve Solver (sol1) -----
Started at Jan 31, 2025, 8:58:39 PM.
Parameter d = 2.0500000000000003E-6 (m).
Geometry shape function: Linear serendipity
Number of vertex elements: 18
Number of boundary elements: 6091
Number of elements: 154069
Minimum element quality: 0.4644
Time: 8 s.
Physical memory: 3.01 GB
Virtual memory: 15.26 GB
Ended at Jan 31, 2025, 8:58:47 PM.
----- Compile Equations: Stationary in study1/Approach Curve Solver (sol1) ---->

<---- Dependent Variables 1 in study1/Approach Curve Solver (sol1) -------------
Started at Jan 31, 2025, 8:58:47 PM.
Solution time: 0 s.
Physical memory: 3.01 GB
Virtual memory: 15.26 GB
Ended at Jan 31, 2025, 8:58:47 PM.
----- Dependent Variables 1 in study1/Approach Curve Solver (sol1) ------------>

<---- Stationary Solver 1 in study1/Approach Curve Solver (sol1) ---------------
Started at Jan 31, 2025, 8:58:47 PM.
Segregated solver
Segregated Step 11 does not have any components solved for.
Number of degrees of freedom solved for: 781748 (plus 11605 internal DOFs).
Segregated solver iteration 1.
Merged variables
Nonsymmetric matrix found.
Scales for dependent variables:
Concentration (comp1.Cl): 1.4e+02
Concentration (comp1.K): 1.4e+02
Electric potential (comp1.V): 1.9e+02
Orthonormal null-space function used.
Iter      SolEst     Damping    Stepsize #Res #Jac #Sol   LinErr   LinRes
   1        0.83   0.3500000        0.83    2    1    1  2.6e-11  2.8e-15
   2        0.81   0.3500000        0.81    3    2    2  4.8e-11  2.7e-15
   3         0.8   0.3500000         0.8    4    3    3  1.4e-11  2.7e-15
Velocity u2, Pressure p
Nonsymmetric matrix found.
Scales for dependent variables:
Pressure (comp1.p): 1
Velocity field (spatial frame) (comp1.u2): 0.00018
Orthonormal null-space function used.
Iter      SolEst     Damping    Stepsize #Res #Jac #Sol   LinErr   LinRes
   1         1.1   0.5000000         1.1    2    1    1  1.5e-15  2.3e-15
Displacement field
Nonsymmetric matrix found.
Scales for dependent variables:
Auxiliary pressure (comp1.solid.lemm1.pw): 1
Displacement field (comp1.u): 2.8e-18
Orthonormal null-space function used.
Iter      SolEst     Damping    Stepsize #Res #Jac #Sol   LinErr   LinRes
   1         1.9   0.5000000         1.9    2    1    1  5.5e-14  1.4e-12
Spatial mesh displacement
```

```
Symmetric matrices found.
Scales for dependent variables:
Spatial mesh displacement (comp1.spatial.disp): 1.2e-09
Orthonormal null-space function used.
Iter      SolEst     Damping    Stepsize #Res #Jac #Sol   LinErr   LinRes
   1     7.9e-07   0.5000000     7.9e-07    2    1    1  1.7e-15  1.8e-15
Solution error estimates for segregated groups
1.4, 0.54, 0.95, 3.9e-07
Residual error estimates for segregated groups
7.8e+03, 8.2e+04, 2.1e+04, 0.12
Segregated solver iteration 2.
Merged variables
Scales for dependent variables:
Concentration (comp1.Cl): 1.4e+02
Concentration (comp1.K): 1.4e+02
Electric potential (comp1.V): 0.064
Iter      SolEst     Damping    Stepsize #Res #Jac #Sol   LinErr   LinRes
   1        0.78   0.3500000        0.78    6    4    4    7e-14  2.9e-15
   2        0.77   0.3500000        0.77    7    5    5  8.6e-14  2.9e-15
   3        0.76   0.3500000        0.76    8    6    6  7.1e-14  3.5e-15
Velocity u2, Pressure p
Scales for dependent variables:
Pressure (comp1.p): 3.7e+02
Velocity field (spatial frame) (comp1.u2): 0.00073
Iter      SolEst     Damping    Stepsize #Res #Jac #Sol   LinErr   LinRes
   1        0.36   0.5000000        0.36    4    2    2    2e-15  7.8e-15
Displacement field
Iter      SolEst     Damping    Stepsize #Res #Jac #Sol   LinErr   LinRes
   1        0.95   0.5000000        0.95    4    2    2  1.3e-13  1.5e-12
Spatial mesh displacement
Iter      SolEst     Damping    Stepsize #Res #Jac #Sol   LinErr   LinRes
   1     1.2e-06   0.5000000     1.2e-06    4    2    2  1.3e-15  1.8e-15
Solution error estimates for segregated groups
1.3, 0.18, 0.48, 5.9e-07
Residual error estimates for segregated groups
7.6e+02, 1.5e+04, 2.1e+04, 0.14
Segregated solver iteration 3.
Merged variables
Iter      SolEst     Damping    Stepsize #Res #Jac #Sol   LinErr   LinRes
   1        0.74   0.3500000        0.74   10    7    7  7.6e-14  2.9e-15
   2        0.73   0.3500000        0.73   11    8    8    8e-14  2.8e-15
   3        0.71   0.3500000        0.71   12    9    9    7e-14    3e-15
Velocity u2, Pressure p
Iter      SolEst     Damping    Stepsize #Res #Jac #Sol   LinErr   LinRes
   1        0.15   0.5000000        0.15    6    3    3  5.4e-15  7.9e-15
Displacement field
Iter      SolEst     Damping    Stepsize #Res #Jac #Sol   LinErr   LinRes
   1        0.52   0.5000000        0.52    6    3    3  5.6e-14  1.5e-12
Spatial mesh displacement
Iter      SolEst     Damping    Stepsize #Res #Jac #Sol   LinErr   LinRes
   1     1.2e-06   0.5000000     1.2e-06    6    3    3  1.3e-15  1.8e-15
Solution error estimates for segregated groups
1.2, 0.077, 0.26, 5.9e-07
Residual error estimates for segregated groups
2.1e+02, 8.7e+03, 1.6e+04, 0.13
Segregated solver iteration 4.
```

```
Merged variables
Iter      SolEst     Damping    Stepsize #Res #Jac #Sol   LinErr   LinRes
   1         0.7   0.3500000         0.7   14   10   10  1.1e-13    3e-15
   2        0.67   0.3500000        0.67   15   11   11  1.1e-13  2.7e-15
   3        0.58   0.3500000        0.58   16   12   12  6.7e-14  3.1e-15
Velocity u2, Pressure p
Iter      SolEst     Damping    Stepsize #Res #Jac #Sol   LinErr   LinRes
   1       0.072   0.5000000       0.072    8    4    4  4.1e-15  7.7e-15
Displacement field
Iter      SolEst     Damping    Stepsize #Res #Jac #Sol   LinErr   LinRes
   1        0.29   0.5000000        0.29    8    4    4    7e-14  1.5e-12
Spatial mesh displacement
Iter      SolEst     Damping    Stepsize #Res #Jac #Sol   LinErr   LinRes
   1     9.8e-07   0.5000000     9.8e-07    8    4    4  1.5e-15  1.8e-15
Solution error estimates for segregated groups
1, 0.036, 0.15, 4.9e-07
Residual error estimates for segregated groups
82, 4.6e+03, 1.1e+04, 0.13
Segregated solver iteration 5.
Merged variables
Iter      SolEst     Damping    Stepsize #Res #Jac #Sol   LinErr   LinRes
   1         0.4   0.3500000         0.4   18   13   13  8.3e-14  2.8e-15
   2        0.27   0.3500000        0.27   19   14   14  4.3e-14  3.2e-15
   3        0.18   0.3500000        0.18   20   15   15  6.2e-14    4e-15
Velocity u2, Pressure p
Iter      SolEst     Damping    Stepsize #Res #Jac #Sol   LinErr   LinRes
   1       0.035   0.5000000       0.035   10    5    5  2.5e-15  7.9e-15
Displacement field
Iter      SolEst     Damping    Stepsize #Res #Jac #Sol   LinErr   LinRes
   1        0.17   0.5000000        0.17   10    5    5  6.1e-14  1.5e-12
Spatial mesh displacement
Iter      SolEst     Damping    Stepsize #Res #Jac #Sol   LinErr   LinRes
   1     7.4e-07   0.5000000     7.4e-07   10    5    5  1.2e-15  1.8e-15
Solution error estimates for segregated groups
0.3, 0.017, 0.084, 3.7e-07
Residual error estimates for segregated groups
32, 2.4e+03, 6.8e+03, 0.14
Segregated solver iteration 6.
Merged variables
Iter      SolEst     Damping    Stepsize #Res #Jac #Sol   LinErr   LinRes
   1        0.12   0.3500000        0.12   22   16   16  1.2e-13    3e-15
   2       0.076   0.3500000       0.076   23   17   17  4.4e-14  2.8e-15
   3        0.05   0.3500000        0.05   24   18   18  1.8e-13  3.5e-15
Velocity u2, Pressure p
Iter      SolEst     Damping    Stepsize #Res #Jac #Sol   LinErr   LinRes
   1       0.017   0.5000000       0.017   12    6    6  2.8e-15    8e-15
Displacement field
Scales for dependent variables:
Auxiliary pressure (comp1.solid.lemm1.pw): 0.37
Displacement field (comp1.u): 2.9e-16
Iter      SolEst     Damping    Stepsize #Res #Jac #Sol   LinErr   LinRes
   1       0.095   0.5000000       0.095   12    6    6  1.2e-13  1.2e-12
Spatial mesh displacement
Iter      SolEst     Damping    Stepsize #Res #Jac #Sol   LinErr   LinRes
   1     5.2e-07   0.5000000     5.2e-07   12    6    6  1.3e-15  1.8e-15
Solution error estimates for segregated groups
```

```
0.085, 0.0086, 0.048, 2.6e-07
Residual error estimates for segregated groups
12, 1.2e+03, 4.2e+03, 0.14
Segregated solver iteration 7.
Merged variables
Iter      SolEst     Damping    Stepsize #Res #Jac #Sol   LinErr   LinRes
   1       0.032   0.3500000       0.032   26   19   19  1.1e-13  2.8e-15
   2       0.021   0.3500000       0.021   27   20   20  6.9e-14  3.2e-15
   3       0.014   0.3500000       0.014   28   21   21  8.2e-14  2.9e-15
Velocity u2, Pressure p
Iter      SolEst     Damping    Stepsize #Res #Jac #Sol   LinErr   LinRes
   1      0.0085   0.5000000      0.0085   14    7    7    3e-15  7.8e-15
Displacement field
Iter      SolEst     Damping    Stepsize #Res #Jac #Sol   LinErr   LinRes
   1       0.054   0.5000000       0.054   14    7    7  1.2e-13  1.2e-12
Spatial mesh displacement
Iter      SolEst     Damping    Stepsize #Res #Jac #Sol   LinErr   LinRes
   1     3.4e-07   0.5000000     3.4e-07   14    7    7  1.5e-15  1.9e-15
Solution error estimates for segregated groups
0.024, 0.0042, 0.027, 1.7e-07
Residual error estimates for segregated groups
4.1, 6e+02, 2.6e+03, 0.13
Segregated solver iteration 8.
Merged variables
Iter      SolEst     Damping    Stepsize #Res #Jac #Sol   LinErr   LinRes
   1      0.0089   0.3500000      0.0089   30   22   22  8.7e-14  3.1e-15
   2      0.0058   0.3500000      0.0058   31   23   23  1.7e-13  4.2e-15
   3      0.0038   0.3500000      0.0038   32   24   24  9.4e-14    3e-15
Velocity u2, Pressure p
Iter      SolEst     Damping    Stepsize #Res #Jac #Sol   LinErr   LinRes
   1      0.0042   0.5000000      0.0042   16    8    8  5.2e-15  7.9e-15
Displacement field
Iter      SolEst     Damping    Stepsize #Res #Jac #Sol   LinErr   LinRes
   1        0.03   0.5000000        0.03   16    8    8  1.1e-13  1.2e-12
Spatial mesh displacement
Iter      SolEst     Damping    Stepsize #Res #Jac #Sol   LinErr   LinRes
   1     2.2e-07   0.5000000     2.2e-07   16    8    8    2e-15  1.8e-15
Solution error estimates for segregated groups
0.0065, 0.0021, 0.015, 1.1e-07
Residual error estimates for segregated groups
1.4, 3e+02, 1.8e+03, 0.14
Segregated solver iteration 9.
Merged variables
Iter      SolEst     Damping    Stepsize #Res #Jac #Sol   LinErr   LinRes
   1      0.0025   0.3500000      0.0025   34   25   25  1.3e-13  2.8e-15
   2      0.0016   0.3500000      0.0016   35   26   26  1.1e-13  2.8e-15
   3       0.001   0.3500000       0.001   36   27   27  8.1e-14  2.8e-15
Velocity u2, Pressure p
Iter      SolEst     Damping    Stepsize #Res #Jac #Sol   LinErr   LinRes
   1      0.0021   0.5000000      0.0021   18    9    9  2.8e-15  7.9e-15
Displacement field
Iter      SolEst     Damping    Stepsize #Res #Jac #Sol   LinErr   LinRes
   1       0.017   0.5000000       0.017   18    9    9  1.3e-13  1.2e-12
Spatial mesh displacement
Iter      SolEst     Damping    Stepsize #Res #Jac #Sol   LinErr   LinRes
   1     1.4e-07   0.5000000     1.4e-07   18    9    9  1.7e-15  1.8e-15
```

```
Solution error estimates for segregated groups
0.0018, 0.0011, 0.0085, 6.9e-08
Residual error estimates for segregated groups
0.44, 1.5e+02, 1.4e+03, 0.12
Segregated solver iteration 10.
Merged variables
Iter      SolEst     Damping    Stepsize #Res #Jac #Sol   LinErr   LinRes
   1     0.00068   0.3500000     0.00068   38   28   28  1.2e-13    4e-15
   2     0.00044   0.3500000     0.00044   39   29   29  7.8e-14  4.2e-15
   3     0.00029   0.3500000     0.00029   40   30   30    1e-13    3e-15
Velocity u2, Pressure p
Iter      SolEst     Damping    Stepsize #Res #Jac #Sol   LinErr   LinRes
   1      0.0011   0.5000000      0.0011   20   10   10  2.3e-15  7.9e-15
Displacement field
Iter      SolEst     Damping    Stepsize #Res #Jac #Sol   LinErr   LinRes
   1      0.0094   0.5000000      0.0094   20   10   10    1e-13  1.2e-12
Spatial mesh displacement
Iter      SolEst     Damping    Stepsize #Res #Jac #Sol   LinErr   LinRes
   1     8.4e-08   0.5000000     8.4e-08   20   10   10  1.5e-15  1.8e-15
Solution error estimates for segregated groups
0.00049, 0.00053, 0.0047, 4.2e-08
Residual error estimates for segregated groups
0.14, 76, 1.2e+03, 0.13
Segregated solver iteration 11.
Merged variables
Iter      SolEst     Damping    Stepsize #Res #Jac #Sol   LinErr   LinRes
   1     0.00019   0.3500000     0.00019   42   31   31  9.1e-14  2.7e-15
   2     0.00012   0.3500000     0.00012   43   32   32  1.2e-13  3.2e-15
   3     7.8e-05   0.3500000     7.8e-05   44   33   33  5.8e-14  3.8e-15
Velocity u2, Pressure p
Iter      SolEst     Damping    Stepsize #Res #Jac #Sol   LinErr   LinRes
   1     0.00053   0.5000000     0.00053   22   11   11  4.3e-15  7.8e-15
Displacement field
Iter      SolEst     Damping    Stepsize #Res #Jac #Sol   LinErr   LinRes
   1      0.0051   0.5000000      0.0051   22   11   11  1.2e-13  1.2e-12
Spatial mesh displacement
Iter      SolEst     Damping    Stepsize #Res #Jac #Sol   LinErr   LinRes
   1     5.1e-08   0.5000000     5.1e-08   22   11   11  1.8e-15  1.8e-15
Solution error estimates for segregated groups
0.00013, 0.00026, 0.0026, 2.5e-08
Residual error estimates for segregated groups
0.043, 38, 1.2e+03, 0.12
Segregated solver iteration 12.
Merged variables
Iter      SolEst     Damping    Stepsize #Res #Jac #Sol   LinErr   LinRes
   1     5.1e-05   0.3500000     5.1e-05   46   34   34  1.2e-13  2.9e-15
   2     3.3e-05   0.3500000     3.3e-05   47   35   35  9.7e-14  2.8e-15
   3     2.2e-05   0.3500000     2.2e-05   48   36   36  8.4e-14  3.2e-15
Velocity u2, Pressure p
Iter      SolEst     Damping    Stepsize #Res #Jac #Sol   LinErr   LinRes
   1     0.00026   0.5000000     0.00026   24   12   12  2.3e-15  7.9e-15
Displacement field
Iter      SolEst     Damping    Stepsize #Res #Jac #Sol   LinErr   LinRes
   1      0.0028   0.5000000      0.0028   24   12   12    1e-13  1.2e-12
Spatial mesh displacement
Iter      SolEst     Damping    Stepsize #Res #Jac #Sol   LinErr   LinRes
```

```
   1        3e-08   0.5000000       3e-08   24    12    12  1.3e-15  1.8e-15
Solution error estimates for segregated groups
3.7e-05, 0.00013, 0.0014, 1.5e-08
Residual error estimates for segregated groups
0.013, 19, 1.2e+03, 0.13
Segregated solver iteration 13.
Merged variables
Iter      SolEst     Damping    Stepsize #Res #Jac #Sol   LinErr   LinRes
   1     1.4e-05   0.3500000     1.4e-05   50    37    37  7.6e-14  2.8e-15
   2     9.1e-06   0.3500000     9.1e-06   51    38    38  8.4e-14  3.5e-15
   3     5.9e-06   0.3500000     5.9e-06   52    39    39  7.7e-14  2.8e-15
Velocity u2, Pressure p
Iter      SolEst     Damping    Stepsize #Res #Jac #Sol   LinErr   LinRes
   1     0.00013   0.5000000     0.00013   26    13    13  2.2e-15  7.9e-15
Displacement field
Iter      SolEst     Damping    Stepsize #Res #Jac #Sol   LinErr   LinRes
   1      0.0015   0.5000000      0.0015   26    13    13  1.2e-13  1.2e-12
Spatial mesh displacement
Iter      SolEst     Damping    Stepsize #Res #Jac #Sol   LinErr   LinRes
   1     1.7e-08   0.5000000     1.7e-08   26    13    13  1.7e-15  1.8e-15
Solution error estimates for segregated groups
1e-05, 6.6e-05, 0.00076, 8.7e-09
Residual error estimates for segregated groups
0.004, 9.5, 1.2e+03, 0.12
Solution time: 154 s. (2 minutes, 34 seconds)
Physical memory: 4.77 GB
Virtual memory: 25.58 GB
Ended at Jan 31, 2025, 9:01:21 PM.
----- Stationary Solver 1 in study1/Approach Curve Solver (sol1) -------------->
```

d=2045 (su93)

GENERAL

Description	Value
Solution	d=2045

LOG

```
<---- Compile Equations: Stationary in study1/Approach Curve Solver (sol1) -----
Started at Jan 31, 2025, 9:01:21 PM.
Parameter d = 2.0450000000000003E-6 (m).
Geometry shape function: Linear serendipity
Number of vertex elements: 18
Number of boundary elements: 6089
Number of elements: 153643
Minimum element quality: 0.4807
Time: 8 s.
Physical memory: 3.01 GB
Virtual memory: 15.25 GB
Ended at Jan 31, 2025, 9:01:30 PM.
----- Compile Equations: Stationary in study1/Approach Curve Solver (sol1) ---->

<---- Dependent Variables 1 in study1/Approach Curve Solver (sol1) -------------
Started at Jan 31, 2025, 9:01:30 PM.
Solution time: 0 s.
Physical memory: 3.01 GB
Virtual memory: 15.25 GB
Ended at Jan 31, 2025, 9:01:30 PM.
----- Dependent Variables 1 in study1/Approach Curve Solver (sol1) ------------>

<---- Stationary Solver 1 in study1/Approach Curve Solver (sol1) ---------------
Started at Jan 31, 2025, 9:01:30 PM.
Segregated solver
Segregated Step 11 does not have any components solved for.
Number of degrees of freedom solved for: 779366 (plus 11601 internal DOFs).
Segregated solver iteration 1.
Merged variables
Nonsymmetric matrix found.
Scales for dependent variables:
Concentration (comp1.Cl): 1.4e+02
Concentration (comp1.K): 1.4e+02
Electric potential (comp1.V): 1.9e+02
Orthonormal null-space function used.
Iter      SolEst     Damping    Stepsize #Res #Jac #Sol   LinErr   LinRes
   1        0.83   0.3500000        0.83    2    1    1  2.3e-11  2.8e-15
   2        0.81   0.3500000        0.81    3    2    2  1.3e-11  2.8e-15
   3         0.8   0.3500000         0.8    4    3    3    5e-11  2.7e-15
Velocity u2, Pressure p
Nonsymmetric matrix found.
Scales for dependent variables:
Pressure (comp1.p): 1
Velocity field (spatial frame) (comp1.u2): 0.00018
Orthonormal null-space function used.
Iter      SolEst     Damping    Stepsize #Res #Jac #Sol   LinErr   LinRes
   1         1.1   0.5000000         1.1    2    1    1  1.6e-15  2.3e-15
Displacement field
Nonsymmetric matrix found.
Scales for dependent variables:
Auxiliary pressure (comp1.solid.lemm1.pw): 1
Displacement field (comp1.u): 2.8e-18
Orthonormal null-space function used.
Iter      SolEst     Damping    Stepsize #Res #Jac #Sol   LinErr   LinRes
   1         1.9   0.5000000         1.9    2    1    1  5.1e-14  1.4e-12
Spatial mesh displacement
```

```
Symmetric matrices found.
Scales for dependent variables:
Spatial mesh displacement (comp1.spatial.disp): 1.2e-09
Orthonormal null-space function used.
Iter      SolEst     Damping    Stepsize #Res #Jac #Sol   LinErr   LinRes
   1       8e-07   0.5000000       8e-07    2    1    1  1.3e-15  1.8e-15
Solution error estimates for segregated groups
1.4, 0.54, 0.95, 4e-07
Residual error estimates for segregated groups
7.8e+03, 8.2e+04, 2.1e+04, 0.12
Segregated solver iteration 2.
Merged variables
Scales for dependent variables:
Concentration (comp1.Cl): 1.4e+02
Concentration (comp1.K): 1.4e+02
Electric potential (comp1.V): 0.064
Iter      SolEst     Damping    Stepsize #Res #Jac #Sol   LinErr   LinRes
   1        0.78   0.3500000        0.78    6    4    4  5.6e-14  2.7e-15
   2        0.77   0.3500000        0.77    7    5    5  8.4e-14  2.8e-15
   3        0.76   0.3500000        0.76    8    6    6  8.5e-14  2.8e-15
Velocity u2, Pressure p
Scales for dependent variables:
Pressure (comp1.p): 3.7e+02
Velocity field (spatial frame) (comp1.u2): 0.00073
Iter      SolEst     Damping    Stepsize #Res #Jac #Sol   LinErr   LinRes
   1        0.36   0.5000000        0.36    4    2    2  4.5e-15  7.8e-15
Displacement field
Iter      SolEst     Damping    Stepsize #Res #Jac #Sol   LinErr   LinRes
   1        0.95   0.5000000        0.95    4    2    2    1e-13  1.5e-12
Spatial mesh displacement
Iter      SolEst     Damping    Stepsize #Res #Jac #Sol   LinErr   LinRes
   1     1.2e-06   0.5000000     1.2e-06    4    2    2  1.8e-15  1.9e-15
Solution error estimates for segregated groups
1.3, 0.18, 0.48, 6e-07
Residual error estimates for segregated groups
7.6e+02, 1.7e+04, 2.1e+04, 0.12
Segregated solver iteration 3.
Merged variables
Iter      SolEst     Damping    Stepsize #Res #Jac #Sol   LinErr   LinRes
   1        0.74   0.3500000        0.74   10    7    7  8.2e-14  2.8e-15
   2        0.73   0.3500000        0.73   11    8    8  6.6e-14  2.9e-15
   3        0.71   0.3500000        0.71   12    9    9  1.1e-13  2.9e-15
Velocity u2, Pressure p
Iter      SolEst     Damping    Stepsize #Res #Jac #Sol   LinErr   LinRes
   1        0.15   0.5000000        0.15    6    3    3  3.5e-15    8e-15
Displacement field
Iter      SolEst     Damping    Stepsize #Res #Jac #Sol   LinErr   LinRes
   1        0.52   0.5000000        0.52    6    3    3  8.5e-14  1.4e-12
Spatial mesh displacement
Iter      SolEst     Damping    Stepsize #Res #Jac #Sol   LinErr   LinRes
   1     1.2e-06   0.5000000     1.2e-06    6    3    3  1.3e-15  1.9e-15
Solution error estimates for segregated groups
1.2, 0.077, 0.26, 6e-07
Residual error estimates for segregated groups
2.1e+02, 9.8e+03, 1.6e+04, 0.12
Segregated solver iteration 4.
```

```
Merged variables
Iter      SolEst     Damping    Stepsize #Res #Jac #Sol   LinErr   LinRes
   1         0.7   0.3500000         0.7   14   10   10  1.1e-13  2.8e-15
   2        0.67   0.3500000        0.67   15   11   11  1.4e-13  2.8e-15
   3        0.58   0.3500000        0.58   16   12   12  1.4e-13  2.9e-15
Velocity u2, Pressure p
Iter      SolEst     Damping    Stepsize #Res #Jac #Sol   LinErr   LinRes
   1       0.072   0.5000000       0.072    8    4    4  2.6e-15  7.9e-15
Displacement field
Iter      SolEst     Damping    Stepsize #Res #Jac #Sol   LinErr   LinRes
   1        0.29   0.5000000        0.29    8    4    4  4.3e-14  1.5e-12
Spatial mesh displacement
Iter      SolEst     Damping    Stepsize #Res #Jac #Sol   LinErr   LinRes
   1       1e-06   0.5000000       1e-06    8    4    4  1.8e-15  1.8e-15
Solution error estimates for segregated groups
1, 0.036, 0.15, 5e-07
Residual error estimates for segregated groups
81, 5.2e+03, 1.1e+04, 0.14
Segregated solver iteration 5.
Merged variables
Iter      SolEst     Damping    Stepsize #Res #Jac #Sol   LinErr   LinRes
   1         0.4   0.3500000         0.4   18   13   13    1e-13  2.8e-15
   2        0.27   0.3500000        0.27   19   14   14  9.6e-14  2.9e-15
   3        0.18   0.3500000        0.18   20   15   15    7e-14  2.6e-15
Velocity u2, Pressure p
Iter      SolEst     Damping    Stepsize #Res #Jac #Sol   LinErr   LinRes
   1       0.035   0.5000000       0.035   10    5    5  4.6e-15    8e-15
Displacement field
Iter      SolEst     Damping    Stepsize #Res #Jac #Sol   LinErr   LinRes
   1        0.17   0.5000000        0.17   10    5    5  1.5e-13  1.5e-12
Spatial mesh displacement
Iter      SolEst     Damping    Stepsize #Res #Jac #Sol   LinErr   LinRes
   1     7.5e-07   0.5000000     7.5e-07   10    5    5  1.3e-15  1.9e-15
Solution error estimates for segregated groups
0.3, 0.017, 0.084, 3.7e-07
Residual error estimates for segregated groups
32, 2.7e+03, 6.8e+03, 0.14
Segregated solver iteration 6.
Merged variables
Iter      SolEst     Damping    Stepsize #Res #Jac #Sol   LinErr   LinRes
   1        0.12   0.3500000        0.12   22   16   16  6.1e-14  2.8e-15
   2       0.076   0.3500000       0.076   23   17   17  8.6e-14  2.8e-15
   3        0.05   0.3500000        0.05   24   18   18  1.2e-13  2.8e-15
Velocity u2, Pressure p
Iter      SolEst     Damping    Stepsize #Res #Jac #Sol   LinErr   LinRes
   1       0.017   0.5000000       0.017   12    6    6  2.5e-15  7.8e-15
Displacement field
Scales for dependent variables:
Auxiliary pressure (comp1.solid.lemm1.pw): 0.37
Displacement field (comp1.u): 3e-16
Iter      SolEst     Damping    Stepsize #Res #Jac #Sol   LinErr   LinRes
   1       0.096   0.5000000       0.096   12    6    6  1.4e-13  1.2e-12
Spatial mesh displacement
Iter      SolEst     Damping    Stepsize #Res #Jac #Sol   LinErr   LinRes
   1     5.2e-07   0.5000000     5.2e-07   12    6    6  1.4e-15  1.8e-15
Solution error estimates for segregated groups
```

```
0.085, 0.0086, 0.048, 2.6e-07
Residual error estimates for segregated groups
12, 1.4e+03, 4.2e+03, 0.13
Segregated solver iteration 7.
Merged variables
Iter      SolEst     Damping    Stepsize #Res #Jac #Sol   LinErr   LinRes
   1       0.032   0.3500000       0.032   26   19   19  1.3e-13  2.8e-15
   2       0.021   0.3500000       0.021   27   20   20  7.9e-14  2.8e-15
   3       0.014   0.3500000       0.014   28   21   21  4.8e-14    3e-15
Velocity u2, Pressure p
Iter      SolEst     Damping    Stepsize #Res #Jac #Sol   LinErr   LinRes
   1      0.0085   0.5000000      0.0085   14    7    7  3.3e-15  7.9e-15
Displacement field
Iter      SolEst     Damping    Stepsize #Res #Jac #Sol   LinErr   LinRes
   1       0.054   0.5000000       0.054   14    7    7  1.3e-13  1.2e-12
Spatial mesh displacement
Iter      SolEst     Damping    Stepsize #Res #Jac #Sol   LinErr   LinRes
   1     3.5e-07   0.5000000     3.5e-07   14    7    7  2.1e-15  1.8e-15
Solution error estimates for segregated groups
0.023, 0.0042, 0.027, 1.7e-07
Residual error estimates for segregated groups
4, 6.8e+02, 2.6e+03, 0.13
Segregated solver iteration 8.
Merged variables
Iter      SolEst     Damping    Stepsize #Res #Jac #Sol   LinErr   LinRes
   1      0.0089   0.3500000      0.0089   30   22   22  8.8e-14  2.8e-15
   2      0.0058   0.3500000      0.0058   31   23   23  9.1e-14  2.7e-15
   3      0.0038   0.3500000      0.0038   32   24   24  6.2e-14  2.8e-15
Velocity u2, Pressure p
Iter      SolEst     Damping    Stepsize #Res #Jac #Sol   LinErr   LinRes
   1      0.0042   0.5000000      0.0042   16    8    8  2.4e-15    8e-15
Displacement field
Iter      SolEst     Damping    Stepsize #Res #Jac #Sol   LinErr   LinRes
   1        0.03   0.5000000        0.03   16    8    8  1.5e-13  1.2e-12
Spatial mesh displacement
Iter      SolEst     Damping    Stepsize #Res #Jac #Sol   LinErr   LinRes
   1     2.2e-07   0.5000000     2.2e-07   16    8    8  1.8e-15  1.8e-15
Solution error estimates for segregated groups
0.0065, 0.0021, 0.015, 1.1e-07
Residual error estimates for segregated groups
1.3, 3.4e+02, 1.8e+03, 0.13
Segregated solver iteration 9.
Merged variables
Iter      SolEst     Damping    Stepsize #Res #Jac #Sol   LinErr   LinRes
   1      0.0024   0.3500000      0.0024   34   25   25  1.5e-13    3e-15
   2      0.0016   0.3500000      0.0016   35   26   26  1.5e-13  2.9e-15
   3       0.001   0.3500000       0.001   36   27   27    7e-14  2.9e-15
Velocity u2, Pressure p
Iter      SolEst     Damping    Stepsize #Res #Jac #Sol   LinErr   LinRes
   1      0.0021   0.5000000      0.0021   18    9    9  3.3e-15  7.8e-15
Displacement field
Iter      SolEst     Damping    Stepsize #Res #Jac #Sol   LinErr   LinRes
   1       0.017   0.5000000       0.017   18    9    9  9.1e-14  1.2e-12
Spatial mesh displacement
Iter      SolEst     Damping    Stepsize #Res #Jac #Sol   LinErr   LinRes
   1     1.4e-07   0.5000000     1.4e-07   18    9    9  1.7e-15  1.8e-15
```

```
Solution error estimates for segregated groups
0.0018, 0.0011, 0.0085, 7e-08
Residual error estimates for segregated groups
0.43, 1.7e+02, 1.4e+03, 0.13
Segregated solver iteration 10.
Merged variables
Iter      SolEst     Damping    Stepsize #Res #Jac #Sol   LinErr   LinRes
   1     0.00067   0.3500000     0.00067   38   28   28    7e-14  2.6e-15
   2     0.00044   0.3500000     0.00044   39   29   29  7.1e-14  2.9e-15
   3     0.00028   0.3500000     0.00028   40   30   30  4.7e-14  2.9e-15
Velocity u2, Pressure p
Iter      SolEst     Damping    Stepsize #Res #Jac #Sol   LinErr   LinRes
   1      0.0011   0.5000000      0.0011   20   10   10  2.2e-15    8e-15
Displacement field
Iter      SolEst     Damping    Stepsize #Res #Jac #Sol   LinErr   LinRes
   1      0.0094   0.5000000      0.0094   20   10   10  2.2e-13  1.2e-12
Spatial mesh displacement
Iter      SolEst     Damping    Stepsize #Res #Jac #Sol   LinErr   LinRes
   1     8.6e-08   0.5000000     8.6e-08   20   10   10  1.9e-15  1.8e-15
Solution error estimates for segregated groups
0.00049, 0.00053, 0.0047, 4.3e-08
Residual error estimates for segregated groups
0.14, 86, 1.2e+03, 0.13
Segregated solver iteration 11.
Merged variables
Iter      SolEst     Damping    Stepsize #Res #Jac #Sol   LinErr   LinRes
   1     0.00018   0.3500000     0.00018   42   31   31  7.5e-14  2.7e-15
   2     0.00012   0.3500000     0.00012   43   32   32  5.1e-14    3e-15
   3     7.8e-05   0.3500000     7.8e-05   44   33   33  9.4e-14  2.8e-15
Velocity u2, Pressure p
Iter      SolEst     Damping    Stepsize #Res #Jac #Sol   LinErr   LinRes
   1     0.00053   0.5000000     0.00053   22   11   11  2.1e-15    8e-15
Displacement field
Iter      SolEst     Damping    Stepsize #Res #Jac #Sol   LinErr   LinRes
   1      0.0051   0.5000000      0.0051   22   11   11  1.2e-13  1.2e-12
Spatial mesh displacement
Iter      SolEst     Damping    Stepsize #Res #Jac #Sol   LinErr   LinRes
   1     5.1e-08   0.5000000     5.1e-08   22   11   11  1.4e-15  1.8e-15
Solution error estimates for segregated groups
0.00013, 0.00026, 0.0026, 2.6e-08
Residual error estimates for segregated groups
0.043, 43, 1.2e+03, 0.12
Segregated solver iteration 12.
Merged variables
Iter      SolEst     Damping    Stepsize #Res #Jac #Sol   LinErr   LinRes
   1     5.1e-05   0.3500000     5.1e-05   46   34   34  7.5e-14    3e-15
   2     3.3e-05   0.3500000     3.3e-05   47   35   35  1.4e-13  3.1e-15
   3     2.1e-05   0.3500000     2.1e-05   48   36   36  6.6e-14  2.8e-15
Velocity u2, Pressure p
Iter      SolEst     Damping    Stepsize #Res #Jac #Sol   LinErr   LinRes
   1     0.00026   0.5000000     0.00026   24   12   12    3e-15  7.9e-15
Displacement field
Iter      SolEst     Damping    Stepsize #Res #Jac #Sol   LinErr   LinRes
   1      0.0028   0.5000000      0.0028   24   12   12  1.3e-13  1.2e-12
Spatial mesh displacement
Iter      SolEst     Damping    Stepsize #Res #Jac #Sol   LinErr   LinRes
```

```
   1       3e-08   0.5000000       3e-08   24   12   12  1.3e-15  1.8e-15
Solution error estimates for segregated groups
3.7e-05, 0.00013, 0.0014, 1.5e-08
Residual error estimates for segregated groups
0.013, 21, 1.2e+03, 0.13
Segregated solver iteration 13.
Merged variables
Iter      SolEst      Damping    Stepsize #Res #Jac #Sol   LinErr   LinRes
   1     1.4e-05   0.3500000     1.4e-05   50   37   37  7.5e-14  2.9e-15
   2       9e-06   0.3500000       9e-06   51   38   38  6.9e-14  2.9e-15
   3     5.9e-06   0.3500000     5.9e-06   52   39   39  7.5e-14  2.9e-15
Velocity u2, Pressure p
Iter      SolEst      Damping    Stepsize #Res #Jac #Sol   LinErr   LinRes
   1     0.00013   0.5000000     0.00013   26   13   13  3.1e-15  7.9e-15
Displacement field
Iter      SolEst      Damping    Stepsize #Res #Jac #Sol   LinErr   LinRes
   1      0.0015   0.5000000      0.0015   26   13   13  1.1e-13  1.2e-12
Spatial mesh displacement
Iter      SolEst      Damping    Stepsize #Res #Jac #Sol   LinErr   LinRes
   1     1.8e-08   0.5000000     1.8e-08   26   13   13  1.5e-15  1.9e-15
Solution error estimates for segregated groups
1e-05, 6.6e-05, 0.00076, 8.9e-09
Residual error estimates for segregated groups
0.004, 11, 1.2e+03, 0.14
Solution time: 155 s. (2 minutes, 35 seconds)
Physical memory: 4.76 GB
Virtual memory: 25.54 GB
Ended at Jan 31, 2025, 9:04:04 PM.
----- Stationary Solver 1 in study1/Approach Curve Solver (sol1) -------------->
```

d=2040 (su94)

GENERAL

Description	Value
Solution	d=2040

LOG

```
<---- Compile Equations: Stationary in study1/Approach Curve Solver (sol1) -----
Started at Jan 31, 2025, 9:04:05 PM.
Parameter d = 2.04E-6 (m).
Geometry shape function: Linear serendipity
Number of vertex elements: 18
Number of boundary elements: 6086
Number of elements: 153772
Minimum element quality: 0.4757
Time: 8 s.
Physical memory: 3.02 GB
Virtual memory: 15.26 GB
Ended at Jan 31, 2025, 9:04:13 PM.
----- Compile Equations: Stationary in study1/Approach Curve Solver (sol1) ---->

<---- Dependent Variables 1 in study1/Approach Curve Solver (sol1) -------------
Started at Jan 31, 2025, 9:04:13 PM.
Solution time: 0 s.
Physical memory: 3.02 GB
Virtual memory: 15.26 GB
Ended at Jan 31, 2025, 9:04:13 PM.
----- Dependent Variables 1 in study1/Approach Curve Solver (sol1) ------------>

<---- Stationary Solver 1 in study1/Approach Curve Solver (sol1) ---------------
Started at Jan 31, 2025, 9:04:13 PM.
Segregated solver
Segregated Step 11 does not have any components solved for.
Number of degrees of freedom solved for: 780134 (plus 11595 internal DOFs).
Segregated solver iteration 1.
Merged variables
Nonsymmetric matrix found.
Scales for dependent variables:
Concentration (comp1.Cl): 1.4e+02
Concentration (comp1.K): 1.4e+02
Electric potential (comp1.V): 1.9e+02
Orthonormal null-space function used.
Iter      SolEst     Damping    Stepsize #Res #Jac #Sol   LinErr   LinRes
   1        0.83   0.3500000        0.83    2    1    1  2.6e-11  2.7e-15
   2        0.81   0.3500000        0.81    3    2    2  4.6e-11  2.7e-15
   3         0.8   0.3500000         0.8    4    3    3  4.2e-11  2.9e-15
Velocity u2, Pressure p
Nonsymmetric matrix found.
Scales for dependent variables:
Pressure (comp1.p): 1
Velocity field (spatial frame) (comp1.u2): 0.00018
Orthonormal null-space function used.
Iter      SolEst     Damping    Stepsize #Res #Jac #Sol   LinErr   LinRes
   1         1.1   0.5000000         1.1    2    1    1  1.7e-15  2.3e-15
Displacement field
Nonsymmetric matrix found.
Scales for dependent variables:
Auxiliary pressure (comp1.solid.lemm1.pw): 1
Displacement field (comp1.u): 2.8e-18
Orthonormal null-space function used.
Iter      SolEst     Damping    Stepsize #Res #Jac #Sol   LinErr   LinRes
   1         1.9   0.5000000         1.9    2    1    1  7.3e-14  1.4e-12
Spatial mesh displacement
```

```
Symmetric matrices found.
Scales for dependent variables:
Spatial mesh displacement (comp1.spatial.disp): 1.2e-09
Orthonormal null-space function used.
Iter      SolEst     Damping    Stepsize #Res #Jac #Sol   LinErr   LinRes
   1     8.1e-07   0.5000000     8.1e-07    2    1    1  1.6e-15  1.8e-15
Solution error estimates for segregated groups
1.4, 0.54, 0.95, 4e-07
Residual error estimates for segregated groups
7.8e+03, 8.2e+04, 2.1e+04, 0.11
Segregated solver iteration 2.
Merged variables
Scales for dependent variables:
Concentration (comp1.Cl): 1.4e+02
Concentration (comp1.K): 1.4e+02
Electric potential (comp1.V): 0.064
Iter      SolEst     Damping    Stepsize #Res #Jac #Sol   LinErr   LinRes
   1        0.78   0.3500000        0.78    6    4    4  5.1e-14  3.2e-15
   2        0.77   0.3500000        0.77    7    5    5    1e-13    4e-15
   3        0.76   0.3500000        0.76    8    6    6  1.6e-13  3.2e-15
Velocity u2, Pressure p
Scales for dependent variables:
Pressure (comp1.p): 3.7e+02
Velocity field (spatial frame) (comp1.u2): 0.00073
Iter      SolEst     Damping    Stepsize #Res #Jac #Sol   LinErr   LinRes
   1        0.36   0.5000000        0.36    4    2    2  2.2e-15  7.8e-15
Displacement field
Iter      SolEst     Damping    Stepsize #Res #Jac #Sol   LinErr   LinRes
   1        0.95   0.5000000        0.95    4    2    2  4.2e-14  1.4e-12
Spatial mesh displacement
Iter      SolEst     Damping    Stepsize #Res #Jac #Sol   LinErr   LinRes
   1     1.2e-06   0.5000000     1.2e-06    4    2    2  1.3e-15  1.9e-15
Solution error estimates for segregated groups
1.3, 0.18, 0.48, 6e-07
Residual error estimates for segregated groups
7.6e+02, 1.6e+04, 2.1e+04, 0.12
Segregated solver iteration 3.
Merged variables
Iter      SolEst     Damping    Stepsize #Res #Jac #Sol   LinErr   LinRes
   1        0.74   0.3500000        0.74   10    7    7  8.5e-14  3.7e-15
   2        0.73   0.3500000        0.73   11    8    8  8.7e-14  3.6e-15
   3        0.71   0.3500000        0.71   12    9    9  1.2e-13  2.9e-15
Velocity u2, Pressure p
Iter      SolEst     Damping    Stepsize #Res #Jac #Sol   LinErr   LinRes
   1        0.15   0.5000000        0.15    6    3    3  2.9e-15  7.8e-15
Displacement field
Iter      SolEst     Damping    Stepsize #Res #Jac #Sol   LinErr   LinRes
   1        0.52   0.5000000        0.52    6    3    3  1.4e-13  1.5e-12
Spatial mesh displacement
Iter      SolEst     Damping    Stepsize #Res #Jac #Sol   LinErr   LinRes
   1     1.2e-06   0.5000000     1.2e-06    6    3    3  1.6e-15  1.9e-15
Solution error estimates for segregated groups
1.2, 0.077, 0.26, 6e-07
Residual error estimates for segregated groups
2.1e+02, 9.3e+03, 1.6e+04, 0.13
Segregated solver iteration 4.
```

```
Merged variables
Iter      SolEst     Damping    Stepsize #Res #Jac #Sol   LinErr   LinRes
   1         0.7   0.3500000         0.7   14   10   10  1.1e-13  3.8e-15
   2        0.67   0.3500000        0.67   15   11   11  1.9e-13  3.7e-15
   3        0.58   0.3500000        0.58   16   12   12  5.2e-14  2.9e-15
Velocity u2, Pressure p
Iter      SolEst     Damping    Stepsize #Res #Jac #Sol   LinErr   LinRes
   1       0.072   0.5000000       0.072    8    4    4  2.9e-15  7.9e-15
Displacement field
Iter      SolEst     Damping    Stepsize #Res #Jac #Sol   LinErr   LinRes
   1        0.29   0.5000000        0.29    8    4    4  4.9e-14  1.5e-12
Spatial mesh displacement
Iter      SolEst     Damping    Stepsize #Res #Jac #Sol   LinErr   LinRes
   1       1e-06   0.5000000       1e-06    8    4    4  1.9e-15  1.8e-15
Solution error estimates for segregated groups
1, 0.036, 0.15, 5e-07
Residual error estimates for segregated groups
82, 4.9e+03, 1.1e+04, 0.14
Segregated solver iteration 5.
Merged variables
Iter      SolEst     Damping    Stepsize #Res #Jac #Sol   LinErr   LinRes
   1         0.4   0.3500000         0.4   18   13   13  9.1e-14  4.6e-15
   2        0.27   0.3500000        0.27   19   14   14  6.7e-14  3.2e-15
   3        0.18   0.3500000        0.18   20   15   15  1.2e-13  2.9e-15
Velocity u2, Pressure p
Iter      SolEst     Damping    Stepsize #Res #Jac #Sol   LinErr   LinRes
   1       0.035   0.5000000       0.035   10    5    5  2.6e-15  7.9e-15
Displacement field
Iter      SolEst     Damping    Stepsize #Res #Jac #Sol   LinErr   LinRes
   1        0.17   0.5000000        0.17   10    5    5  6.5e-14  1.5e-12
Spatial mesh displacement
Iter      SolEst     Damping    Stepsize #Res #Jac #Sol   LinErr   LinRes
   1     7.6e-07   0.5000000     7.6e-07   10    5    5  1.7e-15  1.9e-15
Solution error estimates for segregated groups
0.3, 0.017, 0.084, 3.8e-07
Residual error estimates for segregated groups
32, 2.5e+03, 6.8e+03, 0.13
Segregated solver iteration 6.
Merged variables
Iter      SolEst     Damping    Stepsize #Res #Jac #Sol   LinErr   LinRes
   1        0.12   0.3500000        0.12   22   16   16  6.2e-14  3.8e-15
   2       0.076   0.3500000       0.076   23   17   17  6.5e-14  3.1e-15
   3        0.05   0.3500000        0.05   24   18   18  1.3e-13  3.4e-15
Velocity u2, Pressure p
Iter      SolEst     Damping    Stepsize #Res #Jac #Sol   LinErr   LinRes
   1       0.017   0.5000000       0.017   12    6    6  4.4e-15  7.8e-15
Displacement field
Scales for dependent variables:
Auxiliary pressure (comp1.solid.lemm1.pw): 0.37
Displacement field (comp1.u): 3e-16
Iter      SolEst     Damping    Stepsize #Res #Jac #Sol   LinErr   LinRes
   1       0.096   0.5000000       0.096   12    6    6  1.6e-13  1.2e-12
Spatial mesh displacement
Iter      SolEst     Damping    Stepsize #Res #Jac #Sol   LinErr   LinRes
   1     5.3e-07   0.5000000     5.3e-07   12    6    6  1.6e-15  1.9e-15
Solution error estimates for segregated groups
```

```
0.085, 0.0085, 0.048, 2.6e-07
Residual error estimates for segregated groups
12, 1.3e+03, 4.2e+03, 0.13
Segregated solver iteration 7.
Merged variables
Iter      SolEst     Damping    Stepsize #Res #Jac #Sol   LinErr   LinRes
   1       0.032   0.3500000       0.032   26   19   19  1.3e-13  3.5e-15
   2       0.021   0.3500000       0.021   27   20   20  8.5e-14  6.6e-15
   3       0.014   0.3500000       0.014   28   21   21  8.2e-14  3.3e-15
Velocity u2, Pressure p
Iter      SolEst     Damping    Stepsize #Res #Jac #Sol   LinErr   LinRes
   1      0.0085   0.5000000      0.0085   14    7    7  1.7e-15  7.8e-15
Displacement field
Iter      SolEst     Damping    Stepsize #Res #Jac #Sol   LinErr   LinRes
   1       0.054   0.5000000       0.054   14    7    7  1.1e-13  1.2e-12
Spatial mesh displacement
Iter      SolEst     Damping    Stepsize #Res #Jac #Sol   LinErr   LinRes
   1     3.5e-07   0.5000000     3.5e-07   14    7    7  1.8e-15  1.9e-15
Solution error estimates for segregated groups
0.024, 0.0042, 0.027, 1.8e-07
Residual error estimates for segregated groups
4.1, 6.5e+02, 2.6e+03, 0.13
Segregated solver iteration 8.
Merged variables
Iter      SolEst     Damping    Stepsize #Res #Jac #Sol   LinErr   LinRes
   1      0.0089   0.3500000      0.0089   30   22   22    1e-13  3.1e-15
   2      0.0058   0.3500000      0.0058   31   23   23  1.9e-13  5.1e-15
   3      0.0038   0.3500000      0.0038   32   24   24  5.2e-14  4.3e-15
Velocity u2, Pressure p
Iter      SolEst     Damping    Stepsize #Res #Jac #Sol   LinErr   LinRes
   1      0.0042   0.5000000      0.0042   16    8    8  4.2e-15  7.9e-15
Displacement field
Iter      SolEst     Damping    Stepsize #Res #Jac #Sol   LinErr   LinRes
   1        0.03   0.5000000        0.03   16    8    8  2.3e-13  1.2e-12
Spatial mesh displacement
Iter      SolEst     Damping    Stepsize #Res #Jac #Sol   LinErr   LinRes
   1     2.3e-07   0.5000000     2.3e-07   16    8    8  1.5e-15  1.8e-15
Solution error estimates for segregated groups
0.0065, 0.0021, 0.015, 1.1e-07
Residual error estimates for segregated groups
1.4, 3.3e+02, 1.8e+03, 0.13
Segregated solver iteration 9.
Merged variables
Iter      SolEst     Damping    Stepsize #Res #Jac #Sol   LinErr   LinRes
   1      0.0025   0.3500000      0.0025   34   25   25  1.2e-13  3.6e-15
   2      0.0016   0.3500000      0.0016   35   26   26  1.1e-13  3.2e-15
   3       0.001   0.3500000       0.001   36   27   27  1.5e-13  3.7e-15
Velocity u2, Pressure p
Iter      SolEst     Damping    Stepsize #Res #Jac #Sol   LinErr   LinRes
   1      0.0021   0.5000000      0.0021   18    9    9  2.5e-15    8e-15
Displacement field
Iter      SolEst     Damping    Stepsize #Res #Jac #Sol   LinErr   LinRes
   1       0.017   0.5000000       0.017   18    9    9    1e-13  1.2e-12
Spatial mesh displacement
Iter      SolEst     Damping    Stepsize #Res #Jac #Sol   LinErr   LinRes
   1     1.4e-07   0.5000000     1.4e-07   18    9    9  2.3e-15  1.8e-15
```

```
Solution error estimates for segregated groups
0.0018, 0.0011, 0.0085, 7.1e-08
Residual error estimates for segregated groups
0.44, 1.6e+02, 1.4e+03, 0.13
Segregated solver iteration 10.
Merged variables
Iter      SolEst     Damping    Stepsize #Res #Jac #Sol   LinErr   LinRes
   1     0.00067   0.3500000     0.00067   38   28   28  1.6e-13  3.7e-15
   2     0.00044   0.3500000     0.00044   39   29   29    1e-13  3.4e-15
   3     0.00028   0.3500000     0.00028   40   30   30  9.1e-14  4.3e-15
Velocity u2, Pressure p
Iter      SolEst     Damping    Stepsize #Res #Jac #Sol   LinErr   LinRes
   1      0.0011   0.5000000      0.0011   20   10   10  3.4e-15  7.9e-15
Displacement field
Iter      SolEst     Damping    Stepsize #Res #Jac #Sol   LinErr   LinRes
   1      0.0094   0.5000000      0.0094   20   10   10  1.2e-13  1.2e-12
Spatial mesh displacement
Iter      SolEst     Damping    Stepsize #Res #Jac #Sol   LinErr   LinRes
   1     8.7e-08   0.5000000     8.7e-08   20   10   10  1.4e-15  1.9e-15
Solution error estimates for segregated groups
0.00049, 0.00053, 0.0047, 4.3e-08
Residual error estimates for segregated groups
0.14, 82, 1.2e+03, 0.12
Segregated solver iteration 11.
Merged variables
Iter      SolEst     Damping    Stepsize #Res #Jac #Sol   LinErr   LinRes
   1     0.00018   0.3500000     0.00018   42   31   31  1.2e-13  3.5e-15
   2     0.00012   0.3500000     0.00012   43   32   32  5.3e-14  3.2e-15
   3     7.8e-05   0.3500000     7.8e-05   44   33   33  1.7e-13  3.2e-15
Velocity u2, Pressure p
Iter      SolEst     Damping    Stepsize #Res #Jac #Sol   LinErr   LinRes
   1     0.00053   0.5000000     0.00053   22   11   11    3e-15  7.8e-15
Displacement field
Iter      SolEst     Damping    Stepsize #Res #Jac #Sol   LinErr   LinRes
   1      0.0051   0.5000000      0.0051   22   11   11  1.4e-13  1.2e-12
Spatial mesh displacement
Iter      SolEst     Damping    Stepsize #Res #Jac #Sol   LinErr   LinRes
   1     5.2e-08   0.5000000     5.2e-08   22   11   11  1.6e-15  1.8e-15
Solution error estimates for segregated groups
0.00013, 0.00026, 0.0026, 2.6e-08
Residual error estimates for segregated groups
0.043, 41, 1.2e+03, 0.13
Segregated solver iteration 12.
Merged variables
Iter      SolEst     Damping    Stepsize #Res #Jac #Sol   LinErr   LinRes
   1     5.1e-05   0.3500000     5.1e-05   46   34   34  8.7e-14  3.5e-15
   2     3.3e-05   0.3500000     3.3e-05   47   35   35  5.5e-14    3e-15
   3     2.1e-05   0.3500000     2.1e-05   48   36   36  9.9e-14  4.1e-15
Velocity u2, Pressure p
Iter      SolEst     Damping    Stepsize #Res #Jac #Sol   LinErr   LinRes
   1     0.00026   0.5000000     0.00026   24   12   12  2.4e-15    8e-15
Displacement field
Iter      SolEst     Damping    Stepsize #Res #Jac #Sol   LinErr   LinRes
   1      0.0028   0.5000000      0.0028   24   12   12  1.3e-13  1.2e-12
Spatial mesh displacement
Iter      SolEst     Damping    Stepsize #Res #Jac #Sol   LinErr   LinRes
```

```
   1     3.1e-08   0.5000000     3.1e-08   24   12   12  1.4e-15  1.8e-15
Solution error estimates for segregated groups
3.7e-05, 0.00013, 0.0014, 1.5e-08
Residual error estimates for segregated groups
0.013, 20, 1.2e+03, 0.13
Segregated solver iteration 13.
Merged variables
Iter      SolEst     Damping    Stepsize #Res #Jac #Sol   LinErr   LinRes
   1     1.4e-05   0.3500000     1.4e-05   50   37   37  8.8e-14  3.4e-15
   2     9.1e-06   0.3500000     9.1e-06   51   38   38  1.3e-13  4.3e-15
   3     5.9e-06   0.3500000     5.9e-06   52   39   39  5.7e-14  3.1e-15
Velocity u2, Pressure p
Iter      SolEst     Damping    Stepsize #Res #Jac #Sol   LinErr   LinRes
   1     0.00013   0.5000000     0.00013   26   13   13  3.1e-15  7.8e-15
Displacement field
Iter      SolEst     Damping    Stepsize #Res #Jac #Sol   LinErr   LinRes
   1      0.0015   0.5000000      0.0015   26   13   13  1.1e-13  1.2e-12
Spatial mesh displacement
Iter      SolEst     Damping    Stepsize #Res #Jac #Sol   LinErr   LinRes
   1     1.8e-08   0.5000000     1.8e-08   26   13   13  1.4e-15  1.9e-15
Solution error estimates for segregated groups
1e-05, 6.6e-05, 0.00076, 9e-09
Residual error estimates for segregated groups
0.004, 10, 1.2e+03, 0.14
Solution time: 153 s. (2 minutes, 33 seconds)
Physical memory: 4.72 GB
Virtual memory: 25.56 GB
Ended at Jan 31, 2025, 9:06:46 PM.
----- Stationary Solver 1 in study1/Approach Curve Solver (sol1) -------------->
```

d=2035 (su95)

GENERAL

Description	Value
Solution	d=2035

LOG

```
<---- Compile Equations: Stationary in study1/Approach Curve Solver (sol1) -----
Started at Jan 31, 2025, 9:06:46 PM.
Parameter d = 2.035E-6 (m).
Geometry shape function: Linear serendipity
Number of vertex elements: 18
Number of boundary elements: 6084
Number of elements: 153784
Minimum element quality: 0.486
Time: 8 s.
Physical memory: 3.04 GB
Virtual memory: 15.29 GB
Ended at Jan 31, 2025, 9:06:54 PM.
----- Compile Equations: Stationary in study1/Approach Curve Solver (sol1) ---->

<---- Dependent Variables 1 in study1/Approach Curve Solver (sol1) -------------
Started at Jan 31, 2025, 9:06:54 PM.
Solution time: 0 s.
Physical memory: 3.04 GB
Virtual memory: 15.29 GB
Ended at Jan 31, 2025, 9:06:54 PM.
----- Dependent Variables 1 in study1/Approach Curve Solver (sol1) ------------>

<---- Stationary Solver 1 in study1/Approach Curve Solver (sol1) ---------------
Started at Jan 31, 2025, 9:06:55 PM.
Segregated solver
Segregated Step 11 does not have any components solved for.
Number of degrees of freedom solved for: 780281 (plus 11591 internal DOFs).
Segregated solver iteration 1.
Merged variables
Nonsymmetric matrix found.
Scales for dependent variables:
Concentration (comp1.Cl): 1.4e+02
Concentration (comp1.K): 1.4e+02
Electric potential (comp1.V): 1.9e+02
Orthonormal null-space function used.
Iter      SolEst     Damping    Stepsize #Res #Jac #Sol   LinErr   LinRes
   1        0.83   0.3500000        0.83    2    1    1    2e-11  2.7e-15
   2        0.81   0.3500000        0.81    3    2    2  3.6e-11  2.7e-15
   3         0.8   0.3500000         0.8    4    3    3  1.8e-11  2.7e-15
Velocity u2, Pressure p
Nonsymmetric matrix found.
Scales for dependent variables:
Pressure (comp1.p): 1
Velocity field (spatial frame) (comp1.u2): 0.00018
Orthonormal null-space function used.
Iter      SolEst     Damping    Stepsize #Res #Jac #Sol   LinErr   LinRes
   1         1.1   0.5000000         1.1    2    1    1  1.6e-15  2.3e-15
Displacement field
Nonsymmetric matrix found.
Scales for dependent variables:
Auxiliary pressure (comp1.solid.lemm1.pw): 1
Displacement field (comp1.u): 2.8e-18
Orthonormal null-space function used.
Iter      SolEst     Damping    Stepsize #Res #Jac #Sol   LinErr   LinRes
   1         1.9   0.5000000         1.9    2    1    1  1.6e-13  1.5e-12
Spatial mesh displacement
```

```
Symmetric matrices found.
Scales for dependent variables:
Spatial mesh displacement (comp1.spatial.disp): 1.2e-09
Orthonormal null-space function used.
Iter      SolEst     Damping    Stepsize #Res #Jac #Sol   LinErr   LinRes
   1       8e-07   0.5000000       8e-07    2    1    1  1.6e-15  1.8e-15
Solution error estimates for segregated groups
1.4, 0.54, 0.95, 4e-07
Residual error estimates for segregated groups
7.8e+03, 8.2e+04, 2.1e+04, 0.12
Segregated solver iteration 2.
Merged variables
Scales for dependent variables:
Concentration (comp1.Cl): 1.4e+02
Concentration (comp1.K): 1.4e+02
Electric potential (comp1.V): 0.064
Iter      SolEst     Damping    Stepsize #Res #Jac #Sol   LinErr   LinRes
   1        0.78   0.3500000        0.78    6    4    4    4e-14  2.9e-15
   2        0.77   0.3500000        0.77    7    5    5  7.2e-14  2.8e-15
   3        0.76   0.3500000        0.76    8    6    6    8e-14  2.9e-15
Velocity u2, Pressure p
Scales for dependent variables:
Pressure (comp1.p): 3.7e+02
Velocity field (spatial frame) (comp1.u2): 0.00074
Iter      SolEst     Damping    Stepsize #Res #Jac #Sol   LinErr   LinRes
   1        0.36   0.5000000        0.36    4    2    2  2.3e-15  7.9e-15
Displacement field
Iter      SolEst     Damping    Stepsize #Res #Jac #Sol   LinErr   LinRes
   1        0.95   0.5000000        0.95    4    2    2  4.7e-14  1.5e-12
Spatial mesh displacement
Iter      SolEst     Damping    Stepsize #Res #Jac #Sol   LinErr   LinRes
   1     1.2e-06   0.5000000     1.2e-06    4    2    2  1.9e-15  1.8e-15
Solution error estimates for segregated groups
1.3, 0.18, 0.48, 6e-07
Residual error estimates for segregated groups
7.6e+02, 1.3e+04, 2.1e+04, 0.13
Segregated solver iteration 3.
Merged variables
Iter      SolEst     Damping    Stepsize #Res #Jac #Sol   LinErr   LinRes
   1        0.74   0.3500000        0.74   10    7    7  7.2e-14  2.7e-15
   2        0.73   0.3500000        0.73   11    8    8  5.6e-14  2.8e-15
   3        0.71   0.3500000        0.71   12    9    9  1.1e-13  2.9e-15
Velocity u2, Pressure p
Iter      SolEst     Damping    Stepsize #Res #Jac #Sol   LinErr   LinRes
   1        0.15   0.5000000        0.15    6    3    3  2.4e-15  7.8e-15
Displacement field
Iter      SolEst     Damping    Stepsize #Res #Jac #Sol   LinErr   LinRes
   1        0.52   0.5000000        0.52    6    3    3  8.7e-14  1.5e-12
Spatial mesh displacement
Iter      SolEst     Damping    Stepsize #Res #Jac #Sol   LinErr   LinRes
   1     1.2e-06   0.5000000     1.2e-06    6    3    3    2e-15  1.9e-15
Solution error estimates for segregated groups
1.2, 0.077, 0.26, 6e-07
Residual error estimates for segregated groups
2.1e+02, 7.3e+03, 1.6e+04, 0.13
Segregated solver iteration 4.
```

```
Merged variables
Iter      SolEst     Damping    Stepsize #Res #Jac #Sol   LinErr   LinRes
   1         0.7   0.3500000         0.7   14   10   10    1e-13  3.1e-15
   2        0.67   0.3500000        0.67   15   11   11  8.1e-14  2.9e-15
   3        0.58   0.3500000        0.58   16   12   12  9.1e-14  2.8e-15
Velocity u2, Pressure p
Iter      SolEst     Damping    Stepsize #Res #Jac #Sol   LinErr   LinRes
   1       0.072   0.5000000       0.072    8    4    4  3.3e-15    8e-15
Displacement field
Iter      SolEst     Damping    Stepsize #Res #Jac #Sol   LinErr   LinRes
   1        0.29   0.5000000        0.29    8    4    4  8.4e-14  1.4e-12
Spatial mesh displacement
Iter      SolEst     Damping    Stepsize #Res #Jac #Sol   LinErr   LinRes
   1       1e-06   0.5000000       1e-06    8    4    4  1.4e-15  1.8e-15
Solution error estimates for segregated groups
1, 0.036, 0.15, 5e-07
Residual error estimates for segregated groups
82, 3.8e+03, 1.1e+04, 0.13
Segregated solver iteration 5.
Merged variables
Iter      SolEst     Damping    Stepsize #Res #Jac #Sol   LinErr   LinRes
   1         0.4   0.3500000         0.4   18   13   13  1.3e-13  2.9e-15
   2        0.27   0.3500000        0.27   19   14   14  8.5e-14  2.8e-15
   3        0.18   0.3500000        0.18   20   15   15  8.5e-14  2.8e-15
Velocity u2, Pressure p
Iter      SolEst     Damping    Stepsize #Res #Jac #Sol   LinErr   LinRes
   1       0.035   0.5000000       0.035   10    5    5  2.7e-15  7.9e-15
Displacement field
Iter      SolEst     Damping    Stepsize #Res #Jac #Sol   LinErr   LinRes
   1        0.17   0.5000000        0.17   10    5    5  7.8e-14  1.5e-12
Spatial mesh displacement
Iter      SolEst     Damping    Stepsize #Res #Jac #Sol   LinErr   LinRes
   1     7.5e-07   0.5000000     7.5e-07   10    5    5  1.9e-15  1.8e-15
Solution error estimates for segregated groups
0.3, 0.017, 0.084, 3.8e-07
Residual error estimates for segregated groups
32, 2e+03, 6.8e+03, 0.13
Segregated solver iteration 6.
Merged variables
Iter      SolEst     Damping    Stepsize #Res #Jac #Sol   LinErr   LinRes
   1        0.12   0.3500000        0.12   22   16   16  5.1e-14  2.8e-15
   2       0.076   0.3500000       0.076   23   17   17  1.4e-13  2.8e-15
   3        0.05   0.3500000        0.05   24   18   18    8e-14  3.1e-15
Velocity u2, Pressure p
Iter      SolEst     Damping    Stepsize #Res #Jac #Sol   LinErr   LinRes
   1       0.017   0.5000000       0.017   12    6    6    3e-15  7.8e-15
Displacement field
Scales for dependent variables:
Auxiliary pressure (comp1.solid.lemm1.pw): 0.37
Displacement field (comp1.u): 3e-16
Iter      SolEst     Damping    Stepsize #Res #Jac #Sol   LinErr   LinRes
   1       0.095   0.5000000       0.095   12    6    6  1.2e-13  1.2e-12
Spatial mesh displacement
Iter      SolEst     Damping    Stepsize #Res #Jac #Sol   LinErr   LinRes
   1     5.3e-07   0.5000000     5.3e-07   12    6    6  1.9e-15  1.8e-15
Solution error estimates for segregated groups
```

```
0.085, 0.0086, 0.048, 2.6e-07
Residual error estimates for segregated groups
12, 1e+03, 4.2e+03, 0.12
Segregated solver iteration 7.
Merged variables
Iter      SolEst     Damping    Stepsize #Res #Jac #Sol   LinErr   LinRes
   1       0.032   0.3500000       0.032   26   19   19  5.5e-14  2.8e-15
   2       0.021   0.3500000       0.021   27   20   20  7.5e-14  2.7e-15
   3       0.014   0.3500000       0.014   28   21   21  1.6e-13  2.8e-15
Velocity u2, Pressure p
Iter      SolEst     Damping    Stepsize #Res #Jac #Sol   LinErr   LinRes
   1      0.0085   0.5000000      0.0085   14    7    7  4.5e-15  7.9e-15
Displacement field
Iter      SolEst     Damping    Stepsize #Res #Jac #Sol   LinErr   LinRes
   1       0.054   0.5000000       0.054   14    7    7    1e-13  1.1e-12
Spatial mesh displacement
Iter      SolEst     Damping    Stepsize #Res #Jac #Sol   LinErr   LinRes
   1     3.5e-07   0.5000000     3.5e-07   14    7    7  2.1e-15  1.8e-15
Solution error estimates for segregated groups
0.024, 0.0042, 0.027, 1.8e-07
Residual error estimates for segregated groups
4.1, 5e+02, 2.6e+03, 0.13
Segregated solver iteration 8.
Merged variables
Iter      SolEst     Damping    Stepsize #Res #Jac #Sol   LinErr   LinRes
   1      0.0089   0.3500000      0.0089   30   22   22  1.3e-13  3.1e-15
   2      0.0058   0.3500000      0.0058   31   23   23  1.1e-13  3.3e-15
   3      0.0038   0.3500000      0.0038   32   24   24  1.5e-13  2.9e-15
Velocity u2, Pressure p
Iter      SolEst     Damping    Stepsize #Res #Jac #Sol   LinErr   LinRes
   1      0.0042   0.5000000      0.0042   16    8    8  2.5e-15  7.9e-15
Displacement field
Iter      SolEst     Damping    Stepsize #Res #Jac #Sol   LinErr   LinRes
   1        0.03   0.5000000        0.03   16    8    8  1.7e-13  1.2e-12
Spatial mesh displacement
Iter      SolEst     Damping    Stepsize #Res #Jac #Sol   LinErr   LinRes
   1     2.3e-07   0.5000000     2.3e-07   16    8    8  1.2e-15  1.8e-15
Solution error estimates for segregated groups
0.0065, 0.0021, 0.015, 1.1e-07
Residual error estimates for segregated groups
1.3, 2.5e+02, 1.8e+03, 0.13
Segregated solver iteration 9.
Merged variables
Iter      SolEst     Damping    Stepsize #Res #Jac #Sol   LinErr   LinRes
   1      0.0025   0.3500000      0.0025   34   25   25  7.3e-14  2.9e-15
   2      0.0016   0.3500000      0.0016   35   26   26  8.9e-14  2.8e-15
   3       0.001   0.3500000       0.001   36   27   27  9.8e-14    3e-15
Velocity u2, Pressure p
Iter      SolEst     Damping    Stepsize #Res #Jac #Sol   LinErr   LinRes
   1      0.0021   0.5000000      0.0021   18    9    9    3e-15  7.9e-15
Displacement field
Iter      SolEst     Damping    Stepsize #Res #Jac #Sol   LinErr   LinRes
   1       0.017   0.5000000       0.017   18    9    9  1.2e-13  1.2e-12
Spatial mesh displacement
Iter      SolEst     Damping    Stepsize #Res #Jac #Sol   LinErr   LinRes
   1     1.4e-07   0.5000000     1.4e-07   18    9    9  1.6e-15  1.8e-15
```

```
Solution error estimates for segregated groups
0.0018, 0.0011, 0.0085, 7.1e-08
Residual error estimates for segregated groups
0.44, 1.3e+02, 1.4e+03, 0.13
Segregated solver iteration 10.
Merged variables
Iter      SolEst     Damping    Stepsize #Res #Jac #Sol   LinErr   LinRes
   1     0.00067   0.3500000     0.00067   38   28   28  8.7e-14  3.4e-15
   2     0.00044   0.3500000     0.00044   39   29   29  1.7e-13    3e-15
   3     0.00028   0.3500000     0.00028   40   30   30  6.5e-14  2.9e-15
Velocity u2, Pressure p
Iter      SolEst     Damping    Stepsize #Res #Jac #Sol   LinErr   LinRes
   1      0.0011   0.5000000      0.0011   20   10   10  2.9e-15  7.8e-15
Displacement field
Iter      SolEst     Damping    Stepsize #Res #Jac #Sol   LinErr   LinRes
   1      0.0094   0.5000000      0.0094   20   10   10  1.6e-13  1.2e-12
Spatial mesh displacement
Iter      SolEst     Damping    Stepsize #Res #Jac #Sol   LinErr   LinRes
   1     8.6e-08   0.5000000     8.6e-08   20   10   10  1.4e-15  1.9e-15
Solution error estimates for segregated groups
0.00049, 0.00053, 0.0047, 4.3e-08
Residual error estimates for segregated groups
0.14, 63, 1.3e+03, 0.13
Segregated solver iteration 11.
Merged variables
Iter      SolEst     Damping    Stepsize #Res #Jac #Sol   LinErr   LinRes
   1     0.00019   0.3500000     0.00019   42   31   31  1.4e-13  2.9e-15
   2     0.00012   0.3500000     0.00012   43   32   32  1.3e-13  3.2e-15
   3     7.8e-05   0.3500000     7.8e-05   44   33   33    5e-14  2.8e-15
Velocity u2, Pressure p
Iter      SolEst     Damping    Stepsize #Res #Jac #Sol   LinErr   LinRes
   1     0.00053   0.5000000     0.00053   22   11   11  2.5e-15  7.9e-15
Displacement field
Iter      SolEst     Damping    Stepsize #Res #Jac #Sol   LinErr   LinRes
   1      0.0051   0.5000000      0.0051   22   11   11  1.5e-13  1.2e-12
Spatial mesh displacement
Iter      SolEst     Damping    Stepsize #Res #Jac #Sol   LinErr   LinRes
   1     5.2e-08   0.5000000     5.2e-08   22   11   11  1.5e-15  1.8e-15
Solution error estimates for segregated groups
0.00013, 0.00026, 0.0026, 2.6e-08
Residual error estimates for segregated groups
0.043, 31, 1.2e+03, 0.13
Segregated solver iteration 12.
Merged variables
Iter      SolEst     Damping    Stepsize #Res #Jac #Sol   LinErr   LinRes
   1     5.1e-05   0.3500000     5.1e-05   46   34   34  1.2e-13  3.3e-15
   2     3.3e-05   0.3500000     3.3e-05   47   35   35    1e-13    3e-15
   3     2.1e-05   0.3500000     2.1e-05   48   36   36  1.3e-13  3.1e-15
Velocity u2, Pressure p
Iter      SolEst     Damping    Stepsize #Res #Jac #Sol   LinErr   LinRes
   1     0.00026   0.5000000     0.00026   24   12   12  2.7e-15  7.8e-15
Displacement field
Iter      SolEst     Damping    Stepsize #Res #Jac #Sol   LinErr   LinRes
   1      0.0028   0.5000000      0.0028   24   12   12  2.2e-13  1.2e-12
Spatial mesh displacement
Iter      SolEst     Damping    Stepsize #Res #Jac #Sol   LinErr   LinRes
```

```
   1     3.1e-08   0.5000000     3.1e-08   24   12   12  1.2e-15  1.8e-15
Solution error estimates for segregated groups
3.7e-05, 0.00013, 0.0014, 1.5e-08
Residual error estimates for segregated groups
0.013, 16, 1.2e+03, 0.13
Segregated solver iteration 13.
Merged variables
Iter      SolEst     Damping    Stepsize #Res #Jac #Sol   LinErr   LinRes
   1     1.4e-05   0.3500000     1.4e-05   50   37   37  9.1e-14  2.9e-15
   2     9.1e-06   0.3500000     9.1e-06   51   38   38  1.6e-13  3.3e-15
   3     5.9e-06   0.3500000     5.9e-06   52   39   39  6.6e-14  3.1e-15
Velocity u2, Pressure p
Iter      SolEst     Damping    Stepsize #Res #Jac #Sol   LinErr   LinRes
   1     0.00013   0.5000000     0.00013   26   13   13  2.9e-15  7.9e-15
Displacement field
Iter      SolEst     Damping    Stepsize #Res #Jac #Sol   LinErr   LinRes
   1      0.0015   0.5000000      0.0015   26   13   13  1.2e-13  1.2e-12
Spatial mesh displacement
Iter      SolEst     Damping    Stepsize #Res #Jac #Sol   LinErr   LinRes
   1     1.8e-08   0.5000000     1.8e-08   26   13   13  1.5e-15  1.8e-15
Solution error estimates for segregated groups
1e-05, 6.6e-05, 0.00076, 8.9e-09
Residual error estimates for segregated groups
0.004, 7.9, 1.2e+03, 0.12
Solution time: 157 s. (2 minutes, 37 seconds)
Physical memory: 4.78 GB
Virtual memory: 25.59 GB
Ended at Jan 31, 2025, 9:09:31 PM.
----- Stationary Solver 1 in study1/Approach Curve Solver (sol1) -------------->
```

d=2030 (su96)

GENERAL

Description	Value
Solution	d=2030

LOG

```
<---- Compile Equations: Stationary in study1/Approach Curve Solver (sol1) -----
Started at Jan 31, 2025, 9:09:32 PM.
Parameter d = 2.03E-6 (m).
Geometry shape function: Linear serendipity
Number of vertex elements: 18
Number of boundary elements: 6081
Number of elements: 153813
Minimum element quality: 0.4803
Time: 8 s.
Physical memory: 3.01 GB
Virtual memory: 15.26 GB
Ended at Jan 31, 2025, 9:09:40 PM.
----- Compile Equations: Stationary in study1/Approach Curve Solver (sol1) ---->

<---- Dependent Variables 1 in study1/Approach Curve Solver (sol1) -------------
Started at Jan 31, 2025, 9:09:40 PM.
Solution time: 0 s.
Physical memory: 3.01 GB
Virtual memory: 15.26 GB
Ended at Jan 31, 2025, 9:09:40 PM.
----- Dependent Variables 1 in study1/Approach Curve Solver (sol1) ------------>

<---- Stationary Solver 1 in study1/Approach Curve Solver (sol1) ---------------
Started at Jan 31, 2025, 9:09:40 PM.
Segregated solver
Segregated Step 11 does not have any components solved for.
Number of degrees of freedom solved for: 780319 (plus 11585 internal DOFs).
Segregated solver iteration 1.
Merged variables
Nonsymmetric matrix found.
Scales for dependent variables:
Concentration (comp1.Cl): 1.4e+02
Concentration (comp1.K): 1.4e+02
Electric potential (comp1.V): 1.9e+02
Orthonormal null-space function used.
Iter      SolEst     Damping    Stepsize #Res #Jac #Sol   LinErr   LinRes
   1        0.83   0.3500000        0.83    2    1    1  2.7e-11  2.8e-15
   2        0.81   0.3500000        0.81    3    2    2  2.4e-11  2.7e-15
   3         0.8   0.3500000         0.8    4    3    3    4e-11  2.7e-15
Velocity u2, Pressure p
Nonsymmetric matrix found.
Scales for dependent variables:
Pressure (comp1.p): 1
Velocity field (spatial frame) (comp1.u2): 0.00018
Orthonormal null-space function used.
Iter      SolEst     Damping    Stepsize #Res #Jac #Sol   LinErr   LinRes
   1         1.1   0.5000000         1.1    2    1    1    2e-15  2.2e-15
Displacement field
Nonsymmetric matrix found.
Scales for dependent variables:
Auxiliary pressure (comp1.solid.lemm1.pw): 1
Displacement field (comp1.u): 2.8e-18
Orthonormal null-space function used.
Iter      SolEst     Damping    Stepsize #Res #Jac #Sol   LinErr   LinRes
   1         1.9   0.5000000         1.9    2    1    1  6.7e-14  1.4e-12
Spatial mesh displacement
```

```
Symmetric matrices found.
Scales for dependent variables:
Spatial mesh displacement (comp1.spatial.disp): 1.2e-09
Orthonormal null-space function used.
Iter      SolEst     Damping    Stepsize #Res #Jac #Sol   LinErr   LinRes
   1     8.1e-07   0.5000000     8.1e-07    2    1    1  1.6e-15  1.8e-15
Solution error estimates for segregated groups
1.4, 0.54, 0.95, 4e-07
Residual error estimates for segregated groups
7.8e+03, 8.2e+04, 2.1e+04, 0.11
Segregated solver iteration 2.
Merged variables
Scales for dependent variables:
Concentration (comp1.Cl): 1.4e+02
Concentration (comp1.K): 1.4e+02
Electric potential (comp1.V): 0.064
Iter      SolEst     Damping    Stepsize #Res #Jac #Sol   LinErr   LinRes
   1        0.78   0.3500000        0.78    6    4    4  5.4e-14  2.7e-15
   2        0.77   0.3500000        0.77    7    5    5  7.9e-14  2.7e-15
   3        0.76   0.3500000        0.76    8    6    6  3.8e-14  2.7e-15
Velocity u2, Pressure p
Scales for dependent variables:
Pressure (comp1.p): 3.7e+02
Velocity field (spatial frame) (comp1.u2): 0.00073
Iter      SolEst     Damping    Stepsize #Res #Jac #Sol   LinErr   LinRes
   1        0.36   0.5000000        0.36    4    2    2  2.9e-15  7.9e-15
Displacement field
Iter      SolEst     Damping    Stepsize #Res #Jac #Sol   LinErr   LinRes
   1        0.96   0.5000000        0.96    4    2    2  1.1e-13  1.5e-12
Spatial mesh displacement
Iter      SolEst     Damping    Stepsize #Res #Jac #Sol   LinErr   LinRes
   1     1.2e-06   0.5000000     1.2e-06    4    2    2  1.5e-15  1.8e-15
Solution error estimates for segregated groups
1.3, 0.18, 0.48, 6e-07
Residual error estimates for segregated groups
7.6e+02, 1.4e+04, 2.1e+04, 0.12
Segregated solver iteration 3.
Merged variables
Iter      SolEst     Damping    Stepsize #Res #Jac #Sol   LinErr   LinRes
   1        0.74   0.3500000        0.74   10    7    7  9.1e-14  2.6e-15
   2        0.73   0.3500000        0.73   11    8    8  4.1e-14  2.7e-15
   3        0.71   0.3500000        0.71   12    9    9  1.1e-13  2.6e-15
Velocity u2, Pressure p
Iter      SolEst     Damping    Stepsize #Res #Jac #Sol   LinErr   LinRes
   1        0.15   0.5000000        0.15    6    3    3  3.8e-15  7.9e-15
Displacement field
Iter      SolEst     Damping    Stepsize #Res #Jac #Sol   LinErr   LinRes
   1        0.52   0.5000000        0.52    6    3    3  6.5e-14  1.5e-12
Spatial mesh displacement
Iter      SolEst     Damping    Stepsize #Res #Jac #Sol   LinErr   LinRes
   1     1.2e-06   0.5000000     1.2e-06    6    3    3  1.7e-15  1.9e-15
Solution error estimates for segregated groups
1.2, 0.077, 0.26, 6e-07
Residual error estimates for segregated groups
2.1e+02, 7.7e+03, 1.6e+04, 0.13
Segregated solver iteration 4.
```

```
Merged variables
Iter      SolEst     Damping    Stepsize #Res #Jac #Sol   LinErr   LinRes
   1         0.7   0.3500000         0.7   14   10   10  9.3e-14  2.8e-15
   2        0.67   0.3500000        0.67   15   11   11  5.3e-14  2.7e-15
   3        0.58   0.3500000        0.58   16   12   12  5.2e-14  2.7e-15
Velocity u2, Pressure p
Iter      SolEst     Damping    Stepsize #Res #Jac #Sol   LinErr   LinRes
   1       0.072   0.5000000       0.072    8    4    4    2e-15    8e-15
Displacement field
Iter      SolEst     Damping    Stepsize #Res #Jac #Sol   LinErr   LinRes
   1        0.29   0.5000000        0.29    8    4    4    4e-14  1.4e-12
Spatial mesh displacement
Iter      SolEst     Damping    Stepsize #Res #Jac #Sol   LinErr   LinRes
   1       1e-06   0.5000000       1e-06    8    4    4  1.6e-15  1.8e-15
Solution error estimates for segregated groups
1, 0.036, 0.15, 5e-07
Residual error estimates for segregated groups
81, 4.1e+03, 1.1e+04, 0.13
Segregated solver iteration 5.
Merged variables
Iter      SolEst     Damping    Stepsize #Res #Jac #Sol   LinErr   LinRes
   1         0.4   0.3500000         0.4   18   13   13  4.4e-14  2.6e-15
   2        0.27   0.3500000        0.27   19   14   14  5.8e-14    3e-15
   3        0.18   0.3500000        0.18   20   15   15  8.7e-14  2.6e-15
Velocity u2, Pressure p
Iter      SolEst     Damping    Stepsize #Res #Jac #Sol   LinErr   LinRes
   1       0.035   0.5000000       0.035   10    5    5  2.4e-15  7.8e-15
Displacement field
Iter      SolEst     Damping    Stepsize #Res #Jac #Sol   LinErr   LinRes
   1        0.17   0.5000000        0.17   10    5    5  7.3e-14  1.4e-12
Spatial mesh displacement
Iter      SolEst     Damping    Stepsize #Res #Jac #Sol   LinErr   LinRes
   1     7.6e-07   0.5000000     7.6e-07   10    5    5  1.9e-15  1.8e-15
Solution error estimates for segregated groups
0.3, 0.017, 0.084, 3.8e-07
Residual error estimates for segregated groups
32, 2.1e+03, 6.8e+03, 0.14
Segregated solver iteration 6.
Merged variables
Iter      SolEst     Damping    Stepsize #Res #Jac #Sol   LinErr   LinRes
   1        0.12   0.3500000        0.12   22   16   16  6.6e-14  2.7e-15
   2       0.076   0.3500000       0.076   23   17   17  8.8e-14  2.9e-15
   3        0.05   0.3500000        0.05   24   18   18  3.5e-14  2.7e-15
Velocity u2, Pressure p
Iter      SolEst     Damping    Stepsize #Res #Jac #Sol   LinErr   LinRes
   1       0.017   0.5000000       0.017   12    6    6  2.5e-15  7.9e-15
Displacement field
Scales for dependent variables:
Auxiliary pressure (comp1.solid.lemm1.pw): 0.37
Displacement field (comp1.u): 3e-16
Iter      SolEst     Damping    Stepsize #Res #Jac #Sol   LinErr   LinRes
   1       0.096   0.5000000       0.096   12    6    6  1.4e-13  1.2e-12
Spatial mesh displacement
Iter      SolEst     Damping    Stepsize #Res #Jac #Sol   LinErr   LinRes
   1     5.3e-07   0.5000000     5.3e-07   12    6    6  1.9e-15  1.8e-15
Solution error estimates for segregated groups
```

```
0.085, 0.0086, 0.048, 2.6e-07
Residual error estimates for segregated groups
12, 1.1e+03, 4.2e+03, 0.13
Segregated solver iteration 7.
Merged variables
Iter      SolEst     Damping    Stepsize #Res #Jac #Sol   LinErr   LinRes
   1       0.032   0.3500000       0.032   26   19   19  6.3e-14  2.7e-15
   2       0.021   0.3500000       0.021   27   20   20  6.7e-14  2.7e-15
   3       0.014   0.3500000       0.014   28   21   21  5.8e-14  2.7e-15
Velocity u2, Pressure p
Iter      SolEst     Damping    Stepsize #Res #Jac #Sol   LinErr   LinRes
   1      0.0085   0.5000000      0.0085   14    7    7  2.5e-15  7.9e-15
Displacement field
Iter      SolEst     Damping    Stepsize #Res #Jac #Sol   LinErr   LinRes
   1       0.054   0.5000000       0.054   14    7    7  1.6e-13  1.2e-12
Spatial mesh displacement
Iter      SolEst     Damping    Stepsize #Res #Jac #Sol   LinErr   LinRes
   1     3.5e-07   0.5000000     3.5e-07   14    7    7  1.4e-15  1.8e-15
Solution error estimates for segregated groups
0.024, 0.0042, 0.027, 1.8e-07
Residual error estimates for segregated groups
4.1, 5.3e+02, 2.6e+03, 0.14
Segregated solver iteration 8.
Merged variables
Iter      SolEst     Damping    Stepsize #Res #Jac #Sol   LinErr   LinRes
   1      0.0089   0.3500000      0.0089   30   22   22  6.9e-14  2.7e-15
   2      0.0058   0.3500000      0.0058   31   23   23  7.4e-14  2.7e-15
   3      0.0038   0.3500000      0.0038   32   24   24  4.5e-14  2.7e-15
Velocity u2, Pressure p
Iter      SolEst     Damping    Stepsize #Res #Jac #Sol   LinErr   LinRes
   1      0.0042   0.5000000      0.0042   16    8    8    3e-15  7.9e-15
Displacement field
Iter      SolEst     Damping    Stepsize #Res #Jac #Sol   LinErr   LinRes
   1        0.03   0.5000000        0.03   16    8    8  1.2e-13  1.2e-12
Spatial mesh displacement
Iter      SolEst     Damping    Stepsize #Res #Jac #Sol   LinErr   LinRes
   1     2.3e-07   0.5000000     2.3e-07   16    8    8  1.6e-15  1.8e-15
Solution error estimates for segregated groups
0.0065, 0.0021, 0.015, 1.1e-07
Residual error estimates for segregated groups
1.3, 2.7e+02, 1.8e+03, 0.13
Segregated solver iteration 9.
Merged variables
Iter      SolEst     Damping    Stepsize #Res #Jac #Sol   LinErr   LinRes
   1      0.0025   0.3500000      0.0025   34   25   25  9.2e-14  2.6e-15
   2      0.0016   0.3500000      0.0016   35   26   26  6.7e-14  2.7e-15
   3       0.001   0.3500000       0.001   36   27   27  5.9e-14  2.7e-15
Velocity u2, Pressure p
Iter      SolEst     Damping    Stepsize #Res #Jac #Sol   LinErr   LinRes
   1      0.0021   0.5000000      0.0021   18    9    9  2.5e-15  7.9e-15
Displacement field
Iter      SolEst     Damping    Stepsize #Res #Jac #Sol   LinErr   LinRes
   1       0.017   0.5000000       0.017   18    9    9    1e-13  1.1e-12
Spatial mesh displacement
Iter      SolEst     Damping    Stepsize #Res #Jac #Sol   LinErr   LinRes
   1     1.4e-07   0.5000000     1.4e-07   18    9    9  1.4e-15  1.8e-15
```

```
Solution error estimates for segregated groups
0.0018, 0.0011, 0.0085, 7.1e-08
Residual error estimates for segregated groups
0.44, 1.3e+02, 1.4e+03, 0.13
Segregated solver iteration 10.
Merged variables
Iter      SolEst     Damping    Stepsize #Res #Jac #Sol   LinErr   LinRes
   1     0.00067   0.3500000     0.00067   38   28   28  4.8e-14    3e-15
   2     0.00044   0.3500000     0.00044   39   29   29  6.3e-14  2.6e-15
   3     0.00028   0.3500000     0.00028   40   30   30  5.8e-14  2.6e-15
Velocity u2, Pressure p
Iter      SolEst     Damping    Stepsize #Res #Jac #Sol   LinErr   LinRes
   1      0.0011   0.5000000      0.0011   20   10   10  2.8e-15  7.9e-15
Displacement field
Iter      SolEst     Damping    Stepsize #Res #Jac #Sol   LinErr   LinRes
   1      0.0094   0.5000000      0.0094   20   10   10  1.5e-13  1.2e-12
Spatial mesh displacement
Iter      SolEst     Damping    Stepsize #Res #Jac #Sol   LinErr   LinRes
   1     8.7e-08   0.5000000     8.7e-08   20   10   10    2e-15  1.8e-15
Solution error estimates for segregated groups
0.00049, 0.00053, 0.0047, 4.3e-08
Residual error estimates for segregated groups
0.14, 67, 1.2e+03, 0.12
Segregated solver iteration 11.
Merged variables
Iter      SolEst     Damping    Stepsize #Res #Jac #Sol   LinErr   LinRes
   1     0.00018   0.3500000     0.00018   42   31   31  1.2e-13  2.7e-15
   2     0.00012   0.3500000     0.00012   43   32   32  1.1e-13  3.5e-15
   3     7.8e-05   0.3500000     7.8e-05   44   33   33  6.8e-14  2.6e-15
Velocity u2, Pressure p
Iter      SolEst     Damping    Stepsize #Res #Jac #Sol   LinErr   LinRes
   1     0.00053   0.5000000     0.00053   22   11   11  4.1e-15    8e-15
Displacement field
Iter      SolEst     Damping    Stepsize #Res #Jac #Sol   LinErr   LinRes
   1      0.0051   0.5000000      0.0051   22   11   11    1e-13  1.1e-12
Spatial mesh displacement
Iter      SolEst     Damping    Stepsize #Res #Jac #Sol   LinErr   LinRes
   1     5.2e-08   0.5000000     5.2e-08   22   11   11  1.4e-15  1.8e-15
Solution error estimates for segregated groups
0.00013, 0.00026, 0.0026, 2.6e-08
Residual error estimates for segregated groups
0.043, 34, 1.2e+03, 0.14
Segregated solver iteration 12.
Merged variables
Iter      SolEst     Damping    Stepsize #Res #Jac #Sol   LinErr   LinRes
   1     5.1e-05   0.3500000     5.1e-05   46   34   34  8.1e-14  3.1e-15
   2     3.3e-05   0.3500000     3.3e-05   47   35   35  8.2e-14  2.8e-15
   3     2.1e-05   0.3500000     2.1e-05   48   36   36  7.4e-14  2.6e-15
Velocity u2, Pressure p
Iter      SolEst     Damping    Stepsize #Res #Jac #Sol   LinErr   LinRes
   1     0.00026   0.5000000     0.00026   24   12   12  2.3e-15  7.8e-15
Displacement field
Iter      SolEst     Damping    Stepsize #Res #Jac #Sol   LinErr   LinRes
   1      0.0028   0.5000000      0.0028   24   12   12  1.3e-13  1.2e-12
Spatial mesh displacement
Iter      SolEst     Damping    Stepsize #Res #Jac #Sol   LinErr   LinRes
```

```
   1     3.1e-08   0.5000000     3.1e-08   24   12   12  1.8e-15  1.8e-15
Solution error estimates for segregated groups
3.7e-05, 0.00013, 0.0014, 1.5e-08
Residual error estimates for segregated groups
0.013, 17, 1.2e+03, 0.13
Segregated solver iteration 13.
Merged variables
Iter      SolEst     Damping    Stepsize #Res #Jac #Sol   LinErr   LinRes
   1     1.4e-05   0.3500000     1.4e-05   50   37   37  7.8e-14  2.6e-15
   2     9.1e-06   0.3500000     9.1e-06   51   38   38  9.6e-14  2.6e-15
   3     5.9e-06   0.3500000     5.9e-06   52   39   39  7.4e-14  2.7e-15
Velocity u2, Pressure p
Iter      SolEst     Damping    Stepsize #Res #Jac #Sol   LinErr   LinRes
   1     0.00013   0.5000000     0.00013   26   13   13  2.7e-15  7.9e-15
Displacement field
Iter      SolEst     Damping    Stepsize #Res #Jac #Sol   LinErr   LinRes
   1      0.0015   0.5000000      0.0015   26   13   13    1e-13  1.1e-12
Spatial mesh displacement
Iter      SolEst     Damping    Stepsize #Res #Jac #Sol   LinErr   LinRes
   1     1.8e-08   0.5000000     1.8e-08   26   13   13  1.9e-15  1.8e-15
Solution error estimates for segregated groups
1e-05, 6.6e-05, 0.00076, 9e-09
Residual error estimates for segregated groups
0.004, 8.4, 1.2e+03, 0.13
Solution time: 153 s. (2 minutes, 33 seconds)
Physical memory: 4.78 GB
Virtual memory: 25.58 GB
Ended at Jan 31, 2025, 9:12:13 PM.
----- Stationary Solver 1 in study1/Approach Curve Solver (sol1) -------------->
```

d=2025 (su97)

GENERAL

Description	Value
Solution	d=2025

LOG

```
<---- Compile Equations: Stationary in study1/Approach Curve Solver (sol1) -----
Started at Jan 31, 2025, 9:12:13 PM.
Parameter d = 2.025E-6 (m).
Geometry shape function: Linear serendipity
Number of vertex elements: 18
Number of boundary elements: 6079
Number of elements: 153963
Minimum element quality: 0.482
Time: 8 s.
Physical memory: 3.02 GB
Virtual memory: 15.26 GB
Ended at Jan 31, 2025, 9:12:21 PM.
----- Compile Equations: Stationary in study1/Approach Curve Solver (sol1) ---->

<---- Dependent Variables 1 in study1/Approach Curve Solver (sol1) -------------
Started at Jan 31, 2025, 9:12:21 PM.
Solution time: 0 s.
Physical memory: 3.02 GB
Virtual memory: 15.26 GB
Ended at Jan 31, 2025, 9:12:21 PM.
----- Dependent Variables 1 in study1/Approach Curve Solver (sol1) ------------>

<---- Stationary Solver 1 in study1/Approach Curve Solver (sol1) ---------------
Started at Jan 31, 2025, 9:12:21 PM.
Segregated solver
Segregated Step 11 does not have any components solved for.
Number of degrees of freedom solved for: 780995 (plus 11581 internal DOFs).
Segregated solver iteration 1.
Merged variables
Nonsymmetric matrix found.
Scales for dependent variables:
Concentration (comp1.Cl): 1.4e+02
Concentration (comp1.K): 1.4e+02
Electric potential (comp1.V): 1.9e+02
Orthonormal null-space function used.
Iter      SolEst     Damping    Stepsize #Res #Jac #Sol   LinErr   LinRes
   1        0.83   0.3500000        0.83    2    1    1    6e-11  2.7e-15
   2        0.81   0.3500000        0.81    3    2    2  2.8e-11  2.6e-15
   3         0.8   0.3500000         0.8    4    3    3  4.1e-11  2.7e-15
Velocity u2, Pressure p
Nonsymmetric matrix found.
Scales for dependent variables:
Pressure (comp1.p): 1
Velocity field (spatial frame) (comp1.u2): 0.00018
Orthonormal null-space function used.
Iter      SolEst     Damping    Stepsize #Res #Jac #Sol   LinErr   LinRes
   1         1.1   0.5000000         1.1    2    1    1  1.5e-15  2.3e-15
Displacement field
Nonsymmetric matrix found.
Scales for dependent variables:
Auxiliary pressure (comp1.solid.lemm1.pw): 1
Displacement field (comp1.u): 2.8e-18
Orthonormal null-space function used.
Iter      SolEst     Damping    Stepsize #Res #Jac #Sol   LinErr   LinRes
   1         1.9   0.5000000         1.9    2    1    1  5.9e-14  1.5e-12
Spatial mesh displacement
```

```
Symmetric matrices found.
Scales for dependent variables:
Spatial mesh displacement (comp1.spatial.disp): 1.2e-09
Orthonormal null-space function used.
Iter      SolEst     Damping    Stepsize #Res #Jac #Sol   LinErr   LinRes
   1     8.1e-07   0.5000000     8.1e-07    2    1    1    2e-15  1.8e-15
Solution error estimates for segregated groups
1.4, 0.54, 0.95, 4e-07
Residual error estimates for segregated groups
7.8e+03, 8.2e+04, 2.1e+04, 0.11
Segregated solver iteration 2.
Merged variables
Scales for dependent variables:
Concentration (comp1.Cl): 1.4e+02
Concentration (comp1.K): 1.4e+02
Electric potential (comp1.V): 0.064
Iter      SolEst     Damping    Stepsize #Res #Jac #Sol   LinErr   LinRes
   1        0.78   0.3500000        0.78    6    4    4  4.6e-14    3e-15
   2        0.77   0.3500000        0.77    7    5    5  1.1e-13  3.1e-15
   3        0.76   0.3500000        0.76    8    6    6  1.3e-13    3e-15
Velocity u2, Pressure p
Scales for dependent variables:
Pressure (comp1.p): 3.7e+02
Velocity field (spatial frame) (comp1.u2): 0.00073
Iter      SolEst     Damping    Stepsize #Res #Jac #Sol   LinErr   LinRes
   1        0.36   0.5000000        0.36    4    2    2    2e-15  7.8e-15
Displacement field
Iter      SolEst     Damping    Stepsize #Res #Jac #Sol   LinErr   LinRes
   1        0.95   0.5000000        0.95    4    2    2  1.1e-13  1.5e-12
Spatial mesh displacement
Iter      SolEst     Damping    Stepsize #Res #Jac #Sol   LinErr   LinRes
   1     1.2e-06   0.5000000     1.2e-06    4    2    2  1.8e-15  1.8e-15
Solution error estimates for segregated groups
1.3, 0.18, 0.48, 6.1e-07
Residual error estimates for segregated groups
7.6e+02, 1.6e+04, 2.1e+04, 0.12
Segregated solver iteration 3.
Merged variables
Iter      SolEst     Damping    Stepsize #Res #Jac #Sol   LinErr   LinRes
   1        0.74   0.3500000        0.74   10    7    7  6.4e-14  3.1e-15
   2        0.73   0.3500000        0.73   11    8    8  1.5e-13  3.5e-15
   3        0.71   0.3500000        0.71   12    9    9  4.3e-14  2.8e-15
Velocity u2, Pressure p
Iter      SolEst     Damping    Stepsize #Res #Jac #Sol   LinErr   LinRes
   1        0.15   0.5000000        0.15    6    3    3  2.7e-15  7.9e-15
Displacement field
Iter      SolEst     Damping    Stepsize #Res #Jac #Sol   LinErr   LinRes
   1        0.52   0.5000000        0.52    6    3    3  3.1e-14  1.5e-12
Spatial mesh displacement
Iter      SolEst     Damping    Stepsize #Res #Jac #Sol   LinErr   LinRes
   1     1.2e-06   0.5000000     1.2e-06    6    3    3  1.6e-15  1.8e-15
Solution error estimates for segregated groups
1.2, 0.077, 0.26, 6.1e-07
Residual error estimates for segregated groups
2.1e+02, 9e+03, 1.6e+04, 0.12
Segregated solver iteration 4.
```

```
Merged variables
Iter      SolEst     Damping    Stepsize #Res #Jac #Sol   LinErr   LinRes
   1         0.7   0.3500000         0.7   14   10   10  5.6e-14    3e-15
   2        0.67   0.3500000        0.67   15   11   11  6.2e-14  4.7e-15
   3        0.58   0.3500000        0.58   16   12   12  9.5e-14  3.5e-15
Velocity u2, Pressure p
Iter      SolEst     Damping    Stepsize #Res #Jac #Sol   LinErr   LinRes
   1       0.072   0.5000000       0.072    8    4    4  2.4e-15  7.8e-15
Displacement field
Iter      SolEst     Damping    Stepsize #Res #Jac #Sol   LinErr   LinRes
   1        0.29   0.5000000        0.29    8    4    4  5.5e-14  1.4e-12
Spatial mesh displacement
Iter      SolEst     Damping    Stepsize #Res #Jac #Sol   LinErr   LinRes
   1       1e-06   0.5000000       1e-06    8    4    4  1.7e-15  1.8e-15
Solution error estimates for segregated groups
1, 0.036, 0.15, 5.1e-07
Residual error estimates for segregated groups
81, 4.8e+03, 1.1e+04, 0.13
Segregated solver iteration 5.
Merged variables
Iter      SolEst     Damping    Stepsize #Res #Jac #Sol   LinErr   LinRes
   1         0.4   0.3500000         0.4   18   13   13  4.1e-14    3e-15
   2        0.27   0.3500000        0.27   19   14   14  1.1e-13  2.8e-15
   3        0.18   0.3500000        0.18   20   15   15  1.3e-13  3.2e-15
Velocity u2, Pressure p
Iter      SolEst     Damping    Stepsize #Res #Jac #Sol   LinErr   LinRes
   1       0.035   0.5000000       0.035   10    5    5  2.6e-15  7.8e-15
Displacement field
Scales for dependent variables:
Auxiliary pressure (comp1.solid.lemm1.pw): 0.34
Displacement field (comp1.u): 2.8e-16
Iter      SolEst     Damping    Stepsize #Res #Jac #Sol   LinErr   LinRes
   1        0.17   0.5000000        0.17   10    5    5  1.7e-13  1.2e-12
Spatial mesh displacement
Iter      SolEst     Damping    Stepsize #Res #Jac #Sol   LinErr   LinRes
   1     7.6e-07   0.5000000     7.6e-07   10    5    5  1.1e-15  1.8e-15
Solution error estimates for segregated groups
0.3, 0.017, 0.084, 3.8e-07
Residual error estimates for segregated groups
32, 2.4e+03, 6.8e+03, 0.12
Segregated solver iteration 6.
Merged variables
Iter      SolEst     Damping    Stepsize #Res #Jac #Sol   LinErr   LinRes
   1        0.12   0.3500000        0.12   22   16   16  5.4e-14    3e-15
   2       0.076   0.3500000       0.076   23   17   17  9.3e-14  3.7e-15
   3        0.05   0.3500000        0.05   24   18   18  8.2e-14  2.7e-15
Velocity u2, Pressure p
Iter      SolEst     Damping    Stepsize #Res #Jac #Sol   LinErr   LinRes
   1       0.017   0.5000000       0.017   12    6    6  3.1e-15  7.7e-15
Displacement field
Iter      SolEst     Damping    Stepsize #Res #Jac #Sol   LinErr   LinRes
   1       0.096   0.5000000       0.096   12    6    6    1e-13  1.2e-12
Spatial mesh displacement
Iter      SolEst     Damping    Stepsize #Res #Jac #Sol   LinErr   LinRes
   1     5.3e-07   0.5000000     5.3e-07   12    6    6  1.6e-15  1.8e-15
Solution error estimates for segregated groups
```

```
0.085, 0.0086, 0.048, 2.7e-07
Residual error estimates for segregated groups
12, 1.2e+03, 4.2e+03, 0.12
Segregated solver iteration 7.
Merged variables
Iter      SolEst     Damping    Stepsize #Res #Jac #Sol   LinErr   LinRes
   1       0.032   0.3500000       0.032   26   19   19  6.5e-14  3.3e-15
   2       0.021   0.3500000       0.021   27   20   20  1.1e-13  5.9e-15
   3       0.014   0.3500000       0.014   28   21   21  1.5e-13    3e-15
Velocity u2, Pressure p
Iter      SolEst     Damping    Stepsize #Res #Jac #Sol   LinErr   LinRes
   1      0.0085   0.5000000      0.0085   14    7    7  2.9e-15  7.9e-15
Displacement field
Iter      SolEst     Damping    Stepsize #Res #Jac #Sol   LinErr   LinRes
   1       0.054   0.5000000       0.054   14    7    7  1.2e-13  1.1e-12
Spatial mesh displacement
Iter      SolEst     Damping    Stepsize #Res #Jac #Sol   LinErr   LinRes
   1     3.5e-07   0.5000000     3.5e-07   14    7    7  1.6e-15  1.9e-15
Solution error estimates for segregated groups
0.024, 0.0042, 0.027, 1.8e-07
Residual error estimates for segregated groups
4.1, 6.2e+02, 2.6e+03, 0.13
Segregated solver iteration 8.
Merged variables
Iter      SolEst     Damping    Stepsize #Res #Jac #Sol   LinErr   LinRes
   1      0.0089   0.3500000      0.0089   30   22   22    5e-14  3.2e-15
   2      0.0058   0.3500000      0.0058   31   23   23  7.5e-14  2.9e-15
   3      0.0038   0.3500000      0.0038   32   24   24    9e-14  3.3e-15
Velocity u2, Pressure p
Iter      SolEst     Damping    Stepsize #Res #Jac #Sol   LinErr   LinRes
   1      0.0042   0.5000000      0.0042   16    8    8    5e-15  7.8e-15
Displacement field
Iter      SolEst     Damping    Stepsize #Res #Jac #Sol   LinErr   LinRes
   1        0.03   0.5000000        0.03   16    8    8  1.6e-13  1.2e-12
Spatial mesh displacement
Iter      SolEst     Damping    Stepsize #Res #Jac #Sol   LinErr   LinRes
   1     2.3e-07   0.5000000     2.3e-07   16    8    8  1.3e-15  1.8e-15
Solution error estimates for segregated groups
0.0065, 0.0021, 0.015, 1.1e-07
Residual error estimates for segregated groups
1.3, 3.1e+02, 1.8e+03, 0.12
Segregated solver iteration 9.
Merged variables
Iter      SolEst     Damping    Stepsize #Res #Jac #Sol   LinErr   LinRes
   1      0.0025   0.3500000      0.0025   34   25   25  1.1e-13  3.4e-15
   2      0.0016   0.3500000      0.0016   35   26   26    8e-14  3.5e-15
   3       0.001   0.3500000       0.001   36   27   27  1.3e-13  3.3e-15
Velocity u2, Pressure p
Iter      SolEst     Damping    Stepsize #Res #Jac #Sol   LinErr   LinRes
   1      0.0021   0.5000000      0.0021   18    9    9  3.5e-15    8e-15
Displacement field
Iter      SolEst     Damping    Stepsize #Res #Jac #Sol   LinErr   LinRes
   1       0.017   0.5000000       0.017   18    9    9  1.1e-13  1.2e-12
Spatial mesh displacement
Iter      SolEst     Damping    Stepsize #Res #Jac #Sol   LinErr   LinRes
   1     1.4e-07   0.5000000     1.4e-07   18    9    9  1.3e-15  1.8e-15
```

```
Solution error estimates for segregated groups
0.0018, 0.0011, 0.0085, 7.1e-08
Residual error estimates for segregated groups
0.44, 1.6e+02, 1.4e+03, 0.13
Segregated solver iteration 10.
Merged variables
Iter      SolEst     Damping    Stepsize #Res #Jac #Sol   LinErr   LinRes
   1     0.00067   0.3500000     0.00067   38   28   28  5.5e-14  2.9e-15
   2     0.00044   0.3500000     0.00044   39   29   29  6.7e-14  2.8e-15
   3     0.00028   0.3500000     0.00028   40   30   30  6.2e-14  2.7e-15
Velocity u2, Pressure p
Iter      SolEst     Damping    Stepsize #Res #Jac #Sol   LinErr   LinRes
   1      0.0011   0.5000000      0.0011   20   10   10  3.5e-15  7.7e-15
Displacement field
Iter      SolEst     Damping    Stepsize #Res #Jac #Sol   LinErr   LinRes
   1      0.0094   0.5000000      0.0094   20   10   10  1.6e-13  1.2e-12
Spatial mesh displacement
Iter      SolEst     Damping    Stepsize #Res #Jac #Sol   LinErr   LinRes
   1     8.7e-08   0.5000000     8.7e-08   20   10   10  1.4e-15  1.8e-15
Solution error estimates for segregated groups
0.00049, 0.00053, 0.0047, 4.3e-08
Residual error estimates for segregated groups
0.14, 78, 1.2e+03, 0.12
Segregated solver iteration 11.
Merged variables
Iter      SolEst     Damping    Stepsize #Res #Jac #Sol   LinErr   LinRes
   1     0.00018   0.3500000     0.00018   42   31   31    6e-14  3.4e-15
   2     0.00012   0.3500000     0.00012   43   32   32  8.9e-14  3.4e-15
   3     7.8e-05   0.3500000     7.8e-05   44   33   33    1e-13  4.2e-15
Velocity u2, Pressure p
Iter      SolEst     Damping    Stepsize #Res #Jac #Sol   LinErr   LinRes
   1     0.00053   0.5000000     0.00053   22   11   11  2.2e-15  7.9e-15
Displacement field
Iter      SolEst     Damping    Stepsize #Res #Jac #Sol   LinErr   LinRes
   1      0.0051   0.5000000      0.0051   22   11   11  1.6e-13  1.1e-12
Spatial mesh displacement
Iter      SolEst     Damping    Stepsize #Res #Jac #Sol   LinErr   LinRes
   1     5.2e-08   0.5000000     5.2e-08   22   11   11  1.4e-15  1.8e-15
Solution error estimates for segregated groups
0.00013, 0.00026, 0.0026, 2.6e-08
Residual error estimates for segregated groups
0.043, 39, 1.2e+03, 0.12
Segregated solver iteration 12.
Merged variables
Iter      SolEst     Damping    Stepsize #Res #Jac #Sol   LinErr   LinRes
   1     5.1e-05   0.3500000     5.1e-05   46   34   34  7.3e-14    3e-15
   2     3.3e-05   0.3500000     3.3e-05   47   35   35    5e-14  3.5e-15
   3     2.1e-05   0.3500000     2.1e-05   48   36   36  1.1e-13  3.1e-15
Velocity u2, Pressure p
Iter      SolEst     Damping    Stepsize #Res #Jac #Sol   LinErr   LinRes
   1     0.00026   0.5000000     0.00026   24   12   12  3.1e-15  7.9e-15
Displacement field
Iter      SolEst     Damping    Stepsize #Res #Jac #Sol   LinErr   LinRes
   1      0.0028   0.5000000      0.0028   24   12   12  1.1e-13  1.2e-12
Spatial mesh displacement
Iter      SolEst     Damping    Stepsize #Res #Jac #Sol   LinErr   LinRes
```

```
   1     3.1e-08   0.5000000     3.1e-08   24    12    12  1.4e-15  1.8e-15
Solution error estimates for segregated groups
3.7e-05, 0.00013, 0.0014, 1.5e-08
Residual error estimates for segregated groups
0.013, 20, 1.1e+03, 0.13
Segregated solver iteration 13.
Merged variables
Iter      SolEst      Damping    Stepsize #Res #Jac #Sol   LinErr   LinRes
   1     1.4e-05   0.3500000     1.4e-05   50    37    37  7.2e-14  2.8e-15
   2     9.1e-06   0.3500000     9.1e-06   51    38    38  1.3e-13  4.4e-15
   3     5.9e-06   0.3500000     5.9e-06   52    39    39  8.5e-14  3.5e-15
Velocity u2, Pressure p
Iter      SolEst      Damping    Stepsize #Res #Jac #Sol   LinErr   LinRes
   1     0.00013   0.5000000     0.00013   26    13    13    3e-15  7.7e-15
Displacement field
Iter      SolEst      Damping    Stepsize #Res #Jac #Sol   LinErr   LinRes
   1      0.0015   0.5000000      0.0015   26    13    13    2e-13  1.1e-12
Spatial mesh displacement
Iter      SolEst      Damping    Stepsize #Res #Jac #Sol   LinErr   LinRes
   1     1.8e-08   0.5000000     1.8e-08   26    13    13  1.7e-15  1.8e-15
Solution error estimates for segregated groups
1e-05, 6.6e-05, 0.00076, 9e-09
Residual error estimates for segregated groups
0.004, 9.8, 1.1e+03, 0.12
Solution time: 154 s. (2 minutes, 34 seconds)
Physical memory: 4.83 GB
Virtual memory: 25.65 GB
Ended at Jan 31, 2025, 9:14:55 PM.
----- Stationary Solver 1 in study1/Approach Curve Solver (sol1) -------------->
```

d=2020 (su98)

GENERAL

Description	Value
Solution	d=2020

LOG

```
<---- Compile Equations: Stationary in study1/Approach Curve Solver (sol1) -----
Started at Jan 31, 2025, 9:14:56 PM.
Parameter d = 2.02E-6 (m).
Geometry shape function: Linear serendipity
Number of vertex elements: 18
Number of boundary elements: 6076
Number of elements: 153796
Minimum element quality: 0.4795
Time: 8 s.
Physical memory: 3.06 GB
Virtual memory: 15.29 GB
Ended at Jan 31, 2025, 9:15:03 PM.
----- Compile Equations: Stationary in study1/Approach Curve Solver (sol1) ---->

<---- Dependent Variables 1 in study1/Approach Curve Solver (sol1) -------------
Started at Jan 31, 2025, 9:15:03 PM.
Solution time: 0 s.
Physical memory: 3.06 GB
Virtual memory: 15.29 GB
Ended at Jan 31, 2025, 9:15:04 PM.
----- Dependent Variables 1 in study1/Approach Curve Solver (sol1) ------------>

<---- Stationary Solver 1 in study1/Approach Curve Solver (sol1) ---------------
Started at Jan 31, 2025, 9:15:04 PM.
Segregated solver
Segregated Step 11 does not have any components solved for.
Number of degrees of freedom solved for: 780125 (plus 11575 internal DOFs).
Segregated solver iteration 1.
Merged variables
Nonsymmetric matrix found.
Scales for dependent variables:
Concentration (comp1.Cl): 1.4e+02
Concentration (comp1.K): 1.4e+02
Electric potential (comp1.V): 1.9e+02
Orthonormal null-space function used.
Iter      SolEst     Damping    Stepsize #Res #Jac #Sol   LinErr   LinRes
   1        0.83   0.3500000        0.83    2    1    1  1.5e-11  2.7e-15
   2        0.81   0.3500000        0.81    3    2    2  4.1e-11  2.7e-15
   3         0.8   0.3500000         0.8    4    3    3  2.1e-11  2.7e-15
Velocity u2, Pressure p
Nonsymmetric matrix found.
Scales for dependent variables:
Pressure (comp1.p): 1
Velocity field (spatial frame) (comp1.u2): 0.00018
Orthonormal null-space function used.
Iter      SolEst     Damping    Stepsize #Res #Jac #Sol   LinErr   LinRes
   1         1.1   0.5000000         1.1    2    1    1  1.5e-15  2.2e-15
Displacement field
Nonsymmetric matrix found.
Scales for dependent variables:
Auxiliary pressure (comp1.solid.lemm1.pw): 1
Displacement field (comp1.u): 2.8e-18
Orthonormal null-space function used.
Iter      SolEst     Damping    Stepsize #Res #Jac #Sol   LinErr   LinRes
   1         1.9   0.5000000         1.9    2    1    1  2.6e-14  1.4e-12
Spatial mesh displacement
```

```
Symmetric matrices found.
Scales for dependent variables:
Spatial mesh displacement (comp1.spatial.disp): 1.2e-09
Orthonormal null-space function used.
Iter      SolEst     Damping    Stepsize #Res #Jac #Sol   LinErr   LinRes
   1     8.2e-07   0.5000000     8.2e-07    2    1    1  1.6e-15  1.8e-15
Solution error estimates for segregated groups
1.4, 0.54, 0.95, 4.1e-07
Residual error estimates for segregated groups
7.8e+03, 8.2e+04, 2.1e+04, 0.11
Segregated solver iteration 2.
Merged variables
Scales for dependent variables:
Concentration (comp1.Cl): 1.4e+02
Concentration (comp1.K): 1.4e+02
Electric potential (comp1.V): 0.064
Iter      SolEst     Damping    Stepsize #Res #Jac #Sol   LinErr   LinRes
   1        0.78   0.3500000        0.78    6    4    4  1.3e-13  6.1e-15
   2        0.77   0.3500000        0.77    7    5    5  7.7e-14  3.8e-15
   3        0.76   0.3500000        0.76    8    6    6  7.8e-14  3.8e-15
Velocity u2, Pressure p
Scales for dependent variables:
Pressure (comp1.p): 3.7e+02
Velocity field (spatial frame) (comp1.u2): 0.00074
Iter      SolEst     Damping    Stepsize #Res #Jac #Sol   LinErr   LinRes
   1        0.36   0.5000000        0.36    4    2    2  2.3e-15  7.8e-15
Displacement field
Iter      SolEst     Damping    Stepsize #Res #Jac #Sol   LinErr   LinRes
   1        0.96   0.5000000        0.96    4    2    2  6.1e-14  1.4e-12
Spatial mesh displacement
Iter      SolEst     Damping    Stepsize #Res #Jac #Sol   LinErr   LinRes
   1     1.2e-06   0.5000000     1.2e-06    4    2    2  1.6e-15  1.8e-15
Solution error estimates for segregated groups
1.3, 0.18, 0.48, 6.1e-07
Residual error estimates for segregated groups
7.6e+02, 1.4e+04, 2.1e+04, 0.11
Segregated solver iteration 3.
Merged variables
Iter      SolEst     Damping    Stepsize #Res #Jac #Sol   LinErr   LinRes
   1        0.74   0.3500000        0.74   10    7    7    1e-13  3.5e-15
   2        0.73   0.3500000        0.73   11    8    8  1.1e-13  3.9e-15
   3        0.71   0.3500000        0.71   12    9    9  4.4e-14  3.3e-15
Velocity u2, Pressure p
Iter      SolEst     Damping    Stepsize #Res #Jac #Sol   LinErr   LinRes
   1        0.15   0.5000000        0.15    6    3    3  3.3e-15    8e-15
Displacement field
Iter      SolEst     Damping    Stepsize #Res #Jac #Sol   LinErr   LinRes
   1        0.52   0.5000000        0.52    6    3    3  4.5e-14  1.5e-12
Spatial mesh displacement
Iter      SolEst     Damping    Stepsize #Res #Jac #Sol   LinErr   LinRes
   1     1.2e-06   0.5000000     1.2e-06    6    3    3  1.6e-15  1.8e-15
Solution error estimates for segregated groups
1.2, 0.077, 0.26, 6.1e-07
Residual error estimates for segregated groups
2.1e+02, 7.8e+03, 1.6e+04, 0.13
Segregated solver iteration 4.
```

```
Merged variables
Iter      SolEst     Damping    Stepsize #Res #Jac #Sol   LinErr   LinRes
   1        0.69   0.3500000        0.69   14   10   10  9.4e-14  5.6e-15
   2        0.67   0.3500000        0.67   15   11   11  8.3e-14  4.4e-15
   3        0.58   0.3500000        0.58   16   12   12  8.6e-14  4.4e-15
Velocity u2, Pressure p
Iter      SolEst     Damping    Stepsize #Res #Jac #Sol   LinErr   LinRes
   1       0.072   0.5000000       0.072    8    4    4  2.6e-15  7.8e-15
Displacement field
Iter      SolEst     Damping    Stepsize #Res #Jac #Sol   LinErr   LinRes
   1        0.29   0.5000000        0.29    8    4    4    7e-14  1.4e-12
Spatial mesh displacement
Iter      SolEst     Damping    Stepsize #Res #Jac #Sol   LinErr   LinRes
   1       1e-06   0.5000000       1e-06    8    4    4  1.5e-15  1.8e-15
Solution error estimates for segregated groups
1, 0.036, 0.15, 5.1e-07
Residual error estimates for segregated groups
81, 4.1e+03, 1.1e+04, 0.12
Segregated solver iteration 5.
Merged variables
Iter      SolEst     Damping    Stepsize #Res #Jac #Sol   LinErr   LinRes
   1         0.4   0.3500000         0.4   18   13   13  1.9e-13    3e-15
   2        0.27   0.3500000        0.27   19   14   14    8e-14  3.4e-15
   3        0.18   0.3500000        0.18   20   15   15  6.7e-14  3.5e-15
Velocity u2, Pressure p
Iter      SolEst     Damping    Stepsize #Res #Jac #Sol   LinErr   LinRes
   1       0.035   0.5000000       0.035   10    5    5  2.7e-15  7.7e-15
Displacement field
Scales for dependent variables:
Auxiliary pressure (comp1.solid.lemm1.pw): 0.34
Displacement field (comp1.u): 2.8e-16
Iter      SolEst     Damping    Stepsize #Res #Jac #Sol   LinErr   LinRes
   1        0.17   0.5000000        0.17   10    5    5  1.6e-13  1.2e-12
Spatial mesh displacement
Iter      SolEst     Damping    Stepsize #Res #Jac #Sol   LinErr   LinRes
   1     7.6e-07   0.5000000     7.6e-07   10    5    5  1.7e-15  1.8e-15
Solution error estimates for segregated groups
0.3, 0.017, 0.084, 3.8e-07
Residual error estimates for segregated groups
32, 2.1e+03, 6.8e+03, 0.13
Segregated solver iteration 6.
Merged variables
Iter      SolEst     Damping    Stepsize #Res #Jac #Sol   LinErr   LinRes
   1        0.12   0.3500000        0.12   22   16   16    8e-14  3.7e-15
   2       0.076   0.3500000       0.076   23   17   17  9.1e-14  2.9e-15
   3        0.05   0.3500000        0.05   24   18   18  1.4e-13  3.3e-15
Velocity u2, Pressure p
Iter      SolEst     Damping    Stepsize #Res #Jac #Sol   LinErr   LinRes
   1       0.017   0.5000000       0.017   12    6    6  2.2e-15  7.8e-15
Displacement field
Iter      SolEst     Damping    Stepsize #Res #Jac #Sol   LinErr   LinRes
   1       0.096   0.5000000       0.096   12    6    6  9.6e-14  1.2e-12
Spatial mesh displacement
Iter      SolEst     Damping    Stepsize #Res #Jac #Sol   LinErr   LinRes
   1     5.4e-07   0.5000000     5.4e-07   12    6    6  1.8e-15  1.8e-15
Solution error estimates for segregated groups
```

```
0.085, 0.0086, 0.048, 2.7e-07
Residual error estimates for segregated groups
12, 1.1e+03, 4.2e+03, 0.13
Segregated solver iteration 7.
Merged variables
Iter      SolEst     Damping    Stepsize #Res #Jac #Sol   LinErr   LinRes
   1       0.032   0.3500000       0.032   26   19   19    7e-14  3.6e-15
   2       0.021   0.3500000       0.021   27   20   20  7.1e-14  3.7e-15
   3       0.014   0.3500000       0.014   28   21   21  6.7e-14  5.2e-15
Velocity u2, Pressure p
Iter      SolEst     Damping    Stepsize #Res #Jac #Sol   LinErr   LinRes
   1      0.0085   0.5000000      0.0085   14    7    7  3.4e-15  7.8e-15
Displacement field
Iter      SolEst     Damping    Stepsize #Res #Jac #Sol   LinErr   LinRes
   1       0.054   0.5000000       0.054   14    7    7  1.6e-13  1.2e-12
Spatial mesh displacement
Iter      SolEst     Damping    Stepsize #Res #Jac #Sol   LinErr   LinRes
   1     3.6e-07   0.5000000     3.6e-07   14    7    7  1.7e-15  1.8e-15
Solution error estimates for segregated groups
0.023, 0.0042, 0.027, 1.8e-07
Residual error estimates for segregated groups
4.1, 5.4e+02, 2.6e+03, 0.13
Segregated solver iteration 8.
Merged variables
Iter      SolEst     Damping    Stepsize #Res #Jac #Sol   LinErr   LinRes
   1      0.0089   0.3500000      0.0089   30   22   22  1.2e-13  4.6e-15
   2      0.0058   0.3500000      0.0058   31   23   23  1.1e-13  5.2e-15
   3      0.0038   0.3500000      0.0038   32   24   24  7.1e-14  4.6e-15
Velocity u2, Pressure p
Iter      SolEst     Damping    Stepsize #Res #Jac #Sol   LinErr   LinRes
   1      0.0042   0.5000000      0.0042   16    8    8  4.1e-15  7.8e-15
Displacement field
Iter      SolEst     Damping    Stepsize #Res #Jac #Sol   LinErr   LinRes
   1        0.03   0.5000000        0.03   16    8    8  1.2e-13  1.2e-12
Spatial mesh displacement
Iter      SolEst     Damping    Stepsize #Res #Jac #Sol   LinErr   LinRes
   1     2.3e-07   0.5000000     2.3e-07   16    8    8  1.6e-15  1.8e-15
Solution error estimates for segregated groups
0.0065, 0.0021, 0.015, 1.1e-07
Residual error estimates for segregated groups
1.3, 2.7e+02, 1.8e+03, 0.12
Segregated solver iteration 9.
Merged variables
Iter      SolEst     Damping    Stepsize #Res #Jac #Sol   LinErr   LinRes
   1      0.0024   0.3500000      0.0024   34   25   25  9.3e-14  3.9e-15
   2      0.0016   0.3500000      0.0016   35   26   26  1.3e-13  3.5e-15
   3       0.001   0.3500000       0.001   36   27   27  7.7e-14  5.4e-15
Velocity u2, Pressure p
Iter      SolEst     Damping    Stepsize #Res #Jac #Sol   LinErr   LinRes
   1      0.0021   0.5000000      0.0021   18    9    9  3.1e-15  7.8e-15
Displacement field
Iter      SolEst     Damping    Stepsize #Res #Jac #Sol   LinErr   LinRes
   1       0.017   0.5000000       0.017   18    9    9  1.2e-13  1.1e-12
Spatial mesh displacement
Iter      SolEst     Damping    Stepsize #Res #Jac #Sol   LinErr   LinRes
   1     1.4e-07   0.5000000     1.4e-07   18    9    9  1.5e-15  1.9e-15
```

```
Solution error estimates for segregated groups
0.0018, 0.0011, 0.0085, 7.2e-08
Residual error estimates for segregated groups
0.44, 1.4e+02, 1.4e+03, 0.13
Segregated solver iteration 10.
Merged variables
Iter      SolEst     Damping    Stepsize #Res #Jac #Sol   LinErr   LinRes
   1     0.00067   0.3500000     0.00067   38   28   28  5.4e-14  5.6e-15
   2     0.00044   0.3500000     0.00044   39   29   29  1.1e-13  3.2e-15
   3     0.00028   0.3500000     0.00028   40   30   30  1.2e-13  3.6e-15
Velocity u2, Pressure p
Iter      SolEst     Damping    Stepsize #Res #Jac #Sol   LinErr   LinRes
   1      0.0011   0.5000000      0.0011   20   10   10  1.5e-15  7.8e-15
Displacement field
Iter      SolEst     Damping    Stepsize #Res #Jac #Sol   LinErr   LinRes
   1      0.0094   0.5000000      0.0094   20   10   10  1.9e-13  1.2e-12
Spatial mesh displacement
Iter      SolEst     Damping    Stepsize #Res #Jac #Sol   LinErr   LinRes
   1     8.8e-08   0.5000000     8.8e-08   20   10   10  1.6e-15  1.8e-15
Solution error estimates for segregated groups
0.00049, 0.00053, 0.0047, 4.4e-08
Residual error estimates for segregated groups
0.14, 68, 1.2e+03, 0.12
Segregated solver iteration 11.
Merged variables
Iter      SolEst     Damping    Stepsize #Res #Jac #Sol   LinErr   LinRes
   1     0.00018   0.3500000     0.00018   42   31   31  7.3e-14  3.6e-15
   2     0.00012   0.3500000     0.00012   43   32   32  9.1e-14  3.9e-15
   3     7.8e-05   0.3500000     7.8e-05   44   33   33  1.5e-13  3.9e-15
Velocity u2, Pressure p
Iter      SolEst     Damping    Stepsize #Res #Jac #Sol   LinErr   LinRes
   1     0.00053   0.5000000     0.00053   22   11   11  2.6e-15  7.8e-15
Displacement field
Iter      SolEst     Damping    Stepsize #Res #Jac #Sol   LinErr   LinRes
   1      0.0051   0.5000000      0.0051   22   11   11  2.3e-13  1.1e-12
Spatial mesh displacement
Iter      SolEst     Damping    Stepsize #Res #Jac #Sol   LinErr   LinRes
   1     5.3e-08   0.5000000     5.3e-08   22   11   11  1.3e-15  1.8e-15
Solution error estimates for segregated groups
0.00013, 0.00026, 0.0026, 2.6e-08
Residual error estimates for segregated groups
0.043, 34, 1.2e+03, 0.14
Segregated solver iteration 12.
Merged variables
Iter      SolEst     Damping    Stepsize #Res #Jac #Sol   LinErr   LinRes
   1     5.1e-05   0.3500000     5.1e-05   46   34   34  6.3e-14    4e-15
   2     3.3e-05   0.3500000     3.3e-05   47   35   35  5.9e-14  4.7e-15
   3     2.1e-05   0.3500000     2.1e-05   48   36   36  5.5e-14  3.9e-15
Velocity u2, Pressure p
Iter      SolEst     Damping    Stepsize #Res #Jac #Sol   LinErr   LinRes
   1     0.00026   0.5000000     0.00026   24   12   12  2.3e-15  7.9e-15
Displacement field
Iter      SolEst     Damping    Stepsize #Res #Jac #Sol   LinErr   LinRes
   1      0.0028   0.5000000      0.0028   24   12   12  1.1e-13  1.2e-12
Spatial mesh displacement
Iter      SolEst     Damping    Stepsize #Res #Jac #Sol   LinErr   LinRes
```

```
   1     3.1e-08   0.5000000     3.1e-08   24    12    12  1.5e-15  1.8e-15
Solution error estimates for segregated groups
3.7e-05, 0.00013, 0.0014, 1.6e-08
Residual error estimates for segregated groups
0.013, 17, 1.2e+03, 0.13
Segregated solver iteration 13.
Merged variables
Iter      SolEst     Damping    Stepsize #Res #Jac #Sol   LinErr   LinRes
   1     1.4e-05   0.3500000     1.4e-05   50    37    37    7e-14  3.7e-15
   2       9e-06   0.3500000       9e-06   51    38    38    7e-14  3.6e-15
   3     5.9e-06   0.3500000     5.9e-06   52    39    39  1.1e-13  3.5e-15
Velocity u2, Pressure p
Iter      SolEst     Damping    Stepsize #Res #Jac #Sol   LinErr   LinRes
   1     0.00013   0.5000000     0.00013   26    13    13  4.3e-15  7.8e-15
Displacement field
Iter      SolEst     Damping    Stepsize #Res #Jac #Sol   LinErr   LinRes
   1      0.0015   0.5000000      0.0015   26    13    13    1e-13  1.2e-12
Spatial mesh displacement
Iter      SolEst     Damping    Stepsize #Res #Jac #Sol   LinErr   LinRes
   1     1.8e-08   0.5000000     1.8e-08   26    13    13  1.6e-15  1.8e-15
Solution error estimates for segregated groups
1e-05, 6.6e-05, 0.00076, 9.1e-09
Residual error estimates for segregated groups
0.004, 8.5, 1.2e+03, 0.12
Solution time: 153 s. (2 minutes, 33 seconds)
Physical memory: 4.8 GB
Virtual memory: 25.58 GB
Ended at Jan 31, 2025, 9:17:36 PM.
----- Stationary Solver 1 in study1/Approach Curve Solver (sol1) -------------->
```

d=2015 (su99)

GENERAL

Description	Value
Solution	d=2015

LOG

```
<---- Compile Equations: Stationary in study1/Approach Curve Solver (sol1) -----
Started at Jan 31, 2025, 9:17:37 PM.
Parameter d = 2.015E-6 (m).
Geometry shape function: Linear serendipity
Number of vertex elements: 18
Number of boundary elements: 6074
Number of elements: 153870
Minimum element quality: 0.4799
Time: 8 s.
Physical memory: 3.08 GB
Virtual memory: 15.26 GB
Ended at Jan 31, 2025, 9:17:45 PM.
----- Compile Equations: Stationary in study1/Approach Curve Solver (sol1) ---->

<---- Dependent Variables 1 in study1/Approach Curve Solver (sol1) -------------
Started at Jan 31, 2025, 9:17:45 PM.
Solution time: 0 s.
Physical memory: 3.08 GB
Virtual memory: 15.26 GB
Ended at Jan 31, 2025, 9:17:45 PM.
----- Dependent Variables 1 in study1/Approach Curve Solver (sol1) ------------>

<---- Stationary Solver 1 in study1/Approach Curve Solver (sol1) ---------------
Started at Jan 31, 2025, 9:17:45 PM.
Segregated solver
Segregated Step 11 does not have any components solved for.
Number of degrees of freedom solved for: 780511 (plus 11571 internal DOFs).
Segregated solver iteration 1.
Merged variables
Nonsymmetric matrix found.
Scales for dependent variables:
Concentration (comp1.Cl): 1.4e+02
Concentration (comp1.K): 1.4e+02
Electric potential (comp1.V): 1.9e+02
Orthonormal null-space function used.
Iter      SolEst     Damping    Stepsize #Res #Jac #Sol   LinErr   LinRes
   1        0.83   0.3500000        0.83    2    1    1  1.7e-11  2.7e-15
   2        0.81   0.3500000        0.81    3    2    2  4.5e-11  2.7e-15
   3         0.8   0.3500000         0.8    4    3    3  3.6e-11  2.7e-15
Velocity u2, Pressure p
Nonsymmetric matrix found.
Scales for dependent variables:
Pressure (comp1.p): 1
Velocity field (spatial frame) (comp1.u2): 0.00018
Orthonormal null-space function used.
Iter      SolEst     Damping    Stepsize #Res #Jac #Sol   LinErr   LinRes
   1         1.1   0.5000000         1.1    2    1    1  1.6e-15  2.3e-15
Displacement field
Nonsymmetric matrix found.
Scales for dependent variables:
Auxiliary pressure (comp1.solid.lemm1.pw): 1
Displacement field (comp1.u): 2.8e-18
Orthonormal null-space function used.
Iter      SolEst     Damping    Stepsize #Res #Jac #Sol   LinErr   LinRes
   1         1.9   0.5000000         1.9    2    1    1  2.9e-14  1.5e-12
Spatial mesh displacement
```

```
Symmetric matrices found.
Scales for dependent variables:
Spatial mesh displacement (comp1.spatial.disp): 1.2e-09
Orthonormal null-space function used.
Iter      SolEst     Damping    Stepsize #Res #Jac #Sol   LinErr   LinRes
   1     8.2e-07   0.5000000     8.2e-07    2    1    1  1.3e-15  1.8e-15
Solution error estimates for segregated groups
1.4, 0.54, 0.95, 4.1e-07
Residual error estimates for segregated groups
7.8e+03, 8.2e+04, 2.1e+04, 0.12
Segregated solver iteration 2.
Merged variables
Scales for dependent variables:
Concentration (comp1.Cl): 1.4e+02
Concentration (comp1.K): 1.4e+02
Electric potential (comp1.V): 0.064
Iter      SolEst     Damping    Stepsize #Res #Jac #Sol   LinErr   LinRes
   1        0.78   0.3500000        0.78    6    4    4  9.3e-14  2.7e-15
   2        0.77   0.3500000        0.77    7    5    5  8.5e-14  4.1e-15
   3        0.76   0.3500000        0.76    8    6    6  7.7e-14  2.9e-15
Velocity u2, Pressure p
Scales for dependent variables:
Pressure (comp1.p): 3.7e+02
Velocity field (spatial frame) (comp1.u2): 0.00073
Iter      SolEst     Damping    Stepsize #Res #Jac #Sol   LinErr   LinRes
   1        0.36   0.5000000        0.36    4    2    2  2.9e-15  7.9e-15
Displacement field
Iter      SolEst     Damping    Stepsize #Res #Jac #Sol   LinErr   LinRes
   1        0.95   0.5000000        0.95    4    2    2  9.2e-14  1.5e-12
Spatial mesh displacement
Iter      SolEst     Damping    Stepsize #Res #Jac #Sol   LinErr   LinRes
   1     1.2e-06   0.5000000     1.2e-06    4    2    2  1.4e-15  1.8e-15
Solution error estimates for segregated groups
1.3, 0.18, 0.48, 6.1e-07
Residual error estimates for segregated groups
7.6e+02, 1.4e+04, 2.1e+04, 0.11
Segregated solver iteration 3.
Merged variables
Iter      SolEst     Damping    Stepsize #Res #Jac #Sol   LinErr   LinRes
   1        0.74   0.3500000        0.74   10    7    7  6.8e-14  2.8e-15
   2        0.73   0.3500000        0.73   11    8    8  4.3e-14  2.8e-15
   3        0.71   0.3500000        0.71   12    9    9  7.2e-14  2.7e-15
Velocity u2, Pressure p
Iter      SolEst     Damping    Stepsize #Res #Jac #Sol   LinErr   LinRes
   1        0.15   0.5000000        0.15    6    3    3  2.2e-15  7.9e-15
Displacement field
Iter      SolEst     Damping    Stepsize #Res #Jac #Sol   LinErr   LinRes
   1        0.52   0.5000000        0.52    6    3    3  1.3e-13  1.5e-12
Spatial mesh displacement
Iter      SolEst     Damping    Stepsize #Res #Jac #Sol   LinErr   LinRes
   1     1.2e-06   0.5000000     1.2e-06    6    3    3  1.5e-15  1.8e-15
Solution error estimates for segregated groups
1.2, 0.077, 0.26, 6.1e-07
Residual error estimates for segregated groups
2.1e+02, 7.7e+03, 1.6e+04, 0.13
Segregated solver iteration 4.
```

```
Merged variables
Iter      SolEst     Damping    Stepsize #Res #Jac #Sol   LinErr   LinRes
   1         0.7   0.3500000         0.7   14   10   10  8.6e-14  2.8e-15
   2        0.67   0.3500000        0.67   15   11   11  5.2e-14  3.1e-15
   3        0.58   0.3500000        0.58   16   12   12    6e-14  2.8e-15
Velocity u2, Pressure p
Iter      SolEst     Damping    Stepsize #Res #Jac #Sol   LinErr   LinRes
   1       0.072   0.5000000       0.072    8    4    4  2.6e-15    8e-15
Displacement field
Iter      SolEst     Damping    Stepsize #Res #Jac #Sol   LinErr   LinRes
   1        0.29   0.5000000        0.29    8    4    4  6.4e-14  1.5e-12
Spatial mesh displacement
Iter      SolEst     Damping    Stepsize #Res #Jac #Sol   LinErr   LinRes
   1       1e-06   0.5000000       1e-06    8    4    4  1.8e-15  1.8e-15
Solution error estimates for segregated groups
1, 0.036, 0.15, 5.1e-07
Residual error estimates for segregated groups
81, 4e+03, 1.1e+04, 0.13
Segregated solver iteration 5.
Merged variables
Iter      SolEst     Damping    Stepsize #Res #Jac #Sol   LinErr   LinRes
   1         0.4   0.3500000         0.4   18   13   13  6.5e-14  3.1e-15
   2        0.27   0.3500000        0.27   19   14   14  7.9e-14  2.9e-15
   3        0.18   0.3500000        0.18   20   15   15  6.6e-14  2.8e-15
Velocity u2, Pressure p
Iter      SolEst     Damping    Stepsize #Res #Jac #Sol   LinErr   LinRes
   1       0.035   0.5000000       0.035   10    5    5    4e-15  7.8e-15
Displacement field
Scales for dependent variables:
Auxiliary pressure (comp1.solid.lemm1.pw): 0.34
Displacement field (comp1.u): 2.8e-16
Iter      SolEst     Damping    Stepsize #Res #Jac #Sol   LinErr   LinRes
   1        0.17   0.5000000        0.17   10    5    5  1.5e-13  1.2e-12
Spatial mesh displacement
Iter      SolEst     Damping    Stepsize #Res #Jac #Sol   LinErr   LinRes
   1     7.7e-07   0.5000000     7.7e-07   10    5    5  1.6e-15  1.9e-15
Solution error estimates for segregated groups
0.3, 0.017, 0.084, 3.8e-07
Residual error estimates for segregated groups
32, 2.1e+03, 6.8e+03, 0.12
Segregated solver iteration 6.
Merged variables
Iter      SolEst     Damping    Stepsize #Res #Jac #Sol   LinErr   LinRes
   1        0.12   0.3500000        0.12   22   16   16  1.1e-13    3e-15
   2       0.076   0.3500000       0.076   23   17   17  1.1e-13  2.7e-15
   3        0.05   0.3500000        0.05   24   18   18  4.8e-14  2.9e-15
Velocity u2, Pressure p
Iter      SolEst     Damping    Stepsize #Res #Jac #Sol   LinErr   LinRes
   1       0.017   0.5000000       0.017   12    6    6  2.6e-15  8.1e-15
Displacement field
Iter      SolEst     Damping    Stepsize #Res #Jac #Sol   LinErr   LinRes
   1       0.096   0.5000000       0.096   12    6    6  1.2e-13  1.2e-12
Spatial mesh displacement
Iter      SolEst     Damping    Stepsize #Res #Jac #Sol   LinErr   LinRes
   1     5.4e-07   0.5000000     5.4e-07   12    6    6  2.1e-15  1.8e-15
Solution error estimates for segregated groups
```

```
0.085, 0.0085, 0.048, 2.7e-07
Residual error estimates for segregated groups
12, 1.1e+03, 4.2e+03, 0.13
Segregated solver iteration 7.
Merged variables
Iter      SolEst     Damping    Stepsize #Res #Jac #Sol   LinErr   LinRes
   1       0.032   0.3500000       0.032   26   19   19  1.2e-13  2.8e-15
   2       0.021   0.3500000       0.021   27   20   20  8.5e-14    3e-15
   3       0.014   0.3500000       0.014   28   21   21    5e-14  2.7e-15
Velocity u2, Pressure p
Iter      SolEst     Damping    Stepsize #Res #Jac #Sol   LinErr   LinRes
   1      0.0085   0.5000000      0.0085   14    7    7  2.5e-15  7.9e-15
Displacement field
Iter      SolEst     Damping    Stepsize #Res #Jac #Sol   LinErr   LinRes
   1       0.054   0.5000000       0.054   14    7    7  2.3e-13  1.2e-12
Spatial mesh displacement
Iter      SolEst     Damping    Stepsize #Res #Jac #Sol   LinErr   LinRes
   1     3.6e-07   0.5000000     3.6e-07   14    7    7  1.4e-15  1.8e-15
Solution error estimates for segregated groups
0.024, 0.0042, 0.027, 1.8e-07
Residual error estimates for segregated groups
4.1, 5.3e+02, 2.6e+03, 0.13
Segregated solver iteration 8.
Merged variables
Iter      SolEst     Damping    Stepsize #Res #Jac #Sol   LinErr   LinRes
   1      0.0089   0.3500000      0.0089   30   22   22  5.4e-14    3e-15
   2      0.0058   0.3500000      0.0058   31   23   23  8.5e-14  2.8e-15
   3      0.0038   0.3500000      0.0038   32   24   24  9.1e-14  2.8e-15
Velocity u2, Pressure p
Iter      SolEst     Damping    Stepsize #Res #Jac #Sol   LinErr   LinRes
   1      0.0042   0.5000000      0.0042   16    8    8  6.1e-15  7.9e-15
Displacement field
Iter      SolEst     Damping    Stepsize #Res #Jac #Sol   LinErr   LinRes
   1        0.03   0.5000000        0.03   16    8    8  9.9e-14  1.2e-12
Spatial mesh displacement
Iter      SolEst     Damping    Stepsize #Res #Jac #Sol   LinErr   LinRes
   1     2.3e-07   0.5000000     2.3e-07   16    8    8  1.4e-15  1.8e-15
Solution error estimates for segregated groups
0.0065, 0.0021, 0.015, 1.2e-07
Residual error estimates for segregated groups
1.3, 2.7e+02, 1.8e+03, 0.13
Segregated solver iteration 9.
Merged variables
Iter      SolEst     Damping    Stepsize #Res #Jac #Sol   LinErr   LinRes
   1      0.0024   0.3500000      0.0024   34   25   25  9.1e-14  2.8e-15
   2      0.0016   0.3500000      0.0016   35   26   26  9.4e-14  3.3e-15
   3       0.001   0.3500000       0.001   36   27   27  7.6e-14    3e-15
Velocity u2, Pressure p
Iter      SolEst     Damping    Stepsize #Res #Jac #Sol   LinErr   LinRes
   1      0.0021   0.5000000      0.0021   18    9    9  3.8e-15  7.9e-15
Displacement field
Iter      SolEst     Damping    Stepsize #Res #Jac #Sol   LinErr   LinRes
   1       0.017   0.5000000       0.017   18    9    9  1.5e-13  1.2e-12
Spatial mesh displacement
Iter      SolEst     Damping    Stepsize #Res #Jac #Sol   LinErr   LinRes
   1     1.4e-07   0.5000000     1.4e-07   18    9    9  1.4e-15  1.9e-15
```

```
Solution error estimates for segregated groups
0.0018, 0.0011, 0.0085, 7.2e-08
Residual error estimates for segregated groups
0.44, 1.3e+02, 1.4e+03, 0.12
Segregated solver iteration 10.
Merged variables
Iter      SolEst     Damping    Stepsize #Res #Jac #Sol   LinErr   LinRes
   1     0.00067   0.3500000     0.00067   38   28   28  5.9e-14  2.7e-15
   2     0.00044   0.3500000     0.00044   39   29   29  9.4e-14  2.7e-15
   3     0.00028   0.3500000     0.00028   40   30   30  8.5e-14  2.8e-15
Velocity u2, Pressure p
Iter      SolEst     Damping    Stepsize #Res #Jac #Sol   LinErr   LinRes
   1      0.0011   0.5000000      0.0011   20   10   10  2.3e-15    8e-15
Displacement field
Iter      SolEst     Damping    Stepsize #Res #Jac #Sol   LinErr   LinRes
   1      0.0094   0.5000000      0.0094   20   10   10  1.7e-13  1.2e-12
Spatial mesh displacement
Iter      SolEst     Damping    Stepsize #Res #Jac #Sol   LinErr   LinRes
   1     8.8e-08   0.5000000     8.8e-08   20   10   10  1.6e-15  1.8e-15
Solution error estimates for segregated groups
0.00049, 0.00053, 0.0047, 4.4e-08
Residual error estimates for segregated groups
0.14, 66, 1.3e+03, 0.14
Segregated solver iteration 11.
Merged variables
Iter      SolEst     Damping    Stepsize #Res #Jac #Sol   LinErr   LinRes
   1     0.00018   0.3500000     0.00018   42   31   31  7.5e-14  2.9e-15
   2     0.00012   0.3500000     0.00012   43   32   32  3.8e-14  3.3e-15
   3     7.8e-05   0.3500000     7.8e-05   44   33   33  4.9e-14  2.6e-15
Velocity u2, Pressure p
Iter      SolEst     Damping    Stepsize #Res #Jac #Sol   LinErr   LinRes
   1     0.00053   0.5000000     0.00053   22   11   11  1.9e-15    8e-15
Displacement field
Iter      SolEst     Damping    Stepsize #Res #Jac #Sol   LinErr   LinRes
   1      0.0051   0.5000000      0.0051   22   11   11  1.2e-13  1.2e-12
Spatial mesh displacement
Iter      SolEst     Damping    Stepsize #Res #Jac #Sol   LinErr   LinRes
   1     5.3e-08   0.5000000     5.3e-08   22   11   11  1.6e-15  1.8e-15
Solution error estimates for segregated groups
0.00013, 0.00026, 0.0026, 2.6e-08
Residual error estimates for segregated groups
0.043, 33, 1.2e+03, 0.13
Segregated solver iteration 12.
Merged variables
Iter      SolEst     Damping    Stepsize #Res #Jac #Sol   LinErr   LinRes
   1     5.1e-05   0.3500000     5.1e-05   46   34   34  8.5e-14  3.1e-15
   2     3.3e-05   0.3500000     3.3e-05   47   35   35    1e-13  2.8e-15
   3     2.1e-05   0.3500000     2.1e-05   48   36   36  9.7e-14  2.8e-15
Velocity u2, Pressure p
Iter      SolEst     Damping    Stepsize #Res #Jac #Sol   LinErr   LinRes
   1     0.00026   0.5000000     0.00026   24   12   12    7e-15  7.8e-15
Displacement field
Iter      SolEst     Damping    Stepsize #Res #Jac #Sol   LinErr   LinRes
   1      0.0028   0.5000000      0.0028   24   12   12  1.2e-13  1.1e-12
Spatial mesh displacement
Iter      SolEst     Damping    Stepsize #Res #Jac #Sol   LinErr   LinRes
```

```
   1      3.1e-08   0.5000000     3.1e-08   24    12    12  1.3e-15  1.8e-15
Solution error estimates for segregated groups
3.7e-05, 0.00013, 0.0014, 1.6e-08
Residual error estimates for segregated groups
0.013, 17, 1.2e+03, 0.13
Segregated solver iteration 13.
Merged variables
Iter       SolEst      Damping    Stepsize #Res #Jac #Sol   LinErr   LinRes
   1      1.4e-05   0.3500000     1.4e-05   50    37    37    7e-14    3e-15
   2      9.1e-06   0.3500000     9.1e-06   51    38    38  5.9e-14  2.7e-15
   3      5.9e-06   0.3500000     5.9e-06   52    39    39  9.4e-14  2.7e-15
Velocity u2, Pressure p
Iter       SolEst      Damping    Stepsize #Res #Jac #Sol   LinErr   LinRes
   1      0.00013   0.5000000     0.00013   26    13    13  2.1e-15    8e-15
Displacement field
Iter       SolEst      Damping    Stepsize #Res #Jac #Sol   LinErr   LinRes
   1       0.0015   0.5000000      0.0015   26    13    13  1.2e-13  1.2e-12
Spatial mesh displacement
Iter       SolEst      Damping    Stepsize #Res #Jac #Sol   LinErr   LinRes
   1      1.8e-08   0.5000000     1.8e-08   26    13    13  1.8e-15  1.8e-15
Solution error estimates for segregated groups
1e-05, 6.6e-05, 0.00076, 9.1e-09
Residual error estimates for segregated groups
0.004, 8.3, 1.2e+03, 0.11
Solution time: 154 s. (2 minutes, 34 seconds)
Physical memory: 4.85 GB
Virtual memory: 25.61 GB
Ended at Jan 31, 2025, 9:20:19 PM.
----- Stationary Solver 1 in study1/Approach Curve Solver (sol1) -------------->
```

d=2010 (su100)

GENERAL

Description	Value
Solution	d=2010

LOG

```
<---- Compile Equations: Stationary in study1/Approach Curve Solver (sol1) -----
Started at Jan 31, 2025, 9:20:19 PM.
Parameter d = 2.0100000000000002E-6 (m).
Geometry shape function: Linear serendipity
Number of vertex elements: 18
Number of boundary elements: 6071
Number of elements: 153557
Minimum element quality: 0.4712
Time: 8 s.
Physical memory: 3.11 GB
Virtual memory: 15.3 GB
Ended at Jan 31, 2025, 9:20:27 PM.
----- Compile Equations: Stationary in study1/Approach Curve Solver (sol1) ---->

<---- Dependent Variables 1 in study1/Approach Curve Solver (sol1) -------------
Started at Jan 31, 2025, 9:20:27 PM.
Solution time: 0 s.
Physical memory: 3.11 GB
Virtual memory: 15.3 GB
Ended at Jan 31, 2025, 9:20:27 PM.
----- Dependent Variables 1 in study1/Approach Curve Solver (sol1) ------------>

<---- Stationary Solver 1 in study1/Approach Curve Solver (sol1) ---------------
Started at Jan 31, 2025, 9:20:28 PM.
Segregated solver
Segregated Step 11 does not have any components solved for.
Number of degrees of freedom solved for: 778984 (plus 11565 internal DOFs).
Segregated solver iteration 1.
Merged variables
Nonsymmetric matrix found.
Scales for dependent variables:
Concentration (comp1.Cl): 1.4e+02
Concentration (comp1.K): 1.4e+02
Electric potential (comp1.V): 1.9e+02
Orthonormal null-space function used.
Iter      SolEst     Damping    Stepsize #Res #Jac #Sol   LinErr   LinRes
   1        0.83   0.3500000        0.83    2    1    1  2.5e-11  2.7e-15
   2        0.81   0.3500000        0.81    3    2    2  4.1e-11  2.7e-15
   3         0.8   0.3500000         0.8    4    3    3  2.5e-11  2.7e-15
Velocity u2, Pressure p
Nonsymmetric matrix found.
Scales for dependent variables:
Pressure (comp1.p): 1
Velocity field (spatial frame) (comp1.u2): 0.00018
Orthonormal null-space function used.
Iter      SolEst     Damping    Stepsize #Res #Jac #Sol   LinErr   LinRes
   1         1.1   0.5000000         1.1    2    1    1  1.6e-15  2.3e-15
Displacement field
Nonsymmetric matrix found.
Scales for dependent variables:
Auxiliary pressure (comp1.solid.lemm1.pw): 1
Displacement field (comp1.u): 2.8e-18
Orthonormal null-space function used.
Iter      SolEst     Damping    Stepsize #Res #Jac #Sol   LinErr   LinRes
   1         1.9   0.5000000         1.9    2    1    1  1.1e-13  1.5e-12
Spatial mesh displacement
```

```
Symmetric matrices found.
Scales for dependent variables:
Spatial mesh displacement (comp1.spatial.disp): 1.2e-09
Orthonormal null-space function used.
Iter      SolEst     Damping    Stepsize #Res #Jac #Sol   LinErr   LinRes
   1     8.2e-07   0.5000000     8.2e-07    2    1    1  1.3e-15  1.8e-15
Solution error estimates for segregated groups
1.4, 0.54, 0.95, 4.1e-07
Residual error estimates for segregated groups
7.8e+03, 8.2e+04, 2.1e+04, 0.11
Segregated solver iteration 2.
Merged variables
Scales for dependent variables:
Concentration (comp1.Cl): 1.4e+02
Concentration (comp1.K): 1.4e+02
Electric potential (comp1.V): 0.064
Iter      SolEst     Damping    Stepsize #Res #Jac #Sol   LinErr   LinRes
   1        0.78   0.3500000        0.78    6    4    4  1.4e-13  5.4e-15
   2        0.77   0.3500000        0.77    7    5    5    1e-13  3.4e-15
   3        0.76   0.3500000        0.76    8    6    6  1.6e-13  4.5e-15
Velocity u2, Pressure p
Scales for dependent variables:
Pressure (comp1.p): 3.7e+02
Velocity field (spatial frame) (comp1.u2): 0.00073
Iter      SolEst     Damping    Stepsize #Res #Jac #Sol   LinErr   LinRes
   1        0.36   0.5000000        0.36    4    2    2  1.8e-15  7.7e-15
Displacement field
Iter      SolEst     Damping    Stepsize #Res #Jac #Sol   LinErr   LinRes
   1        0.96   0.5000000        0.96    4    2    2  4.6e-14  1.4e-12
Spatial mesh displacement
Iter      SolEst     Damping    Stepsize #Res #Jac #Sol   LinErr   LinRes
   1     1.2e-06   0.5000000     1.2e-06    4    2    2  1.5e-15  1.8e-15
Solution error estimates for segregated groups
1.3, 0.18, 0.48, 6.2e-07
Residual error estimates for segregated groups
7.6e+02, 1.5e+04, 2.1e+04, 0.13
Segregated solver iteration 3.
Merged variables
Iter      SolEst     Damping    Stepsize #Res #Jac #Sol   LinErr   LinRes
   1        0.74   0.3500000        0.74   10    7    7  8.6e-14  4.3e-15
   2        0.73   0.3500000        0.73   11    8    8  1.3e-13    6e-15
   3        0.71   0.3500000        0.71   12    9    9  7.3e-14  6.8e-15
Velocity u2, Pressure p
Iter      SolEst     Damping    Stepsize #Res #Jac #Sol   LinErr   LinRes
   1        0.15   0.5000000        0.15    6    3    3    4e-15  7.9e-15
Displacement field
Iter      SolEst     Damping    Stepsize #Res #Jac #Sol   LinErr   LinRes
   1        0.52   0.5000000        0.52    6    3    3  1.2e-13  1.5e-12
Spatial mesh displacement
Iter      SolEst     Damping    Stepsize #Res #Jac #Sol   LinErr   LinRes
   1     1.2e-06   0.5000000     1.2e-06    6    3    3  1.4e-15  1.8e-15
Solution error estimates for segregated groups
1.2, 0.077, 0.26, 6.2e-07
Residual error estimates for segregated groups
2.1e+02, 8.3e+03, 1.6e+04, 0.14
Segregated solver iteration 4.
```

```
Merged variables
Iter      SolEst     Damping    Stepsize #Res #Jac #Sol   LinErr   LinRes
   1        0.69   0.3500000        0.69   14   10   10  1.5e-13  2.8e-15
   2        0.67   0.3500000        0.67   15   11   11    1e-13    3e-15
   3        0.58   0.3500000        0.58   16   12   12  5.2e-14  2.8e-15
Velocity u2, Pressure p
Iter      SolEst     Damping    Stepsize #Res #Jac #Sol   LinErr   LinRes
   1       0.072   0.5000000       0.072    8    4    4  2.7e-15  7.9e-15
Displacement field
Iter      SolEst     Damping    Stepsize #Res #Jac #Sol   LinErr   LinRes
   1        0.29   0.5000000        0.29    8    4    4  1.2e-13  1.5e-12
Spatial mesh displacement
Iter      SolEst     Damping    Stepsize #Res #Jac #Sol   LinErr   LinRes
   1       1e-06   0.5000000       1e-06    8    4    4  1.6e-15  1.8e-15
Solution error estimates for segregated groups
1, 0.036, 0.15, 5.1e-07
Residual error estimates for segregated groups
81, 4.4e+03, 1.1e+04, 0.13
Segregated solver iteration 5.
Merged variables
Iter      SolEst     Damping    Stepsize #Res #Jac #Sol   LinErr   LinRes
   1         0.4   0.3500000         0.4   18   13   13  1.3e-13  4.1e-15
   2        0.27   0.3500000        0.27   19   14   14    9e-14  2.8e-15
   3        0.18   0.3500000        0.18   20   15   15  1.1e-13  3.6e-15
Velocity u2, Pressure p
Iter      SolEst     Damping    Stepsize #Res #Jac #Sol   LinErr   LinRes
   1       0.035   0.5000000       0.035   10    5    5  2.1e-15  7.7e-15
Displacement field
Scales for dependent variables:
Auxiliary pressure (comp1.solid.lemm1.pw): 0.35
Displacement field (comp1.u): 2.8e-16
Iter      SolEst     Damping    Stepsize #Res #Jac #Sol   LinErr   LinRes
   1        0.17   0.5000000        0.17   10    5    5  1.3e-13  1.2e-12
Spatial mesh displacement
Iter      SolEst     Damping    Stepsize #Res #Jac #Sol   LinErr   LinRes
   1     7.7e-07   0.5000000     7.7e-07   10    5    5  1.3e-15  1.8e-15
Solution error estimates for segregated groups
0.3, 0.017, 0.084, 3.9e-07
Residual error estimates for segregated groups
32, 2.3e+03, 6.8e+03, 0.13
Segregated solver iteration 6.
Merged variables
Iter      SolEst     Damping    Stepsize #Res #Jac #Sol   LinErr   LinRes
   1        0.12   0.3500000        0.12   22   16   16    4e-14  2.8e-15
   2       0.076   0.3500000       0.076   23   17   17  1.2e-13  3.4e-15
   3        0.05   0.3500000        0.05   24   18   18    8e-14  3.6e-15
Velocity u2, Pressure p
Iter      SolEst     Damping    Stepsize #Res #Jac #Sol   LinErr   LinRes
   1       0.017   0.5000000       0.017   12    6    6  2.6e-15  7.9e-15
Displacement field
Iter      SolEst     Damping    Stepsize #Res #Jac #Sol   LinErr   LinRes
   1       0.096   0.5000000       0.096   12    6    6  1.2e-13  1.2e-12
Spatial mesh displacement
Iter      SolEst     Damping    Stepsize #Res #Jac #Sol   LinErr   LinRes
   1     5.4e-07   0.5000000     5.4e-07   12    6    6  1.8e-15  1.8e-15
Solution error estimates for segregated groups
```

```
0.085, 0.0086, 0.048, 2.7e-07
Residual error estimates for segregated groups
12, 1.1e+03, 4.2e+03, 0.13
Segregated solver iteration 7.
Merged variables
Iter      SolEst     Damping    Stepsize #Res #Jac #Sol   LinErr   LinRes
   1       0.032   0.3500000       0.032   26   19   19    1e-13  5.1e-15
   2       0.021   0.3500000       0.021   27   20   20  8.2e-14  3.2e-15
   3       0.014   0.3500000       0.014   28   21   21  1.1e-13  3.2e-15
Velocity u2, Pressure p
Iter      SolEst     Damping    Stepsize #Res #Jac #Sol   LinErr   LinRes
   1      0.0085   0.5000000      0.0085   14    7    7    2e-15  7.8e-15
Displacement field
Iter      SolEst     Damping    Stepsize #Res #Jac #Sol   LinErr   LinRes
   1       0.054   0.5000000       0.054   14    7    7  1.5e-13  1.2e-12
Spatial mesh displacement
Iter      SolEst     Damping    Stepsize #Res #Jac #Sol   LinErr   LinRes
   1     3.6e-07   0.5000000     3.6e-07   14    7    7  2.2e-15  1.8e-15
Solution error estimates for segregated groups
0.023, 0.0042, 0.027, 1.8e-07
Residual error estimates for segregated groups
4.1, 5.8e+02, 2.6e+03, 0.12
Segregated solver iteration 8.
Merged variables
Iter      SolEst     Damping    Stepsize #Res #Jac #Sol   LinErr   LinRes
   1      0.0089   0.3500000      0.0089   30   22   22  7.8e-14  4.4e-15
   2      0.0058   0.3500000      0.0058   31   23   23  6.7e-14    3e-15
   3      0.0038   0.3500000      0.0038   32   24   24  1.2e-13  2.9e-15
Velocity u2, Pressure p
Iter      SolEst     Damping    Stepsize #Res #Jac #Sol   LinErr   LinRes
   1      0.0042   0.5000000      0.0042   16    8    8  2.7e-15  7.9e-15
Displacement field
Iter      SolEst     Damping    Stepsize #Res #Jac #Sol   LinErr   LinRes
   1        0.03   0.5000000        0.03   16    8    8  1.8e-13  1.2e-12
Spatial mesh displacement
Iter      SolEst     Damping    Stepsize #Res #Jac #Sol   LinErr   LinRes
   1     2.3e-07   0.5000000     2.3e-07   16    8    8  1.8e-15  1.8e-15
Solution error estimates for segregated groups
0.0065, 0.0021, 0.015, 1.2e-07
Residual error estimates for segregated groups
1.3, 2.9e+02, 1.8e+03, 0.13
Segregated solver iteration 9.
Merged variables
Iter      SolEst     Damping    Stepsize #Res #Jac #Sol   LinErr   LinRes
   1      0.0024   0.3500000      0.0024   34   25   25  7.9e-14  2.8e-15
   2      0.0016   0.3500000      0.0016   35   26   26  6.9e-14  5.1e-15
   3       0.001   0.3500000       0.001   36   27   27  9.3e-14  3.7e-15
Velocity u2, Pressure p
Iter      SolEst     Damping    Stepsize #Res #Jac #Sol   LinErr   LinRes
   1      0.0021   0.5000000      0.0021   18    9    9    3e-15  7.8e-15
Displacement field
Iter      SolEst     Damping    Stepsize #Res #Jac #Sol   LinErr   LinRes
   1       0.017   0.5000000       0.017   18    9    9  1.5e-13  1.2e-12
Spatial mesh displacement
Iter      SolEst     Damping    Stepsize #Res #Jac #Sol   LinErr   LinRes
   1     1.4e-07   0.5000000     1.4e-07   18    9    9  1.7e-15  1.8e-15
```

```
Solution error estimates for segregated groups
0.0018, 0.0011, 0.0085, 7.2e-08
Residual error estimates for segregated groups
0.44, 1.4e+02, 1.4e+03, 0.13
Segregated solver iteration 10.
Merged variables
Iter      SolEst     Damping    Stepsize #Res #Jac #Sol   LinErr   LinRes
   1     0.00067   0.3500000     0.00067   38   28   28    9e-14  3.1e-15
   2     0.00044   0.3500000     0.00044   39   29   29  1.1e-13  3.3e-15
   3     0.00028   0.3500000     0.00028   40   30   30  8.9e-14  2.9e-15
Velocity u2, Pressure p
Iter      SolEst     Damping    Stepsize #Res #Jac #Sol   LinErr   LinRes
   1      0.0011   0.5000000      0.0011   20   10   10  3.2e-15  7.9e-15
Displacement field
Iter      SolEst     Damping    Stepsize #Res #Jac #Sol   LinErr   LinRes
   1      0.0094   0.5000000      0.0094   20   10   10    1e-13  1.2e-12
Spatial mesh displacement
Iter      SolEst     Damping    Stepsize #Res #Jac #Sol   LinErr   LinRes
   1     8.9e-08   0.5000000     8.9e-08   20   10   10  1.5e-15  1.8e-15
Solution error estimates for segregated groups
0.00049, 0.00053, 0.0047, 4.4e-08
Residual error estimates for segregated groups
0.14, 72, 1.2e+03, 0.12
Segregated solver iteration 11.
Merged variables
Iter      SolEst     Damping    Stepsize #Res #Jac #Sol   LinErr   LinRes
   1     0.00018   0.3500000     0.00018   42   31   31  7.1e-14  3.7e-15
   2     0.00012   0.3500000     0.00012   43   32   32  3.4e-14  5.5e-15
   3     7.8e-05   0.3500000     7.8e-05   44   33   33    8e-14  3.2e-15
Velocity u2, Pressure p
Iter      SolEst     Damping    Stepsize #Res #Jac #Sol   LinErr   LinRes
   1     0.00053   0.5000000     0.00053   22   11   11    4e-15  7.8e-15
Displacement field
Iter      SolEst     Damping    Stepsize #Res #Jac #Sol   LinErr   LinRes
   1      0.0051   0.5000000      0.0051   22   11   11  1.2e-13  1.1e-12
Spatial mesh displacement
Iter      SolEst     Damping    Stepsize #Res #Jac #Sol   LinErr   LinRes
   1     5.3e-08   0.5000000     5.3e-08   22   11   11  1.6e-15  1.8e-15
Solution error estimates for segregated groups
0.00013, 0.00026, 0.0026, 2.7e-08
Residual error estimates for segregated groups
0.043, 36, 1.2e+03, 0.13
Segregated solver iteration 12.
Merged variables
Iter      SolEst     Damping    Stepsize #Res #Jac #Sol   LinErr   LinRes
   1     5.1e-05   0.3500000     5.1e-05   46   34   34    1e-13  4.2e-15
   2     3.3e-05   0.3500000     3.3e-05   47   35   35  1.9e-13  4.7e-15
   3     2.1e-05   0.3500000     2.1e-05   48   36   36  1.4e-13  2.8e-15
Velocity u2, Pressure p
Iter      SolEst     Damping    Stepsize #Res #Jac #Sol   LinErr   LinRes
   1     0.00026   0.5000000     0.00026   24   12   12  2.8e-15  7.8e-15
Displacement field
Iter      SolEst     Damping    Stepsize #Res #Jac #Sol   LinErr   LinRes
   1      0.0028   0.5000000      0.0028   24   12   12  1.2e-13  1.1e-12
Spatial mesh displacement
Iter      SolEst     Damping    Stepsize #Res #Jac #Sol   LinErr   LinRes
```

```
   1     3.1e-08   0.5000000     3.1e-08   24    12    12  1.5e-15  1.8e-15
Solution error estimates for segregated groups
3.7e-05, 0.00013, 0.0014, 1.6e-08
Residual error estimates for segregated groups
0.013, 18, 1.1e+03, 0.12
Segregated solver iteration 13.
Merged variables
Iter      SolEst      Damping    Stepsize #Res #Jac #Sol   LinErr   LinRes
   1     1.4e-05   0.3500000     1.4e-05   50    37    37    9e-14  2.7e-15
   2       9e-06   0.3500000       9e-06   51    38    38  8.6e-14  3.7e-15
   3     5.9e-06   0.3500000     5.9e-06   52    39    39  9.6e-14    3e-15
Velocity u2, Pressure p
Iter      SolEst      Damping    Stepsize #Res #Jac #Sol   LinErr   LinRes
   1     0.00013   0.5000000     0.00013   26    13    13  1.9e-15  7.9e-15
Displacement field
Iter      SolEst      Damping    Stepsize #Res #Jac #Sol   LinErr   LinRes
   1      0.0015   0.5000000      0.0015   26    13    13  1.1e-13  1.2e-12
Spatial mesh displacement
Iter      SolEst      Damping    Stepsize #Res #Jac #Sol   LinErr   LinRes
   1     1.8e-08   0.5000000     1.8e-08   26    13    13  2.1e-15  1.8e-15
Solution error estimates for segregated groups
1e-05, 6.6e-05, 0.00076, 9.2e-09
Residual error estimates for segregated groups
0.004, 9, 1.2e+03, 0.12
Solution time: 155 s. (2 minutes, 35 seconds)
Physical memory: 4.74 GB
Virtual memory: 26.03 GB
Ended at Jan 31, 2025, 9:23:03 PM.
----- Stationary Solver 1 in study1/Approach Curve Solver (sol1) -------------->
```

d=2005 (su101)

GENERAL

Description	Value
Solution	d=2005

LOG

```
<---- Compile Equations: Stationary in study1/Approach Curve Solver (sol1) -----
Started at Jan 31, 2025, 9:23:03 PM.
Parameter d = 2.0050000000000003E-6 (m).
Geometry shape function: Linear serendipity
Number of vertex elements: 18
Number of boundary elements: 6069
Number of elements: 153149
Minimum element quality: 0.4853
Time: 8 s.
Physical memory: 3.09 GB
Virtual memory: 15.27 GB
Ended at Jan 31, 2025, 9:23:11 PM.
----- Compile Equations: Stationary in study1/Approach Curve Solver (sol1) ---->

<---- Dependent Variables 1 in study1/Approach Curve Solver (sol1) -------------
Started at Jan 31, 2025, 9:23:11 PM.
Solution time: 0 s.
Physical memory: 3.09 GB
Virtual memory: 15.27 GB
Ended at Jan 31, 2025, 9:23:11 PM.
----- Dependent Variables 1 in study1/Approach Curve Solver (sol1) ------------>

<---- Stationary Solver 1 in study1/Approach Curve Solver (sol1) ---------------
Started at Jan 31, 2025, 9:23:11 PM.
Segregated solver
Segregated Step 11 does not have any components solved for.
Number of degrees of freedom solved for: 776729 (plus 11561 internal DOFs).
Segregated solver iteration 1.
Merged variables
Nonsymmetric matrix found.
Scales for dependent variables:
Concentration (comp1.Cl): 1.4e+02
Concentration (comp1.K): 1.4e+02
Electric potential (comp1.V): 1.9e+02
Orthonormal null-space function used.
Iter      SolEst     Damping    Stepsize #Res #Jac #Sol   LinErr   LinRes
   1        0.82   0.3500000        0.82    2    1    1  2.3e-11  2.7e-15
   2        0.81   0.3500000        0.81    3    2    2    6e-11  2.8e-15
   3         0.8   0.3500000         0.8    4    3    3  3.4e-11  2.6e-15
Velocity u2, Pressure p
Nonsymmetric matrix found.
Scales for dependent variables:
Pressure (comp1.p): 1
Velocity field (spatial frame) (comp1.u2): 0.00018
Orthonormal null-space function used.
Iter      SolEst     Damping    Stepsize #Res #Jac #Sol   LinErr   LinRes
   1         1.1   0.5000000         1.1    2    1    1  1.8e-15  2.3e-15
Displacement field
Nonsymmetric matrix found.
Scales for dependent variables:
Auxiliary pressure (comp1.solid.lemm1.pw): 1
Displacement field (comp1.u): 2.8e-18
Orthonormal null-space function used.
Iter      SolEst     Damping    Stepsize #Res #Jac #Sol   LinErr   LinRes
   1         1.9   0.5000000         1.9    2    1    1  2.7e-14  1.4e-12
Spatial mesh displacement
```

```
Symmetric matrices found.
Scales for dependent variables:
Spatial mesh displacement (comp1.spatial.disp): 1.2e-09
Orthonormal null-space function used.
Iter      SolEst     Damping    Stepsize #Res #Jac #Sol   LinErr   LinRes
   1     8.3e-07   0.5000000     8.3e-07    2    1    1  1.3e-15  1.8e-15
Solution error estimates for segregated groups
1.4, 0.54, 0.95, 4.1e-07
Residual error estimates for segregated groups
7.8e+03, 8.1e+04, 2.1e+04, 0.11
Segregated solver iteration 2.
Merged variables
Scales for dependent variables:
Concentration (comp1.Cl): 1.4e+02
Concentration (comp1.K): 1.4e+02
Electric potential (comp1.V): 0.064
Iter      SolEst     Damping    Stepsize #Res #Jac #Sol   LinErr   LinRes
   1        0.78   0.3500000        0.78    6    4    4  7.3e-14  4.3e-15
   2        0.77   0.3500000        0.77    7    5    5  7.4e-14  3.1e-15
   3        0.75   0.3500000        0.75    8    6    6  9.8e-14  4.5e-15
Velocity u2, Pressure p
Scales for dependent variables:
Pressure (comp1.p): 3.7e+02
Velocity field (spatial frame) (comp1.u2): 0.00074
Iter      SolEst     Damping    Stepsize #Res #Jac #Sol   LinErr   LinRes
   1        0.36   0.5000000        0.36    4    2    2  3.2e-15  7.8e-15
Displacement field
Iter      SolEst     Damping    Stepsize #Res #Jac #Sol   LinErr   LinRes
   1        0.96   0.5000000        0.96    4    2    2  6.4e-14  1.4e-12
Spatial mesh displacement
Iter      SolEst     Damping    Stepsize #Res #Jac #Sol   LinErr   LinRes
   1     1.2e-06   0.5000000     1.2e-06    4    2    2  1.4e-15  1.8e-15
Solution error estimates for segregated groups
1.3, 0.18, 0.48, 6.2e-07
Residual error estimates for segregated groups
7.5e+02, 1.4e+04, 2.1e+04, 0.11
Segregated solver iteration 3.
Merged variables
Iter      SolEst     Damping    Stepsize #Res #Jac #Sol   LinErr   LinRes
   1        0.74   0.3500000        0.74   10    7    7  8.9e-14  3.3e-15
   2        0.73   0.3500000        0.73   11    8    8    9e-14  3.2e-15
   3        0.71   0.3500000        0.71   12    9    9  9.6e-14  2.9e-15
Velocity u2, Pressure p
Iter      SolEst     Damping    Stepsize #Res #Jac #Sol   LinErr   LinRes
   1        0.15   0.5000000        0.15    6    3    3  3.1e-15  7.9e-15
Displacement field
Iter      SolEst     Damping    Stepsize #Res #Jac #Sol   LinErr   LinRes
   1        0.52   0.5000000        0.52    6    3    3  8.6e-14  1.5e-12
Spatial mesh displacement
Iter      SolEst     Damping    Stepsize #Res #Jac #Sol   LinErr   LinRes
   1     1.2e-06   0.5000000     1.2e-06    6    3    3  1.8e-15  1.8e-15
Solution error estimates for segregated groups
1.2, 0.077, 0.26, 6.2e-07
Residual error estimates for segregated groups
2.1e+02, 8e+03, 1.6e+04, 0.13
Segregated solver iteration 4.
```

```
Merged variables
Iter      SolEst     Damping    Stepsize #Res #Jac #Sol   LinErr   LinRes
   1        0.69   0.3500000        0.69   14   10   10    1e-13  3.3e-15
   2        0.67   0.3500000        0.67   15   11   11    1e-13  3.7e-15
   3        0.58   0.3500000        0.58   16   12   12  1.1e-13  3.2e-15
Velocity u2, Pressure p
Iter      SolEst     Damping    Stepsize #Res #Jac #Sol   LinErr   LinRes
   1       0.072   0.5000000       0.072    8    4    4  2.8e-15  7.8e-15
Displacement field
Iter      SolEst     Damping    Stepsize #Res #Jac #Sol   LinErr   LinRes
   1        0.29   0.5000000        0.29    8    4    4  6.6e-14  1.4e-12
Spatial mesh displacement
Iter      SolEst     Damping    Stepsize #Res #Jac #Sol   LinErr   LinRes
   1       1e-06   0.5000000       1e-06    8    4    4  1.7e-15  1.8e-15
Solution error estimates for segregated groups
0.99, 0.036, 0.15, 5.2e-07
Residual error estimates for segregated groups
81, 4.2e+03, 1.1e+04, 0.13
Segregated solver iteration 5.
Merged variables
Iter      SolEst     Damping    Stepsize #Res #Jac #Sol   LinErr   LinRes
   1         0.4   0.3500000         0.4   18   13   13  8.5e-14  4.6e-15
   2        0.27   0.3500000        0.27   19   14   14  6.7e-14    3e-15
   3        0.18   0.3500000        0.18   20   15   15  1.5e-13  3.2e-15
Velocity u2, Pressure p
Iter      SolEst     Damping    Stepsize #Res #Jac #Sol   LinErr   LinRes
   1       0.035   0.5000000       0.035   10    5    5    4e-15  7.9e-15
Displacement field
Scales for dependent variables:
Auxiliary pressure (comp1.solid.lemm1.pw): 0.35
Displacement field (comp1.u): 2.8e-16
Iter      SolEst     Damping    Stepsize #Res #Jac #Sol   LinErr   LinRes
   1        0.17   0.5000000        0.17   10    5    5  1.4e-13  1.1e-12
Spatial mesh displacement
Iter      SolEst     Damping    Stepsize #Res #Jac #Sol   LinErr   LinRes
   1     7.7e-07   0.5000000     7.7e-07   10    5    5  1.2e-15  1.8e-15
Solution error estimates for segregated groups
0.3, 0.017, 0.084, 3.9e-07
Residual error estimates for segregated groups
32, 2.2e+03, 6.8e+03, 0.12
Segregated solver iteration 6.
Merged variables
Iter      SolEst     Damping    Stepsize #Res #Jac #Sol   LinErr   LinRes
   1        0.12   0.3500000        0.12   22   16   16  7.4e-14  3.9e-15
   2       0.075   0.3500000       0.075   23   17   17  8.4e-14    3e-15
   3       0.049   0.3500000       0.049   24   18   18  1.2e-13  3.2e-15
Velocity u2, Pressure p
Iter      SolEst     Damping    Stepsize #Res #Jac #Sol   LinErr   LinRes
   1       0.017   0.5000000       0.017   12    6    6  2.1e-15  7.8e-15
Displacement field
Iter      SolEst     Damping    Stepsize #Res #Jac #Sol   LinErr   LinRes
   1       0.096   0.5000000       0.096   12    6    6  1.1e-13  1.1e-12
Spatial mesh displacement
Iter      SolEst     Damping    Stepsize #Res #Jac #Sol   LinErr   LinRes
   1     5.4e-07   0.5000000     5.4e-07   12    6    6  1.8e-15  1.8e-15
Solution error estimates for segregated groups
```

```
0.085, 0.0086, 0.048, 2.7e-07
Residual error estimates for segregated groups
12, 1.1e+03, 4.2e+03, 0.12
Segregated solver iteration 7.
Merged variables
Iter      SolEst     Damping    Stepsize #Res #Jac #Sol   LinErr   LinRes
   1       0.032   0.3500000       0.032   26   19   19  1.8e-13  4.2e-15
   2       0.021   0.3500000       0.021   27   20   20  8.6e-14    4e-15
   3       0.014   0.3500000       0.014   28   21   21    1e-13  3.8e-15
Velocity u2, Pressure p
Iter      SolEst     Damping    Stepsize #Res #Jac #Sol   LinErr   LinRes
   1      0.0085   0.5000000      0.0085   14    7    7  1.9e-15  7.7e-15
Displacement field
Iter      SolEst     Damping    Stepsize #Res #Jac #Sol   LinErr   LinRes
   1       0.054   0.5000000       0.054   14    7    7  9.4e-14  1.2e-12
Spatial mesh displacement
Iter      SolEst     Damping    Stepsize #Res #Jac #Sol   LinErr   LinRes
   1     3.6e-07   0.5000000     3.6e-07   14    7    7  1.8e-15  1.8e-15
Solution error estimates for segregated groups
0.023, 0.0042, 0.027, 1.8e-07
Residual error estimates for segregated groups
4, 5.6e+02, 2.6e+03, 0.13
Segregated solver iteration 8.
Merged variables
Iter      SolEst     Damping    Stepsize #Res #Jac #Sol   LinErr   LinRes
   1      0.0088   0.3500000      0.0088   30   22   22  9.7e-14    3e-15
   2      0.0058   0.3500000      0.0058   31   23   23  1.4e-13    3e-15
   3      0.0037   0.3500000      0.0037   32   24   24  6.1e-14  3.3e-15
Velocity u2, Pressure p
Iter      SolEst     Damping    Stepsize #Res #Jac #Sol   LinErr   LinRes
   1      0.0042   0.5000000      0.0042   16    8    8  2.7e-15  7.8e-15
Displacement field
Iter      SolEst     Damping    Stepsize #Res #Jac #Sol   LinErr   LinRes
   1        0.03   0.5000000        0.03   16    8    8  1.3e-13  1.2e-12
Spatial mesh displacement
Iter      SolEst     Damping    Stepsize #Res #Jac #Sol   LinErr   LinRes
   1     2.3e-07   0.5000000     2.3e-07   16    8    8  1.4e-15  1.8e-15
Solution error estimates for segregated groups
0.0064, 0.0021, 0.015, 1.2e-07
Residual error estimates for segregated groups
1.3, 2.8e+02, 1.8e+03, 0.13
Segregated solver iteration 9.
Merged variables
Iter      SolEst     Damping    Stepsize #Res #Jac #Sol   LinErr   LinRes
   1      0.0024   0.3500000      0.0024   34   25   25    1e-13    3e-15
   2      0.0016   0.3500000      0.0016   35   26   26  7.6e-14  3.4e-15
   3       0.001   0.3500000       0.001   36   27   27  1.3e-13  3.1e-15
Velocity u2, Pressure p
Iter      SolEst     Damping    Stepsize #Res #Jac #Sol   LinErr   LinRes
   1      0.0021   0.5000000      0.0021   18    9    9  4.1e-15  7.8e-15
Displacement field
Iter      SolEst     Damping    Stepsize #Res #Jac #Sol   LinErr   LinRes
   1       0.017   0.5000000       0.017   18    9    9  1.9e-13  1.2e-12
Spatial mesh displacement
Iter      SolEst     Damping    Stepsize #Res #Jac #Sol   LinErr   LinRes
   1     1.5e-07   0.5000000     1.5e-07   18    9    9  1.4e-15  1.8e-15
```

```
Solution error estimates for segregated groups
0.0018, 0.0011, 0.0085, 7.3e-08
Residual error estimates for segregated groups
0.43, 1.4e+02, 1.4e+03, 0.13
Segregated solver iteration 10.
Merged variables
Iter      SolEst     Damping    Stepsize #Res #Jac #Sol   LinErr   LinRes
   1     0.00067   0.3500000     0.00067   38   28   28  1.3e-13    3e-15
   2     0.00043   0.3500000     0.00043   39   29   29  7.9e-14    3e-15
   3     0.00028   0.3500000     0.00028   40   30   30    1e-13  4.1e-15
Velocity u2, Pressure p
Iter      SolEst     Damping    Stepsize #Res #Jac #Sol   LinErr   LinRes
   1      0.0011   0.5000000      0.0011   20   10   10  2.2e-15  7.8e-15
Displacement field
Iter      SolEst     Damping    Stepsize #Res #Jac #Sol   LinErr   LinRes
   1      0.0094   0.5000000      0.0094   20   10   10    2e-13  1.1e-12
Spatial mesh displacement
Iter      SolEst     Damping    Stepsize #Res #Jac #Sol   LinErr   LinRes
   1     8.9e-08   0.5000000     8.9e-08   20   10   10  1.6e-15  1.8e-15
Solution error estimates for segregated groups
0.00048, 0.00053, 0.0047, 4.4e-08
Residual error estimates for segregated groups
0.14, 70, 1.2e+03, 0.12
Segregated solver iteration 11.
Merged variables
Iter      SolEst     Damping    Stepsize #Res #Jac #Sol   LinErr   LinRes
   1     0.00018   0.3500000     0.00018   42   31   31  8.2e-14  3.3e-15
   2     0.00012   0.3500000     0.00012   43   32   32  6.8e-14  3.9e-15
   3     7.8e-05   0.3500000     7.8e-05   44   33   33    1e-13  5.4e-15
Velocity u2, Pressure p
Iter      SolEst     Damping    Stepsize #Res #Jac #Sol   LinErr   LinRes
   1     0.00053   0.5000000     0.00053   22   11   11  2.8e-15  7.9e-15
Displacement field
Iter      SolEst     Damping    Stepsize #Res #Jac #Sol   LinErr   LinRes
   1      0.0051   0.5000000      0.0051   22   11   11  1.3e-13  1.2e-12
Spatial mesh displacement
Iter      SolEst     Damping    Stepsize #Res #Jac #Sol   LinErr   LinRes
   1     5.3e-08   0.5000000     5.3e-08   22   11   11  1.6e-15  1.8e-15
Solution error estimates for segregated groups
0.00013, 0.00026, 0.0026, 2.7e-08
Residual error estimates for segregated groups
0.043, 35, 1.2e+03, 0.12
Segregated solver iteration 12.
Merged variables
Iter      SolEst     Damping    Stepsize #Res #Jac #Sol   LinErr   LinRes
   1       5e-05   0.3500000       5e-05   46   34   34  1.5e-13  3.5e-15
   2     3.3e-05   0.3500000     3.3e-05   47   35   35  9.3e-14  3.3e-15
   3     2.1e-05   0.3500000     2.1e-05   48   36   36  9.1e-14  2.9e-15
Velocity u2, Pressure p
Iter      SolEst     Damping    Stepsize #Res #Jac #Sol   LinErr   LinRes
   1     0.00026   0.5000000     0.00026   24   12   12    5e-15  7.8e-15
Displacement field
Iter      SolEst     Damping    Stepsize #Res #Jac #Sol   LinErr   LinRes
   1      0.0028   0.5000000      0.0028   24   12   12  1.3e-13  1.2e-12
Spatial mesh displacement
Iter      SolEst     Damping    Stepsize #Res #Jac #Sol   LinErr   LinRes
```

```
   1     3.1e-08   0.5000000     3.1e-08   24    12    12  1.3e-15  1.8e-15
Solution error estimates for segregated groups
3.7e-05, 0.00013, 0.0014, 1.6e-08
Residual error estimates for segregated groups
0.013, 17, 1.1e+03, 0.13
Segregated solver iteration 13.
Merged variables
Iter      SolEst     Damping    Stepsize #Res #Jac #Sol   LinErr   LinRes
   1     1.4e-05   0.3500000     1.4e-05   50    37    37  8.6e-14  3.3e-15
   2       9e-06   0.3500000       9e-06   51    38    38  1.1e-13  3.2e-15
   3     5.8e-06   0.3500000     5.8e-06   52    39    39  8.7e-14  2.9e-15
Velocity u2, Pressure p
Iter      SolEst     Damping    Stepsize #Res #Jac #Sol   LinErr   LinRes
   1     0.00013   0.5000000     0.00013   26    13    13  3.8e-15  7.8e-15
Displacement field
Iter      SolEst     Damping    Stepsize #Res #Jac #Sol   LinErr   LinRes
   1      0.0015   0.5000000      0.0015   26    13    13  1.1e-13  1.1e-12
Spatial mesh displacement
Iter      SolEst     Damping    Stepsize #Res #Jac #Sol   LinErr   LinRes
   1     1.8e-08   0.5000000     1.8e-08   26    13    13  1.5e-15  1.9e-15
Solution error estimates for segregated groups
1e-05, 6.6e-05, 0.00076, 9.2e-09
Residual error estimates for segregated groups
0.004, 8.7, 1.2e+03, 0.13
Solution time: 152 s. (2 minutes, 32 seconds)
Physical memory: 4.78 GB
Virtual memory: 25.88 GB
Ended at Jan 31, 2025, 9:25:43 PM.
----- Stationary Solver 1 in study1/Approach Curve Solver (sol1) -------------->
```

d=2000 (su102)

GENERAL

Description	Value
Solution	d=2000

LOG

```
<---- Compile Equations: Stationary in study1/Approach Curve Solver (sol1) -----
Started at Jan 31, 2025, 9:25:43 PM.
Parameter d = 2.0000000000000003E-6 (m).
Geometry shape function: Linear serendipity
Number of vertex elements: 18
Number of boundary elements: 6066
Number of elements: 153762
Minimum element quality: 0.4832
Time: 8 s.
Physical memory: 3.06 GB
Virtual memory: 15.23 GB
Ended at Jan 31, 2025, 9:25:51 PM.
----- Compile Equations: Stationary in study1/Approach Curve Solver (sol1) ---->

<---- Dependent Variables 1 in study1/Approach Curve Solver (sol1) -------------
Started at Jan 31, 2025, 9:25:51 PM.
Solution time: 0 s.
Physical memory: 3.06 GB
Virtual memory: 15.23 GB
Ended at Jan 31, 2025, 9:25:51 PM.
----- Dependent Variables 1 in study1/Approach Curve Solver (sol1) ------------>

<---- Stationary Solver 1 in study1/Approach Curve Solver (sol1) ---------------
Started at Jan 31, 2025, 9:25:51 PM.
Segregated solver
Segregated Step 11 does not have any components solved for.
Number of degrees of freedom solved for: 779901 (plus 11555 internal DOFs).
Segregated solver iteration 1.
Merged variables
Nonsymmetric matrix found.
Scales for dependent variables:
Concentration (comp1.Cl): 1.4e+02
Concentration (comp1.K): 1.4e+02
Electric potential (comp1.V): 1.9e+02
Orthonormal null-space function used.
Iter      SolEst     Damping    Stepsize #Res #Jac #Sol   LinErr   LinRes
   1        0.83   0.3500000        0.83    2    1    1  4.5e-11  2.7e-15
   2        0.81   0.3500000        0.81    3    2    2  5.6e-11  2.7e-15
   3         0.8   0.3500000         0.8    4    3    3  4.9e-11  2.7e-15
Velocity u2, Pressure p
Nonsymmetric matrix found.
Scales for dependent variables:
Pressure (comp1.p): 1
Velocity field (spatial frame) (comp1.u2): 0.00018
Orthonormal null-space function used.
Iter      SolEst     Damping    Stepsize #Res #Jac #Sol   LinErr   LinRes
   1         1.1   0.5000000         1.1    2    1    1  1.8e-15  2.2e-15
Displacement field
Nonsymmetric matrix found.
Scales for dependent variables:
Auxiliary pressure (comp1.solid.lemm1.pw): 1
Displacement field (comp1.u): 2.8e-18
Orthonormal null-space function used.
Iter      SolEst     Damping    Stepsize #Res #Jac #Sol   LinErr   LinRes
   1         1.9   0.5000000         1.9    2    1    1  7.4e-14  1.4e-12
Spatial mesh displacement
```

```
Symmetric matrices found.
Scales for dependent variables:
Spatial mesh displacement (comp1.spatial.disp): 1.2e-09
Orthonormal null-space function used.
Iter      SolEst     Damping    Stepsize #Res #Jac #Sol   LinErr   LinRes
   1     8.3e-07   0.5000000     8.3e-07    2    1    1  1.8e-15  1.8e-15
Solution error estimates for segregated groups
1.4, 0.54, 0.95, 4.1e-07
Residual error estimates for segregated groups
7.8e+03, 8.2e+04, 2.1e+04, 0.11
Segregated solver iteration 2.
Merged variables
Scales for dependent variables:
Concentration (comp1.Cl): 1.4e+02
Concentration (comp1.K): 1.4e+02
Electric potential (comp1.V): 0.064
Iter      SolEst     Damping    Stepsize #Res #Jac #Sol   LinErr   LinRes
   1        0.78   0.3500000        0.78    6    4    4    2e-13  7.8e-15
   2        0.77   0.3500000        0.77    7    5    5  1.1e-13  5.1e-15
   3        0.76   0.3500000        0.76    8    6    6  1.3e-13  6.8e-15
Velocity u2, Pressure p
Scales for dependent variables:
Pressure (comp1.p): 3.7e+02
Velocity field (spatial frame) (comp1.u2): 0.00074
Iter      SolEst     Damping    Stepsize #Res #Jac #Sol   LinErr   LinRes
   1        0.36   0.5000000        0.36    4    2    2  3.1e-15    8e-15
Displacement field
Iter      SolEst     Damping    Stepsize #Res #Jac #Sol   LinErr   LinRes
   1        0.95   0.5000000        0.95    4    2    2    6e-14  1.5e-12
Spatial mesh displacement
Iter      SolEst     Damping    Stepsize #Res #Jac #Sol   LinErr   LinRes
   1     1.2e-06   0.5000000     1.2e-06    4    2    2  1.3e-15  1.8e-15
Solution error estimates for segregated groups
1.3, 0.18, 0.48, 6.2e-07
Residual error estimates for segregated groups
7.6e+02, 1.6e+04, 2.1e+04, 0.12
Segregated solver iteration 3.
Merged variables
Iter      SolEst     Damping    Stepsize #Res #Jac #Sol   LinErr   LinRes
   1        0.74   0.3500000        0.74   10    7    7  1.6e-13  5.6e-15
   2        0.73   0.3500000        0.73   11    8    8  2.6e-13  1.3e-14
   3        0.71   0.3500000        0.71   12    9    9  1.2e-13  7.5e-15
Velocity u2, Pressure p
Iter      SolEst     Damping    Stepsize #Res #Jac #Sol   LinErr   LinRes
   1        0.15   0.5000000        0.15    6    3    3  4.1e-15  7.9e-15
Displacement field
Iter      SolEst     Damping    Stepsize #Res #Jac #Sol   LinErr   LinRes
   1        0.52   0.5000000        0.52    6    3    3  7.4e-14  1.5e-12
Spatial mesh displacement
Iter      SolEst     Damping    Stepsize #Res #Jac #Sol   LinErr   LinRes
   1     1.2e-06   0.5000000     1.2e-06    6    3    3  2.4e-15  1.8e-15
Solution error estimates for segregated groups
1.2, 0.077, 0.26, 6.2e-07
Residual error estimates for segregated groups
2.1e+02, 8.9e+03, 1.6e+04, 0.13
Segregated solver iteration 4.
```

```
Merged variables
Iter      SolEst     Damping    Stepsize #Res #Jac #Sol   LinErr   LinRes
   1        0.69   0.3500000        0.69   14   10   10  8.3e-14  5.8e-15
   2        0.67   0.3500000        0.67   15   11   11  6.8e-14  7.4e-15
   3        0.58   0.3500000        0.58   16   12   12  8.8e-14  6.8e-15
Velocity u2, Pressure p
Iter      SolEst     Damping    Stepsize #Res #Jac #Sol   LinErr   LinRes
   1       0.072   0.5000000       0.072    8    4    4  3.3e-15  7.9e-15
Displacement field
Iter      SolEst     Damping    Stepsize #Res #Jac #Sol   LinErr   LinRes
   1        0.29   0.5000000        0.29    8    4    4  1.3e-13  1.5e-12
Spatial mesh displacement
Iter      SolEst     Damping    Stepsize #Res #Jac #Sol   LinErr   LinRes
   1       1e-06   0.5000000       1e-06    8    4    4  1.6e-15  1.8e-15
Solution error estimates for segregated groups
1, 0.036, 0.15, 5.2e-07
Residual error estimates for segregated groups
81, 4.7e+03, 1.1e+04, 0.12
Segregated solver iteration 5.
Merged variables
Iter      SolEst     Damping    Stepsize #Res #Jac #Sol   LinErr   LinRes
   1         0.4   0.3500000         0.4   18   13   13  1.2e-13  4.4e-15
   2        0.27   0.3500000        0.27   19   14   14  1.7e-13  1.1e-14
   3        0.18   0.3500000        0.18   20   15   15  1.8e-13  1.5e-14
Velocity u2, Pressure p
Iter      SolEst     Damping    Stepsize #Res #Jac #Sol   LinErr   LinRes
   1       0.035   0.5000000       0.035   10    5    5  2.8e-15    8e-15
Displacement field
Scales for dependent variables:
Auxiliary pressure (comp1.solid.lemm1.pw): 0.35
Displacement field (comp1.u): 2.8e-16
Iter      SolEst     Damping    Stepsize #Res #Jac #Sol   LinErr   LinRes
   1        0.17   0.5000000        0.17   10    5    5  1.1e-13  1.1e-12
Spatial mesh displacement
Iter      SolEst     Damping    Stepsize #Res #Jac #Sol   LinErr   LinRes
   1     7.8e-07   0.5000000     7.8e-07   10    5    5  1.3e-15  1.8e-15
Solution error estimates for segregated groups
0.3, 0.017, 0.084, 3.9e-07
Residual error estimates for segregated groups
32, 2.4e+03, 6.8e+03, 0.12
Segregated solver iteration 6.
Merged variables
Iter      SolEst     Damping    Stepsize #Res #Jac #Sol   LinErr   LinRes
   1        0.12   0.3500000        0.12   22   16   16  7.1e-14  1.1e-14
   2       0.076   0.3500000       0.076   23   17   17  2.2e-13  9.7e-15
   3        0.05   0.3500000        0.05   24   18   18  1.2e-13    5e-15
Velocity u2, Pressure p
Iter      SolEst     Damping    Stepsize #Res #Jac #Sol   LinErr   LinRes
   1       0.017   0.5000000       0.017   12    6    6    3e-15  7.9e-15
Displacement field
Iter      SolEst     Damping    Stepsize #Res #Jac #Sol   LinErr   LinRes
   1       0.096   0.5000000       0.096   12    6    6  1.7e-13  1.2e-12
Spatial mesh displacement
Iter      SolEst     Damping    Stepsize #Res #Jac #Sol   LinErr   LinRes
   1     5.4e-07   0.5000000     5.4e-07   12    6    6  1.2e-15  1.8e-15
Solution error estimates for segregated groups
```

```
0.085, 0.0086, 0.048, 2.7e-07
Residual error estimates for segregated groups
12, 1.2e+03, 4.2e+03, 0.13
Segregated solver iteration 7.
Merged variables
Iter      SolEst     Damping    Stepsize #Res #Jac #Sol   LinErr   LinRes
   1       0.032   0.3500000       0.032   26   19   19  2.8e-13  5.2e-15
   2       0.021   0.3500000       0.021   27   20   20  7.7e-14  1.2e-14
   3       0.014   0.3500000       0.014   28   21   21  8.8e-14  5.4e-15
Velocity u2, Pressure p
Iter      SolEst     Damping    Stepsize #Res #Jac #Sol   LinErr   LinRes
   1      0.0085   0.5000000      0.0085   14    7    7  2.2e-15  7.8e-15
Displacement field
Iter      SolEst     Damping    Stepsize #Res #Jac #Sol   LinErr   LinRes
   1       0.054   0.5000000       0.054   14    7    7  1.3e-13  1.2e-12
Spatial mesh displacement
Iter      SolEst     Damping    Stepsize #Res #Jac #Sol   LinErr   LinRes
   1     3.6e-07   0.5000000     3.6e-07   14    7    7    2e-15  1.9e-15
Solution error estimates for segregated groups
0.023, 0.0042, 0.027, 1.8e-07
Residual error estimates for segregated groups
4.1, 6.2e+02, 2.6e+03, 0.12
Segregated solver iteration 8.
Merged variables
Iter      SolEst     Damping    Stepsize #Res #Jac #Sol   LinErr   LinRes
   1      0.0089   0.3500000      0.0089   30   22   22  5.9e-14  1.2e-14
   2      0.0058   0.3500000      0.0058   31   23   23  2.4e-13  6.3e-15
   3      0.0038   0.3500000      0.0038   32   24   24  2.3e-13  5.2e-15
Velocity u2, Pressure p
Iter      SolEst     Damping    Stepsize #Res #Jac #Sol   LinErr   LinRes
   1      0.0042   0.5000000      0.0042   16    8    8  3.3e-15  7.8e-15
Displacement field
Iter      SolEst     Damping    Stepsize #Res #Jac #Sol   LinErr   LinRes
   1        0.03   0.5000000        0.03   16    8    8  1.1e-13  1.2e-12
Spatial mesh displacement
Iter      SolEst     Damping    Stepsize #Res #Jac #Sol   LinErr   LinRes
   1     2.3e-07   0.5000000     2.3e-07   16    8    8  1.5e-15  1.8e-15
Solution error estimates for segregated groups
0.0065, 0.0021, 0.015, 1.2e-07
Residual error estimates for segregated groups
1.3, 3.1e+02, 1.8e+03, 0.13
Segregated solver iteration 9.
Merged variables
Iter      SolEst     Damping    Stepsize #Res #Jac #Sol   LinErr   LinRes
   1      0.0024   0.3500000      0.0024   34   25   25  2.1e-13  6.3e-15
   2      0.0016   0.3500000      0.0016   35   26   26  1.5e-13  6.2e-15
   3       0.001   0.3500000       0.001   36   27   27  2.2e-13  1.1e-14
Velocity u2, Pressure p
Iter      SolEst     Damping    Stepsize #Res #Jac #Sol   LinErr   LinRes
   1      0.0021   0.5000000      0.0021   18    9    9  4.7e-15  7.8e-15
Displacement field
Iter      SolEst     Damping    Stepsize #Res #Jac #Sol   LinErr   LinRes
   1       0.017   0.5000000       0.017   18    9    9  2.1e-13  1.2e-12
Spatial mesh displacement
Iter      SolEst     Damping    Stepsize #Res #Jac #Sol   LinErr   LinRes
   1     1.5e-07   0.5000000     1.5e-07   18    9    9  1.4e-15  1.8e-15
```

```
Solution error estimates for segregated groups
0.0018, 0.0011, 0.0085, 7.3e-08
Residual error estimates for segregated groups
0.44, 1.6e+02, 1.4e+03, 0.12
Segregated solver iteration 10.
Merged variables
Iter      SolEst     Damping    Stepsize #Res #Jac #Sol   LinErr   LinRes
   1     0.00067   0.3500000     0.00067   38   28   28  1.3e-13  7.3e-15
   2     0.00044   0.3500000     0.00044   39   29   29  8.8e-14  4.2e-15
   3     0.00028   0.3500000     0.00028   40   30   30  3.2e-13  1.3e-14
Velocity u2, Pressure p
Iter      SolEst     Damping    Stepsize #Res #Jac #Sol   LinErr   LinRes
   1      0.0011   0.5000000      0.0011   20   10   10  2.3e-15    8e-15
Displacement field
Iter      SolEst     Damping    Stepsize #Res #Jac #Sol   LinErr   LinRes
   1      0.0094   0.5000000      0.0094   20   10   10  1.3e-13  1.2e-12
Spatial mesh displacement
Iter      SolEst     Damping    Stepsize #Res #Jac #Sol   LinErr   LinRes
   1     8.9e-08   0.5000000     8.9e-08   20   10   10  1.6e-15  1.8e-15
Solution error estimates for segregated groups
0.00049, 0.00053, 0.0047, 4.5e-08
Residual error estimates for segregated groups
0.14, 78, 1.2e+03, 0.12
Segregated solver iteration 11.
Merged variables
Iter      SolEst     Damping    Stepsize #Res #Jac #Sol   LinErr   LinRes
   1     0.00018   0.3500000     0.00018   42   31   31  1.7e-13  9.5e-15
   2     0.00012   0.3500000     0.00012   43   32   32  1.7e-13  6.7e-15
   3     7.8e-05   0.3500000     7.8e-05   44   33   33  1.3e-13  9.7e-15
Velocity u2, Pressure p
Iter      SolEst     Damping    Stepsize #Res #Jac #Sol   LinErr   LinRes
   1     0.00053   0.5000000     0.00053   22   11   11  2.4e-15  7.8e-15
Displacement field
Iter      SolEst     Damping    Stepsize #Res #Jac #Sol   LinErr   LinRes
   1      0.0051   0.5000000      0.0051   22   11   11  1.3e-13  1.2e-12
Spatial mesh displacement
Iter      SolEst     Damping    Stepsize #Res #Jac #Sol   LinErr   LinRes
   1     5.3e-08   0.5000000     5.3e-08   22   11   11  1.5e-15  1.8e-15
Solution error estimates for segregated groups
0.00013, 0.00026, 0.0026, 2.7e-08
Residual error estimates for segregated groups
0.043, 39, 1.2e+03, 0.13
Segregated solver iteration 12.
Merged variables
Iter      SolEst     Damping    Stepsize #Res #Jac #Sol   LinErr   LinRes
   1     5.1e-05   0.3500000     5.1e-05   46   34   34  2.1e-13  6.6e-15
   2     3.3e-05   0.3500000     3.3e-05   47   35   35  1.4e-13  4.7e-15
   3     2.1e-05   0.3500000     2.1e-05   48   36   36  7.9e-14  5.2e-15
Velocity u2, Pressure p
Iter      SolEst     Damping    Stepsize #Res #Jac #Sol   LinErr   LinRes
   1     0.00026   0.5000000     0.00026   24   12   12  3.3e-15  7.9e-15
Displacement field
Iter      SolEst     Damping    Stepsize #Res #Jac #Sol   LinErr   LinRes
   1      0.0028   0.5000000      0.0028   24   12   12  1.6e-13  1.2e-12
Spatial mesh displacement
Iter      SolEst     Damping    Stepsize #Res #Jac #Sol   LinErr   LinRes
```

```
   1     3.2e-08   0.5000000     3.2e-08   24    12    12  1.9e-15  1.9e-15
Solution error estimates for segregated groups
3.7e-05, 0.00013, 0.0014, 1.6e-08
Residual error estimates for segregated groups
0.013, 19, 1.2e+03, 0.12
Segregated solver iteration 13.
Merged variables
Iter      SolEst     Damping    Stepsize #Res #Jac #Sol   LinErr   LinRes
   1     1.4e-05   0.3500000     1.4e-05   50    37    37  5.1e-14  1.5e-14
   2       9e-06   0.3500000       9e-06   51    38    38  2.3e-13  6.2e-15
   3     5.9e-06   0.3500000     5.9e-06   52    39    39  1.7e-13  7.2e-15
Velocity u2, Pressure p
Iter      SolEst     Damping    Stepsize #Res #Jac #Sol   LinErr   LinRes
   1     0.00013   0.5000000     0.00013   26    13    13  1.9e-15  7.8e-15
Displacement field
Iter      SolEst     Damping    Stepsize #Res #Jac #Sol   LinErr   LinRes
   1      0.0015   0.5000000      0.0015   26    13    13    1e-13  1.2e-12
Spatial mesh displacement
Iter      SolEst     Damping    Stepsize #Res #Jac #Sol   LinErr   LinRes
   1     1.8e-08   0.5000000     1.8e-08   26    13    13  2.1e-15  1.9e-15
Solution error estimates for segregated groups
1e-05, 6.6e-05, 0.00076, 9.2e-09
Residual error estimates for segregated groups
0.004, 9.7, 1.2e+03, 0.13
Solution time: 154 s. (2 minutes, 34 seconds)
Physical memory: 4.85 GB
Virtual memory: 25.58 GB
Ended at Jan 31, 2025, 9:28:25 PM.
----- Stationary Solver 1 in study1/Approach Curve Solver (sol1) -------------->
```

d=1995 (su103)

GENERAL

Description	Value
Solution	d=1995

LOG

```
<---- Compile Equations: Stationary in study1/Approach Curve Solver (sol1) -----
Started at Jan 31, 2025, 9:28:26 PM.
Parameter d = 1.995E-6 (m).
Geometry shape function: Linear serendipity
Number of vertex elements: 18
Number of boundary elements: 6064
Number of elements: 153650
Minimum element quality: 0.4775
Time: 8 s.
Physical memory: 3.09 GB
Virtual memory: 15.25 GB
Ended at Jan 31, 2025, 9:28:34 PM.
----- Compile Equations: Stationary in study1/Approach Curve Solver (sol1) ---->

<---- Dependent Variables 1 in study1/Approach Curve Solver (sol1) -------------
Started at Jan 31, 2025, 9:28:34 PM.
Solution time: 0 s.
Physical memory: 3.09 GB
Virtual memory: 15.25 GB
Ended at Jan 31, 2025, 9:28:34 PM.
----- Dependent Variables 1 in study1/Approach Curve Solver (sol1) ------------>

<---- Stationary Solver 1 in study1/Approach Curve Solver (sol1) ---------------
Started at Jan 31, 2025, 9:28:34 PM.
Segregated solver
Segregated Step 11 does not have any components solved for.
Number of degrees of freedom solved for: 779352 (plus 11551 internal DOFs).
Segregated solver iteration 1.
Merged variables
Nonsymmetric matrix found.
Scales for dependent variables:
Concentration (comp1.Cl): 1.4e+02
Concentration (comp1.K): 1.4e+02
Electric potential (comp1.V): 1.9e+02
Orthonormal null-space function used.
Iter      SolEst     Damping    Stepsize #Res #Jac #Sol   LinErr   LinRes
   1        0.83   0.3500000        0.83    2    1    1  2.9e-11  2.8e-15
   2        0.81   0.3500000        0.81    3    2    2  5.4e-11  2.6e-15
   3         0.8   0.3500000         0.8    4    3    3  2.1e-11  2.6e-15
Velocity u2, Pressure p
Nonsymmetric matrix found.
Scales for dependent variables:
Pressure (comp1.p): 1
Velocity field (spatial frame) (comp1.u2): 0.00018
Orthonormal null-space function used.
Iter      SolEst     Damping    Stepsize #Res #Jac #Sol   LinErr   LinRes
   1         1.1   0.5000000         1.1    2    1    1  2.1e-15  2.3e-15
Displacement field
Nonsymmetric matrix found.
Scales for dependent variables:
Auxiliary pressure (comp1.solid.lemm1.pw): 1
Displacement field (comp1.u): 2.8e-18
Orthonormal null-space function used.
Iter      SolEst     Damping    Stepsize #Res #Jac #Sol   LinErr   LinRes
   1         1.9   0.5000000         1.9    2    1    1  1.3e-13  1.4e-12
Spatial mesh displacement
```

```
Symmetric matrices found.
Scales for dependent variables:
Spatial mesh displacement (comp1.spatial.disp): 1.2e-09
Orthonormal null-space function used.
Iter      SolEst     Damping    Stepsize #Res #Jac #Sol   LinErr   LinRes
   1     8.3e-07   0.5000000     8.3e-07    2    1    1  1.7e-15  1.8e-15
Solution error estimates for segregated groups
1.4, 0.54, 0.95, 4.1e-07
Residual error estimates for segregated groups
7.8e+03, 8.2e+04, 2.1e+04, 0.11
Segregated solver iteration 2.
Merged variables
Scales for dependent variables:
Concentration (comp1.Cl): 1.4e+02
Concentration (comp1.K): 1.4e+02
Electric potential (comp1.V): 0.064
Iter      SolEst     Damping    Stepsize #Res #Jac #Sol   LinErr   LinRes
   1        0.78   0.3500000        0.78    6    4    4  5.9e-14  2.6e-15
   2        0.77   0.3500000        0.77    7    5    5  5.9e-14  2.8e-15
   3        0.76   0.3500000        0.76    8    6    6  4.3e-14  2.7e-15
Velocity u2, Pressure p
Scales for dependent variables:
Pressure (comp1.p): 3.7e+02
Velocity field (spatial frame) (comp1.u2): 0.00074
Iter      SolEst     Damping    Stepsize #Res #Jac #Sol   LinErr   LinRes
   1        0.36   0.5000000        0.36    4    2    2  3.1e-15  7.9e-15
Displacement field
Iter      SolEst     Damping    Stepsize #Res #Jac #Sol   LinErr   LinRes
   1        0.96   0.5000000        0.96    4    2    2  7.5e-14  1.4e-12
Spatial mesh displacement
Iter      SolEst     Damping    Stepsize #Res #Jac #Sol   LinErr   LinRes
   1     1.2e-06   0.5000000     1.2e-06    4    2    2  1.4e-15  1.8e-15
Solution error estimates for segregated groups
1.3, 0.18, 0.48, 6.2e-07
Residual error estimates for segregated groups
7.6e+02, 1.5e+04, 2.1e+04, 0.13
Segregated solver iteration 3.
Merged variables
Iter      SolEst     Damping    Stepsize #Res #Jac #Sol   LinErr   LinRes
   1        0.74   0.3500000        0.74   10    7    7  7.6e-14    3e-15
   2        0.73   0.3500000        0.73   11    8    8    1e-13  3.4e-15
   3        0.71   0.3500000        0.71   12    9    9  7.5e-14  3.1e-15
Velocity u2, Pressure p
Iter      SolEst     Damping    Stepsize #Res #Jac #Sol   LinErr   LinRes
   1        0.15   0.5000000        0.15    6    3    3  2.1e-15    8e-15
Displacement field
Iter      SolEst     Damping    Stepsize #Res #Jac #Sol   LinErr   LinRes
   1        0.52   0.5000000        0.52    6    3    3  8.8e-14  1.4e-12
Spatial mesh displacement
Iter      SolEst     Damping    Stepsize #Res #Jac #Sol   LinErr   LinRes
   1     1.2e-06   0.5000000     1.2e-06    6    3    3  1.4e-15  1.8e-15
Solution error estimates for segregated groups
1.2, 0.077, 0.26, 6.2e-07
Residual error estimates for segregated groups
2.1e+02, 8.3e+03, 1.6e+04, 0.12
Segregated solver iteration 4.
```

```
Merged variables
Iter      SolEst     Damping    Stepsize #Res #Jac #Sol   LinErr   LinRes
   1        0.69   0.3500000        0.69   14   10   10  4.4e-14  3.3e-15
   2        0.67   0.3500000        0.67   15   11   11  6.5e-14  2.7e-15
   3        0.58   0.3500000        0.58   16   12   12  1.5e-13  5.6e-15
Velocity u2, Pressure p
Iter      SolEst     Damping    Stepsize #Res #Jac #Sol   LinErr   LinRes
   1       0.072   0.5000000       0.072    8    4    4  2.6e-15  7.9e-15
Displacement field
Iter      SolEst     Damping    Stepsize #Res #Jac #Sol   LinErr   LinRes
   1        0.29   0.5000000        0.29    8    4    4  7.4e-14  1.5e-12
Spatial mesh displacement
Iter      SolEst     Damping    Stepsize #Res #Jac #Sol   LinErr   LinRes
   1       1e-06   0.5000000       1e-06    8    4    4  1.6e-15  1.8e-15
Solution error estimates for segregated groups
1, 0.036, 0.15, 5.2e-07
Residual error estimates for segregated groups
81, 4.4e+03, 1.1e+04, 0.13
Segregated solver iteration 5.
Merged variables
Iter      SolEst     Damping    Stepsize #Res #Jac #Sol   LinErr   LinRes
   1         0.4   0.3500000         0.4   18   13   13  7.7e-14  3.3e-15
   2        0.27   0.3500000        0.27   19   14   14  3.8e-14  2.7e-15
   3        0.18   0.3500000        0.18   20   15   15  5.4e-14  6.3e-15
Velocity u2, Pressure p
Iter      SolEst     Damping    Stepsize #Res #Jac #Sol   LinErr   LinRes
   1       0.035   0.5000000       0.035   10    5    5  4.5e-15  7.7e-15
Displacement field
Scales for dependent variables:
Auxiliary pressure (comp1.solid.lemm1.pw): 0.35
Displacement field (comp1.u): 2.8e-16
Iter      SolEst     Damping    Stepsize #Res #Jac #Sol   LinErr   LinRes
   1        0.17   0.5000000        0.17   10    5    5  1.7e-13  1.2e-12
Spatial mesh displacement
Iter      SolEst     Damping    Stepsize #Res #Jac #Sol   LinErr   LinRes
   1     7.8e-07   0.5000000     7.8e-07   10    5    5  1.4e-15  1.8e-15
Solution error estimates for segregated groups
0.3, 0.017, 0.084, 3.9e-07
Residual error estimates for segregated groups
32, 2.3e+03, 6.8e+03, 0.12
Segregated solver iteration 6.
Merged variables
Iter      SolEst     Damping    Stepsize #Res #Jac #Sol   LinErr   LinRes
   1        0.12   0.3500000        0.12   22   16   16  7.6e-14  5.6e-15
   2       0.076   0.3500000       0.076   23   17   17  9.2e-14  3.7e-15
   3        0.05   0.3500000        0.05   24   18   18  5.9e-14  2.7e-15
Velocity u2, Pressure p
Iter      SolEst     Damping    Stepsize #Res #Jac #Sol   LinErr   LinRes
   1       0.017   0.5000000       0.017   12    6    6  3.4e-15  7.9e-15
Displacement field
Iter      SolEst     Damping    Stepsize #Res #Jac #Sol   LinErr   LinRes
   1       0.096   0.5000000       0.096   12    6    6  1.5e-13  1.2e-12
Spatial mesh displacement
Iter      SolEst     Damping    Stepsize #Res #Jac #Sol   LinErr   LinRes
   1     5.4e-07   0.5000000     5.4e-07   12    6    6  1.4e-15  1.8e-15
Solution error estimates for segregated groups
```

```
0.085, 0.0086, 0.048, 2.7e-07
Residual error estimates for segregated groups
12, 1.1e+03, 4.2e+03, 0.13
Segregated solver iteration 7.
Merged variables
Iter      SolEst     Damping    Stepsize #Res #Jac #Sol   LinErr   LinRes
   1       0.032   0.3500000       0.032   26   19   19  1.1e-13  2.9e-15
   2       0.021   0.3500000       0.021   27   20   20  4.4e-14  3.3e-15
   3       0.014   0.3500000       0.014   28   21   21  5.9e-14  4.4e-15
Velocity u2, Pressure p
Iter      SolEst     Damping    Stepsize #Res #Jac #Sol   LinErr   LinRes
   1      0.0085   0.5000000      0.0085   14    7    7  3.3e-15  7.9e-15
Displacement field
Iter      SolEst     Damping    Stepsize #Res #Jac #Sol   LinErr   LinRes
   1       0.054   0.5000000       0.054   14    7    7  1.4e-13  1.1e-12
Spatial mesh displacement
Iter      SolEst     Damping    Stepsize #Res #Jac #Sol   LinErr   LinRes
   1     3.6e-07   0.5000000     3.6e-07   14    7    7  1.4e-15  1.8e-15
Solution error estimates for segregated groups
0.023, 0.0042, 0.027, 1.8e-07
Residual error estimates for segregated groups
4, 5.7e+02, 2.6e+03, 0.13
Segregated solver iteration 8.
Merged variables
Iter      SolEst     Damping    Stepsize #Res #Jac #Sol   LinErr   LinRes
   1      0.0089   0.3500000      0.0089   30   22   22  7.3e-14  3.4e-15
   2      0.0058   0.3500000      0.0058   31   23   23  4.2e-14    4e-15
   3      0.0038   0.3500000      0.0038   32   24   24  9.5e-14  3.6e-15
Velocity u2, Pressure p
Iter      SolEst     Damping    Stepsize #Res #Jac #Sol   LinErr   LinRes
   1      0.0042   0.5000000      0.0042   16    8    8  2.3e-15  7.7e-15
Displacement field
Iter      SolEst     Damping    Stepsize #Res #Jac #Sol   LinErr   LinRes
   1        0.03   0.5000000        0.03   16    8    8  1.1e-13  1.1e-12
Spatial mesh displacement
Iter      SolEst     Damping    Stepsize #Res #Jac #Sol   LinErr   LinRes
   1     2.3e-07   0.5000000     2.3e-07   16    8    8  1.5e-15  1.8e-15
Solution error estimates for segregated groups
0.0065, 0.0021, 0.015, 1.2e-07
Residual error estimates for segregated groups
1.3, 2.9e+02, 1.8e+03, 0.13
Segregated solver iteration 9.
Merged variables
Iter      SolEst     Damping    Stepsize #Res #Jac #Sol   LinErr   LinRes
   1      0.0024   0.3500000      0.0024   34   25   25  6.1e-14  4.1e-15
   2      0.0016   0.3500000      0.0016   35   26   26  7.9e-14  5.7e-15
   3       0.001   0.3500000       0.001   36   27   27  7.2e-14  2.8e-15
Velocity u2, Pressure p
Iter      SolEst     Damping    Stepsize #Res #Jac #Sol   LinErr   LinRes
   1      0.0021   0.5000000      0.0021   18    9    9  2.5e-15  7.9e-15
Displacement field
Iter      SolEst     Damping    Stepsize #Res #Jac #Sol   LinErr   LinRes
   1       0.017   0.5000000       0.017   18    9    9  1.3e-13  1.1e-12
Spatial mesh displacement
Iter      SolEst     Damping    Stepsize #Res #Jac #Sol   LinErr   LinRes
   1     1.5e-07   0.5000000     1.5e-07   18    9    9  1.6e-15  1.8e-15
```

```
Solution error estimates for segregated groups
0.0018, 0.0011, 0.0085, 7.3e-08
Residual error estimates for segregated groups
0.43, 1.4e+02, 1.4e+03, 0.13
Segregated solver iteration 10.
Merged variables
Iter      SolEst     Damping    Stepsize #Res #Jac #Sol   LinErr   LinRes
   1     0.00067   0.3500000     0.00067   38   28   28  1.1e-13  3.6e-15
   2     0.00044   0.3500000     0.00044   39   29   29  1.1e-13  3.7e-15
   3     0.00028   0.3500000     0.00028   40   30   30    7e-14  3.6e-15
Velocity u2, Pressure p
Iter      SolEst     Damping    Stepsize #Res #Jac #Sol   LinErr   LinRes
   1      0.0011   0.5000000      0.0011   20   10   10  4.6e-15  7.8e-15
Displacement field
Iter      SolEst     Damping    Stepsize #Res #Jac #Sol   LinErr   LinRes
   1      0.0094   0.5000000      0.0094   20   10   10  1.4e-13  1.2e-12
Spatial mesh displacement
Iter      SolEst     Damping    Stepsize #Res #Jac #Sol   LinErr   LinRes
   1     8.9e-08   0.5000000     8.9e-08   20   10   10  1.5e-15  1.8e-15
Solution error estimates for segregated groups
0.00049, 0.00053, 0.0047, 4.5e-08
Residual error estimates for segregated groups
0.14, 72, 1.2e+03, 0.11
Segregated solver iteration 11.
Merged variables
Iter      SolEst     Damping    Stepsize #Res #Jac #Sol   LinErr   LinRes
   1     0.00018   0.3500000     0.00018   42   31   31  1.1e-13  4.4e-15
   2     0.00012   0.3500000     0.00012   43   32   32  9.3e-14  3.9e-15
   3     7.8e-05   0.3500000     7.8e-05   44   33   33  7.6e-14    3e-15
Velocity u2, Pressure p
Iter      SolEst     Damping    Stepsize #Res #Jac #Sol   LinErr   LinRes
   1     0.00053   0.5000000     0.00053   22   11   11  3.5e-15  7.9e-15
Displacement field
Iter      SolEst     Damping    Stepsize #Res #Jac #Sol   LinErr   LinRes
   1      0.0051   0.5000000      0.0051   22   11   11  1.4e-13  1.2e-12
Spatial mesh displacement
Iter      SolEst     Damping    Stepsize #Res #Jac #Sol   LinErr   LinRes
   1     5.3e-08   0.5000000     5.3e-08   22   11   11  1.7e-15  1.8e-15
Solution error estimates for segregated groups
0.00013, 0.00026, 0.0026, 2.7e-08
Residual error estimates for segregated groups
0.043, 36, 1.2e+03, 0.12
Segregated solver iteration 12.
Merged variables
Iter      SolEst     Damping    Stepsize #Res #Jac #Sol   LinErr   LinRes
   1     5.1e-05   0.3500000     5.1e-05   46   34   34  5.6e-14  3.6e-15
   2     3.3e-05   0.3500000     3.3e-05   47   35   35  6.9e-14  6.2e-15
   3     2.1e-05   0.3500000     2.1e-05   48   36   36  1.5e-13  8.2e-15
Velocity u2, Pressure p
Iter      SolEst     Damping    Stepsize #Res #Jac #Sol   LinErr   LinRes
   1     0.00026   0.5000000     0.00026   24   12   12  2.6e-15  7.9e-15
Displacement field
Iter      SolEst     Damping    Stepsize #Res #Jac #Sol   LinErr   LinRes
   1      0.0028   0.5000000      0.0028   24   12   12  1.2e-13  1.2e-12
Spatial mesh displacement
Iter      SolEst     Damping    Stepsize #Res #Jac #Sol   LinErr   LinRes
```

```
   1     3.2e-08   0.5000000     3.2e-08   24   12   12  1.4e-15  1.8e-15
Solution error estimates for segregated groups
3.7e-05, 0.00013, 0.0014, 1.6e-08
Residual error estimates for segregated groups
0.013, 18, 1.2e+03, 0.12
Segregated solver iteration 13.
Merged variables
Iter      SolEst     Damping    Stepsize #Res #Jac #Sol   LinErr   LinRes
   1     1.4e-05   0.3500000     1.4e-05   50   37   37  5.5e-14  3.6e-15
   2       9e-06   0.3500000       9e-06   51   38   38  3.7e-14  3.5e-15
   3     5.9e-06   0.3500000     5.9e-06   52   39   39  1.2e-13  4.7e-15
Velocity u2, Pressure p
Iter      SolEst     Damping    Stepsize #Res #Jac #Sol   LinErr   LinRes
   1     0.00013   0.5000000     0.00013   26   13   13  3.7e-15  7.8e-15
Displacement field
Iter      SolEst     Damping    Stepsize #Res #Jac #Sol   LinErr   LinRes
   1      0.0015   0.5000000      0.0015   26   13   13    1e-13  1.2e-12
Spatial mesh displacement
Iter      SolEst     Damping    Stepsize #Res #Jac #Sol   LinErr   LinRes
   1     1.8e-08   0.5000000     1.8e-08   26   13   13  1.7e-15  1.8e-15
Solution error estimates for segregated groups
1e-05, 6.6e-05, 0.00076, 9.2e-09
Residual error estimates for segregated groups
0.004, 9, 1.2e+03, 0.13
Solution time: 156 s. (2 minutes, 36 seconds)
Physical memory: 4.93 GB
Virtual memory: 25.63 GB
Ended at Jan 31, 2025, 9:31:10 PM.
----- Stationary Solver 1 in study1/Approach Curve Solver (sol1) -------------->
```

d=1990 (su104)

GENERAL

Description	Value
Solution	d=1990

LOG

```
<---- Compile Equations: Stationary in study1/Approach Curve Solver (sol1) -----
Started at Jan 31, 2025, 9:31:10 PM.
Parameter d = 1.99E-6 (m).
Geometry shape function: Linear serendipity
Number of vertex elements: 18
Number of boundary elements: 6061
Number of elements: 153239
Minimum element quality: 0.4868
Time: 8 s.
Physical memory: 3.1 GB
Virtual memory: 15.26 GB
Ended at Jan 31, 2025, 9:31:18 PM.
----- Compile Equations: Stationary in study1/Approach Curve Solver (sol1) ---->

<---- Dependent Variables 1 in study1/Approach Curve Solver (sol1) -------------
Started at Jan 31, 2025, 9:31:18 PM.
Solution time: 0 s.
Physical memory: 3.1 GB
Virtual memory: 15.26 GB
Ended at Jan 31, 2025, 9:31:18 PM.
----- Dependent Variables 1 in study1/Approach Curve Solver (sol1) ------------>

<---- Stationary Solver 1 in study1/Approach Curve Solver (sol1) ---------------
Started at Jan 31, 2025, 9:31:18 PM.
Segregated solver
Segregated Step 11 does not have any components solved for.
Number of degrees of freedom solved for: 777254 (plus 11545 internal DOFs).
Segregated solver iteration 1.
Merged variables
Nonsymmetric matrix found.
Scales for dependent variables:
Concentration (comp1.Cl): 1.4e+02
Concentration (comp1.K): 1.4e+02
Electric potential (comp1.V): 1.9e+02
Orthonormal null-space function used.
Iter      SolEst     Damping    Stepsize #Res #Jac #Sol   LinErr   LinRes
   1        0.82   0.3500000        0.82    2    1    1  1.6e-11  2.7e-15
   2        0.81   0.3500000        0.81    3    2    2  3.5e-11  2.7e-15
   3         0.8   0.3500000         0.8    4    3    3  4.2e-11  2.6e-15
Velocity u2, Pressure p
Nonsymmetric matrix found.
Scales for dependent variables:
Pressure (comp1.p): 1
Velocity field (spatial frame) (comp1.u2): 0.00018
Orthonormal null-space function used.
Iter      SolEst     Damping    Stepsize #Res #Jac #Sol   LinErr   LinRes
   1         1.1   0.5000000         1.1    2    1    1  1.7e-15  2.2e-15
Displacement field
Nonsymmetric matrix found.
Scales for dependent variables:
Auxiliary pressure (comp1.solid.lemm1.pw): 1
Displacement field (comp1.u): 2.8e-18
Orthonormal null-space function used.
Iter      SolEst     Damping    Stepsize #Res #Jac #Sol   LinErr   LinRes
   1         1.9   0.5000000         1.9    2    1    1  1.1e-13  1.4e-12
Spatial mesh displacement
```

```
Symmetric matrices found.
Scales for dependent variables:
Spatial mesh displacement (comp1.spatial.disp): 1.2e-09
Orthonormal null-space function used.
Iter      SolEst     Damping    Stepsize #Res #Jac #Sol   LinErr   LinRes
   1     8.4e-07   0.5000000     8.4e-07    2    1    1  1.8e-15  1.8e-15
Solution error estimates for segregated groups
1.4, 0.54, 0.95, 4.2e-07
Residual error estimates for segregated groups
7.8e+03, 8.1e+04, 2.1e+04, 0.11
Segregated solver iteration 2.
Merged variables
Scales for dependent variables:
Concentration (comp1.Cl): 1.4e+02
Concentration (comp1.K): 1.4e+02
Electric potential (comp1.V): 0.064
Iter      SolEst     Damping    Stepsize #Res #Jac #Sol   LinErr   LinRes
   1        0.78   0.3500000        0.78    6    4    4  2.1e-13  3.8e-15
   2        0.77   0.3500000        0.77    7    5    5  9.5e-14  4.1e-15
   3        0.75   0.3500000        0.75    8    6    6  9.2e-14  4.4e-15
Velocity u2, Pressure p
Scales for dependent variables:
Pressure (comp1.p): 3.7e+02
Velocity field (spatial frame) (comp1.u2): 0.00074
Iter      SolEst     Damping    Stepsize #Res #Jac #Sol   LinErr   LinRes
   1        0.36   0.5000000        0.36    4    2    2  3.5e-15  7.8e-15
Displacement field
Iter      SolEst     Damping    Stepsize #Res #Jac #Sol   LinErr   LinRes
   1        0.96   0.5000000        0.96    4    2    2  5.9e-14  1.4e-12
Spatial mesh displacement
Iter      SolEst     Damping    Stepsize #Res #Jac #Sol   LinErr   LinRes
   1     1.3e-06   0.5000000     1.3e-06    4    2    2  1.4e-15  1.8e-15
Solution error estimates for segregated groups
1.3, 0.18, 0.48, 6.3e-07
Residual error estimates for segregated groups
7.5e+02, 1.6e+04, 2.1e+04, 0.12
Segregated solver iteration 3.
Merged variables
Iter      SolEst     Damping    Stepsize #Res #Jac #Sol   LinErr   LinRes
   1        0.74   0.3500000        0.74   10    7    7  1.3e-13  3.9e-15
   2        0.73   0.3500000        0.73   11    8    8  1.2e-13  5.1e-15
   3        0.71   0.3500000        0.71   12    9    9  8.5e-14  3.8e-15
Velocity u2, Pressure p
Iter      SolEst     Damping    Stepsize #Res #Jac #Sol   LinErr   LinRes
   1        0.15   0.5000000        0.15    6    3    3  4.7e-15  7.7e-15
Displacement field
Iter      SolEst     Damping    Stepsize #Res #Jac #Sol   LinErr   LinRes
   1        0.52   0.5000000        0.52    6    3    3  5.6e-14  1.4e-12
Spatial mesh displacement
Iter      SolEst     Damping    Stepsize #Res #Jac #Sol   LinErr   LinRes
   1     1.3e-06   0.5000000     1.3e-06    6    3    3  1.3e-15  1.9e-15
Solution error estimates for segregated groups
1.2, 0.077, 0.26, 6.3e-07
Residual error estimates for segregated groups
2.1e+02, 8.9e+03, 1.6e+04, 0.13
Segregated solver iteration 4.
```

```
Merged variables
Iter      SolEst     Damping    Stepsize #Res #Jac #Sol   LinErr   LinRes
   1        0.69   0.3500000        0.69   14   10   10  8.8e-14  4.1e-15
   2        0.67   0.3500000        0.67   15   11   11  1.1e-13  4.9e-15
   3        0.58   0.3500000        0.58   16   12   12  1.6e-13  3.3e-15
Velocity u2, Pressure p
Iter      SolEst     Damping    Stepsize #Res #Jac #Sol   LinErr   LinRes
   1       0.072   0.5000000       0.072    8    4    4  4.3e-15  7.9e-15
Displacement field
Iter      SolEst     Damping    Stepsize #Res #Jac #Sol   LinErr   LinRes
   1        0.29   0.5000000        0.29    8    4    4  5.9e-14  1.4e-12
Spatial mesh displacement
Iter      SolEst     Damping    Stepsize #Res #Jac #Sol   LinErr   LinRes
   1       1e-06   0.5000000       1e-06    8    4    4  1.4e-15  1.8e-15
Solution error estimates for segregated groups
0.99, 0.036, 0.15, 5.2e-07
Residual error estimates for segregated groups
81, 4.7e+03, 1.1e+04, 0.12
Segregated solver iteration 5.
Merged variables
Iter      SolEst     Damping    Stepsize #Res #Jac #Sol   LinErr   LinRes
   1         0.4   0.3500000         0.4   18   13   13  8.7e-14  5.8e-15
   2        0.27   0.3500000        0.27   19   14   14  1.3e-13  4.9e-15
   3        0.18   0.3500000        0.18   20   15   15  8.4e-14  3.7e-15
Velocity u2, Pressure p
Iter      SolEst     Damping    Stepsize #Res #Jac #Sol   LinErr   LinRes
   1       0.035   0.5000000       0.035   10    5    5  2.9e-15  7.9e-15
Displacement field
Scales for dependent variables:
Auxiliary pressure (comp1.solid.lemm1.pw): 0.35
Displacement field (comp1.u): 2.8e-16
Iter      SolEst     Damping    Stepsize #Res #Jac #Sol   LinErr   LinRes
   1        0.17   0.5000000        0.17   10    5    5    1e-13  1.2e-12
Spatial mesh displacement
Iter      SolEst     Damping    Stepsize #Res #Jac #Sol   LinErr   LinRes
   1     7.8e-07   0.5000000     7.8e-07   10    5    5  1.2e-15  1.9e-15
Solution error estimates for segregated groups
0.3, 0.017, 0.084, 3.9e-07
Residual error estimates for segregated groups
32, 2.4e+03, 6.8e+03, 0.13
Segregated solver iteration 6.
Merged variables
Iter      SolEst     Damping    Stepsize #Res #Jac #Sol   LinErr   LinRes
   1        0.12   0.3500000        0.12   22   16   16  1.3e-13  4.5e-15
   2       0.076   0.3500000       0.076   23   17   17  2.2e-13  4.3e-15
   3       0.049   0.3500000       0.049   24   18   18  4.7e-14  3.6e-15
Velocity u2, Pressure p
Iter      SolEst     Damping    Stepsize #Res #Jac #Sol   LinErr   LinRes
   1       0.017   0.5000000       0.017   12    6    6  2.7e-15  7.7e-15
Displacement field
Iter      SolEst     Damping    Stepsize #Res #Jac #Sol   LinErr   LinRes
   1       0.096   0.5000000       0.096   12    6    6  1.3e-13  1.1e-12
Spatial mesh displacement
Iter      SolEst     Damping    Stepsize #Res #Jac #Sol   LinErr   LinRes
   1     5.5e-07   0.5000000     5.5e-07   12    6    6  1.6e-15  1.8e-15
Solution error estimates for segregated groups
```

```
0.085, 0.0086, 0.048, 2.7e-07
Residual error estimates for segregated groups
12, 1.2e+03, 4.2e+03, 0.12
Segregated solver iteration 7.
Merged variables
Iter      SolEst     Damping    Stepsize #Res #Jac #Sol   LinErr   LinRes
   1       0.032   0.3500000       0.032   26   19   19  1.2e-13  4.3e-15
   2       0.021   0.3500000       0.021   27   20   20  1.4e-13  4.2e-15
   3       0.014   0.3500000       0.014   28   21   21  8.7e-14  5.3e-15
Velocity u2, Pressure p
Iter      SolEst     Damping    Stepsize #Res #Jac #Sol   LinErr   LinRes
   1      0.0085   0.5000000      0.0085   14    7    7    3e-15  7.8e-15
Displacement field
Iter      SolEst     Damping    Stepsize #Res #Jac #Sol   LinErr   LinRes
   1       0.054   0.5000000       0.054   14    7    7  9.5e-14  1.1e-12
Spatial mesh displacement
Iter      SolEst     Damping    Stepsize #Res #Jac #Sol   LinErr   LinRes
   1     3.7e-07   0.5000000     3.7e-07   14    7    7  1.4e-15  1.8e-15
Solution error estimates for segregated groups
0.023, 0.0042, 0.027, 1.8e-07
Residual error estimates for segregated groups
4, 6.2e+02, 2.6e+03, 0.13
Segregated solver iteration 8.
Merged variables
Iter      SolEst     Damping    Stepsize #Res #Jac #Sol   LinErr   LinRes
   1      0.0089   0.3500000      0.0089   30   22   22  5.6e-14  5.7e-15
   2      0.0058   0.3500000      0.0058   31   23   23  1.1e-13    5e-15
   3      0.0037   0.3500000      0.0037   32   24   24  9.2e-14  3.9e-15
Velocity u2, Pressure p
Iter      SolEst     Damping    Stepsize #Res #Jac #Sol   LinErr   LinRes
   1      0.0042   0.5000000      0.0042   16    8    8  5.4e-15  7.8e-15
Displacement field
Iter      SolEst     Damping    Stepsize #Res #Jac #Sol   LinErr   LinRes
   1        0.03   0.5000000        0.03   16    8    8  1.1e-13  1.2e-12
Spatial mesh displacement
Iter      SolEst     Damping    Stepsize #Res #Jac #Sol   LinErr   LinRes
   1     2.4e-07   0.5000000     2.4e-07   16    8    8  1.8e-15  1.8e-15
Solution error estimates for segregated groups
0.0064, 0.0021, 0.015, 1.2e-07
Residual error estimates for segregated groups
1.3, 3.1e+02, 1.8e+03, 0.12
Segregated solver iteration 9.
Merged variables
Iter      SolEst     Damping    Stepsize #Res #Jac #Sol   LinErr   LinRes
   1      0.0024   0.3500000      0.0024   34   25   25  2.4e-13  5.3e-15
   2      0.0016   0.3500000      0.0016   35   26   26  1.8e-13  4.4e-15
   3       0.001   0.3500000       0.001   36   27   27  1.2e-13  4.1e-15
Velocity u2, Pressure p
Iter      SolEst     Damping    Stepsize #Res #Jac #Sol   LinErr   LinRes
   1      0.0021   0.5000000      0.0021   18    9    9  2.4e-15  7.8e-15
Displacement field
Iter      SolEst     Damping    Stepsize #Res #Jac #Sol   LinErr   LinRes
   1       0.017   0.5000000       0.017   18    9    9  2.5e-13  1.2e-12
Spatial mesh displacement
Iter      SolEst     Damping    Stepsize #Res #Jac #Sol   LinErr   LinRes
   1     1.5e-07   0.5000000     1.5e-07   18    9    9  1.7e-15  1.8e-15
```

```
Solution error estimates for segregated groups
0.0018, 0.0011, 0.0085, 7.4e-08
Residual error estimates for segregated groups
0.43, 1.6e+02, 1.4e+03, 0.12
Segregated solver iteration 10.
Merged variables
Iter      SolEst     Damping    Stepsize #Res #Jac #Sol   LinErr   LinRes
   1     0.00067   0.3500000     0.00067   38   28   28  5.7e-14  3.5e-15
   2     0.00044   0.3500000     0.00044   39   29   29  1.3e-13  5.2e-15
   3     0.00028   0.3500000     0.00028   40   30   30  1.5e-13  4.5e-15
Velocity u2, Pressure p
Iter      SolEst     Damping    Stepsize #Res #Jac #Sol   LinErr   LinRes
   1      0.0011   0.5000000      0.0011   20   10   10  3.2e-15  7.9e-15
Displacement field
Iter      SolEst     Damping    Stepsize #Res #Jac #Sol   LinErr   LinRes
   1      0.0094   0.5000000      0.0094   20   10   10  1.3e-13  1.2e-12
Spatial mesh displacement
Iter      SolEst     Damping    Stepsize #Res #Jac #Sol   LinErr   LinRes
   1       9e-08   0.5000000       9e-08   20   10   10  1.7e-15  1.8e-15
Solution error estimates for segregated groups
0.00049, 0.00053, 0.0047, 4.5e-08
Residual error estimates for segregated groups
0.14, 78, 1.3e+03, 0.12
Segregated solver iteration 11.
Merged variables
Iter      SolEst     Damping    Stepsize #Res #Jac #Sol   LinErr   LinRes
   1     0.00018   0.3500000     0.00018   42   31   31  1.5e-13  3.7e-15
   2     0.00012   0.3500000     0.00012   43   32   32  1.4e-13  3.9e-15
   3     7.8e-05   0.3500000     7.8e-05   44   33   33    7e-14  4.1e-15
Velocity u2, Pressure p
Iter      SolEst     Damping    Stepsize #Res #Jac #Sol   LinErr   LinRes
   1     0.00053   0.5000000     0.00053   22   11   11  3.8e-15  7.8e-15
Displacement field
Iter      SolEst     Damping    Stepsize #Res #Jac #Sol   LinErr   LinRes
   1      0.0051   0.5000000      0.0051   22   11   11  1.3e-13  1.2e-12
Spatial mesh displacement
Iter      SolEst     Damping    Stepsize #Res #Jac #Sol   LinErr   LinRes
   1     5.4e-08   0.5000000     5.4e-08   22   11   11  1.4e-15  1.8e-15
Solution error estimates for segregated groups
0.00013, 0.00026, 0.0026, 2.7e-08
Residual error estimates for segregated groups
0.043, 39, 1.2e+03, 0.12
Segregated solver iteration 12.
Merged variables
Iter      SolEst     Damping    Stepsize #Res #Jac #Sol   LinErr   LinRes
   1       5e-05   0.3500000       5e-05   46   34   34  1.4e-13  3.7e-15
   2     3.3e-05   0.3500000     3.3e-05   47   35   35  1.5e-13  5.4e-15
   3     2.1e-05   0.3500000     2.1e-05   48   36   36  1.5e-13  3.9e-15
Velocity u2, Pressure p
Iter      SolEst     Damping    Stepsize #Res #Jac #Sol   LinErr   LinRes
   1     0.00026   0.5000000     0.00026   24   12   12    4e-15  7.8e-15
Displacement field
Iter      SolEst     Damping    Stepsize #Res #Jac #Sol   LinErr   LinRes
   1      0.0028   0.5000000      0.0028   24   12   12  1.2e-13  1.2e-12
Spatial mesh displacement
Iter      SolEst     Damping    Stepsize #Res #Jac #Sol   LinErr   LinRes
```

```
   1     3.2e-08   0.5000000     3.2e-08   24   12   12  1.6e-15  1.8e-15
Solution error estimates for segregated groups
3.7e-05, 0.00013, 0.0014, 1.6e-08
Residual error estimates for segregated groups
0.013, 19, 1.2e+03, 0.12
Segregated solver iteration 13.
Merged variables
Iter      SolEst     Damping    Stepsize #Res #Jac #Sol   LinErr   LinRes
   1     1.4e-05   0.3500000     1.4e-05   50   37   37  1.2e-13    5e-15
   2       9e-06   0.3500000       9e-06   51   38   38  1.1e-13  4.5e-15
   3     5.9e-06   0.3500000     5.9e-06   52   39   39  8.2e-14  4.3e-15
Velocity u2, Pressure p
Iter      SolEst     Damping    Stepsize #Res #Jac #Sol   LinErr   LinRes
   1     0.00013   0.5000000     0.00013   26   13   13  3.9e-15  7.9e-15
Displacement field
Iter      SolEst     Damping    Stepsize #Res #Jac #Sol   LinErr   LinRes
   1      0.0015   0.5000000      0.0015   26   13   13  1.3e-13  1.2e-12
Spatial mesh displacement
Iter      SolEst     Damping    Stepsize #Res #Jac #Sol   LinErr   LinRes
   1     1.9e-08   0.5000000     1.9e-08   26   13   13  1.3e-15  1.8e-15
Solution error estimates for segregated groups
1e-05, 6.6e-05, 0.00076, 9.3e-09
Residual error estimates for segregated groups
0.004, 9.7, 1.2e+03, 0.13
Solution time: 152 s. (2 minutes, 32 seconds)
Physical memory: 4.79 GB
Virtual memory: 25.55 GB
Ended at Jan 31, 2025, 9:33:51 PM.
----- Stationary Solver 1 in study1/Approach Curve Solver (sol1) -------------->
```

d=1985 (su105)

GENERAL

Description	Value
Solution	d=1985

LOG

```
<---- Compile Equations: Stationary in study1/Approach Curve Solver (sol1) -----
Started at Jan 31, 2025, 9:33:51 PM.
Parameter d = 1.985E-6 (m).
Geometry shape function: Linear serendipity
Number of vertex elements: 18
Number of boundary elements: 6059
Number of elements: 153479
Minimum element quality: 0.4619
Time: 8 s.
Physical memory: 3.11 GB
Virtual memory: 15.27 GB
Ended at Jan 31, 2025, 9:33:59 PM.
----- Compile Equations: Stationary in study1/Approach Curve Solver (sol1) ---->

<---- Dependent Variables 1 in study1/Approach Curve Solver (sol1) -------------
Started at Jan 31, 2025, 9:33:59 PM.
Solution time: 0 s.
Physical memory: 3.11 GB
Virtual memory: 15.27 GB
Ended at Jan 31, 2025, 9:33:59 PM.
----- Dependent Variables 1 in study1/Approach Curve Solver (sol1) ------------>

<---- Stationary Solver 1 in study1/Approach Curve Solver (sol1) ---------------
Started at Jan 31, 2025, 9:33:59 PM.
Segregated solver
Segregated Step 11 does not have any components solved for.
Number of degrees of freedom solved for: 778419 (plus 11541 internal DOFs).
Segregated solver iteration 1.
Merged variables
Nonsymmetric matrix found.
Scales for dependent variables:
Concentration (comp1.Cl): 1.4e+02
Concentration (comp1.K): 1.4e+02
Electric potential (comp1.V): 1.9e+02
Orthonormal null-space function used.
Iter      SolEst     Damping    Stepsize #Res #Jac #Sol   LinErr   LinRes
   1        0.82   0.3500000        0.82    2    1    1  1.9e-11  2.7e-15
   2        0.81   0.3500000        0.81    3    2    2  4.3e-11  2.7e-15
   3         0.8   0.3500000         0.8    4    3    3  2.6e-11  2.7e-15
Velocity u2, Pressure p
Nonsymmetric matrix found.
Scales for dependent variables:
Pressure (comp1.p): 1
Velocity field (spatial frame) (comp1.u2): 0.00018
Orthonormal null-space function used.
Iter      SolEst     Damping    Stepsize #Res #Jac #Sol   LinErr   LinRes
   1         1.1   0.5000000         1.1    2    1    1  1.5e-15  2.2e-15
Displacement field
Nonsymmetric matrix found.
Scales for dependent variables:
Auxiliary pressure (comp1.solid.lemm1.pw): 1
Displacement field (comp1.u): 2.8e-18
Orthonormal null-space function used.
Iter      SolEst     Damping    Stepsize #Res #Jac #Sol   LinErr   LinRes
   1         1.9   0.5000000         1.9    2    1    1  6.7e-14  1.4e-12
Spatial mesh displacement
```

```
Symmetric matrices found.
Scales for dependent variables:
Spatial mesh displacement (comp1.spatial.disp): 1.2e-09
Orthonormal null-space function used.
Iter      SolEst     Damping    Stepsize #Res #Jac #Sol   LinErr   LinRes
   1     8.4e-07   0.5000000     8.4e-07    2    1    1  1.8e-15  1.8e-15
Solution error estimates for segregated groups
1.4, 0.54, 0.95, 4.2e-07
Residual error estimates for segregated groups
7.8e+03, 8.1e+04, 2.1e+04, 0.11
Segregated solver iteration 2.
Merged variables
Scales for dependent variables:
Concentration (comp1.Cl): 1.4e+02
Concentration (comp1.K): 1.4e+02
Electric potential (comp1.V): 0.064
Iter      SolEst     Damping    Stepsize #Res #Jac #Sol   LinErr   LinRes
   1        0.78   0.3500000        0.78    6    4    4  8.7e-14  2.6e-15
   2        0.77   0.3500000        0.77    7    5    5  8.3e-14  2.7e-15
   3        0.75   0.3500000        0.75    8    6    6  7.2e-14  2.7e-15
Velocity u2, Pressure p
Scales for dependent variables:
Pressure (comp1.p): 3.7e+02
Velocity field (spatial frame) (comp1.u2): 0.00074
Iter      SolEst     Damping    Stepsize #Res #Jac #Sol   LinErr   LinRes
   1        0.36   0.5000000        0.36    4    2    2    3e-15  7.9e-15
Displacement field
Iter      SolEst     Damping    Stepsize #Res #Jac #Sol   LinErr   LinRes
   1        0.96   0.5000000        0.96    4    2    2  4.2e-14  1.5e-12
Spatial mesh displacement
Iter      SolEst     Damping    Stepsize #Res #Jac #Sol   LinErr   LinRes
   1     1.3e-06   0.5000000     1.3e-06    4    2    2  1.5e-15  1.8e-15
Solution error estimates for segregated groups
1.3, 0.18, 0.48, 6.3e-07
Residual error estimates for segregated groups
7.6e+02, 1.7e+04, 2.1e+04, 0.12
Segregated solver iteration 3.
Merged variables
Iter      SolEst     Damping    Stepsize #Res #Jac #Sol   LinErr   LinRes
   1        0.74   0.3500000        0.74   10    7    7  8.2e-14  2.7e-15
   2        0.73   0.3500000        0.73   11    8    8  5.7e-14  2.7e-15
   3        0.71   0.3500000        0.71   12    9    9  1.4e-13  2.7e-15
Velocity u2, Pressure p
Iter      SolEst     Damping    Stepsize #Res #Jac #Sol   LinErr   LinRes
   1        0.15   0.5000000        0.15    6    3    3    3e-15  7.9e-15
Displacement field
Iter      SolEst     Damping    Stepsize #Res #Jac #Sol   LinErr   LinRes
   1        0.52   0.5000000        0.52    6    3    3  3.1e-14  1.5e-12
Spatial mesh displacement
Iter      SolEst     Damping    Stepsize #Res #Jac #Sol   LinErr   LinRes
   1     1.3e-06   0.5000000     1.3e-06    6    3    3  1.4e-15  1.9e-15
Solution error estimates for segregated groups
1.2, 0.077, 0.26, 6.3e-07
Residual error estimates for segregated groups
2.1e+02, 9.5e+03, 1.6e+04, 0.12
Segregated solver iteration 4.
```

```
Merged variables
Iter      SolEst     Damping    Stepsize #Res #Jac #Sol   LinErr   LinRes
   1        0.69   0.3500000        0.69   14   10   10  8.3e-14  2.7e-15
   2        0.67   0.3500000        0.67   15   11   11    1e-13  2.7e-15
   3        0.58   0.3500000        0.58   16   12   12  2.8e-14  2.6e-15
Velocity u2, Pressure p
Iter      SolEst     Damping    Stepsize #Res #Jac #Sol   LinErr   LinRes
   1       0.072   0.5000000       0.072    8    4    4    3e-15  7.8e-15
Displacement field
Iter      SolEst     Damping    Stepsize #Res #Jac #Sol   LinErr   LinRes
   1        0.29   0.5000000        0.29    8    4    4  3.5e-14  1.5e-12
Spatial mesh displacement
Iter      SolEst     Damping    Stepsize #Res #Jac #Sol   LinErr   LinRes
   1       1e-06   0.5000000       1e-06    8    4    4  1.9e-15  1.8e-15
Solution error estimates for segregated groups
0.99, 0.036, 0.15, 5.2e-07
Residual error estimates for segregated groups
81, 5e+03, 1.1e+04, 0.12
Segregated solver iteration 5.
Merged variables
Iter      SolEst     Damping    Stepsize #Res #Jac #Sol   LinErr   LinRes
   1         0.4   0.3500000         0.4   18   13   13  7.8e-14  2.7e-15
   2        0.27   0.3500000        0.27   19   14   14  1.1e-13  2.7e-15
   3        0.18   0.3500000        0.18   20   15   15    4e-14  2.7e-15
Velocity u2, Pressure p
Iter      SolEst     Damping    Stepsize #Res #Jac #Sol   LinErr   LinRes
   1       0.035   0.5000000       0.035   10    5    5  2.6e-15  7.7e-15
Displacement field
Scales for dependent variables:
Auxiliary pressure (comp1.solid.lemm1.pw): 0.35
Displacement field (comp1.u): 2.9e-16
Iter      SolEst     Damping    Stepsize #Res #Jac #Sol   LinErr   LinRes
   1        0.17   0.5000000        0.17   10    5    5    1e-13  1.2e-12
Spatial mesh displacement
Iter      SolEst     Damping    Stepsize #Res #Jac #Sol   LinErr   LinRes
   1     7.9e-07   0.5000000     7.9e-07   10    5    5  1.5e-15  1.8e-15
Solution error estimates for segregated groups
0.3, 0.017, 0.084, 3.9e-07
Residual error estimates for segregated groups
32, 2.6e+03, 6.8e+03, 0.12
Segregated solver iteration 6.
Merged variables
Iter      SolEst     Damping    Stepsize #Res #Jac #Sol   LinErr   LinRes
   1        0.12   0.3500000        0.12   22   16   16  5.4e-14  2.9e-15
   2       0.076   0.3500000       0.076   23   17   17    6e-14  2.6e-15
   3       0.049   0.3500000       0.049   24   18   18  1.1e-13  2.7e-15
Velocity u2, Pressure p
Iter      SolEst     Damping    Stepsize #Res #Jac #Sol   LinErr   LinRes
   1       0.017   0.5000000       0.017   12    6    6  3.3e-15  7.8e-15
Displacement field
Iter      SolEst     Damping    Stepsize #Res #Jac #Sol   LinErr   LinRes
   1       0.096   0.5000000       0.096   12    6    6  1.2e-13  1.1e-12
Spatial mesh displacement
Iter      SolEst     Damping    Stepsize #Res #Jac #Sol   LinErr   LinRes
   1     5.5e-07   0.5000000     5.5e-07   12    6    6  1.6e-15  1.8e-15
Solution error estimates for segregated groups
```

```
0.085, 0.0086, 0.048, 2.8e-07
Residual error estimates for segregated groups
12, 1.3e+03, 4.2e+03, 0.11
Segregated solver iteration 7.
Merged variables
Iter      SolEst     Damping    Stepsize #Res #Jac #Sol   LinErr   LinRes
   1       0.032   0.3500000       0.032   26   19   19  8.3e-14  2.7e-15
   2       0.021   0.3500000       0.021   27   20   20  7.2e-14  2.6e-15
   3       0.014   0.3500000       0.014   28   21   21    4e-14  2.8e-15
Velocity u2, Pressure p
Iter      SolEst     Damping    Stepsize #Res #Jac #Sol   LinErr   LinRes
   1      0.0085   0.5000000      0.0085   14    7    7  3.7e-15  7.8e-15
Displacement field
Iter      SolEst     Damping    Stepsize #Res #Jac #Sol   LinErr   LinRes
   1       0.054   0.5000000       0.054   14    7    7  1.1e-13  1.1e-12
Spatial mesh displacement
Iter      SolEst     Damping    Stepsize #Res #Jac #Sol   LinErr   LinRes
   1     3.7e-07   0.5000000     3.7e-07   14    7    7  1.4e-15  1.8e-15
Solution error estimates for segregated groups
0.023, 0.0042, 0.027, 1.8e-07
Residual error estimates for segregated groups
4, 6.6e+02, 2.6e+03, 0.13
Segregated solver iteration 8.
Merged variables
Iter      SolEst     Damping    Stepsize #Res #Jac #Sol   LinErr   LinRes
   1      0.0089   0.3500000      0.0089   30   22   22  9.9e-14  2.7e-15
   2      0.0058   0.3500000      0.0058   31   23   23  5.8e-14  2.7e-15
   3      0.0038   0.3500000      0.0038   32   24   24  5.8e-14  2.7e-15
Velocity u2, Pressure p
Iter      SolEst     Damping    Stepsize #Res #Jac #Sol   LinErr   LinRes
   1      0.0042   0.5000000      0.0042   16    8    8  2.7e-15  7.9e-15
Displacement field
Iter      SolEst     Damping    Stepsize #Res #Jac #Sol   LinErr   LinRes
   1        0.03   0.5000000        0.03   16    8    8  1.8e-13  1.2e-12
Spatial mesh displacement
Iter      SolEst     Damping    Stepsize #Res #Jac #Sol   LinErr   LinRes
   1     2.4e-07   0.5000000     2.4e-07   16    8    8  1.6e-15  1.8e-15
Solution error estimates for segregated groups
0.0064, 0.0021, 0.015, 1.2e-07
Residual error estimates for segregated groups
1.3, 3.3e+02, 1.8e+03, 0.12
Segregated solver iteration 9.
Merged variables
Iter      SolEst     Damping    Stepsize #Res #Jac #Sol   LinErr   LinRes
   1      0.0024   0.3500000      0.0024   34   25   25  1.3e-13  2.7e-15
   2      0.0016   0.3500000      0.0016   35   26   26  8.5e-14  2.7e-15
   3       0.001   0.3500000       0.001   36   27   27  9.2e-14  2.7e-15
Velocity u2, Pressure p
Iter      SolEst     Damping    Stepsize #Res #Jac #Sol   LinErr   LinRes
   1      0.0021   0.5000000      0.0021   18    9    9  3.1e-15  7.8e-15
Displacement field
Iter      SolEst     Damping    Stepsize #Res #Jac #Sol   LinErr   LinRes
   1       0.017   0.5000000       0.017   18    9    9  1.3e-13  1.1e-12
Spatial mesh displacement
Iter      SolEst     Damping    Stepsize #Res #Jac #Sol   LinErr   LinRes
   1     1.5e-07   0.5000000     1.5e-07   18    9    9  1.5e-15  1.9e-15
```

```
Solution error estimates for segregated groups
0.0018, 0.0011, 0.0085, 7.4e-08
Residual error estimates for segregated groups
0.43, 1.7e+02, 1.4e+03, 0.12
Segregated solver iteration 10.
Merged variables
Iter      SolEst     Damping    Stepsize #Res #Jac #Sol   LinErr   LinRes
   1     0.00067   0.3500000     0.00067   38   28   28  5.6e-14  2.7e-15
   2     0.00044   0.3500000     0.00044   39   29   29  6.9e-14  2.7e-15
   3     0.00028   0.3500000     0.00028   40   30   30  5.5e-14  2.7e-15
Velocity u2, Pressure p
Iter      SolEst     Damping    Stepsize #Res #Jac #Sol   LinErr   LinRes
   1      0.0011   0.5000000      0.0011   20   10   10  3.9e-15  7.7e-15
Displacement field
Iter      SolEst     Damping    Stepsize #Res #Jac #Sol   LinErr   LinRes
   1      0.0094   0.5000000      0.0094   20   10   10  1.2e-13  1.2e-12
Spatial mesh displacement
Iter      SolEst     Damping    Stepsize #Res #Jac #Sol   LinErr   LinRes
   1       9e-08   0.5000000       9e-08   20   10   10  1.5e-15  1.9e-15
Solution error estimates for segregated groups
0.00049, 0.00053, 0.0047, 4.5e-08
Residual error estimates for segregated groups
0.14, 83, 1.2e+03, 0.12
Segregated solver iteration 11.
Merged variables
Iter      SolEst     Damping    Stepsize #Res #Jac #Sol   LinErr   LinRes
   1     0.00018   0.3500000     0.00018   42   31   31  7.4e-14  2.6e-15
   2     0.00012   0.3500000     0.00012   43   32   32  9.7e-14  2.7e-15
   3     7.8e-05   0.3500000     7.8e-05   44   33   33  5.5e-14  2.6e-15
Velocity u2, Pressure p
Iter      SolEst     Damping    Stepsize #Res #Jac #Sol   LinErr   LinRes
   1     0.00053   0.5000000     0.00053   22   11   11  1.9e-15  7.8e-15
Displacement field
Iter      SolEst     Damping    Stepsize #Res #Jac #Sol   LinErr   LinRes
   1      0.0051   0.5000000      0.0051   22   11   11  1.1e-13  1.2e-12
Spatial mesh displacement
Iter      SolEst     Damping    Stepsize #Res #Jac #Sol   LinErr   LinRes
   1     5.4e-08   0.5000000     5.4e-08   22   11   11  1.8e-15  1.8e-15
Solution error estimates for segregated groups
0.00013, 0.00026, 0.0026, 2.7e-08
Residual error estimates for segregated groups
0.043, 42, 1.2e+03, 0.12
Segregated solver iteration 12.
Merged variables
Iter      SolEst     Damping    Stepsize #Res #Jac #Sol   LinErr   LinRes
   1     5.1e-05   0.3500000     5.1e-05   46   34   34  4.2e-14  2.7e-15
   2     3.3e-05   0.3500000     3.3e-05   47   35   35  1.4e-13  2.7e-15
   3     2.1e-05   0.3500000     2.1e-05   48   36   36  1.3e-13  2.7e-15
Velocity u2, Pressure p
Iter      SolEst     Damping    Stepsize #Res #Jac #Sol   LinErr   LinRes
   1     0.00026   0.5000000     0.00026   24   12   12  2.9e-15  7.8e-15
Displacement field
Iter      SolEst     Damping    Stepsize #Res #Jac #Sol   LinErr   LinRes
   1      0.0028   0.5000000      0.0028   24   12   12    1e-13  1.2e-12
Spatial mesh displacement
Iter      SolEst     Damping    Stepsize #Res #Jac #Sol   LinErr   LinRes
```

```
   1      3.2e-08   0.5000000     3.2e-08   24    12    12  1.8e-15  1.8e-15
Solution error estimates for segregated groups
3.7e-05, 0.00013, 0.0014, 1.6e-08
Residual error estimates for segregated groups
0.013, 21, 1.2e+03, 0.12
Segregated solver iteration 13.
Merged variables
Iter      SolEst      Damping    Stepsize #Res #Jac #Sol   LinErr   LinRes
   1      1.4e-05   0.3500000     1.4e-05   50    37    37  1.5e-13  2.7e-15
   2        9e-06   0.3500000       9e-06   51    38    38  6.5e-14  2.7e-15
   3      5.9e-06   0.3500000     5.9e-06   52    39    39  7.5e-14  2.7e-15
Velocity u2, Pressure p
Iter      SolEst      Damping    Stepsize #Res #Jac #Sol   LinErr   LinRes
   1      0.00013   0.5000000     0.00013   26    13    13  4.7e-15  7.8e-15
Displacement field
Iter      SolEst      Damping    Stepsize #Res #Jac #Sol   LinErr   LinRes
   1       0.0015   0.5000000      0.0015   26    13    13    1e-13  1.2e-12
Spatial mesh displacement
Iter      SolEst      Damping    Stepsize #Res #Jac #Sol   LinErr   LinRes
   1      1.9e-08   0.5000000     1.9e-08   26    13    13  1.3e-15  1.8e-15
Solution error estimates for segregated groups
1e-05, 6.6e-05, 0.00076, 9.3e-09
Residual error estimates for segregated groups
0.004, 10, 1.2e+03, 0.11
Solution time: 151 s. (2 minutes, 31 seconds)
Physical memory: 4.84 GB
Virtual memory: 25.54 GB
Ended at Jan 31, 2025, 9:36:30 PM.
----- Stationary Solver 1 in study1/Approach Curve Solver (sol1) -------------->
```

d=1980 (su106)

GENERAL

Description	Value
Solution	d=1980

LOG

```
<---- Compile Equations: Stationary in study1/Approach Curve Solver (sol1) -----
Started at Jan 31, 2025, 9:36:31 PM.
Parameter d = 1.98E-6 (m).
Geometry shape function: Linear serendipity
Number of vertex elements: 18
Number of boundary elements: 6056
Number of elements: 153282
Minimum element quality: 0.4609
Time: 8 s.
Physical memory: 3.09 GB
Virtual memory: 15.25 GB
Ended at Jan 31, 2025, 9:36:39 PM.
----- Compile Equations: Stationary in study1/Approach Curve Solver (sol1) ---->

<---- Dependent Variables 1 in study1/Approach Curve Solver (sol1) -------------
Started at Jan 31, 2025, 9:36:39 PM.
Solution time: 0 s.
Physical memory: 3.09 GB
Virtual memory: 15.25 GB
Ended at Jan 31, 2025, 9:36:39 PM.
----- Dependent Variables 1 in study1/Approach Curve Solver (sol1) ------------>

<---- Stationary Solver 1 in study1/Approach Curve Solver (sol1) ---------------
Started at Jan 31, 2025, 9:36:39 PM.
Segregated solver
Segregated Step 11 does not have any components solved for.
Number of degrees of freedom solved for: 777312 (plus 11535 internal DOFs).
Segregated solver iteration 1.
Merged variables
Nonsymmetric matrix found.
Scales for dependent variables:
Concentration (comp1.Cl): 1.4e+02
Concentration (comp1.K): 1.4e+02
Electric potential (comp1.V): 1.9e+02
Orthonormal null-space function used.
Iter      SolEst     Damping    Stepsize #Res #Jac #Sol   LinErr   LinRes
   1        0.82   0.3500000        0.82    2    1    1  4.7e-11  2.7e-15
   2        0.81   0.3500000        0.81    3    2    2  2.9e-11  2.6e-15
   3         0.8   0.3500000         0.8    4    3    3  6.5e-11  2.6e-15
Velocity u2, Pressure p
Nonsymmetric matrix found.
Scales for dependent variables:
Pressure (comp1.p): 1
Velocity field (spatial frame) (comp1.u2): 0.00018
Orthonormal null-space function used.
Iter      SolEst     Damping    Stepsize #Res #Jac #Sol   LinErr   LinRes
   1         1.1   0.5000000         1.1    2    1    1  1.6e-15  2.3e-15
Displacement field
Nonsymmetric matrix found.
Scales for dependent variables:
Auxiliary pressure (comp1.solid.lemm1.pw): 1
Displacement field (comp1.u): 2.8e-18
Orthonormal null-space function used.
Iter      SolEst     Damping    Stepsize #Res #Jac #Sol   LinErr   LinRes
   1         1.9   0.5000000         1.9    2    1    1  1.3e-13  1.4e-12
Spatial mesh displacement
```

```
Symmetric matrices found.
Scales for dependent variables:
Spatial mesh displacement (comp1.spatial.disp): 1.2e-09
Orthonormal null-space function used.
Iter      SolEst     Damping    Stepsize #Res #Jac #Sol   LinErr   LinRes
   1     8.4e-07   0.5000000     8.4e-07    2    1    1  1.3e-15  1.8e-15
Solution error estimates for segregated groups
1.4, 0.54, 0.96, 4.2e-07
Residual error estimates for segregated groups
7.8e+03, 8.1e+04, 2.1e+04, 0.11
Segregated solver iteration 2.
Merged variables
Scales for dependent variables:
Concentration (comp1.Cl): 1.4e+02
Concentration (comp1.K): 1.4e+02
Electric potential (comp1.V): 0.064
Iter      SolEst     Damping    Stepsize #Res #Jac #Sol   LinErr   LinRes
   1        0.78   0.3500000        0.78    6    4    4  1.1e-13  2.6e-15
   2        0.77   0.3500000        0.77    7    5    5  5.6e-14  2.8e-15
   3        0.75   0.3500000        0.75    8    6    6  8.6e-14  2.7e-15
Velocity u2, Pressure p
Scales for dependent variables:
Pressure (comp1.p): 3.7e+02
Velocity field (spatial frame) (comp1.u2): 0.00074
Iter      SolEst     Damping    Stepsize #Res #Jac #Sol   LinErr   LinRes
   1        0.36   0.5000000        0.36    4    2    2    3e-15  7.9e-15
Displacement field
Iter      SolEst     Damping    Stepsize #Res #Jac #Sol   LinErr   LinRes
   1        0.96   0.5000000        0.96    4    2    2  4.2e-14  1.4e-12
Spatial mesh displacement
Iter      SolEst     Damping    Stepsize #Res #Jac #Sol   LinErr   LinRes
   1     1.3e-06   0.5000000     1.3e-06    4    2    2  1.5e-15  1.9e-15
Solution error estimates for segregated groups
1.3, 0.18, 0.48, 6.3e-07
Residual error estimates for segregated groups
7.5e+02, 1.7e+04, 2.1e+04, 0.11
Segregated solver iteration 3.
Merged variables
Iter      SolEst     Damping    Stepsize #Res #Jac #Sol   LinErr   LinRes
   1        0.74   0.3500000        0.74   10    7    7  1.2e-13  2.6e-15
   2        0.73   0.3500000        0.73   11    8    8  5.4e-14  2.7e-15
   3        0.71   0.3500000        0.71   12    9    9    1e-13  2.8e-15
Velocity u2, Pressure p
Iter      SolEst     Damping    Stepsize #Res #Jac #Sol   LinErr   LinRes
   1        0.15   0.5000000        0.15    6    3    3    2e-15  7.8e-15
Displacement field
Iter      SolEst     Damping    Stepsize #Res #Jac #Sol   LinErr   LinRes
   1        0.52   0.5000000        0.52    6    3    3  4.6e-14  1.4e-12
Spatial mesh displacement
Iter      SolEst     Damping    Stepsize #Res #Jac #Sol   LinErr   LinRes
   1     1.3e-06   0.5000000     1.3e-06    6    3    3  1.6e-15  1.8e-15
Solution error estimates for segregated groups
1.2, 0.077, 0.26, 6.3e-07
Residual error estimates for segregated groups
2.1e+02, 9.6e+03, 1.6e+04, 0.12
Segregated solver iteration 4.
```

```
Merged variables
Iter      SolEst     Damping    Stepsize #Res #Jac #Sol   LinErr   LinRes
   1        0.69   0.3500000        0.69   14   10   10  5.5e-14  2.7e-15
   2        0.67   0.3500000        0.67   15   11   11  7.6e-14  2.8e-15
   3        0.58   0.3500000        0.58   16   12   12  7.3e-14  2.6e-15
Velocity u2, Pressure p
Iter      SolEst     Damping    Stepsize #Res #Jac #Sol   LinErr   LinRes
   1       0.072   0.5000000       0.072    8    4    4  4.2e-15  7.9e-15
Displacement field
Iter      SolEst     Damping    Stepsize #Res #Jac #Sol   LinErr   LinRes
   1        0.29   0.5000000        0.29    8    4    4  8.1e-14  1.5e-12
Spatial mesh displacement
Iter      SolEst     Damping    Stepsize #Res #Jac #Sol   LinErr   LinRes
   1     1.1e-06   0.5000000     1.1e-06    8    4    4  1.3e-15  1.8e-15
Solution error estimates for segregated groups
0.99, 0.036, 0.15, 5.3e-07
Residual error estimates for segregated groups
81, 5.1e+03, 1.1e+04, 0.12
Segregated solver iteration 5.
Merged variables
Iter      SolEst     Damping    Stepsize #Res #Jac #Sol   LinErr   LinRes
   1         0.4   0.3500000         0.4   18   13   13    7e-14  2.7e-15
   2        0.27   0.3500000        0.27   19   14   14  6.8e-14  2.7e-15
   3        0.18   0.3500000        0.18   20   15   15  7.3e-14  2.6e-15
Velocity u2, Pressure p
Iter      SolEst     Damping    Stepsize #Res #Jac #Sol   LinErr   LinRes
   1       0.035   0.5000000       0.035   10    5    5  2.8e-15  7.8e-15
Displacement field
Scales for dependent variables:
Auxiliary pressure (comp1.solid.lemm1.pw): 0.35
Displacement field (comp1.u): 2.9e-16
Iter      SolEst     Damping    Stepsize #Res #Jac #Sol   LinErr   LinRes
   1        0.17   0.5000000        0.17   10    5    5    1e-13  1.1e-12
Spatial mesh displacement
Iter      SolEst     Damping    Stepsize #Res #Jac #Sol   LinErr   LinRes
   1     7.9e-07   0.5000000     7.9e-07   10    5    5  1.7e-15  1.8e-15
Solution error estimates for segregated groups
0.3, 0.017, 0.084, 3.9e-07
Residual error estimates for segregated groups
32, 2.6e+03, 6.8e+03, 0.13
Segregated solver iteration 6.
Merged variables
Iter      SolEst     Damping    Stepsize #Res #Jac #Sol   LinErr   LinRes
   1        0.12   0.3500000        0.12   22   16   16  4.6e-14  2.7e-15
   2       0.075   0.3500000       0.075   23   17   17    6e-14  2.6e-15
   3       0.049   0.3500000       0.049   24   18   18  8.2e-14  2.8e-15
Velocity u2, Pressure p
Iter      SolEst     Damping    Stepsize #Res #Jac #Sol   LinErr   LinRes
   1       0.017   0.5000000       0.017   12    6    6  4.8e-15  7.7e-15
Displacement field
Iter      SolEst     Damping    Stepsize #Res #Jac #Sol   LinErr   LinRes
   1       0.096   0.5000000       0.096   12    6    6  1.5e-13  1.2e-12
Spatial mesh displacement
Iter      SolEst     Damping    Stepsize #Res #Jac #Sol   LinErr   LinRes
   1     5.5e-07   0.5000000     5.5e-07   12    6    6  1.4e-15  1.8e-15
Solution error estimates for segregated groups
```

```
0.085, 0.0086, 0.048, 2.8e-07
Residual error estimates for segregated groups
12, 1.3e+03, 4.2e+03, 0.12
Segregated solver iteration 7.
Merged variables
Iter      SolEst     Damping    Stepsize #Res #Jac #Sol   LinErr   LinRes
   1       0.032   0.3500000       0.032   26   19   19  9.3e-14  2.8e-15
   2       0.021   0.3500000       0.021   27   20   20    1e-13  2.7e-15
   3       0.014   0.3500000       0.014   28   21   21  1.2e-13  2.7e-15
Velocity u2, Pressure p
Iter      SolEst     Damping    Stepsize #Res #Jac #Sol   LinErr   LinRes
   1      0.0085   0.5000000      0.0085   14    7    7  2.3e-15  7.9e-15
Displacement field
Iter      SolEst     Damping    Stepsize #Res #Jac #Sol   LinErr   LinRes
   1       0.054   0.5000000       0.054   14    7    7  1.1e-13  1.2e-12
Spatial mesh displacement
Iter      SolEst     Damping    Stepsize #Res #Jac #Sol   LinErr   LinRes
   1     3.7e-07   0.5000000     3.7e-07   14    7    7  2.2e-15  1.8e-15
Solution error estimates for segregated groups
0.023, 0.0042, 0.027, 1.8e-07
Residual error estimates for segregated groups
4, 6.7e+02, 2.6e+03, 0.13
Segregated solver iteration 8.
Merged variables
Iter      SolEst     Damping    Stepsize #Res #Jac #Sol   LinErr   LinRes
   1      0.0089   0.3500000      0.0089   30   22   22  6.2e-14    3e-15
   2      0.0058   0.3500000      0.0058   31   23   23  8.3e-14  2.6e-15
   3      0.0037   0.3500000      0.0037   32   24   24  4.4e-14  2.6e-15
Velocity u2, Pressure p
Iter      SolEst     Damping    Stepsize #Res #Jac #Sol   LinErr   LinRes
   1      0.0042   0.5000000      0.0042   16    8    8  3.9e-15  7.9e-15
Displacement field
Iter      SolEst     Damping    Stepsize #Res #Jac #Sol   LinErr   LinRes
   1        0.03   0.5000000        0.03   16    8    8  9.8e-14  1.1e-12
Spatial mesh displacement
Iter      SolEst     Damping    Stepsize #Res #Jac #Sol   LinErr   LinRes
   1     2.4e-07   0.5000000     2.4e-07   16    8    8  1.5e-15  1.8e-15
Solution error estimates for segregated groups
0.0064, 0.0021, 0.015, 1.2e-07
Residual error estimates for segregated groups
1.3, 3.4e+02, 1.8e+03, 0.12
Segregated solver iteration 9.
Merged variables
Iter      SolEst     Damping    Stepsize #Res #Jac #Sol   LinErr   LinRes
   1      0.0024   0.3500000      0.0024   34   25   25  9.8e-14  2.8e-15
   2      0.0016   0.3500000      0.0016   35   26   26  6.8e-14  2.8e-15
   3       0.001   0.3500000       0.001   36   27   27    1e-13  2.9e-15
Velocity u2, Pressure p
Iter      SolEst     Damping    Stepsize #Res #Jac #Sol   LinErr   LinRes
   1      0.0021   0.5000000      0.0021   18    9    9  2.7e-15  7.8e-15
Displacement field
Iter      SolEst     Damping    Stepsize #Res #Jac #Sol   LinErr   LinRes
   1       0.017   0.5000000       0.017   18    9    9  1.5e-13  1.1e-12
Spatial mesh displacement
Iter      SolEst     Damping    Stepsize #Res #Jac #Sol   LinErr   LinRes
   1     1.5e-07   0.5000000     1.5e-07   18    9    9  1.4e-15  1.8e-15
```

```
Solution error estimates for segregated groups
0.0018, 0.0011, 0.0085, 7.4e-08
Residual error estimates for segregated groups
0.43, 1.7e+02, 1.4e+03, 0.12
Segregated solver iteration 10.
Merged variables
Iter      SolEst     Damping    Stepsize #Res #Jac #Sol   LinErr   LinRes
   1     0.00067   0.3500000     0.00067   38   28   28    6e-14  2.6e-15
   2     0.00043   0.3500000     0.00043   39   29   29  6.2e-14  2.8e-15
   3     0.00028   0.3500000     0.00028   40   30   30  7.6e-14  2.7e-15
Velocity u2, Pressure p
Iter      SolEst     Damping    Stepsize #Res #Jac #Sol   LinErr   LinRes
   1      0.0011   0.5000000      0.0011   20   10   10  4.9e-15    8e-15
Displacement field
Iter      SolEst     Damping    Stepsize #Res #Jac #Sol   LinErr   LinRes
   1      0.0094   0.5000000      0.0094   20   10   10    1e-13  1.1e-12
Spatial mesh displacement
Iter      SolEst     Damping    Stepsize #Res #Jac #Sol   LinErr   LinRes
   1       9e-08   0.5000000       9e-08   20   10   10  1.7e-15  1.8e-15
Solution error estimates for segregated groups
0.00048, 0.00053, 0.0047, 4.5e-08
Residual error estimates for segregated groups
0.14, 85, 1.2e+03, 0.13
Segregated solver iteration 11.
Merged variables
Iter      SolEst     Damping    Stepsize #Res #Jac #Sol   LinErr   LinRes
   1     0.00018   0.3500000     0.00018   42   31   31  6.9e-14  2.6e-15
   2     0.00012   0.3500000     0.00012   43   32   32  6.8e-14  2.8e-15
   3     7.8e-05   0.3500000     7.8e-05   44   33   33  4.7e-14  2.7e-15
Velocity u2, Pressure p
Iter      SolEst     Damping    Stepsize #Res #Jac #Sol   LinErr   LinRes
   1     0.00053   0.5000000     0.00053   22   11   11  4.6e-15  7.8e-15
Displacement field
Iter      SolEst     Damping    Stepsize #Res #Jac #Sol   LinErr   LinRes
   1      0.0051   0.5000000      0.0051   22   11   11  1.7e-13  1.2e-12
Spatial mesh displacement
Iter      SolEst     Damping    Stepsize #Res #Jac #Sol   LinErr   LinRes
   1     5.4e-08   0.5000000     5.4e-08   22   11   11  1.7e-15  1.8e-15
Solution error estimates for segregated groups
0.00013, 0.00026, 0.0026, 2.7e-08
Residual error estimates for segregated groups
0.043, 42, 1.2e+03, 0.12
Segregated solver iteration 12.
Merged variables
Iter      SolEst     Damping    Stepsize #Res #Jac #Sol   LinErr   LinRes
   1       5e-05   0.3500000       5e-05   46   34   34  6.5e-14  2.7e-15
   2     3.3e-05   0.3500000     3.3e-05   47   35   35  1.5e-13  2.6e-15
   3     2.1e-05   0.3500000     2.1e-05   48   36   36  5.5e-14  2.6e-15
Velocity u2, Pressure p
Iter      SolEst     Damping    Stepsize #Res #Jac #Sol   LinErr   LinRes
   1     0.00026   0.5000000     0.00026   24   12   12  3.3e-15  7.9e-15
Displacement field
Iter      SolEst     Damping    Stepsize #Res #Jac #Sol   LinErr   LinRes
   1      0.0028   0.5000000      0.0028   24   12   12  9.5e-14  1.1e-12
Spatial mesh displacement
Iter      SolEst     Damping    Stepsize #Res #Jac #Sol   LinErr   LinRes
```

```
   1     3.2e-08   0.5000000     3.2e-08   24   12   12  1.6e-15  1.8e-15
Solution error estimates for segregated groups
3.7e-05, 0.00013, 0.0014, 1.6e-08
Residual error estimates for segregated groups
0.013, 21, 1.2e+03, 0.12
Segregated solver iteration 13.
Merged variables
Iter      SolEst     Damping    Stepsize #Res #Jac #Sol   LinErr   LinRes
   1     1.4e-05   0.3500000     1.4e-05   50   37   37  8.5e-14  2.7e-15
   2       9e-06   0.3500000       9e-06   51   38   38  6.4e-14  2.7e-15
   3     5.9e-06   0.3500000     5.9e-06   52   39   39  4.4e-14  2.6e-15
Velocity u2, Pressure p
Iter      SolEst     Damping    Stepsize #Res #Jac #Sol   LinErr   LinRes
   1     0.00013   0.5000000     0.00013   26   13   13  4.4e-15  7.8e-15
Displacement field
Iter      SolEst     Damping    Stepsize #Res #Jac #Sol   LinErr   LinRes
   1      0.0015   0.5000000      0.0015   26   13   13  9.8e-14  1.1e-12
Spatial mesh displacement
Iter      SolEst     Damping    Stepsize #Res #Jac #Sol   LinErr   LinRes
   1     1.9e-08   0.5000000     1.9e-08   26   13   13  1.6e-15  1.8e-15
Solution error estimates for segregated groups
1e-05, 6.6e-05, 0.00076, 9.4e-09
Residual error estimates for segregated groups
0.004, 11, 1.2e+03, 0.13
Solution time: 156 s. (2 minutes, 36 seconds)
Physical memory: 4.82 GB
Virtual memory: 25.57 GB
Ended at Jan 31, 2025, 9:39:15 PM.
----- Stationary Solver 1 in study1/Approach Curve Solver (sol1) -------------->
```

d=1975 (su107)

GENERAL

Description	Value
Solution	d=1975

LOG

```
<---- Compile Equations: Stationary in study1/Approach Curve Solver (sol1) -----
Started at Jan 31, 2025, 9:39:15 PM.
Parameter d = 1.975E-6 (m).
Geometry shape function: Linear serendipity
Number of vertex elements: 18
Number of boundary elements: 6054
Number of elements: 153596
Minimum element quality: 0.4815
Time: 8 s.
Physical memory: 3.16 GB
Virtual memory: 15.32 GB
Ended at Jan 31, 2025, 9:39:23 PM.
----- Compile Equations: Stationary in study1/Approach Curve Solver (sol1) ---->

<---- Dependent Variables 1 in study1/Approach Curve Solver (sol1) -------------
Started at Jan 31, 2025, 9:39:23 PM.
Solution time: 0 s.
Physical memory: 3.16 GB
Virtual memory: 15.32 GB
Ended at Jan 31, 2025, 9:39:23 PM.
----- Dependent Variables 1 in study1/Approach Curve Solver (sol1) ------------>

<---- Stationary Solver 1 in study1/Approach Curve Solver (sol1) ---------------
Started at Jan 31, 2025, 9:39:23 PM.
Segregated solver
Segregated Step 11 does not have any components solved for.
Number of degrees of freedom solved for: 778898 (plus 11531 internal DOFs).
Segregated solver iteration 1.
Merged variables
Nonsymmetric matrix found.
Scales for dependent variables:
Concentration (comp1.Cl): 1.4e+02
Concentration (comp1.K): 1.4e+02
Electric potential (comp1.V): 1.9e+02
Orthonormal null-space function used.
Iter      SolEst     Damping    Stepsize #Res #Jac #Sol   LinErr   LinRes
   1        0.82   0.3500000        0.82    2    1    1  1.8e-11  2.7e-15
   2        0.81   0.3500000        0.81    3    2    2  4.1e-11  2.7e-15
   3         0.8   0.3500000         0.8    4    3    3  1.6e-11  2.6e-15
Velocity u2, Pressure p
Nonsymmetric matrix found.
Scales for dependent variables:
Pressure (comp1.p): 1
Velocity field (spatial frame) (comp1.u2): 0.00018
Orthonormal null-space function used.
Iter      SolEst     Damping    Stepsize #Res #Jac #Sol   LinErr   LinRes
   1         1.1   0.5000000         1.1    2    1    1  1.7e-15  2.3e-15
Displacement field
Nonsymmetric matrix found.
Scales for dependent variables:
Auxiliary pressure (comp1.solid.lemm1.pw): 1
Displacement field (comp1.u): 2.8e-18
Orthonormal null-space function used.
Iter      SolEst     Damping    Stepsize #Res #Jac #Sol   LinErr   LinRes
   1         1.9   0.5000000         1.9    2    1    1  3.9e-14  1.4e-12
Spatial mesh displacement
```

```
Symmetric matrices found.
Scales for dependent variables:
Spatial mesh displacement (comp1.spatial.disp): 1.2e-09
Orthonormal null-space function used.
Iter      SolEst     Damping    Stepsize #Res #Jac #Sol   LinErr   LinRes
   1     8.4e-07   0.5000000     8.4e-07    2    1    1  1.4e-15  1.8e-15
Solution error estimates for segregated groups
1.4, 0.54, 0.96, 4.2e-07
Residual error estimates for segregated groups
7.8e+03, 8.1e+04, 2.1e+04, 0.11
Segregated solver iteration 2.
Merged variables
Scales for dependent variables:
Concentration (comp1.Cl): 1.4e+02
Concentration (comp1.K): 1.4e+02
Electric potential (comp1.V): 0.064
Iter      SolEst     Damping    Stepsize #Res #Jac #Sol   LinErr   LinRes
   1        0.78   0.3500000        0.78    6    4    4    5e-14  2.8e-15
   2        0.77   0.3500000        0.77    7    5    5  1.1e-13  3.2e-15
   3        0.75   0.3500000        0.75    8    6    6  6.2e-14    4e-15
Velocity u2, Pressure p
Scales for dependent variables:
Pressure (comp1.p): 3.7e+02
Velocity field (spatial frame) (comp1.u2): 0.00074
Iter      SolEst     Damping    Stepsize #Res #Jac #Sol   LinErr   LinRes
   1        0.36   0.5000000        0.36    4    2    2  2.8e-15  7.9e-15
Displacement field
Iter      SolEst     Damping    Stepsize #Res #Jac #Sol   LinErr   LinRes
   1        0.96   0.5000000        0.96    4    2    2  7.1e-14  1.4e-12
Spatial mesh displacement
Iter      SolEst     Damping    Stepsize #Res #Jac #Sol   LinErr   LinRes
   1     1.3e-06   0.5000000     1.3e-06    4    2    2  1.6e-15  1.9e-15
Solution error estimates for segregated groups
1.3, 0.18, 0.48, 6.3e-07
Residual error estimates for segregated groups
7.5e+02, 1.6e+04, 2.1e+04, 0.13
Segregated solver iteration 3.
Merged variables
Iter      SolEst     Damping    Stepsize #Res #Jac #Sol   LinErr   LinRes
   1        0.74   0.3500000        0.74   10    7    7  7.8e-14  3.4e-15
   2        0.73   0.3500000        0.73   11    8    8  1.6e-13  3.7e-15
   3        0.71   0.3500000        0.71   12    9    9  1.1e-13    3e-15
Velocity u2, Pressure p
Iter      SolEst     Damping    Stepsize #Res #Jac #Sol   LinErr   LinRes
   1        0.15   0.5000000        0.15    6    3    3  2.1e-15  7.8e-15
Displacement field
Iter      SolEst     Damping    Stepsize #Res #Jac #Sol   LinErr   LinRes
   1        0.52   0.5000000        0.52    6    3    3  6.1e-14  1.4e-12
Spatial mesh displacement
Iter      SolEst     Damping    Stepsize #Res #Jac #Sol   LinErr   LinRes
   1     1.3e-06   0.5000000     1.3e-06    6    3    3  1.3e-15  1.8e-15
Solution error estimates for segregated groups
1.2, 0.077, 0.26, 6.3e-07
Residual error estimates for segregated groups
2.1e+02, 9.3e+03, 1.6e+04, 0.12
Segregated solver iteration 4.
```

```
Merged variables
Iter      SolEst     Damping    Stepsize #Res #Jac #Sol   LinErr   LinRes
   1        0.69   0.3500000        0.69   14   10   10  1.3e-13    3e-15
   2        0.67   0.3500000        0.67   15   11   11  6.2e-14  5.4e-15
   3        0.58   0.3500000        0.58   16   12   12  3.5e-14  4.1e-15
Velocity u2, Pressure p
Iter      SolEst     Damping    Stepsize #Res #Jac #Sol   LinErr   LinRes
   1       0.072   0.5000000       0.072    8    4    4  3.5e-15    8e-15
Displacement field
Iter      SolEst     Damping    Stepsize #Res #Jac #Sol   LinErr   LinRes
   1        0.29   0.5000000        0.29    8    4    4  7.7e-14  1.5e-12
Spatial mesh displacement
Iter      SolEst     Damping    Stepsize #Res #Jac #Sol   LinErr   LinRes
   1     1.1e-06   0.5000000     1.1e-06    8    4    4  1.4e-15  1.8e-15
Solution error estimates for segregated groups
0.99, 0.036, 0.15, 5.3e-07
Residual error estimates for segregated groups
81, 4.9e+03, 1.1e+04, 0.13
Segregated solver iteration 5.
Merged variables
Iter      SolEst     Damping    Stepsize #Res #Jac #Sol   LinErr   LinRes
   1         0.4   0.3500000         0.4   18   13   13  5.4e-14  4.4e-15
   2        0.27   0.3500000        0.27   19   14   14  8.5e-14    4e-15
   3        0.18   0.3500000        0.18   20   15   15  4.2e-14  4.2e-15
Velocity u2, Pressure p
Iter      SolEst     Damping    Stepsize #Res #Jac #Sol   LinErr   LinRes
   1       0.035   0.5000000       0.035   10    5    5  2.3e-15    8e-15
Displacement field
Scales for dependent variables:
Auxiliary pressure (comp1.solid.lemm1.pw): 0.35
Displacement field (comp1.u): 2.9e-16
Iter      SolEst     Damping    Stepsize #Res #Jac #Sol   LinErr   LinRes
   1        0.17   0.5000000        0.17   10    5    5    2e-13  1.2e-12
Spatial mesh displacement
Iter      SolEst     Damping    Stepsize #Res #Jac #Sol   LinErr   LinRes
   1     7.9e-07   0.5000000     7.9e-07   10    5    5  1.5e-15  1.8e-15
Solution error estimates for segregated groups
0.3, 0.017, 0.084, 3.9e-07
Residual error estimates for segregated groups
32, 2.5e+03, 6.8e+03, 0.12
Segregated solver iteration 6.
Merged variables
Iter      SolEst     Damping    Stepsize #Res #Jac #Sol   LinErr   LinRes
   1        0.12   0.3500000        0.12   22   16   16  8.1e-14  5.3e-15
   2       0.076   0.3500000       0.076   23   17   17  5.6e-14  5.7e-15
   3       0.049   0.3500000       0.049   24   18   18  5.7e-14    3e-15
Velocity u2, Pressure p
Iter      SolEst     Damping    Stepsize #Res #Jac #Sol   LinErr   LinRes
   1       0.017   0.5000000       0.017   12    6    6  2.5e-15  7.7e-15
Displacement field
Iter      SolEst     Damping    Stepsize #Res #Jac #Sol   LinErr   LinRes
   1       0.096   0.5000000       0.096   12    6    6  1.6e-13  1.2e-12
Spatial mesh displacement
Iter      SolEst     Damping    Stepsize #Res #Jac #Sol   LinErr   LinRes
   1     5.5e-07   0.5000000     5.5e-07   12    6    6  1.5e-15  1.9e-15
Solution error estimates for segregated groups
```

```
0.085, 0.0086, 0.048, 2.8e-07
Residual error estimates for segregated groups
12, 1.3e+03, 4.2e+03, 0.13
Segregated solver iteration 7.
Merged variables
Iter      SolEst     Damping    Stepsize #Res #Jac #Sol   LinErr   LinRes
   1       0.032   0.3500000       0.032   26   19   19  4.5e-14    3e-15
   2       0.021   0.3500000       0.021   27   20   20  8.6e-14    3e-15
   3       0.014   0.3500000       0.014   28   21   21  4.3e-14  5.7e-15
Velocity u2, Pressure p
Iter      SolEst     Damping    Stepsize #Res #Jac #Sol   LinErr   LinRes
   1      0.0085   0.5000000      0.0085   14    7    7  3.7e-15  7.8e-15
Displacement field
Iter      SolEst     Damping    Stepsize #Res #Jac #Sol   LinErr   LinRes
   1       0.054   0.5000000       0.054   14    7    7  1.4e-13  1.2e-12
Spatial mesh displacement
Iter      SolEst     Damping    Stepsize #Res #Jac #Sol   LinErr   LinRes
   1     3.7e-07   0.5000000     3.7e-07   14    7    7  1.7e-15  1.8e-15
Solution error estimates for segregated groups
0.023, 0.0042, 0.027, 1.8e-07
Residual error estimates for segregated groups
4, 6.4e+02, 2.6e+03, 0.13
Segregated solver iteration 8.
Merged variables
Iter      SolEst     Damping    Stepsize #Res #Jac #Sol   LinErr   LinRes
   1      0.0089   0.3500000      0.0089   30   22   22  1.1e-13  3.8e-15
   2      0.0058   0.3500000      0.0058   31   23   23  8.9e-14  3.3e-15
   3      0.0038   0.3500000      0.0038   32   24   24  1.1e-13  3.8e-15
Velocity u2, Pressure p
Iter      SolEst     Damping    Stepsize #Res #Jac #Sol   LinErr   LinRes
   1      0.0042   0.5000000      0.0042   16    8    8  3.2e-15  7.9e-15
Displacement field
Iter      SolEst     Damping    Stepsize #Res #Jac #Sol   LinErr   LinRes
   1        0.03   0.5000000        0.03   16    8    8  1.1e-13  1.2e-12
Spatial mesh displacement
Iter      SolEst     Damping    Stepsize #Res #Jac #Sol   LinErr   LinRes
   1     2.4e-07   0.5000000     2.4e-07   16    8    8  2.4e-15  1.8e-15
Solution error estimates for segregated groups
0.0064, 0.0021, 0.015, 1.2e-07
Residual error estimates for segregated groups
1.3, 3.2e+02, 1.8e+03, 0.12
Segregated solver iteration 9.
Merged variables
Iter      SolEst     Damping    Stepsize #Res #Jac #Sol   LinErr   LinRes
   1      0.0024   0.3500000      0.0024   34   25   25  9.3e-14  5.5e-15
   2      0.0016   0.3500000      0.0016   35   26   26  7.6e-14    5e-15
   3       0.001   0.3500000       0.001   36   27   27  1.4e-13  3.4e-15
Velocity u2, Pressure p
Iter      SolEst     Damping    Stepsize #Res #Jac #Sol   LinErr   LinRes
   1      0.0021   0.5000000      0.0021   18    9    9  2.6e-15  7.8e-15
Displacement field
Iter      SolEst     Damping    Stepsize #Res #Jac #Sol   LinErr   LinRes
   1       0.017   0.5000000       0.017   18    9    9  1.1e-13  1.2e-12
Spatial mesh displacement
Iter      SolEst     Damping    Stepsize #Res #Jac #Sol   LinErr   LinRes
   1     1.5e-07   0.5000000     1.5e-07   18    9    9  1.4e-15  1.8e-15
```

```
Solution error estimates for segregated groups
0.0018, 0.0011, 0.0085, 7.4e-08
Residual error estimates for segregated groups
0.43, 1.6e+02, 1.4e+03, 0.12
Segregated solver iteration 10.
Merged variables
Iter      SolEst     Damping    Stepsize #Res #Jac #Sol   LinErr   LinRes
   1     0.00067   0.3500000     0.00067   38   28   28  5.2e-14  3.6e-15
   2     0.00044   0.3500000     0.00044   39   29   29  6.1e-14  2.8e-15
   3     0.00028   0.3500000     0.00028   40   30   30  7.1e-14  4.3e-15
Velocity u2, Pressure p
Iter      SolEst     Damping    Stepsize #Res #Jac #Sol   LinErr   LinRes
   1      0.0011   0.5000000      0.0011   20   10   10  2.6e-15  7.8e-15
Displacement field
Iter      SolEst     Damping    Stepsize #Res #Jac #Sol   LinErr   LinRes
   1      0.0094   0.5000000      0.0094   20   10   10    1e-13  1.1e-12
Spatial mesh displacement
Iter      SolEst     Damping    Stepsize #Res #Jac #Sol   LinErr   LinRes
   1     9.1e-08   0.5000000     9.1e-08   20   10   10  1.2e-15  1.8e-15
Solution error estimates for segregated groups
0.00049, 0.00053, 0.0047, 4.5e-08
Residual error estimates for segregated groups
0.14, 81, 1.2e+03, 0.11
Segregated solver iteration 11.
Merged variables
Iter      SolEst     Damping    Stepsize #Res #Jac #Sol   LinErr   LinRes
   1     0.00018   0.3500000     0.00018   42   31   31  4.4e-14  5.2e-15
   2     0.00012   0.3500000     0.00012   43   32   32  6.9e-14  3.9e-15
   3     7.8e-05   0.3500000     7.8e-05   44   33   33  5.7e-14  4.5e-15
Velocity u2, Pressure p
Iter      SolEst     Damping    Stepsize #Res #Jac #Sol   LinErr   LinRes
   1     0.00053   0.5000000     0.00053   22   11   11  3.6e-15  7.9e-15
Displacement field
Iter      SolEst     Damping    Stepsize #Res #Jac #Sol   LinErr   LinRes
   1      0.0051   0.5000000      0.0051   22   11   11    1e-13  1.2e-12
Spatial mesh displacement
Iter      SolEst     Damping    Stepsize #Res #Jac #Sol   LinErr   LinRes
   1     5.4e-08   0.5000000     5.4e-08   22   11   11  1.5e-15  1.8e-15
Solution error estimates for segregated groups
0.00013, 0.00026, 0.0026, 2.7e-08
Residual error estimates for segregated groups
0.043, 41, 1.2e+03, 0.13
Segregated solver iteration 12.
Merged variables
Iter      SolEst     Damping    Stepsize #Res #Jac #Sol   LinErr   LinRes
   1     5.1e-05   0.3500000     5.1e-05   46   34   34  6.6e-14  3.4e-15
   2     3.3e-05   0.3500000     3.3e-05   47   35   35  6.5e-14  2.9e-15
   3     2.1e-05   0.3500000     2.1e-05   48   36   36    1e-13  3.2e-15
Velocity u2, Pressure p
Iter      SolEst     Damping    Stepsize #Res #Jac #Sol   LinErr   LinRes
   1     0.00026   0.5000000     0.00026   24   12   12  5.5e-15  7.9e-15
Displacement field
Iter      SolEst     Damping    Stepsize #Res #Jac #Sol   LinErr   LinRes
   1      0.0028   0.5000000      0.0028   24   12   12    1e-13  1.2e-12
Spatial mesh displacement
Iter      SolEst     Damping    Stepsize #Res #Jac #Sol   LinErr   LinRes
```

```
   1     3.2e-08   0.5000000     3.2e-08   24   12   12  1.7e-15  1.8e-15
Solution error estimates for segregated groups
3.7e-05, 0.00013, 0.0014, 1.6e-08
Residual error estimates for segregated groups
0.013, 20, 1.2e+03, 0.12
Segregated solver iteration 13.
Merged variables
Iter      SolEst     Damping    Stepsize #Res #Jac #Sol   LinErr   LinRes
   1     1.4e-05   0.3500000     1.4e-05   50   37   37  4.2e-14  3.6e-15
   2       9e-06   0.3500000       9e-06   51   38   38  8.7e-14  3.2e-15
   3     5.9e-06   0.3500000     5.9e-06   52   39   39  1.3e-13  4.3e-15
Velocity u2, Pressure p
Iter      SolEst     Damping    Stepsize #Res #Jac #Sol   LinErr   LinRes
   1     0.00013   0.5000000     0.00013   26   13   13  2.9e-15  7.9e-15
Displacement field
Iter      SolEst     Damping    Stepsize #Res #Jac #Sol   LinErr   LinRes
   1      0.0015   0.5000000      0.0015   26   13   13  1.2e-13  1.2e-12
Spatial mesh displacement
Iter      SolEst     Damping    Stepsize #Res #Jac #Sol   LinErr   LinRes
   1     1.9e-08   0.5000000     1.9e-08   26   13   13  1.8e-15  1.8e-15
Solution error estimates for segregated groups
1e-05, 6.6e-05, 0.00076, 9.4e-09
Residual error estimates for segregated groups
0.004, 10, 1.2e+03, 0.12
Solution time: 155 s. (2 minutes, 35 seconds)
Physical memory: 4.84 GB
Virtual memory: 25.57 GB
Ended at Jan 31, 2025, 9:41:58 PM.
----- Stationary Solver 1 in study1/Approach Curve Solver (sol1) -------------->
```

d=1970 (su108)

GENERAL

Description	Value
Solution	d=1970

LOG

```
<---- Compile Equations: Stationary in study1/Approach Curve Solver (sol1) -----
Started at Jan 31, 2025, 9:41:58 PM.
Parameter d = 1.97E-6 (m).
Geometry shape function: Linear serendipity
Number of vertex elements: 18
Number of boundary elements: 6051
Number of elements: 153049
Minimum element quality: 0.4924
Time: 8 s.
Physical memory: 3.09 GB
Virtual memory: 15.24 GB
Ended at Jan 31, 2025, 9:42:06 PM.
----- Compile Equations: Stationary in study1/Approach Curve Solver (sol1) ---->

<---- Dependent Variables 1 in study1/Approach Curve Solver (sol1) -------------
Started at Jan 31, 2025, 9:42:06 PM.
Solution time: 0 s.
Physical memory: 3.09 GB
Virtual memory: 15.24 GB
Ended at Jan 31, 2025, 9:42:06 PM.
----- Dependent Variables 1 in study1/Approach Curve Solver (sol1) ------------>

<---- Stationary Solver 1 in study1/Approach Curve Solver (sol1) ---------------
Started at Jan 31, 2025, 9:42:06 PM.
Segregated solver
Segregated Step 11 does not have any components solved for.
Number of degrees of freedom solved for: 776126 (plus 11525 internal DOFs).
Segregated solver iteration 1.
Merged variables
Nonsymmetric matrix found.
Scales for dependent variables:
Concentration (comp1.Cl): 1.4e+02
Concentration (comp1.K): 1.4e+02
Electric potential (comp1.V): 1.9e+02
Orthonormal null-space function used.
Iter      SolEst     Damping    Stepsize #Res #Jac #Sol   LinErr   LinRes
   1        0.82   0.3500000        0.82    2    1    1  2.5e-11  2.7e-15
   2        0.81   0.3500000        0.81    3    2    2  3.8e-11  2.7e-15
   3         0.8   0.3500000         0.8    4    3    3  2.6e-11  2.6e-15
Velocity u2, Pressure p
Nonsymmetric matrix found.
Scales for dependent variables:
Pressure (comp1.p): 1
Velocity field (spatial frame) (comp1.u2): 0.00018
Orthonormal null-space function used.
Iter      SolEst     Damping    Stepsize #Res #Jac #Sol   LinErr   LinRes
   1         1.1   0.5000000         1.1    2    1    1  1.7e-15  2.2e-15
Displacement field
Nonsymmetric matrix found.
Scales for dependent variables:
Auxiliary pressure (comp1.solid.lemm1.pw): 1
Displacement field (comp1.u): 2.8e-18
Orthonormal null-space function used.
Iter      SolEst     Damping    Stepsize #Res #Jac #Sol   LinErr   LinRes
   1         1.9   0.5000000         1.9    2    1    1  9.7e-14  1.4e-12
Spatial mesh displacement
```

```
Symmetric matrices found.
Scales for dependent variables:
Spatial mesh displacement (comp1.spatial.disp): 1.2e-09
Orthonormal null-space function used.
Iter      SolEst     Damping    Stepsize #Res #Jac #Sol   LinErr   LinRes
   1     8.5e-07   0.5000000     8.5e-07    2    1    1  1.5e-15  1.8e-15
Solution error estimates for segregated groups
1.4, 0.54, 0.96, 4.2e-07
Residual error estimates for segregated groups
7.8e+03, 8.1e+04, 2.1e+04, 0.1
Segregated solver iteration 2.
Merged variables
Scales for dependent variables:
Concentration (comp1.Cl): 1.4e+02
Concentration (comp1.K): 1.4e+02
Electric potential (comp1.V): 0.064
Iter      SolEst     Damping    Stepsize #Res #Jac #Sol   LinErr   LinRes
   1        0.78   0.3500000        0.78    6    4    4  3.9e-14  2.6e-15
   2        0.77   0.3500000        0.77    7    5    5  8.6e-14  2.6e-15
   3        0.75   0.3500000        0.75    8    6    6  5.1e-14  2.6e-15
Velocity u2, Pressure p
Scales for dependent variables:
Pressure (comp1.p): 3.7e+02
Velocity field (spatial frame) (comp1.u2): 0.00074
Iter      SolEst     Damping    Stepsize #Res #Jac #Sol   LinErr   LinRes
   1        0.36   0.5000000        0.36    4    2    2  4.6e-15  7.7e-15
Displacement field
Iter      SolEst     Damping    Stepsize #Res #Jac #Sol   LinErr   LinRes
   1        0.96   0.5000000        0.96    4    2    2    5e-14  1.5e-12
Spatial mesh displacement
Iter      SolEst     Damping    Stepsize #Res #Jac #Sol   LinErr   LinRes
   1     1.3e-06   0.5000000     1.3e-06    4    2    2  1.5e-15  1.8e-15
Solution error estimates for segregated groups
1.3, 0.18, 0.48, 6.4e-07
Residual error estimates for segregated groups
7.5e+02, 1.5e+04, 2.1e+04, 0.11
Segregated solver iteration 3.
Merged variables
Iter      SolEst     Damping    Stepsize #Res #Jac #Sol   LinErr   LinRes
   1        0.74   0.3500000        0.74   10    7    7  9.1e-14  2.6e-15
   2        0.73   0.3500000        0.73   11    8    8    1e-13  2.6e-15
   3        0.71   0.3500000        0.71   12    9    9  5.2e-14  2.6e-15
Velocity u2, Pressure p
Iter      SolEst     Damping    Stepsize #Res #Jac #Sol   LinErr   LinRes
   1        0.15   0.5000000        0.15    6    3    3  4.6e-15  7.8e-15
Displacement field
Iter      SolEst     Damping    Stepsize #Res #Jac #Sol   LinErr   LinRes
   1        0.52   0.5000000        0.52    6    3    3    1e-13  1.5e-12
Spatial mesh displacement
Iter      SolEst     Damping    Stepsize #Res #Jac #Sol   LinErr   LinRes
   1     1.3e-06   0.5000000     1.3e-06    6    3    3  1.5e-15  1.8e-15
Solution error estimates for segregated groups
1.2, 0.077, 0.26, 6.4e-07
Residual error estimates for segregated groups
2.1e+02, 8.4e+03, 1.6e+04, 0.12
Segregated solver iteration 4.
```

```
Merged variables
Iter      SolEst     Damping    Stepsize #Res #Jac #Sol   LinErr   LinRes
   1        0.69   0.3500000        0.69   14   10   10  6.9e-14  2.6e-15
   2        0.67   0.3500000        0.67   15   11   11  4.1e-14  2.6e-15
   3        0.58   0.3500000        0.58   16   12   12  1.3e-13  2.6e-15
Velocity u2, Pressure p
Iter      SolEst     Damping    Stepsize #Res #Jac #Sol   LinErr   LinRes
   1       0.072   0.5000000       0.072    8    4    4  3.4e-15  7.8e-15
Displacement field
Iter      SolEst     Damping    Stepsize #Res #Jac #Sol   LinErr   LinRes
   1        0.29   0.5000000        0.29    8    4    4  6.1e-14  1.4e-12
Spatial mesh displacement
Iter      SolEst     Damping    Stepsize #Res #Jac #Sol   LinErr   LinRes
   1     1.1e-06   0.5000000     1.1e-06    8    4    4  1.9e-15  1.8e-15
Solution error estimates for segregated groups
0.99, 0.036, 0.15, 5.3e-07
Residual error estimates for segregated groups
81, 4.4e+03, 1.1e+04, 0.13
Segregated solver iteration 5.
Merged variables
Iter      SolEst     Damping    Stepsize #Res #Jac #Sol   LinErr   LinRes
   1         0.4   0.3500000         0.4   18   13   13    4e-14  2.6e-15
   2        0.27   0.3500000        0.27   19   14   14    1e-13  2.6e-15
   3        0.18   0.3500000        0.18   20   15   15  5.2e-14  2.5e-15
Velocity u2, Pressure p
Iter      SolEst     Damping    Stepsize #Res #Jac #Sol   LinErr   LinRes
   1       0.035   0.5000000       0.035   10    5    5    3e-15  7.8e-15
Displacement field
Scales for dependent variables:
Auxiliary pressure (comp1.solid.lemm1.pw): 0.35
Displacement field (comp1.u): 2.9e-16
Iter      SolEst     Damping    Stepsize #Res #Jac #Sol   LinErr   LinRes
   1        0.17   0.5000000        0.17   10    5    5  1.4e-13  1.2e-12
Spatial mesh displacement
Iter      SolEst     Damping    Stepsize #Res #Jac #Sol   LinErr   LinRes
   1       8e-07   0.5000000       8e-07   10    5    5  1.6e-15  1.8e-15
Solution error estimates for segregated groups
0.3, 0.017, 0.084, 4e-07
Residual error estimates for segregated groups
32, 2.3e+03, 6.8e+03, 0.13
Segregated solver iteration 6.
Merged variables
Iter      SolEst     Damping    Stepsize #Res #Jac #Sol   LinErr   LinRes
   1        0.12   0.3500000        0.12   22   16   16    5e-14  2.6e-15
   2       0.075   0.3500000       0.075   23   17   17  7.4e-14  2.6e-15
   3       0.049   0.3500000       0.049   24   18   18  7.1e-14  2.7e-15
Velocity u2, Pressure p
Iter      SolEst     Damping    Stepsize #Res #Jac #Sol   LinErr   LinRes
   1       0.017   0.5000000       0.017   12    6    6  2.4e-15  7.8e-15
Displacement field
Iter      SolEst     Damping    Stepsize #Res #Jac #Sol   LinErr   LinRes
   1       0.096   0.5000000       0.096   12    6    6  1.1e-13  1.2e-12
Spatial mesh displacement
Iter      SolEst     Damping    Stepsize #Res #Jac #Sol   LinErr   LinRes
   1     5.6e-07   0.5000000     5.6e-07   12    6    6  1.4e-15  1.9e-15
Solution error estimates for segregated groups
```

```
0.084, 0.0086, 0.048, 2.8e-07
Residual error estimates for segregated groups
12, 1.2e+03, 4.2e+03, 0.13
Segregated solver iteration 7.
Merged variables
Iter      SolEst     Damping    Stepsize #Res #Jac #Sol   LinErr   LinRes
   1       0.032   0.3500000       0.032   26   19   19  5.9e-14  2.6e-15
   2       0.021   0.3500000       0.021   27   20   20  6.2e-14  2.6e-15
   3       0.014   0.3500000       0.014   28   21   21  9.9e-14  2.6e-15
Velocity u2, Pressure p
Iter      SolEst     Damping    Stepsize #Res #Jac #Sol   LinErr   LinRes
   1      0.0085   0.5000000      0.0085   14    7    7  2.6e-15  7.8e-15
Displacement field
Iter      SolEst     Damping    Stepsize #Res #Jac #Sol   LinErr   LinRes
   1       0.054   0.5000000       0.054   14    7    7  1.1e-13  1.1e-12
Spatial mesh displacement
Iter      SolEst     Damping    Stepsize #Res #Jac #Sol   LinErr   LinRes
   1     3.7e-07   0.5000000     3.7e-07   14    7    7  2.3e-15  1.8e-15
Solution error estimates for segregated groups
0.023, 0.0043, 0.027, 1.9e-07
Residual error estimates for segregated groups
4, 5.8e+02, 2.6e+03, 0.12
Segregated solver iteration 8.
Merged variables
Iter      SolEst     Damping    Stepsize #Res #Jac #Sol   LinErr   LinRes
   1      0.0088   0.3500000      0.0088   30   22   22  7.6e-14  2.6e-15
   2      0.0057   0.3500000      0.0057   31   23   23  5.8e-14  2.5e-15
   3      0.0037   0.3500000      0.0037   32   24   24  8.9e-14  2.6e-15
Velocity u2, Pressure p
Iter      SolEst     Damping    Stepsize #Res #Jac #Sol   LinErr   LinRes
   1      0.0042   0.5000000      0.0042   16    8    8  2.7e-15  7.9e-15
Displacement field
Iter      SolEst     Damping    Stepsize #Res #Jac #Sol   LinErr   LinRes
   1        0.03   0.5000000        0.03   16    8    8  1.2e-13  1.1e-12
Spatial mesh displacement
Iter      SolEst     Damping    Stepsize #Res #Jac #Sol   LinErr   LinRes
   1     2.4e-07   0.5000000     2.4e-07   16    8    8  1.7e-15  1.8e-15
Solution error estimates for segregated groups
0.0064, 0.0021, 0.015, 1.2e-07
Residual error estimates for segregated groups
1.3, 2.9e+02, 1.8e+03, 0.13
Segregated solver iteration 9.
Merged variables
Iter      SolEst     Damping    Stepsize #Res #Jac #Sol   LinErr   LinRes
   1      0.0024   0.3500000      0.0024   34   25   25  4.2e-14  2.6e-15
   2      0.0016   0.3500000      0.0016   35   26   26  7.2e-14  2.7e-15
   3       0.001   0.3500000       0.001   36   27   27    1e-13  2.6e-15
Velocity u2, Pressure p
Iter      SolEst     Damping    Stepsize #Res #Jac #Sol   LinErr   LinRes
   1      0.0021   0.5000000      0.0021   18    9    9  3.1e-15  7.8e-15
Displacement field
Iter      SolEst     Damping    Stepsize #Res #Jac #Sol   LinErr   LinRes
   1       0.017   0.5000000       0.017   18    9    9  1.2e-13  1.1e-12
Spatial mesh displacement
Iter      SolEst     Damping    Stepsize #Res #Jac #Sol   LinErr   LinRes
   1     1.5e-07   0.5000000     1.5e-07   18    9    9    2e-15  1.8e-15
```

```
Solution error estimates for segregated groups
0.0018, 0.0011, 0.0085, 7.5e-08
Residual error estimates for segregated groups
0.43, 1.5e+02, 1.4e+03, 0.13
Segregated solver iteration 10.
Merged variables
Iter      SolEst     Damping    Stepsize #Res #Jac #Sol   LinErr   LinRes
   1     0.00067   0.3500000     0.00067   38   28   28  1.1e-13  2.6e-15
   2     0.00043   0.3500000     0.00043   39   29   29  7.9e-14  2.6e-15
   3     0.00028   0.3500000     0.00028   40   30   30  5.5e-14  2.6e-15
Velocity u2, Pressure p
Iter      SolEst     Damping    Stepsize #Res #Jac #Sol   LinErr   LinRes
   1      0.0011   0.5000000      0.0011   20   10   10  2.4e-15  7.9e-15
Displacement field
Iter      SolEst     Damping    Stepsize #Res #Jac #Sol   LinErr   LinRes
   1      0.0094   0.5000000      0.0094   20   10   10  1.5e-13  1.1e-12
Spatial mesh displacement
Iter      SolEst     Damping    Stepsize #Res #Jac #Sol   LinErr   LinRes
   1     9.1e-08   0.5000000     9.1e-08   20   10   10  1.5e-15  1.8e-15
Solution error estimates for segregated groups
0.00048, 0.00053, 0.0047, 4.6e-08
Residual error estimates for segregated groups
0.14, 73, 1.2e+03, 0.12
Segregated solver iteration 11.
Merged variables
Iter      SolEst     Damping    Stepsize #Res #Jac #Sol   LinErr   LinRes
   1     0.00018   0.3500000     0.00018   42   31   31  8.4e-14  2.6e-15
   2     0.00012   0.3500000     0.00012   43   32   32  7.6e-14  2.6e-15
   3     7.7e-05   0.3500000     7.7e-05   44   33   33  3.5e-14  2.6e-15
Velocity u2, Pressure p
Iter      SolEst     Damping    Stepsize #Res #Jac #Sol   LinErr   LinRes
   1     0.00053   0.5000000     0.00053   22   11   11  2.1e-15  7.9e-15
Displacement field
Iter      SolEst     Damping    Stepsize #Res #Jac #Sol   LinErr   LinRes
   1      0.0051   0.5000000      0.0051   22   11   11  1.3e-13  1.2e-12
Spatial mesh displacement
Iter      SolEst     Damping    Stepsize #Res #Jac #Sol   LinErr   LinRes
   1     5.5e-08   0.5000000     5.5e-08   22   11   11  1.6e-15  1.8e-15
Solution error estimates for segregated groups
0.00013, 0.00026, 0.0026, 2.7e-08
Residual error estimates for segregated groups
0.043, 36, 1.2e+03, 0.11
Segregated solver iteration 12.
Merged variables
Iter      SolEst     Damping    Stepsize #Res #Jac #Sol   LinErr   LinRes
   1       5e-05   0.3500000       5e-05   46   34   34  5.8e-14  2.6e-15
   2     3.3e-05   0.3500000     3.3e-05   47   35   35    1e-13  2.6e-15
   3     2.1e-05   0.3500000     2.1e-05   48   36   36  6.5e-14  2.5e-15
Velocity u2, Pressure p
Iter      SolEst     Damping    Stepsize #Res #Jac #Sol   LinErr   LinRes
   1     0.00026   0.5000000     0.00026   24   12   12  2.5e-15  7.7e-15
Displacement field
Iter      SolEst     Damping    Stepsize #Res #Jac #Sol   LinErr   LinRes
   1      0.0028   0.5000000      0.0028   24   12   12  1.1e-13  1.1e-12
Spatial mesh displacement
Iter      SolEst     Damping    Stepsize #Res #Jac #Sol   LinErr   LinRes
```

```
   1     3.2e-08   0.5000000     3.2e-08   24   12   12  1.4e-15  1.8e-15
Solution error estimates for segregated groups
3.7e-05, 0.00013, 0.0014, 1.6e-08
Residual error estimates for segregated groups
0.013, 18, 1.1e+03, 0.12
Segregated solver iteration 13.
Merged variables
Iter      SolEst     Damping    Stepsize #Res #Jac #Sol   LinErr   LinRes
   1     1.4e-05   0.3500000     1.4e-05   50   37   37  9.3e-14  2.6e-15
   2       9e-06   0.3500000       9e-06   51   38   38    3e-14  2.6e-15
   3     5.8e-06   0.3500000     5.8e-06   52   39   39  6.9e-14  2.6e-15
Velocity u2, Pressure p
Iter      SolEst     Damping    Stepsize #Res #Jac #Sol   LinErr   LinRes
   1     0.00013   0.5000000     0.00013   26   13   13  2.5e-15  7.9e-15
Displacement field
Iter      SolEst     Damping    Stepsize #Res #Jac #Sol   LinErr   LinRes
   1      0.0015   0.5000000      0.0015   26   13   13  1.4e-13  1.1e-12
Spatial mesh displacement
Iter      SolEst     Damping    Stepsize #Res #Jac #Sol   LinErr   LinRes
   1     1.9e-08   0.5000000     1.9e-08   26   13   13  1.5e-15  1.8e-15
Solution error estimates for segregated groups
1e-05, 6.6e-05, 0.00076, 9.4e-09
Residual error estimates for segregated groups
0.004, 9.1, 1.2e+03, 0.11
Solution time: 153 s. (2 minutes, 33 seconds)
Physical memory: 4.85 GB
Virtual memory: 25.57 GB
Ended at Jan 31, 2025, 9:44:39 PM.
----- Stationary Solver 1 in study1/Approach Curve Solver (sol1) -------------->
```

d=1965 (su109)

GENERAL

Description	Value
Solution	d=1965

LOG

```
<---- Compile Equations: Stationary in study1/Approach Curve Solver (sol1) -----
Started at Jan 31, 2025, 9:44:40 PM.
Parameter d = 1.9650000000000002E-6 (m).
Geometry shape function: Linear serendipity
Number of vertex elements: 18
Number of boundary elements: 6049
Number of elements: 153129
Minimum element quality: 0.4803
Time: 8 s.
Physical memory: 3.1 GB
Virtual memory: 15.25 GB
Ended at Jan 31, 2025, 9:44:47 PM.
----- Compile Equations: Stationary in study1/Approach Curve Solver (sol1) ---->

<---- Dependent Variables 1 in study1/Approach Curve Solver (sol1) -------------
Started at Jan 31, 2025, 9:44:47 PM.
Solution time: 0 s.
Physical memory: 3.1 GB
Virtual memory: 15.25 GB
Ended at Jan 31, 2025, 9:44:47 PM.
----- Dependent Variables 1 in study1/Approach Curve Solver (sol1) ------------>

<---- Stationary Solver 1 in study1/Approach Curve Solver (sol1) ---------------
Started at Jan 31, 2025, 9:44:48 PM.
Segregated solver
Segregated Step 11 does not have any components solved for.
Number of degrees of freedom solved for: 776379 (plus 11521 internal DOFs).
Segregated solver iteration 1.
Merged variables
Nonsymmetric matrix found.
Scales for dependent variables:
Concentration (comp1.Cl): 1.4e+02
Concentration (comp1.K): 1.4e+02
Electric potential (comp1.V): 1.9e+02
Orthonormal null-space function used.
Iter      SolEst     Damping    Stepsize #Res #Jac #Sol   LinErr   LinRes
   1        0.82   0.3500000        0.82    2    1    1  1.5e-11  2.7e-15
   2        0.81   0.3500000        0.81    3    2    2  2.6e-11  2.6e-15
   3         0.8   0.3500000         0.8    4    3    3  3.2e-11  2.9e-15
Velocity u2, Pressure p
Nonsymmetric matrix found.
Scales for dependent variables:
Pressure (comp1.p): 1
Velocity field (spatial frame) (comp1.u2): 0.00018
Orthonormal null-space function used.
Iter      SolEst     Damping    Stepsize #Res #Jac #Sol   LinErr   LinRes
   1         1.1   0.5000000         1.1    2    1    1    2e-15  2.3e-15
Displacement field
Nonsymmetric matrix found.
Scales for dependent variables:
Auxiliary pressure (comp1.solid.lemm1.pw): 1
Displacement field (comp1.u): 2.8e-18
Orthonormal null-space function used.
Iter      SolEst     Damping    Stepsize #Res #Jac #Sol   LinErr   LinRes
   1         1.9   0.5000000         1.9    2    1    1  8.8e-14  1.4e-12
Spatial mesh displacement
```

```
Symmetric matrices found.
Scales for dependent variables:
Spatial mesh displacement (comp1.spatial.disp): 1.2e-09
Orthonormal null-space function used.
Iter      SolEst     Damping    Stepsize #Res #Jac #Sol   LinErr   LinRes
   1     8.5e-07   0.5000000     8.5e-07    2    1    1  1.3e-15  1.8e-15
Solution error estimates for segregated groups
1.4, 0.54, 0.96, 4.3e-07
Residual error estimates for segregated groups
7.8e+03, 8.1e+04, 2.1e+04, 0.11
Segregated solver iteration 2.
Merged variables
Scales for dependent variables:
Concentration (comp1.Cl): 1.4e+02
Concentration (comp1.K): 1.4e+02
Electric potential (comp1.V): 0.064
Iter      SolEst     Damping    Stepsize #Res #Jac #Sol   LinErr   LinRes
   1        0.78   0.3500000        0.78    6    4    4  1.3e-13  2.9e-15
   2        0.77   0.3500000        0.77    7    5    5  1.1e-13  3.7e-15
   3        0.75   0.3500000        0.75    8    6    6    1e-13  7.6e-15
Velocity u2, Pressure p
Scales for dependent variables:
Pressure (comp1.p): 3.7e+02
Velocity field (spatial frame) (comp1.u2): 0.00074
Iter      SolEst     Damping    Stepsize #Res #Jac #Sol   LinErr   LinRes
   1        0.36   0.5000000        0.36    4    2    2  2.7e-15  7.6e-15
Displacement field
Iter      SolEst     Damping    Stepsize #Res #Jac #Sol   LinErr   LinRes
   1        0.96   0.5000000        0.96    4    2    2  1.2e-13  1.4e-12
Spatial mesh displacement
Iter      SolEst     Damping    Stepsize #Res #Jac #Sol   LinErr   LinRes
   1     1.3e-06   0.5000000     1.3e-06    4    2    2  1.8e-15  1.8e-15
Solution error estimates for segregated groups
1.3, 0.18, 0.48, 6.4e-07
Residual error estimates for segregated groups
7.5e+02, 1.5e+04, 2.1e+04, 0.12
Segregated solver iteration 3.
Merged variables
Iter      SolEst     Damping    Stepsize #Res #Jac #Sol   LinErr   LinRes
   1        0.74   0.3500000        0.74   10    7    7  6.5e-14  3.1e-15
   2        0.73   0.3500000        0.73   11    8    8  1.1e-13  2.9e-15
   3        0.71   0.3500000        0.71   12    9    9  6.9e-14    3e-15
Velocity u2, Pressure p
Iter      SolEst     Damping    Stepsize #Res #Jac #Sol   LinErr   LinRes
   1        0.15   0.5000000        0.15    6    3    3  2.4e-15  7.8e-15
Displacement field
Iter      SolEst     Damping    Stepsize #Res #Jac #Sol   LinErr   LinRes
   1        0.52   0.5000000        0.52    6    3    3  7.3e-14  1.4e-12
Spatial mesh displacement
Iter      SolEst     Damping    Stepsize #Res #Jac #Sol   LinErr   LinRes
   1     1.3e-06   0.5000000     1.3e-06    6    3    3  1.6e-15  1.8e-15
Solution error estimates for segregated groups
1.2, 0.077, 0.26, 6.4e-07
Residual error estimates for segregated groups
2.1e+02, 8.6e+03, 1.6e+04, 0.12
Segregated solver iteration 4.
```

```
Merged variables
Iter      SolEst     Damping    Stepsize #Res #Jac #Sol   LinErr   LinRes
   1        0.69   0.3500000        0.69   14   10   10  7.1e-14  3.5e-15
   2        0.67   0.3500000        0.67   15   11   11  8.8e-14  4.7e-15
   3        0.58   0.3500000        0.58   16   12   12  9.6e-14  2.6e-15
Velocity u2, Pressure p
Iter      SolEst     Damping    Stepsize #Res #Jac #Sol   LinErr   LinRes
   1       0.072   0.5000000       0.072    8    4    4  2.8e-15  7.9e-15
Displacement field
Iter      SolEst     Damping    Stepsize #Res #Jac #Sol   LinErr   LinRes
   1        0.29   0.5000000        0.29    8    4    4  1.7e-13  1.5e-12
Spatial mesh displacement
Iter      SolEst     Damping    Stepsize #Res #Jac #Sol   LinErr   LinRes
   1     1.1e-06   0.5000000     1.1e-06    8    4    4  1.6e-15  1.8e-15
Solution error estimates for segregated groups
0.99, 0.036, 0.15, 5.3e-07
Residual error estimates for segregated groups
81, 4.6e+03, 1.1e+04, 0.13
Segregated solver iteration 5.
Merged variables
Iter      SolEst     Damping    Stepsize #Res #Jac #Sol   LinErr   LinRes
   1         0.4   0.3500000         0.4   18   13   13  5.8e-14    4e-15
   2        0.27   0.3500000        0.27   19   14   14  8.8e-14  3.5e-15
   3        0.18   0.3500000        0.18   20   15   15  6.6e-14  2.9e-15
Velocity u2, Pressure p
Iter      SolEst     Damping    Stepsize #Res #Jac #Sol   LinErr   LinRes
   1       0.035   0.5000000       0.035   10    5    5  3.5e-15  7.8e-15
Displacement field
Scales for dependent variables:
Auxiliary pressure (comp1.solid.lemm1.pw): 0.36
Displacement field (comp1.u): 2.9e-16
Iter      SolEst     Damping    Stepsize #Res #Jac #Sol   LinErr   LinRes
   1        0.17   0.5000000        0.17   10    5    5    1e-13  1.1e-12
Spatial mesh displacement
Iter      SolEst     Damping    Stepsize #Res #Jac #Sol   LinErr   LinRes
   1       8e-07   0.5000000       8e-07   10    5    5  2.4e-15  1.8e-15
Solution error estimates for segregated groups
0.3, 0.017, 0.084, 4e-07
Residual error estimates for segregated groups
32, 2.4e+03, 6.8e+03, 0.12
Segregated solver iteration 6.
Merged variables
Iter      SolEst     Damping    Stepsize #Res #Jac #Sol   LinErr   LinRes
   1        0.12   0.3500000        0.12   22   16   16  9.9e-14    4e-15
   2       0.075   0.3500000       0.075   23   17   17  7.1e-14  3.8e-15
   3       0.049   0.3500000       0.049   24   18   18  8.9e-14  3.5e-15
Velocity u2, Pressure p
Iter      SolEst     Damping    Stepsize #Res #Jac #Sol   LinErr   LinRes
   1       0.017   0.5000000       0.017   12    6    6  3.5e-15  7.9e-15
Displacement field
Iter      SolEst     Damping    Stepsize #Res #Jac #Sol   LinErr   LinRes
   1       0.096   0.5000000       0.096   12    6    6  1.4e-13  1.1e-12
Spatial mesh displacement
Iter      SolEst     Damping    Stepsize #Res #Jac #Sol   LinErr   LinRes
   1     5.6e-07   0.5000000     5.6e-07   12    6    6  1.6e-15  1.8e-15
Solution error estimates for segregated groups
```

```
0.084, 0.0086, 0.048, 2.8e-07
Residual error estimates for segregated groups
12, 1.2e+03, 4.2e+03, 0.11
Segregated solver iteration 7.
Merged variables
Iter      SolEst     Damping    Stepsize #Res #Jac #Sol   LinErr   LinRes
   1       0.032   0.3500000       0.032   26   19   19  4.9e-14  3.8e-15
   2       0.021   0.3500000       0.021   27   20   20  5.4e-14    5e-15
   3       0.014   0.3500000       0.014   28   21   21  7.3e-14  3.4e-15
Velocity u2, Pressure p
Iter      SolEst     Damping    Stepsize #Res #Jac #Sol   LinErr   LinRes
   1      0.0085   0.5000000      0.0085   14    7    7  2.9e-15  7.8e-15
Displacement field
Iter      SolEst     Damping    Stepsize #Res #Jac #Sol   LinErr   LinRes
   1       0.054   0.5000000       0.054   14    7    7  1.1e-13  1.1e-12
Spatial mesh displacement
Iter      SolEst     Damping    Stepsize #Res #Jac #Sol   LinErr   LinRes
   1     3.7e-07   0.5000000     3.7e-07   14    7    7  1.5e-15  1.8e-15
Solution error estimates for segregated groups
0.023, 0.0043, 0.027, 1.9e-07
Residual error estimates for segregated groups
4, 6e+02, 2.6e+03, 0.11
Segregated solver iteration 8.
Merged variables
Iter      SolEst     Damping    Stepsize #Res #Jac #Sol   LinErr   LinRes
   1      0.0088   0.3500000      0.0088   30   22   22  9.3e-14  3.2e-15
   2      0.0057   0.3500000      0.0057   31   23   23  3.7e-14  3.3e-15
   3      0.0037   0.3500000      0.0037   32   24   24  9.9e-14  2.7e-15
Velocity u2, Pressure p
Iter      SolEst     Damping    Stepsize #Res #Jac #Sol   LinErr   LinRes
   1      0.0042   0.5000000      0.0042   16    8    8    3e-15    8e-15
Displacement field
Iter      SolEst     Damping    Stepsize #Res #Jac #Sol   LinErr   LinRes
   1        0.03   0.5000000        0.03   16    8    8  1.2e-13  1.2e-12
Spatial mesh displacement
Iter      SolEst     Damping    Stepsize #Res #Jac #Sol   LinErr   LinRes
   1     2.4e-07   0.5000000     2.4e-07   16    8    8  1.4e-15  1.8e-15
Solution error estimates for segregated groups
0.0064, 0.0021, 0.015, 1.2e-07
Residual error estimates for segregated groups
1.3, 3e+02, 1.8e+03, 0.13
Segregated solver iteration 9.
Merged variables
Iter      SolEst     Damping    Stepsize #Res #Jac #Sol   LinErr   LinRes
   1      0.0024   0.3500000      0.0024   34   25   25  6.3e-14    3e-15
   2      0.0016   0.3500000      0.0016   35   26   26  3.9e-14  3.6e-15
   3       0.001   0.3500000       0.001   36   27   27  5.6e-14  3.5e-15
Velocity u2, Pressure p
Iter      SolEst     Damping    Stepsize #Res #Jac #Sol   LinErr   LinRes
   1      0.0021   0.5000000      0.0021   18    9    9  2.6e-15  7.8e-15
Displacement field
Iter      SolEst     Damping    Stepsize #Res #Jac #Sol   LinErr   LinRes
   1       0.017   0.5000000       0.017   18    9    9  1.2e-13  1.1e-12
Spatial mesh displacement
Iter      SolEst     Damping    Stepsize #Res #Jac #Sol   LinErr   LinRes
   1     1.5e-07   0.5000000     1.5e-07   18    9    9  1.7e-15  1.8e-15
```

```
Solution error estimates for segregated groups
0.0018, 0.0011, 0.0085, 7.5e-08
Residual error estimates for segregated groups
0.43, 1.5e+02, 1.4e+03, 0.12
Segregated solver iteration 10.
Merged variables
Iter      SolEst     Damping    Stepsize #Res #Jac #Sol   LinErr   LinRes
   1     0.00067   0.3500000     0.00067   38   28   28  5.7e-14  3.2e-15
   2     0.00043   0.3500000     0.00043   39   29   29    6e-14  3.8e-15
   3     0.00028   0.3500000     0.00028   40   30   30  6.8e-14  3.2e-15
Velocity u2, Pressure p
Iter      SolEst     Damping    Stepsize #Res #Jac #Sol   LinErr   LinRes
   1      0.0011   0.5000000      0.0011   20   10   10    6e-15  7.8e-15
Displacement field
Iter      SolEst     Damping    Stepsize #Res #Jac #Sol   LinErr   LinRes
   1      0.0094   0.5000000      0.0094   20   10   10  1.5e-13  1.2e-12
Spatial mesh displacement
Iter      SolEst     Damping    Stepsize #Res #Jac #Sol   LinErr   LinRes
   1     9.2e-08   0.5000000     9.2e-08   20   10   10  1.7e-15  1.8e-15
Solution error estimates for segregated groups
0.00048, 0.00053, 0.0047, 4.6e-08
Residual error estimates for segregated groups
0.14, 75, 1.2e+03, 0.11
Segregated solver iteration 11.
Merged variables
Iter      SolEst     Damping    Stepsize #Res #Jac #Sol   LinErr   LinRes
   1     0.00018   0.3500000     0.00018   42   31   31  5.3e-14  2.9e-15
   2     0.00012   0.3500000     0.00012   43   32   32  5.6e-14  3.8e-15
   3     7.7e-05   0.3500000     7.7e-05   44   33   33  7.8e-14    3e-15
Velocity u2, Pressure p
Iter      SolEst     Damping    Stepsize #Res #Jac #Sol   LinErr   LinRes
   1     0.00053   0.5000000     0.00053   22   11   11  3.7e-15    8e-15
Displacement field
Iter      SolEst     Damping    Stepsize #Res #Jac #Sol   LinErr   LinRes
   1      0.0052   0.5000000      0.0052   22   11   11  1.1e-13  1.2e-12
Spatial mesh displacement
Iter      SolEst     Damping    Stepsize #Res #Jac #Sol   LinErr   LinRes
   1     5.5e-08   0.5000000     5.5e-08   22   11   11  1.4e-15  1.8e-15
Solution error estimates for segregated groups
0.00013, 0.00026, 0.0026, 2.8e-08
Residual error estimates for segregated groups
0.043, 38, 1.2e+03, 0.13
Segregated solver iteration 12.
Merged variables
Iter      SolEst     Damping    Stepsize #Res #Jac #Sol   LinErr   LinRes
   1       5e-05   0.3500000       5e-05   46   34   34    6e-14  3.6e-15
   2     3.3e-05   0.3500000     3.3e-05   47   35   35  6.9e-14  3.6e-15
   3     2.1e-05   0.3500000     2.1e-05   48   36   36  4.8e-14  3.6e-15
Velocity u2, Pressure p
Iter      SolEst     Damping    Stepsize #Res #Jac #Sol   LinErr   LinRes
   1     0.00026   0.5000000     0.00026   24   12   12  2.3e-15  7.8e-15
Displacement field
Iter      SolEst     Damping    Stepsize #Res #Jac #Sol   LinErr   LinRes
   1      0.0028   0.5000000      0.0028   24   12   12  1.2e-13  1.2e-12
Spatial mesh displacement
Iter      SolEst     Damping    Stepsize #Res #Jac #Sol   LinErr   LinRes
```

```
   1     3.3e-08   0.5000000     3.3e-08   24   12   12  1.4e-15  1.8e-15
Solution error estimates for segregated groups
3.6e-05, 0.00013, 0.0014, 1.6e-08
Residual error estimates for segregated groups
0.013, 19, 1.2e+03, 0.13
Segregated solver iteration 13.
Merged variables
Iter      SolEst     Damping    Stepsize #Res #Jac #Sol   LinErr   LinRes
   1     1.4e-05   0.3500000     1.4e-05   50   37   37  6.6e-14  2.8e-15
   2       9e-06   0.3500000       9e-06   51   38   38  7.2e-14  3.3e-15
   3     5.8e-06   0.3500000     5.8e-06   52   39   39  6.4e-14  3.4e-15
Velocity u2, Pressure p
Iter      SolEst     Damping    Stepsize #Res #Jac #Sol   LinErr   LinRes
   1     0.00013   0.5000000     0.00013   26   13   13  2.8e-15    8e-15
Displacement field
Iter      SolEst     Damping    Stepsize #Res #Jac #Sol   LinErr   LinRes
   1      0.0015   0.5000000      0.0015   26   13   13    1e-13  1.2e-12
Spatial mesh displacement
Iter      SolEst     Damping    Stepsize #Res #Jac #Sol   LinErr   LinRes
   1     1.9e-08   0.5000000     1.9e-08   26   13   13  1.8e-15  1.8e-15
Solution error estimates for segregated groups
1e-05, 6.6e-05, 0.00076, 9.5e-09
Residual error estimates for segregated groups
0.004, 9.4, 1.2e+03, 0.12
Solution time: 151 s. (2 minutes, 31 seconds)
Physical memory: 4.8 GB
Virtual memory: 25.91 GB
Ended at Jan 31, 2025, 9:47:19 PM.
----- Stationary Solver 1 in study1/Approach Curve Solver (sol1) -------------->
```

d=1960 (su110)

GENERAL

Description	Value
Solution	d=1960

LOG

```
<---- Compile Equations: Stationary in study1/Approach Curve Solver (sol1) -----
Started at Jan 31, 2025, 9:47:19 PM.
Parameter d = 1.9600000000000003E-6 (m).
Geometry shape function: Linear serendipity
Number of vertex elements: 18
Number of boundary elements: 6045
Number of elements: 153181
Minimum element quality: 0.4675
Time: 8 s.
Physical memory: 3.11 GB
Virtual memory: 15.27 GB
Ended at Jan 31, 2025, 9:47:27 PM.
----- Compile Equations: Stationary in study1/Approach Curve Solver (sol1) ---->

<---- Dependent Variables 1 in study1/Approach Curve Solver (sol1) -------------
Started at Jan 31, 2025, 9:47:27 PM.
Solution time: 0 s.
Physical memory: 3.11 GB
Virtual memory: 15.27 GB
Ended at Jan 31, 2025, 9:47:27 PM.
----- Dependent Variables 1 in study1/Approach Curve Solver (sol1) ------------>

<---- Stationary Solver 1 in study1/Approach Curve Solver (sol1) ---------------
Started at Jan 31, 2025, 9:47:27 PM.
Segregated solver
Segregated Step 11 does not have any components solved for.
Number of degrees of freedom solved for: 776615 (plus 11513 internal DOFs).
Segregated solver iteration 1.
Merged variables
Nonsymmetric matrix found.
Scales for dependent variables:
Concentration (comp1.Cl): 1.4e+02
Concentration (comp1.K): 1.4e+02
Electric potential (comp1.V): 1.9e+02
Orthonormal null-space function used.
Iter      SolEst     Damping    Stepsize #Res #Jac #Sol   LinErr   LinRes
   1        0.82   0.3500000        0.82    2    1    1  2.5e-11  2.6e-15
   2        0.81   0.3500000        0.81    3    2    2    2e-11  2.6e-15
   3         0.8   0.3500000         0.8    4    3    3  4.4e-11  2.6e-15
Velocity u2, Pressure p
Nonsymmetric matrix found.
Scales for dependent variables:
Pressure (comp1.p): 1
Velocity field (spatial frame) (comp1.u2): 0.00018
Orthonormal null-space function used.
Iter      SolEst     Damping    Stepsize #Res #Jac #Sol   LinErr   LinRes
   1         1.1   0.5000000         1.1    2    1    1  2.3e-15  2.3e-15
Displacement field
Nonsymmetric matrix found.
Scales for dependent variables:
Auxiliary pressure (comp1.solid.lemm1.pw): 1
Displacement field (comp1.u): 2.8e-18
Orthonormal null-space function used.
Iter      SolEst     Damping    Stepsize #Res #Jac #Sol   LinErr   LinRes
   1         1.9   0.5000000         1.9    2    1    1  5.9e-14  1.4e-12
Spatial mesh displacement
```

```
Symmetric matrices found.
Scales for dependent variables:
Spatial mesh displacement (comp1.spatial.disp): 1.2e-09
Orthonormal null-space function used.
Iter      SolEst     Damping    Stepsize #Res #Jac #Sol   LinErr   LinRes
   1     8.6e-07   0.5000000     8.6e-07    2    1    1  1.5e-15  1.8e-15
Solution error estimates for segregated groups
1.4, 0.54, 0.96, 4.3e-07
Residual error estimates for segregated groups
7.8e+03, 8.1e+04, 2.1e+04, 0.11
Segregated solver iteration 2.
Merged variables
Scales for dependent variables:
Concentration (comp1.Cl): 1.4e+02
Concentration (comp1.K): 1.4e+02
Electric potential (comp1.V): 0.064
Iter      SolEst     Damping    Stepsize #Res #Jac #Sol   LinErr   LinRes
   1        0.78   0.3500000        0.78    6    4    4  2.6e-13  4.3e-15
   2        0.77   0.3500000        0.77    7    5    5  2.6e-13  1.5e-14
   3        0.75   0.3500000        0.75    8    6    6  1.9e-13  5.6e-15
Velocity u2, Pressure p
Scales for dependent variables:
Pressure (comp1.p): 3.7e+02
Velocity field (spatial frame) (comp1.u2): 0.00074
Iter      SolEst     Damping    Stepsize #Res #Jac #Sol   LinErr   LinRes
   1        0.36   0.5000000        0.36    4    2    2  3.6e-15  7.8e-15
Displacement field
Iter      SolEst     Damping    Stepsize #Res #Jac #Sol   LinErr   LinRes
   1        0.96   0.5000000        0.96    4    2    2  1.2e-13  1.4e-12
Spatial mesh displacement
Iter      SolEst     Damping    Stepsize #Res #Jac #Sol   LinErr   LinRes
   1     1.3e-06   0.5000000     1.3e-06    4    2    2  1.8e-15  1.8e-15
Solution error estimates for segregated groups
1.3, 0.18, 0.48, 6.5e-07
Residual error estimates for segregated groups
7.5e+02, 1.5e+04, 2.1e+04, 0.12
Segregated solver iteration 3.
Merged variables
Iter      SolEst     Damping    Stepsize #Res #Jac #Sol   LinErr   LinRes
   1        0.74   0.3500000        0.74   10    7    7  3.1e-13  1.8e-14
   2        0.73   0.3500000        0.73   11    8    8  2.4e-13  4.4e-15
   3        0.71   0.3500000        0.71   12    9    9  1.1e-13  7.1e-15
Velocity u2, Pressure p
Iter      SolEst     Damping    Stepsize #Res #Jac #Sol   LinErr   LinRes
   1        0.15   0.5000000        0.15    6    3    3  4.9e-15  7.7e-15
Displacement field
Iter      SolEst     Damping    Stepsize #Res #Jac #Sol   LinErr   LinRes
   1        0.52   0.5000000        0.52    6    3    3    2e-13  1.4e-12
Spatial mesh displacement
Iter      SolEst     Damping    Stepsize #Res #Jac #Sol   LinErr   LinRes
   1     1.3e-06   0.5000000     1.3e-06    6    3    3  1.3e-15  1.8e-15
Solution error estimates for segregated groups
1.2, 0.077, 0.26, 6.5e-07
Residual error estimates for segregated groups
2.1e+02, 8.5e+03, 1.6e+04, 0.12
Segregated solver iteration 4.
```

```
Merged variables
Iter      SolEst     Damping    Stepsize #Res #Jac #Sol   LinErr   LinRes
   1        0.69   0.3500000        0.69   14   10   10  2.9e-13  1.5e-14
   2        0.67   0.3500000        0.67   15   11   11    2e-13  1.9e-14
   3        0.58   0.3500000        0.58   16   12   12  3.5e-13  1.1e-14
Velocity u2, Pressure p
Iter      SolEst     Damping    Stepsize #Res #Jac #Sol   LinErr   LinRes
   1       0.072   0.5000000       0.072    8    4    4  4.7e-15  7.8e-15
Displacement field
Iter      SolEst     Damping    Stepsize #Res #Jac #Sol   LinErr   LinRes
   1        0.29   0.5000000        0.29    8    4    4  8.7e-14  1.4e-12
Spatial mesh displacement
Iter      SolEst     Damping    Stepsize #Res #Jac #Sol   LinErr   LinRes
   1     1.1e-06   0.5000000     1.1e-06    8    4    4  1.4e-15  1.8e-15
Solution error estimates for segregated groups
0.99, 0.036, 0.15, 5.4e-07
Residual error estimates for segregated groups
81, 4.5e+03, 1.1e+04, 0.13
Segregated solver iteration 5.
Merged variables
Iter      SolEst     Damping    Stepsize #Res #Jac #Sol   LinErr   LinRes
   1         0.4   0.3500000         0.4   18   13   13  1.1e-13  3.8e-15
   2        0.27   0.3500000        0.27   19   14   14  4.6e-13  6.2e-15
   3        0.18   0.3500000        0.18   20   15   15    1e-13  6.7e-15
Velocity u2, Pressure p
Iter      SolEst     Damping    Stepsize #Res #Jac #Sol   LinErr   LinRes
   1       0.035   0.5000000       0.035   10    5    5  3.5e-15  7.8e-15
Displacement field
Scales for dependent variables:
Auxiliary pressure (comp1.solid.lemm1.pw): 0.36
Displacement field (comp1.u): 2.9e-16
Iter      SolEst     Damping    Stepsize #Res #Jac #Sol   LinErr   LinRes
   1        0.17   0.5000000        0.17   10    5    5  1.2e-13  1.1e-12
Spatial mesh displacement
Iter      SolEst     Damping    Stepsize #Res #Jac #Sol   LinErr   LinRes
   1     8.1e-07   0.5000000     8.1e-07   10    5    5  1.4e-15  1.8e-15
Solution error estimates for segregated groups
0.3, 0.017, 0.084, 4e-07
Residual error estimates for segregated groups
32, 2.3e+03, 6.8e+03, 0.11
Segregated solver iteration 6.
Merged variables
Iter      SolEst     Damping    Stepsize #Res #Jac #Sol   LinErr   LinRes
   1        0.12   0.3500000        0.12   22   16   16  2.3e-13  5.4e-15
   2       0.075   0.3500000       0.075   23   17   17  4.4e-13  4.5e-15
   3       0.049   0.3500000       0.049   24   18   18  2.9e-13    9e-15
Velocity u2, Pressure p
Iter      SolEst     Damping    Stepsize #Res #Jac #Sol   LinErr   LinRes
   1       0.017   0.5000000       0.017   12    6    6  9.1e-15  7.8e-15
Displacement field
Iter      SolEst     Damping    Stepsize #Res #Jac #Sol   LinErr   LinRes
   1       0.096   0.5000000       0.096   12    6    6  2.1e-13  1.2e-12
Spatial mesh displacement
Iter      SolEst     Damping    Stepsize #Res #Jac #Sol   LinErr   LinRes
   1     5.7e-07   0.5000000     5.7e-07   12    6    6  1.3e-15  1.8e-15
Solution error estimates for segregated groups
```

```
0.084, 0.0086, 0.048, 2.8e-07
Residual error estimates for segregated groups
12, 1.2e+03, 4.2e+03, 0.11
Segregated solver iteration 7.
Merged variables
Iter      SolEst     Damping    Stepsize #Res #Jac #Sol   LinErr   LinRes
   1       0.032   0.3500000       0.032   26   19   19  3.6e-13  5.7e-15
   2       0.021   0.3500000       0.021   27   20   20  2.3e-13    1e-14
   3       0.014   0.3500000       0.014   28   21   21    3e-13  6.1e-15
Velocity u2, Pressure p
Iter      SolEst     Damping    Stepsize #Res #Jac #Sol   LinErr   LinRes
   1      0.0085   0.5000000      0.0085   14    7    7  4.5e-15  7.9e-15
Displacement field
Iter      SolEst     Damping    Stepsize #Res #Jac #Sol   LinErr   LinRes
   1       0.054   0.5000000       0.054   14    7    7  1.3e-13  1.2e-12
Spatial mesh displacement
Iter      SolEst     Damping    Stepsize #Res #Jac #Sol   LinErr   LinRes
   1     3.8e-07   0.5000000     3.8e-07   14    7    7  1.3e-15  1.8e-15
Solution error estimates for segregated groups
0.023, 0.0042, 0.027, 1.9e-07
Residual error estimates for segregated groups
4, 5.9e+02, 2.6e+03, 0.11
Segregated solver iteration 8.
Merged variables
Iter      SolEst     Damping    Stepsize #Res #Jac #Sol   LinErr   LinRes
   1      0.0088   0.3500000      0.0088   30   22   22  4.6e-13  1.3e-14
   2      0.0057   0.3500000      0.0057   31   23   23  3.6e-13  6.5e-15
   3      0.0037   0.3500000      0.0037   32   24   24  4.3e-13  7.9e-15
Velocity u2, Pressure p
Iter      SolEst     Damping    Stepsize #Res #Jac #Sol   LinErr   LinRes
   1      0.0042   0.5000000      0.0042   16    8    8  3.4e-15  7.9e-15
Displacement field
Iter      SolEst     Damping    Stepsize #Res #Jac #Sol   LinErr   LinRes
   1        0.03   0.5000000        0.03   16    8    8  1.1e-13  1.1e-12
Spatial mesh displacement
Iter      SolEst     Damping    Stepsize #Res #Jac #Sol   LinErr   LinRes
   1     2.4e-07   0.5000000     2.4e-07   16    8    8  1.1e-15  1.8e-15
Solution error estimates for segregated groups
0.0064, 0.0021, 0.015, 1.2e-07
Residual error estimates for segregated groups
1.3, 3e+02, 1.8e+03, 0.13
Segregated solver iteration 9.
Merged variables
Iter      SolEst     Damping    Stepsize #Res #Jac #Sol   LinErr   LinRes
   1      0.0024   0.3500000      0.0024   34   25   25  2.2e-13  9.9e-15
   2      0.0016   0.3500000      0.0016   35   26   26  1.7e-13  5.1e-15
   3       0.001   0.3500000       0.001   36   27   27  1.9e-13  4.3e-15
Velocity u2, Pressure p
Iter      SolEst     Damping    Stepsize #Res #Jac #Sol   LinErr   LinRes
   1      0.0021   0.5000000      0.0021   18    9    9  5.1e-15  7.9e-15
Displacement field
Iter      SolEst     Damping    Stepsize #Res #Jac #Sol   LinErr   LinRes
   1       0.017   0.5000000       0.017   18    9    9  1.3e-13  1.1e-12
Spatial mesh displacement
Iter      SolEst     Damping    Stepsize #Res #Jac #Sol   LinErr   LinRes
   1     1.5e-07   0.5000000     1.5e-07   18    9    9    2e-15  1.8e-15
```

```
Solution error estimates for segregated groups
0.0018, 0.0011, 0.0085, 7.6e-08
Residual error estimates for segregated groups
0.43, 1.5e+02, 1.4e+03, 0.12
Segregated solver iteration 10.
Merged variables
Iter      SolEst     Damping    Stepsize #Res #Jac #Sol   LinErr   LinRes
   1     0.00067   0.3500000     0.00067   38   28   28    2e-13  7.7e-15
   2     0.00043   0.3500000     0.00043   39   29   29  3.8e-13    5e-15
   3     0.00028   0.3500000     0.00028   40   30   30  2.1e-13  8.5e-15
Velocity u2, Pressure p
Iter      SolEst     Damping    Stepsize #Res #Jac #Sol   LinErr   LinRes
   1      0.0011   0.5000000      0.0011   20   10   10  3.5e-15    8e-15
Displacement field
Iter      SolEst     Damping    Stepsize #Res #Jac #Sol   LinErr   LinRes
   1      0.0094   0.5000000      0.0094   20   10   10  1.5e-13  1.1e-12
Spatial mesh displacement
Iter      SolEst     Damping    Stepsize #Res #Jac #Sol   LinErr   LinRes
   1     9.3e-08   0.5000000     9.3e-08   20   10   10  1.5e-15  1.8e-15
Solution error estimates for segregated groups
0.00048, 0.00053, 0.0047, 4.6e-08
Residual error estimates for segregated groups
0.14, 74, 1.2e+03, 0.12
Segregated solver iteration 11.
Merged variables
Iter      SolEst     Damping    Stepsize #Res #Jac #Sol   LinErr   LinRes
   1     0.00018   0.3500000     0.00018   42   31   31    3e-13  4.4e-15
   2     0.00012   0.3500000     0.00012   43   32   32  1.3e-13  1.3e-14
   3     7.7e-05   0.3500000     7.7e-05   44   33   33  1.1e-13  2.8e-14
Velocity u2, Pressure p
Iter      SolEst     Damping    Stepsize #Res #Jac #Sol   LinErr   LinRes
   1     0.00053   0.5000000     0.00053   22   11   11  4.2e-15  7.8e-15
Displacement field
Iter      SolEst     Damping    Stepsize #Res #Jac #Sol   LinErr   LinRes
   1      0.0052   0.5000000      0.0052   22   11   11  1.2e-13  1.2e-12
Spatial mesh displacement
Iter      SolEst     Damping    Stepsize #Res #Jac #Sol   LinErr   LinRes
   1     5.6e-08   0.5000000     5.6e-08   22   11   11  1.5e-15  1.8e-15
Solution error estimates for segregated groups
0.00013, 0.00026, 0.0026, 2.8e-08
Residual error estimates for segregated groups
0.043, 37, 1.2e+03, 0.12
Segregated solver iteration 12.
Merged variables
Iter      SolEst     Damping    Stepsize #Res #Jac #Sol   LinErr   LinRes
   1       5e-05   0.3500000       5e-05   46   34   34  3.1e-13    5e-15
   2     3.3e-05   0.3500000     3.3e-05   47   35   35  2.2e-13  6.9e-15
   3     2.1e-05   0.3500000     2.1e-05   48   36   36  3.2e-13  4.9e-15
Velocity u2, Pressure p
Iter      SolEst     Damping    Stepsize #Res #Jac #Sol   LinErr   LinRes
   1     0.00026   0.5000000     0.00026   24   12   12  2.3e-15  7.8e-15
Displacement field
Iter      SolEst     Damping    Stepsize #Res #Jac #Sol   LinErr   LinRes
   1      0.0028   0.5000000      0.0028   24   12   12  1.4e-13  1.1e-12
Spatial mesh displacement
Iter      SolEst     Damping    Stepsize #Res #Jac #Sol   LinErr   LinRes
```

```
   1      3.3e-08   0.5000000     3.3e-08   24    12    12  1.5e-15  1.8e-15
Solution error estimates for segregated groups
3.6e-05, 0.00013, 0.0014, 1.6e-08
Residual error estimates for segregated groups
0.013, 19, 1.2e+03, 0.12
Segregated solver iteration 13.
Merged variables
Iter      SolEst     Damping    Stepsize #Res #Jac #Sol   LinErr   LinRes
   1      1.4e-05   0.3500000     1.4e-05   50    37    37  2.3e-13  4.3e-15
   2        9e-06   0.3500000       9e-06   51    38    38  1.1e-13  1.5e-14
   3      5.8e-06   0.3500000     5.8e-06   52    39    39  1.7e-13  2.2e-14
Velocity u2, Pressure p
Iter      SolEst     Damping    Stepsize #Res #Jac #Sol   LinErr   LinRes
   1      0.00013   0.5000000     0.00013   26    13    13  2.2e-15  7.9e-15
Displacement field
Iter      SolEst     Damping    Stepsize #Res #Jac #Sol   LinErr   LinRes
   1       0.0015   0.5000000      0.0015   26    13    13  1.1e-13  1.1e-12
Spatial mesh displacement
Iter      SolEst     Damping    Stepsize #Res #Jac #Sol   LinErr   LinRes
   1      1.9e-08   0.5000000     1.9e-08   26    13    13  1.8e-15  1.8e-15
Solution error estimates for segregated groups
1e-05, 6.6e-05, 0.00076, 9.6e-09
Residual error estimates for segregated groups
0.004, 9.3, 1.2e+03, 0.12
Solution time: 153 s. (2 minutes, 33 seconds)
Physical memory: 4.84 GB
Virtual memory: 25.55 GB
Ended at Jan 31, 2025, 9:50:00 PM.
----- Stationary Solver 1 in study1/Approach Curve Solver (sol1) -------------->
```

d=1955 (su111)

GENERAL

Description	Value
Solution	d=1955

LOG

```
<---- Compile Equations: Stationary in study1/Approach Curve Solver (sol1) -----
Started at Jan 31, 2025, 9:50:00 PM.
Parameter d = 1.955E-6 (m).
Geometry shape function: Linear serendipity
Number of vertex elements: 18
Number of boundary elements: 6043
Number of elements: 153199
Minimum element quality: 0.4732
Time: 8 s.
Physical memory: 3.1 GB
Virtual memory: 15.25 GB
Ended at Jan 31, 2025, 9:50:08 PM.
----- Compile Equations: Stationary in study1/Approach Curve Solver (sol1) ---->

<---- Dependent Variables 1 in study1/Approach Curve Solver (sol1) -------------
Started at Jan 31, 2025, 9:50:08 PM.
Solution time: 0 s.
Physical memory: 3.1 GB
Virtual memory: 15.25 GB
Ended at Jan 31, 2025, 9:50:08 PM.
----- Dependent Variables 1 in study1/Approach Curve Solver (sol1) ------------>

<---- Stationary Solver 1 in study1/Approach Curve Solver (sol1) ---------------
Started at Jan 31, 2025, 9:50:08 PM.
Segregated solver
Segregated Step 11 does not have any components solved for.
Number of degrees of freedom solved for: 776727 (plus 11509 internal DOFs).
Segregated solver iteration 1.
Merged variables
Nonsymmetric matrix found.
Scales for dependent variables:
Concentration (comp1.Cl): 1.4e+02
Concentration (comp1.K): 1.4e+02
Electric potential (comp1.V): 1.9e+02
Orthonormal null-space function used.
Iter      SolEst     Damping    Stepsize #Res #Jac #Sol   LinErr   LinRes
   1        0.82   0.3500000        0.82    2    1    1  3.3e-11  2.7e-15
   2        0.81   0.3500000        0.81    3    2    2  1.5e-11  2.7e-15
   3         0.8   0.3500000         0.8    4    3    3  1.8e-11  2.6e-15
Velocity u2, Pressure p
Nonsymmetric matrix found.
Scales for dependent variables:
Pressure (comp1.p): 1
Velocity field (spatial frame) (comp1.u2): 0.00018
Orthonormal null-space function used.
Iter      SolEst     Damping    Stepsize #Res #Jac #Sol   LinErr   LinRes
   1         1.1   0.5000000         1.1    2    1    1  2.1e-15  2.2e-15
Displacement field
Nonsymmetric matrix found.
Scales for dependent variables:
Auxiliary pressure (comp1.solid.lemm1.pw): 1
Displacement field (comp1.u): 2.8e-18
Orthonormal null-space function used.
Iter      SolEst     Damping    Stepsize #Res #Jac #Sol   LinErr   LinRes
   1         1.9   0.5000000         1.9    2    1    1    1e-13  1.5e-12
Spatial mesh displacement
```

```
Symmetric matrices found.
Scales for dependent variables:
Spatial mesh displacement (comp1.spatial.disp): 1.2e-09
Orthonormal null-space function used.
Iter      SolEst     Damping    Stepsize #Res #Jac #Sol   LinErr   LinRes
   1     8.7e-07   0.5000000     8.7e-07    2    1    1  1.8e-15  1.9e-15
Solution error estimates for segregated groups
1.4, 0.54, 0.96, 4.3e-07
Residual error estimates for segregated groups
7.8e+03, 8.1e+04, 2.1e+04, 0.1
Segregated solver iteration 2.
Merged variables
Scales for dependent variables:
Concentration (comp1.Cl): 1.4e+02
Concentration (comp1.K): 1.4e+02
Electric potential (comp1.V): 0.064
Iter      SolEst     Damping    Stepsize #Res #Jac #Sol   LinErr   LinRes
   1        0.78   0.3500000        0.78    6    4    4  1.2e-13  6.3e-15
   2        0.77   0.3500000        0.77    7    5    5  8.9e-14  3.4e-15
   3        0.75   0.3500000        0.75    8    6    6  7.9e-14  6.4e-15
Velocity u2, Pressure p
Scales for dependent variables:
Pressure (comp1.p): 3.7e+02
Velocity field (spatial frame) (comp1.u2): 0.00074
Iter      SolEst     Damping    Stepsize #Res #Jac #Sol   LinErr   LinRes
   1        0.36   0.5000000        0.36    4    2    2  2.3e-15  7.9e-15
Displacement field
Iter      SolEst     Damping    Stepsize #Res #Jac #Sol   LinErr   LinRes
   1        0.96   0.5000000        0.96    4    2    2  6.4e-14  1.4e-12
Spatial mesh displacement
Iter      SolEst     Damping    Stepsize #Res #Jac #Sol   LinErr   LinRes
   1     1.3e-06   0.5000000     1.3e-06    4    2    2  1.8e-15  1.9e-15
Solution error estimates for segregated groups
1.3, 0.18, 0.48, 6.5e-07
Residual error estimates for segregated groups
7.5e+02, 1.3e+04, 2.1e+04, 0.11
Segregated solver iteration 3.
Merged variables
Iter      SolEst     Damping    Stepsize #Res #Jac #Sol   LinErr   LinRes
   1        0.74   0.3500000        0.74   10    7    7  5.9e-14  3.4e-15
   2        0.73   0.3500000        0.73   11    8    8  1.1e-13  6.1e-15
   3        0.71   0.3500000        0.71   12    9    9    8e-14  5.6e-15
Velocity u2, Pressure p
Iter      SolEst     Damping    Stepsize #Res #Jac #Sol   LinErr   LinRes
   1        0.15   0.5000000        0.15    6    3    3  2.2e-15  7.9e-15
Displacement field
Iter      SolEst     Damping    Stepsize #Res #Jac #Sol   LinErr   LinRes
   1        0.52   0.5000000        0.52    6    3    3  6.6e-14  1.4e-12
Spatial mesh displacement
Iter      SolEst     Damping    Stepsize #Res #Jac #Sol   LinErr   LinRes
   1     1.3e-06   0.5000000     1.3e-06    6    3    3  1.9e-15  1.9e-15
Solution error estimates for segregated groups
1.2, 0.077, 0.26, 6.5e-07
Residual error estimates for segregated groups
2.1e+02, 7.4e+03, 1.6e+04, 0.12
Segregated solver iteration 4.
```

```
Merged variables
Iter      SolEst     Damping    Stepsize #Res #Jac #Sol   LinErr   LinRes
   1        0.69   0.3500000        0.69   14   10   10  1.5e-13  5.9e-15
   2        0.67   0.3500000        0.67   15   11   11  5.3e-14  4.9e-15
   3        0.58   0.3500000        0.58   16   12   12  1.1e-13  4.3e-15
Velocity u2, Pressure p
Iter      SolEst     Damping    Stepsize #Res #Jac #Sol   LinErr   LinRes
   1       0.072   0.5000000       0.072    8    4    4  2.2e-15  7.8e-15
Displacement field
Iter      SolEst     Damping    Stepsize #Res #Jac #Sol   LinErr   LinRes
   1        0.29   0.5000000        0.29    8    4    4    9e-14  1.4e-12
Spatial mesh displacement
Iter      SolEst     Damping    Stepsize #Res #Jac #Sol   LinErr   LinRes
   1     1.1e-06   0.5000000     1.1e-06    8    4    4  1.6e-15  1.9e-15
Solution error estimates for segregated groups
0.99, 0.036, 0.15, 5.4e-07
Residual error estimates for segregated groups
81, 3.9e+03, 1.1e+04, 0.12
Segregated solver iteration 5.
Merged variables
Iter      SolEst     Damping    Stepsize #Res #Jac #Sol   LinErr   LinRes
   1         0.4   0.3500000         0.4   18   13   13  8.3e-14  4.6e-15
   2        0.27   0.3500000        0.27   19   14   14  1.2e-13  5.4e-15
   3        0.18   0.3500000        0.18   20   15   15  9.8e-14  3.6e-15
Velocity u2, Pressure p
Iter      SolEst     Damping    Stepsize #Res #Jac #Sol   LinErr   LinRes
   1       0.035   0.5000000       0.035   10    5    5  2.8e-15  8.1e-15
Displacement field
Scales for dependent variables:
Auxiliary pressure (comp1.solid.lemm1.pw): 0.36
Displacement field (comp1.u): 3e-16
Iter      SolEst     Damping    Stepsize #Res #Jac #Sol   LinErr   LinRes
   1        0.17   0.5000000        0.17   10    5    5  1.3e-13  1.1e-12
Spatial mesh displacement
Iter      SolEst     Damping    Stepsize #Res #Jac #Sol   LinErr   LinRes
   1     8.2e-07   0.5000000     8.2e-07   10    5    5  1.6e-15  1.8e-15
Solution error estimates for segregated groups
0.3, 0.017, 0.084, 4.1e-07
Residual error estimates for segregated groups
32, 2e+03, 6.8e+03, 0.12
Segregated solver iteration 6.
Merged variables
Iter      SolEst     Damping    Stepsize #Res #Jac #Sol   LinErr   LinRes
   1        0.12   0.3500000        0.12   22   16   16  1.3e-13  4.7e-15
   2       0.075   0.3500000       0.075   23   17   17  8.2e-14  4.7e-15
   3       0.049   0.3500000       0.049   24   18   18  1.6e-13  6.5e-15
Velocity u2, Pressure p
Iter      SolEst     Damping    Stepsize #Res #Jac #Sol   LinErr   LinRes
   1       0.017   0.5000000       0.017   12    6    6  2.6e-15  7.8e-15
Displacement field
Iter      SolEst     Damping    Stepsize #Res #Jac #Sol   LinErr   LinRes
   1       0.096   0.5000000       0.096   12    6    6  1.4e-13  1.1e-12
Spatial mesh displacement
Iter      SolEst     Damping    Stepsize #Res #Jac #Sol   LinErr   LinRes
   1     5.7e-07   0.5000000     5.7e-07   12    6    6  1.8e-15  1.8e-15
Solution error estimates for segregated groups
```

```
0.084, 0.0086, 0.048, 2.9e-07
Residual error estimates for segregated groups
12, 1e+03, 4.2e+03, 0.12
Segregated solver iteration 7.
Merged variables
Iter      SolEst     Damping    Stepsize #Res #Jac #Sol   LinErr   LinRes
   1       0.032   0.3500000       0.032   26   19   19  9.3e-14  3.6e-15
   2       0.021   0.3500000       0.021   27   20   20  1.1e-13  3.7e-15
   3       0.014   0.3500000       0.014   28   21   21    1e-13    4e-15
Velocity u2, Pressure p
Iter      SolEst     Damping    Stepsize #Res #Jac #Sol   LinErr   LinRes
   1      0.0085   0.5000000      0.0085   14    7    7  3.4e-15    8e-15
Displacement field
Iter      SolEst     Damping    Stepsize #Res #Jac #Sol   LinErr   LinRes
   1       0.054   0.5000000       0.054   14    7    7  1.1e-13  1.1e-12
Spatial mesh displacement
Iter      SolEst     Damping    Stepsize #Res #Jac #Sol   LinErr   LinRes
   1     3.8e-07   0.5000000     3.8e-07   14    7    7  1.5e-15  1.9e-15
Solution error estimates for segregated groups
0.023, 0.0042, 0.027, 1.9e-07
Residual error estimates for segregated groups
4, 5.1e+02, 2.6e+03, 0.11
Segregated solver iteration 8.
Merged variables
Iter      SolEst     Damping    Stepsize #Res #Jac #Sol   LinErr   LinRes
   1      0.0088   0.3500000      0.0088   30   22   22    9e-14  4.3e-15
   2      0.0057   0.3500000      0.0057   31   23   23  2.1e-13  4.7e-15
   3      0.0037   0.3500000      0.0037   32   24   24    1e-13  3.9e-15
Velocity u2, Pressure p
Iter      SolEst     Damping    Stepsize #Res #Jac #Sol   LinErr   LinRes
   1      0.0042   0.5000000      0.0042   16    8    8  2.3e-15  8.1e-15
Displacement field
Iter      SolEst     Damping    Stepsize #Res #Jac #Sol   LinErr   LinRes
   1        0.03   0.5000000        0.03   16    8    8  1.3e-13  1.2e-12
Spatial mesh displacement
Iter      SolEst     Damping    Stepsize #Res #Jac #Sol   LinErr   LinRes
   1     2.4e-07   0.5000000     2.4e-07   16    8    8  1.4e-15  1.8e-15
Solution error estimates for segregated groups
0.0064, 0.0021, 0.015, 1.2e-07
Residual error estimates for segregated groups
1.3, 2.6e+02, 1.8e+03, 0.11
Segregated solver iteration 9.
Merged variables
Iter      SolEst     Damping    Stepsize #Res #Jac #Sol   LinErr   LinRes
   1      0.0024   0.3500000      0.0024   34   25   25  7.2e-14  5.3e-15
   2      0.0016   0.3500000      0.0016   35   26   26  1.4e-13  3.7e-15
   3       0.001   0.3500000       0.001   36   27   27  7.6e-14  3.6e-15
Velocity u2, Pressure p
Iter      SolEst     Damping    Stepsize #Res #Jac #Sol   LinErr   LinRes
   1      0.0021   0.5000000      0.0021   18    9    9  2.9e-15  7.9e-15
Displacement field
Iter      SolEst     Damping    Stepsize #Res #Jac #Sol   LinErr   LinRes
   1       0.017   0.5000000       0.017   18    9    9  1.2e-13  1.1e-12
Spatial mesh displacement
Iter      SolEst     Damping    Stepsize #Res #Jac #Sol   LinErr   LinRes
   1     1.5e-07   0.5000000     1.5e-07   18    9    9  1.7e-15  1.9e-15
```

```
Solution error estimates for segregated groups
0.0018, 0.0011, 0.0085, 7.6e-08
Residual error estimates for segregated groups
0.43, 1.3e+02, 1.4e+03, 0.12
Segregated solver iteration 10.
Merged variables
Iter      SolEst     Damping    Stepsize #Res #Jac #Sol   LinErr   LinRes
   1     0.00067   0.3500000     0.00067   38   28   28  6.9e-14  3.4e-15
   2     0.00043   0.3500000     0.00043   39   29   29  1.4e-13  4.4e-15
   3     0.00028   0.3500000     0.00028   40   30   30  1.1e-13  3.4e-15
Velocity u2, Pressure p
Iter      SolEst     Damping    Stepsize #Res #Jac #Sol   LinErr   LinRes
   1      0.0011   0.5000000      0.0011   20   10   10  3.4e-15  7.9e-15
Displacement field
Iter      SolEst     Damping    Stepsize #Res #Jac #Sol   LinErr   LinRes
   1      0.0094   0.5000000      0.0094   20   10   10  1.5e-13  1.1e-12
Spatial mesh displacement
Iter      SolEst     Damping    Stepsize #Res #Jac #Sol   LinErr   LinRes
   1     9.3e-08   0.5000000     9.3e-08   20   10   10  1.7e-15  1.8e-15
Solution error estimates for segregated groups
0.00048, 0.00053, 0.0047, 4.7e-08
Residual error estimates for segregated groups
0.14, 64, 1.3e+03, 0.11
Segregated solver iteration 11.
Merged variables
Iter      SolEst     Damping    Stepsize #Res #Jac #Sol   LinErr   LinRes
   1     0.00018   0.3500000     0.00018   42   31   31  1.2e-13  6.6e-15
   2     0.00012   0.3500000     0.00012   43   32   32  1.1e-13  3.3e-15
   3     7.7e-05   0.3500000     7.7e-05   44   33   33  1.8e-13  4.4e-15
Velocity u2, Pressure p
Iter      SolEst     Damping    Stepsize #Res #Jac #Sol   LinErr   LinRes
   1     0.00053   0.5000000     0.00053   22   11   11  3.5e-15  7.9e-15
Displacement field
Iter      SolEst     Damping    Stepsize #Res #Jac #Sol   LinErr   LinRes
   1      0.0052   0.5000000      0.0052   22   11   11    1e-13  1.1e-12
Spatial mesh displacement
Iter      SolEst     Damping    Stepsize #Res #Jac #Sol   LinErr   LinRes
   1     5.6e-08   0.5000000     5.6e-08   22   11   11  1.9e-15  1.8e-15
Solution error estimates for segregated groups
0.00013, 0.00026, 0.0026, 2.8e-08
Residual error estimates for segregated groups
0.043, 32, 1.2e+03, 0.12
Segregated solver iteration 12.
Merged variables
Iter      SolEst     Damping    Stepsize #Res #Jac #Sol   LinErr   LinRes
   1       5e-05   0.3500000       5e-05   46   34   34  1.1e-13  3.9e-15
   2     3.3e-05   0.3500000     3.3e-05   47   35   35  1.6e-13  6.7e-15
   3     2.1e-05   0.3500000     2.1e-05   48   36   36  1.1e-13  3.5e-15
Velocity u2, Pressure p
Iter      SolEst     Damping    Stepsize #Res #Jac #Sol   LinErr   LinRes
   1     0.00026   0.5000000     0.00026   24   12   12  2.5e-15  7.9e-15
Displacement field
Iter      SolEst     Damping    Stepsize #Res #Jac #Sol   LinErr   LinRes
   1      0.0028   0.5000000      0.0028   24   12   12  1.9e-13  1.1e-12
Spatial mesh displacement
Iter      SolEst     Damping    Stepsize #Res #Jac #Sol   LinErr   LinRes
```

```
   1     3.3e-08   0.5000000     3.3e-08   24   12   12  1.6e-15  1.8e-15
Solution error estimates for segregated groups
3.6e-05, 0.00013, 0.0014, 1.7e-08
Residual error estimates for segregated groups
0.013, 16, 1.2e+03, 0.12
Segregated solver iteration 13.
Merged variables
Iter      SolEst     Damping    Stepsize #Res #Jac #Sol   LinErr   LinRes
   1     1.4e-05   0.3500000     1.4e-05   50   37   37  6.5e-14  5.7e-15
   2       9e-06   0.3500000       9e-06   51   38   38  1.1e-13  3.4e-15
   3     5.8e-06   0.3500000     5.8e-06   52   39   39  1.6e-13  5.8e-15
Velocity u2, Pressure p
Iter      SolEst     Damping    Stepsize #Res #Jac #Sol   LinErr   LinRes
   1     0.00013   0.5000000     0.00013   26   13   13  2.2e-15    8e-15
Displacement field
Iter      SolEst     Damping    Stepsize #Res #Jac #Sol   LinErr   LinRes
   1      0.0015   0.5000000      0.0015   26   13   13  1.4e-13  1.1e-12
Spatial mesh displacement
Iter      SolEst     Damping    Stepsize #Res #Jac #Sol   LinErr   LinRes
   1     1.9e-08   0.5000000     1.9e-08   26   13   13  1.3e-15  1.9e-15
Solution error estimates for segregated groups
1e-05, 6.6e-05, 0.00076, 9.7e-09
Residual error estimates for segregated groups
0.004, 8, 1.2e+03, 0.11
Solution time: 152 s. (2 minutes, 32 seconds)
Physical memory: 4.86 GB
Virtual memory: 25.91 GB
Ended at Jan 31, 2025, 9:52:40 PM.
----- Stationary Solver 1 in study1/Approach Curve Solver (sol1) -------------->
```

d=1950 (su112)

GENERAL

Description	Value
Solution	d=1950

LOG

```
<---- Compile Equations: Stationary in study1/Approach Curve Solver (sol1) -----
Started at Jan 31, 2025, 9:52:40 PM.
Parameter d = 1.95E-6 (m).
Geometry shape function: Linear serendipity
Number of vertex elements: 18
Number of boundary elements: 6040
Number of elements: 153266
Minimum element quality: 0.4827
Time: 8 s.
Physical memory: 3.1 GB
Virtual memory: 15.25 GB
Ended at Jan 31, 2025, 9:52:48 PM.
----- Compile Equations: Stationary in study1/Approach Curve Solver (sol1) ---->

<---- Dependent Variables 1 in study1/Approach Curve Solver (sol1) -------------
Started at Jan 31, 2025, 9:52:48 PM.
Solution time: 0 s.
Physical memory: 3.1 GB
Virtual memory: 15.25 GB
Ended at Jan 31, 2025, 9:52:48 PM.
----- Dependent Variables 1 in study1/Approach Curve Solver (sol1) ------------>

<---- Stationary Solver 1 in study1/Approach Curve Solver (sol1) ---------------
Started at Jan 31, 2025, 9:52:48 PM.
Segregated solver
Segregated Step 11 does not have any components solved for.
Number of degrees of freedom solved for: 776906 (plus 11503 internal DOFs).
Segregated solver iteration 1.
Merged variables
Nonsymmetric matrix found.
Scales for dependent variables:
Concentration (comp1.Cl): 1.4e+02
Concentration (comp1.K): 1.4e+02
Electric potential (comp1.V): 1.9e+02
Orthonormal null-space function used.
Iter      SolEst     Damping    Stepsize #Res #Jac #Sol   LinErr   LinRes
   1        0.82   0.3500000        0.82    2    1    1  6.5e-11  2.7e-15
   2        0.81   0.3500000        0.81    3    2    2  4.5e-11  2.6e-15
   3        0.79   0.3500000        0.79    4    3    3  2.1e-11  2.6e-15
Velocity u2, Pressure p
Nonsymmetric matrix found.
Scales for dependent variables:
Pressure (comp1.p): 1
Velocity field (spatial frame) (comp1.u2): 0.00018
Orthonormal null-space function used.
Iter      SolEst     Damping    Stepsize #Res #Jac #Sol   LinErr   LinRes
   1         1.1   0.5000000         1.1    2    1    1  1.6e-15  2.3e-15
Displacement field
Nonsymmetric matrix found.
Scales for dependent variables:
Auxiliary pressure (comp1.solid.lemm1.pw): 1
Displacement field (comp1.u): 2.8e-18
Orthonormal null-space function used.
Iter      SolEst     Damping    Stepsize #Res #Jac #Sol   LinErr   LinRes
   1         1.9   0.5000000         1.9    2    1    1  4.6e-14  1.5e-12
Spatial mesh displacement
```

```
Symmetric matrices found.
Scales for dependent variables:
Spatial mesh displacement (comp1.spatial.disp): 1.2e-09
Orthonormal null-space function used.
Iter      SolEst     Damping    Stepsize #Res #Jac #Sol   LinErr   LinRes
   1     8.6e-07   0.5000000     8.6e-07    2    1    1  1.4e-15  1.8e-15
Solution error estimates for segregated groups
1.4, 0.54, 0.96, 4.3e-07
Residual error estimates for segregated groups
7.8e+03, 8.1e+04, 2.1e+04, 0.11
Segregated solver iteration 2.
Merged variables
Scales for dependent variables:
Concentration (comp1.Cl): 1.4e+02
Concentration (comp1.K): 1.4e+02
Electric potential (comp1.V): 0.064
Iter      SolEst     Damping    Stepsize #Res #Jac #Sol   LinErr   LinRes
   1        0.78   0.3500000        0.78    6    4    4  9.7e-14  2.6e-15
   2        0.77   0.3500000        0.77    7    5    5  7.4e-14  2.7e-15
   3        0.75   0.3500000        0.75    8    6    6  8.3e-14  2.8e-15
Velocity u2, Pressure p
Scales for dependent variables:
Pressure (comp1.p): 3.7e+02
Velocity field (spatial frame) (comp1.u2): 0.00074
Iter      SolEst     Damping    Stepsize #Res #Jac #Sol   LinErr   LinRes
   1        0.36   0.5000000        0.36    4    2    2  3.6e-15  7.8e-15
Displacement field
Iter      SolEst     Damping    Stepsize #Res #Jac #Sol   LinErr   LinRes
   1        0.96   0.5000000        0.96    4    2    2  4.1e-14  1.4e-12
Spatial mesh displacement
Iter      SolEst     Damping    Stepsize #Res #Jac #Sol   LinErr   LinRes
   1     1.3e-06   0.5000000     1.3e-06    4    2    2  1.7e-15  1.8e-15
Solution error estimates for segregated groups
1.3, 0.18, 0.48, 6.5e-07
Residual error estimates for segregated groups
7.5e+02, 1.3e+04, 2.1e+04, 0.11
Segregated solver iteration 3.
Merged variables
Iter      SolEst     Damping    Stepsize #Res #Jac #Sol   LinErr   LinRes
   1        0.74   0.3500000        0.74   10    7    7  6.4e-14  2.6e-15
   2        0.73   0.3500000        0.73   11    8    8  5.5e-14  2.8e-15
   3        0.71   0.3500000        0.71   12    9    9  6.8e-14  2.7e-15
Velocity u2, Pressure p
Iter      SolEst     Damping    Stepsize #Res #Jac #Sol   LinErr   LinRes
   1        0.15   0.5000000        0.15    6    3    3  2.2e-15  7.9e-15
Displacement field
Iter      SolEst     Damping    Stepsize #Res #Jac #Sol   LinErr   LinRes
   1        0.52   0.5000000        0.52    6    3    3    8e-14  1.5e-12
Spatial mesh displacement
Iter      SolEst     Damping    Stepsize #Res #Jac #Sol   LinErr   LinRes
   1     1.3e-06   0.5000000     1.3e-06    6    3    3  1.6e-15  1.9e-15
Solution error estimates for segregated groups
1.2, 0.077, 0.26, 6.5e-07
Residual error estimates for segregated groups
2.1e+02, 7.6e+03, 1.6e+04, 0.12
Segregated solver iteration 4.
```

```
Merged variables
Iter      SolEst     Damping    Stepsize #Res #Jac #Sol   LinErr   LinRes
   1        0.69   0.3500000        0.69   14   10   10  1.3e-13  2.7e-15
   2        0.67   0.3500000        0.67   15   11   11  1.1e-13  3.8e-15
   3        0.58   0.3500000        0.58   16   12   12  7.5e-14  3.1e-15
Velocity u2, Pressure p
Iter      SolEst     Damping    Stepsize #Res #Jac #Sol   LinErr   LinRes
   1       0.072   0.5000000       0.072    8    4    4  1.9e-15  7.9e-15
Displacement field
Iter      SolEst     Damping    Stepsize #Res #Jac #Sol   LinErr   LinRes
   1        0.29   0.5000000        0.29    8    4    4  5.2e-14  1.5e-12
Spatial mesh displacement
Iter      SolEst     Damping    Stepsize #Res #Jac #Sol   LinErr   LinRes
   1     1.1e-06   0.5000000     1.1e-06    8    4    4    2e-15  1.8e-15
Solution error estimates for segregated groups
0.99, 0.036, 0.15, 5.4e-07
Residual error estimates for segregated groups
81, 4e+03, 1.1e+04, 0.12
Segregated solver iteration 5.
Merged variables
Iter      SolEst     Damping    Stepsize #Res #Jac #Sol   LinErr   LinRes
   1         0.4   0.3500000         0.4   18   13   13  8.3e-14  2.8e-15
   2        0.26   0.3500000        0.26   19   14   14  9.9e-14  2.7e-15
   3        0.18   0.3500000        0.18   20   15   15  6.3e-14  2.7e-15
Velocity u2, Pressure p
Iter      SolEst     Damping    Stepsize #Res #Jac #Sol   LinErr   LinRes
   1       0.035   0.5000000       0.035   10    5    5  3.3e-15  7.8e-15
Displacement field
Scales for dependent variables:
Auxiliary pressure (comp1.solid.lemm1.pw): 0.36
Displacement field (comp1.u): 3e-16
Iter      SolEst     Damping    Stepsize #Res #Jac #Sol   LinErr   LinRes
   1        0.17   0.5000000        0.17   10    5    5  1.1e-13  1.1e-12
Spatial mesh displacement
Iter      SolEst     Damping    Stepsize #Res #Jac #Sol   LinErr   LinRes
   1     8.1e-07   0.5000000     8.1e-07   10    5    5  1.8e-15  1.9e-15
Solution error estimates for segregated groups
0.3, 0.017, 0.084, 4e-07
Residual error estimates for segregated groups
32, 2e+03, 6.8e+03, 0.12
Segregated solver iteration 6.
Merged variables
Iter      SolEst     Damping    Stepsize #Res #Jac #Sol   LinErr   LinRes
   1        0.11   0.3500000        0.11   22   16   16    7e-14  2.8e-15
   2       0.075   0.3500000       0.075   23   17   17  4.4e-14  2.9e-15
   3       0.049   0.3500000       0.049   24   18   18    7e-14  2.7e-15
Velocity u2, Pressure p
Iter      SolEst     Damping    Stepsize #Res #Jac #Sol   LinErr   LinRes
   1       0.017   0.5000000       0.017   12    6    6  3.1e-15  7.8e-15
Displacement field
Iter      SolEst     Damping    Stepsize #Res #Jac #Sol   LinErr   LinRes
   1       0.096   0.5000000       0.096   12    6    6  1.5e-13  1.2e-12
Spatial mesh displacement
Iter      SolEst     Damping    Stepsize #Res #Jac #Sol   LinErr   LinRes
   1     5.7e-07   0.5000000     5.7e-07   12    6    6  1.3e-15  1.8e-15
Solution error estimates for segregated groups
```

```
0.084, 0.0086, 0.048, 2.8e-07
Residual error estimates for segregated groups
12, 1e+03, 4.2e+03, 0.12
Segregated solver iteration 7.
Merged variables
Iter      SolEst     Damping    Stepsize #Res #Jac #Sol   LinErr   LinRes
   1       0.032   0.3500000       0.032   26   19   19  4.6e-14  2.7e-15
   2       0.021   0.3500000       0.021   27   20   20  5.5e-14  2.8e-15
   3       0.014   0.3500000       0.014   28   21   21    6e-14  2.8e-15
Velocity u2, Pressure p
Iter      SolEst     Damping    Stepsize #Res #Jac #Sol   LinErr   LinRes
   1      0.0085   0.5000000      0.0085   14    7    7  2.9e-15  7.8e-15
Displacement field
Iter      SolEst     Damping    Stepsize #Res #Jac #Sol   LinErr   LinRes
   1       0.054   0.5000000       0.054   14    7    7  9.6e-14  1.2e-12
Spatial mesh displacement
Iter      SolEst     Damping    Stepsize #Res #Jac #Sol   LinErr   LinRes
   1     3.8e-07   0.5000000     3.8e-07   14    7    7  1.8e-15  1.8e-15
Solution error estimates for segregated groups
0.023, 0.0042, 0.027, 1.9e-07
Residual error estimates for segregated groups
4, 5.2e+02, 2.6e+03, 0.11
Segregated solver iteration 8.
Merged variables
Iter      SolEst     Damping    Stepsize #Res #Jac #Sol   LinErr   LinRes
   1      0.0088   0.3500000      0.0088   30   22   22  8.5e-14  2.8e-15
   2      0.0057   0.3500000      0.0057   31   23   23  4.1e-14  2.7e-15
   3      0.0037   0.3500000      0.0037   32   24   24  1.9e-13  2.7e-15
Velocity u2, Pressure p
Iter      SolEst     Damping    Stepsize #Res #Jac #Sol   LinErr   LinRes
   1      0.0042   0.5000000      0.0042   16    8    8  2.5e-15  7.9e-15
Displacement field
Iter      SolEst     Damping    Stepsize #Res #Jac #Sol   LinErr   LinRes
   1        0.03   0.5000000        0.03   16    8    8  1.4e-13  1.2e-12
Spatial mesh displacement
Iter      SolEst     Damping    Stepsize #Res #Jac #Sol   LinErr   LinRes
   1     2.4e-07   0.5000000     2.4e-07   16    8    8  1.5e-15  1.8e-15
Solution error estimates for segregated groups
0.0064, 0.0021, 0.015, 1.2e-07
Residual error estimates for segregated groups
1.3, 2.6e+02, 1.8e+03, 0.12
Segregated solver iteration 9.
Merged variables
Iter      SolEst     Damping    Stepsize #Res #Jac #Sol   LinErr   LinRes
   1      0.0024   0.3500000      0.0024   34   25   25  1.1e-13  3.1e-15
   2      0.0016   0.3500000      0.0016   35   26   26  4.8e-14  2.7e-15
   3       0.001   0.3500000       0.001   36   27   27  6.6e-14  2.7e-15
Velocity u2, Pressure p
Iter      SolEst     Damping    Stepsize #Res #Jac #Sol   LinErr   LinRes
   1      0.0021   0.5000000      0.0021   18    9    9  2.6e-15  7.7e-15
Displacement field
Iter      SolEst     Damping    Stepsize #Res #Jac #Sol   LinErr   LinRes
   1       0.017   0.5000000       0.017   18    9    9  1.2e-13  1.2e-12
Spatial mesh displacement
Iter      SolEst     Damping    Stepsize #Res #Jac #Sol   LinErr   LinRes
   1     1.5e-07   0.5000000     1.5e-07   18    9    9  1.3e-15  1.8e-15
```

```
Solution error estimates for segregated groups
0.0018, 0.0011, 0.0085, 7.6e-08
Residual error estimates for segregated groups
0.43, 1.3e+02, 1.4e+03, 0.13
Segregated solver iteration 10.
Merged variables
Iter      SolEst     Damping    Stepsize #Res #Jac #Sol   LinErr   LinRes
   1     0.00067   0.3500000     0.00067   38   28   28  7.9e-14  2.8e-15
   2     0.00043   0.3500000     0.00043   39   29   29  8.3e-14    3e-15
   3     0.00028   0.3500000     0.00028   40   30   30  9.5e-14  2.6e-15
Velocity u2, Pressure p
Iter      SolEst     Damping    Stepsize #Res #Jac #Sol   LinErr   LinRes
   1      0.0011   0.5000000      0.0011   20   10   10    4e-15  7.9e-15
Displacement field
Iter      SolEst     Damping    Stepsize #Res #Jac #Sol   LinErr   LinRes
   1      0.0094   0.5000000      0.0094   20   10   10  1.5e-13  1.1e-12
Spatial mesh displacement
Iter      SolEst     Damping    Stepsize #Res #Jac #Sol   LinErr   LinRes
   1     9.3e-08   0.5000000     9.3e-08   20   10   10  1.4e-15  1.8e-15
Solution error estimates for segregated groups
0.00048, 0.00053, 0.0047, 4.6e-08
Residual error estimates for segregated groups
0.14, 65, 1.2e+03, 0.12
Segregated solver iteration 11.
Merged variables
Iter      SolEst     Damping    Stepsize #Res #Jac #Sol   LinErr   LinRes
   1     0.00018   0.3500000     0.00018   42   31   31  6.6e-14  2.6e-15
   2     0.00012   0.3500000     0.00012   43   32   32  8.6e-14  2.9e-15
   3     7.7e-05   0.3500000     7.7e-05   44   33   33  6.6e-14  2.7e-15
Velocity u2, Pressure p
Iter      SolEst     Damping    Stepsize #Res #Jac #Sol   LinErr   LinRes
   1     0.00053   0.5000000     0.00053   22   11   11  2.4e-15  7.8e-15
Displacement field
Iter      SolEst     Damping    Stepsize #Res #Jac #Sol   LinErr   LinRes
   1      0.0052   0.5000000      0.0052   22   11   11  1.2e-13  1.2e-12
Spatial mesh displacement
Iter      SolEst     Damping    Stepsize #Res #Jac #Sol   LinErr   LinRes
   1     5.6e-08   0.5000000     5.6e-08   22   11   11  1.3e-15  1.8e-15
Solution error estimates for segregated groups
0.00013, 0.00026, 0.0026, 2.8e-08
Residual error estimates for segregated groups
0.043, 33, 1.2e+03, 0.12
Segregated solver iteration 12.
Merged variables
Iter      SolEst     Damping    Stepsize #Res #Jac #Sol   LinErr   LinRes
   1       5e-05   0.3500000       5e-05   46   34   34  8.8e-14    3e-15
   2     3.3e-05   0.3500000     3.3e-05   47   35   35  4.5e-14  2.8e-15
   3     2.1e-05   0.3500000     2.1e-05   48   36   36  4.6e-14  2.8e-15
Velocity u2, Pressure p
Iter      SolEst     Damping    Stepsize #Res #Jac #Sol   LinErr   LinRes
   1     0.00026   0.5000000     0.00026   24   12   12    2e-15  7.9e-15
Displacement field
Iter      SolEst     Damping    Stepsize #Res #Jac #Sol   LinErr   LinRes
   1      0.0028   0.5000000      0.0028   24   12   12  9.1e-14  1.1e-12
Spatial mesh displacement
Iter      SolEst     Damping    Stepsize #Res #Jac #Sol   LinErr   LinRes
```

```
   1     3.3e-08   0.5000000     3.3e-08   24    12    12  1.6e-15  1.8e-15
Solution error estimates for segregated groups
3.6e-05, 0.00013, 0.0014, 1.6e-08
Residual error estimates for segregated groups
0.013, 16, 1.2e+03, 0.11
Segregated solver iteration 13.
Merged variables
Iter      SolEst     Damping    Stepsize #Res #Jac #Sol   LinErr   LinRes
   1     1.4e-05   0.3500000     1.4e-05   50    37    37    1e-13    3e-15
   2       9e-06   0.3500000       9e-06   51    38    38  4.2e-14    3e-15
   3     5.8e-06   0.3500000     5.8e-06   52    39    39  1.1e-13  2.8e-15
Velocity u2, Pressure p
Iter      SolEst     Damping    Stepsize #Res #Jac #Sol   LinErr   LinRes
   1     0.00013   0.5000000     0.00013   26    13    13  2.5e-15  7.9e-15
Displacement field
Iter      SolEst     Damping    Stepsize #Res #Jac #Sol   LinErr   LinRes
   1      0.0015   0.5000000      0.0015   26    13    13  1.1e-13  1.2e-12
Spatial mesh displacement
Iter      SolEst     Damping    Stepsize #Res #Jac #Sol   LinErr   LinRes
   1     1.9e-08   0.5000000     1.9e-08   26    13    13  1.6e-15  1.8e-15
Solution error estimates for segregated groups
1e-05, 6.6e-05, 0.00076, 9.6e-09
Residual error estimates for segregated groups
0.004, 8.2, 1.2e+03, 0.11
Solution time: 155 s. (2 minutes, 35 seconds)
Physical memory: 4.9 GB
Virtual memory: 25.88 GB
Ended at Jan 31, 2025, 9:55:23 PM.
----- Stationary Solver 1 in study1/Approach Curve Solver (sol1) -------------->
```

d=1945 (su113)

GENERAL

Description	Value
Solution	d=1945

LOG

```
<---- Compile Equations: Stationary in study1/Approach Curve Solver (sol1) -----
Started at Jan 31, 2025, 9:55:24 PM.
Parameter d = 1.945E-6 (m).
Geometry shape function: Linear serendipity
Number of vertex elements: 18
Number of boundary elements: 6038
Number of elements: 153264
Minimum element quality: 0.4767
Time: 8 s.
Physical memory: 3.1 GB
Virtual memory: 15.25 GB
Ended at Jan 31, 2025, 9:55:31 PM.
----- Compile Equations: Stationary in study1/Approach Curve Solver (sol1) ---->

<---- Dependent Variables 1 in study1/Approach Curve Solver (sol1) -------------
Started at Jan 31, 2025, 9:55:31 PM.
Solution time: 0 s.
Physical memory: 3.1 GB
Virtual memory: 15.25 GB
Ended at Jan 31, 2025, 9:55:31 PM.
----- Dependent Variables 1 in study1/Approach Curve Solver (sol1) ------------>

<---- Stationary Solver 1 in study1/Approach Curve Solver (sol1) ---------------
Started at Jan 31, 2025, 9:55:32 PM.
Segregated solver
Segregated Step 11 does not have any components solved for.
Number of degrees of freedom solved for: 776972 (plus 11499 internal DOFs).
Segregated solver iteration 1.
Merged variables
Nonsymmetric matrix found.
Scales for dependent variables:
Concentration (comp1.Cl): 1.4e+02
Concentration (comp1.K): 1.4e+02
Electric potential (comp1.V): 1.9e+02
Orthonormal null-space function used.
Iter      SolEst     Damping    Stepsize #Res #Jac #Sol   LinErr   LinRes
   1        0.82   0.3500000        0.82    2    1    1  3.9e-11  2.7e-15
   2        0.81   0.3500000        0.81    3    2    2  3.3e-11  2.6e-15
   3         0.8   0.3500000         0.8    4    3    3  1.7e-11  2.7e-15
Velocity u2, Pressure p
Nonsymmetric matrix found.
Scales for dependent variables:
Pressure (comp1.p): 1
Velocity field (spatial frame) (comp1.u2): 0.00018
Orthonormal null-space function used.
Iter      SolEst     Damping    Stepsize #Res #Jac #Sol   LinErr   LinRes
   1         1.1   0.5000000         1.1    2    1    1  1.9e-15  2.3e-15
Displacement field
Nonsymmetric matrix found.
Scales for dependent variables:
Auxiliary pressure (comp1.solid.lemm1.pw): 1
Displacement field (comp1.u): 2.7e-18
Orthonormal null-space function used.
Iter      SolEst     Damping    Stepsize #Res #Jac #Sol   LinErr   LinRes
   1         1.9   0.5000000         1.9    2    1    1  5.6e-14  1.4e-12
Spatial mesh displacement
```

```
Symmetric matrices found.
Scales for dependent variables:
Spatial mesh displacement (comp1.spatial.disp): 1.2e-09
Orthonormal null-space function used.
Iter      SolEst     Damping    Stepsize #Res #Jac #Sol   LinErr   LinRes
   1     8.7e-07   0.5000000     8.7e-07    2    1    1  1.7e-15  1.8e-15
Solution error estimates for segregated groups
1.4, 0.54, 0.96, 4.4e-07
Residual error estimates for segregated groups
7.8e+03, 8.1e+04, 2.1e+04, 0.11
Segregated solver iteration 2.
Merged variables
Scales for dependent variables:
Concentration (comp1.Cl): 1.4e+02
Concentration (comp1.K): 1.4e+02
Electric potential (comp1.V): 0.064
Iter      SolEst     Damping    Stepsize #Res #Jac #Sol   LinErr   LinRes
   1        0.78   0.3500000        0.78    6    4    4  1.6e-13  3.8e-15
   2        0.77   0.3500000        0.77    7    5    5  1.1e-13  1.2e-14
   3        0.75   0.3500000        0.75    8    6    6  7.2e-14  9.9e-15
Velocity u2, Pressure p
Scales for dependent variables:
Pressure (comp1.p): 3.7e+02
Velocity field (spatial frame) (comp1.u2): 0.00074
Iter      SolEst     Damping    Stepsize #Res #Jac #Sol   LinErr   LinRes
   1        0.36   0.5000000        0.36    4    2    2  3.8e-15  7.8e-15
Displacement field
Iter      SolEst     Damping    Stepsize #Res #Jac #Sol   LinErr   LinRes
   1        0.96   0.5000000        0.96    4    2    2  2.8e-14  1.5e-12
Spatial mesh displacement
Iter      SolEst     Damping    Stepsize #Res #Jac #Sol   LinErr   LinRes
   1     1.3e-06   0.5000000     1.3e-06    4    2    2  1.6e-15  1.8e-15
Solution error estimates for segregated groups
1.3, 0.18, 0.48, 6.6e-07
Residual error estimates for segregated groups
7.5e+02, 1.4e+04, 2.1e+04, 0.12
Segregated solver iteration 3.
Merged variables
Iter      SolEst     Damping    Stepsize #Res #Jac #Sol   LinErr   LinRes
   1        0.74   0.3500000        0.74   10    7    7    9e-14  8.4e-15
   2        0.73   0.3500000        0.73   11    8    8  5.4e-14  1.2e-14
   3        0.71   0.3500000        0.71   12    9    9  8.9e-14  3.4e-15
Velocity u2, Pressure p
Iter      SolEst     Damping    Stepsize #Res #Jac #Sol   LinErr   LinRes
   1        0.15   0.5000000        0.15    6    3    3  2.1e-15  7.8e-15
Displacement field
Iter      SolEst     Damping    Stepsize #Res #Jac #Sol   LinErr   LinRes
   1        0.52   0.5000000        0.52    6    3    3  1.5e-13  1.5e-12
Spatial mesh displacement
Iter      SolEst     Damping    Stepsize #Res #Jac #Sol   LinErr   LinRes
   1     1.3e-06   0.5000000     1.3e-06    6    3    3  1.9e-15  1.8e-15
Solution error estimates for segregated groups
1.2, 0.077, 0.26, 6.6e-07
Residual error estimates for segregated groups
2.1e+02, 7.7e+03, 1.6e+04, 0.13
Segregated solver iteration 4.
```

```
Merged variables
Iter      SolEst     Damping    Stepsize #Res #Jac #Sol   LinErr   LinRes
   1        0.69   0.3500000        0.69   14   10   10  6.1e-14  4.6e-15
   2        0.67   0.3500000        0.67   15   11   11  1.2e-13  1.2e-14
   3        0.58   0.3500000        0.58   16   12   12  8.3e-14  4.6e-15
Velocity u2, Pressure p
Iter      SolEst     Damping    Stepsize #Res #Jac #Sol   LinErr   LinRes
   1       0.072   0.5000000       0.072    8    4    4  3.1e-15  7.9e-15
Displacement field
Iter      SolEst     Damping    Stepsize #Res #Jac #Sol   LinErr   LinRes
   1        0.29   0.5000000        0.29    8    4    4  8.7e-14  1.5e-12
Spatial mesh displacement
Iter      SolEst     Damping    Stepsize #Res #Jac #Sol   LinErr   LinRes
   1     1.1e-06   0.5000000     1.1e-06    8    4    4  2.2e-15  1.8e-15
Solution error estimates for segregated groups
0.99, 0.036, 0.15, 5.5e-07
Residual error estimates for segregated groups
81, 4.1e+03, 1.1e+04, 0.13
Segregated solver iteration 5.
Merged variables
Iter      SolEst     Damping    Stepsize #Res #Jac #Sol   LinErr   LinRes
   1         0.4   0.3500000         0.4   18   13   13  7.8e-14  5.9e-15
   2        0.27   0.3500000        0.27   19   14   14    1e-13  4.9e-15
   3        0.18   0.3500000        0.18   20   15   15  7.9e-14  1.1e-14
Velocity u2, Pressure p
Iter      SolEst     Damping    Stepsize #Res #Jac #Sol   LinErr   LinRes
   1       0.035   0.5000000       0.035   10    5    5    3e-15  7.9e-15
Displacement field
Scales for dependent variables:
Auxiliary pressure (comp1.solid.lemm1.pw): 0.36
Displacement field (comp1.u): 3e-16
Iter      SolEst     Damping    Stepsize #Res #Jac #Sol   LinErr   LinRes
   1        0.17   0.5000000        0.17   10    5    5  1.2e-13  1.1e-12
Spatial mesh displacement
Iter      SolEst     Damping    Stepsize #Res #Jac #Sol   LinErr   LinRes
   1     8.2e-07   0.5000000     8.2e-07   10    5    5  1.8e-15  1.8e-15
Solution error estimates for segregated groups
0.3, 0.017, 0.084, 4.1e-07
Residual error estimates for segregated groups
32, 2.1e+03, 6.8e+03, 0.13
Segregated solver iteration 6.
Merged variables
Iter      SolEst     Damping    Stepsize #Res #Jac #Sol   LinErr   LinRes
   1        0.12   0.3500000        0.12   22   16   16  6.7e-14  5.7e-15
   2       0.075   0.3500000       0.075   23   17   17  8.6e-14    7e-15
   3       0.049   0.3500000       0.049   24   18   18    1e-13  1.6e-14
Velocity u2, Pressure p
Iter      SolEst     Damping    Stepsize #Res #Jac #Sol   LinErr   LinRes
   1       0.017   0.5000000       0.017   12    6    6  3.3e-15  7.9e-15
Displacement field
Iter      SolEst     Damping    Stepsize #Res #Jac #Sol   LinErr   LinRes
   1       0.096   0.5000000       0.096   12    6    6  1.2e-13  1.1e-12
Spatial mesh displacement
Iter      SolEst     Damping    Stepsize #Res #Jac #Sol   LinErr   LinRes
   1     5.7e-07   0.5000000     5.7e-07   12    6    6  1.9e-15  1.9e-15
Solution error estimates for segregated groups
```

```
0.084, 0.0086, 0.048, 2.9e-07
Residual error estimates for segregated groups
12, 1.1e+03, 4.2e+03, 0.12
Segregated solver iteration 7.
Merged variables
Iter      SolEst     Damping    Stepsize #Res #Jac #Sol   LinErr   LinRes
   1       0.032   0.3500000       0.032   26   19   19  9.1e-14  1.2e-14
   2       0.021   0.3500000       0.021   27   20   20  8.3e-14  1.4e-14
   3       0.014   0.3500000       0.014   28   21   21  7.2e-14  7.7e-15
Velocity u2, Pressure p
Iter      SolEst     Damping    Stepsize #Res #Jac #Sol   LinErr   LinRes
   1      0.0085   0.5000000      0.0085   14    7    7  2.2e-15  7.8e-15
Displacement field
Iter      SolEst     Damping    Stepsize #Res #Jac #Sol   LinErr   LinRes
   1       0.054   0.5000000       0.054   14    7    7  1.3e-13  1.1e-12
Spatial mesh displacement
Iter      SolEst     Damping    Stepsize #Res #Jac #Sol   LinErr   LinRes
   1     3.8e-07   0.5000000     3.8e-07   14    7    7  1.5e-15  1.8e-15
Solution error estimates for segregated groups
0.023, 0.0042, 0.027, 1.9e-07
Residual error estimates for segregated groups
4, 5.3e+02, 2.6e+03, 0.11
Segregated solver iteration 8.
Merged variables
Iter      SolEst     Damping    Stepsize #Res #Jac #Sol   LinErr   LinRes
   1      0.0088   0.3500000      0.0088   30   22   22  9.8e-14  1.2e-14
   2      0.0057   0.3500000      0.0057   31   23   23  1.3e-13  7.7e-15
   3      0.0037   0.3500000      0.0037   32   24   24  1.1e-13  6.6e-15
Velocity u2, Pressure p
Iter      SolEst     Damping    Stepsize #Res #Jac #Sol   LinErr   LinRes
   1      0.0042   0.5000000      0.0042   16    8    8  3.9e-15  7.9e-15
Displacement field
Iter      SolEst     Damping    Stepsize #Res #Jac #Sol   LinErr   LinRes
   1        0.03   0.5000000        0.03   16    8    8  1.2e-13  1.1e-12
Spatial mesh displacement
Iter      SolEst     Damping    Stepsize #Res #Jac #Sol   LinErr   LinRes
   1     2.5e-07   0.5000000     2.5e-07   16    8    8  1.9e-15  1.8e-15
Solution error estimates for segregated groups
0.0064, 0.0021, 0.015, 1.2e-07
Residual error estimates for segregated groups
1.3, 2.7e+02, 1.8e+03, 0.11
Segregated solver iteration 9.
Merged variables
Iter      SolEst     Damping    Stepsize #Res #Jac #Sol   LinErr   LinRes
   1      0.0024   0.3500000      0.0024   34   25   25  1.1e-13  1.1e-14
   2      0.0016   0.3500000      0.0016   35   26   26  1.3e-13  9.9e-15
   3       0.001   0.3500000       0.001   36   27   27  7.2e-14  7.8e-15
Velocity u2, Pressure p
Iter      SolEst     Damping    Stepsize #Res #Jac #Sol   LinErr   LinRes
   1      0.0021   0.5000000      0.0021   18    9    9  2.6e-15    8e-15
Displacement field
Iter      SolEst     Damping    Stepsize #Res #Jac #Sol   LinErr   LinRes
   1       0.017   0.5000000       0.017   18    9    9  1.1e-13  1.2e-12
Spatial mesh displacement
Iter      SolEst     Damping    Stepsize #Res #Jac #Sol   LinErr   LinRes
   1     1.5e-07   0.5000000     1.5e-07   18    9    9  1.6e-15  1.9e-15
```

```
Solution error estimates for segregated groups
0.0018, 0.0011, 0.0085, 7.7e-08
Residual error estimates for segregated groups
0.43, 1.3e+02, 1.4e+03, 0.12
Segregated solver iteration 10.
Merged variables
Iter      SolEst     Damping    Stepsize #Res #Jac #Sol   LinErr   LinRes
   1     0.00067   0.3500000     0.00067   38   28   28  1.3e-13  1.3e-14
   2     0.00043   0.3500000     0.00043   39   29   29    1e-13  9.3e-15
   3     0.00028   0.3500000     0.00028   40   30   30  5.6e-14  4.1e-15
Velocity u2, Pressure p
Iter      SolEst     Damping    Stepsize #Res #Jac #Sol   LinErr   LinRes
   1      0.0011   0.5000000      0.0011   20   10   10  4.2e-15  7.9e-15
Displacement field
Iter      SolEst     Damping    Stepsize #Res #Jac #Sol   LinErr   LinRes
   1      0.0094   0.5000000      0.0094   20   10   10  1.7e-13  1.1e-12
Spatial mesh displacement
Iter      SolEst     Damping    Stepsize #Res #Jac #Sol   LinErr   LinRes
   1     9.4e-08   0.5000000     9.4e-08   20   10   10  1.5e-15  1.8e-15
Solution error estimates for segregated groups
0.00048, 0.00053, 0.0047, 4.7e-08
Residual error estimates for segregated groups
0.14, 67, 1.2e+03, 0.12
Segregated solver iteration 11.
Merged variables
Iter      SolEst     Damping    Stepsize #Res #Jac #Sol   LinErr   LinRes
   1     0.00018   0.3500000     0.00018   42   31   31  1.6e-13  6.9e-15
   2     0.00012   0.3500000     0.00012   43   32   32  1.2e-13    1e-14
   3     7.7e-05   0.3500000     7.7e-05   44   33   33  1.2e-13  9.4e-15
Velocity u2, Pressure p
Iter      SolEst     Damping    Stepsize #Res #Jac #Sol   LinErr   LinRes
   1     0.00053   0.5000000     0.00053   22   11   11  1.9e-15  7.8e-15
Displacement field
Iter      SolEst     Damping    Stepsize #Res #Jac #Sol   LinErr   LinRes
   1      0.0052   0.5000000      0.0052   22   11   11  1.3e-13  1.1e-12
Spatial mesh displacement
Iter      SolEst     Damping    Stepsize #Res #Jac #Sol   LinErr   LinRes
   1     5.6e-08   0.5000000     5.6e-08   22   11   11  1.6e-15  1.8e-15
Solution error estimates for segregated groups
0.00013, 0.00026, 0.0026, 2.8e-08
Residual error estimates for segregated groups
0.043, 33, 1.2e+03, 0.11
Segregated solver iteration 12.
Merged variables
Iter      SolEst     Damping    Stepsize #Res #Jac #Sol   LinErr   LinRes
   1       5e-05   0.3500000       5e-05   46   34   34  9.3e-14  9.2e-15
   2     3.3e-05   0.3500000     3.3e-05   47   35   35  4.9e-14  8.2e-15
   3     2.1e-05   0.3500000     2.1e-05   48   36   36  6.2e-14  5.6e-15
Velocity u2, Pressure p
Iter      SolEst     Damping    Stepsize #Res #Jac #Sol   LinErr   LinRes
   1     0.00026   0.5000000     0.00026   24   12   12  2.5e-15  7.8e-15
Displacement field
Iter      SolEst     Damping    Stepsize #Res #Jac #Sol   LinErr   LinRes
   1      0.0028   0.5000000      0.0028   24   12   12  9.9e-14  1.1e-12
Spatial mesh displacement
Iter      SolEst     Damping    Stepsize #Res #Jac #Sol   LinErr   LinRes
```

```
   1      3.3e-08   0.5000000     3.3e-08   24    12    12  1.3e-15   1.9e-15
Solution error estimates for segregated groups
3.6e-05, 0.00013, 0.0014, 1.7e-08
Residual error estimates for segregated groups
0.013, 17, 1.2e+03, 0.11
Segregated solver iteration 13.
Merged variables
Iter      SolEst     Damping    Stepsize #Res #Jac #Sol   LinErr   LinRes
   1      1.4e-05   0.3500000     1.4e-05   50    37    37  1.6e-13   1.3e-14
   2        9e-06   0.3500000       9e-06   51    38    38    9e-14   6.2e-15
   3      5.8e-06   0.3500000     5.8e-06   52    39    39  1.3e-13   6.9e-15
Velocity u2, Pressure p
Iter      SolEst     Damping    Stepsize #Res #Jac #Sol   LinErr   LinRes
   1      0.00013   0.5000000     0.00013   26    13    13  2.3e-15   7.8e-15
Displacement field
Iter      SolEst     Damping    Stepsize #Res #Jac #Sol   LinErr   LinRes
   1       0.0015   0.5000000      0.0015   26    13    13  1.3e-13   1.2e-12
Spatial mesh displacement
Iter      SolEst     Damping    Stepsize #Res #Jac #Sol   LinErr   LinRes
   1      1.9e-08   0.5000000     1.9e-08   26    13    13  1.4e-15   1.8e-15
Solution error estimates for segregated groups
1e-05, 6.6e-05, 0.00076, 9.7e-09
Residual error estimates for segregated groups
0.004, 8.4, 1.2e+03, 0.11
Solution time: 152 s. (2 minutes, 32 seconds)
Physical memory: 4.75 GB
Virtual memory: 25.48 GB
Ended at Jan 31, 2025, 9:58:03 PM.
----- Stationary Solver 1 in study1/Approach Curve Solver (sol1) -------------->
```

d=1940 (su114)

GENERAL

Description	Value
Solution	d=1940

LOG

```
<---- Compile Equations: Stationary in study1/Approach Curve Solver (sol1) -----
Started at Jan 31, 2025, 9:58:03 PM.
Parameter d = 1.94E-6 (m).
Geometry shape function: Linear serendipity
Number of vertex elements: 18
Number of boundary elements: 6035
Number of elements: 153025
Minimum element quality: 0.4952
Time: 8 s.
Physical memory: 3.15 GB
Virtual memory: 15.3 GB
Ended at Jan 31, 2025, 9:58:11 PM.
----- Compile Equations: Stationary in study1/Approach Curve Solver (sol1) ---->

<---- Dependent Variables 1 in study1/Approach Curve Solver (sol1) -------------
Started at Jan 31, 2025, 9:58:11 PM.
Solution time: 0 s.
Physical memory: 3.15 GB
Virtual memory: 15.3 GB
Ended at Jan 31, 2025, 9:58:11 PM.
----- Dependent Variables 1 in study1/Approach Curve Solver (sol1) ------------>

<---- Stationary Solver 1 in study1/Approach Curve Solver (sol1) ---------------
Started at Jan 31, 2025, 9:58:11 PM.
Segregated solver
Segregated Step 11 does not have any components solved for.
Number of degrees of freedom solved for: 775926 (plus 11493 internal DOFs).
Segregated solver iteration 1.
Merged variables
Nonsymmetric matrix found.
Scales for dependent variables:
Concentration (comp1.Cl): 1.4e+02
Concentration (comp1.K): 1.4e+02
Electric potential (comp1.V): 1.9e+02
Orthonormal null-space function used.
Iter      SolEst     Damping    Stepsize #Res #Jac #Sol   LinErr   LinRes
   1        0.82   0.3500000        0.82    2    1    1  2.6e-11  2.7e-15
   2        0.81   0.3500000        0.81    3    2    2  4.1e-11  2.6e-15
   3         0.8   0.3500000         0.8    4    3    3  6.7e-11  2.6e-15
Velocity u2, Pressure p
Nonsymmetric matrix found.
Scales for dependent variables:
Pressure (comp1.p): 1
Velocity field (spatial frame) (comp1.u2): 0.00018
Orthonormal null-space function used.
Iter      SolEst     Damping    Stepsize #Res #Jac #Sol   LinErr   LinRes
   1         1.1   0.5000000         1.1    2    1    1  1.8e-15  2.2e-15
Displacement field
Nonsymmetric matrix found.
Scales for dependent variables:
Auxiliary pressure (comp1.solid.lemm1.pw): 1
Displacement field (comp1.u): 2.8e-18
Orthonormal null-space function used.
Iter      SolEst     Damping    Stepsize #Res #Jac #Sol   LinErr   LinRes
   1         1.9   0.5000000         1.9    2    1    1    3e-14  1.4e-12
Spatial mesh displacement
```

```
Symmetric matrices found.
Scales for dependent variables:
Spatial mesh displacement (comp1.spatial.disp): 1.2e-09
Orthonormal null-space function used.
Iter      SolEst     Damping    Stepsize #Res #Jac #Sol   LinErr   LinRes
   1     8.8e-07   0.5000000     8.8e-07    2    1    1    2e-15  1.8e-15
Solution error estimates for segregated groups
1.4, 0.54, 0.96, 4.4e-07
Residual error estimates for segregated groups
7.8e+03, 8.1e+04, 2.1e+04, 0.099
Segregated solver iteration 2.
Merged variables
Scales for dependent variables:
Concentration (comp1.Cl): 1.4e+02
Concentration (comp1.K): 1.4e+02
Electric potential (comp1.V): 0.064
Iter      SolEst     Damping    Stepsize #Res #Jac #Sol   LinErr   LinRes
   1        0.78   0.3500000        0.78    6    4    4  7.2e-14  2.7e-15
   2        0.77   0.3500000        0.77    7    5    5  6.9e-14  2.7e-15
   3        0.75   0.3500000        0.75    8    6    6  7.3e-14  2.7e-15
Velocity u2, Pressure p
```
[truncated: 418,698 more chars]
